# Repeatability, Reproducibility, Separative Power and Subjectivity of Different Fish Morphometric Analysis Methods

**DOI:** 10.1371/journal.pone.0157890

**Published:** 2016-06-21

**Authors:** Péter Takács, Zoltán Vitál, Árpád Ferincz, Ádám Staszny

**Affiliations:** 1 MTA, Centre for Ecological Research, Balaton Limnological Institute, Tihany, Hungary; 2 Szent István University, Department of Aquaculture, Gödöllő, Hungary; Karlsruhe Institute of Technology, GERMANY

## Abstract

We compared the repeatability, reproducibility (intra- and inter-measurer similarity), separative power and subjectivity (measurer effect on results) of four morphometric methods frequently used in ichthyological research, the “traditional” caliper-based (TRA) and truss-network (TRU) distance methods and two geometric methods that compare landmark coordinates on the body (GMB) and scales (GMS). In each case, measurements were performed three times by three measurers on the same specimen of three common cyprinid species (roach *Rutilus rutilus* (Linnaeus, 1758), bleak *Alburnus alburnus* (Linnaeus, 1758) and Prussian carp *Carassius gibelio* (Bloch, 1782)) collected from three closely-situated sites in the Lake Balaton catchment (Hungary) in 2014. TRA measurements were made on conserved specimens using a digital caliper, while TRU, GMB and GMS measurements were undertaken on digital images of the bodies and scales. In most cases, intra-measurer repeatability was similar. While all four methods were able to differentiate the source populations, significant differences were observed in their repeatability, reproducibility and subjectivity. GMB displayed highest overall repeatability and reproducibility and was least burdened by measurer effect. While GMS showed similar repeatability to GMB when fish scales had a characteristic shape, it showed significantly lower reproducability (compared with its repeatability) for each species than the other methods. TRU showed similar repeatability as the GMS. TRA was the least applicable method as measurements were obtained from the fish itself, resulting in poor repeatability and reproducibility. Although all four methods showed some degree of subjectivity, TRA was the only method where population-level detachment was entirely overwritten by measurer effect. Based on these results, we recommend a) avoidance of aggregating different measurer’s datasets when using TRA and GMS methods; and b) use of image-based methods for morphometric surveys. Automation of the morphometric workflow would also reduce any measurer effect and eliminate measurement and data-input errors.

## Introduction

**Fig 1:**
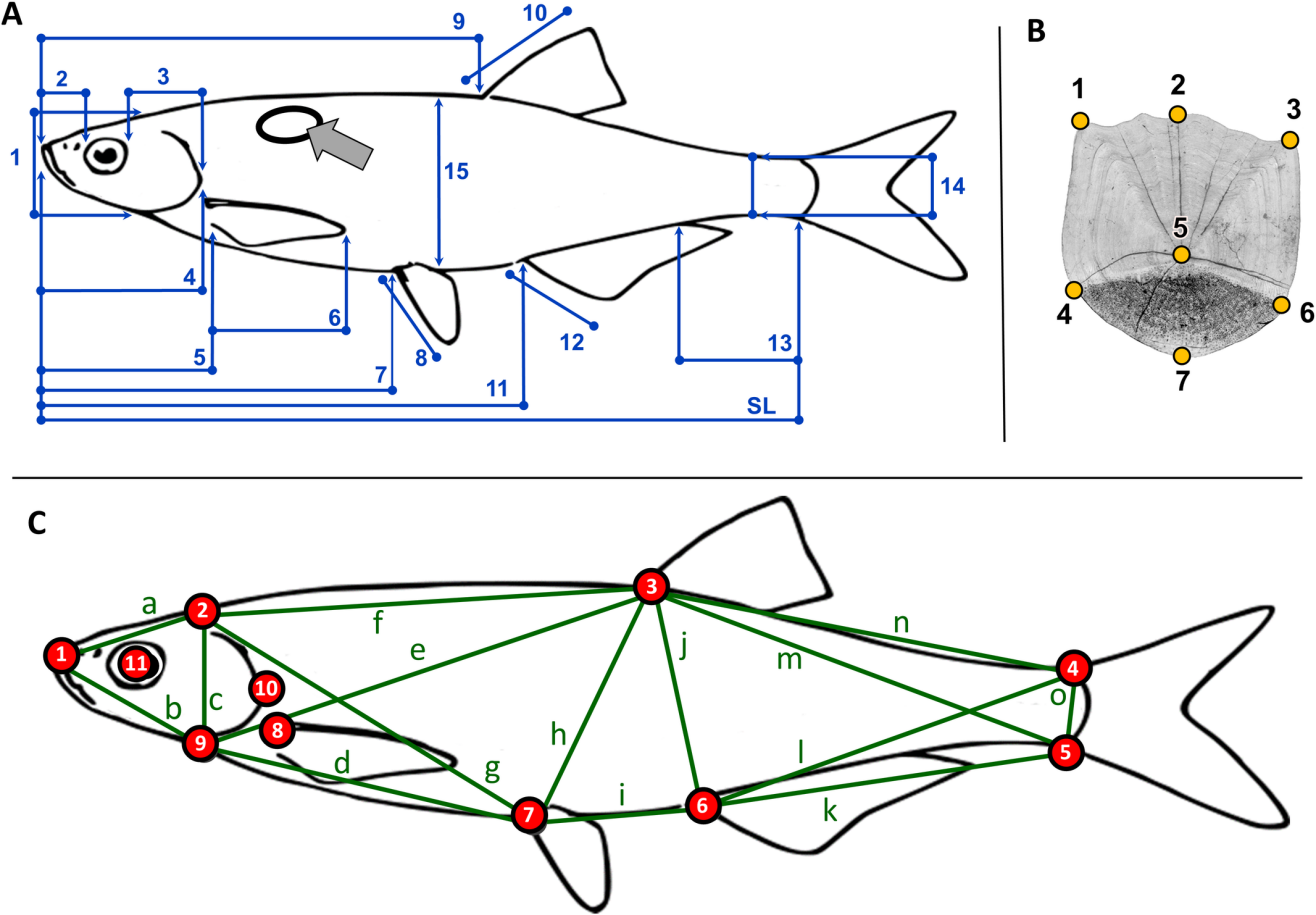
Morphometric landmarks and distances recorded by the three measurers. **A:** Distances measured when using the “traditional” (TRA) method (blue lines): 1—height of head, 2—preorbital distance, 3—postorbital distance, 4—head length, 5—prepectoral distance, 6—length of pectoral fin, 7—prepelvic distance, 8—length of pelvic fin, 9—predorsal distance, 10—length of dorsal fin, 11—preanal distance, 12—length of anal fin, 13—length of caudal peduncle, 14—minimum body depth, 15—maximum body depth, SL—standard length. The oval and arrow indicate the scale sampling area. **B:** codes for the seven landmarks recorded on scales (orange): 1—left cranial edge, 2—cranial end, 3—right cranial edge, 4—left caudal edge, 5—focus, 6—right caudal edge, 7—caudal peak. **C:** codes for the 11 landmarks recorded on the body (red): 1—tip of snout, 2 –occiput, 3—base of dorsal fin, 4—upper base of caudal fin, 5—lower base of caudal fin, 6—base of anal fin, 7—base of pelvic fin, 8—base of pectoral fin, 9—lower part of head, 10—posterior point of opercule, 11—middle point of eye. Fifteen between-landmark distances were used for truss-network (TRU) analysis, indicated by green letters and lines. Color codes corresponds with Figs 2 and 3. (For the raw datasets see S1–S4 Tables).

Morphological characteristics have been of fundamental importance in biology since the beginnings of the discipline. Indeed, the taxonomic classification of organisms [1] and the first steps in understanding the evolution of life [2] both came about through morphological descriptions of different forms. Morphologic investigations compare and analyse “meristic” and/or continuous “measureable” morphometric variables [3]. In the latter case, the morphometric characteristics selected are translated into numeric values so they can be analysed using appropriate statistical methods [4,5]. Morphologic investigations can be applied at various levels. Until recently, for example, morphological methods were generally used to differentiate species [6, 7, 8] or to describe intraspecific differences, such as sexual dimorphism [9,10] and/or population level detachments [11, 12]. Moreover, morphometric surveys can be applied to the entire body [13, 14] or to individual body parts, e.g. a fish scale, vertebra or otolith [15,16, 17, 18], depending on the goal of the survey. Over the last century, however, new morphometric methods have been developed. The oldest of these, the “traditional” distance-based method (hereafter TRA), measures the size (e.g. minimum body height, eye diameter, head length) and/or distance between specific body parts (e.g. prepelvic distance) of an individual [19] (e.g. see Fig 1A) and analyses these data further. The instruments used to measure distance will vary (e.g. tape measure or calipers) and the variables selected for measurement will vary depending on the size and the shape of the individual surveyed.

The TRA method was used exclusively until the introduction of the box-truss network (hereafter TRU) method at the begining of the 1980s [20]. While the TRU method also uses distance data, it relies on specifically identifiable, homologous points in order to eliminate many of the uncertainties inherent in the TRA method. For example, in ichthyological studies, individual variation in body shape may result in a shift of maximum and minimum body height (Fig 1A) along the fish’s body. The distance between the base of the dorsal and anal fin (see: Fig 1C) can be more precisely definied, however, as the distance is homologous in each individual measured. For more datails see [21].

A new family of morphometric methodolgies was developed at the end of the 20th century [22]. These ‘landmark-based geometric methods’ use coordinates of homologous points taken from digital images of the study objects and the data transformations and standardisations necessary require a strong computational background. As the use of personal computers has become wide-spread, however, such methods have quickly become the most widely used [23], though distance-based methods are also still applied [24, 25].

Despite the limited information available on the suitability and/or sensitivity of the different methods available, it is a generally accepted ‘fact’ that geometric methods display higher separative power than distance-based methods [26, 27]. Moreover, geometric methods are also generally considered less destructive, faster and cheaper than TRA methods [28, 29, 30]. While there have been some methodological studies assessing the quality of data and results obtained using these methods [31, 32, 33], very few have compared and quantified the applicability of the different methods now used in ichthyological studies (see: [34, 35]); and those that have were usually been unable to provide any statistical confirmation. It is a generally accepted tenant of science that unrepeatable or unreproducible measurements have no validity [36]; yet there is still very little information available regarding the repeatability (i.e. closeness of agreement between independent results obtained on identical subjects using the same method under the same conditions) or reproducibility (i.e. closeness of agreement between independent results obtained using the same method on identical subjects but under different conditions [37, 38]) of the different morphometric methods used today in ichthyology. Moreover, numerous studies compare or combine the datasets of different measurers [35]; meaning that, in most cases, the results will be more or less biased by a ‘measurer effect’, despite the use of well-defined protocols [39, 40, 41]. As this phenomenon is generally well-recognised, it is frequently advised that the data of just one measurer is used, especially in the case of population-level studies [42, 43, 44, 45]. Less well-known, however, is how measurer variability affects the results of different morphometric methods and how this can change based on a species’ physical characteristics.

In this study, our aim is to survey the applicability of four different morphometric methods based on five aspects: 1) repeatability and 2) reproducibility of measurement, 3) separative power, 4) degree of measurer effect and 5) how such features change depending on the physical characteristics of the different species analysed.

## Material and Methods

### Ethics statement

This study was undertaken following all relevant national and international guidelines pertaining to the care and welfare of fish. Fish collection was authorised by the Ministry of Rural Development (Permit no.: EHVF/188-1/2014). All procedures used in this study were approved by the Committee on the Ethics of Animal Experiments of the Hungarian Academy of Sciences’ Centre for Ecological Research (Permit no.: VE-I-001/01890-3/2013). During sampling, every effort was made to minimise the suffering of fish and all fish were anaesthetised with a lethal dose of clove oil prior to analysis. No endangered species (according to the IUCN Red List of Threatened Species v. 2015–4 [www.iucnredlist.org]) were caught during this study.

### Sample collection and data management

In this study we applied two distance based morphometric methods (TRA, TRU) and two geometric methods using body (GMB) and scale (GMS) landmarks to three common and wide-spread cyprinid fish species. Thirty specimens each of roach *Rutilus rutilus* (Linnaeus, 1758), bleak *Alburnus alburnus* (Linnaeus, 1758) and Prussian (gibel) carp *Carassius gibelio* (Bloch, 1782) were collected by electrofishing from three sampling sites (Site1: N46.63474 E17.17433, Site2: N46.79983 E17.38822, Site3: N46.75362 E17.56720) in the Lake Balaton catchment area (Hungary) in 2014. The sampling sites were situated relatively close to each other, with a straight-line distance between sites 1 and 2 of 24 km, 33 km between sites 1 and 3, and 15 km between sites 2 and 3. All fish were euthenised with a lethal dose of clove oil before fixing in 4% formaldehyde [46], whereupon the samples were moved to the laboratory and tagged with a unique identification code for further analysis. After tagging, each fish was placed flat on a table surface and the left side was photographed from a perpendicular angle using a tripod-mounted Nikon D50 digital camera angle for GMB and TRU analysis. A single scale was then removed from the area anterior to the dorsal fin of each specimen for GMS analysis (see. Fig 1). All scales were placed between two glass slides and scanned with a Hewlet Packard ScanJet 5300C XPA scanner at 2400 dpi.

Eleven easily defined landmarks were recorded on body and scale images for GMB, and seven for GMS (Fig 1A and 1B), using tpsUtil and tpsDig2 digital imaging software, both of which were specifically developed for digitising landmarks and outlines for geometric morphometric analysis (for more details see: [47, 48]). Sixteen inter-landmark distances were recorded on the digital images for TRU analysis, the distances being measured using freeware imageJ software (for more details see: [49]). For TRA analysis, 16 commonly-used taxonomic measurements [50, 51, 35] were taken from the left side of each individual (i.e. not from a photographic image) using a digital caliper, all data being recorded to the nearest 0.01 mm. In total, 26,730 measurements (three species x three populations x 30 individuals x three measurers x three repeats x 11 variables) were undertaken during GMB analysis, 17,010 measurements during GMS analysis (three species x three populations x 30 individuals x three measurers x three repeats x seven variables), and 38,880 measurements each during TRU and TRA analysis (three species x three populations x 30 individuals x three measurers x three repeats x 16 variables). For definitions of the measured characteristics, and for the most important features of the methods tested and compared in this study see Table 1.

**Table 1:** Summary description of the four morphometric methods tested and compared in our study.

Abbreviation	Method type	Method description	Object measured	Data extraction equipment	No. of variables
**TRA**	Distance-based	“Traditional” method—distance between specific body parts	Actual body	Digital calipers	16
**TRU**	Distance-based	Distance between homologous body landmarks	Body image	imageJ software	16
**GMB**	Geometric	Distance between homologous body landmark coordinates	Body image	tpsUtil and tpsDig2 digital imaging softwares	11
**GMS**	Geometric	Distance between homologous scale landmark coordinates	Scale image	tpsUtil and tpsDig2 digital imaging softwares	7

All measurements were undertaken by the same three measurers (M1, M2, M3), the whole sample set being repeated three times. Before measurement started, the three measurers discussed the actual methods of measurement (TRA, TRU) and image marking (GMB, GMS) prior to the survey in order to anticipate any discrepancies originating from different conventions and/or personal methods (see: [52]). All three measurers were right handed, thereby preventing bias in the results from left or right-handedness (see: [53]). During TRA measurement, the measurers paid special attention to avoid discrepencies from individual fish degradation. Since all measurements were made within a relatively short period (two months) after sample collection, the chances of preservative-caused morphometric differences were considered negligible [46]. All specimens measured have been deposited within the institution’s fish collection and all fish and images therefrom are accessible from the corresponding author.

To eliminate any size-effect in the TRA and TRU datasets [54], we used the allometric formula of Elliott et al. [55], i.e. M_adj_ = M (L_s_—L_0_)^b^ where M is the original measurement, M_adj_ is the size adjusted measurement, L_o_ is the standard length (SL) of the fish, and Ls is the overall mean SL for all fish from all samples in each analysis. The standardised data were rechecked by correlating against the original SL values. For GMB and GMS, a full Procrustes fit was undertaken on the landmark data, followed by multivariate regression analysis on the logarithm of Centroid Size (logCS) [56]. Statistical analysis was performed on the residuals of the regression analysis in order to eliminate any size effect.

For testing repeatability and reproducibility there is no generally accepted protocol [38]. In some cases each measured morphometric variable is tested independently on some selected specimens [57, 58, 59], or the repeteadly recorded entire datasets of the whole analysed stocks can be compared [60, 61]. In our case, we chose to follow the latter method, with repeatability calculated as ‘intra-measurer similarity of three independently recorded datasets obtained from the same population by the same method’. Intra-measurer similarity was computed as follows: each of the three datasets derived from the same population was converted into a distance matrix using Euclidean distance and compared by pairwise Mantel-tests [62]. Pairwise comparison correlation coefficients (R) and significance (p) values were used to characterise the level of similarity between the three repeated measurements. Values for R ranged between 0 and 1, with 0 representing complete differentiation and 1 representing complete agreement (repeatability) between the two datasets. The R values were arranged into groups in order to assess repeatability at three different consecutive levels: I—measurer, II—species and III—method. Reproducibility was computed in a similar manner, but with inter-measurer similarity of independent datasets compared using the same method from the same population. The R values of pairwise Mantel-tests were then arranged into groups at two consecutive levels: I–species, and II—method. Differences found between groups at consecutive levels were tested using the non-parametric Kruskal-Wallis test.

Separative power and subjectivity (i.e. measurer’s influence on the results) were assessed *via* a datamatrix containing a randomly chosen dataset from the three repeats on the same individual. In each case, the results were analysed using Canonical Variate Analyses (CVA) and two-way permutational ANOVA (PERMANOVA) [63] of Euclidean distance with 9 999 permutations. The analysis was performed independently for each method and for each species. All statistical analyses were carried out using PAST v.2.17c software [64].

## Results

### Raw data and standardisation process

TRA measurement indicated SLs ranging from 61.1 to 130.9 mm for bleak, 64.5 to 135.7 mm for roach and 43.7 to 157.7 mm for Prussian carp. All raw data for GMB and GMS landmark analysis and the variables measured for TRU and TRA analysis are presented in supplementary S1–S4 Tables. None of the variables measured showed any significant correlation with SL data after standarisation; hence, all variables were used for further analysis.

### Repeatability and reproducibility

Measurement repeatability (correlation coefficient of pairwise Mantel-tests) varied between 0.015 and 0.986, with 308 of 324 (95.1%) semi-matrix pairwise comparisons from repeated measurements displaying significant correlations (p < 0.05). For GMB and TRU analysis, all comparisons were significant, while just one result was not significant using GMS. TRA indicated just 65 of 81 pairwise comparisons (80.2%) as significant (S5 Table).

**Fig 2:**
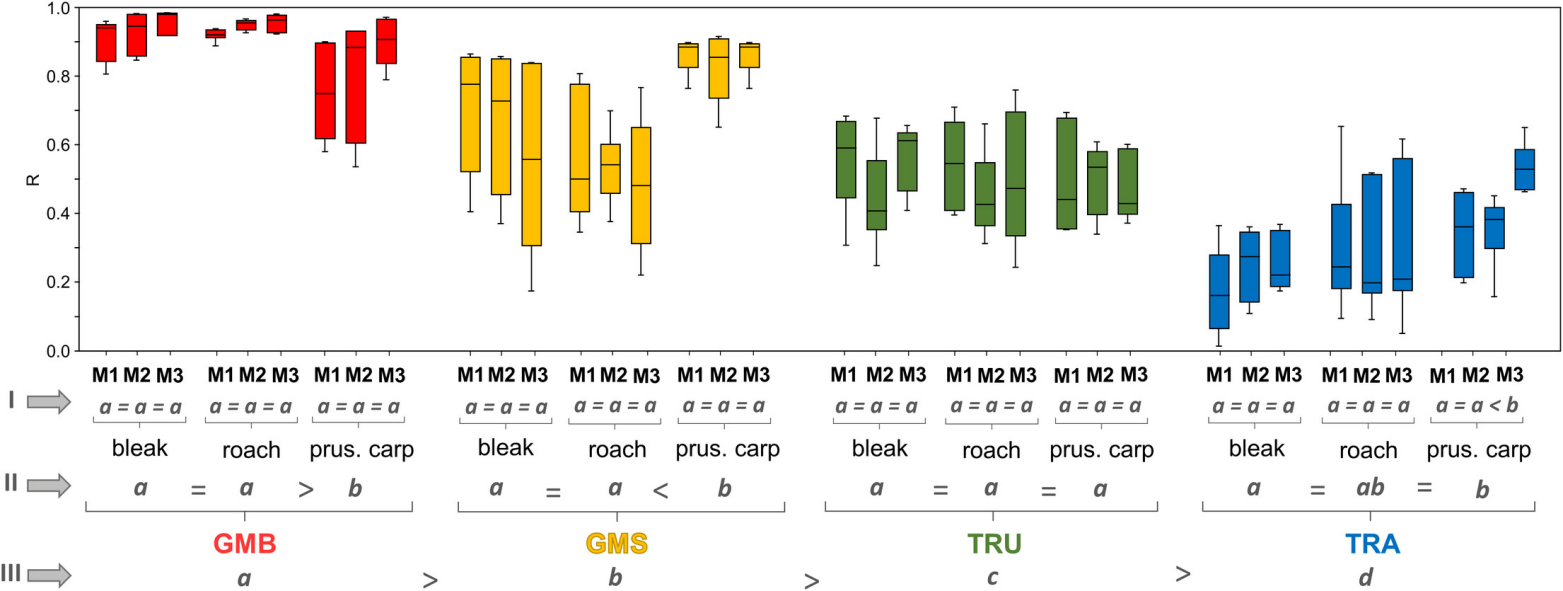
Boxplots for repeatability derived from pairwise Mantel tests of intra-measurer R values (for data see S5 Table). Nine pairwise R values, obtained from the same measurer’s data, were used for each box. Each box represents the 25% and 75% quartiles while the line in the box represents the median. The whiskers show the highest and lowest values within the dataset. In rows indicated by grey Roman numerals, the datasets were analysed at different levels, i.e. I (measurer; n = 9), II (species; n = 27) and III (method; n = 81). Groups with the same letter did not differ significantly (p < 0.05) using the Kruskal-Wallis test. Color codes corresponds with Figs 1 and 3.

Mean repeatability values indicated only slight differences between most comparisons at level 1 (measurer), with only one of 36 crosschecks (2.7%) showing significant inter-measurer differentiation (Fig 2, level I). Moreover, most R values derived from different measurer datasets showed a similar range-spread for each species.

In comparison, major differences were found in repeatability at the two higher levels (Fig 2, levels II and III). At level II (species), mean repeatability (mean ± SD) of GMB measurements on Prussian carp was significantly lower (0.820 ± 0.13) than that of the other two species (roach 0.943 ± 0.02, bleak 0.934 ± 0.05; no significant difference between roach and bleak). On the other hand, both bleak and roach displayed significantly lower GMS repeatability (bleak 0.658 ± 0.20, roach 0.528 ± 0.20) than Prussian carp (0.854 ± 0.06). Using TRA, significant differences were observed between repeatability values for bleak (0.226 ± 0.11) and Prussian carp (0.363 ± 0.16), but not between roach and bleak or Prussian carp (0.292 ± 0.17). No significant species-level differences were observed in mean repeatability using TRU (bleak 0.508 ± 0.14, roach 0.482 ± 0.14, Prussian carp 0.493 ± 0.11). At level III (method), measurement repeatibility improved from TRA (0.294 ± 0.16) through TRU (0.439 ± 0.13) and GMS (0.680 ± 0.20) to GMB (0.899 ± 0.10), with crosschecks between each method being significant (p < 0.05; Fig 2, level III).

**Fig 3:**
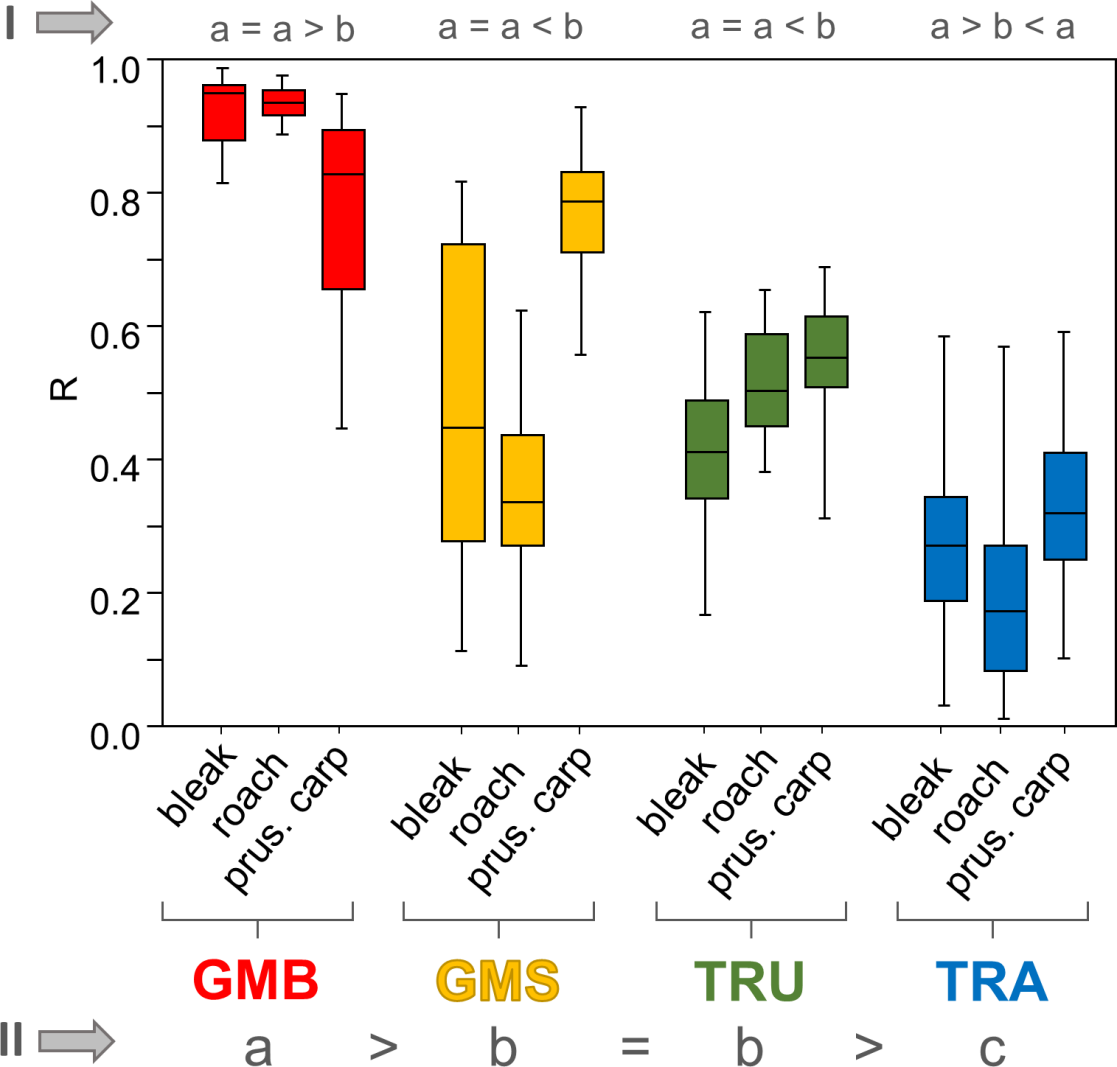
Boxplots of reproducibility derived from pairwise Mantel tests of inter-measurer R values (for data see S6 Table). Each box presents 81 pairwise R values obtained from a comparison of datasets derived from the same subjects by different measurers. The box represents the 25% and 75% quartiles, with the line in the box representing the median. The whiskers show the highest and lowest values within the dataset. In rows indicated by grey Roman numerals, datasets were analysed at different levels, i.e. I (species; n = 81) and II (method; n = 243). Groups with the same letter did not differ significantly (p < 0.05) using the Kruskal-Wallis test. Color codes corresponds with Figs 1 and 2.

Measurement reproducibility ranged between 0.01 and 0.99, with 882 of 972 pairwise comparisons (90.7%) exhibiting significant correlations. All pairwise comparsions for GMB were significant for all three species investigated, while 239 of 243 (98.4%) were significant for GMS and 230 of 243 (94.78%) for TRU. TRA displayed the lowest number of significant pairwise comparisons, with 170 of 243 (69.9%) (S6 Table). At the species level (I), mean (± SD) reproducibility of GMB measurements on Prussian carp was significantly lower (0.774 ± 0.14) than than that for roach and bleak (roach 0.935 ± 0.02, bleak 0.926 ± 0.05), with no significant difference between these species (Fig 3).

Mean (± SD) GMS measurement reproducibility for Prussian carp (0.769 ± 0.08) was significantly higher than that for bleak (0.6481 ± 0.22) or roach (0.360 ± 0.13). A similar trend was observed for TRU reproducibility, with highest mean (± SD) values observed for Prussian carp (0.578 ± 0.126), followed by roach (0.450 ± 0.13) and bleak (0.421 ± 0.14). For TRA, however, roach (0.187 ± 0.12) showed significantly lower reproducibility than either Prussian carp (0.324 ± 0.12) or bleak (0.274 ± 0.13). At the method level (II), GMB showed significantly higher reproducibility (0.878 ± 0.11) than either GMS (0.536 ± 0.23) or TRU (0.483 ± 0.15), with lowest mean values recorded for TRA (0.262 ± 0.14).

A comparison of repeatability and reproducibility data indicated lower mean values for reproducibility, both at the species and method level, though significant differences were only observed for GMS in all three species investigated (Kruskal-Wallis test, p < 0.05).

### Separative power and subjectivity

In almost all cases, CVA analysis indicated significant differentiation of the three study populations. For GMB, 26 out of 27 pairwise population comparisons showed significant isolation (Table 2), with all three populations differing from each other significantly in eight cases out of nine. For GMS, 21 of 27 comparisons were significantly different, and 23 of 27 comparisons for TRU, with all three populations differing significantly from each other in five cases each. For TRA, 21 pairwise comparisons were significantly isolated, and in six cases all populations were significantly isolated from each other. In just two cases, the populations showed no significant detachment (roach-GMS-M1 and Prussian carp-TRA-M2).

**Table 2:** Level of detachment of the three study populations using canonical variate analysis.

species	method/ measurer	GMB	GMS	TRU	TRA
**Bleak**	M1	***	***	***	***
** **	M2	***	**	**	***
** **	M3	***	***	**	***
**Roach**	M1	**	-	***	***
** **	M2	***	**	**	***
** **	M3	***	***	**	***
**Prussian carp**	M1	***	***	***	*
** **	M2	***	**	***	-
** **	M3	***	***	***	**
**all pops. differ significantly**		8/9	5/9	5/9	6/9
**significant differences**		26/27	21/27	23/27	21/27

*** = all three populations significantly separated

** = two of three populations significantly isolated

* = one population significantly isolated

- = no significant isolaton between populations; all at p < 0.05.

**Fig 4:**
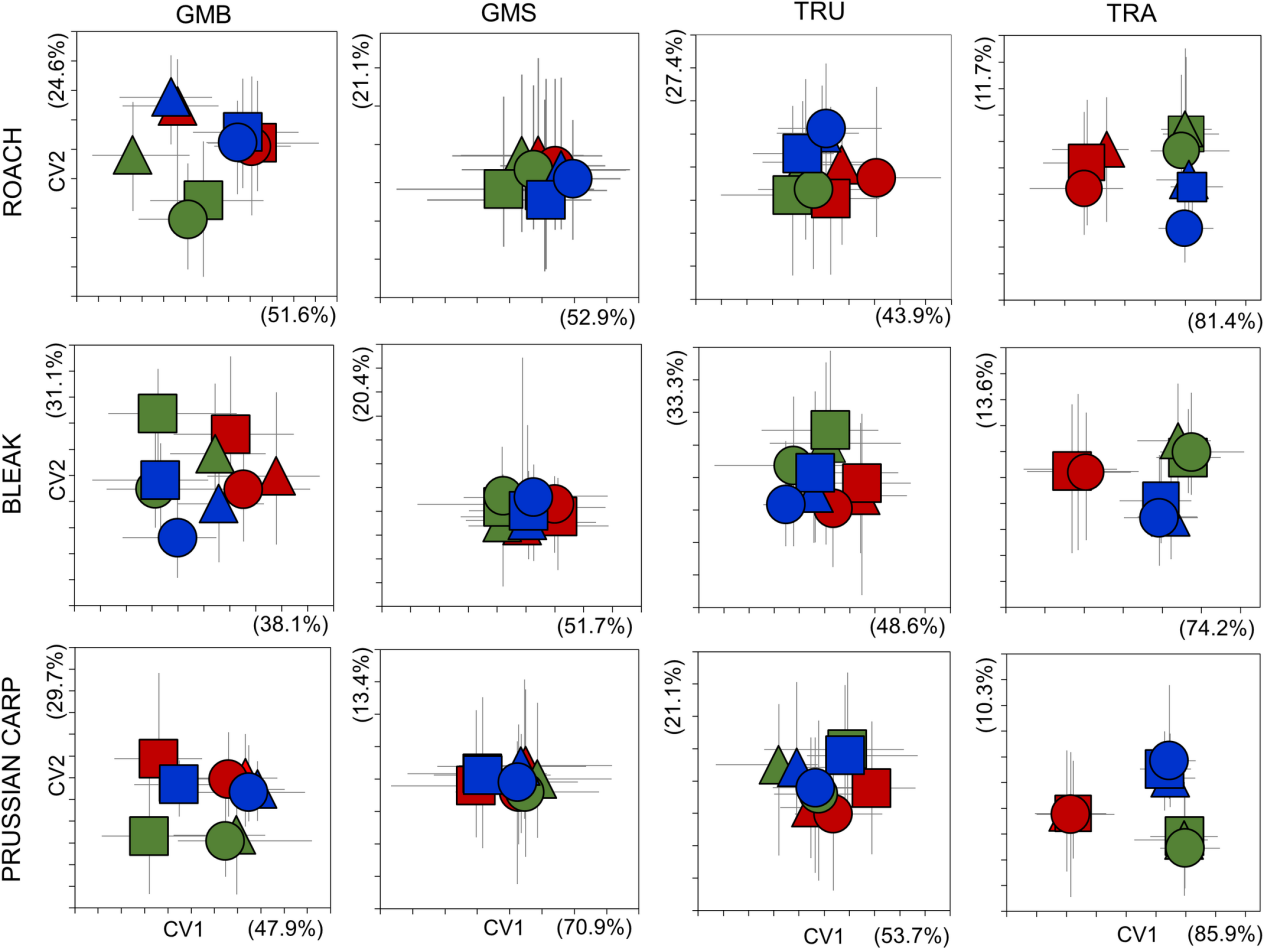
Canonical variate analysis scatterplots of standardised morphometric data derived from three species by three measurers using four different methods. For clarity, only the group centroids are indicated, with vertical and horizontal whiskers indicating the maximum and minimum values. Each measurer is represented by a different colour (M1—red, M2—green, M3—blue), while each site is represented by a different shape (Site1—△, Site2—⬜, Site3—○). Numbers in parentheses show the variance on each axis.

Analysis of subjectivity indicated strong differences between the methods studied. Using the GMB method, CVA scatter plots indicated that all three study populations were separated from each other in the same manner by all three measurers, and that this pattern was detected for all three species (Fig 4). Note, however, that the relative positions of the different measurers group centroids were shifted slightly (ghosting) along the y and/or x axes in all cases. A similar effect was noted for both TRU and GMS, though the study populations were much less separated. This was especially true in case of GMS, where differentiation was much weaker and the datasets overlapped much more than GMB for all three species. In the case of TRA, a very different pattern was detected, the group centroids being aggregated according to measurer rather than sampling site (Fig 4).

Overall, therefore, CVA scatterplots indicated measurer impact on differentiation with all methods tested, though measurer role in separation was only important for all three species in TRA analysis. These findings were supported by the results of two-way PERMANOVA analysis, with both site and measurer having a significant effect on population differentiation in most cases, independent of method used (Table 3). In the case of GMB, higher F values were calculated for site for all three species, whereas only Prussian carp showed this pattern using GMS and TRU. Using GMS, the role of measurer was higher than that for sampling site in the differentiation of roach and bleak populations, while the effect of measurer was notably higher than site for all three species using TRA.

**Table 3:** Results of two-way PERMANOVA analysis (9 999 permutations) for effect of site and measurer. High F values with bold letters indicate a significant effect (p < 0.05) of site and/or measurer.

Method	Species	Source	Sum of sqrs	df	Mean square	F	p
**GMB**	Roach	**sampling site**	0.043	2	0.021	**9.81**	**0.0001**
		measurer	0.025	2	0.013	5.84	**0.0001**
		Interaction	0.003	4	0.001	0.32	0.9995
		Residual	0.567	261	0.002		
		Total	0.638	269			
	Bleak	**sampling site**	0.034	2	0.017	**7.91**	**0.0001**
		measurer	0.027	2	0.014	6.28	**0.0002**
		Interaction	0.003	4	0.001	0.36	0.989
		Residual	0.568	261	0.002		
		Total	0.633	269			
	Prussian Carp	**sampling site**	0.074	2	0.037	**17.88**	**0.0001**
		measurer	0.025	2	0.012	5.98	**0.0001**
		Interaction	0.004	4	0.001	0.45	0.9989
		Residual	0.542	261	0.002		
		Total	0.644	269			
**GMS**	Roach	sampling site	0.021	2	0.011	2.42	**0.0085**
		**measurer**	0.059	2	0.029	**6.67**	**0.0001**
		Interaction	0.016	4	0.004	0.88	0.6123
		Residual	1.15	261	0.004		
		Total	1.246	269			
	Bleak	sampling site	0.07	2	0.035	3.22	**0.0004**
		**measurer**	0.097	2	0.048	**4.44**	**0.0001**
		Interaction	0.038	4	0.009	0.87	0.6325
		Residual	2.85	261	0.011		
		Total	3.055	269			
	Prussian Carp	**sampling site**	0.095	2	0.047	**9.80**	**0.0001**
		measurer	0.017	2	0.008	1.73	0.0558
		Interaction	0.008	4	0.002	0.42	0.9962
		Residual	1.258	261	0.005		
		Total	1.378	269			
**TRU**	Roach	sampling site	0.108	2	0.054	6.05	**0.0001**
		**measurer**	0.143	2	0.072	**8.04**	**0.0001**
		Interaction	0.019	4	0.005	0.53	0.9899
		Residual	2.322	261	0.009		
		Total	2.591	269			
	Bleak	sampling site	0.112	2	0.056	6.30	**0.0001**
		**measurer**	0.202	2	0.101	**11.43**	**0.0001**
		Interaction	0.025	4	0.006	0.7	0.908
		Residual	2.311	261	0.009		
		Total	2.649	269			
	Prussian Carp	**sampling site**	0.32331	2	0.162	**14.79**	**0.0001**
		measurer	0.068632	2	0.034	3.14	**0.0001**
		Interaction	0.036716	4	0.009	0.84	0.7093
		Residual	2.8531	261	0.011		
		Total	3.2818	269			
**TRA**	Roach	sampling site	0.095	2	0.047	5.79	**0.0002**
		**measurer**	2.238	2	1.119	**136.66**	**0.0001**
		Interaction	0.05	4	0.012	1.52	0.1082
		Residual	2.137	261	0.008		
		Total	4.519	269			
	Bleak	sampling site	0.148	2	0.074	7.06	**0.0001**
		**measurer**	1.961	2	0.98	**93.66**	**0.0001**
		Interaction	0.082	4	0.021	1.97	**0.0179**
		Residual	2.732	261	0.01		
		Total	4.924	269			
	Prussian Carp	sampling site	0.043	2	0.021	1.74	0.1077
		**measurer**	4.686	2	2.343	**190.62**	**0.0001**
		Interaction	0.055	4	0.014	1.13	0.3114
		Residual	3.208	261	0.012		
		Total	7.993	269			

## Discussion

Our results indicate that all four methods tested were able to detect morphometric differences between the different fish populations, despite the relatively narrow geographic scale. Nevertheless, the features examined showed considerable differences in some cases, with mean repeatability using GMB, for example, three times higher than that for TRA. Our results correspond with those of Parsons et al. [29], who showed that geometric/morphometric methods had higher separative power, making them more applicable than traditional, distance-based methods. At the same time, all four methods were more-or-less burdened by different negative effects; hence, all the methods studied had weaknesses and strengths, affecting their applicability.

In most cases, measurement repeatability did not differ between measurers. Hence, as long as measurements are carried out by competent analysts, all methods are equally usable as regards repeatability. At the species level, repeatability and reproducibility showed similar trends, though reproducibility was lower in each case. In roach and bleak, both repeatability and reproducibility decreased from the GMB through GMS and TRU to TRA. For both species, GMB measurements showed > 90% repeatability, which corresponds well with the literature (e.g. [65]). There was no significant difference in repeatability using GMB and GMS for Prussian carp, presumably due to its more characteristic scale shape [66]. Therefore GMS appear to be equally applicable as GMB, as long as the species examined has a characteristic scale shape. Moreover as Staszny et al. [67] discussed, body shape is more influenced by the conditional status of a fish than scale shape; hence, scale shape is less sensitive to short-term environmental effects (e.g. starvation). On the other hand, unlike the other three methods, GMS measurements showed significantly lower reproducability (compared with its repeatability) for each species. In this case, therefore, it would appear important that a single measurer’s dataset is used. When using TRU and GMB, however, datasets of different measurers may be combined if the methodology and other influencing factors are the same (e.g. for species level differentiation, supraspecific taxonomic researche using a large number of individuals).

Analysis of subjectivity indicated that all the morphometric methods were influenced by measurer effect to a greater or lesser degree. Even in the case of GMB, which is generally less burdened by measurer effect, the relative position of different measurer group centroids were shifted along the y and/or x axes in all cases. At the same time, TRA was the only method where population level detachment was entirely overwritten by measurer effect. Despite the lack of any significant difference between repeatability and reproducibility using TRA, therefore, calculation of subjectivity indicated that different measurers could have a crucial affect on analysis results; hence, it is recommended to avoid from datasets aggregation of different measurers in this case. Very low levels of repeatability and reproducibility were detected for TRA in some cases (≈1%), possibly due to errors in measurement or during the data entry process (clerical errors). Direct data entry [68] (rather than transfer from paper to computer, as in our case) can reduce the number of data entry errors during fish morphometric survey. TRA repeatability did not correspond absolutely with that of the other three methods tested, possibly as the fish were handled during measurement, the other three methods utilising static images for markings and measurement. In this case, slight differences in the positioning of the fish between measurements may have affected measurement repeatability (for more details see [31]). The use of image analysis techniques instead of actual body measurement, therefore, clearly improved the applicability of distance-based methods.

Our findings (with distance-based TRA and TRU methods showing similar separative power to GMS) partially contradict those of Medebacher [26], who stated that “traditional morphometrics is often at its limit when closely related entities are analysed”. On the other hand, our results correspond with those of Franklin et al. [69], where analysis using some carefully selected TRA variables can provide similar results to those from geometric morphometric methods using numerous landmarks. Moreover, a combination of meristic variables and the TRA method could further increase the separative power of the analysis [70, 71]. Similarly, Ibáñez & O’Higgins [72] shows that GMS separative power could be strengthened with the use of form (shape and size data) instead of shape alone.

Our results draw attention to the importance of measurer skill and expertise, especially when planning morphometric studies. Furthermore, when deciding on the morphometric method to be used, factors such as the selection of variables should be considered alongside the function of specific physical characteristics (e.g. scale shape) for each species examined. Our data showed that, whereas the same set of variables appeared appropriate for differentiating roach and bleak populations, they were less suitabe for discerning Prussian carp populations (see Table 2). For best results, therefore, the most appropriate method and morphometric variables to be used will depend on the species studied. Our study showed that all the morphometric methods tested are appropriate for detecting even population level differences. Athough the methods differed considerably in their sensitivity, separative power and subjectivity, the final results were strongly influenced by attributes of the species investigated and by the measurer’s skill and expertise. The considerable impact of measurer effect on the results provides some weight to the need for greater automation of morphometric analysis, including distance measurement and landmark processing, data standardisation and statistical analysis. This would also help reduce the level of measurment and data input errors. The methodologies of other disciplines (e.g. medicine and astronomy) that are already largely automated could prove useful in the automatisation of morphometric assessment [73,74, 75], though further methodological studies are needed in order to identify the most appropriate methods (or combination of methods) and measurement variables for individual species and for the goals of individual studies.

## Supporting Information

S1 Table Raw dataset of the GMB analyses, for codes see text. (DOCX)

S2 Table Raw dataset of the GMS analyses, for codes see text. (DOCX)

S3 Table Raw dataset of the TRU analyses, for codes see text. (DOCX)

S4 Table Raw dataset of the TRA analyses, for codes see text. (DOCX)

S5 Table Results of repeatability computations. „R” values and significance levels (* = p<0.05; ** = p<0.01) of pairwise Mantel tests made on Euclidean distance matrices of the repeated measurement data of the same measurers.
(DOCX)

S6 Table Results of reproduciblity computations. „R” values and significance levels (* = p<0.05; ** = p<0.01) of pairwise Mantel tests made on Euclidean distance matrices of the datasets of two different measurers.
(DOCX)

## References

[1] Linnaeus C. Systema naturae per regna tria naturae secundum classes ordines genera species (Vol. 1). impensis Georg Emanuel Beer; 1788.

[2] Darwin C. On the origins of species by means of natural selection. London: Murray 247

[3] Turan C. Stock identification of Mediterranean horse mackerel (Trachurus mediterraneus) using morphometric and meristic characters. ICES Journal of Marine Science: Journal du Conseil. 2004;61(5): 774–781.

[4] Goodall C. Procrustes methods in the statistical analysis of shape. J R Stat Soc B. 1991;285–339.

[5] Carpenter KE, Sommer H.J III, Marcus LF. Converting truss interlandmark distances to Cartesian coordinates In Advances in Morphometrics (pp. 103–111). Springer US; 1996

[6] Lindsey CC. Sympatric occurrence of two species of humpback whitefish in Squanga Lake Yukon Territory. J Fish Board Can. 1963; 20.3: 749–767.

[7] Creech S. A multivariate morphometric investigation of Atherina boyeri Risso 1810 and A. presbyter Cuvier 1829 (Teleostei: Atherinidae): morphometric evidence in support of the two species. J Fish Biol 1992;41(3): 341–353.

[8] Doadrio I, Carmona JA, Fernandez-Delgado C. Morphometric study of the Iberian Aphanius (Actinopterygii Cyprinodontiformes) with description of a new species. Fol Zool. 2002;51(1): 67–80.

[9] Rohlf FJ. Morphometrics. Annu Rev Ecol S. 1990;21: 299–316.

[10] Kitano J, Mori S, Peichel CL. Sexual dimorphism in the external morphology of the threespine stickleback (*Gasterosteus aculeatus*). Copeia 2007;2007(2): 336–349.

[11] Herler J, Kerschbaumer M, Mitteroecker P, Postl L, Sturmbauer C. Sexual dimorphism and population divergence in the Lake Tanganyika cichlid fish genus Tropheus. Front Zool. 2010;7(1): 4. 20205752 10.1186/1742-9994-7-4 PMC2822758

[12] Jørgensen HB, Pertoldi C, Hansen MM, Ruzzante DE, Loeschcke V. Genetic and environmental correlates of morphological variation in a marine fish: the case of Baltic Sea herring (Clupea harengus). Can. J Fish Aquat Sci. 2008;65(3): 389–400.

[13] Trapani J. Geometric morphometric analysis of body-form variability in Cichlasoma minckleyi the Cuatro Cienegas cichlid. Environ. Biol Fish. 2003;68(4): 357–369.

[14] Nowak M, Mendel J, Szczerbik P, Klaczak A, Mikołajczyk T, Ozga K, et al Contributions to the morphological variation of the common gudgeon *Gobio gobio* complex (Teleostei: Cyprinidae) in the upper Vistula drainage (southeast Poland). Arch Pol Fish. 2011;19(1): 37–49.

[15] Ponton D. Is geometric morphometrics efficient for comparing otolith shape of different fish species? J Morph. 2006;267(6): 750–757. 16526058 10.1002/jmor.10439

[16] Ibañez AL, Cowx IG, O'Higgins P. Geometric morphometric analysis of fish scales for identifying genera species and local populations within the Mugilidae. Can J Fish Aquat Sci. 2007;64(8): 1091–1100.

[17] Huber HR, Jorgensen JC, Butler VL, Baker G, Stevens R. Can salmonids (*Oncorhynchus spp*.) be identified to species using vertebral morphometrics? J Archaeol Sci. 2011;38(1): 136–146.

[18] Agüera A, Brophy D. Use of saggital otolith shape analysis to discriminate Northeast Atlantic and Western Mediterranean stocks of Atlantic saury *Scomberesox saurus saurus* (Walbaum). Fish Res. 2011; 110(3): 465–471.

[19] Schaefer MB, Walford LA. Biometric Comparison Between Yellowfin Tunas (*Necthunnus*) of Angola and of the Pacific Coast of Central America. US Government Printing Office; 1950

[20] Strauss RE, Bookstein FL. The truss: body form reconstructions in morphometrics. Syst Biol. 1982;31(2): 113–135.

[21] Zelditch ML, Swiederski DL, Sheets HD, Fink WL. Geometric Morphometrics for Biologists: A primer. New York: Elsevier Academic Press; 2004.

[22] Rohlf FJ, Marcus LF. A revolution in morphometrics. Trends Ecol Evol. 1993;8: 129–132. 10.1016/0169-5347(93)90024-J 21236128

[23] Adams DC, Rohlf FJ, Slice DE. Geometric morphometrics: ten years of progress following the ‘revolution’. Ital J Zool. 2004;71(1): 5–16.

[24] Sajina AM, Chakraborty SK, Jaiswar AK, Pazhayamadam DG, Sudheesan D. Stock structure analysis of *Megalaspis cordyla* (Linnaeus 1758) along the Indian coast based on truss network analysis. Fish Res. 2011;108(1): 100–105.

[25] Szlachciak J, Nowak M. Morphology of the only known population of Kessler’s gudgeon *Romanogobio kesslerii* (Teleostei: Cyprinidae) outside the Black Sea basin. Biologia. 2015;70(1): 121–131.

[26] Maderbacher M. Bauer C. Herler J. Postl L. Makasa L. Sturmbauer C. Assessment of traditional versus geometric morphometrics for discriminating populations of the Tropheus moorii species complex (Teleostei: Cichlidae) a Lake Tanganyika model for allopatric speciation. J Zool Syst Evol Res. 2008;46(2): 153–161.

[27] Ginter CC, DeWitt TJ, Fish FE, Marshall CD. Fused Traditional and Geometric Morphometrics Demonstrate Pinniped Whisker Diversity. PLoS ONE. 2012;7(4): e34481 10.1371/journal.pone.0034481 22509310 PMC3317988

[28] Cadrin SX, Friedland KD. The utility of image processing techniques for morphometric analysis and stock identification. Fish Res. 1999;43(1): 129–139.

[29] Parsons K.J. Robinson B.W. Hrbek T. Getting into shape: an empirical comparison of traditional truss-based morphometric methods with a newer geometric method applied to New World cichlids. Environ. Biol. Fish. 2003;67(4) 417–431.

[30] Viscosi V, Lepais O, Gerber S, Fortini P. Leaf morphological analyses in four European oak species (*Quercus*) and their hybrids: A comparison of traditional and geometric morphometric methods. Plant Biosys. 2009;143(3): 564–574.

[31] Arnqvist G, Martensson T. Measurement error in geometric morphometrics: empirical strategies to assess and reduce its impact on measures of shape. Acta Zool Acad Sci Hung. 1998; 44(1–2): 73–96.

[32] Adams GL, Gansky SA, Miller AJ, Harrell WE, Hatcher DC. Comparison between traditional 2-dimensional cephalometry and a 3-dimensional approach on human dry skulls. Am J Orthod Dentofac. 2004b;126(4): 397–409. 10.1016/j.ajodo.2004.03.023 15470343

[33] Kocovsky PM, Adams JV, Bronte CR. The effect of sample size on the stability of principal components analysis of truss-based fish morphometrics. T Am Fish Soc. 2009;138(3): 487–496.

[34] González-Castro M, Ibáñez AL, Heras S, Roldán MI, Cousseau MB. Assessment of lineal versus landmark-based morphometry for discriminating species of Mugilidae (Actinopterygii). Zool Stud. 2012;51(8): 1515–1528.

[35] Petrtýl M, Kalous L, Memis D. Comparison of Manual Measurements and Computer Assisted Image Analysis in Fish Morphometry. Turk J Vet Anim Sci. 2014;38: 88–94.

[36] Chinn S. Statistics in respiratory medicine. 2. Repeatability and method comparison. Thorax. 1991;46(6): 454–456. 1858087 10.1136/thx.46.6.454 PMC463197

[37] Bartlett JW, Frost C. Reliability repeatability and reproducibility: analysis of measurement errors in continuous variables. Ultrasound Obst Gyn. 2008;31(4): 466–475. 10.1002/uog.5256 18306169

[38] Slezák P, Waczulíková I. Reproducibility and repeatability. Physiol Res. 2011;60: 203–205. 21469910

[39] Rabinovich S. Measurement errors Theory and practice. New York: American Institute of Physics c1995 1; 1995

[40] Yezerinac SM, Lougheed SC, Handford P. Measurement error and morphometric studies: statistical power and observer experience. Syst Biol. 1992;41(4): 471–482.

[41] Roitberg ES, Orlova VF, Kuranova VN, Bulakhova NA, Zinenko OI, Ljubisavljevic K, et al Inter-observer and intra-observer differences in measuring body length: A test in the common lizard *Zootoca vivipara*. Amphibia-Reptilia. 2011;32(4): 477–484.

[42] Rohlf FJ. On applications of geometric morphometrics to studies of ontogeny and phylogeny. Syst Biol. 1998;147–158. 12064235 10.1080/106351598261094

[43] Lee JC. Accuracy and precision in anuran morphometrics: artifacts of preservation. Syst Biol. 1982;31(3): 266–281.

[44] Dujardin JPA, Kaba D, Henry AB. The exchangeability of shape. BMC research notes. 2010;3(1) 266. 20964872 10.1186/1756-0500-3-266 PMC2987866

[45] Lehtinen RM, Glaw F, Andreone F, Pabijan M, Vences M. A new species of putatively pond breeding frog of the genus Guibemantis from southeastern Madagascar. Copeia. 2012;2012(4): 648–662.

[46] Distefano RJ, Roell MJ, Wagner BA, Decoske JJ. Relative performances of four preservatives on fish and crayfish. T Am Fish Soc. 1994;123(5): 817–823.

[47] Rohlf FJ. tpsUtil file utility program. version 1.46 Department of Ecology and Evolution state university of new york at stony brook; 2010a.

[48] Rohlf FJ. tpsdig2 digitize landmarks and outlines version 2.16 department of ecology and evolution state university of new york at stony brook; 2010b.

[49] Rasband WS. ImageJ: Image processing and analysis in Java. Astrophysics Source Code Library 1 06013; 2012.

[50] Berinkey L. Halak-Pisces Fauna Hungarie Akadémiai Kiadó Budapest 132; (1966)

[51] Villéger S, Miranda JR, Hernández DF, Mouillot D. Contrasting changes in taxonomic vs. functional diversity of tropical fish communities after habitat degradation. Ecol Appl. 2010;20: 1512–1522. 20945756 10.1890/09-1310.1

[52] Howe JC. Standard length: not quite so standard. Fish Res. 2002;56(1): 1–7.

[53] Helm B, Albrecht H. Human handedness causes directional asymmetry in avian wing length measurements. Anim Behav. 2000;60(6): 899–902. 11124889 10.1006/anbe.2000.1534

[54] Bookstein FL, Chernoff B, Elder RL, Humphries JM Jr. Smith GR, Strauss RE. Morphometrics in evolutionary biology. Special publication 15 Academy of Natural Sciences Press Philadelphia; 1985

[55] Elliott NG, Haskard K, Koslow JA. Morphometric analysis of orange roughy (*Hoplostethus atlanticus*) off the continental slope of southern Australia. J Fish Biol. 1995;46: 202–220.

[56] Klingenberg CP. MorphoJ: an integrated software package for geometric morphometrics. Mol Ecol Res. 2011;11(2): 353–357. 10.1111/j.1755-0998.2010.02924.x 21429143

[57] Bailey RC, Byrnes J. A new old method for assessing measurement error in both univariate andmultivariate morphometric studies. Syst Zool. 1990;39: 124–130.

[58] Arechavala-Lopez P, Sanchez-Jerez P, Bayle-Sempere JT, Sfakianakis DG, Somarakis S. Morphological differences between wild and farmed Mediterranean fish. Hydrobiologia. 2012;679(1): 217–231.

[59] Essner RL Jr, Patel R, Reilly SM. Ontogeny of Body Shape and Diet in Freshwater Drum (*Aplodinotus grunniens*). Trans Ill St Acad Sci. 2014: 107: 27–30.

[60] Martinón-Torres M, Bastir M, De Castro JB, Gómez A, Sarmiento S, Muela A, et al Hominin lower second premolar morphology: evolutionary inferences through geometric morphometric analysis. J Hum Evol. 2006;50(5): 523–533. 16472839 10.1016/j.jhevol.2005.12.004

[61] Gómez-Robles A, Martinón-Torres M, De Castro JB, Margvelashvili A, Bastir M, Arsuaga JL, et al A geometric morphometric analysis of hominin upper first molar shape. J Hum Evol. 2007;53(3): 272–285. 17599390 10.1016/j.jhevol.2007.02.002

[62] Mantel NA. The detection of disease clustering and a generalized regression approach. Can Res. 1967;27: 209–220. 6018555

[63] Anderson MJ. A new method for non-parametric multivariate analysis of variance. Austral Ecol. 2001;26: 32–46.

[64] Hammer Ø, Harper DAT, Ryan PD. PAST: paleontological statistics software package education and data analysis. Palaeontol Electron. 2001;4: 9p

[65] Gómez GF, Márquez EJ, Gutiérrez LA, Conn JE, Correa MM, Geometric morphometric analysis of Colombian *Anopheles albimanus* (Diptera: Culicidae) reveals significant effect of environmental factors on wing traits and presence of a metapopulation. Acta tropica. 2014;135: 75–85. 10.1016/j.actatropica.2014.03.020 24704285 PMC4464773

[66] Staszny Á, Ferincz Á, Weiperth A, Havas E, Urbányi B, Paulovits G. Scale-morphometry study to discriminate gibel carp (*Carassius gibelio*) populations in the balaton-catchment (Hungary). Acta Zool Acad Sci Hung. 2012;58: 19–27.

[67] Staszny Á, Havas E, Kovács R, Urbányi B, Paulovits G, Bencsik D, et al Impact of Environmental and Genetic Factors on the Scale Shape of Zebrafish *Danio rerio* (Hamilton 1822): A Geometric Morphometric Study. Acta Biol Hung. 2013;64(4): 462–475. 10.1556/ABiol.64.2013.4.6 24275592

[68] Lynnerup N, Lynnerup O. Automatic data acquisition of anthropological measurements. Comput Biol Med. 1993;23(2): 143–147. 8513665 10.1016/0010-4825(93)90145-q

[69] Franklin D, Cardini A, Flavel A, Kuliukas A. The application of traditional and geometric morphometric analyses for forensic quantification of sexual dimorphism: preliminary investigations in a Western Australian population. Int J Legal Med. 2012;126(4): 549–558. 10.1007/s00414-012-0684-8 22399102

[70] Takács P. Morphometric differentiation of gudgeon species inhabiting the Carpathian Basin. Ann Limnol-Int J Lim. 2012;48: 53–61.

[71] Pinheiro A, Teixeira CM, Rego AL, Marques JF, Cabral HN. Genetic and morphological variation of *Solea lascaris* (Risso 1810) along the Portuguese coast. Fish Res. 2005;73(1): 67–78.

[72] Ibáñez AL, O’Higgins P. Identifying fish scales: The influence of allometry on scale shape and classification. Fish Res. 2011;109: 54–60.

[73] Heckemann RA, Keihaninejad S, Aljabar P, Gray KR, Nielsen C, Rueckert D, et al Automatic morphometry in Alzheimer's disease and mild cognitive impairment. Neuroimage 2011;56.4: 2024–2037. 21397703 10.1016/j.neuroimage.2011.03.014 PMC3153069

[74] Calès P, Chaigneau J, Hunault G, Michalak S, Cavaro-Menard C, Fasquel JB, et al Automated morphometry provides accurate and reproducible virtual staging of liver fibrosis in chronic hepatitis C. J Pathol Inform, 2015;6: 20 10.4103/2153-3539.157782 26110088 PMC4466784

[75] Bertin E. Automated Morphometry with SExtractor and PSFEx. In Astronomical Data Analysis Software and Systems XX 2011;442: 435.

