# Supplementary material for: Repeatability, Reproducibility, Separative Power and Subjectivity of Different Fish Morphometric Analysis Methods
Source: PLoS One. 2016 Jun 21;11(6):e0157890. doi: 10.1371/journal.pone.0157890 (PMC4915670; doi:10.1371/journal.pone.0157890)
Supplement: S1 Table — (DOCX) [file pone.0157890.s001.docx]

Supplementary Table 1 Raw dataset of the GMB analyses, for codes see text.

| Species | individual code | Measurer | Site | repeat | x1 | y1 | x2 | y2 | x3 | y3 | x4 | y4 | x5 | y5 | x6 | y6 | x7 | y7 | x8 | y8 | x9 | y9 | x10 | y10 | x11 | y11 |
| --- | --- | --- | --- | --- | --- | --- | --- | --- | --- | --- | --- | --- | --- | --- | --- | --- | --- | --- | --- | --- | --- | --- | --- | --- | --- | --- |
| Bleak | 1031 | 1 | 1 | 1 | -0.350 | 0.032 | -0.226 | 0.076 | 0.138 | 0.127 | 0.518 | 0.057 | 0.511 | -0.032 | 0.211 | -0.072 | 0.038 | -0.094 | -0.156 | -0.041 | -0.233 | -0.065 | -0.167 | -0.005 | -0.282 | 0.016 |
| Bleak | 1032 | 1 | 1 | 1 | -0.346 | 0.029 | -0.222 | 0.086 | 0.130 | 0.109 | 0.502 | 0.070 | 0.521 | -0.018 | 0.217 | -0.102 | 0.049 | -0.109 | -0.155 | -0.035 | -0.235 | -0.054 | -0.178 | -0.002 | -0.282 | 0.026 |
| Bleak | 1033 | 1 | 1 | 1 | -0.343 | 0.053 | -0.220 | 0.083 | 0.119 | 0.101 | 0.521 | 0.074 | 0.508 | -0.013 | 0.211 | -0.095 | 0.051 | -0.115 | -0.162 | -0.051 | -0.231 | -0.057 | -0.169 | -0.006 | -0.286 | 0.025 |
| Bleak | 1034 | 1 | 1 | 1 | -0.339 | 0.060 | -0.211 | 0.086 | 0.139 | 0.105 | 0.498 | 0.082 | 0.505 | -0.008 | 0.229 | -0.106 | 0.059 | -0.131 | -0.170 | -0.060 | -0.240 | -0.053 | -0.184 | -0.009 | -0.287 | 0.033 |
| Bleak | 1035 | 1 | 1 | 1 | -0.350 | 0.056 | -0.207 | 0.090 | 0.104 | 0.096 | 0.513 | 0.081 | 0.517 | -0.007 | 0.208 | -0.106 | 0.054 | -0.125 | -0.156 | -0.057 | -0.257 | -0.062 | -0.152 | 0.002 | -0.274 | 0.032 |
| Bleak | 1036 | 1 | 1 | 1 | -0.355 | 0.045 | -0.219 | 0.080 | 0.122 | 0.109 | 0.511 | 0.072 | 0.518 | -0.016 | 0.211 | -0.096 | 0.041 | -0.114 | -0.160 | -0.050 | -0.237 | -0.065 | -0.160 | 0.007 | -0.273 | 0.028 |
| Bleak | 1037 | 1 | 1 | 1 | -0.354 | 0.023 | -0.215 | 0.080 | 0.133 | 0.117 | 0.510 | 0.064 | 0.519 | -0.025 | 0.211 | -0.087 | 0.038 | -0.100 | -0.157 | -0.037 | -0.238 | -0.055 | -0.167 | -0.005 | -0.281 | 0.024 |
| Bleak | 1038 | 1 | 1 | 1 | -0.345 | 0.045 | -0.226 | 0.084 | 0.146 | 0.099 | 0.502 | 0.069 | 0.505 | -0.015 | 0.226 | -0.088 | 0.059 | -0.108 | -0.171 | -0.054 | -0.234 | -0.050 | -0.172 | -0.005 | -0.291 | 0.023 |
| Bleak | 1039 | 1 | 1 | 1 | -0.344 | 0.068 | -0.215 | 0.085 | 0.131 | 0.100 | 0.499 | 0.086 | 0.508 | -0.006 | 0.235 | -0.110 | 0.039 | -0.126 | -0.159 | -0.066 | -0.247 | -0.054 | -0.166 | -0.008 | -0.280 | 0.032 |
| Bleak | 1040 | 1 | 1 | 1 | -0.360 | 0.029 | -0.219 | 0.086 | 0.137 | 0.109 | 0.513 | 0.064 | 0.512 | -0.020 | 0.208 | -0.089 | 0.046 | -0.095 | -0.158 | -0.049 | -0.232 | -0.059 | -0.169 | 0.002 | -0.279 | 0.022 |
| Bleak | 1041 | 1 | 1 | 1 | -0.357 | 0.037 | -0.207 | 0.095 | 0.131 | 0.115 | 0.511 | 0.068 | 0.511 | -0.019 | 0.208 | -0.090 | 0.049 | -0.114 | -0.162 | -0.052 | -0.222 | -0.058 | -0.174 | -0.003 | -0.289 | 0.021 |
| Bleak | 1042 | 1 | 1 | 1 | -0.342 | 0.052 | -0.224 | 0.079 | 0.115 | 0.106 | 0.524 | 0.063 | 0.512 | -0.012 | 0.219 | -0.087 | 0.033 | -0.105 | -0.157 | -0.051 | -0.228 | -0.061 | -0.169 | -0.005 | -0.283 | 0.022 |
| Bleak | 1043 | 1 | 1 | 1 | -0.347 | 0.037 | -0.234 | 0.080 | 0.123 | 0.113 | 0.519 | 0.058 | 0.528 | -0.020 | 0.188 | -0.087 | 0.050 | -0.089 | -0.153 | -0.050 | -0.231 | -0.057 | -0.167 | -0.004 | -0.275 | 0.017 |
| Bleak | 1044 | 1 | 1 | 1 | -0.352 | 0.029 | -0.224 | 0.075 | 0.142 | 0.118 | 0.509 | 0.061 | 0.507 | -0.027 | 0.216 | -0.082 | 0.055 | -0.097 | -0.163 | -0.040 | -0.235 | -0.062 | -0.171 | 0.002 | -0.285 | 0.023 |
| Bleak | 1045 | 1 | 1 | 1 | -0.339 | 0.040 | -0.233 | 0.082 | 0.093 | 0.128 | 0.521 | 0.063 | 0.513 | -0.039 | 0.225 | -0.087 | 0.058 | -0.097 | -0.169 | -0.045 | -0.213 | -0.059 | -0.170 | -0.002 | -0.286 | 0.016 |
| Bleak | 1046 | 1 | 1 | 1 | -0.351 | 0.054 | -0.214 | 0.087 | 0.133 | 0.100 | 0.492 | 0.083 | 0.533 | -0.007 | 0.212 | -0.113 | 0.033 | -0.116 | -0.164 | -0.051 | -0.240 | -0.055 | -0.160 | -0.010 | -0.273 | 0.029 |
| Bleak | 1047 | 1 | 1 | 1 | -0.358 | 0.047 | -0.208 | 0.089 | 0.125 | 0.095 | 0.509 | 0.077 | 0.509 | -0.009 | 0.218 | -0.101 | 0.055 | -0.117 | -0.160 | -0.052 | -0.240 | -0.057 | -0.166 | -0.005 | -0.283 | 0.033 |
| Bleak | 1048 | 1 | 1 | 1 | -0.351 | 0.029 | -0.241 | 0.068 | 0.129 | 0.135 | 0.524 | 0.046 | 0.505 | -0.032 | 0.210 | -0.072 | 0.054 | -0.088 | -0.154 | -0.044 | -0.226 | -0.060 | -0.169 | 0.001 | -0.281 | 0.017 |
| Bleak | 1049 | 1 | 1 | 1 | -0.339 | 0.058 | -0.214 | 0.085 | 0.127 | 0.101 | 0.509 | 0.078 | 0.512 | -0.008 | 0.221 | -0.100 | 0.044 | -0.125 | -0.166 | -0.058 | -0.233 | -0.060 | -0.176 | 0.002 | -0.285 | 0.028 |
| Bleak | 1050 | 1 | 1 | 1 | -0.350 | 0.022 | -0.239 | 0.088 | 0.134 | 0.134 | 0.516 | 0.058 | 0.499 | -0.033 | 0.222 | -0.088 | 0.050 | -0.095 | -0.151 | -0.044 | -0.232 | -0.057 | -0.165 | -0.001 | -0.282 | 0.016 |
| Bleak | 1051 | 1 | 1 | 1 | -0.350 | 0.021 | -0.229 | 0.085 | 0.115 | 0.136 | 0.522 | 0.048 | 0.507 | -0.036 | 0.214 | -0.074 | 0.056 | -0.094 | -0.154 | -0.038 | -0.222 | -0.062 | -0.173 | 0.001 | -0.288 | 0.013 |
| Bleak | 1052 | 1 | 1 | 1 | -0.343 | 0.030 | -0.218 | 0.078 | 0.136 | 0.103 | 0.514 | 0.063 | 0.513 | -0.012 | 0.211 | -0.092 | 0.053 | -0.103 | -0.164 | -0.039 | -0.241 | -0.056 | -0.176 | 0.008 | -0.284 | 0.020 |
| Bleak | 1053 | 1 | 1 | 1 | -0.356 | 0.017 | -0.231 | 0.078 | 0.130 | 0.127 | 0.520 | 0.044 | 0.514 | -0.034 | 0.201 | -0.070 | 0.059 | -0.076 | -0.152 | -0.034 | -0.230 | -0.056 | -0.166 | -0.008 | -0.289 | 0.013 |
| Bleak | 1054 | 1 | 1 | 1 | -0.352 | 0.037 | -0.225 | 0.090 | 0.121 | 0.125 | 0.512 | 0.063 | 0.518 | -0.022 | 0.206 | -0.097 | 0.046 | -0.099 | -0.149 | -0.050 | -0.239 | -0.061 | -0.162 | -0.002 | -0.276 | 0.017 |
| Bleak | 1055 | 1 | 1 | 1 | -0.356 | 0.014 | -0.253 | 0.073 | 0.140 | 0.138 | 0.513 | 0.050 | 0.512 | -0.047 | 0.214 | -0.072 | 0.041 | -0.080 | -0.139 | -0.034 | -0.213 | -0.058 | -0.169 | 0.007 | -0.290 | 0.009 |
| Bleak | 1056 | 1 | 1 | 1 | -0.351 | 0.025 | -0.229 | 0.082 | 0.133 | 0.121 | 0.517 | 0.060 | 0.510 | -0.024 | 0.209 | -0.093 | 0.048 | -0.094 | -0.153 | -0.030 | -0.232 | -0.054 | -0.166 | -0.004 | -0.286 | 0.012 |
| Bleak | 1057 | 1 | 1 | 1 | -0.347 | 0.062 | -0.220 | 0.077 | 0.151 | 0.076 | 0.493 | 0.087 | 0.510 | 0.003 | 0.232 | -0.113 | 0.048 | -0.122 | -0.167 | -0.046 | -0.239 | -0.051 | -0.172 | -0.006 | -0.288 | 0.034 |
| Bleak | 1058 | 1 | 1 | 1 | -0.357 | 0.049 | -0.191 | 0.093 | 0.111 | 0.091 | 0.511 | 0.088 | 0.535 | -0.011 | 0.188 | -0.106 | 0.038 | -0.118 | -0.158 | -0.049 | -0.239 | -0.061 | -0.163 | -0.002 | -0.275 | 0.025 |
| Bleak | 1059 | 1 | 1 | 1 | -0.340 | 0.047 | -0.200 | 0.087 | 0.136 | 0.089 | 0.502 | 0.083 | 0.510 | 0.006 | 0.227 | -0.114 | 0.045 | -0.139 | -0.168 | -0.049 | -0.239 | -0.052 | -0.183 | 0.005 | -0.289 | 0.037 |
| Bleak | 1060 | 1 | 1 | 1 | -0.333 | 0.068 | -0.219 | 0.084 | 0.140 | 0.071 | 0.502 | 0.088 | 0.510 | 0.004 | 0.237 | -0.108 | 0.039 | -0.121 | -0.176 | -0.056 | -0.244 | -0.051 | -0.176 | -0.010 | -0.281 | 0.031 |
| Bleak | 1031 | 1 | 1 | 2 | -0.350 | 0.027 | -0.227 | 0.075 | 0.137 | 0.123 | 0.518 | 0.053 | 0.512 | -0.029 | 0.213 | -0.071 | 0.039 | -0.093 | -0.148 | -0.038 | -0.237 | -0.059 | -0.171 | -0.002 | -0.284 | 0.015 |
| Bleak | 1032 | 1 | 1 | 2 | -0.345 | 0.035 | -0.217 | 0.086 | 0.119 | 0.107 | 0.509 | 0.069 | 0.523 | -0.014 | 0.213 | -0.105 | 0.042 | -0.103 | -0.161 | -0.042 | -0.226 | -0.062 | -0.172 | 0.002 | -0.285 | 0.027 |
| Bleak | 1033 | 1 | 1 | 2 | -0.340 | 0.049 | -0.217 | 0.083 | 0.121 | 0.101 | 0.514 | 0.069 | 0.518 | -0.012 | 0.203 | -0.091 | 0.063 | -0.112 | -0.162 | -0.050 | -0.239 | -0.052 | -0.177 | -0.011 | -0.284 | 0.024 |
| Bleak | 1034 | 1 | 1 | 2 | -0.334 | 0.059 | -0.207 | 0.089 | 0.136 | 0.109 | 0.504 | 0.083 | 0.500 | -0.007 | 0.223 | -0.109 | 0.064 | -0.129 | -0.170 | -0.064 | -0.244 | -0.055 | -0.184 | -0.010 | -0.288 | 0.034 |
| Bleak | 1035 | 1 | 1 | 2 | -0.354 | 0.053 | -0.207 | 0.087 | 0.121 | 0.099 | 0.508 | 0.080 | 0.517 | -0.005 | 0.209 | -0.108 | 0.051 | -0.122 | -0.161 | -0.056 | -0.248 | -0.058 | -0.160 | -0.003 | -0.275 | 0.032 |
| Bleak | 1036 | 1 | 1 | 2 | -0.351 | 0.042 | -0.226 | 0.079 | 0.130 | 0.113 | 0.519 | 0.071 | 0.509 | -0.015 | 0.206 | -0.094 | 0.042 | -0.113 | -0.153 | -0.051 | -0.254 | -0.057 | -0.154 | -0.002 | -0.268 | 0.026 |
| Bleak | 1037 | 1 | 1 | 2 | -0.353 | 0.024 | -0.219 | 0.085 | 0.131 | 0.117 | 0.517 | 0.057 | 0.518 | -0.021 | 0.208 | -0.084 | 0.035 | -0.098 | -0.161 | -0.039 | -0.226 | -0.057 | -0.166 | -0.003 | -0.283 | 0.018 |
| Bleak | 1038 | 1 | 1 | 2 | -0.343 | 0.049 | -0.229 | 0.084 | 0.145 | 0.094 | 0.504 | 0.066 | 0.510 | -0.009 | 0.220 | -0.089 | 0.062 | -0.107 | -0.172 | -0.051 | -0.230 | -0.052 | -0.181 | -0.006 | -0.285 | 0.021 |
| Bleak | 1039 | 1 | 1 | 2 | -0.344 | 0.066 | -0.213 | 0.089 | 0.122 | 0.100 | 0.494 | 0.080 | 0.522 | -0.003 | 0.230 | -0.107 | 0.041 | -0.130 | -0.162 | -0.066 | -0.244 | -0.055 | -0.170 | -0.005 | -0.276 | 0.032 |
| Bleak | 1040 | 1 | 1 | 2 | -0.362 | 0.032 | -0.234 | 0.084 | 0.128 | 0.105 | 0.513 | 0.060 | 0.514 | -0.020 | 0.211 | -0.090 | 0.053 | -0.092 | -0.158 | -0.038 | -0.222 | -0.062 | -0.165 | -0.004 | -0.278 | 0.024 |
| Bleak | 1041 | 1 | 1 | 2 | -0.352 | 0.038 | -0.209 | 0.093 | 0.144 | 0.115 | 0.507 | 0.068 | 0.508 | -0.016 | 0.220 | -0.097 | 0.039 | -0.106 | -0.164 | -0.049 | -0.226 | -0.052 | -0.176 | -0.006 | -0.291 | 0.013 |
| Bleak | 1042 | 1 | 1 | 2 | -0.343 | 0.050 | -0.233 | 0.078 | 0.123 | 0.106 | 0.518 | 0.063 | 0.511 | -0.015 | 0.221 | -0.085 | 0.039 | -0.106 | -0.160 | -0.051 | -0.229 | -0.057 | -0.167 | -0.008 | -0.280 | 0.023 |
| Bleak | 1043 | 1 | 1 | 2 | -0.338 | 0.031 | -0.231 | 0.079 | 0.125 | 0.117 | 0.521 | 0.057 | 0.525 | -0.018 | 0.193 | -0.085 | 0.044 | -0.090 | -0.158 | -0.051 | -0.241 | -0.053 | -0.164 | -0.008 | -0.276 | 0.022 |
| Bleak | 1044 | 1 | 1 | 2 | -0.354 | 0.029 | -0.229 | 0.077 | 0.139 | 0.116 | 0.509 | 0.055 | 0.502 | -0.023 | 0.226 | -0.081 | 0.058 | -0.099 | -0.158 | -0.037 | -0.234 | -0.060 | -0.174 | 0.001 | -0.287 | 0.022 |
| Bleak | 1045 | 1 | 1 | 2 | -0.334 | 0.040 | -0.242 | 0.076 | 0.112 | 0.128 | 0.528 | 0.058 | 0.513 | -0.026 | 0.206 | -0.088 | 0.051 | -0.097 | -0.171 | -0.050 | -0.217 | -0.057 | -0.170 | 0.001 | -0.276 | 0.016 |
| Bleak | 1046 | 1 | 1 | 2 | -0.352 | 0.057 | -0.213 | 0.090 | 0.123 | 0.098 | 0.497 | 0.082 | 0.520 | -0.004 | 0.219 | -0.115 | 0.051 | -0.120 | -0.157 | -0.059 | -0.252 | -0.049 | -0.162 | -0.009 | -0.274 | 0.029 |
| Bleak | 1047 | 1 | 1 | 2 | -0.359 | 0.048 | -0.214 | 0.085 | 0.123 | 0.097 | 0.519 | 0.075 | 0.510 | -0.009 | 0.212 | -0.098 | 0.038 | -0.116 | -0.164 | -0.050 | -0.226 | -0.059 | -0.160 | -0.001 | -0.279 | 0.029 |
| Bleak | 1048 | 1 | 1 | 2 | -0.355 | 0.030 | -0.232 | 0.072 | 0.124 | 0.136 | 0.524 | 0.041 | 0.504 | -0.028 | 0.211 | -0.071 | 0.059 | -0.086 | -0.155 | -0.047 | -0.230 | -0.060 | -0.168 | -0.003 | -0.283 | 0.017 |
| Bleak | 1049 | 1 | 1 | 2 | -0.337 | 0.050 | -0.213 | 0.089 | 0.118 | 0.099 | 0.523 | 0.071 | 0.511 | -0.007 | 0.223 | -0.091 | 0.031 | -0.120 | -0.176 | -0.057 | -0.225 | -0.054 | -0.176 | -0.003 | -0.278 | 0.022 |
| Bleak | 1050 | 1 | 1 | 2 | -0.351 | 0.027 | -0.239 | 0.081 | 0.106 | 0.130 | 0.523 | 0.048 | 0.504 | -0.028 | 0.227 | -0.085 | 0.051 | -0.094 | -0.139 | -0.042 | -0.234 | -0.057 | -0.167 | 0.005 | -0.281 | 0.014 |
| Bleak | 1051 | 1 | 1 | 2 | -0.348 | 0.028 | -0.243 | 0.078 | 0.101 | 0.135 | 0.521 | 0.048 | 0.511 | -0.040 | 0.225 | -0.074 | 0.054 | -0.090 | -0.145 | -0.043 | -0.222 | -0.056 | -0.168 | -0.001 | -0.285 | 0.016 |
| Bleak | 1052 | 1 | 1 | 2 | -0.343 | 0.031 | -0.217 | 0.081 | 0.119 | 0.104 | 0.517 | 0.061 | 0.514 | -0.016 | 0.216 | -0.089 | 0.055 | -0.107 | -0.163 | -0.038 | -0.234 | -0.051 | -0.178 | 0.007 | -0.286 | 0.016 |
| Bleak | 1053 | 1 | 1 | 2 | -0.354 | 0.010 | -0.227 | 0.076 | 0.132 | 0.129 | 0.514 | 0.046 | 0.513 | -0.036 | 0.206 | -0.074 | 0.066 | -0.078 | -0.150 | -0.032 | -0.240 | -0.057 | -0.168 | 0.000 | -0.290 | 0.015 |
| Bleak | 1054 | 1 | 1 | 2 | -0.355 | 0.032 | -0.228 | 0.087 | 0.135 | 0.130 | 0.511 | 0.061 | 0.520 | -0.019 | 0.200 | -0.099 | 0.035 | -0.099 | -0.147 | -0.050 | -0.239 | -0.058 | -0.157 | 0.000 | -0.273 | 0.016 |
| Bleak | 1055 | 1 | 1 | 2 | -0.357 | 0.008 | -0.251 | 0.074 | 0.139 | 0.142 | 0.517 | 0.043 | 0.513 | -0.045 | 0.205 | -0.067 | 0.046 | -0.078 | -0.141 | -0.033 | -0.218 | -0.058 | -0.165 | 0.010 | -0.286 | 0.007 |
| Bleak | 1056 | 1 | 1 | 2 | -0.352 | 0.023 | -0.217 | 0.084 | 0.122 | 0.121 | 0.518 | 0.061 | 0.514 | -0.027 | 0.213 | -0.090 | 0.043 | -0.095 | -0.151 | -0.035 | -0.236 | -0.052 | -0.167 | 0.000 | -0.285 | 0.012 |
| Bleak | 1057 | 1 | 1 | 2 | -0.346 | 0.065 | -0.220 | 0.081 | 0.148 | 0.078 | 0.500 | 0.088 | 0.510 | 0.006 | 0.220 | -0.115 | 0.047 | -0.128 | -0.165 | -0.049 | -0.239 | -0.048 | -0.168 | -0.010 | -0.286 | 0.031 |
| Bleak | 1058 | 1 | 1 | 2 | -0.351 | 0.050 | -0.218 | 0.091 | 0.110 | 0.097 | 0.504 | 0.082 | 0.532 | -0.010 | 0.204 | -0.112 | 0.043 | -0.112 | -0.150 | -0.044 | -0.243 | -0.058 | -0.158 | -0.014 | -0.272 | 0.031 |
| Bleak | 1059 | 1 | 1 | 2 | -0.332 | 0.057 | -0.225 | 0.088 | 0.145 | 0.090 | 0.496 | 0.083 | 0.514 | 0.007 | 0.230 | -0.114 | 0.036 | -0.136 | -0.168 | -0.049 | -0.238 | -0.051 | -0.177 | -0.011 | -0.281 | 0.037 |
| Bleak | 1060 | 1 | 1 | 2 | -0.330 | 0.074 | -0.211 | 0.078 | 0.128 | 0.071 | 0.501 | 0.085 | 0.520 | 0.009 | 0.232 | -0.109 | 0.045 | -0.121 | -0.171 | -0.061 | -0.250 | -0.045 | -0.187 | -0.014 | -0.277 | 0.033 |
| Bleak | 1031 | 1 | 1 | 3 | -0.355 | 0.023 | -0.232 | 0.073 | 0.142 | 0.128 | 0.512 | 0.052 | 0.512 | -0.034 | 0.218 | -0.069 | 0.037 | -0.092 | -0.153 | -0.039 | -0.230 | -0.061 | -0.169 | 0.002 | -0.282 | 0.017 |
| Bleak | 1032 | 1 | 1 | 3 | -0.348 | 0.033 | -0.222 | 0.081 | 0.129 | 0.112 | 0.509 | 0.071 | 0.514 | -0.019 | 0.216 | -0.102 | 0.049 | -0.107 | -0.158 | -0.040 | -0.233 | -0.058 | -0.173 | 0.003 | -0.281 | 0.026 |
| Bleak | 1033 | 1 | 1 | 3 | -0.340 | 0.049 | -0.238 | 0.084 | 0.135 | 0.104 | 0.511 | 0.073 | 0.507 | -0.015 | 0.212 | -0.096 | 0.062 | -0.115 | -0.161 | -0.044 | -0.233 | -0.055 | -0.173 | -0.010 | -0.283 | 0.026 |
| Bleak | 1034 | 1 | 1 | 3 | -0.338 | 0.064 | -0.217 | 0.085 | 0.131 | 0.105 | 0.507 | 0.079 | 0.498 | -0.005 | 0.231 | -0.110 | 0.061 | -0.125 | -0.167 | -0.061 | -0.236 | -0.056 | -0.183 | -0.009 | -0.288 | 0.033 |
| Bleak | 1035 | 1 | 1 | 3 | -0.355 | 0.051 | -0.211 | 0.084 | 0.124 | 0.100 | 0.507 | 0.074 | 0.515 | -0.004 | 0.212 | -0.104 | 0.054 | -0.121 | -0.161 | -0.052 | -0.248 | -0.058 | -0.161 | -0.002 | -0.275 | 0.033 |
| Bleak | 1036 | 1 | 1 | 3 | -0.353 | 0.043 | -0.222 | 0.082 | 0.135 | 0.109 | 0.515 | 0.071 | 0.507 | -0.014 | 0.212 | -0.091 | 0.043 | -0.117 | -0.150 | -0.050 | -0.250 | -0.059 | -0.165 | -0.003 | -0.271 | 0.028 |
| Bleak | 1037 | 1 | 1 | 3 | -0.353 | 0.024 | -0.220 | 0.080 | 0.136 | 0.117 | 0.514 | 0.059 | 0.511 | -0.022 | 0.216 | -0.086 | 0.039 | -0.097 | -0.160 | -0.035 | -0.232 | -0.057 | -0.165 | -0.003 | -0.287 | 0.019 |
| Bleak | 1038 | 1 | 1 | 3 | -0.341 | 0.049 | -0.226 | 0.080 | 0.144 | 0.096 | 0.509 | 0.071 | 0.509 | -0.014 | 0.217 | -0.089 | 0.056 | -0.107 | -0.164 | -0.051 | -0.233 | -0.051 | -0.182 | -0.008 | -0.288 | 0.023 |
| Bleak | 1039 | 1 | 1 | 3 | -0.346 | 0.071 | -0.212 | 0.088 | 0.122 | 0.099 | 0.491 | 0.079 | 0.515 | -0.001 | 0.241 | -0.107 | 0.046 | -0.131 | -0.158 | -0.061 | -0.250 | -0.054 | -0.169 | -0.015 | -0.281 | 0.033 |
| Bleak | 1040 | 1 | 1 | 3 | -0.355 | 0.030 | -0.222 | 0.086 | 0.131 | 0.109 | 0.515 | 0.062 | 0.515 | -0.022 | 0.206 | -0.088 | 0.052 | -0.093 | -0.156 | -0.044 | -0.229 | -0.060 | -0.180 | -0.002 | -0.276 | 0.021 |
| Bleak | 1041 | 1 | 1 | 3 | -0.350 | 0.036 | -0.205 | 0.087 | 0.136 | 0.114 | 0.511 | 0.067 | 0.515 | -0.015 | 0.212 | -0.093 | 0.036 | -0.110 | -0.159 | -0.050 | -0.227 | -0.049 | -0.179 | -0.003 | -0.289 | 0.016 |
| Bleak | 1042 | 1 | 1 | 3 | -0.342 | 0.051 | -0.229 | 0.078 | 0.115 | 0.107 | 0.518 | 0.063 | 0.517 | -0.014 | 0.217 | -0.087 | 0.038 | -0.106 | -0.158 | -0.046 | -0.231 | -0.060 | -0.165 | -0.005 | -0.282 | 0.022 |
| Bleak | 1043 | 1 | 1 | 3 | -0.348 | 0.036 | -0.238 | 0.081 | 0.119 | 0.116 | 0.515 | 0.056 | 0.531 | -0.024 | 0.188 | -0.082 | 0.055 | -0.090 | -0.150 | -0.050 | -0.231 | -0.057 | -0.166 | -0.004 | -0.274 | 0.019 |
| Bleak | 1044 | 1 | 1 | 3 | -0.357 | 0.032 | -0.225 | 0.076 | 0.148 | 0.119 | 0.503 | 0.061 | 0.505 | -0.026 | 0.223 | -0.083 | 0.055 | -0.099 | -0.157 | -0.041 | -0.236 | -0.060 | -0.171 | 0.000 | -0.288 | 0.021 |
| Bleak | 1045 | 1 | 1 | 3 | -0.346 | 0.036 | -0.222 | 0.082 | 0.104 | 0.128 | 0.524 | 0.062 | 0.514 | -0.035 | 0.208 | -0.082 | 0.057 | -0.099 | -0.160 | -0.056 | -0.222 | -0.055 | -0.174 | 0.001 | -0.282 | 0.018 |
| Bleak | 1046 | 1 | 1 | 3 | -0.354 | 0.056 | -0.217 | 0.087 | 0.127 | 0.100 | 0.499 | 0.081 | 0.518 | -0.006 | 0.218 | -0.113 | 0.044 | -0.121 | -0.143 | -0.053 | -0.247 | -0.050 | -0.166 | -0.010 | -0.280 | 0.030 |
| Bleak | 1047 | 1 | 1 | 3 | -0.355 | 0.047 | -0.208 | 0.085 | 0.119 | 0.096 | 0.514 | 0.077 | 0.512 | -0.012 | 0.216 | -0.095 | 0.046 | -0.117 | -0.153 | -0.056 | -0.247 | -0.050 | -0.165 | -0.003 | -0.280 | 0.030 |
| Bleak | 1048 | 1 | 1 | 3 | -0.350 | 0.025 | -0.236 | 0.070 | 0.121 | 0.132 | 0.529 | 0.045 | 0.504 | -0.029 | 0.212 | -0.077 | 0.053 | -0.085 | -0.153 | -0.042 | -0.233 | -0.058 | -0.166 | 0.002 | -0.279 | 0.015 |
| Bleak | 1049 | 1 | 1 | 3 | -0.340 | 0.052 | -0.209 | 0.079 | 0.127 | 0.097 | 0.510 | 0.076 | 0.517 | -0.010 | 0.222 | -0.093 | 0.038 | -0.118 | -0.165 | -0.052 | -0.240 | -0.052 | -0.180 | -0.005 | -0.280 | 0.027 |
| Bleak | 1050 | 1 | 1 | 3 | -0.353 | 0.023 | -0.233 | 0.084 | 0.122 | 0.137 | 0.524 | 0.043 | 0.498 | -0.025 | 0.218 | -0.082 | 0.056 | -0.095 | -0.148 | -0.046 | -0.232 | -0.062 | -0.166 | 0.005 | -0.285 | 0.017 |
| Bleak | 1051 | 1 | 1 | 3 | -0.349 | 0.024 | -0.242 | 0.077 | 0.109 | 0.133 | 0.523 | 0.045 | 0.512 | -0.035 | 0.219 | -0.074 | 0.050 | -0.090 | -0.152 | -0.035 | -0.218 | -0.063 | -0.167 | 0.003 | -0.283 | 0.016 |
| Bleak | 1052 | 1 | 1 | 3 | -0.343 | 0.028 | -0.216 | 0.078 | 0.129 | 0.107 | 0.509 | 0.060 | 0.516 | -0.014 | 0.224 | -0.091 | 0.045 | -0.105 | -0.158 | -0.038 | -0.247 | -0.050 | -0.176 | 0.007 | -0.283 | 0.017 |
| Bleak | 1053 | 1 | 1 | 3 | -0.352 | 0.017 | -0.232 | 0.079 | 0.132 | 0.132 | 0.526 | 0.048 | 0.507 | -0.038 | 0.200 | -0.072 | 0.059 | -0.074 | -0.151 | -0.035 | -0.235 | -0.057 | -0.167 | -0.013 | -0.286 | 0.013 |
| Bleak | 1054 | 1 | 1 | 3 | -0.349 | 0.029 | -0.232 | 0.088 | 0.131 | 0.127 | 0.509 | 0.062 | 0.524 | -0.022 | 0.204 | -0.095 | 0.032 | -0.103 | -0.154 | -0.046 | -0.230 | -0.061 | -0.158 | 0.001 | -0.275 | 0.020 |
| Bleak | 1055 | 1 | 1 | 3 | -0.362 | 0.009 | -0.242 | 0.079 | 0.136 | 0.140 | 0.521 | 0.044 | 0.505 | -0.039 | 0.206 | -0.075 | 0.050 | -0.084 | -0.139 | -0.035 | -0.219 | -0.061 | -0.166 | 0.016 | -0.288 | 0.006 |
| Bleak | 1056 | 1 | 1 | 3 | -0.354 | 0.023 | -0.226 | 0.081 | 0.135 | 0.126 | 0.514 | 0.065 | 0.507 | -0.031 | 0.213 | -0.093 | 0.049 | -0.098 | -0.150 | -0.036 | -0.233 | -0.056 | -0.167 | 0.010 | -0.287 | 0.009 |
| Bleak | 1057 | 1 | 1 | 3 | -0.349 | 0.060 | -0.216 | 0.082 | 0.154 | 0.078 | 0.492 | 0.087 | 0.507 | 0.006 | 0.228 | -0.116 | 0.053 | -0.128 | -0.166 | -0.053 | -0.242 | -0.048 | -0.172 | -0.003 | -0.290 | 0.034 |
| Bleak | 1058 | 1 | 1 | 3 | -0.357 | 0.045 | -0.202 | 0.091 | 0.118 | 0.099 | 0.513 | 0.081 | 0.526 | -0.007 | 0.198 | -0.111 | 0.034 | -0.115 | -0.152 | -0.050 | -0.244 | -0.059 | -0.159 | 0.002 | -0.273 | 0.025 |
| Bleak | 1059 | 1 | 1 | 3 | -0.333 | 0.050 | -0.214 | 0.080 | 0.135 | 0.088 | 0.505 | 0.083 | 0.515 | 0.007 | 0.219 | -0.117 | 0.049 | -0.134 | -0.175 | -0.045 | -0.242 | -0.049 | -0.181 | 0.001 | -0.277 | 0.036 |
| Bleak | 1060 | 1 | 1 | 3 | -0.330 | 0.067 | -0.211 | 0.085 | 0.128 | 0.074 | 0.500 | 0.082 | 0.525 | 0.010 | 0.227 | -0.106 | 0.037 | -0.126 | -0.169 | -0.059 | -0.247 | -0.048 | -0.182 | -0.015 | -0.279 | 0.035 |
| Bleak | 1131 | 1 | 2 | 1 | -0.342 | 0.043 | -0.239 | 0.076 | 0.135 | 0.113 | 0.510 | 0.069 | 0.509 | -0.028 | 0.229 | -0.089 | 0.038 | -0.100 | -0.165 | -0.042 | -0.216 | -0.059 | -0.172 | -0.006 | -0.287 | 0.023 |
| Bleak | 1132 | 1 | 2 | 1 | -0.352 | 0.019 | -0.241 | 0.076 | 0.127 | 0.114 | 0.517 | 0.049 | 0.515 | -0.027 | 0.215 | -0.079 | 0.049 | -0.082 | -0.160 | -0.037 | -0.218 | -0.058 | -0.167 | 0.008 | -0.286 | 0.017 |
| Bleak | 1133 | 1 | 2 | 1 | -0.339 | 0.037 | -0.217 | 0.081 | 0.144 | 0.116 | 0.516 | 0.070 | 0.512 | -0.014 | 0.199 | -0.102 | 0.047 | -0.103 | -0.166 | -0.046 | -0.227 | -0.059 | -0.175 | -0.005 | -0.291 | 0.024 |
| Bleak | 1134 | 1 | 2 | 1 | -0.348 | 0.029 | -0.224 | 0.077 | 0.130 | 0.108 | 0.515 | 0.065 | 0.519 | -0.028 | 0.212 | -0.081 | 0.038 | -0.093 | -0.158 | -0.037 | -0.230 | -0.057 | -0.171 | 0.000 | -0.284 | 0.018 |
| Bleak | 1135 | 1 | 2 | 1 | -0.346 | 0.016 | -0.228 | 0.075 | 0.139 | 0.119 | 0.518 | 0.051 | 0.506 | -0.024 | 0.227 | -0.077 | 0.030 | -0.093 | -0.158 | -0.032 | -0.238 | -0.055 | -0.162 | 0.005 | -0.287 | 0.015 |
| Bleak | 1136 | 1 | 2 | 1 | -0.344 | 0.021 | -0.229 | 0.077 | 0.139 | 0.132 | 0.508 | 0.065 | 0.525 | -0.036 | 0.196 | -0.089 | 0.044 | -0.092 | -0.160 | -0.036 | -0.224 | -0.062 | -0.169 | 0.001 | -0.286 | 0.019 |
| Bleak | 1137 | 1 | 2 | 1 | -0.350 | 0.037 | -0.236 | 0.075 | 0.139 | 0.110 | 0.513 | 0.065 | 0.509 | -0.022 | 0.210 | -0.089 | 0.055 | -0.099 | -0.159 | -0.039 | -0.229 | -0.059 | -0.169 | -0.004 | -0.284 | 0.025 |
| Bleak | 1138 | 1 | 2 | 1 | -0.351 | 0.036 | -0.234 | 0.082 | 0.130 | 0.126 | 0.518 | 0.059 | 0.507 | -0.026 | 0.216 | -0.087 | 0.042 | -0.098 | -0.161 | -0.044 | -0.215 | -0.059 | -0.166 | -0.005 | -0.287 | 0.016 |
| Bleak | 1139 | 1 | 2 | 1 | -0.343 | 0.029 | -0.225 | 0.076 | 0.120 | 0.120 | 0.513 | 0.060 | 0.517 | -0.028 | 0.221 | -0.086 | 0.041 | -0.096 | -0.157 | -0.037 | -0.229 | -0.057 | -0.171 | 0.000 | -0.288 | 0.020 |
| Bleak | 1140 | 1 | 2 | 1 | -0.346 | 0.022 | -0.237 | 0.076 | 0.135 | 0.133 | 0.525 | 0.049 | 0.494 | -0.033 | 0.214 | -0.077 | 0.070 | -0.095 | -0.166 | -0.031 | -0.220 | -0.058 | -0.178 | 0.005 | -0.291 | 0.010 |
| Bleak | 1141 | 1 | 2 | 1 | -0.346 | 0.026 | -0.237 | 0.074 | 0.133 | 0.120 | 0.518 | 0.055 | 0.502 | -0.029 | 0.226 | -0.075 | 0.050 | -0.098 | -0.162 | -0.040 | -0.236 | -0.053 | -0.165 | -0.004 | -0.283 | 0.022 |
| Bleak | 1142 | 1 | 2 | 1 | -0.345 | 0.036 | -0.221 | 0.077 | 0.118 | 0.109 | 0.507 | 0.071 | 0.525 | -0.020 | 0.208 | -0.098 | 0.054 | -0.108 | -0.160 | -0.043 | -0.243 | -0.052 | -0.162 | 0.002 | -0.281 | 0.024 |
| Bleak | 1143 | 1 | 2 | 1 | -0.342 | 0.039 | -0.230 | 0.071 | 0.144 | 0.114 | 0.507 | 0.070 | 0.518 | -0.021 | 0.211 | -0.094 | 0.044 | -0.101 | -0.172 | -0.044 | -0.229 | -0.050 | -0.170 | -0.006 | -0.281 | 0.022 |
| Bleak | 1144 | 1 | 2 | 1 | -0.347 | 0.044 | -0.224 | 0.073 | 0.140 | 0.099 | 0.513 | 0.064 | 0.512 | -0.019 | 0.216 | -0.081 | 0.050 | -0.090 | -0.167 | -0.049 | -0.231 | -0.056 | -0.176 | -0.005 | -0.286 | 0.020 |
| Bleak | 1145 | 1 | 2 | 1 | -0.345 | 0.032 | -0.228 | 0.077 | 0.124 | 0.115 | 0.516 | 0.062 | 0.517 | -0.021 | 0.207 | -0.087 | 0.050 | -0.104 | -0.166 | -0.046 | -0.229 | -0.053 | -0.167 | 0.003 | -0.279 | 0.022 |
| Bleak | 1146 | 1 | 2 | 1 | -0.342 | 0.048 | -0.231 | 0.079 | 0.121 | 0.097 | 0.506 | 0.073 | 0.520 | -0.017 | 0.224 | -0.100 | 0.045 | -0.100 | -0.158 | -0.049 | -0.232 | -0.056 | -0.167 | 0.000 | -0.285 | 0.026 |
| Bleak | 1147 | 1 | 2 | 1 | -0.350 | 0.048 | -0.218 | 0.078 | 0.128 | 0.098 | 0.501 | 0.068 | 0.516 | -0.013 | 0.214 | -0.102 | 0.075 | -0.099 | -0.166 | -0.047 | -0.224 | -0.057 | -0.178 | -0.004 | -0.298 | 0.030 |
| Bleak | 1148 | 1 | 2 | 1 | -0.350 | 0.022 | -0.232 | 0.078 | 0.135 | 0.121 | 0.510 | 0.052 | 0.502 | -0.033 | 0.237 | -0.082 | 0.051 | -0.079 | -0.160 | -0.037 | -0.227 | -0.062 | -0.176 | 0.004 | -0.292 | 0.017 |
| Bleak | 1149 | 1 | 2 | 1 | -0.352 | 0.036 | -0.223 | 0.082 | 0.151 | 0.114 | 0.509 | 0.070 | 0.499 | -0.018 | 0.225 | -0.095 | 0.047 | -0.109 | -0.173 | -0.046 | -0.229 | -0.056 | -0.169 | -0.004 | -0.284 | 0.026 |
| Bleak | 1150 | 1 | 2 | 1 | -0.344 | 0.028 | -0.224 | 0.072 | 0.124 | 0.109 | 0.524 | 0.060 | 0.517 | -0.023 | 0.209 | -0.084 | 0.041 | -0.089 | -0.162 | -0.039 | -0.229 | -0.055 | -0.174 | 0.002 | -0.281 | 0.019 |
| Bleak | 1151 | 1 | 2 | 1 | -0.340 | 0.051 | -0.215 | 0.080 | 0.141 | 0.084 | 0.505 | 0.081 | 0.514 | -0.010 | 0.226 | -0.099 | 0.043 | -0.106 | -0.175 | -0.049 | -0.232 | -0.052 | -0.177 | -0.007 | -0.288 | 0.027 |
| Bleak | 1152 | 1 | 2 | 1 | -0.338 | 0.031 | -0.237 | 0.072 | 0.136 | 0.122 | 0.519 | 0.052 | 0.507 | -0.022 | 0.220 | -0.075 | 0.048 | -0.096 | -0.172 | -0.047 | -0.233 | -0.055 | -0.169 | -0.006 | -0.280 | 0.026 |
| Bleak | 1153 | 1 | 2 | 1 | -0.349 | 0.038 | -0.238 | 0.080 | 0.134 | 0.105 | 0.510 | 0.066 | 0.517 | -0.017 | 0.215 | -0.092 | 0.036 | -0.103 | -0.150 | -0.037 | -0.224 | -0.057 | -0.169 | -0.004 | -0.284 | 0.022 |
| Bleak | 1154 | 1 | 2 | 1 | -0.344 | 0.033 | -0.222 | 0.076 | 0.114 | 0.116 | 0.522 | 0.065 | 0.510 | -0.026 | 0.214 | -0.092 | 0.056 | -0.101 | -0.161 | -0.040 | -0.225 | -0.059 | -0.175 | 0.007 | -0.288 | 0.021 |
| Bleak | 1155 | 1 | 2 | 1 | -0.345 | 0.036 | -0.226 | 0.080 | 0.123 | 0.119 | 0.511 | 0.062 | 0.516 | -0.024 | 0.222 | -0.090 | 0.044 | -0.099 | -0.164 | -0.045 | -0.218 | -0.054 | -0.174 | -0.005 | -0.288 | 0.022 |
| Bleak | 1156 | 1 | 2 | 1 | -0.357 | 0.029 | -0.230 | 0.076 | 0.123 | 0.114 | 0.515 | 0.061 | 0.508 | -0.030 | 0.227 | -0.085 | 0.044 | -0.092 | -0.157 | -0.035 | -0.228 | -0.061 | -0.165 | 0.005 | -0.280 | 0.019 |
| Bleak | 1157 | 1 | 2 | 1 | -0.345 | 0.044 | -0.218 | 0.083 | 0.140 | 0.094 | 0.491 | 0.072 | 0.531 | -0.001 | 0.216 | -0.107 | 0.037 | -0.110 | -0.164 | -0.053 | -0.237 | -0.051 | -0.168 | -0.002 | -0.284 | 0.031 |
| Bleak | 1158 | 1 | 2 | 1 | -0.359 | 0.032 | -0.240 | 0.077 | 0.149 | 0.123 | 0.502 | 0.059 | 0.494 | -0.022 | 0.235 | -0.093 | 0.055 | -0.104 | -0.155 | -0.042 | -0.239 | -0.060 | -0.156 | 0.005 | -0.287 | 0.025 |
| Bleak | 1159 | 1 | 2 | 1 | -0.349 | 0.030 | -0.232 | 0.068 | 0.130 | 0.102 | 0.517 | 0.059 | 0.507 | -0.024 | 0.230 | -0.080 | 0.045 | -0.088 | -0.159 | -0.035 | -0.244 | -0.057 | -0.164 | -0.001 | -0.280 | 0.025 |
| Bleak | 1160 | 1 | 2 | 1 | -0.345 | 0.041 | -0.225 | 0.078 | 0.135 | 0.116 | 0.507 | 0.068 | 0.513 | -0.019 | 0.222 | -0.094 | 0.039 | -0.110 | -0.163 | -0.044 | -0.232 | -0.056 | -0.165 | 0.001 | -0.285 | 0.019 |
| Bleak | 1131 | 1 | 2 | 2 | -0.345 | 0.044 | -0.228 | 0.078 | 0.141 | 0.119 | 0.511 | 0.067 | 0.503 | -0.021 | 0.226 | -0.092 | 0.042 | -0.102 | -0.162 | -0.045 | -0.236 | -0.060 | -0.166 | -0.007 | -0.286 | 0.020 |
| Bleak | 1132 | 1 | 2 | 2 | -0.346 | 0.022 | -0.244 | 0.076 | 0.122 | 0.115 | 0.518 | 0.050 | 0.514 | -0.031 | 0.220 | -0.078 | 0.049 | -0.080 | -0.157 | -0.036 | -0.225 | -0.059 | -0.165 | 0.007 | -0.284 | 0.015 |
| Bleak | 1133 | 1 | 2 | 2 | -0.348 | 0.037 | -0.214 | 0.084 | 0.153 | 0.119 | 0.502 | 0.067 | 0.504 | -0.014 | 0.218 | -0.100 | 0.056 | -0.108 | -0.173 | -0.044 | -0.231 | -0.057 | -0.172 | -0.009 | -0.294 | 0.025 |
| Bleak | 1134 | 1 | 2 | 2 | -0.349 | 0.032 | -0.223 | 0.078 | 0.127 | 0.116 | 0.506 | 0.063 | 0.519 | -0.029 | 0.225 | -0.083 | 0.039 | -0.095 | -0.159 | -0.041 | -0.233 | -0.057 | -0.170 | -0.004 | -0.284 | 0.020 |
| Bleak | 1135 | 1 | 2 | 2 | -0.345 | 0.022 | -0.235 | 0.070 | 0.140 | 0.121 | 0.514 | 0.048 | 0.507 | -0.020 | 0.232 | -0.075 | 0.031 | -0.099 | -0.159 | -0.040 | -0.233 | -0.054 | -0.169 | 0.005 | -0.284 | 0.023 |
| Bleak | 1136 | 1 | 2 | 2 | -0.339 | 0.025 | -0.238 | 0.072 | 0.125 | 0.128 | 0.516 | 0.061 | 0.526 | -0.035 | 0.199 | -0.084 | 0.041 | -0.090 | -0.155 | -0.038 | -0.221 | -0.058 | -0.171 | 0.000 | -0.284 | 0.021 |
| Bleak | 1137 | 1 | 2 | 2 | -0.347 | 0.037 | -0.239 | 0.073 | 0.137 | 0.112 | 0.511 | 0.066 | 0.518 | -0.027 | 0.208 | -0.086 | 0.048 | -0.093 | -0.159 | -0.042 | -0.226 | -0.057 | -0.170 | -0.002 | -0.281 | 0.020 |
| Bleak | 1138 | 1 | 2 | 2 | -0.351 | 0.033 | -0.231 | 0.084 | 0.129 | 0.131 | 0.513 | 0.056 | 0.507 | -0.028 | 0.223 | -0.086 | 0.044 | -0.096 | -0.154 | -0.046 | -0.223 | -0.055 | -0.175 | -0.010 | -0.284 | 0.017 |
| Bleak | 1139 | 1 | 2 | 2 | -0.343 | 0.028 | -0.231 | 0.070 | 0.126 | 0.129 | 0.508 | 0.061 | 0.515 | -0.033 | 0.230 | -0.088 | 0.038 | -0.097 | -0.155 | -0.034 | -0.233 | -0.052 | -0.168 | -0.006 | -0.286 | 0.021 |
| Bleak | 1140 | 1 | 2 | 2 | -0.345 | 0.019 | -0.240 | 0.078 | 0.134 | 0.139 | 0.525 | 0.049 | 0.495 | -0.036 | 0.215 | -0.079 | 0.062 | -0.088 | -0.162 | -0.038 | -0.217 | -0.055 | -0.174 | -0.002 | -0.294 | 0.014 |
| Bleak | 1141 | 1 | 2 | 2 | -0.345 | 0.029 | -0.243 | 0.072 | 0.132 | 0.121 | 0.523 | 0.055 | 0.511 | -0.028 | 0.206 | -0.076 | 0.051 | -0.093 | -0.161 | -0.039 | -0.229 | -0.053 | -0.168 | -0.009 | -0.278 | 0.021 |
| Bleak | 1142 | 1 | 2 | 2 | -0.343 | 0.042 | -0.221 | 0.076 | 0.118 | 0.111 | 0.513 | 0.066 | 0.523 | -0.017 | 0.208 | -0.095 | 0.047 | -0.103 | -0.161 | -0.041 | -0.236 | -0.057 | -0.164 | -0.005 | -0.284 | 0.023 |
| Bleak | 1143 | 1 | 2 | 2 | -0.338 | 0.036 | -0.221 | 0.079 | 0.110 | 0.117 | 0.511 | 0.065 | 0.533 | -0.022 | 0.210 | -0.097 | 0.037 | -0.100 | -0.164 | -0.044 | -0.222 | -0.054 | -0.173 | -0.001 | -0.283 | 0.021 |
| Bleak | 1144 | 1 | 2 | 2 | -0.348 | 0.044 | -0.221 | 0.074 | 0.122 | 0.099 | 0.520 | 0.059 | 0.513 | -0.018 | 0.214 | -0.082 | 0.055 | -0.085 | -0.161 | -0.051 | -0.227 | -0.058 | -0.180 | -0.004 | -0.287 | 0.022 |
| Bleak | 1145 | 1 | 2 | 2 | -0.345 | 0.030 | -0.222 | 0.076 | 0.139 | 0.115 | 0.519 | 0.065 | 0.513 | -0.023 | 0.203 | -0.083 | 0.041 | -0.104 | -0.165 | -0.041 | -0.228 | -0.057 | -0.169 | 0.003 | -0.287 | 0.020 |
| Bleak | 1146 | 1 | 2 | 2 | -0.345 | 0.045 | -0.206 | 0.079 | 0.120 | 0.099 | 0.509 | 0.071 | 0.514 | -0.012 | 0.230 | -0.105 | 0.041 | -0.096 | -0.166 | -0.045 | -0.235 | -0.056 | -0.168 | -0.003 | -0.294 | 0.023 |
| Bleak | 1147 | 1 | 2 | 2 | -0.343 | 0.051 | -0.228 | 0.078 | 0.122 | 0.106 | 0.512 | 0.066 | 0.510 | -0.011 | 0.219 | -0.102 | 0.061 | -0.101 | -0.163 | -0.049 | -0.226 | -0.058 | -0.173 | -0.009 | -0.290 | 0.030 |
| Bleak | 1148 | 1 | 2 | 2 | -0.350 | 0.019 | -0.218 | 0.080 | 0.130 | 0.128 | 0.524 | 0.056 | 0.500 | -0.036 | 0.222 | -0.080 | 0.046 | -0.083 | -0.157 | -0.038 | -0.232 | -0.065 | -0.171 | 0.002 | -0.292 | 0.016 |
| Bleak | 1149 | 1 | 2 | 2 | -0.351 | 0.034 | -0.224 | 0.084 | 0.140 | 0.114 | 0.507 | 0.066 | 0.501 | -0.018 | 0.229 | -0.096 | 0.055 | -0.107 | -0.173 | -0.044 | -0.231 | -0.053 | -0.169 | -0.005 | -0.283 | 0.025 |
| Bleak | 1150 | 1 | 2 | 2 | -0.345 | 0.027 | -0.222 | 0.076 | 0.118 | 0.107 | 0.526 | 0.066 | 0.517 | -0.031 | 0.211 | -0.080 | 0.040 | -0.088 | -0.164 | -0.039 | -0.229 | -0.055 | -0.171 | 0.000 | -0.280 | 0.018 |
| Bleak | 1151 | 1 | 2 | 2 | -0.338 | 0.055 | -0.219 | 0.078 | 0.130 | 0.082 | 0.504 | 0.074 | 0.518 | -0.005 | 0.228 | -0.097 | 0.048 | -0.108 | -0.172 | -0.046 | -0.232 | -0.053 | -0.178 | -0.007 | -0.289 | 0.027 |
| Bleak | 1152 | 1 | 2 | 2 | -0.339 | 0.032 | -0.230 | 0.077 | 0.120 | 0.123 | 0.523 | 0.056 | 0.506 | -0.029 | 0.225 | -0.074 | 0.047 | -0.096 | -0.168 | -0.047 | -0.232 | -0.057 | -0.168 | -0.008 | -0.284 | 0.024 |
| Bleak | 1153 | 1 | 2 | 2 | -0.351 | 0.037 | -0.223 | 0.082 | 0.117 | 0.109 | 0.514 | 0.064 | 0.518 | -0.019 | 0.216 | -0.093 | 0.042 | -0.100 | -0.156 | -0.042 | -0.221 | -0.055 | -0.170 | -0.003 | -0.287 | 0.021 |
| Bleak | 1154 | 1 | 2 | 2 | -0.342 | 0.034 | -0.240 | 0.076 | 0.122 | 0.121 | 0.510 | 0.058 | 0.518 | -0.026 | 0.215 | -0.088 | 0.055 | -0.102 | -0.156 | -0.038 | -0.222 | -0.056 | -0.174 | 0.002 | -0.286 | 0.019 |
| Bleak | 1155 | 1 | 2 | 2 | -0.343 | 0.030 | -0.230 | 0.078 | 0.132 | 0.115 | 0.516 | 0.063 | 0.511 | -0.023 | 0.223 | -0.089 | 0.034 | -0.097 | -0.158 | -0.043 | -0.220 | -0.053 | -0.178 | -0.003 | -0.287 | 0.024 |
| Bleak | 1156 | 1 | 2 | 2 | -0.355 | 0.027 | -0.228 | 0.075 | 0.137 | 0.116 | 0.512 | 0.057 | 0.506 | -0.025 | 0.230 | -0.086 | 0.042 | -0.088 | -0.158 | -0.038 | -0.234 | -0.054 | -0.170 | -0.001 | -0.282 | 0.017 |
| Bleak | 1157 | 1 | 2 | 2 | -0.344 | 0.050 | -0.219 | 0.085 | 0.133 | 0.107 | 0.503 | 0.071 | 0.518 | -0.004 | 0.215 | -0.112 | 0.042 | -0.113 | -0.161 | -0.047 | -0.227 | -0.053 | -0.173 | -0.011 | -0.287 | 0.026 |
| Bleak | 1158 | 1 | 2 | 2 | -0.362 | 0.040 | -0.233 | 0.079 | 0.145 | 0.130 | 0.490 | 0.058 | 0.499 | -0.026 | 0.238 | -0.095 | 0.071 | -0.102 | -0.165 | -0.041 | -0.224 | -0.069 | -0.165 | 0.000 | -0.295 | 0.027 |
| Bleak | 1159 | 1 | 2 | 2 | -0.348 | 0.031 | -0.232 | 0.063 | 0.137 | 0.103 | 0.517 | 0.055 | 0.505 | -0.019 | 0.234 | -0.079 | 0.039 | -0.086 | -0.155 | -0.036 | -0.242 | -0.057 | -0.172 | -0.003 | -0.282 | 0.027 |
| Bleak | 1160 | 1 | 2 | 2 | -0.341 | 0.042 | -0.237 | 0.074 | 0.127 | 0.113 | 0.510 | 0.061 | 0.517 | -0.015 | 0.222 | -0.092 | 0.041 | -0.106 | -0.163 | -0.037 | -0.228 | -0.053 | -0.166 | -0.005 | -0.282 | 0.021 |
| Bleak | 1131 | 1 | 2 | 3 | -0.342 | 0.040 | -0.225 | 0.079 | 0.136 | 0.119 | 0.508 | 0.068 | 0.504 | -0.024 | 0.230 | -0.097 | 0.050 | -0.097 | -0.165 | -0.047 | -0.234 | -0.056 | -0.175 | -0.007 | -0.287 | 0.021 |
| Bleak | 1132 | 1 | 2 | 3 | -0.352 | 0.020 | -0.245 | 0.077 | 0.122 | 0.115 | 0.515 | 0.049 | 0.513 | -0.029 | 0.221 | -0.081 | 0.054 | -0.084 | -0.156 | -0.036 | -0.219 | -0.055 | -0.166 | 0.006 | -0.287 | 0.019 |
| Bleak | 1133 | 1 | 2 | 3 | -0.346 | 0.037 | -0.225 | 0.081 | 0.148 | 0.113 | 0.504 | 0.058 | 0.511 | -0.009 | 0.213 | -0.097 | 0.055 | -0.100 | -0.161 | -0.043 | -0.233 | -0.056 | -0.174 | -0.007 | -0.292 | 0.023 |
| Bleak | 1134 | 1 | 2 | 3 | -0.348 | 0.032 | -0.229 | 0.077 | 0.127 | 0.116 | 0.517 | 0.058 | 0.516 | -0.025 | 0.215 | -0.081 | 0.039 | -0.094 | -0.157 | -0.038 | -0.223 | -0.056 | -0.171 | -0.006 | -0.286 | 0.017 |
| Bleak | 1135 | 1 | 2 | 3 | -0.349 | 0.020 | -0.240 | 0.068 | 0.130 | 0.122 | 0.512 | 0.052 | 0.506 | -0.031 | 0.234 | -0.081 | 0.050 | -0.089 | -0.162 | -0.039 | -0.229 | -0.050 | -0.166 | 0.010 | -0.287 | 0.018 |
| Bleak | 1136 | 1 | 2 | 3 | -0.341 | 0.022 | -0.231 | 0.076 | 0.130 | 0.131 | 0.518 | 0.060 | 0.520 | -0.034 | 0.201 | -0.084 | 0.040 | -0.088 | -0.159 | -0.043 | -0.226 | -0.058 | -0.167 | -0.004 | -0.285 | 0.021 |
| Bleak | 1137 | 1 | 2 | 3 | -0.344 | 0.041 | -0.251 | 0.073 | 0.134 | 0.115 | 0.512 | 0.061 | 0.510 | -0.025 | 0.218 | -0.089 | 0.052 | -0.093 | -0.157 | -0.043 | -0.223 | -0.057 | -0.171 | -0.006 | -0.282 | 0.022 |
| Bleak | 1138 | 1 | 2 | 3 | -0.347 | 0.038 | -0.241 | 0.078 | 0.118 | 0.130 | 0.518 | 0.057 | 0.510 | -0.027 | 0.217 | -0.090 | 0.046 | -0.100 | -0.154 | -0.045 | -0.218 | -0.053 | -0.166 | -0.006 | -0.284 | 0.018 |
| Bleak | 1139 | 1 | 2 | 3 | -0.341 | 0.030 | -0.246 | 0.080 | 0.114 | 0.127 | 0.514 | 0.056 | 0.510 | -0.032 | 0.227 | -0.087 | 0.054 | -0.100 | -0.151 | -0.037 | -0.223 | -0.057 | -0.171 | 0.000 | -0.287 | 0.021 |
| Bleak | 1140 | 1 | 2 | 3 | -0.342 | 0.020 | -0.250 | 0.071 | 0.128 | 0.142 | 0.532 | 0.044 | 0.497 | -0.036 | 0.212 | -0.075 | 0.059 | -0.088 | -0.163 | -0.036 | -0.213 | -0.054 | -0.172 | 0.003 | -0.288 | 0.010 |
| Bleak | 1141 | 1 | 2 | 3 | -0.344 | 0.030 | -0.244 | 0.073 | 0.131 | 0.121 | 0.515 | 0.052 | 0.507 | -0.027 | 0.232 | -0.075 | 0.043 | -0.098 | -0.164 | -0.040 | -0.229 | -0.054 | -0.167 | -0.005 | -0.279 | 0.021 |
| Bleak | 1142 | 1 | 2 | 3 | -0.347 | 0.031 | -0.211 | 0.077 | 0.120 | 0.112 | 0.510 | 0.067 | 0.520 | -0.021 | 0.210 | -0.092 | 0.055 | -0.107 | -0.164 | -0.044 | -0.242 | -0.051 | -0.168 | 0.001 | -0.285 | 0.026 |
| Bleak | 1143 | 1 | 2 | 3 | -0.334 | 0.039 | -0.231 | 0.069 | 0.127 | 0.115 | 0.517 | 0.069 | 0.520 | -0.020 | 0.208 | -0.097 | 0.040 | -0.099 | -0.165 | -0.047 | -0.237 | -0.049 | -0.166 | -0.002 | -0.279 | 0.021 |
| Bleak | 1144 | 1 | 2 | 3 | -0.343 | 0.041 | -0.233 | 0.073 | 0.135 | 0.102 | 0.518 | 0.064 | 0.512 | -0.024 | 0.214 | -0.078 | 0.051 | -0.089 | -0.166 | -0.050 | -0.232 | -0.048 | -0.173 | -0.012 | -0.282 | 0.020 |
| Bleak | 1145 | 1 | 2 | 3 | -0.342 | 0.036 | -0.225 | 0.077 | 0.132 | 0.118 | 0.522 | 0.060 | 0.510 | -0.015 | 0.206 | -0.089 | 0.046 | -0.103 | -0.164 | -0.046 | -0.227 | -0.051 | -0.173 | -0.008 | -0.284 | 0.022 |
| Bleak | 1146 | 1 | 2 | 3 | -0.344 | 0.044 | -0.228 | 0.079 | 0.117 | 0.098 | 0.507 | 0.067 | 0.528 | -0.014 | 0.213 | -0.097 | 0.045 | -0.099 | -0.162 | -0.050 | -0.221 | -0.056 | -0.169 | -0.001 | -0.286 | 0.028 |
| Bleak | 1147 | 1 | 2 | 3 | -0.350 | 0.046 | -0.225 | 0.076 | 0.121 | 0.106 | 0.512 | 0.067 | 0.508 | -0.016 | 0.217 | -0.101 | 0.066 | -0.100 | -0.160 | -0.044 | -0.225 | -0.058 | -0.173 | -0.008 | -0.293 | 0.032 |
| Bleak | 1148 | 1 | 2 | 3 | -0.350 | 0.019 | -0.228 | 0.074 | 0.126 | 0.130 | 0.523 | 0.047 | 0.503 | -0.034 | 0.227 | -0.073 | 0.043 | -0.083 | -0.155 | -0.037 | -0.233 | -0.060 | -0.170 | 0.002 | -0.285 | 0.014 |
| Bleak | 1149 | 1 | 2 | 3 | -0.351 | 0.037 | -0.234 | 0.083 | 0.125 | 0.112 | 0.508 | 0.060 | 0.501 | -0.018 | 0.231 | -0.094 | 0.071 | -0.104 | -0.170 | -0.041 | -0.229 | -0.054 | -0.168 | -0.008 | -0.286 | 0.028 |
| Bleak | 1150 | 1 | 2 | 3 | -0.340 | 0.028 | -0.225 | 0.077 | 0.122 | 0.105 | 0.528 | 0.060 | 0.517 | -0.020 | 0.210 | -0.084 | 0.034 | -0.087 | -0.161 | -0.040 | -0.225 | -0.051 | -0.178 | -0.009 | -0.282 | 0.022 |
| Bleak | 1151 | 1 | 2 | 3 | -0.340 | 0.050 | -0.214 | 0.081 | 0.129 | 0.086 | 0.501 | 0.073 | 0.514 | -0.008 | 0.231 | -0.098 | 0.064 | -0.107 | -0.173 | -0.046 | -0.239 | -0.047 | -0.184 | -0.012 | -0.290 | 0.028 |
| Bleak | 1152 | 1 | 2 | 3 | -0.338 | 0.029 | -0.229 | 0.073 | 0.124 | 0.122 | 0.526 | 0.056 | 0.504 | -0.029 | 0.221 | -0.074 | 0.048 | -0.094 | -0.174 | -0.046 | -0.225 | -0.056 | -0.173 | -0.009 | -0.286 | 0.028 |
| Bleak | 1153 | 1 | 2 | 3 | -0.347 | 0.037 | -0.246 | 0.079 | 0.128 | 0.108 | 0.513 | 0.062 | 0.516 | -0.018 | 0.216 | -0.093 | 0.040 | -0.099 | -0.150 | -0.039 | -0.224 | -0.056 | -0.165 | -0.004 | -0.281 | 0.023 |
| Bleak | 1154 | 1 | 2 | 3 | -0.340 | 0.034 | -0.233 | 0.076 | 0.123 | 0.122 | 0.508 | 0.062 | 0.515 | -0.027 | 0.218 | -0.088 | 0.060 | -0.105 | -0.164 | -0.039 | -0.229 | -0.056 | -0.172 | -0.001 | -0.286 | 0.022 |
| Bleak | 1155 | 1 | 2 | 3 | -0.343 | 0.038 | -0.225 | 0.080 | 0.116 | 0.119 | 0.509 | 0.064 | 0.516 | -0.026 | 0.228 | -0.090 | 0.045 | -0.104 | -0.164 | -0.044 | -0.217 | -0.055 | -0.179 | -0.005 | -0.287 | 0.023 |
| Bleak | 1156 | 1 | 2 | 3 | -0.355 | 0.026 | -0.238 | 0.074 | 0.135 | 0.116 | 0.508 | 0.056 | 0.513 | -0.028 | 0.223 | -0.081 | 0.049 | -0.089 | -0.158 | -0.038 | -0.230 | -0.054 | -0.166 | -0.002 | -0.280 | 0.019 |
| Bleak | 1157 | 1 | 2 | 3 | -0.344 | 0.046 | -0.217 | 0.082 | 0.130 | 0.100 | 0.500 | 0.071 | 0.522 | -0.001 | 0.220 | -0.112 | 0.042 | -0.113 | -0.166 | -0.051 | -0.232 | -0.050 | -0.168 | -0.004 | -0.287 | 0.031 |
| Bleak | 1158 | 1 | 2 | 3 | -0.356 | 0.035 | -0.243 | 0.076 | 0.143 | 0.131 | 0.497 | 0.059 | 0.493 | -0.025 | 0.242 | -0.097 | 0.065 | -0.106 | -0.156 | -0.041 | -0.235 | -0.060 | -0.159 | -0.004 | -0.290 | 0.032 |
| Bleak | 1159 | 1 | 2 | 3 | -0.350 | 0.033 | -0.241 | 0.060 | 0.139 | 0.108 | 0.519 | 0.055 | 0.501 | -0.022 | 0.237 | -0.082 | 0.033 | -0.082 | -0.153 | -0.035 | -0.230 | -0.058 | -0.172 | -0.007 | -0.284 | 0.031 |
| Bleak | 1160 | 1 | 2 | 3 | -0.343 | 0.040 | -0.239 | 0.075 | 0.130 | 0.116 | 0.509 | 0.067 | 0.514 | -0.020 | 0.218 | -0.091 | 0.042 | -0.118 | -0.157 | -0.038 | -0.227 | -0.052 | -0.164 | -0.001 | -0.284 | 0.022 |
| Bleak | 1221 | 1 | 3 | 1 | -0.353 | 0.046 | -0.209 | 0.092 | 0.126 | 0.121 | 0.507 | 0.072 | 0.518 | -0.025 | 0.223 | -0.090 | 0.019 | -0.107 | -0.156 | -0.055 | -0.234 | -0.069 | -0.160 | -0.007 | -0.280 | 0.022 |
| Bleak | 1222 | 1 | 3 | 1 | -0.352 | 0.044 | -0.233 | 0.076 | 0.137 | 0.114 | 0.515 | 0.056 | 0.503 | -0.028 | 0.224 | -0.070 | 0.053 | -0.096 | -0.161 | -0.043 | -0.221 | -0.059 | -0.177 | -0.013 | -0.288 | 0.018 |
| Bleak | 1223 | 1 | 3 | 1 | -0.351 | 0.020 | -0.234 | 0.073 | 0.128 | 0.122 | 0.514 | 0.042 | 0.519 | -0.036 | 0.215 | -0.063 | 0.051 | -0.074 | -0.150 | -0.035 | -0.224 | -0.057 | -0.175 | -0.006 | -0.293 | 0.013 |
| Bleak | 1224 | 1 | 3 | 1 | -0.344 | 0.053 | -0.218 | 0.083 | 0.130 | 0.092 | 0.507 | 0.080 | 0.520 | -0.008 | 0.213 | -0.107 | 0.041 | -0.108 | -0.162 | -0.045 | -0.229 | -0.063 | -0.169 | -0.006 | -0.288 | 0.030 |
| Bleak | 1225 | 1 | 3 | 1 | -0.343 | 0.074 | -0.198 | 0.076 | 0.094 | 0.079 | 0.513 | 0.079 | 0.521 | -0.002 | 0.233 | -0.098 | 0.043 | -0.121 | -0.169 | -0.050 | -0.233 | -0.055 | -0.172 | -0.016 | -0.288 | 0.034 |
| Bleak | 1226 | 1 | 3 | 1 | -0.341 | 0.044 | -0.215 | 0.080 | 0.136 | 0.105 | 0.510 | 0.071 | 0.504 | -0.015 | 0.229 | -0.098 | 0.046 | -0.106 | -0.158 | -0.048 | -0.243 | -0.055 | -0.179 | 0.000 | -0.289 | 0.021 |
| Bleak | 1227 | 1 | 3 | 1 | -0.347 | 0.047 | -0.218 | 0.075 | 0.136 | 0.110 | 0.504 | 0.070 | 0.509 | -0.019 | 0.235 | -0.092 | 0.033 | -0.107 | -0.154 | -0.045 | -0.232 | -0.064 | -0.175 | 0.000 | -0.291 | 0.027 |
| Bleak | 1228 | 1 | 3 | 1 | -0.355 | 0.026 | -0.240 | 0.072 | 0.126 | 0.147 | 0.524 | 0.037 | 0.507 | -0.048 | 0.213 | -0.053 | 0.050 | -0.073 | -0.147 | -0.045 | -0.226 | -0.062 | -0.166 | -0.008 | -0.285 | 0.007 |
| Bleak | 1229 | 1 | 3 | 1 | -0.350 | 0.052 | -0.215 | 0.082 | 0.111 | 0.112 | 0.518 | 0.064 | 0.511 | -0.021 | 0.216 | -0.080 | 0.050 | -0.105 | -0.153 | -0.057 | -0.234 | -0.069 | -0.168 | -0.015 | -0.286 | 0.035 |
| Bleak | 1230 | 1 | 3 | 1 | -0.355 | 0.053 | -0.241 | 0.080 | 0.139 | 0.115 | 0.515 | 0.066 | 0.512 | -0.019 | 0.204 | -0.092 | 0.035 | -0.098 | -0.149 | -0.050 | -0.211 | -0.066 | -0.162 | -0.012 | -0.287 | 0.023 |
| Bleak | 1232 | 1 | 3 | 1 | -0.356 | 0.040 | -0.215 | 0.073 | 0.141 | 0.116 | 0.511 | 0.068 | 0.507 | -0.022 | 0.213 | -0.092 | 0.052 | -0.099 | -0.164 | -0.039 | -0.230 | -0.058 | -0.166 | -0.014 | -0.292 | 0.026 |
| Bleak | 1233 | 1 | 3 | 1 | -0.350 | 0.036 | -0.222 | 0.079 | 0.138 | 0.119 | 0.505 | 0.061 | 0.520 | -0.027 | 0.213 | -0.086 | 0.046 | -0.088 | -0.159 | -0.043 | -0.229 | -0.061 | -0.174 | -0.001 | -0.287 | 0.014 |
| Bleak | 1234 | 1 | 3 | 1 | -0.355 | 0.034 | -0.225 | 0.080 | 0.155 | 0.124 | 0.511 | 0.068 | 0.506 | -0.026 | 0.211 | -0.094 | 0.034 | -0.093 | -0.157 | -0.046 | -0.227 | -0.058 | -0.170 | -0.005 | -0.284 | 0.015 |
| Bleak | 1235 | 1 | 3 | 1 | -0.345 | 0.060 | -0.205 | 0.084 | 0.108 | 0.093 | 0.514 | 0.070 | 0.511 | -0.011 | 0.234 | -0.087 | 0.048 | -0.110 | -0.173 | -0.058 | -0.239 | -0.059 | -0.168 | -0.014 | -0.285 | 0.031 |
| Bleak | 1236 | 1 | 3 | 1 | -0.357 | 0.029 | -0.226 | 0.085 | 0.118 | 0.121 | 0.523 | 0.053 | 0.516 | -0.030 | 0.211 | -0.077 | 0.035 | -0.083 | -0.143 | -0.050 | -0.224 | -0.054 | -0.165 | -0.003 | -0.286 | 0.009 |
| Bleak | 1237 | 1 | 3 | 1 | -0.360 | 0.023 | -0.217 | 0.077 | 0.141 | 0.132 | 0.526 | 0.050 | 0.498 | -0.027 | 0.206 | -0.077 | 0.048 | -0.091 | -0.158 | -0.042 | -0.224 | -0.065 | -0.170 | 0.003 | -0.291 | 0.018 |
| Bleak | 1238 | 1 | 3 | 1 | -0.348 | 0.030 | -0.224 | 0.086 | 0.113 | 0.130 | 0.524 | 0.056 | 0.495 | -0.026 | 0.234 | -0.090 | 0.050 | -0.098 | -0.157 | -0.044 | -0.236 | -0.057 | -0.169 | -0.007 | -0.282 | 0.020 |
| Bleak | 1239 | 1 | 3 | 1 | -0.354 | 0.046 | -0.227 | 0.080 | 0.141 | 0.113 | 0.507 | 0.061 | 0.499 | -0.020 | 0.227 | -0.082 | 0.060 | -0.108 | -0.161 | -0.044 | -0.229 | -0.059 | -0.171 | -0.009 | -0.292 | 0.022 |
| Bleak | 1240 | 1 | 3 | 1 | -0.350 | 0.028 | -0.227 | 0.074 | 0.135 | 0.125 | 0.515 | 0.051 | 0.512 | -0.038 | 0.214 | -0.069 | 0.051 | -0.077 | -0.148 | -0.044 | -0.229 | -0.057 | -0.179 | -0.002 | -0.293 | 0.010 |
| Bleak | 1241 | 1 | 3 | 1 | -0.347 | 0.034 | -0.222 | 0.085 | 0.128 | 0.116 | 0.521 | 0.063 | 0.515 | -0.028 | 0.217 | -0.080 | 0.023 | -0.085 | -0.154 | -0.057 | -0.232 | -0.057 | -0.167 | -0.011 | -0.281 | 0.020 |
| Bleak | 1242 | 1 | 3 | 1 | -0.346 | 0.048 | -0.222 | 0.079 | 0.132 | 0.109 | 0.510 | 0.068 | 0.514 | -0.010 | 0.214 | -0.100 | 0.042 | -0.108 | -0.158 | -0.049 | -0.226 | -0.064 | -0.172 | -0.011 | -0.287 | 0.037 |
| Bleak | 1243 | 1 | 3 | 1 | -0.347 | 0.056 | -0.208 | 0.080 | 0.120 | 0.086 | 0.499 | 0.079 | 0.522 | 0.000 | 0.235 | -0.107 | 0.034 | -0.126 | -0.165 | -0.044 | -0.236 | -0.053 | -0.172 | -0.002 | -0.282 | 0.032 |
| Bleak | 1244 | 1 | 3 | 1 | -0.354 | 0.032 | -0.213 | 0.084 | 0.113 | 0.132 | 0.519 | 0.062 | 0.529 | -0.030 | 0.189 | -0.085 | 0.041 | -0.099 | -0.154 | -0.046 | -0.226 | -0.057 | -0.165 | -0.006 | -0.278 | 0.014 |
| Bleak | 1245 | 1 | 3 | 1 | -0.367 | 0.004 | -0.253 | 0.074 | 0.154 | 0.155 | 0.512 | 0.028 | 0.491 | -0.049 | 0.220 | -0.059 | 0.070 | -0.071 | -0.146 | -0.026 | -0.216 | -0.060 | -0.162 | -0.002 | -0.303 | 0.005 |
| Bleak | 1246 | 1 | 3 | 1 | -0.343 | 0.063 | -0.207 | 0.086 | 0.127 | 0.088 | 0.504 | 0.087 | 0.529 | 0.007 | 0.204 | -0.122 | 0.035 | -0.123 | -0.173 | -0.047 | -0.232 | -0.058 | -0.165 | -0.016 | -0.279 | 0.034 |
| Bleak | 1247 | 1 | 3 | 1 | -0.350 | 0.024 | -0.233 | 0.085 | 0.125 | 0.131 | 0.522 | 0.052 | 0.512 | -0.030 | 0.209 | -0.082 | 0.037 | -0.086 | -0.151 | -0.046 | -0.220 | -0.059 | -0.164 | -0.010 | -0.287 | 0.020 |
| Bleak | 1248 | 1 | 3 | 1 | -0.355 | 0.055 | -0.200 | 0.084 | 0.108 | 0.098 | 0.505 | 0.078 | 0.522 | -0.003 | 0.227 | -0.115 | 0.029 | -0.114 | -0.155 | -0.055 | -0.238 | -0.058 | -0.159 | -0.008 | -0.284 | 0.037 |
| Bleak | 1249 | 1 | 3 | 1 | -0.348 | 0.060 | -0.230 | 0.085 | 0.140 | 0.097 | 0.499 | 0.072 | 0.514 | -0.004 | 0.227 | -0.103 | 0.041 | -0.111 | -0.157 | -0.053 | -0.234 | -0.056 | -0.168 | -0.017 | -0.284 | 0.029 |
| Bleak | 1250 | 1 | 3 | 1 | -0.347 | 0.045 | -0.223 | 0.079 | 0.137 | 0.105 | 0.510 | 0.068 | 0.503 | -0.020 | 0.231 | -0.085 | 0.044 | -0.101 | -0.154 | -0.047 | -0.241 | -0.058 | -0.168 | -0.012 | -0.292 | 0.027 |
| Bleak | 1250 | 1 | 3 | 1 | -0.349 | 0.040 | -0.219 | 0.092 | 0.124 | 0.114 | 0.505 | 0.065 | 0.512 | -0.022 | 0.232 | -0.091 | 0.039 | -0.106 | -0.152 | -0.047 | -0.224 | -0.063 | -0.173 | -0.006 | -0.294 | 0.024 |
| Bleak | 1221 | 1 | 3 | 2 | -0.358 | 0.048 | -0.215 | 0.088 | 0.129 | 0.121 | 0.505 | 0.063 | 0.513 | -0.020 | 0.218 | -0.087 | 0.046 | -0.106 | -0.159 | -0.058 | -0.235 | -0.065 | -0.164 | -0.003 | -0.280 | 0.020 |
| Bleak | 1222 | 1 | 3 | 2 | -0.350 | 0.035 | -0.236 | 0.071 | 0.148 | 0.114 | 0.514 | 0.053 | 0.502 | -0.026 | 0.224 | -0.072 | 0.049 | -0.089 | -0.157 | -0.037 | -0.226 | -0.058 | -0.174 | -0.002 | -0.293 | 0.013 |
| Bleak | 1223 | 1 | 3 | 2 | -0.347 | 0.021 | -0.246 | 0.069 | 0.140 | 0.124 | 0.515 | 0.039 | 0.505 | -0.034 | 0.227 | -0.063 | 0.056 | -0.074 | -0.150 | -0.033 | -0.230 | -0.057 | -0.175 | -0.005 | -0.294 | 0.013 |
| Bleak | 1224 | 1 | 3 | 2 | -0.341 | 0.051 | -0.226 | 0.082 | 0.128 | 0.096 | 0.505 | 0.078 | 0.525 | -0.010 | 0.211 | -0.109 | 0.037 | -0.104 | -0.158 | -0.045 | -0.225 | -0.058 | -0.170 | -0.010 | -0.287 | 0.029 |
| Bleak | 1225 | 1 | 3 | 2 | -0.345 | 0.074 | -0.195 | 0.077 | 0.109 | 0.075 | 0.511 | 0.080 | 0.523 | 0.003 | 0.225 | -0.098 | 0.038 | -0.118 | -0.170 | -0.052 | -0.239 | -0.057 | -0.173 | -0.017 | -0.285 | 0.033 |
| Bleak | 1226 | 1 | 3 | 2 | -0.345 | 0.046 | -0.229 | 0.080 | 0.149 | 0.105 | 0.498 | 0.071 | 0.505 | -0.020 | 0.227 | -0.093 | 0.058 | -0.102 | -0.157 | -0.048 | -0.231 | -0.058 | -0.178 | -0.012 | -0.298 | 0.031 |
| Bleak | 1227 | 1 | 3 | 2 | -0.352 | 0.049 | -0.222 | 0.081 | 0.132 | 0.107 | 0.498 | 0.068 | 0.503 | -0.019 | 0.241 | -0.094 | 0.058 | -0.109 | -0.153 | -0.052 | -0.231 | -0.060 | -0.177 | -0.004 | -0.297 | 0.032 |
| Bleak | 1228 | 1 | 3 | 2 | -0.358 | 0.028 | -0.245 | 0.071 | 0.124 | 0.144 | 0.524 | 0.036 | 0.509 | -0.049 | 0.210 | -0.050 | 0.051 | -0.071 | -0.145 | -0.045 | -0.221 | -0.061 | -0.163 | -0.007 | -0.287 | 0.004 |
| Bleak | 1229 | 1 | 3 | 2 | -0.343 | 0.056 | -0.231 | 0.078 | 0.108 | 0.116 | 0.513 | 0.063 | 0.515 | -0.024 | 0.222 | -0.085 | 0.054 | -0.101 | -0.154 | -0.058 | -0.231 | -0.068 | -0.169 | -0.009 | -0.283 | 0.032 |
| Bleak | 1230 | 1 | 3 | 2 | -0.355 | 0.048 | -0.233 | 0.082 | 0.128 | 0.114 | 0.506 | 0.073 | 0.519 | -0.028 | 0.211 | -0.095 | 0.041 | -0.101 | -0.144 | -0.045 | -0.219 | -0.064 | -0.165 | -0.005 | -0.290 | 0.021 |
| Bleak | 1232 | 1 | 3 | 2 | -0.350 | 0.051 | -0.234 | 0.073 | 0.141 | 0.115 | 0.500 | 0.066 | 0.508 | -0.019 | 0.226 | -0.093 | 0.051 | -0.109 | -0.156 | -0.042 | -0.227 | -0.064 | -0.168 | -0.004 | -0.291 | 0.026 |
| Bleak | 1233 | 1 | 3 | 2 | -0.355 | 0.036 | -0.224 | 0.075 | 0.149 | 0.117 | 0.500 | 0.063 | 0.514 | -0.028 | 0.218 | -0.086 | 0.051 | -0.090 | -0.157 | -0.044 | -0.231 | -0.059 | -0.171 | -0.001 | -0.291 | 0.017 |
| Bleak | 1234 | 1 | 3 | 2 | -0.358 | 0.044 | -0.226 | 0.078 | 0.154 | 0.125 | 0.510 | 0.068 | 0.513 | -0.027 | 0.196 | -0.089 | 0.039 | -0.091 | -0.144 | -0.053 | -0.238 | -0.057 | -0.164 | -0.008 | -0.281 | 0.011 |
| Bleak | 1235 | 1 | 3 | 2 | -0.343 | 0.059 | -0.208 | 0.073 | 0.111 | 0.100 | 0.513 | 0.068 | 0.517 | -0.017 | 0.222 | -0.080 | 0.052 | -0.106 | -0.163 | -0.057 | -0.248 | -0.057 | -0.172 | -0.013 | -0.282 | 0.029 |
| Bleak | 1236 | 1 | 3 | 2 | -0.353 | 0.026 | -0.219 | 0.084 | 0.112 | 0.116 | 0.534 | 0.056 | 0.512 | -0.030 | 0.205 | -0.077 | 0.036 | -0.082 | -0.146 | -0.043 | -0.228 | -0.054 | -0.164 | -0.009 | -0.288 | 0.012 |
| Bleak | 1237 | 1 | 3 | 2 | -0.360 | 0.022 | -0.219 | 0.084 | 0.136 | 0.132 | 0.517 | 0.053 | 0.507 | -0.034 | 0.212 | -0.076 | 0.041 | -0.085 | -0.153 | -0.040 | -0.226 | -0.065 | -0.165 | -0.008 | -0.292 | 0.017 |
| Bleak | 1238 | 1 | 3 | 2 | -0.354 | 0.030 | -0.230 | 0.083 | 0.128 | 0.128 | 0.510 | 0.053 | 0.504 | -0.025 | 0.232 | -0.090 | 0.046 | -0.092 | -0.153 | -0.046 | -0.229 | -0.055 | -0.165 | -0.007 | -0.289 | 0.021 |
| Bleak | 1239 | 1 | 3 | 2 | -0.353 | 0.047 | -0.232 | 0.082 | 0.138 | 0.112 | 0.515 | 0.055 | 0.495 | -0.015 | 0.223 | -0.083 | 0.063 | -0.100 | -0.152 | -0.052 | -0.233 | -0.060 | -0.172 | -0.006 | -0.291 | 0.022 |
| Bleak | 1240 | 1 | 3 | 2 | -0.353 | 0.026 | -0.220 | 0.082 | 0.123 | 0.132 | 0.513 | 0.049 | 0.520 | -0.039 | 0.210 | -0.072 | 0.049 | -0.079 | -0.154 | -0.042 | -0.218 | -0.062 | -0.176 | -0.004 | -0.293 | 0.008 |
| Bleak | 1241 | 1 | 3 | 2 | -0.358 | 0.033 | -0.212 | 0.084 | 0.134 | 0.117 | 0.519 | 0.067 | 0.515 | -0.026 | 0.201 | -0.085 | 0.034 | -0.091 | -0.152 | -0.048 | -0.233 | -0.059 | -0.165 | -0.008 | -0.284 | 0.018 |
| Bleak | 1242 | 1 | 3 | 2 | -0.350 | 0.038 | -0.221 | 0.082 | 0.138 | 0.111 | 0.513 | 0.068 | 0.507 | -0.012 | 0.210 | -0.095 | 0.046 | -0.114 | -0.157 | -0.048 | -0.230 | -0.062 | -0.168 | 0.001 | -0.289 | 0.031 |
| Bleak | 1243 | 1 | 3 | 2 | -0.339 | 0.065 | -0.211 | 0.077 | 0.128 | 0.092 | 0.507 | 0.084 | 0.523 | 0.000 | 0.209 | -0.110 | 0.041 | -0.121 | -0.159 | -0.055 | -0.244 | -0.046 | -0.174 | -0.013 | -0.281 | 0.028 |
| Bleak | 1244 | 1 | 3 | 2 | -0.350 | 0.035 | -0.223 | 0.083 | 0.102 | 0.125 | 0.519 | 0.060 | 0.524 | -0.030 | 0.209 | -0.087 | 0.043 | -0.095 | -0.152 | -0.040 | -0.231 | -0.055 | -0.163 | -0.006 | -0.278 | 0.011 |
| Bleak | 1245 | 1 | 3 | 2 | -0.361 | 0.008 | -0.258 | 0.074 | 0.143 | 0.163 | 0.516 | 0.028 | 0.487 | -0.052 | 0.228 | -0.064 | 0.071 | -0.068 | -0.147 | -0.031 | -0.218 | -0.059 | -0.160 | -0.007 | -0.301 | 0.008 |
| Bleak | 1246 | 1 | 3 | 2 | -0.337 | 0.060 | -0.208 | 0.085 | 0.117 | 0.089 | 0.510 | 0.087 | 0.521 | 0.003 | 0.220 | -0.118 | 0.030 | -0.128 | -0.162 | -0.049 | -0.240 | -0.050 | -0.172 | -0.017 | -0.279 | 0.036 |
| Bleak | 1247 | 1 | 3 | 2 | -0.350 | 0.024 | -0.222 | 0.083 | 0.117 | 0.127 | 0.523 | 0.053 | 0.512 | -0.031 | 0.210 | -0.080 | 0.047 | -0.087 | -0.154 | -0.045 | -0.221 | -0.059 | -0.172 | -0.005 | -0.291 | 0.020 |
| Bleak | 1248 | 1 | 3 | 2 | -0.352 | 0.061 | -0.197 | 0.083 | 0.109 | 0.096 | 0.500 | 0.076 | 0.527 | 0.000 | 0.226 | -0.111 | 0.036 | -0.119 | -0.165 | -0.054 | -0.232 | -0.062 | -0.169 | -0.009 | -0.282 | 0.038 |
| Bleak | 1249 | 1 | 3 | 2 | -0.350 | 0.068 | -0.235 | 0.077 | 0.151 | 0.094 | 0.500 | 0.068 | 0.509 | 0.003 | 0.225 | -0.102 | 0.041 | -0.110 | -0.157 | -0.056 | -0.225 | -0.059 | -0.172 | -0.014 | -0.287 | 0.031 |
| Bleak | 1250 | 1 | 3 | 2 | -0.343 | 0.046 | -0.211 | 0.071 | 0.133 | 0.107 | 0.521 | 0.071 | 0.509 | -0.017 | 0.215 | -0.089 | 0.035 | -0.102 | -0.162 | -0.050 | -0.232 | -0.052 | -0.174 | -0.009 | -0.292 | 0.025 |
| Bleak | 1250 | 1 | 3 | 2 | -0.343 | 0.044 | -0.217 | 0.086 | 0.131 | 0.116 | 0.509 | 0.067 | 0.508 | -0.019 | 0.223 | -0.092 | 0.047 | -0.107 | -0.155 | -0.048 | -0.241 | -0.059 | -0.170 | -0.009 | -0.291 | 0.022 |
| Bleak | 1221 | 1 | 3 | 3 | -0.356 | 0.049 | -0.213 | 0.091 | 0.127 | 0.120 | 0.504 | 0.066 | 0.515 | -0.021 | 0.217 | -0.089 | 0.046 | -0.105 | -0.158 | -0.056 | -0.237 | -0.067 | -0.162 | -0.013 | -0.283 | 0.026 |
| Bleak | 1222 | 1 | 3 | 3 | -0.352 | 0.041 | -0.221 | 0.077 | 0.135 | 0.118 | 0.519 | 0.054 | 0.500 | -0.026 | 0.226 | -0.073 | 0.050 | -0.093 | -0.159 | -0.042 | -0.226 | -0.060 | -0.179 | -0.013 | -0.291 | 0.016 |
| Bleak | 1223 | 1 | 3 | 3 | -0.347 | 0.020 | -0.247 | 0.068 | 0.141 | 0.129 | 0.508 | 0.037 | 0.512 | -0.034 | 0.222 | -0.062 | 0.054 | -0.080 | -0.144 | -0.031 | -0.228 | -0.058 | -0.176 | -0.003 | -0.296 | 0.014 |
| Bleak | 1224 | 1 | 3 | 3 | -0.346 | 0.054 | -0.214 | 0.081 | 0.124 | 0.094 | 0.513 | 0.084 | 0.524 | -0.011 | 0.203 | -0.109 | 0.037 | -0.106 | -0.161 | -0.048 | -0.223 | -0.061 | -0.170 | -0.010 | -0.287 | 0.033 |
| Bleak | 1225 | 1 | 3 | 3 | -0.347 | 0.067 | -0.201 | 0.079 | 0.121 | 0.082 | 0.506 | 0.081 | 0.519 | 0.000 | 0.225 | -0.098 | 0.038 | -0.120 | -0.159 | -0.060 | -0.236 | -0.051 | -0.177 | -0.016 | -0.288 | 0.037 |
| Bleak | 1226 | 1 | 3 | 3 | -0.345 | 0.044 | -0.218 | 0.079 | 0.137 | 0.103 | 0.514 | 0.070 | 0.508 | -0.013 | 0.219 | -0.094 | 0.038 | -0.106 | -0.155 | -0.046 | -0.227 | -0.058 | -0.177 | -0.004 | -0.293 | 0.026 |
| Bleak | 1227 | 1 | 3 | 3 | -0.349 | 0.048 | -0.212 | 0.077 | 0.127 | 0.107 | 0.495 | 0.072 | 0.516 | -0.020 | 0.234 | -0.096 | 0.050 | -0.108 | -0.156 | -0.046 | -0.235 | -0.059 | -0.175 | -0.006 | -0.295 | 0.031 |
| Bleak | 1228 | 1 | 3 | 3 | -0.356 | 0.021 | -0.236 | 0.074 | 0.133 | 0.146 | 0.529 | 0.038 | 0.505 | -0.047 | 0.206 | -0.050 | 0.043 | -0.075 | -0.147 | -0.045 | -0.218 | -0.064 | -0.170 | -0.002 | -0.290 | 0.005 |
| Bleak | 1229 | 1 | 3 | 3 | -0.349 | 0.056 | -0.211 | 0.080 | 0.123 | 0.114 | 0.519 | 0.064 | 0.511 | -0.021 | 0.208 | -0.080 | 0.046 | -0.098 | -0.158 | -0.060 | -0.228 | -0.067 | -0.172 | -0.018 | -0.291 | 0.029 |
| Bleak | 1230 | 1 | 3 | 3 | -0.351 | 0.052 | -0.229 | 0.081 | 0.128 | 0.112 | 0.520 | 0.069 | 0.513 | -0.022 | 0.206 | -0.091 | 0.038 | -0.096 | -0.152 | -0.047 | -0.221 | -0.064 | -0.168 | -0.017 | -0.283 | 0.023 |
| Bleak | 1232 | 1 | 3 | 3 | -0.355 | 0.043 | -0.222 | 0.074 | 0.145 | 0.116 | 0.508 | 0.069 | 0.504 | -0.021 | 0.218 | -0.093 | 0.051 | -0.104 | -0.164 | -0.039 | -0.229 | -0.058 | -0.164 | -0.014 | -0.292 | 0.027 |
| Bleak | 1233 | 1 | 3 | 3 | -0.354 | 0.033 | -0.219 | 0.082 | 0.149 | 0.120 | 0.504 | 0.060 | 0.514 | -0.025 | 0.208 | -0.085 | 0.050 | -0.090 | -0.159 | -0.049 | -0.234 | -0.065 | -0.171 | 0.005 | -0.287 | 0.014 |
| Bleak | 1234 | 1 | 3 | 3 | -0.351 | 0.036 | -0.225 | 0.081 | 0.153 | 0.125 | 0.506 | 0.067 | 0.513 | -0.024 | 0.211 | -0.092 | 0.033 | -0.096 | -0.153 | -0.051 | -0.246 | -0.050 | -0.162 | -0.007 | -0.278 | 0.011 |
| Bleak | 1235 | 1 | 3 | 3 | -0.344 | 0.059 | -0.207 | 0.087 | 0.110 | 0.095 | 0.511 | 0.071 | 0.512 | -0.014 | 0.229 | -0.085 | 0.056 | -0.114 | -0.166 | -0.052 | -0.239 | -0.058 | -0.175 | -0.019 | -0.287 | 0.029 |
| Bleak | 1236 | 1 | 3 | 3 | -0.360 | 0.029 | -0.237 | 0.083 | 0.132 | 0.127 | 0.529 | 0.051 | 0.499 | -0.032 | 0.213 | -0.076 | 0.035 | -0.085 | -0.141 | -0.047 | -0.221 | -0.059 | -0.160 | 0.001 | -0.289 | 0.007 |
| Bleak | 1237 | 1 | 3 | 3 | -0.358 | 0.027 | -0.232 | 0.080 | 0.142 | 0.132 | 0.527 | 0.049 | 0.494 | -0.029 | 0.211 | -0.077 | 0.046 | -0.086 | -0.152 | -0.045 | -0.219 | -0.062 | -0.172 | -0.004 | -0.288 | 0.015 |
| Bleak | 1238 | 1 | 3 | 3 | -0.353 | 0.034 | -0.232 | 0.081 | 0.128 | 0.124 | 0.514 | 0.055 | 0.505 | -0.027 | 0.224 | -0.086 | 0.051 | -0.091 | -0.156 | -0.048 | -0.226 | -0.055 | -0.169 | -0.004 | -0.287 | 0.017 |
| Bleak | 1239 | 1 | 3 | 3 | -0.351 | 0.046 | -0.228 | 0.080 | 0.139 | 0.113 | 0.508 | 0.063 | 0.501 | -0.018 | 0.228 | -0.087 | 0.050 | -0.106 | -0.154 | -0.048 | -0.237 | -0.059 | -0.168 | -0.006 | -0.288 | 0.024 |
| Bleak | 1240 | 1 | 3 | 3 | -0.354 | 0.030 | -0.225 | 0.075 | 0.139 | 0.126 | 0.517 | 0.049 | 0.506 | -0.033 | 0.218 | -0.072 | 0.048 | -0.078 | -0.153 | -0.045 | -0.231 | -0.058 | -0.176 | -0.004 | -0.289 | 0.009 |
| Bleak | 1241 | 1 | 3 | 3 | -0.353 | 0.035 | -0.214 | 0.080 | 0.140 | 0.117 | 0.512 | 0.068 | 0.513 | -0.027 | 0.212 | -0.083 | 0.036 | -0.095 | -0.154 | -0.050 | -0.250 | -0.055 | -0.161 | -0.008 | -0.281 | 0.019 |
| Bleak | 1242 | 1 | 3 | 3 | -0.351 | 0.035 | -0.221 | 0.083 | 0.144 | 0.104 | 0.512 | 0.066 | 0.511 | -0.008 | 0.208 | -0.094 | 0.041 | -0.108 | -0.155 | -0.045 | -0.232 | -0.061 | -0.170 | -0.005 | -0.287 | 0.034 |
| Bleak | 1243 | 1 | 3 | 3 | -0.344 | 0.060 | -0.207 | 0.079 | 0.103 | 0.090 | 0.504 | 0.079 | 0.516 | -0.004 | 0.248 | -0.107 | 0.041 | -0.125 | -0.165 | -0.051 | -0.245 | -0.046 | -0.173 | -0.008 | -0.278 | 0.033 |
| Bleak | 1244 | 1 | 3 | 3 | -0.355 | 0.034 | -0.214 | 0.084 | 0.117 | 0.126 | 0.519 | 0.059 | 0.515 | -0.026 | 0.209 | -0.086 | 0.043 | -0.098 | -0.161 | -0.041 | -0.222 | -0.059 | -0.167 | -0.010 | -0.284 | 0.016 |
| Bleak | 1245 | 1 | 3 | 3 | -0.360 | 0.002 | -0.255 | 0.075 | 0.145 | 0.160 | 0.518 | 0.029 | 0.492 | -0.054 | 0.221 | -0.065 | 0.067 | -0.059 | -0.151 | -0.028 | -0.212 | -0.062 | -0.165 | -0.004 | -0.301 | 0.005 |
| Bleak | 1246 | 1 | 3 | 3 | -0.338 | 0.061 | -0.206 | 0.082 | 0.123 | 0.083 | 0.507 | 0.090 | 0.529 | 0.004 | 0.208 | -0.119 | 0.029 | -0.124 | -0.169 | -0.050 | -0.230 | -0.052 | -0.169 | -0.009 | -0.284 | 0.034 |
| Bleak | 1247 | 1 | 3 | 3 | -0.351 | 0.024 | -0.229 | 0.081 | 0.133 | 0.120 | 0.527 | 0.052 | 0.501 | -0.028 | 0.214 | -0.077 | 0.046 | -0.084 | -0.154 | -0.043 | -0.221 | -0.059 | -0.175 | -0.003 | -0.291 | 0.016 |
| Bleak | 1248 | 1 | 3 | 3 | -0.353 | 0.059 | -0.200 | 0.084 | 0.121 | 0.093 | 0.500 | 0.083 | 0.525 | -0.003 | 0.216 | -0.111 | 0.042 | -0.118 | -0.160 | -0.057 | -0.241 | -0.057 | -0.170 | -0.012 | -0.280 | 0.038 |
| Bleak | 1249 | 1 | 3 | 3 | -0.351 | 0.062 | -0.220 | 0.080 | 0.145 | 0.089 | 0.504 | 0.075 | 0.514 | 0.002 | 0.220 | -0.102 | 0.033 | -0.111 | -0.160 | -0.058 | -0.237 | -0.052 | -0.168 | -0.014 | -0.281 | 0.030 |
| Bleak | 1250 | 1 | 3 | 3 | -0.344 | 0.046 | -0.225 | 0.080 | 0.137 | 0.113 | 0.513 | 0.067 | 0.506 | -0.020 | 0.223 | -0.088 | 0.041 | -0.102 | -0.157 | -0.048 | -0.233 | -0.059 | -0.171 | -0.014 | -0.289 | 0.026 |
| Bleak | 1250 | 1 | 3 | 3 | -0.349 | 0.046 | -0.214 | 0.079 | 0.137 | 0.120 | 0.498 | 0.067 | 0.513 | -0.024 | 0.231 | -0.089 | 0.037 | -0.105 | -0.156 | -0.047 | -0.232 | -0.058 | -0.171 | -0.011 | -0.295 | 0.022 |
| Bleak | 1031 | 2 | 1 | 1 | -0.357 | 0.024 | -0.222 | 0.077 | 0.135 | 0.126 | 0.515 | 0.056 | 0.516 | -0.036 | 0.215 | -0.071 | 0.028 | -0.087 | -0.153 | -0.035 | -0.234 | -0.066 | -0.162 | 0.000 | -0.282 | 0.013 |
| Bleak | 1032 | 2 | 1 | 1 | -0.350 | 0.038 | -0.213 | 0.085 | 0.118 | 0.111 | 0.517 | 0.077 | 0.516 | -0.020 | 0.210 | -0.108 | 0.039 | -0.105 | -0.165 | -0.039 | -0.227 | -0.067 | -0.165 | 0.004 | -0.280 | 0.023 |
| Bleak | 1033 | 2 | 1 | 1 | -0.343 | 0.046 | -0.215 | 0.084 | 0.126 | 0.102 | 0.517 | 0.077 | 0.516 | -0.015 | 0.215 | -0.092 | 0.027 | -0.114 | -0.166 | -0.047 | -0.238 | -0.056 | -0.162 | -0.006 | -0.278 | 0.021 |
| Bleak | 1034 | 2 | 1 | 1 | -0.333 | 0.066 | -0.222 | 0.085 | 0.132 | 0.105 | 0.505 | 0.083 | 0.499 | -0.009 | 0.236 | -0.108 | 0.055 | -0.126 | -0.169 | -0.058 | -0.253 | -0.051 | -0.168 | -0.018 | -0.282 | 0.029 |
| Bleak | 1035 | 2 | 1 | 1 | -0.349 | 0.051 | -0.226 | 0.084 | 0.114 | 0.100 | 0.511 | 0.077 | 0.519 | -0.009 | 0.211 | -0.109 | 0.046 | -0.110 | -0.161 | -0.053 | -0.252 | -0.059 | -0.147 | -0.001 | -0.268 | 0.028 |
| Bleak | 1036 | 2 | 1 | 1 | -0.352 | 0.046 | -0.220 | 0.079 | 0.120 | 0.108 | 0.523 | 0.075 | 0.512 | -0.017 | 0.207 | -0.099 | 0.038 | -0.105 | -0.159 | -0.050 | -0.250 | -0.060 | -0.155 | -0.001 | -0.264 | 0.023 |
| Bleak | 1037 | 2 | 1 | 1 | -0.354 | 0.028 | -0.218 | 0.082 | 0.128 | 0.114 | 0.515 | 0.064 | 0.510 | -0.025 | 0.220 | -0.089 | 0.039 | -0.097 | -0.163 | -0.038 | -0.230 | -0.063 | -0.166 | 0.005 | -0.283 | 0.019 |
| Bleak | 1038 | 2 | 1 | 1 | -0.344 | 0.048 | -0.219 | 0.084 | 0.147 | 0.097 | 0.510 | 0.074 | 0.509 | -0.014 | 0.219 | -0.091 | 0.040 | -0.104 | -0.170 | -0.048 | -0.228 | -0.055 | -0.176 | -0.012 | -0.287 | 0.021 |
| Bleak | 1039 | 2 | 1 | 1 | -0.344 | 0.068 | -0.209 | 0.083 | 0.120 | 0.099 | 0.491 | 0.085 | 0.524 | -0.007 | 0.238 | -0.108 | 0.032 | -0.128 | -0.164 | -0.065 | -0.253 | -0.053 | -0.158 | -0.005 | -0.276 | 0.031 |
| Bleak | 1040 | 2 | 1 | 1 | -0.365 | 0.028 | -0.211 | 0.085 | 0.148 | 0.111 | 0.515 | 0.070 | 0.502 | -0.023 | 0.209 | -0.090 | 0.043 | -0.098 | -0.159 | -0.044 | -0.241 | -0.063 | -0.160 | 0.006 | -0.281 | 0.018 |
| Bleak | 1041 | 2 | 1 | 1 | -0.355 | 0.039 | -0.201 | 0.091 | 0.140 | 0.118 | 0.513 | 0.078 | 0.508 | -0.025 | 0.199 | -0.097 | 0.054 | -0.108 | -0.166 | -0.043 | -0.234 | -0.063 | -0.167 | 0.000 | -0.290 | 0.011 |
| Bleak | 1042 | 2 | 1 | 1 | -0.347 | 0.050 | -0.208 | 0.078 | 0.128 | 0.107 | 0.518 | 0.073 | 0.509 | -0.018 | 0.219 | -0.089 | 0.035 | -0.108 | -0.164 | -0.050 | -0.243 | -0.061 | -0.164 | -0.001 | -0.281 | 0.018 |
| Bleak | 1043 | 2 | 1 | 1 | -0.347 | 0.035 | -0.224 | 0.079 | 0.122 | 0.117 | 0.518 | 0.063 | 0.535 | -0.027 | 0.184 | -0.086 | 0.037 | -0.085 | -0.158 | -0.043 | -0.235 | -0.062 | -0.160 | -0.006 | -0.274 | 0.014 |
| Bleak | 1044 | 2 | 1 | 1 | -0.357 | 0.029 | -0.220 | 0.078 | 0.142 | 0.117 | 0.512 | 0.059 | 0.502 | -0.026 | 0.224 | -0.083 | 0.049 | -0.092 | -0.162 | -0.042 | -0.241 | -0.063 | -0.165 | 0.007 | -0.285 | 0.016 |
| Bleak | 1045 | 2 | 1 | 1 | -0.336 | 0.040 | -0.227 | 0.079 | 0.109 | 0.128 | 0.524 | 0.065 | 0.515 | -0.036 | 0.216 | -0.084 | 0.042 | -0.094 | -0.171 | -0.048 | -0.226 | -0.056 | -0.167 | -0.003 | -0.279 | 0.010 |
| Bleak | 1046 | 2 | 1 | 1 | -0.350 | 0.052 | -0.219 | 0.087 | 0.131 | 0.099 | 0.495 | 0.080 | 0.527 | -0.005 | 0.214 | -0.114 | 0.040 | -0.116 | -0.161 | -0.053 | -0.245 | -0.051 | -0.156 | -0.002 | -0.274 | 0.024 |
| Bleak | 1047 | 2 | 1 | 1 | -0.356 | 0.046 | -0.200 | 0.084 | 0.124 | 0.095 | 0.521 | 0.081 | 0.508 | -0.011 | 0.214 | -0.100 | 0.035 | -0.113 | -0.167 | -0.050 | -0.240 | -0.058 | -0.158 | 0.002 | -0.280 | 0.025 |
| Bleak | 1048 | 2 | 1 | 1 | -0.358 | 0.028 | -0.230 | 0.073 | 0.135 | 0.136 | 0.527 | 0.048 | 0.501 | -0.031 | 0.210 | -0.077 | 0.045 | -0.081 | -0.157 | -0.039 | -0.228 | -0.065 | -0.162 | -0.003 | -0.281 | 0.012 |
| Bleak | 1049 | 2 | 1 | 1 | -0.342 | 0.054 | -0.205 | 0.083 | 0.132 | 0.098 | 0.525 | 0.082 | 0.501 | -0.007 | 0.219 | -0.097 | 0.029 | -0.123 | -0.175 | -0.054 | -0.234 | -0.054 | -0.169 | -0.007 | -0.281 | 0.025 |
| Bleak | 1050 | 2 | 1 | 1 | -0.353 | 0.026 | -0.232 | 0.082 | 0.128 | 0.134 | 0.531 | 0.054 | 0.497 | -0.029 | 0.213 | -0.087 | 0.039 | -0.086 | -0.149 | -0.045 | -0.235 | -0.062 | -0.163 | 0.001 | -0.277 | 0.012 |
| Bleak | 1051 | 2 | 1 | 1 | -0.340 | 0.026 | -0.260 | 0.068 | 0.121 | 0.136 | 0.532 | 0.054 | 0.515 | -0.039 | 0.187 | -0.079 | 0.055 | -0.089 | -0.154 | -0.036 | -0.220 | -0.059 | -0.161 | 0.007 | -0.275 | 0.012 |
| Bleak | 1052 | 2 | 1 | 1 | -0.341 | 0.024 | -0.206 | 0.081 | 0.126 | 0.107 | 0.518 | 0.072 | 0.518 | -0.021 | 0.214 | -0.094 | 0.034 | -0.100 | -0.167 | -0.037 | -0.244 | -0.055 | -0.172 | 0.009 | -0.280 | 0.015 |
| Bleak | 1053 | 2 | 1 | 1 | -0.356 | 0.018 | -0.228 | 0.079 | 0.134 | 0.129 | 0.525 | 0.050 | 0.504 | -0.036 | 0.206 | -0.074 | 0.053 | -0.078 | -0.159 | -0.034 | -0.232 | -0.064 | -0.161 | -0.002 | -0.287 | 0.011 |
| Bleak | 1054 | 2 | 1 | 1 | -0.355 | 0.036 | -0.223 | 0.087 | 0.136 | 0.129 | 0.514 | 0.069 | 0.513 | -0.026 | 0.202 | -0.097 | 0.038 | -0.101 | -0.160 | -0.047 | -0.237 | -0.065 | -0.154 | 0.000 | -0.273 | 0.015 |
| Bleak | 1055 | 2 | 1 | 1 | -0.361 | 0.007 | -0.236 | 0.080 | 0.134 | 0.138 | 0.532 | 0.051 | 0.500 | -0.047 | 0.206 | -0.072 | 0.039 | -0.077 | -0.148 | -0.036 | -0.219 | -0.062 | -0.162 | 0.013 | -0.286 | 0.004 |
| Bleak | 1056 | 2 | 1 | 1 | -0.354 | 0.020 | -0.211 | 0.084 | 0.128 | 0.122 | 0.512 | 0.066 | 0.519 | -0.034 | 0.211 | -0.089 | 0.038 | -0.090 | -0.155 | -0.033 | -0.242 | -0.057 | -0.163 | 0.004 | -0.284 | 0.008 |
| Bleak | 1057 | 2 | 1 | 1 | -0.345 | 0.060 | -0.217 | 0.080 | 0.156 | 0.079 | 0.497 | 0.092 | 0.513 | 0.001 | 0.229 | -0.116 | 0.025 | -0.120 | -0.166 | -0.043 | -0.239 | -0.054 | -0.167 | -0.007 | -0.285 | 0.028 |
| Bleak | 1058 | 2 | 1 | 1 | -0.354 | 0.049 | -0.201 | 0.091 | 0.121 | 0.097 | 0.509 | 0.084 | 0.527 | -0.010 | 0.201 | -0.111 | 0.037 | -0.108 | -0.160 | -0.046 | -0.258 | -0.060 | -0.153 | -0.005 | -0.270 | 0.018 |
| Bleak | 1059 | 2 | 1 | 1 | -0.333 | 0.054 | -0.203 | 0.086 | 0.137 | 0.088 | 0.502 | 0.091 | 0.517 | 0.003 | 0.220 | -0.120 | 0.038 | -0.131 | -0.175 | -0.040 | -0.243 | -0.059 | -0.177 | -0.005 | -0.282 | 0.034 |
| Bleak | 1060 | 2 | 1 | 1 | -0.332 | 0.072 | -0.216 | 0.081 | 0.137 | 0.072 | 0.496 | 0.088 | 0.526 | 0.007 | 0.221 | -0.113 | 0.043 | -0.119 | -0.179 | -0.059 | -0.246 | -0.050 | -0.171 | -0.006 | -0.278 | 0.028 |
| Bleak | 1031 | 2 | 1 | 2 | -0.354 | 0.031 | -0.221 | 0.077 | 0.135 | 0.128 | 0.516 | 0.057 | 0.513 | -0.034 | 0.215 | -0.073 | 0.029 | -0.093 | -0.154 | -0.034 | -0.235 | -0.068 | -0.159 | -0.006 | -0.284 | 0.013 |
| Bleak | 1032 | 2 | 1 | 2 | -0.350 | 0.028 | -0.209 | 0.087 | 0.130 | 0.109 | 0.511 | 0.075 | 0.522 | -0.018 | 0.203 | -0.105 | 0.037 | -0.106 | -0.165 | -0.039 | -0.233 | -0.063 | -0.165 | 0.006 | -0.282 | 0.025 |
| Bleak | 1033 | 2 | 1 | 2 | -0.343 | 0.049 | -0.207 | 0.084 | 0.129 | 0.102 | 0.513 | 0.079 | 0.516 | -0.014 | 0.214 | -0.097 | 0.034 | -0.113 | -0.170 | -0.048 | -0.240 | -0.056 | -0.165 | -0.006 | -0.281 | 0.020 |
| Bleak | 1034 | 2 | 1 | 2 | -0.335 | 0.067 | -0.199 | 0.084 | 0.134 | 0.101 | 0.501 | 0.086 | 0.511 | -0.005 | 0.226 | -0.109 | 0.047 | -0.125 | -0.175 | -0.056 | -0.253 | -0.053 | -0.171 | -0.019 | -0.285 | 0.029 |
| Bleak | 1035 | 2 | 1 | 2 | -0.350 | 0.053 | -0.214 | 0.084 | 0.124 | 0.101 | 0.507 | 0.084 | 0.520 | -0.012 | 0.206 | -0.107 | 0.045 | -0.113 | -0.164 | -0.051 | -0.249 | -0.060 | -0.152 | -0.010 | -0.274 | 0.031 |
| Bleak | 1036 | 2 | 1 | 2 | -0.354 | 0.039 | -0.225 | 0.081 | 0.134 | 0.110 | 0.511 | 0.073 | 0.516 | -0.018 | 0.209 | -0.097 | 0.037 | -0.104 | -0.161 | -0.047 | -0.246 | -0.059 | -0.155 | -0.002 | -0.267 | 0.025 |
| Bleak | 1037 | 2 | 1 | 2 | -0.350 | 0.027 | -0.220 | 0.081 | 0.131 | 0.117 | 0.522 | 0.066 | 0.512 | -0.026 | 0.214 | -0.086 | 0.023 | -0.096 | -0.158 | -0.038 | -0.233 | -0.058 | -0.161 | -0.005 | -0.280 | 0.018 |
| Bleak | 1038 | 2 | 1 | 2 | -0.347 | 0.050 | -0.225 | 0.087 | 0.147 | 0.100 | 0.502 | 0.071 | 0.513 | -0.014 | 0.218 | -0.091 | 0.048 | -0.104 | -0.172 | -0.050 | -0.229 | -0.057 | -0.169 | -0.014 | -0.288 | 0.022 |
| Bleak | 1039 | 2 | 1 | 2 | -0.345 | 0.065 | -0.205 | 0.085 | 0.129 | 0.098 | 0.491 | 0.086 | 0.526 | -0.008 | 0.230 | -0.107 | 0.030 | -0.122 | -0.168 | -0.063 | -0.246 | -0.057 | -0.165 | -0.009 | -0.277 | 0.030 |
| Bleak | 1040 | 2 | 1 | 2 | -0.362 | 0.026 | -0.220 | 0.085 | 0.145 | 0.111 | 0.515 | 0.068 | 0.511 | -0.026 | 0.207 | -0.089 | 0.034 | -0.088 | -0.160 | -0.042 | -0.234 | -0.061 | -0.157 | -0.001 | -0.279 | 0.017 |
| Bleak | 1041 | 2 | 1 | 2 | -0.356 | 0.039 | -0.211 | 0.093 | 0.136 | 0.115 | 0.510 | 0.075 | 0.515 | -0.028 | 0.201 | -0.094 | 0.048 | -0.097 | -0.160 | -0.050 | -0.227 | -0.058 | -0.167 | -0.010 | -0.289 | 0.015 |
| Bleak | 1042 | 2 | 1 | 2 | -0.347 | 0.052 | -0.208 | 0.078 | 0.118 | 0.109 | 0.522 | 0.075 | 0.512 | -0.022 | 0.216 | -0.086 | 0.035 | -0.107 | -0.162 | -0.045 | -0.242 | -0.058 | -0.163 | -0.018 | -0.280 | 0.022 |
| Bleak | 1043 | 2 | 1 | 2 | -0.345 | 0.039 | -0.223 | 0.079 | 0.126 | 0.119 | 0.521 | 0.062 | 0.531 | -0.026 | 0.183 | -0.083 | 0.038 | -0.082 | -0.160 | -0.055 | -0.238 | -0.063 | -0.160 | -0.007 | -0.273 | 0.016 |
| Bleak | 1044 | 2 | 1 | 2 | -0.352 | 0.033 | -0.228 | 0.074 | 0.138 | 0.120 | 0.509 | 0.063 | 0.511 | -0.032 | 0.216 | -0.080 | 0.052 | -0.094 | -0.158 | -0.040 | -0.243 | -0.061 | -0.162 | 0.003 | -0.283 | 0.016 |
| Bleak | 1045 | 2 | 1 | 2 | -0.339 | 0.035 | -0.221 | 0.083 | 0.108 | 0.128 | 0.532 | 0.065 | 0.512 | -0.037 | 0.213 | -0.082 | 0.033 | -0.090 | -0.166 | -0.049 | -0.225 | -0.059 | -0.170 | -0.005 | -0.278 | 0.010 |
| Bleak | 1046 | 2 | 1 | 2 | -0.349 | 0.052 | -0.214 | 0.087 | 0.136 | 0.098 | 0.494 | 0.082 | 0.530 | -0.005 | 0.211 | -0.114 | 0.035 | -0.114 | -0.165 | -0.052 | -0.246 | -0.052 | -0.157 | -0.007 | -0.274 | 0.025 |
| Bleak | 1047 | 2 | 1 | 2 | -0.355 | 0.048 | -0.203 | 0.085 | 0.125 | 0.095 | 0.510 | 0.082 | 0.517 | -0.011 | 0.212 | -0.100 | 0.038 | -0.118 | -0.165 | -0.050 | -0.245 | -0.053 | -0.158 | -0.005 | -0.279 | 0.027 |
| Bleak | 1048 | 2 | 1 | 2 | -0.352 | 0.027 | -0.227 | 0.074 | 0.130 | 0.137 | 0.527 | 0.048 | 0.507 | -0.034 | 0.207 | -0.074 | 0.042 | -0.080 | -0.160 | -0.038 | -0.230 | -0.065 | -0.164 | -0.006 | -0.281 | 0.011 |
| Bleak | 1049 | 2 | 1 | 2 | -0.342 | 0.056 | -0.204 | 0.083 | 0.130 | 0.096 | 0.514 | 0.083 | 0.515 | -0.010 | 0.218 | -0.097 | 0.027 | -0.117 | -0.173 | -0.053 | -0.236 | -0.055 | -0.171 | -0.009 | -0.279 | 0.023 |
| Bleak | 1050 | 2 | 1 | 2 | -0.355 | 0.023 | -0.226 | 0.086 | 0.131 | 0.133 | 0.517 | 0.056 | 0.510 | -0.032 | 0.212 | -0.085 | 0.040 | -0.086 | -0.152 | -0.043 | -0.232 | -0.063 | -0.163 | -0.004 | -0.281 | 0.014 |
| Bleak | 1051 | 2 | 1 | 2 | -0.349 | 0.027 | -0.228 | 0.085 | 0.119 | 0.137 | 0.522 | 0.053 | 0.513 | -0.039 | 0.207 | -0.073 | 0.047 | -0.089 | -0.161 | -0.043 | -0.221 | -0.064 | -0.166 | -0.004 | -0.283 | 0.010 |
| Bleak | 1052 | 2 | 1 | 2 | -0.346 | 0.031 | -0.209 | 0.078 | 0.131 | 0.109 | 0.512 | 0.066 | 0.511 | -0.016 | 0.216 | -0.095 | 0.056 | -0.107 | -0.170 | -0.030 | -0.244 | -0.055 | -0.171 | 0.002 | -0.286 | 0.018 |
| Bleak | 1053 | 2 | 1 | 2 | -0.357 | 0.023 | -0.227 | 0.081 | 0.140 | 0.133 | 0.518 | 0.051 | 0.505 | -0.038 | 0.209 | -0.074 | 0.052 | -0.077 | -0.156 | -0.036 | -0.231 | -0.066 | -0.163 | -0.005 | -0.289 | 0.008 |
| Bleak | 1054 | 2 | 1 | 2 | -0.353 | 0.038 | -0.229 | 0.086 | 0.140 | 0.128 | 0.512 | 0.070 | 0.515 | -0.025 | 0.197 | -0.097 | 0.041 | -0.100 | -0.157 | -0.048 | -0.240 | -0.065 | -0.153 | -0.003 | -0.273 | 0.016 |
| Bleak | 1055 | 2 | 1 | 2 | -0.363 | 0.010 | -0.243 | 0.082 | 0.127 | 0.142 | 0.521 | 0.049 | 0.507 | -0.049 | 0.206 | -0.076 | 0.053 | -0.077 | -0.146 | -0.031 | -0.216 | -0.064 | -0.161 | 0.007 | -0.285 | 0.006 |
| Bleak | 1056 | 2 | 1 | 2 | -0.353 | 0.019 | -0.219 | 0.085 | 0.140 | 0.125 | 0.509 | 0.065 | 0.515 | -0.032 | 0.210 | -0.091 | 0.043 | -0.093 | -0.159 | -0.034 | -0.236 | -0.057 | -0.163 | 0.001 | -0.285 | 0.011 |
| Bleak | 1057 | 2 | 1 | 2 | -0.345 | 0.061 | -0.216 | 0.081 | 0.154 | 0.080 | 0.496 | 0.091 | 0.512 | 0.002 | 0.227 | -0.116 | 0.036 | -0.123 | -0.171 | -0.042 | -0.240 | -0.051 | -0.168 | -0.013 | -0.285 | 0.030 |
| Bleak | 1058 | 2 | 1 | 2 | -0.353 | 0.049 | -0.209 | 0.089 | 0.119 | 0.096 | 0.503 | 0.083 | 0.537 | -0.010 | 0.199 | -0.109 | 0.031 | -0.110 | -0.160 | -0.045 | -0.247 | -0.058 | -0.150 | -0.007 | -0.270 | 0.023 |
| Bleak | 1059 | 2 | 1 | 2 | -0.334 | 0.062 | -0.196 | 0.082 | 0.136 | 0.091 | 0.501 | 0.093 | 0.520 | 0.002 | 0.221 | -0.122 | 0.031 | -0.128 | -0.179 | -0.042 | -0.240 | -0.060 | -0.175 | -0.010 | -0.284 | 0.032 |
| Bleak | 1060 | 2 | 1 | 2 | -0.330 | 0.072 | -0.217 | 0.083 | 0.147 | 0.074 | 0.500 | 0.091 | 0.524 | 0.007 | 0.218 | -0.112 | 0.031 | -0.117 | -0.182 | -0.053 | -0.242 | -0.054 | -0.173 | -0.023 | -0.276 | 0.032 |
| Bleak | 1031 | 2 | 1 | 3 | -0.352 | 0.030 | -0.228 | 0.074 | 0.140 | 0.125 | 0.519 | 0.055 | 0.512 | -0.033 | 0.212 | -0.071 | 0.030 | -0.087 | -0.158 | -0.032 | -0.229 | -0.068 | -0.160 | -0.003 | -0.285 | 0.010 |
| Bleak | 1032 | 2 | 1 | 3 | -0.351 | 0.035 | -0.207 | 0.086 | 0.122 | 0.107 | 0.509 | 0.080 | 0.531 | -0.022 | 0.198 | -0.103 | 0.036 | -0.105 | -0.160 | -0.038 | -0.228 | -0.063 | -0.166 | -0.002 | -0.282 | 0.024 |
| Bleak | 1033 | 2 | 1 | 3 | -0.339 | 0.052 | -0.215 | 0.083 | 0.127 | 0.105 | 0.518 | 0.077 | 0.514 | -0.014 | 0.213 | -0.095 | 0.032 | -0.112 | -0.165 | -0.046 | -0.237 | -0.054 | -0.166 | -0.015 | -0.283 | 0.020 |
| Bleak | 1034 | 2 | 1 | 3 | -0.325 | 0.069 | -0.228 | 0.080 | 0.134 | 0.102 | 0.509 | 0.085 | 0.515 | -0.009 | 0.209 | -0.108 | 0.051 | -0.116 | -0.170 | -0.053 | -0.248 | -0.052 | -0.170 | -0.027 | -0.278 | 0.027 |
| Bleak | 1035 | 2 | 1 | 3 | -0.346 | 0.053 | -0.208 | 0.083 | 0.110 | 0.098 | 0.512 | 0.079 | 0.528 | -0.009 | 0.203 | -0.106 | 0.043 | -0.108 | -0.165 | -0.050 | -0.253 | -0.059 | -0.152 | -0.009 | -0.272 | 0.029 |
| Bleak | 1036 | 2 | 1 | 3 | -0.352 | 0.044 | -0.223 | 0.081 | 0.130 | 0.113 | 0.516 | 0.071 | 0.517 | -0.018 | 0.208 | -0.095 | 0.030 | -0.100 | -0.156 | -0.049 | -0.250 | -0.061 | -0.154 | -0.010 | -0.265 | 0.024 |
| Bleak | 1037 | 2 | 1 | 3 | -0.350 | 0.025 | -0.219 | 0.077 | 0.127 | 0.115 | 0.514 | 0.067 | 0.519 | -0.029 | 0.214 | -0.089 | 0.035 | -0.096 | -0.162 | -0.034 | -0.240 | -0.057 | -0.160 | 0.003 | -0.278 | 0.016 |
| Bleak | 1038 | 2 | 1 | 3 | -0.347 | 0.051 | -0.227 | 0.084 | 0.161 | 0.102 | 0.500 | 0.068 | 0.508 | -0.010 | 0.219 | -0.089 | 0.049 | -0.104 | -0.171 | -0.054 | -0.236 | -0.053 | -0.170 | -0.016 | -0.286 | 0.021 |
| Bleak | 1039 | 2 | 1 | 3 | -0.349 | 0.067 | -0.207 | 0.085 | 0.129 | 0.101 | 0.492 | 0.081 | 0.517 | -0.006 | 0.240 | -0.108 | 0.031 | -0.121 | -0.168 | -0.064 | -0.243 | -0.059 | -0.165 | -0.007 | -0.278 | 0.030 |
| Bleak | 1040 | 2 | 1 | 3 | -0.360 | 0.038 | -0.227 | 0.084 | 0.140 | 0.112 | 0.510 | 0.068 | 0.514 | -0.026 | 0.207 | -0.088 | 0.044 | -0.091 | -0.165 | -0.042 | -0.230 | -0.063 | -0.153 | -0.008 | -0.280 | 0.017 |
| Bleak | 1041 | 2 | 1 | 3 | -0.357 | 0.043 | -0.202 | 0.088 | 0.129 | 0.114 | 0.514 | 0.076 | 0.511 | -0.022 | 0.204 | -0.099 | 0.053 | -0.108 | -0.165 | -0.041 | -0.232 | -0.057 | -0.166 | -0.006 | -0.288 | 0.012 |
| Bleak | 1042 | 2 | 1 | 3 | -0.344 | 0.050 | -0.211 | 0.080 | 0.120 | 0.107 | 0.522 | 0.076 | 0.512 | -0.024 | 0.216 | -0.086 | 0.037 | -0.104 | -0.168 | -0.047 | -0.241 | -0.058 | -0.160 | -0.014 | -0.280 | 0.020 |
| Bleak | 1043 | 2 | 1 | 3 | -0.351 | 0.037 | -0.222 | 0.080 | 0.126 | 0.114 | 0.516 | 0.066 | 0.531 | -0.027 | 0.192 | -0.088 | 0.033 | -0.082 | -0.162 | -0.045 | -0.231 | -0.062 | -0.155 | -0.008 | -0.277 | 0.014 |
| Bleak | 1044 | 2 | 1 | 3 | -0.356 | 0.033 | -0.223 | 0.076 | 0.135 | 0.117 | 0.516 | 0.058 | 0.506 | -0.027 | 0.218 | -0.080 | 0.049 | -0.087 | -0.160 | -0.036 | -0.237 | -0.060 | -0.164 | -0.011 | -0.284 | 0.017 |
| Bleak | 1045 | 2 | 1 | 3 | -0.337 | 0.038 | -0.222 | 0.078 | 0.103 | 0.127 | 0.531 | 0.066 | 0.512 | -0.037 | 0.217 | -0.086 | 0.039 | -0.088 | -0.176 | -0.046 | -0.221 | -0.060 | -0.166 | -0.004 | -0.279 | 0.012 |
| Bleak | 1046 | 2 | 1 | 3 | -0.349 | 0.056 | -0.213 | 0.087 | 0.132 | 0.099 | 0.496 | 0.081 | 0.529 | -0.004 | 0.212 | -0.113 | 0.033 | -0.114 | -0.163 | -0.051 | -0.244 | -0.054 | -0.158 | -0.009 | -0.275 | 0.023 |
| Bleak | 1047 | 2 | 1 | 3 | -0.355 | 0.052 | -0.200 | 0.085 | 0.125 | 0.097 | 0.515 | 0.081 | 0.510 | -0.009 | 0.219 | -0.100 | 0.032 | -0.117 | -0.167 | -0.050 | -0.244 | -0.060 | -0.154 | -0.008 | -0.281 | 0.027 |
| Bleak | 1048 | 2 | 1 | 3 | -0.353 | 0.026 | -0.224 | 0.075 | 0.134 | 0.138 | 0.527 | 0.048 | 0.501 | -0.032 | 0.211 | -0.074 | 0.045 | -0.086 | -0.160 | -0.045 | -0.229 | -0.063 | -0.167 | 0.003 | -0.284 | 0.010 |
| Bleak | 1049 | 2 | 1 | 3 | -0.341 | 0.055 | -0.208 | 0.087 | 0.133 | 0.097 | 0.515 | 0.081 | 0.510 | -0.007 | 0.220 | -0.100 | 0.031 | -0.119 | -0.171 | -0.054 | -0.235 | -0.052 | -0.170 | -0.013 | -0.282 | 0.026 |
| Bleak | 1050 | 2 | 1 | 3 | -0.349 | 0.025 | -0.231 | 0.086 | 0.123 | 0.132 | 0.534 | 0.057 | 0.499 | -0.033 | 0.215 | -0.083 | 0.035 | -0.087 | -0.154 | -0.041 | -0.232 | -0.063 | -0.162 | -0.005 | -0.277 | 0.011 |
| Bleak | 1051 | 2 | 1 | 3 | -0.346 | 0.026 | -0.230 | 0.081 | 0.110 | 0.140 | 0.523 | 0.051 | 0.519 | -0.039 | 0.203 | -0.074 | 0.050 | -0.090 | -0.157 | -0.039 | -0.226 | -0.061 | -0.164 | -0.007 | -0.282 | 0.011 |
| Bleak | 1052 | 2 | 1 | 3 | -0.345 | 0.028 | -0.204 | 0.082 | 0.155 | 0.108 | 0.506 | 0.070 | 0.514 | -0.015 | 0.215 | -0.093 | 0.035 | -0.103 | -0.168 | -0.036 | -0.250 | -0.053 | -0.173 | -0.005 | -0.283 | 0.018 |
| Bleak | 1053 | 2 | 1 | 3 | -0.358 | 0.019 | -0.224 | 0.079 | 0.136 | 0.132 | 0.519 | 0.050 | 0.507 | -0.037 | 0.209 | -0.075 | 0.052 | -0.078 | -0.154 | -0.033 | -0.235 | -0.064 | -0.162 | -0.002 | -0.289 | 0.010 |
| Bleak | 1054 | 2 | 1 | 3 | -0.348 | 0.042 | -0.243 | 0.086 | 0.132 | 0.126 | 0.514 | 0.069 | 0.516 | -0.026 | 0.203 | -0.098 | 0.036 | -0.098 | -0.153 | -0.043 | -0.237 | -0.064 | -0.147 | -0.009 | -0.273 | 0.016 |
| Bleak | 1055 | 2 | 1 | 3 | -0.363 | 0.007 | -0.235 | 0.087 | 0.133 | 0.141 | 0.524 | 0.051 | 0.505 | -0.047 | 0.205 | -0.076 | 0.042 | -0.075 | -0.150 | -0.033 | -0.219 | -0.066 | -0.156 | 0.006 | -0.288 | 0.005 |
| Bleak | 1056 | 2 | 1 | 3 | -0.354 | 0.025 | -0.214 | 0.084 | 0.132 | 0.122 | 0.514 | 0.064 | 0.510 | -0.029 | 0.217 | -0.090 | 0.040 | -0.094 | -0.155 | -0.033 | -0.239 | -0.058 | -0.165 | 0.000 | -0.287 | 0.010 |
| Bleak | 1057 | 2 | 1 | 3 | -0.347 | 0.061 | -0.210 | 0.080 | 0.153 | 0.079 | 0.506 | 0.093 | 0.506 | 0.001 | 0.228 | -0.113 | 0.027 | -0.121 | -0.171 | -0.042 | -0.237 | -0.054 | -0.167 | -0.016 | -0.286 | 0.031 |
| Bleak | 1058 | 2 | 1 | 3 | -0.354 | 0.053 | -0.198 | 0.091 | 0.126 | 0.100 | 0.501 | 0.085 | 0.535 | -0.011 | 0.198 | -0.110 | 0.030 | -0.108 | -0.164 | -0.045 | -0.247 | -0.064 | -0.154 | -0.013 | -0.273 | 0.023 |
| Bleak | 1059 | 2 | 1 | 3 | -0.330 | 0.061 | -0.197 | 0.084 | 0.132 | 0.091 | 0.507 | 0.095 | 0.516 | -0.001 | 0.221 | -0.116 | 0.031 | -0.134 | -0.177 | -0.045 | -0.243 | -0.052 | -0.175 | -0.016 | -0.285 | 0.033 |
| Bleak | 1060 | 2 | 1 | 3 | -0.333 | 0.069 | -0.210 | 0.084 | 0.132 | 0.075 | 0.505 | 0.091 | 0.524 | 0.003 | 0.216 | -0.113 | 0.039 | -0.115 | -0.182 | -0.056 | -0.242 | -0.054 | -0.169 | -0.016 | -0.278 | 0.031 |
| Bleak | 1131 | 2 | 2 | 1 | -0.341 | 0.042 | -0.227 | 0.072 | 0.135 | 0.116 | 0.509 | 0.073 | 0.511 | -0.029 | 0.221 | -0.096 | 0.044 | -0.094 | -0.166 | -0.047 | -0.236 | -0.063 | -0.163 | 0.008 | -0.288 | 0.017 |
| Bleak | 1132 | 2 | 2 | 1 | -0.354 | 0.014 | -0.237 | 0.074 | 0.134 | 0.115 | 0.521 | 0.050 | 0.511 | -0.030 | 0.210 | -0.079 | 0.049 | -0.077 | -0.165 | -0.036 | -0.219 | -0.062 | -0.164 | 0.016 | -0.286 | 0.015 |
| Bleak | 1133 | 2 | 2 | 1 | -0.345 | 0.034 | -0.229 | 0.077 | 0.157 | 0.118 | 0.501 | 0.067 | 0.513 | -0.020 | 0.219 | -0.098 | 0.036 | -0.093 | -0.162 | -0.045 | -0.236 | -0.057 | -0.167 | -0.003 | -0.287 | 0.020 |
| Bleak | 1134 | 2 | 2 | 1 | -0.350 | 0.027 | -0.220 | 0.073 | 0.130 | 0.111 | 0.509 | 0.063 | 0.524 | -0.029 | 0.218 | -0.081 | 0.032 | -0.093 | -0.164 | -0.035 | -0.232 | -0.058 | -0.166 | 0.008 | -0.282 | 0.014 |
| Bleak | 1135 | 2 | 2 | 1 | -0.348 | 0.018 | -0.226 | 0.077 | 0.129 | 0.119 | 0.513 | 0.054 | 0.512 | -0.028 | 0.232 | -0.081 | 0.029 | -0.093 | -0.166 | -0.033 | -0.225 | -0.061 | -0.162 | 0.014 | -0.289 | 0.013 |
| Bleak | 1136 | 2 | 2 | 1 | -0.350 | 0.014 | -0.221 | 0.076 | 0.135 | 0.133 | 0.520 | 0.059 | 0.512 | -0.034 | 0.207 | -0.089 | 0.035 | -0.084 | -0.163 | -0.042 | -0.225 | -0.065 | -0.163 | 0.018 | -0.287 | 0.014 |
| Bleak | 1137 | 2 | 2 | 1 | -0.350 | 0.033 | -0.237 | 0.071 | 0.142 | 0.116 | 0.514 | 0.067 | 0.515 | -0.027 | 0.199 | -0.087 | 0.047 | -0.097 | -0.162 | -0.041 | -0.237 | -0.061 | -0.152 | 0.007 | -0.281 | 0.018 |
| Bleak | 1138 | 2 | 2 | 1 | -0.354 | 0.034 | -0.233 | 0.083 | 0.128 | 0.128 | 0.512 | 0.059 | 0.510 | -0.027 | 0.218 | -0.089 | 0.041 | -0.098 | -0.160 | -0.048 | -0.221 | -0.057 | -0.157 | 0.003 | -0.285 | 0.013 |
| Bleak | 1139 | 2 | 2 | 1 | -0.344 | 0.029 | -0.226 | 0.074 | 0.139 | 0.124 | 0.514 | 0.064 | 0.511 | -0.033 | 0.218 | -0.089 | 0.037 | -0.084 | -0.162 | -0.039 | -0.233 | -0.061 | -0.168 | 0.001 | -0.286 | 0.013 |
| Bleak | 1140 | 2 | 2 | 1 | -0.344 | 0.019 | -0.245 | 0.070 | 0.148 | 0.136 | 0.531 | 0.050 | 0.488 | -0.032 | 0.221 | -0.084 | 0.041 | -0.082 | -0.164 | -0.036 | -0.218 | -0.059 | -0.167 | 0.010 | -0.291 | 0.009 |
| Bleak | 1141 | 2 | 2 | 1 | -0.345 | 0.032 | -0.246 | 0.069 | 0.137 | 0.122 | 0.513 | 0.058 | 0.513 | -0.031 | 0.221 | -0.081 | 0.036 | -0.088 | -0.164 | -0.045 | -0.232 | -0.057 | -0.154 | 0.000 | -0.279 | 0.020 |
| Bleak | 1142 | 2 | 2 | 1 | -0.344 | 0.039 | -0.218 | 0.071 | 0.110 | 0.108 | 0.511 | 0.071 | 0.532 | -0.021 | 0.202 | -0.096 | 0.045 | -0.101 | -0.159 | -0.050 | -0.247 | -0.053 | -0.155 | 0.008 | -0.278 | 0.023 |
| Bleak | 1143 | 2 | 2 | 1 | -0.338 | 0.031 | -0.221 | 0.074 | 0.134 | 0.115 | 0.507 | 0.069 | 0.527 | -0.020 | 0.213 | -0.097 | 0.028 | -0.097 | -0.171 | -0.048 | -0.235 | -0.053 | -0.166 | 0.005 | -0.279 | 0.021 |
| Bleak | 1144 | 2 | 2 | 1 | -0.348 | 0.045 | -0.220 | 0.066 | 0.138 | 0.098 | 0.517 | 0.068 | 0.516 | -0.022 | 0.210 | -0.085 | 0.042 | -0.081 | -0.167 | -0.047 | -0.233 | -0.058 | -0.171 | -0.001 | -0.284 | 0.018 |
| Bleak | 1145 | 2 | 2 | 1 | -0.332 | 0.014 | -0.206 | 0.071 | 0.135 | 0.117 | 0.516 | 0.069 | 0.525 | -0.025 | 0.219 | -0.096 | 0.047 | -0.106 | -0.223 | -0.066 | -0.148 | 0.000 | -0.265 | 0.010 | -0.267 | 0.011 |
| Bleak | 1146 | 2 | 2 | 1 | -0.342 | 0.044 | -0.213 | 0.075 | 0.135 | 0.098 | 0.506 | 0.074 | 0.525 | -0.013 | 0.214 | -0.098 | 0.032 | -0.099 | -0.167 | -0.047 | -0.241 | -0.056 | -0.161 | 0.003 | -0.287 | 0.019 |
| Bleak | 1147 | 2 | 2 | 1 | -0.347 | 0.047 | -0.221 | 0.075 | 0.130 | 0.102 | 0.507 | 0.073 | 0.513 | -0.016 | 0.210 | -0.104 | 0.068 | -0.099 | -0.171 | -0.045 | -0.228 | -0.063 | -0.167 | 0.002 | -0.293 | 0.027 |
| Bleak | 1148 | 2 | 2 | 1 | -0.350 | 0.019 | -0.224 | 0.074 | 0.129 | 0.127 | 0.525 | 0.051 | 0.509 | -0.035 | 0.217 | -0.076 | 0.036 | -0.075 | -0.165 | -0.038 | -0.220 | -0.064 | -0.169 | 0.007 | -0.288 | 0.012 |
| Bleak | 1149 | 2 | 2 | 1 | -0.349 | 0.034 | -0.223 | 0.083 | 0.143 | 0.114 | 0.505 | 0.073 | 0.510 | -0.021 | 0.225 | -0.098 | 0.036 | -0.107 | -0.171 | -0.049 | -0.237 | -0.054 | -0.160 | 0.001 | -0.278 | 0.023 |
| Bleak | 1150 | 2 | 2 | 1 | -0.341 | 0.030 | -0.220 | 0.075 | 0.130 | 0.104 | 0.525 | 0.065 | 0.517 | -0.023 | 0.213 | -0.083 | 0.028 | -0.084 | -0.176 | -0.039 | -0.229 | -0.056 | -0.168 | -0.005 | -0.280 | 0.017 |
| Bleak | 1151 | 2 | 2 | 1 | -0.333 | 0.050 | -0.242 | 0.073 | 0.145 | 0.084 | 0.493 | 0.079 | 0.526 | -0.015 | 0.223 | -0.099 | 0.052 | -0.101 | -0.173 | -0.039 | -0.239 | -0.051 | -0.171 | -0.006 | -0.280 | 0.024 |
| Bleak | 1152 | 2 | 2 | 1 | -0.337 | 0.028 | -0.236 | 0.072 | 0.129 | 0.119 | 0.519 | 0.059 | 0.508 | -0.030 | 0.221 | -0.078 | 0.050 | -0.094 | -0.177 | -0.043 | -0.229 | -0.057 | -0.166 | -0.001 | -0.283 | 0.024 |
| Bleak | 1153 | 2 | 2 | 1 | -0.350 | 0.036 | -0.235 | 0.081 | 0.143 | 0.107 | 0.520 | 0.067 | 0.510 | -0.015 | 0.207 | -0.096 | 0.029 | -0.097 | -0.154 | -0.042 | -0.224 | -0.056 | -0.163 | -0.004 | -0.282 | 0.018 |
| Bleak | 1154 | 2 | 2 | 1 | -0.344 | 0.028 | -0.229 | 0.070 | 0.130 | 0.120 | 0.521 | 0.063 | 0.516 | -0.029 | 0.210 | -0.090 | 0.030 | -0.085 | -0.163 | -0.035 | -0.227 | -0.063 | -0.162 | 0.003 | -0.284 | 0.016 |
| Bleak | 1155 | 2 | 2 | 1 | -0.343 | 0.033 | -0.233 | 0.075 | 0.135 | 0.120 | 0.515 | 0.064 | 0.516 | -0.025 | 0.211 | -0.091 | 0.037 | -0.096 | -0.165 | -0.045 | -0.222 | -0.053 | -0.165 | 0.000 | -0.286 | 0.017 |
| Bleak | 1156 | 2 | 2 | 1 | -0.355 | 0.029 | -0.225 | 0.071 | 0.137 | 0.115 | 0.518 | 0.061 | 0.512 | -0.027 | 0.217 | -0.087 | 0.032 | -0.081 | -0.167 | -0.037 | -0.231 | -0.059 | -0.159 | 0.001 | -0.280 | 0.014 |
| Bleak | 1157 | 2 | 2 | 1 | -0.349 | 0.046 | -0.226 | 0.082 | 0.133 | 0.098 | 0.486 | 0.072 | 0.533 | -0.005 | 0.217 | -0.117 | 0.050 | -0.106 | -0.167 | -0.043 | -0.233 | -0.058 | -0.160 | 0.003 | -0.284 | 0.027 |
| Bleak | 1158 | 2 | 2 | 1 | -0.359 | 0.029 | -0.228 | 0.079 | 0.131 | 0.123 | 0.490 | 0.061 | 0.505 | -0.026 | 0.249 | -0.099 | 0.058 | -0.102 | -0.158 | -0.039 | -0.250 | -0.054 | -0.151 | 0.003 | -0.286 | 0.024 |
| Bleak | 1159 | 2 | 2 | 1 | -0.347 | 0.035 | -0.236 | 0.064 | 0.134 | 0.102 | 0.518 | 0.061 | 0.509 | -0.021 | 0.228 | -0.087 | 0.033 | -0.080 | -0.156 | -0.036 | -0.245 | -0.062 | -0.160 | 0.003 | -0.278 | 0.023 |
| Bleak | 1160 | 2 | 2 | 1 | -0.344 | 0.043 | -0.234 | 0.073 | 0.128 | 0.112 | 0.512 | 0.066 | 0.519 | -0.020 | 0.218 | -0.093 | 0.035 | -0.100 | -0.166 | -0.040 | -0.230 | -0.051 | -0.158 | -0.009 | -0.280 | 0.018 |
| Bleak | 1131 | 2 | 2 | 2 | -0.350 | 0.043 | -0.222 | 0.079 | 0.139 | 0.114 | 0.505 | 0.069 | 0.507 | -0.029 | 0.223 | -0.088 | 0.060 | -0.097 | -0.177 | -0.040 | -0.223 | -0.064 | -0.170 | -0.008 | -0.291 | 0.021 |
| Bleak | 1132 | 2 | 2 | 2 | -0.354 | 0.019 | -0.230 | 0.079 | 0.123 | 0.113 | 0.526 | 0.054 | 0.509 | -0.030 | 0.211 | -0.080 | 0.049 | -0.080 | -0.165 | -0.036 | -0.224 | -0.059 | -0.159 | 0.006 | -0.286 | 0.014 |
| Bleak | 1133 | 2 | 2 | 2 | -0.343 | 0.040 | -0.216 | 0.079 | 0.151 | 0.114 | 0.509 | 0.074 | 0.509 | -0.018 | 0.215 | -0.103 | 0.035 | -0.094 | -0.167 | -0.046 | -0.241 | -0.058 | -0.165 | -0.008 | -0.287 | 0.020 |
| Bleak | 1134 | 2 | 2 | 2 | -0.354 | 0.025 | -0.221 | 0.075 | 0.129 | 0.108 | 0.512 | 0.063 | 0.519 | -0.029 | 0.219 | -0.081 | 0.037 | -0.091 | -0.164 | -0.034 | -0.231 | -0.057 | -0.159 | 0.005 | -0.287 | 0.016 |
| Bleak | 1135 | 2 | 2 | 2 | -0.354 | 0.016 | -0.210 | 0.078 | 0.141 | 0.115 | 0.510 | 0.055 | 0.517 | -0.026 | 0.222 | -0.078 | 0.030 | -0.084 | -0.173 | -0.033 | -0.231 | -0.061 | -0.162 | 0.005 | -0.291 | 0.015 |
| Bleak | 1136 | 2 | 2 | 2 | -0.349 | 0.015 | -0.204 | 0.083 | 0.134 | 0.129 | 0.521 | 0.064 | 0.513 | -0.031 | 0.207 | -0.088 | 0.028 | -0.091 | -0.168 | -0.041 | -0.232 | -0.063 | -0.161 | 0.008 | -0.288 | 0.015 |
| Bleak | 1137 | 2 | 2 | 2 | -0.351 | 0.031 | -0.229 | 0.074 | 0.135 | 0.111 | 0.513 | 0.066 | 0.516 | -0.024 | 0.206 | -0.090 | 0.047 | -0.095 | -0.161 | -0.043 | -0.236 | -0.060 | -0.158 | 0.006 | -0.282 | 0.023 |
| Bleak | 1138 | 2 | 2 | 2 | -0.354 | 0.032 | -0.216 | 0.089 | 0.123 | 0.127 | 0.514 | 0.061 | 0.514 | -0.028 | 0.215 | -0.088 | 0.041 | -0.096 | -0.167 | -0.046 | -0.228 | -0.058 | -0.159 | -0.006 | -0.284 | 0.013 |
| Bleak | 1139 | 2 | 2 | 2 | -0.345 | 0.032 | -0.223 | 0.074 | 0.127 | 0.122 | 0.511 | 0.062 | 0.518 | -0.032 | 0.219 | -0.086 | 0.038 | -0.086 | -0.159 | -0.036 | -0.236 | -0.062 | -0.165 | -0.005 | -0.286 | 0.017 |
| Bleak | 1140 | 2 | 2 | 2 | -0.344 | 0.014 | -0.234 | 0.073 | 0.139 | 0.137 | 0.527 | 0.050 | 0.499 | -0.039 | 0.215 | -0.076 | 0.047 | -0.080 | -0.168 | -0.035 | -0.220 | -0.056 | -0.171 | 0.005 | -0.291 | 0.006 |
| Bleak | 1141 | 2 | 2 | 2 | -0.353 | 0.025 | -0.216 | 0.079 | 0.133 | 0.122 | 0.511 | 0.061 | 0.513 | -0.032 | 0.213 | -0.079 | 0.051 | -0.094 | -0.168 | -0.042 | -0.238 | -0.056 | -0.162 | -0.001 | -0.285 | 0.018 |
| Bleak | 1142 | 2 | 2 | 2 | -0.342 | 0.037 | -0.224 | 0.075 | 0.111 | 0.109 | 0.520 | 0.070 | 0.525 | -0.021 | 0.202 | -0.094 | 0.043 | -0.100 | -0.161 | -0.045 | -0.241 | -0.056 | -0.154 | 0.002 | -0.278 | 0.024 |
| Bleak | 1143 | 2 | 2 | 2 | -0.341 | 0.034 | -0.213 | 0.078 | 0.138 | 0.113 | 0.511 | 0.070 | 0.526 | -0.019 | 0.204 | -0.094 | 0.028 | -0.095 | -0.172 | -0.046 | -0.233 | -0.059 | -0.165 | -0.001 | -0.281 | 0.019 |
| Bleak | 1144 | 2 | 2 | 2 | -0.345 | 0.041 | -0.223 | 0.071 | 0.136 | 0.099 | 0.515 | 0.070 | 0.519 | -0.027 | 0.210 | -0.079 | 0.040 | -0.083 | -0.168 | -0.046 | -0.244 | -0.056 | -0.158 | -0.009 | -0.283 | 0.019 |
| Bleak | 1145 | 2 | 2 | 2 | -0.348 | 0.024 | -0.211 | 0.083 | 0.133 | 0.115 | 0.518 | 0.065 | 0.518 | -0.022 | 0.206 | -0.085 | 0.034 | -0.099 | -0.173 | -0.041 | -0.234 | -0.057 | -0.163 | -0.002 | -0.280 | 0.020 |
| Bleak | 1146 | 2 | 2 | 2 | -0.346 | 0.045 | -0.211 | 0.078 | 0.159 | 0.093 | 0.501 | 0.079 | 0.517 | -0.011 | 0.215 | -0.099 | 0.034 | -0.097 | -0.169 | -0.043 | -0.247 | -0.056 | -0.164 | -0.010 | -0.288 | 0.022 |
| Bleak | 1147 | 2 | 2 | 2 | -0.347 | 0.049 | -0.215 | 0.077 | 0.132 | 0.100 | 0.504 | 0.075 | 0.526 | -0.015 | 0.214 | -0.102 | 0.033 | -0.094 | -0.166 | -0.050 | -0.225 | -0.060 | -0.167 | -0.007 | -0.288 | 0.028 |
| Bleak | 1148 | 2 | 2 | 2 | -0.349 | 0.023 | -0.217 | 0.074 | 0.128 | 0.126 | 0.524 | 0.059 | 0.514 | -0.039 | 0.210 | -0.077 | 0.033 | -0.074 | -0.160 | -0.038 | -0.231 | -0.062 | -0.166 | -0.004 | -0.286 | 0.012 |
| Bleak | 1149 | 2 | 2 | 2 | -0.350 | 0.035 | -0.209 | 0.089 | 0.140 | 0.114 | 0.509 | 0.072 | 0.508 | -0.018 | 0.225 | -0.098 | 0.032 | -0.103 | -0.177 | -0.047 | -0.231 | -0.056 | -0.163 | -0.007 | -0.284 | 0.019 |
| Bleak | 1150 | 2 | 2 | 2 | -0.345 | 0.023 | -0.214 | 0.077 | 0.123 | 0.102 | 0.525 | 0.066 | 0.519 | -0.027 | 0.212 | -0.081 | 0.031 | -0.085 | -0.171 | -0.036 | -0.232 | -0.051 | -0.168 | -0.006 | -0.281 | 0.018 |
| Bleak | 1151 | 2 | 2 | 2 | -0.338 | 0.049 | -0.217 | 0.077 | 0.141 | 0.085 | 0.501 | 0.081 | 0.522 | -0.013 | 0.221 | -0.097 | 0.040 | -0.105 | -0.181 | -0.040 | -0.233 | -0.054 | -0.171 | -0.007 | -0.286 | 0.024 |
| Bleak | 1152 | 2 | 2 | 2 | -0.335 | 0.029 | -0.227 | 0.074 | 0.133 | 0.119 | 0.526 | 0.058 | 0.503 | -0.028 | 0.221 | -0.074 | 0.042 | -0.092 | -0.176 | -0.044 | -0.236 | -0.057 | -0.168 | -0.004 | -0.283 | 0.020 |
| Bleak | 1153 | 2 | 2 | 2 | -0.352 | 0.037 | -0.226 | 0.083 | 0.134 | 0.104 | 0.521 | 0.071 | 0.511 | -0.020 | 0.206 | -0.094 | 0.038 | -0.095 | -0.160 | -0.040 | -0.229 | -0.060 | -0.160 | -0.003 | -0.283 | 0.017 |
| Bleak | 1154 | 2 | 2 | 2 | -0.345 | 0.031 | -0.211 | 0.076 | 0.130 | 0.114 | 0.516 | 0.069 | 0.520 | -0.026 | 0.213 | -0.092 | 0.026 | -0.088 | -0.165 | -0.039 | -0.230 | -0.060 | -0.169 | 0.001 | -0.287 | 0.015 |
| Bleak | 1155 | 2 | 2 | 2 | -0.343 | 0.030 | -0.224 | 0.077 | 0.136 | 0.114 | 0.529 | 0.066 | 0.499 | -0.023 | 0.220 | -0.089 | 0.030 | -0.095 | -0.166 | -0.043 | -0.228 | -0.055 | -0.167 | 0.000 | -0.286 | 0.017 |
| Bleak | 1156 | 2 | 2 | 2 | -0.354 | 0.032 | -0.217 | 0.079 | 0.115 | 0.110 | 0.516 | 0.062 | 0.521 | -0.027 | 0.219 | -0.087 | 0.039 | -0.086 | -0.165 | -0.038 | -0.230 | -0.057 | -0.165 | -0.004 | -0.279 | 0.015 |
| Bleak | 1157 | 2 | 2 | 2 | -0.348 | 0.051 | -0.213 | 0.080 | 0.139 | 0.093 | 0.491 | 0.079 | 0.534 | -0.003 | 0.212 | -0.113 | 0.036 | -0.109 | -0.173 | -0.048 | -0.230 | -0.054 | -0.162 | -0.003 | -0.286 | 0.026 |
| Bleak | 1158 | 2 | 2 | 2 | -0.363 | 0.033 | -0.229 | 0.077 | 0.164 | 0.123 | 0.491 | 0.062 | 0.496 | -0.024 | 0.242 | -0.093 | 0.048 | -0.100 | -0.164 | -0.038 | -0.236 | -0.065 | -0.158 | 0.003 | -0.292 | 0.022 |
| Bleak | 1159 | 2 | 2 | 2 | -0.349 | 0.032 | -0.227 | 0.065 | 0.132 | 0.100 | 0.518 | 0.061 | 0.507 | -0.021 | 0.238 | -0.084 | 0.027 | -0.085 | -0.166 | -0.031 | -0.243 | -0.060 | -0.156 | 0.000 | -0.280 | 0.022 |
| Bleak | 1160 | 2 | 2 | 2 | -0.346 | 0.039 | -0.227 | 0.074 | 0.139 | 0.111 | 0.510 | 0.069 | 0.515 | -0.019 | 0.222 | -0.095 | 0.027 | -0.101 | -0.170 | -0.036 | -0.232 | -0.057 | -0.158 | -0.001 | -0.281 | 0.016 |
| Bleak | 1131 | 2 | 2 | 3 | -0.344 | 0.043 | -0.213 | 0.076 | 0.134 | 0.113 | 0.501 | 0.070 | 0.524 | -0.025 | 0.217 | -0.089 | 0.043 | -0.096 | -0.170 | -0.045 | -0.244 | -0.057 | -0.160 | -0.006 | -0.286 | 0.017 |
| Bleak | 1132 | 2 | 2 | 3 | -0.353 | 0.014 | -0.236 | 0.080 | 0.131 | 0.111 | 0.517 | 0.053 | 0.516 | -0.031 | 0.211 | -0.078 | 0.050 | -0.078 | -0.167 | -0.031 | -0.224 | -0.058 | -0.160 | 0.004 | -0.285 | 0.015 |
| Bleak | 1133 | 2 | 2 | 3 | -0.344 | 0.039 | -0.208 | 0.080 | 0.146 | 0.113 | 0.498 | 0.071 | 0.525 | -0.017 | 0.218 | -0.097 | 0.026 | -0.100 | -0.171 | -0.047 | -0.235 | -0.057 | -0.166 | -0.005 | -0.289 | 0.019 |
| Bleak | 1134 | 2 | 2 | 3 | -0.349 | 0.026 | -0.222 | 0.075 | 0.131 | 0.111 | 0.514 | 0.064 | 0.518 | -0.029 | 0.216 | -0.082 | 0.034 | -0.091 | -0.165 | -0.037 | -0.237 | -0.058 | -0.158 | 0.003 | -0.283 | 0.017 |
| Bleak | 1135 | 2 | 2 | 3 | -0.353 | 0.016 | -0.211 | 0.078 | 0.134 | 0.121 | 0.517 | 0.051 | 0.508 | -0.027 | 0.223 | -0.074 | 0.040 | -0.090 | -0.172 | -0.034 | -0.233 | -0.060 | -0.164 | 0.008 | -0.290 | 0.013 |
| Bleak | 1136 | 2 | 2 | 3 | -0.347 | 0.015 | -0.211 | 0.082 | 0.142 | 0.127 | 0.520 | 0.066 | 0.521 | -0.031 | 0.192 | -0.088 | 0.027 | -0.088 | -0.164 | -0.038 | -0.228 | -0.062 | -0.165 | 0.001 | -0.287 | 0.016 |
| Bleak | 1137 | 2 | 2 | 3 | -0.350 | 0.033 | -0.225 | 0.075 | 0.140 | 0.108 | 0.516 | 0.068 | 0.513 | -0.023 | 0.204 | -0.087 | 0.045 | -0.099 | -0.163 | -0.044 | -0.233 | -0.060 | -0.164 | 0.006 | -0.283 | 0.022 |
| Bleak | 1138 | 2 | 2 | 3 | -0.352 | 0.034 | -0.228 | 0.083 | 0.132 | 0.131 | 0.517 | 0.059 | 0.508 | -0.028 | 0.210 | -0.088 | 0.045 | -0.093 | -0.164 | -0.047 | -0.226 | -0.055 | -0.159 | -0.008 | -0.285 | 0.012 |
| Bleak | 1139 | 2 | 2 | 3 | -0.353 | 0.028 | -0.220 | 0.078 | 0.147 | 0.121 | 0.509 | 0.067 | 0.508 | -0.031 | 0.213 | -0.085 | 0.048 | -0.097 | -0.165 | -0.039 | -0.228 | -0.060 | -0.168 | 0.003 | -0.292 | 0.017 |
| Bleak | 1140 | 2 | 2 | 3 | -0.346 | 0.019 | -0.231 | 0.076 | 0.143 | 0.135 | 0.533 | 0.050 | 0.486 | -0.031 | 0.218 | -0.082 | 0.050 | -0.081 | -0.163 | -0.038 | -0.227 | -0.060 | -0.171 | 0.007 | -0.292 | 0.007 |
| Bleak | 1141 | 2 | 2 | 3 | -0.355 | 0.030 | -0.221 | 0.080 | 0.143 | 0.121 | 0.511 | 0.061 | 0.508 | -0.032 | 0.217 | -0.075 | 0.042 | -0.093 | -0.164 | -0.044 | -0.235 | -0.058 | -0.159 | -0.010 | -0.288 | 0.018 |
| Bleak | 1142 | 2 | 2 | 3 | -0.344 | 0.035 | -0.220 | 0.077 | 0.122 | 0.107 | 0.511 | 0.072 | 0.527 | -0.021 | 0.204 | -0.097 | 0.042 | -0.099 | -0.164 | -0.041 | -0.238 | -0.053 | -0.161 | -0.002 | -0.280 | 0.023 |
| Bleak | 1143 | 2 | 2 | 3 | -0.334 | 0.036 | -0.221 | 0.076 | 0.135 | 0.111 | 0.508 | 0.071 | 0.532 | -0.020 | 0.200 | -0.096 | 0.034 | -0.096 | -0.171 | -0.045 | -0.231 | -0.058 | -0.168 | 0.006 | -0.283 | 0.016 |
| Bleak | 1144 | 2 | 2 | 3 | -0.347 | 0.039 | -0.218 | 0.073 | 0.143 | 0.100 | 0.515 | 0.068 | 0.518 | -0.025 | 0.201 | -0.077 | 0.047 | -0.085 | -0.172 | -0.048 | -0.235 | -0.058 | -0.165 | -0.007 | -0.287 | 0.020 |
| Bleak | 1145 | 2 | 2 | 3 | -0.349 | 0.029 | -0.221 | 0.081 | 0.134 | 0.117 | 0.516 | 0.063 | 0.517 | -0.019 | 0.203 | -0.089 | 0.037 | -0.104 | -0.168 | -0.041 | -0.227 | -0.059 | -0.164 | 0.000 | -0.279 | 0.021 |
| Bleak | 1146 | 2 | 2 | 3 | -0.345 | 0.043 | -0.212 | 0.078 | 0.145 | 0.094 | 0.503 | 0.078 | 0.524 | -0.014 | 0.209 | -0.100 | 0.035 | -0.099 | -0.167 | -0.045 | -0.239 | -0.057 | -0.163 | -0.001 | -0.290 | 0.022 |
| Bleak | 1147 | 2 | 2 | 3 | -0.349 | 0.048 | -0.218 | 0.078 | 0.147 | 0.105 | 0.497 | 0.075 | 0.524 | -0.015 | 0.209 | -0.101 | 0.040 | -0.098 | -0.164 | -0.051 | -0.233 | -0.061 | -0.166 | -0.009 | -0.287 | 0.029 |
| Bleak | 1148 | 2 | 2 | 3 | -0.350 | 0.018 | -0.221 | 0.076 | 0.127 | 0.127 | 0.520 | 0.053 | 0.509 | -0.037 | 0.221 | -0.078 | 0.040 | -0.074 | -0.163 | -0.034 | -0.229 | -0.062 | -0.166 | 0.000 | -0.289 | 0.012 |
| Bleak | 1149 | 2 | 2 | 3 | -0.347 | 0.033 | -0.216 | 0.084 | 0.140 | 0.116 | 0.504 | 0.072 | 0.512 | -0.025 | 0.228 | -0.092 | 0.036 | -0.105 | -0.172 | -0.044 | -0.236 | -0.056 | -0.165 | -0.003 | -0.282 | 0.020 |
| Bleak | 1150 | 2 | 2 | 3 | -0.344 | 0.025 | -0.216 | 0.075 | 0.138 | 0.104 | 0.520 | 0.066 | 0.520 | -0.025 | 0.211 | -0.083 | 0.025 | -0.081 | -0.173 | -0.040 | -0.234 | -0.057 | -0.166 | -0.001 | -0.281 | 0.016 |
| Bleak | 1151 | 2 | 2 | 3 | -0.339 | 0.051 | -0.218 | 0.076 | 0.148 | 0.087 | 0.504 | 0.080 | 0.516 | -0.010 | 0.221 | -0.098 | 0.042 | -0.103 | -0.180 | -0.047 | -0.237 | -0.050 | -0.171 | -0.010 | -0.286 | 0.023 |
| Bleak | 1152 | 2 | 2 | 3 | -0.339 | 0.032 | -0.220 | 0.073 | 0.139 | 0.123 | 0.520 | 0.060 | 0.504 | -0.029 | 0.218 | -0.075 | 0.047 | -0.093 | -0.177 | -0.050 | -0.234 | -0.059 | -0.173 | -0.003 | -0.285 | 0.021 |
| Bleak | 1153 | 2 | 2 | 3 | -0.350 | 0.036 | -0.227 | 0.084 | 0.142 | 0.103 | 0.511 | 0.068 | 0.518 | -0.014 | 0.206 | -0.097 | 0.033 | -0.097 | -0.160 | -0.043 | -0.228 | -0.059 | -0.161 | 0.000 | -0.285 | 0.019 |
| Bleak | 1154 | 2 | 2 | 3 | -0.342 | 0.031 | -0.215 | 0.073 | 0.132 | 0.122 | 0.516 | 0.067 | 0.516 | -0.027 | 0.205 | -0.088 | 0.046 | -0.102 | -0.163 | -0.035 | -0.238 | -0.061 | -0.172 | 0.001 | -0.285 | 0.018 |
| Bleak | 1155 | 2 | 2 | 3 | -0.347 | 0.034 | -0.225 | 0.080 | 0.145 | 0.112 | 0.515 | 0.067 | 0.507 | -0.021 | 0.218 | -0.090 | 0.035 | -0.099 | -0.166 | -0.044 | -0.221 | -0.056 | -0.171 | -0.002 | -0.289 | 0.018 |
| Bleak | 1156 | 2 | 2 | 3 | -0.354 | 0.028 | -0.228 | 0.080 | 0.134 | 0.115 | 0.516 | 0.063 | 0.506 | -0.030 | 0.223 | -0.084 | 0.043 | -0.086 | -0.170 | -0.045 | -0.233 | -0.055 | -0.154 | -0.004 | -0.282 | 0.018 |
| Bleak | 1157 | 2 | 2 | 3 | -0.348 | 0.048 | -0.209 | 0.084 | 0.144 | 0.095 | 0.495 | 0.077 | 0.531 | -0.001 | 0.208 | -0.111 | 0.033 | -0.109 | -0.171 | -0.051 | -0.233 | -0.054 | -0.163 | -0.004 | -0.286 | 0.026 |
| Bleak | 1158 | 2 | 2 | 3 | -0.363 | 0.026 | -0.212 | 0.082 | 0.149 | 0.125 | 0.491 | 0.063 | 0.502 | -0.028 | 0.240 | -0.093 | 0.053 | -0.102 | -0.163 | -0.039 | -0.248 | -0.063 | -0.160 | 0.007 | -0.289 | 0.020 |
| Bleak | 1159 | 2 | 2 | 3 | -0.354 | 0.030 | -0.230 | 0.069 | 0.147 | 0.102 | 0.511 | 0.060 | 0.502 | -0.024 | 0.240 | -0.084 | 0.034 | -0.077 | -0.162 | -0.036 | -0.244 | -0.061 | -0.161 | -0.003 | -0.284 | 0.022 |
| Bleak | 1160 | 2 | 2 | 3 | -0.352 | 0.039 | -0.221 | 0.079 | 0.135 | 0.112 | 0.515 | 0.069 | 0.510 | -0.018 | 0.218 | -0.095 | 0.031 | -0.104 | -0.167 | -0.039 | -0.227 | -0.057 | -0.159 | -0.003 | -0.285 | 0.018 |
| Bleak | 1221 | 2 | 3 | 1 | -0.357 | 0.045 | -0.204 | 0.088 | 0.133 | 0.120 | 0.501 | 0.070 | 0.518 | -0.026 | 0.226 | -0.088 | 0.028 | -0.103 | -0.167 | -0.056 | -0.234 | -0.071 | -0.160 | 0.004 | -0.282 | 0.018 |
| Bleak | 1222 | 2 | 3 | 1 | -0.354 | 0.041 | -0.222 | 0.074 | 0.142 | 0.114 | 0.514 | 0.062 | 0.510 | -0.034 | 0.217 | -0.072 | 0.044 | -0.083 | -0.164 | -0.041 | -0.225 | -0.061 | -0.171 | -0.012 | -0.291 | 0.012 |
| Bleak | 1223 | 2 | 3 | 1 | -0.353 | 0.020 | -0.226 | 0.075 | 0.139 | 0.127 | 0.529 | 0.043 | 0.508 | -0.034 | 0.201 | -0.065 | 0.043 | -0.067 | -0.155 | -0.036 | -0.225 | -0.066 | -0.169 | -0.004 | -0.293 | 0.007 |
| Bleak | 1224 | 2 | 3 | 1 | -0.347 | 0.050 | -0.207 | 0.078 | 0.136 | 0.096 | 0.512 | 0.083 | 0.522 | -0.011 | 0.203 | -0.107 | 0.029 | -0.099 | -0.168 | -0.042 | -0.231 | -0.062 | -0.162 | -0.014 | -0.288 | 0.029 |
| Bleak | 1225 | 2 | 3 | 1 | -0.347 | 0.067 | -0.201 | 0.078 | 0.130 | 0.081 | 0.512 | 0.085 | 0.512 | -0.002 | 0.226 | -0.100 | 0.030 | -0.114 | -0.171 | -0.054 | -0.243 | -0.055 | -0.165 | -0.020 | -0.283 | 0.033 |
| Bleak | 1226 | 2 | 3 | 1 | -0.349 | 0.042 | -0.208 | 0.078 | 0.160 | 0.102 | 0.509 | 0.073 | 0.508 | -0.013 | 0.213 | -0.097 | 0.034 | -0.094 | -0.168 | -0.050 | -0.234 | -0.064 | -0.173 | 0.001 | -0.292 | 0.022 |
| Bleak | 1227 | 2 | 3 | 1 | -0.351 | 0.044 | -0.203 | 0.079 | 0.139 | 0.106 | 0.510 | 0.072 | 0.507 | -0.016 | 0.227 | -0.096 | 0.034 | -0.098 | -0.166 | -0.045 | -0.233 | -0.064 | -0.172 | -0.009 | -0.292 | 0.027 |
| Bleak | 1228 | 2 | 3 | 1 | -0.358 | 0.015 | -0.223 | 0.076 | 0.137 | 0.145 | 0.540 | 0.041 | 0.501 | -0.046 | 0.197 | -0.052 | 0.034 | -0.068 | -0.155 | -0.043 | -0.223 | -0.066 | -0.162 | -0.007 | -0.289 | 0.005 |
| Bleak | 1229 | 2 | 3 | 1 | -0.346 | 0.055 | -0.205 | 0.077 | 0.123 | 0.115 | 0.523 | 0.064 | 0.511 | -0.019 | 0.211 | -0.082 | 0.037 | -0.094 | -0.164 | -0.057 | -0.235 | -0.073 | -0.168 | -0.008 | -0.287 | 0.023 |
| Bleak | 1230 | 2 | 3 | 1 | -0.356 | 0.049 | -0.225 | 0.083 | 0.138 | 0.115 | 0.519 | 0.070 | 0.508 | -0.022 | 0.205 | -0.092 | 0.031 | -0.097 | -0.160 | -0.044 | -0.214 | -0.072 | -0.156 | -0.009 | -0.291 | 0.019 |
| Bleak | 1232 | 2 | 3 | 1 | -0.355 | 0.049 | -0.213 | 0.074 | 0.135 | 0.117 | 0.511 | 0.069 | 0.510 | -0.023 | 0.211 | -0.094 | 0.048 | -0.093 | -0.164 | -0.045 | -0.229 | -0.067 | -0.162 | -0.008 | -0.293 | 0.021 |
| Bleak | 1233 | 2 | 3 | 1 | -0.357 | 0.034 | -0.217 | 0.080 | 0.149 | 0.121 | 0.506 | 0.062 | 0.514 | -0.026 | 0.211 | -0.086 | 0.039 | -0.085 | -0.163 | -0.044 | -0.235 | -0.064 | -0.161 | -0.006 | -0.287 | 0.015 |
| Bleak | 1234 | 2 | 3 | 1 | -0.363 | 0.041 | -0.226 | 0.081 | 0.154 | 0.124 | 0.518 | 0.063 | 0.501 | -0.024 | 0.206 | -0.090 | 0.033 | -0.088 | -0.155 | -0.053 | -0.222 | -0.060 | -0.156 | -0.008 | -0.288 | 0.013 |
| Bleak | 1235 | 2 | 3 | 1 | -0.348 | 0.055 | -0.203 | 0.078 | 0.118 | 0.099 | 0.512 | 0.073 | 0.520 | -0.015 | 0.220 | -0.087 | 0.036 | -0.105 | -0.172 | -0.055 | -0.241 | -0.057 | -0.160 | -0.013 | -0.282 | 0.026 |
| Bleak | 1236 | 2 | 3 | 1 | -0.358 | 0.019 | -0.217 | 0.088 | 0.133 | 0.123 | 0.528 | 0.055 | 0.510 | -0.029 | 0.203 | -0.079 | 0.030 | -0.081 | -0.162 | -0.042 | -0.223 | -0.062 | -0.155 | 0.004 | -0.289 | 0.006 |
| Bleak | 1237 | 2 | 3 | 1 | -0.356 | 0.035 | -0.218 | 0.081 | 0.152 | 0.128 | 0.525 | 0.058 | 0.496 | -0.031 | 0.204 | -0.077 | 0.045 | -0.082 | -0.163 | -0.047 | -0.227 | -0.069 | -0.164 | -0.007 | -0.293 | 0.011 |
| Bleak | 1238 | 2 | 3 | 1 | -0.354 | 0.026 | -0.221 | 0.080 | 0.151 | 0.122 | 0.515 | 0.060 | 0.502 | -0.028 | 0.221 | -0.085 | 0.035 | -0.084 | -0.166 | -0.045 | -0.232 | -0.059 | -0.162 | -0.001 | -0.289 | 0.014 |
| Bleak | 1239 | 2 | 3 | 1 | -0.350 | 0.048 | -0.218 | 0.078 | 0.140 | 0.116 | 0.522 | 0.066 | 0.499 | -0.022 | 0.216 | -0.083 | 0.040 | -0.097 | -0.165 | -0.049 | -0.234 | -0.064 | -0.162 | -0.012 | -0.288 | 0.019 |
| Bleak | 1240 | 2 | 3 | 1 | -0.354 | 0.032 | -0.224 | 0.076 | 0.141 | 0.130 | 0.522 | 0.054 | 0.510 | -0.037 | 0.195 | -0.073 | 0.050 | -0.076 | -0.156 | -0.043 | -0.226 | -0.062 | -0.167 | -0.005 | -0.292 | 0.004 |
| Bleak | 1241 | 2 | 3 | 1 | -0.357 | 0.034 | -0.213 | 0.086 | 0.137 | 0.118 | 0.518 | 0.068 | 0.516 | -0.030 | 0.211 | -0.085 | 0.018 | -0.082 | -0.166 | -0.054 | -0.228 | -0.066 | -0.157 | -0.004 | -0.279 | 0.016 |
| Bleak | 1242 | 2 | 3 | 1 | -0.351 | 0.043 | -0.212 | 0.081 | 0.149 | 0.103 | 0.510 | 0.072 | 0.512 | -0.011 | 0.210 | -0.095 | 0.034 | -0.105 | -0.165 | -0.042 | -0.230 | -0.066 | -0.166 | -0.009 | -0.290 | 0.029 |
| Bleak | 1243 | 2 | 3 | 1 | -0.340 | 0.063 | -0.202 | 0.078 | 0.114 | 0.090 | 0.501 | 0.080 | 0.530 | -0.001 | 0.227 | -0.108 | 0.033 | -0.119 | -0.170 | -0.049 | -0.245 | -0.050 | -0.170 | -0.013 | -0.278 | 0.029 |
| Bleak | 1244 | 2 | 3 | 1 | -0.350 | 0.038 | -0.218 | 0.084 | 0.130 | 0.127 | 0.522 | 0.066 | 0.518 | -0.027 | 0.196 | -0.089 | 0.029 | -0.090 | -0.159 | -0.045 | -0.229 | -0.062 | -0.158 | -0.014 | -0.281 | 0.013 |
| Bleak | 1245 | 2 | 3 | 1 | -0.367 | 0.000 | -0.254 | 0.074 | 0.170 | 0.155 | 0.521 | 0.030 | 0.481 | -0.049 | 0.214 | -0.063 | 0.064 | -0.057 | -0.148 | -0.034 | -0.215 | -0.061 | -0.163 | 0.005 | -0.303 | 0.001 |
| Bleak | 1246 | 2 | 3 | 1 | -0.343 | 0.060 | -0.197 | 0.082 | 0.137 | 0.086 | 0.492 | 0.092 | 0.538 | 0.003 | 0.207 | -0.122 | 0.025 | -0.122 | -0.176 | -0.046 | -0.234 | -0.056 | -0.165 | -0.009 | -0.283 | 0.030 |
| Bleak | 1247 | 2 | 3 | 1 | -0.353 | 0.025 | -0.219 | 0.078 | 0.144 | 0.125 | 0.515 | 0.061 | 0.509 | -0.033 | 0.211 | -0.082 | 0.039 | -0.082 | -0.165 | -0.043 | -0.237 | -0.060 | -0.157 | -0.003 | -0.288 | 0.013 |
| Bleak | 1248 | 2 | 3 | 1 | -0.357 | 0.055 | -0.213 | 0.081 | 0.125 | 0.091 | 0.499 | 0.082 | 0.532 | -0.006 | 0.207 | -0.115 | 0.033 | -0.107 | -0.161 | -0.047 | -0.230 | -0.064 | -0.158 | -0.006 | -0.278 | 0.033 |
| Bleak | 1249 | 2 | 3 | 1 | -0.348 | 0.065 | -0.214 | 0.081 | 0.145 | 0.092 | 0.495 | 0.079 | 0.531 | -0.003 | 0.205 | -0.105 | 0.033 | -0.104 | -0.166 | -0.057 | -0.234 | -0.062 | -0.165 | -0.010 | -0.284 | 0.025 |
| Bleak | 1250 | 2 | 3 | 1 | -0.343 | 0.045 | -0.227 | 0.074 | 0.139 | 0.105 | 0.500 | 0.066 | 0.522 | -0.017 | 0.223 | -0.093 | 0.038 | -0.093 | -0.164 | -0.046 | -0.235 | -0.057 | -0.165 | -0.008 | -0.287 | 0.023 |
| Bleak | 1250 | 2 | 3 | 1 | -0.348 | 0.042 | -0.218 | 0.085 | 0.140 | 0.111 | 0.507 | 0.067 | 0.508 | -0.021 | 0.220 | -0.088 | 0.049 | -0.098 | -0.168 | -0.051 | -0.237 | -0.059 | -0.164 | -0.005 | -0.290 | 0.018 |
| Bleak | 1221 | 2 | 3 | 2 | -0.356 | 0.047 | -0.208 | 0.089 | 0.128 | 0.121 | 0.507 | 0.070 | 0.520 | -0.026 | 0.214 | -0.087 | 0.030 | -0.101 | -0.164 | -0.055 | -0.233 | -0.070 | -0.158 | -0.009 | -0.279 | 0.020 |
| Bleak | 1222 | 2 | 3 | 2 | -0.354 | 0.041 | -0.225 | 0.076 | 0.149 | 0.115 | 0.507 | 0.063 | 0.511 | -0.035 | 0.221 | -0.072 | 0.038 | -0.085 | -0.168 | -0.047 | -0.218 | -0.061 | -0.171 | -0.008 | -0.291 | 0.012 |
| Bleak | 1223 | 2 | 3 | 2 | -0.350 | 0.020 | -0.229 | 0.072 | 0.146 | 0.129 | 0.519 | 0.048 | 0.514 | -0.041 | 0.202 | -0.064 | 0.044 | -0.068 | -0.157 | -0.036 | -0.225 | -0.065 | -0.171 | -0.005 | -0.294 | 0.009 |
| Bleak | 1224 | 2 | 3 | 2 | -0.347 | 0.051 | -0.206 | 0.080 | 0.137 | 0.096 | 0.511 | 0.083 | 0.520 | -0.010 | 0.207 | -0.109 | 0.029 | -0.099 | -0.166 | -0.045 | -0.232 | -0.063 | -0.164 | -0.010 | -0.289 | 0.027 |
| Bleak | 1225 | 2 | 3 | 2 | -0.348 | 0.070 | -0.198 | 0.077 | 0.129 | 0.085 | 0.509 | 0.085 | 0.512 | -0.003 | 0.230 | -0.101 | 0.030 | -0.115 | -0.174 | -0.051 | -0.236 | -0.057 | -0.167 | -0.024 | -0.287 | 0.034 |
| Bleak | 1226 | 2 | 3 | 2 | -0.345 | 0.048 | -0.209 | 0.077 | 0.151 | 0.104 | 0.512 | 0.077 | 0.510 | -0.016 | 0.212 | -0.097 | 0.032 | -0.096 | -0.166 | -0.045 | -0.232 | -0.062 | -0.171 | -0.013 | -0.294 | 0.023 |
| Bleak | 1227 | 2 | 3 | 2 | -0.350 | 0.046 | -0.207 | 0.078 | 0.140 | 0.106 | 0.508 | 0.073 | 0.509 | -0.015 | 0.225 | -0.097 | 0.032 | -0.103 | -0.164 | -0.045 | -0.233 | -0.065 | -0.167 | -0.005 | -0.292 | 0.025 |
| Bleak | 1228 | 2 | 3 | 2 | -0.360 | 0.017 | -0.221 | 0.077 | 0.139 | 0.143 | 0.532 | 0.042 | 0.505 | -0.049 | 0.201 | -0.050 | 0.035 | -0.068 | -0.159 | -0.043 | -0.221 | -0.065 | -0.161 | -0.005 | -0.291 | 0.002 |
| Bleak | 1229 | 2 | 3 | 2 | -0.351 | 0.055 | -0.207 | 0.078 | 0.127 | 0.118 | 0.522 | 0.066 | 0.511 | -0.023 | 0.205 | -0.081 | 0.040 | -0.095 | -0.159 | -0.053 | -0.235 | -0.072 | -0.165 | -0.016 | -0.288 | 0.024 |
| Bleak | 1230 | 2 | 3 | 2 | -0.354 | 0.045 | -0.213 | 0.082 | 0.144 | 0.117 | 0.511 | 0.071 | 0.515 | -0.021 | 0.206 | -0.095 | 0.029 | -0.093 | -0.161 | -0.047 | -0.233 | -0.066 | -0.155 | -0.012 | -0.288 | 0.018 |
| Bleak | 1232 | 2 | 3 | 2 | -0.350 | 0.049 | -0.214 | 0.070 | 0.140 | 0.115 | 0.513 | 0.070 | 0.504 | -0.022 | 0.217 | -0.093 | 0.048 | -0.096 | -0.163 | -0.040 | -0.241 | -0.062 | -0.163 | -0.011 | -0.292 | 0.021 |
| Bleak | 1233 | 2 | 3 | 2 | -0.353 | 0.032 | -0.213 | 0.081 | 0.141 | 0.119 | 0.510 | 0.069 | 0.518 | -0.033 | 0.208 | -0.086 | 0.036 | -0.085 | -0.165 | -0.045 | -0.236 | -0.062 | -0.161 | -0.004 | -0.284 | 0.014 |
| Bleak | 1234 | 2 | 3 | 2 | -0.361 | 0.039 | -0.222 | 0.079 | 0.154 | 0.127 | 0.510 | 0.069 | 0.512 | -0.029 | 0.199 | -0.092 | 0.038 | -0.090 | -0.156 | -0.048 | -0.227 | -0.060 | -0.158 | -0.005 | -0.287 | 0.011 |
| Bleak | 1235 | 2 | 3 | 2 | -0.345 | 0.057 | -0.201 | 0.077 | 0.124 | 0.099 | 0.513 | 0.074 | 0.518 | -0.014 | 0.219 | -0.085 | 0.036 | -0.106 | -0.175 | -0.051 | -0.243 | -0.057 | -0.165 | -0.019 | -0.282 | 0.026 |
| Bleak | 1236 | 2 | 3 | 2 | -0.358 | 0.025 | -0.224 | 0.081 | 0.129 | 0.123 | 0.529 | 0.058 | 0.513 | -0.035 | 0.203 | -0.075 | 0.025 | -0.082 | -0.156 | -0.043 | -0.220 | -0.060 | -0.152 | -0.002 | -0.287 | 0.010 |
| Bleak | 1237 | 2 | 3 | 2 | -0.356 | 0.036 | -0.220 | 0.083 | 0.154 | 0.131 | 0.522 | 0.056 | 0.496 | -0.028 | 0.210 | -0.079 | 0.040 | -0.081 | -0.162 | -0.044 | -0.227 | -0.068 | -0.164 | -0.017 | -0.294 | 0.012 |
| Bleak | 1238 | 2 | 3 | 2 | -0.355 | 0.025 | -0.225 | 0.081 | 0.149 | 0.122 | 0.509 | 0.063 | 0.508 | -0.032 | 0.218 | -0.084 | 0.040 | -0.086 | -0.168 | -0.044 | -0.229 | -0.060 | -0.161 | 0.000 | -0.287 | 0.015 |
| Bleak | 1239 | 2 | 3 | 2 | -0.349 | 0.046 | -0.220 | 0.079 | 0.146 | 0.116 | 0.516 | 0.066 | 0.497 | -0.020 | 0.222 | -0.086 | 0.044 | -0.099 | -0.167 | -0.051 | -0.239 | -0.062 | -0.161 | -0.007 | -0.289 | 0.020 |
| Bleak | 1240 | 2 | 3 | 2 | -0.354 | 0.029 | -0.224 | 0.076 | 0.146 | 0.130 | 0.518 | 0.056 | 0.515 | -0.040 | 0.197 | -0.072 | 0.039 | -0.073 | -0.157 | -0.043 | -0.224 | -0.062 | -0.166 | -0.007 | -0.291 | 0.005 |
| Bleak | 1241 | 2 | 3 | 2 | -0.354 | 0.040 | -0.214 | 0.083 | 0.147 | 0.119 | 0.514 | 0.071 | 0.513 | -0.029 | 0.210 | -0.086 | 0.022 | -0.085 | -0.168 | -0.053 | -0.236 | -0.066 | -0.154 | -0.013 | -0.281 | 0.018 |
| Bleak | 1242 | 2 | 3 | 2 | -0.353 | 0.040 | -0.205 | 0.081 | 0.154 | 0.105 | 0.509 | 0.076 | 0.509 | -0.014 | 0.209 | -0.096 | 0.036 | -0.107 | -0.165 | -0.040 | -0.232 | -0.066 | -0.168 | -0.010 | -0.294 | 0.031 |
| Bleak | 1243 | 2 | 3 | 2 | -0.341 | 0.060 | -0.200 | 0.079 | 0.146 | 0.087 | 0.500 | 0.086 | 0.522 | 0.000 | 0.219 | -0.107 | 0.029 | -0.116 | -0.177 | -0.051 | -0.248 | -0.049 | -0.168 | -0.017 | -0.282 | 0.028 |
| Bleak | 1244 | 2 | 3 | 2 | -0.353 | 0.038 | -0.216 | 0.087 | 0.129 | 0.125 | 0.518 | 0.065 | 0.514 | -0.028 | 0.206 | -0.088 | 0.034 | -0.093 | -0.162 | -0.045 | -0.228 | -0.061 | -0.161 | -0.012 | -0.284 | 0.012 |
| Bleak | 1245 | 2 | 3 | 2 | -0.365 | 0.002 | -0.256 | 0.074 | 0.171 | 0.155 | 0.515 | 0.033 | 0.487 | -0.049 | 0.213 | -0.065 | 0.061 | -0.061 | -0.147 | -0.032 | -0.217 | -0.064 | -0.161 | 0.000 | -0.301 | 0.006 |
| Bleak | 1246 | 2 | 3 | 2 | -0.341 | 0.059 | -0.200 | 0.084 | 0.145 | 0.090 | 0.495 | 0.093 | 0.540 | 0.004 | 0.197 | -0.120 | 0.017 | -0.123 | -0.174 | -0.045 | -0.236 | -0.057 | -0.162 | -0.018 | -0.281 | 0.032 |
| Bleak | 1247 | 2 | 3 | 2 | -0.353 | 0.023 | -0.219 | 0.077 | 0.150 | 0.124 | 0.515 | 0.061 | 0.509 | -0.031 | 0.203 | -0.082 | 0.045 | -0.083 | -0.161 | -0.040 | -0.239 | -0.058 | -0.161 | -0.008 | -0.289 | 0.016 |
| Bleak | 1248 | 2 | 3 | 2 | -0.357 | 0.056 | -0.198 | 0.082 | 0.133 | 0.092 | 0.494 | 0.081 | 0.532 | -0.002 | 0.214 | -0.112 | 0.028 | -0.109 | -0.165 | -0.052 | -0.238 | -0.064 | -0.161 | -0.007 | -0.281 | 0.034 |
| Bleak | 1249 | 2 | 3 | 2 | -0.351 | 0.064 | -0.208 | 0.077 | 0.140 | 0.088 | 0.500 | 0.081 | 0.526 | -0.006 | 0.213 | -0.100 | 0.030 | -0.105 | -0.170 | -0.055 | -0.236 | -0.059 | -0.157 | -0.014 | -0.286 | 0.028 |
| Bleak | 1250 | 2 | 3 | 2 | -0.350 | 0.045 | -0.217 | 0.081 | 0.146 | 0.104 | 0.502 | 0.071 | 0.511 | -0.021 | 0.230 | -0.089 | 0.037 | -0.096 | -0.164 | -0.050 | -0.236 | -0.057 | -0.165 | -0.013 | -0.291 | 0.025 |
| Bleak | 1250 | 2 | 3 | 2 | -0.347 | 0.044 | -0.220 | 0.080 | 0.142 | 0.106 | 0.506 | 0.076 | 0.515 | -0.026 | 0.214 | -0.091 | 0.041 | -0.100 | -0.161 | -0.044 | -0.234 | -0.064 | -0.167 | 0.002 | -0.289 | 0.016 |
| Bleak | 1221 | 2 | 3 | 3 | -0.356 | 0.046 | -0.208 | 0.088 | 0.132 | 0.121 | 0.500 | 0.069 | 0.523 | -0.024 | 0.214 | -0.088 | 0.033 | -0.107 | -0.161 | -0.057 | -0.235 | -0.070 | -0.162 | 0.003 | -0.280 | 0.019 |
| Bleak | 1222 | 2 | 3 | 3 | -0.357 | 0.039 | -0.223 | 0.073 | 0.148 | 0.116 | 0.509 | 0.061 | 0.504 | -0.028 | 0.222 | -0.077 | 0.048 | -0.094 | -0.166 | -0.045 | -0.230 | -0.057 | -0.165 | -0.001 | -0.291 | 0.013 |
| Bleak | 1223 | 2 | 3 | 3 | -0.348 | 0.022 | -0.231 | 0.070 | 0.138 | 0.127 | 0.519 | 0.044 | 0.519 | -0.036 | 0.209 | -0.066 | 0.034 | -0.070 | -0.154 | -0.036 | -0.221 | -0.067 | -0.172 | 0.005 | -0.291 | 0.008 |
| Bleak | 1224 | 2 | 3 | 3 | -0.346 | 0.056 | -0.206 | 0.080 | 0.132 | 0.094 | 0.510 | 0.084 | 0.525 | -0.012 | 0.205 | -0.107 | 0.029 | -0.099 | -0.165 | -0.044 | -0.231 | -0.064 | -0.163 | -0.014 | -0.289 | 0.026 |
| Bleak | 1225 | 2 | 3 | 3 | -0.347 | 0.071 | -0.200 | 0.075 | 0.127 | 0.083 | 0.511 | 0.086 | 0.509 | -0.003 | 0.229 | -0.103 | 0.037 | -0.119 | -0.168 | -0.056 | -0.242 | -0.055 | -0.168 | -0.010 | -0.288 | 0.031 |
| Bleak | 1226 | 2 | 3 | 3 | -0.351 | 0.048 | -0.207 | 0.078 | 0.146 | 0.103 | 0.510 | 0.073 | 0.509 | -0.015 | 0.214 | -0.095 | 0.040 | -0.099 | -0.166 | -0.047 | -0.230 | -0.061 | -0.170 | -0.007 | -0.296 | 0.022 |
| Bleak | 1227 | 2 | 3 | 3 | -0.349 | 0.047 | -0.204 | 0.080 | 0.140 | 0.104 | 0.511 | 0.077 | 0.506 | -0.016 | 0.227 | -0.098 | 0.032 | -0.103 | -0.169 | -0.046 | -0.233 | -0.063 | -0.171 | -0.008 | -0.289 | 0.027 |
| Bleak | 1228 | 2 | 3 | 3 | -0.359 | 0.023 | -0.233 | 0.070 | 0.141 | 0.143 | 0.531 | 0.042 | 0.506 | -0.047 | 0.200 | -0.050 | 0.039 | -0.074 | -0.155 | -0.041 | -0.219 | -0.066 | -0.163 | -0.004 | -0.286 | 0.003 |
| Bleak | 1229 | 2 | 3 | 3 | -0.342 | 0.061 | -0.207 | 0.075 | 0.122 | 0.113 | 0.531 | 0.072 | 0.506 | -0.024 | 0.207 | -0.081 | 0.041 | -0.093 | -0.169 | -0.056 | -0.241 | -0.071 | -0.164 | -0.022 | -0.283 | 0.025 |
| Bleak | 1230 | 2 | 3 | 3 | -0.354 | 0.047 | -0.214 | 0.077 | 0.143 | 0.115 | 0.513 | 0.071 | 0.512 | -0.021 | 0.208 | -0.095 | 0.033 | -0.097 | -0.161 | -0.047 | -0.233 | -0.065 | -0.159 | 0.000 | -0.288 | 0.014 |
| Bleak | 1232 | 2 | 3 | 3 | -0.350 | 0.047 | -0.211 | 0.072 | 0.128 | 0.113 | 0.518 | 0.070 | 0.503 | -0.021 | 0.216 | -0.094 | 0.054 | -0.098 | -0.163 | -0.044 | -0.244 | -0.061 | -0.162 | -0.004 | -0.289 | 0.021 |
| Bleak | 1233 | 2 | 3 | 3 | -0.352 | 0.034 | -0.213 | 0.080 | 0.137 | 0.119 | 0.506 | 0.068 | 0.524 | -0.033 | 0.207 | -0.085 | 0.037 | -0.088 | -0.160 | -0.048 | -0.235 | -0.063 | -0.167 | 0.002 | -0.283 | 0.013 |
| Bleak | 1234 | 2 | 3 | 3 | -0.364 | 0.039 | -0.223 | 0.079 | 0.159 | 0.128 | 0.517 | 0.064 | 0.500 | -0.021 | 0.205 | -0.096 | 0.030 | -0.092 | -0.154 | -0.047 | -0.222 | -0.062 | -0.160 | -0.004 | -0.288 | 0.013 |
| Bleak | 1235 | 2 | 3 | 3 | -0.347 | 0.056 | -0.206 | 0.079 | 0.130 | 0.097 | 0.510 | 0.072 | 0.514 | -0.014 | 0.225 | -0.084 | 0.039 | -0.105 | -0.176 | -0.053 | -0.245 | -0.058 | -0.164 | -0.014 | -0.281 | 0.025 |
| Bleak | 1236 | 2 | 3 | 3 | -0.358 | 0.025 | -0.226 | 0.081 | 0.120 | 0.121 | 0.534 | 0.056 | 0.506 | -0.032 | 0.207 | -0.079 | 0.031 | -0.086 | -0.151 | -0.039 | -0.220 | -0.062 | -0.156 | 0.010 | -0.288 | 0.005 |
| Bleak | 1237 | 2 | 3 | 3 | -0.352 | 0.032 | -0.218 | 0.080 | 0.146 | 0.127 | 0.530 | 0.056 | 0.498 | -0.031 | 0.200 | -0.077 | 0.043 | -0.076 | -0.160 | -0.051 | -0.230 | -0.070 | -0.166 | -0.003 | -0.291 | 0.012 |
| Bleak | 1238 | 2 | 3 | 3 | -0.353 | 0.027 | -0.223 | 0.077 | 0.150 | 0.122 | 0.508 | 0.060 | 0.518 | -0.029 | 0.209 | -0.085 | 0.032 | -0.081 | -0.162 | -0.047 | -0.230 | -0.060 | -0.162 | 0.003 | -0.287 | 0.013 |
| Bleak | 1239 | 2 | 3 | 3 | -0.352 | 0.048 | -0.219 | 0.079 | 0.141 | 0.113 | 0.520 | 0.065 | 0.494 | -0.018 | 0.216 | -0.086 | 0.056 | -0.103 | -0.163 | -0.048 | -0.237 | -0.063 | -0.165 | -0.007 | -0.291 | 0.020 |
| Bleak | 1240 | 2 | 3 | 3 | -0.354 | 0.029 | -0.226 | 0.074 | 0.137 | 0.128 | 0.515 | 0.054 | 0.520 | -0.037 | 0.203 | -0.075 | 0.039 | -0.076 | -0.154 | -0.040 | -0.220 | -0.063 | -0.168 | 0.002 | -0.291 | 0.004 |
| Bleak | 1241 | 2 | 3 | 3 | -0.354 | 0.039 | -0.213 | 0.083 | 0.145 | 0.118 | 0.517 | 0.070 | 0.511 | -0.027 | 0.206 | -0.086 | 0.026 | -0.088 | -0.166 | -0.054 | -0.230 | -0.065 | -0.158 | -0.007 | -0.284 | 0.016 |
| Bleak | 1242 | 2 | 3 | 3 | -0.352 | 0.042 | -0.208 | 0.081 | 0.155 | 0.104 | 0.506 | 0.077 | 0.512 | -0.015 | 0.209 | -0.094 | 0.034 | -0.108 | -0.163 | -0.043 | -0.238 | -0.066 | -0.165 | -0.006 | -0.290 | 0.027 |
| Bleak | 1243 | 2 | 3 | 3 | -0.345 | 0.063 | -0.208 | 0.079 | 0.151 | 0.087 | 0.498 | 0.085 | 0.507 | -0.002 | 0.239 | -0.105 | 0.029 | -0.117 | -0.175 | -0.052 | -0.250 | -0.052 | -0.165 | -0.009 | -0.282 | 0.024 |
| Bleak | 1244 | 2 | 3 | 3 | -0.351 | 0.040 | -0.210 | 0.084 | 0.138 | 0.125 | 0.522 | 0.064 | 0.514 | -0.023 | 0.200 | -0.086 | 0.024 | -0.095 | -0.159 | -0.045 | -0.230 | -0.062 | -0.166 | -0.012 | -0.283 | 0.010 |
| Bleak | 1245 | 2 | 3 | 3 | -0.364 | 0.004 | -0.250 | 0.074 | 0.169 | 0.154 | 0.515 | 0.031 | 0.485 | -0.046 | 0.215 | -0.066 | 0.067 | -0.064 | -0.149 | -0.030 | -0.221 | -0.063 | -0.164 | 0.001 | -0.302 | 0.004 |
| Bleak | 1246 | 2 | 3 | 3 | -0.340 | 0.062 | -0.204 | 0.081 | 0.145 | 0.089 | 0.495 | 0.095 | 0.531 | 0.001 | 0.213 | -0.119 | 0.016 | -0.126 | -0.174 | -0.044 | -0.232 | -0.053 | -0.167 | -0.018 | -0.281 | 0.032 |
| Bleak | 1247 | 2 | 3 | 3 | -0.357 | 0.023 | -0.210 | 0.083 | 0.147 | 0.126 | 0.513 | 0.064 | 0.510 | -0.033 | 0.206 | -0.085 | 0.040 | -0.084 | -0.163 | -0.043 | -0.231 | -0.061 | -0.163 | -0.003 | -0.292 | 0.014 |
| Bleak | 1248 | 2 | 3 | 3 | -0.356 | 0.057 | -0.205 | 0.082 | 0.132 | 0.097 | 0.496 | 0.080 | 0.532 | -0.002 | 0.204 | -0.116 | 0.035 | -0.109 | -0.162 | -0.051 | -0.235 | -0.061 | -0.161 | -0.011 | -0.280 | 0.033 |
| Bleak | 1249 | 2 | 3 | 3 | -0.347 | 0.061 | -0.214 | 0.077 | 0.146 | 0.093 | 0.492 | 0.079 | 0.527 | -0.001 | 0.213 | -0.105 | 0.038 | -0.115 | -0.167 | -0.047 | -0.245 | -0.056 | -0.161 | -0.012 | -0.284 | 0.027 |
| Bleak | 1250 | 2 | 3 | 3 | -0.349 | 0.048 | -0.217 | 0.076 | 0.144 | 0.105 | 0.509 | 0.071 | 0.506 | -0.019 | 0.224 | -0.092 | 0.041 | -0.095 | -0.165 | -0.045 | -0.238 | -0.060 | -0.163 | -0.009 | -0.291 | 0.020 |
| Bleak | 1250 | 2 | 3 | 3 | -0.349 | 0.040 | -0.214 | 0.087 | 0.143 | 0.110 | 0.506 | 0.071 | 0.511 | -0.021 | 0.216 | -0.093 | 0.043 | -0.102 | -0.165 | -0.047 | -0.232 | -0.066 | -0.168 | 0.002 | -0.291 | 0.018 |
| Bleak | 1031 | 3 | 2 | 1 | -0.361 | 0.023 | -0.223 | 0.076 | 0.145 | 0.127 | 0.519 | 0.052 | 0.505 | -0.032 | 0.216 | -0.069 | 0.031 | -0.087 | -0.151 | -0.038 | -0.228 | -0.063 | -0.169 | -0.003 | -0.284 | 0.015 |
| Bleak | 1032 | 3 | 2 | 1 | -0.354 | 0.029 | -0.212 | 0.086 | 0.136 | 0.110 | 0.504 | 0.075 | 0.520 | -0.019 | 0.211 | -0.106 | 0.042 | -0.104 | -0.165 | -0.041 | -0.228 | -0.063 | -0.173 | 0.007 | -0.280 | 0.026 |
| Bleak | 1033 | 3 | 2 | 1 | -0.349 | 0.043 | -0.212 | 0.087 | 0.131 | 0.105 | 0.511 | 0.073 | 0.515 | -0.015 | 0.215 | -0.094 | 0.037 | -0.110 | -0.164 | -0.050 | -0.233 | -0.057 | -0.170 | -0.007 | -0.282 | 0.024 |
| Bleak | 1034 | 3 | 2 | 1 | -0.340 | 0.056 | -0.209 | 0.084 | 0.140 | 0.106 | 0.499 | 0.079 | 0.510 | -0.009 | 0.227 | -0.105 | 0.049 | -0.120 | -0.171 | -0.056 | -0.243 | -0.054 | -0.182 | -0.013 | -0.282 | 0.030 |
| Bleak | 1035 | 3 | 2 | 1 | -0.358 | 0.045 | -0.211 | 0.087 | 0.128 | 0.101 | 0.506 | 0.078 | 0.518 | -0.008 | 0.207 | -0.107 | 0.046 | -0.115 | -0.163 | -0.055 | -0.245 | -0.061 | -0.156 | 0.004 | -0.272 | 0.031 |
| Bleak | 1036 | 3 | 2 | 1 | -0.362 | 0.036 | -0.216 | 0.082 | 0.149 | 0.110 | 0.512 | 0.072 | 0.505 | -0.015 | 0.210 | -0.094 | 0.040 | -0.111 | -0.159 | -0.046 | -0.247 | -0.063 | -0.160 | 0.004 | -0.271 | 0.025 |
| Bleak | 1037 | 3 | 2 | 1 | -0.359 | 0.017 | -0.216 | 0.084 | 0.133 | 0.116 | 0.517 | 0.062 | 0.510 | -0.025 | 0.215 | -0.088 | 0.036 | -0.096 | -0.158 | -0.035 | -0.226 | -0.060 | -0.170 | 0.004 | -0.283 | 0.021 |
| Bleak | 1038 | 3 | 2 | 1 | -0.351 | 0.043 | -0.217 | 0.086 | 0.151 | 0.098 | 0.500 | 0.070 | 0.512 | -0.014 | 0.220 | -0.092 | 0.054 | -0.102 | -0.172 | -0.048 | -0.229 | -0.055 | -0.181 | -0.008 | -0.287 | 0.021 |
| Bleak | 1039 | 3 | 2 | 1 | -0.348 | 0.066 | -0.205 | 0.084 | 0.134 | 0.100 | 0.484 | 0.082 | 0.526 | -0.004 | 0.234 | -0.107 | 0.033 | -0.124 | -0.165 | -0.065 | -0.245 | -0.059 | -0.170 | -0.003 | -0.279 | 0.032 |
| Bleak | 1040 | 3 | 2 | 1 | -0.363 | 0.023 | -0.207 | 0.088 | 0.145 | 0.110 | 0.515 | 0.066 | 0.515 | -0.025 | 0.199 | -0.084 | 0.040 | -0.091 | -0.161 | -0.043 | -0.229 | -0.064 | -0.172 | 0.002 | -0.281 | 0.018 |
| Bleak | 1041 | 3 | 2 | 1 | -0.362 | 0.033 | -0.206 | 0.098 | 0.150 | 0.117 | 0.510 | 0.073 | 0.508 | -0.025 | 0.202 | -0.096 | 0.047 | -0.099 | -0.163 | -0.052 | -0.217 | -0.062 | -0.177 | -0.001 | -0.292 | 0.014 |
| Bleak | 1042 | 3 | 2 | 1 | -0.351 | 0.043 | -0.210 | 0.082 | 0.126 | 0.108 | 0.521 | 0.068 | 0.506 | -0.018 | 0.219 | -0.087 | 0.037 | -0.104 | -0.164 | -0.049 | -0.233 | -0.061 | -0.168 | -0.005 | -0.283 | 0.023 |
| Bleak | 1043 | 3 | 2 | 1 | -0.352 | 0.039 | -0.220 | 0.081 | 0.131 | 0.116 | 0.529 | 0.063 | 0.512 | -0.023 | 0.192 | -0.087 | 0.043 | -0.083 | -0.162 | -0.054 | -0.229 | -0.064 | -0.168 | -0.005 | -0.276 | 0.015 |
| Bleak | 1044 | 3 | 2 | 1 | -0.361 | 0.025 | -0.217 | 0.080 | 0.151 | 0.120 | 0.507 | 0.056 | 0.502 | -0.025 | 0.223 | -0.083 | 0.051 | -0.091 | -0.162 | -0.042 | -0.236 | -0.062 | -0.171 | 0.002 | -0.288 | 0.020 |
| Bleak | 1045 | 3 | 2 | 1 | -0.344 | 0.033 | -0.225 | 0.084 | 0.125 | 0.129 | 0.529 | 0.061 | 0.501 | -0.031 | 0.219 | -0.085 | 0.039 | -0.093 | -0.170 | -0.050 | -0.218 | -0.061 | -0.175 | 0.000 | -0.281 | 0.012 |
| Bleak | 1046 | 3 | 2 | 1 | -0.354 | 0.055 | -0.211 | 0.087 | 0.135 | 0.098 | 0.493 | 0.078 | 0.524 | -0.002 | 0.217 | -0.113 | 0.043 | -0.118 | -0.160 | -0.052 | -0.246 | -0.056 | -0.164 | -0.001 | -0.276 | 0.024 |
| Bleak | 1047 | 3 | 2 | 1 | -0.356 | 0.043 | -0.211 | 0.085 | 0.126 | 0.095 | 0.515 | 0.077 | 0.511 | -0.011 | 0.215 | -0.097 | 0.043 | -0.115 | -0.160 | -0.051 | -0.237 | -0.058 | -0.167 | 0.004 | -0.278 | 0.029 |
| Bleak | 1048 | 3 | 2 | 1 | -0.357 | 0.022 | -0.222 | 0.074 | 0.135 | 0.136 | 0.523 | 0.048 | 0.502 | -0.033 | 0.212 | -0.075 | 0.050 | -0.083 | -0.155 | -0.040 | -0.235 | -0.063 | -0.171 | -0.001 | -0.282 | 0.014 |
| Bleak | 1049 | 3 | 2 | 1 | -0.344 | 0.050 | -0.205 | 0.085 | 0.137 | 0.097 | 0.513 | 0.079 | 0.510 | -0.008 | 0.220 | -0.096 | 0.031 | -0.121 | -0.172 | -0.053 | -0.234 | -0.058 | -0.175 | 0.001 | -0.282 | 0.024 |
| Bleak | 1050 | 3 | 2 | 1 | -0.356 | 0.021 | -0.227 | 0.087 | 0.132 | 0.134 | 0.523 | 0.053 | 0.502 | -0.031 | 0.212 | -0.083 | 0.044 | -0.092 | -0.151 | -0.045 | -0.229 | -0.062 | -0.169 | 0.005 | -0.281 | 0.013 |
| Bleak | 1051 | 3 | 2 | 1 | -0.350 | 0.017 | -0.224 | 0.083 | 0.124 | 0.138 | 0.528 | 0.055 | 0.509 | -0.043 | 0.202 | -0.072 | 0.045 | -0.085 | -0.153 | -0.039 | -0.225 | -0.063 | -0.174 | -0.001 | -0.283 | 0.010 |
| Bleak | 1052 | 3 | 2 | 1 | -0.346 | 0.023 | -0.207 | 0.081 | 0.148 | 0.107 | 0.510 | 0.068 | 0.511 | -0.017 | 0.215 | -0.092 | 0.045 | -0.104 | -0.168 | -0.035 | -0.244 | -0.055 | -0.179 | 0.005 | -0.283 | 0.018 |
| Bleak | 1053 | 3 | 2 | 1 | -0.359 | 0.012 | -0.224 | 0.082 | 0.134 | 0.131 | 0.529 | 0.049 | 0.502 | -0.038 | 0.202 | -0.072 | 0.055 | -0.073 | -0.155 | -0.036 | -0.230 | -0.060 | -0.167 | -0.006 | -0.287 | 0.013 |
| Bleak | 1054 | 3 | 2 | 1 | -0.358 | 0.038 | -0.223 | 0.089 | 0.139 | 0.127 | 0.516 | 0.069 | 0.511 | -0.021 | 0.201 | -0.100 | 0.035 | -0.101 | -0.158 | -0.050 | -0.228 | -0.065 | -0.159 | -0.001 | -0.275 | 0.015 |
| Bleak | 1055 | 3 | 2 | 1 | -0.366 | 0.006 | -0.232 | 0.084 | 0.137 | 0.141 | 0.518 | 0.048 | 0.505 | -0.045 | 0.212 | -0.076 | 0.046 | -0.079 | -0.149 | -0.034 | -0.213 | -0.064 | -0.169 | 0.015 | -0.289 | 0.004 |
| Bleak | 1056 | 3 | 2 | 1 | -0.357 | 0.018 | -0.219 | 0.087 | 0.136 | 0.124 | 0.515 | 0.062 | 0.506 | -0.031 | 0.217 | -0.091 | 0.044 | -0.091 | -0.157 | -0.032 | -0.227 | -0.060 | -0.172 | 0.003 | -0.286 | 0.013 |
| Bleak | 1057 | 3 | 2 | 1 | -0.352 | 0.056 | -0.215 | 0.083 | 0.160 | 0.080 | 0.494 | 0.090 | 0.505 | 0.002 | 0.231 | -0.116 | 0.040 | -0.123 | -0.166 | -0.048 | -0.235 | -0.052 | -0.176 | -0.003 | -0.287 | 0.032 |
| Bleak | 1058 | 3 | 2 | 1 | -0.362 | 0.043 | -0.200 | 0.093 | 0.125 | 0.099 | 0.499 | 0.082 | 0.535 | -0.011 | 0.196 | -0.111 | 0.038 | -0.109 | -0.156 | -0.047 | -0.241 | -0.063 | -0.161 | -0.003 | -0.274 | 0.026 |
| Bleak | 1059 | 3 | 2 | 1 | -0.337 | 0.049 | -0.203 | 0.085 | 0.140 | 0.090 | 0.502 | 0.089 | 0.515 | 0.001 | 0.221 | -0.118 | 0.039 | -0.130 | -0.178 | -0.043 | -0.236 | -0.058 | -0.179 | 0.000 | -0.283 | 0.034 |
| Bleak | 1060 | 3 | 2 | 1 | -0.339 | 0.060 | -0.206 | 0.083 | 0.143 | 0.074 | 0.498 | 0.087 | 0.518 | 0.006 | 0.221 | -0.114 | 0.047 | -0.120 | -0.180 | -0.052 | -0.244 | -0.054 | -0.179 | -0.002 | -0.280 | 0.031 |
| Bleak | 1031 | 3 | 2 | 2 | -0.359 | 0.025 | -0.223 | 0.077 | 0.142 | 0.126 | 0.520 | 0.052 | 0.506 | -0.032 | 0.216 | -0.069 | 0.032 | -0.088 | -0.157 | -0.040 | -0.229 | -0.067 | -0.169 | 0.000 | -0.280 | 0.015 |
| Bleak | 1032 | 3 | 2 | 2 | -0.353 | 0.035 | -0.210 | 0.085 | 0.135 | 0.108 | 0.507 | 0.075 | 0.522 | -0.017 | 0.205 | -0.104 | 0.037 | -0.105 | -0.163 | -0.041 | -0.228 | -0.064 | -0.172 | 0.004 | -0.280 | 0.022 |
| Bleak | 1033 | 3 | 2 | 2 | -0.345 | 0.044 | -0.209 | 0.085 | 0.130 | 0.102 | 0.517 | 0.077 | 0.515 | -0.015 | 0.211 | -0.093 | 0.032 | -0.111 | -0.166 | -0.049 | -0.232 | -0.057 | -0.173 | -0.005 | -0.281 | 0.023 |
| Bleak | 1034 | 3 | 2 | 2 | -0.338 | 0.063 | -0.209 | 0.083 | 0.134 | 0.105 | 0.504 | 0.084 | 0.506 | -0.010 | 0.227 | -0.106 | 0.052 | -0.123 | -0.168 | -0.059 | -0.244 | -0.056 | -0.180 | -0.010 | -0.283 | 0.030 |
| Bleak | 1035 | 3 | 2 | 2 | -0.354 | 0.047 | -0.209 | 0.086 | 0.125 | 0.100 | 0.511 | 0.081 | 0.520 | -0.008 | 0.202 | -0.107 | 0.041 | -0.117 | -0.161 | -0.055 | -0.247 | -0.061 | -0.157 | 0.004 | -0.271 | 0.030 |
| Bleak | 1036 | 3 | 2 | 2 | -0.359 | 0.041 | -0.214 | 0.081 | 0.140 | 0.110 | 0.523 | 0.075 | 0.499 | -0.015 | 0.213 | -0.096 | 0.033 | -0.108 | -0.159 | -0.047 | -0.249 | -0.063 | -0.160 | 0.000 | -0.267 | 0.023 |
| Bleak | 1037 | 3 | 2 | 2 | -0.354 | 0.025 | -0.216 | 0.082 | 0.143 | 0.116 | 0.516 | 0.066 | 0.511 | -0.026 | 0.210 | -0.085 | 0.032 | -0.098 | -0.161 | -0.039 | -0.228 | -0.061 | -0.170 | 0.004 | -0.282 | 0.017 |
| Bleak | 1038 | 3 | 2 | 2 | -0.350 | 0.050 | -0.218 | 0.084 | 0.153 | 0.100 | 0.501 | 0.069 | 0.510 | -0.012 | 0.220 | -0.091 | 0.048 | -0.102 | -0.170 | -0.053 | -0.226 | -0.058 | -0.179 | -0.007 | -0.289 | 0.021 |
| Bleak | 1039 | 3 | 2 | 2 | -0.350 | 0.067 | -0.206 | 0.087 | 0.138 | 0.102 | 0.488 | 0.084 | 0.515 | -0.006 | 0.236 | -0.108 | 0.037 | -0.128 | -0.162 | -0.066 | -0.245 | -0.058 | -0.173 | -0.004 | -0.278 | 0.031 |
| Bleak | 1040 | 3 | 2 | 2 | -0.362 | 0.024 | -0.214 | 0.088 | 0.145 | 0.112 | 0.516 | 0.065 | 0.511 | -0.025 | 0.201 | -0.085 | 0.043 | -0.091 | -0.157 | -0.043 | -0.235 | -0.060 | -0.168 | -0.003 | -0.279 | 0.018 |
| Bleak | 1041 | 3 | 2 | 2 | -0.361 | 0.033 | -0.199 | 0.095 | 0.150 | 0.119 | 0.511 | 0.075 | 0.512 | -0.022 | 0.200 | -0.096 | 0.032 | -0.105 | -0.162 | -0.051 | -0.223 | -0.060 | -0.174 | -0.001 | -0.286 | 0.013 |
| Bleak | 1042 | 3 | 2 | 2 | -0.353 | 0.046 | -0.211 | 0.079 | 0.123 | 0.109 | 0.525 | 0.071 | 0.506 | -0.021 | 0.217 | -0.087 | 0.034 | -0.106 | -0.163 | -0.049 | -0.228 | -0.063 | -0.168 | -0.002 | -0.282 | 0.021 |
| Bleak | 1043 | 3 | 2 | 2 | -0.349 | 0.038 | -0.221 | 0.080 | 0.126 | 0.114 | 0.527 | 0.065 | 0.525 | -0.025 | 0.186 | -0.085 | 0.034 | -0.082 | -0.158 | -0.054 | -0.230 | -0.062 | -0.166 | -0.004 | -0.273 | 0.014 |
| Bleak | 1044 | 3 | 2 | 2 | -0.360 | 0.028 | -0.219 | 0.081 | 0.135 | 0.117 | 0.511 | 0.058 | 0.504 | -0.029 | 0.227 | -0.083 | 0.050 | -0.087 | -0.158 | -0.043 | -0.235 | -0.063 | -0.171 | 0.002 | -0.285 | 0.019 |
| Bleak | 1045 | 3 | 2 | 2 | -0.344 | 0.037 | -0.229 | 0.085 | 0.117 | 0.129 | 0.527 | 0.064 | 0.507 | -0.035 | 0.216 | -0.084 | 0.039 | -0.094 | -0.168 | -0.052 | -0.213 | -0.061 | -0.172 | 0.000 | -0.280 | 0.011 |
| Bleak | 1046 | 3 | 2 | 2 | -0.354 | 0.052 | -0.212 | 0.088 | 0.133 | 0.099 | 0.501 | 0.081 | 0.518 | -0.005 | 0.218 | -0.114 | 0.036 | -0.115 | -0.163 | -0.052 | -0.241 | -0.055 | -0.161 | -0.006 | -0.275 | 0.026 |
| Bleak | 1047 | 3 | 2 | 2 | -0.358 | 0.046 | -0.207 | 0.086 | 0.127 | 0.093 | 0.516 | 0.079 | 0.507 | -0.011 | 0.220 | -0.099 | 0.036 | -0.113 | -0.163 | -0.054 | -0.233 | -0.059 | -0.164 | 0.003 | -0.280 | 0.029 |
| Bleak | 1048 | 3 | 2 | 2 | -0.355 | 0.024 | -0.221 | 0.073 | 0.140 | 0.136 | 0.525 | 0.047 | 0.502 | -0.030 | 0.213 | -0.075 | 0.040 | -0.083 | -0.159 | -0.041 | -0.232 | -0.063 | -0.170 | 0.001 | -0.283 | 0.012 |
| Bleak | 1049 | 3 | 2 | 2 | -0.345 | 0.053 | -0.210 | 0.087 | 0.142 | 0.099 | 0.516 | 0.080 | 0.504 | -0.007 | 0.221 | -0.098 | 0.030 | -0.120 | -0.170 | -0.053 | -0.232 | -0.056 | -0.175 | -0.009 | -0.280 | 0.024 |
| Bleak | 1050 | 3 | 2 | 2 | -0.355 | 0.021 | -0.228 | 0.086 | 0.135 | 0.134 | 0.523 | 0.055 | 0.499 | -0.031 | 0.215 | -0.084 | 0.042 | -0.094 | -0.150 | -0.042 | -0.231 | -0.061 | -0.168 | 0.003 | -0.281 | 0.012 |
| Bleak | 1051 | 3 | 2 | 2 | -0.353 | 0.017 | -0.217 | 0.088 | 0.126 | 0.137 | 0.524 | 0.051 | 0.510 | -0.038 | 0.208 | -0.074 | 0.041 | -0.084 | -0.157 | -0.041 | -0.225 | -0.064 | -0.173 | -0.002 | -0.284 | 0.009 |
| Bleak | 1052 | 3 | 2 | 2 | -0.347 | 0.026 | -0.216 | 0.080 | 0.164 | 0.109 | 0.508 | 0.068 | 0.506 | -0.017 | 0.214 | -0.093 | 0.044 | -0.099 | -0.170 | -0.035 | -0.235 | -0.062 | -0.182 | 0.007 | -0.286 | 0.017 |
| Bleak | 1053 | 3 | 2 | 2 | -0.356 | 0.012 | -0.229 | 0.079 | 0.132 | 0.129 | 0.533 | 0.049 | 0.502 | -0.039 | 0.201 | -0.070 | 0.052 | -0.071 | -0.157 | -0.033 | -0.229 | -0.060 | -0.164 | -0.005 | -0.286 | 0.011 |
| Bleak | 1054 | 3 | 2 | 2 | -0.358 | 0.036 | -0.226 | 0.089 | 0.134 | 0.126 | 0.517 | 0.068 | 0.512 | -0.022 | 0.201 | -0.099 | 0.035 | -0.100 | -0.159 | -0.046 | -0.229 | -0.067 | -0.154 | -0.001 | -0.274 | 0.017 |
| Bleak | 1055 | 3 | 2 | 2 | -0.365 | 0.008 | -0.236 | 0.083 | 0.138 | 0.141 | 0.519 | 0.048 | 0.507 | -0.045 | 0.208 | -0.075 | 0.043 | -0.078 | -0.149 | -0.032 | -0.209 | -0.065 | -0.170 | 0.011 | -0.286 | 0.003 |
| Bleak | 1056 | 3 | 2 | 2 | -0.359 | 0.021 | -0.219 | 0.086 | 0.133 | 0.124 | 0.512 | 0.063 | 0.512 | -0.032 | 0.215 | -0.091 | 0.043 | -0.091 | -0.155 | -0.034 | -0.228 | -0.057 | -0.170 | 0.001 | -0.285 | 0.011 |
| Bleak | 1057 | 3 | 2 | 2 | -0.350 | 0.062 | -0.218 | 0.082 | 0.156 | 0.080 | 0.494 | 0.089 | 0.510 | 0.003 | 0.230 | -0.115 | 0.037 | -0.123 | -0.168 | -0.043 | -0.233 | -0.051 | -0.175 | -0.012 | -0.285 | 0.030 |
| Bleak | 1058 | 3 | 2 | 2 | -0.359 | 0.049 | -0.207 | 0.091 | 0.122 | 0.100 | 0.504 | 0.082 | 0.534 | -0.011 | 0.196 | -0.112 | 0.033 | -0.109 | -0.158 | -0.046 | -0.230 | -0.065 | -0.161 | -0.003 | -0.273 | 0.024 |
| Bleak | 1059 | 3 | 2 | 2 | -0.334 | 0.051 | -0.204 | 0.084 | 0.141 | 0.090 | 0.508 | 0.093 | 0.512 | 0.001 | 0.219 | -0.118 | 0.031 | -0.136 | -0.176 | -0.043 | -0.237 | -0.059 | -0.178 | 0.005 | -0.281 | 0.031 |
| Bleak | 1060 | 3 | 2 | 2 | -0.340 | 0.063 | -0.212 | 0.083 | 0.142 | 0.075 | 0.499 | 0.088 | 0.518 | 0.004 | 0.224 | -0.111 | 0.042 | -0.120 | -0.176 | -0.056 | -0.241 | -0.052 | -0.177 | -0.007 | -0.279 | 0.032 |
| Bleak | 1031 | 3 | 2 | 3 | -0.360 | 0.024 | -0.223 | 0.077 | 0.142 | 0.126 | 0.522 | 0.053 | 0.506 | -0.032 | 0.213 | -0.071 | 0.030 | -0.088 | -0.155 | -0.037 | -0.224 | -0.064 | -0.165 | -0.005 | -0.286 | 0.016 |
| Bleak | 1032 | 3 | 2 | 3 | -0.352 | 0.030 | -0.216 | 0.085 | 0.131 | 0.107 | 0.512 | 0.075 | 0.521 | -0.019 | 0.203 | -0.103 | 0.037 | -0.104 | -0.163 | -0.038 | -0.224 | -0.063 | -0.169 | 0.008 | -0.281 | 0.022 |
| Bleak | 1033 | 3 | 2 | 3 | -0.349 | 0.041 | -0.206 | 0.087 | 0.126 | 0.103 | 0.512 | 0.077 | 0.522 | -0.018 | 0.205 | -0.094 | 0.040 | -0.110 | -0.168 | -0.048 | -0.228 | -0.058 | -0.172 | -0.002 | -0.283 | 0.023 |
| Bleak | 1034 | 3 | 2 | 3 | -0.346 | 0.051 | -0.202 | 0.087 | 0.143 | 0.107 | 0.503 | 0.082 | 0.501 | -0.010 | 0.227 | -0.105 | 0.053 | -0.122 | -0.173 | -0.058 | -0.242 | -0.056 | -0.180 | -0.006 | -0.284 | 0.032 |
| Bleak | 1035 | 3 | 2 | 3 | -0.357 | 0.045 | -0.207 | 0.087 | 0.119 | 0.100 | 0.513 | 0.079 | 0.517 | -0.009 | 0.207 | -0.106 | 0.041 | -0.116 | -0.161 | -0.054 | -0.246 | -0.062 | -0.154 | 0.004 | -0.273 | 0.031 |
| Bleak | 1036 | 3 | 2 | 3 | -0.361 | 0.031 | -0.211 | 0.082 | 0.138 | 0.109 | 0.521 | 0.071 | 0.506 | -0.014 | 0.207 | -0.095 | 0.030 | -0.107 | -0.160 | -0.047 | -0.245 | -0.064 | -0.156 | 0.008 | -0.270 | 0.026 |
| Bleak | 1037 | 3 | 2 | 3 | -0.355 | 0.020 | -0.216 | 0.083 | 0.128 | 0.115 | 0.515 | 0.065 | 0.521 | -0.029 | 0.209 | -0.085 | 0.029 | -0.094 | -0.157 | -0.035 | -0.226 | -0.062 | -0.166 | 0.003 | -0.282 | 0.020 |
| Bleak | 1038 | 3 | 2 | 3 | -0.349 | 0.043 | -0.217 | 0.082 | 0.150 | 0.097 | 0.509 | 0.071 | 0.511 | -0.014 | 0.215 | -0.090 | 0.041 | -0.099 | -0.171 | -0.050 | -0.223 | -0.056 | -0.178 | -0.004 | -0.288 | 0.020 |
| Bleak | 1039 | 3 | 2 | 3 | -0.349 | 0.066 | -0.208 | 0.086 | 0.125 | 0.098 | 0.491 | 0.085 | 0.524 | -0.007 | 0.233 | -0.108 | 0.032 | -0.128 | -0.167 | -0.059 | -0.237 | -0.062 | -0.166 | -0.003 | -0.277 | 0.033 |
| Bleak | 1040 | 3 | 2 | 3 | -0.367 | 0.025 | -0.210 | 0.085 | 0.135 | 0.110 | 0.516 | 0.066 | 0.514 | -0.025 | 0.204 | -0.086 | 0.040 | -0.094 | -0.159 | -0.044 | -0.230 | -0.065 | -0.164 | 0.011 | -0.279 | 0.018 |
| Bleak | 1041 | 3 | 2 | 3 | -0.361 | 0.033 | -0.201 | 0.094 | 0.156 | 0.118 | 0.509 | 0.076 | 0.510 | -0.024 | 0.199 | -0.095 | 0.038 | -0.103 | -0.167 | -0.054 | -0.216 | -0.062 | -0.174 | 0.001 | -0.291 | 0.016 |
| Bleak | 1042 | 3 | 2 | 3 | -0.353 | 0.041 | -0.213 | 0.082 | 0.124 | 0.108 | 0.526 | 0.068 | 0.503 | -0.019 | 0.220 | -0.087 | 0.031 | -0.105 | -0.162 | -0.046 | -0.229 | -0.063 | -0.166 | -0.001 | -0.282 | 0.022 |
| Bleak | 1043 | 3 | 2 | 3 | -0.353 | 0.031 | -0.219 | 0.083 | 0.122 | 0.115 | 0.524 | 0.065 | 0.530 | -0.029 | 0.186 | -0.085 | 0.032 | -0.081 | -0.161 | -0.050 | -0.220 | -0.063 | -0.165 | -0.003 | -0.275 | 0.017 |
| Bleak | 1044 | 3 | 2 | 3 | -0.361 | 0.025 | -0.219 | 0.082 | 0.151 | 0.119 | 0.510 | 0.057 | 0.502 | -0.027 | 0.222 | -0.080 | 0.045 | -0.090 | -0.161 | -0.042 | -0.231 | -0.063 | -0.172 | -0.002 | -0.285 | 0.020 |
| Bleak | 1045 | 3 | 2 | 3 | -0.346 | 0.030 | -0.217 | 0.086 | 0.113 | 0.127 | 0.524 | 0.064 | 0.513 | -0.038 | 0.216 | -0.080 | 0.039 | -0.094 | -0.171 | -0.051 | -0.213 | -0.061 | -0.176 | 0.005 | -0.282 | 0.012 |
| Bleak | 1046 | 3 | 2 | 3 | -0.353 | 0.051 | -0.213 | 0.089 | 0.131 | 0.097 | 0.494 | 0.079 | 0.530 | -0.004 | 0.215 | -0.112 | 0.033 | -0.115 | -0.165 | -0.050 | -0.240 | -0.057 | -0.159 | -0.005 | -0.274 | 0.027 |
| Bleak | 1047 | 3 | 2 | 3 | -0.357 | 0.042 | -0.207 | 0.087 | 0.127 | 0.095 | 0.512 | 0.079 | 0.515 | -0.013 | 0.213 | -0.097 | 0.037 | -0.113 | -0.166 | -0.049 | -0.234 | -0.059 | -0.164 | -0.002 | -0.278 | 0.030 |
| Bleak | 1048 | 3 | 2 | 3 | -0.357 | 0.022 | -0.226 | 0.073 | 0.137 | 0.135 | 0.524 | 0.047 | 0.505 | -0.033 | 0.208 | -0.073 | 0.044 | -0.080 | -0.158 | -0.039 | -0.227 | -0.065 | -0.168 | -0.001 | -0.282 | 0.015 |
| Bleak | 1049 | 3 | 2 | 3 | -0.347 | 0.051 | -0.211 | 0.088 | 0.144 | 0.099 | 0.513 | 0.079 | 0.509 | -0.008 | 0.217 | -0.098 | 0.027 | -0.119 | -0.172 | -0.054 | -0.226 | -0.059 | -0.172 | -0.001 | -0.281 | 0.022 |
| Bleak | 1050 | 3 | 2 | 3 | -0.360 | 0.019 | -0.226 | 0.090 | 0.128 | 0.133 | 0.526 | 0.051 | 0.499 | -0.031 | 0.214 | -0.081 | 0.043 | -0.091 | -0.155 | -0.043 | -0.222 | -0.064 | -0.165 | 0.004 | -0.282 | 0.013 |
| Bleak | 1051 | 3 | 2 | 3 | -0.354 | 0.021 | -0.221 | 0.086 | 0.119 | 0.138 | 0.527 | 0.052 | 0.509 | -0.041 | 0.211 | -0.073 | 0.040 | -0.085 | -0.157 | -0.041 | -0.220 | -0.066 | -0.168 | -0.004 | -0.286 | 0.012 |
| Bleak | 1052 | 3 | 2 | 3 | -0.347 | 0.024 | -0.217 | 0.081 | 0.140 | 0.107 | 0.521 | 0.069 | 0.510 | -0.021 | 0.209 | -0.090 | 0.038 | -0.097 | -0.166 | -0.034 | -0.234 | -0.055 | -0.171 | -0.003 | -0.282 | 0.020 |
| Bleak | 1053 | 3 | 2 | 3 | -0.362 | 0.012 | -0.222 | 0.084 | 0.135 | 0.130 | 0.527 | 0.048 | 0.503 | -0.038 | 0.204 | -0.071 | 0.054 | -0.074 | -0.159 | -0.033 | -0.224 | -0.062 | -0.165 | -0.010 | -0.289 | 0.013 |
| Bleak | 1054 | 3 | 2 | 3 | -0.358 | 0.028 | -0.223 | 0.090 | 0.142 | 0.130 | 0.515 | 0.067 | 0.512 | -0.025 | 0.201 | -0.096 | 0.031 | -0.100 | -0.161 | -0.049 | -0.225 | -0.066 | -0.158 | 0.003 | -0.275 | 0.017 |
| Bleak | 1055 | 3 | 2 | 3 | -0.366 | 0.005 | -0.236 | 0.085 | 0.140 | 0.140 | 0.516 | 0.051 | 0.507 | -0.047 | 0.209 | -0.075 | 0.041 | -0.082 | -0.147 | -0.034 | -0.209 | -0.064 | -0.166 | 0.017 | -0.290 | 0.005 |
| Bleak | 1056 | 3 | 2 | 3 | -0.361 | 0.017 | -0.218 | 0.090 | 0.132 | 0.124 | 0.510 | 0.062 | 0.512 | -0.033 | 0.216 | -0.091 | 0.042 | -0.091 | -0.154 | -0.032 | -0.223 | -0.060 | -0.169 | 0.003 | -0.288 | 0.012 |
| Bleak | 1057 | 3 | 2 | 3 | -0.352 | 0.059 | -0.215 | 0.084 | 0.158 | 0.080 | 0.493 | 0.089 | 0.510 | 0.003 | 0.231 | -0.115 | 0.032 | -0.122 | -0.166 | -0.046 | -0.231 | -0.053 | -0.173 | -0.012 | -0.287 | 0.034 |
| Bleak | 1058 | 3 | 2 | 3 | -0.364 | 0.045 | -0.198 | 0.092 | 0.117 | 0.099 | 0.503 | 0.082 | 0.536 | -0.012 | 0.202 | -0.111 | 0.026 | -0.107 | -0.164 | -0.047 | -0.224 | -0.066 | -0.159 | -0.004 | -0.276 | 0.029 |
| Bleak | 1059 | 3 | 2 | 3 | -0.339 | 0.045 | -0.206 | 0.089 | 0.140 | 0.091 | 0.504 | 0.090 | 0.514 | -0.001 | 0.222 | -0.116 | 0.032 | -0.131 | -0.179 | -0.046 | -0.231 | -0.060 | -0.173 | 0.002 | -0.284 | 0.037 |
| Bleak | 1060 | 3 | 2 | 3 | -0.338 | 0.062 | -0.208 | 0.086 | 0.141 | 0.076 | 0.499 | 0.087 | 0.520 | 0.003 | 0.224 | -0.110 | 0.038 | -0.117 | -0.179 | -0.053 | -0.236 | -0.053 | -0.182 | -0.016 | -0.280 | 0.033 |
| Bleak | 1131 | 3 | 1 | 1 | -0.347 | 0.041 | -0.217 | 0.077 | 0.155 | 0.114 | 0.505 | 0.064 | 0.509 | -0.018 | 0.220 | -0.089 | 0.041 | -0.093 | -0.171 | -0.044 | -0.233 | -0.063 | -0.172 | -0.013 | -0.290 | 0.022 |
| Bleak | 1132 | 3 | 1 | 1 | -0.355 | 0.014 | -0.239 | 0.078 | 0.133 | 0.114 | 0.522 | 0.045 | 0.510 | -0.028 | 0.212 | -0.074 | 0.053 | -0.077 | -0.165 | -0.034 | -0.214 | -0.057 | -0.170 | 0.002 | -0.286 | 0.016 |
| Bleak | 1133 | 3 | 1 | 1 | -0.345 | 0.036 | -0.211 | 0.083 | 0.146 | 0.111 | 0.510 | 0.061 | 0.512 | -0.008 | 0.213 | -0.099 | 0.041 | -0.093 | -0.166 | -0.045 | -0.233 | -0.058 | -0.178 | -0.012 | -0.289 | 0.024 |
| Bleak | 1134 | 3 | 1 | 1 | -0.352 | 0.026 | -0.226 | 0.077 | 0.141 | 0.108 | 0.513 | 0.058 | 0.518 | -0.024 | 0.212 | -0.078 | 0.033 | -0.088 | -0.160 | -0.035 | -0.225 | -0.058 | -0.172 | -0.006 | -0.285 | 0.019 |
| Bleak | 1135 | 3 | 1 | 1 | -0.352 | 0.013 | -0.219 | 0.077 | 0.141 | 0.118 | 0.517 | 0.049 | 0.511 | -0.023 | 0.222 | -0.077 | 0.031 | -0.087 | -0.168 | -0.030 | -0.226 | -0.060 | -0.170 | 0.004 | -0.287 | 0.016 |
| Bleak | 1136 | 3 | 1 | 1 | -0.347 | 0.015 | -0.214 | 0.082 | 0.149 | 0.127 | 0.520 | 0.055 | 0.519 | -0.022 | 0.191 | -0.085 | 0.031 | -0.086 | -0.163 | -0.041 | -0.224 | -0.061 | -0.174 | -0.001 | -0.287 | 0.017 |
| Bleak | 1137 | 3 | 1 | 1 | -0.357 | 0.027 | -0.222 | 0.078 | 0.157 | 0.109 | 0.505 | 0.062 | 0.518 | -0.018 | 0.205 | -0.088 | 0.038 | -0.093 | -0.160 | -0.041 | -0.226 | -0.060 | -0.173 | -0.002 | -0.284 | 0.025 |
| Bleak | 1138 | 3 | 1 | 1 | -0.352 | 0.031 | -0.229 | 0.085 | 0.132 | 0.126 | 0.523 | 0.055 | 0.504 | -0.022 | 0.212 | -0.088 | 0.041 | -0.093 | -0.159 | -0.043 | -0.219 | -0.058 | -0.169 | -0.009 | -0.285 | 0.016 |
| Bleak | 1139 | 3 | 1 | 1 | -0.352 | 0.024 | -0.224 | 0.079 | 0.155 | 0.124 | 0.513 | 0.059 | 0.504 | -0.025 | 0.218 | -0.087 | 0.033 | -0.090 | -0.160 | -0.037 | -0.221 | -0.059 | -0.175 | -0.006 | -0.291 | 0.018 |
| Bleak | 1140 | 3 | 1 | 1 | -0.346 | 0.016 | -0.238 | 0.075 | 0.148 | 0.137 | 0.529 | 0.047 | 0.488 | -0.032 | 0.217 | -0.080 | 0.055 | -0.084 | -0.167 | -0.031 | -0.219 | -0.058 | -0.177 | 0.001 | -0.290 | 0.009 |
| Bleak | 1141 | 3 | 1 | 1 | -0.357 | 0.022 | -0.224 | 0.082 | 0.157 | 0.120 | 0.512 | 0.053 | 0.503 | -0.024 | 0.219 | -0.076 | 0.038 | -0.089 | -0.169 | -0.042 | -0.225 | -0.059 | -0.168 | -0.006 | -0.287 | 0.018 |
| Bleak | 1142 | 3 | 1 | 1 | -0.349 | 0.035 | -0.218 | 0.077 | 0.137 | 0.108 | 0.509 | 0.066 | 0.524 | -0.014 | 0.198 | -0.096 | 0.048 | -0.097 | -0.166 | -0.042 | -0.236 | -0.058 | -0.164 | -0.002 | -0.283 | 0.023 |
| Bleak | 1143 | 3 | 1 | 1 | -0.344 | 0.030 | -0.218 | 0.079 | 0.146 | 0.113 | 0.513 | 0.068 | 0.521 | -0.020 | 0.201 | -0.093 | 0.032 | -0.093 | -0.172 | -0.044 | -0.224 | -0.058 | -0.172 | -0.001 | -0.283 | 0.019 |
| Bleak | 1144 | 3 | 1 | 1 | -0.351 | 0.037 | -0.225 | 0.076 | 0.145 | 0.096 | 0.508 | 0.063 | 0.517 | -0.023 | 0.209 | -0.078 | 0.058 | -0.083 | -0.170 | -0.049 | -0.233 | -0.057 | -0.172 | -0.004 | -0.286 | 0.021 |
| Bleak | 1145 | 3 | 1 | 1 | -0.350 | 0.026 | -0.216 | 0.079 | 0.139 | 0.115 | 0.520 | 0.058 | 0.514 | -0.017 | 0.205 | -0.085 | 0.036 | -0.095 | -0.169 | -0.040 | -0.225 | -0.055 | -0.173 | -0.007 | -0.281 | 0.021 |
| Bleak | 1146 | 3 | 1 | 1 | -0.351 | 0.039 | -0.211 | 0.081 | 0.152 | 0.095 | 0.504 | 0.074 | 0.518 | -0.014 | 0.212 | -0.096 | 0.036 | -0.096 | -0.169 | -0.042 | -0.230 | -0.060 | -0.172 | -0.004 | -0.289 | 0.022 |
| Bleak | 1147 | 3 | 1 | 1 | -0.355 | 0.041 | -0.221 | 0.079 | 0.146 | 0.102 | 0.500 | 0.069 | 0.509 | -0.014 | 0.219 | -0.102 | 0.060 | -0.096 | -0.165 | -0.044 | -0.226 | -0.059 | -0.176 | -0.007 | -0.291 | 0.030 |
| Bleak | 1148 | 3 | 1 | 1 | -0.355 | 0.018 | -0.224 | 0.078 | 0.141 | 0.129 | 0.520 | 0.051 | 0.503 | -0.036 | 0.217 | -0.077 | 0.044 | -0.074 | -0.160 | -0.037 | -0.220 | -0.063 | -0.176 | -0.007 | -0.291 | 0.018 |
| Bleak | 1149 | 3 | 1 | 1 | -0.351 | 0.034 | -0.224 | 0.085 | 0.146 | 0.112 | 0.504 | 0.067 | 0.506 | -0.019 | 0.223 | -0.094 | 0.051 | -0.104 | -0.176 | -0.045 | -0.229 | -0.058 | -0.168 | -0.002 | -0.283 | 0.023 |
| Bleak | 1150 | 3 | 1 | 1 | -0.348 | 0.023 | -0.216 | 0.075 | 0.139 | 0.102 | 0.521 | 0.063 | 0.519 | -0.023 | 0.208 | -0.080 | 0.029 | -0.083 | -0.172 | -0.036 | -0.224 | -0.056 | -0.175 | -0.003 | -0.282 | 0.019 |
| Bleak | 1151 | 3 | 1 | 1 | -0.342 | 0.042 | -0.215 | 0.080 | 0.156 | 0.085 | 0.508 | 0.076 | 0.512 | -0.006 | 0.219 | -0.097 | 0.039 | -0.102 | -0.177 | -0.046 | -0.230 | -0.052 | -0.182 | -0.007 | -0.287 | 0.027 |
| Bleak | 1152 | 3 | 1 | 1 | -0.342 | 0.025 | -0.217 | 0.078 | 0.145 | 0.121 | 0.518 | 0.055 | 0.503 | -0.025 | 0.219 | -0.075 | 0.049 | -0.093 | -0.177 | -0.047 | -0.234 | -0.058 | -0.175 | -0.005 | -0.287 | 0.022 |
| Bleak | 1153 | 3 | 1 | 1 | -0.355 | 0.033 | -0.220 | 0.083 | 0.143 | 0.105 | 0.515 | 0.067 | 0.517 | -0.015 | 0.205 | -0.093 | 0.030 | -0.098 | -0.160 | -0.039 | -0.223 | -0.058 | -0.168 | -0.006 | -0.284 | 0.021 |
| Bleak | 1154 | 3 | 1 | 1 | -0.347 | 0.024 | -0.217 | 0.079 | 0.142 | 0.120 | 0.516 | 0.060 | 0.514 | -0.023 | 0.212 | -0.088 | 0.033 | -0.089 | -0.163 | -0.037 | -0.230 | -0.059 | -0.174 | -0.005 | -0.285 | 0.019 |
| Bleak | 1155 | 3 | 1 | 1 | -0.347 | 0.028 | -0.227 | 0.080 | 0.135 | 0.115 | 0.521 | 0.059 | 0.512 | -0.021 | 0.212 | -0.086 | 0.031 | -0.093 | -0.163 | -0.039 | -0.215 | -0.058 | -0.172 | -0.005 | -0.288 | 0.020 |
| Bleak | 1156 | 3 | 1 | 1 | -0.359 | 0.024 | -0.227 | 0.077 | 0.148 | 0.112 | 0.512 | 0.060 | 0.509 | -0.027 | 0.218 | -0.085 | 0.039 | -0.083 | -0.165 | -0.037 | -0.220 | -0.059 | -0.168 | 0.001 | -0.286 | 0.017 |
| Bleak | 1157 | 3 | 1 | 1 | -0.353 | 0.043 | -0.208 | 0.087 | 0.150 | 0.095 | 0.492 | 0.072 | 0.529 | 0.000 | 0.208 | -0.108 | 0.041 | -0.108 | -0.172 | -0.043 | -0.224 | -0.057 | -0.174 | -0.009 | -0.288 | 0.029 |
| Bleak | 1158 | 3 | 1 | 1 | -0.370 | 0.029 | -0.226 | 0.082 | 0.165 | 0.123 | 0.490 | 0.057 | 0.499 | -0.019 | 0.234 | -0.093 | 0.053 | -0.101 | -0.164 | -0.039 | -0.225 | -0.067 | -0.163 | 0.000 | -0.292 | 0.027 |
| Bleak | 1159 | 3 | 1 | 1 | -0.360 | 0.025 | -0.219 | 0.069 | 0.158 | 0.103 | 0.515 | 0.060 | 0.500 | -0.024 | 0.225 | -0.078 | 0.039 | -0.080 | -0.163 | -0.032 | -0.236 | -0.065 | -0.173 | -0.003 | -0.285 | 0.023 |
| Bleak | 1160 | 3 | 1 | 1 | -0.355 | 0.036 | -0.219 | 0.080 | 0.136 | 0.110 | 0.506 | 0.061 | 0.512 | -0.016 | 0.217 | -0.093 | 0.057 | -0.102 | -0.171 | -0.039 | -0.224 | -0.059 | -0.171 | 0.001 | -0.287 | 0.021 |
| Bleak | 1131 | 3 | 1 | 2 | -0.347 | 0.039 | -0.215 | 0.076 | 0.156 | 0.115 | 0.509 | 0.068 | 0.508 | -0.019 | 0.217 | -0.090 | 0.034 | -0.095 | -0.167 | -0.045 | -0.238 | -0.059 | -0.169 | -0.011 | -0.288 | 0.021 |
| Bleak | 1132 | 3 | 1 | 2 | -0.355 | 0.014 | -0.227 | 0.080 | 0.138 | 0.112 | 0.522 | 0.050 | 0.511 | -0.028 | 0.208 | -0.076 | 0.048 | -0.075 | -0.166 | -0.035 | -0.222 | -0.060 | -0.169 | 0.001 | -0.287 | 0.015 |
| Bleak | 1133 | 3 | 1 | 2 | -0.346 | 0.034 | -0.218 | 0.082 | 0.153 | 0.114 | 0.504 | 0.063 | 0.510 | -0.014 | 0.217 | -0.097 | 0.045 | -0.096 | -0.165 | -0.046 | -0.231 | -0.056 | -0.180 | -0.006 | -0.289 | 0.022 |
| Bleak | 1134 | 3 | 1 | 2 | -0.353 | 0.022 | -0.222 | 0.077 | 0.146 | 0.110 | 0.512 | 0.064 | 0.513 | -0.029 | 0.217 | -0.080 | 0.033 | -0.088 | -0.160 | -0.035 | -0.226 | -0.059 | -0.175 | -0.002 | -0.285 | 0.019 |
| Bleak | 1135 | 3 | 1 | 2 | -0.353 | 0.012 | -0.221 | 0.075 | 0.153 | 0.118 | 0.514 | 0.049 | 0.507 | -0.022 | 0.224 | -0.075 | 0.031 | -0.088 | -0.172 | -0.034 | -0.230 | -0.055 | -0.167 | 0.004 | -0.286 | 0.017 |
| Bleak | 1136 | 3 | 1 | 2 | -0.348 | 0.013 | -0.216 | 0.080 | 0.149 | 0.127 | 0.516 | 0.060 | 0.522 | -0.029 | 0.194 | -0.084 | 0.027 | -0.084 | -0.161 | -0.036 | -0.226 | -0.061 | -0.173 | -0.002 | -0.285 | 0.016 |
| Bleak | 1137 | 3 | 1 | 2 | -0.358 | 0.027 | -0.225 | 0.078 | 0.153 | 0.111 | 0.514 | 0.067 | 0.509 | -0.023 | 0.203 | -0.089 | 0.045 | -0.095 | -0.161 | -0.044 | -0.226 | -0.061 | -0.173 | 0.006 | -0.281 | 0.022 |
| Bleak | 1138 | 3 | 1 | 2 | -0.357 | 0.035 | -0.230 | 0.085 | 0.134 | 0.128 | 0.515 | 0.058 | 0.508 | -0.026 | 0.215 | -0.089 | 0.040 | -0.094 | -0.160 | -0.042 | -0.216 | -0.057 | -0.168 | -0.012 | -0.282 | 0.014 |
| Bleak | 1139 | 3 | 1 | 2 | -0.349 | 0.027 | -0.224 | 0.077 | 0.152 | 0.122 | 0.510 | 0.064 | 0.507 | -0.030 | 0.220 | -0.086 | 0.035 | -0.089 | -0.161 | -0.038 | -0.227 | -0.060 | -0.174 | -0.004 | -0.288 | 0.017 |
| Bleak | 1140 | 3 | 1 | 2 | -0.349 | 0.015 | -0.236 | 0.076 | 0.152 | 0.137 | 0.532 | 0.047 | 0.486 | -0.032 | 0.219 | -0.080 | 0.043 | -0.079 | -0.162 | -0.036 | -0.216 | -0.059 | -0.178 | 0.002 | -0.291 | 0.008 |
| Bleak | 1141 | 3 | 1 | 2 | -0.356 | 0.022 | -0.221 | 0.078 | 0.158 | 0.122 | 0.513 | 0.057 | 0.505 | -0.026 | 0.214 | -0.077 | 0.037 | -0.089 | -0.166 | -0.046 | -0.229 | -0.057 | -0.168 | -0.003 | -0.287 | 0.019 |
| Bleak | 1142 | 3 | 1 | 2 | -0.350 | 0.037 | -0.217 | 0.078 | 0.126 | 0.107 | 0.511 | 0.067 | 0.526 | -0.016 | 0.203 | -0.095 | 0.040 | -0.101 | -0.162 | -0.045 | -0.231 | -0.057 | -0.165 | 0.000 | -0.282 | 0.023 |
| Bleak | 1143 | 3 | 1 | 2 | -0.343 | 0.033 | -0.219 | 0.079 | 0.142 | 0.112 | 0.510 | 0.069 | 0.523 | -0.021 | 0.206 | -0.093 | 0.034 | -0.093 | -0.171 | -0.048 | -0.227 | -0.056 | -0.175 | -0.003 | -0.280 | 0.022 |
| Bleak | 1144 | 3 | 1 | 2 | -0.351 | 0.034 | -0.218 | 0.076 | 0.142 | 0.097 | 0.515 | 0.067 | 0.517 | -0.024 | 0.208 | -0.079 | 0.041 | -0.085 | -0.167 | -0.051 | -0.229 | -0.056 | -0.174 | -0.001 | -0.283 | 0.020 |
| Bleak | 1145 | 3 | 1 | 2 | -0.352 | 0.025 | -0.216 | 0.083 | 0.135 | 0.114 | 0.520 | 0.063 | 0.515 | -0.020 | 0.203 | -0.087 | 0.036 | -0.099 | -0.167 | -0.042 | -0.220 | -0.060 | -0.174 | 0.002 | -0.281 | 0.021 |
| Bleak | 1146 | 3 | 1 | 2 | -0.349 | 0.040 | -0.216 | 0.078 | 0.163 | 0.096 | 0.499 | 0.075 | 0.521 | -0.012 | 0.210 | -0.100 | 0.030 | -0.097 | -0.171 | -0.044 | -0.227 | -0.059 | -0.171 | 0.001 | -0.289 | 0.021 |
| Bleak | 1147 | 3 | 1 | 2 | -0.352 | 0.044 | -0.220 | 0.077 | 0.146 | 0.101 | 0.496 | 0.075 | 0.516 | -0.017 | 0.218 | -0.103 | 0.054 | -0.096 | -0.167 | -0.048 | -0.226 | -0.060 | -0.176 | -0.004 | -0.290 | 0.031 |
| Bleak | 1148 | 3 | 1 | 2 | -0.355 | 0.019 | -0.222 | 0.074 | 0.137 | 0.127 | 0.523 | 0.057 | 0.509 | -0.039 | 0.209 | -0.077 | 0.038 | -0.075 | -0.161 | -0.037 | -0.219 | -0.065 | -0.171 | 0.002 | -0.289 | 0.015 |
| Bleak | 1149 | 3 | 1 | 2 | -0.352 | 0.034 | -0.221 | 0.085 | 0.142 | 0.114 | 0.508 | 0.069 | 0.504 | -0.021 | 0.226 | -0.095 | 0.046 | -0.104 | -0.172 | -0.045 | -0.226 | -0.057 | -0.171 | -0.001 | -0.284 | 0.021 |
| Bleak | 1150 | 3 | 1 | 2 | -0.347 | 0.026 | -0.219 | 0.075 | 0.140 | 0.102 | 0.520 | 0.063 | 0.518 | -0.022 | 0.210 | -0.082 | 0.030 | -0.083 | -0.171 | -0.037 | -0.224 | -0.056 | -0.174 | -0.007 | -0.284 | 0.020 |
| Bleak | 1151 | 3 | 1 | 2 | -0.342 | 0.050 | -0.216 | 0.080 | 0.153 | 0.088 | 0.504 | 0.078 | 0.511 | -0.009 | 0.223 | -0.096 | 0.042 | -0.103 | -0.176 | -0.049 | -0.232 | -0.053 | -0.180 | -0.011 | -0.287 | 0.025 |
| Bleak | 1152 | 3 | 1 | 2 | -0.343 | 0.029 | -0.224 | 0.076 | 0.149 | 0.122 | 0.520 | 0.055 | 0.500 | -0.025 | 0.220 | -0.075 | 0.044 | -0.092 | -0.174 | -0.046 | -0.234 | -0.059 | -0.174 | -0.007 | -0.283 | 0.021 |
| Bleak | 1153 | 3 | 1 | 2 | -0.357 | 0.034 | -0.219 | 0.082 | 0.150 | 0.106 | 0.514 | 0.070 | 0.510 | -0.017 | 0.206 | -0.094 | 0.035 | -0.098 | -0.160 | -0.041 | -0.226 | -0.058 | -0.169 | -0.004 | -0.285 | 0.020 |
| Bleak | 1154 | 3 | 1 | 2 | -0.346 | 0.028 | -0.220 | 0.077 | 0.149 | 0.118 | 0.517 | 0.063 | 0.509 | -0.021 | 0.210 | -0.087 | 0.033 | -0.094 | -0.160 | -0.040 | -0.231 | -0.059 | -0.179 | -0.003 | -0.284 | 0.018 |
| Bleak | 1155 | 3 | 1 | 2 | -0.346 | 0.029 | -0.226 | 0.078 | 0.145 | 0.115 | 0.517 | 0.062 | 0.508 | -0.021 | 0.217 | -0.086 | 0.031 | -0.095 | -0.162 | -0.044 | -0.225 | -0.056 | -0.174 | -0.002 | -0.286 | 0.021 |
| Bleak | 1156 | 3 | 1 | 2 | -0.361 | 0.029 | -0.229 | 0.078 | 0.144 | 0.113 | 0.514 | 0.060 | 0.507 | -0.027 | 0.219 | -0.084 | 0.038 | -0.086 | -0.162 | -0.039 | -0.217 | -0.061 | -0.168 | -0.001 | -0.285 | 0.018 |
| Bleak | 1157 | 3 | 1 | 2 | -0.351 | 0.046 | -0.212 | 0.084 | 0.150 | 0.095 | 0.499 | 0.076 | 0.521 | -0.002 | 0.211 | -0.108 | 0.035 | -0.109 | -0.168 | -0.044 | -0.231 | -0.054 | -0.170 | -0.009 | -0.284 | 0.026 |
| Bleak | 1158 | 3 | 1 | 2 | -0.367 | 0.031 | -0.223 | 0.079 | 0.168 | 0.123 | 0.492 | 0.059 | 0.495 | -0.022 | 0.237 | -0.093 | 0.050 | -0.096 | -0.162 | -0.041 | -0.229 | -0.068 | -0.170 | 0.007 | -0.293 | 0.022 |
| Bleak | 1159 | 3 | 1 | 2 | -0.358 | 0.023 | -0.220 | 0.071 | 0.163 | 0.104 | 0.519 | 0.062 | 0.503 | -0.021 | 0.209 | -0.080 | 0.037 | -0.081 | -0.162 | -0.037 | -0.235 | -0.064 | -0.173 | 0.000 | -0.283 | 0.023 |
| Bleak | 1160 | 3 | 1 | 2 | -0.348 | 0.038 | -0.230 | 0.077 | 0.136 | 0.111 | 0.510 | 0.065 | 0.518 | -0.017 | 0.211 | -0.093 | 0.040 | -0.103 | -0.164 | -0.043 | -0.226 | -0.053 | -0.168 | 0.000 | -0.280 | 0.018 |
| Bleak | 1131 | 3 | 1 | 3 | -0.350 | 0.042 | -0.215 | 0.080 | 0.146 | 0.115 | 0.502 | 0.069 | 0.515 | -0.025 | 0.218 | -0.090 | 0.042 | -0.095 | -0.173 | -0.045 | -0.221 | -0.063 | -0.173 | -0.008 | -0.291 | 0.021 |
| Bleak | 1132 | 3 | 1 | 3 | -0.359 | 0.012 | -0.228 | 0.080 | 0.138 | 0.112 | 0.517 | 0.051 | 0.513 | -0.029 | 0.211 | -0.078 | 0.046 | -0.075 | -0.164 | -0.036 | -0.215 | -0.059 | -0.170 | 0.005 | -0.290 | 0.017 |
| Bleak | 1133 | 3 | 1 | 3 | -0.349 | 0.034 | -0.214 | 0.083 | 0.152 | 0.114 | 0.504 | 0.065 | 0.513 | -0.015 | 0.218 | -0.098 | 0.036 | -0.093 | -0.165 | -0.045 | -0.228 | -0.059 | -0.176 | -0.009 | -0.290 | 0.023 |
| Bleak | 1134 | 3 | 1 | 3 | -0.358 | 0.020 | -0.220 | 0.080 | 0.141 | 0.112 | 0.512 | 0.062 | 0.518 | -0.029 | 0.214 | -0.081 | 0.027 | -0.087 | -0.161 | -0.036 | -0.222 | -0.061 | -0.167 | -0.001 | -0.284 | 0.020 |
| Bleak | 1135 | 3 | 1 | 3 | -0.354 | 0.013 | -0.219 | 0.075 | 0.144 | 0.117 | 0.516 | 0.051 | 0.506 | -0.026 | 0.230 | -0.075 | 0.028 | -0.089 | -0.171 | -0.033 | -0.222 | -0.060 | -0.169 | 0.011 | -0.289 | 0.016 |
| Bleak | 1136 | 3 | 1 | 3 | -0.350 | 0.012 | -0.218 | 0.078 | 0.148 | 0.124 | 0.521 | 0.059 | 0.520 | -0.028 | 0.191 | -0.084 | 0.027 | -0.084 | -0.163 | -0.037 | -0.216 | -0.063 | -0.172 | 0.005 | -0.287 | 0.017 |
| Bleak | 1137 | 3 | 1 | 3 | -0.361 | 0.025 | -0.226 | 0.077 | 0.149 | 0.111 | 0.512 | 0.064 | 0.516 | -0.022 | 0.201 | -0.090 | 0.037 | -0.093 | -0.163 | -0.041 | -0.214 | -0.061 | -0.168 | 0.006 | -0.284 | 0.023 |
| Bleak | 1138 | 3 | 1 | 3 | -0.359 | 0.029 | -0.228 | 0.086 | 0.142 | 0.130 | 0.516 | 0.058 | 0.507 | -0.029 | 0.210 | -0.088 | 0.038 | -0.090 | -0.160 | -0.046 | -0.211 | -0.060 | -0.171 | -0.006 | -0.284 | 0.014 |
| Bleak | 1139 | 3 | 1 | 3 | -0.354 | 0.029 | -0.224 | 0.079 | 0.143 | 0.122 | 0.504 | 0.062 | 0.512 | -0.032 | 0.226 | -0.089 | 0.038 | -0.084 | -0.162 | -0.039 | -0.219 | -0.062 | -0.173 | -0.003 | -0.291 | 0.017 |
| Bleak | 1140 | 3 | 1 | 3 | -0.349 | 0.013 | -0.235 | 0.078 | 0.144 | 0.136 | 0.526 | 0.045 | 0.499 | -0.033 | 0.216 | -0.075 | 0.041 | -0.083 | -0.167 | -0.034 | -0.208 | -0.060 | -0.175 | 0.004 | -0.292 | 0.009 |
| Bleak | 1141 | 3 | 1 | 3 | -0.360 | 0.020 | -0.221 | 0.080 | 0.157 | 0.120 | 0.512 | 0.056 | 0.502 | -0.026 | 0.222 | -0.077 | 0.036 | -0.088 | -0.168 | -0.043 | -0.224 | -0.058 | -0.169 | -0.005 | -0.286 | 0.021 |
| Bleak | 1142 | 3 | 1 | 3 | -0.351 | 0.032 | -0.216 | 0.075 | 0.137 | 0.107 | 0.507 | 0.067 | 0.528 | -0.018 | 0.203 | -0.094 | 0.034 | -0.096 | -0.169 | -0.041 | -0.228 | -0.060 | -0.163 | 0.006 | -0.282 | 0.022 |
| Bleak | 1143 | 3 | 1 | 3 | -0.343 | 0.030 | -0.215 | 0.080 | 0.143 | 0.114 | 0.514 | 0.074 | 0.523 | -0.024 | 0.198 | -0.093 | 0.030 | -0.097 | -0.169 | -0.045 | -0.226 | -0.058 | -0.173 | 0.000 | -0.281 | 0.020 |
| Bleak | 1144 | 3 | 1 | 3 | -0.353 | 0.037 | -0.219 | 0.072 | 0.146 | 0.096 | 0.517 | 0.066 | 0.514 | -0.022 | 0.205 | -0.079 | 0.047 | -0.083 | -0.170 | -0.050 | -0.229 | -0.059 | -0.172 | 0.002 | -0.286 | 0.020 |
| Bleak | 1145 | 3 | 1 | 3 | -0.352 | 0.027 | -0.218 | 0.081 | 0.136 | 0.114 | 0.520 | 0.065 | 0.515 | -0.023 | 0.205 | -0.089 | 0.034 | -0.093 | -0.168 | -0.041 | -0.219 | -0.059 | -0.170 | -0.002 | -0.283 | 0.020 |
| Bleak | 1146 | 3 | 1 | 3 | -0.352 | 0.038 | -0.211 | 0.078 | 0.149 | 0.094 | 0.507 | 0.075 | 0.519 | -0.015 | 0.210 | -0.096 | 0.032 | -0.096 | -0.168 | -0.041 | -0.226 | -0.060 | -0.169 | 0.000 | -0.290 | 0.023 |
| Bleak | 1147 | 3 | 1 | 3 | -0.358 | 0.044 | -0.215 | 0.077 | 0.145 | 0.103 | 0.498 | 0.071 | 0.513 | -0.015 | 0.214 | -0.103 | 0.062 | -0.096 | -0.168 | -0.046 | -0.223 | -0.060 | -0.176 | -0.007 | -0.293 | 0.031 |
| Bleak | 1148 | 3 | 1 | 3 | -0.354 | 0.018 | -0.219 | 0.075 | 0.134 | 0.123 | 0.526 | 0.055 | 0.514 | -0.038 | 0.204 | -0.073 | 0.032 | -0.070 | -0.163 | -0.040 | -0.219 | -0.064 | -0.167 | 0.000 | -0.290 | 0.013 |
| Bleak | 1149 | 3 | 1 | 3 | -0.353 | 0.035 | -0.215 | 0.088 | 0.143 | 0.114 | 0.513 | 0.069 | 0.504 | -0.020 | 0.223 | -0.093 | 0.035 | -0.102 | -0.173 | -0.044 | -0.226 | -0.060 | -0.168 | -0.009 | -0.282 | 0.021 |
| Bleak | 1150 | 3 | 1 | 3 | -0.349 | 0.023 | -0.218 | 0.076 | 0.137 | 0.104 | 0.522 | 0.064 | 0.516 | -0.026 | 0.212 | -0.080 | 0.028 | -0.083 | -0.168 | -0.038 | -0.224 | -0.055 | -0.173 | -0.005 | -0.283 | 0.021 |
| Bleak | 1151 | 3 | 1 | 3 | -0.345 | 0.042 | -0.216 | 0.080 | 0.153 | 0.085 | 0.505 | 0.075 | 0.513 | -0.008 | 0.223 | -0.096 | 0.036 | -0.101 | -0.176 | -0.043 | -0.227 | -0.054 | -0.177 | -0.004 | -0.289 | 0.024 |
| Bleak | 1152 | 3 | 1 | 3 | -0.344 | 0.026 | -0.219 | 0.078 | 0.142 | 0.123 | 0.518 | 0.055 | 0.508 | -0.026 | 0.218 | -0.074 | 0.040 | -0.092 | -0.177 | -0.046 | -0.228 | -0.060 | -0.172 | -0.005 | -0.284 | 0.021 |
| Bleak | 1153 | 3 | 1 | 3 | -0.359 | 0.032 | -0.220 | 0.085 | 0.139 | 0.105 | 0.521 | 0.070 | 0.514 | -0.019 | 0.201 | -0.092 | 0.030 | -0.095 | -0.160 | -0.039 | -0.217 | -0.060 | -0.165 | -0.005 | -0.285 | 0.020 |
| Bleak | 1154 | 3 | 1 | 3 | -0.350 | 0.026 | -0.217 | 0.079 | 0.139 | 0.119 | 0.520 | 0.058 | 0.511 | -0.023 | 0.211 | -0.085 | 0.037 | -0.090 | -0.165 | -0.041 | -0.225 | -0.061 | -0.174 | 0.001 | -0.286 | 0.017 |
| Bleak | 1155 | 3 | 1 | 3 | -0.348 | 0.027 | -0.229 | 0.076 | 0.143 | 0.113 | 0.518 | 0.063 | 0.513 | -0.026 | 0.215 | -0.084 | 0.025 | -0.092 | -0.161 | -0.042 | -0.216 | -0.057 | -0.172 | 0.004 | -0.286 | 0.018 |
| Bleak | 1156 | 3 | 1 | 3 | -0.359 | 0.024 | -0.221 | 0.079 | 0.135 | 0.110 | 0.524 | 0.059 | 0.511 | -0.024 | 0.211 | -0.081 | 0.030 | -0.085 | -0.166 | -0.039 | -0.216 | -0.057 | -0.167 | -0.002 | -0.283 | 0.018 |
| Bleak | 1157 | 3 | 1 | 3 | -0.357 | 0.043 | -0.207 | 0.085 | 0.144 | 0.095 | 0.499 | 0.074 | 0.522 | -0.004 | 0.206 | -0.106 | 0.046 | -0.109 | -0.169 | -0.046 | -0.227 | -0.055 | -0.170 | -0.005 | -0.289 | 0.028 |
| Bleak | 1158 | 3 | 1 | 3 | -0.368 | 0.029 | -0.223 | 0.081 | 0.160 | 0.121 | 0.491 | 0.059 | 0.498 | -0.025 | 0.235 | -0.090 | 0.059 | -0.099 | -0.163 | -0.038 | -0.234 | -0.065 | -0.165 | 0.003 | -0.292 | 0.023 |
| Bleak | 1159 | 3 | 1 | 3 | -0.359 | 0.022 | -0.215 | 0.068 | 0.153 | 0.101 | 0.517 | 0.059 | 0.505 | -0.024 | 0.224 | -0.076 | 0.030 | -0.075 | -0.162 | -0.034 | -0.237 | -0.064 | -0.171 | -0.001 | -0.287 | 0.024 |
| Bleak | 1160 | 3 | 1 | 3 | -0.351 | 0.036 | -0.223 | 0.078 | 0.133 | 0.111 | 0.513 | 0.064 | 0.515 | -0.017 | 0.219 | -0.092 | 0.030 | -0.102 | -0.171 | -0.040 | -0.218 | -0.058 | -0.166 | 0.001 | -0.282 | 0.019 |
| Bleak | 1221 | 3 | 3 | 1 | -0.369 | 0.035 | -0.207 | 0.092 | 0.139 | 0.122 | 0.499 | 0.066 | 0.515 | -0.025 | 0.224 | -0.087 | 0.031 | -0.103 | -0.161 | -0.053 | -0.231 | -0.068 | -0.162 | -0.003 | -0.278 | 0.024 |
| Bleak | 1222 | 3 | 3 | 1 | -0.362 | 0.039 | -0.223 | 0.078 | 0.149 | 0.116 | 0.515 | 0.057 | 0.494 | -0.026 | 0.226 | -0.076 | 0.050 | -0.089 | -0.162 | -0.043 | -0.217 | -0.062 | -0.177 | -0.011 | -0.294 | 0.018 |
| Bleak | 1223 | 3 | 3 | 1 | -0.353 | 0.014 | -0.228 | 0.074 | 0.147 | 0.128 | 0.516 | 0.042 | 0.514 | -0.038 | 0.209 | -0.062 | 0.043 | -0.068 | -0.154 | -0.034 | -0.220 | -0.066 | -0.178 | -0.001 | -0.295 | 0.010 |
| Bleak | 1224 | 3 | 3 | 1 | -0.352 | 0.048 | -0.207 | 0.082 | 0.147 | 0.098 | 0.505 | 0.080 | 0.521 | -0.010 | 0.204 | -0.105 | 0.031 | -0.103 | -0.165 | -0.046 | -0.225 | -0.064 | -0.172 | -0.009 | -0.288 | 0.029 |
| Bleak | 1225 | 3 | 3 | 1 | -0.355 | 0.065 | -0.198 | 0.081 | 0.123 | 0.081 | 0.504 | 0.081 | 0.518 | -0.003 | 0.230 | -0.099 | 0.036 | -0.115 | -0.169 | -0.053 | -0.232 | -0.060 | -0.174 | -0.013 | -0.283 | 0.035 |
| Bleak | 1226 | 3 | 3 | 1 | -0.350 | 0.041 | -0.211 | 0.083 | 0.154 | 0.102 | 0.506 | 0.072 | 0.511 | -0.015 | 0.212 | -0.093 | 0.042 | -0.099 | -0.161 | -0.045 | -0.233 | -0.061 | -0.178 | -0.008 | -0.293 | 0.023 |
| Bleak | 1227 | 3 | 3 | 1 | -0.357 | 0.043 | -0.210 | 0.079 | 0.147 | 0.107 | 0.506 | 0.071 | 0.500 | -0.016 | 0.231 | -0.095 | 0.042 | -0.105 | -0.160 | -0.046 | -0.236 | -0.061 | -0.175 | -0.007 | -0.288 | 0.029 |
| Bleak | 1228 | 3 | 3 | 1 | -0.364 | 0.015 | -0.222 | 0.079 | 0.145 | 0.142 | 0.523 | 0.039 | 0.505 | -0.046 | 0.208 | -0.051 | 0.039 | -0.069 | -0.152 | -0.041 | -0.217 | -0.066 | -0.172 | -0.006 | -0.293 | 0.005 |
| Bleak | 1229 | 3 | 3 | 1 | -0.357 | 0.047 | -0.205 | 0.082 | 0.135 | 0.116 | 0.513 | 0.064 | 0.513 | -0.021 | 0.209 | -0.082 | 0.042 | -0.093 | -0.163 | -0.054 | -0.229 | -0.071 | -0.170 | -0.017 | -0.287 | 0.029 |
| Bleak | 1230 | 3 | 3 | 1 | -0.359 | 0.043 | -0.217 | 0.082 | 0.156 | 0.116 | 0.510 | 0.070 | 0.512 | -0.021 | 0.204 | -0.090 | 0.029 | -0.094 | -0.156 | -0.044 | -0.225 | -0.064 | -0.169 | -0.017 | -0.286 | 0.019 |
| Bleak | 1232 | 3 | 3 | 1 | -0.358 | 0.044 | -0.215 | 0.076 | 0.147 | 0.116 | 0.504 | 0.068 | 0.507 | -0.021 | 0.217 | -0.094 | 0.052 | -0.098 | -0.163 | -0.042 | -0.230 | -0.064 | -0.170 | -0.009 | -0.291 | 0.024 |
| Bleak | 1233 | 3 | 3 | 1 | -0.359 | 0.029 | -0.215 | 0.083 | 0.152 | 0.121 | 0.504 | 0.065 | 0.515 | -0.030 | 0.208 | -0.085 | 0.041 | -0.086 | -0.158 | -0.042 | -0.234 | -0.061 | -0.170 | -0.009 | -0.285 | 0.017 |
| Bleak | 1234 | 3 | 3 | 1 | -0.365 | 0.033 | -0.224 | 0.082 | 0.161 | 0.127 | 0.511 | 0.065 | 0.508 | -0.026 | 0.203 | -0.092 | 0.028 | -0.090 | -0.157 | -0.044 | -0.216 | -0.062 | -0.164 | -0.006 | -0.286 | 0.013 |
| Bleak | 1235 | 3 | 3 | 1 | -0.351 | 0.051 | -0.204 | 0.081 | 0.131 | 0.098 | 0.510 | 0.072 | 0.511 | -0.015 | 0.227 | -0.086 | 0.041 | -0.103 | -0.174 | -0.053 | -0.238 | -0.059 | -0.172 | -0.015 | -0.283 | 0.030 |
| Bleak | 1236 | 3 | 3 | 1 | -0.364 | 0.021 | -0.225 | 0.087 | 0.135 | 0.124 | 0.526 | 0.051 | 0.507 | -0.031 | 0.204 | -0.074 | 0.036 | -0.082 | -0.153 | -0.043 | -0.210 | -0.060 | -0.166 | -0.004 | -0.290 | 0.011 |
| Bleak | 1237 | 3 | 3 | 1 | -0.362 | 0.031 | -0.223 | 0.084 | 0.156 | 0.129 | 0.518 | 0.052 | 0.495 | -0.028 | 0.211 | -0.078 | 0.050 | -0.081 | -0.159 | -0.044 | -0.219 | -0.068 | -0.174 | -0.011 | -0.293 | 0.014 |
| Bleak | 1238 | 3 | 3 | 1 | -0.357 | 0.029 | -0.222 | 0.084 | 0.157 | 0.122 | 0.507 | 0.058 | 0.504 | -0.025 | 0.220 | -0.084 | 0.042 | -0.089 | -0.162 | -0.045 | -0.225 | -0.062 | -0.173 | -0.004 | -0.290 | 0.016 |
| Bleak | 1239 | 3 | 3 | 1 | -0.357 | 0.043 | -0.219 | 0.083 | 0.149 | 0.116 | 0.509 | 0.063 | 0.498 | -0.021 | 0.222 | -0.084 | 0.051 | -0.102 | -0.162 | -0.050 | -0.230 | -0.065 | -0.168 | -0.007 | -0.292 | 0.023 |
| Bleak | 1240 | 3 | 3 | 1 | -0.356 | 0.024 | -0.225 | 0.079 | 0.148 | 0.132 | 0.517 | 0.053 | 0.511 | -0.038 | 0.204 | -0.072 | 0.037 | -0.076 | -0.152 | -0.040 | -0.219 | -0.062 | -0.174 | -0.007 | -0.292 | 0.007 |
| Bleak | 1241 | 3 | 3 | 1 | -0.360 | 0.033 | -0.220 | 0.084 | 0.150 | 0.118 | 0.508 | 0.066 | 0.516 | -0.029 | 0.213 | -0.084 | 0.024 | -0.088 | -0.165 | -0.049 | -0.227 | -0.064 | -0.161 | -0.006 | -0.278 | 0.017 |
| Bleak | 1242 | 3 | 3 | 1 | -0.356 | 0.036 | -0.208 | 0.083 | 0.153 | 0.106 | 0.511 | 0.071 | 0.508 | -0.012 | 0.209 | -0.095 | 0.038 | -0.106 | -0.164 | -0.039 | -0.227 | -0.065 | -0.177 | -0.009 | -0.286 | 0.029 |
| Bleak | 1243 | 3 | 3 | 1 | -0.347 | 0.060 | -0.203 | 0.081 | 0.152 | 0.089 | 0.500 | 0.085 | 0.511 | 0.000 | 0.225 | -0.106 | 0.034 | -0.120 | -0.169 | -0.050 | -0.246 | -0.051 | -0.177 | -0.018 | -0.281 | 0.030 |
| Bleak | 1244 | 3 | 3 | 1 | -0.356 | 0.035 | -0.223 | 0.085 | 0.139 | 0.128 | 0.514 | 0.063 | 0.518 | -0.028 | 0.197 | -0.087 | 0.039 | -0.091 | -0.157 | -0.040 | -0.223 | -0.061 | -0.167 | -0.017 | -0.280 | 0.014 |
| Bleak | 1245 | 3 | 3 | 1 | -0.370 | 0.001 | -0.246 | 0.079 | 0.168 | 0.153 | 0.520 | 0.024 | 0.486 | -0.045 | 0.213 | -0.061 | 0.062 | -0.053 | -0.150 | -0.032 | -0.212 | -0.068 | -0.170 | -0.001 | -0.301 | 0.002 |
| Bleak | 1246 | 3 | 3 | 1 | -0.346 | 0.059 | -0.201 | 0.087 | 0.145 | 0.089 | 0.501 | 0.090 | 0.524 | 0.005 | 0.205 | -0.119 | 0.029 | -0.123 | -0.173 | -0.049 | -0.231 | -0.056 | -0.172 | -0.015 | -0.282 | 0.032 |
| Bleak | 1247 | 3 | 3 | 1 | -0.359 | 0.025 | -0.217 | 0.082 | 0.147 | 0.124 | 0.515 | 0.060 | 0.508 | -0.031 | 0.206 | -0.081 | 0.043 | -0.084 | -0.160 | -0.042 | -0.223 | -0.063 | -0.171 | -0.007 | -0.290 | 0.017 |
| Bleak | 1248 | 3 | 3 | 1 | -0.363 | 0.059 | -0.200 | 0.085 | 0.140 | 0.092 | 0.494 | 0.079 | 0.514 | -0.002 | 0.230 | -0.110 | 0.037 | -0.110 | -0.163 | -0.051 | -0.237 | -0.064 | -0.167 | -0.011 | -0.285 | 0.034 |
| Bleak | 1249 | 3 | 3 | 1 | -0.359 | 0.058 | -0.209 | 0.081 | 0.148 | 0.088 | 0.494 | 0.074 | 0.520 | 0.000 | 0.218 | -0.101 | 0.039 | -0.109 | -0.163 | -0.052 | -0.237 | -0.061 | -0.167 | -0.009 | -0.285 | 0.031 |
| Bleak | 1250 | 3 | 3 | 1 | -0.352 | 0.039 | -0.212 | 0.081 | 0.148 | 0.101 | 0.511 | 0.065 | 0.505 | -0.015 | 0.223 | -0.086 | 0.040 | -0.095 | -0.163 | -0.042 | -0.233 | -0.063 | -0.175 | -0.009 | -0.292 | 0.023 |
| Bleak | 1250 | 3 | 3 | 1 | -0.354 | 0.038 | -0.221 | 0.087 | 0.148 | 0.112 | 0.503 | 0.068 | 0.510 | -0.022 | 0.215 | -0.090 | 0.048 | -0.100 | -0.163 | -0.043 | -0.226 | -0.063 | -0.172 | -0.008 | -0.289 | 0.022 |
| Bleak | 1221 | 3 | 3 | 2 | -0.357 | 0.049 | -0.211 | 0.090 | 0.137 | 0.119 | 0.502 | 0.069 | 0.518 | -0.023 | 0.218 | -0.086 | 0.031 | -0.104 | -0.164 | -0.056 | -0.231 | -0.070 | -0.162 | -0.010 | -0.280 | 0.022 |
| Bleak | 1222 | 3 | 3 | 2 | -0.358 | 0.038 | -0.218 | 0.078 | 0.148 | 0.116 | 0.513 | 0.061 | 0.502 | -0.029 | 0.221 | -0.076 | 0.047 | -0.089 | -0.163 | -0.045 | -0.221 | -0.059 | -0.178 | -0.010 | -0.291 | 0.015 |
| Bleak | 1223 | 3 | 3 | 2 | -0.352 | 0.018 | -0.231 | 0.076 | 0.148 | 0.126 | 0.516 | 0.043 | 0.516 | -0.038 | 0.205 | -0.063 | 0.042 | -0.067 | -0.154 | -0.036 | -0.220 | -0.067 | -0.176 | 0.000 | -0.293 | 0.008 |
| Bleak | 1224 | 3 | 3 | 2 | -0.350 | 0.051 | -0.208 | 0.083 | 0.142 | 0.096 | 0.506 | 0.081 | 0.523 | -0.009 | 0.202 | -0.106 | 0.031 | -0.102 | -0.165 | -0.046 | -0.224 | -0.064 | -0.170 | -0.009 | -0.288 | 0.027 |
| Bleak | 1225 | 3 | 3 | 2 | -0.350 | 0.069 | -0.198 | 0.078 | 0.124 | 0.080 | 0.511 | 0.083 | 0.514 | -0.002 | 0.228 | -0.099 | 0.035 | -0.111 | -0.171 | -0.052 | -0.237 | -0.054 | -0.173 | -0.022 | -0.282 | 0.032 |
| Bleak | 1226 | 3 | 3 | 2 | -0.348 | 0.044 | -0.208 | 0.082 | 0.153 | 0.103 | 0.511 | 0.077 | 0.510 | -0.016 | 0.208 | -0.096 | 0.039 | -0.099 | -0.164 | -0.045 | -0.232 | -0.064 | -0.178 | -0.010 | -0.291 | 0.024 |
| Bleak | 1227 | 3 | 3 | 2 | -0.353 | 0.043 | -0.209 | 0.077 | 0.144 | 0.107 | 0.514 | 0.072 | 0.502 | -0.016 | 0.226 | -0.095 | 0.033 | -0.101 | -0.165 | -0.048 | -0.229 | -0.062 | -0.175 | -0.004 | -0.287 | 0.026 |
| Bleak | 1228 | 3 | 3 | 2 | -0.364 | 0.020 | -0.226 | 0.077 | 0.144 | 0.143 | 0.523 | 0.040 | 0.507 | -0.049 | 0.209 | -0.050 | 0.035 | -0.068 | -0.154 | -0.041 | -0.214 | -0.067 | -0.170 | -0.005 | -0.289 | 0.001 |
| Bleak | 1229 | 3 | 3 | 2 | -0.350 | 0.051 | -0.203 | 0.080 | 0.127 | 0.113 | 0.525 | 0.064 | 0.510 | -0.019 | 0.205 | -0.080 | 0.039 | -0.092 | -0.163 | -0.057 | -0.233 | -0.072 | -0.172 | -0.010 | -0.284 | 0.023 |
| Bleak | 1230 | 3 | 3 | 2 | -0.358 | 0.045 | -0.220 | 0.080 | 0.148 | 0.116 | 0.514 | 0.070 | 0.514 | -0.023 | 0.201 | -0.091 | 0.028 | -0.096 | -0.154 | -0.045 | -0.217 | -0.066 | -0.168 | -0.011 | -0.287 | 0.019 |
| Bleak | 1232 | 3 | 3 | 2 | -0.353 | 0.045 | -0.216 | 0.075 | 0.147 | 0.115 | 0.507 | 0.068 | 0.507 | -0.021 | 0.216 | -0.093 | 0.050 | -0.097 | -0.165 | -0.042 | -0.233 | -0.063 | -0.170 | -0.009 | -0.290 | 0.022 |
| Bleak | 1233 | 3 | 3 | 2 | -0.359 | 0.033 | -0.217 | 0.083 | 0.143 | 0.121 | 0.503 | 0.064 | 0.517 | -0.031 | 0.212 | -0.087 | 0.044 | -0.088 | -0.160 | -0.042 | -0.225 | -0.068 | -0.172 | -0.002 | -0.286 | 0.017 |
| Bleak | 1234 | 3 | 3 | 2 | -0.366 | 0.029 | -0.224 | 0.080 | 0.157 | 0.125 | 0.511 | 0.065 | 0.512 | -0.029 | 0.202 | -0.090 | 0.030 | -0.088 | -0.156 | -0.043 | -0.213 | -0.060 | -0.167 | -0.003 | -0.284 | 0.014 |
| Bleak | 1235 | 3 | 3 | 2 | -0.351 | 0.054 | -0.206 | 0.082 | 0.135 | 0.099 | 0.507 | 0.069 | 0.510 | -0.012 | 0.226 | -0.086 | 0.045 | -0.107 | -0.172 | -0.056 | -0.239 | -0.060 | -0.171 | -0.014 | -0.284 | 0.030 |
| Bleak | 1236 | 3 | 3 | 2 | -0.364 | 0.024 | -0.219 | 0.087 | 0.132 | 0.125 | 0.527 | 0.054 | 0.507 | -0.030 | 0.203 | -0.077 | 0.037 | -0.085 | -0.157 | -0.042 | -0.216 | -0.061 | -0.164 | -0.001 | -0.287 | 0.008 |
| Bleak | 1237 | 3 | 3 | 2 | -0.363 | 0.031 | -0.224 | 0.081 | 0.153 | 0.129 | 0.519 | 0.052 | 0.500 | -0.030 | 0.204 | -0.077 | 0.051 | -0.082 | -0.158 | -0.041 | -0.221 | -0.069 | -0.170 | -0.005 | -0.290 | 0.012 |
| Bleak | 1238 | 3 | 3 | 2 | -0.357 | 0.025 | -0.220 | 0.083 | 0.158 | 0.123 | 0.508 | 0.060 | 0.506 | -0.026 | 0.218 | -0.085 | 0.035 | -0.088 | -0.163 | -0.045 | -0.228 | -0.059 | -0.172 | -0.006 | -0.287 | 0.017 |
| Bleak | 1239 | 3 | 3 | 2 | -0.355 | 0.039 | -0.218 | 0.081 | 0.154 | 0.117 | 0.514 | 0.063 | 0.500 | -0.021 | 0.213 | -0.081 | 0.048 | -0.100 | -0.165 | -0.049 | -0.235 | -0.061 | -0.168 | -0.007 | -0.287 | 0.020 |
| Bleak | 1240 | 3 | 3 | 2 | -0.358 | 0.020 | -0.226 | 0.078 | 0.143 | 0.129 | 0.520 | 0.051 | 0.517 | -0.038 | 0.196 | -0.070 | 0.038 | -0.072 | -0.153 | -0.040 | -0.220 | -0.062 | -0.170 | -0.002 | -0.287 | 0.006 |
| Bleak | 1241 | 3 | 3 | 2 | -0.360 | 0.031 | -0.215 | 0.085 | 0.145 | 0.117 | 0.511 | 0.068 | 0.519 | -0.032 | 0.211 | -0.083 | 0.019 | -0.082 | -0.166 | -0.049 | -0.224 | -0.068 | -0.163 | -0.004 | -0.277 | 0.016 |
| Bleak | 1242 | 3 | 3 | 2 | -0.355 | 0.034 | -0.207 | 0.084 | 0.146 | 0.106 | 0.511 | 0.069 | 0.512 | -0.013 | 0.209 | -0.091 | 0.036 | -0.107 | -0.163 | -0.040 | -0.229 | -0.062 | -0.172 | -0.006 | -0.288 | 0.026 |
| Bleak | 1243 | 3 | 3 | 2 | -0.347 | 0.056 | -0.204 | 0.079 | 0.146 | 0.088 | 0.503 | 0.083 | 0.513 | 0.001 | 0.226 | -0.106 | 0.032 | -0.121 | -0.172 | -0.048 | -0.242 | -0.054 | -0.176 | -0.006 | -0.279 | 0.029 |
| Bleak | 1244 | 3 | 3 | 2 | -0.358 | 0.033 | -0.216 | 0.087 | 0.131 | 0.126 | 0.514 | 0.065 | 0.518 | -0.030 | 0.204 | -0.087 | 0.037 | -0.094 | -0.158 | -0.040 | -0.225 | -0.058 | -0.168 | -0.014 | -0.280 | 0.013 |
| Bleak | 1245 | 3 | 3 | 2 | -0.370 | -0.004 | -0.244 | 0.079 | 0.167 | 0.155 | 0.517 | 0.026 | 0.489 | -0.045 | 0.215 | -0.060 | 0.058 | -0.064 | -0.151 | -0.030 | -0.209 | -0.064 | -0.171 | 0.004 | -0.301 | 0.002 |
| Bleak | 1246 | 3 | 3 | 2 | -0.349 | 0.051 | -0.197 | 0.089 | 0.141 | 0.089 | 0.502 | 0.091 | 0.525 | 0.000 | 0.211 | -0.117 | 0.021 | -0.121 | -0.170 | -0.049 | -0.234 | -0.054 | -0.171 | -0.012 | -0.279 | 0.032 |
| Bleak | 1247 | 3 | 3 | 2 | -0.358 | 0.017 | -0.219 | 0.082 | 0.148 | 0.124 | 0.514 | 0.058 | 0.515 | -0.031 | 0.199 | -0.081 | 0.041 | -0.082 | -0.161 | -0.039 | -0.223 | -0.062 | -0.169 | 0.001 | -0.287 | 0.015 |
| Bleak | 1248 | 3 | 3 | 2 | -0.366 | 0.056 | -0.196 | 0.085 | 0.136 | 0.094 | 0.496 | 0.082 | 0.525 | -0.002 | 0.211 | -0.116 | 0.033 | -0.112 | -0.161 | -0.052 | -0.231 | -0.068 | -0.164 | 0.002 | -0.283 | 0.031 |
| Bleak | 1249 | 3 | 3 | 2 | -0.359 | 0.058 | -0.202 | 0.082 | 0.138 | 0.090 | 0.497 | 0.076 | 0.523 | -0.004 | 0.220 | -0.103 | 0.036 | -0.107 | -0.170 | -0.050 | -0.231 | -0.061 | -0.167 | -0.007 | -0.285 | 0.027 |
| Bleak | 1250 | 3 | 3 | 2 | -0.357 | 0.041 | -0.213 | 0.082 | 0.148 | 0.103 | 0.510 | 0.070 | 0.500 | -0.020 | 0.229 | -0.088 | 0.041 | -0.097 | -0.162 | -0.043 | -0.230 | -0.062 | -0.176 | -0.010 | -0.291 | 0.024 |
| Bleak | 1250 | 3 | 3 | 2 | -0.353 | 0.037 | -0.218 | 0.085 | 0.148 | 0.112 | 0.508 | 0.072 | 0.510 | -0.023 | 0.216 | -0.092 | 0.036 | -0.101 | -0.163 | -0.041 | -0.228 | -0.062 | -0.169 | -0.008 | -0.287 | 0.019 |
| Bleak | 1221 | 3 | 3 | 3 | -0.363 | 0.042 | -0.207 | 0.091 | 0.139 | 0.120 | 0.505 | 0.068 | 0.517 | -0.024 | 0.212 | -0.085 | 0.029 | -0.102 | -0.163 | -0.055 | -0.224 | -0.070 | -0.162 | -0.009 | -0.282 | 0.024 |
| Bleak | 1222 | 3 | 3 | 3 | -0.364 | 0.036 | -0.219 | 0.082 | 0.146 | 0.114 | 0.515 | 0.058 | 0.501 | -0.028 | 0.219 | -0.072 | 0.048 | -0.089 | -0.163 | -0.045 | -0.220 | -0.061 | -0.173 | -0.010 | -0.290 | 0.016 |
| Bleak | 1223 | 3 | 3 | 3 | -0.354 | 0.009 | -0.228 | 0.074 | 0.147 | 0.127 | 0.519 | 0.040 | 0.515 | -0.036 | 0.207 | -0.061 | 0.035 | -0.066 | -0.157 | -0.033 | -0.215 | -0.062 | -0.174 | 0.000 | -0.296 | 0.010 |
| Bleak | 1224 | 3 | 3 | 3 | -0.355 | 0.046 | -0.206 | 0.085 | 0.139 | 0.096 | 0.506 | 0.082 | 0.525 | -0.011 | 0.202 | -0.107 | 0.029 | -0.102 | -0.163 | -0.041 | -0.220 | -0.062 | -0.169 | -0.019 | -0.288 | 0.033 |
| Bleak | 1225 | 3 | 3 | 3 | -0.354 | 0.064 | -0.198 | 0.082 | 0.122 | 0.081 | 0.510 | 0.082 | 0.517 | -0.004 | 0.226 | -0.097 | 0.033 | -0.113 | -0.171 | -0.047 | -0.228 | -0.059 | -0.175 | -0.024 | -0.283 | 0.036 |
| Bleak | 1226 | 3 | 3 | 3 | -0.350 | 0.038 | -0.212 | 0.083 | 0.148 | 0.104 | 0.513 | 0.071 | 0.511 | -0.015 | 0.208 | -0.094 | 0.035 | -0.099 | -0.165 | -0.045 | -0.225 | -0.064 | -0.170 | -0.004 | -0.293 | 0.025 |
| Bleak | 1227 | 3 | 3 | 3 | -0.355 | 0.043 | -0.203 | 0.079 | 0.144 | 0.106 | 0.512 | 0.071 | 0.503 | -0.016 | 0.228 | -0.091 | 0.032 | -0.104 | -0.162 | -0.045 | -0.228 | -0.063 | -0.178 | -0.012 | -0.292 | 0.031 |
| Bleak | 1228 | 3 | 3 | 3 | -0.365 | 0.013 | -0.224 | 0.078 | 0.145 | 0.144 | 0.525 | 0.039 | 0.507 | -0.049 | 0.202 | -0.051 | 0.039 | -0.066 | -0.155 | -0.042 | -0.213 | -0.063 | -0.170 | -0.008 | -0.292 | 0.005 |
| Bleak | 1229 | 3 | 3 | 3 | -0.357 | 0.046 | -0.206 | 0.080 | 0.128 | 0.114 | 0.523 | 0.062 | 0.510 | -0.021 | 0.207 | -0.078 | 0.037 | -0.093 | -0.163 | -0.052 | -0.227 | -0.071 | -0.170 | -0.015 | -0.284 | 0.028 |
| Bleak | 1230 | 3 | 3 | 3 | -0.361 | 0.038 | -0.215 | 0.084 | 0.150 | 0.116 | 0.513 | 0.067 | 0.514 | -0.020 | 0.204 | -0.091 | 0.025 | -0.095 | -0.158 | -0.043 | -0.216 | -0.064 | -0.169 | -0.012 | -0.287 | 0.020 |
| Bleak | 1232 | 3 | 3 | 3 | -0.361 | 0.040 | -0.210 | 0.075 | 0.152 | 0.112 | 0.513 | 0.070 | 0.502 | -0.021 | 0.211 | -0.091 | 0.047 | -0.095 | -0.166 | -0.041 | -0.229 | -0.064 | -0.171 | -0.009 | -0.289 | 0.025 |
| Bleak | 1233 | 3 | 3 | 3 | -0.359 | 0.031 | -0.218 | 0.084 | 0.148 | 0.120 | 0.508 | 0.064 | 0.515 | -0.030 | 0.209 | -0.083 | 0.036 | -0.087 | -0.160 | -0.043 | -0.227 | -0.063 | -0.168 | -0.011 | -0.284 | 0.017 |
| Bleak | 1234 | 3 | 3 | 3 | -0.364 | 0.032 | -0.223 | 0.079 | 0.156 | 0.127 | 0.517 | 0.060 | 0.505 | -0.022 | 0.207 | -0.091 | 0.027 | -0.089 | -0.160 | -0.044 | -0.215 | -0.062 | -0.166 | -0.005 | -0.285 | 0.015 |
| Bleak | 1235 | 3 | 3 | 3 | -0.351 | 0.049 | -0.201 | 0.083 | 0.129 | 0.099 | 0.513 | 0.070 | 0.514 | -0.015 | 0.222 | -0.082 | 0.037 | -0.102 | -0.176 | -0.051 | -0.234 | -0.060 | -0.172 | -0.022 | -0.282 | 0.031 |
| Bleak | 1236 | 3 | 3 | 3 | -0.364 | 0.019 | -0.224 | 0.088 | 0.131 | 0.121 | 0.529 | 0.053 | 0.506 | -0.032 | 0.206 | -0.076 | 0.036 | -0.081 | -0.156 | -0.040 | -0.211 | -0.061 | -0.165 | -0.001 | -0.288 | 0.010 |
| Bleak | 1237 | 3 | 3 | 3 | -0.366 | 0.024 | -0.217 | 0.084 | 0.152 | 0.129 | 0.522 | 0.050 | 0.500 | -0.029 | 0.207 | -0.073 | 0.040 | -0.081 | -0.161 | -0.041 | -0.218 | -0.065 | -0.169 | -0.013 | -0.291 | 0.015 |
| Bleak | 1238 | 3 | 3 | 3 | -0.360 | 0.022 | -0.222 | 0.084 | 0.161 | 0.121 | 0.510 | 0.057 | 0.503 | -0.025 | 0.219 | -0.083 | 0.034 | -0.084 | -0.164 | -0.041 | -0.223 | -0.060 | -0.170 | -0.008 | -0.288 | 0.018 |
| Bleak | 1239 | 3 | 3 | 3 | -0.356 | 0.038 | -0.218 | 0.084 | 0.148 | 0.119 | 0.514 | 0.059 | 0.501 | -0.021 | 0.214 | -0.080 | 0.048 | -0.098 | -0.163 | -0.049 | -0.229 | -0.062 | -0.170 | -0.016 | -0.290 | 0.025 |
| Bleak | 1240 | 3 | 3 | 3 | -0.358 | 0.023 | -0.224 | 0.080 | 0.140 | 0.131 | 0.520 | 0.052 | 0.515 | -0.039 | 0.200 | -0.071 | 0.039 | -0.074 | -0.153 | -0.040 | -0.217 | -0.060 | -0.172 | -0.009 | -0.289 | 0.008 |
| Bleak | 1241 | 3 | 3 | 3 | -0.360 | 0.030 | -0.218 | 0.087 | 0.142 | 0.114 | 0.518 | 0.065 | 0.515 | -0.028 | 0.208 | -0.080 | 0.022 | -0.085 | -0.165 | -0.047 | -0.223 | -0.064 | -0.160 | -0.011 | -0.278 | 0.019 |
| Bleak | 1242 | 3 | 3 | 3 | -0.356 | 0.035 | -0.207 | 0.084 | 0.150 | 0.104 | 0.512 | 0.068 | 0.511 | -0.011 | 0.208 | -0.092 | 0.031 | -0.105 | -0.166 | -0.040 | -0.221 | -0.066 | -0.173 | -0.003 | -0.289 | 0.027 |
| Bleak | 1243 | 3 | 3 | 3 | -0.346 | 0.055 | -0.202 | 0.081 | 0.138 | 0.088 | 0.502 | 0.081 | 0.514 | -0.002 | 0.235 | -0.102 | 0.026 | -0.116 | -0.173 | -0.049 | -0.240 | -0.053 | -0.173 | -0.015 | -0.282 | 0.032 |
| Bleak | 1244 | 3 | 3 | 3 | -0.357 | 0.033 | -0.217 | 0.087 | 0.132 | 0.124 | 0.517 | 0.063 | 0.522 | -0.030 | 0.201 | -0.083 | 0.025 | -0.089 | -0.161 | -0.041 | -0.216 | -0.062 | -0.165 | -0.017 | -0.281 | 0.014 |
| Bleak | 1245 | 3 | 3 | 3 | -0.371 | -0.003 | -0.244 | 0.082 | 0.175 | 0.154 | 0.507 | 0.030 | 0.492 | -0.049 | 0.217 | -0.063 | 0.058 | -0.060 | -0.153 | -0.030 | -0.210 | -0.067 | -0.168 | 0.001 | -0.304 | 0.004 |
| Bleak | 1246 | 3 | 3 | 3 | -0.349 | 0.053 | -0.196 | 0.091 | 0.155 | 0.089 | 0.500 | 0.095 | 0.528 | 0.004 | 0.198 | -0.121 | 0.016 | -0.122 | -0.173 | -0.047 | -0.231 | -0.058 | -0.167 | -0.018 | -0.281 | 0.033 |
| Bleak | 1247 | 3 | 3 | 3 | -0.360 | 0.019 | -0.216 | 0.082 | 0.143 | 0.123 | 0.520 | 0.058 | 0.511 | -0.032 | 0.197 | -0.079 | 0.043 | -0.083 | -0.161 | -0.041 | -0.219 | -0.063 | -0.167 | 0.001 | -0.291 | 0.014 |
| Bleak | 1248 | 3 | 3 | 3 | -0.365 | 0.062 | -0.196 | 0.088 | 0.133 | 0.094 | 0.502 | 0.081 | 0.522 | 0.000 | 0.208 | -0.114 | 0.033 | -0.112 | -0.163 | -0.049 | -0.225 | -0.067 | -0.166 | -0.014 | -0.283 | 0.033 |
| Bleak | 1249 | 3 | 3 | 3 | -0.355 | 0.059 | -0.202 | 0.086 | 0.141 | 0.089 | 0.502 | 0.078 | 0.518 | -0.003 | 0.220 | -0.099 | 0.031 | -0.111 | -0.173 | -0.052 | -0.231 | -0.063 | -0.165 | -0.014 | -0.285 | 0.029 |
| Bleak | 1250 | 3 | 3 | 3 | -0.353 | 0.038 | -0.209 | 0.081 | 0.142 | 0.101 | 0.512 | 0.068 | 0.510 | -0.018 | 0.219 | -0.088 | 0.039 | -0.091 | -0.165 | -0.041 | -0.228 | -0.057 | -0.175 | -0.019 | -0.291 | 0.026 |
| Bleak | 1250 | 3 | 3 | 3 | -0.356 | 0.035 | -0.219 | 0.086 | 0.149 | 0.113 | 0.504 | 0.071 | 0.510 | -0.025 | 0.214 | -0.088 | 0.045 | -0.103 | -0.165 | -0.041 | -0.226 | -0.063 | -0.167 | -0.007 | -0.289 | 0.022 |
| Roach | 1001 | 1 | 1 | 1 | -0.344 | 0.015 | -0.214 | 0.085 | 0.076 | 0.167 | 0.488 | 0.080 | 0.506 | -0.011 | 0.271 | -0.105 | 0.049 | -0.130 | -0.162 | -0.052 | -0.229 | -0.069 | -0.158 | -0.002 | -0.284 | 0.021 |
| Roach | 1002 | 1 | 1 | 1 | -0.357 | 0.017 | -0.224 | 0.097 | 0.086 | 0.154 | 0.477 | 0.079 | 0.499 | -0.012 | 0.273 | -0.101 | 0.081 | -0.123 | -0.162 | -0.058 | -0.236 | -0.069 | -0.154 | -0.002 | -0.283 | 0.018 |
| Roach | 1003 | 1 | 1 | 1 | -0.351 | -0.008 | -0.242 | 0.082 | 0.070 | 0.189 | 0.520 | 0.047 | 0.480 | -0.035 | 0.264 | -0.070 | 0.067 | -0.097 | -0.154 | -0.051 | -0.224 | -0.074 | -0.155 | 0.011 | -0.274 | 0.006 |
| Roach | 1004 | 1 | 1 | 1 | -0.352 | 0.005 | -0.229 | 0.095 | 0.052 | 0.178 | 0.498 | 0.067 | 0.485 | -0.033 | 0.275 | -0.091 | 0.090 | -0.116 | -0.158 | -0.052 | -0.226 | -0.073 | -0.152 | 0.009 | -0.282 | 0.013 |
| Roach | 1005 | 1 | 1 | 1 | -0.344 | -0.003 | -0.232 | 0.077 | 0.091 | 0.170 | 0.488 | 0.070 | 0.484 | -0.029 | 0.283 | -0.092 | 0.082 | -0.111 | -0.169 | -0.045 | -0.225 | -0.074 | -0.166 | 0.022 | -0.294 | 0.015 |
| Roach | 1006 | 1 | 1 | 1 | -0.358 | 0.011 | -0.224 | 0.099 | 0.075 | 0.163 | 0.492 | 0.082 | 0.489 | -0.018 | 0.274 | -0.107 | 0.066 | -0.124 | -0.153 | -0.058 | -0.226 | -0.071 | -0.152 | 0.009 | -0.284 | 0.013 |
| Roach | 1007 | 1 | 1 | 1 | -0.356 | -0.019 | -0.234 | 0.095 | 0.062 | 0.215 | 0.509 | 0.053 | 0.477 | -0.057 | 0.270 | -0.073 | 0.071 | -0.094 | -0.151 | -0.052 | -0.228 | -0.077 | -0.141 | 0.013 | -0.278 | -0.003 |
| Roach | 1008 | 1 | 1 | 1 | -0.350 | 0.011 | -0.231 | 0.097 | 0.065 | 0.176 | 0.502 | 0.080 | 0.485 | -0.030 | 0.276 | -0.097 | 0.060 | -0.123 | -0.157 | -0.057 | -0.216 | -0.074 | -0.149 | 0.003 | -0.283 | 0.014 |
| Roach | 1009 | 1 | 1 | 1 | -0.353 | 0.016 | -0.224 | 0.096 | 0.075 | 0.164 | 0.488 | 0.079 | 0.489 | -0.022 | 0.280 | -0.097 | 0.071 | -0.122 | -0.159 | -0.060 | -0.233 | -0.074 | -0.150 | 0.001 | -0.283 | 0.018 |
| Roach | 1010 | 1 | 1 | 1 | -0.355 | 0.030 | -0.222 | 0.099 | 0.075 | 0.165 | 0.483 | 0.087 | 0.486 | -0.018 | 0.274 | -0.103 | 0.090 | -0.136 | -0.166 | -0.064 | -0.225 | -0.075 | -0.155 | -0.008 | -0.285 | 0.023 |
| Roach | 1011 | 1 | 1 | 1 | -0.343 | 0.020 | -0.223 | 0.097 | 0.075 | 0.166 | 0.496 | 0.081 | 0.498 | -0.017 | 0.266 | -0.100 | 0.058 | -0.124 | -0.169 | -0.060 | -0.214 | -0.075 | -0.157 | -0.005 | -0.287 | 0.017 |
| Roach | 1012 | 1 | 1 | 1 | -0.350 | 0.021 | -0.241 | 0.088 | 0.082 | 0.148 | 0.478 | 0.083 | 0.506 | -0.009 | 0.275 | -0.106 | 0.070 | -0.128 | -0.163 | -0.059 | -0.233 | -0.064 | -0.149 | 0.004 | -0.274 | 0.023 |
| Roach | 1013 | 1 | 1 | 1 | -0.354 | 0.010 | -0.226 | 0.091 | 0.092 | 0.168 | 0.493 | 0.079 | 0.495 | -0.025 | 0.262 | -0.090 | 0.063 | -0.117 | -0.159 | -0.061 | -0.227 | -0.069 | -0.159 | -0.001 | -0.281 | 0.015 |
| Roach | 1014 | 1 | 1 | 1 | -0.360 | 0.017 | -0.230 | 0.093 | 0.092 | 0.146 | 0.485 | 0.087 | 0.500 | -0.006 | 0.261 | -0.110 | 0.066 | -0.129 | -0.158 | -0.056 | -0.227 | -0.077 | -0.150 | 0.008 | -0.280 | 0.025 |
| Roach | 1015 | 1 | 1 | 1 | -0.361 | -0.006 | -0.231 | 0.098 | 0.077 | 0.175 | 0.500 | 0.065 | 0.498 | -0.032 | 0.262 | -0.084 | 0.053 | -0.103 | -0.145 | -0.051 | -0.222 | -0.082 | -0.150 | 0.010 | -0.280 | 0.009 |
| Roach | 1016 | 1 | 1 | 1 | -0.346 | 0.013 | -0.231 | 0.095 | 0.090 | 0.192 | 0.493 | 0.074 | 0.482 | -0.027 | 0.264 | -0.096 | 0.080 | -0.119 | -0.162 | -0.068 | -0.228 | -0.071 | -0.158 | 0.000 | -0.285 | 0.007 |
| Roach | 1017 | 1 | 1 | 1 | -0.351 | 0.008 | -0.232 | 0.098 | 0.049 | 0.173 | 0.500 | 0.061 | 0.491 | -0.028 | 0.280 | -0.086 | 0.070 | -0.117 | -0.160 | -0.054 | -0.216 | -0.068 | -0.146 | 0.001 | -0.285 | 0.011 |
| Roach | 1018 | 1 | 1 | 1 | -0.344 | 0.003 | -0.227 | 0.095 | 0.078 | 0.181 | 0.500 | 0.064 | 0.488 | -0.029 | 0.268 | -0.086 | 0.073 | -0.107 | -0.169 | -0.055 | -0.222 | -0.075 | -0.158 | -0.001 | -0.288 | 0.010 |
| Roach | 1019 | 1 | 1 | 1 | -0.347 | 0.013 | -0.235 | 0.094 | 0.101 | 0.177 | 0.493 | 0.074 | 0.481 | -0.030 | 0.274 | -0.082 | 0.071 | -0.119 | -0.168 | -0.055 | -0.215 | -0.074 | -0.163 | -0.004 | -0.291 | 0.007 |
| Roach | 1020 | 1 | 1 | 1 | -0.353 | 0.006 | -0.229 | 0.096 | 0.096 | 0.173 | 0.490 | 0.071 | 0.486 | -0.024 | 0.272 | -0.086 | 0.069 | -0.117 | -0.162 | -0.057 | -0.226 | -0.072 | -0.155 | -0.002 | -0.289 | 0.013 |
| Roach | 1021 | 1 | 1 | 1 | -0.346 | 0.002 | -0.239 | 0.088 | 0.095 | 0.165 | 0.498 | 0.075 | 0.489 | -0.022 | 0.264 | -0.093 | 0.068 | -0.114 | -0.160 | -0.049 | -0.226 | -0.071 | -0.158 | 0.004 | -0.285 | 0.015 |
| Roach | 1022 | 1 | 1 | 1 | -0.357 | 0.008 | -0.226 | 0.095 | 0.090 | 0.164 | 0.490 | 0.068 | 0.489 | -0.026 | 0.278 | -0.079 | 0.065 | -0.112 | -0.161 | -0.059 | -0.214 | -0.079 | -0.161 | 0.003 | -0.292 | 0.017 |
| Roach | 1023 | 1 | 1 | 1 | -0.356 | 0.018 | -0.227 | 0.096 | 0.077 | 0.153 | 0.494 | 0.078 | 0.494 | -0.014 | 0.277 | -0.096 | 0.054 | -0.118 | -0.166 | -0.060 | -0.214 | -0.073 | -0.152 | -0.006 | -0.284 | 0.023 |
| Roach | 1024 | 1 | 1 | 1 | -0.364 | 0.003 | -0.226 | 0.105 | 0.056 | 0.178 | 0.504 | 0.077 | 0.487 | -0.037 | 0.267 | -0.088 | 0.062 | -0.123 | -0.146 | -0.063 | -0.217 | -0.078 | -0.150 | 0.009 | -0.274 | 0.016 |
| Roach | 1025 | 1 | 1 | 1 | -0.344 | 0.017 | -0.225 | 0.098 | 0.083 | 0.176 | 0.496 | 0.075 | 0.486 | -0.020 | 0.266 | -0.095 | 0.077 | -0.123 | -0.165 | -0.058 | -0.225 | -0.075 | -0.159 | -0.003 | -0.290 | 0.009 |
| Roach | 1026 | 1 | 1 | 1 | -0.361 | -0.011 | -0.247 | 0.092 | 0.098 | 0.197 | 0.508 | 0.039 | 0.469 | -0.046 | 0.265 | -0.056 | 0.088 | -0.083 | -0.151 | -0.054 | -0.219 | -0.071 | -0.158 | -0.004 | -0.293 | -0.003 |
| Roach | 1027 | 1 | 1 | 1 | -0.353 | 0.040 | -0.230 | 0.095 | 0.095 | 0.140 | 0.469 | 0.084 | 0.503 | 0.004 | 0.281 | -0.104 | 0.069 | -0.140 | -0.170 | -0.065 | -0.217 | -0.069 | -0.155 | -0.011 | -0.291 | 0.027 |
| Roach | 1028 | 1 | 1 | 1 | -0.352 | 0.003 | -0.226 | 0.090 | 0.077 | 0.161 | 0.491 | 0.073 | 0.501 | -0.027 | 0.272 | -0.087 | 0.064 | -0.111 | -0.163 | -0.048 | -0.232 | -0.071 | -0.149 | 0.004 | -0.283 | 0.013 |
| Roach | 1029 | 1 | 1 | 1 | -0.343 | -0.001 | -0.232 | 0.091 | 0.075 | 0.179 | 0.514 | 0.052 | 0.474 | -0.036 | 0.275 | -0.071 | 0.079 | -0.097 | -0.165 | -0.057 | -0.213 | -0.074 | -0.173 | 0.009 | -0.291 | 0.003 |
| Roach | 1030 | 1 | 1 | 1 | -0.346 | 0.005 | -0.254 | 0.083 | 0.120 | 0.172 | 0.488 | 0.079 | 0.479 | -0.021 | 0.270 | -0.097 | 0.071 | -0.126 | -0.159 | -0.051 | -0.223 | -0.071 | -0.158 | 0.007 | -0.288 | 0.019 |
| Roach | 1001 | 1 | 1 | 2 | -0.343 | 0.014 | -0.213 | 0.084 | 0.072 | 0.167 | 0.492 | 0.080 | 0.501 | -0.013 | 0.279 | -0.101 | 0.044 | -0.131 | -0.164 | -0.055 | -0.229 | -0.071 | -0.157 | 0.006 | -0.282 | 0.019 |
| Roach | 1002 | 1 | 1 | 2 | -0.358 | 0.019 | -0.234 | 0.093 | 0.091 | 0.158 | 0.480 | 0.082 | 0.495 | -0.015 | 0.274 | -0.101 | 0.072 | -0.123 | -0.158 | -0.058 | -0.229 | -0.071 | -0.150 | -0.007 | -0.281 | 0.024 |
| Roach | 1003 | 1 | 1 | 2 | -0.349 | -0.009 | -0.247 | 0.079 | 0.070 | 0.190 | 0.522 | 0.045 | 0.481 | -0.034 | 0.261 | -0.071 | 0.065 | -0.098 | -0.148 | -0.051 | -0.225 | -0.070 | -0.153 | 0.014 | -0.276 | 0.005 |
| Roach | 1004 | 1 | 1 | 2 | -0.351 | 0.007 | -0.243 | 0.093 | 0.057 | 0.181 | 0.493 | 0.064 | 0.489 | -0.034 | 0.269 | -0.089 | 0.096 | -0.118 | -0.154 | -0.052 | -0.223 | -0.072 | -0.152 | 0.004 | -0.281 | 0.016 |
| Roach | 1005 | 1 | 1 | 2 | -0.339 | -0.004 | -0.230 | 0.072 | 0.051 | 0.170 | 0.500 | 0.067 | 0.492 | -0.035 | 0.280 | -0.091 | 0.084 | -0.115 | -0.162 | -0.036 | -0.222 | -0.073 | -0.165 | 0.025 | -0.289 | 0.020 |
| Roach | 1006 | 1 | 1 | 2 | -0.361 | 0.011 | -0.223 | 0.098 | 0.085 | 0.167 | 0.490 | 0.083 | 0.483 | -0.019 | 0.278 | -0.105 | 0.063 | -0.121 | -0.153 | -0.063 | -0.227 | -0.075 | -0.150 | 0.006 | -0.285 | 0.019 |
| Roach | 1007 | 1 | 1 | 2 | -0.348 | -0.009 | -0.258 | 0.080 | 0.043 | 0.207 | 0.524 | 0.046 | 0.470 | -0.051 | 0.278 | -0.079 | 0.073 | -0.091 | -0.140 | -0.051 | -0.215 | -0.073 | -0.147 | 0.018 | -0.278 | 0.003 |
| Roach | 1008 | 1 | 1 | 2 | -0.350 | 0.010 | -0.227 | 0.100 | 0.059 | 0.173 | 0.493 | 0.073 | 0.492 | -0.026 | 0.288 | -0.098 | 0.054 | -0.121 | -0.158 | -0.057 | -0.208 | -0.070 | -0.155 | -0.002 | -0.287 | 0.017 |
| Roach | 1009 | 1 | 1 | 2 | -0.354 | 0.021 | -0.235 | 0.089 | 0.071 | 0.157 | 0.490 | 0.078 | 0.488 | -0.025 | 0.285 | -0.092 | 0.075 | -0.119 | -0.154 | -0.055 | -0.232 | -0.071 | -0.155 | 0.002 | -0.278 | 0.016 |
| Roach | 1010 | 1 | 1 | 2 | -0.351 | 0.031 | -0.226 | 0.095 | 0.083 | 0.166 | 0.479 | 0.083 | 0.490 | -0.017 | 0.274 | -0.103 | 0.090 | -0.132 | -0.167 | -0.062 | -0.222 | -0.075 | -0.160 | -0.006 | -0.289 | 0.018 |
| Roach | 1011 | 1 | 1 | 2 | -0.343 | 0.025 | -0.244 | 0.089 | 0.064 | 0.173 | 0.499 | 0.075 | 0.491 | -0.028 | 0.267 | -0.096 | 0.079 | -0.116 | -0.162 | -0.061 | -0.211 | -0.072 | -0.158 | -0.004 | -0.284 | 0.017 |
| Roach | 1012 | 1 | 1 | 2 | -0.352 | 0.016 | -0.220 | 0.092 | 0.081 | 0.159 | 0.484 | 0.084 | 0.498 | -0.010 | 0.275 | -0.107 | 0.065 | -0.130 | -0.159 | -0.053 | -0.233 | -0.067 | -0.156 | -0.010 | -0.281 | 0.025 |
| Roach | 1013 | 1 | 1 | 2 | -0.351 | 0.013 | -0.228 | 0.089 | 0.060 | 0.173 | 0.502 | 0.073 | 0.488 | -0.027 | 0.272 | -0.092 | 0.073 | -0.117 | -0.152 | -0.062 | -0.239 | -0.067 | -0.150 | 0.000 | -0.276 | 0.016 |
| Roach | 1014 | 1 | 1 | 2 | -0.359 | 0.022 | -0.230 | 0.089 | 0.067 | 0.141 | 0.493 | 0.087 | 0.503 | -0.009 | 0.261 | -0.112 | 0.071 | -0.125 | -0.152 | -0.054 | -0.224 | -0.073 | -0.151 | 0.007 | -0.279 | 0.026 |
| Roach | 1015 | 1 | 1 | 2 | -0.359 | -0.004 | -0.229 | 0.101 | 0.075 | 0.173 | 0.504 | 0.066 | 0.496 | -0.028 | 0.260 | -0.086 | 0.055 | -0.104 | -0.146 | -0.054 | -0.223 | -0.078 | -0.154 | 0.005 | -0.279 | 0.012 |
| Roach | 1016 | 1 | 1 | 2 | -0.342 | 0.011 | -0.254 | 0.083 | 0.079 | 0.195 | 0.492 | 0.067 | 0.486 | -0.031 | 0.260 | -0.096 | 0.095 | -0.122 | -0.159 | -0.055 | -0.218 | -0.069 | -0.154 | 0.006 | -0.287 | 0.012 |
| Roach | 1017 | 1 | 1 | 2 | -0.353 | 0.006 | -0.243 | 0.090 | 0.042 | 0.180 | 0.498 | 0.061 | 0.492 | -0.033 | 0.281 | -0.089 | 0.074 | -0.117 | -0.156 | -0.048 | -0.213 | -0.069 | -0.140 | 0.003 | -0.281 | 0.017 |
| Roach | 1018 | 1 | 1 | 2 | -0.348 | 0.012 | -0.226 | 0.096 | 0.071 | 0.178 | 0.505 | 0.067 | 0.486 | -0.027 | 0.265 | -0.085 | 0.076 | -0.115 | -0.166 | -0.059 | -0.219 | -0.073 | -0.157 | -0.003 | -0.287 | 0.009 |
| Roach | 1019 | 1 | 1 | 2 | -0.349 | 0.016 | -0.238 | 0.079 | 0.097 | 0.178 | 0.501 | 0.073 | 0.471 | -0.031 | 0.276 | -0.084 | 0.077 | -0.112 | -0.162 | -0.059 | -0.218 | -0.068 | -0.161 | -0.004 | -0.294 | 0.013 |
| Roach | 1020 | 1 | 1 | 2 | -0.353 | 0.012 | -0.231 | 0.091 | 0.081 | 0.169 | 0.492 | 0.071 | 0.489 | -0.023 | 0.270 | -0.089 | 0.078 | -0.120 | -0.161 | -0.051 | -0.226 | -0.070 | -0.152 | -0.004 | -0.287 | 0.016 |
| Roach | 1021 | 1 | 1 | 2 | -0.348 | 0.013 | -0.239 | 0.084 | 0.087 | 0.174 | 0.499 | 0.074 | 0.487 | -0.027 | 0.264 | -0.088 | 0.071 | -0.116 | -0.155 | -0.056 | -0.225 | -0.070 | -0.153 | -0.003 | -0.288 | 0.015 |
| Roach | 1022 | 1 | 1 | 2 | -0.358 | 0.010 | -0.222 | 0.098 | 0.087 | 0.167 | 0.485 | 0.067 | 0.483 | -0.026 | 0.282 | -0.083 | 0.079 | -0.114 | -0.156 | -0.054 | -0.226 | -0.084 | -0.159 | 0.004 | -0.296 | 0.014 |
| Roach | 1023 | 1 | 1 | 2 | -0.346 | 0.022 | -0.237 | 0.092 | 0.081 | 0.149 | 0.496 | 0.077 | 0.497 | -0.011 | 0.275 | -0.096 | 0.055 | -0.116 | -0.165 | -0.057 | -0.222 | -0.075 | -0.155 | -0.004 | -0.279 | 0.019 |
| Roach | 1024 | 1 | 1 | 2 | -0.365 | 0.003 | -0.233 | 0.103 | 0.067 | 0.183 | 0.505 | 0.076 | 0.484 | -0.038 | 0.261 | -0.089 | 0.062 | -0.115 | -0.147 | -0.056 | -0.217 | -0.081 | -0.140 | 0.000 | -0.278 | 0.015 |
| Roach | 1025 | 1 | 1 | 2 | -0.344 | 0.013 | -0.237 | 0.097 | 0.084 | 0.177 | 0.496 | 0.077 | 0.478 | -0.024 | 0.276 | -0.098 | 0.076 | -0.126 | -0.158 | -0.052 | -0.219 | -0.074 | -0.167 | 0.000 | -0.286 | 0.011 |
| Roach | 1026 | 1 | 1 | 2 | -0.361 | -0.012 | -0.241 | 0.093 | 0.102 | 0.197 | 0.505 | 0.048 | 0.473 | -0.053 | 0.259 | -0.053 | 0.088 | -0.087 | -0.150 | -0.052 | -0.224 | -0.074 | -0.161 | -0.005 | -0.290 | -0.002 |
| Roach | 1027 | 1 | 1 | 2 | -0.352 | 0.042 | -0.235 | 0.096 | 0.091 | 0.143 | 0.473 | 0.090 | 0.497 | -0.001 | 0.281 | -0.106 | 0.071 | -0.141 | -0.168 | -0.071 | -0.218 | -0.068 | -0.153 | -0.011 | -0.286 | 0.028 |
| Roach | 1028 | 1 | 1 | 2 | -0.352 | 0.001 | -0.226 | 0.089 | 0.078 | 0.158 | 0.493 | 0.069 | 0.498 | -0.022 | 0.274 | -0.088 | 0.068 | -0.109 | -0.167 | -0.046 | -0.233 | -0.064 | -0.149 | 0.001 | -0.283 | 0.012 |
| Roach | 1029 | 1 | 1 | 2 | -0.344 | -0.003 | -0.245 | 0.086 | 0.071 | 0.178 | 0.510 | 0.049 | 0.472 | -0.038 | 0.276 | -0.072 | 0.102 | -0.092 | -0.165 | -0.050 | -0.216 | -0.067 | -0.169 | 0.000 | -0.292 | 0.011 |
| Roach | 1030 | 1 | 1 | 2 | -0.344 | 0.006 | -0.221 | 0.098 | 0.051 | 0.171 | 0.496 | 0.074 | 0.482 | -0.019 | 0.285 | -0.102 | 0.078 | -0.139 | -0.153 | -0.054 | -0.226 | -0.071 | -0.157 | 0.015 | -0.292 | 0.022 |
| Roach | 1001 | 1 | 1 | 3 | -0.346 | 0.017 | -0.221 | 0.080 | 0.070 | 0.162 | 0.493 | 0.082 | 0.504 | -0.015 | 0.269 | -0.103 | 0.055 | -0.128 | -0.158 | -0.049 | -0.230 | -0.070 | -0.154 | 0.005 | -0.283 | 0.021 |
| Roach | 1002 | 1 | 1 | 3 | -0.355 | 0.024 | -0.247 | 0.096 | 0.080 | 0.164 | 0.487 | 0.081 | 0.487 | -0.019 | 0.283 | -0.103 | 0.063 | -0.120 | -0.151 | -0.059 | -0.230 | -0.069 | -0.139 | -0.011 | -0.278 | 0.018 |
| Roach | 1003 | 1 | 1 | 3 | -0.353 | -0.007 | -0.250 | 0.085 | 0.067 | 0.199 | 0.519 | 0.048 | 0.476 | -0.039 | 0.266 | -0.073 | 0.067 | -0.099 | -0.147 | -0.048 | -0.223 | -0.074 | -0.148 | 0.002 | -0.273 | 0.006 |
| Roach | 1004 | 1 | 1 | 3 | -0.351 | 0.013 | -0.241 | 0.085 | 0.081 | 0.183 | 0.489 | 0.073 | 0.481 | -0.035 | 0.277 | -0.088 | 0.084 | -0.117 | -0.155 | -0.061 | -0.228 | -0.069 | -0.154 | 0.001 | -0.285 | 0.015 |
| Roach | 1005 | 1 | 1 | 3 | -0.338 | -0.001 | -0.237 | 0.080 | 0.067 | 0.172 | 0.484 | 0.071 | 0.493 | -0.037 | 0.291 | -0.092 | 0.082 | -0.117 | -0.162 | -0.037 | -0.225 | -0.072 | -0.165 | 0.014 | -0.290 | 0.017 |
| Roach | 1006 | 1 | 1 | 3 | -0.356 | 0.017 | -0.254 | 0.082 | 0.085 | 0.165 | 0.491 | 0.079 | 0.481 | -0.020 | 0.283 | -0.104 | 0.067 | -0.122 | -0.150 | -0.054 | -0.213 | -0.070 | -0.152 | 0.005 | -0.283 | 0.022 |
| Roach | 1007 | 1 | 1 | 3 | -0.358 | -0.022 | -0.238 | 0.090 | 0.061 | 0.209 | 0.521 | 0.048 | 0.468 | -0.052 | 0.271 | -0.076 | 0.075 | -0.089 | -0.152 | -0.046 | -0.213 | -0.073 | -0.150 | 0.010 | -0.284 | 0.000 |
| Roach | 1008 | 1 | 1 | 3 | -0.344 | 0.017 | -0.254 | 0.086 | 0.064 | 0.175 | 0.498 | 0.078 | 0.490 | -0.033 | 0.279 | -0.094 | 0.063 | -0.124 | -0.155 | -0.054 | -0.209 | -0.067 | -0.154 | 0.000 | -0.278 | 0.016 |
| Roach | 1009 | 1 | 1 | 3 | -0.353 | 0.022 | -0.230 | 0.095 | 0.071 | 0.166 | 0.484 | 0.076 | 0.491 | -0.024 | 0.283 | -0.096 | 0.077 | -0.117 | -0.160 | -0.061 | -0.229 | -0.073 | -0.151 | -0.005 | -0.283 | 0.017 |
| Roach | 1010 | 1 | 1 | 3 | -0.355 | 0.033 | -0.223 | 0.099 | 0.086 | 0.164 | 0.480 | 0.085 | 0.485 | -0.017 | 0.276 | -0.101 | 0.090 | -0.132 | -0.166 | -0.065 | -0.219 | -0.077 | -0.162 | -0.009 | -0.292 | 0.021 |
| Roach | 1011 | 1 | 1 | 3 | -0.339 | 0.026 | -0.250 | 0.083 | 0.079 | 0.165 | 0.495 | 0.071 | 0.488 | -0.020 | 0.267 | -0.095 | 0.095 | -0.117 | -0.169 | -0.058 | -0.223 | -0.070 | -0.159 | -0.003 | -0.284 | 0.018 |
| Roach | 1012 | 1 | 1 | 3 | -0.353 | 0.024 | -0.219 | 0.089 | 0.076 | 0.157 | 0.475 | 0.083 | 0.501 | -0.012 | 0.278 | -0.106 | 0.081 | -0.131 | -0.163 | -0.061 | -0.236 | -0.069 | -0.159 | 0.004 | -0.281 | 0.022 |
| Roach | 1013 | 1 | 1 | 3 | -0.356 | 0.020 | -0.229 | 0.096 | 0.085 | 0.170 | 0.494 | 0.078 | 0.490 | -0.023 | 0.265 | -0.092 | 0.068 | -0.117 | -0.160 | -0.061 | -0.224 | -0.075 | -0.152 | -0.013 | -0.282 | 0.016 |
| Roach | 1014 | 1 | 1 | 3 | -0.362 | 0.022 | -0.230 | 0.095 | 0.076 | 0.148 | 0.490 | 0.086 | 0.497 | -0.010 | 0.263 | -0.108 | 0.074 | -0.129 | -0.160 | -0.053 | -0.214 | -0.079 | -0.151 | 0.000 | -0.282 | 0.029 |
| Roach | 1015 | 1 | 1 | 3 | -0.360 | 0.002 | -0.248 | 0.086 | 0.060 | 0.174 | 0.508 | 0.063 | 0.493 | -0.037 | 0.265 | -0.083 | 0.063 | -0.099 | -0.143 | -0.053 | -0.218 | -0.077 | -0.145 | 0.009 | -0.275 | 0.015 |
| Roach | 1016 | 1 | 1 | 3 | -0.352 | 0.012 | -0.228 | 0.092 | 0.089 | 0.192 | 0.485 | 0.072 | 0.482 | -0.026 | 0.263 | -0.096 | 0.099 | -0.127 | -0.158 | -0.062 | -0.227 | -0.069 | -0.164 | 0.001 | -0.289 | 0.011 |
| Roach | 1017 | 1 | 1 | 3 | -0.345 | 0.009 | -0.239 | 0.095 | 0.046 | 0.185 | 0.503 | 0.061 | 0.493 | -0.033 | 0.267 | -0.087 | 0.081 | -0.115 | -0.156 | -0.055 | -0.214 | -0.068 | -0.155 | -0.012 | -0.281 | 0.018 |
| Roach | 1018 | 1 | 1 | 3 | -0.349 | 0.006 | -0.227 | 0.094 | 0.074 | 0.180 | 0.498 | 0.067 | 0.489 | -0.035 | 0.267 | -0.085 | 0.081 | -0.106 | -0.163 | -0.057 | -0.214 | -0.075 | -0.168 | 0.003 | -0.288 | 0.009 |
| Roach | 1019 | 1 | 1 | 3 | -0.347 | 0.012 | -0.239 | 0.088 | 0.100 | 0.172 | 0.499 | 0.076 | 0.478 | -0.030 | 0.271 | -0.086 | 0.074 | -0.115 | -0.166 | -0.055 | -0.221 | -0.067 | -0.162 | -0.003 | -0.287 | 0.007 |
| Roach | 1020 | 1 | 1 | 3 | -0.355 | 0.012 | -0.240 | 0.088 | 0.084 | 0.169 | 0.491 | 0.072 | 0.484 | -0.030 | 0.273 | -0.088 | 0.085 | -0.111 | -0.158 | -0.056 | -0.220 | -0.073 | -0.153 | -0.001 | -0.291 | 0.019 |
| Roach | 1021 | 1 | 1 | 3 | -0.346 | 0.007 | -0.234 | 0.088 | 0.084 | 0.164 | 0.497 | 0.076 | 0.489 | -0.028 | 0.267 | -0.089 | 0.074 | -0.116 | -0.156 | -0.059 | -0.224 | -0.071 | -0.163 | 0.011 | -0.288 | 0.017 |
| Roach | 1022 | 1 | 1 | 3 | -0.359 | 0.016 | -0.226 | 0.092 | 0.085 | 0.164 | 0.489 | 0.070 | 0.485 | -0.029 | 0.283 | -0.082 | 0.076 | -0.110 | -0.162 | -0.055 | -0.215 | -0.079 | -0.165 | -0.004 | -0.290 | 0.015 |
| Roach | 1023 | 1 | 1 | 3 | -0.354 | 0.018 | -0.218 | 0.095 | 0.070 | 0.151 | 0.490 | 0.077 | 0.501 | -0.017 | 0.279 | -0.092 | 0.056 | -0.119 | -0.159 | -0.059 | -0.223 | -0.072 | -0.156 | -0.004 | -0.286 | 0.021 |
| Roach | 1024 | 1 | 1 | 3 | -0.362 | 0.008 | -0.233 | 0.097 | 0.066 | 0.186 | 0.503 | 0.080 | 0.488 | -0.039 | 0.255 | -0.085 | 0.066 | -0.127 | -0.135 | -0.060 | -0.225 | -0.075 | -0.144 | -0.006 | -0.278 | 0.021 |
| Roach | 1025 | 1 | 1 | 3 | -0.347 | 0.020 | -0.239 | 0.087 | 0.077 | 0.177 | 0.495 | 0.075 | 0.485 | -0.029 | 0.270 | -0.092 | 0.080 | -0.123 | -0.158 | -0.061 | -0.215 | -0.073 | -0.165 | 0.005 | -0.284 | 0.014 |
| Roach | 1026 | 1 | 1 | 3 | -0.362 | -0.007 | -0.253 | 0.081 | 0.093 | 0.195 | 0.499 | 0.047 | 0.472 | -0.053 | 0.271 | -0.059 | 0.095 | -0.089 | -0.148 | -0.053 | -0.215 | -0.068 | -0.158 | 0.004 | -0.293 | 0.002 |
| Roach | 1027 | 1 | 1 | 3 | -0.352 | 0.040 | -0.232 | 0.096 | 0.096 | 0.141 | 0.468 | 0.087 | 0.499 | 0.003 | 0.281 | -0.107 | 0.075 | -0.142 | -0.164 | -0.069 | -0.217 | -0.068 | -0.159 | -0.009 | -0.294 | 0.029 |
| Roach | 1028 | 1 | 1 | 3 | -0.352 | 0.006 | -0.229 | 0.082 | 0.077 | 0.167 | 0.490 | 0.069 | 0.497 | -0.027 | 0.273 | -0.089 | 0.072 | -0.108 | -0.162 | -0.052 | -0.225 | -0.070 | -0.154 | 0.001 | -0.287 | 0.019 |
| Roach | 1029 | 1 | 1 | 3 | -0.343 | -0.003 | -0.246 | 0.085 | 0.075 | 0.181 | 0.510 | 0.048 | 0.478 | -0.038 | 0.269 | -0.069 | 0.091 | -0.094 | -0.164 | -0.054 | -0.215 | -0.070 | -0.164 | 0.003 | -0.291 | 0.010 |
| Roach | 1030 | 1 | 1 | 3 | -0.342 | 0.009 | -0.243 | 0.092 | 0.060 | 0.171 | 0.486 | 0.079 | 0.488 | -0.025 | 0.287 | -0.105 | 0.079 | -0.136 | -0.151 | -0.051 | -0.223 | -0.068 | -0.155 | 0.011 | -0.286 | 0.024 |
| Roach | 1101 | 1 | 2 | 1 | -0.346 | 0.006 | -0.240 | 0.074 | 0.072 | 0.173 | 0.509 | 0.057 | 0.487 | -0.038 | 0.270 | -0.074 | 0.077 | -0.092 | -0.154 | -0.046 | -0.211 | -0.076 | -0.170 | 0.004 | -0.293 | 0.012 |
| Roach | 1102 | 1 | 2 | 1 | -0.365 | 0.018 | -0.224 | 0.087 | 0.075 | 0.139 | 0.490 | 0.073 | 0.505 | -0.015 | 0.267 | -0.083 | 0.066 | -0.118 | -0.152 | -0.059 | -0.226 | -0.069 | -0.149 | 0.004 | -0.287 | 0.023 |
| Roach | 1103 | 1 | 2 | 1 | -0.357 | 0.021 | -0.227 | 0.087 | 0.079 | 0.147 | 0.483 | 0.075 | 0.492 | -0.026 | 0.286 | -0.083 | 0.081 | -0.113 | -0.159 | -0.057 | -0.218 | -0.074 | -0.164 | -0.002 | -0.296 | 0.026 |
| Roach | 1104 | 1 | 2 | 1 | -0.359 | 0.014 | -0.222 | 0.093 | 0.068 | 0.161 | 0.498 | 0.066 | 0.491 | -0.024 | 0.274 | -0.079 | 0.069 | -0.114 | -0.159 | -0.054 | -0.216 | -0.081 | -0.154 | -0.002 | -0.291 | 0.021 |
| Roach | 1105 | 1 | 2 | 1 | -0.368 | 0.015 | -0.221 | 0.090 | 0.078 | 0.160 | 0.489 | 0.071 | 0.495 | -0.028 | 0.268 | -0.081 | 0.078 | -0.105 | -0.153 | -0.063 | -0.232 | -0.079 | -0.150 | -0.004 | -0.283 | 0.024 |
| Roach | 1106 | 1 | 2 | 1 | -0.357 | 0.024 | -0.215 | 0.090 | 0.081 | 0.156 | 0.477 | 0.075 | 0.515 | -0.009 | 0.266 | -0.097 | 0.061 | -0.119 | -0.155 | -0.062 | -0.225 | -0.071 | -0.160 | -0.014 | -0.288 | 0.027 |
| Roach | 1107 | 1 | 2 | 1 | -0.363 | 0.035 | -0.204 | 0.092 | 0.076 | 0.134 | 0.486 | 0.087 | 0.507 | -0.007 | 0.271 | -0.094 | 0.058 | -0.124 | -0.159 | -0.068 | -0.229 | -0.077 | -0.158 | -0.005 | -0.285 | 0.028 |
| Roach | 1108 | 1 | 2 | 1 | -0.359 | 0.016 | -0.233 | 0.085 | 0.090 | 0.153 | 0.488 | 0.068 | 0.493 | -0.023 | 0.274 | -0.077 | 0.075 | -0.114 | -0.157 | -0.056 | -0.222 | -0.070 | -0.158 | -0.001 | -0.290 | 0.019 |
| Roach | 1109 | 1 | 2 | 1 | -0.364 | 0.005 | -0.248 | 0.095 | 0.081 | 0.170 | 0.493 | 0.070 | 0.493 | -0.026 | 0.264 | -0.089 | 0.065 | -0.120 | -0.144 | -0.056 | -0.220 | -0.074 | -0.144 | 0.008 | -0.276 | 0.016 |
| Roach | 1110 | 1 | 2 | 1 | -0.358 | 0.026 | -0.235 | 0.088 | 0.068 | 0.145 | 0.492 | 0.074 | 0.502 | -0.019 | 0.272 | -0.085 | 0.070 | -0.119 | -0.151 | -0.060 | -0.223 | -0.062 | -0.155 | -0.007 | -0.282 | 0.018 |
| Roach | 1111 | 1 | 2 | 1 | -0.360 | 0.010 | -0.219 | 0.096 | 0.069 | 0.155 | 0.495 | 0.076 | 0.506 | -0.024 | 0.261 | -0.084 | 0.060 | -0.116 | -0.152 | -0.062 | -0.223 | -0.067 | -0.155 | -0.005 | -0.283 | 0.021 |
| Roach | 1112 | 1 | 2 | 1 | -0.367 | 0.022 | -0.232 | 0.088 | 0.076 | 0.148 | 0.492 | 0.070 | 0.487 | -0.022 | 0.281 | -0.082 | 0.077 | -0.112 | -0.148 | -0.059 | -0.226 | -0.072 | -0.153 | 0.001 | -0.287 | 0.019 |
| Roach | 1113 | 1 | 2 | 1 | -0.372 | 0.018 | -0.216 | 0.099 | 0.077 | 0.157 | 0.496 | 0.078 | 0.488 | -0.026 | 0.266 | -0.079 | 0.069 | -0.123 | -0.154 | -0.066 | -0.223 | -0.078 | -0.148 | 0.004 | -0.283 | 0.017 |
| Roach | 1114 | 1 | 2 | 1 | -0.363 | 0.001 | -0.230 | 0.083 | 0.077 | 0.178 | 0.502 | 0.063 | 0.489 | -0.038 | 0.267 | -0.073 | 0.060 | -0.104 | -0.144 | -0.056 | -0.225 | -0.079 | -0.149 | 0.004 | -0.285 | 0.018 |
| Roach | 1115 | 1 | 2 | 1 | -0.360 | 0.001 | -0.233 | 0.093 | 0.076 | 0.178 | 0.500 | 0.068 | 0.494 | -0.034 | 0.260 | -0.084 | 0.064 | -0.106 | -0.150 | -0.057 | -0.215 | -0.069 | -0.152 | -0.002 | -0.285 | 0.012 |
| Roach | 1116 | 1 | 2 | 1 | -0.351 | 0.026 | -0.222 | 0.081 | 0.087 | 0.143 | 0.491 | 0.081 | 0.496 | -0.008 | 0.276 | -0.096 | 0.060 | -0.123 | -0.154 | -0.056 | -0.227 | -0.075 | -0.166 | 0.001 | -0.290 | 0.027 |
| Roach | 1117 | 1 | 2 | 1 | -0.346 | 0.022 | -0.221 | 0.083 | 0.067 | 0.155 | 0.495 | 0.076 | 0.486 | -0.029 | 0.286 | -0.079 | 0.082 | -0.119 | -0.166 | -0.056 | -0.228 | -0.072 | -0.164 | -0.006 | -0.291 | 0.026 |
| Roach | 1118 | 1 | 2 | 1 | -0.360 | 0.016 | -0.230 | 0.087 | 0.077 | 0.154 | 0.491 | 0.071 | 0.495 | -0.024 | 0.272 | -0.083 | 0.075 | -0.112 | -0.153 | -0.057 | -0.223 | -0.073 | -0.156 | 0.000 | -0.288 | 0.021 |
| Roach | 1119 | 1 | 2 | 1 | -0.358 | 0.040 | -0.242 | 0.084 | 0.073 | 0.143 | 0.490 | 0.082 | 0.501 | -0.019 | 0.267 | -0.085 | 0.076 | -0.129 | -0.156 | -0.069 | -0.212 | -0.068 | -0.158 | -0.003 | -0.281 | 0.024 |
| Roach | 1120 | 1 | 2 | 1 | -0.359 | 0.011 | -0.249 | 0.073 | 0.061 | 0.137 | 0.494 | 0.065 | 0.511 | -0.027 | 0.278 | -0.078 | 0.060 | -0.100 | -0.150 | -0.043 | -0.222 | -0.069 | -0.143 | 0.009 | -0.279 | 0.021 |
| Roach | 1121 | 1 | 2 | 1 | -0.372 | -0.002 | -0.240 | 0.081 | 0.095 | 0.162 | 0.491 | 0.057 | 0.489 | -0.036 | 0.263 | -0.077 | 0.093 | -0.086 | -0.149 | -0.047 | -0.213 | -0.073 | -0.162 | 0.008 | -0.296 | 0.012 |
| Roach | 1122 | 1 | 2 | 1 | -0.351 | 0.022 | -0.230 | 0.085 | 0.092 | 0.135 | 0.480 | 0.082 | 0.496 | -0.002 | 0.292 | -0.102 | 0.055 | -0.126 | -0.155 | -0.054 | -0.225 | -0.069 | -0.165 | -0.001 | -0.290 | 0.032 |
| Roach | 1123 | 1 | 2 | 1 | -0.352 | 0.021 | -0.222 | 0.088 | 0.091 | 0.128 | 0.480 | 0.084 | 0.498 | -0.002 | 0.274 | -0.103 | 0.085 | -0.128 | -0.171 | -0.048 | -0.224 | -0.072 | -0.164 | 0.003 | -0.296 | 0.030 |
| Roach | 1124 | 1 | 2 | 1 | -0.355 | -0.008 | -0.242 | 0.077 | 0.088 | 0.164 | 0.486 | 0.057 | 0.483 | -0.038 | 0.290 | -0.074 | 0.089 | -0.097 | -0.158 | -0.039 | -0.223 | -0.071 | -0.156 | 0.009 | -0.302 | 0.021 |
| Roach | 1125 | 1 | 2 | 1 | -0.360 | 0.032 | -0.244 | 0.079 | 0.080 | 0.154 | 0.496 | 0.071 | 0.490 | -0.027 | 0.258 | -0.077 | 0.101 | -0.112 | -0.147 | -0.050 | -0.228 | -0.079 | -0.159 | -0.018 | -0.286 | 0.027 |
| Roach | 1126 | 1 | 2 | 1 | -0.374 | 0.027 | -0.226 | 0.092 | 0.066 | 0.135 | 0.497 | 0.075 | 0.494 | -0.017 | 0.260 | -0.091 | 0.095 | -0.104 | -0.148 | -0.068 | -0.234 | -0.072 | -0.147 | -0.004 | -0.283 | 0.026 |
| Roach | 1127 | 1 | 2 | 1 | -0.357 | 0.016 | -0.223 | 0.088 | 0.082 | 0.143 | 0.475 | 0.077 | 0.500 | -0.015 | 0.281 | -0.094 | 0.083 | -0.128 | -0.158 | -0.052 | -0.218 | -0.071 | -0.170 | 0.009 | -0.295 | 0.026 |
| Roach | 1128 | 1 | 2 | 1 | -0.375 | 0.012 | -0.232 | 0.083 | 0.069 | 0.178 | 0.488 | 0.056 | 0.482 | -0.041 | 0.273 | -0.067 | 0.109 | -0.106 | -0.151 | -0.061 | -0.233 | -0.074 | -0.146 | 0.001 | -0.283 | 0.019 |
| Roach | 1129 | 1 | 2 | 1 | -0.365 | -0.005 | -0.246 | 0.089 | 0.075 | 0.189 | 0.500 | 0.043 | 0.487 | -0.054 | 0.262 | -0.058 | 0.092 | -0.075 | -0.143 | -0.060 | -0.209 | -0.075 | -0.155 | 0.000 | -0.297 | 0.007 |
| Roach | 1130 | 1 | 2 | 1 | -0.360 | -0.018 | -0.243 | 0.090 | 0.073 | 0.190 | 0.508 | 0.025 | 0.465 | -0.047 | 0.277 | -0.052 | 0.115 | -0.077 | -0.146 | -0.040 | -0.213 | -0.074 | -0.170 | -0.002 | -0.307 | 0.006 |
| Roach | 1101 | 1 | 2 | 2 | -0.351 | 0.003 | -0.243 | 0.078 | 0.077 | 0.178 | 0.504 | 0.056 | 0.488 | -0.036 | 0.268 | -0.076 | 0.073 | -0.094 | -0.153 | -0.049 | -0.210 | -0.074 | -0.160 | 0.002 | -0.294 | 0.012 |
| Roach | 1102 | 1 | 2 | 2 | -0.366 | 0.015 | -0.220 | 0.093 | 0.078 | 0.140 | 0.489 | 0.076 | 0.500 | -0.019 | 0.265 | -0.082 | 0.075 | -0.122 | -0.155 | -0.055 | -0.229 | -0.072 | -0.150 | 0.004 | -0.289 | 0.021 |
| Roach | 1103 | 1 | 2 | 2 | -0.355 | 0.020 | -0.227 | 0.088 | 0.080 | 0.143 | 0.490 | 0.076 | 0.488 | -0.024 | 0.285 | -0.084 | 0.077 | -0.113 | -0.160 | -0.053 | -0.218 | -0.074 | -0.165 | 0.001 | -0.296 | 0.021 |
| Roach | 1104 | 1 | 2 | 2 | -0.365 | 0.014 | -0.217 | 0.098 | 0.075 | 0.160 | 0.490 | 0.070 | 0.491 | -0.023 | 0.277 | -0.086 | 0.068 | -0.112 | -0.152 | -0.059 | -0.217 | -0.081 | -0.157 | 0.002 | -0.293 | 0.018 |
| Roach | 1105 | 1 | 2 | 2 | -0.365 | 0.014 | -0.225 | 0.093 | 0.076 | 0.163 | 0.484 | 0.072 | 0.499 | -0.033 | 0.265 | -0.081 | 0.084 | -0.103 | -0.152 | -0.066 | -0.231 | -0.078 | -0.152 | -0.005 | -0.284 | 0.024 |
| Roach | 1106 | 1 | 2 | 2 | -0.355 | 0.023 | -0.224 | 0.089 | 0.082 | 0.157 | 0.484 | 0.080 | 0.504 | -0.013 | 0.265 | -0.099 | 0.066 | -0.125 | -0.147 | -0.065 | -0.224 | -0.071 | -0.161 | 0.000 | -0.289 | 0.024 |
| Roach | 1107 | 1 | 2 | 2 | -0.362 | 0.036 | -0.213 | 0.091 | 0.080 | 0.131 | 0.488 | 0.087 | 0.498 | -0.008 | 0.273 | -0.093 | 0.072 | -0.126 | -0.155 | -0.069 | -0.231 | -0.068 | -0.163 | -0.008 | -0.286 | 0.028 |
| Roach | 1108 | 1 | 2 | 2 | -0.355 | 0.020 | -0.241 | 0.080 | 0.077 | 0.151 | 0.489 | 0.067 | 0.496 | -0.025 | 0.282 | -0.079 | 0.072 | -0.114 | -0.159 | -0.049 | -0.215 | -0.072 | -0.157 | 0.002 | -0.290 | 0.018 |
| Roach | 1109 | 1 | 2 | 2 | -0.366 | 0.005 | -0.242 | 0.097 | 0.075 | 0.172 | 0.489 | 0.074 | 0.497 | -0.035 | 0.271 | -0.084 | 0.056 | -0.119 | -0.143 | -0.058 | -0.215 | -0.077 | -0.145 | 0.012 | -0.277 | 0.014 |
| Roach | 1110 | 1 | 2 | 2 | -0.355 | 0.024 | -0.244 | 0.082 | 0.075 | 0.143 | 0.495 | 0.074 | 0.500 | -0.021 | 0.269 | -0.084 | 0.074 | -0.116 | -0.150 | -0.060 | -0.223 | -0.059 | -0.158 | 0.000 | -0.282 | 0.017 |
| Roach | 1111 | 1 | 2 | 2 | -0.358 | 0.014 | -0.217 | 0.093 | 0.067 | 0.153 | 0.498 | 0.078 | 0.501 | -0.022 | 0.269 | -0.087 | 0.057 | -0.118 | -0.151 | -0.060 | -0.229 | -0.068 | -0.154 | -0.003 | -0.282 | 0.019 |
| Roach | 1112 | 1 | 2 | 2 | -0.361 | 0.015 | -0.240 | 0.084 | 0.075 | 0.147 | 0.493 | 0.069 | 0.490 | -0.025 | 0.285 | -0.080 | 0.074 | -0.110 | -0.152 | -0.051 | -0.230 | -0.070 | -0.155 | 0.000 | -0.278 | 0.021 |
| Roach | 1113 | 1 | 2 | 2 | -0.369 | 0.018 | -0.221 | 0.090 | 0.078 | 0.162 | 0.491 | 0.074 | 0.496 | -0.026 | 0.268 | -0.079 | 0.064 | -0.119 | -0.153 | -0.066 | -0.224 | -0.075 | -0.150 | 0.002 | -0.280 | 0.019 |
| Roach | 1114 | 1 | 2 | 2 | -0.364 | 0.002 | -0.233 | 0.081 | 0.071 | 0.180 | 0.501 | 0.063 | 0.482 | -0.038 | 0.274 | -0.076 | 0.069 | -0.109 | -0.144 | -0.060 | -0.220 | -0.071 | -0.149 | 0.008 | -0.289 | 0.019 |
| Roach | 1115 | 1 | 2 | 2 | -0.360 | 0.001 | -0.232 | 0.090 | 0.073 | 0.179 | 0.508 | 0.067 | 0.489 | -0.036 | 0.261 | -0.084 | 0.061 | -0.103 | -0.155 | -0.059 | -0.203 | -0.075 | -0.157 | 0.005 | -0.285 | 0.015 |
| Roach | 1116 | 1 | 2 | 2 | -0.351 | 0.028 | -0.226 | 0.084 | 0.063 | 0.140 | 0.491 | 0.080 | 0.499 | -0.013 | 0.283 | -0.095 | 0.065 | -0.125 | -0.153 | -0.059 | -0.223 | -0.071 | -0.159 | 0.002 | -0.289 | 0.030 |
| Roach | 1117 | 1 | 2 | 2 | -0.348 | 0.019 | -0.230 | 0.078 | 0.079 | 0.160 | 0.484 | 0.074 | 0.485 | -0.029 | 0.295 | -0.082 | 0.080 | -0.119 | -0.160 | -0.059 | -0.225 | -0.073 | -0.169 | -0.001 | -0.291 | 0.031 |
| Roach | 1118 | 1 | 2 | 2 | -0.349 | -0.005 | -0.244 | 0.079 | 0.097 | 0.159 | 0.490 | 0.068 | 0.491 | -0.036 | 0.281 | -0.074 | 0.066 | -0.106 | -0.147 | -0.044 | -0.214 | -0.070 | -0.172 | 0.011 | -0.298 | 0.019 |
| Roach | 1119 | 1 | 2 | 2 | -0.350 | 0.042 | -0.253 | 0.084 | 0.068 | 0.141 | 0.491 | 0.077 | 0.499 | -0.013 | 0.275 | -0.088 | 0.073 | -0.128 | -0.158 | -0.071 | -0.212 | -0.068 | -0.155 | 0.000 | -0.280 | 0.025 |
| Roach | 1120 | 1 | 2 | 2 | -0.366 | 0.014 | -0.226 | 0.086 | 0.056 | 0.145 | 0.496 | 0.068 | 0.495 | -0.027 | 0.286 | -0.081 | 0.068 | -0.107 | -0.150 | -0.048 | -0.218 | -0.074 | -0.150 | 0.004 | -0.290 | 0.021 |
| Roach | 1121 | 1 | 2 | 2 | -0.368 | -0.009 | -0.241 | 0.084 | 0.081 | 0.164 | 0.492 | 0.059 | 0.493 | -0.042 | 0.262 | -0.075 | 0.091 | -0.093 | -0.145 | -0.043 | -0.208 | -0.074 | -0.159 | 0.014 | -0.299 | 0.015 |
| Roach | 1122 | 1 | 2 | 2 | -0.355 | 0.024 | -0.204 | 0.087 | 0.080 | 0.133 | 0.478 | 0.085 | 0.507 | -0.005 | 0.285 | -0.102 | 0.058 | -0.124 | -0.157 | -0.052 | -0.231 | -0.077 | -0.170 | 0.000 | -0.291 | 0.031 |
| Roach | 1123 | 1 | 2 | 2 | -0.352 | 0.019 | -0.212 | 0.088 | 0.087 | 0.127 | 0.481 | 0.087 | 0.498 | -0.005 | 0.278 | -0.101 | 0.079 | -0.133 | -0.157 | -0.042 | -0.233 | -0.075 | -0.169 | 0.005 | -0.299 | 0.030 |
| Roach | 1124 | 1 | 2 | 2 | -0.360 | -0.005 | -0.230 | 0.087 | 0.085 | 0.165 | 0.488 | 0.058 | 0.483 | -0.036 | 0.285 | -0.072 | 0.087 | -0.102 | -0.155 | -0.043 | -0.221 | -0.076 | -0.161 | 0.008 | -0.300 | 0.016 |
| Roach | 1125 | 1 | 2 | 2 | -0.360 | 0.027 | -0.226 | 0.084 | 0.073 | 0.155 | 0.492 | 0.068 | 0.493 | -0.021 | 0.270 | -0.081 | 0.082 | -0.113 | -0.147 | -0.057 | -0.229 | -0.082 | -0.158 | -0.003 | -0.291 | 0.025 |
| Roach | 1126 | 1 | 2 | 2 | -0.372 | 0.022 | -0.224 | 0.091 | 0.066 | 0.137 | 0.495 | 0.077 | 0.498 | -0.020 | 0.263 | -0.090 | 0.085 | -0.105 | -0.153 | -0.061 | -0.228 | -0.071 | -0.147 | -0.006 | -0.284 | 0.025 |
| Roach | 1127 | 1 | 2 | 2 | -0.354 | 0.016 | -0.217 | 0.090 | 0.074 | 0.142 | 0.475 | 0.079 | 0.504 | -0.015 | 0.280 | -0.098 | 0.083 | -0.124 | -0.157 | -0.055 | -0.223 | -0.069 | -0.169 | 0.008 | -0.296 | 0.026 |
| Roach | 1128 | 1 | 2 | 2 | -0.373 | 0.008 | -0.230 | 0.090 | 0.063 | 0.176 | 0.491 | 0.058 | 0.485 | -0.042 | 0.272 | -0.072 | 0.104 | -0.097 | -0.149 | -0.063 | -0.229 | -0.076 | -0.150 | -0.004 | -0.283 | 0.022 |
| Roach | 1129 | 1 | 2 | 2 | -0.363 | -0.005 | -0.254 | 0.083 | 0.070 | 0.188 | 0.503 | 0.038 | 0.482 | -0.053 | 0.271 | -0.058 | 0.087 | -0.075 | -0.138 | -0.054 | -0.210 | -0.078 | -0.153 | 0.007 | -0.296 | 0.007 |
| Roach | 1130 | 1 | 2 | 2 | -0.364 | -0.020 | -0.247 | 0.090 | 0.081 | 0.183 | 0.494 | 0.032 | 0.475 | -0.052 | 0.280 | -0.053 | 0.110 | -0.076 | -0.140 | -0.045 | -0.216 | -0.075 | -0.166 | 0.009 | -0.306 | 0.008 |
| Roach | 1101 | 1 | 2 | 3 | -0.349 | 0.002 | -0.238 | 0.078 | 0.071 | 0.173 | 0.500 | 0.055 | 0.487 | -0.042 | 0.270 | -0.073 | 0.098 | -0.090 | -0.151 | -0.049 | -0.216 | -0.071 | -0.176 | 0.002 | -0.296 | 0.016 |
| Roach | 1102 | 1 | 2 | 3 | -0.369 | 0.022 | -0.222 | 0.093 | 0.072 | 0.141 | 0.488 | 0.073 | 0.502 | -0.018 | 0.261 | -0.083 | 0.090 | -0.118 | -0.157 | -0.055 | -0.222 | -0.070 | -0.154 | -0.008 | -0.288 | 0.024 |
| Roach | 1103 | 1 | 2 | 3 | -0.358 | 0.019 | -0.226 | 0.090 | 0.084 | 0.146 | 0.480 | 0.074 | 0.491 | -0.026 | 0.289 | -0.083 | 0.079 | -0.112 | -0.154 | -0.057 | -0.212 | -0.078 | -0.173 | 0.002 | -0.300 | 0.025 |
| Roach | 1104 | 1 | 2 | 3 | -0.356 | 0.016 | -0.220 | 0.094 | 0.075 | 0.159 | 0.497 | 0.070 | 0.493 | -0.023 | 0.271 | -0.080 | 0.064 | -0.111 | -0.152 | -0.064 | -0.227 | -0.081 | -0.158 | -0.001 | -0.289 | 0.021 |
| Roach | 1105 | 1 | 2 | 3 | -0.365 | 0.020 | -0.224 | 0.091 | 0.067 | 0.158 | 0.494 | 0.067 | 0.499 | -0.027 | 0.264 | -0.080 | 0.079 | -0.100 | -0.152 | -0.063 | -0.228 | -0.081 | -0.151 | -0.007 | -0.282 | 0.023 |
| Roach | 1106 | 1 | 2 | 3 | -0.358 | 0.024 | -0.221 | 0.090 | 0.085 | 0.152 | 0.477 | 0.078 | 0.516 | -0.010 | 0.259 | -0.098 | 0.062 | -0.123 | -0.150 | -0.065 | -0.220 | -0.073 | -0.162 | 0.000 | -0.288 | 0.024 |
| Roach | 1107 | 1 | 2 | 3 | -0.358 | 0.036 | -0.215 | 0.093 | 0.074 | 0.133 | 0.490 | 0.086 | 0.503 | -0.008 | 0.271 | -0.093 | 0.062 | -0.125 | -0.152 | -0.070 | -0.228 | -0.073 | -0.161 | -0.008 | -0.286 | 0.030 |
| Roach | 1108 | 1 | 2 | 3 | -0.357 | 0.016 | -0.233 | 0.084 | 0.086 | 0.152 | 0.484 | 0.068 | 0.498 | -0.024 | 0.280 | -0.079 | 0.072 | -0.110 | -0.155 | -0.052 | -0.222 | -0.070 | -0.163 | -0.001 | -0.290 | 0.018 |
| Roach | 1109 | 1 | 2 | 3 | -0.362 | 0.008 | -0.262 | 0.085 | 0.082 | 0.171 | 0.495 | 0.070 | 0.493 | -0.031 | 0.268 | -0.088 | 0.060 | -0.114 | -0.139 | -0.055 | -0.220 | -0.071 | -0.144 | 0.008 | -0.272 | 0.016 |
| Roach | 1110 | 1 | 2 | 3 | -0.351 | 0.022 | -0.255 | 0.081 | 0.080 | 0.144 | 0.493 | 0.072 | 0.501 | -0.018 | 0.271 | -0.086 | 0.067 | -0.117 | -0.153 | -0.054 | -0.213 | -0.065 | -0.157 | 0.001 | -0.283 | 0.021 |
| Roach | 1111 | 1 | 2 | 3 | -0.360 | 0.014 | -0.218 | 0.097 | 0.066 | 0.152 | 0.498 | 0.075 | 0.502 | -0.019 | 0.265 | -0.090 | 0.058 | -0.115 | -0.149 | -0.064 | -0.225 | -0.068 | -0.153 | -0.002 | -0.284 | 0.020 |
| Roach | 1112 | 1 | 2 | 3 | -0.368 | 0.021 | -0.236 | 0.086 | 0.078 | 0.145 | 0.495 | 0.069 | 0.487 | -0.023 | 0.283 | -0.079 | 0.071 | -0.106 | -0.146 | -0.057 | -0.225 | -0.072 | -0.155 | -0.003 | -0.284 | 0.020 |
| Roach | 1113 | 1 | 2 | 3 | -0.368 | 0.017 | -0.223 | 0.093 | 0.079 | 0.158 | 0.494 | 0.073 | 0.494 | -0.025 | 0.265 | -0.077 | 0.068 | -0.119 | -0.150 | -0.064 | -0.226 | -0.075 | -0.150 | -0.002 | -0.282 | 0.019 |
| Roach | 1114 | 1 | 2 | 3 | -0.367 | 0.000 | -0.232 | 0.087 | 0.075 | 0.178 | 0.494 | 0.061 | 0.494 | -0.043 | 0.269 | -0.070 | 0.068 | -0.101 | -0.149 | -0.053 | -0.211 | -0.079 | -0.154 | 0.005 | -0.288 | 0.015 |
| Roach | 1115 | 1 | 2 | 3 | -0.362 | 0.008 | -0.232 | 0.088 | 0.069 | 0.175 | 0.504 | 0.064 | 0.492 | -0.034 | 0.259 | -0.081 | 0.072 | -0.102 | -0.152 | -0.057 | -0.213 | -0.070 | -0.150 | -0.003 | -0.288 | 0.012 |
| Roach | 1116 | 1 | 2 | 3 | -0.354 | 0.024 | -0.222 | 0.083 | 0.081 | 0.140 | 0.486 | 0.080 | 0.493 | -0.013 | 0.281 | -0.096 | 0.077 | -0.122 | -0.149 | -0.054 | -0.226 | -0.074 | -0.171 | 0.003 | -0.295 | 0.029 |
| Roach | 1117 | 1 | 2 | 3 | -0.348 | 0.023 | -0.223 | 0.084 | 0.075 | 0.157 | 0.487 | 0.076 | 0.485 | -0.029 | 0.284 | -0.080 | 0.095 | -0.121 | -0.169 | -0.060 | -0.226 | -0.072 | -0.167 | -0.006 | -0.292 | 0.028 |
| Roach | 1118 | 1 | 2 | 3 | -0.348 | -0.008 | -0.231 | 0.081 | 0.085 | 0.159 | 0.496 | 0.063 | 0.488 | -0.033 | 0.285 | -0.076 | 0.066 | -0.103 | -0.155 | -0.041 | -0.214 | -0.071 | -0.175 | 0.008 | -0.298 | 0.021 |
| Roach | 1119 | 1 | 2 | 3 | -0.352 | 0.040 | -0.255 | 0.082 | 0.078 | 0.140 | 0.495 | 0.078 | 0.492 | -0.014 | 0.271 | -0.088 | 0.082 | -0.128 | -0.159 | -0.062 | -0.212 | -0.065 | -0.159 | -0.007 | -0.281 | 0.023 |
| Roach | 1120 | 1 | 2 | 3 | -0.360 | 0.011 | -0.216 | 0.090 | 0.047 | 0.143 | 0.502 | 0.069 | 0.502 | -0.026 | 0.280 | -0.082 | 0.061 | -0.104 | -0.151 | -0.047 | -0.224 | -0.070 | -0.156 | -0.003 | -0.285 | 0.020 |
| Roach | 1121 | 1 | 2 | 3 | -0.365 | -0.002 | -0.234 | 0.082 | 0.090 | 0.159 | 0.497 | 0.058 | 0.488 | -0.036 | 0.266 | -0.073 | 0.085 | -0.084 | -0.150 | -0.050 | -0.221 | -0.073 | -0.159 | 0.007 | -0.298 | 0.011 |
| Roach | 1122 | 1 | 2 | 3 | -0.348 | 0.022 | -0.225 | 0.084 | 0.078 | 0.130 | 0.485 | 0.084 | 0.498 | -0.004 | 0.286 | -0.102 | 0.066 | -0.133 | -0.154 | -0.054 | -0.232 | -0.070 | -0.164 | 0.013 | -0.289 | 0.030 |
| Roach | 1123 | 1 | 2 | 3 | -0.349 | 0.024 | -0.223 | 0.085 | 0.083 | 0.132 | 0.478 | 0.091 | 0.503 | -0.013 | 0.267 | -0.102 | 0.094 | -0.132 | -0.159 | -0.050 | -0.232 | -0.069 | -0.168 | 0.003 | -0.295 | 0.029 |
| Roach | 1124 | 1 | 2 | 3 | -0.357 | -0.010 | -0.240 | 0.083 | 0.093 | 0.169 | 0.492 | 0.057 | 0.482 | -0.036 | 0.276 | -0.071 | 0.088 | -0.100 | -0.151 | -0.048 | -0.224 | -0.069 | -0.155 | 0.004 | -0.302 | 0.021 |
| Roach | 1125 | 1 | 2 | 3 | -0.365 | 0.025 | -0.218 | 0.083 | 0.070 | 0.153 | 0.493 | 0.069 | 0.488 | -0.025 | 0.261 | -0.080 | 0.111 | -0.112 | -0.149 | -0.058 | -0.237 | -0.079 | -0.160 | -0.001 | -0.293 | 0.025 |
| Roach | 1126 | 1 | 2 | 3 | -0.369 | 0.026 | -0.224 | 0.090 | 0.067 | 0.134 | 0.492 | 0.075 | 0.497 | -0.018 | 0.262 | -0.089 | 0.101 | -0.105 | -0.152 | -0.061 | -0.241 | -0.071 | -0.154 | -0.005 | -0.279 | 0.025 |
| Roach | 1127 | 1 | 2 | 3 | -0.355 | 0.017 | -0.225 | 0.088 | 0.079 | 0.142 | 0.477 | 0.080 | 0.497 | -0.016 | 0.284 | -0.096 | 0.085 | -0.128 | -0.157 | -0.051 | -0.224 | -0.070 | -0.173 | 0.009 | -0.289 | 0.024 |
| Roach | 1128 | 1 | 2 | 3 | -0.376 | 0.013 | -0.244 | 0.086 | 0.071 | 0.176 | 0.492 | 0.051 | 0.471 | -0.037 | 0.280 | -0.068 | 0.113 | -0.100 | -0.143 | -0.064 | -0.233 | -0.074 | -0.149 | -0.005 | -0.282 | 0.022 |
| Roach | 1129 | 1 | 2 | 3 | -0.362 | -0.006 | -0.252 | 0.083 | 0.077 | 0.189 | 0.506 | 0.042 | 0.481 | -0.054 | 0.259 | -0.055 | 0.099 | -0.077 | -0.141 | -0.054 | -0.221 | -0.075 | -0.154 | 0.000 | -0.293 | 0.007 |
| Roach | 1130 | 1 | 2 | 3 | -0.355 | -0.016 | -0.248 | 0.083 | 0.089 | 0.189 | 0.510 | 0.028 | 0.471 | -0.046 | 0.269 | -0.049 | 0.099 | -0.076 | -0.140 | -0.041 | -0.213 | -0.077 | -0.175 | 0.002 | -0.305 | 0.005 |
| Roach | 1191 | 1 | 3 | 1 | -0.363 | 0.025 | -0.220 | 0.089 | 0.073 | 0.143 | 0.483 | 0.070 | 0.501 | -0.021 | 0.277 | -0.083 | 0.081 | -0.110 | -0.156 | -0.057 | -0.215 | -0.075 | -0.162 | -0.003 | -0.298 | 0.021 |
| Roach | 1192 | 1 | 3 | 1 | -0.360 | 0.001 | -0.242 | 0.085 | 0.090 | 0.178 | 0.485 | 0.068 | 0.491 | -0.028 | 0.269 | -0.093 | 0.071 | -0.114 | -0.148 | -0.044 | -0.210 | -0.072 | -0.149 | 0.004 | -0.298 | 0.016 |
| Roach | 1193 | 1 | 3 | 1 | -0.349 | 0.005 | -0.226 | 0.083 | 0.072 | 0.182 | 0.488 | 0.062 | 0.494 | -0.037 | 0.271 | -0.077 | 0.087 | -0.111 | -0.157 | -0.054 | -0.226 | -0.077 | -0.159 | 0.008 | -0.295 | 0.016 |
| Roach | 1194 | 1 | 3 | 1 | -0.361 | 0.037 | -0.249 | 0.082 | 0.081 | 0.154 | 0.483 | 0.074 | 0.487 | -0.018 | 0.274 | -0.092 | 0.099 | -0.122 | -0.152 | -0.063 | -0.224 | -0.073 | -0.156 | -0.006 | -0.282 | 0.026 |
| Roach | 1195 | 1 | 3 | 1 | -0.352 | 0.031 | -0.232 | 0.087 | 0.060 | 0.151 | 0.486 | 0.078 | 0.500 | -0.020 | 0.277 | -0.094 | 0.076 | -0.123 | -0.153 | -0.060 | -0.220 | -0.078 | -0.153 | -0.002 | -0.291 | 0.030 |
| Roach | 1196 | 1 | 3 | 1 | -0.352 | 0.013 | -0.232 | 0.088 | 0.068 | 0.161 | 0.508 | 0.063 | 0.482 | -0.024 | 0.272 | -0.080 | 0.076 | -0.110 | -0.152 | -0.055 | -0.213 | -0.076 | -0.161 | -0.003 | -0.296 | 0.022 |
| Roach | 1197 | 1 | 3 | 1 | -0.371 | 0.006 | -0.250 | 0.096 | 0.066 | 0.180 | 0.486 | 0.058 | 0.476 | -0.041 | 0.277 | -0.084 | 0.106 | -0.116 | -0.136 | -0.046 | -0.199 | -0.077 | -0.152 | 0.013 | -0.304 | 0.010 |
| Roach | 1198 | 1 | 3 | 1 | -0.359 | 0.005 | -0.242 | 0.082 | 0.103 | 0.144 | 0.481 | 0.076 | 0.486 | -0.018 | 0.283 | -0.092 | 0.084 | -0.119 | -0.155 | -0.046 | -0.224 | -0.072 | -0.164 | 0.014 | -0.291 | 0.027 |
| Roach | 1199 | 1 | 3 | 1 | -0.355 | 0.009 | -0.223 | 0.088 | 0.070 | 0.154 | 0.495 | 0.067 | 0.497 | -0.021 | 0.269 | -0.084 | 0.076 | -0.116 | -0.149 | -0.053 | -0.228 | -0.071 | -0.165 | 0.009 | -0.288 | 0.019 |
| Roach | 1200 | 1 | 3 | 1 | -0.346 | 0.031 | -0.226 | 0.085 | 0.087 | 0.147 | 0.478 | 0.075 | 0.504 | -0.012 | 0.271 | -0.093 | 0.084 | -0.118 | -0.163 | -0.060 | -0.221 | -0.075 | -0.168 | -0.005 | -0.300 | 0.025 |
| Roach | 1201 | 1 | 3 | 1 | -0.366 | 0.030 | -0.193 | 0.105 | 0.088 | 0.143 | 0.469 | 0.093 | 0.501 | -0.002 | 0.278 | -0.105 | 0.066 | -0.142 | -0.161 | -0.067 | -0.241 | -0.078 | -0.153 | -0.006 | -0.288 | 0.028 |
| Roach | 1202 | 1 | 3 | 1 | -0.355 | -0.022 | -0.243 | 0.097 | 0.095 | 0.189 | 0.483 | 0.063 | 0.490 | -0.039 | 0.259 | -0.094 | 0.090 | -0.105 | -0.151 | -0.041 | -0.209 | -0.080 | -0.160 | 0.018 | -0.298 | 0.013 |
| Roach | 1203 | 1 | 3 | 1 | -0.367 | 0.007 | -0.242 | 0.090 | 0.078 | 0.172 | 0.498 | 0.061 | 0.487 | -0.027 | 0.262 | -0.079 | 0.080 | -0.117 | -0.144 | -0.047 | -0.218 | -0.081 | -0.152 | 0.004 | -0.281 | 0.018 |
| Roach | 1204 | 1 | 3 | 1 | -0.361 | 0.024 | -0.229 | 0.089 | 0.085 | 0.159 | 0.485 | 0.068 | 0.484 | -0.018 | 0.283 | -0.085 | 0.080 | -0.121 | -0.152 | -0.054 | -0.208 | -0.081 | -0.166 | -0.002 | -0.301 | 0.023 |
| Roach | 1205 | 1 | 3 | 1 | -0.367 | 0.027 | -0.219 | 0.091 | 0.065 | 0.147 | 0.489 | 0.077 | 0.495 | -0.026 | 0.269 | -0.077 | 0.089 | -0.127 | -0.149 | -0.056 | -0.226 | -0.078 | -0.155 | -0.002 | -0.290 | 0.024 |
| Roach | 1206 | 1 | 3 | 1 | -0.366 | 0.008 | -0.240 | 0.087 | 0.058 | 0.177 | 0.496 | 0.059 | 0.496 | -0.040 | 0.267 | -0.074 | 0.077 | -0.103 | -0.148 | -0.057 | -0.221 | -0.072 | -0.138 | 0.001 | -0.282 | 0.013 |
| Roach | 1207 | 1 | 3 | 1 | -0.351 | 0.000 | -0.245 | 0.083 | 0.063 | 0.171 | 0.486 | 0.072 | 0.493 | -0.036 | 0.283 | -0.094 | 0.075 | -0.118 | -0.149 | -0.035 | -0.212 | -0.083 | -0.155 | 0.016 | -0.288 | 0.025 |
| Roach | 1208 | 1 | 3 | 1 | -0.352 | -0.015 | -0.240 | 0.081 | 0.063 | 0.183 | 0.503 | 0.050 | 0.499 | -0.049 | 0.264 | -0.071 | 0.074 | -0.085 | -0.153 | -0.035 | -0.206 | -0.072 | -0.155 | 0.008 | -0.297 | 0.008 |
| Roach | 1209 | 1 | 3 | 1 | -0.367 | 0.006 | -0.239 | 0.087 | 0.072 | 0.153 | 0.498 | 0.068 | 0.497 | -0.018 | 0.265 | -0.090 | 0.067 | -0.119 | -0.148 | -0.043 | -0.212 | -0.068 | -0.152 | 0.006 | -0.281 | 0.018 |
| Roach | 1210 | 1 | 3 | 1 | -0.356 | 0.009 | -0.235 | 0.082 | 0.079 | 0.161 | 0.496 | 0.062 | 0.492 | -0.033 | 0.265 | -0.077 | 0.091 | -0.098 | -0.151 | -0.052 | -0.221 | -0.072 | -0.165 | 0.002 | -0.294 | 0.016 |
| Roach | 1211 | 1 | 3 | 1 | -0.371 | 0.037 | -0.208 | 0.093 | 0.088 | 0.124 | 0.483 | 0.088 | 0.489 | 0.003 | 0.287 | -0.100 | 0.065 | -0.135 | -0.153 | -0.058 | -0.241 | -0.079 | -0.150 | 0.003 | -0.288 | 0.024 |
| Roach | 1212 | 1 | 3 | 1 | -0.372 | 0.013 | -0.216 | 0.094 | 0.075 | 0.151 | 0.491 | 0.082 | 0.501 | -0.017 | 0.260 | -0.098 | 0.065 | -0.122 | -0.147 | -0.052 | -0.226 | -0.074 | -0.146 | 0.002 | -0.285 | 0.022 |
| Roach | 1213 | 1 | 3 | 1 | -0.342 | 0.016 | -0.228 | 0.081 | 0.075 | 0.163 | 0.504 | 0.065 | 0.484 | -0.021 | 0.272 | -0.083 | 0.081 | -0.116 | -0.154 | -0.061 | -0.224 | -0.069 | -0.170 | 0.002 | -0.298 | 0.020 |
| Roach | 1214 | 1 | 3 | 1 | -0.349 | 0.014 | -0.226 | 0.077 | 0.058 | 0.159 | 0.507 | 0.058 | 0.496 | -0.028 | 0.272 | -0.067 | 0.071 | -0.110 | -0.150 | -0.059 | -0.224 | -0.070 | -0.165 | 0.007 | -0.289 | 0.020 |
| Roach | 1215 | 1 | 3 | 1 | -0.371 | 0.014 | -0.225 | 0.090 | 0.061 | 0.167 | 0.503 | 0.051 | 0.502 | -0.029 | 0.257 | -0.068 | 0.072 | -0.094 | -0.144 | -0.058 | -0.217 | -0.078 | -0.153 | -0.008 | -0.284 | 0.013 |
| Roach | 1216 | 1 | 3 | 1 | -0.357 | 0.015 | -0.212 | 0.088 | 0.079 | 0.148 | 0.492 | 0.074 | 0.492 | -0.018 | 0.271 | -0.088 | 0.082 | -0.116 | -0.154 | -0.056 | -0.232 | -0.076 | -0.165 | 0.008 | -0.295 | 0.021 |
| Roach | 1217 | 1 | 3 | 1 | -0.380 | -0.012 | -0.250 | 0.089 | 0.096 | 0.186 | 0.496 | 0.041 | 0.470 | -0.045 | 0.277 | -0.058 | 0.083 | -0.086 | -0.132 | -0.050 | -0.230 | -0.084 | -0.144 | 0.015 | -0.286 | 0.004 |
| Roach | 1218 | 1 | 3 | 1 | -0.345 | 0.003 | -0.231 | 0.090 | 0.072 | 0.174 | 0.501 | 0.069 | 0.486 | -0.036 | 0.271 | -0.082 | 0.072 | -0.114 | -0.150 | -0.047 | -0.208 | -0.079 | -0.166 | 0.002 | -0.302 | 0.020 |
| Roach | 1219 | 1 | 3 | 1 | -0.364 | 0.013 | -0.231 | 0.089 | 0.067 | 0.176 | 0.500 | 0.057 | 0.475 | -0.042 | 0.280 | -0.071 | 0.092 | -0.097 | -0.145 | -0.056 | -0.215 | -0.083 | -0.159 | 0.000 | -0.299 | 0.014 |
| Roach | 1220 | 1 | 3 | 1 | -0.363 | 0.017 | -0.238 | 0.088 | 0.078 | 0.162 | 0.480 | 0.070 | 0.499 | -0.026 | 0.277 | -0.090 | 0.074 | -0.113 | -0.138 | -0.053 | -0.224 | -0.071 | -0.158 | 0.002 | -0.287 | 0.015 |
| Roach | 1191 | 1 | 3 | 2 | -0.363 | 0.021 | -0.229 | 0.091 | 0.080 | 0.146 | 0.479 | 0.069 | 0.500 | -0.022 | 0.276 | -0.084 | 0.086 | -0.111 | -0.156 | -0.054 | -0.209 | -0.077 | -0.162 | 0.000 | -0.300 | 0.021 |
| Roach | 1192 | 1 | 3 | 2 | -0.356 | -0.001 | -0.238 | 0.086 | 0.090 | 0.175 | 0.488 | 0.067 | 0.493 | -0.025 | 0.269 | -0.096 | 0.068 | -0.110 | -0.148 | -0.046 | -0.215 | -0.070 | -0.156 | 0.006 | -0.296 | 0.014 |
| Roach | 1193 | 1 | 3 | 2 | -0.351 | -0.001 | -0.219 | 0.085 | 0.073 | 0.179 | 0.492 | 0.059 | 0.489 | -0.035 | 0.274 | -0.079 | 0.087 | -0.106 | -0.160 | -0.050 | -0.222 | -0.080 | -0.169 | 0.014 | -0.294 | 0.015 |
| Roach | 1194 | 1 | 3 | 2 | -0.365 | 0.033 | -0.236 | 0.085 | 0.087 | 0.153 | 0.488 | 0.076 | 0.483 | -0.015 | 0.273 | -0.091 | 0.090 | -0.123 | -0.156 | -0.060 | -0.222 | -0.079 | -0.157 | -0.006 | -0.284 | 0.028 |
| Roach | 1195 | 1 | 3 | 2 | -0.355 | 0.024 | -0.221 | 0.089 | 0.063 | 0.152 | 0.486 | 0.077 | 0.506 | -0.019 | 0.271 | -0.094 | 0.072 | -0.119 | -0.154 | -0.060 | -0.227 | -0.072 | -0.153 | -0.002 | -0.287 | 0.024 |
| Roach | 1196 | 1 | 3 | 2 | -0.355 | 0.012 | -0.234 | 0.086 | 0.076 | 0.159 | 0.506 | 0.067 | 0.483 | -0.024 | 0.264 | -0.082 | 0.080 | -0.111 | -0.149 | -0.056 | -0.215 | -0.072 | -0.163 | 0.002 | -0.294 | 0.020 |
| Roach | 1197 | 1 | 3 | 2 | -0.373 | 0.000 | -0.247 | 0.095 | 0.074 | 0.182 | 0.486 | 0.058 | 0.478 | -0.039 | 0.272 | -0.083 | 0.102 | -0.117 | -0.138 | -0.048 | -0.200 | -0.078 | -0.150 | 0.017 | -0.303 | 0.012 |
| Roach | 1198 | 1 | 3 | 2 | -0.361 | 0.005 | -0.229 | 0.088 | 0.093 | 0.149 | 0.481 | 0.073 | 0.489 | -0.010 | 0.281 | -0.100 | 0.076 | -0.125 | -0.158 | -0.048 | -0.219 | -0.075 | -0.158 | 0.013 | -0.296 | 0.029 |
| Roach | 1199 | 1 | 3 | 2 | -0.352 | 0.009 | -0.220 | 0.086 | 0.065 | 0.154 | 0.497 | 0.068 | 0.501 | -0.024 | 0.265 | -0.081 | 0.078 | -0.115 | -0.158 | -0.055 | -0.225 | -0.072 | -0.161 | 0.011 | -0.289 | 0.020 |
| Roach | 1200 | 1 | 3 | 2 | -0.346 | 0.028 | -0.229 | 0.084 | 0.090 | 0.147 | 0.479 | 0.077 | 0.508 | -0.015 | 0.269 | -0.093 | 0.074 | -0.111 | -0.157 | -0.061 | -0.228 | -0.073 | -0.162 | -0.005 | -0.297 | 0.021 |
| Roach | 1201 | 1 | 3 | 2 | -0.364 | 0.027 | -0.213 | 0.095 | 0.092 | 0.143 | 0.473 | 0.093 | 0.498 | -0.003 | 0.268 | -0.107 | 0.079 | -0.145 | -0.156 | -0.062 | -0.240 | -0.077 | -0.149 | 0.003 | -0.287 | 0.032 |
| Roach | 1202 | 1 | 3 | 2 | -0.354 | -0.021 | -0.245 | 0.091 | 0.098 | 0.188 | 0.492 | 0.067 | 0.489 | -0.036 | 0.250 | -0.094 | 0.080 | -0.108 | -0.146 | -0.045 | -0.214 | -0.080 | -0.155 | 0.021 | -0.296 | 0.016 |
| Roach | 1203 | 1 | 3 | 2 | -0.368 | 0.004 | -0.244 | 0.088 | 0.076 | 0.171 | 0.501 | 0.058 | 0.482 | -0.025 | 0.261 | -0.081 | 0.092 | -0.116 | -0.146 | -0.047 | -0.221 | -0.079 | -0.150 | 0.007 | -0.281 | 0.020 |
| Roach | 1204 | 1 | 3 | 2 | -0.357 | 0.018 | -0.223 | 0.089 | 0.083 | 0.159 | 0.485 | 0.071 | 0.487 | -0.022 | 0.283 | -0.085 | 0.077 | -0.120 | -0.152 | -0.058 | -0.223 | -0.076 | -0.163 | 0.002 | -0.297 | 0.021 |
| Roach | 1205 | 1 | 3 | 2 | -0.374 | 0.029 | -0.226 | 0.094 | 0.067 | 0.153 | 0.484 | 0.070 | 0.490 | -0.022 | 0.278 | -0.081 | 0.087 | -0.126 | -0.148 | -0.057 | -0.212 | -0.085 | -0.152 | -0.001 | -0.293 | 0.027 |
| Roach | 1206 | 1 | 3 | 2 | -0.368 | 0.004 | -0.237 | 0.093 | 0.065 | 0.180 | 0.493 | 0.054 | 0.496 | -0.035 | 0.261 | -0.074 | 0.085 | -0.105 | -0.146 | -0.054 | -0.224 | -0.074 | -0.141 | -0.006 | -0.283 | 0.016 |
| Roach | 1207 | 1 | 3 | 2 | -0.357 | -0.002 | -0.249 | 0.080 | 0.066 | 0.166 | 0.496 | 0.066 | 0.481 | -0.029 | 0.286 | -0.095 | 0.076 | -0.113 | -0.143 | -0.042 | -0.211 | -0.078 | -0.157 | 0.018 | -0.288 | 0.029 |
| Roach | 1208 | 1 | 3 | 2 | -0.352 | -0.019 | -0.242 | 0.079 | 0.070 | 0.184 | 0.502 | 0.048 | 0.492 | -0.043 | 0.270 | -0.075 | 0.074 | -0.092 | -0.147 | -0.040 | -0.207 | -0.071 | -0.163 | 0.018 | -0.297 | 0.011 |
| Roach | 1209 | 1 | 3 | 2 | -0.368 | 0.011 | -0.230 | 0.096 | 0.058 | 0.150 | 0.495 | 0.070 | 0.500 | -0.021 | 0.270 | -0.090 | 0.069 | -0.119 | -0.148 | -0.049 | -0.208 | -0.068 | -0.153 | 0.000 | -0.285 | 0.020 |
| Roach | 1210 | 1 | 3 | 2 | -0.356 | 0.009 | -0.225 | 0.086 | 0.080 | 0.156 | 0.497 | 0.065 | 0.502 | -0.032 | 0.251 | -0.075 | 0.090 | -0.097 | -0.158 | -0.051 | -0.221 | -0.074 | -0.165 | 0.002 | -0.294 | 0.011 |
| Roach | 1211 | 1 | 3 | 2 | -0.373 | 0.037 | -0.208 | 0.096 | 0.088 | 0.126 | 0.478 | 0.086 | 0.499 | 0.003 | 0.282 | -0.096 | 0.058 | -0.133 | -0.157 | -0.070 | -0.233 | -0.075 | -0.149 | 0.002 | -0.285 | 0.025 |
| Roach | 1212 | 1 | 3 | 2 | -0.369 | 0.012 | -0.218 | 0.095 | 0.072 | 0.154 | 0.481 | 0.080 | 0.504 | -0.021 | 0.264 | -0.096 | 0.072 | -0.125 | -0.131 | -0.058 | -0.236 | -0.073 | -0.151 | 0.008 | -0.288 | 0.023 |
| Roach | 1213 | 1 | 3 | 2 | -0.345 | 0.019 | -0.220 | 0.082 | 0.067 | 0.162 | 0.497 | 0.067 | 0.493 | -0.025 | 0.273 | -0.083 | 0.081 | -0.112 | -0.155 | -0.061 | -0.224 | -0.070 | -0.166 | 0.003 | -0.300 | 0.017 |
| Roach | 1214 | 1 | 3 | 2 | -0.351 | 0.015 | -0.225 | 0.075 | 0.054 | 0.162 | 0.507 | 0.055 | 0.491 | -0.029 | 0.280 | -0.067 | 0.072 | -0.108 | -0.150 | -0.058 | -0.223 | -0.071 | -0.166 | 0.005 | -0.289 | 0.021 |
| Roach | 1215 | 1 | 3 | 2 | -0.369 | 0.012 | -0.242 | 0.092 | 0.070 | 0.171 | 0.498 | 0.054 | 0.491 | -0.036 | 0.262 | -0.070 | 0.085 | -0.096 | -0.141 | -0.059 | -0.216 | -0.079 | -0.151 | -0.005 | -0.287 | 0.014 |
| Roach | 1216 | 1 | 3 | 2 | -0.359 | 0.015 | -0.218 | 0.085 | 0.083 | 0.148 | 0.490 | 0.071 | 0.486 | -0.016 | 0.281 | -0.088 | 0.079 | -0.117 | -0.154 | -0.055 | -0.227 | -0.075 | -0.164 | 0.006 | -0.296 | 0.025 |
| Roach | 1217 | 1 | 3 | 2 | -0.380 | -0.016 | -0.239 | 0.096 | 0.085 | 0.183 | 0.502 | 0.040 | 0.472 | -0.046 | 0.273 | -0.055 | 0.082 | -0.084 | -0.131 | -0.053 | -0.231 | -0.085 | -0.146 | 0.012 | -0.288 | 0.008 |
| Roach | 1218 | 1 | 3 | 2 | -0.353 | 0.003 | -0.239 | 0.088 | 0.079 | 0.174 | 0.495 | 0.063 | 0.484 | -0.033 | 0.271 | -0.080 | 0.083 | -0.116 | -0.152 | -0.046 | -0.204 | -0.079 | -0.166 | 0.007 | -0.298 | 0.019 |
| Roach | 1219 | 1 | 3 | 2 | -0.366 | 0.016 | -0.228 | 0.089 | 0.070 | 0.179 | 0.502 | 0.054 | 0.475 | -0.039 | 0.277 | -0.065 | 0.084 | -0.097 | -0.146 | -0.061 | -0.217 | -0.077 | -0.152 | -0.009 | -0.300 | 0.012 |
| Roach | 1220 | 1 | 3 | 2 | -0.357 | 0.020 | -0.255 | 0.078 | 0.085 | 0.160 | 0.478 | 0.072 | 0.501 | -0.027 | 0.275 | -0.089 | 0.076 | -0.114 | -0.134 | -0.052 | -0.225 | -0.063 | -0.157 | -0.003 | -0.288 | 0.018 |
| Roach | 1191 | 1 | 3 | 3 | -0.362 | 0.018 | -0.226 | 0.088 | 0.079 | 0.145 | 0.476 | 0.071 | 0.506 | -0.023 | 0.278 | -0.084 | 0.077 | -0.109 | -0.158 | -0.055 | -0.216 | -0.071 | -0.157 | -0.002 | -0.298 | 0.021 |
| Roach | 1192 | 1 | 3 | 3 | -0.353 | 0.000 | -0.224 | 0.090 | 0.072 | 0.177 | 0.485 | 0.069 | 0.500 | -0.029 | 0.269 | -0.091 | 0.073 | -0.119 | -0.144 | -0.046 | -0.227 | -0.069 | -0.153 | 0.006 | -0.297 | 0.013 |
| Roach | 1193 | 1 | 3 | 3 | -0.351 | 0.003 | -0.223 | 0.082 | 0.074 | 0.183 | 0.483 | 0.061 | 0.494 | -0.040 | 0.271 | -0.076 | 0.097 | -0.109 | -0.157 | -0.048 | -0.224 | -0.081 | -0.168 | 0.008 | -0.296 | 0.017 |
| Roach | 1194 | 1 | 3 | 3 | -0.361 | 0.030 | -0.240 | 0.084 | 0.081 | 0.150 | 0.486 | 0.076 | 0.487 | -0.020 | 0.273 | -0.088 | 0.097 | -0.121 | -0.151 | -0.063 | -0.232 | -0.069 | -0.156 | -0.005 | -0.285 | 0.026 |
| Roach | 1195 | 1 | 3 | 3 | -0.352 | 0.025 | -0.223 | 0.088 | 0.069 | 0.150 | 0.488 | 0.074 | 0.501 | -0.016 | 0.272 | -0.089 | 0.075 | -0.121 | -0.156 | -0.062 | -0.226 | -0.074 | -0.158 | -0.002 | -0.289 | 0.027 |
| Roach | 1196 | 1 | 3 | 3 | -0.356 | 0.013 | -0.244 | 0.085 | 0.079 | 0.157 | 0.495 | 0.061 | 0.485 | -0.026 | 0.283 | -0.076 | 0.074 | -0.111 | -0.149 | -0.052 | -0.208 | -0.073 | -0.163 | 0.000 | -0.296 | 0.022 |
| Roach | 1197 | 1 | 3 | 3 | -0.374 | 0.005 | -0.247 | 0.097 | 0.079 | 0.184 | 0.482 | 0.057 | 0.474 | -0.039 | 0.278 | -0.083 | 0.107 | -0.113 | -0.137 | -0.046 | -0.199 | -0.072 | -0.158 | 0.000 | -0.305 | 0.011 |
| Roach | 1198 | 1 | 3 | 3 | -0.359 | 0.006 | -0.225 | 0.093 | 0.088 | 0.145 | 0.477 | 0.072 | 0.494 | -0.014 | 0.284 | -0.094 | 0.082 | -0.118 | -0.151 | -0.048 | -0.225 | -0.072 | -0.167 | 0.001 | -0.296 | 0.029 |
| Roach | 1199 | 1 | 3 | 3 | -0.353 | 0.011 | -0.216 | 0.090 | 0.056 | 0.151 | 0.494 | 0.069 | 0.506 | -0.024 | 0.267 | -0.084 | 0.078 | -0.113 | -0.150 | -0.053 | -0.226 | -0.071 | -0.166 | 0.005 | -0.290 | 0.021 |
| Roach | 1200 | 1 | 3 | 3 | -0.347 | 0.026 | -0.224 | 0.086 | 0.087 | 0.150 | 0.479 | 0.075 | 0.501 | -0.017 | 0.272 | -0.091 | 0.084 | -0.115 | -0.159 | -0.060 | -0.222 | -0.072 | -0.170 | -0.006 | -0.301 | 0.024 |
| Roach | 1201 | 1 | 3 | 3 | -0.367 | 0.034 | -0.213 | 0.097 | 0.093 | 0.141 | 0.473 | 0.089 | 0.494 | 0.000 | 0.275 | -0.106 | 0.078 | -0.139 | -0.164 | -0.067 | -0.234 | -0.073 | -0.149 | -0.004 | -0.286 | 0.028 |
| Roach | 1202 | 1 | 3 | 3 | -0.357 | -0.023 | -0.239 | 0.095 | 0.099 | 0.188 | 0.480 | 0.068 | 0.486 | -0.040 | 0.267 | -0.094 | 0.088 | -0.111 | -0.149 | -0.040 | -0.220 | -0.077 | -0.158 | 0.023 | -0.297 | 0.012 |
| Roach | 1203 | 1 | 3 | 3 | -0.366 | 0.005 | -0.246 | 0.087 | 0.077 | 0.167 | 0.504 | 0.061 | 0.477 | -0.022 | 0.270 | -0.083 | 0.080 | -0.118 | -0.143 | -0.048 | -0.233 | -0.072 | -0.145 | 0.004 | -0.275 | 0.017 |
| Roach | 1204 | 1 | 3 | 3 | -0.356 | 0.020 | -0.225 | 0.087 | 0.085 | 0.157 | 0.489 | 0.070 | 0.489 | -0.022 | 0.271 | -0.079 | 0.088 | -0.120 | -0.154 | -0.055 | -0.225 | -0.072 | -0.167 | -0.009 | -0.295 | 0.024 |
| Roach | 1205 | 1 | 3 | 3 | -0.373 | 0.025 | -0.223 | 0.097 | 0.074 | 0.154 | 0.484 | 0.072 | 0.488 | -0.025 | 0.274 | -0.078 | 0.092 | -0.122 | -0.147 | -0.056 | -0.223 | -0.080 | -0.152 | -0.010 | -0.293 | 0.024 |
| Roach | 1206 | 1 | 3 | 3 | -0.372 | 0.008 | -0.241 | 0.095 | 0.054 | 0.178 | 0.492 | 0.055 | 0.498 | -0.041 | 0.260 | -0.076 | 0.095 | -0.095 | -0.135 | -0.066 | -0.224 | -0.071 | -0.144 | -0.001 | -0.281 | 0.016 |
| Roach | 1207 | 1 | 3 | 3 | -0.356 | 0.001 | -0.231 | 0.089 | 0.058 | 0.170 | 0.480 | 0.067 | 0.509 | -0.034 | 0.272 | -0.091 | 0.079 | -0.114 | -0.152 | -0.049 | -0.208 | -0.078 | -0.157 | 0.014 | -0.293 | 0.024 |
| Roach | 1208 | 1 | 3 | 3 | -0.350 | -0.015 | -0.241 | 0.074 | 0.079 | 0.184 | 0.516 | 0.044 | 0.485 | -0.036 | 0.259 | -0.068 | 0.069 | -0.094 | -0.144 | -0.037 | -0.211 | -0.070 | -0.165 | 0.007 | -0.298 | 0.010 |
| Roach | 1209 | 1 | 3 | 3 | -0.364 | 0.002 | -0.234 | 0.093 | 0.081 | 0.153 | 0.496 | 0.068 | 0.492 | -0.015 | 0.268 | -0.089 | 0.069 | -0.120 | -0.144 | -0.055 | -0.217 | -0.062 | -0.160 | 0.004 | -0.287 | 0.022 |
| Roach | 1210 | 1 | 3 | 3 | -0.357 | 0.008 | -0.246 | 0.085 | 0.084 | 0.160 | 0.492 | 0.059 | 0.496 | -0.032 | 0.262 | -0.076 | 0.091 | -0.094 | -0.138 | -0.051 | -0.223 | -0.067 | -0.165 | -0.005 | -0.296 | 0.014 |
| Roach | 1211 | 1 | 3 | 3 | -0.369 | 0.031 | -0.209 | 0.092 | 0.083 | 0.123 | 0.479 | 0.087 | 0.495 | 0.000 | 0.289 | -0.095 | 0.064 | -0.138 | -0.156 | -0.055 | -0.239 | -0.075 | -0.152 | 0.005 | -0.285 | 0.027 |
| Roach | 1212 | 1 | 3 | 3 | -0.376 | 0.013 | -0.215 | 0.097 | 0.071 | 0.151 | 0.488 | 0.081 | 0.496 | -0.018 | 0.269 | -0.098 | 0.065 | -0.122 | -0.142 | -0.054 | -0.224 | -0.078 | -0.147 | 0.004 | -0.286 | 0.024 |
| Roach | 1213 | 1 | 3 | 3 | -0.339 | 0.007 | -0.225 | 0.086 | 0.078 | 0.160 | 0.496 | 0.064 | 0.491 | -0.022 | 0.273 | -0.081 | 0.082 | -0.115 | -0.158 | -0.055 | -0.221 | -0.065 | -0.175 | -0.001 | -0.301 | 0.021 |
| Roach | 1214 | 1 | 3 | 3 | -0.351 | 0.019 | -0.234 | 0.074 | 0.074 | 0.165 | 0.505 | 0.058 | 0.487 | -0.026 | 0.274 | -0.066 | 0.072 | -0.111 | -0.151 | -0.057 | -0.223 | -0.071 | -0.164 | -0.006 | -0.289 | 0.022 |
| Roach | 1215 | 1 | 3 | 3 | -0.364 | 0.015 | -0.244 | 0.083 | 0.065 | 0.168 | 0.503 | 0.049 | 0.501 | -0.031 | 0.254 | -0.070 | 0.079 | -0.088 | -0.141 | -0.057 | -0.219 | -0.079 | -0.153 | -0.005 | -0.282 | 0.017 |
| Roach | 1216 | 1 | 3 | 3 | -0.359 | 0.015 | -0.217 | 0.090 | 0.064 | 0.143 | 0.497 | 0.070 | 0.496 | -0.017 | 0.267 | -0.088 | 0.082 | -0.114 | -0.143 | -0.056 | -0.225 | -0.076 | -0.165 | 0.009 | -0.298 | 0.023 |
| Roach | 1217 | 1 | 3 | 3 | -0.376 | -0.020 | -0.252 | 0.088 | 0.082 | 0.181 | 0.502 | 0.035 | 0.473 | -0.049 | 0.268 | -0.053 | 0.101 | -0.080 | -0.132 | -0.052 | -0.228 | -0.085 | -0.153 | 0.025 | -0.285 | 0.010 |
| Roach | 1218 | 1 | 3 | 3 | -0.348 | 0.005 | -0.245 | 0.077 | 0.071 | 0.171 | 0.499 | 0.061 | 0.484 | -0.032 | 0.280 | -0.083 | 0.082 | -0.110 | -0.148 | -0.039 | -0.210 | -0.070 | -0.166 | 0.003 | -0.298 | 0.019 |
| Roach | 1219 | 1 | 3 | 3 | -0.358 | 0.013 | -0.239 | 0.084 | 0.070 | 0.177 | 0.502 | 0.051 | 0.479 | -0.040 | 0.277 | -0.065 | 0.087 | -0.092 | -0.146 | -0.060 | -0.216 | -0.076 | -0.160 | -0.008 | -0.297 | 0.016 |
| Roach | 1220 | 1 | 3 | 3 | -0.360 | 0.017 | -0.234 | 0.085 | 0.079 | 0.161 | 0.484 | 0.067 | 0.492 | -0.023 | 0.280 | -0.085 | 0.077 | -0.113 | -0.140 | -0.065 | -0.233 | -0.064 | -0.153 | 0.000 | -0.291 | 0.020 |
| Roach | 1001 | 2 | 1 | 1 | -0.345 | 0.016 | -0.226 | 0.084 | 0.086 | 0.161 | 0.490 | 0.084 | 0.505 | -0.010 | 0.269 | -0.106 | 0.045 | -0.127 | -0.158 | -0.047 | -0.232 | -0.072 | -0.154 | -0.002 | -0.280 | 0.019 |
| Roach | 1002 | 2 | 1 | 1 | -0.355 | 0.017 | -0.231 | 0.093 | 0.085 | 0.154 | 0.480 | 0.084 | 0.499 | -0.017 | 0.279 | -0.101 | 0.066 | -0.123 | -0.161 | -0.054 | -0.240 | -0.068 | -0.142 | -0.003 | -0.279 | 0.017 |
| Roach | 1003 | 2 | 1 | 1 | -0.354 | -0.011 | -0.245 | 0.083 | 0.077 | 0.191 | 0.516 | 0.051 | 0.476 | -0.037 | 0.269 | -0.074 | 0.069 | -0.095 | -0.152 | -0.047 | -0.230 | -0.072 | -0.149 | 0.005 | -0.276 | 0.006 |
| Roach | 1004 | 2 | 1 | 1 | -0.357 | 0.006 | -0.230 | 0.091 | 0.085 | 0.175 | 0.495 | 0.071 | 0.482 | -0.031 | 0.274 | -0.090 | 0.076 | -0.107 | -0.160 | -0.051 | -0.232 | -0.076 | -0.150 | 0.002 | -0.282 | 0.009 |
| Roach | 1005 | 2 | 1 | 1 | -0.347 | -0.006 | -0.230 | 0.081 | 0.091 | 0.168 | 0.489 | 0.067 | 0.478 | -0.029 | 0.285 | -0.094 | 0.091 | -0.105 | -0.164 | -0.032 | -0.237 | -0.077 | -0.159 | 0.012 | -0.297 | 0.014 |
| Roach | 1006 | 2 | 1 | 1 | -0.355 | 0.014 | -0.223 | 0.096 | 0.080 | 0.165 | 0.492 | 0.082 | 0.486 | -0.018 | 0.278 | -0.103 | 0.065 | -0.122 | -0.154 | -0.055 | -0.235 | -0.070 | -0.151 | -0.001 | -0.283 | 0.014 |
| Roach | 1007 | 2 | 1 | 1 | -0.359 | -0.023 | -0.232 | 0.097 | 0.093 | 0.211 | 0.518 | 0.053 | 0.465 | -0.051 | 0.262 | -0.072 | 0.068 | -0.085 | -0.149 | -0.053 | -0.225 | -0.078 | -0.156 | 0.009 | -0.285 | -0.007 |
| Roach | 1008 | 2 | 1 | 1 | -0.352 | 0.012 | -0.230 | 0.100 | 0.073 | 0.178 | 0.483 | 0.078 | 0.497 | -0.032 | 0.268 | -0.094 | 0.076 | -0.122 | -0.161 | -0.055 | -0.219 | -0.076 | -0.150 | -0.004 | -0.285 | 0.014 |
| Roach | 1009 | 2 | 1 | 1 | -0.357 | 0.015 | -0.224 | 0.096 | 0.086 | 0.162 | 0.484 | 0.078 | 0.483 | -0.021 | 0.284 | -0.096 | 0.081 | -0.120 | -0.163 | -0.053 | -0.238 | -0.072 | -0.153 | -0.004 | -0.283 | 0.015 |
| Roach | 1010 | 2 | 1 | 1 | -0.355 | 0.021 | -0.217 | 0.102 | 0.090 | 0.165 | 0.475 | 0.085 | 0.486 | -0.017 | 0.282 | -0.103 | 0.080 | -0.131 | -0.168 | -0.060 | -0.234 | -0.078 | -0.149 | -0.002 | -0.290 | 0.017 |
| Roach | 1011 | 2 | 1 | 1 | -0.347 | 0.024 | -0.231 | 0.092 | 0.073 | 0.166 | 0.491 | 0.080 | 0.505 | -0.025 | 0.248 | -0.097 | 0.088 | -0.118 | -0.165 | -0.065 | -0.220 | -0.072 | -0.153 | 0.003 | -0.288 | 0.013 |
| Roach | 1012 | 2 | 1 | 1 | -0.356 | 0.018 | -0.217 | 0.094 | 0.095 | 0.153 | 0.485 | 0.087 | 0.489 | -0.009 | 0.272 | -0.107 | 0.077 | -0.126 | -0.170 | -0.054 | -0.244 | -0.069 | -0.151 | -0.005 | -0.280 | 0.019 |
| Roach | 1013 | 2 | 1 | 1 | -0.356 | 0.012 | -0.233 | 0.091 | 0.085 | 0.164 | 0.496 | 0.076 | 0.492 | -0.023 | 0.262 | -0.091 | 0.073 | -0.115 | -0.161 | -0.051 | -0.230 | -0.072 | -0.148 | -0.006 | -0.280 | 0.015 |
| Roach | 1014 | 2 | 1 | 1 | -0.360 | 0.018 | -0.235 | 0.088 | 0.096 | 0.147 | 0.484 | 0.088 | 0.500 | -0.008 | 0.264 | -0.111 | 0.065 | -0.122 | -0.158 | -0.054 | -0.236 | -0.073 | -0.143 | 0.004 | -0.277 | 0.023 |
| Roach | 1015 | 2 | 1 | 1 | -0.360 | -0.007 | -0.226 | 0.097 | 0.074 | 0.175 | 0.509 | 0.066 | 0.490 | -0.031 | 0.260 | -0.087 | 0.056 | -0.101 | -0.150 | -0.049 | -0.230 | -0.081 | -0.146 | 0.010 | -0.277 | 0.007 |
| Roach | 1016 | 2 | 1 | 1 | -0.351 | 0.006 | -0.227 | 0.097 | 0.098 | 0.189 | 0.491 | 0.076 | 0.478 | -0.026 | 0.264 | -0.096 | 0.084 | -0.127 | -0.164 | -0.064 | -0.226 | -0.069 | -0.156 | 0.007 | -0.289 | 0.006 |
| Roach | 1017 | 2 | 1 | 1 | -0.351 | 0.001 | -0.230 | 0.094 | 0.094 | 0.174 | 0.489 | 0.067 | 0.490 | -0.027 | 0.269 | -0.083 | 0.074 | -0.114 | -0.165 | -0.052 | -0.228 | -0.071 | -0.153 | 0.001 | -0.289 | 0.010 |
| Roach | 1018 | 2 | 1 | 1 | -0.348 | 0.002 | -0.221 | 0.097 | 0.080 | 0.178 | 0.507 | 0.068 | 0.483 | -0.028 | 0.272 | -0.085 | 0.058 | -0.108 | -0.164 | -0.051 | -0.224 | -0.074 | -0.157 | -0.004 | -0.285 | 0.006 |
| Roach | 1019 | 2 | 1 | 1 | -0.350 | 0.011 | -0.227 | 0.095 | 0.103 | 0.176 | 0.497 | 0.073 | 0.474 | -0.026 | 0.274 | -0.085 | 0.076 | -0.111 | -0.163 | -0.061 | -0.235 | -0.067 | -0.158 | -0.010 | -0.290 | 0.005 |
| Roach | 1020 | 2 | 1 | 1 | -0.351 | 0.008 | -0.226 | 0.092 | 0.084 | 0.169 | 0.497 | 0.075 | 0.486 | -0.023 | 0.277 | -0.090 | 0.059 | -0.115 | -0.162 | -0.057 | -0.235 | -0.073 | -0.144 | 0.002 | -0.284 | 0.013 |
| Roach | 1021 | 2 | 1 | 1 | -0.352 | 0.003 | -0.230 | 0.087 | 0.093 | 0.163 | 0.503 | 0.076 | 0.481 | -0.026 | 0.270 | -0.091 | 0.067 | -0.109 | -0.162 | -0.051 | -0.228 | -0.073 | -0.155 | 0.009 | -0.287 | 0.011 |
| Roach | 1022 | 2 | 1 | 1 | -0.364 | 0.013 | -0.223 | 0.095 | 0.092 | 0.164 | 0.490 | 0.068 | 0.481 | -0.024 | 0.278 | -0.082 | 0.076 | -0.110 | -0.159 | -0.048 | -0.225 | -0.083 | -0.154 | -0.005 | -0.292 | 0.013 |
| Roach | 1023 | 2 | 1 | 1 | -0.353 | 0.016 | -0.226 | 0.091 | 0.100 | 0.145 | 0.493 | 0.081 | 0.500 | -0.011 | 0.261 | -0.091 | 0.057 | -0.120 | -0.167 | -0.056 | -0.229 | -0.071 | -0.152 | -0.001 | -0.284 | 0.018 |
| Roach | 1024 | 2 | 1 | 1 | -0.366 | 0.004 | -0.226 | 0.105 | 0.066 | 0.182 | 0.506 | 0.074 | 0.485 | -0.033 | 0.265 | -0.087 | 0.055 | -0.118 | -0.149 | -0.056 | -0.223 | -0.081 | -0.139 | -0.004 | -0.274 | 0.014 |
| Roach | 1025 | 2 | 1 | 1 | -0.345 | 0.011 | -0.225 | 0.095 | 0.084 | 0.173 | 0.500 | 0.080 | 0.486 | -0.024 | 0.268 | -0.097 | 0.065 | -0.118 | -0.165 | -0.052 | -0.225 | -0.074 | -0.153 | -0.002 | -0.288 | 0.009 |
| Roach | 1026 | 2 | 1 | 1 | -0.360 | -0.009 | -0.260 | 0.082 | 0.087 | 0.197 | 0.520 | 0.036 | 0.468 | -0.046 | 0.263 | -0.054 | 0.079 | -0.084 | -0.152 | -0.046 | -0.209 | -0.072 | -0.146 | -0.004 | -0.289 | 0.000 |
| Roach | 1027 | 2 | 1 | 1 | -0.353 | 0.034 | -0.221 | 0.097 | 0.091 | 0.140 | 0.468 | 0.088 | 0.501 | 0.000 | 0.289 | -0.105 | 0.068 | -0.138 | -0.172 | -0.061 | -0.230 | -0.066 | -0.152 | -0.013 | -0.289 | 0.024 |
| Roach | 1028 | 2 | 1 | 1 | -0.353 | 0.005 | -0.222 | 0.089 | 0.076 | 0.159 | 0.498 | 0.073 | 0.498 | -0.026 | 0.269 | -0.087 | 0.062 | -0.106 | -0.164 | -0.046 | -0.230 | -0.074 | -0.149 | 0.000 | -0.284 | 0.014 |
| Roach | 1029 | 2 | 1 | 1 | -0.344 | -0.001 | -0.240 | 0.088 | 0.093 | 0.178 | 0.506 | 0.057 | 0.478 | -0.037 | 0.275 | -0.073 | 0.073 | -0.091 | -0.170 | -0.046 | -0.216 | -0.075 | -0.162 | -0.003 | -0.293 | 0.004 |
| Roach | 1030 | 2 | 1 | 1 | -0.349 | -0.002 | -0.228 | 0.097 | 0.121 | 0.171 | 0.488 | 0.082 | 0.481 | -0.017 | 0.269 | -0.101 | 0.061 | -0.123 | -0.167 | -0.046 | -0.229 | -0.078 | -0.154 | 0.003 | -0.292 | 0.013 |
| Roach | 1001 | 2 | 1 | 2 | -0.349 | 0.009 | -0.216 | 0.092 | 0.092 | 0.161 | 0.481 | 0.079 | 0.507 | -0.012 | 0.273 | -0.102 | 0.049 | -0.118 | -0.163 | -0.050 | -0.233 | -0.075 | -0.154 | 0.001 | -0.286 | 0.015 |
| Roach | 1002 | 2 | 1 | 2 | -0.359 | 0.013 | -0.223 | 0.097 | 0.088 | 0.159 | 0.474 | 0.080 | 0.503 | -0.013 | 0.274 | -0.103 | 0.070 | -0.125 | -0.158 | -0.054 | -0.242 | -0.067 | -0.145 | -0.003 | -0.281 | 0.014 |
| Roach | 1003 | 2 | 1 | 2 | -0.357 | -0.014 | -0.228 | 0.093 | 0.069 | 0.191 | 0.510 | 0.052 | 0.487 | -0.039 | 0.266 | -0.074 | 0.064 | -0.095 | -0.158 | -0.050 | -0.228 | -0.074 | -0.145 | 0.008 | -0.279 | 0.002 |
| Roach | 1004 | 2 | 1 | 2 | -0.357 | 0.003 | -0.225 | 0.098 | 0.066 | 0.168 | 0.499 | 0.070 | 0.486 | -0.033 | 0.272 | -0.086 | 0.086 | -0.107 | -0.164 | -0.053 | -0.236 | -0.072 | -0.148 | 0.001 | -0.280 | 0.011 |
| Roach | 1005 | 2 | 1 | 2 | -0.348 | -0.009 | -0.229 | 0.082 | 0.092 | 0.167 | 0.488 | 0.069 | 0.481 | -0.031 | 0.283 | -0.094 | 0.089 | -0.107 | -0.162 | -0.028 | -0.236 | -0.078 | -0.160 | 0.020 | -0.297 | 0.009 |
| Roach | 1006 | 2 | 1 | 2 | -0.359 | 0.017 | -0.230 | 0.092 | 0.080 | 0.164 | 0.489 | 0.080 | 0.485 | -0.018 | 0.281 | -0.107 | 0.068 | -0.119 | -0.159 | -0.052 | -0.223 | -0.072 | -0.147 | -0.001 | -0.285 | 0.015 |
| Roach | 1007 | 2 | 1 | 2 | -0.361 | -0.018 | -0.237 | 0.099 | 0.084 | 0.208 | 0.510 | 0.050 | 0.469 | -0.048 | 0.268 | -0.074 | 0.073 | -0.087 | -0.149 | -0.049 | -0.231 | -0.076 | -0.146 | 0.004 | -0.281 | -0.008 |
| Roach | 1008 | 2 | 1 | 2 | -0.351 | 0.011 | -0.223 | 0.100 | 0.074 | 0.174 | 0.494 | 0.080 | 0.491 | -0.028 | 0.274 | -0.094 | 0.055 | -0.120 | -0.161 | -0.054 | -0.225 | -0.078 | -0.141 | 0.001 | -0.288 | 0.009 |
| Roach | 1009 | 2 | 1 | 2 | -0.357 | 0.012 | -0.222 | 0.094 | 0.086 | 0.158 | 0.490 | 0.083 | 0.485 | -0.024 | 0.281 | -0.095 | 0.069 | -0.118 | -0.163 | -0.053 | -0.236 | -0.072 | -0.149 | 0.001 | -0.283 | 0.013 |
| Roach | 1010 | 2 | 1 | 2 | -0.356 | 0.025 | -0.215 | 0.103 | 0.090 | 0.165 | 0.475 | 0.086 | 0.490 | -0.014 | 0.277 | -0.104 | 0.077 | -0.132 | -0.172 | -0.060 | -0.231 | -0.079 | -0.145 | -0.004 | -0.290 | 0.016 |
| Roach | 1011 | 2 | 1 | 2 | -0.347 | 0.026 | -0.230 | 0.092 | 0.077 | 0.164 | 0.492 | 0.079 | 0.494 | -0.025 | 0.260 | -0.095 | 0.090 | -0.117 | -0.172 | -0.066 | -0.220 | -0.074 | -0.154 | 0.001 | -0.288 | 0.013 |
| Roach | 1012 | 2 | 1 | 2 | -0.356 | 0.021 | -0.215 | 0.095 | 0.092 | 0.152 | 0.477 | 0.083 | 0.499 | -0.004 | 0.277 | -0.106 | 0.066 | -0.128 | -0.172 | -0.057 | -0.239 | -0.071 | -0.146 | -0.004 | -0.283 | 0.019 |
| Roach | 1013 | 2 | 1 | 2 | -0.353 | 0.012 | -0.228 | 0.092 | 0.070 | 0.162 | 0.497 | 0.075 | 0.493 | -0.025 | 0.266 | -0.091 | 0.076 | -0.117 | -0.160 | -0.050 | -0.234 | -0.072 | -0.146 | -0.001 | -0.282 | 0.014 |
| Roach | 1014 | 2 | 1 | 2 | -0.363 | 0.016 | -0.226 | 0.094 | 0.104 | 0.147 | 0.480 | 0.090 | 0.500 | -0.008 | 0.265 | -0.109 | 0.063 | -0.121 | -0.163 | -0.051 | -0.230 | -0.075 | -0.148 | -0.002 | -0.281 | 0.022 |
| Roach | 1015 | 2 | 1 | 2 | -0.359 | 0.001 | -0.231 | 0.097 | 0.077 | 0.174 | 0.512 | 0.064 | 0.484 | -0.029 | 0.264 | -0.085 | 0.057 | -0.099 | -0.153 | -0.054 | -0.225 | -0.085 | -0.147 | 0.009 | -0.278 | 0.008 |
| Roach | 1016 | 2 | 1 | 2 | -0.348 | 0.005 | -0.223 | 0.102 | 0.089 | 0.191 | 0.492 | 0.073 | 0.486 | -0.028 | 0.262 | -0.095 | 0.073 | -0.120 | -0.162 | -0.061 | -0.223 | -0.076 | -0.154 | 0.006 | -0.291 | 0.004 |
| Roach | 1017 | 2 | 1 | 2 | -0.351 | 0.000 | -0.227 | 0.096 | 0.097 | 0.173 | 0.495 | 0.067 | 0.489 | -0.026 | 0.266 | -0.083 | 0.065 | -0.108 | -0.169 | -0.057 | -0.222 | -0.073 | -0.151 | 0.003 | -0.292 | 0.008 |
| Roach | 1018 | 2 | 1 | 2 | -0.348 | 0.004 | -0.226 | 0.096 | 0.079 | 0.177 | 0.506 | 0.068 | 0.484 | -0.029 | 0.271 | -0.085 | 0.062 | -0.108 | -0.167 | -0.051 | -0.220 | -0.074 | -0.154 | -0.004 | -0.287 | 0.007 |
| Roach | 1019 | 2 | 1 | 2 | -0.349 | 0.007 | -0.224 | 0.093 | 0.103 | 0.176 | 0.498 | 0.073 | 0.474 | -0.026 | 0.271 | -0.087 | 0.079 | -0.112 | -0.158 | -0.061 | -0.245 | -0.062 | -0.159 | -0.004 | -0.289 | 0.003 |
| Roach | 1020 | 2 | 1 | 2 | -0.353 | 0.010 | -0.230 | 0.094 | 0.089 | 0.172 | 0.489 | 0.074 | 0.486 | -0.028 | 0.276 | -0.090 | 0.072 | -0.112 | -0.160 | -0.056 | -0.231 | -0.072 | -0.149 | -0.005 | -0.289 | 0.013 |
| Roach | 1021 | 2 | 1 | 2 | -0.351 | 0.008 | -0.224 | 0.086 | 0.090 | 0.167 | 0.497 | 0.074 | 0.482 | -0.024 | 0.274 | -0.092 | 0.071 | -0.111 | -0.165 | -0.051 | -0.233 | -0.078 | -0.153 | 0.008 | -0.288 | 0.011 |
| Roach | 1022 | 2 | 1 | 2 | -0.364 | 0.013 | -0.232 | 0.091 | 0.094 | 0.159 | 0.487 | 0.070 | 0.487 | -0.025 | 0.277 | -0.083 | 0.076 | -0.110 | -0.161 | -0.048 | -0.217 | -0.079 | -0.157 | -0.002 | -0.290 | 0.015 |
| Roach | 1023 | 2 | 1 | 2 | -0.356 | 0.014 | -0.213 | 0.096 | 0.099 | 0.148 | 0.493 | 0.081 | 0.496 | -0.010 | 0.268 | -0.095 | 0.054 | -0.114 | -0.167 | -0.057 | -0.234 | -0.070 | -0.154 | -0.006 | -0.286 | 0.014 |
| Roach | 1024 | 2 | 1 | 2 | -0.366 | 0.002 | -0.228 | 0.100 | 0.062 | 0.181 | 0.502 | 0.076 | 0.491 | -0.041 | 0.264 | -0.083 | 0.056 | -0.118 | -0.150 | -0.056 | -0.223 | -0.082 | -0.134 | 0.011 | -0.275 | 0.010 |
| Roach | 1025 | 2 | 1 | 2 | -0.343 | 0.013 | -0.228 | 0.094 | 0.084 | 0.175 | 0.495 | 0.077 | 0.488 | -0.024 | 0.265 | -0.094 | 0.077 | -0.123 | -0.166 | -0.055 | -0.225 | -0.074 | -0.158 | -0.002 | -0.288 | 0.012 |
| Roach | 1026 | 2 | 1 | 2 | -0.363 | -0.009 | -0.252 | 0.085 | 0.086 | 0.197 | 0.512 | 0.039 | 0.471 | -0.050 | 0.268 | -0.054 | 0.080 | -0.084 | -0.154 | -0.052 | -0.210 | -0.075 | -0.149 | 0.005 | -0.289 | -0.002 |
| Roach | 1027 | 2 | 1 | 2 | -0.356 | 0.034 | -0.211 | 0.099 | 0.097 | 0.139 | 0.472 | 0.091 | 0.503 | 0.006 | 0.275 | -0.109 | 0.063 | -0.139 | -0.173 | -0.067 | -0.231 | -0.067 | -0.149 | -0.008 | -0.291 | 0.023 |
| Roach | 1028 | 2 | 1 | 2 | -0.354 | 0.004 | -0.217 | 0.090 | 0.079 | 0.156 | 0.499 | 0.076 | 0.496 | -0.025 | 0.268 | -0.089 | 0.062 | -0.106 | -0.166 | -0.052 | -0.235 | -0.072 | -0.144 | 0.006 | -0.287 | 0.012 |
| Roach | 1029 | 2 | 1 | 2 | -0.344 | -0.004 | -0.234 | 0.091 | 0.097 | 0.179 | 0.507 | 0.049 | 0.473 | -0.030 | 0.277 | -0.071 | 0.077 | -0.091 | -0.170 | -0.053 | -0.218 | -0.073 | -0.174 | 0.001 | -0.292 | 0.003 |
| Roach | 1030 | 2 | 1 | 2 | -0.348 | 0.000 | -0.235 | 0.093 | 0.114 | 0.170 | 0.486 | 0.081 | 0.485 | -0.023 | 0.272 | -0.096 | 0.063 | -0.124 | -0.164 | -0.051 | -0.225 | -0.076 | -0.156 | 0.010 | -0.290 | 0.014 |
| Roach | 1001 | 2 | 1 | 3 | -0.348 | 0.011 | -0.227 | 0.087 | 0.097 | 0.166 | 0.479 | 0.078 | 0.508 | -0.012 | 0.270 | -0.104 | 0.049 | -0.118 | -0.160 | -0.048 | -0.232 | -0.077 | -0.152 | -0.001 | -0.284 | 0.017 |
| Roach | 1002 | 2 | 1 | 3 | -0.358 | 0.016 | -0.226 | 0.096 | 0.091 | 0.158 | 0.483 | 0.082 | 0.495 | -0.012 | 0.277 | -0.102 | 0.058 | -0.123 | -0.163 | -0.056 | -0.235 | -0.069 | -0.140 | -0.006 | -0.282 | 0.015 |
| Roach | 1003 | 2 | 1 | 3 | -0.355 | -0.011 | -0.231 | 0.095 | 0.072 | 0.194 | 0.512 | 0.050 | 0.481 | -0.036 | 0.267 | -0.075 | 0.064 | -0.096 | -0.155 | -0.051 | -0.229 | -0.079 | -0.145 | 0.007 | -0.280 | 0.003 |
| Roach | 1004 | 2 | 1 | 3 | -0.355 | 0.010 | -0.232 | 0.089 | 0.077 | 0.173 | 0.492 | 0.071 | 0.487 | -0.033 | 0.275 | -0.086 | 0.082 | -0.108 | -0.165 | -0.052 | -0.230 | -0.073 | -0.149 | -0.001 | -0.282 | 0.012 |
| Roach | 1005 | 2 | 1 | 3 | -0.350 | -0.004 | -0.230 | 0.081 | 0.092 | 0.166 | 0.489 | 0.068 | 0.480 | -0.032 | 0.283 | -0.091 | 0.089 | -0.102 | -0.167 | -0.033 | -0.232 | -0.082 | -0.157 | 0.019 | -0.298 | 0.010 |
| Roach | 1006 | 2 | 1 | 3 | -0.360 | 0.016 | -0.219 | 0.101 | 0.084 | 0.166 | 0.489 | 0.083 | 0.487 | -0.021 | 0.276 | -0.104 | 0.069 | -0.114 | -0.160 | -0.054 | -0.229 | -0.071 | -0.152 | -0.013 | -0.284 | 0.012 |
| Roach | 1007 | 2 | 1 | 3 | -0.361 | -0.021 | -0.231 | 0.101 | 0.096 | 0.209 | 0.513 | 0.053 | 0.461 | -0.045 | 0.268 | -0.072 | 0.069 | -0.094 | -0.157 | -0.053 | -0.231 | -0.079 | -0.144 | 0.005 | -0.283 | -0.004 |
| Roach | 1008 | 2 | 1 | 3 | -0.354 | 0.009 | -0.224 | 0.100 | 0.076 | 0.178 | 0.487 | 0.080 | 0.497 | -0.032 | 0.272 | -0.095 | 0.060 | -0.117 | -0.159 | -0.053 | -0.218 | -0.072 | -0.151 | -0.010 | -0.287 | 0.012 |
| Roach | 1009 | 2 | 1 | 3 | -0.354 | 0.017 | -0.232 | 0.091 | 0.089 | 0.162 | 0.490 | 0.077 | 0.485 | -0.019 | 0.278 | -0.094 | 0.074 | -0.117 | -0.167 | -0.050 | -0.236 | -0.069 | -0.147 | -0.015 | -0.282 | 0.017 |
| Roach | 1010 | 2 | 1 | 3 | -0.358 | 0.027 | -0.217 | 0.104 | 0.092 | 0.165 | 0.468 | 0.088 | 0.498 | -0.020 | 0.273 | -0.102 | 0.081 | -0.126 | -0.170 | -0.058 | -0.230 | -0.079 | -0.147 | -0.016 | -0.289 | 0.017 |
| Roach | 1011 | 2 | 1 | 3 | -0.352 | 0.024 | -0.216 | 0.105 | 0.079 | 0.168 | 0.490 | 0.081 | 0.494 | -0.025 | 0.259 | -0.094 | 0.082 | -0.117 | -0.167 | -0.061 | -0.222 | -0.077 | -0.152 | -0.015 | -0.294 | 0.010 |
| Roach | 1012 | 2 | 1 | 3 | -0.357 | 0.021 | -0.218 | 0.095 | 0.095 | 0.154 | 0.479 | 0.086 | 0.503 | -0.007 | 0.269 | -0.106 | 0.062 | -0.122 | -0.170 | -0.055 | -0.237 | -0.068 | -0.144 | -0.018 | -0.281 | 0.020 |
| Roach | 1013 | 2 | 1 | 3 | -0.357 | 0.013 | -0.222 | 0.096 | 0.085 | 0.166 | 0.491 | 0.078 | 0.495 | -0.023 | 0.261 | -0.091 | 0.077 | -0.117 | -0.163 | -0.055 | -0.236 | -0.069 | -0.151 | -0.012 | -0.280 | 0.014 |
| Roach | 1014 | 2 | 1 | 3 | -0.361 | 0.022 | -0.233 | 0.092 | 0.092 | 0.148 | 0.486 | 0.088 | 0.503 | -0.005 | 0.263 | -0.110 | 0.053 | -0.124 | -0.156 | -0.049 | -0.231 | -0.072 | -0.137 | -0.012 | -0.278 | 0.023 |
| Roach | 1015 | 2 | 1 | 3 | -0.360 | -0.004 | -0.229 | 0.100 | 0.079 | 0.176 | 0.511 | 0.067 | 0.490 | -0.031 | 0.259 | -0.083 | 0.046 | -0.101 | -0.147 | -0.054 | -0.228 | -0.078 | -0.145 | 0.001 | -0.276 | 0.007 |
| Roach | 1016 | 2 | 1 | 3 | -0.348 | 0.005 | -0.224 | 0.098 | 0.092 | 0.189 | 0.498 | 0.079 | 0.479 | -0.031 | 0.262 | -0.093 | 0.074 | -0.123 | -0.166 | -0.062 | -0.227 | -0.070 | -0.155 | 0.001 | -0.285 | 0.007 |
| Roach | 1017 | 2 | 1 | 3 | -0.355 | 0.003 | -0.222 | 0.098 | 0.084 | 0.177 | 0.492 | 0.064 | 0.490 | -0.026 | 0.262 | -0.080 | 0.084 | -0.117 | -0.165 | -0.058 | -0.229 | -0.067 | -0.150 | -0.003 | -0.292 | 0.010 |
| Roach | 1018 | 2 | 1 | 3 | -0.347 | 0.002 | -0.216 | 0.101 | 0.072 | 0.177 | 0.507 | 0.070 | 0.489 | -0.029 | 0.274 | -0.085 | 0.047 | -0.110 | -0.162 | -0.055 | -0.221 | -0.072 | -0.156 | -0.006 | -0.286 | 0.006 |
| Roach | 1019 | 2 | 1 | 3 | -0.352 | 0.011 | -0.212 | 0.103 | 0.099 | 0.174 | 0.497 | 0.074 | 0.478 | -0.024 | 0.270 | -0.086 | 0.075 | -0.111 | -0.172 | -0.064 | -0.227 | -0.071 | -0.160 | -0.010 | -0.295 | 0.005 |
| Roach | 1020 | 2 | 1 | 3 | -0.355 | 0.013 | -0.230 | 0.097 | 0.091 | 0.175 | 0.492 | 0.077 | 0.480 | -0.028 | 0.279 | -0.090 | 0.068 | -0.114 | -0.156 | -0.058 | -0.229 | -0.071 | -0.149 | -0.014 | -0.290 | 0.014 |
| Roach | 1021 | 2 | 1 | 3 | -0.349 | 0.011 | -0.234 | 0.087 | 0.094 | 0.167 | 0.500 | 0.076 | 0.483 | -0.024 | 0.269 | -0.091 | 0.067 | -0.111 | -0.163 | -0.053 | -0.228 | -0.074 | -0.152 | 0.001 | -0.287 | 0.011 |
| Roach | 1022 | 2 | 1 | 3 | -0.362 | 0.012 | -0.224 | 0.096 | 0.085 | 0.159 | 0.487 | 0.071 | 0.487 | -0.026 | 0.285 | -0.080 | 0.068 | -0.114 | -0.162 | -0.049 | -0.222 | -0.079 | -0.151 | -0.006 | -0.291 | 0.016 |
| Roach | 1023 | 2 | 1 | 3 | -0.354 | 0.017 | -0.219 | 0.094 | 0.092 | 0.147 | 0.495 | 0.078 | 0.499 | -0.009 | 0.267 | -0.093 | 0.053 | -0.115 | -0.166 | -0.060 | -0.231 | -0.071 | -0.152 | -0.001 | -0.283 | 0.014 |
| Roach | 1024 | 2 | 1 | 3 | -0.365 | 0.002 | -0.230 | 0.106 | 0.056 | 0.180 | 0.505 | 0.075 | 0.491 | -0.039 | 0.266 | -0.085 | 0.053 | -0.114 | -0.148 | -0.058 | -0.222 | -0.077 | -0.131 | -0.004 | -0.275 | 0.015 |
| Roach | 1025 | 2 | 1 | 3 | -0.350 | 0.013 | -0.222 | 0.098 | 0.089 | 0.174 | 0.497 | 0.078 | 0.486 | -0.023 | 0.264 | -0.095 | 0.068 | -0.118 | -0.167 | -0.054 | -0.221 | -0.075 | -0.153 | -0.009 | -0.292 | 0.010 |
| Roach | 1026 | 2 | 1 | 3 | -0.362 | -0.006 | -0.242 | 0.093 | 0.093 | 0.198 | 0.509 | 0.047 | 0.470 | -0.049 | 0.265 | -0.055 | 0.081 | -0.088 | -0.158 | -0.056 | -0.220 | -0.074 | -0.144 | -0.006 | -0.291 | -0.003 |
| Roach | 1027 | 2 | 1 | 3 | -0.354 | 0.036 | -0.210 | 0.100 | 0.097 | 0.143 | 0.471 | 0.090 | 0.507 | 0.007 | 0.276 | -0.111 | 0.055 | -0.139 | -0.170 | -0.063 | -0.231 | -0.070 | -0.148 | -0.015 | -0.291 | 0.022 |
| Roach | 1028 | 2 | 1 | 3 | -0.353 | 0.001 | -0.220 | 0.091 | 0.077 | 0.158 | 0.495 | 0.073 | 0.502 | -0.027 | 0.270 | -0.086 | 0.061 | -0.104 | -0.168 | -0.049 | -0.235 | -0.070 | -0.146 | 0.003 | -0.281 | 0.011 |
| Roach | 1029 | 2 | 1 | 3 | -0.346 | -0.005 | -0.228 | 0.096 | 0.095 | 0.179 | 0.506 | 0.056 | 0.474 | -0.032 | 0.280 | -0.073 | 0.069 | -0.095 | -0.173 | -0.053 | -0.217 | -0.074 | -0.164 | -0.001 | -0.294 | 0.004 |
| Roach | 1030 | 2 | 1 | 3 | -0.354 | -0.002 | -0.220 | 0.100 | 0.115 | 0.171 | 0.487 | 0.082 | 0.482 | -0.017 | 0.269 | -0.101 | 0.063 | -0.125 | -0.170 | -0.047 | -0.222 | -0.081 | -0.156 | 0.007 | -0.294 | 0.013 |
| Roach | 1101 | 2 | 2 | 1 | -0.349 | 0.003 | -0.239 | 0.077 | 0.076 | 0.177 | 0.505 | 0.057 | 0.488 | -0.041 | 0.268 | -0.075 | 0.077 | -0.088 | -0.156 | -0.044 | -0.215 | -0.074 | -0.159 | -0.004 | -0.296 | 0.012 |
| Roach | 1102 | 2 | 2 | 1 | -0.365 | 0.021 | -0.224 | 0.090 | 0.076 | 0.138 | 0.493 | 0.076 | 0.498 | -0.018 | 0.269 | -0.084 | 0.074 | -0.116 | -0.153 | -0.051 | -0.234 | -0.069 | -0.149 | -0.008 | -0.284 | 0.020 |
| Roach | 1103 | 2 | 2 | 1 | -0.358 | 0.018 | -0.216 | 0.093 | 0.083 | 0.145 | 0.484 | 0.077 | 0.487 | -0.025 | 0.293 | -0.083 | 0.074 | -0.112 | -0.161 | -0.057 | -0.228 | -0.074 | -0.159 | -0.004 | -0.298 | 0.022 |
| Roach | 1104 | 2 | 2 | 1 | -0.358 | 0.014 | -0.220 | 0.092 | 0.077 | 0.161 | 0.495 | 0.070 | 0.495 | -0.026 | 0.272 | -0.080 | 0.061 | -0.109 | -0.157 | -0.057 | -0.225 | -0.082 | -0.149 | -0.002 | -0.289 | 0.019 |
| Roach | 1105 | 2 | 2 | 1 | -0.367 | 0.013 | -0.219 | 0.092 | 0.082 | 0.162 | 0.490 | 0.072 | 0.491 | -0.029 | 0.268 | -0.081 | 0.079 | -0.103 | -0.163 | -0.065 | -0.239 | -0.078 | -0.140 | -0.005 | -0.284 | 0.022 |
| Roach | 1106 | 2 | 2 | 1 | -0.354 | 0.022 | -0.219 | 0.086 | 0.079 | 0.150 | 0.482 | 0.078 | 0.512 | -0.013 | 0.272 | -0.098 | 0.054 | -0.114 | -0.159 | -0.051 | -0.228 | -0.078 | -0.155 | -0.003 | -0.285 | 0.021 |
| Roach | 1107 | 2 | 2 | 1 | -0.361 | 0.034 | -0.215 | 0.088 | 0.084 | 0.136 | 0.488 | 0.087 | 0.497 | -0.008 | 0.272 | -0.097 | 0.067 | -0.120 | -0.161 | -0.067 | -0.238 | -0.075 | -0.148 | -0.005 | -0.284 | 0.027 |
| Roach | 1108 | 2 | 2 | 1 | -0.360 | 0.015 | -0.230 | 0.082 | 0.082 | 0.150 | 0.499 | 0.068 | 0.486 | -0.021 | 0.278 | -0.078 | 0.069 | -0.113 | -0.151 | -0.049 | -0.232 | -0.071 | -0.153 | 0.002 | -0.289 | 0.015 |
| Roach | 1109 | 2 | 2 | 1 | -0.367 | 0.008 | -0.253 | 0.094 | 0.075 | 0.170 | 0.501 | 0.068 | 0.484 | -0.026 | 0.267 | -0.090 | 0.068 | -0.115 | -0.144 | -0.052 | -0.223 | -0.073 | -0.131 | 0.003 | -0.276 | 0.013 |
| Roach | 1110 | 2 | 2 | 1 | -0.358 | 0.018 | -0.230 | 0.087 | 0.074 | 0.141 | 0.501 | 0.077 | 0.498 | -0.019 | 0.269 | -0.085 | 0.058 | -0.115 | -0.162 | -0.052 | -0.224 | -0.069 | -0.144 | 0.001 | -0.283 | 0.016 |
| Roach | 1111 | 2 | 2 | 1 | -0.359 | 0.008 | -0.219 | 0.093 | 0.064 | 0.151 | 0.507 | 0.077 | 0.492 | -0.024 | 0.273 | -0.090 | 0.056 | -0.108 | -0.162 | -0.058 | -0.232 | -0.070 | -0.142 | 0.003 | -0.279 | 0.017 |
| Roach | 1112 | 2 | 2 | 1 | -0.367 | 0.017 | -0.231 | 0.089 | 0.083 | 0.150 | 0.506 | 0.074 | 0.481 | -0.021 | 0.271 | -0.086 | 0.064 | -0.105 | -0.156 | -0.056 | -0.229 | -0.077 | -0.141 | -0.004 | -0.282 | 0.018 |
| Roach | 1113 | 2 | 2 | 1 | -0.370 | 0.017 | -0.215 | 0.093 | 0.082 | 0.158 | 0.498 | 0.078 | 0.491 | -0.026 | 0.264 | -0.079 | 0.064 | -0.115 | -0.156 | -0.061 | -0.239 | -0.077 | -0.141 | -0.003 | -0.277 | 0.015 |
| Roach | 1114 | 2 | 2 | 1 | -0.364 | -0.004 | -0.228 | 0.082 | 0.084 | 0.181 | 0.504 | 0.067 | 0.484 | -0.043 | 0.267 | -0.072 | 0.061 | -0.099 | -0.147 | -0.052 | -0.236 | -0.076 | -0.140 | 0.003 | -0.286 | 0.013 |
| Roach | 1115 | 2 | 2 | 1 | -0.361 | -0.002 | -0.221 | 0.095 | 0.079 | 0.178 | 0.499 | 0.070 | 0.496 | -0.033 | 0.261 | -0.086 | 0.054 | -0.102 | -0.154 | -0.056 | -0.223 | -0.072 | -0.146 | 0.000 | -0.286 | 0.010 |
| Roach | 1116 | 2 | 2 | 1 | -0.351 | 0.023 | -0.221 | 0.082 | 0.062 | 0.140 | 0.498 | 0.082 | 0.500 | -0.017 | 0.272 | -0.098 | 0.069 | -0.112 | -0.159 | -0.048 | -0.231 | -0.074 | -0.150 | -0.002 | -0.288 | 0.025 |
| Roach | 1117 | 2 | 2 | 1 | -0.353 | 0.019 | -0.212 | 0.089 | 0.076 | 0.158 | 0.496 | 0.076 | 0.477 | -0.024 | 0.292 | -0.084 | 0.072 | -0.117 | -0.166 | -0.058 | -0.229 | -0.078 | -0.157 | -0.003 | -0.295 | 0.023 |
| Roach | 1118 | 2 | 2 | 1 | -0.350 | -0.012 | -0.232 | 0.086 | 0.092 | 0.161 | 0.499 | 0.068 | 0.488 | -0.033 | 0.284 | -0.080 | 0.049 | -0.100 | -0.165 | -0.037 | -0.214 | -0.080 | -0.154 | 0.013 | -0.296 | 0.015 |
| Roach | 1119 | 2 | 2 | 1 | -0.361 | 0.036 | -0.222 | 0.090 | 0.085 | 0.143 | 0.489 | 0.086 | 0.496 | -0.013 | 0.271 | -0.090 | 0.068 | -0.126 | -0.168 | -0.068 | -0.225 | -0.070 | -0.149 | -0.008 | -0.284 | 0.020 |
| Roach | 1120 | 2 | 2 | 1 | -0.365 | 0.011 | -0.212 | 0.095 | 0.049 | 0.141 | 0.504 | 0.073 | 0.499 | -0.025 | 0.280 | -0.086 | 0.051 | -0.102 | -0.151 | -0.054 | -0.229 | -0.073 | -0.141 | 0.002 | -0.286 | 0.017 |
| Roach | 1121 | 2 | 2 | 1 | -0.370 | -0.006 | -0.235 | 0.086 | 0.087 | 0.162 | 0.497 | 0.059 | 0.485 | -0.038 | 0.275 | -0.076 | 0.072 | -0.083 | -0.156 | -0.047 | -0.219 | -0.078 | -0.143 | 0.008 | -0.294 | 0.013 |
| Roach | 1122 | 2 | 2 | 1 | -0.358 | 0.020 | -0.201 | 0.090 | 0.080 | 0.137 | 0.469 | 0.084 | 0.508 | -0.004 | 0.293 | -0.106 | 0.059 | -0.128 | -0.162 | -0.045 | -0.240 | -0.076 | -0.157 | 0.004 | -0.291 | 0.025 |
| Roach | 1123 | 2 | 2 | 1 | -0.351 | 0.017 | -0.220 | 0.089 | 0.093 | 0.132 | 0.485 | 0.091 | 0.499 | -0.009 | 0.269 | -0.108 | 0.074 | -0.116 | -0.168 | -0.049 | -0.238 | -0.074 | -0.149 | 0.003 | -0.294 | 0.025 |
| Roach | 1124 | 2 | 2 | 1 | -0.352 | -0.007 | -0.229 | 0.082 | 0.079 | 0.163 | 0.497 | 0.060 | 0.497 | -0.037 | 0.276 | -0.069 | 0.057 | -0.097 | -0.158 | -0.041 | -0.219 | -0.078 | -0.150 | 0.009 | -0.298 | 0.015 |
| Roach | 1125 | 2 | 2 | 1 | -0.364 | 0.019 | -0.213 | 0.086 | 0.073 | 0.153 | 0.498 | 0.075 | 0.503 | -0.024 | 0.268 | -0.081 | 0.045 | -0.105 | -0.150 | -0.063 | -0.231 | -0.084 | -0.145 | 0.003 | -0.284 | 0.021 |
| Roach | 1126 | 2 | 2 | 1 | -0.378 | 0.020 | -0.218 | 0.095 | 0.069 | 0.137 | 0.497 | 0.079 | 0.500 | -0.017 | 0.256 | -0.094 | 0.075 | -0.105 | -0.158 | -0.058 | -0.225 | -0.080 | -0.130 | 0.003 | -0.287 | 0.021 |
| Roach | 1127 | 2 | 2 | 1 | -0.357 | 0.018 | -0.221 | 0.092 | 0.074 | 0.142 | 0.478 | 0.079 | 0.499 | -0.015 | 0.284 | -0.098 | 0.079 | -0.124 | -0.161 | -0.050 | -0.223 | -0.071 | -0.156 | 0.002 | -0.295 | 0.024 |
| Roach | 1128 | 2 | 2 | 1 | -0.369 | 0.005 | -0.232 | 0.087 | 0.068 | 0.177 | 0.496 | 0.059 | 0.487 | -0.042 | 0.265 | -0.068 | 0.096 | -0.100 | -0.163 | -0.057 | -0.233 | -0.076 | -0.138 | -0.005 | -0.279 | 0.019 |
| Roach | 1129 | 2 | 2 | 1 | -0.363 | -0.011 | -0.243 | 0.083 | 0.080 | 0.189 | 0.515 | 0.043 | 0.480 | -0.053 | 0.268 | -0.058 | 0.064 | -0.070 | -0.150 | -0.045 | -0.209 | -0.081 | -0.148 | 0.002 | -0.294 | 0.000 |
| Roach | 1130 | 2 | 2 | 1 | -0.359 | -0.023 | -0.240 | 0.090 | 0.088 | 0.188 | 0.512 | 0.032 | 0.469 | -0.050 | 0.273 | -0.050 | 0.091 | -0.070 | -0.154 | -0.043 | -0.221 | -0.079 | -0.161 | 0.000 | -0.297 | 0.003 |
| Roach | 1101 | 2 | 2 | 2 | -0.350 | 0.002 | -0.225 | 0.082 | 0.074 | 0.176 | 0.506 | 0.058 | 0.487 | -0.038 | 0.268 | -0.074 | 0.075 | -0.091 | -0.154 | -0.049 | -0.221 | -0.076 | -0.164 | 0.000 | -0.296 | 0.009 |
| Roach | 1102 | 2 | 2 | 2 | -0.368 | 0.019 | -0.222 | 0.091 | 0.071 | 0.138 | 0.493 | 0.075 | 0.504 | -0.018 | 0.263 | -0.085 | 0.070 | -0.113 | -0.153 | -0.054 | -0.225 | -0.071 | -0.145 | -0.003 | -0.289 | 0.019 |
| Roach | 1103 | 2 | 2 | 2 | -0.359 | 0.017 | -0.217 | 0.089 | 0.083 | 0.145 | 0.483 | 0.074 | 0.486 | -0.025 | 0.294 | -0.083 | 0.078 | -0.110 | -0.162 | -0.056 | -0.227 | -0.075 | -0.160 | 0.003 | -0.299 | 0.021 |
| Roach | 1104 | 2 | 2 | 2 | -0.361 | 0.014 | -0.226 | 0.092 | 0.087 | 0.164 | 0.496 | 0.068 | 0.485 | -0.022 | 0.274 | -0.083 | 0.066 | -0.107 | -0.159 | -0.058 | -0.224 | -0.083 | -0.146 | -0.006 | -0.292 | 0.021 |
| Roach | 1105 | 2 | 2 | 2 | -0.370 | 0.012 | -0.216 | 0.090 | 0.079 | 0.161 | 0.485 | 0.074 | 0.493 | -0.031 | 0.271 | -0.083 | 0.081 | -0.104 | -0.156 | -0.063 | -0.237 | -0.078 | -0.142 | 0.000 | -0.288 | 0.022 |
| Roach | 1106 | 2 | 2 | 2 | -0.353 | 0.024 | -0.218 | 0.087 | 0.085 | 0.154 | 0.480 | 0.078 | 0.519 | -0.009 | 0.258 | -0.097 | 0.054 | -0.122 | -0.158 | -0.057 | -0.225 | -0.073 | -0.153 | -0.008 | -0.290 | 0.022 |
| Roach | 1107 | 2 | 2 | 2 | -0.364 | 0.032 | -0.203 | 0.092 | 0.073 | 0.134 | 0.481 | 0.086 | 0.512 | -0.008 | 0.270 | -0.096 | 0.065 | -0.124 | -0.166 | -0.064 | -0.236 | -0.072 | -0.147 | -0.007 | -0.284 | 0.026 |
| Roach | 1108 | 2 | 2 | 2 | -0.363 | 0.017 | -0.228 | 0.087 | 0.079 | 0.150 | 0.488 | 0.069 | 0.496 | -0.021 | 0.276 | -0.080 | 0.073 | -0.114 | -0.158 | -0.055 | -0.227 | -0.070 | -0.146 | 0.002 | -0.290 | 0.016 |
| Roach | 1109 | 2 | 2 | 2 | -0.368 | 0.007 | -0.242 | 0.096 | 0.068 | 0.170 | 0.493 | 0.071 | 0.494 | -0.029 | 0.269 | -0.091 | 0.064 | -0.118 | -0.144 | -0.052 | -0.220 | -0.075 | -0.137 | 0.009 | -0.277 | 0.013 |
| Roach | 1110 | 2 | 2 | 2 | -0.361 | 0.023 | -0.232 | 0.085 | 0.083 | 0.144 | 0.497 | 0.079 | 0.491 | -0.021 | 0.271 | -0.085 | 0.072 | -0.116 | -0.158 | -0.059 | -0.222 | -0.067 | -0.157 | 0.001 | -0.285 | 0.016 |
| Roach | 1111 | 2 | 2 | 2 | -0.364 | 0.008 | -0.215 | 0.091 | 0.070 | 0.152 | 0.501 | 0.078 | 0.498 | -0.024 | 0.267 | -0.090 | 0.056 | -0.109 | -0.156 | -0.054 | -0.232 | -0.068 | -0.144 | 0.001 | -0.281 | 0.014 |
| Roach | 1112 | 2 | 2 | 2 | -0.368 | 0.013 | -0.223 | 0.090 | 0.086 | 0.151 | 0.497 | 0.074 | 0.494 | -0.023 | 0.265 | -0.085 | 0.063 | -0.102 | -0.156 | -0.052 | -0.231 | -0.076 | -0.140 | -0.006 | -0.287 | 0.016 |
| Roach | 1113 | 2 | 2 | 2 | -0.374 | 0.020 | -0.217 | 0.094 | 0.084 | 0.160 | 0.492 | 0.074 | 0.486 | -0.026 | 0.271 | -0.077 | 0.071 | -0.119 | -0.155 | -0.064 | -0.229 | -0.078 | -0.143 | 0.003 | -0.285 | 0.013 |
| Roach | 1114 | 2 | 2 | 2 | -0.366 | 0.001 | -0.227 | 0.085 | 0.080 | 0.179 | 0.500 | 0.063 | 0.486 | -0.035 | 0.272 | -0.076 | 0.058 | -0.107 | -0.150 | -0.052 | -0.220 | -0.079 | -0.145 | 0.006 | -0.289 | 0.014 |
| Roach | 1115 | 2 | 2 | 2 | -0.361 | 0.003 | -0.226 | 0.093 | 0.077 | 0.179 | 0.504 | 0.068 | 0.490 | -0.034 | 0.260 | -0.089 | 0.065 | -0.097 | -0.158 | -0.059 | -0.217 | -0.074 | -0.148 | 0.001 | -0.285 | 0.008 |
| Roach | 1116 | 2 | 2 | 2 | -0.352 | 0.021 | -0.224 | 0.079 | 0.093 | 0.146 | 0.490 | 0.083 | 0.500 | -0.008 | 0.265 | -0.099 | 0.062 | -0.125 | -0.157 | -0.048 | -0.230 | -0.074 | -0.159 | 0.003 | -0.288 | 0.024 |
| Roach | 1117 | 2 | 2 | 2 | -0.348 | 0.023 | -0.210 | 0.086 | 0.077 | 0.158 | 0.493 | 0.079 | 0.488 | -0.032 | 0.285 | -0.078 | 0.072 | -0.112 | -0.169 | -0.065 | -0.231 | -0.076 | -0.162 | -0.005 | -0.294 | 0.021 |
| Roach | 1118 | 2 | 2 | 2 | -0.349 | -0.001 | -0.241 | 0.076 | 0.090 | 0.159 | 0.500 | 0.064 | 0.480 | -0.031 | 0.291 | -0.080 | 0.061 | -0.097 | -0.162 | -0.037 | -0.217 | -0.077 | -0.155 | 0.007 | -0.298 | 0.017 |
| Roach | 1119 | 2 | 2 | 2 | -0.359 | 0.035 | -0.227 | 0.090 | 0.088 | 0.146 | 0.491 | 0.081 | 0.491 | -0.014 | 0.274 | -0.087 | 0.067 | -0.122 | -0.172 | -0.067 | -0.218 | -0.075 | -0.151 | -0.006 | -0.286 | 0.019 |
| Roach | 1120 | 2 | 2 | 2 | -0.367 | 0.009 | -0.216 | 0.093 | 0.051 | 0.142 | 0.508 | 0.071 | 0.499 | -0.025 | 0.275 | -0.086 | 0.051 | -0.099 | -0.153 | -0.051 | -0.220 | -0.073 | -0.140 | 0.002 | -0.288 | 0.016 |
| Roach | 1121 | 2 | 2 | 2 | -0.368 | -0.002 | -0.243 | 0.083 | 0.085 | 0.162 | 0.495 | 0.059 | 0.490 | -0.039 | 0.269 | -0.075 | 0.081 | -0.084 | -0.149 | -0.048 | -0.219 | -0.071 | -0.148 | 0.001 | -0.293 | 0.013 |
| Roach | 1122 | 2 | 2 | 2 | -0.354 | 0.020 | -0.200 | 0.091 | 0.079 | 0.134 | 0.472 | 0.083 | 0.515 | -0.003 | 0.285 | -0.104 | 0.056 | -0.124 | -0.162 | -0.046 | -0.236 | -0.077 | -0.166 | -0.002 | -0.290 | 0.026 |
| Roach | 1123 | 2 | 2 | 2 | -0.352 | 0.014 | -0.211 | 0.093 | 0.092 | 0.132 | 0.479 | 0.090 | 0.507 | -0.009 | 0.267 | -0.105 | 0.075 | -0.120 | -0.171 | -0.051 | -0.233 | -0.074 | -0.159 | 0.003 | -0.295 | 0.027 |
| Roach | 1124 | 2 | 2 | 2 | -0.353 | -0.011 | -0.237 | 0.079 | 0.095 | 0.167 | 0.498 | 0.058 | 0.484 | -0.035 | 0.279 | -0.070 | 0.066 | -0.098 | -0.157 | -0.040 | -0.218 | -0.077 | -0.156 | 0.009 | -0.300 | 0.018 |
| Roach | 1125 | 2 | 2 | 2 | -0.364 | 0.022 | -0.218 | 0.083 | 0.079 | 0.153 | 0.505 | 0.072 | 0.489 | -0.021 | 0.269 | -0.081 | 0.062 | -0.103 | -0.147 | -0.058 | -0.237 | -0.080 | -0.153 | -0.008 | -0.285 | 0.021 |
| Roach | 1126 | 2 | 2 | 2 | -0.377 | 0.024 | -0.239 | 0.091 | 0.070 | 0.136 | 0.493 | 0.074 | 0.494 | -0.018 | 0.261 | -0.094 | 0.093 | -0.103 | -0.150 | -0.055 | -0.227 | -0.075 | -0.134 | -0.005 | -0.283 | 0.026 |
| Roach | 1127 | 2 | 2 | 2 | -0.357 | 0.018 | -0.218 | 0.091 | 0.067 | 0.141 | 0.478 | 0.081 | 0.499 | -0.017 | 0.281 | -0.100 | 0.087 | -0.125 | -0.161 | -0.052 | -0.225 | -0.071 | -0.158 | 0.009 | -0.294 | 0.025 |
| Roach | 1128 | 2 | 2 | 2 | -0.373 | 0.010 | -0.237 | 0.088 | 0.070 | 0.178 | 0.493 | 0.059 | 0.481 | -0.044 | 0.269 | -0.070 | 0.102 | -0.102 | -0.153 | -0.058 | -0.222 | -0.080 | -0.147 | 0.001 | -0.283 | 0.016 |
| Roach | 1129 | 2 | 2 | 2 | -0.368 | -0.012 | -0.241 | 0.091 | 0.071 | 0.190 | 0.509 | 0.042 | 0.472 | -0.054 | 0.269 | -0.064 | 0.097 | -0.073 | -0.152 | -0.048 | -0.207 | -0.081 | -0.151 | 0.006 | -0.298 | 0.003 |
| Roach | 1130 | 2 | 2 | 2 | -0.363 | -0.020 | -0.238 | 0.089 | 0.082 | 0.191 | 0.516 | 0.034 | 0.464 | -0.049 | 0.274 | -0.054 | 0.083 | -0.076 | -0.145 | -0.045 | -0.216 | -0.081 | -0.157 | 0.011 | -0.303 | 0.001 |
| Roach | 1101 | 2 | 2 | 3 | -0.352 | 0.001 | -0.227 | 0.082 | 0.075 | 0.173 | 0.510 | 0.057 | 0.481 | -0.037 | 0.269 | -0.077 | 0.081 | -0.086 | -0.158 | -0.050 | -0.217 | -0.074 | -0.163 | 0.003 | -0.299 | 0.008 |
| Roach | 1102 | 2 | 2 | 3 | -0.368 | 0.021 | -0.216 | 0.092 | 0.075 | 0.140 | 0.490 | 0.076 | 0.500 | -0.014 | 0.272 | -0.086 | 0.066 | -0.120 | -0.155 | -0.053 | -0.234 | -0.069 | -0.144 | -0.005 | -0.287 | 0.018 |
| Roach | 1103 | 2 | 2 | 3 | -0.361 | 0.021 | -0.206 | 0.095 | 0.086 | 0.148 | 0.490 | 0.080 | 0.480 | -0.021 | 0.289 | -0.086 | 0.072 | -0.115 | -0.164 | -0.057 | -0.226 | -0.077 | -0.158 | -0.009 | -0.301 | 0.021 |
| Roach | 1104 | 2 | 2 | 3 | -0.359 | 0.018 | -0.221 | 0.096 | 0.083 | 0.161 | 0.496 | 0.068 | 0.489 | -0.021 | 0.274 | -0.081 | 0.062 | -0.111 | -0.159 | -0.058 | -0.222 | -0.083 | -0.153 | -0.008 | -0.290 | 0.018 |
| Roach | 1105 | 2 | 2 | 3 | -0.373 | 0.014 | -0.216 | 0.092 | 0.089 | 0.163 | 0.487 | 0.072 | 0.487 | -0.027 | 0.271 | -0.083 | 0.079 | -0.101 | -0.158 | -0.064 | -0.242 | -0.078 | -0.140 | -0.010 | -0.285 | 0.021 |
| Roach | 1106 | 2 | 2 | 3 | -0.358 | 0.024 | -0.217 | 0.091 | 0.078 | 0.156 | 0.480 | 0.076 | 0.509 | -0.009 | 0.268 | -0.101 | 0.065 | -0.120 | -0.155 | -0.058 | -0.225 | -0.071 | -0.157 | -0.010 | -0.288 | 0.023 |
| Roach | 1107 | 2 | 2 | 3 | -0.363 | 0.037 | -0.205 | 0.094 | 0.083 | 0.135 | 0.490 | 0.091 | 0.492 | -0.005 | 0.281 | -0.095 | 0.056 | -0.128 | -0.163 | -0.066 | -0.239 | -0.075 | -0.147 | -0.014 | -0.284 | 0.026 |
| Roach | 1108 | 2 | 2 | 3 | -0.363 | 0.017 | -0.224 | 0.085 | 0.082 | 0.152 | 0.498 | 0.071 | 0.493 | -0.024 | 0.269 | -0.079 | 0.068 | -0.109 | -0.159 | -0.049 | -0.225 | -0.073 | -0.150 | -0.005 | -0.289 | 0.012 |
| Roach | 1109 | 2 | 2 | 3 | -0.365 | 0.005 | -0.241 | 0.100 | 0.072 | 0.170 | 0.492 | 0.071 | 0.491 | -0.033 | 0.273 | -0.081 | 0.061 | -0.121 | -0.140 | -0.052 | -0.230 | -0.073 | -0.133 | 0.002 | -0.281 | 0.012 |
| Roach | 1110 | 2 | 2 | 3 | -0.364 | 0.021 | -0.224 | 0.088 | 0.081 | 0.148 | 0.491 | 0.076 | 0.493 | -0.021 | 0.275 | -0.084 | 0.073 | -0.113 | -0.169 | -0.058 | -0.219 | -0.067 | -0.152 | -0.007 | -0.287 | 0.018 |
| Roach | 1111 | 2 | 2 | 3 | -0.362 | 0.008 | -0.209 | 0.098 | 0.074 | 0.153 | 0.503 | 0.077 | 0.494 | -0.021 | 0.270 | -0.086 | 0.051 | -0.113 | -0.160 | -0.058 | -0.233 | -0.068 | -0.146 | -0.004 | -0.283 | 0.014 |
| Roach | 1112 | 2 | 2 | 3 | -0.365 | 0.021 | -0.219 | 0.092 | 0.083 | 0.150 | 0.497 | 0.071 | 0.484 | -0.020 | 0.277 | -0.082 | 0.072 | -0.106 | -0.162 | -0.057 | -0.238 | -0.077 | -0.142 | -0.007 | -0.287 | 0.014 |
| Roach | 1113 | 2 | 2 | 3 | -0.372 | 0.020 | -0.218 | 0.093 | 0.082 | 0.161 | 0.490 | 0.077 | 0.495 | -0.028 | 0.270 | -0.079 | 0.060 | -0.116 | -0.158 | -0.059 | -0.226 | -0.077 | -0.140 | -0.006 | -0.283 | 0.014 |
| Roach | 1114 | 2 | 2 | 3 | -0.365 | -0.002 | -0.220 | 0.091 | 0.076 | 0.181 | 0.504 | 0.062 | 0.486 | -0.037 | 0.269 | -0.071 | 0.058 | -0.104 | -0.147 | -0.053 | -0.227 | -0.077 | -0.143 | -0.002 | -0.290 | 0.014 |
| Roach | 1115 | 2 | 2 | 3 | -0.363 | 0.000 | -0.226 | 0.093 | 0.082 | 0.178 | 0.502 | 0.065 | 0.489 | -0.028 | 0.260 | -0.085 | 0.066 | -0.103 | -0.157 | -0.056 | -0.223 | -0.071 | -0.145 | -0.001 | -0.285 | 0.009 |
| Roach | 1116 | 2 | 2 | 3 | -0.355 | 0.016 | -0.213 | 0.087 | 0.098 | 0.147 | 0.492 | 0.082 | 0.489 | -0.008 | 0.271 | -0.099 | 0.063 | -0.121 | -0.158 | -0.054 | -0.237 | -0.077 | -0.157 | 0.003 | -0.293 | 0.022 |
| Roach | 1117 | 2 | 2 | 3 | -0.350 | 0.026 | -0.216 | 0.087 | 0.086 | 0.161 | 0.489 | 0.074 | 0.484 | -0.023 | 0.286 | -0.077 | 0.073 | -0.120 | -0.163 | -0.064 | -0.232 | -0.077 | -0.165 | -0.012 | -0.292 | 0.024 |
| Roach | 1118 | 2 | 2 | 3 | -0.351 | -0.014 | -0.228 | 0.091 | 0.090 | 0.156 | 0.494 | 0.066 | 0.492 | -0.034 | 0.280 | -0.076 | 0.061 | -0.100 | -0.162 | -0.035 | -0.218 | -0.076 | -0.160 | 0.006 | -0.298 | 0.016 |
| Roach | 1119 | 2 | 2 | 3 | -0.363 | 0.031 | -0.218 | 0.093 | 0.095 | 0.147 | 0.493 | 0.085 | 0.493 | -0.013 | 0.268 | -0.085 | 0.056 | -0.129 | -0.165 | -0.065 | -0.222 | -0.072 | -0.150 | -0.011 | -0.286 | 0.018 |
| Roach | 1120 | 2 | 2 | 3 | -0.366 | 0.011 | -0.213 | 0.096 | 0.062 | 0.143 | 0.503 | 0.071 | 0.501 | -0.021 | 0.271 | -0.083 | 0.054 | -0.105 | -0.156 | -0.055 | -0.224 | -0.070 | -0.143 | -0.004 | -0.288 | 0.016 |
| Roach | 1121 | 2 | 2 | 3 | -0.371 | -0.006 | -0.230 | 0.087 | 0.088 | 0.165 | 0.494 | 0.056 | 0.487 | -0.038 | 0.277 | -0.074 | 0.073 | -0.081 | -0.153 | -0.046 | -0.218 | -0.078 | -0.154 | 0.005 | -0.294 | 0.010 |
| Roach | 1122 | 2 | 2 | 3 | -0.350 | 0.021 | -0.204 | 0.090 | 0.081 | 0.133 | 0.481 | 0.089 | 0.506 | -0.005 | 0.285 | -0.105 | 0.054 | -0.126 | -0.165 | -0.046 | -0.233 | -0.073 | -0.168 | -0.006 | -0.288 | 0.027 |
| Roach | 1123 | 2 | 2 | 3 | -0.353 | 0.023 | -0.208 | 0.084 | 0.084 | 0.129 | 0.489 | 0.092 | 0.500 | -0.007 | 0.273 | -0.104 | 0.064 | -0.123 | -0.165 | -0.050 | -0.237 | -0.074 | -0.152 | 0.001 | -0.296 | 0.028 |
| Roach | 1124 | 2 | 2 | 3 | -0.357 | -0.006 | -0.225 | 0.086 | 0.097 | 0.166 | 0.497 | 0.059 | 0.477 | -0.032 | 0.284 | -0.071 | 0.069 | -0.099 | -0.161 | -0.045 | -0.220 | -0.078 | -0.154 | 0.004 | -0.307 | 0.017 |
| Roach | 1125 | 2 | 2 | 3 | -0.361 | 0.026 | -0.218 | 0.080 | 0.077 | 0.154 | 0.503 | 0.078 | 0.497 | -0.023 | 0.263 | -0.083 | 0.051 | -0.109 | -0.148 | -0.064 | -0.227 | -0.083 | -0.152 | 0.001 | -0.286 | 0.023 |
| Roach | 1126 | 2 | 2 | 3 | -0.378 | 0.025 | -0.224 | 0.092 | 0.061 | 0.137 | 0.495 | 0.076 | 0.502 | -0.020 | 0.262 | -0.091 | 0.072 | -0.103 | -0.149 | -0.054 | -0.226 | -0.082 | -0.128 | -0.002 | -0.287 | 0.022 |
| Roach | 1127 | 2 | 2 | 3 | -0.356 | 0.018 | -0.221 | 0.088 | 0.080 | 0.142 | 0.478 | 0.080 | 0.499 | -0.014 | 0.276 | -0.097 | 0.087 | -0.126 | -0.160 | -0.047 | -0.230 | -0.069 | -0.159 | 0.002 | -0.295 | 0.024 |
| Roach | 1128 | 2 | 2 | 3 | -0.375 | 0.006 | -0.226 | 0.094 | 0.072 | 0.178 | 0.497 | 0.061 | 0.483 | -0.046 | 0.261 | -0.065 | 0.100 | -0.096 | -0.155 | -0.065 | -0.228 | -0.077 | -0.141 | -0.005 | -0.287 | 0.017 |
| Roach | 1129 | 2 | 2 | 3 | -0.369 | -0.006 | -0.242 | 0.086 | 0.084 | 0.187 | 0.511 | 0.044 | 0.479 | -0.048 | 0.259 | -0.061 | 0.078 | -0.073 | -0.147 | -0.051 | -0.210 | -0.079 | -0.145 | 0.001 | -0.298 | -0.001 |
| Roach | 1130 | 2 | 2 | 3 | -0.360 | -0.018 | -0.240 | 0.095 | 0.077 | 0.192 | 0.509 | 0.028 | 0.467 | -0.048 | 0.275 | -0.052 | 0.099 | -0.076 | -0.147 | -0.046 | -0.212 | -0.083 | -0.163 | 0.003 | -0.304 | 0.005 |
| Roach | 1191 | 2 | 3 | 1 | -0.359 | 0.021 | -0.227 | 0.087 | 0.072 | 0.141 | 0.482 | 0.071 | 0.509 | -0.021 | 0.271 | -0.084 | 0.079 | -0.110 | -0.160 | -0.051 | -0.223 | -0.074 | -0.150 | 0.001 | -0.294 | 0.020 |
| Roach | 1192 | 2 | 3 | 1 | -0.360 | -0.007 | -0.222 | 0.092 | 0.082 | 0.175 | 0.488 | 0.070 | 0.498 | -0.031 | 0.266 | -0.093 | 0.063 | -0.109 | -0.153 | -0.047 | -0.221 | -0.072 | -0.146 | 0.010 | -0.296 | 0.011 |
| Roach | 1193 | 2 | 3 | 1 | -0.348 | -0.003 | -0.219 | 0.086 | 0.067 | 0.177 | 0.500 | 0.062 | 0.499 | -0.040 | 0.264 | -0.075 | 0.075 | -0.101 | -0.164 | -0.051 | -0.229 | -0.076 | -0.157 | 0.011 | -0.288 | 0.010 |
| Roach | 1194 | 2 | 3 | 1 | -0.362 | 0.027 | -0.234 | 0.081 | 0.085 | 0.152 | 0.482 | 0.079 | 0.497 | -0.018 | 0.272 | -0.093 | 0.079 | -0.117 | -0.155 | -0.062 | -0.242 | -0.072 | -0.141 | -0.001 | -0.281 | 0.024 |
| Roach | 1195 | 2 | 3 | 1 | -0.355 | 0.019 | -0.219 | 0.087 | 0.094 | 0.151 | 0.480 | 0.081 | 0.497 | -0.014 | 0.274 | -0.095 | 0.070 | -0.115 | -0.160 | -0.061 | -0.252 | -0.070 | -0.141 | -0.004 | -0.290 | 0.020 |
| Roach | 1196 | 2 | 3 | 1 | -0.361 | 0.011 | -0.232 | 0.085 | 0.100 | 0.163 | 0.498 | 0.067 | 0.471 | -0.022 | 0.281 | -0.080 | 0.070 | -0.113 | -0.154 | -0.054 | -0.226 | -0.080 | -0.148 | 0.005 | -0.299 | 0.017 |
| Roach | 1197 | 2 | 3 | 1 | -0.375 | -0.003 | -0.228 | 0.108 | 0.083 | 0.183 | 0.486 | 0.062 | 0.469 | -0.037 | 0.276 | -0.085 | 0.100 | -0.111 | -0.147 | -0.048 | -0.216 | -0.080 | -0.144 | 0.007 | -0.305 | 0.005 |
| Roach | 1198 | 2 | 3 | 1 | -0.362 | -0.005 | -0.208 | 0.098 | 0.105 | 0.147 | 0.478 | 0.077 | 0.487 | -0.016 | 0.283 | -0.094 | 0.077 | -0.113 | -0.162 | -0.043 | -0.240 | -0.079 | -0.161 | 0.009 | -0.297 | 0.021 |
| Roach | 1199 | 2 | 3 | 1 | -0.350 | 0.006 | -0.229 | 0.081 | 0.072 | 0.153 | 0.500 | 0.068 | 0.501 | -0.023 | 0.264 | -0.082 | 0.068 | -0.110 | -0.158 | -0.053 | -0.231 | -0.070 | -0.154 | 0.011 | -0.285 | 0.018 |
| Roach | 1200 | 2 | 3 | 1 | -0.345 | 0.025 | -0.222 | 0.088 | 0.094 | 0.150 | 0.481 | 0.075 | 0.500 | -0.012 | 0.273 | -0.094 | 0.073 | -0.111 | -0.161 | -0.064 | -0.234 | -0.076 | -0.161 | -0.004 | -0.298 | 0.021 |
| Roach | 1201 | 2 | 3 | 1 | -0.369 | 0.027 | -0.209 | 0.099 | 0.093 | 0.146 | 0.477 | 0.095 | 0.492 | -0.006 | 0.272 | -0.110 | 0.071 | -0.135 | -0.163 | -0.062 | -0.240 | -0.081 | -0.138 | 0.001 | -0.286 | 0.026 |
| Roach | 1202 | 2 | 3 | 1 | -0.355 | -0.020 | -0.249 | 0.089 | 0.110 | 0.189 | 0.484 | 0.067 | 0.489 | -0.041 | 0.264 | -0.091 | 0.070 | -0.097 | -0.157 | -0.044 | -0.215 | -0.080 | -0.147 | 0.019 | -0.294 | 0.010 |
| Roach | 1203 | 2 | 3 | 1 | -0.371 | 0.006 | -0.229 | 0.095 | 0.076 | 0.170 | 0.504 | 0.066 | 0.482 | -0.024 | 0.261 | -0.082 | 0.070 | -0.120 | -0.143 | -0.052 | -0.230 | -0.083 | -0.136 | 0.011 | -0.283 | 0.013 |
| Roach | 1204 | 2 | 3 | 1 | -0.359 | 0.017 | -0.210 | 0.090 | 0.069 | 0.156 | 0.494 | 0.070 | 0.492 | -0.023 | 0.273 | -0.081 | 0.075 | -0.115 | -0.156 | -0.057 | -0.234 | -0.077 | -0.150 | 0.005 | -0.296 | 0.015 |
| Roach | 1205 | 2 | 3 | 1 | -0.373 | 0.024 | -0.215 | 0.095 | 0.078 | 0.150 | 0.485 | 0.077 | 0.487 | -0.024 | 0.277 | -0.078 | 0.081 | -0.125 | -0.151 | -0.060 | -0.237 | -0.081 | -0.142 | 0.003 | -0.290 | 0.018 |
| Roach | 1206 | 2 | 3 | 1 | -0.371 | 0.001 | -0.227 | 0.095 | 0.062 | 0.177 | 0.496 | 0.059 | 0.497 | -0.040 | 0.265 | -0.077 | 0.070 | -0.092 | -0.147 | -0.060 | -0.228 | -0.079 | -0.136 | 0.007 | -0.281 | 0.009 |
| Roach | 1207 | 2 | 3 | 1 | -0.361 | -0.003 | -0.234 | 0.088 | 0.062 | 0.170 | 0.488 | 0.073 | 0.494 | -0.033 | 0.276 | -0.099 | 0.074 | -0.116 | -0.154 | -0.036 | -0.214 | -0.082 | -0.139 | 0.015 | -0.292 | 0.021 |
| Roach | 1208 | 2 | 3 | 1 | -0.355 | -0.019 | -0.236 | 0.082 | 0.076 | 0.185 | 0.512 | 0.051 | 0.488 | -0.044 | 0.260 | -0.075 | 0.068 | -0.082 | -0.157 | -0.037 | -0.211 | -0.078 | -0.147 | 0.012 | -0.296 | 0.004 |
| Roach | 1209 | 2 | 3 | 1 | -0.372 | 0.003 | -0.228 | 0.095 | 0.088 | 0.155 | 0.497 | 0.074 | 0.491 | -0.017 | 0.263 | -0.092 | 0.059 | -0.116 | -0.151 | -0.046 | -0.220 | -0.074 | -0.141 | 0.002 | -0.286 | 0.017 |
| Roach | 1210 | 2 | 3 | 1 | -0.360 | 0.005 | -0.228 | 0.088 | 0.082 | 0.162 | 0.492 | 0.063 | 0.489 | -0.032 | 0.270 | -0.081 | 0.087 | -0.095 | -0.156 | -0.056 | -0.232 | -0.076 | -0.150 | 0.008 | -0.294 | 0.013 |
| Roach | 1211 | 2 | 3 | 1 | -0.366 | 0.032 | -0.218 | 0.091 | 0.081 | 0.121 | 0.477 | 0.088 | 0.503 | -0.002 | 0.289 | -0.100 | 0.056 | -0.129 | -0.156 | -0.058 | -0.243 | -0.077 | -0.142 | 0.010 | -0.281 | 0.024 |
| Roach | 1212 | 2 | 3 | 1 | -0.371 | 0.013 | -0.215 | 0.096 | 0.077 | 0.152 | 0.488 | 0.087 | 0.498 | -0.021 | 0.269 | -0.101 | 0.059 | -0.117 | -0.154 | -0.049 | -0.234 | -0.080 | -0.132 | 0.003 | -0.284 | 0.017 |
| Roach | 1213 | 2 | 3 | 1 | -0.345 | 0.015 | -0.220 | 0.088 | 0.081 | 0.163 | 0.501 | 0.069 | 0.483 | -0.022 | 0.271 | -0.083 | 0.079 | -0.113 | -0.161 | -0.060 | -0.230 | -0.070 | -0.160 | -0.001 | -0.300 | 0.015 |
| Roach | 1214 | 2 | 3 | 1 | -0.356 | 0.012 | -0.216 | 0.086 | 0.070 | 0.162 | 0.502 | 0.063 | 0.485 | -0.027 | 0.280 | -0.071 | 0.069 | -0.112 | -0.159 | -0.057 | -0.231 | -0.074 | -0.155 | 0.000 | -0.289 | 0.017 |
| Roach | 1215 | 2 | 3 | 1 | -0.370 | 0.013 | -0.226 | 0.090 | 0.075 | 0.172 | 0.498 | 0.058 | 0.488 | -0.035 | 0.270 | -0.069 | 0.073 | -0.093 | -0.154 | -0.064 | -0.230 | -0.079 | -0.144 | 0.000 | -0.281 | 0.007 |
| Roach | 1216 | 2 | 3 | 1 | -0.354 | 0.013 | -0.215 | 0.085 | 0.066 | 0.144 | 0.493 | 0.073 | 0.494 | -0.020 | 0.274 | -0.090 | 0.089 | -0.114 | -0.164 | -0.047 | -0.234 | -0.077 | -0.155 | 0.013 | -0.293 | 0.020 |
| Roach | 1217 | 2 | 3 | 1 | -0.382 | -0.014 | -0.238 | 0.098 | 0.090 | 0.182 | 0.500 | 0.042 | 0.471 | -0.047 | 0.271 | -0.054 | 0.087 | -0.082 | -0.145 | -0.055 | -0.231 | -0.088 | -0.136 | 0.018 | -0.287 | 0.000 |
| Roach | 1218 | 2 | 3 | 1 | -0.354 | 0.000 | -0.221 | 0.094 | 0.084 | 0.174 | 0.497 | 0.068 | 0.484 | -0.031 | 0.271 | -0.083 | 0.071 | -0.113 | -0.156 | -0.047 | -0.224 | -0.082 | -0.154 | 0.009 | -0.298 | 0.010 |
| Roach | 1219 | 2 | 3 | 1 | -0.369 | 0.011 | -0.235 | 0.089 | 0.068 | 0.177 | 0.504 | 0.054 | 0.473 | -0.040 | 0.283 | -0.068 | 0.079 | -0.094 | -0.147 | -0.054 | -0.218 | -0.084 | -0.141 | -0.002 | -0.296 | 0.012 |
| Roach | 1220 | 2 | 3 | 1 | -0.361 | 0.015 | -0.248 | 0.082 | 0.082 | 0.160 | 0.486 | 0.071 | 0.486 | -0.028 | 0.275 | -0.090 | 0.098 | -0.109 | -0.152 | -0.053 | -0.235 | -0.067 | -0.147 | 0.000 | -0.282 | 0.019 |
| Roach | 1191 | 2 | 3 | 2 | -0.366 | 0.018 | -0.216 | 0.092 | 0.081 | 0.146 | 0.477 | 0.070 | 0.502 | -0.022 | 0.273 | -0.083 | 0.088 | -0.108 | -0.162 | -0.051 | -0.228 | -0.074 | -0.150 | -0.007 | -0.299 | 0.019 |
| Roach | 1192 | 2 | 3 | 2 | -0.356 | -0.005 | -0.223 | 0.093 | 0.086 | 0.173 | 0.492 | 0.070 | 0.493 | -0.027 | 0.273 | -0.094 | 0.056 | -0.106 | -0.152 | -0.047 | -0.223 | -0.072 | -0.150 | 0.005 | -0.296 | 0.009 |
| Roach | 1193 | 2 | 3 | 2 | -0.351 | 0.001 | -0.219 | 0.083 | 0.073 | 0.181 | 0.494 | 0.062 | 0.496 | -0.041 | 0.266 | -0.074 | 0.081 | -0.101 | -0.160 | -0.052 | -0.234 | -0.078 | -0.155 | 0.009 | -0.291 | 0.010 |
| Roach | 1194 | 2 | 3 | 2 | -0.365 | 0.029 | -0.221 | 0.087 | 0.084 | 0.153 | 0.483 | 0.079 | 0.494 | -0.016 | 0.270 | -0.091 | 0.080 | -0.122 | -0.158 | -0.064 | -0.235 | -0.075 | -0.148 | -0.002 | -0.286 | 0.022 |
| Roach | 1195 | 2 | 3 | 2 | -0.358 | 0.022 | -0.215 | 0.088 | 0.091 | 0.153 | 0.480 | 0.081 | 0.495 | -0.016 | 0.276 | -0.094 | 0.072 | -0.119 | -0.157 | -0.061 | -0.242 | -0.075 | -0.150 | 0.000 | -0.293 | 0.021 |
| Roach | 1196 | 2 | 3 | 2 | -0.363 | 0.012 | -0.220 | 0.093 | 0.092 | 0.163 | 0.496 | 0.069 | 0.472 | -0.022 | 0.282 | -0.083 | 0.075 | -0.111 | -0.155 | -0.060 | -0.228 | -0.077 | -0.150 | 0.003 | -0.300 | 0.014 |
| Roach | 1197 | 2 | 3 | 2 | -0.378 | 0.000 | -0.232 | 0.107 | 0.084 | 0.186 | 0.485 | 0.061 | 0.470 | -0.037 | 0.275 | -0.085 | 0.099 | -0.112 | -0.147 | -0.046 | -0.206 | -0.079 | -0.145 | -0.001 | -0.305 | 0.007 |
| Roach | 1198 | 2 | 3 | 2 | -0.359 | 0.001 | -0.214 | 0.094 | 0.089 | 0.145 | 0.481 | 0.076 | 0.488 | -0.020 | 0.292 | -0.091 | 0.072 | -0.114 | -0.160 | -0.049 | -0.234 | -0.078 | -0.160 | 0.015 | -0.295 | 0.021 |
| Roach | 1199 | 2 | 3 | 2 | -0.354 | 0.003 | -0.204 | 0.094 | 0.061 | 0.149 | 0.504 | 0.070 | 0.505 | -0.021 | 0.262 | -0.083 | 0.061 | -0.110 | -0.161 | -0.052 | -0.229 | -0.072 | -0.156 | 0.006 | -0.289 | 0.015 |
| Roach | 1200 | 2 | 3 | 2 | -0.348 | 0.025 | -0.219 | 0.091 | 0.093 | 0.149 | 0.478 | 0.077 | 0.506 | -0.012 | 0.270 | -0.094 | 0.069 | -0.111 | -0.165 | -0.063 | -0.227 | -0.076 | -0.159 | -0.005 | -0.299 | 0.020 |
| Roach | 1201 | 2 | 3 | 2 | -0.369 | 0.029 | -0.199 | 0.100 | 0.092 | 0.142 | 0.475 | 0.093 | 0.494 | -0.004 | 0.278 | -0.103 | 0.068 | -0.138 | -0.166 | -0.064 | -0.242 | -0.081 | -0.142 | 0.000 | -0.287 | 0.026 |
| Roach | 1202 | 2 | 3 | 2 | -0.357 | -0.024 | -0.240 | 0.094 | 0.106 | 0.188 | 0.486 | 0.064 | 0.491 | -0.037 | 0.259 | -0.091 | 0.071 | -0.100 | -0.154 | -0.042 | -0.217 | -0.081 | -0.148 | 0.019 | -0.297 | 0.009 |
| Roach | 1203 | 2 | 3 | 2 | -0.372 | 0.006 | -0.219 | 0.099 | 0.078 | 0.172 | 0.507 | 0.063 | 0.474 | -0.020 | 0.265 | -0.082 | 0.074 | -0.118 | -0.144 | -0.051 | -0.238 | -0.083 | -0.142 | 0.003 | -0.282 | 0.012 |
| Roach | 1204 | 2 | 3 | 2 | -0.361 | 0.014 | -0.215 | 0.092 | 0.086 | 0.156 | 0.505 | 0.069 | 0.477 | -0.017 | 0.270 | -0.080 | 0.075 | -0.114 | -0.160 | -0.059 | -0.228 | -0.076 | -0.153 | -0.003 | -0.297 | 0.018 |
| Roach | 1205 | 2 | 3 | 2 | -0.368 | 0.026 | -0.221 | 0.091 | 0.070 | 0.148 | 0.481 | 0.077 | 0.500 | -0.026 | 0.274 | -0.077 | 0.082 | -0.123 | -0.147 | -0.058 | -0.238 | -0.073 | -0.148 | -0.006 | -0.285 | 0.021 |
| Roach | 1206 | 2 | 3 | 2 | -0.372 | 0.003 | -0.229 | 0.095 | 0.066 | 0.178 | 0.505 | 0.059 | 0.488 | -0.036 | 0.265 | -0.078 | 0.068 | -0.095 | -0.147 | -0.057 | -0.225 | -0.079 | -0.137 | 0.002 | -0.281 | 0.008 |
| Roach | 1207 | 2 | 3 | 2 | -0.361 | -0.003 | -0.225 | 0.089 | 0.067 | 0.171 | 0.484 | 0.072 | 0.501 | -0.033 | 0.277 | -0.096 | 0.061 | -0.109 | -0.151 | -0.040 | -0.224 | -0.086 | -0.139 | 0.014 | -0.291 | 0.020 |
| Roach | 1208 | 2 | 3 | 2 | -0.353 | -0.019 | -0.233 | 0.084 | 0.074 | 0.183 | 0.502 | 0.053 | 0.501 | -0.049 | 0.258 | -0.072 | 0.068 | -0.080 | -0.156 | -0.035 | -0.214 | -0.078 | -0.150 | 0.010 | -0.297 | 0.003 |
| Roach | 1209 | 2 | 3 | 2 | -0.369 | 0.002 | -0.224 | 0.095 | 0.084 | 0.153 | 0.486 | 0.074 | 0.508 | -0.017 | 0.260 | -0.093 | 0.059 | -0.116 | -0.154 | -0.045 | -0.219 | -0.068 | -0.145 | -0.001 | -0.286 | 0.017 |
| Roach | 1210 | 2 | 3 | 2 | -0.362 | 0.008 | -0.227 | 0.087 | 0.083 | 0.162 | 0.496 | 0.063 | 0.490 | -0.033 | 0.264 | -0.075 | 0.086 | -0.094 | -0.158 | -0.057 | -0.223 | -0.076 | -0.153 | 0.002 | -0.296 | 0.013 |
| Roach | 1211 | 2 | 3 | 2 | -0.369 | 0.034 | -0.207 | 0.091 | 0.089 | 0.121 | 0.484 | 0.089 | 0.492 | 0.001 | 0.288 | -0.097 | 0.058 | -0.129 | -0.157 | -0.061 | -0.244 | -0.077 | -0.151 | 0.005 | -0.283 | 0.024 |
| Roach | 1212 | 2 | 3 | 2 | -0.374 | 0.018 | -0.211 | 0.097 | 0.072 | 0.150 | 0.497 | 0.086 | 0.495 | -0.017 | 0.262 | -0.100 | 0.059 | -0.121 | -0.143 | -0.052 | -0.227 | -0.083 | -0.144 | 0.000 | -0.286 | 0.022 |
| Roach | 1213 | 2 | 3 | 2 | -0.340 | 0.018 | -0.221 | 0.083 | 0.082 | 0.160 | 0.510 | 0.069 | 0.478 | -0.023 | 0.268 | -0.080 | 0.082 | -0.110 | -0.161 | -0.062 | -0.227 | -0.071 | -0.168 | 0.000 | -0.302 | 0.016 |
| Roach | 1214 | 2 | 3 | 2 | -0.357 | 0.014 | -0.223 | 0.082 | 0.069 | 0.164 | 0.508 | 0.061 | 0.482 | -0.025 | 0.277 | -0.069 | 0.067 | -0.114 | -0.155 | -0.059 | -0.226 | -0.075 | -0.152 | 0.000 | -0.290 | 0.023 |
| Roach | 1215 | 2 | 3 | 2 | -0.369 | 0.016 | -0.230 | 0.092 | 0.061 | 0.168 | 0.504 | 0.057 | 0.494 | -0.036 | 0.265 | -0.069 | 0.070 | -0.090 | -0.152 | -0.061 | -0.224 | -0.078 | -0.140 | -0.007 | -0.279 | 0.008 |
| Roach | 1216 | 2 | 3 | 2 | -0.355 | 0.017 | -0.215 | 0.088 | 0.070 | 0.148 | 0.500 | 0.074 | 0.496 | -0.018 | 0.263 | -0.090 | 0.079 | -0.110 | -0.161 | -0.051 | -0.228 | -0.076 | -0.156 | -0.004 | -0.294 | 0.023 |
| Roach | 1217 | 2 | 3 | 2 | -0.386 | -0.007 | -0.234 | 0.098 | 0.083 | 0.184 | 0.502 | 0.043 | 0.467 | -0.047 | 0.274 | -0.056 | 0.088 | -0.084 | -0.136 | -0.053 | -0.233 | -0.090 | -0.135 | 0.010 | -0.288 | 0.001 |
| Roach | 1218 | 2 | 3 | 2 | -0.354 | -0.002 | -0.219 | 0.097 | 0.075 | 0.176 | 0.497 | 0.066 | 0.485 | -0.031 | 0.274 | -0.084 | 0.067 | -0.113 | -0.156 | -0.048 | -0.217 | -0.080 | -0.151 | 0.008 | -0.300 | 0.012 |
| Roach | 1219 | 2 | 3 | 2 | -0.366 | 0.013 | -0.226 | 0.090 | 0.069 | 0.177 | 0.505 | 0.055 | 0.478 | -0.038 | 0.274 | -0.069 | 0.080 | -0.094 | -0.149 | -0.058 | -0.218 | -0.084 | -0.150 | -0.001 | -0.297 | 0.009 |
| Roach | 1220 | 2 | 3 | 2 | -0.363 | 0.013 | -0.224 | 0.090 | 0.088 | 0.161 | 0.485 | 0.075 | 0.496 | -0.025 | 0.272 | -0.088 | 0.066 | -0.111 | -0.159 | -0.053 | -0.232 | -0.074 | -0.143 | 0.000 | -0.287 | 0.013 |
| Roach | 1191 | 2 | 3 | 3 | -0.361 | 0.024 | -0.218 | 0.090 | 0.075 | 0.143 | 0.483 | 0.074 | 0.507 | -0.019 | 0.269 | -0.085 | 0.076 | -0.110 | -0.161 | -0.056 | -0.224 | -0.072 | -0.149 | -0.009 | -0.296 | 0.021 |
| Roach | 1192 | 2 | 3 | 3 | -0.360 | -0.004 | -0.220 | 0.099 | 0.078 | 0.176 | 0.488 | 0.069 | 0.495 | -0.030 | 0.274 | -0.094 | 0.059 | -0.109 | -0.155 | -0.046 | -0.215 | -0.077 | -0.147 | 0.005 | -0.297 | 0.010 |
| Roach | 1193 | 2 | 3 | 3 | -0.351 | 0.002 | -0.217 | 0.090 | 0.070 | 0.180 | 0.495 | 0.062 | 0.496 | -0.039 | 0.267 | -0.074 | 0.077 | -0.101 | -0.161 | -0.050 | -0.231 | -0.077 | -0.156 | -0.005 | -0.290 | 0.013 |
| Roach | 1194 | 2 | 3 | 3 | -0.369 | 0.031 | -0.214 | 0.092 | 0.074 | 0.154 | 0.481 | 0.080 | 0.500 | -0.019 | 0.266 | -0.092 | 0.083 | -0.119 | -0.163 | -0.063 | -0.229 | -0.077 | -0.143 | -0.008 | -0.287 | 0.021 |
| Roach | 1195 | 2 | 3 | 3 | -0.358 | 0.024 | -0.217 | 0.086 | 0.089 | 0.153 | 0.483 | 0.080 | 0.495 | -0.012 | 0.274 | -0.096 | 0.069 | -0.119 | -0.159 | -0.059 | -0.240 | -0.074 | -0.142 | -0.004 | -0.293 | 0.021 |
| Roach | 1196 | 2 | 3 | 3 | -0.359 | 0.019 | -0.212 | 0.095 | 0.084 | 0.161 | 0.495 | 0.069 | 0.478 | -0.022 | 0.284 | -0.081 | 0.072 | -0.110 | -0.157 | -0.060 | -0.230 | -0.080 | -0.153 | -0.008 | -0.300 | 0.017 |
| Roach | 1197 | 2 | 3 | 3 | -0.376 | 0.002 | -0.239 | 0.100 | 0.088 | 0.187 | 0.485 | 0.060 | 0.469 | -0.037 | 0.273 | -0.082 | 0.105 | -0.111 | -0.153 | -0.045 | -0.208 | -0.075 | -0.140 | -0.008 | -0.304 | 0.011 |
| Roach | 1198 | 2 | 3 | 3 | -0.362 | 0.000 | -0.212 | 0.098 | 0.088 | 0.146 | 0.477 | 0.078 | 0.490 | -0.018 | 0.288 | -0.096 | 0.076 | -0.118 | -0.161 | -0.047 | -0.235 | -0.076 | -0.154 | 0.008 | -0.294 | 0.024 |
| Roach | 1199 | 2 | 3 | 3 | -0.352 | 0.007 | -0.215 | 0.090 | 0.064 | 0.151 | 0.500 | 0.068 | 0.511 | -0.022 | 0.257 | -0.082 | 0.064 | -0.108 | -0.161 | -0.049 | -0.226 | -0.073 | -0.154 | -0.001 | -0.287 | 0.018 |
| Roach | 1200 | 2 | 3 | 3 | -0.349 | 0.025 | -0.217 | 0.088 | 0.088 | 0.150 | 0.480 | 0.076 | 0.503 | -0.016 | 0.273 | -0.091 | 0.075 | -0.108 | -0.166 | -0.060 | -0.231 | -0.075 | -0.160 | -0.010 | -0.297 | 0.021 |
| Roach | 1201 | 2 | 3 | 3 | -0.372 | 0.033 | -0.197 | 0.104 | 0.097 | 0.142 | 0.474 | 0.093 | 0.496 | -0.001 | 0.275 | -0.101 | 0.063 | -0.138 | -0.166 | -0.062 | -0.243 | -0.077 | -0.142 | -0.023 | -0.286 | 0.027 |
| Roach | 1202 | 2 | 3 | 3 | -0.359 | -0.022 | -0.242 | 0.095 | 0.104 | 0.189 | 0.490 | 0.066 | 0.483 | -0.037 | 0.265 | -0.092 | 0.069 | -0.102 | -0.154 | -0.042 | -0.217 | -0.079 | -0.145 | 0.017 | -0.295 | 0.009 |
| Roach | 1203 | 2 | 3 | 3 | -0.371 | 0.006 | -0.226 | 0.097 | 0.085 | 0.171 | 0.501 | 0.064 | 0.477 | -0.021 | 0.260 | -0.080 | 0.084 | -0.120 | -0.149 | -0.051 | -0.238 | -0.080 | -0.140 | 0.001 | -0.283 | 0.013 |
| Roach | 1204 | 2 | 3 | 3 | -0.358 | 0.017 | -0.211 | 0.093 | 0.075 | 0.157 | 0.498 | 0.070 | 0.493 | -0.023 | 0.267 | -0.079 | 0.074 | -0.112 | -0.161 | -0.056 | -0.226 | -0.078 | -0.155 | -0.007 | -0.295 | 0.018 |
| Roach | 1205 | 2 | 3 | 3 | -0.370 | 0.027 | -0.221 | 0.096 | 0.075 | 0.149 | 0.491 | 0.076 | 0.488 | -0.022 | 0.271 | -0.078 | 0.080 | -0.124 | -0.150 | -0.055 | -0.234 | -0.079 | -0.139 | -0.008 | -0.290 | 0.019 |
| Roach | 1206 | 2 | 3 | 3 | -0.374 | 0.003 | -0.220 | 0.101 | 0.068 | 0.178 | 0.497 | 0.062 | 0.496 | -0.037 | 0.264 | -0.077 | 0.063 | -0.095 | -0.153 | -0.058 | -0.226 | -0.079 | -0.133 | -0.004 | -0.282 | 0.007 |
| Roach | 1207 | 2 | 3 | 3 | -0.359 | -0.008 | -0.222 | 0.091 | 0.063 | 0.171 | 0.492 | 0.072 | 0.494 | -0.032 | 0.277 | -0.097 | 0.063 | -0.109 | -0.155 | -0.039 | -0.228 | -0.084 | -0.133 | 0.017 | -0.292 | 0.019 |
| Roach | 1208 | 2 | 3 | 3 | -0.354 | -0.018 | -0.232 | 0.085 | 0.076 | 0.181 | 0.513 | 0.052 | 0.494 | -0.045 | 0.249 | -0.068 | 0.069 | -0.086 | -0.152 | -0.039 | -0.215 | -0.077 | -0.151 | 0.013 | -0.297 | 0.002 |
| Roach | 1209 | 2 | 3 | 3 | -0.369 | 0.007 | -0.224 | 0.096 | 0.076 | 0.153 | 0.504 | 0.071 | 0.498 | -0.017 | 0.253 | -0.089 | 0.063 | -0.113 | -0.152 | -0.047 | -0.219 | -0.070 | -0.144 | -0.003 | -0.286 | 0.013 |
| Roach | 1210 | 2 | 3 | 3 | -0.365 | 0.010 | -0.219 | 0.091 | 0.082 | 0.161 | 0.501 | 0.064 | 0.486 | -0.028 | 0.260 | -0.077 | 0.086 | -0.097 | -0.159 | -0.055 | -0.223 | -0.077 | -0.151 | -0.005 | -0.298 | 0.013 |
| Roach | 1211 | 2 | 3 | 3 | -0.371 | 0.035 | -0.203 | 0.092 | 0.097 | 0.121 | 0.477 | 0.091 | 0.496 | 0.000 | 0.286 | -0.096 | 0.058 | -0.127 | -0.162 | -0.066 | -0.244 | -0.074 | -0.151 | 0.002 | -0.285 | 0.022 |
| Roach | 1212 | 2 | 3 | 3 | -0.377 | 0.014 | -0.209 | 0.097 | 0.073 | 0.152 | 0.485 | 0.085 | 0.497 | -0.021 | 0.269 | -0.099 | 0.066 | -0.120 | -0.151 | -0.051 | -0.233 | -0.078 | -0.132 | -0.001 | -0.289 | 0.021 |
| Roach | 1213 | 2 | 3 | 3 | -0.342 | 0.016 | -0.220 | 0.085 | 0.078 | 0.162 | 0.503 | 0.069 | 0.485 | -0.024 | 0.270 | -0.082 | 0.079 | -0.108 | -0.162 | -0.058 | -0.224 | -0.073 | -0.167 | -0.003 | -0.300 | 0.017 |
| Roach | 1214 | 2 | 3 | 3 | -0.355 | 0.016 | -0.222 | 0.085 | 0.070 | 0.164 | 0.501 | 0.065 | 0.490 | -0.027 | 0.277 | -0.072 | 0.063 | -0.111 | -0.158 | -0.057 | -0.232 | -0.074 | -0.146 | -0.006 | -0.288 | 0.017 |
| Roach | 1215 | 2 | 3 | 3 | -0.368 | 0.013 | -0.227 | 0.086 | 0.068 | 0.167 | 0.506 | 0.056 | 0.497 | -0.034 | 0.262 | -0.067 | 0.064 | -0.087 | -0.150 | -0.058 | -0.228 | -0.079 | -0.143 | -0.006 | -0.281 | 0.009 |
| Roach | 1216 | 2 | 3 | 3 | -0.354 | 0.014 | -0.206 | 0.092 | 0.070 | 0.145 | 0.501 | 0.075 | 0.492 | -0.015 | 0.269 | -0.089 | 0.074 | -0.117 | -0.162 | -0.050 | -0.234 | -0.078 | -0.155 | 0.001 | -0.294 | 0.020 |
| Roach | 1217 | 2 | 3 | 3 | -0.388 | -0.015 | -0.233 | 0.104 | 0.090 | 0.188 | 0.504 | 0.040 | 0.465 | -0.046 | 0.272 | -0.054 | 0.082 | -0.080 | -0.135 | -0.053 | -0.232 | -0.090 | -0.135 | 0.002 | -0.289 | 0.004 |
| Roach | 1218 | 2 | 3 | 3 | -0.354 | 0.000 | -0.215 | 0.100 | 0.077 | 0.176 | 0.499 | 0.069 | 0.484 | -0.031 | 0.272 | -0.084 | 0.069 | -0.111 | -0.161 | -0.050 | -0.222 | -0.078 | -0.152 | -0.001 | -0.298 | 0.010 |
| Roach | 1219 | 2 | 3 | 3 | -0.364 | 0.017 | -0.225 | 0.091 | 0.066 | 0.177 | 0.510 | 0.057 | 0.474 | -0.041 | 0.275 | -0.064 | 0.078 | -0.094 | -0.152 | -0.060 | -0.220 | -0.081 | -0.147 | -0.011 | -0.297 | 0.010 |
| Roach | 1220 | 2 | 3 | 3 | -0.363 | 0.012 | -0.209 | 0.094 | 0.080 | 0.163 | 0.488 | 0.076 | 0.491 | -0.022 | 0.277 | -0.091 | 0.070 | -0.119 | -0.162 | -0.053 | -0.230 | -0.071 | -0.151 | -0.001 | -0.291 | 0.012 |
| Roach | 1001 | 3 | 2 | 1 | -0.346 | 0.014 | -0.218 | 0.088 | 0.080 | 0.157 | 0.486 | 0.076 | 0.508 | -0.007 | 0.272 | -0.102 | 0.055 | -0.123 | -0.166 | -0.050 | -0.232 | -0.069 | -0.157 | -0.001 | -0.282 | 0.018 |
| Roach | 1002 | 3 | 2 | 1 | -0.358 | 0.014 | -0.225 | 0.097 | 0.088 | 0.157 | 0.480 | 0.081 | 0.492 | -0.015 | 0.282 | -0.100 | 0.070 | -0.124 | -0.158 | -0.057 | -0.238 | -0.066 | -0.149 | -0.003 | -0.283 | 0.017 |
| Roach | 1003 | 3 | 2 | 1 | -0.354 | -0.012 | -0.235 | 0.088 | 0.071 | 0.188 | 0.508 | 0.050 | 0.483 | -0.039 | 0.272 | -0.071 | 0.071 | -0.097 | -0.151 | -0.048 | -0.229 | -0.073 | -0.158 | 0.010 | -0.278 | 0.004 |
| Roach | 1004 | 3 | 2 | 1 | -0.357 | 0.002 | -0.229 | 0.096 | 0.082 | 0.175 | 0.493 | 0.067 | 0.483 | -0.030 | 0.272 | -0.086 | 0.083 | -0.111 | -0.156 | -0.053 | -0.231 | -0.075 | -0.154 | 0.006 | -0.286 | 0.011 |
| Roach | 1005 | 3 | 2 | 1 | -0.346 | -0.006 | -0.231 | 0.083 | 0.093 | 0.168 | 0.485 | 0.068 | 0.482 | -0.030 | 0.285 | -0.090 | 0.088 | -0.110 | -0.168 | -0.034 | -0.231 | -0.076 | -0.162 | 0.013 | -0.296 | 0.014 |
| Roach | 1006 | 3 | 2 | 1 | -0.355 | 0.014 | -0.227 | 0.094 | 0.081 | 0.165 | 0.487 | 0.081 | 0.496 | -0.018 | 0.271 | -0.102 | 0.064 | -0.126 | -0.150 | -0.055 | -0.232 | -0.070 | -0.153 | 0.003 | -0.281 | 0.015 |
| Roach | 1007 | 3 | 2 | 1 | -0.360 | -0.019 | -0.248 | 0.092 | 0.087 | 0.210 | 0.505 | 0.048 | 0.472 | -0.048 | 0.272 | -0.074 | 0.072 | -0.090 | -0.149 | -0.049 | -0.220 | -0.076 | -0.149 | 0.007 | -0.281 | 0.000 |
| Roach | 1008 | 3 | 2 | 1 | -0.356 | 0.006 | -0.221 | 0.106 | 0.074 | 0.179 | 0.493 | 0.077 | 0.485 | -0.029 | 0.273 | -0.096 | 0.070 | -0.121 | -0.162 | -0.054 | -0.215 | -0.076 | -0.151 | -0.004 | -0.290 | 0.013 |
| Roach | 1009 | 3 | 2 | 1 | -0.356 | 0.014 | -0.225 | 0.095 | 0.084 | 0.160 | 0.482 | 0.079 | 0.492 | -0.023 | 0.280 | -0.093 | 0.076 | -0.123 | -0.161 | -0.054 | -0.234 | -0.071 | -0.154 | 0.003 | -0.283 | 0.014 |
| Roach | 1010 | 3 | 2 | 1 | -0.358 | 0.022 | -0.217 | 0.105 | 0.086 | 0.165 | 0.474 | 0.085 | 0.486 | -0.017 | 0.283 | -0.104 | 0.083 | -0.134 | -0.167 | -0.062 | -0.227 | -0.078 | -0.155 | 0.000 | -0.288 | 0.018 |
| Roach | 1011 | 3 | 2 | 1 | -0.353 | 0.019 | -0.230 | 0.098 | 0.082 | 0.169 | 0.490 | 0.076 | 0.492 | -0.022 | 0.254 | -0.097 | 0.096 | -0.117 | -0.157 | -0.066 | -0.223 | -0.071 | -0.163 | -0.004 | -0.288 | 0.015 |
| Roach | 1012 | 3 | 2 | 1 | -0.354 | 0.016 | -0.219 | 0.096 | 0.098 | 0.152 | 0.478 | 0.084 | 0.498 | -0.004 | 0.278 | -0.107 | 0.059 | -0.127 | -0.161 | -0.058 | -0.242 | -0.068 | -0.156 | -0.004 | -0.280 | 0.020 |
| Roach | 1013 | 3 | 2 | 1 | -0.355 | 0.014 | -0.228 | 0.092 | 0.092 | 0.162 | 0.493 | 0.075 | 0.496 | -0.021 | 0.255 | -0.090 | 0.079 | -0.116 | -0.163 | -0.053 | -0.225 | -0.072 | -0.160 | -0.005 | -0.283 | 0.013 |
| Roach | 1014 | 3 | 2 | 1 | -0.360 | 0.017 | -0.234 | 0.090 | 0.096 | 0.146 | 0.484 | 0.087 | 0.497 | -0.007 | 0.264 | -0.111 | 0.072 | -0.124 | -0.155 | -0.050 | -0.233 | -0.074 | -0.153 | 0.002 | -0.278 | 0.025 |
| Roach | 1015 | 3 | 2 | 1 | -0.362 | 0.001 | -0.237 | 0.097 | 0.078 | 0.174 | 0.506 | 0.064 | 0.489 | -0.032 | 0.263 | -0.082 | 0.058 | -0.101 | -0.145 | -0.052 | -0.220 | -0.081 | -0.153 | 0.004 | -0.276 | 0.009 |
| Roach | 1016 | 3 | 2 | 1 | -0.350 | 0.009 | -0.229 | 0.096 | 0.104 | 0.188 | 0.486 | 0.076 | 0.482 | -0.028 | 0.261 | -0.094 | 0.086 | -0.121 | -0.168 | -0.061 | -0.223 | -0.076 | -0.160 | 0.005 | -0.290 | 0.006 |
| Roach | 1017 | 3 | 2 | 1 | -0.356 | 0.001 | -0.229 | 0.096 | 0.093 | 0.177 | 0.491 | 0.063 | 0.486 | -0.026 | 0.270 | -0.084 | 0.078 | -0.111 | -0.163 | -0.052 | -0.220 | -0.072 | -0.159 | -0.003 | -0.291 | 0.010 |
| Roach | 1018 | 3 | 2 | 1 | -0.349 | 0.003 | -0.222 | 0.099 | 0.081 | 0.176 | 0.502 | 0.067 | 0.488 | -0.029 | 0.269 | -0.083 | 0.062 | -0.109 | -0.163 | -0.053 | -0.219 | -0.075 | -0.164 | -0.004 | -0.286 | 0.007 |
| Roach | 1019 | 3 | 2 | 1 | -0.352 | 0.009 | -0.227 | 0.095 | 0.103 | 0.176 | 0.498 | 0.066 | 0.473 | -0.024 | 0.272 | -0.083 | 0.081 | -0.109 | -0.159 | -0.061 | -0.228 | -0.069 | -0.166 | -0.007 | -0.294 | 0.007 |
| Roach | 1020 | 3 | 2 | 1 | -0.353 | 0.006 | -0.231 | 0.095 | 0.095 | 0.172 | 0.490 | 0.072 | 0.483 | -0.026 | 0.275 | -0.087 | 0.074 | -0.116 | -0.158 | -0.053 | -0.228 | -0.072 | -0.157 | -0.004 | -0.289 | 0.014 |
| Roach | 1021 | 3 | 2 | 1 | -0.353 | 0.003 | -0.229 | 0.091 | 0.093 | 0.167 | 0.502 | 0.075 | 0.478 | -0.025 | 0.271 | -0.092 | 0.068 | -0.112 | -0.158 | -0.051 | -0.225 | -0.074 | -0.159 | 0.003 | -0.289 | 0.014 |
| Roach | 1022 | 3 | 2 | 1 | -0.364 | 0.008 | -0.227 | 0.101 | 0.092 | 0.165 | 0.489 | 0.070 | 0.480 | -0.028 | 0.280 | -0.082 | 0.074 | -0.113 | -0.158 | -0.053 | -0.214 | -0.080 | -0.158 | -0.006 | -0.294 | 0.017 |
| Roach | 1023 | 3 | 2 | 1 | -0.358 | 0.012 | -0.222 | 0.098 | 0.104 | 0.148 | 0.494 | 0.078 | 0.486 | -0.010 | 0.278 | -0.091 | 0.053 | -0.116 | -0.165 | -0.058 | -0.225 | -0.073 | -0.161 | -0.009 | -0.284 | 0.020 |
| Roach | 1024 | 3 | 2 | 1 | -0.370 | -0.001 | -0.222 | 0.111 | 0.064 | 0.185 | 0.508 | 0.074 | 0.486 | -0.037 | 0.259 | -0.087 | 0.055 | -0.117 | -0.146 | -0.059 | -0.210 | -0.083 | -0.145 | -0.001 | -0.277 | 0.015 |
| Roach | 1025 | 3 | 2 | 1 | -0.344 | 0.009 | -0.227 | 0.098 | 0.091 | 0.178 | 0.495 | 0.074 | 0.488 | -0.021 | 0.261 | -0.097 | 0.075 | -0.120 | -0.160 | -0.055 | -0.221 | -0.073 | -0.167 | -0.006 | -0.290 | 0.012 |
| Roach | 1026 | 3 | 2 | 1 | -0.366 | -0.010 | -0.246 | 0.095 | 0.090 | 0.197 | 0.511 | 0.038 | 0.467 | -0.046 | 0.266 | -0.056 | 0.086 | -0.084 | -0.147 | -0.051 | -0.209 | -0.073 | -0.159 | -0.011 | -0.294 | 0.001 |
| Roach | 1027 | 3 | 2 | 1 | -0.355 | 0.035 | -0.225 | 0.100 | 0.103 | 0.142 | 0.469 | 0.087 | 0.500 | 0.005 | 0.280 | -0.109 | 0.064 | -0.137 | -0.162 | -0.066 | -0.225 | -0.070 | -0.160 | -0.012 | -0.290 | 0.025 |
| Roach | 1028 | 3 | 2 | 1 | -0.352 | 0.002 | -0.223 | 0.091 | 0.080 | 0.162 | 0.496 | 0.071 | 0.497 | -0.026 | 0.268 | -0.085 | 0.064 | -0.108 | -0.162 | -0.048 | -0.229 | -0.071 | -0.154 | -0.002 | -0.285 | 0.014 |
| Roach | 1029 | 3 | 2 | 1 | -0.345 | -0.004 | -0.236 | 0.091 | 0.093 | 0.181 | 0.505 | 0.052 | 0.472 | -0.033 | 0.281 | -0.074 | 0.078 | -0.091 | -0.167 | -0.055 | -0.219 | -0.070 | -0.169 | -0.001 | -0.292 | 0.005 |
| Roach | 1030 | 3 | 2 | 1 | -0.354 | 0.001 | -0.231 | 0.099 | 0.111 | 0.170 | 0.487 | 0.078 | 0.480 | -0.016 | 0.268 | -0.098 | 0.074 | -0.130 | -0.158 | -0.046 | -0.223 | -0.076 | -0.161 | 0.002 | -0.293 | 0.016 |
| Roach | 1001 | 3 | 2 | 2 | -0.351 | 0.009 | -0.214 | 0.090 | 0.088 | 0.160 | 0.484 | 0.078 | 0.503 | -0.010 | 0.275 | -0.104 | 0.053 | -0.121 | -0.163 | -0.051 | -0.228 | -0.073 | -0.162 | 0.005 | -0.286 | 0.017 |
| Roach | 1002 | 3 | 2 | 2 | -0.362 | 0.018 | -0.220 | 0.103 | 0.091 | 0.155 | 0.476 | 0.082 | 0.497 | -0.014 | 0.278 | -0.101 | 0.066 | -0.123 | -0.160 | -0.057 | -0.229 | -0.076 | -0.152 | -0.003 | -0.284 | 0.016 |
| Roach | 1003 | 3 | 2 | 2 | -0.359 | -0.012 | -0.230 | 0.095 | 0.074 | 0.190 | 0.509 | 0.049 | 0.480 | -0.034 | 0.269 | -0.075 | 0.069 | -0.099 | -0.152 | -0.050 | -0.222 | -0.077 | -0.158 | 0.007 | -0.280 | 0.005 |
| Roach | 1004 | 3 | 2 | 2 | -0.363 | 0.007 | -0.227 | 0.095 | 0.088 | 0.177 | 0.494 | 0.070 | 0.479 | -0.030 | 0.271 | -0.089 | 0.080 | -0.111 | -0.154 | -0.055 | -0.222 | -0.080 | -0.158 | 0.006 | -0.289 | 0.011 |
| Roach | 1005 | 3 | 2 | 2 | -0.351 | -0.004 | -0.229 | 0.083 | 0.098 | 0.169 | 0.485 | 0.069 | 0.478 | -0.030 | 0.285 | -0.093 | 0.088 | -0.109 | -0.165 | -0.035 | -0.221 | -0.080 | -0.169 | 0.015 | -0.299 | 0.014 |
| Roach | 1006 | 3 | 2 | 2 | -0.357 | 0.015 | -0.223 | 0.097 | 0.082 | 0.164 | 0.489 | 0.082 | 0.493 | -0.018 | 0.272 | -0.102 | 0.063 | -0.123 | -0.156 | -0.058 | -0.224 | -0.073 | -0.154 | 0.001 | -0.284 | 0.015 |
| Roach | 1007 | 3 | 2 | 2 | -0.364 | -0.021 | -0.243 | 0.101 | 0.087 | 0.209 | 0.508 | 0.049 | 0.467 | -0.049 | 0.268 | -0.073 | 0.074 | -0.092 | -0.146 | -0.052 | -0.211 | -0.080 | -0.156 | 0.008 | -0.285 | -0.001 |
| Roach | 1008 | 3 | 2 | 2 | -0.356 | 0.009 | -0.221 | 0.104 | 0.081 | 0.179 | 0.495 | 0.076 | 0.482 | -0.028 | 0.274 | -0.090 | 0.066 | -0.122 | -0.157 | -0.060 | -0.220 | -0.075 | -0.153 | -0.008 | -0.290 | 0.014 |
| Roach | 1009 | 3 | 2 | 2 | -0.360 | 0.013 | -0.226 | 0.097 | 0.089 | 0.162 | 0.489 | 0.077 | 0.484 | -0.020 | 0.278 | -0.093 | 0.072 | -0.121 | -0.161 | -0.055 | -0.225 | -0.077 | -0.157 | 0.003 | -0.283 | 0.014 |
| Roach | 1010 | 3 | 2 | 2 | -0.359 | 0.023 | -0.216 | 0.104 | 0.091 | 0.163 | 0.479 | 0.086 | 0.484 | -0.015 | 0.280 | -0.102 | 0.077 | -0.135 | -0.167 | -0.065 | -0.227 | -0.077 | -0.156 | -0.001 | -0.286 | 0.018 |
| Roach | 1011 | 3 | 2 | 2 | -0.353 | 0.022 | -0.225 | 0.101 | 0.078 | 0.167 | 0.485 | 0.079 | 0.499 | -0.022 | 0.261 | -0.098 | 0.082 | -0.120 | -0.161 | -0.065 | -0.217 | -0.074 | -0.162 | -0.005 | -0.287 | 0.015 |
| Roach | 1012 | 3 | 2 | 2 | -0.360 | 0.019 | -0.217 | 0.096 | 0.100 | 0.150 | 0.476 | 0.083 | 0.498 | -0.005 | 0.276 | -0.105 | 0.063 | -0.125 | -0.164 | -0.059 | -0.232 | -0.074 | -0.159 | -0.001 | -0.281 | 0.020 |
| Roach | 1013 | 3 | 2 | 2 | -0.356 | 0.010 | -0.222 | 0.098 | 0.090 | 0.164 | 0.498 | 0.076 | 0.487 | -0.021 | 0.261 | -0.090 | 0.077 | -0.116 | -0.162 | -0.056 | -0.231 | -0.069 | -0.159 | -0.007 | -0.283 | 0.011 |
| Roach | 1014 | 3 | 2 | 2 | -0.364 | 0.019 | -0.230 | 0.094 | 0.098 | 0.147 | 0.481 | 0.086 | 0.498 | -0.005 | 0.264 | -0.111 | 0.070 | -0.125 | -0.156 | -0.051 | -0.227 | -0.076 | -0.154 | -0.002 | -0.281 | 0.025 |
| Roach | 1015 | 3 | 2 | 2 | -0.362 | 0.000 | -0.232 | 0.098 | 0.078 | 0.176 | 0.511 | 0.062 | 0.487 | -0.029 | 0.260 | -0.083 | 0.055 | -0.100 | -0.147 | -0.052 | -0.219 | -0.082 | -0.151 | 0.002 | -0.278 | 0.008 |
| Roach | 1016 | 3 | 2 | 2 | -0.351 | 0.007 | -0.225 | 0.095 | 0.101 | 0.191 | 0.497 | 0.075 | 0.477 | -0.026 | 0.258 | -0.094 | 0.081 | -0.121 | -0.167 | -0.062 | -0.222 | -0.075 | -0.163 | 0.006 | -0.286 | 0.005 |
| Roach | 1017 | 3 | 2 | 2 | -0.357 | 0.006 | -0.227 | 0.097 | 0.085 | 0.174 | 0.496 | 0.063 | 0.483 | -0.026 | 0.271 | -0.081 | 0.076 | -0.113 | -0.162 | -0.053 | -0.218 | -0.077 | -0.155 | -0.003 | -0.291 | 0.012 |
| Roach | 1018 | 3 | 2 | 2 | -0.352 | 0.003 | -0.223 | 0.098 | 0.078 | 0.176 | 0.508 | 0.067 | 0.481 | -0.029 | 0.271 | -0.084 | 0.063 | -0.108 | -0.161 | -0.053 | -0.217 | -0.076 | -0.161 | -0.005 | -0.287 | 0.010 |
| Roach | 1019 | 3 | 2 | 2 | -0.356 | 0.012 | -0.232 | 0.094 | 0.106 | 0.179 | 0.499 | 0.067 | 0.470 | -0.024 | 0.272 | -0.084 | 0.074 | -0.111 | -0.161 | -0.062 | -0.214 | -0.076 | -0.166 | -0.005 | -0.292 | 0.009 |
| Roach | 1020 | 3 | 2 | 2 | -0.358 | 0.007 | -0.228 | 0.094 | 0.096 | 0.173 | 0.492 | 0.073 | 0.477 | -0.025 | 0.281 | -0.087 | 0.069 | -0.116 | -0.158 | -0.055 | -0.229 | -0.073 | -0.153 | -0.003 | -0.288 | 0.014 |
| Roach | 1021 | 3 | 2 | 2 | -0.354 | 0.004 | -0.231 | 0.089 | 0.096 | 0.162 | 0.500 | 0.074 | 0.484 | -0.023 | 0.267 | -0.090 | 0.069 | -0.110 | -0.158 | -0.052 | -0.224 | -0.073 | -0.162 | 0.005 | -0.288 | 0.014 |
| Roach | 1022 | 3 | 2 | 2 | -0.368 | 0.010 | -0.225 | 0.098 | 0.098 | 0.165 | 0.487 | 0.068 | 0.475 | -0.027 | 0.285 | -0.080 | 0.080 | -0.112 | -0.162 | -0.057 | -0.212 | -0.079 | -0.166 | -0.001 | -0.293 | 0.014 |
| Roach | 1023 | 3 | 2 | 2 | -0.360 | 0.013 | -0.219 | 0.095 | 0.104 | 0.147 | 0.494 | 0.080 | 0.489 | -0.011 | 0.274 | -0.093 | 0.055 | -0.114 | -0.166 | -0.059 | -0.228 | -0.074 | -0.161 | 0.001 | -0.282 | 0.015 |
| Roach | 1024 | 3 | 2 | 2 | -0.369 | 0.002 | -0.228 | 0.106 | 0.065 | 0.186 | 0.506 | 0.072 | 0.485 | -0.035 | 0.263 | -0.086 | 0.056 | -0.118 | -0.146 | -0.058 | -0.215 | -0.083 | -0.145 | 0.000 | -0.273 | 0.015 |
| Roach | 1025 | 3 | 2 | 2 | -0.349 | 0.013 | -0.223 | 0.096 | 0.094 | 0.176 | 0.495 | 0.078 | 0.486 | -0.022 | 0.268 | -0.096 | 0.067 | -0.119 | -0.161 | -0.055 | -0.220 | -0.071 | -0.170 | -0.013 | -0.287 | 0.012 |
| Roach | 1026 | 3 | 2 | 2 | -0.365 | -0.012 | -0.247 | 0.096 | 0.089 | 0.197 | 0.509 | 0.036 | 0.471 | -0.049 | 0.268 | -0.051 | 0.085 | -0.082 | -0.149 | -0.049 | -0.209 | -0.074 | -0.160 | -0.013 | -0.291 | 0.000 |
| Roach | 1027 | 3 | 2 | 2 | -0.355 | 0.035 | -0.217 | 0.099 | 0.098 | 0.140 | 0.472 | 0.086 | 0.500 | 0.003 | 0.282 | -0.105 | 0.065 | -0.135 | -0.167 | -0.063 | -0.223 | -0.071 | -0.164 | -0.011 | -0.290 | 0.023 |
| Roach | 1028 | 3 | 2 | 2 | -0.357 | 0.006 | -0.219 | 0.091 | 0.082 | 0.159 | 0.494 | 0.073 | 0.500 | -0.024 | 0.269 | -0.089 | 0.061 | -0.105 | -0.162 | -0.049 | -0.223 | -0.073 | -0.160 | -0.002 | -0.284 | 0.014 |
| Roach | 1029 | 3 | 2 | 2 | -0.347 | -0.003 | -0.232 | 0.093 | 0.098 | 0.180 | 0.509 | 0.052 | 0.468 | -0.031 | 0.279 | -0.070 | 0.077 | -0.094 | -0.169 | -0.056 | -0.216 | -0.073 | -0.175 | -0.003 | -0.292 | 0.004 |
| Roach | 1030 | 3 | 2 | 2 | -0.354 | -0.001 | -0.221 | 0.103 | 0.122 | 0.168 | 0.488 | 0.080 | 0.479 | -0.016 | 0.270 | -0.094 | 0.063 | -0.127 | -0.164 | -0.048 | -0.220 | -0.082 | -0.169 | 0.004 | -0.293 | 0.014 |
| Roach | 1001 | 3 | 2 | 3 | -0.350 | 0.011 | -0.219 | 0.089 | 0.086 | 0.162 | 0.492 | 0.078 | 0.498 | -0.012 | 0.274 | -0.103 | 0.048 | -0.120 | -0.161 | -0.051 | -0.228 | -0.076 | -0.157 | 0.004 | -0.282 | 0.018 |
| Roach | 1002 | 3 | 2 | 3 | -0.363 | 0.016 | -0.221 | 0.103 | 0.092 | 0.157 | 0.477 | 0.082 | 0.497 | -0.015 | 0.277 | -0.102 | 0.063 | -0.121 | -0.160 | -0.057 | -0.229 | -0.073 | -0.149 | -0.006 | -0.285 | 0.015 |
| Roach | 1003 | 3 | 2 | 3 | -0.359 | -0.014 | -0.229 | 0.096 | 0.072 | 0.190 | 0.512 | 0.048 | 0.479 | -0.035 | 0.271 | -0.075 | 0.066 | -0.094 | -0.155 | -0.049 | -0.220 | -0.079 | -0.160 | 0.010 | -0.278 | 0.004 |
| Roach | 1004 | 3 | 2 | 3 | -0.363 | 0.005 | -0.223 | 0.099 | 0.075 | 0.174 | 0.498 | 0.069 | 0.481 | -0.032 | 0.274 | -0.086 | 0.073 | -0.110 | -0.161 | -0.054 | -0.216 | -0.082 | -0.153 | 0.004 | -0.287 | 0.013 |
| Roach | 1005 | 3 | 2 | 3 | -0.350 | -0.009 | -0.227 | 0.084 | 0.089 | 0.167 | 0.486 | 0.070 | 0.486 | -0.031 | 0.283 | -0.093 | 0.083 | -0.108 | -0.163 | -0.033 | -0.225 | -0.079 | -0.168 | 0.019 | -0.294 | 0.014 |
| Roach | 1006 | 3 | 2 | 3 | -0.359 | 0.011 | -0.224 | 0.095 | 0.077 | 0.166 | 0.493 | 0.081 | 0.490 | -0.020 | 0.272 | -0.104 | 0.063 | -0.122 | -0.155 | -0.056 | -0.221 | -0.074 | -0.151 | 0.007 | -0.285 | 0.017 |
| Roach | 1007 | 3 | 2 | 3 | -0.365 | -0.020 | -0.245 | 0.098 | 0.083 | 0.211 | 0.509 | 0.048 | 0.470 | -0.052 | 0.270 | -0.072 | 0.067 | -0.087 | -0.147 | -0.053 | -0.210 | -0.079 | -0.151 | 0.008 | -0.282 | -0.002 |
| Roach | 1008 | 3 | 2 | 3 | -0.357 | 0.001 | -0.227 | 0.102 | 0.082 | 0.178 | 0.492 | 0.075 | 0.489 | -0.030 | 0.274 | -0.096 | 0.056 | -0.115 | -0.154 | -0.054 | -0.206 | -0.076 | -0.160 | 0.003 | -0.290 | 0.014 |
| Roach | 1009 | 3 | 2 | 3 | -0.361 | 0.013 | -0.223 | 0.096 | 0.087 | 0.160 | 0.486 | 0.077 | 0.483 | -0.020 | 0.284 | -0.095 | 0.074 | -0.120 | -0.159 | -0.058 | -0.229 | -0.076 | -0.161 | 0.006 | -0.281 | 0.016 |
| Roach | 1010 | 3 | 2 | 3 | -0.359 | 0.022 | -0.217 | 0.104 | 0.087 | 0.166 | 0.478 | 0.084 | 0.486 | -0.016 | 0.280 | -0.103 | 0.077 | -0.131 | -0.166 | -0.065 | -0.224 | -0.080 | -0.157 | 0.002 | -0.286 | 0.018 |
| Roach | 1011 | 3 | 2 | 3 | -0.349 | 0.020 | -0.227 | 0.094 | 0.082 | 0.169 | 0.498 | 0.078 | 0.498 | -0.022 | 0.259 | -0.095 | 0.060 | -0.118 | -0.162 | -0.062 | -0.210 | -0.078 | -0.165 | 0.005 | -0.285 | 0.009 |
| Roach | 1012 | 3 | 2 | 3 | -0.359 | 0.019 | -0.217 | 0.094 | 0.095 | 0.153 | 0.481 | 0.084 | 0.498 | -0.007 | 0.276 | -0.104 | 0.057 | -0.125 | -0.164 | -0.058 | -0.231 | -0.075 | -0.155 | -0.001 | -0.281 | 0.020 |
| Roach | 1013 | 3 | 2 | 3 | -0.359 | 0.012 | -0.220 | 0.098 | 0.091 | 0.165 | 0.503 | 0.077 | 0.489 | -0.020 | 0.257 | -0.090 | 0.063 | -0.114 | -0.160 | -0.055 | -0.223 | -0.076 | -0.159 | -0.008 | -0.282 | 0.012 |
| Roach | 1014 | 3 | 2 | 3 | -0.363 | 0.016 | -0.226 | 0.094 | 0.102 | 0.144 | 0.486 | 0.089 | 0.498 | -0.006 | 0.263 | -0.108 | 0.059 | -0.124 | -0.158 | -0.053 | -0.228 | -0.077 | -0.155 | 0.003 | -0.278 | 0.023 |
| Roach | 1015 | 3 | 2 | 3 | -0.364 | -0.003 | -0.231 | 0.101 | 0.080 | 0.177 | 0.508 | 0.065 | 0.490 | -0.031 | 0.259 | -0.084 | 0.051 | -0.100 | -0.150 | -0.053 | -0.211 | -0.084 | -0.154 | 0.006 | -0.278 | 0.008 |
| Roach | 1016 | 3 | 2 | 3 | -0.350 | 0.006 | -0.223 | 0.100 | 0.089 | 0.187 | 0.496 | 0.076 | 0.483 | -0.030 | 0.261 | -0.094 | 0.078 | -0.122 | -0.169 | -0.060 | -0.215 | -0.076 | -0.163 | 0.005 | -0.286 | 0.007 |
| Roach | 1017 | 3 | 2 | 3 | -0.356 | -0.001 | -0.226 | 0.098 | 0.089 | 0.173 | 0.496 | 0.064 | 0.484 | -0.026 | 0.269 | -0.083 | 0.077 | -0.112 | -0.166 | -0.054 | -0.217 | -0.076 | -0.161 | 0.005 | -0.288 | 0.010 |
| Roach | 1018 | 3 | 2 | 3 | -0.352 | 0.002 | -0.224 | 0.099 | 0.079 | 0.177 | 0.506 | 0.066 | 0.484 | -0.029 | 0.271 | -0.084 | 0.061 | -0.107 | -0.163 | -0.052 | -0.210 | -0.078 | -0.164 | -0.005 | -0.287 | 0.010 |
| Roach | 1019 | 3 | 2 | 3 | -0.356 | 0.009 | -0.229 | 0.095 | 0.106 | 0.181 | 0.502 | 0.069 | 0.470 | -0.024 | 0.272 | -0.086 | 0.069 | -0.110 | -0.164 | -0.062 | -0.213 | -0.076 | -0.166 | -0.004 | -0.290 | 0.009 |
| Roach | 1020 | 3 | 2 | 3 | -0.359 | 0.008 | -0.228 | 0.095 | 0.093 | 0.172 | 0.494 | 0.074 | 0.482 | -0.026 | 0.271 | -0.088 | 0.071 | -0.115 | -0.156 | -0.057 | -0.221 | -0.075 | -0.160 | 0.000 | -0.288 | 0.014 |
| Roach | 1021 | 3 | 2 | 3 | -0.354 | 0.004 | -0.230 | 0.091 | 0.098 | 0.162 | 0.498 | 0.075 | 0.484 | -0.022 | 0.268 | -0.092 | 0.069 | -0.110 | -0.161 | -0.053 | -0.224 | -0.074 | -0.158 | 0.007 | -0.288 | 0.013 |
| Roach | 1022 | 3 | 2 | 3 | -0.363 | 0.006 | -0.223 | 0.099 | 0.093 | 0.162 | 0.490 | 0.069 | 0.485 | -0.027 | 0.277 | -0.080 | 0.071 | -0.111 | -0.160 | -0.054 | -0.213 | -0.078 | -0.166 | 0.003 | -0.291 | 0.013 |
| Roach | 1023 | 3 | 2 | 3 | -0.360 | 0.015 | -0.215 | 0.097 | 0.099 | 0.148 | 0.487 | 0.075 | 0.497 | -0.009 | 0.276 | -0.091 | 0.053 | -0.117 | -0.169 | -0.060 | -0.221 | -0.075 | -0.165 | -0.001 | -0.283 | 0.017 |
| Roach | 1024 | 3 | 2 | 3 | -0.368 | -0.001 | -0.224 | 0.108 | 0.063 | 0.182 | 0.504 | 0.075 | 0.489 | -0.038 | 0.263 | -0.086 | 0.055 | -0.116 | -0.150 | -0.059 | -0.215 | -0.082 | -0.143 | 0.002 | -0.273 | 0.014 |
| Roach | 1025 | 3 | 2 | 3 | -0.351 | 0.011 | -0.224 | 0.098 | 0.094 | 0.176 | 0.494 | 0.079 | 0.483 | -0.025 | 0.268 | -0.094 | 0.073 | -0.122 | -0.164 | -0.056 | -0.218 | -0.072 | -0.167 | -0.009 | -0.287 | 0.014 |
| Roach | 1026 | 3 | 2 | 3 | -0.366 | -0.015 | -0.245 | 0.097 | 0.094 | 0.198 | 0.511 | 0.038 | 0.471 | -0.049 | 0.264 | -0.052 | 0.078 | -0.083 | -0.151 | -0.052 | -0.200 | -0.074 | -0.162 | -0.006 | -0.294 | -0.002 |
| Roach | 1027 | 3 | 2 | 3 | -0.358 | 0.034 | -0.211 | 0.101 | 0.095 | 0.142 | 0.475 | 0.090 | 0.499 | 0.002 | 0.282 | -0.108 | 0.057 | -0.139 | -0.170 | -0.066 | -0.219 | -0.073 | -0.161 | -0.008 | -0.288 | 0.024 |
| Roach | 1028 | 3 | 2 | 3 | -0.354 | 0.007 | -0.224 | 0.092 | 0.077 | 0.159 | 0.494 | 0.074 | 0.499 | -0.027 | 0.269 | -0.089 | 0.066 | -0.105 | -0.164 | -0.050 | -0.227 | -0.074 | -0.155 | -0.002 | -0.282 | 0.014 |
| Roach | 1029 | 3 | 2 | 3 | -0.348 | -0.003 | -0.237 | 0.092 | 0.094 | 0.179 | 0.507 | 0.052 | 0.470 | -0.033 | 0.278 | -0.071 | 0.081 | -0.094 | -0.167 | -0.055 | -0.214 | -0.074 | -0.171 | 0.001 | -0.293 | 0.006 |
| Roach | 1030 | 3 | 2 | 3 | -0.356 | 0.000 | -0.220 | 0.100 | 0.104 | 0.173 | 0.487 | 0.079 | 0.486 | -0.019 | 0.267 | -0.098 | 0.068 | -0.127 | -0.164 | -0.050 | -0.216 | -0.079 | -0.164 | 0.007 | -0.292 | 0.013 |
| Roach | 1101 | 3 | 1 | 1 | -0.351 | 0.001 | -0.232 | 0.082 | 0.078 | 0.175 | 0.496 | 0.057 | 0.486 | -0.042 | 0.272 | -0.076 | 0.097 | -0.091 | -0.156 | -0.047 | -0.218 | -0.074 | -0.173 | 0.004 | -0.297 | 0.010 |
| Roach | 1102 | 3 | 1 | 1 | -0.366 | 0.017 | -0.220 | 0.093 | 0.075 | 0.140 | 0.491 | 0.075 | 0.502 | -0.016 | 0.268 | -0.085 | 0.067 | -0.116 | -0.152 | -0.055 | -0.229 | -0.069 | -0.151 | -0.005 | -0.286 | 0.021 |
| Roach | 1103 | 3 | 1 | 1 | -0.361 | 0.020 | -0.215 | 0.091 | 0.088 | 0.147 | 0.482 | 0.075 | 0.485 | -0.024 | 0.286 | -0.083 | 0.089 | -0.111 | -0.162 | -0.056 | -0.224 | -0.075 | -0.169 | -0.009 | -0.300 | 0.024 |
| Roach | 1104 | 3 | 1 | 1 | -0.361 | 0.013 | -0.217 | 0.096 | 0.081 | 0.160 | 0.500 | 0.067 | 0.486 | -0.022 | 0.273 | -0.079 | 0.064 | -0.109 | -0.155 | -0.057 | -0.221 | -0.083 | -0.159 | -0.006 | -0.291 | 0.020 |
| Roach | 1105 | 3 | 1 | 1 | -0.369 | 0.014 | -0.217 | 0.093 | 0.077 | 0.161 | 0.490 | 0.071 | 0.494 | -0.027 | 0.267 | -0.084 | 0.078 | -0.104 | -0.152 | -0.063 | -0.231 | -0.082 | -0.153 | -0.003 | -0.283 | 0.023 |
| Roach | 1106 | 3 | 1 | 1 | -0.356 | 0.025 | -0.218 | 0.091 | 0.082 | 0.153 | 0.483 | 0.077 | 0.504 | -0.011 | 0.268 | -0.097 | 0.067 | -0.119 | -0.155 | -0.059 | -0.222 | -0.078 | -0.164 | -0.007 | -0.290 | 0.025 |
| Roach | 1107 | 3 | 1 | 1 | -0.359 | 0.031 | -0.213 | 0.090 | 0.086 | 0.133 | 0.490 | 0.086 | 0.498 | -0.004 | 0.274 | -0.096 | 0.062 | -0.123 | -0.161 | -0.069 | -0.232 | -0.074 | -0.161 | -0.001 | -0.283 | 0.027 |
| Roach | 1108 | 3 | 1 | 1 | -0.359 | 0.013 | -0.221 | 0.089 | 0.081 | 0.150 | 0.496 | 0.070 | 0.495 | -0.024 | 0.271 | -0.077 | 0.069 | -0.107 | -0.155 | -0.051 | -0.226 | -0.070 | -0.163 | -0.011 | -0.289 | 0.018 |
| Roach | 1109 | 3 | 1 | 1 | -0.369 | 0.006 | -0.244 | 0.101 | 0.076 | 0.171 | 0.494 | 0.070 | 0.483 | -0.031 | 0.274 | -0.088 | 0.071 | -0.115 | -0.143 | -0.050 | -0.217 | -0.077 | -0.145 | 0.000 | -0.280 | 0.013 |
| Roach | 1110 | 3 | 1 | 1 | -0.363 | 0.018 | -0.237 | 0.087 | 0.082 | 0.143 | 0.495 | 0.073 | 0.490 | -0.018 | 0.277 | -0.087 | 0.067 | -0.116 | -0.154 | -0.050 | -0.217 | -0.070 | -0.157 | 0.000 | -0.285 | 0.018 |
| Roach | 1111 | 3 | 1 | 1 | -0.363 | 0.010 | -0.218 | 0.098 | 0.075 | 0.154 | 0.498 | 0.077 | 0.494 | -0.023 | 0.268 | -0.089 | 0.063 | -0.113 | -0.157 | -0.053 | -0.220 | -0.074 | -0.156 | -0.006 | -0.285 | 0.019 |
| Roach | 1112 | 3 | 1 | 1 | -0.370 | 0.019 | -0.233 | 0.087 | 0.088 | 0.149 | 0.497 | 0.071 | 0.485 | -0.024 | 0.274 | -0.079 | 0.073 | -0.106 | -0.151 | -0.053 | -0.226 | -0.075 | -0.157 | -0.008 | -0.282 | 0.018 |
| Roach | 1113 | 3 | 1 | 1 | -0.376 | 0.016 | -0.218 | 0.095 | 0.081 | 0.159 | 0.491 | 0.076 | 0.493 | -0.028 | 0.266 | -0.078 | 0.067 | -0.119 | -0.154 | -0.061 | -0.220 | -0.078 | -0.150 | -0.001 | -0.282 | 0.019 |
| Roach | 1114 | 3 | 1 | 1 | -0.365 | -0.001 | -0.226 | 0.086 | 0.075 | 0.177 | 0.506 | 0.061 | 0.485 | -0.038 | 0.269 | -0.072 | 0.065 | -0.098 | -0.146 | -0.049 | -0.223 | -0.079 | -0.151 | -0.004 | -0.288 | 0.017 |
| Roach | 1115 | 3 | 1 | 1 | -0.363 | 0.003 | -0.228 | 0.091 | 0.079 | 0.176 | 0.503 | 0.068 | 0.494 | -0.034 | 0.259 | -0.082 | 0.057 | -0.102 | -0.150 | -0.052 | -0.216 | -0.074 | -0.152 | -0.006 | -0.284 | 0.012 |
| Roach | 1116 | 3 | 1 | 1 | -0.357 | 0.018 | -0.218 | 0.087 | 0.082 | 0.144 | 0.487 | 0.079 | 0.497 | -0.011 | 0.270 | -0.098 | 0.076 | -0.120 | -0.156 | -0.056 | -0.229 | -0.074 | -0.157 | 0.002 | -0.295 | 0.028 |
| Roach | 1117 | 3 | 1 | 1 | -0.353 | 0.017 | -0.217 | 0.090 | 0.091 | 0.161 | 0.493 | 0.075 | 0.476 | -0.024 | 0.283 | -0.081 | 0.083 | -0.118 | -0.162 | -0.061 | -0.228 | -0.079 | -0.172 | -0.006 | -0.294 | 0.026 |
| Roach | 1118 | 3 | 1 | 1 | -0.355 | -0.006 | -0.228 | 0.090 | 0.088 | 0.159 | 0.496 | 0.066 | 0.488 | -0.034 | 0.278 | -0.076 | 0.067 | -0.101 | -0.156 | -0.037 | -0.211 | -0.079 | -0.166 | 0.004 | -0.301 | 0.016 |
| Roach | 1119 | 3 | 1 | 1 | -0.365 | 0.030 | -0.224 | 0.092 | 0.090 | 0.145 | 0.497 | 0.081 | 0.488 | -0.013 | 0.265 | -0.087 | 0.070 | -0.125 | -0.156 | -0.065 | -0.221 | -0.072 | -0.157 | -0.008 | -0.288 | 0.020 |
| Roach | 1120 | 3 | 1 | 1 | -0.363 | 0.007 | -0.218 | 0.096 | 0.067 | 0.141 | 0.507 | 0.073 | 0.492 | -0.025 | 0.277 | -0.082 | 0.053 | -0.101 | -0.152 | -0.046 | -0.223 | -0.075 | -0.153 | -0.009 | -0.287 | 0.019 |
| Roach | 1121 | 3 | 1 | 1 | -0.374 | -0.006 | -0.233 | 0.087 | 0.095 | 0.162 | 0.498 | 0.057 | 0.485 | -0.035 | 0.267 | -0.073 | 0.077 | -0.083 | -0.152 | -0.044 | -0.207 | -0.076 | -0.158 | -0.001 | -0.298 | 0.013 |
| Roach | 1122 | 3 | 1 | 1 | -0.359 | 0.017 | -0.200 | 0.089 | 0.082 | 0.132 | 0.481 | 0.084 | 0.499 | -0.002 | 0.287 | -0.104 | 0.062 | -0.126 | -0.156 | -0.047 | -0.235 | -0.075 | -0.165 | 0.004 | -0.296 | 0.030 |
| Roach | 1123 | 3 | 1 | 1 | -0.354 | 0.015 | -0.215 | 0.088 | 0.093 | 0.130 | 0.488 | 0.090 | 0.496 | -0.008 | 0.269 | -0.103 | 0.076 | -0.125 | -0.166 | -0.045 | -0.226 | -0.074 | -0.165 | 0.002 | -0.295 | 0.030 |
| Roach | 1124 | 3 | 1 | 1 | -0.357 | -0.011 | -0.224 | 0.088 | 0.097 | 0.164 | 0.495 | 0.058 | 0.483 | -0.034 | 0.276 | -0.069 | 0.077 | -0.095 | -0.160 | -0.041 | -0.220 | -0.077 | -0.163 | 0.000 | -0.305 | 0.018 |
| Roach | 1125 | 3 | 1 | 1 | -0.365 | 0.016 | -0.214 | 0.083 | 0.083 | 0.155 | 0.508 | 0.074 | 0.495 | -0.024 | 0.255 | -0.077 | 0.054 | -0.105 | -0.144 | -0.059 | -0.230 | -0.083 | -0.155 | -0.001 | -0.288 | 0.021 |
| Roach | 1126 | 3 | 1 | 1 | -0.381 | 0.017 | -0.219 | 0.097 | 0.075 | 0.138 | 0.495 | 0.075 | 0.491 | -0.016 | 0.268 | -0.093 | 0.077 | -0.105 | -0.149 | -0.056 | -0.227 | -0.080 | -0.144 | -0.002 | -0.285 | 0.023 |
| Roach | 1127 | 3 | 1 | 1 | -0.360 | 0.016 | -0.221 | 0.088 | 0.087 | 0.143 | 0.476 | 0.079 | 0.496 | -0.015 | 0.278 | -0.097 | 0.091 | -0.123 | -0.159 | -0.051 | -0.223 | -0.072 | -0.168 | 0.006 | -0.296 | 0.025 |
| Roach | 1128 | 3 | 1 | 1 | -0.376 | 0.009 | -0.223 | 0.092 | 0.067 | 0.177 | 0.495 | 0.058 | 0.486 | -0.043 | 0.262 | -0.067 | 0.101 | -0.097 | -0.149 | -0.062 | -0.229 | -0.079 | -0.152 | -0.004 | -0.282 | 0.016 |
| Roach | 1129 | 3 | 1 | 1 | -0.366 | -0.012 | -0.232 | 0.096 | 0.076 | 0.189 | 0.511 | 0.041 | 0.479 | -0.050 | 0.268 | -0.060 | 0.073 | -0.069 | -0.144 | -0.052 | -0.210 | -0.084 | -0.156 | -0.003 | -0.297 | 0.003 |
| Roach | 1130 | 3 | 1 | 1 | -0.366 | -0.018 | -0.234 | 0.095 | 0.076 | 0.190 | 0.497 | 0.029 | 0.477 | -0.057 | 0.266 | -0.047 | 0.122 | -0.070 | -0.147 | -0.045 | -0.220 | -0.079 | -0.165 | -0.003 | -0.306 | 0.004 |
| Roach | 1101 | 3 | 1 | 2 | -0.349 | 0.001 | -0.230 | 0.083 | 0.079 | 0.175 | 0.500 | 0.057 | 0.488 | -0.039 | 0.275 | -0.075 | 0.077 | -0.093 | -0.155 | -0.048 | -0.209 | -0.075 | -0.178 | 0.002 | -0.296 | 0.011 |
| Roach | 1102 | 3 | 1 | 2 | -0.369 | 0.018 | -0.217 | 0.093 | 0.076 | 0.139 | 0.489 | 0.076 | 0.501 | -0.017 | 0.269 | -0.084 | 0.072 | -0.120 | -0.154 | -0.055 | -0.228 | -0.071 | -0.152 | 0.001 | -0.286 | 0.020 |
| Roach | 1103 | 3 | 1 | 2 | -0.359 | 0.018 | -0.220 | 0.088 | 0.094 | 0.148 | 0.491 | 0.074 | 0.480 | -0.022 | 0.287 | -0.083 | 0.076 | -0.111 | -0.163 | -0.058 | -0.218 | -0.079 | -0.173 | 0.004 | -0.294 | 0.022 |
| Roach | 1104 | 3 | 1 | 2 | -0.361 | 0.016 | -0.217 | 0.097 | 0.079 | 0.162 | 0.500 | 0.066 | 0.486 | -0.022 | 0.276 | -0.076 | 0.062 | -0.109 | -0.158 | -0.059 | -0.217 | -0.083 | -0.159 | -0.013 | -0.289 | 0.023 |
| Roach | 1105 | 3 | 1 | 2 | -0.369 | 0.016 | -0.215 | 0.093 | 0.090 | 0.162 | 0.488 | 0.073 | 0.492 | -0.026 | 0.267 | -0.081 | 0.074 | -0.102 | -0.156 | -0.064 | -0.233 | -0.080 | -0.154 | -0.014 | -0.284 | 0.024 |
| Roach | 1106 | 3 | 1 | 2 | -0.357 | 0.022 | -0.211 | 0.093 | 0.086 | 0.152 | 0.488 | 0.082 | 0.504 | -0.013 | 0.262 | -0.096 | 0.063 | -0.119 | -0.158 | -0.061 | -0.224 | -0.078 | -0.165 | -0.004 | -0.287 | 0.022 |
| Roach | 1107 | 3 | 1 | 2 | -0.363 | 0.036 | -0.213 | 0.089 | 0.090 | 0.135 | 0.484 | 0.084 | 0.501 | -0.004 | 0.272 | -0.093 | 0.064 | -0.125 | -0.160 | -0.066 | -0.230 | -0.077 | -0.161 | -0.004 | -0.284 | 0.025 |
| Roach | 1108 | 3 | 1 | 2 | -0.362 | 0.013 | -0.224 | 0.087 | 0.088 | 0.151 | 0.495 | 0.070 | 0.492 | -0.023 | 0.273 | -0.078 | 0.069 | -0.108 | -0.157 | -0.051 | -0.222 | -0.071 | -0.162 | -0.007 | -0.288 | 0.018 |
| Roach | 1109 | 3 | 1 | 2 | -0.370 | 0.011 | -0.242 | 0.103 | 0.078 | 0.172 | 0.494 | 0.070 | 0.487 | -0.028 | 0.272 | -0.088 | 0.063 | -0.117 | -0.145 | -0.055 | -0.213 | -0.077 | -0.147 | -0.006 | -0.276 | 0.015 |
| Roach | 1110 | 3 | 1 | 2 | -0.364 | 0.016 | -0.218 | 0.092 | 0.080 | 0.143 | 0.495 | 0.078 | 0.497 | -0.018 | 0.273 | -0.087 | 0.058 | -0.117 | -0.161 | -0.050 | -0.218 | -0.072 | -0.156 | 0.001 | -0.286 | 0.014 |
| Roach | 1111 | 3 | 1 | 2 | -0.365 | 0.011 | -0.217 | 0.097 | 0.078 | 0.154 | 0.497 | 0.076 | 0.495 | -0.021 | 0.270 | -0.087 | 0.057 | -0.113 | -0.158 | -0.053 | -0.220 | -0.073 | -0.154 | -0.007 | -0.283 | 0.018 |
| Roach | 1112 | 3 | 1 | 2 | -0.372 | 0.016 | -0.223 | 0.093 | 0.090 | 0.150 | 0.501 | 0.073 | 0.484 | -0.024 | 0.269 | -0.081 | 0.068 | -0.104 | -0.156 | -0.056 | -0.219 | -0.079 | -0.154 | -0.002 | -0.288 | 0.014 |
| Roach | 1113 | 3 | 1 | 2 | -0.374 | 0.020 | -0.216 | 0.098 | 0.083 | 0.161 | 0.493 | 0.078 | 0.493 | -0.028 | 0.262 | -0.079 | 0.069 | -0.116 | -0.158 | -0.061 | -0.222 | -0.079 | -0.149 | -0.014 | -0.281 | 0.019 |
| Roach | 1114 | 3 | 1 | 2 | -0.369 | -0.002 | -0.221 | 0.090 | 0.079 | 0.179 | 0.504 | 0.061 | 0.484 | -0.038 | 0.267 | -0.071 | 0.064 | -0.102 | -0.147 | -0.051 | -0.218 | -0.082 | -0.153 | 0.001 | -0.290 | 0.015 |
| Roach | 1115 | 3 | 1 | 2 | -0.364 | 0.003 | -0.231 | 0.095 | 0.080 | 0.178 | 0.499 | 0.067 | 0.491 | -0.033 | 0.258 | -0.085 | 0.072 | -0.103 | -0.152 | -0.056 | -0.215 | -0.073 | -0.155 | -0.005 | -0.284 | 0.013 |
| Roach | 1116 | 3 | 1 | 2 | -0.357 | 0.019 | -0.221 | 0.084 | 0.102 | 0.146 | 0.489 | 0.081 | 0.493 | -0.009 | 0.270 | -0.095 | 0.063 | -0.120 | -0.156 | -0.059 | -0.230 | -0.075 | -0.163 | 0.005 | -0.290 | 0.023 |
| Roach | 1117 | 3 | 1 | 2 | -0.353 | 0.021 | -0.217 | 0.090 | 0.085 | 0.159 | 0.491 | 0.075 | 0.478 | -0.024 | 0.289 | -0.079 | 0.079 | -0.120 | -0.160 | -0.065 | -0.229 | -0.076 | -0.170 | -0.008 | -0.293 | 0.028 |
| Roach | 1118 | 3 | 1 | 2 | -0.352 | -0.010 | -0.229 | 0.090 | 0.093 | 0.160 | 0.497 | 0.066 | 0.482 | -0.033 | 0.285 | -0.078 | 0.067 | -0.102 | -0.159 | -0.039 | -0.216 | -0.076 | -0.171 | 0.005 | -0.296 | 0.017 |
| Roach | 1119 | 3 | 1 | 2 | -0.365 | 0.033 | -0.220 | 0.091 | 0.097 | 0.143 | 0.494 | 0.083 | 0.487 | -0.012 | 0.270 | -0.083 | 0.066 | -0.128 | -0.161 | -0.067 | -0.219 | -0.074 | -0.161 | -0.006 | -0.287 | 0.020 |
| Roach | 1120 | 3 | 1 | 2 | -0.365 | 0.008 | -0.224 | 0.089 | 0.076 | 0.143 | 0.504 | 0.073 | 0.495 | -0.024 | 0.274 | -0.083 | 0.048 | -0.100 | -0.153 | -0.050 | -0.214 | -0.074 | -0.156 | 0.000 | -0.286 | 0.018 |
| Roach | 1121 | 3 | 1 | 2 | -0.372 | -0.005 | -0.228 | 0.088 | 0.097 | 0.161 | 0.497 | 0.060 | 0.485 | -0.036 | 0.272 | -0.073 | 0.072 | -0.083 | -0.156 | -0.046 | -0.211 | -0.076 | -0.162 | -0.001 | -0.295 | 0.012 |
| Roach | 1122 | 3 | 1 | 2 | -0.358 | 0.022 | -0.199 | 0.089 | 0.084 | 0.133 | 0.480 | 0.084 | 0.505 | -0.001 | 0.284 | -0.104 | 0.055 | -0.126 | -0.160 | -0.051 | -0.230 | -0.075 | -0.168 | 0.002 | -0.292 | 0.026 |
| Roach | 1123 | 3 | 1 | 2 | -0.356 | 0.018 | -0.211 | 0.090 | 0.093 | 0.130 | 0.487 | 0.090 | 0.495 | -0.009 | 0.271 | -0.102 | 0.076 | -0.123 | -0.161 | -0.047 | -0.230 | -0.074 | -0.171 | 0.002 | -0.294 | 0.026 |
| Roach | 1124 | 3 | 1 | 2 | -0.358 | -0.008 | -0.228 | 0.084 | 0.101 | 0.165 | 0.494 | 0.058 | 0.480 | -0.034 | 0.281 | -0.069 | 0.075 | -0.096 | -0.157 | -0.044 | -0.224 | -0.076 | -0.165 | 0.006 | -0.300 | 0.013 |
| Roach | 1125 | 3 | 1 | 2 | -0.363 | 0.021 | -0.220 | 0.083 | 0.076 | 0.152 | 0.507 | 0.074 | 0.494 | -0.027 | 0.259 | -0.078 | 0.060 | -0.103 | -0.145 | -0.061 | -0.224 | -0.085 | -0.157 | 0.002 | -0.287 | 0.021 |
| Roach | 1126 | 3 | 1 | 2 | -0.385 | 0.025 | -0.223 | 0.097 | 0.069 | 0.137 | 0.491 | 0.075 | 0.492 | -0.018 | 0.272 | -0.093 | 0.078 | -0.103 | -0.150 | -0.059 | -0.215 | -0.081 | -0.144 | -0.005 | -0.286 | 0.025 |
| Roach | 1127 | 3 | 1 | 2 | -0.358 | 0.017 | -0.218 | 0.090 | 0.087 | 0.141 | 0.479 | 0.080 | 0.496 | -0.012 | 0.279 | -0.098 | 0.082 | -0.124 | -0.161 | -0.051 | -0.224 | -0.071 | -0.167 | 0.004 | -0.294 | 0.024 |
| Roach | 1128 | 3 | 1 | 2 | -0.375 | 0.009 | -0.232 | 0.092 | 0.070 | 0.179 | 0.499 | 0.057 | 0.477 | -0.041 | 0.266 | -0.069 | 0.101 | -0.098 | -0.149 | -0.061 | -0.222 | -0.081 | -0.153 | -0.003 | -0.283 | 0.018 |
| Roach | 1129 | 3 | 1 | 2 | -0.372 | -0.008 | -0.241 | 0.092 | 0.087 | 0.192 | 0.501 | 0.043 | 0.479 | -0.054 | 0.268 | -0.060 | 0.080 | -0.071 | -0.143 | -0.054 | -0.200 | -0.083 | -0.160 | 0.001 | -0.299 | 0.002 |
| Roach | 1130 | 3 | 1 | 2 | -0.365 | -0.022 | -0.238 | 0.094 | 0.084 | 0.191 | 0.505 | 0.025 | 0.464 | -0.049 | 0.272 | -0.052 | 0.119 | -0.072 | -0.152 | -0.045 | -0.212 | -0.079 | -0.171 | 0.007 | -0.305 | 0.002 |
| Roach | 1101 | 3 | 1 | 3 | -0.350 | -0.001 | -0.230 | 0.082 | 0.076 | 0.176 | 0.501 | 0.057 | 0.489 | -0.042 | 0.272 | -0.074 | 0.080 | -0.091 | -0.156 | -0.048 | -0.208 | -0.075 | -0.177 | 0.005 | -0.296 | 0.009 |
| Roach | 1102 | 3 | 1 | 3 | -0.368 | 0.017 | -0.215 | 0.093 | 0.074 | 0.138 | 0.491 | 0.076 | 0.499 | -0.017 | 0.271 | -0.084 | 0.072 | -0.117 | -0.157 | -0.055 | -0.228 | -0.071 | -0.152 | 0.002 | -0.287 | 0.019 |
| Roach | 1103 | 3 | 1 | 3 | -0.363 | 0.020 | -0.208 | 0.095 | 0.088 | 0.146 | 0.487 | 0.075 | 0.481 | -0.022 | 0.292 | -0.081 | 0.076 | -0.112 | -0.162 | -0.058 | -0.221 | -0.078 | -0.170 | -0.004 | -0.300 | 0.021 |
| Roach | 1104 | 3 | 1 | 3 | -0.364 | 0.015 | -0.215 | 0.097 | 0.076 | 0.160 | 0.504 | 0.065 | 0.483 | -0.020 | 0.274 | -0.081 | 0.064 | -0.108 | -0.157 | -0.059 | -0.215 | -0.087 | -0.159 | 0.000 | -0.290 | 0.019 |
| Roach | 1105 | 3 | 1 | 3 | -0.371 | 0.014 | -0.214 | 0.094 | 0.076 | 0.162 | 0.493 | 0.070 | 0.492 | -0.028 | 0.264 | -0.081 | 0.081 | -0.103 | -0.159 | -0.063 | -0.227 | -0.084 | -0.150 | -0.006 | -0.284 | 0.024 |
| Roach | 1106 | 3 | 1 | 3 | -0.360 | 0.022 | -0.211 | 0.093 | 0.083 | 0.152 | 0.479 | 0.078 | 0.511 | -0.011 | 0.268 | -0.097 | 0.059 | -0.119 | -0.160 | -0.059 | -0.220 | -0.079 | -0.162 | 0.000 | -0.287 | 0.021 |
| Roach | 1107 | 3 | 1 | 3 | -0.366 | 0.033 | -0.200 | 0.092 | 0.081 | 0.133 | 0.486 | 0.086 | 0.500 | -0.004 | 0.277 | -0.095 | 0.059 | -0.125 | -0.164 | -0.068 | -0.230 | -0.079 | -0.158 | 0.002 | -0.285 | 0.026 |
| Roach | 1108 | 3 | 1 | 3 | -0.364 | 0.015 | -0.228 | 0.087 | 0.084 | 0.152 | 0.490 | 0.068 | 0.495 | -0.024 | 0.275 | -0.079 | 0.070 | -0.109 | -0.158 | -0.052 | -0.215 | -0.076 | -0.162 | 0.001 | -0.288 | 0.018 |
| Roach | 1109 | 3 | 1 | 3 | -0.372 | 0.007 | -0.243 | 0.098 | 0.074 | 0.170 | 0.491 | 0.069 | 0.484 | -0.028 | 0.275 | -0.091 | 0.073 | -0.118 | -0.145 | -0.052 | -0.215 | -0.079 | -0.145 | 0.011 | -0.278 | 0.013 |
| Roach | 1110 | 3 | 1 | 3 | -0.362 | 0.021 | -0.234 | 0.087 | 0.083 | 0.143 | 0.497 | 0.077 | 0.491 | -0.020 | 0.276 | -0.084 | 0.061 | -0.117 | -0.159 | -0.052 | -0.215 | -0.071 | -0.157 | -0.001 | -0.283 | 0.017 |
| Roach | 1111 | 3 | 1 | 3 | -0.365 | 0.009 | -0.213 | 0.097 | 0.077 | 0.153 | 0.500 | 0.077 | 0.496 | -0.021 | 0.268 | -0.088 | 0.054 | -0.113 | -0.154 | -0.054 | -0.221 | -0.075 | -0.158 | -0.001 | -0.282 | 0.016 |
| Roach | 1112 | 3 | 1 | 3 | -0.372 | 0.017 | -0.221 | 0.094 | 0.083 | 0.150 | 0.499 | 0.074 | 0.486 | -0.025 | 0.273 | -0.082 | 0.067 | -0.105 | -0.157 | -0.056 | -0.216 | -0.080 | -0.156 | -0.004 | -0.286 | 0.017 |
| Roach | 1113 | 3 | 1 | 3 | -0.376 | 0.020 | -0.216 | 0.096 | 0.080 | 0.159 | 0.492 | 0.078 | 0.496 | -0.031 | 0.264 | -0.077 | 0.064 | -0.116 | -0.158 | -0.060 | -0.215 | -0.083 | -0.149 | -0.002 | -0.282 | 0.016 |
| Roach | 1114 | 3 | 1 | 3 | -0.369 | -0.002 | -0.221 | 0.087 | 0.078 | 0.178 | 0.507 | 0.060 | 0.482 | -0.038 | 0.270 | -0.070 | 0.060 | -0.101 | -0.149 | -0.051 | -0.217 | -0.083 | -0.152 | 0.004 | -0.289 | 0.015 |
| Roach | 1115 | 3 | 1 | 3 | -0.362 | 0.001 | -0.231 | 0.093 | 0.079 | 0.178 | 0.504 | 0.066 | 0.496 | -0.035 | 0.256 | -0.082 | 0.058 | -0.099 | -0.155 | -0.054 | -0.209 | -0.077 | -0.152 | -0.004 | -0.283 | 0.012 |
| Roach | 1116 | 3 | 1 | 3 | -0.359 | 0.020 | -0.218 | 0.086 | 0.095 | 0.143 | 0.491 | 0.080 | 0.491 | -0.010 | 0.271 | -0.096 | 0.072 | -0.116 | -0.156 | -0.053 | -0.231 | -0.077 | -0.167 | -0.002 | -0.290 | 0.025 |
| Roach | 1117 | 3 | 1 | 3 | -0.352 | 0.021 | -0.220 | 0.090 | 0.091 | 0.161 | 0.496 | 0.074 | 0.474 | -0.023 | 0.285 | -0.080 | 0.076 | -0.118 | -0.162 | -0.064 | -0.226 | -0.078 | -0.170 | -0.010 | -0.292 | 0.026 |
| Roach | 1118 | 3 | 1 | 3 | -0.357 | -0.015 | -0.219 | 0.095 | 0.083 | 0.157 | 0.501 | 0.065 | 0.484 | -0.034 | 0.279 | -0.076 | 0.067 | -0.102 | -0.160 | -0.038 | -0.212 | -0.079 | -0.168 | 0.009 | -0.299 | 0.018 |
| Roach | 1119 | 3 | 1 | 3 | -0.366 | 0.034 | -0.221 | 0.092 | 0.089 | 0.143 | 0.492 | 0.082 | 0.488 | -0.013 | 0.269 | -0.085 | 0.079 | -0.128 | -0.162 | -0.068 | -0.219 | -0.073 | -0.163 | -0.005 | -0.285 | 0.022 |
| Roach | 1120 | 3 | 1 | 3 | -0.365 | 0.008 | -0.219 | 0.092 | 0.058 | 0.141 | 0.509 | 0.071 | 0.499 | -0.026 | 0.271 | -0.082 | 0.052 | -0.101 | -0.153 | -0.048 | -0.214 | -0.074 | -0.154 | 0.002 | -0.285 | 0.017 |
| Roach | 1121 | 3 | 1 | 3 | -0.373 | -0.007 | -0.235 | 0.087 | 0.090 | 0.160 | 0.500 | 0.058 | 0.487 | -0.039 | 0.265 | -0.073 | 0.078 | -0.085 | -0.148 | -0.045 | -0.205 | -0.076 | -0.162 | 0.009 | -0.297 | 0.011 |
| Roach | 1122 | 3 | 1 | 3 | -0.360 | 0.019 | -0.192 | 0.092 | 0.072 | 0.134 | 0.483 | 0.083 | 0.503 | -0.003 | 0.287 | -0.102 | 0.057 | -0.128 | -0.164 | -0.049 | -0.231 | -0.076 | -0.165 | 0.001 | -0.289 | 0.027 |
| Roach | 1123 | 3 | 1 | 3 | -0.355 | 0.019 | -0.215 | 0.087 | 0.097 | 0.130 | 0.486 | 0.091 | 0.498 | -0.008 | 0.268 | -0.103 | 0.072 | -0.121 | -0.164 | -0.048 | -0.227 | -0.077 | -0.167 | 0.004 | -0.293 | 0.028 |
| Roach | 1124 | 3 | 1 | 3 | -0.356 | -0.010 | -0.230 | 0.083 | 0.101 | 0.163 | 0.498 | 0.058 | 0.484 | -0.035 | 0.275 | -0.066 | 0.071 | -0.098 | -0.162 | -0.039 | -0.217 | -0.079 | -0.163 | 0.007 | -0.300 | 0.016 |
| Roach | 1125 | 3 | 1 | 3 | -0.372 | 0.021 | -0.213 | 0.085 | 0.083 | 0.155 | 0.495 | 0.072 | 0.498 | -0.026 | 0.267 | -0.077 | 0.054 | -0.105 | -0.144 | -0.058 | -0.222 | -0.087 | -0.157 | -0.001 | -0.289 | 0.020 |
| Roach | 1126 | 3 | 1 | 3 | -0.383 | 0.024 | -0.223 | 0.095 | 0.074 | 0.140 | 0.493 | 0.075 | 0.494 | -0.016 | 0.266 | -0.092 | 0.077 | -0.104 | -0.153 | -0.057 | -0.217 | -0.081 | -0.142 | -0.006 | -0.285 | 0.024 |
| Roach | 1127 | 3 | 1 | 3 | -0.359 | 0.019 | -0.219 | 0.090 | 0.085 | 0.141 | 0.479 | 0.080 | 0.497 | -0.014 | 0.280 | -0.097 | 0.080 | -0.123 | -0.160 | -0.052 | -0.223 | -0.073 | -0.165 | 0.005 | -0.294 | 0.023 |
| Roach | 1128 | 3 | 1 | 3 | -0.379 | 0.008 | -0.225 | 0.094 | 0.065 | 0.177 | 0.496 | 0.057 | 0.480 | -0.042 | 0.267 | -0.068 | 0.104 | -0.101 | -0.152 | -0.060 | -0.223 | -0.081 | -0.153 | 0.001 | -0.281 | 0.015 |
| Roach | 1129 | 3 | 1 | 3 | -0.371 | -0.009 | -0.241 | 0.095 | 0.081 | 0.189 | 0.507 | 0.041 | 0.474 | -0.050 | 0.266 | -0.062 | 0.088 | -0.070 | -0.146 | -0.055 | -0.203 | -0.083 | -0.158 | 0.000 | -0.298 | 0.004 |
| Roach | 1130 | 3 | 1 | 3 | -0.365 | -0.024 | -0.237 | 0.095 | 0.078 | 0.191 | 0.508 | 0.031 | 0.477 | -0.056 | 0.269 | -0.049 | 0.091 | -0.072 | -0.145 | -0.043 | -0.206 | -0.080 | -0.168 | 0.008 | -0.300 | -0.001 |
| Roach | 1191 | 3 | 3 | 1 | -0.363 | 0.019 | -0.223 | 0.090 | 0.087 | 0.144 | 0.482 | 0.070 | 0.500 | -0.020 | 0.269 | -0.082 | 0.085 | -0.108 | -0.158 | -0.055 | -0.223 | -0.076 | -0.160 | -0.003 | -0.296 | 0.021 |
| Roach | 1192 | 3 | 3 | 1 | -0.358 | -0.005 | -0.225 | 0.095 | 0.094 | 0.175 | 0.483 | 0.069 | 0.497 | -0.028 | 0.269 | -0.090 | 0.065 | -0.109 | -0.148 | -0.043 | -0.222 | -0.072 | -0.158 | -0.004 | -0.296 | 0.013 |
| Roach | 1193 | 3 | 3 | 1 | -0.352 | 0.002 | -0.218 | 0.084 | 0.072 | 0.180 | 0.491 | 0.060 | 0.496 | -0.038 | 0.269 | -0.075 | 0.084 | -0.103 | -0.158 | -0.049 | -0.229 | -0.075 | -0.166 | -0.003 | -0.291 | 0.016 |
| Roach | 1194 | 3 | 3 | 1 | -0.368 | 0.024 | -0.227 | 0.090 | 0.089 | 0.155 | 0.487 | 0.075 | 0.487 | -0.016 | 0.269 | -0.089 | 0.087 | -0.120 | -0.156 | -0.057 | -0.230 | -0.076 | -0.153 | -0.010 | -0.284 | 0.025 |
| Roach | 1195 | 3 | 3 | 1 | -0.359 | 0.021 | -0.219 | 0.089 | 0.095 | 0.152 | 0.483 | 0.080 | 0.492 | -0.014 | 0.274 | -0.094 | 0.073 | -0.117 | -0.156 | -0.060 | -0.237 | -0.073 | -0.156 | -0.008 | -0.290 | 0.025 |
| Roach | 1196 | 3 | 3 | 1 | -0.364 | 0.009 | -0.213 | 0.093 | 0.095 | 0.161 | 0.493 | 0.065 | 0.477 | -0.022 | 0.284 | -0.076 | 0.073 | -0.109 | -0.156 | -0.056 | -0.222 | -0.079 | -0.165 | -0.003 | -0.301 | 0.018 |
| Roach | 1197 | 3 | 3 | 1 | -0.376 | 0.000 | -0.236 | 0.102 | 0.081 | 0.183 | 0.487 | 0.059 | 0.470 | -0.037 | 0.273 | -0.086 | 0.106 | -0.109 | -0.138 | -0.045 | -0.210 | -0.076 | -0.153 | -0.001 | -0.304 | 0.009 |
| Roach | 1198 | 3 | 3 | 1 | -0.363 | 0.001 | -0.219 | 0.093 | 0.098 | 0.147 | 0.481 | 0.076 | 0.489 | -0.019 | 0.279 | -0.093 | 0.079 | -0.110 | -0.153 | -0.046 | -0.234 | -0.077 | -0.161 | -0.001 | -0.297 | 0.028 |
| Roach | 1199 | 3 | 3 | 1 | -0.353 | 0.004 | -0.217 | 0.087 | 0.070 | 0.150 | 0.503 | 0.070 | 0.504 | -0.023 | 0.259 | -0.083 | 0.066 | -0.108 | -0.152 | -0.050 | -0.228 | -0.070 | -0.163 | 0.003 | -0.288 | 0.019 |
| Roach | 1200 | 3 | 3 | 1 | -0.348 | 0.023 | -0.222 | 0.088 | 0.087 | 0.147 | 0.481 | 0.074 | 0.499 | -0.016 | 0.285 | -0.090 | 0.067 | -0.110 | -0.159 | -0.058 | -0.225 | -0.075 | -0.167 | -0.006 | -0.298 | 0.023 |
| Roach | 1201 | 3 | 3 | 1 | -0.372 | 0.029 | -0.202 | 0.101 | 0.093 | 0.141 | 0.474 | 0.091 | 0.490 | -0.002 | 0.274 | -0.107 | 0.086 | -0.134 | -0.163 | -0.063 | -0.241 | -0.077 | -0.151 | -0.009 | -0.288 | 0.030 |
| Roach | 1202 | 3 | 3 | 1 | -0.361 | -0.025 | -0.237 | 0.098 | 0.109 | 0.186 | 0.483 | 0.063 | 0.481 | -0.039 | 0.270 | -0.087 | 0.081 | -0.100 | -0.153 | -0.038 | -0.218 | -0.078 | -0.157 | 0.008 | -0.298 | 0.012 |
| Roach | 1203 | 3 | 3 | 1 | -0.371 | 0.007 | -0.230 | 0.095 | 0.073 | 0.168 | 0.500 | 0.064 | 0.480 | -0.027 | 0.269 | -0.081 | 0.081 | -0.115 | -0.145 | -0.048 | -0.226 | -0.081 | -0.151 | -0.001 | -0.281 | 0.017 |
| Roach | 1204 | 3 | 3 | 1 | -0.363 | 0.017 | -0.214 | 0.092 | 0.097 | 0.157 | 0.491 | 0.069 | 0.481 | -0.016 | 0.274 | -0.081 | 0.078 | -0.118 | -0.153 | -0.057 | -0.227 | -0.076 | -0.165 | -0.005 | -0.299 | 0.018 |
| Roach | 1205 | 3 | 3 | 1 | -0.373 | 0.022 | -0.216 | 0.096 | 0.074 | 0.152 | 0.490 | 0.073 | 0.485 | -0.023 | 0.276 | -0.078 | 0.085 | -0.121 | -0.146 | -0.057 | -0.238 | -0.075 | -0.147 | -0.011 | -0.289 | 0.022 |
| Roach | 1206 | 3 | 3 | 1 | -0.371 | 0.003 | -0.228 | 0.097 | 0.065 | 0.176 | 0.495 | 0.058 | 0.495 | -0.037 | 0.270 | -0.077 | 0.070 | -0.096 | -0.148 | -0.055 | -0.224 | -0.080 | -0.142 | 0.001 | -0.280 | 0.010 |
| Roach | 1207 | 3 | 3 | 1 | -0.363 | -0.009 | -0.216 | 0.095 | 0.093 | 0.171 | 0.482 | 0.073 | 0.488 | -0.028 | 0.277 | -0.093 | 0.071 | -0.114 | -0.150 | -0.042 | -0.234 | -0.080 | -0.152 | 0.009 | -0.295 | 0.018 |
| Roach | 1208 | 3 | 3 | 1 | -0.355 | -0.021 | -0.234 | 0.085 | 0.083 | 0.183 | 0.511 | 0.048 | 0.486 | -0.041 | 0.260 | -0.068 | 0.072 | -0.087 | -0.151 | -0.033 | -0.213 | -0.073 | -0.160 | 0.001 | -0.298 | 0.006 |
| Roach | 1209 | 3 | 3 | 1 | -0.369 | 0.005 | -0.221 | 0.094 | 0.077 | 0.154 | 0.498 | 0.069 | 0.494 | -0.019 | 0.269 | -0.086 | 0.061 | -0.115 | -0.150 | -0.046 | -0.220 | -0.070 | -0.155 | -0.001 | -0.284 | 0.015 |
| Roach | 1210 | 3 | 3 | 1 | -0.363 | 0.004 | -0.223 | 0.089 | 0.097 | 0.163 | 0.492 | 0.059 | 0.487 | -0.029 | 0.266 | -0.073 | 0.089 | -0.092 | -0.156 | -0.051 | -0.229 | -0.076 | -0.164 | -0.007 | -0.296 | 0.013 |
| Roach | 1211 | 3 | 3 | 1 | -0.374 | 0.031 | -0.209 | 0.096 | 0.099 | 0.122 | 0.477 | 0.087 | 0.491 | 0.000 | 0.294 | -0.093 | 0.055 | -0.130 | -0.159 | -0.060 | -0.239 | -0.079 | -0.149 | 0.002 | -0.286 | 0.025 |
| Roach | 1212 | 3 | 3 | 1 | -0.379 | 0.014 | -0.215 | 0.098 | 0.077 | 0.152 | 0.486 | 0.081 | 0.498 | -0.018 | 0.269 | -0.099 | 0.059 | -0.117 | -0.144 | -0.053 | -0.211 | -0.083 | -0.153 | 0.001 | -0.288 | 0.025 |
| Roach | 1213 | 3 | 3 | 1 | -0.348 | 0.009 | -0.220 | 0.084 | 0.083 | 0.164 | 0.502 | 0.065 | 0.485 | -0.024 | 0.267 | -0.081 | 0.081 | -0.107 | -0.155 | -0.057 | -0.229 | -0.069 | -0.170 | -0.002 | -0.298 | 0.017 |
| Roach | 1214 | 3 | 3 | 1 | -0.355 | 0.013 | -0.218 | 0.082 | 0.072 | 0.164 | 0.496 | 0.064 | 0.495 | -0.030 | 0.276 | -0.069 | 0.068 | -0.109 | -0.155 | -0.058 | -0.228 | -0.074 | -0.161 | -0.003 | -0.290 | 0.021 |
| Roach | 1215 | 3 | 3 | 1 | -0.370 | 0.012 | -0.232 | 0.093 | 0.068 | 0.167 | 0.494 | 0.054 | 0.502 | -0.034 | 0.269 | -0.067 | 0.065 | -0.091 | -0.146 | -0.056 | -0.220 | -0.081 | -0.149 | -0.007 | -0.282 | 0.012 |
| Roach | 1216 | 3 | 3 | 1 | -0.359 | 0.012 | -0.211 | 0.092 | 0.076 | 0.147 | 0.491 | 0.072 | 0.489 | -0.019 | 0.272 | -0.087 | 0.091 | -0.114 | -0.158 | -0.050 | -0.231 | -0.077 | -0.163 | 0.000 | -0.298 | 0.024 |
| Roach | 1217 | 3 | 3 | 1 | -0.391 | -0.014 | -0.233 | 0.103 | 0.095 | 0.185 | 0.499 | 0.035 | 0.465 | -0.043 | 0.269 | -0.055 | 0.098 | -0.077 | -0.139 | -0.049 | -0.223 | -0.091 | -0.149 | 0.004 | -0.292 | 0.002 |
| Roach | 1218 | 3 | 3 | 1 | -0.354 | 0.002 | -0.224 | 0.096 | 0.081 | 0.174 | 0.496 | 0.065 | 0.483 | -0.031 | 0.269 | -0.080 | 0.081 | -0.114 | -0.153 | -0.048 | -0.217 | -0.080 | -0.163 | 0.000 | -0.300 | 0.016 |
| Roach | 1219 | 3 | 3 | 1 | -0.367 | 0.008 | -0.227 | 0.089 | 0.080 | 0.178 | 0.502 | 0.051 | 0.476 | -0.037 | 0.275 | -0.065 | 0.082 | -0.091 | -0.149 | -0.056 | -0.219 | -0.084 | -0.159 | -0.006 | -0.294 | 0.013 |
| Roach | 1220 | 3 | 3 | 1 | -0.365 | 0.009 | -0.223 | 0.093 | 0.088 | 0.161 | 0.483 | 0.068 | 0.492 | -0.023 | 0.278 | -0.086 | 0.076 | -0.109 | -0.150 | -0.054 | -0.231 | -0.069 | -0.157 | -0.004 | -0.291 | 0.014 |
| Roach | 1191 | 3 | 3 | 2 | -0.365 | 0.020 | -0.219 | 0.090 | 0.088 | 0.144 | 0.484 | 0.071 | 0.496 | -0.019 | 0.271 | -0.082 | 0.085 | -0.110 | -0.162 | -0.056 | -0.221 | -0.075 | -0.162 | -0.001 | -0.296 | 0.018 |
| Roach | 1192 | 3 | 3 | 2 | -0.358 | -0.005 | -0.219 | 0.095 | 0.088 | 0.174 | 0.495 | 0.071 | 0.492 | -0.028 | 0.266 | -0.092 | 0.059 | -0.107 | -0.152 | -0.045 | -0.218 | -0.072 | -0.160 | -0.002 | -0.294 | 0.011 |
| Roach | 1193 | 3 | 3 | 2 | -0.351 | 0.000 | -0.219 | 0.085 | 0.083 | 0.181 | 0.496 | 0.060 | 0.491 | -0.037 | 0.267 | -0.072 | 0.079 | -0.101 | -0.158 | -0.050 | -0.231 | -0.079 | -0.167 | 0.000 | -0.289 | 0.014 |
| Roach | 1194 | 3 | 3 | 2 | -0.370 | 0.032 | -0.215 | 0.094 | 0.080 | 0.153 | 0.484 | 0.079 | 0.494 | -0.017 | 0.269 | -0.090 | 0.084 | -0.121 | -0.154 | -0.064 | -0.227 | -0.076 | -0.159 | -0.013 | -0.285 | 0.024 |
| Roach | 1195 | 3 | 3 | 2 | -0.357 | 0.021 | -0.219 | 0.088 | 0.094 | 0.150 | 0.488 | 0.078 | 0.491 | -0.013 | 0.273 | -0.090 | 0.071 | -0.118 | -0.154 | -0.063 | -0.238 | -0.071 | -0.160 | -0.006 | -0.289 | 0.023 |
| Roach | 1196 | 3 | 3 | 2 | -0.363 | 0.013 | -0.217 | 0.093 | 0.099 | 0.161 | 0.490 | 0.066 | 0.479 | -0.023 | 0.281 | -0.076 | 0.074 | -0.109 | -0.156 | -0.057 | -0.219 | -0.080 | -0.170 | -0.006 | -0.300 | 0.018 |
| Roach | 1197 | 3 | 3 | 2 | -0.375 | 0.003 | -0.240 | 0.099 | 0.086 | 0.184 | 0.481 | 0.059 | 0.473 | -0.041 | 0.275 | -0.081 | 0.109 | -0.109 | -0.141 | -0.050 | -0.211 | -0.075 | -0.156 | 0.002 | -0.302 | 0.009 |
| Roach | 1198 | 3 | 3 | 2 | -0.363 | -0.001 | -0.222 | 0.093 | 0.096 | 0.147 | 0.483 | 0.074 | 0.480 | -0.019 | 0.292 | -0.095 | 0.077 | -0.111 | -0.159 | -0.048 | -0.224 | -0.079 | -0.164 | 0.010 | -0.295 | 0.028 |
| Roach | 1199 | 3 | 3 | 2 | -0.355 | 0.008 | -0.218 | 0.088 | 0.075 | 0.154 | 0.501 | 0.069 | 0.502 | -0.021 | 0.262 | -0.082 | 0.060 | -0.110 | -0.155 | -0.052 | -0.222 | -0.073 | -0.165 | 0.001 | -0.286 | 0.018 |
| Roach | 1200 | 3 | 3 | 2 | -0.350 | 0.023 | -0.221 | 0.090 | 0.094 | 0.148 | 0.481 | 0.076 | 0.497 | -0.014 | 0.279 | -0.091 | 0.072 | -0.113 | -0.164 | -0.062 | -0.225 | -0.076 | -0.168 | -0.004 | -0.296 | 0.023 |
| Roach | 1201 | 3 | 3 | 2 | -0.372 | 0.029 | -0.208 | 0.100 | 0.103 | 0.142 | 0.474 | 0.091 | 0.491 | -0.001 | 0.274 | -0.105 | 0.074 | -0.135 | -0.161 | -0.066 | -0.237 | -0.078 | -0.152 | -0.006 | -0.286 | 0.028 |
| Roach | 1202 | 3 | 3 | 2 | -0.359 | -0.026 | -0.241 | 0.099 | 0.111 | 0.188 | 0.490 | 0.065 | 0.485 | -0.037 | 0.254 | -0.089 | 0.078 | -0.101 | -0.155 | -0.041 | -0.213 | -0.082 | -0.156 | 0.013 | -0.294 | 0.012 |
| Roach | 1203 | 3 | 3 | 2 | -0.372 | 0.005 | -0.226 | 0.096 | 0.080 | 0.170 | 0.502 | 0.063 | 0.478 | -0.024 | 0.269 | -0.080 | 0.074 | -0.117 | -0.145 | -0.050 | -0.226 | -0.080 | -0.154 | 0.000 | -0.280 | 0.016 |
| Roach | 1204 | 3 | 3 | 2 | -0.359 | 0.018 | -0.214 | 0.090 | 0.083 | 0.156 | 0.495 | 0.067 | 0.488 | -0.019 | 0.271 | -0.077 | 0.077 | -0.115 | -0.153 | -0.057 | -0.227 | -0.076 | -0.167 | -0.007 | -0.295 | 0.020 |
| Roach | 1205 | 3 | 3 | 2 | -0.373 | 0.021 | -0.215 | 0.094 | 0.076 | 0.151 | 0.494 | 0.071 | 0.480 | -0.020 | 0.278 | -0.078 | 0.084 | -0.122 | -0.148 | -0.059 | -0.237 | -0.076 | -0.151 | -0.002 | -0.288 | 0.020 |
| Roach | 1206 | 3 | 3 | 2 | -0.370 | 0.003 | -0.230 | 0.094 | 0.068 | 0.176 | 0.500 | 0.058 | 0.493 | -0.036 | 0.267 | -0.076 | 0.066 | -0.096 | -0.144 | -0.054 | -0.224 | -0.078 | -0.144 | 0.000 | -0.281 | 0.009 |
| Roach | 1207 | 3 | 3 | 2 | -0.366 | -0.007 | -0.220 | 0.094 | 0.098 | 0.173 | 0.486 | 0.072 | 0.481 | -0.026 | 0.275 | -0.097 | 0.076 | -0.113 | -0.153 | -0.041 | -0.223 | -0.086 | -0.160 | 0.010 | -0.294 | 0.020 |
| Roach | 1208 | 3 | 3 | 2 | -0.357 | -0.020 | -0.235 | 0.084 | 0.089 | 0.184 | 0.507 | 0.050 | 0.483 | -0.040 | 0.263 | -0.071 | 0.073 | -0.090 | -0.154 | -0.035 | -0.210 | -0.075 | -0.162 | 0.006 | -0.299 | 0.007 |
| Roach | 1209 | 3 | 3 | 2 | -0.370 | 0.003 | -0.222 | 0.096 | 0.084 | 0.153 | 0.492 | 0.071 | 0.496 | -0.019 | 0.268 | -0.090 | 0.066 | -0.111 | -0.151 | -0.045 | -0.221 | -0.072 | -0.156 | -0.001 | -0.285 | 0.015 |
| Roach | 1210 | 3 | 3 | 2 | -0.364 | 0.006 | -0.221 | 0.089 | 0.094 | 0.161 | 0.490 | 0.062 | 0.488 | -0.031 | 0.267 | -0.075 | 0.090 | -0.096 | -0.158 | -0.056 | -0.223 | -0.078 | -0.169 | 0.008 | -0.295 | 0.010 |
| Roach | 1211 | 3 | 3 | 2 | -0.373 | 0.034 | -0.213 | 0.092 | 0.100 | 0.122 | 0.481 | 0.087 | 0.485 | -0.001 | 0.294 | -0.093 | 0.061 | -0.129 | -0.157 | -0.061 | -0.236 | -0.080 | -0.155 | 0.002 | -0.286 | 0.027 |
| Roach | 1212 | 3 | 3 | 2 | -0.374 | 0.014 | -0.219 | 0.098 | 0.075 | 0.151 | 0.483 | 0.081 | 0.498 | -0.018 | 0.270 | -0.100 | 0.072 | -0.121 | -0.145 | -0.051 | -0.230 | -0.079 | -0.148 | 0.004 | -0.283 | 0.023 |
| Roach | 1213 | 3 | 3 | 2 | -0.347 | 0.017 | -0.221 | 0.085 | 0.079 | 0.161 | 0.498 | 0.069 | 0.485 | -0.023 | 0.273 | -0.083 | 0.085 | -0.112 | -0.157 | -0.060 | -0.228 | -0.071 | -0.170 | -0.002 | -0.297 | 0.018 |
| Roach | 1214 | 3 | 3 | 2 | -0.358 | 0.015 | -0.217 | 0.086 | 0.069 | 0.165 | 0.500 | 0.063 | 0.490 | -0.026 | 0.277 | -0.070 | 0.064 | -0.114 | -0.152 | -0.060 | -0.226 | -0.077 | -0.163 | -0.001 | -0.285 | 0.020 |
| Roach | 1215 | 3 | 3 | 2 | -0.372 | 0.013 | -0.224 | 0.094 | 0.076 | 0.169 | 0.496 | 0.056 | 0.494 | -0.032 | 0.267 | -0.068 | 0.073 | -0.093 | -0.145 | -0.060 | -0.228 | -0.081 | -0.155 | -0.005 | -0.281 | 0.009 |
| Roach | 1216 | 3 | 3 | 2 | -0.359 | 0.013 | -0.213 | 0.088 | 0.080 | 0.148 | 0.494 | 0.072 | 0.487 | -0.016 | 0.269 | -0.089 | 0.090 | -0.117 | -0.158 | -0.051 | -0.228 | -0.079 | -0.168 | 0.010 | -0.295 | 0.021 |
| Roach | 1217 | 3 | 3 | 2 | -0.387 | -0.017 | -0.237 | 0.097 | 0.092 | 0.183 | 0.497 | 0.040 | 0.473 | -0.048 | 0.275 | -0.054 | 0.082 | -0.081 | -0.132 | -0.049 | -0.230 | -0.089 | -0.146 | 0.013 | -0.287 | 0.003 |
| Roach | 1218 | 3 | 3 | 2 | -0.357 | 0.003 | -0.224 | 0.094 | 0.092 | 0.176 | 0.501 | 0.067 | 0.471 | -0.030 | 0.280 | -0.081 | 0.071 | -0.111 | -0.153 | -0.048 | -0.215 | -0.081 | -0.167 | -0.003 | -0.298 | 0.015 |
| Roach | 1219 | 3 | 3 | 2 | -0.367 | 0.010 | -0.220 | 0.092 | 0.074 | 0.178 | 0.503 | 0.053 | 0.476 | -0.039 | 0.279 | -0.066 | 0.080 | -0.092 | -0.150 | -0.059 | -0.213 | -0.082 | -0.167 | -0.007 | -0.294 | 0.013 |
| Roach | 1220 | 3 | 3 | 2 | -0.368 | 0.012 | -0.230 | 0.088 | 0.096 | 0.161 | 0.484 | 0.070 | 0.493 | -0.022 | 0.272 | -0.087 | 0.070 | -0.111 | -0.149 | -0.056 | -0.222 | -0.073 | -0.155 | 0.005 | -0.290 | 0.014 |
| Roach | 1191 | 3 | 3 | 3 | -0.365 | 0.020 | -0.220 | 0.091 | 0.085 | 0.143 | 0.487 | 0.071 | 0.498 | -0.018 | 0.269 | -0.082 | 0.079 | -0.109 | -0.160 | -0.056 | -0.218 | -0.076 | -0.159 | -0.003 | -0.295 | 0.020 |
| Roach | 1192 | 3 | 3 | 3 | -0.359 | -0.007 | -0.225 | 0.094 | 0.088 | 0.173 | 0.491 | 0.070 | 0.497 | -0.031 | 0.265 | -0.089 | 0.061 | -0.109 | -0.151 | -0.044 | -0.210 | -0.075 | -0.165 | 0.006 | -0.292 | 0.011 |
| Roach | 1193 | 3 | 3 | 3 | -0.350 | 0.000 | -0.216 | 0.087 | 0.075 | 0.181 | 0.495 | 0.060 | 0.498 | -0.036 | 0.264 | -0.074 | 0.074 | -0.102 | -0.159 | -0.048 | -0.229 | -0.077 | -0.163 | -0.005 | -0.290 | 0.013 |
| Roach | 1194 | 3 | 3 | 3 | -0.371 | 0.030 | -0.216 | 0.094 | 0.082 | 0.153 | 0.483 | 0.078 | 0.492 | -0.016 | 0.275 | -0.093 | 0.080 | -0.120 | -0.159 | -0.061 | -0.218 | -0.083 | -0.160 | -0.003 | -0.286 | 0.022 |
| Roach | 1195 | 3 | 3 | 3 | -0.360 | 0.019 | -0.217 | 0.089 | 0.096 | 0.151 | 0.482 | 0.078 | 0.495 | -0.014 | 0.274 | -0.091 | 0.068 | -0.116 | -0.159 | -0.057 | -0.229 | -0.078 | -0.159 | -0.006 | -0.292 | 0.025 |
| Roach | 1196 | 3 | 3 | 3 | -0.367 | 0.008 | -0.215 | 0.097 | 0.110 | 0.165 | 0.498 | 0.066 | 0.469 | -0.020 | 0.277 | -0.076 | 0.070 | -0.110 | -0.160 | -0.058 | -0.217 | -0.081 | -0.164 | -0.008 | -0.301 | 0.017 |
| Roach | 1197 | 3 | 3 | 3 | -0.376 | 0.001 | -0.240 | 0.099 | 0.088 | 0.185 | 0.486 | 0.058 | 0.471 | -0.037 | 0.275 | -0.081 | 0.098 | -0.111 | -0.143 | -0.044 | -0.207 | -0.076 | -0.148 | -0.004 | -0.303 | 0.009 |
| Roach | 1198 | 3 | 3 | 3 | -0.368 | 0.000 | -0.219 | 0.097 | 0.096 | 0.148 | 0.482 | 0.075 | 0.481 | -0.018 | 0.292 | -0.096 | 0.073 | -0.113 | -0.157 | -0.046 | -0.223 | -0.082 | -0.160 | 0.008 | -0.295 | 0.028 |
| Roach | 1199 | 3 | 3 | 3 | -0.356 | 0.006 | -0.215 | 0.088 | 0.077 | 0.153 | 0.500 | 0.070 | 0.502 | -0.021 | 0.258 | -0.082 | 0.064 | -0.113 | -0.158 | -0.053 | -0.222 | -0.076 | -0.163 | 0.011 | -0.288 | 0.016 |
| Roach | 1200 | 3 | 3 | 3 | -0.350 | 0.024 | -0.219 | 0.090 | 0.088 | 0.146 | 0.488 | 0.076 | 0.497 | -0.014 | 0.278 | -0.092 | 0.062 | -0.107 | -0.162 | -0.062 | -0.219 | -0.079 | -0.167 | -0.002 | -0.296 | 0.020 |
| Roach | 1201 | 3 | 3 | 3 | -0.373 | 0.026 | -0.208 | 0.101 | 0.095 | 0.141 | 0.473 | 0.090 | 0.495 | -0.003 | 0.271 | -0.104 | 0.078 | -0.136 | -0.161 | -0.065 | -0.234 | -0.079 | -0.150 | 0.001 | -0.286 | 0.029 |
| Roach | 1202 | 3 | 3 | 3 | -0.361 | -0.026 | -0.244 | 0.097 | 0.112 | 0.189 | 0.486 | 0.064 | 0.484 | -0.038 | 0.260 | -0.090 | 0.080 | -0.102 | -0.154 | -0.041 | -0.208 | -0.082 | -0.157 | 0.020 | -0.296 | 0.012 |
| Roach | 1203 | 3 | 3 | 3 | -0.375 | 0.003 | -0.224 | 0.101 | 0.076 | 0.172 | 0.501 | 0.060 | 0.479 | -0.025 | 0.268 | -0.080 | 0.078 | -0.115 | -0.149 | -0.049 | -0.220 | -0.085 | -0.148 | 0.001 | -0.284 | 0.017 |
| Roach | 1204 | 3 | 3 | 3 | -0.363 | 0.016 | -0.214 | 0.093 | 0.093 | 0.158 | 0.495 | 0.070 | 0.481 | -0.019 | 0.274 | -0.080 | 0.075 | -0.115 | -0.157 | -0.055 | -0.221 | -0.078 | -0.168 | -0.012 | -0.296 | 0.020 |
| Roach | 1205 | 3 | 3 | 3 | -0.376 | 0.023 | -0.217 | 0.094 | 0.076 | 0.151 | 0.491 | 0.073 | 0.480 | -0.022 | 0.280 | -0.079 | 0.083 | -0.123 | -0.149 | -0.059 | -0.230 | -0.080 | -0.150 | 0.003 | -0.288 | 0.020 |
| Roach | 1206 | 3 | 3 | 3 | -0.373 | 0.003 | -0.233 | 0.093 | 0.069 | 0.178 | 0.500 | 0.057 | 0.493 | -0.038 | 0.264 | -0.076 | 0.068 | -0.090 | -0.148 | -0.055 | -0.217 | -0.084 | -0.144 | 0.003 | -0.280 | 0.009 |
| Roach | 1207 | 3 | 3 | 3 | -0.367 | -0.009 | -0.218 | 0.095 | 0.093 | 0.172 | 0.487 | 0.072 | 0.485 | -0.029 | 0.277 | -0.095 | 0.062 | -0.108 | -0.150 | -0.045 | -0.217 | -0.087 | -0.159 | 0.016 | -0.295 | 0.018 |
| Roach | 1208 | 3 | 3 | 3 | -0.359 | -0.021 | -0.236 | 0.087 | 0.086 | 0.186 | 0.509 | 0.049 | 0.484 | -0.041 | 0.259 | -0.071 | 0.071 | -0.092 | -0.155 | -0.035 | -0.200 | -0.077 | -0.161 | 0.009 | -0.299 | 0.007 |
| Roach | 1209 | 3 | 3 | 3 | -0.373 | 0.001 | -0.223 | 0.096 | 0.082 | 0.150 | 0.497 | 0.069 | 0.496 | -0.015 | 0.263 | -0.089 | 0.061 | -0.116 | -0.150 | -0.045 | -0.213 | -0.071 | -0.151 | 0.003 | -0.288 | 0.017 |
| Roach | 1210 | 3 | 3 | 3 | -0.365 | 0.002 | -0.229 | 0.090 | 0.099 | 0.161 | 0.490 | 0.062 | 0.492 | -0.032 | 0.261 | -0.074 | 0.086 | -0.095 | -0.158 | -0.055 | -0.217 | -0.078 | -0.164 | 0.007 | -0.295 | 0.012 |
| Roach | 1211 | 3 | 3 | 3 | -0.379 | 0.032 | -0.208 | 0.094 | 0.099 | 0.122 | 0.478 | 0.086 | 0.488 | -0.002 | 0.294 | -0.092 | 0.057 | -0.125 | -0.159 | -0.060 | -0.231 | -0.087 | -0.152 | 0.004 | -0.288 | 0.028 |
| Roach | 1212 | 3 | 3 | 3 | -0.374 | 0.015 | -0.216 | 0.096 | 0.075 | 0.149 | 0.492 | 0.084 | 0.500 | -0.020 | 0.263 | -0.098 | 0.057 | -0.118 | -0.145 | -0.052 | -0.215 | -0.083 | -0.150 | 0.008 | -0.286 | 0.020 |
| Roach | 1213 | 3 | 3 | 3 | -0.348 | 0.007 | -0.219 | 0.086 | 0.067 | 0.160 | 0.506 | 0.065 | 0.485 | -0.024 | 0.274 | -0.082 | 0.077 | -0.111 | -0.160 | -0.057 | -0.218 | -0.072 | -0.171 | 0.008 | -0.295 | 0.020 |
| Roach | 1214 | 3 | 3 | 3 | -0.359 | 0.014 | -0.217 | 0.085 | 0.067 | 0.163 | 0.504 | 0.061 | 0.490 | -0.028 | 0.275 | -0.067 | 0.065 | -0.111 | -0.154 | -0.057 | -0.222 | -0.079 | -0.161 | -0.002 | -0.288 | 0.021 |
| Roach | 1215 | 3 | 3 | 3 | -0.376 | 0.013 | -0.233 | 0.087 | 0.095 | 0.172 | 0.492 | 0.056 | 0.491 | -0.032 | 0.263 | -0.070 | 0.072 | -0.090 | -0.149 | -0.061 | -0.219 | -0.085 | -0.153 | 0.004 | -0.284 | 0.007 |
| Roach | 1216 | 3 | 3 | 3 | -0.360 | 0.011 | -0.211 | 0.090 | 0.082 | 0.147 | 0.495 | 0.072 | 0.492 | -0.015 | 0.272 | -0.087 | 0.070 | -0.117 | -0.159 | -0.051 | -0.220 | -0.081 | -0.165 | 0.010 | -0.296 | 0.021 |
| Roach | 1217 | 3 | 3 | 3 | -0.389 | -0.013 | -0.231 | 0.102 | 0.079 | 0.183 | 0.501 | 0.037 | 0.474 | -0.048 | 0.274 | -0.053 | 0.082 | -0.079 | -0.139 | -0.049 | -0.214 | -0.090 | -0.147 | 0.006 | -0.290 | 0.003 |
| Roach | 1218 | 3 | 3 | 3 | -0.358 | 0.002 | -0.224 | 0.094 | 0.084 | 0.174 | 0.498 | 0.067 | 0.478 | -0.034 | 0.277 | -0.079 | 0.072 | -0.112 | -0.156 | -0.048 | -0.208 | -0.082 | -0.164 | 0.000 | -0.298 | 0.017 |
| Roach | 1219 | 3 | 3 | 3 | -0.368 | 0.006 | -0.224 | 0.090 | 0.076 | 0.178 | 0.502 | 0.052 | 0.475 | -0.039 | 0.278 | -0.066 | 0.081 | -0.095 | -0.152 | -0.060 | -0.207 | -0.087 | -0.163 | 0.006 | -0.297 | 0.013 |
| Roach | 1220 | 3 | 3 | 3 | -0.370 | 0.010 | -0.230 | 0.090 | 0.108 | 0.163 | 0.478 | 0.070 | 0.492 | -0.021 | 0.272 | -0.087 | 0.071 | -0.110 | -0.153 | -0.054 | -0.223 | -0.075 | -0.154 | 0.002 | -0.292 | 0.014 |
| Prussian carp | 1061 | 1 | 1 | 1 | -0.373 | 0.005 | -0.219 | 0.124 | 0.031 | 0.218 | 0.476 | 0.109 | 0.490 | -0.029 | 0.281 | -0.118 | 0.021 | -0.138 | -0.129 | -0.080 | -0.204 | -0.109 | -0.108 | 0.000 | -0.267 | 0.017 |
| Prussian carp | 1062 | 1 | 1 | 1 | -0.363 | -0.004 | -0.214 | 0.119 | 0.015 | 0.211 | 0.489 | 0.107 | 0.482 | -0.043 | 0.286 | -0.114 | 0.040 | -0.129 | -0.134 | -0.068 | -0.197 | -0.102 | -0.125 | 0.004 | -0.281 | 0.017 |
| Prussian carp | 1063 | 1 | 1 | 1 | -0.353 | 0.006 | -0.221 | 0.107 | 0.042 | 0.214 | 0.481 | 0.104 | 0.491 | -0.036 | 0.278 | -0.106 | 0.044 | -0.138 | -0.137 | -0.069 | -0.200 | -0.104 | -0.142 | 0.007 | -0.284 | 0.014 |
| Prussian carp | 1064 | 1 | 1 | 1 | -0.354 | -0.008 | -0.218 | 0.120 | 0.009 | 0.232 | 0.484 | 0.097 | 0.482 | -0.048 | 0.287 | -0.112 | 0.046 | -0.129 | -0.124 | -0.077 | -0.203 | -0.098 | -0.125 | 0.012 | -0.284 | 0.012 |
| Prussian carp | 1065 | 1 | 1 | 1 | -0.361 | 0.001 | -0.208 | 0.128 | 0.020 | 0.237 | 0.473 | 0.101 | 0.474 | -0.040 | 0.296 | -0.115 | 0.045 | -0.145 | -0.128 | -0.079 | -0.206 | -0.105 | -0.124 | 0.009 | -0.280 | 0.009 |
| Prussian carp | 1066 | 1 | 1 | 1 | -0.371 | 0.004 | -0.218 | 0.127 | 0.031 | 0.213 | 0.464 | 0.107 | 0.489 | -0.036 | 0.292 | -0.122 | 0.049 | -0.129 | -0.130 | -0.081 | -0.207 | -0.099 | -0.122 | 0.001 | -0.276 | 0.015 |
| Prussian carp | 1067 | 1 | 1 | 1 | -0.351 | 0.000 | -0.211 | 0.112 | 0.028 | 0.228 | 0.476 | 0.101 | 0.484 | -0.035 | 0.285 | -0.111 | 0.057 | -0.150 | -0.137 | -0.075 | -0.216 | -0.098 | -0.135 | 0.012 | -0.280 | 0.015 |
| Prussian carp | 1068 | 1 | 1 | 1 | -0.359 | -0.008 | -0.221 | 0.115 | 0.051 | 0.219 | 0.472 | 0.109 | 0.483 | -0.045 | 0.291 | -0.111 | 0.038 | -0.125 | -0.132 | -0.070 | -0.210 | -0.103 | -0.128 | 0.011 | -0.286 | 0.008 |
| Prussian carp | 1069 | 1 | 1 | 1 | -0.359 | 0.010 | -0.217 | 0.129 | 0.030 | 0.214 | 0.467 | 0.114 | 0.488 | -0.041 | 0.288 | -0.115 | 0.044 | -0.142 | -0.133 | -0.082 | -0.206 | -0.107 | -0.121 | 0.007 | -0.282 | 0.011 |
| Prussian carp | 1070 | 1 | 1 | 1 | -0.336 | 0.009 | -0.218 | 0.111 | 0.036 | 0.223 | 0.493 | 0.126 | 0.478 | -0.024 | 0.272 | -0.133 | 0.029 | -0.164 | -0.147 | -0.069 | -0.204 | -0.107 | -0.123 | 0.017 | -0.280 | 0.012 |
| Prussian carp | 1071 | 1 | 1 | 1 | -0.352 | 0.006 | -0.221 | 0.116 | 0.035 | 0.225 | 0.459 | 0.109 | 0.474 | -0.045 | 0.316 | -0.121 | 0.060 | -0.137 | -0.146 | -0.070 | -0.205 | -0.097 | -0.133 | -0.003 | -0.288 | 0.016 |
| Prussian carp | 1072 | 1 | 1 | 1 | -0.375 | -0.005 | -0.216 | 0.128 | 0.025 | 0.220 | 0.471 | 0.100 | 0.469 | -0.049 | 0.305 | -0.110 | 0.061 | -0.126 | -0.126 | -0.067 | -0.205 | -0.105 | -0.123 | 0.000 | -0.286 | 0.015 |
| Prussian carp | 1073 | 1 | 1 | 1 | -0.364 | 0.000 | -0.219 | 0.110 | 0.101 | 0.209 | 0.466 | 0.118 | 0.465 | -0.024 | 0.296 | -0.114 | 0.043 | -0.134 | -0.141 | -0.078 | -0.215 | -0.113 | -0.139 | 0.005 | -0.294 | 0.020 |
| Prussian carp | 1074 | 1 | 1 | 1 | -0.359 | 0.007 | -0.215 | 0.115 | 0.035 | 0.213 | 0.485 | 0.114 | 0.486 | -0.033 | 0.282 | -0.116 | 0.024 | -0.139 | -0.128 | -0.072 | -0.203 | -0.098 | -0.124 | 0.003 | -0.283 | 0.008 |
| Prussian carp | 1075 | 1 | 1 | 1 | -0.345 | 0.004 | -0.211 | 0.115 | 0.057 | 0.234 | 0.458 | 0.116 | 0.470 | -0.041 | 0.315 | -0.116 | 0.047 | -0.148 | -0.147 | -0.080 | -0.210 | -0.100 | -0.146 | -0.002 | -0.287 | 0.018 |
| Prussian carp | 1076 | 1 | 1 | 1 | -0.349 | -0.017 | -0.210 | 0.121 | 0.058 | 0.241 | 0.484 | 0.099 | 0.466 | -0.038 | 0.282 | -0.107 | 0.056 | -0.138 | -0.145 | -0.074 | -0.199 | -0.101 | -0.147 | 0.005 | -0.296 | 0.009 |
| Prussian carp | 1077 | 1 | 1 | 1 | -0.358 | -0.012 | -0.221 | 0.117 | 0.023 | 0.221 | 0.483 | 0.099 | 0.484 | -0.047 | 0.287 | -0.111 | 0.049 | -0.124 | -0.126 | -0.066 | -0.206 | -0.093 | -0.130 | 0.004 | -0.286 | 0.013 |
| Prussian carp | 1078 | 1 | 1 | 1 | -0.344 | 0.013 | -0.226 | 0.120 | 0.062 | 0.242 | 0.469 | 0.109 | 0.467 | -0.037 | 0.287 | -0.118 | 0.058 | -0.144 | -0.140 | -0.086 | -0.188 | -0.109 | -0.148 | -0.003 | -0.298 | 0.013 |
| Prussian carp | 1079 | 1 | 1 | 1 | -0.355 | 0.027 | -0.209 | 0.124 | 0.047 | 0.228 | 0.457 | 0.121 | 0.475 | -0.033 | 0.306 | -0.124 | 0.035 | -0.152 | -0.136 | -0.094 | -0.200 | -0.109 | -0.134 | 0.002 | -0.286 | 0.010 |
| Prussian carp | 1080 | 1 | 1 | 1 | -0.373 | 0.012 | -0.208 | 0.125 | 0.052 | 0.223 | 0.453 | 0.113 | 0.477 | -0.033 | 0.296 | -0.122 | 0.053 | -0.142 | -0.134 | -0.096 | -0.196 | -0.107 | -0.130 | 0.009 | -0.292 | 0.018 |
| Prussian carp | 1081 | 1 | 1 | 1 | -0.374 | 0.019 | -0.212 | 0.120 | 0.033 | 0.216 | 0.474 | 0.125 | 0.476 | -0.027 | 0.288 | -0.127 | 0.025 | -0.157 | -0.117 | -0.075 | -0.218 | -0.112 | -0.106 | 0.003 | -0.269 | 0.016 |
| Prussian carp | 1082 | 1 | 1 | 1 | -0.380 | -0.004 | -0.212 | 0.138 | 0.012 | 0.217 | 0.476 | 0.099 | 0.488 | -0.041 | 0.288 | -0.115 | 0.021 | -0.128 | -0.116 | -0.071 | -0.182 | -0.117 | -0.115 | 0.005 | -0.280 | 0.015 |
| Prussian carp | 1083 | 1 | 1 | 1 | -0.373 | -0.001 | -0.220 | 0.121 | 0.047 | 0.239 | 0.466 | 0.108 | 0.466 | -0.046 | 0.298 | -0.114 | 0.044 | -0.132 | -0.120 | -0.083 | -0.226 | -0.107 | -0.108 | 0.010 | -0.274 | 0.005 |
| Prussian carp | 1084 | 1 | 1 | 1 | -0.367 | -0.006 | -0.237 | 0.123 | 0.044 | 0.245 | 0.478 | 0.095 | 0.476 | -0.051 | 0.278 | -0.103 | 0.043 | -0.126 | -0.119 | -0.076 | -0.187 | -0.106 | -0.123 | -0.003 | -0.286 | 0.009 |
| Prussian carp | 1085 | 1 | 1 | 1 | -0.360 | 0.001 | -0.218 | 0.112 | 0.063 | 0.216 | 0.475 | 0.110 | 0.481 | -0.028 | 0.290 | -0.115 | 0.028 | -0.138 | -0.139 | -0.078 | -0.206 | -0.099 | -0.135 | 0.010 | -0.280 | 0.008 |
| Prussian carp | 1086 | 1 | 1 | 1 | -0.369 | -0.006 | -0.228 | 0.117 | 0.035 | 0.222 | 0.475 | 0.104 | 0.469 | -0.052 | 0.296 | -0.111 | 0.057 | -0.124 | -0.127 | -0.072 | -0.203 | -0.105 | -0.120 | 0.014 | -0.286 | 0.014 |
| Prussian carp | 1087 | 1 | 1 | 1 | -0.343 | -0.001 | -0.219 | 0.112 | 0.045 | 0.235 | 0.472 | 0.104 | 0.480 | -0.037 | 0.292 | -0.110 | 0.044 | -0.145 | -0.137 | -0.082 | -0.218 | -0.098 | -0.132 | 0.010 | -0.283 | 0.012 |
| Prussian carp | 1088 | 1 | 1 | 1 | -0.357 | 0.000 | -0.211 | 0.117 | 0.025 | 0.221 | 0.478 | 0.102 | 0.487 | -0.035 | 0.289 | -0.112 | 0.034 | -0.141 | -0.130 | -0.073 | -0.205 | -0.106 | -0.129 | 0.012 | -0.282 | 0.015 |
| Prussian carp | 1089 | 1 | 1 | 1 | -0.355 | -0.006 | -0.208 | 0.126 | 0.026 | 0.227 | 0.485 | 0.100 | 0.475 | -0.035 | 0.287 | -0.114 | 0.048 | -0.135 | -0.134 | -0.080 | -0.210 | -0.096 | -0.130 | 0.003 | -0.284 | 0.009 |
| Prussian carp | 1090 | 1 | 1 | 1 | -0.351 | -0.008 | -0.226 | 0.118 | 0.036 | 0.227 | 0.468 | 0.096 | 0.480 | -0.047 | 0.292 | -0.108 | 0.072 | -0.133 | -0.131 | -0.073 | -0.213 | -0.095 | -0.138 | 0.012 | -0.289 | 0.012 |
| Prussian carp | 1061 | 1 | 1 | 2 | -0.372 | 0.000 | -0.214 | 0.126 | 0.039 | 0.219 | 0.476 | 0.111 | 0.484 | -0.028 | 0.281 | -0.117 | 0.029 | -0.142 | -0.134 | -0.085 | -0.213 | -0.102 | -0.111 | 0.003 | -0.265 | 0.015 |
| Prussian carp | 1062 | 1 | 1 | 2 | -0.369 | 0.000 | -0.212 | 0.119 | 0.011 | 0.212 | 0.478 | 0.103 | 0.489 | -0.042 | 0.294 | -0.115 | 0.039 | -0.129 | -0.133 | -0.072 | -0.191 | -0.100 | -0.125 | 0.006 | -0.281 | 0.019 |
| Prussian carp | 1063 | 1 | 1 | 2 | -0.350 | 0.002 | -0.221 | 0.108 | 0.037 | 0.214 | 0.482 | 0.099 | 0.488 | -0.035 | 0.285 | -0.107 | 0.047 | -0.134 | -0.140 | -0.072 | -0.201 | -0.099 | -0.139 | 0.011 | -0.287 | 0.013 |
| Prussian carp | 1064 | 1 | 1 | 2 | -0.354 | -0.009 | -0.208 | 0.129 | 0.010 | 0.235 | 0.493 | 0.102 | 0.465 | -0.050 | 0.291 | -0.112 | 0.053 | -0.130 | -0.136 | -0.079 | -0.201 | -0.099 | -0.127 | 0.003 | -0.286 | 0.011 |
| Prussian carp | 1065 | 1 | 1 | 2 | -0.362 | 0.009 | -0.222 | 0.119 | 0.030 | 0.234 | 0.470 | 0.105 | 0.473 | -0.040 | 0.301 | -0.116 | 0.042 | -0.140 | -0.121 | -0.076 | -0.213 | -0.101 | -0.122 | -0.001 | -0.277 | 0.009 |
| Prussian carp | 1066 | 1 | 1 | 2 | -0.372 | 0.008 | -0.235 | 0.119 | 0.034 | 0.209 | 0.472 | 0.107 | 0.488 | -0.037 | 0.285 | -0.120 | 0.049 | -0.129 | -0.129 | -0.078 | -0.194 | -0.098 | -0.121 | 0.001 | -0.276 | 0.018 |
| Prussian carp | 1067 | 1 | 1 | 2 | -0.351 | -0.005 | -0.208 | 0.115 | 0.045 | 0.232 | 0.471 | 0.105 | 0.482 | -0.033 | 0.284 | -0.110 | 0.049 | -0.149 | -0.135 | -0.086 | -0.226 | -0.096 | -0.131 | 0.014 | -0.280 | 0.013 |
| Prussian carp | 1068 | 1 | 1 | 2 | -0.363 | -0.003 | -0.215 | 0.119 | 0.041 | 0.224 | 0.481 | 0.103 | 0.469 | -0.040 | 0.297 | -0.112 | 0.044 | -0.123 | -0.129 | -0.075 | -0.206 | -0.100 | -0.131 | -0.004 | -0.289 | 0.012 |
| Prussian carp | 1069 | 1 | 1 | 2 | -0.356 | 0.009 | -0.218 | 0.126 | 0.033 | 0.213 | 0.465 | 0.110 | 0.490 | -0.041 | 0.295 | -0.113 | 0.037 | -0.133 | -0.137 | -0.088 | -0.192 | -0.108 | -0.132 | 0.008 | -0.285 | 0.017 |
| Prussian carp | 1070 | 1 | 1 | 2 | -0.349 | 0.006 | -0.210 | 0.118 | 0.061 | 0.225 | 0.475 | 0.130 | 0.482 | -0.021 | 0.275 | -0.133 | 0.020 | -0.163 | -0.141 | -0.076 | -0.198 | -0.106 | -0.129 | 0.001 | -0.286 | 0.018 |
| Prussian carp | 1071 | 1 | 1 | 2 | -0.352 | 0.002 | -0.237 | 0.104 | 0.042 | 0.230 | 0.465 | 0.106 | 0.478 | -0.047 | 0.303 | -0.121 | 0.056 | -0.129 | -0.147 | -0.071 | -0.196 | -0.101 | -0.131 | 0.011 | -0.282 | 0.016 |
| Prussian carp | 1072 | 1 | 1 | 2 | -0.378 | -0.007 | -0.221 | 0.133 | 0.032 | 0.227 | 0.459 | 0.095 | 0.467 | -0.049 | 0.308 | -0.116 | 0.065 | -0.130 | -0.122 | -0.077 | -0.191 | -0.109 | -0.128 | 0.022 | -0.290 | 0.011 |
| Prussian carp | 1073 | 1 | 1 | 2 | -0.358 | -0.002 | -0.207 | 0.120 | 0.071 | 0.219 | 0.468 | 0.116 | 0.474 | -0.029 | 0.288 | -0.118 | 0.045 | -0.136 | -0.137 | -0.079 | -0.213 | -0.109 | -0.138 | 0.001 | -0.292 | 0.018 |
| Prussian carp | 1074 | 1 | 1 | 2 | -0.362 | 0.010 | -0.216 | 0.115 | 0.043 | 0.222 | 0.484 | 0.117 | 0.480 | -0.036 | 0.279 | -0.119 | 0.027 | -0.138 | -0.132 | -0.071 | -0.194 | -0.103 | -0.124 | -0.003 | -0.285 | 0.006 |
| Prussian carp | 1075 | 1 | 1 | 2 | -0.337 | 0.001 | -0.212 | 0.118 | 0.047 | 0.224 | 0.459 | 0.119 | 0.479 | -0.043 | 0.310 | -0.115 | 0.044 | -0.149 | -0.145 | -0.088 | -0.213 | -0.101 | -0.145 | 0.015 | -0.286 | 0.020 |
| Prussian carp | 1076 | 1 | 1 | 2 | -0.350 | -0.015 | -0.217 | 0.123 | 0.051 | 0.242 | 0.486 | 0.099 | 0.465 | -0.038 | 0.286 | -0.110 | 0.043 | -0.131 | -0.133 | -0.087 | -0.199 | -0.099 | -0.142 | 0.002 | -0.291 | 0.014 |
| Prussian carp | 1077 | 1 | 1 | 2 | -0.356 | -0.013 | -0.219 | 0.112 | 0.034 | 0.223 | 0.494 | 0.103 | 0.465 | -0.042 | 0.295 | -0.110 | 0.046 | -0.124 | -0.128 | -0.070 | -0.224 | -0.089 | -0.124 | -0.002 | -0.284 | 0.012 |
| Prussian carp | 1078 | 1 | 1 | 2 | -0.344 | 0.014 | -0.228 | 0.119 | 0.081 | 0.242 | 0.464 | 0.113 | 0.467 | -0.035 | 0.283 | -0.114 | 0.061 | -0.145 | -0.141 | -0.090 | -0.188 | -0.111 | -0.152 | -0.005 | -0.302 | 0.013 |
| Prussian carp | 1079 | 1 | 1 | 2 | -0.358 | 0.022 | -0.211 | 0.125 | 0.049 | 0.228 | 0.459 | 0.117 | 0.472 | -0.031 | 0.305 | -0.124 | 0.037 | -0.148 | -0.135 | -0.091 | -0.197 | -0.108 | -0.129 | 0.000 | -0.292 | 0.010 |
| Prussian carp | 1080 | 1 | 1 | 2 | -0.369 | 0.011 | -0.203 | 0.121 | 0.048 | 0.220 | 0.455 | 0.115 | 0.485 | -0.033 | 0.297 | -0.121 | 0.040 | -0.144 | -0.134 | -0.082 | -0.202 | -0.107 | -0.127 | 0.005 | -0.290 | 0.016 |
| Prussian carp | 1081 | 1 | 1 | 2 | -0.375 | 0.016 | -0.211 | 0.125 | 0.030 | 0.216 | 0.457 | 0.113 | 0.496 | -0.022 | 0.285 | -0.128 | 0.029 | -0.158 | -0.115 | -0.079 | -0.212 | -0.112 | -0.115 | 0.012 | -0.268 | 0.018 |
| Prussian carp | 1082 | 1 | 1 | 2 | -0.374 | -0.004 | -0.214 | 0.131 | 0.011 | 0.215 | 0.471 | 0.099 | 0.490 | -0.043 | 0.298 | -0.112 | 0.024 | -0.127 | -0.118 | -0.072 | -0.198 | -0.110 | -0.114 | 0.007 | -0.277 | 0.016 |
| Prussian carp | 1083 | 1 | 1 | 2 | -0.375 | -0.002 | -0.217 | 0.130 | 0.037 | 0.241 | 0.462 | 0.107 | 0.465 | -0.047 | 0.302 | -0.117 | 0.043 | -0.138 | -0.121 | -0.084 | -0.210 | -0.107 | -0.108 | 0.010 | -0.278 | 0.006 |
| Prussian carp | 1084 | 1 | 1 | 2 | -0.367 | -0.008 | -0.232 | 0.119 | 0.068 | 0.247 | 0.474 | 0.100 | 0.469 | -0.045 | 0.281 | -0.106 | 0.038 | -0.126 | -0.124 | -0.082 | -0.194 | -0.109 | -0.122 | 0.008 | -0.291 | 0.003 |
| Prussian carp | 1085 | 1 | 1 | 2 | -0.359 | 0.007 | -0.216 | 0.113 | 0.044 | 0.210 | 0.475 | 0.111 | 0.489 | -0.030 | 0.290 | -0.118 | 0.028 | -0.136 | -0.138 | -0.077 | -0.197 | -0.102 | -0.136 | 0.010 | -0.281 | 0.012 |
| Prussian carp | 1086 | 1 | 1 | 2 | -0.371 | -0.009 | -0.211 | 0.129 | 0.034 | 0.223 | 0.470 | 0.108 | 0.477 | -0.051 | 0.293 | -0.112 | 0.045 | -0.127 | -0.130 | -0.073 | -0.197 | -0.110 | -0.119 | 0.010 | -0.291 | 0.011 |
| Prussian carp | 1087 | 1 | 1 | 2 | -0.346 | -0.003 | -0.210 | 0.117 | 0.041 | 0.239 | 0.475 | 0.102 | 0.475 | -0.037 | 0.294 | -0.109 | 0.047 | -0.146 | -0.139 | -0.075 | -0.214 | -0.099 | -0.137 | -0.001 | -0.285 | 0.012 |
| Prussian carp | 1088 | 1 | 1 | 2 | -0.353 | -0.002 | -0.208 | 0.124 | 0.022 | 0.222 | 0.483 | 0.097 | 0.473 | -0.038 | 0.299 | -0.106 | 0.041 | -0.140 | -0.123 | -0.073 | -0.213 | -0.106 | -0.133 | 0.009 | -0.289 | 0.013 |
| Prussian carp | 1089 | 1 | 1 | 2 | -0.351 | -0.005 | -0.215 | 0.122 | 0.025 | 0.227 | 0.493 | 0.098 | 0.471 | -0.033 | 0.286 | -0.114 | 0.053 | -0.134 | -0.137 | -0.074 | -0.213 | -0.090 | -0.131 | -0.009 | -0.282 | 0.011 |
| Prussian carp | 1090 | 1 | 1 | 2 | -0.357 | 0.001 | -0.212 | 0.122 | 0.027 | 0.235 | 0.470 | 0.097 | 0.465 | -0.049 | 0.313 | -0.108 | 0.061 | -0.134 | -0.135 | -0.079 | -0.204 | -0.098 | -0.135 | 0.004 | -0.293 | 0.008 |
| Prussian carp | 1061 | 1 | 1 | 3 | -0.372 | 0.002 | -0.212 | 0.130 | 0.022 | 0.214 | 0.472 | 0.111 | 0.483 | -0.034 | 0.293 | -0.121 | 0.034 | -0.139 | -0.128 | -0.080 | -0.214 | -0.104 | -0.107 | 0.004 | -0.272 | 0.016 |
| Prussian carp | 1062 | 1 | 1 | 3 | -0.364 | -0.004 | -0.204 | 0.124 | 0.008 | 0.211 | 0.485 | 0.102 | 0.487 | -0.040 | 0.291 | -0.112 | 0.040 | -0.129 | -0.140 | -0.070 | -0.201 | -0.100 | -0.125 | 0.002 | -0.279 | 0.016 |
| Prussian carp | 1063 | 1 | 1 | 3 | -0.351 | -0.001 | -0.217 | 0.115 | 0.041 | 0.211 | 0.483 | 0.099 | 0.488 | -0.032 | 0.280 | -0.106 | 0.050 | -0.135 | -0.136 | -0.074 | -0.209 | -0.100 | -0.143 | 0.009 | -0.285 | 0.013 |
| Prussian carp | 1064 | 1 | 1 | 3 | -0.356 | -0.009 | -0.215 | 0.126 | 0.016 | 0.231 | 0.489 | 0.094 | 0.475 | -0.044 | 0.285 | -0.110 | 0.051 | -0.130 | -0.125 | -0.073 | -0.195 | -0.101 | -0.135 | 0.007 | -0.289 | 0.010 |
| Prussian carp | 1065 | 1 | 1 | 3 | -0.365 | 0.005 | -0.208 | 0.130 | 0.025 | 0.228 | 0.475 | 0.101 | 0.469 | -0.037 | 0.294 | -0.115 | 0.062 | -0.142 | -0.131 | -0.072 | -0.207 | -0.110 | -0.131 | 0.002 | -0.283 | 0.009 |
| Prussian carp | 1066 | 1 | 1 | 3 | -0.369 | 0.006 | -0.221 | 0.125 | 0.035 | 0.215 | 0.470 | 0.103 | 0.486 | -0.028 | 0.289 | -0.119 | 0.046 | -0.136 | -0.130 | -0.076 | -0.206 | -0.098 | -0.124 | -0.008 | -0.277 | 0.016 |
| Prussian carp | 1067 | 1 | 1 | 3 | -0.354 | -0.002 | -0.209 | 0.116 | 0.045 | 0.232 | 0.474 | 0.101 | 0.478 | -0.029 | 0.284 | -0.110 | 0.056 | -0.152 | -0.136 | -0.079 | -0.212 | -0.105 | -0.145 | 0.010 | -0.281 | 0.018 |
| Prussian carp | 1068 | 1 | 1 | 3 | -0.364 | 0.001 | -0.218 | 0.116 | 0.039 | 0.223 | 0.478 | 0.101 | 0.469 | -0.038 | 0.302 | -0.115 | 0.043 | -0.126 | -0.126 | -0.073 | -0.203 | -0.103 | -0.132 | 0.005 | -0.289 | 0.009 |
| Prussian carp | 1069 | 1 | 1 | 3 | -0.356 | 0.008 | -0.210 | 0.130 | 0.029 | 0.214 | 0.464 | 0.107 | 0.486 | -0.037 | 0.303 | -0.114 | 0.041 | -0.137 | -0.132 | -0.086 | -0.207 | -0.102 | -0.134 | 0.003 | -0.285 | 0.014 |
| Prussian carp | 1070 | 1 | 1 | 3 | -0.339 | 0.004 | -0.213 | 0.118 | 0.044 | 0.218 | 0.483 | 0.123 | 0.492 | -0.019 | 0.267 | -0.134 | 0.025 | -0.160 | -0.140 | -0.066 | -0.202 | -0.104 | -0.133 | 0.003 | -0.285 | 0.015 |
| Prussian carp | 1071 | 1 | 1 | 3 | -0.351 | 0.002 | -0.214 | 0.120 | 0.037 | 0.226 | 0.460 | 0.105 | 0.472 | -0.041 | 0.315 | -0.119 | 0.057 | -0.141 | -0.142 | -0.074 | -0.201 | -0.099 | -0.141 | 0.007 | -0.292 | 0.015 |
| Prussian carp | 1072 | 1 | 1 | 3 | -0.375 | -0.007 | -0.221 | 0.129 | 0.028 | 0.222 | 0.473 | 0.097 | 0.466 | -0.048 | 0.303 | -0.109 | 0.057 | -0.130 | -0.115 | -0.075 | -0.200 | -0.105 | -0.128 | 0.010 | -0.288 | 0.014 |
| Prussian carp | 1073 | 1 | 1 | 3 | -0.358 | 0.001 | -0.214 | 0.116 | 0.058 | 0.211 | 0.468 | 0.113 | 0.471 | -0.031 | 0.299 | -0.118 | 0.055 | -0.141 | -0.136 | -0.074 | -0.217 | -0.109 | -0.136 | 0.011 | -0.290 | 0.021 |
| Prussian carp | 1074 | 1 | 1 | 3 | -0.361 | 0.008 | -0.212 | 0.114 | 0.037 | 0.216 | 0.488 | 0.109 | 0.478 | -0.028 | 0.285 | -0.117 | 0.030 | -0.139 | -0.129 | -0.071 | -0.201 | -0.100 | -0.128 | -0.002 | -0.286 | 0.010 |
| Prussian carp | 1075 | 1 | 1 | 3 | -0.345 | 0.003 | -0.214 | 0.114 | 0.056 | 0.226 | 0.461 | 0.114 | 0.474 | -0.041 | 0.308 | -0.115 | 0.049 | -0.148 | -0.151 | -0.077 | -0.197 | -0.104 | -0.153 | 0.013 | -0.288 | 0.015 |
| Prussian carp | 1076 | 1 | 1 | 3 | -0.353 | -0.016 | -0.217 | 0.120 | 0.040 | 0.249 | 0.473 | 0.097 | 0.474 | -0.044 | 0.289 | -0.114 | 0.055 | -0.137 | -0.129 | -0.074 | -0.203 | -0.096 | -0.143 | -0.001 | -0.286 | 0.016 |
| Prussian carp | 1077 | 1 | 1 | 3 | -0.357 | -0.014 | -0.216 | 0.116 | 0.029 | 0.223 | 0.490 | 0.101 | 0.477 | -0.043 | 0.284 | -0.112 | 0.047 | -0.127 | -0.130 | -0.062 | -0.209 | -0.097 | -0.130 | 0.007 | -0.286 | 0.008 |
| Prussian carp | 1078 | 1 | 1 | 3 | -0.348 | 0.013 | -0.223 | 0.121 | 0.057 | 0.243 | 0.464 | 0.109 | 0.469 | -0.040 | 0.286 | -0.117 | 0.070 | -0.146 | -0.144 | -0.087 | -0.185 | -0.111 | -0.145 | 0.000 | -0.300 | 0.015 |
| Prussian carp | 1079 | 1 | 1 | 3 | -0.354 | 0.026 | -0.218 | 0.125 | 0.052 | 0.229 | 0.467 | 0.112 | 0.473 | -0.025 | 0.300 | -0.121 | 0.027 | -0.148 | -0.136 | -0.089 | -0.193 | -0.109 | -0.127 | -0.008 | -0.290 | 0.007 |
| Prussian carp | 1080 | 1 | 1 | 3 | -0.366 | 0.004 | -0.208 | 0.124 | 0.047 | 0.220 | 0.458 | 0.113 | 0.486 | -0.033 | 0.293 | -0.122 | 0.041 | -0.143 | -0.134 | -0.080 | -0.198 | -0.108 | -0.132 | 0.005 | -0.286 | 0.019 |
| Prussian carp | 1081 | 1 | 1 | 3 | -0.378 | 0.025 | -0.223 | 0.121 | 0.026 | 0.214 | 0.463 | 0.116 | 0.489 | -0.020 | 0.282 | -0.131 | 0.031 | -0.162 | -0.107 | -0.077 | -0.205 | -0.112 | -0.108 | 0.005 | -0.271 | 0.023 |
| Prussian carp | 1082 | 1 | 1 | 3 | -0.373 | -0.004 | -0.204 | 0.141 | 0.008 | 0.216 | 0.479 | 0.103 | 0.489 | -0.042 | 0.287 | -0.114 | 0.027 | -0.124 | -0.120 | -0.076 | -0.200 | -0.110 | -0.115 | -0.002 | -0.278 | 0.013 |
| Prussian carp | 1083 | 1 | 1 | 3 | -0.376 | 0.004 | -0.214 | 0.126 | 0.033 | 0.242 | 0.465 | 0.110 | 0.472 | -0.047 | 0.298 | -0.119 | 0.035 | -0.136 | -0.123 | -0.078 | -0.201 | -0.107 | -0.114 | -0.003 | -0.276 | 0.008 |
| Prussian carp | 1084 | 1 | 1 | 3 | -0.364 | -0.006 | -0.233 | 0.121 | 0.057 | 0.243 | 0.468 | 0.094 | 0.474 | -0.046 | 0.284 | -0.107 | 0.054 | -0.124 | -0.125 | -0.073 | -0.197 | -0.107 | -0.127 | 0.004 | -0.292 | 0.004 |
| Prussian carp | 1085 | 1 | 1 | 3 | -0.360 | 0.006 | -0.222 | 0.105 | 0.051 | 0.211 | 0.482 | 0.103 | 0.482 | -0.026 | 0.297 | -0.110 | 0.027 | -0.134 | -0.138 | -0.073 | -0.200 | -0.096 | -0.137 | 0.004 | -0.281 | 0.011 |
| Prussian carp | 1086 | 1 | 1 | 3 | -0.371 | -0.007 | -0.214 | 0.130 | 0.028 | 0.215 | 0.474 | 0.106 | 0.474 | -0.051 | 0.298 | -0.107 | 0.049 | -0.131 | -0.126 | -0.064 | -0.197 | -0.105 | -0.125 | -0.001 | -0.290 | 0.016 |
| Prussian carp | 1087 | 1 | 1 | 3 | -0.344 | -0.007 | -0.213 | 0.112 | 0.037 | 0.236 | 0.478 | 0.096 | 0.474 | -0.036 | 0.299 | -0.105 | 0.047 | -0.148 | -0.142 | -0.075 | -0.214 | -0.093 | -0.136 | 0.004 | -0.286 | 0.015 |
| Prussian carp | 1088 | 1 | 1 | 3 | -0.349 | 0.002 | -0.217 | 0.114 | 0.023 | 0.223 | 0.475 | 0.098 | 0.490 | -0.034 | 0.289 | -0.114 | 0.049 | -0.144 | -0.126 | -0.067 | -0.211 | -0.102 | -0.140 | 0.009 | -0.282 | 0.015 |
| Prussian carp | 1089 | 1 | 1 | 3 | -0.356 | -0.005 | -0.212 | 0.116 | 0.028 | 0.230 | 0.492 | 0.098 | 0.469 | -0.034 | 0.292 | -0.114 | 0.045 | -0.133 | -0.137 | -0.071 | -0.202 | -0.093 | -0.134 | -0.004 | -0.285 | 0.011 |
| Prussian carp | 1090 | 1 | 1 | 3 | -0.357 | -0.002 | -0.215 | 0.121 | 0.024 | 0.228 | 0.474 | 0.095 | 0.474 | -0.046 | 0.293 | -0.110 | 0.081 | -0.130 | -0.129 | -0.072 | -0.208 | -0.094 | -0.144 | -0.003 | -0.292 | 0.012 |
| Prussian carp | 1161 | 1 | 2 | 1 | -0.388 | 0.006 | -0.235 | 0.122 | 0.034 | 0.218 | 0.473 | 0.101 | 0.470 | -0.040 | 0.287 | -0.107 | 0.054 | -0.142 | -0.109 | -0.085 | -0.199 | -0.103 | -0.108 | 0.009 | -0.279 | 0.023 |
| Prussian carp | 1162 | 1 | 2 | 1 | -0.389 | 0.016 | -0.212 | 0.131 | 0.031 | 0.196 | 0.464 | 0.108 | 0.488 | -0.038 | 0.288 | -0.101 | 0.049 | -0.141 | -0.120 | -0.084 | -0.204 | -0.105 | -0.107 | 0.001 | -0.286 | 0.018 |
| Prussian carp | 1163 | 1 | 2 | 1 | -0.371 | 0.004 | -0.248 | 0.112 | 0.038 | 0.207 | 0.465 | 0.092 | 0.482 | -0.049 | 0.295 | -0.099 | 0.068 | -0.128 | -0.125 | -0.077 | -0.192 | -0.102 | -0.121 | 0.022 | -0.292 | 0.018 |
| Prussian carp | 1164 | 1 | 2 | 1 | -0.385 | -0.002 | -0.219 | 0.132 | 0.017 | 0.230 | 0.467 | 0.094 | 0.483 | -0.043 | 0.278 | -0.102 | 0.057 | -0.144 | -0.122 | -0.082 | -0.210 | -0.107 | -0.091 | 0.010 | -0.275 | 0.015 |
| Prussian carp | 1165 | 1 | 2 | 1 | -0.371 | 0.006 | -0.201 | 0.136 | 0.015 | 0.219 | 0.470 | 0.110 | 0.479 | -0.037 | 0.288 | -0.117 | 0.049 | -0.151 | -0.124 | -0.087 | -0.200 | -0.107 | -0.118 | 0.007 | -0.287 | 0.021 |
| Prussian carp | 1166 | 1 | 2 | 1 | -0.374 | -0.009 | -0.224 | 0.113 | 0.044 | 0.192 | 0.481 | 0.094 | 0.487 | -0.033 | 0.291 | -0.097 | 0.035 | -0.126 | -0.127 | -0.064 | -0.202 | -0.101 | -0.122 | 0.009 | -0.289 | 0.020 |
| Prussian carp | 1167 | 1 | 2 | 1 | -0.397 | 0.007 | -0.225 | 0.130 | 0.058 | 0.207 | 0.455 | 0.108 | 0.473 | -0.028 | 0.289 | -0.114 | 0.051 | -0.151 | -0.114 | -0.082 | -0.199 | -0.111 | -0.105 | 0.011 | -0.287 | 0.023 |
| Prussian carp | 1168 | 1 | 2 | 1 | -0.371 | 0.007 | -0.211 | 0.119 | 0.028 | 0.205 | 0.478 | 0.103 | 0.479 | -0.036 | 0.296 | -0.108 | 0.043 | -0.135 | -0.131 | -0.075 | -0.194 | -0.102 | -0.124 | 0.005 | -0.294 | 0.017 |
| Prussian carp | 1169 | 1 | 2 | 1 | -0.386 | 0.001 | -0.231 | 0.127 | 0.044 | 0.233 | 0.459 | 0.099 | 0.468 | -0.041 | 0.290 | -0.114 | 0.060 | -0.142 | -0.103 | -0.076 | -0.202 | -0.110 | -0.109 | 0.002 | -0.289 | 0.019 |
| Prussian carp | 1170 | 1 | 2 | 1 | -0.381 | 0.011 | -0.233 | 0.112 | 0.043 | 0.219 | 0.459 | 0.110 | 0.475 | -0.037 | 0.294 | -0.116 | 0.049 | -0.146 | -0.116 | -0.072 | -0.213 | -0.113 | -0.092 | 0.005 | -0.284 | 0.026 |
| Prussian carp | 1171 | 1 | 2 | 1 | -0.385 | -0.003 | -0.217 | 0.121 | 0.053 | 0.220 | 0.454 | 0.105 | 0.475 | -0.039 | 0.304 | -0.108 | 0.047 | -0.137 | -0.118 | -0.079 | -0.231 | -0.106 | -0.106 | 0.010 | -0.276 | 0.016 |
| Prussian carp | 1172 | 1 | 2 | 1 | -0.383 | 0.004 | -0.226 | 0.114 | 0.037 | 0.222 | 0.473 | 0.098 | 0.479 | -0.052 | 0.288 | -0.096 | 0.048 | -0.127 | -0.118 | -0.080 | -0.189 | -0.105 | -0.124 | 0.009 | -0.286 | 0.013 |
| Prussian carp | 1173 | 1 | 2 | 1 | -0.380 | 0.012 | -0.233 | 0.105 | 0.058 | 0.211 | 0.470 | 0.110 | 0.492 | -0.037 | 0.276 | -0.103 | 0.028 | -0.135 | -0.121 | -0.079 | -0.196 | -0.104 | -0.113 | 0.001 | -0.282 | 0.018 |
| Prussian carp | 1174 | 1 | 2 | 1 | -0.377 | -0.002 | -0.248 | 0.118 | 0.017 | 0.236 | 0.470 | 0.092 | 0.477 | -0.053 | 0.288 | -0.109 | 0.055 | -0.139 | -0.105 | -0.075 | -0.190 | -0.097 | -0.109 | 0.014 | -0.279 | 0.015 |
| Prussian carp | 1175 | 1 | 2 | 1 | -0.378 | 0.011 | -0.220 | 0.122 | 0.035 | 0.214 | 0.468 | 0.105 | 0.481 | -0.039 | 0.291 | -0.105 | 0.051 | -0.139 | -0.126 | -0.079 | -0.205 | -0.101 | -0.118 | -0.010 | -0.279 | 0.021 |
| Prussian carp | 1176 | 1 | 2 | 1 | -0.371 | 0.011 | -0.217 | 0.116 | 0.027 | 0.203 | 0.475 | 0.107 | 0.491 | -0.009 | 0.284 | -0.123 | 0.022 | -0.166 | -0.130 | -0.080 | -0.206 | -0.101 | -0.105 | 0.008 | -0.271 | 0.032 |
| Prussian carp | 1177 | 1 | 2 | 1 | -0.381 | 0.007 | -0.205 | 0.128 | 0.038 | 0.208 | 0.459 | 0.114 | 0.490 | -0.031 | 0.283 | -0.116 | 0.041 | -0.148 | -0.119 | -0.082 | -0.208 | -0.106 | -0.106 | 0.001 | -0.290 | 0.024 |
| Prussian carp | 1178 | 1 | 2 | 1 | -0.369 | 0.015 | -0.219 | 0.124 | 0.019 | 0.198 | 0.463 | 0.100 | 0.474 | -0.043 | 0.305 | -0.105 | 0.085 | -0.146 | -0.126 | -0.075 | -0.196 | -0.100 | -0.135 | 0.007 | -0.303 | 0.024 |
| Prussian carp | 1179 | 1 | 2 | 1 | -0.384 | -0.010 | -0.218 | 0.120 | 0.027 | 0.211 | 0.475 | 0.099 | 0.485 | -0.046 | 0.286 | -0.103 | 0.046 | -0.130 | -0.124 | -0.067 | -0.189 | -0.101 | -0.115 | 0.005 | -0.288 | 0.022 |
| Prussian carp | 1180 | 1 | 2 | 1 | -0.374 | 0.042 | -0.205 | 0.120 | 0.027 | 0.199 | 0.474 | 0.112 | 0.480 | -0.022 | 0.294 | -0.106 | 0.047 | -0.155 | -0.138 | -0.099 | -0.199 | -0.099 | -0.120 | -0.011 | -0.284 | 0.018 |
| Prussian carp | 1181 | 1 | 2 | 1 | -0.380 | -0.005 | -0.221 | 0.114 | 0.057 | 0.219 | 0.462 | 0.107 | 0.475 | -0.042 | 0.290 | -0.112 | 0.059 | -0.142 | -0.119 | -0.066 | -0.197 | -0.105 | -0.135 | 0.013 | -0.292 | 0.019 |
| Prussian carp | 1182 | 1 | 2 | 1 | -0.386 | 0.016 | -0.219 | 0.119 | 0.032 | 0.184 | 0.468 | 0.111 | 0.481 | -0.029 | 0.298 | -0.108 | 0.047 | -0.143 | -0.110 | -0.082 | -0.223 | -0.108 | -0.110 | 0.005 | -0.278 | 0.034 |
| Prussian carp | 1183 | 1 | 2 | 1 | -0.374 | -0.004 | -0.233 | 0.115 | 0.076 | 0.212 | 0.464 | 0.106 | 0.474 | -0.040 | 0.302 | -0.097 | 0.029 | -0.136 | -0.141 | -0.077 | -0.196 | -0.108 | -0.110 | 0.012 | -0.290 | 0.016 |
| Prussian carp | 1184 | 1 | 2 | 1 | -0.381 | 0.019 | -0.216 | 0.117 | 0.029 | 0.193 | 0.464 | 0.107 | 0.474 | -0.028 | 0.305 | -0.119 | 0.055 | -0.141 | -0.115 | -0.083 | -0.207 | -0.106 | -0.112 | 0.015 | -0.297 | 0.026 |
| Prussian carp | 1185 | 1 | 2 | 1 | -0.384 | 0.012 | -0.215 | 0.123 | 0.046 | 0.203 | 0.458 | 0.098 | 0.459 | -0.032 | 0.315 | -0.114 | 0.092 | -0.132 | -0.147 | -0.089 | -0.205 | -0.099 | -0.127 | 0.014 | -0.292 | 0.015 |
| Prussian carp | 1186 | 1 | 2 | 1 | -0.402 | -0.004 | -0.219 | 0.134 | 0.042 | 0.227 | 0.455 | 0.107 | 0.478 | -0.042 | 0.288 | -0.121 | 0.041 | -0.133 | -0.109 | -0.082 | -0.191 | -0.113 | -0.104 | 0.010 | -0.279 | 0.017 |
| Prussian carp | 1187 | 1 | 2 | 1 | -0.387 | -0.001 | -0.224 | 0.125 | 0.040 | 0.211 | 0.473 | 0.104 | 0.474 | -0.050 | 0.280 | -0.102 | 0.069 | -0.127 | -0.123 | -0.075 | -0.200 | -0.113 | -0.118 | 0.009 | -0.285 | 0.019 |
| Prussian carp | 1188 | 1 | 2 | 1 | -0.369 | 0.005 | -0.230 | 0.119 | 0.065 | 0.222 | 0.464 | 0.101 | 0.455 | -0.034 | 0.306 | -0.108 | 0.068 | -0.141 | -0.134 | -0.085 | -0.201 | -0.107 | -0.128 | 0.003 | -0.297 | 0.025 |
| Prussian carp | 1189 | 1 | 2 | 1 | -0.374 | 0.018 | -0.238 | 0.116 | 0.042 | 0.223 | 0.474 | 0.106 | 0.474 | -0.035 | 0.277 | -0.110 | 0.051 | -0.139 | -0.110 | -0.092 | -0.204 | -0.109 | -0.107 | -0.009 | -0.285 | 0.030 |
| Prussian carp | 1190 | 1 | 2 | 1 | -0.371 | -0.004 | -0.220 | 0.113 | 0.047 | 0.201 | 0.473 | 0.102 | 0.475 | -0.046 | 0.312 | -0.092 | 0.035 | -0.129 | -0.125 | -0.077 | -0.196 | -0.100 | -0.139 | 0.006 | -0.291 | 0.026 |
| Prussian carp | 1161 | 1 | 2 | 2 | -0.387 | 0.002 | -0.224 | 0.129 | 0.025 | 0.215 | 0.465 | 0.099 | 0.479 | -0.039 | 0.291 | -0.110 | 0.051 | -0.142 | -0.118 | -0.084 | -0.201 | -0.105 | -0.102 | 0.015 | -0.279 | 0.020 |
| Prussian carp | 1162 | 1 | 2 | 2 | -0.390 | 0.018 | -0.213 | 0.126 | 0.031 | 0.198 | 0.471 | 0.107 | 0.479 | -0.036 | 0.287 | -0.102 | 0.056 | -0.136 | -0.124 | -0.086 | -0.207 | -0.106 | -0.106 | -0.005 | -0.285 | 0.021 |
| Prussian carp | 1163 | 1 | 2 | 2 | -0.372 | -0.002 | -0.239 | 0.120 | 0.047 | 0.212 | 0.461 | 0.095 | 0.478 | -0.046 | 0.297 | -0.101 | 0.069 | -0.134 | -0.131 | -0.078 | -0.191 | -0.102 | -0.127 | 0.017 | -0.292 | 0.020 |
| Prussian carp | 1164 | 1 | 2 | 2 | -0.385 | -0.009 | -0.221 | 0.124 | 0.030 | 0.229 | 0.474 | 0.097 | 0.483 | -0.040 | 0.272 | -0.105 | 0.046 | -0.139 | -0.116 | -0.082 | -0.211 | -0.105 | -0.095 | 0.012 | -0.277 | 0.017 |
| Prussian carp | 1165 | 1 | 2 | 2 | -0.372 | 0.006 | -0.202 | 0.136 | 0.033 | 0.223 | 0.468 | 0.110 | 0.473 | -0.032 | 0.290 | -0.115 | 0.043 | -0.153 | -0.127 | -0.088 | -0.198 | -0.105 | -0.115 | -0.001 | -0.293 | 0.019 |
| Prussian carp | 1166 | 1 | 2 | 2 | -0.372 | -0.004 | -0.233 | 0.111 | 0.034 | 0.196 | 0.477 | 0.092 | 0.491 | -0.036 | 0.289 | -0.100 | 0.044 | -0.130 | -0.118 | -0.067 | -0.199 | -0.100 | -0.124 | 0.012 | -0.288 | 0.026 |
| Prussian carp | 1167 | 1 | 2 | 2 | -0.400 | 0.006 | -0.216 | 0.133 | 0.074 | 0.210 | 0.453 | 0.114 | 0.472 | -0.028 | 0.286 | -0.111 | 0.047 | -0.147 | -0.120 | -0.085 | -0.198 | -0.113 | -0.110 | -0.001 | -0.288 | 0.023 |
| Prussian carp | 1168 | 1 | 2 | 2 | -0.370 | 0.012 | -0.218 | 0.119 | 0.019 | 0.202 | 0.480 | 0.101 | 0.480 | -0.037 | 0.297 | -0.110 | 0.045 | -0.132 | -0.129 | -0.075 | -0.188 | -0.102 | -0.123 | 0.004 | -0.293 | 0.018 |
| Prussian carp | 1169 | 1 | 2 | 2 | -0.380 | 0.006 | -0.242 | 0.113 | 0.048 | 0.235 | 0.459 | 0.096 | 0.470 | -0.043 | 0.298 | -0.113 | 0.054 | -0.136 | -0.115 | -0.073 | -0.194 | -0.107 | -0.110 | 0.009 | -0.288 | 0.012 |
| Prussian carp | 1170 | 1 | 2 | 2 | -0.378 | 0.010 | -0.239 | 0.108 | 0.042 | 0.220 | 0.460 | 0.107 | 0.475 | -0.038 | 0.300 | -0.115 | 0.045 | -0.144 | -0.115 | -0.073 | -0.208 | -0.106 | -0.099 | 0.002 | -0.283 | 0.029 |
| Prussian carp | 1171 | 1 | 2 | 2 | -0.383 | 0.002 | -0.226 | 0.127 | 0.023 | 0.213 | 0.467 | 0.100 | 0.478 | -0.041 | 0.299 | -0.114 | 0.043 | -0.130 | -0.115 | -0.087 | -0.203 | -0.111 | -0.108 | 0.018 | -0.275 | 0.023 |
| Prussian carp | 1172 | 1 | 2 | 2 | -0.381 | 0.001 | -0.233 | 0.120 | 0.031 | 0.223 | 0.469 | 0.093 | 0.479 | -0.052 | 0.297 | -0.095 | 0.049 | -0.129 | -0.121 | -0.072 | -0.189 | -0.100 | -0.115 | -0.003 | -0.286 | 0.015 |
| Prussian carp | 1173 | 1 | 2 | 2 | -0.369 | 0.012 | -0.219 | 0.108 | 0.052 | 0.208 | 0.484 | 0.116 | 0.500 | -0.036 | 0.259 | -0.099 | 0.017 | -0.141 | -0.118 | -0.082 | -0.199 | -0.098 | -0.120 | 0.003 | -0.286 | 0.009 |
| Prussian carp | 1174 | 1 | 2 | 2 | -0.376 | -0.009 | -0.259 | 0.119 | 0.046 | 0.237 | 0.467 | 0.092 | 0.475 | -0.046 | 0.282 | -0.106 | 0.056 | -0.140 | -0.111 | -0.073 | -0.195 | -0.099 | -0.104 | 0.008 | -0.281 | 0.017 |
| Prussian carp | 1175 | 1 | 2 | 2 | -0.374 | 0.006 | -0.225 | 0.113 | 0.031 | 0.207 | 0.469 | 0.107 | 0.488 | -0.041 | 0.290 | -0.106 | 0.041 | -0.142 | -0.116 | -0.080 | -0.211 | -0.096 | -0.113 | 0.012 | -0.281 | 0.019 |
| Prussian carp | 1176 | 1 | 2 | 2 | -0.373 | 0.015 | -0.228 | 0.109 | 0.037 | 0.204 | 0.468 | 0.109 | 0.473 | -0.013 | 0.291 | -0.127 | 0.065 | -0.163 | -0.137 | -0.076 | -0.220 | -0.102 | -0.100 | 0.009 | -0.275 | 0.035 |
| Prussian carp | 1177 | 1 | 2 | 2 | -0.370 | 0.013 | -0.221 | 0.122 | 0.035 | 0.205 | 0.465 | 0.116 | 0.493 | -0.029 | 0.276 | -0.120 | 0.042 | -0.152 | -0.117 | -0.083 | -0.205 | -0.104 | -0.109 | 0.004 | -0.289 | 0.026 |
| Prussian carp | 1178 | 1 | 2 | 2 | -0.365 | 0.012 | -0.221 | 0.126 | 0.021 | 0.203 | 0.464 | 0.104 | 0.478 | -0.048 | 0.295 | -0.107 | 0.090 | -0.137 | -0.128 | -0.069 | -0.203 | -0.101 | -0.130 | -0.006 | -0.303 | 0.023 |
| Prussian carp | 1179 | 1 | 2 | 2 | -0.390 | -0.003 | -0.212 | 0.125 | 0.023 | 0.199 | 0.478 | 0.096 | 0.475 | -0.042 | 0.291 | -0.106 | 0.068 | -0.124 | -0.127 | -0.069 | -0.201 | -0.102 | -0.117 | 0.012 | -0.288 | 0.013 |
| Prussian carp | 1180 | 1 | 2 | 2 | -0.376 | 0.033 | -0.206 | 0.129 | 0.030 | 0.199 | 0.463 | 0.114 | 0.485 | -0.019 | 0.291 | -0.112 | 0.049 | -0.160 | -0.133 | -0.104 | -0.204 | -0.094 | -0.113 | -0.009 | -0.287 | 0.024 |
| Prussian carp | 1181 | 1 | 2 | 2 | -0.379 | 0.004 | -0.233 | 0.104 | 0.063 | 0.216 | 0.463 | 0.103 | 0.476 | -0.038 | 0.299 | -0.108 | 0.047 | -0.137 | -0.119 | -0.060 | -0.197 | -0.102 | -0.129 | 0.002 | -0.290 | 0.016 |
| Prussian carp | 1182 | 1 | 2 | 2 | -0.395 | 0.019 | -0.224 | 0.116 | 0.046 | 0.183 | 0.463 | 0.112 | 0.474 | -0.033 | 0.301 | -0.105 | 0.057 | -0.134 | -0.114 | -0.084 | -0.222 | -0.112 | -0.103 | 0.006 | -0.283 | 0.032 |
| Prussian carp | 1183 | 1 | 2 | 2 | -0.371 | 0.005 | -0.218 | 0.121 | 0.028 | 0.211 | 0.467 | 0.100 | 0.485 | -0.046 | 0.304 | -0.098 | 0.038 | -0.131 | -0.128 | -0.084 | -0.195 | -0.103 | -0.123 | 0.003 | -0.286 | 0.021 |
| Prussian carp | 1184 | 1 | 2 | 2 | -0.378 | 0.015 | -0.202 | 0.123 | 0.029 | 0.188 | 0.462 | 0.105 | 0.482 | -0.028 | 0.306 | -0.113 | 0.055 | -0.140 | -0.129 | -0.075 | -0.209 | -0.105 | -0.118 | 0.011 | -0.298 | 0.019 |
| Prussian carp | 1185 | 1 | 2 | 2 | -0.378 | 0.019 | -0.213 | 0.129 | 0.025 | 0.203 | 0.455 | 0.100 | 0.468 | -0.041 | 0.312 | -0.114 | 0.105 | -0.131 | -0.144 | -0.081 | -0.211 | -0.094 | -0.129 | 0.000 | -0.292 | 0.010 |
| Prussian carp | 1186 | 1 | 2 | 2 | -0.390 | 0.002 | -0.227 | 0.128 | 0.030 | 0.222 | 0.463 | 0.108 | 0.484 | -0.043 | 0.287 | -0.119 | 0.039 | -0.127 | -0.117 | -0.082 | -0.203 | -0.106 | -0.095 | 0.002 | -0.272 | 0.015 |
| Prussian carp | 1187 | 1 | 2 | 2 | -0.386 | 0.006 | -0.231 | 0.116 | 0.027 | 0.205 | 0.483 | 0.101 | 0.466 | -0.050 | 0.281 | -0.101 | 0.083 | -0.131 | -0.123 | -0.082 | -0.194 | -0.109 | -0.120 | 0.017 | -0.286 | 0.026 |
| Prussian carp | 1188 | 1 | 2 | 2 | -0.373 | 0.003 | -0.238 | 0.118 | 0.053 | 0.217 | 0.456 | 0.099 | 0.462 | -0.045 | 0.303 | -0.109 | 0.091 | -0.140 | -0.133 | -0.071 | -0.197 | -0.108 | -0.120 | 0.013 | -0.303 | 0.024 |
| Prussian carp | 1189 | 1 | 2 | 2 | -0.379 | 0.016 | -0.228 | 0.119 | 0.049 | 0.221 | 0.465 | 0.105 | 0.474 | -0.034 | 0.283 | -0.110 | 0.051 | -0.143 | -0.113 | -0.084 | -0.202 | -0.117 | -0.110 | 0.006 | -0.290 | 0.020 |
| Prussian carp | 1190 | 1 | 2 | 2 | -0.368 | -0.002 | -0.218 | 0.118 | 0.028 | 0.199 | 0.472 | 0.103 | 0.488 | -0.048 | 0.296 | -0.096 | 0.047 | -0.134 | -0.119 | -0.063 | -0.206 | -0.102 | -0.127 | 0.002 | -0.293 | 0.023 |
| Prussian carp | 1161 | 1 | 2 | 3 | -0.389 | 0.006 | -0.237 | 0.125 | 0.021 | 0.221 | 0.467 | 0.098 | 0.476 | -0.043 | 0.287 | -0.110 | 0.058 | -0.143 | -0.106 | -0.082 | -0.197 | -0.100 | -0.100 | 0.008 | -0.281 | 0.022 |
| Prussian carp | 1162 | 1 | 2 | 3 | -0.390 | 0.016 | -0.216 | 0.124 | 0.031 | 0.196 | 0.471 | 0.104 | 0.477 | -0.036 | 0.292 | -0.101 | 0.058 | -0.138 | -0.121 | -0.081 | -0.207 | -0.104 | -0.109 | -0.002 | -0.285 | 0.022 |
| Prussian carp | 1163 | 1 | 2 | 3 | -0.375 | 0.001 | -0.231 | 0.122 | 0.045 | 0.211 | 0.464 | 0.096 | 0.476 | -0.045 | 0.297 | -0.101 | 0.065 | -0.128 | -0.125 | -0.078 | -0.196 | -0.103 | -0.125 | 0.009 | -0.295 | 0.017 |
| Prussian carp | 1164 | 1 | 2 | 3 | -0.385 | -0.006 | -0.209 | 0.136 | 0.027 | 0.230 | 0.476 | 0.097 | 0.478 | -0.036 | 0.274 | -0.108 | 0.043 | -0.140 | -0.119 | -0.080 | -0.206 | -0.110 | -0.094 | 0.006 | -0.284 | 0.012 |
| Prussian carp | 1165 | 1 | 2 | 3 | -0.371 | 0.011 | -0.202 | 0.133 | 0.020 | 0.221 | 0.473 | 0.109 | 0.489 | -0.036 | 0.274 | -0.110 | 0.041 | -0.147 | -0.127 | -0.082 | -0.192 | -0.109 | -0.116 | -0.007 | -0.289 | 0.018 |
| Prussian carp | 1166 | 1 | 2 | 3 | -0.377 | -0.009 | -0.214 | 0.125 | 0.035 | 0.196 | 0.484 | 0.093 | 0.480 | -0.033 | 0.291 | -0.099 | 0.042 | -0.131 | -0.119 | -0.061 | -0.200 | -0.101 | -0.131 | 0.005 | -0.291 | 0.016 |
| Prussian carp | 1167 | 1 | 2 | 3 | -0.394 | 0.008 | -0.236 | 0.119 | 0.078 | 0.205 | 0.458 | 0.114 | 0.477 | -0.028 | 0.280 | -0.112 | 0.048 | -0.146 | -0.118 | -0.077 | -0.202 | -0.108 | -0.107 | 0.005 | -0.284 | 0.021 |
| Prussian carp | 1168 | 1 | 2 | 3 | -0.371 | 0.011 | -0.206 | 0.120 | 0.022 | 0.200 | 0.474 | 0.100 | 0.485 | -0.034 | 0.296 | -0.109 | 0.050 | -0.132 | -0.126 | -0.076 | -0.198 | -0.100 | -0.128 | -0.001 | -0.298 | 0.019 |
| Prussian carp | 1169 | 1 | 2 | 3 | -0.380 | 0.010 | -0.241 | 0.114 | 0.038 | 0.228 | 0.461 | 0.099 | 0.471 | -0.040 | 0.292 | -0.117 | 0.062 | -0.143 | -0.100 | -0.073 | -0.202 | -0.109 | -0.111 | 0.011 | -0.290 | 0.019 |
| Prussian carp | 1170 | 1 | 2 | 3 | -0.374 | 0.016 | -0.243 | 0.101 | 0.043 | 0.214 | 0.463 | 0.108 | 0.475 | -0.038 | 0.302 | -0.114 | 0.043 | -0.146 | -0.109 | -0.075 | -0.211 | -0.108 | -0.110 | 0.017 | -0.280 | 0.024 |
| Prussian carp | 1171 | 1 | 2 | 3 | -0.390 | -0.003 | -0.227 | 0.120 | 0.059 | 0.222 | 0.454 | 0.104 | 0.456 | -0.044 | 0.319 | -0.106 | 0.051 | -0.142 | -0.115 | -0.080 | -0.206 | -0.108 | -0.116 | 0.009 | -0.285 | 0.027 |
| Prussian carp | 1172 | 1 | 2 | 3 | -0.379 | 0.013 | -0.235 | 0.111 | 0.014 | 0.217 | 0.485 | 0.095 | 0.480 | -0.053 | 0.289 | -0.093 | 0.048 | -0.126 | -0.118 | -0.072 | -0.193 | -0.101 | -0.114 | -0.009 | -0.278 | 0.018 |
| Prussian carp | 1173 | 1 | 2 | 3 | -0.371 | 0.014 | -0.217 | 0.111 | 0.018 | 0.205 | 0.479 | 0.109 | 0.501 | -0.036 | 0.271 | -0.109 | 0.035 | -0.142 | -0.128 | -0.076 | -0.197 | -0.102 | -0.113 | 0.007 | -0.279 | 0.018 |
| Prussian carp | 1174 | 1 | 2 | 3 | -0.386 | -0.004 | -0.236 | 0.124 | 0.031 | 0.239 | 0.464 | 0.092 | 0.480 | -0.051 | 0.271 | -0.111 | 0.078 | -0.135 | -0.108 | -0.080 | -0.180 | -0.103 | -0.128 | 0.013 | -0.285 | 0.016 |
| Prussian carp | 1175 | 1 | 2 | 3 | -0.377 | 0.009 | -0.216 | 0.126 | 0.030 | 0.219 | 0.458 | 0.101 | 0.484 | -0.035 | 0.290 | -0.112 | 0.072 | -0.137 | -0.123 | -0.075 | -0.212 | -0.102 | -0.120 | -0.026 | -0.285 | 0.032 |
| Prussian carp | 1176 | 1 | 2 | 3 | -0.378 | 0.012 | -0.238 | 0.105 | 0.044 | 0.207 | 0.467 | 0.108 | 0.480 | -0.016 | 0.291 | -0.124 | 0.036 | -0.164 | -0.124 | -0.064 | -0.201 | -0.108 | -0.098 | 0.009 | -0.278 | 0.035 |
| Prussian carp | 1177 | 1 | 2 | 3 | -0.371 | 0.010 | -0.213 | 0.122 | 0.026 | 0.207 | 0.467 | 0.110 | 0.496 | -0.027 | 0.280 | -0.116 | 0.041 | -0.150 | -0.124 | -0.085 | -0.207 | -0.094 | -0.114 | -0.005 | -0.282 | 0.028 |
| Prussian carp | 1178 | 1 | 2 | 3 | -0.374 | 0.010 | -0.219 | 0.123 | 0.037 | 0.200 | 0.455 | 0.104 | 0.478 | -0.044 | 0.306 | -0.104 | 0.076 | -0.136 | -0.124 | -0.077 | -0.213 | -0.095 | -0.122 | -0.001 | -0.299 | 0.021 |
| Prussian carp | 1179 | 1 | 2 | 3 | -0.386 | 0.003 | -0.215 | 0.120 | 0.017 | 0.210 | 0.479 | 0.097 | 0.468 | -0.045 | 0.298 | -0.106 | 0.058 | -0.126 | -0.114 | -0.078 | -0.196 | -0.104 | -0.119 | 0.007 | -0.291 | 0.022 |
| Prussian carp | 1180 | 1 | 2 | 3 | -0.369 | 0.043 | -0.226 | 0.126 | 0.024 | 0.188 | 0.473 | 0.115 | 0.480 | -0.024 | 0.288 | -0.118 | 0.061 | -0.158 | -0.121 | -0.088 | -0.192 | -0.101 | -0.126 | -0.002 | -0.290 | 0.020 |
| Prussian carp | 1181 | 1 | 2 | 3 | -0.380 | 0.004 | -0.229 | 0.113 | 0.039 | 0.212 | 0.468 | 0.097 | 0.475 | -0.032 | 0.302 | -0.109 | 0.047 | -0.143 | -0.116 | -0.078 | -0.198 | -0.093 | -0.124 | 0.009 | -0.284 | 0.021 |
| Prussian carp | 1182 | 1 | 2 | 3 | -0.388 | 0.020 | -0.224 | 0.118 | 0.035 | 0.182 | 0.464 | 0.111 | 0.483 | -0.030 | 0.297 | -0.108 | 0.055 | -0.138 | -0.114 | -0.085 | -0.221 | -0.106 | -0.109 | 0.001 | -0.279 | 0.034 |
| Prussian carp | 1183 | 1 | 2 | 3 | -0.370 | 0.008 | -0.233 | 0.112 | 0.061 | 0.214 | 0.458 | 0.105 | 0.470 | -0.047 | 0.315 | -0.097 | 0.047 | -0.135 | -0.128 | -0.073 | -0.199 | -0.107 | -0.127 | 0.000 | -0.294 | 0.021 |
| Prussian carp | 1184 | 1 | 2 | 3 | -0.376 | 0.017 | -0.221 | 0.123 | 0.043 | 0.193 | 0.460 | 0.108 | 0.476 | -0.027 | 0.302 | -0.116 | 0.062 | -0.143 | -0.124 | -0.079 | -0.206 | -0.105 | -0.116 | 0.005 | -0.299 | 0.025 |
| Prussian carp | 1185 | 1 | 2 | 3 | -0.380 | 0.014 | -0.220 | 0.127 | 0.034 | 0.207 | 0.462 | 0.101 | 0.459 | -0.041 | 0.309 | -0.115 | 0.101 | -0.132 | -0.132 | -0.081 | -0.206 | -0.094 | -0.134 | 0.001 | -0.294 | 0.013 |
| Prussian carp | 1186 | 1 | 2 | 3 | -0.390 | -0.001 | -0.223 | 0.138 | 0.025 | 0.224 | 0.453 | 0.101 | 0.482 | -0.045 | 0.305 | -0.117 | 0.035 | -0.129 | -0.112 | -0.085 | -0.192 | -0.111 | -0.107 | 0.004 | -0.276 | 0.022 |
| Prussian carp | 1187 | 1 | 2 | 3 | -0.392 | -0.001 | -0.235 | 0.121 | 0.051 | 0.208 | 0.471 | 0.105 | 0.465 | -0.050 | 0.282 | -0.100 | 0.082 | -0.132 | -0.109 | -0.074 | -0.200 | -0.103 | -0.125 | -0.002 | -0.291 | 0.028 |
| Prussian carp | 1188 | 1 | 2 | 3 | -0.367 | -0.003 | -0.236 | 0.116 | 0.061 | 0.214 | 0.460 | 0.096 | 0.463 | -0.033 | 0.314 | -0.107 | 0.056 | -0.142 | -0.123 | -0.073 | -0.201 | -0.107 | -0.130 | 0.010 | -0.299 | 0.030 |
| Prussian carp | 1189 | 1 | 2 | 3 | -0.371 | 0.014 | -0.244 | 0.115 | 0.044 | 0.225 | 0.468 | 0.102 | 0.468 | -0.038 | 0.288 | -0.112 | 0.062 | -0.145 | -0.109 | -0.076 | -0.203 | -0.111 | -0.110 | 0.003 | -0.291 | 0.022 |
| Prussian carp | 1190 | 1 | 2 | 3 | -0.369 | -0.001 | -0.238 | 0.115 | 0.028 | 0.200 | 0.473 | 0.094 | 0.471 | -0.049 | 0.309 | -0.100 | 0.073 | -0.124 | -0.118 | -0.077 | -0.202 | -0.094 | -0.134 | 0.003 | -0.292 | 0.032 |
| Prussian carp | 1251 | 1 | 3 | 1 | -0.365 | 0.006 | -0.224 | 0.123 | 0.043 | 0.244 | 0.467 | 0.107 | 0.478 | -0.033 | 0.281 | -0.124 | 0.034 | -0.152 | -0.125 | -0.078 | -0.185 | -0.113 | -0.117 | 0.011 | -0.286 | 0.011 |
| Prussian carp | 1252 | 1 | 3 | 1 | -0.364 | 0.007 | -0.215 | 0.128 | 0.036 | 0.245 | 0.479 | 0.116 | 0.458 | -0.044 | 0.283 | -0.119 | 0.051 | -0.157 | -0.130 | -0.088 | -0.188 | -0.111 | -0.122 | 0.008 | -0.288 | 0.014 |
| Prussian carp | 1253 | 1 | 3 | 1 | -0.351 | 0.011 | -0.208 | 0.123 | 0.039 | 0.234 | 0.459 | 0.114 | 0.478 | -0.033 | 0.296 | -0.123 | 0.052 | -0.161 | -0.147 | -0.086 | -0.203 | -0.110 | -0.131 | 0.016 | -0.284 | 0.015 |
| Prussian carp | 1254 | 1 | 3 | 1 | -0.377 | -0.030 | -0.220 | 0.138 | 0.026 | 0.226 | 0.485 | 0.083 | 0.480 | -0.054 | 0.276 | -0.088 | 0.051 | -0.125 | -0.125 | -0.069 | -0.190 | -0.105 | -0.117 | 0.014 | -0.289 | 0.009 |
| Prussian carp | 1255 | 1 | 3 | 1 | -0.359 | 0.002 | -0.203 | 0.127 | 0.049 | 0.223 | 0.453 | 0.113 | 0.490 | -0.024 | 0.299 | -0.125 | 0.022 | -0.156 | -0.138 | -0.081 | -0.200 | -0.109 | -0.128 | 0.014 | -0.284 | 0.016 |
| Prussian carp | 1256 | 1 | 3 | 1 | -0.378 | 0.005 | -0.219 | 0.123 | 0.047 | 0.222 | 0.473 | 0.112 | 0.469 | -0.040 | 0.290 | -0.107 | 0.040 | -0.147 | -0.135 | -0.081 | -0.194 | -0.112 | -0.111 | 0.006 | -0.282 | 0.018 |
| Prussian carp | 1257 | 1 | 3 | 1 | -0.360 | -0.006 | -0.229 | 0.109 | 0.047 | 0.225 | 0.479 | 0.101 | 0.477 | -0.037 | 0.289 | -0.115 | 0.039 | -0.126 | -0.127 | -0.069 | -0.206 | -0.105 | -0.121 | 0.010 | -0.288 | 0.013 |
| Prussian carp | 1258 | 1 | 3 | 1 | -0.364 | 0.012 | -0.217 | 0.120 | 0.055 | 0.206 | 0.453 | 0.108 | 0.500 | -0.009 | 0.300 | -0.120 | 0.013 | -0.156 | -0.136 | -0.074 | -0.213 | -0.104 | -0.115 | -0.007 | -0.275 | 0.024 |
| Prussian carp | 1259 | 1 | 3 | 1 | -0.348 | -0.004 | -0.226 | 0.121 | 0.029 | 0.217 | 0.490 | 0.096 | 0.474 | -0.046 | 0.295 | -0.098 | 0.048 | -0.130 | -0.137 | -0.074 | -0.198 | -0.101 | -0.140 | 0.005 | -0.288 | 0.012 |
| Prussian carp | 1260 | 1 | 3 | 1 | -0.361 | 0.001 | -0.211 | 0.123 | 0.003 | 0.215 | 0.474 | 0.104 | 0.492 | -0.048 | 0.298 | -0.109 | 0.036 | -0.135 | -0.130 | -0.075 | -0.202 | -0.102 | -0.119 | 0.006 | -0.280 | 0.019 |
| Prussian carp | 1261 | 1 | 3 | 1 | -0.352 | -0.002 | -0.222 | 0.120 | 0.081 | 0.211 | 0.477 | 0.112 | 0.469 | -0.034 | 0.291 | -0.097 | 0.039 | -0.145 | -0.143 | -0.078 | -0.202 | -0.104 | -0.143 | 0.004 | -0.296 | 0.013 |
| Prussian carp | 1262 | 1 | 3 | 1 | -0.366 | 0.018 | -0.210 | 0.124 | 0.048 | 0.205 | 0.465 | 0.121 | 0.478 | -0.026 | 0.297 | -0.118 | 0.033 | -0.160 | -0.130 | -0.083 | -0.198 | -0.107 | -0.129 | 0.008 | -0.288 | 0.019 |
| Prussian carp | 1263 | 1 | 3 | 1 | -0.363 | 0.010 | -0.215 | 0.130 | 0.047 | 0.220 | 0.458 | 0.109 | 0.476 | -0.041 | 0.301 | -0.105 | 0.051 | -0.148 | -0.139 | -0.087 | -0.199 | -0.111 | -0.125 | 0.007 | -0.291 | 0.016 |
| Prussian carp | 1264 | 1 | 3 | 1 | -0.371 | 0.005 | -0.225 | 0.121 | 0.025 | 0.236 | 0.479 | 0.116 | 0.471 | -0.047 | 0.282 | -0.117 | 0.038 | -0.144 | -0.125 | -0.086 | -0.202 | -0.114 | -0.103 | 0.009 | -0.269 | 0.020 |
| Prussian carp | 1265 | 1 | 3 | 1 | -0.340 | 0.007 | -0.215 | 0.100 | 0.020 | 0.220 | 0.484 | 0.103 | 0.489 | -0.049 | 0.294 | -0.105 | 0.036 | -0.132 | -0.135 | -0.069 | -0.194 | -0.107 | -0.145 | 0.020 | -0.295 | 0.012 |
| Prussian carp | 1266 | 1 | 3 | 1 | -0.363 | -0.018 | -0.242 | 0.119 | 0.008 | 0.250 | 0.495 | 0.091 | 0.476 | -0.061 | 0.283 | -0.102 | 0.025 | -0.123 | -0.122 | -0.068 | -0.196 | -0.104 | -0.101 | 0.012 | -0.263 | 0.005 |
| Prussian carp | 1267 | 1 | 3 | 1 | -0.376 | -0.018 | -0.235 | 0.131 | -0.011 | 0.240 | 0.490 | 0.094 | 0.480 | -0.056 | 0.281 | -0.117 | 0.028 | -0.129 | -0.114 | -0.065 | -0.193 | -0.108 | -0.089 | 0.017 | -0.262 | 0.011 |
| Prussian carp | 1268 | 1 | 3 | 1 | -0.373 | 0.004 | -0.234 | 0.126 | 0.026 | 0.250 | 0.484 | 0.128 | 0.466 | -0.045 | 0.277 | -0.126 | 0.003 | -0.163 | -0.116 | -0.090 | -0.202 | -0.121 | -0.085 | 0.019 | -0.247 | 0.018 |
| Prussian carp | 1269 | 1 | 3 | 1 | -0.364 | -0.012 | -0.230 | 0.120 | 0.039 | 0.215 | 0.477 | 0.097 | 0.475 | -0.052 | 0.287 | -0.104 | 0.068 | -0.121 | -0.133 | -0.073 | -0.200 | -0.105 | -0.126 | 0.021 | -0.293 | 0.013 |
| Prussian carp | 1270 | 1 | 3 | 1 | -0.362 | -0.020 | -0.238 | 0.117 | 0.036 | 0.253 | 0.499 | 0.087 | 0.456 | -0.046 | 0.286 | -0.100 | 0.036 | -0.137 | -0.124 | -0.067 | -0.193 | -0.103 | -0.113 | 0.007 | -0.281 | 0.010 |
| Prussian carp | 1271 | 1 | 3 | 1 | -0.397 | 0.018 | -0.196 | 0.125 | 0.048 | 0.202 | 0.455 | 0.115 | 0.481 | -0.023 | 0.299 | -0.120 | 0.040 | -0.148 | -0.126 | -0.082 | -0.195 | -0.112 | -0.125 | -0.003 | -0.283 | 0.026 |
| Prussian carp | 1272 | 1 | 3 | 1 | -0.355 | 0.019 | -0.214 | 0.117 | 0.066 | 0.226 | 0.459 | 0.118 | 0.472 | -0.026 | 0.302 | -0.114 | 0.036 | -0.165 | -0.138 | -0.088 | -0.205 | -0.104 | -0.137 | 0.000 | -0.286 | 0.018 |
| Prussian carp | 1273 | 1 | 3 | 1 | -0.359 | 0.014 | -0.231 | 0.112 | 0.074 | 0.226 | 0.440 | 0.104 | 0.478 | -0.022 | 0.315 | -0.124 | 0.049 | -0.150 | -0.133 | -0.084 | -0.208 | -0.096 | -0.136 | 0.009 | -0.290 | 0.011 |
| Prussian carp | 1274 | 1 | 3 | 1 | -0.362 | -0.007 | -0.215 | 0.119 | 0.067 | 0.223 | 0.460 | 0.107 | 0.470 | -0.041 | 0.306 | -0.107 | 0.042 | -0.139 | -0.128 | -0.074 | -0.204 | -0.109 | -0.137 | 0.012 | -0.299 | 0.016 |
| Prussian carp | 1275 | 1 | 3 | 1 | -0.360 | -0.021 | -0.233 | 0.122 | 0.050 | 0.244 | 0.484 | 0.099 | 0.474 | -0.037 | 0.276 | -0.114 | 0.027 | -0.147 | -0.118 | -0.070 | -0.192 | -0.089 | -0.127 | 0.009 | -0.282 | 0.005 |
| Prussian carp | 4030 | 1 | 3 | 1 | -0.383 | -0.002 | -0.227 | 0.124 | 0.038 | 0.234 | 0.475 | 0.099 | 0.472 | -0.046 | 0.286 | -0.095 | 0.033 | -0.143 | -0.114 | -0.080 | -0.203 | -0.108 | -0.101 | 0.003 | -0.276 | 0.014 |
| Prussian carp | 4031 | 1 | 3 | 1 | -0.370 | 0.001 | -0.221 | 0.120 | 0.007 | 0.234 | 0.479 | 0.093 | 0.473 | -0.048 | 0.300 | -0.108 | 0.039 | -0.139 | -0.115 | -0.063 | -0.186 | -0.110 | -0.123 | 0.005 | -0.284 | 0.016 |
| Prussian carp | 4032 | 1 | 3 | 1 | -0.349 | -0.012 | -0.228 | 0.108 | 0.064 | 0.235 | 0.459 | 0.106 | 0.477 | -0.035 | 0.298 | -0.125 | 0.052 | -0.139 | -0.136 | -0.060 | -0.218 | -0.103 | -0.131 | 0.008 | -0.287 | 0.017 |
| Prussian carp | 4033 | 1 | 3 | 1 | -0.340 | -0.009 | -0.212 | 0.121 | 0.002 | 0.237 | 0.470 | 0.108 | 0.480 | -0.045 | 0.300 | -0.137 | 0.057 | -0.150 | -0.136 | -0.064 | -0.197 | -0.100 | -0.141 | 0.022 | -0.284 | 0.017 |
| Prussian carp | 4034 | 1 | 3 | 1 | -0.346 | 0.006 | -0.223 | 0.114 | 0.069 | 0.226 | 0.466 | 0.117 | 0.461 | -0.029 | 0.313 | -0.122 | 0.041 | -0.154 | -0.149 | -0.081 | -0.201 | -0.099 | -0.140 | 0.002 | -0.290 | 0.018 |
| Prussian carp | 1251 | 1 | 3 | 2 | -0.364 | 0.013 | -0.228 | 0.120 | 0.041 | 0.244 | 0.465 | 0.104 | 0.482 | -0.033 | 0.275 | -0.123 | 0.041 | -0.152 | -0.122 | -0.081 | -0.184 | -0.117 | -0.122 | 0.015 | -0.286 | 0.010 |
| Prussian carp | 1252 | 1 | 3 | 2 | -0.361 | 0.007 | -0.216 | 0.126 | 0.040 | 0.240 | 0.470 | 0.115 | 0.467 | -0.041 | 0.287 | -0.119 | 0.049 | -0.154 | -0.125 | -0.088 | -0.202 | -0.108 | -0.125 | 0.010 | -0.284 | 0.012 |
| Prussian carp | 1253 | 1 | 3 | 2 | -0.352 | 0.015 | -0.204 | 0.121 | 0.039 | 0.230 | 0.454 | 0.113 | 0.482 | -0.032 | 0.301 | -0.118 | 0.048 | -0.163 | -0.145 | -0.085 | -0.203 | -0.108 | -0.132 | 0.011 | -0.288 | 0.018 |
| Prussian carp | 1254 | 1 | 3 | 2 | -0.376 | -0.028 | -0.213 | 0.137 | 0.024 | 0.223 | 0.474 | 0.083 | 0.488 | -0.055 | 0.278 | -0.090 | 0.058 | -0.126 | -0.128 | -0.070 | -0.191 | -0.106 | -0.124 | 0.023 | -0.291 | 0.009 |
| Prussian carp | 1255 | 1 | 3 | 2 | -0.358 | 0.007 | -0.212 | 0.124 | 0.050 | 0.224 | 0.448 | 0.111 | 0.492 | -0.023 | 0.297 | -0.127 | 0.037 | -0.158 | -0.139 | -0.081 | -0.206 | -0.106 | -0.128 | 0.013 | -0.281 | 0.016 |
| Prussian carp | 1256 | 1 | 3 | 2 | -0.382 | 0.012 | -0.213 | 0.128 | 0.023 | 0.215 | 0.472 | 0.112 | 0.474 | -0.039 | 0.291 | -0.114 | 0.046 | -0.149 | -0.125 | -0.078 | -0.195 | -0.110 | -0.112 | 0.004 | -0.280 | 0.019 |
[truncated: 133,382 more chars]
