# Supplementary material for: Repeatability, Reproducibility, Separative Power and Subjectivity of Different Fish Morphometric Analysis Methods
Source: PLoS One. 2016 Jun 21;11(6):e0157890. doi: 10.1371/journal.pone.0157890 (PMC4915670; doi:10.1371/journal.pone.0157890)
Supplement: S3 Table — (DOCX) [file pone.0157890.s003.docx]

Supplementary Table 3 Raw dataset of the TRU analyses, for codes see text.

| Species | individual code | Measu-rer | Site | repeat | a | b | c | d | e | f | g | h | i | j | k | l | m | n | o |
| --- | --- | --- | --- | --- | --- | --- | --- | --- | --- | --- | --- | --- | --- | --- | --- | --- | --- | --- | --- |
| Bleak | 1031 | 1 | 1 | 1 | 17.22 | 15.21 | 15.40 | 31.78 | 47.27 | 41.55 | 34.51 | 26.97 | 20.92 | 33.58 | 37.91 | 24.63 | 45.24 | 41.93 | 10.88 |
| Bleak | 1032 | 1 | 1 | 1 | 20.51 | 18.51 | 19.04 | 40.67 | 57.88 | 53.88 | 47.45 | 29.56 | 20.99 | 45.16 | 48.35 | 29.04 | 58.86 | 55.98 | 10.66 |
| Bleak | 1033 | 1 | 1 | 1 | 24.19 | 20.49 | 20.49 | 40.73 | 61.98 | 55.57 | 48.56 | 35.27 | 24.21 | 44.59 | 49.96 | 34.19 | 58.54 | 53.91 | 11.92 |
| Bleak | 1034 | 1 | 1 | 1 | 17.05 | 15.75 | 15.27 | 29.56 | 43.86 | 39.13 | 35.06 | 24.93 | 19.22 | 34.37 | 37.48 | 23.75 | 46.39 | 42.99 | 9.88 |
| Bleak | 1035 | 1 | 1 | 1 | 12.65 | 12.59 | 12.68 | 22.13 | 36.05 | 31.13 | 26.71 | 21.57 | 15.69 | 27.64 | 30.38 | 18.96 | 36.74 | 34.89 | 7.84 |
| Bleak | 1036 | 1 | 1 | 1 | 19.85 | 18.87 | 18.69 | 35.55 | 54.90 | 48.90 | 40.66 | 33.32 | 23.36 | 45.52 | 48.36 | 30.51 | 58.82 | 54.65 | 12.89 |
| Bleak | 1037 | 1 | 1 | 1 | 19.37 | 16.65 | 16.71 | 32.28 | 49.53 | 45.07 | 39.15 | 26.91 | 18.91 | 38.86 | 42.05 | 25.94 | 51.11 | 47.23 | 10.68 |
| Bleak | 1038 | 1 | 1 | 1 | 20.36 | 16.02 | 16.80 | 32.49 | 47.01 | 43.15 | 38.96 | 28.41 | 20.59 | 36.13 | 40.75 | 27.24 | 48.99 | 47.69 | 10.48 |
| Bleak | 1039 | 1 | 1 | 1 | 16.05 | 14.85 | 14.87 | 28.27 | 38.96 | 33.90 | 32.61 | 24.02 | 15.91 | 33.83 | 35.77 | 22.39 | 44.31 | 41.60 | 9.28 |
| Bleak | 1040 | 1 | 1 | 1 | 22.64 | 18.71 | 19.42 | 39.93 | 58.44 | 54.71 | 49.50 | 35.49 | 23.38 | 41.43 | 49.74 | 32.55 | 58.23 | 59.72 | 13.21 |
| Bleak | 1041 | 1 | 1 | 1 | 13.60 | 12.59 | 13.07 | 24.94 | 37.74 | 33.73 | 30.04 | 21.86 | 14.70 | 29.32 | 31.63 | 19.59 | 39.46 | 36.49 | 8.24 |
| Bleak | 1042 | 1 | 1 | 1 | 18.01 | 16.53 | 17.10 | 32.13 | 46.22 | 40.67 | 39.35 | 27.44 | 16.43 | 40.90 | 42.70 | 25.94 | 51.81 | 47.91 | 10.48 |
| Bleak | 1043 | 1 | 1 | 1 | 18.53 | 16.38 | 17.32 | 33.51 | 51.29 | 47.57 | 39.18 | 30.87 | 22.63 | 40.80 | 43.98 | 28.14 | 53.42 | 49.26 | 11.70 |
| Bleak | 1044 | 1 | 1 | 1 | 15.35 | 13.84 | 14.13 | 30.01 | 41.79 | 37.40 | 33.67 | 21.48 | 15.47 | 34.33 | 36.91 | 20.34 | 42.96 | 41.43 | 9.85 |
| Bleak | 1045 | 1 | 1 | 1 | 15.28 | 15.75 | 15.50 | 29.65 | 45.09 | 39.05 | 35.00 | 26.87 | 18.62 | 38.51 | 41.53 | 24.48 | 46.37 | 45.11 | 10.05 |
| Bleak | 1046 | 1 | 1 | 1 | 21.23 | 18.00 | 18.54 | 36.94 | 52.53 | 46.33 | 41.57 | 29.42 | 22.94 | 43.36 | 45.97 | 26.95 | 51.37 | 47.45 | 11.74 |
| Bleak | 1047 | 1 | 1 | 1 | 22.79 | 18.44 | 18.69 | 40.55 | 53.38 | 48.43 | 46.26 | 29.29 | 19.12 | 43.67 | 45.88 | 28.58 | 54.53 | 50.06 | 11.96 |
| Bleak | 1048 | 1 | 1 | 1 | 16.23 | 16.80 | 16.49 | 29.94 | 46.18 | 40.04 | 35.50 | 24.72 | 17.88 | 36.38 | 39.45 | 25.28 | 49.39 | 46.01 | 10.50 |
| Bleak | 1049 | 1 | 1 | 1 | 21.87 | 20.27 | 20.33 | 44.41 | 59.82 | 52.97 | 53.78 | 34.85 | 21.09 | 43.52 | 48.00 | 32.69 | 58.14 | 55.16 | 13.07 |
| Bleak | 1050 | 1 | 1 | 1 | 15.68 | 14.02 | 14.32 | 28.35 | 41.95 | 36.67 | 31.50 | 23.81 | 18.20 | 35.53 | 37.81 | 22.14 | 44.69 | 42.63 | 9.25 |
| Bleak | 1051 | 1 | 1 | 1 | 19.61 | 16.43 | 17.34 | 37.36 | 52.99 | 47.22 | 41.63 | 28.40 | 22.95 | 41.23 | 44.84 | 25.67 | 53.22 | 48.45 | 11.48 |
| Bleak | 1052 | 1 | 1 | 1 | 20.18 | 17.84 | 19.70 | 41.27 | 57.87 | 52.57 | 45.59 | 32.17 | 23.16 | 43.37 | 46.83 | 29.69 | 57.08 | 55.70 | 10.92 |
| Bleak | 1053 | 1 | 1 | 1 | 21.86 | 17.60 | 19.17 | 32.80 | 51.67 | 47.37 | 41.35 | 29.63 | 21.98 | 42.45 | 47.02 | 27.55 | 54.40 | 51.92 | 11.45 |
| Bleak | 1054 | 1 | 1 | 1 | 14.44 | 13.96 | 14.50 | 27.79 | 39.60 | 35.35 | 34.08 | 23.34 | 14.31 | 31.83 | 35.37 | 22.02 | 43.42 | 41.23 | 9.25 |
| Bleak | 1055 | 1 | 1 | 1 | 18.25 | 15.15 | 16.11 | 29.64 | 44.66 | 41.27 | 36.91 | 25.68 | 19.29 | 36.40 | 40.27 | 26.12 | 47.79 | 45.91 | 10.66 |
| Bleak | 1056 | 1 | 1 | 1 | 13.50 | 13.55 | 12.46 | 21.76 | 33.17 | 30.18 | 25.25 | 19.41 | 16.48 | 26.96 | 28.85 | 18.82 | 36.67 | 35.75 | 7.45 |
| Bleak | 1057 | 1 | 1 | 1 | 14.83 | 13.73 | 13.46 | 28.78 | 35.54 | 31.60 | 32.80 | 20.01 | 10.34 | 33.08 | 33.45 | 20.04 | 39.23 | 35.29 | 7.88 |
| Bleak | 1058 | 1 | 1 | 1 | 20.34 | 17.67 | 17.78 | 29.64 | 46.35 | 41.83 | 39.22 | 28.13 | 18.17 | 32.35 | 35.72 | 27.60 | 45.91 | 42.34 | 9.85 |
| Bleak | 1059 | 1 | 1 | 1 | 15.51 | 16.77 | 15.36 | 29.41 | 44.75 | 40.05 | 33.25 | 25.26 | 21.35 | 33.93 | 37.74 | 23.40 | 46.27 | 45.21 | 8.88 |
| Bleak | 1060 | 1 | 1 | 1 | 17.00 | 15.30 | 15.29 | 31.55 | 45.09 | 40.24 | 37.14 | 25.54 | 18.33 | 35.77 | 39.07 | 24.63 | 46.95 | 45.21 | 10.47 |
| Bleak | 1031 | 1 | 1 | 2 | 17.94 | 15.45 | 16.70 | 29.73 | 46.10 | 40.90 | 35.11 | 27.16 | 20.79 | 35.33 | 39.48 | 25.33 | 45.63 | 43.05 | 10.47 |
| Bleak | 1032 | 1 | 1 | 2 | 20.79 | 19.30 | 20.06 | 39.61 | 56.33 | 51.64 | 47.00 | 30.12 | 23.23 | 42.61 | 47.10 | 30.19 | 58.33 | 56.05 | 11.66 |
| Bleak | 1033 | 1 | 1 | 2 | 21.58 | 20.26 | 20.71 | 41.70 | 62.26 | 53.79 | 45.84 | 35.11 | 24.70 | 47.02 | 51.23 | 31.98 | 58.87 | 55.48 | 12.47 |
| Bleak | 1034 | 1 | 1 | 2 | 18.51 | 16.20 | 16.09 | 28.03 | 43.43 | 39.28 | 34.06 | 25.12 | 19.14 | 36.18 | 39.28 | 23.64 | 47.54 | 44.95 | 10.05 |
| Bleak | 1035 | 1 | 1 | 2 | 12.72 | 12.94 | 12.26 | 23.34 | 35.91 | 31.02 | 26.03 | 21.01 | 16.62 | 27.05 | 29.12 | 18.69 | 37.13 | 34.46 | 7.65 |
| Bleak | 1036 | 1 | 1 | 2 | 19.68 | 20.04 | 19.32 | 35.53 | 55.58 | 49.15 | 40.70 | 32.50 | 21.92 | 46.03 | 48.26 | 30.83 | 57.65 | 54.14 | 11.90 |
| Bleak | 1037 | 1 | 1 | 2 | 18.97 | 16.97 | 16.68 | 32.65 | 48.38 | 45.35 | 38.40 | 26.97 | 18.91 | 40.47 | 42.16 | 24.53 | 49.82 | 46.56 | 11.27 |
| Bleak | 1038 | 1 | 1 | 2 | 18.13 | 16.35 | 16.77 | 32.66 | 48.63 | 44.73 | 40.30 | 28.57 | 18.67 | 36.70 | 41.53 | 27.55 | 49.50 | 47.53 | 10.46 |
| Bleak | 1039 | 1 | 1 | 2 | 16.28 | 15.30 | 15.28 | 27.71 | 40.48 | 35.67 | 32.71 | 24.40 | 16.91 | 33.18 | 36.49 | 24.17 | 45.51 | 43.17 | 9.45 |
| Bleak | 1040 | 1 | 1 | 2 | 22.75 | 17.70 | 19.24 | 40.30 | 61.38 | 57.18 | 49.30 | 35.56 | 23.15 | 41.69 | 50.15 | 34.38 | 60.03 | 60.24 | 12.22 |
| Bleak | 1041 | 1 | 1 | 2 | 14.13 | 13.75 | 12.86 | 24.56 | 36.64 | 33.27 | 29.74 | 20.75 | 14.41 | 29.16 | 32.18 | 18.73 | 38.32 | 36.49 | 8.04 |
| Bleak | 1042 | 1 | 1 | 2 | 17.86 | 16.84 | 16.18 | 33.67 | 45.84 | 39.41 | 38.94 | 26.46 | 15.78 | 39.53 | 42.27 | 26.09 | 50.98 | 48.13 | 9.85 |
| Bleak | 1043 | 1 | 1 | 2 | 17.29 | 16.79 | 17.08 | 33.47 | 50.37 | 44.83 | 38.77 | 30.26 | 21.79 | 42.46 | 46.50 | 27.61 | 52.78 | 49.06 | 11.28 |
| Bleak | 1044 | 1 | 1 | 2 | 16.50 | 14.54 | 13.94 | 29.64 | 40.11 | 35.78 | 33.72 | 20.79 | 15.27 | 34.10 | 36.99 | 19.83 | 42.51 | 41.01 | 9.05 |
| Bleak | 1045 | 1 | 1 | 2 | 15.88 | 15.10 | 14.87 | 30.56 | 44.87 | 39.71 | 35.53 | 26.62 | 18.44 | 37.24 | 40.91 | 24.04 | 46.90 | 45.46 | 9.85 |
| Bleak | 1046 | 1 | 1 | 2 | 19.91 | 18.00 | 18.90 | 35.29 | 54.13 | 48.96 | 40.81 | 30.64 | 25.75 | 42.27 | 44.94 | 28.10 | 54.58 | 49.87 | 11.72 |
| Bleak | 1047 | 1 | 1 | 2 | 22.22 | 18.63 | 17.89 | 41.77 | 52.12 | 47.51 | 46.65 | 28.75 | 18.29 | 42.58 | 46.99 | 29.10 | 53.81 | 50.88 | 11.54 |
| Bleak | 1048 | 1 | 1 | 2 | 17.28 | 16.90 | 15.88 | 31.56 | 46.01 | 39.25 | 34.88 | 24.21 | 17.30 | 35.71 | 39.46 | 24.70 | 47.51 | 46.18 | 10.90 |
| Bleak | 1049 | 1 | 1 | 2 | 22.56 | 20.65 | 20.32 | 43.70 | 59.72 | 52.37 | 50.76 | 35.11 | 24.18 | 43.67 | 49.28 | 33.28 | 57.34 | 56.03 | 13.38 |
| Bleak | 1050 | 1 | 1 | 2 | 15.56 | 14.92 | 14.07 | 28.36 | 42.10 | 37.43 | 32.65 | 22.91 | 17.97 | 35.30 | 38.26 | 22.47 | 44.69 | 43.01 | 10.30 |
| Bleak | 1051 | 1 | 1 | 2 | 19.33 | 15.84 | 17.20 | 37.10 | 52.61 | 47.82 | 42.56 | 28.04 | 23.92 | 39.09 | 43.15 | 26.81 | 51.92 | 49.27 | 11.06 |
| Bleak | 1052 | 1 | 1 | 2 | 18.97 | 18.34 | 19.53 | 39.50 | 58.32 | 51.92 | 45.45 | 33.14 | 22.08 | 43.21 | 48.05 | 29.74 | 56.96 | 55.67 | 11.43 |
| Bleak | 1053 | 1 | 1 | 2 | 20.79 | 18.96 | 18.69 | 34.30 | 50.95 | 45.83 | 41.47 | 30.24 | 22.38 | 41.35 | 46.51 | 27.81 | 53.10 | 51.71 | 11.66 |
| Bleak | 1054 | 1 | 1 | 2 | 14.55 | 13.59 | 13.86 | 29.20 | 39.46 | 35.78 | 34.25 | 23.62 | 15.83 | 32.62 | 35.74 | 23.56 | 44.22 | 42.05 | 9.25 |
| Bleak | 1055 | 1 | 1 | 2 | 18.01 | 15.07 | 15.67 | 31.35 | 45.74 | 40.63 | 37.06 | 27.26 | 18.54 | 37.64 | 40.98 | 26.28 | 50.52 | 48.08 | 11.54 |
| Bleak | 1056 | 1 | 1 | 2 | 13.75 | 11.31 | 11.92 | 22.18 | 32.01 | 29.11 | 26.54 | 18.18 | 15.27 | 26.56 | 29.30 | 18.44 | 37.36 | 34.75 | 7.85 |
| Bleak | 1057 | 1 | 1 | 2 | 14.39 | 13.62 | 13.47 | 26.20 | 36.36 | 31.91 | 30.19 | 21.50 | 13.44 | 32.28 | 33.73 | 19.79 | 36.89 | 34.27 | 7.57 |
| Bleak | 1058 | 1 | 1 | 2 | 18.22 | 16.90 | 17.10 | 33.95 | 49.01 | 44.23 | 39.33 | 28.85 | 19.84 | 33.43 | 34.72 | 26.31 | 43.93 | 40.61 | 9.05 |
| Bleak | 1059 | 1 | 1 | 2 | 16.13 | 14.88 | 14.68 | 29.64 | 44.84 | 39.28 | 33.15 | 24.28 | 20.93 | 32.67 | 37.65 | 22.93 | 44.63 | 44.11 | 10.10 |
| Bleak | 1060 | 1 | 1 | 2 | 17.05 | 14.30 | 15.54 | 30.51 | 43.01 | 39.11 | 38.36 | 26.57 | 17.50 | 37.17 | 39.85 | 24.36 | 47.52 | 44.81 | 10.68 |
| Bleak | 1031 | 1 | 1 | 3 | 17.83 | 14.74 | 16.09 | 29.96 | 47.10 | 41.43 | 35.45 | 26.79 | 20.13 | 35.67 | 38.63 | 25.12 | 46.01 | 42.55 | 11.05 |
| Bleak | 1032 | 1 | 1 | 3 | 21.96 | 19.23 | 21.12 | 38.40 | 56.18 | 52.92 | 46.73 | 30.32 | 21.96 | 43.90 | 48.80 | 29.84 | 59.24 | 57.60 | 11.54 |
| Bleak | 1033 | 1 | 1 | 3 | 21.31 | 20.50 | 21.51 | 40.50 | 61.54 | 54.47 | 46.90 | 35.63 | 26.47 | 44.71 | 49.06 | 32.73 | 56.77 | 54.07 | 12.30 |
| Bleak | 1034 | 1 | 1 | 3 | 15.91 | 15.49 | 15.68 | 29.31 | 44.40 | 40.10 | 34.95 | 25.41 | 19.47 | 34.72 | 37.66 | 23.19 | 46.77 | 43.32 | 10.06 |
| Bleak | 1035 | 1 | 1 | 3 | 13.66 | 13.30 | 12.06 | 22.54 | 35.39 | 31.33 | 27.02 | 21.05 | 15.80 | 27.94 | 29.38 | 18.51 | 36.42 | 34.89 | 7.84 |
| Bleak | 1036 | 1 | 1 | 3 | 19.23 | 18.33 | 18.49 | 35.31 | 54.86 | 49.39 | 41.11 | 33.61 | 21.55 | 48.56 | 50.17 | 30.26 | 58.36 | 53.06 | 12.28 |
| Bleak | 1037 | 1 | 1 | 3 | 17.36 | 16.49 | 17.28 | 34.07 | 48.36 | 43.55 | 38.18 | 27.51 | 19.10 | 39.45 | 42.60 | 25.94 | 51.35 | 49.02 | 10.66 |
| Bleak | 1038 | 1 | 1 | 3 | 19.34 | 16.36 | 17.25 | 31.47 | 48.35 | 45.04 | 40.20 | 27.85 | 19.27 | 37.67 | 40.91 | 26.99 | 49.12 | 47.49 | 10.87 |
| Bleak | 1039 | 1 | 1 | 3 | 16.56 | 14.78 | 15.07 | 28.16 | 40.33 | 35.05 | 32.89 | 24.62 | 16.88 | 32.87 | 36.30 | 25.44 | 45.79 | 43.74 | 9.85 |
| Bleak | 1040 | 1 | 1 | 3 | 21.62 | 17.63 | 19.49 | 40.13 | 58.52 | 54.11 | 48.90 | 36.16 | 24.78 | 41.77 | 48.84 | 35.30 | 61.02 | 60.97 | 12.54 |
| Bleak | 1041 | 1 | 1 | 3 | 13.76 | 13.57 | 13.68 | 23.94 | 37.49 | 32.98 | 27.37 | 22.48 | 15.89 | 30.93 | 33.61 | 19.59 | 38.57 | 36.75 | 8.06 |
| Bleak | 1042 | 1 | 1 | 3 | 17.22 | 16.77 | 17.18 | 33.77 | 45.77 | 39.28 | 37.29 | 27.71 | 19.77 | 39.00 | 42.22 | 26.42 | 52.39 | 48.34 | 9.72 |
| Bleak | 1043 | 1 | 1 | 3 | 17.29 | 15.83 | 16.72 | 33.33 | 50.30 | 45.49 | 39.47 | 31.64 | 21.34 | 42.34 | 45.73 | 27.73 | 53.24 | 49.88 | 11.52 |
| Bleak | 1044 | 1 | 1 | 3 | 15.65 | 15.50 | 14.08 | 29.60 | 42.85 | 36.60 | 34.06 | 20.98 | 14.67 | 33.90 | 36.60 | 20.71 | 43.72 | 41.87 | 9.24 |
| Bleak | 1045 | 1 | 1 | 3 | 15.89 | 15.83 | 16.08 | 31.29 | 45.87 | 40.39 | 35.70 | 26.82 | 18.37 | 38.70 | 40.98 | 24.48 | 48.66 | 46.71 | 10.06 |
| Bleak | 1046 | 1 | 1 | 3 | 19.79 | 17.67 | 19.13 | 37.26 | 54.31 | 48.63 | 41.41 | 30.32 | 24.35 | 41.96 | 46.77 | 28.44 | 53.57 | 51.28 | 12.46 |
| Bleak | 1047 | 1 | 1 | 3 | 21.95 | 16.74 | 17.82 | 40.34 | 53.92 | 47.96 | 44.26 | 28.50 | 21.41 | 42.04 | 45.50 | 28.99 | 54.39 | 52.17 | 11.55 |
| Bleak | 1048 | 1 | 1 | 3 | 16.89 | 17.00 | 16.28 | 29.74 | 44.59 | 39.28 | 33.43 | 26.43 | 20.13 | 36.24 | 40.19 | 24.12 | 49.58 | 46.91 | 10.70 |
| Bleak | 1049 | 1 | 1 | 3 | 22.31 | 20.71 | 20.79 | 43.78 | 60.72 | 53.80 | 50.20 | 35.35 | 23.60 | 45.57 | 51.16 | 33.87 | 58.88 | 55.64 | 13.47 |
| Bleak | 1050 | 1 | 1 | 3 | 15.30 | 14.92 | 14.50 | 27.96 | 42.64 | 38.35 | 31.93 | 23.20 | 18.64 | 36.50 | 37.91 | 21.93 | 44.74 | 43.01 | 10.72 |
| Bleak | 1051 | 1 | 1 | 3 | 19.04 | 16.39 | 16.60 | 36.57 | 50.27 | 45.41 | 41.40 | 28.13 | 24.56 | 41.19 | 43.74 | 25.99 | 51.81 | 48.84 | 11.45 |
| Bleak | 1052 | 1 | 1 | 3 | 19.17 | 17.00 | 18.69 | 39.64 | 56.88 | 49.82 | 45.01 | 33.82 | 23.56 | 41.56 | 47.23 | 30.47 | 58.70 | 57.29 | 11.32 |
| Bleak | 1053 | 1 | 1 | 3 | 20.37 | 18.68 | 17.89 | 33.03 | 51.04 | 46.31 | 40.74 | 29.85 | 22.38 | 42.01 | 46.16 | 27.55 | 51.81 | 49.72 | 11.66 |
| Bleak | 1054 | 1 | 1 | 3 | 14.95 | 13.55 | 13.46 | 28.60 | 39.01 | 34.56 | 33.53 | 22.27 | 14.81 | 32.37 | 35.99 | 22.47 | 43.34 | 41.86 | 9.64 |
| Bleak | 1055 | 1 | 1 | 3 | 16.24 | 15.32 | 15.52 | 29.21 | 44.31 | 41.90 | 36.00 | 27.16 | 18.94 | 37.91 | 41.61 | 24.35 | 47.47 | 44.07 | 10.26 |
| Bleak | 1056 | 1 | 1 | 3 | 13.78 | 13.22 | 12.68 | 21.36 | 31.50 | 28.34 | 26.09 | 18.13 | 15.07 | 28.25 | 30.11 | 19.41 | 37.55 | 36.01 | 7.64 |
| Bleak | 1057 | 1 | 1 | 3 | 14.55 | 14.22 | 13.90 | 28.60 | 36.36 | 31.75 | 30.23 | 19.94 | 13.98 | 31.01 | 32.08 | 19.79 | 35.92 | 32.44 | 8.21 |
| Bleak | 1058 | 1 | 1 | 3 | 19.23 | 16.49 | 17.14 | 31.87 | 46.83 | 41.98 | 38.36 | 28.79 | 19.94 | 32.99 | 35.28 | 28.32 | 46.54 | 41.80 | 10.50 |
| Bleak | 1059 | 1 | 1 | 3 | 16.26 | 16.65 | 15.83 | 30.02 | 44.66 | 38.28 | 34.46 | 24.33 | 16.98 | 35.94 | 39.72 | 21.29 | 43.66 | 42.73 | 9.70 |
| Bleak | 1060 | 1 | 1 | 3 | 16.52 | 15.26 | 15.28 | 49.63 | 44.64 | 40.76 | 39.02 | 26.21 | 16.79 | 36.85 | 38.51 | 24.81 | 47.53 | 44.21 | 10.30 |
| Bleak | 1131 | 1 | 2 | 1 | 27.59 | 26.57 | 26.84 | 53.51 | 82.53 | 72.25 | 59.81 | 49.36 | 35.64 | 58.02 | 63.60 | 39.74 | 79.06 | 74.77 | 15.56 |
| Bleak | 1132 | 1 | 2 | 1 | 22.79 | 23.33 | 23.58 | 42.44 | 60.90 | 50.60 | 48.00 | 34.65 | 25.62 | 47.31 | 52.00 | 34.10 | 62.13 | 60.29 | 12.96 |
| Bleak | 1133 | 1 | 2 | 1 | 23.45 | 21.06 | 22.30 | 45.03 | 63.02 | 54.59 | 53.24 | 36.98 | 26.21 | 49.57 | 55.14 | 33.64 | 63.29 | 60.61 | 12.96 |
| Bleak | 1134 | 1 | 2 | 1 | 20.38 | 17.65 | 18.80 | 41.45 | 55.35 | 47.93 | 48.25 | 32.60 | 22.10 | 41.08 | 45.48 | 30.31 | 50.99 | 48.71 | 12.66 |
| Bleak | 1135 | 1 | 2 | 1 | 20.34 | 22.37 | 22.95 | 49.14 | 63.64 | 50.98 | 49.94 | 34.71 | 25.46 | 46.14 | 53.40 | 32.26 | 60.69 | 59.10 | 13.59 |
| Bleak | 1136 | 1 | 2 | 1 | 22.00 | 19.16 | 19.63 | 39.49 | 56.28 | 49.59 | 45.63 | 34.67 | 24.43 | 44.09 | 50.14 | 29.79 | 57.10 | 55.90 | 11.82 |
| Bleak | 1137 | 1 | 2 | 1 | 23.49 | 22.65 | 22.00 | 45.49 | 66.46 | 58.85 | 50.82 | 38.84 | 30.06 | 51.43 | 55.94 | 35.59 | 72.63 | 68.66 | 14.78 |
| Bleak | 1138 | 1 | 2 | 1 | 26.46 | 22.56 | 24.41 | 47.19 | 68.27 | 63.78 | 58.25 | 38.32 | 27.72 | 51.57 | 56.87 | 33.02 | 64.27 | 62.39 | 13.02 |
| Bleak | 1139 | 1 | 2 | 1 | 32.34 | 27.45 | 30.27 | 62.87 | 87.94 | 73.10 | 69.50 | 51.89 | 43.46 | 58.99 | 65.77 | 50.04 | 85.60 | 79.90 | 18.88 |
| Bleak | 1140 | 1 | 2 | 1 | 25.43 | 24.40 | 23.82 | 44.71 | 63.18 | 55.93 | 52.31 | 35.57 | 26.51 | 51.48 | 56.04 | 35.15 | 68.72 | 66.92 | 13.29 |
| Bleak | 1141 | 1 | 2 | 1 | 20.34 | 19.26 | 17.81 | 29.20 | 45.83 | 39.88 | 35.78 | 27.27 | 20.55 | 36.55 | 40.05 | 25.08 | 47.00 | 44.60 | 10.25 |
| Bleak | 1142 | 1 | 2 | 1 | 27.56 | 25.42 | 26.39 | 52.08 | 75.80 | 71.34 | 63.73 | 45.87 | 37.77 | 58.28 | 66.48 | 41.13 | 79.59 | 78.22 | 15.37 |
| Bleak | 1143 | 1 | 2 | 1 | 23.21 | 20.94 | 22.31 | 41.33 | 59.14 | 51.93 | 49.53 | 34.43 | 22.65 | 52.21 | 55.81 | 31.56 | 66.75 | 62.25 | 13.48 |
| Bleak | 1144 | 1 | 2 | 1 | 23.77 | 23.46 | 24.26 | 46.94 | 70.45 | 62.32 | 53.93 | 38.55 | 28.46 | 47.67 | 53.06 | 34.54 | 66.45 | 63.08 | 14.13 |
| Bleak | 1145 | 1 | 2 | 1 | 20.99 | 18.20 | 20.12 | 39.00 | 51.74 | 47.35 | 45.58 | 32.31 | 24.34 | 42.34 | 46.55 | 33.87 | 62.52 | 59.90 | 13.18 |
| Bleak | 1146 | 1 | 2 | 1 | 28.37 | 26.70 | 27.41 | 50.22 | 77.21 | 68.25 | 61.44 | 47.07 | 37.55 | 57.48 | 63.07 | 43.30 | 75.08 | 70.26 | 15.57 |
| Bleak | 1147 | 1 | 2 | 1 | 30.45 | 29.56 | 27.86 | 54.09 | 75.37 | 66.51 | 63.95 | 43.94 | 34.05 | 57.01 | 64.80 | 41.31 | 77.91 | 75.35 | 15.98 |
| Bleak | 1148 | 1 | 2 | 1 | 28.94 | 26.36 | 26.61 | 49.73 | 71.23 | 65.75 | 61.05 | 44.70 | 32.27 | 55.97 | 64.25 | 42.08 | 81.01 | 79.93 | 14.61 |
| Bleak | 1149 | 1 | 2 | 1 | 27.96 | 26.07 | 26.76 | 53.87 | 74.08 | 65.71 | 64.31 | 46.38 | 37.13 | 56.02 | 65.25 | 40.71 | 76.19 | 76.24 | 15.56 |
| Bleak | 1150 | 1 | 2 | 1 | 22.32 | 22.85 | 23.82 | 45.77 | 66.40 | 57.68 | 52.94 | 38.89 | 27.51 | 43.94 | 52.04 | 38.29 | 63.53 | 64.46 | 12.95 |
| Bleak | 1151 | 1 | 2 | 1 | 24.35 | 21.61 | 22.36 | 41.61 | 60.37 | 50.29 | 46.06 | 34.59 | 24.53 | 47.02 | 50.55 | 34.17 | 69.30 | 66.61 | 14.96 |
| Bleak | 1152 | 1 | 2 | 1 | 23.74 | 25.34 | 25.06 | 51.07 | 75.89 | 67.01 | 58.72 | 42.99 | 32.65 | 60.49 | 65.20 | 39.11 | 77.13 | 75.98 | 15.40 |
| Bleak | 1153 | 1 | 2 | 1 | 27.63 | 27.51 | 26.33 | 54.57 | 80.17 | 72.01 | 66.39 | 41.73 | 25.18 | 62.01 | 66.88 | 39.69 | 78.82 | 75.82 | 14.55 |
| Bleak | 1154 | 1 | 2 | 1 | 23.35 | 20.36 | 22.03 | 41.10 | 58.41 | 53.51 | 47.56 | 33.63 | 22.68 | 46.25 | 51.90 | 33.64 | 59.99 | 57.10 | 12.66 |
| Bleak | 1155 | 1 | 2 | 1 | 29.17 | 26.22 | 27.28 | 48.53 | 75.16 | 71.93 | 62.31 | 45.58 | 33.70 | 54.51 | 60.66 | 42.60 | 79.66 | 75.19 | 16.98 |
| Bleak | 1156 | 1 | 2 | 1 | 25.34 | 24.74 | 25.04 | 49.22 | 72.37 | 64.80 | 57.71 | 43.02 | 26.72 | 56.89 | 62.24 | 38.87 | 68.74 | 67.93 | 16.58 |
| Bleak | 1157 | 1 | 2 | 1 | 27.72 | 23.35 | 25.20 | 51.17 | 79.31 | 72.66 | 60.49 | 46.49 | 42.71 | 52.18 | 60.40 | 39.91 | 67.86 | 64.27 | 15.50 |
| Bleak | 1158 | 1 | 2 | 1 | 25.83 | 25.83 | 25.13 | 42.47 | 62.92 | 52.83 | 50.21 | 36.55 | 28.10 | 55.53 | 60.76 | 35.34 | 68.68 | 64.08 | 14.70 |
| Bleak | 1159 | 1 | 2 | 1 | 18.98 | 17.81 | 19.70 | 37.94 | 54.97 | 48.57 | 46.37 | 32.07 | 23.15 | 43.46 | 48.71 | 28.43 | 50.95 | 48.27 | 10.66 |
| Bleak | 1160 | 1 | 2 | 1 | 24.67 | 19.81 | 23.74 | 45.92 | 65.12 | 57.27 | 54.65 | 36.94 | 32.65 | 50.82 | 54.43 | 33.91 | 65.56 | 60.69 | 13.46 |
| Bleak | 1131 | 1 | 2 | 2 | 27.59 | 26.84 | 25.92 | 50.93 | 80.65 | 73.49 | 61.27 | 46.95 | 35.68 | 56.24 | 62.98 | 40.76 | 78.42 | 74.77 | 16.30 |
| Bleak | 1132 | 1 | 2 | 2 | 21.83 | 23.47 | 23.00 | 43.14 | 60.42 | 51.79 | 48.85 | 35.28 | 26.03 | 49.11 | 52.67 | 34.11 | 62.21 | 59.07 | 13.68 |
| Bleak | 1133 | 1 | 2 | 2 | 24.30 | 21.35 | 21.28 | 46.42 | 61.23 | 53.40 | 56.27 | 35.09 | 21.91 | 50.95 | 55.54 | 33.37 | 64.62 | 60.82 | 13.86 |
| Bleak | 1134 | 1 | 2 | 2 | 21.00 | 17.05 | 18.64 | 42.52 | 54.54 | 47.62 | 46.39 | 32.74 | 23.37 | 41.69 | 45.39 | 29.89 | 52.52 | 51.67 | 12.79 |
| Bleak | 1135 | 1 | 2 | 2 | 21.23 | 21.49 | 23.18 | 46.52 | 61.46 | 50.09 | 50.23 | 35.74 | 23.67 | 46.51 | 53.09 | 32.53 | 60.67 | 59.45 | 12.36 |
| Bleak | 1136 | 1 | 2 | 2 | 21.74 | 20.91 | 20.33 | 39.13 | 56.99 | 48.62 | 43.65 | 32.41 | 23.00 | 42.56 | 49.61 | 31.01 | 56.14 | 55.45 | 12.09 |
| Bleak | 1137 | 1 | 2 | 2 | 24.78 | 23.46 | 22.05 | 43.91 | 68.07 | 59.22 | 50.67 | 38.71 | 28.16 | 53.22 | 56.21 | 35.10 | 67.97 | 64.27 | 12.99 |
| Bleak | 1138 | 1 | 2 | 2 | 26.17 | 21.35 | 22.61 | 46.81 | 69.45 | 63.04 | 56.14 | 38.06 | 28.55 | 50.42 | 54.51 | 32.30 | 61.41 | 60.58 | 14.22 |
| Bleak | 1139 | 1 | 2 | 2 | 29.90 | 27.45 | 28.61 | 64.46 | 86.81 | 71.88 | 70.12 | 52.35 | 42.52 | 64.70 | 66.75 | 47.89 | 81.29 | 76.89 | 17.61 |
| Bleak | 1140 | 1 | 2 | 2 | 25.95 | 25.75 | 25.32 | 42.84 | 62.81 | 54.36 | 50.09 | 37.65 | 27.01 | 50.47 | 57.47 | 32.90 | 65.14 | 63.07 | 12.66 |
| Bleak | 1141 | 1 | 2 | 2 | 20.74 | 20.59 | 17.95 | 30.44 | 47.99 | 40.85 | 36.39 | 28.71 | 20.00 | 36.19 | 40.19 | 25.45 | 46.25 | 43.67 | 10.85 |
| Bleak | 1142 | 1 | 2 | 2 | 29.41 | 25.88 | 27.59 | 52.49 | 75.93 | 68.59 | 63.54 | 45.51 | 37.47 | 55.05 | 64.97 | 41.93 | 80.35 | 77.92 | 14.92 |
| Bleak | 1143 | 1 | 2 | 2 | 23.74 | 21.03 | 21.42 | 42.60 | 61.83 | 53.22 | 47.97 | 35.49 | 25.24 | 51.18 | 55.69 | 32.58 | 65.58 | 60.71 | 14.28 |
| Bleak | 1144 | 1 | 2 | 2 | 25.04 | 22.89 | 22.38 | 46.46 | 68.03 | 60.47 | 54.10 | 39.37 | 28.71 | 48.31 | 52.56 | 35.28 | 64.27 | 61.42 | 13.02 |
| Bleak | 1145 | 1 | 2 | 2 | 22.33 | 17.26 | 19.14 | 36.95 | 53.84 | 49.48 | 46.91 | 32.27 | 22.77 | 41.53 | 47.37 | 33.44 | 58.81 | 58.81 | 12.29 |
| Bleak | 1146 | 1 | 2 | 2 | 27.71 | 26.32 | 25.77 | 54.47 | 76.58 | 67.34 | 62.63 | 44.56 | 34.11 | 61.57 | 64.40 | 43.66 | 73.91 | 69.65 | 16.98 |
| Bleak | 1147 | 1 | 2 | 2 | 28.48 | 28.07 | 26.13 | 54.97 | 73.73 | 62.68 | 63.42 | 44.51 | 35.02 | 58.43 | 66.07 | 40.90 | 77.58 | 76.56 | 16.89 |
| Bleak | 1148 | 1 | 2 | 2 | 27.93 | 25.75 | 25.32 | 50.89 | 75.97 | 68.93 | 61.42 | 45.48 | 30.89 | 55.51 | 63.08 | 42.90 | 78.25 | 76.39 | 13.34 |
| Bleak | 1149 | 1 | 2 | 2 | 29.49 | 25.28 | 26.19 | 53.58 | 78.75 | 68.42 | 62.27 | 47.45 | 38.62 | 58.34 | 66.60 | 41.51 | 77.05 | 76.22 | 16.02 |
| Bleak | 1150 | 1 | 2 | 2 | 23.24 | 22.54 | 24.11 | 45.11 | 65.35 | 58.27 | 53.96 | 39.29 | 27.45 | 44.53 | 52.16 | 37.07 | 65.09 | 65.65 | 13.25 |
| Bleak | 1151 | 1 | 2 | 2 | 22.85 | 20.83 | 21.80 | 41.95 | 62.30 | 57.18 | 50.95 | 36.00 | 22.60 | 49.23 | 52.48 | 32.75 | 66.46 | 62.88 | 12.95 |
| Bleak | 1152 | 1 | 2 | 2 | 24.85 | 24.97 | 24.64 | 54.14 | 78.45 | 69.48 | 60.12 | 42.15 | 31.81 | 60.98 | 64.76 | 39.63 | 76.10 | 75.39 | 16.28 |
| Bleak | 1153 | 1 | 2 | 2 | 27.77 | 28.85 | 27.49 | 55.20 | 82.34 | 70.98 | 61.58 | 43.37 | 30.09 | 60.17 | 65.70 | 39.63 | 81.88 | 77.17 | 16.32 |
| Bleak | 1154 | 1 | 2 | 2 | 20.86 | 22.11 | 21.70 | 38.75 | 57.64 | 51.14 | 45.14 | 35.95 | 24.94 | 45.58 | 50.92 | 32.46 | 58.46 | 56.18 | 13.26 |
| Bleak | 1155 | 1 | 2 | 2 | 31.18 | 25.38 | 28.07 | 49.73 | 79.34 | 74.49 | 61.82 | 46.33 | 32.75 | 56.74 | 61.06 | 42.46 | 79.03 | 74.04 | 16.41 |
| Bleak | 1156 | 1 | 2 | 2 | 24.41 | 23.07 | 22.92 | 48.64 | 71.64 | 62.90 | 55.45 | 41.46 | 29.29 | 53.21 | 62.14 | 38.69 | 73.28 | 70.57 | 16.16 |
| Bleak | 1157 | 1 | 2 | 2 | 27.49 | 22.76 | 23.63 | 52.79 | 77.51 | 69.05 | 61.38 | 44.50 | 37.35 | 54.63 | 61.95 | 38.57 | 67.15 | 64.62 | 16.80 |
| Bleak | 1158 | 1 | 2 | 2 | 25.81 | 24.41 | 25.70 | 46.07 | 64.23 | 52.79 | 50.64 | 38.71 | 28.99 | 55.80 | 59.86 | 36.24 | 70.85 | 67.08 | 14.87 |
| Bleak | 1159 | 1 | 2 | 2 | 18.75 | 16.62 | 20.82 | 39.04 | 54.50 | 47.68 | 46.27 | 33.09 | 23.89 | 42.51 | 48.47 | 30.08 | 52.37 | 50.36 | 12.09 |
| Bleak | 1160 | 1 | 2 | 2 | 24.20 | 20.42 | 23.00 | 46.93 | 66.64 | 60.58 | 58.17 | 35.56 | 29.14 | 53.45 | 57.88 | 33.08 | 64.53 | 63.50 | 14.25 |
| Bleak | 1131 | 1 | 2 | 3 | 26.35 | 26.02 | 26.52 | 51.49 | 81.17 | 72.74 | 59.91 | 47.77 | 38.90 | 54.31 | 60.84 | 40.71 | 78.25 | 73.28 | 15.45 |
| Bleak | 1132 | 1 | 2 | 3 | 22.07 | 22.11 | 20.53 | 42.07 | 59.20 | 50.81 | 47.73 | 34.90 | 26.63 | 48.72 | 52.10 | 35.45 | 64.99 | 59.99 | 13.29 |
| Bleak | 1133 | 1 | 2 | 3 | 22.43 | 21.57 | 20.47 | 45.04 | 61.42 | 55.16 | 52.34 | 37.00 | 27.50 | 50.31 | 54.73 | 32.35 | 63.48 | 62.02 | 14.17 |
| Bleak | 1134 | 1 | 2 | 3 | 19.72 | 16.74 | 20.27 | 40.74 | 54.23 | 46.73 | 46.59 | 34.09 | 23.33 | 39.84 | 46.33 | 30.21 | 50.24 | 49.99 | 12.15 |
| Bleak | 1135 | 1 | 2 | 3 | 21.78 | 23.25 | 22.98 | 45.64 | 60.63 | 49.76 | 49.99 | 34.22 | 24.78 | 46.51 | 52.98 | 35.05 | 63.10 | 62.16 | 13.28 |
| Bleak | 1136 | 1 | 2 | 3 | 20.68 | 20.02 | 19.63 | 40.87 | 56.92 | 48.41 | 45.39 | 32.81 | 23.15 | 42.99 | 48.40 | 29.50 | 55.58 | 54.14 | 12.36 |
| Bleak | 1137 | 1 | 2 | 3 | 24.08 | 23.23 | 22.68 | 44.16 | 67.53 | 58.99 | 51.74 | 40.06 | 28.30 | 52.84 | 56.22 | 33.30 | 67.96 | 64.10 | 15.98 |
| Bleak | 1138 | 1 | 2 | 3 | 27.37 | 22.23 | 22.61 | 45.57 | 68.63 | 63.34 | 55.31 | 37.44 | 31.39 | 49.28 | 55.09 | 32.22 | 63.98 | 59.98 | 13.32 |
| Bleak | 1139 | 1 | 2 | 3 | 33.34 | 25.48 | 29.20 | 61.81 | 86.86 | 75.72 | 70.94 | 52.35 | 42.92 | 61.96 | 66.79 | 49.12 | 83.41 | 77.49 | 18.33 |
| Bleak | 1140 | 1 | 2 | 3 | 24.58 | 24.53 | 24.16 | 43.75 | 64.15 | 57.29 | 50.66 | 35.17 | 26.77 | 49.23 | 56.30 | 33.36 | 63.02 | 62.44 | 12.67 |
| Bleak | 1141 | 1 | 2 | 3 | 19.06 | 20.02 | 18.33 | 32.04 | 43.94 | 36.23 | 36.12 | 27.59 | 19.70 | 37.36 | 41.98 | 25.55 | 48.13 | 43.94 | 10.55 |
| Bleak | 1142 | 1 | 2 | 3 | 29.20 | 24.64 | 26.69 | 54.56 | 76.82 | 68.27 | 62.58 | 45.17 | 37.31 | 54.90 | 65.78 | 40.99 | 75.49 | 76.45 | 15.99 |
| Bleak | 1143 | 1 | 2 | 3 | 22.97 | 21.73 | 22.91 | 41.65 | 34.99 | 53.49 | 48.39 | 33.93 | 24.83 | 49.43 | 52.87 | 33.03 | 66.75 | 63.72 | 12.54 |
| Bleak | 1144 | 1 | 2 | 3 | 24.62 | 23.27 | 23.70 | 51.68 | 71.99 | 63.78 | 56.01 | 40.31 | 29.01 | 47.53 | 52.72 | 35.10 | 64.15 | 61.42 | 12.75 |
| Bleak | 1145 | 1 | 2 | 3 | 21.34 | 17.93 | 20.38 | 35.97 | 51.34 | 46.29 | 46.67 | 33.00 | 22.70 | 43.79 | 49.34 | 32.88 | 61.20 | 60.20 | 12.19 |
| Bleak | 1146 | 1 | 2 | 3 | 27.74 | 25.46 | 26.26 | 56.59 | 78.64 | 67.01 | 61.95 | 45.84 | 36.57 | 60.29 | 62.76 | 43.83 | 76.61 | 69.36 | 16.51 |
| Bleak | 1147 | 1 | 2 | 3 | 31.45 | 28.41 | 28.60 | 57.29 | 75.50 | 65.60 | 64.40 | 44.70 | 36.24 | 56.94 | 65.88 | 41.41 | 76.31 | 75.65 | 16.58 |
| Bleak | 1148 | 1 | 2 | 3 | 28.10 | 24.52 | 26.53 | 51.95 | 78.02 | 71.36 | 62.43 | 44.63 | 31.49 | 58.10 | 65.79 | 41.42 | 80.89 | 78.27 | 14.49 |
| Bleak | 1149 | 1 | 2 | 3 | 29.22 | 26.07 | 26.78 | 53.58 | 76.13 | 67.21 | 63.03 | 46.24 | 39.38 | 54.94 | 63.29 | 41.38 | 75.81 | 74.69 | 15.78 |
| Bleak | 1150 | 1 | 2 | 3 | 23.42 | 22.11 | 22.02 | 45.61 | 65.98 | 58.52 | 54.70 | 39.58 | 28.69 | 46.11 | 51.91 | 38.70 | 66.77 | 65.91 | 12.59 |
| Bleak | 1151 | 1 | 2 | 3 | 22.49 | 20.76 | 22.17 | 40.99 | 57.09 | 49.71 | 47.37 | 36.65 | 22.37 | 51.29 | 55.73 | 33.43 | 69.49 | 66.07 | 13.35 |
| Bleak | 1152 | 1 | 2 | 3 | 23.09 | 24.40 | 24.56 | 54.14 | 77.39 | 67.44 | 59.62 | 41.32 | 34.59 | 59.99 | 63.44 | 39.59 | 78.30 | 76.28 | 15.37 |
| Bleak | 1153 | 1 | 2 | 3 | 28.99 | 28.00 | 28.00 | 53.72 | 78.25 | 70.69 | 62.63 | 42.25 | 28.35 | 62.54 | 67.14 | 41.11 | 81.11 | 78.04 | 19.61 |
| Bleak | 1154 | 1 | 2 | 3 | 21.76 | 20.23 | 22.05 | 38.84 | 57.10 | 51.25 | 47.92 | 34.36 | 20.81 | 47.49 | 52.75 | 31.85 | 59.70 | 56.89 | 12.15 |
| Bleak | 1155 | 1 | 2 | 3 | 29.67 | 25.26 | 25.93 | 49.43 | 76.42 | 72.67 | 62.56 | 47.47 | 30.57 | 57.98 | 63.38 | 41.53 | 77.18 | 74.28 | 15.99 |
| Bleak | 1156 | 1 | 2 | 3 | 24.86 | 22.33 | 24.78 | 48.28 | 70.23 | 61.95 | 56.04 | 41.32 | 29.85 | 53.78 | 59.65 | 38.56 | 72.20 | 69.39 | 16.88 |
| Bleak | 1157 | 1 | 2 | 3 | 29.85 | 24.68 | 24.16 | 52.21 | 75.35 | 69.34 | 61.15 | 45.30 | 36.68 | 57.23 | 64.02 | 37.50 | 66.27 | 63.12 | 15.14 |
| Bleak | 1158 | 1 | 2 | 3 | 26.36 | 26.14 | 26.54 | 42.93 | 62.60 | 53.46 | 52.42 | 37.18 | 25.72 | 56.48 | 59.86 | 36.26 | 69.67 | 65.50 | 15.07 |
| Bleak | 1159 | 1 | 2 | 3 | 20.53 | 17.35 | 19.57 | 40.87 | 53.47 | 47.97 | 47.12 | 33.17 | 25.67 | 42.91 | 47.55 | 31.81 | 54.81 | 51.27 | 12.72 |
| Bleak | 1160 | 1 | 2 | 3 | 27.59 | 20.51 | 24.68 | 48.12 | 66.27 | 59.68 | 56.06 | 34.54 | 32.52 | 49.27 | 55.70 | 34.52 | 65.69 | 62.51 | 13.34 |
| Bleak | 1221 | 1 | 3 | 1 | 30.38 | 27.13 | 26.97 | 46.30 | 69.41 | 58.82 | 53.77 | 42.86 | 33.25 | 50.84 | 55.83 | 37.64 | 71.02 | 66.89 | 15.40 |
| Bleak | 1222 | 1 | 3 | 1 | 21.08 | 19.85 | 20.65 | 34.70 | 55.84 | 52.57 | 45.17 | 32.97 | 26.09 | 39.80 | 44.47 | 28.85 | 55.71 | 53.03 | 13.02 |
| Bleak | 1223 | 1 | 3 | 1 | 18.08 | 17.63 | 18.48 | 39.06 | 58.06 | 52.60 | 44.91 | 31.87 | 23.52 | 45.22 | 48.03 | 28.43 | 60.93 | 58.48 | 10.59 |
| Bleak | 1224 | 1 | 3 | 1 | 26.86 | 23.05 | 23.00 | 46.46 | 66.05 | 57.92 | 52.39 | 37.35 | 30.16 | 56.06 | 59.49 | 35.54 | 68.86 | 66.02 | 15.08 |
| Bleak | 1225 | 1 | 3 | 1 | 28.18 | 24.13 | 22.30 | 46.11 | 65.22 | 55.54 | 51.73 | 37.68 | 33.89 | 49.14 | 55.11 | 35.87 | 71.06 | 67.28 | 13.35 |
| Bleak | 1226 | 1 | 3 | 1 | 20.15 | 17.36 | 18.39 | 33.92 | 49.61 | 43.37 | 40.13 | 29.61 | 22.65 | 37.18 | 41.51 | 25.48 | 49.49 | 47.63 | 11.49 |
| Bleak | 1227 | 1 | 3 | 1 | 20.92 | 22.61 | 20.30 | 38.11 | 56.88 | 49.76 | 44.82 | 33.48 | 27.33 | 40.71 | 45.56 | 32.62 | 56.73 | 54.12 | 11.76 |
| Bleak | 1228 | 1 | 3 | 1 | 24.41 | 20.54 | 19.35 | 39.48 | 58.01 | 51.54 | 44.35 | 33.65 | 24.13 | 45.27 | 49.09 | 29.81 | 61.27 | 59.47 | 13.54 |
| Bleak | 1229 | 1 | 3 | 1 | 24.52 | 21.11 | 21.61 | 38.58 | 55.00 | 46.91 | 43.94 | 32.03 | 24.43 | 44.55 | 47.30 | 29.38 | 61.19 | 58.36 | 12.24 |
| Bleak | 1230 | 1 | 3 | 1 | 28.48 | 23.76 | 23.81 | 41.24 | 65.28 | 59.55 | 52.58 | 39.69 | 27.37 | 52.34 | 56.77 | 35.38 | 65.10 | 62.68 | 13.57 |
| Bleak | 1232 | 1 | 3 | 1 | 23.23 | 20.04 | 20.00 | 40.68 | 57.02 | 49.11 | 45.31 | 34.22 | 23.40 | 43.72 | 47.87 | 32.38 | 59.87 | 56.23 | 12.66 |
| Bleak | 1233 | 1 | 3 | 1 | 33.29 | 31.46 | 29.24 | 59.19 | 89.70 | 80.04 | 66.49 | 50.69 | 37.22 | 62.94 | 68.94 | 45.79 | 84.45 | 78.43 | 19.93 |
| Bleak | 1234 | 1 | 3 | 1 | 24.91 | 22.44 | 23.26 | 41.40 | 63.89 | 57.78 | 49.68 | 37.22 | 26.87 | 46.37 | 51.85 | 35.84 | 61.91 | 57.95 | 15.07 |
| Bleak | 1235 | 1 | 3 | 1 | 24.96 | 22.35 | 22.70 | 44.05 | 62.56 | 52.61 | 50.04 | 35.48 | 30.23 | 47.52 | 50.88 | 33.69 | 66.81 | 63.97 | 12.97 |
| Bleak | 1236 | 1 | 3 | 1 | 26.44 | 21.97 | 22.81 | 36.51 | 58.57 | 53.76 | 46.42 | 34.04 | 27.71 | 45.34 | 51.14 | 33.61 | 64.34 | 64.41 | 12.89 |
| Bleak | 1237 | 1 | 3 | 1 | 22.91 | 22.78 | 22.62 | 39.20 | 58.98 | 49.95 | 42.08 | 34.25 | 23.67 | 43.86 | 48.94 | 31.56 | 59.33 | 57.33 | 12.69 |
| Bleak | 1238 | 1 | 3 | 1 | 24.25 | 21.03 | 22.31 | 44.27 | 64.57 | 57.08 | 49.88 | 38.06 | 28.23 | 48.77 | 51.76 | 36.01 | 67.01 | 65.65 | 13.83 |
| Bleak | 1239 | 1 | 3 | 1 | 25.15 | 22.74 | 23.67 | 48.23 | 67.68 | 60.39 | 54.38 | 38.75 | 28.79 | 46.64 | 53.28 | 35.77 | 64.71 | 64.19 | 12.68 |
| Bleak | 1240 | 1 | 3 | 1 | 24.67 | 22.03 | 21.70 | 39.22 | 60.96 | 56.54 | 47.32 | 34.78 | 24.41 | 48.57 | 52.04 | 31.65 | 66.46 | 60.61 | 13.35 |
| Bleak | 1241 | 1 | 3 | 1 | 23.58 | 23.12 | 22.61 | 35.35 | 59.03 | 52.18 | 43.58 | 33.60 | 25.70 | 45.47 | 50.55 | 31.03 | 59.50 | 56.76 | 13.58 |
| Bleak | 1242 | 1 | 3 | 1 | 27.01 | 26.20 | 26.27 | 47.44 | 73.46 | 65.77 | 58.10 | 42.44 | 30.98 | 56.55 | 58.41 | 36.21 | 70.77 | 64.86 | 14.55 |
| Bleak | 1243 | 1 | 3 | 1 | 23.48 | 23.77 | 23.11 | 47.18 | 66.63 | 56.72 | 52.87 | 39.58 | 33.65 | 49.95 | 53.61 | 37.54 | 67.29 | 63.03 | 13.83 |
| Bleak | 1244 | 1 | 3 | 1 | 23.39 | 22.28 | 23.24 | 43.44 | 61.20 | 54.18 | 52.00 | 39.12 | 26.66 | 50.36 | 56.48 | 38.15 | 71.79 | 66.75 | 14.20 |
| Bleak | 1245 | 1 | 3 | 1 | 23.72 | 20.42 | 21.46 | 40.14 | 61.80 | 60.50 | 50.14 | 35.99 | 23.86 | 39.49 | 43.39 | 34.15 | 59.49 | 57.38 | 12.01 |
| Bleak | 1246 | 1 | 3 | 1 | 23.50 | 23.10 | 22.70 | 39.11 | 58.10 | 49.26 | 46.70 | 35.60 | 30.36 | 50.04 | 54.37 | 34.84 | 64.49 | 59.30 | 15.07 |
| Bleak | 1247 | 1 | 3 | 1 | 23.58 | 22.16 | 21.80 | 41.48 | 58.80 | 50.19 | 46.48 | 33.08 | 22.61 | 45.31 | 50.32 | 31.84 | 61.11 | 59.11 | 11.89 |
| Bleak | 1248 | 1 | 3 | 1 | 25.81 | 24.06 | 23.24 | 40.61 | 59.34 | 49.78 | 47.50 | 34.22 | 26.61 | 49.60 | 54.00 | 33.70 | 63.13 | 58.79 | 12.21 |
| Bleak | 1249 | 1 | 3 | 1 | 17.37 | 14.39 | 15.40 | 28.67 | 42.24 | 37.24 | 32.64 | 23.64 | 17.26 | 32.89 | 35.31 | 20.58 | 40.20 | 39.90 | 9.07 |
| Bleak | 1250 | 1 | 3 | 1 | 18.77 | 18.02 | 17.82 | 32.36 | 48.71 | 42.37 | 39.29 | 26.25 | 20.04 | 38.63 | 42.40 | 25.75 | 49.20 | 47.92 | 12.37 |
| Bleak | 1250 | 1 | 3 | 1 | 21.04 | 21.10 | 20.64 | 35.38 | 53.67 | 46.17 | 42.57 | 31.93 | 23.40 | 41.45 | 46.11 | 30.06 | 58.05 | 53.32 | 12.09 |
| Bleak | 1221 | 1 | 3 | 2 | 29.64 | 25.88 | 28.26 | 44.81 | 67.68 | 57.31 | 54.30 | 41.66 | 28.76 | 52.13 | 56.40 | 36.34 | 70.35 | 64.22 | 15.07 |
| Bleak | 1222 | 1 | 3 | 2 | 23.54 | 21.57 | 19.90 | 37.58 | 56.22 | 51.07 | 45.08 | 32.93 | 24.48 | 40.79 | 45.51 | 28.58 | 56.16 | 55.59 | 11.94 |
| Bleak | 1223 | 1 | 3 | 2 | 19.39 | 18.31 | 19.89 | 37.45 | 56.62 | 51.16 | 45.02 | 30.84 | 25.08 | 40.73 | 44.75 | 28.99 | 60.54 | 58.48 | 10.59 |
| Bleak | 1224 | 1 | 3 | 2 | 27.28 | 22.66 | 25.63 | 43.95 | 66.50 | 60.96 | 54.31 | 38.39 | 29.01 | 55.23 | 60.12 | 34.51 | 66.54 | 63.92 | 14.77 |
| Bleak | 1225 | 1 | 3 | 2 | 28.37 | 24.48 | 22.65 | 46.52 | 62.69 | 53.70 | 49.86 | 34.71 | 31.07 | 50.06 | 55.76 | 34.97 | 69.92 | 69.42 | 14.80 |
| Bleak | 1226 | 1 | 3 | 2 | 17.98 | 17.54 | 18.08 | 32.86 | 49.65 | 43.78 | 38.70 | 29.01 | 23.10 | 38.63 | 42.08 | 26.62 | 52.64 | 50.34 | 11.49 |
| Bleak | 1227 | 1 | 3 | 2 | 20.76 | 22.97 | 20.53 | 36.92 | 57.56 | 51.59 | 44.47 | 33.87 | 26.69 | 44.17 | 47.41 | 30.19 | 55.81 | 53.09 | 12.67 |
| Bleak | 1228 | 1 | 3 | 2 | 21.57 | 17.66 | 19.92 | 39.18 | 59.11 | 53.07 | 43.81 | 34.71 | 24.48 | 45.27 | 50.61 | 29.72 | 63.17 | 61.01 | 12.13 |
| Bleak | 1229 | 1 | 3 | 2 | 23.50 | 19.52 | 20.30 | 37.51 | 54.86 | 46.88 | 44.43 | 32.03 | 23.90 | 41.91 | 48.00 | 30.87 | 60.15 | 57.62 | 12.89 |
| Bleak | 1230 | 1 | 3 | 2 | 26.51 | 24.40 | 24.43 | 41.43 | 67.53 | 61.01 | 49.68 | 40.30 | 31.59 | 50.44 | 56.50 | 34.71 | 64.44 | 61.68 | 14.52 |
| Bleak | 1232 | 1 | 3 | 2 | 22.17 | 21.81 | 20.12 | 39.26 | 57.84 | 50.05 | 45.82 | 33.84 | 21.84 | 43.72 | 49.22 | 30.73 | 56.14 | 55.15 | 12.37 |
| Bleak | 1233 | 1 | 3 | 2 | 31.61 | 28.99 | 29.84 | 60.69 | 91.40 | 82.45 | 68.39 | 51.81 | 38.58 | 62.58 | 69.71 | 45.24 | 84.83 | 78.79 | 19.00 |
| Bleak | 1234 | 1 | 3 | 2 | 24.83 | 22.44 | 22.94 | 39.62 | 66.10 | 60.11 | 49.42 | 38.90 | 26.61 | 47.45 | 52.37 | 34.69 | 61.49 | 58.28 | 13.87 |
| Bleak | 1235 | 1 | 3 | 2 | 23.45 | 23.05 | 22.46 | 46.77 | 60.40 | 52.29 | 52.10 | 35.74 | 30.86 | 46.65 | 51.22 | 34.66 | 67.55 | 65.50 | 12.66 |
| Bleak | 1236 | 1 | 3 | 2 | 23.74 | 21.98 | 21.48 | 38.05 | 58.00 | 51.54 | 45.40 | 34.43 | 27.26 | 46.34 | 52.44 | 31.68 | 62.24 | 61.31 | 13.38 |
| Bleak | 1237 | 1 | 3 | 2 | 22.43 | 21.49 | 22.01 | 36.48 | 58.12 | 50.30 | 42.40 | 34.09 | 24.26 | 41.56 | 48.00 | 31.43 | 59.05 | 57.84 | 13.43 |
| Bleak | 1238 | 1 | 3 | 2 | 22.40 | 21.61 | 21.42 | 42.81 | 64.31 | 57.46 | 51.12 | 36.06 | 26.58 | 47.76 | 51.90 | 36.13 | 65.49 | 63.00 | 11.89 |
| Bleak | 1239 | 1 | 3 | 2 | 26.43 | 22.30 | 24.20 | 48.18 | 67.15 | 58.97 | 54.74 | 39.97 | 30.06 | 45.16 | 50.96 | 35.78 | 64.64 | 62.25 | 12.79 |
| Bleak | 1240 | 1 | 3 | 2 | 24.58 | 20.70 | 21.42 | 40.94 | 61.76 | 53.89 | 45.89 | 34.64 | 25.02 | 48.92 | 52.71 | 32.89 | 65.24 | 62.43 | 13.73 |
| Bleak | 1241 | 1 | 3 | 2 | 19.49 | 21.58 | 21.75 | 36.85 | 59.49 | 53.03 | 44.30 | 33.72 | 26.01 | 46.13 | 50.32 | 31.16 | 59.50 | 56.48 | 13.32 |
| Bleak | 1242 | 1 | 3 | 2 | 28.02 | 24.59 | 26.55 | 48.08 | 73.46 | 65.43 | 56.60 | 42.43 | 30.33 | 54.25 | 58.31 | 36.73 | 68.07 | 64.62 | 13.32 |
| Bleak | 1243 | 1 | 3 | 2 | 25.15 | 22.65 | 22.52 | 49.60 | 68.40 | 58.81 | 52.84 | 39.07 | 34.24 | 49.43 | 53.89 | 35.53 | 67.44 | 63.38 | 13.35 |
| Bleak | 1244 | 1 | 3 | 2 | 26.04 | 23.07 | 23.22 | 45.83 | 62.18 | 54.11 | 54.48 | 38.54 | 24.30 | 49.01 | 55.54 | 36.00 | 68.73 | 65.95 | 13.56 |
| Bleak | 1245 | 1 | 3 | 2 | 24.26 | 20.32 | 21.70 | 40.11 | 61.05 | 57.48 | 48.80 | 35.32 | 21.70 | 41.90 | 46.16 | 32.04 | 56.94 | 53.97 | 11.73 |
| Bleak | 1246 | 1 | 3 | 2 | 24.94 | 21.87 | 22.17 | 38.31 | 60.14 | 51.63 | 45.77 | 36.42 | 27.83 | 54.18 | 57.21 | 33.25 | 63.68 | 58.87 | 13.09 |
| Bleak | 1247 | 1 | 3 | 2 | 22.31 | 21.94 | 21.50 | 39.85 | 59.49 | 52.53 | 47.15 | 33.18 | 25.02 | 43.18 | 48.36 | 32.60 | 60.54 | 56.95 | 12.09 |
| Bleak | 1248 | 1 | 3 | 2 | 26.43 | 24.38 | 22.76 | 40.38 | 59.05 | 48.89 | 46.36 | 34.82 | 28.41 | 48.58 | 51.74 | 34.37 | 62.51 | 59.99 | 11.77 |
| Bleak | 1249 | 1 | 3 | 2 | 17.13 | 14.78 | 15.40 | 28.05 | 41.09 | 35.24 | 31.12 | 23.54 | 17.20 | 32.89 | 34.89 | 21.30 | 40.85 | 37.65 | 8.20 |
| Bleak | 1250 | 1 | 3 | 2 | 19.26 | 17.57 | 18.21 | 33.29 | 50.01 | 42.37 | 36.24 | 26.48 | 22.98 | 36.35 | 41.25 | 27.35 | 52.19 | 48.83 | 12.12 |
| Bleak | 1250 | 1 | 3 | 2 | 22.13 | 21.07 | 19.78 | 39.39 | 53.78 | 46.28 | 42.74 | 31.23 | 24.51 | 40.57 | 44.36 | 29.38 | 56.81 | 51.41 | 12.24 |
| Bleak | 1221 | 1 | 3 | 3 | 27.29 | 26.03 | 26.69 | 45.28 | 68.77 | 59.12 | 53.55 | 42.61 | 29.72 | 53.50 | 56.55 | 37.29 | 71.76 | 66.16 | 15.37 |
| Bleak | 1222 | 1 | 3 | 3 | 21.38 | 18.98 | 21.10 | 38.10 | 59.04 | 52.49 | 44.57 | 32.53 | 24.94 | 40.04 | 45.05 | 27.77 | 56.73 | 53.12 | 12.15 |
| Bleak | 1223 | 1 | 3 | 3 | 20.68 | 19.28 | 20.50 | 39.11 | 57.30 | 50.87 | 45.39 | 30.67 | 22.32 | 44.31 | 47.30 | 28.26 | 59.60 | 57.46 | 10.06 |
| Bleak | 1224 | 1 | 3 | 3 | 28.56 | 23.64 | 24.60 | 44.75 | 67.52 | 60.04 | 51.84 | 37.22 | 30.74 | 56.15 | 61.57 | 36.27 | 68.86 | 64.56 | 14.50 |
| Bleak | 1225 | 1 | 3 | 3 | 29.66 | 25.06 | 22.60 | 44.45 | 64.06 | 55.02 | 50.65 | 37.39 | 34.14 | 50.16 | 54.45 | 35.19 | 68.53 | 66.35 | 13.65 |
| Bleak | 1226 | 1 | 3 | 3 | 20.15 | 18.21 | 18.75 | 32.12 | 47.87 | 42.14 | 39.88 | 29.00 | 22.21 | 38.22 | 42.59 | 26.56 | 50.66 | 48.23 | 11.17 |
| Bleak | 1227 | 1 | 3 | 3 | 22.69 | 19.92 | 19.31 | 39.39 | 57.70 | 51.26 | 44.47 | 33.53 | 27.59 | 40.33 | 44.73 | 31.85 | 56.68 | 55.56 | 12.37 |
| Bleak | 1228 | 1 | 3 | 3 | 24.51 | 20.15 | 19.78 | 38.58 | 58.01 | 52.19 | 44.90 | 34.04 | 22.30 | 44.95 | 48.80 | 28.77 | 61.58 | 59.52 | 12.59 |
| Bleak | 1229 | 1 | 3 | 3 | 24.09 | 19.33 | 20.88 | 38.66 | 55.94 | 47.53 | 45.34 | 32.03 | 23.40 | 43.95 | 49.00 | 29.93 | 63.38 | 59.85 | 12.49 |
| Bleak | 1230 | 1 | 3 | 3 | 27.87 | 23.73 | 24.43 | 41.84 | 66.20 | 60.15 | 52.52 | 39.30 | 28.49 | 51.10 | 54.77 | 36.42 | 66.24 | 63.58 | 13.87 |
| Bleak | 1232 | 1 | 3 | 3 | 23.54 | 20.99 | 18.81 | 41.18 | 57.15 | 48.86 | 45.64 | 32.89 | 24.78 | 41.32 | 47.89 | 33.43 | 58.82 | 54.96 | 12.36 |
| Bleak | 1233 | 1 | 3 | 3 | 33.64 | 29.10 | 30.16 | 56.76 | 91.05 | 81.66 | 67.45 | 50.07 | 37.55 | 62.21 | 71.40 | 45.73 | 83.21 | 79.93 | 19.41 |
| Bleak | 1234 | 1 | 3 | 3 | 24.58 | 23.57 | 22.00 | 40.11 | 65.38 | 59.76 | 48.69 | 38.42 | 27.86 | 47.24 | 52.46 | 35.10 | 62.83 | 58.58 | 13.88 |
| Bleak | 1235 | 1 | 3 | 3 | 25.80 | 22.30 | 21.95 | 44.45 | 61.64 | 53.47 | 52.10 | 33.95 | 30.68 | 47.08 | 50.21 | 34.06 | 67.33 | 64.85 | 13.28 |
| Bleak | 1236 | 1 | 3 | 3 | 24.83 | 23.07 | 22.08 | 38.54 | 57.24 | 50.64 | 46.94 | 32.68 | 25.53 | 45.10 | 51.96 | 32.67 | 63.38 | 63.81 | 13.01 |
| Bleak | 1237 | 1 | 3 | 3 | 21.89 | 21.07 | 20.80 | 36.17 | 58.72 | 50.60 | 41.63 | 33.37 | 25.16 | 42.15 | 47.59 | 31.07 | 59.72 | 55.97 | 12.96 |
| Bleak | 1238 | 1 | 3 | 3 | 23.45 | 21.10 | 22.01 | 41.45 | 64.87 | 58.92 | 53.98 | 37.56 | 24.64 | 46.82 | 53.65 | 34.38 | 64.43 | 63.97 | 13.48 |
| Bleak | 1239 | 1 | 3 | 3 | 27.92 | 20.27 | 22.68 | 48.94 | 68.67 | 60.09 | 54.87 | 39.12 | 29.99 | 43.68 | 47.99 | 37.09 | 64.81 | 62.29 | 13.05 |
| Bleak | 1240 | 1 | 3 | 3 | 25.43 | 21.74 | 21.17 | 41.27 | 62.86 | 57.14 | 48.90 | 35.73 | 25.02 | 48.87 | 52.53 | 32.25 | 64.57 | 60.61 | 15.15 |
| Bleak | 1241 | 1 | 3 | 3 | 23.03 | 21.55 | 21.70 | 37.13 | 58.93 | 51.44 | 43.48 | 34.31 | 26.47 | 45.18 | 50.62 | 31.42 | 62.47 | 58.85 | 14.47 |
| Bleak | 1242 | 1 | 3 | 3 | 28.29 | 24.78 | 25.06 | 48.42 | 71.44 | 63.07 | 56.87 | 43.10 | 31.81 | 54.91 | 59.82 | 36.85 | 72.01 | 66.06 | 14.17 |
| Bleak | 1243 | 1 | 3 | 3 | 25.37 | 22.89 | 22.15 | 49.14 | 68.43 | 57.65 | 53.36 | 39.97 | 34.64 | 50.07 | 53.66 | 36.11 | 68.42 | 63.98 | 10.97 |
| Bleak | 1244 | 1 | 3 | 3 | 26.17 | 23.36 | 24.13 | 46.73 | 61.63 | 53.27 | 54.12 | 38.38 | 23.30 | 52.31 | 57.42 | 37.24 | 70.56 | 67.48 | 14.47 |
| Bleak | 1245 | 1 | 3 | 3 | 23.99 | 20.38 | 20.64 | 40.70 | 63.07 | 57.99 | 47.20 | 35.26 | 23.84 | 41.60 | 43.97 | 32.31 | 58.69 | 54.57 | 11.15 |
| Bleak | 1246 | 1 | 3 | 3 | 23.48 | 21.78 | 22.47 | 39.56 | 59.60 | 51.41 | 47.40 | 36.27 | 29.24 | 51.57 | 54.93 | 33.43 | 65.17 | 57.72 | 13.38 |
| Bleak | 1247 | 1 | 3 | 3 | 22.60 | 21.58 | 22.68 | 40.64 | 57.83 | 51.05 | 48.15 | 33.76 | 23.81 | 43.82 | 49.60 | 33.16 | 63.99 | 60.94 | 12.45 |
| Bleak | 1248 | 1 | 3 | 3 | 26.65 | 26.07 | 23.18 | 41.91 | 58.15 | 48.00 | 45.77 | 35.04 | 27.15 | 50.20 | 53.43 | 33.08 | 62.25 | 56.97 | 11.82 |
| Bleak | 1249 | 1 | 3 | 3 | 16.05 | 14.78 | 14.77 | 27.44 | 41.77 | 38.01 | 32.91 | 24.21 | 17.62 | 33.98 | 35.74 | 20.62 | 40.91 | 36.98 | 7.63 |
| Bleak | 1250 | 1 | 3 | 3 | 19.17 | 18.02 | 17.57 | 32.72 | 49.75 | 44.16 | 38.62 | 26.78 | 20.97 | 38.02 | 43.01 | 26.10 | 52.05 | 49.73 | 11.77 |
| Bleak | 1250 | 1 | 3 | 3 | 21.42 | 19.83 | 19.70 | 36.96 | 53.51 | 48.46 | 43.81 | 31.19 | 25.11 | 40.43 | 44.76 | 29.26 | 53.76 | 51.69 | 12.36 |
| Bleak | 1031 | 2 | 1 | 1 | 19.28 | 16.42 | 16.43 | 30.05 | 45.56 | 39.86 | 34.33 | 26.17 | 20.50 | 35.49 | 39.08 | 24.32 | 46.82 | 41.94 | 11.74 |
| Bleak | 1032 | 2 | 1 | 1 | 22.50 | 20.25 | 20.23 | 38.72 | 56.87 | 51.56 | 45.33 | 31.13 | 24.95 | 42.73 | 46.37 | 28.90 | 56.41 | 54.41 | 11.91 |
| Bleak | 1033 | 2 | 1 | 1 | 24.19 | 21.77 | 22.60 | 40.05 | 59.86 | 53.57 | 46.10 | 34.41 | 26.13 | 45.55 | 49.99 | 31.87 | 58.99 | 54.81 | 15.33 |
| Bleak | 1034 | 2 | 1 | 1 | 15.85 | 16.85 | 15.91 | 29.23 | 42.40 | 37.38 | 34.16 | 25.24 | 19.58 | 34.60 | 37.78 | 23.49 | 45.73 | 44.68 | 11.26 |
| Bleak | 1035 | 2 | 1 | 1 | 12.56 | 13.96 | 12.84 | 23.22 | 35.70 | 30.14 | 27.25 | 19.39 | 15.32 | 27.11 | 30.23 | 18.47 | 38.10 | 34.92 | 8.23 |
| Bleak | 1036 | 2 | 1 | 1 | 20.25 | 19.95 | 19.67 | 35.95 | 52.42 | 44.92 | 40.83 | 32.28 | 23.92 | 44.19 | 47.43 | 29.71 | 59.56 | 53.72 | 13.00 |
| Bleak | 1037 | 2 | 1 | 1 | 20.01 | 17.37 | 16.80 | 33.74 | 48.19 | 44.54 | 38.61 | 27.12 | 17.74 | 39.75 | 42.61 | 25.36 | 49.65 | 45.46 | 10.35 |
| Bleak | 1038 | 2 | 1 | 1 | 21.22 | 18.97 | 17.22 | 29.78 | 45.67 | 42.13 | 39.25 | 27.77 | 20.10 | 36.46 | 39.75 | 27.56 | 48.45 | 46.50 | 10.23 |
| Bleak | 1039 | 2 | 1 | 1 | 17.06 | 16.25 | 15.18 | 26.67 | 40.41 | 36.41 | 33.14 | 23.92 | 19.97 | 30.62 | 32.02 | 23.04 | 41.76 | 40.40 | 10.33 |
| Bleak | 1040 | 2 | 1 | 1 | 22.57 | 18.99 | 20.01 | 39.68 | 59.93 | 57.37 | 49.95 | 35.12 | 22.11 | 42.18 | 49.29 | 33.78 | 57.30 | 59.09 | 14.76 |
| Bleak | 1041 | 2 | 1 | 1 | 13.57 | 13.60 | 13.31 | 25.34 | 38.21 | 32.55 | 27.52 | 22.10 | 15.56 | 29.68 | 31.90 | 18.42 | 35.52 | 34.05 | 7.33 |
| Bleak | 1042 | 2 | 1 | 1 | 16.84 | 17.14 | 16.70 | 31.43 | 45.91 | 39.77 | 38.18 | 26.56 | 18.51 | 39.25 | 40.64 | 26.77 | 52.05 | 47.86 | 13.30 |
| Bleak | 1043 | 2 | 1 | 1 | 17.37 | 16.80 | 16.20 | 34.48 | 50.60 | 45.38 | 39.03 | 29.90 | 22.03 | 41.63 | 44.51 | 26.58 | 50.80 | 47.73 | 12.34 |
| Bleak | 1044 | 2 | 1 | 1 | 15.81 | 15.33 | 15.10 | 28.68 | 42.93 | 36.62 | 31.30 | 20.74 | 17.10 | 33.84 | 37.41 | 19.89 | 41.68 | 39.86 | 10.68 |
| Bleak | 1045 | 2 | 1 | 1 | 17.73 | 16.19 | 15.81 | 27.59 | 43.07 | 38.06 | 34.70 | 24.81 | 18.92 | 35.30 | 39.95 | 24.17 | 45.52 | 42.69 | 10.06 |
| Bleak | 1046 | 2 | 1 | 1 | 20.27 | 18.78 | 17.83 | 37.32 | 55.60 | 47.19 | 40.53 | 29.72 | 23.11 | 42.86 | 46.28 | 27.10 | 52.63 | 47.12 | 13.15 |
| Bleak | 1047 | 2 | 1 | 1 | 22.21 | 19.55 | 18.58 | 38.14 | 52.96 | 47.63 | 45.13 | 27.40 | 19.20 | 45.94 | 46.50 | 29.36 | 56.16 | 49.32 | 13.44 |
| Bleak | 1048 | 2 | 1 | 1 | 17.37 | 17.18 | 16.80 | 31.01 | 45.56 | 39.63 | 34.53 | 23.66 | 20.21 | 33.15 | 37.75 | 25.58 | 47.92 | 45.46 | 10.21 |
| Bleak | 1049 | 2 | 1 | 1 | 21.49 | 19.09 | 22.01 | 41.35 | 58.98 | 54.51 | 48.81 | 34.21 | 26.25 | 44.96 | 50.21 | 32.39 | 55.57 | 52.48 | 16.10 |
| Bleak | 1050 | 2 | 1 | 1 | 14.92 | 15.16 | 14.86 | 26.86 | 41.66 | 36.26 | 31.62 | 21.11 | 16.72 | 36.60 | 38.33 | 19.31 | 42.90 | 41.25 | 10.69 |
| Bleak | 1051 | 2 | 1 | 1 | 18.61 | 16.33 | 17.30 | 36.46 | 51.99 | 45.75 | 41.32 | 29.21 | 23.43 | 41.01 | 44.62 | 25.66 | 50.95 | 49.53 | 13.27 |
| Bleak | 1052 | 2 | 1 | 1 | 19.27 | 17.65 | 18.59 | 40.14 | 57.84 | 51.17 | 45.13 | 32.62 | 24.57 | 42.72 | 47.12 | 28.61 | 54.63 | 52.21 | 12.74 |
| Bleak | 1053 | 2 | 1 | 1 | 22.42 | 18.94 | 19.20 | 34.46 | 48.49 | 43.79 | 41.39 | 29.38 | 22.73 | 39.59 | 44.16 | 27.26 | 52.15 | 48.75 | 10.85 |
| Bleak | 1054 | 2 | 1 | 1 | 15.49 | 14.42 | 13.81 | 29.09 | 39.19 | 34.56 | 31.78 | 21.57 | 15.47 | 32.75 | 36.70 | 20.98 | 39.35 | 38.31 | 9.53 |
| Bleak | 1055 | 2 | 1 | 1 | 19.11 | 15.35 | 16.27 | 26.96 | 43.21 | 41.40 | 36.00 | 26.93 | 20.03 | 34.86 | 41.28 | 24.24 | 47.14 | 43.75 | 10.39 |
| Bleak | 1056 | 2 | 1 | 1 | 13.03 | 12.48 | 12.62 | 22.28 | 30.78 | 27.25 | 25.67 | 18.03 | 15.90 | 25.63 | 28.59 | 17.72 | 34.64 | 33.74 | 9.79 |
| Bleak | 1057 | 2 | 1 | 1 | 14.37 | 14.58 | 14.22 | 24.64 | 36.84 | 32.73 | 28.40 | 20.86 | 16.26 | 31.60 | 31.91 | 19.91 | 36.89 | 33.41 | 9.25 |
| Bleak | 1058 | 2 | 1 | 1 | 16.26 | 17.55 | 17.29 | 31.78 | 47.49 | 41.92 | 36.10 | 26.79 | 23.71 | 30.91 | 34.71 | 25.94 | 43.00 | 39.28 | 10.05 |
| Bleak | 1059 | 2 | 1 | 1 | 16.44 | 13.11 | 13.32 | 29.70 | 44.02 | 42.28 | 34.62 | 20.22 | 22.60 | 28.82 | 34.17 | 22.27 | 41.87 | 41.46 | 11.07 |
| Bleak | 1060 | 2 | 1 | 1 | 17.02 | 14.97 | 15.75 | 36.42 | 46.72 | 40.75 | 37.10 | 26.76 | 19.21 | 36.49 | 38.74 | 26.08 | 45.05 | 43.60 | 9.61 |
| Bleak | 1031 | 2 | 1 | 2 | 18.67 | 17.32 | 17.43 | 27.71 | 44.63 | 38.08 | 34.12 | 26.44 | 20.46 | 33.82 | 36.90 | 25.86 | 47.12 | 44.12 | 11.02 |
| Bleak | 1032 | 2 | 1 | 2 | 20.67 | 19.64 | 19.95 | 37.83 | 55.49 | 51.41 | 45.96 | 29.89 | 24.07 | 41.61 | 46.96 | 29.47 | 57.02 | 54.16 | 12.16 |
| Bleak | 1033 | 2 | 1 | 2 | 21.53 | 21.12 | 21.81 | 39.52 | 59.84 | 53.01 | 44.61 | 35.77 | 27.92 | 46.43 | 50.98 | 31.91 | 59.86 | 55.21 | 15.42 |
| Bleak | 1034 | 2 | 1 | 2 | 19.27 | 17.47 | 14.90 | 28.38 | 43.27 | 37.87 | 32.84 | 25.50 | 21.22 | 34.40 | 38.12 | 23.21 | 44.73 | 41.09 | 10.82 |
| Bleak | 1035 | 2 | 1 | 2 | 13.02 | 14.59 | 13.83 | 21.71 | 35.76 | 30.70 | 27.12 | 20.46 | 15.08 | 28.11 | 30.91 | 18.70 | 36.57 | 35.54 | 8.50 |
| Bleak | 1036 | 2 | 1 | 2 | 20.31 | 20.04 | 20.04 | 34.73 | 55.00 | 48.41 | 41.44 | 32.10 | 22.89 | 44.33 | 47.41 | 30.62 | 58.11 | 52.81 | 13.30 |
| Bleak | 1037 | 2 | 1 | 2 | 17.65 | 16.39 | 18.06 | 32.50 | 47.68 | 43.77 | 39.56 | 25.93 | 18.42 | 37.19 | 42.22 | 25.06 | 48.37 | 44.16 | 11.65 |
| Bleak | 1038 | 2 | 1 | 2 | 18.21 | 17.09 | 17.86 | 31.79 | 47.30 | 43.64 | 38.56 | 27.26 | 20.99 | 36.00 | 40.34 | 26.84 | 48.40 | 48.21 | 10.85 |
| Bleak | 1039 | 2 | 1 | 2 | 16.08 | 15.94 | 14.53 | 28.37 | 40.99 | 35.50 | 31.75 | 23.39 | 20.64 | 30.29 | 32.94 | 22.07 | 41.32 | 39.78 | 10.65 |
| Bleak | 1040 | 2 | 1 | 2 | 24.52 | 18.99 | 20.32 | 39.68 | 59.12 | 55.06 | 48.96 | 35.64 | 22.68 | 44.49 | 51.25 | 32.31 | 57.43 | 58.15 | 13.28 |
| Bleak | 1041 | 2 | 1 | 2 | 14.39 | 15.04 | 12.34 | 24.85 | 38.37 | 34.30 | 29.67 | 22.55 | 15.51 | 27.58 | 31.31 | 19.15 | 36.12 | 34.97 | 8.60 |
| Bleak | 1042 | 2 | 1 | 2 | 17.67 | 17.08 | 15.86 | 34.41 | 46.57 | 39.94 | 37.16 | 26.12 | 17.36 | 39.91 | 41.83 | 25.67 | 50.96 | 47.36 | 10.45 |
| Bleak | 1043 | 2 | 1 | 2 | 17.37 | 17.69 | 16.65 | 34.84 | 50.99 | 46.07 | 40.64 | 29.78 | 20.39 | 42.70 | 46.18 | 27.14 | 51.14 | 47.89 | 12.62 |
| Bleak | 1044 | 2 | 1 | 2 | 16.55 | 14.53 | 14.28 | 28.83 | 42.05 | 38.41 | 33.08 | 21.41 | 16.95 | 33.89 | 36.80 | 19.74 | 41.66 | 39.10 | 9.01 |
| Bleak | 1045 | 2 | 1 | 2 | 17.73 | 16.51 | 17.01 | 28.54 | 42.84 | 37.07 | 34.12 | 25.62 | 17.54 | 38.47 | 42.34 | 23.69 | 49.29 | 45.54 | 9.92 |
| Bleak | 1046 | 2 | 1 | 2 | 22.38 | 19.12 | 18.25 | 36.28 | 54.41 | 49.43 | 41.49 | 30.34 | 23.89 | 43.94 | 47.08 | 27.19 | 54.73 | 48.48 | 12.86 |
| Bleak | 1047 | 2 | 1 | 2 | 21.65 | 19.04 | 18.23 | 39.89 | 50.27 | 46.67 | 47.77 | 27.28 | 19.62 | 42.13 | 45.39 | 29.52 | 55.60 | 51.43 | 12.56 |
| Bleak | 1048 | 2 | 1 | 2 | 17.60 | 16.60 | 16.00 | 28.21 | 45.70 | 41.64 | 35.63 | 25.87 | 20.65 | 33.03 | 36.68 | 24.46 | 49.21 | 45.82 | 12.01 |
| Bleak | 1049 | 2 | 1 | 2 | 21.68 | 20.28 | 21.70 | 42.48 | 60.67 | 52.69 | 51.16 | 34.81 | 25.28 | 42.12 | 46.65 | 32.95 | 60.02 | 53.76 | 14.83 |
| Bleak | 1050 | 2 | 1 | 2 | 14.93 | 15.83 | 14.52 | 27.22 | 41.08 | 35.22 | 30.26 | 22.26 | 19.50 | 33.90 | 37.77 | 20.75 | 43.13 | 40.31 | 9.40 |
| Bleak | 1051 | 2 | 1 | 2 | 16.47 | 17.39 | 18.74 | 38.81 | 53.56 | 47.57 | 43.00 | 27.36 | 23.16 | 41.55 | 44.51 | 25.53 | 53.29 | 45.92 | 11.44 |
| Bleak | 1052 | 2 | 1 | 2 | 19.12 | 17.65 | 18.61 | 40.16 | 58.37 | 50.45 | 44.02 | 34.35 | 25.50 | 41.21 | 46.94 | 29.42 | 56.97 | 55.39 | 11.93 |
| Bleak | 1053 | 2 | 1 | 2 | 20.86 | 20.39 | 20.32 | 36.62 | 54.48 | 47.16 | 41.25 | 29.92 | 22.18 | 44.30 | 45.90 | 26.71 | 51.05 | 50.72 | 13.21 |
| Bleak | 1054 | 2 | 1 | 2 | 15.40 | 13.82 | 13.60 | 29.48 | 40.95 | 34.86 | 32.14 | 22.96 | 17.44 | 31.16 | 34.37 | 23.21 | 44.18 | 41.79 | 9.91 |
| Bleak | 1055 | 2 | 1 | 2 | 20.07 | 15.63 | 16.20 | 28.71 | 45.96 | 42.13 | 36.16 | 27.36 | 21.05 | 36.34 | 40.13 | 23.34 | 46.68 | 44.77 | 11.27 |
| Bleak | 1056 | 2 | 1 | 2 | 15.87 | 14.47 | 12.48 | 23.32 | 31.87 | 28.54 | 26.09 | 19.79 | 15.00 | 27.39 | 29.79 | 18.42 | 35.59 | 34.95 | 8.26 |
| Bleak | 1057 | 2 | 1 | 2 | 16.31 | 13.89 | 14.80 | 24.32 | 35.52 | 31.16 | 28.64 | 20.81 | 14.88 | 32.58 | 31.12 | 18.62 | 37.26 | 32.74 | 11.05 |
| Bleak | 1058 | 2 | 1 | 2 | 19.89 | 18.86 | 16.35 | 31.92 | 47.90 | 42.22 | 38.21 | 28.11 | 21.31 | 33.59 | 36.92 | 25.54 | 39.34 | 37.09 | 11.91 |
| Bleak | 1059 | 2 | 1 | 2 | 17.39 | 15.47 | 15.26 | 31.71 | 47.20 | 41.66 | 35.44 | 23.48 | 18.67 | 33.22 | 36.30 | 21.23 | 41.58 | 41.93 | 11.33 |
| Bleak | 1060 | 2 | 1 | 2 | 18.52 | 16.37 | 16.24 | 27.69 | 43.36 | 39.84 | 38.05 | 25.90 | 17.61 | 33.79 | 40.45 | 24.78 | 45.33 | 44.13 | 10.66 |
| Bleak | 1031 | 2 | 1 | 3 | 20.56 | 17.28 | 19.20 | 26.06 | 45.28 | 39.25 | 34.56 | 24.82 | 18.47 | 34.50 | 38.63 | 24.59 | 45.73 | 43.41 | 10.83 |
| Bleak | 1032 | 2 | 1 | 3 | 21.29 | 20.91 | 21.34 | 38.81 | 55.50 | 48.68 | 44.66 | 31.32 | 25.12 | 41.98 | 44.91 | 30.87 | 57.69 | 56.77 | 12.22 |
| Bleak | 1033 | 2 | 1 | 3 | 21.67 | 21.81 | 22.20 | 38.48 | 58.90 | 54.26 | 47.39 | 34.62 | 27.77 | 44.67 | 51.15 | 33.10 | 59.77 | 55.81 | 14.87 |
| Bleak | 1034 | 2 | 1 | 3 | 18.78 | 17.14 | 15.90 | 27.00 | 44.35 | 39.21 | 33.31 | 24.82 | 20.05 | 38.04 | 40.47 | 22.78 | 45.50 | 42.46 | 10.21 |
| Bleak | 1035 | 2 | 1 | 3 | 13.89 | 14.25 | 12.90 | 21.62 | 34.48 | 29.30 | 25.51 | 19.67 | 17.23 | 25.19 | 28.81 | 19.41 | 37.57 | 34.87 | 8.47 |
| Bleak | 1036 | 2 | 1 | 3 | 19.70 | 20.46 | 19.15 | 34.96 | 54.37 | 45.87 | 40.29 | 31.48 | 22.86 | 44.96 | 46.09 | 30.38 | 59.07 | 53.29 | 14.46 |
| Bleak | 1037 | 2 | 1 | 3 | 17.32 | 16.52 | 16.21 | 31.74 | 47.32 | 44.70 | 40.03 | 27.41 | 18.67 | 36.86 | 43.01 | 25.05 | 47.87 | 45.54 | 11.90 |
| Bleak | 1038 | 2 | 1 | 3 | 19.70 | 17.17 | 19.13 | 29.60 | 47.88 | 42.49 | 38.39 | 27.32 | 19.27 | 37.02 | 40.90 | 26.27 | 45.61 | 44.36 | 10.25 |
| Bleak | 1039 | 2 | 1 | 3 | 15.37 | 14.49 | 15.10 | 27.73 | 41.66 | 37.20 | 33.16 | 23.38 | 18.42 | 31.03 | 34.44 | 22.50 | 42.66 | 38.22 | 11.18 |
| Bleak | 1040 | 2 | 1 | 3 | 23.04 | 20.73 | 20.59 | 40.04 | 59.07 | 53.77 | 47.26 | 36.33 | 23.59 | 45.33 | 51.71 | 33.86 | 58.45 | 58.91 | 14.47 |
| Bleak | 1041 | 2 | 1 | 3 | 14.54 | 14.77 | 13.73 | 25.65 | 37.41 | 30.62 | 28.32 | 20.07 | 15.26 | 27.12 | 31.72 | 19.16 | 36.50 | 35.04 | 8.73 |
| Bleak | 1042 | 2 | 1 | 3 | 16.12 | 17.41 | 17.15 | 35.41 | 47.90 | 40.03 | 37.93 | 26.23 | 18.16 | 40.79 | 40.64 | 24.76 | 50.25 | 47.02 | 10.82 |
| Bleak | 1043 | 2 | 1 | 3 | 17.52 | 16.57 | 18.16 | 33.65 | 50.85 | 43.83 | 38.61 | 29.20 | 22.35 | 39.82 | 44.90 | 27.47 | 51.76 | 45.98 | 12.14 |
| Bleak | 1044 | 2 | 1 | 3 | 17.25 | 15.54 | 14.38 | 31.99 | 40.84 | 34.81 | 31.15 | 22.41 | 17.56 | 33.88 | 37.70 | 19.51 | 41.57 | 38.68 | 9.57 |
| Bleak | 1045 | 2 | 1 | 3 | 18.48 | 16.37 | 17.97 | 27.63 | 40.90 | 36.02 | 35.51 | 26.58 | 17.51 | 38.13 | 41.85 | 24.81 | 46.54 | 43.69 | 10.77 |
| Bleak | 1046 | 2 | 1 | 3 | 18.25 | 18.65 | 19.16 | 35.59 | 53.82 | 47.37 | 41.57 | 28.48 | 22.68 | 45.20 | 45.80 | 26.14 | 50.77 | 46.21 | 12.12 |
| Bleak | 1047 | 2 | 1 | 3 | 22.64 | 19.30 | 18.21 | 42.34 | 53.76 | 45.80 | 45.66 | 28.63 | 19.41 | 44.09 | 47.48 | 28.80 | 52.42 | 49.25 | 11.46 |
| Bleak | 1048 | 2 | 1 | 3 | 17.40 | 17.65 | 16.54 | 32.70 | 45.44 | 40.03 | 36.77 | 24.53 | 19.37 | 31.57 | 37.44 | 27.17 | 48.54 | 46.72 | 10.95 |
| Bleak | 1049 | 2 | 1 | 3 | 21.72 | 21.00 | 20.65 | 39.38 | 61.25 | 55.34 | 46.84 | 34.80 | 26.24 | 43.91 | 49.47 | 30.67 | 55.27 | 54.47 | 15.01 |
| Bleak | 1050 | 2 | 1 | 3 | 15.62 | 16.01 | 14.43 | 27.34 | 41.58 | 36.70 | 31.60 | 22.44 | 18.11 | 34.52 | 38.32 | 21.16 | 45.58 | 40.10 | 9.26 |
| Bleak | 1051 | 2 | 1 | 3 | 19.95 | 16.21 | 17.30 | 38.34 | 52.31 | 47.21 | 42.69 | 27.30 | 22.73 | 39.30 | 42.16 | 23.33 | 47.51 | 46.42 | 12.64 |
| Bleak | 1052 | 2 | 1 | 3 | 18.87 | 17.37 | 18.61 | 38.63 | 57.63 | 51.17 | 43.94 | 32.21 | 26.08 | 44.52 | 50.01 | 29.04 | 55.72 | 54.06 | 13.00 |
| Bleak | 1053 | 2 | 1 | 3 | 21.34 | 19.65 | 18.60 | 33.32 | 47.86 | 42.57 | 40.57 | 30.37 | 20.48 | 40.81 | 48.96 | 27.17 | 52.76 | 47.29 | 12.44 |
| Bleak | 1054 | 2 | 1 | 3 | 14.19 | 14.49 | 15.01 | 27.23 | 39.60 | 32.93 | 33.32 | 22.18 | 14.06 | 33.30 | 38.92 | 22.24 | 42.80 | 41.19 | 10.74 |
| Bleak | 1055 | 2 | 1 | 3 | 20.43 | 16.47 | 16.36 | 29.05 | 45.02 | 42.56 | 36.62 | 25.66 | 19.38 | 35.52 | 41.26 | 24.68 | 49.39 | 45.86 | 9.01 |
| Bleak | 1056 | 2 | 1 | 3 | 14.04 | 12.93 | 13.02 | 23.76 | 33.99 | 29.90 | 26.36 | 16.97 | 14.82 | 28.60 | 30.77 | 20.60 | 39.29 | 38.64 | 9.31 |
| Bleak | 1057 | 2 | 1 | 3 | 14.23 | 12.82 | 13.39 | 24.64 | 34.23 | 29.69 | 28.38 | 19.10 | 14.75 | 31.27 | 33.15 | 19.92 | 35.49 | 31.10 | 9.42 |
| Bleak | 1058 | 2 | 1 | 3 | 17.57 | 17.17 | 15.72 | 34.29 | 48.97 | 42.10 | 38.87 | 27.59 | 18.99 | 33.32 | 34.82 | 26.85 | 45.16 | 38.85 | 10.19 |
| Bleak | 1059 | 2 | 1 | 3 | 16.04 | 14.60 | 14.97 | 31.61 | 47.45 | 42.44 | 37.35 | 22.35 | 17.32 | 31.30 | 36.41 | 21.35 | 42.14 | 40.34 | 8.98 |
| Bleak | 1060 | 2 | 1 | 3 | 16.81 | 14.63 | 16.73 | 31.26 | 46.64 | 43.31 | 38.31 | 25.35 | 18.55 | 39.24 | 38.53 | 24.69 | 47.37 | 43.97 | 10.50 |
| Bleak | 1131 | 2 | 2 | 1 | 28.30 | 29.67 | 26.99 | 50.76 | 82.66 | 76.70 | 60.26 | 46.97 | 37.77 | 54.70 | 58.90 | 41.73 | 78.39 | 73.66 | 18.80 |
| Bleak | 1132 | 2 | 2 | 1 | 25.75 | 26.71 | 24.88 | 41.63 | 56.39 | 48.86 | 50.02 | 34.39 | 23.58 | 49.92 | 53.09 | 34.04 | 62.36 | 58.06 | 14.89 |
| Bleak | 1133 | 2 | 2 | 1 | 22.38 | 21.45 | 21.95 | 43.59 | 61.98 | 56.57 | 52.25 | 35.91 | 28.40 | 49.08 | 54.49 | 33.43 | 64.28 | 60.97 | 15.90 |
| Bleak | 1134 | 2 | 2 | 1 | 20.21 | 19.14 | 19.67 | 43.45 | 55.52 | 44.71 | 43.09 | 28.94 | 24.02 | 40.13 | 46.88 | 31.76 | 53.51 | 51.99 | 13.55 |
| Bleak | 1135 | 2 | 2 | 1 | 23.76 | 23.72 | 22.46 | 45.70 | 60.73 | 49.85 | 49.49 | 31.89 | 24.43 | 47.78 | 53.47 | 34.26 | 67.00 | 62.50 | 13.01 |
| Bleak | 1136 | 2 | 2 | 1 | 20.80 | 20.69 | 21.09 | 40.25 | 58.55 | 50.28 | 46.50 | 33.32 | 21.94 | 44.10 | 48.92 | 29.23 | 55.57 | 51.19 | 13.72 |
| Bleak | 1137 | 2 | 2 | 1 | 24.36 | 24.27 | 22.09 | 47.39 | 69.42 | 59.49 | 50.37 | 38.79 | 31.68 | 49.44 | 54.04 | 35.69 | 66.35 | 64.39 | 16.58 |
| Bleak | 1138 | 2 | 2 | 1 | 26.51 | 23.41 | 23.26 | 49.14 | 68.00 | 61.68 | 54.83 | 35.33 | 29.46 | 51.93 | 56.91 | 30.01 | 60.50 | 57.43 | 16.87 |
| Bleak | 1139 | 2 | 2 | 1 | 31.20 | 26.56 | 28.52 | 63.58 | 87.88 | 76.20 | 70.19 | 52.13 | 41.84 | 63.08 | 68.34 | 49.60 | 87.32 | 76.25 | 17.12 |
| Bleak | 1140 | 2 | 2 | 1 | 25.24 | 25.98 | 25.07 | 48.13 | 68.70 | 58.03 | 51.67 | 36.83 | 27.19 | 49.73 | 55.06 | 32.23 | 63.11 | 63.04 | 18.05 |
| Bleak | 1141 | 2 | 2 | 1 | 22.22 | 20.32 | 16.80 | 28.72 | 45.38 | 41.26 | 37.63 | 26.42 | 20.41 | 34.07 | 38.03 | 28.48 | 47.41 | 45.15 | 9.31 |
| Bleak | 1142 | 2 | 2 | 1 | 30.73 | 26.73 | 27.59 | 55.67 | 78.42 | 68.74 | 60.49 | 44.91 | 37.84 | 52.30 | 66.11 | 40.08 | 76.22 | 76.19 | 18.54 |
| Bleak | 1143 | 2 | 2 | 1 | 22.05 | 23.70 | 21.74 | 41.45 | 61.32 | 53.38 | 48.41 | 34.88 | 22.86 | 51.58 | 54.69 | 32.83 | 62.18 | 60.48 | 14.62 |
| Bleak | 1144 | 2 | 2 | 1 | 26.02 | 25.43 | 24.01 | 51.43 | 70.44 | 62.95 | 55.87 | 35.79 | 28.60 | 45.94 | 50.72 | 36.08 | 63.98 | 59.86 | 15.41 |
| Bleak | 1145 | 2 | 2 | 1 | 24.90 | 20.94 | 21.74 | 35.86 | 50.93 | 46.12 | 43.13 | 30.72 | 23.56 | 37.78 | 48.31 | 32.12 | 62.43 | 58.06 | 14.73 |
| Bleak | 1146 | 2 | 2 | 1 | 24.42 | 25.81 | 28.73 | 52.02 | 77.35 | 67.54 | 63.91 | 45.41 | 34.61 | 60.19 | 62.65 | 42.74 | 77.07 | 65.12 | 18.73 |
| Bleak | 1147 | 2 | 2 | 1 | 32.82 | 28.77 | 28.22 | 52.38 | 73.14 | 61.25 | 62.41 | 41.66 | 37.73 | 52.50 | 62.99 | 41.47 | 74.88 | 72.01 | 16.77 |
| Bleak | 1148 | 2 | 2 | 1 | 30.05 | 27.03 | 22.84 | 55.75 | 77.43 | 67.77 | 61.52 | 41.47 | 30.06 | 57.46 | 66.62 | 40.45 | 76.59 | 76.55 | 14.80 |
| Bleak | 1149 | 2 | 2 | 1 | 32.27 | 25.36 | 27.30 | 53.08 | 76.59 | 66.61 | 62.44 | 47.36 | 38.27 | 56.84 | 65.90 | 39.68 | 75.91 | 74.88 | 18.76 |
| Bleak | 1150 | 2 | 2 | 1 | 23.02 | 21.92 | 22.40 | 44.02 | 65.07 | 57.96 | 51.80 | 37.26 | 28.23 | 45.59 | 55.02 | 34.67 | 62.59 | 61.20 | 13.62 |
| Bleak | 1151 | 2 | 2 | 1 | 22.56 | 21.82 | 22.60 | 42.83 | 59.69 | 51.08 | 50.46 | 35.32 | 23.46 | 46.53 | 51.18 | 34.82 | 64.07 | 61.25 | 15.69 |
| Bleak | 1152 | 2 | 2 | 1 | 25.15 | 25.05 | 23.82 | 53.27 | 75.70 | 64.41 | 58.99 | 42.65 | 32.36 | 61.30 | 62.90 | 36.45 | 68.25 | 67.08 | 16.70 |
| Bleak | 1153 | 2 | 2 | 1 | 26.70 | 26.88 | 26.19 | 55.53 | 79.41 | 69.15 | 62.55 | 42.96 | 30.24 | 58.78 | 63.47 | 41.33 | 77.99 | 74.55 | 19.26 |
| Bleak | 1154 | 2 | 2 | 1 | 22.90 | 21.75 | 21.65 | 39.31 | 57.97 | 50.09 | 47.83 | 34.91 | 21.14 | 46.10 | 49.96 | 32.84 | 60.64 | 54.93 | 14.71 |
| Bleak | 1155 | 2 | 2 | 1 | 28.37 | 30.47 | 28.66 | 45.35 | 75.13 | 70.60 | 62.68 | 46.88 | 29.18 | 59.15 | 66.20 | 39.48 | 79.62 | 74.69 | 15.35 |
| Bleak | 1156 | 2 | 2 | 1 | 25.49 | 25.73 | 24.42 | 49.28 | 70.52 | 60.73 | 53.96 | 39.11 | 28.78 | 58.03 | 57.21 | 40.79 | 77.90 | 68.02 | 19.70 |
| Bleak | 1157 | 2 | 2 | 1 | 27.72 | 24.80 | 22.44 | 53.09 | 78.21 | 70.41 | 63.79 | 44.73 | 36.41 | 53.24 | 61.26 | 37.78 | 66.06 | 64.29 | 19.05 |
| Bleak | 1158 | 2 | 2 | 1 | 27.13 | 29.57 | 25.49 | 43.47 | 64.16 | 59.25 | 53.62 | 37.32 | 27.99 | 55.79 | 59.89 | 36.92 | 70.87 | 65.80 | 16.46 |
| Bleak | 1159 | 2 | 2 | 1 | 18.82 | 18.42 | 20.13 | 39.10 | 55.44 | 45.65 | 45.60 | 34.06 | 24.21 | 44.60 | 50.29 | 28.75 | 53.17 | 50.13 | 12.40 |
| Bleak | 1160 | 2 | 2 | 1 | 24.09 | 21.93 | 23.22 | 48.27 | 66.04 | 57.21 | 53.45 | 36.27 | 30.15 | 55.64 | 59.57 | 32.44 | 63.96 | 62.66 | 15.90 |
| Bleak | 1131 | 2 | 2 | 2 | 27.80 | 27.64 | 23.89 | 52.19 | 79.31 | 68.96 | 61.70 | 44.34 | 35.22 | 54.56 | 62.57 | 41.77 | 76.00 | 73.75 | 16.72 |
| Bleak | 1132 | 2 | 2 | 2 | 22.89 | 22.44 | 21.77 | 39.68 | 59.92 | 53.29 | 49.35 | 33.63 | 24.34 | 49.48 | 54.61 | 34.42 | 63.13 | 59.12 | 14.83 |
| Bleak | 1133 | 2 | 2 | 2 | 22.17 | 20.02 | 22.96 | 43.77 | 63.32 | 55.10 | 47.78 | 36.90 | 28.07 | 48.34 | 52.44 | 32.51 | 63.10 | 60.57 | 15.03 |
| Bleak | 1134 | 2 | 2 | 2 | 22.71 | 20.13 | 19.48 | 41.11 | 54.82 | 47.71 | 46.50 | 34.74 | 23.50 | 42.57 | 46.86 | 29.81 | 54.39 | 52.78 | 13.03 |
| Bleak | 1135 | 2 | 2 | 2 | 24.33 | 23.15 | 21.93 | 46.24 | 58.84 | 49.61 | 50.58 | 33.15 | 25.18 | 49.00 | 52.62 | 32.99 | 63.05 | 56.92 | 14.62 |
| Bleak | 1136 | 2 | 2 | 2 | 21.64 | 20.21 | 22.78 | 39.04 | 58.03 | 49.90 | 43.24 | 31.97 | 22.98 | 46.21 | 48.78 | 28.62 | 54.18 | 52.59 | 14.42 |
| Bleak | 1137 | 2 | 2 | 2 | 24.70 | 24.15 | 23.02 | 44.69 | 67.09 | 60.04 | 52.43 | 39.82 | 30.55 | 53.42 | 56.38 | 36.51 | 64.99 | 61.48 | 18.47 |
| Bleak | 1138 | 2 | 2 | 2 | 25.40 | 24.48 | 22.63 | 43.60 | 67.97 | 64.60 | 55.82 | 35.18 | 29.65 | 48.78 | 55.10 | 33.43 | 59.38 | 58.46 | 13.28 |
| Bleak | 1139 | 2 | 2 | 2 | 29.92 | 27.89 | 28.53 | 64.05 | 86.25 | 74.99 | 74.22 | 53.90 | 38.88 | 60.89 | 66.04 | 47.49 | 81.36 | 77.25 | 16.67 |
| Bleak | 1140 | 2 | 2 | 2 | 24.36 | 25.59 | 21.83 | 48.22 | 69.51 | 59.62 | 54.31 | 35.09 | 24.17 | 50.72 | 54.90 | 32.08 | 62.56 | 59.42 | 16.52 |
| Bleak | 1141 | 2 | 2 | 2 | 18.40 | 21.28 | 17.86 | 33.07 | 48.05 | 41.50 | 38.61 | 28.56 | 17.33 | 36.47 | 41.63 | 26.28 | 47.81 | 44.98 | 11.56 |
| Bleak | 1142 | 2 | 2 | 2 | 25.78 | 23.53 | 26.63 | 52.65 | 74.76 | 66.77 | 61.01 | 42.81 | 37.00 | 53.39 | 66.53 | 39.45 | 72.13 | 76.29 | 20.20 |
| Bleak | 1143 | 2 | 2 | 2 | 23.47 | 21.22 | 22.23 | 40.63 | 61.83 | 51.74 | 47.04 | 33.91 | 22.83 | 52.65 | 54.92 | 32.58 | 64.54 | 63.53 | 14.95 |
| Bleak | 1144 | 2 | 2 | 2 | 24.15 | 24.17 | 23.19 | 47.98 | 69.16 | 62.07 | 57.03 | 39.77 | 27.49 | 48.58 | 53.95 | 36.11 | 68.09 | 64.85 | 16.62 |
| Bleak | 1145 | 2 | 2 | 2 | 19.97 | 20.16 | 21.32 | 37.07 | 53.50 | 48.64 | 44.00 | 33.16 | 26.13 | 47.02 | 51.08 | 31.98 | 61.29 | 60.07 | 13.72 |
| Bleak | 1146 | 2 | 2 | 2 | 29.07 | 24.90 | 26.65 | 43.23 | 72.88 | 63.47 | 59.05 | 42.71 | 34.27 | 66.51 | 65.08 | 38.28 | 73.50 | 68.41 | 17.08 |
| Bleak | 1147 | 2 | 2 | 2 | 28.86 | 26.38 | 29.10 | 53.72 | 75.29 | 65.22 | 62.47 | 44.04 | 35.58 | 58.54 | 64.12 | 40.34 | 78.82 | 74.11 | 15.08 |
| Bleak | 1148 | 2 | 2 | 2 | 33.01 | 26.08 | 27.87 | 50.88 | 73.53 | 66.11 | 62.10 | 46.11 | 31.91 | 53.30 | 64.64 | 42.53 | 78.78 | 79.45 | 15.25 |
| Bleak | 1149 | 2 | 2 | 2 | 27.58 | 23.78 | 24.54 | 48.81 | 76.38 | 67.21 | 59.26 | 46.84 | 41.86 | 55.38 | 64.99 | 41.37 | 77.90 | 75.05 | 18.16 |
| Bleak | 1150 | 2 | 2 | 2 | 23.52 | 22.54 | 25.03 | 44.72 | 67.66 | 60.11 | 52.67 | 40.82 | 29.03 | 44.74 | 52.78 | 38.12 | 66.41 | 64.81 | 15.41 |
| Bleak | 1151 | 2 | 2 | 2 | 23.27 | 25.96 | 25.36 | 40.85 | 59.25 | 51.92 | 50.19 | 35.32 | 23.14 | 49.17 | 54.13 | 34.36 | 69.67 | 63.14 | 15.53 |
| Bleak | 1152 | 2 | 2 | 2 | 26.07 | 28.27 | 25.55 | 53.04 | 75.94 | 64.55 | 60.00 | 41.03 | 31.97 | 59.79 | 66.04 | 39.09 | 77.87 | 73.86 | 17.00 |
| Bleak | 1153 | 2 | 2 | 2 | 29.43 | 29.46 | 29.10 | 56.88 | 84.06 | 73.16 | 60.11 | 44.14 | 32.26 | 58.74 | 65.48 | 42.19 | 81.13 | 80.58 | 16.24 |
| Bleak | 1154 | 2 | 2 | 2 | 23.47 | 20.66 | 22.05 | 39.90 | 59.28 | 51.32 | 47.47 | 36.82 | 22.39 | 46.88 | 51.03 | 31.93 | 58.69 | 54.08 | 13.86 |
| Bleak | 1155 | 2 | 2 | 2 | 30.42 | 28.60 | 27.86 | 51.02 | 76.84 | 73.55 | 62.80 | 43.39 | 33.35 | 57.12 | 63.71 | 40.71 | 76.18 | 77.76 | 21.15 |
| Bleak | 1156 | 2 | 2 | 2 | 26.90 | 28.68 | 26.46 | 49.10 | 69.27 | 59.55 | 56.36 | 38.59 | 27.13 | 54.48 | 58.63 | 38.22 | 70.10 | 67.27 | 17.87 |
| Bleak | 1157 | 2 | 2 | 2 | 29.14 | 25.16 | 25.50 | 51.30 | 79.32 | 69.62 | 59.58 | 44.83 | 34.73 | 55.11 | 67.47 | 38.65 | 64.34 | 62.84 | 15.38 |
| Bleak | 1158 | 2 | 2 | 2 | 26.00 | 25.43 | 24.36 | 47.93 | 70.31 | 56.01 | 51.98 | 37.35 | 25.30 | 57.28 | 62.54 | 37.29 | 70.12 | 61.70 | 17.35 |
| Bleak | 1159 | 2 | 2 | 2 | 20.49 | 19.02 | 20.72 | 39.30 | 54.71 | 44.07 | 44.00 | 35.17 | 24.80 | 46.84 | 50.20 | 29.67 | 53.51 | 50.81 | 12.61 |
| Bleak | 1160 | 2 | 2 | 2 | 25.48 | 23.74 | 22.29 | 51.00 | 70.54 | 60.01 | 52.14 | 38.31 | 34.91 | 52.07 | 56.33 | 33.84 | 63.89 | 61.31 | 16.38 |
| Bleak | 1131 | 2 | 2 | 3 | 27.35 | 28.45 | 28.00 | 51.43 | 80.19 | 71.60 | 57.91 | 44.82 | 37.62 | 57.59 | 63.44 | 38.98 | 78.60 | 74.40 | 17.80 |
| Bleak | 1132 | 2 | 2 | 3 | 23.23 | 20.80 | 22.35 | 43.36 | 60.02 | 52.83 | 50.97 | 35.74 | 23.78 | 49.15 | 53.38 | 33.36 | 64.48 | 59.45 | 14.56 |
| Bleak | 1133 | 2 | 2 | 3 | 22.60 | 21.41 | 21.92 | 44.39 | 60.73 | 54.32 | 51.50 | 37.58 | 30.30 | 47.35 | 52.16 | 32.54 | 62.62 | 59.68 | 13.68 |
| Bleak | 1134 | 2 | 2 | 3 | 19.45 | 18.34 | 19.46 | 39.32 | 54.16 | 45.91 | 45.33 | 30.65 | 19.14 | 44.83 | 50.48 | 28.66 | 55.60 | 51.11 | 11.74 |
| Bleak | 1135 | 2 | 2 | 3 | 22.98 | 22.61 | 23.98 | 46.80 | 62.66 | 49.64 | 49.64 | 33.04 | 23.97 | 48.08 | 52.77 | 32.24 | 59.44 | 57.73 | 11.84 |
| Bleak | 1136 | 2 | 2 | 3 | 21.74 | 21.95 | 21.00 | 40.66 | 56.11 | 46.34 | 44.47 | 23.33 | 24.76 | 42.18 | 48.77 | 29.53 | 56.45 | 55.13 | 13.29 |
| Bleak | 1137 | 2 | 2 | 3 | 27.14 | 26.24 | 23.85 | 44.95 | 68.16 | 59.26 | 50.87 | 38.64 | 29.37 | 53.04 | 56.27 | 33.69 | 66.42 | 63.02 | 16.35 |
| Bleak | 1138 | 2 | 2 | 3 | 25.51 | 23.55 | 23.37 | 47.04 | 69.34 | 63.33 | 54.99 | 36.69 | 27.50 | 50.16 | 54.89 | 31.60 | 64.20 | 62.41 | 14.62 |
| Bleak | 1139 | 2 | 2 | 3 | 31.27 | 26.85 | 30.96 | 62.92 | 89.09 | 77.32 | 72.75 | 52.41 | 42.22 | 63.59 | 65.02 | 47.23 | 78.19 | 73.29 | 16.95 |
| Bleak | 1140 | 2 | 2 | 3 | 22.31 | 26.30 | 24.40 | 44.83 | 66.04 | 58.02 | 52.11 | 34.25 | 26.65 | 48.38 | 56.53 | 33.28 | 65.49 | 61.83 | 15.62 |
| Bleak | 1141 | 2 | 2 | 3 | 20.77 | 21.35 | 17.10 | 33.83 | 47.65 | 41.55 | 37.26 | 28.27 | 17.42 | 39.70 | 42.53 | 26.10 | 45.17 | 41.90 | 11.17 |
| Bleak | 1142 | 2 | 2 | 3 | 28.02 | 25.67 | 27.99 | 53.04 | 75.08 | 68.18 | 62.01 | 45.76 | 36.15 | 53.52 | 66.20 | 39.12 | 77.76 | 73.98 | 18.95 |
| Bleak | 1143 | 2 | 2 | 3 | 21.81 | 21.43 | 22.20 | 40.61 | 58.54 | 48.61 | 48.47 | 34.01 | 22.32 | 55.48 | 55.18 | 32.73 | 65.56 | 60.52 | 13.10 |
| Bleak | 1144 | 2 | 2 | 3 | 25.22 | 24.32 | 22.21 | 48.11 | 67.55 | 60.18 | 56.55 | 36.69 | 26.42 | 47.55 | 52.54 | 35.42 | 63.10 | 58.38 | 14.96 |
| Bleak | 1145 | 2 | 2 | 3 | 21.85 | 18.10 | 22.47 | 43.01 | 55.37 | 49.60 | 48.84 | 31.53 | 20.72 | 41.27 | 50.27 | 33.63 | 62.31 | 62.49 | 15.20 |
| Bleak | 1146 | 2 | 2 | 3 | 28.99 | 25.76 | 24.32 | 55.89 | 77.22 | 68.52 | 61.71 | 46.31 | 37.97 | 62.34 | 64.28 | 42.56 | 79.63 | 69.31 | 18.60 |
| Bleak | 1147 | 2 | 2 | 3 | 32.47 | 30.05 | 27.24 | 56.60 | 76.63 | 67.00 | 61.21 | 44.35 | 35.25 | 57.78 | 65.97 | 39.52 | 80.74 | 75.91 | 15.93 |
| Bleak | 1148 | 2 | 2 | 3 | 24.49 | 22.78 | 23.62 | 54.26 | 79.73 | 67.77 | 61.70 | 44.95 | 32.65 | 57.61 | 61.96 | 44.18 | 77.95 | 75.73 | 13.65 |
| Bleak | 1149 | 2 | 2 | 3 | 30.34 | 25.77 | 28.48 | 54.00 | 79.47 | 72.31 | 64.63 | 45.22 | 41.50 | 56.20 | 62.42 | 40.07 | 75.34 | 71.68 | 16.87 |
| Bleak | 1150 | 2 | 2 | 3 | 23.24 | 23.44 | 22.61 | 42.85 | 65.79 | 57.82 | 52.43 | 39.60 | 29.23 | 45.78 | 53.94 | 35.61 | 65.83 | 60.76 | 15.61 |
| Bleak | 1151 | 2 | 2 | 3 | 22.75 | 24.56 | 23.49 | 43.02 | 58.20 | 50.02 | 47.96 | 32.96 | 24.07 | 46.04 | 51.53 | 35.11 | 68.22 | 66.12 | 15.61 |
| Bleak | 1152 | 2 | 2 | 3 | 22.35 | 27.66 | 26.40 | 52.73 | 70.21 | 58.13 | 56.20 | 42.95 | 34.02 | 59.43 | 64.80 | 38.67 | 69.43 | 67.65 | 17.23 |
| Bleak | 1153 | 2 | 2 | 3 | 24.16 | 28.30 | 27.04 | 59.45 | 80.73 | 70.10 | 62.60 | 41.84 | 29.15 | 55.89 | 62.80 | 39.73 | 75.34 | 77.69 | 20.67 |
| Bleak | 1154 | 2 | 2 | 3 | 21.22 | 20.64 | 22.54 | 37.79 | 60.38 | 52.83 | 45.19 | 35.73 | 22.99 | 46.08 | 52.51 | 33.35 | 56.55 | 54.45 | 14.32 |
| Bleak | 1155 | 2 | 2 | 3 | 31.56 | 33.74 | 30.30 | 48.22 | 79.74 | 72.72 | 59.88 | 46.00 | 31.58 | 61.53 | 65.58 | 40.03 | 78.41 | 76.43 | 20.27 |
| Bleak | 1156 | 2 | 2 | 3 | 24.56 | 28.58 | 25.50 | 46.33 | 69.76 | 57.39 | 53.65 | 38.91 | 28.94 | 55.70 | 61.28 | 38.02 | 70.18 | 67.86 | 17.07 |
| Bleak | 1157 | 2 | 2 | 3 | 25.46 | 22.56 | 23.77 | 48.41 | 78.18 | 70.61 | 61.13 | 46.79 | 37.46 | 59.36 | 64.58 | 37.03 | 66.61 | 63.01 | 18.79 |
| Bleak | 1158 | 2 | 2 | 3 | 24.77 | 27.58 | 25.01 | 44.09 | 63.82 | 53.08 | 52.04 | 35.27 | 25.25 | 60.73 | 62.04 | 36.59 | 74.83 | 64.47 | 17.90 |
| Bleak | 1159 | 2 | 2 | 3 | 21.21 | 19.22 | 19.61 | 38.22 | 52.56 | 42.64 | 44.27 | 34.66 | 25.82 | 44.36 | 48.82 | 30.08 | 55.12 | 50.65 | 13.16 |
| Bleak | 1160 | 2 | 2 | 3 | 25.77 | 23.97 | 25.71 | 52.83 | 69.64 | 57.02 | 55.52 | 37.93 | 31.37 | 52.09 | 55.01 | 34.41 | 62.55 | 62.21 | 13.15 |
| Bleak | 1221 | 2 | 3 | 1 | 28.94 | 25.49 | 26.54 | 48.41 | 69.34 | 56.42 | 53.24 | 41.16 | 30.38 | 51.69 | 54.82 | 34.81 | 68.68 | 63.96 | 16.75 |
| Bleak | 1222 | 2 | 3 | 1 | 21.53 | 22.15 | 17.76 | 40.09 | 57.44 | 51.29 | 43.85 | 29.21 | 24.17 | 41.71 | 44.68 | 27.11 | 56.24 | 55.20 | 12.47 |
| Bleak | 1223 | 2 | 3 | 1 | 20.68 | 19.53 | 20.14 | 36.81 | 57.57 | 53.91 | 46.11 | 30.55 | 21.61 | 43.97 | 47.95 | 28.42 | 59.65 | 56.69 | 11.93 |
| Bleak | 1224 | 2 | 3 | 1 | 29.28 | 23.08 | 25.57 | 42.98 | 69.10 | 61.65 | 52.15 | 36.05 | 28.81 | 54.94 | 61.49 | 36.17 | 66.81 | 60.41 | 17.00 |
| Bleak | 1225 | 2 | 3 | 1 | 27.87 | 26.10 | 22.32 | 46.93 | 62.96 | 53.84 | 51.98 | 37.01 | 33.19 | 52.06 | 55.27 | 33.29 | 67.58 | 64.64 | 12.76 |
| Bleak | 1226 | 2 | 3 | 1 | 18.40 | 20.56 | 19.75 | 33.65 | 51.81 | 44.71 | 38.45 | 28.53 | 21.90 | 40.04 | 44.54 | 25.92 | 50.01 | 47.56 | 12.68 |
| Bleak | 1227 | 2 | 3 | 1 | 23.70 | 19.77 | 19.22 | 38.57 | 58.18 | 49.20 | 42.96 | 33.34 | 28.43 | 41.99 | 47.72 | 30.48 | 52.43 | 49.09 | 13.36 |
| Bleak | 1228 | 2 | 3 | 1 | 24.30 | 23.22 | 21.60 | 35.58 | 60.63 | 55.24 | 44.90 | 32.98 | 24.13 | 45.46 | 46.28 | 28.92 | 62.20 | 59.20 | 14.38 |
| Bleak | 1229 | 2 | 3 | 1 | 23.32 | 20.96 | 20.78 | 39.05 | 55.55 | 46.72 | 45.17 | 33.27 | 22.81 | 42.91 | 49.74 | 28.47 | 56.04 | 55.99 | 13.80 |
| Bleak | 1230 | 2 | 3 | 1 | 30.02 | 26.20 | 23.20 | 40.76 | 64.61 | 58.87 | 50.82 | 36.22 | 26.62 | 51.72 | 56.57 | 35.81 | 60.34 | 59.99 | 15.21 |
| Bleak | 1232 | 2 | 3 | 1 | 21.61 | 21.87 | 21.25 | 40.33 | 56.51 | 50.25 | 47.83 | 31.88 | 19.78 | 43.33 | 47.70 | 29.13 | 53.09 | 52.22 | 12.15 |
| Bleak | 1233 | 2 | 3 | 1 | 31.80 | 31.67 | 29.00 | 55.67 | 92.32 | 83.20 | 66.10 | 50.67 | 39.77 | 60.83 | 67.05 | 44.50 | 80.25 | 78.00 | 16.61 |
| Bleak | 1234 | 2 | 3 | 1 | 25.14 | 23.25 | 20.27 | 41.70 | 65.82 | 58.96 | 47.93 | 38.25 | 27.08 | 46.32 | 53.34 | 33.15 | 57.75 | 55.62 | 15.28 |
| Bleak | 1235 | 2 | 3 | 1 | 26.10 | 22.89 | 23.84 | 45.60 | 63.63 | 51.67 | 50.37 | 36.01 | 28.04 | 50.39 | 54.16 | 32.94 | 65.24 | 62.76 | 15.31 |
| Bleak | 1236 | 2 | 3 | 1 | 20.42 | 21.39 | 20.23 | 40.22 | 62.53 | 55.71 | 47.99 | 33.52 | 25.18 | 45.24 | 51.89 | 34.10 | 64.41 | 62.97 | 11.15 |
| Bleak | 1237 | 2 | 3 | 1 | 21.92 | 22.82 | 22.08 | 36.91 | 60.01 | 51.55 | 44.43 | 32.14 | 22.54 | 38.28 | 47.51 | 29.46 | 53.05 | 51.59 | 11.12 |
| Bleak | 1238 | 2 | 3 | 1 | 23.80 | 21.88 | 21.24 | 44.44 | 68.17 | 61.79 | 50.08 | 38.30 | 28.38 | 48.37 | 53.15 | 34.37 | 62.67 | 58.76 | 12.03 |
| Bleak | 1239 | 2 | 3 | 1 | 25.04 | 23.11 | 23.33 | 44.56 | 67.73 | 61.28 | 53.81 | 39.09 | 29.45 | 45.77 | 52.79 | 34.77 | 60.56 | 59.06 | 12.91 |
| Bleak | 1240 | 2 | 3 | 1 | 24.30 | 21.96 | 19.29 | 40.20 | 64.93 | 58.39 | 47.05 | 34.99 | 26.61 | 48.43 | 49.09 | 30.76 | 59.77 | 57.68 | 14.44 |
| Bleak | 1241 | 2 | 3 | 1 | 23.89 | 23.29 | 22.03 | 36.73 | 62.27 | 54.35 | 42.40 | 33.76 | 26.69 | 46.05 | 48.53 | 29.93 | 58.35 | 54.57 | 14.10 |
| Bleak | 1242 | 2 | 3 | 1 | 28.50 | 25.72 | 25.94 | 45.03 | 72.72 | 65.54 | 57.50 | 43.81 | 30.54 | 52.02 | 61.25 | 36.45 | 68.83 | 66.12 | 16.08 |
| Bleak | 1243 | 2 | 3 | 1 | 24.61 | 22.07 | 22.43 | 44.76 | 69.28 | 58.60 | 49.92 | 38.66 | 34.63 | 46.86 | 53.85 | 35.25 | 61.31 | 58.65 | 13.85 |
| Bleak | 1244 | 2 | 3 | 1 | 31.94 | 24.11 | 22.89 | 43.05 | 58.63 | 53.92 | 49.21 | 37.27 | 31.06 | 43.27 | 49.95 | 39.48 | 73.47 | 68.18 | 14.24 |
| Bleak | 1245 | 2 | 3 | 1 | 22.15 | 20.31 | 18.64 | 41.94 | 61.37 | 55.10 | 47.39 | 34.02 | 22.62 | 37.82 | 44.02 | 30.53 | 52.60 | 53.27 | 12.97 |
| Bleak | 1246 | 2 | 3 | 1 | 23.87 | 22.64 | 22.05 | 41.31 | 61.99 | 52.64 | 46.34 | 37.73 | 29.85 | 54.01 | 54.79 | 33.25 | 63.26 | 56.71 | 14.88 |
| Bleak | 1247 | 2 | 3 | 1 | 23.26 | 22.65 | 21.77 | 39.76 | 61.20 | 55.08 | 46.93 | 33.05 | 21.63 | 47.88 | 51.71 | 30.64 | 58.32 | 58.02 | 13.82 |
| Bleak | 1248 | 2 | 3 | 1 | 27.16 | 24.75 | 24.28 | 41.86 | 62.51 | 54.24 | 50.25 | 35.55 | 23.26 | 53.40 | 55.99 | 32.01 | 61.14 | 56.41 | 13.79 |
| Bleak | 1249 | 2 | 3 | 1 | 19.56 | 16.98 | 15.03 | 25.96 | 40.77 | 35.71 | 32.31 | 22.32 | 15.69 | 34.58 | 36.92 | 21.03 | 39.89 | 35.79 | 9.20 |
| Bleak | 1250 | 2 | 3 | 1 | 20.24 | 19.23 | 18.09 | 31.41 | 47.79 | 40.12 | 37.11 | 27.33 | 19.86 | 38.47 | 40.00 | 25.24 | 47.26 | 43.03 | 10.89 |
| Bleak | 1250 | 2 | 3 | 1 | 20.70 | 20.62 | 19.58 | 38.05 | 52.25 | 46.10 | 43.41 | 30.63 | 22.63 | 41.31 | 45.54 | 28.23 | 55.66 | 49.42 | 12.66 |
| Bleak | 1221 | 2 | 3 | 2 | 26.40 | 24.95 | 27.21 | 45.98 | 71.52 | 62.25 | 54.86 | 39.08 | 27.61 | 53.61 | 56.40 | 39.16 | 71.50 | 66.37 | 16.63 |
| Bleak | 1222 | 2 | 3 | 2 | 23.88 | 20.19 | 19.41 | 38.44 | 54.87 | 49.53 | 44.25 | 30.11 | 24.41 | 42.09 | 44.65 | 27.95 | 53.64 | 51.80 | 13.38 |
| Bleak | 1223 | 2 | 3 | 2 | 19.87 | 18.64 | 19.73 | 36.84 | 56.69 | 52.72 | 43.78 | 30.32 | 22.35 | 47.12 | 49.96 | 27.65 | 58.84 | 56.40 | 10.87 |
| Bleak | 1224 | 2 | 3 | 2 | 29.73 | 23.56 | 24.45 | 42.86 | 65.63 | 57.16 | 53.85 | 37.01 | 24.62 | 56.13 | 63.45 | 35.08 | 66.44 | 63.91 | 18.00 |
| Bleak | 1225 | 2 | 3 | 2 | 28.29 | 25.41 | 22.46 | 44.87 | 61.73 | 51.65 | 51.30 | 36.44 | 30.21 | 52.69 | 56.64 | 33.08 | 65.52 | 65.61 | 12.00 |
| Bleak | 1226 | 2 | 3 | 2 | 20.63 | 18.69 | 18.57 | 31.74 | 48.09 | 41.37 | 40.40 | 27.99 | 19.61 | 40.67 | 44.77 | 27.28 | 49.85 | 45.98 | 12.06 |
| Bleak | 1227 | 2 | 3 | 2 | 23.69 | 22.28 | 21.86 | 38.87 | 58.93 | 49.51 | 43.99 | 34.85 | 27.67 | 40.70 | 44.03 | 31.00 | 55.93 | 52.44 | 12.93 |
| Bleak | 1228 | 2 | 3 | 2 | 25.64 | 22.41 | 21.51 | 36.15 | 59.05 | 53.66 | 44.03 | 33.59 | 24.92 | 43.70 | 49.99 | 29.56 | 61.87 | 57.94 | 13.29 |
| Bleak | 1229 | 2 | 3 | 2 | 23.97 | 21.94 | 23.01 | 40.69 | 57.70 | 47.66 | 44.92 | 33.51 | 23.97 | 44.52 | 48.38 | 29.51 | 57.08 | 53.83 | 14.18 |
| Bleak | 1230 | 2 | 3 | 2 | 31.57 | 27.28 | 25.78 | 41.38 | 67.22 | 59.82 | 50.13 | 38.14 | 30.00 | 48.00 | 52.09 | 33.27 | 61.17 | 57.97 | 16.45 |
| Bleak | 1232 | 2 | 3 | 2 | 21.96 | 21.21 | 21.14 | 43.56 | 60.52 | 49.63 | 43.58 | 33.39 | 24.91 | 40.74 | 45.59 | 32.18 | 57.41 | 54.69 | 12.47 |
| Bleak | 1233 | 2 | 3 | 2 | 31.65 | 32.08 | 29.44 | 52.57 | 86.06 | 76.38 | 64.69 | 50.45 | 38.52 | 62.29 | 71.02 | 45.52 | 83.80 | 77.44 | 18.96 |
| Bleak | 1234 | 2 | 3 | 2 | 26.63 | 22.06 | 21.02 | 40.50 | 66.17 | 59.78 | 48.22 | 39.11 | 26.42 | 44.74 | 54.48 | 33.13 | 54.88 | 53.83 | 15.86 |
| Bleak | 1235 | 2 | 3 | 2 | 23.87 | 23.54 | 23.72 | 46.47 | 63.24 | 51.83 | 49.18 | 36.10 | 29.60 | 49.79 | 53.68 | 33.96 | 63.87 | 62.62 | 14.86 |
| Bleak | 1236 | 2 | 3 | 2 | 26.50 | 24.44 | 23.03 | 37.62 | 58.11 | 50.36 | 44.83 | 34.81 | 27.59 | 46.00 | 48.38 | 30.96 | 62.49 | 60.19 | 15.09 |
| Bleak | 1237 | 2 | 3 | 2 | 22.93 | 22.36 | 21.94 | 38.65 | 59.39 | 51.94 | 44.01 | 32.74 | 22.57 | 40.75 | 48.33 | 29.48 | 52.48 | 50.48 | 13.51 |
| Bleak | 1238 | 2 | 3 | 2 | 23.45 | 21.93 | 23.64 | 42.72 | 66.47 | 60.23 | 52.62 | 35.38 | 25.87 | 42.89 | 52.52 | 35.48 | 59.10 | 60.90 | 14.25 |
| Bleak | 1239 | 2 | 3 | 2 | 27.16 | 22.22 | 26.25 | 42.55 | 68.44 | 57.51 | 53.10 | 39.79 | 25.56 | 48.93 | 56.37 | 34.74 | 61.99 | 64.44 | 15.94 |
| Bleak | 1240 | 2 | 3 | 2 | 25.21 | 20.53 | 20.40 | 40.69 | 63.61 | 57.35 | 47.21 | 35.63 | 26.65 | 50.47 | 53.34 | 32.24 | 63.24 | 58.26 | 16.45 |
| Bleak | 1241 | 2 | 3 | 2 | 23.44 | 21.62 | 22.40 | 37.28 | 61.27 | 52.63 | 42.19 | 35.70 | 28.79 | 44.06 | 49.59 | 31.64 | 59.25 | 53.58 | 15.90 |
| Bleak | 1242 | 2 | 3 | 2 | 28.74 | 25.33 | 25.22 | 45.19 | 70.72 | 64.81 | 54.69 | 41.47 | 30.30 | 55.81 | 62.94 | 35.78 | 66.31 | 63.96 | 14.41 |
| Bleak | 1243 | 2 | 3 | 2 | 25.91 | 24.06 | 23.42 | 44.31 | 70.23 | 60.78 | 53.30 | 41.38 | 32.13 | 51.91 | 56.29 | 34.54 | 57.74 | 54.25 | 13.79 |
| Bleak | 1244 | 2 | 3 | 2 | 35.24 | 26.22 | 26.42 | 36.32 | 60.58 | 54.88 | 51.98 | 38.52 | 27.49 | 46.58 | 51.08 | 40.14 | 71.12 | 68.67 | 16.92 |
| Bleak | 1245 | 2 | 3 | 2 | 23.08 | 19.97 | 20.75 | 37.27 | 65.85 | 59.10 | 46.35 | 35.34 | 21.44 | 42.01 | 47.07 | 30.33 | 55.76 | 53.89 | 14.22 |
| Bleak | 1246 | 2 | 3 | 2 | 21.75 | 22.28 | 21.89 | 41.13 | 58.52 | 49.00 | 46.99 | 36.61 | 27.16 | 54.74 | 56.33 | 32.20 | 61.61 | 54.72 | 15.43 |
| Bleak | 1247 | 2 | 3 | 2 | 19.50 | 21.34 | 21.65 | 39.40 | 60.18 | 53.98 | 47.82 | 35.17 | 24.05 | 45.63 | 50.02 | 33.08 | 60.48 | 58.65 | 14.45 |
| Bleak | 1248 | 2 | 3 | 2 | 26.31 | 23.99 | 22.59 | 44.17 | 63.74 | 53.06 | 48.89 | 35.77 | 24.54 | 55.03 | 56.20 | 31.88 | 63.70 | 54.81 | 15.06 |
| Bleak | 1249 | 2 | 3 | 2 | 18.06 | 15.99 | 16.65 | 28.36 | 42.61 | 36.28 | 30.23 | 23.76 | 18.40 | 32.21 | 33.50 | 21.07 | 39.36 | 37.25 | 11.07 |
| Bleak | 1250 | 2 | 3 | 2 | 20.14 | 19.36 | 18.78 | 33.30 | 48.48 | 40.39 | 37.29 | 25.54 | 21.47 | 37.21 | 40.07 | 24.43 | 47.15 | 45.21 | 13.30 |
| Bleak | 1250 | 2 | 3 | 2 | 20.44 | 18.97 | 21.61 | 33.71 | 54.13 | 46.98 | 45.61 | 31.49 | 25.63 | 41.21 | 42.28 | 30.44 | 54.30 | 51.33 | 11.69 |
| Bleak | 1221 | 2 | 3 | 3 | 29.70 | 26.21 | 27.17 | 47.82 | 71.41 | 58.37 | 52.71 | 40.03 | 30.90 | 50.19 | 51.52 | 36.45 | 67.29 | 60.23 | 15.53 |
| Bleak | 1222 | 2 | 3 | 3 | 22.58 | 19.24 | 20.94 | 35.10 | 53.97 | 48.68 | 44.40 | 29.50 | 22.92 | 42.92 | 45.35 | 27.56 | 55.39 | 53.80 | 14.21 |
| Bleak | 1223 | 2 | 3 | 3 | 21.34 | 20.70 | 18.92 | 37.98 | 58.94 | 52.67 | 45.36 | 29.38 | 21.00 | 47.87 | 49.13 | 28.67 | 57.56 | 56.03 | 13.20 |
| Bleak | 1224 | 2 | 3 | 3 | 26.10 | 25.62 | 25.01 | 44.79 | 64.61 | 57.67 | 53.42 | 36.77 | 30.48 | 53.79 | 60.62 | 34.38 | 66.52 | 61.52 | 16.04 |
| Bleak | 1225 | 2 | 3 | 3 | 28.01 | 24.63 | 23.28 | 47.02 | 63.67 | 51.42 | 51.25 | 35.84 | 33.42 | 51.63 | 55.60 | 33.63 | 64.03 | 62.22 | 14.62 |
| Bleak | 1226 | 2 | 3 | 3 | 19.59 | 18.91 | 19.42 | 36.93 | 53.07 | 47.07 | 39.86 | 29.15 | 23.17 | 41.92 | 43.28 | 25.57 | 51.08 | 49.66 | 12.90 |
| Bleak | 1227 | 2 | 3 | 3 | 23.14 | 23.60 | 20.10 | 38.62 | 59.33 | 50.51 | 43.72 | 33.35 | 26.94 | 39.90 | 45.02 | 29.08 | 57.86 | 52.41 | 13.38 |
| Bleak | 1228 | 2 | 3 | 3 | 27.11 | 23.71 | 21.41 | 36.01 | 57.04 | 50.51 | 41.68 | 35.21 | 23.78 | 45.63 | 48.14 | 29.29 | 63.60 | 60.89 | 13.25 |
| Bleak | 1229 | 2 | 3 | 3 | 24.36 | 21.64 | 22.91 | 41.55 | 58.18 | 46.75 | 44.74 | 30.98 | 22.45 | 45.35 | 50.90 | 28.75 | 58.11 | 56.27 | 13.07 |
| Bleak | 1230 | 2 | 3 | 3 | 28.93 | 26.06 | 24.65 | 39.79 | 65.50 | 57.78 | 49.49 | 39.82 | 27.41 | 54.46 | 58.87 | 35.49 | 62.37 | 62.71 | 17.12 |
| Bleak | 1232 | 2 | 3 | 3 | 23.16 | 22.24 | 21.48 | 42.24 | 58.93 | 51.04 | 47.66 | 32.50 | 22.86 | 42.04 | 46.73 | 33.16 | 56.85 | 55.56 | 13.80 |
| Bleak | 1233 | 2 | 3 | 3 | 32.94 | 33.03 | 31.87 | 58.21 | 89.99 | 79.22 | 69.89 | 47.73 | 35.13 | 61.31 | 70.09 | 41.13 | 77.72 | 73.06 | 20.32 |
| Bleak | 1234 | 2 | 3 | 3 | 25.26 | 24.57 | 24.31 | 38.02 | 66.22 | 59.76 | 47.47 | 38.52 | 26.97 | 45.97 | 53.08 | 33.99 | 54.30 | 53.44 | 15.09 |
| Bleak | 1235 | 2 | 3 | 3 | 25.42 | 20.79 | 22.13 | 46.14 | 63.51 | 52.64 | 47.92 | 36.21 | 29.35 | 50.91 | 55.25 | 32.54 | 63.41 | 62.48 | 14.73 |
| Bleak | 1236 | 2 | 3 | 3 | 27.79 | 24.24 | 20.82 | 36.89 | 60.83 | 53.66 | 44.71 | 34.39 | 25.91 | 45.82 | 53.47 | 31.28 | 62.39 | 64.17 | 14.56 |
| Bleak | 1237 | 2 | 3 | 3 | 22.57 | 21.83 | 23.26 | 36.15 | 57.56 | 48.94 | 43.51 | 31.85 | 21.83 | 41.54 | 49.07 | 29.74 | 58.51 | 51.25 | 12.02 |
| Bleak | 1238 | 2 | 3 | 3 | 23.75 | 20.86 | 21.40 | 44.02 | 63.96 | 55.89 | 47.88 | 36.60 | 27.91 | 47.54 | 53.67 | 34.96 | 63.78 | 59.44 | 17.34 |
| Bleak | 1239 | 2 | 3 | 3 | 27.02 | 23.74 | 24.28 | 43.07 | 68.54 | 60.03 | 53.62 | 37.75 | 26.93 | 46.87 | 56.76 | 32.67 | 60.42 | 61.84 | 17.21 |
| Bleak | 1240 | 2 | 3 | 3 | 25.09 | 19.37 | 20.01 | 41.05 | 63.98 | 59.25 | 48.34 | 34.53 | 25.01 | 48.26 | 52.48 | 30.91 | 62.03 | 57.10 | 14.61 |
| Bleak | 1241 | 2 | 3 | 3 | 23.26 | 21.41 | 22.44 | 34.43 | 58.20 | 50.54 | 42.76 | 32.94 | 24.29 | 49.42 | 53.48 | 29.05 | 57.23 | 55.11 | 15.84 |
| Bleak | 1242 | 2 | 3 | 3 | 28.74 | 26.75 | 27.62 | 45.33 | 73.26 | 64.73 | 54.92 | 42.80 | 31.53 | 54.61 | 60.51 | 36.35 | 69.61 | 64.86 | 16.30 |
| Bleak | 1243 | 2 | 3 | 3 | 27.02 | 22.86 | 23.79 | 50.38 | 75.54 | 59.77 | 50.86 | 41.09 | 32.13 | 47.51 | 54.82 | 35.96 | 64.55 | 59.54 | 15.18 |
| Bleak | 1244 | 2 | 3 | 3 | 25.81 | 25.59 | 24.42 | 40.44 | 60.44 | 53.04 | 52.65 | 38.15 | 27.39 | 50.96 | 54.48 | 36.74 | 65.00 | 63.28 | 15.20 |
| Bleak | 1245 | 2 | 3 | 3 | 25.53 | 22.83 | 20.05 | 38.77 | 60.41 | 55.81 | 47.36 | 34.53 | 21.52 | 38.20 | 42.44 | 31.15 | 51.26 | 49.37 | 12.53 |
| Bleak | 1246 | 2 | 3 | 3 | 23.90 | 21.33 | 23.95 | 40.25 | 60.19 | 50.64 | 46.76 | 35.80 | 29.46 | 52.49 | 55.01 | 33.45 | 64.48 | 57.78 | 15.39 |
| Bleak | 1247 | 2 | 3 | 3 | 20.51 | 20.20 | 19.21 | 42.20 | 58.90 | 52.20 | 45.14 | 34.28 | 23.56 | 46.83 | 51.50 | 30.67 | 58.72 | 54.63 | 15.45 |
| Bleak | 1248 | 2 | 3 | 3 | 25.74 | 25.23 | 23.79 | 43.41 | 63.87 | 52.29 | 47.39 | 34.06 | 23.83 | 53.77 | 56.57 | 32.31 | 60.02 | 55.22 | 14.50 |
| Bleak | 1249 | 2 | 3 | 3 | 16.14 | 16.91 | 16.66 | 26.10 | 40.53 | 34.58 | 32.58 | 20.23 | 15.30 | 35.91 | 37.42 | 21.12 | 38.76 | 35.30 | 9.37 |
| Bleak | 1250 | 2 | 3 | 3 | 19.70 | 19.99 | 18.03 | 30.22 | 49.95 | 43.37 | 36.49 | 26.48 | 21.56 | 36.12 | 41.96 | 25.41 | 49.49 | 46.43 | 11.21 |
| Bleak | 1250 | 2 | 3 | 3 | 25.30 | 19.60 | 18.83 | 34.34 | 53.05 | 46.05 | 42.98 | 31.42 | 26.34 | 39.10 | 43.84 | 27.34 | 52.75 | 50.15 | 12.17 |
| Bleak | 1031 | 3 | 1 | 1 | 18.87 | 16.13 | 16.80 | 29.63 | 45.34 | 41.52 | 36.33 | 25.66 | 19.15 | 33.42 | 36.72 | 24.23 | 43.35 | 39.73 | 10.62 |
| Bleak | 1032 | 3 | 1 | 1 | 22.89 | 20.94 | 20.67 | 37.51 | 55.49 | 53.02 | 46.76 | 29.89 | 22.42 | 43.84 | 47.64 | 29.06 | 55.69 | 53.33 | 11.87 |
| Bleak | 1033 | 3 | 1 | 1 | 23.14 | 21.14 | 21.15 | 39.01 | 60.88 | 54.80 | 46.60 | 34.43 | 25.73 | 45.11 | 49.10 | 32.70 | 56.43 | 53.00 | 12.33 |
| Bleak | 1034 | 3 | 1 | 1 | 18.03 | 16.69 | 16.31 | 28.06 | 44.12 | 40.50 | 34.82 | 24.59 | 19.21 | 34.30 | 37.97 | 22.71 | 45.45 | 42.34 | 10.85 |
| Bleak | 1035 | 3 | 1 | 1 | 13.86 | 14.45 | 13.13 | 22.36 | 34.46 | 30.48 | 26.33 | 19.83 | 15.99 | 27.01 | 29.42 | 18.95 | 36.53 | 34.22 | 7.97 |
| Bleak | 1036 | 3 | 1 | 1 | 20.27 | 20.08 | 19.52 | 33.71 | 53.94 | 48.54 | 40.91 | 32.28 | 22.97 | 42.81 | 46.29 | 29.85 | 55.10 | 51.69 | 12.31 |
| Bleak | 1037 | 3 | 1 | 1 | 19.84 | 17.55 | 17.42 | 31.94 | 48.40 | 44.93 | 38.47 | 27.29 | 18.62 | 38.09 | 40.92 | 24.66 | 47.63 | 43.38 | 11.15 |
| Bleak | 1038 | 3 | 1 | 1 | 20.41 | 17.23 | 17.85 | 30.59 | 47.77 | 43.83 | 39.21 | 28.68 | 19.68 | 36.17 | 39.83 | 27.51 | 48.99 | 46.51 | 10.80 |
| Bleak | 1039 | 3 | 1 | 1 | 16.23 | 15.58 | 15.10 | 26.92 | 40.81 | 35.97 | 31.90 | 23.82 | 18.00 | 30.79 | 33.91 | 23.20 | 41.14 | 38.26 | 10.02 |
| Bleak | 1040 | 3 | 1 | 1 | 22.16 | 19.26 | 20.71 | 39.06 | 59.94 | 56.43 | 47.91 | 34.19 | 24.47 | 40.82 | 48.59 | 33.11 | 57.17 | 57.96 | 13.57 |
| Bleak | 1041 | 3 | 1 | 1 | 15.01 | 14.54 | 13.60 | 23.62 | 36.72 | 30.15 | 28.89 | 16.75 | 18.91 | 28.35 | 31.49 | 18.93 | 35.99 | 33.90 | 7.70 |
| Bleak | 1042 | 3 | 1 | 1 | 17.71 | 17.42 | 16.92 | 32.98 | 46.43 | 39.87 | 37.43 | 26.19 | 18.04 | 38.84 | 41.66 | 25.22 | 49.11 | 45.48 | 10.69 |
| Bleak | 1043 | 3 | 1 | 1 | 18.37 | 17.22 | 17.60 | 33.08 | 50.70 | 45.02 | 39.30 | 29.14 | 21.35 | 41.00 | 44.59 | 27.79 | 51.70 | 48.44 | 11.74 |
| Bleak | 1044 | 3 | 1 | 1 | 16.71 | 15.40 | 14.42 | 29.05 | 43.45 | 37.92 | 31.68 | 21.61 | 15.80 | 33.43 | 36.27 | 19.38 | 40.43 | 38.21 | 9.91 |
| Bleak | 1045 | 3 | 1 | 1 | 16.59 | 16.91 | 16.40 | 29.39 | 44.67 | 39.39 | 35.16 | 25.97 | 18.37 | 35.40 | 39.67 | 24.01 | 45.97 | 44.19 | 9.70 |
| Bleak | 1046 | 3 | 1 | 1 | 20.91 | 18.00 | 18.60 | 34.85 | 53.20 | 48.42 | 42.22 | 29.70 | 23.22 | 41.44 | 44.58 | 27.45 | 50.75 | 46.83 | 12.81 |
| Bleak | 1047 | 3 | 1 | 1 | 22.39 | 19.78 | 19.52 | 37.04 | 53.47 | 47.53 | 43.56 | 29.04 | 21.48 | 40.70 | 44.39 | 29.11 | 53.06 | 49.41 | 12.06 |
| Bleak | 1048 | 3 | 1 | 1 | 17.64 | 18.12 | 16.50 | 30.45 | 46.15 | 40.43 | 35.23 | 25.20 | 17.41 | 34.60 | 38.91 | 25.10 | 47.41 | 45.00 | 11.03 |
| Bleak | 1049 | 3 | 1 | 1 | 23.37 | 21.62 | 21.94 | 40.47 | 60.96 | 53.81 | 48.19 | 35.64 | 27.35 | 41.88 | 47.43 | 33.32 | 56.35 | 54.06 | 13.24 |
| Bleak | 1050 | 3 | 1 | 1 | 15.88 | 15.83 | 14.61 | 26.31 | 41.30 | 37.09 | 31.68 | 23.03 | 18.88 | 33.60 | 37.13 | 21.69 | 43.08 | 41.41 | 9.62 |
| Bleak | 1051 | 3 | 1 | 1 | 19.84 | 18.19 | 17.98 | 36.50 | 51.82 | 47.30 | 42.35 | 28.28 | 22.61 | 39.37 | 43.02 | 25.89 | 49.59 | 46.64 | 11.38 |
| Bleak | 1052 | 3 | 1 | 1 | 20.28 | 18.95 | 19.88 | 39.08 | 57.97 | 50.37 | 43.69 | 33.31 | 24.80 | 40.05 | 45.39 | 29.56 | 54.74 | 53.86 | 11.83 |
| Bleak | 1053 | 3 | 1 | 1 | 22.17 | 20.45 | 19.81 | 34.29 | 51.71 | 46.18 | 41.17 | 29.81 | 21.85 | 40.48 | 45.17 | 27.62 | 51.03 | 48.24 | 10.97 |
| Bleak | 1054 | 3 | 1 | 1 | 15.42 | 14.27 | 14.21 | 27.23 | 41.27 | 35.83 | 31.75 | 23.41 | 15.78 | 32.48 | 35.80 | 22.30 | 41.31 | 39.43 | 9.03 |
| Bleak | 1055 | 3 | 1 | 1 | 17.64 | 16.25 | 16.50 | 29.19 | 45.55 | 40.87 | 35.79 | 27.37 | 20.29 | 35.26 | 39.38 | 24.57 | 45.14 | 43.67 | 10.12 |
| Bleak | 1056 | 3 | 1 | 1 | 14.55 | 12.64 | 12.53 | 22.46 | 34.15 | 30.62 | 26.34 | 18.52 | 14.85 | 27.07 | 29.59 | 18.07 | 35.43 | 33.46 | 8.03 |
| Bleak | 1057 | 3 | 1 | 1 | 15.27 | 14.51 | 14.00 | 23.74 | 36.35 | 31.60 | 28.33 | 20.69 | 15.19 | 31.55 | 31.63 | 19.73 | 36.67 | 32.27 | 8.27 |
| Bleak | 1058 | 3 | 1 | 1 | 20.72 | 18.40 | 18.30 | 32.98 | 48.33 | 43.38 | 38.83 | 28.77 | 20.29 | 31.45 | 34.76 | 27.11 | 44.34 | 41.06 | 10.21 |
| Bleak | 1059 | 3 | 1 | 1 | 16.96 | 16.95 | 15.42 | 30.83 | 38.57 | 33.41 | 33.77 | 22.93 | 21.70 | 32.93 | 38.02 | 21.46 | 41.97 | 40.86 | 10.20 |
| Bleak | 1060 | 3 | 1 | 1 | 18.08 | 16.07 | 16.03 | 30.34 | 45.97 | 41.22 | 37.04 | 26.20 | 18.74 | 35.33 | 38.22 | 24.91 | 46.72 | 43.71 | 10.71 |
| Bleak | 1031 | 3 | 1 | 2 | 17.38 | 15.70 | 16.51 | 31.15 | 45.97 | 40.36 | 34.62 | 25.85 | 19.22 | 34.82 | 37.70 | 24.15 | 44.28 | 40.68 | 11.26 |
| Bleak | 1032 | 3 | 1 | 2 | 22.01 | 19.81 | 20.10 | 38.56 | 56.14 | 52.57 | 46.49 | 29.46 | 23.00 | 42.19 | 46.69 | 29.53 | 56.50 | 54.44 | 12.16 |
| Bleak | 1033 | 3 | 1 | 2 | 22.18 | 20.73 | 21.16 | 39.93 | 61.03 | 53.91 | 46.28 | 34.10 | 26.00 | 45.13 | 48.38 | 33.78 | 56.44 | 53.00 | 12.41 |
| Bleak | 1034 | 3 | 1 | 2 | 18.05 | 16.96 | 16.02 | 27.98 | 43.53 | 40.18 | 34.46 | 24.84 | 19.25 | 34.68 | 37.76 | 22.62 | 44.24 | 41.65 | 10.30 |
| Bleak | 1035 | 3 | 1 | 2 | 13.62 | 14.14 | 12.98 | 22.52 | 34.42 | 30.32 | 26.68 | 19.48 | 16.31 | 26.24 | 29.05 | 19.09 | 35.53 | 34.36 | 7.88 |
| Bleak | 1036 | 3 | 1 | 2 | 20.34 | 20.31 | 19.70 | 34.19 | 55.51 | 48.97 | 40.41 | 32.62 | 22.34 | 44.03 | 47.47 | 29.58 | 54.77 | 51.67 | 12.82 |
| Bleak | 1037 | 3 | 1 | 2 | 19.64 | 17.72 | 16.95 | 32.25 | 49.61 | 45.14 | 38.24 | 28.05 | 18.93 | 37.36 | 39.72 | 24.91 | 47.74 | 44.64 | 10.06 |
| Bleak | 1038 | 3 | 1 | 2 | 20.82 | 17.58 | 18.14 | 30.66 | 46.61 | 43.06 | 39.26 | 28.99 | 19.58 | 36.02 | 40.38 | 27.72 | 49.10 | 46.69 | 10.95 |
| Bleak | 1039 | 3 | 1 | 2 | 16.43 | 15.49 | 15.31 | 26.31 | 41.35 | 37.08 | 32.15 | 24.02 | 17.91 | 31.44 | 34.09 | 23.46 | 43.31 | 39.56 | 10.01 |
| Bleak | 1040 | 3 | 1 | 2 | 22.72 | 19.52 | 20.53 | 39.84 | 60.20 | 56.58 | 47.91 | 35.14 | 24.32 | 41.18 | 49.46 | 33.08 | 56.64 | 57.74 | 13.02 |
| Bleak | 1041 | 3 | 1 | 2 | 14.69 | 13.69 | 13.42 | 24.13 | 37.51 | 32.93 | 28.32 | 21.23 | 14.88 | 28.25 | 31.18 | 18.96 | 36.53 | 34.98 | 7.82 |
| Bleak | 1042 | 3 | 1 | 2 | 17.46 | 17.77 | 17.14 | 33.83 | 46.74 | 40.08 | 37.43 | 25.84 | 17.73 | 39.14 | 41.99 | 25.72 | 49.22 | 45.90 | 10.31 |
| Bleak | 1043 | 3 | 1 | 2 | 18.32 | 17.22 | 17.40 | 32.81 | 51.04 | 45.51 | 39.23 | 29.09 | 21.45 | 40.80 | 44.44 | 28.03 | 51.75 | 48.54 | 12.05 |
| Bleak | 1044 | 3 | 1 | 2 | 16.01 | 15.19 | 14.74 | 28.80 | 42.92 | 38.61 | 32.48 | 21.89 | 16.10 | 34.26 | 36.98 | 19.81 | 40.54 | 39.04 | 10.10 |
| Bleak | 1045 | 3 | 1 | 2 | 18.75 | 17.31 | 16.70 | 29.29 | 44.01 | 39.33 | 35.03 | 25.94 | 18.15 | 36.01 | 39.80 | 23.75 | 44.85 | 43.29 | 9.72 |
| Bleak | 1046 | 3 | 1 | 2 | 20.50 | 18.83 | 18.95 | 34.33 | 55.28 | 49.48 | 41.44 | 30.41 | 23.57 | 42.35 | 45.21 | 26.90 | 51.25 | 47.12 | 12.31 |
| Bleak | 1047 | 3 | 1 | 2 | 22.39 | 18.98 | 19.06 | 38.23 | 54.07 | 48.27 | 43.66 | 29.47 | 21.47 | 41.79 | 45.60 | 29.08 | 52.71 | 48.96 | 12.16 |
| Bleak | 1048 | 3 | 1 | 2 | 17.72 | 17.78 | 16.51 | 31.20 | 46.08 | 39.66 | 34.24 | 25.16 | 18.46 | 34.51 | 38.51 | 25.10 | 46.55 | 44.87 | 10.59 |
| Bleak | 1049 | 3 | 1 | 2 | 23.01 | 21.03 | 21.38 | 41.10 | 60.77 | 53.96 | 48.42 | 36.23 | 27.94 | 41.52 | 47.97 | 33.35 | 56.91 | 53.02 | 13.51 |
| Bleak | 1050 | 3 | 1 | 2 | 16.10 | 15.70 | 14.55 | 28.36 | 42.11 | 37.39 | 31.95 | 22.95 | 18.26 | 33.86 | 36.64 | 21.54 | 44.07 | 42.16 | 9.96 |
| Bleak | 1051 | 3 | 1 | 2 | 19.65 | 17.34 | 17.71 | 36.13 | 52.29 | 48.00 | 43.04 | 28.41 | 22.62 | 38.81 | 42.93 | 26.04 | 49.04 | 47.24 | 11.44 |
| Bleak | 1052 | 3 | 1 | 2 | 19.50 | 19.15 | 19.76 | 39.54 | 58.70 | 50.96 | 44.23 | 33.47 | 24.38 | 39.69 | 45.33 | 29.67 | 54.38 | 53.57 | 11.50 |
| Bleak | 1053 | 3 | 1 | 2 | 21.53 | 19.63 | 19.40 | 33.52 | 51.12 | 46.59 | 41.39 | 30.01 | 22.13 | 40.42 | 45.17 | 27.64 | 51.46 | 49.40 | 11.40 |
| Bleak | 1054 | 3 | 1 | 2 | 15.21 | 14.49 | 14.60 | 27.64 | 40.88 | 35.81 | 31.99 | 23.15 | 15.78 | 32.22 | 35.55 | 22.01 | 41.41 | 39.50 | 9.30 |
| Bleak | 1055 | 3 | 1 | 2 | 17.72 | 15.92 | 16.05 | 29.81 | 45.96 | 40.86 | 34.71 | 26.90 | 20.88 | 34.87 | 38.40 | 25.07 | 45.83 | 44.03 | 9.45 |
| Bleak | 1056 | 3 | 1 | 2 | 14.17 | 12.97 | 12.75 | 21.95 | 34.22 | 31.03 | 26.73 | 18.31 | 14.03 | 27.54 | 30.19 | 18.26 | 35.82 | 33.66 | 8.10 |
| Bleak | 1057 | 3 | 1 | 2 | 15.29 | 14.05 | 14.00 | 25.34 | 36.64 | 32.22 | 28.70 | 20.98 | 14.99 | 31.06 | 32.15 | 19.69 | 35.90 | 31.77 | 8.36 |
| Bleak | 1058 | 3 | 1 | 2 | 18.93 | 17.49 | 17.44 | 31.58 | 42.43 | 35.96 | 30.81 | 34.04 | 26.30 | 32.04 | 34.91 | 26.92 | 43.15 | 39.19 | 9.82 |
| Bleak | 1059 | 3 | 1 | 2 | 16.54 | 16.42 | 15.35 | 31.63 | 48.25 | 42.17 | 33.98 | 24.10 | 17.51 | 33.37 | 38.38 | 21.17 | 41.08 | 40.25 | 9.89 |
| Bleak | 1060 | 3 | 1 | 2 | 17.88 | 16.46 | 16.12 | 29.83 | 45.75 | 41.30 | 38.36 | 26.22 | 17.97 | 35.39 | 38.78 | 24.87 | 45.94 | 42.91 | 10.63 |
| Bleak | 1031 | 3 | 1 | 3 | 18.20 | 16.32 | 16.80 | 29.10 | 45.42 | 40.63 | 36.26 | 24.74 | 18.16 | 33.48 | 36.30 | 24.44 | 45.51 | 41.40 | 10.64 |
| Bleak | 1032 | 3 | 1 | 3 | 22.48 | 20.77 | 20.52 | 37.96 | 55.02 | 52.27 | 46.63 | 29.94 | 22.89 | 43.25 | 47.31 | 28.88 | 56.59 | 54.17 | 11.85 |
| Bleak | 1033 | 3 | 1 | 3 | 23.46 | 22.01 | 21.91 | 39.65 | 60.44 | 54.04 | 46.24 | 34.84 | 26.69 | 44.28 | 48.77 | 33.10 | 56.92 | 53.45 | 12.88 |
| Bleak | 1034 | 3 | 1 | 3 | 17.65 | 16.51 | 16.16 | 27.68 | 42.66 | 39.77 | 34.80 | 24.55 | 18.90 | 33.93 | 37.40 | 23.05 | 45.57 | 42.96 | 10.51 |
| Bleak | 1035 | 3 | 1 | 3 | 13.87 | 14.37 | 13.00 | 22.32 | 34.75 | 30.58 | 26.59 | 20.03 | 15.64 | 27.20 | 29.65 | 19.05 | 36.15 | 35.09 | 7.41 |
| Bleak | 1036 | 3 | 1 | 3 | 19.89 | 20.68 | 20.11 | 34.44 | 55.00 | 48.20 | 40.36 | 32.28 | 23.14 | 42.48 | 46.49 | 29.30 | 54.10 | 50.79 | 12.16 |
| Bleak | 1037 | 3 | 1 | 3 | 20.61 | 17.72 | 17.48 | 30.71 | 47.46 | 45.56 | 38.64 | 26.97 | 18.91 | 37.56 | 40.97 | 24.60 | 46.38 | 42.98 | 10.82 |
| Bleak | 1038 | 3 | 1 | 3 | 20.01 | 17.38 | 18.37 | 30.65 | 46.98 | 42.84 | 38.46 | 28.14 | 20.00 | 36.54 | 40.86 | 28.33 | 49.95 | 47.71 | 10.81 |
| Bleak | 1039 | 3 | 1 | 3 | 16.73 | 15.23 | 15.00 | 26.81 | 42.18 | 37.46 | 31.82 | 24.38 | 18.90 | 29.95 | 33.64 | 23.40 | 41.58 | 38.85 | 9.72 |
| Bleak | 1040 | 3 | 1 | 3 | 22.78 | 19.77 | 20.07 | 40.22 | 60.04 | 55.98 | 48.23 | 35.14 | 24.02 | 41.02 | 48.96 | 33.34 | 56.54 | 57.39 | 12.52 |
| Bleak | 1041 | 3 | 1 | 3 | 14.74 | 14.86 | 13.81 | 23.94 | 38.21 | 33.36 | 27.88 | 20.96 | 15.44 | 28.50 | 31.37 | 19.11 | 35.91 | 34.38 | 7.89 |
| Bleak | 1042 | 3 | 1 | 3 | 18.06 | 17.41 | 17.02 | 33.04 | 46.39 | 39.58 | 36.78 | 26.13 | 17.67 | 40.10 | 42.09 | 25.46 | 49.33 | 46.10 | 10.14 |
| Bleak | 1043 | 3 | 1 | 3 | 18.72 | 17.67 | 17.81 | 33.80 | 51.83 | 46.04 | 38.90 | 29.38 | 21.35 | 41.79 | 44.33 | 27.82 | 51.61 | 48.16 | 11.77 |
| Bleak | 1044 | 3 | 1 | 3 | 16.34 | 15.53 | 14.58 | 28.25 | 42.74 | 37.81 | 31.90 | 21.68 | 16.00 | 33.55 | 36.28 | 19.41 | 41.10 | 38.87 | 9.80 |
| Bleak | 1045 | 3 | 1 | 3 | 18.20 | 16.98 | 15.90 | 29.19 | 44.15 | 39.02 | 35.17 | 26.09 | 17.61 | 36.72 | 39.41 | 23.76 | 46.05 | 43.75 | 9.81 |
| Bleak | 1046 | 3 | 1 | 3 | 21.16 | 19.21 | 18.94 | 34.89 | 54.59 | 49.03 | 40.99 | 30.14 | 23.88 | 42.78 | 45.16 | 27.23 | 51.58 | 47.29 | 12.63 |
| Bleak | 1047 | 3 | 1 | 3 | 22.15 | 19.69 | 19.05 | 36.59 | 53.54 | 47.99 | 43.58 | 29.53 | 20.85 | 41.37 | 45.68 | 29.53 | 53.43 | 49.56 | 11.95 |
| Bleak | 1048 | 3 | 1 | 3 | 17.92 | 17.71 | 16.80 | 30.45 | 46.36 | 40.58 | 35.03 | 25.20 | 17.26 | 34.73 | 39.26 | 24.60 | 47.50 | 45.46 | 10.44 |
| Bleak | 1049 | 3 | 1 | 3 | 22.92 | 20.75 | 21.50 | 41.49 | 61.52 | 54.71 | 48.00 | 35.83 | 28.38 | 42.13 | 47.52 | 33.57 | 58.14 | 55.08 | 13.41 |
| Bleak | 1050 | 3 | 1 | 3 | 16.25 | 15.63 | 14.70 | 27.46 | 41.91 | 37.54 | 31.48 | 22.77 | 18.54 | 34.21 | 37.49 | 21.21 | 43.34 | 41.41 | 9.97 |
| Bleak | 1051 | 3 | 1 | 3 | 19.67 | 16.86 | 17.63 | 36.86 | 52.52 | 47.40 | 41.20 | 28.15 | 24.16 | 39.65 | 43.40 | 25.80 | 49.06 | 46.25 | 11.20 |
| Bleak | 1052 | 3 | 1 | 3 | 19.67 | 18.96 | 19.49 | 39.17 | 58.64 | 51.73 | 44.80 | 33.23 | 24.41 | 39.70 | 45.15 | 29.90 | 53.89 | 53.02 | 11.72 |
| Bleak | 1053 | 3 | 1 | 3 | 21.26 | 19.23 | 19.22 | 33.53 | 51.99 | 47.13 | 41.15 | 29.95 | 22.56 | 40.41 | 44.82 | 27.39 | 51.31 | 48.29 | 10.99 |
| Bleak | 1054 | 3 | 1 | 3 | 14.95 | 15.23 | 14.56 | 26.45 | 40.68 | 35.32 | 30.52 | 23.76 | 17.33 | 31.95 | 34.97 | 21.88 | 40.28 | 38.51 | 9.31 |
| Bleak | 1055 | 3 | 1 | 3 | 17.78 | 15.77 | 16.20 | 29.67 | 45.93 | 41.51 | 36.11 | 27.11 | 20.43 | 35.27 | 38.21 | 24.98 | 46.02 | 43.01 | 10.42 |
| Bleak | 1056 | 3 | 1 | 3 | 14.07 | 13.41 | 12.91 | 21.86 | 33.02 | 29.00 | 26.72 | 18.34 | 13.35 | 28.35 | 30.70 | 17.96 | 34.85 | 32.86 | 7.95 |
| Bleak | 1057 | 3 | 1 | 3 | 15.17 | 14.27 | 13.81 | 24.80 | 36.68 | 32.51 | 29.06 | 21.16 | 14.89 | 31.04 | 31.52 | 19.50 | 36.15 | 31.89 | 8.36 |
| Bleak | 1058 | 3 | 1 | 3 | 19.32 | 18.50 | 17.55 | 32.34 | 50.09 | 44.45 | 37.97 | 28.68 | 20.32 | 32.52 | 35.59 | 26.33 | 42.65 | 39.00 | 10.08 |
| Bleak | 1059 | 3 | 1 | 3 | 17.06 | 16.42 | 15.24 | 30.04 | 47.87 | 41.17 | 32.57 | 24.12 | 18.48 | 34.46 | 38.38 | 21.37 | 43.26 | 42.00 | 10.21 |
| Bleak | 1060 | 3 | 1 | 3 | 18.07 | 16.64 | 16.38 | 32.33 | 45.26 | 40.62 | 37.80 | 25.95 | 17.61 | 35.72 | 39.41 | 24.82 | 46.50 | 43.97 | 10.53 |
| Bleak | 1131 | 3 | 2 | 1 | 30.54 | 28.07 | 27.61 | 49.34 | 81.02 | 72.93 | 60.28 | 47.40 | 36.72 | 53.57 | 61.81 | 40.47 | 75.49 | 74.01 | 16.60 |
| Bleak | 1132 | 3 | 2 | 1 | 24.75 | 23.82 | 22.53 | 42.20 | 61.05 | 52.72 | 48.35 | 34.92 | 25.00 | 47.78 | 52.81 | 34.38 | 63.34 | 57.17 | 14.63 |
| Bleak | 1133 | 3 | 2 | 1 | 23.41 | 23.86 | 23.54 | 41.70 | 61.81 | 51.92 | 47.59 | 37.64 | 29.97 | 48.29 | 54.09 | 33.47 | 61.20 | 59.10 | 14.11 |
| Bleak | 1134 | 3 | 2 | 1 | 19.75 | 19.39 | 19.83 | 41.05 | 54.85 | 46.22 | 45.87 | 32.64 | 23.80 | 39.47 | 45.40 | 31.45 | 52.77 | 50.31 | 13.80 |
| Bleak | 1135 | 3 | 2 | 1 | 24.19 | 24.34 | 23.78 | 44.63 | 61.32 | 51.04 | 49.32 | 34.54 | 25.11 | 49.03 | 53.82 | 33.86 | 61.51 | 58.00 | 13.77 |
| Bleak | 1136 | 3 | 2 | 1 | 21.32 | 22.30 | 21.54 | 38.87 | 58.87 | 49.49 | 43.45 | 32.69 | 23.62 | 43.84 | 48.86 | 29.80 | 55.12 | 52.44 | 12.77 |
| Bleak | 1137 | 3 | 2 | 1 | 25.16 | 25.18 | 23.43 | 43.71 | 64.89 | 56.51 | 51.35 | 38.56 | 28.86 | 52.87 | 57.03 | 35.90 | 70.83 | 66.44 | 16.35 |
| Bleak | 1138 | 3 | 2 | 1 | 26.58 | 22.16 | 23.26 | 46.67 | 69.92 | 64.10 | 55.68 | 38.45 | 28.73 | 50.34 | 54.56 | 33.33 | 65.78 | 59.57 | 14.50 |
| Bleak | 1139 | 3 | 2 | 1 | 34.09 | 30.99 | 32.22 | 60.40 | 87.65 | 74.16 | 68.77 | 53.15 | 45.15 | 61.81 | 65.66 | 49.23 | 85.14 | 76.82 | 19.37 |
| Bleak | 1140 | 3 | 2 | 1 | 26.24 | 27.98 | 25.20 | 43.08 | 67.13 | 59.68 | 51.15 | 37.84 | 27.05 | 50.77 | 58.00 | 34.08 | 65.73 | 62.61 | 15.15 |
| Bleak | 1141 | 3 | 2 | 1 | 20.35 | 21.62 | 19.48 | 30.83 | 46.16 | 39.20 | 37.34 | 28.78 | 17.44 | 37.90 | 41.96 | 26.09 | 46.22 | 43.06 | 12.32 |
| Bleak | 1142 | 3 | 2 | 1 | 30.72 | 27.51 | 28.16 | 50.84 | 76.68 | 66.18 | 58.95 | 45.74 | 36.89 | 54.30 | 63.24 | 41.51 | 77.81 | 74.80 | 15.47 |
| Bleak | 1143 | 3 | 2 | 1 | 23.90 | 23.41 | 23.25 | 40.50 | 61.13 | 53.39 | 47.36 | 34.11 | 23.15 | 52.76 | 55.64 | 33.01 | 64.62 | 61.50 | 14.22 |
| Bleak | 1144 | 3 | 2 | 1 | 26.01 | 25.62 | 24.84 | 46.65 | 68.74 | 59.70 | 53.28 | 38.84 | 28.22 | 47.29 | 53.01 | 36.44 | 65.22 | 62.46 | 14.03 |
| Bleak | 1145 | 3 | 2 | 1 | 22.77 | 20.06 | 21.38 | 36.21 | 54.46 | 48.69 | 44.97 | 33.92 | 24.86 | 41.82 | 49.46 | 32.97 | 59.68 | 58.59 | 14.59 |
| Bleak | 1146 | 3 | 2 | 1 | 30.45 | 26.98 | 27.81 | 53.07 | 77.72 | 67.30 | 60.79 | 45.39 | 36.06 | 61.90 | 62.93 | 42.79 | 75.34 | 67.04 | 17.14 |
| Bleak | 1147 | 3 | 2 | 1 | 30.96 | 30.49 | 28.38 | 53.24 | 76.22 | 66.20 | 63.11 | 44.47 | 34.89 | 57.51 | 64.52 | 41.09 | 76.09 | 73.21 | 17.01 |
| Bleak | 1148 | 3 | 2 | 1 | 30.81 | 28.16 | 26.02 | 51.96 | 79.23 | 71.26 | 60.21 | 45.54 | 32.32 | 54.93 | 62.34 | 42.05 | 75.82 | 75.26 | 15.44 |
| Bleak | 1149 | 3 | 2 | 1 | 30.66 | 27.22 | 27.62 | 51.50 | 78.92 | 69.41 | 62.59 | 47.86 | 36.67 | 57.34 | 64.81 | 40.82 | 74.58 | 72.92 | 16.84 |
| Bleak | 1150 | 3 | 2 | 1 | 23.22 | 23.12 | 24.02 | 44.35 | 66.86 | 58.93 | 52.50 | 39.16 | 27.74 | 47.58 | 55.48 | 37.80 | 65.11 | 64.42 | 13.82 |
| Bleak | 1151 | 3 | 2 | 1 | 23.30 | 22.95 | 23.73 | 40.80 | 60.65 | 52.72 | 48.20 | 35.54 | 25.04 | 45.18 | 51.87 | 34.64 | 64.75 | 62.95 | 14.28 |
| Bleak | 1152 | 3 | 2 | 1 | 25.36 | 25.50 | 25.63 | 52.44 | 79.08 | 68.51 | 57.91 | 43.55 | 32.56 | 57.26 | 62.71 | 37.64 | 69.62 | 64.12 | 15.98 |
| Bleak | 1153 | 3 | 2 | 1 | 30.16 | 29.18 | 27.75 | 52.71 | 79.34 | 73.35 | 63.07 | 43.68 | 31.24 | 57.00 | 63.96 | 41.06 | 78.31 | 78.00 | 18.25 |
| Bleak | 1154 | 3 | 2 | 1 | 23.33 | 22.19 | 22.41 | 36.70 | 58.50 | 51.33 | 45.95 | 34.47 | 23.31 | 45.31 | 49.51 | 33.25 | 58.71 | 55.21 | 13.08 |
| Bleak | 1155 | 3 | 2 | 1 | 32.83 | 28.49 | 27.95 | 48.81 | 76.10 | 71.80 | 61.47 | 45.99 | 32.52 | 54.42 | 62.26 | 42.25 | 75.74 | 72.39 | 18.01 |
| Bleak | 1156 | 3 | 2 | 1 | 26.71 | 26.80 | 24.94 | 46.03 | 69.02 | 61.43 | 54.53 | 41.49 | 30.32 | 51.77 | 57.37 | 38.47 | 68.28 | 64.96 | 15.42 |
| Bleak | 1157 | 3 | 2 | 1 | 30.76 | 25.46 | 25.12 | 49.19 | 77.57 | 71.42 | 61.05 | 44.88 | 36.40 | 55.77 | 60.57 | 37.53 | 63.82 | 62.12 | 16.10 |
| Bleak | 1158 | 3 | 2 | 1 | 29.29 | 27.49 | 26.21 | 42.36 | 64.01 | 53.12 | 51.70 | 37.70 | 25.83 | 58.09 | 59.81 | 35.79 | 69.68 | 62.51 | 15.94 |
| Bleak | 1159 | 3 | 2 | 1 | 19.75 | 18.97 | 20.52 | 38.79 | 55.61 | 47.47 | 44.33 | 33.23 | 24.95 | 44.03 | 48.10 | 30.20 | 52.49 | 49.23 | 12.09 |
| Bleak | 1160 | 3 | 2 | 1 | 26.32 | 22.76 | 23.39 | 47.51 | 68.08 | 58.41 | 52.92 | 36.59 | 31.76 | 53.45 | 55.49 | 33.99 | 64.62 | 60.51 | 14.12 |
| Bleak | 1131 | 3 | 2 | 2 | 30.70 | 28.58 | 27.44 | 48.97 | 78.92 | 71.59 | 59.01 | 47.43 | 37.16 | 53.76 | 61.98 | 39.93 | 74.81 | 72.41 | 16.50 |
| Bleak | 1132 | 3 | 2 | 2 | 23.37 | 23.55 | 23.00 | 41.09 | 58.21 | 48.97 | 47.82 | 34.60 | 24.48 | 49.65 | 53.26 | 33.92 | 62.19 | 57.61 | 14.17 |
| Bleak | 1133 | 3 | 2 | 2 | 23.94 | 24.12 | 23.24 | 42.11 | 61.48 | 52.24 | 49.43 | 37.25 | 28.16 | 47.74 | 52.79 | 33.36 | 62.75 | 60.47 | 14.41 |
| Bleak | 1134 | 3 | 2 | 2 | 19.20 | 20.20 | 20.28 | 41.30 | 55.99 | 47.27 | 46.87 | 33.35 | 21.70 | 41.05 | 46.66 | 31.17 | 52.59 | 49.98 | 13.95 |
| Bleak | 1135 | 3 | 2 | 2 | 23.78 | 24.26 | 23.35 | 45.07 | 60.09 | 47.65 | 47.12 | 34.86 | 25.03 | 48.14 | 54.03 | 33.88 | 61.14 | 58.11 | 13.22 |
| Bleak | 1136 | 3 | 2 | 2 | 19.92 | 21.02 | 20.74 | 38.79 | 56.51 | 32.74 | 44.08 | 32.57 | 22.90 | 41.88 | 47.66 | 30.48 | 55.32 | 54.42 | 12.67 |
| Bleak | 1137 | 3 | 2 | 2 | 23.99 | 25.14 | 23.04 | 45.35 | 67.63 | 60.06 | 51.98 | 39.32 | 28.66 | 52.49 | 56.71 | 34.93 | 68.69 | 64.74 | 15.43 |
| Bleak | 1138 | 3 | 2 | 2 | 26.49 | 23.16 | 23.85 | 46.22 | 69.04 | 63.77 | 55.50 | 39.59 | 29.84 | 49.91 | 53.80 | 33.07 | 62.03 | 58.37 | 14.63 |
| Bleak | 1139 | 3 | 2 | 2 | 34.17 | 30.78 | 31.29 | 60.03 | 87.07 | 70.60 | 68.22 | 52.93 | 44.85 | 61.23 | 65.13 | 49.57 | 82.67 | 75.07 | 18.42 |
| Bleak | 1140 | 3 | 2 | 2 | 24.97 | 27.46 | 25.04 | 43.84 | 68.13 | 57.99 | 49.34 | 37.19 | 27.84 | 48.88 | 56.62 | 33.73 | 64.37 | 62.89 | 14.80 |
| Bleak | 1141 | 3 | 2 | 2 | 20.18 | 20.84 | 19.47 | 30.41 | 47.30 | 41.32 | 38.05 | 28.72 | 18.39 | 37.72 | 41.34 | 25.58 | 46.61 | 43.50 | 12.02 |
| Bleak | 1142 | 3 | 2 | 2 | 31.63 | 27.26 | 27.73 | 51.20 | 76.68 | 66.21 | 58.67 | 46.26 | 38.26 | 55.98 | 63.24 | 41.69 | 76.99 | 74.16 | 16.80 |
| Bleak | 1143 | 3 | 2 | 2 | 23.44 | 22.54 | 22.95 | 41.85 | 62.03 | 54.31 | 48.03 | 34.39 | 23.48 | 51.70 | 55.10 | 32.62 | 65.53 | 61.04 | 13.94 |
| Bleak | 1144 | 3 | 2 | 2 | 26.40 | 25.30 | 24.44 | 47.66 | 69.60 | 61.34 | 52.50 | 39.00 | 29.85 | 46.87 | 53.64 | 35.59 | 66.15 | 64.18 | 13.99 |
| Bleak | 1145 | 3 | 2 | 2 | 23.26 | 19.50 | 20.70 | 35.91 | 54.31 | 48.72 | 45.21 | 34.32 | 24.73 | 42.52 | 49.30 | 32.57 | 59.28 | 58.84 | 14.83 |
| Bleak | 1146 | 3 | 2 | 2 | 28.07 | 27.71 | 27.86 | 55.07 | 78.77 | 68.51 | 62.09 | 45.17 | 35.07 | 59.57 | 62.94 | 43.17 | 76.03 | 67.65 | 16.13 |
| Bleak | 1147 | 3 | 2 | 2 | 31.49 | 29.73 | 28.38 | 53.21 | 76.94 | 67.02 | 62.90 | 45.00 | 36.34 | 56.44 | 62.86 | 41.75 | 75.94 | 71.41 | 16.30 |
| Bleak | 1148 | 3 | 2 | 2 | 30.64 | 27.82 | 26.61 | 51.74 | 77.21 | 69.06 | 59.77 | 45.39 | 31.93 | 54.88 | 62.48 | 41.79 | 76.00 | 75.11 | 15.58 |
| Bleak | 1149 | 3 | 2 | 2 | 30.13 | 26.54 | 27.39 | 52.34 | 79.48 | 71.01 | 62.83 | 47.60 | 37.44 | 57.00 | 64.99 | 40.41 | 73.75 | 71.31 | 16.82 |
| Bleak | 1150 | 3 | 2 | 2 | 23.39 | 22.56 | 23.86 | 45.15 | 67.66 | 60.23 | 52.97 | 39.43 | 28.10 | 48.68 | 53.69 | 37.07 | 65.94 | 64.37 | 14.39 |
| Bleak | 1151 | 3 | 2 | 2 | 24.02 | 23.26 | 23.45 | 40.05 | 58.72 | 51.20 | 47.86 | 35.77 | 24.84 | 45.21 | 51.73 | 34.36 | 64.46 | 63.65 | 14.85 |
| Bleak | 1152 | 3 | 2 | 2 | 24.69 | 26.57 | 25.18 | 52.15 | 79.88 | 70.89 | 59.62 | 45.10 | 33.61 | 55.74 | 63.02 | 38.69 | 71.72 | 68.66 | 16.46 |
| Bleak | 1153 | 3 | 2 | 2 | 30.09 | 30.18 | 27.89 | 54.31 | 79.83 | 72.04 | 62.59 | 42.81 | 31.74 | 55.24 | 64.68 | 41.87 | 79.77 | 78.44 | 18.58 |
| Bleak | 1154 | 3 | 2 | 2 | 23.20 | 21.65 | 22.40 | 37.31 | 58.12 | 50.51 | 45.72 | 36.01 | 22.73 | 46.41 | 50.53 | 33.03 | 59.08 | 54.63 | 13.30 |
| Bleak | 1155 | 3 | 2 | 2 | 32.01 | 27.53 | 28.41 | 48.23 | 75.73 | 69.81 | 61.82 | 45.39 | 31.40 | 54.25 | 62.26 | 43.03 | 77.59 | 74.93 | 18.14 |
| Bleak | 1156 | 3 | 2 | 2 | 25.94 | 26.14 | 24.65 | 47.05 | 69.25 | 60.73 | 54.32 | 40.69 | 30.86 | 50.56 | 56.26 | 39.46 | 69.68 | 65.94 | 15.21 |
| Bleak | 1157 | 3 | 2 | 2 | 30.15 | 24.17 | 24.47 | 52.93 | 77.88 | 70.62 | 61.17 | 44.22 | 36.96 | 55.03 | 60.64 | 38.35 | 65.36 | 62.55 | 16.80 |
| Bleak | 1158 | 3 | 2 | 2 | 29.23 | 26.93 | 26.81 | 42.71 | 63.70 | 54.36 | 51.39 | 38.52 | 27.99 | 56.72 | 58.70 | 37.07 | 71.99 | 64.31 | 15.84 |
| Bleak | 1159 | 3 | 2 | 2 | 19.87 | 19.47 | 20.74 | 38.52 | 55.40 | 45.95 | 43.58 | 33.52 | 25.42 | 43.12 | 47.37 | 30.24 | 52.46 | 48.63 | 12.74 |
| Bleak | 1160 | 3 | 2 | 2 | 25.47 | 22.60 | 23.39 | 48.21 | 67.77 | 58.61 | 53.32 | 37.05 | 31.39 | 52.34 | 55.13 | 33.54 | 64.16 | 59.10 | 14.07 |
| Bleak | 1131 | 3 | 2 | 3 | 29.98 | 27.68 | 26.62 | 51.13 | 81.11 | 73.07 | 59.45 | 48.04 | 36.34 | 56.18 | 62.11 | 39.66 | 77.03 | 74.61 | 16.38 |
| Bleak | 1132 | 3 | 2 | 3 | 24.44 | 25.32 | 23.44 | 40.36 | 59.18 | 50.69 | 46.99 | 34.98 | 25.27 | 49.41 | 53.70 | 34.57 | 61.13 | 57.23 | 13.93 |
| Bleak | 1133 | 3 | 2 | 3 | 23.50 | 23.48 | 23.27 | 41.59 | 63.53 | 53.29 | 48.11 | 37.71 | 28.96 | 75.96 | 83.31 | 38.65 | 63.66 | 61.02 | 14.25 |
| Bleak | 1134 | 3 | 2 | 3 | 20.55 | 19.83 | 20.40 | 41.59 | 55.05 | 45.77 | 45.48 | 32.74 | 24.12 | 39.96 | 46.58 | 31.24 | 54.84 | 52.36 | 13.65 |
| Bleak | 1135 | 3 | 2 | 3 | 23.37 | 24.01 | 23.76 | 44.35 | 61.05 | 50.29 | 49.32 | 35.02 | 24.53 | 47.78 | 53.05 | 33.60 | 61.70 | 59.20 | 13.44 |
| Bleak | 1136 | 3 | 2 | 3 | 20.97 | 22.01 | 21.25 | 38.87 | 57.87 | 48.59 | 42.82 | 33.01 | 24.20 | 43.01 | 49.46 | 30.06 | 55.03 | 52.01 | 12.30 |
| Bleak | 1137 | 3 | 2 | 3 | 26.04 | 25.71 | 23.73 | 42.56 | 64.76 | 56.38 | 51.36 | 38.49 | 29.07 | 52.75 | 57.30 | 35.45 | 70.07 | 66.13 | 16.06 |
| Bleak | 1138 | 3 | 2 | 3 | 27.00 | 24.04 | 23.83 | 46.89 | 70.15 | 63.31 | 54.83 | 38.98 | 29.99 | 50.93 | 54.42 | 33.57 | 65.06 | 59.81 | 15.19 |
| Bleak | 1139 | 3 | 2 | 3 | 33.97 | 31.13 | 31.44 | 60.03 | 85.54 | 72.17 | 67.85 | 52.48 | 44.94 | 61.18 | 64.76 | 48.35 | 81.79 | 73.30 | 17.85 |
| Bleak | 1140 | 3 | 2 | 3 | 27.84 | 26.77 | 24.92 | 45.17 | 66.41 | 56.56 | 50.14 | 37.22 | 27.20 | 51.05 | 57.12 | 33.71 | 64.35 | 62.16 | 15.16 |
| Bleak | 1141 | 3 | 2 | 3 | 20.44 | 19.80 | 19.15 | 31.24 | 47.50 | 40.57 | 37.47 | 28.34 | 18.06 | 37.61 | 41.53 | 25.89 | 46.82 | 43.32 | 12.05 |
| Bleak | 1142 | 3 | 2 | 3 | 31.92 | 28.74 | 28.26 | 50.32 | 76.51 | 64.66 | 57.78 | 46.03 | 38.96 | 55.26 | 62.53 | 41.73 | 77.99 | 76.21 | 15.59 |
| Bleak | 1143 | 3 | 2 | 3 | 22.88 | 22.73 | 23.22 | 40.80 | 60.75 | 53.27 | 47.17 | 33.60 | 22.85 | 52.98 | 55.82 | 32.10 | 64.76 | 61.56 | 13.87 |
| Bleak | 1144 | 3 | 2 | 3 | 26.88 | 24.23 | 23.72 | 46.65 | 69.21 | 61.15 | 53.48 | 37.58 | 29.41 | 46.73 | 53.49 | 35.47 | 63.67 | 62.10 | 13.69 |
| Bleak | 1145 | 3 | 2 | 3 | 22.64 | 19.52 | 20.92 | 36.36 | 55.26 | 49.72 | 44.33 | 33.87 | 26.20 | 41.67 | 48.67 | 33.10 | 59.12 | 58.19 | 13.79 |
| Bleak | 1146 | 3 | 2 | 3 | 28.48 | 29.03 | 28.48 | 53.55 | 78.75 | 67.94 | 60.55 | 45.27 | 35.45 | 61.12 | 63.62 | 43.46 | 76.63 | 68.16 | 16.33 |
| Bleak | 1147 | 3 | 2 | 3 | 30.49 | 30.31 | 28.49 | 54.75 | 77.64 | 65.90 | 62.71 | 44.65 | 35.14 | 57.14 | 63.91 | 41.35 | 75.62 | 72.31 | 16.66 |
| Bleak | 1148 | 3 | 2 | 3 | 30.88 | 28.30 | 26.40 | 50.88 | 78.42 | 69.75 | 57.58 | 45.42 | 31.89 | 56.39 | 63.42 | 41.37 | 75.57 | 74.30 | 14.90 |
| Bleak | 1149 | 3 | 2 | 3 | 30.84 | 26.69 | 28.09 | 52.12 | 78.64 | 70.21 | 63.20 | 48.14 | 37.71 | 57.10 | 64.27 | 40.97 | 74.07 | 71.89 | 16.68 |
| Bleak | 1150 | 3 | 2 | 3 | 23.54 | 23.00 | 23.71 | 44.10 | 67.13 | 60.08 | 52.72 | 38.90 | 28.21 | 45.40 | 52.60 | 37.97 | 65.77 | 65.11 | 14.58 |
| Bleak | 1151 | 3 | 2 | 3 | 23.26 | 23.33 | 23.01 | 40.61 | 59.55 | 51.23 | 48.05 | 34.51 | 24.05 | 45.21 | 51.89 | 34.41 | 67.38 | 64.06 | 14.85 |
| Bleak | 1152 | 3 | 2 | 3 | 25.69 | 25.68 | 24.90 | 52.75 | 78.99 | 69.13 | 59.10 | 44.82 | 32.73 | 56.63 | 62.57 | 38.35 | 69.49 | 67.74 | 14.61 |
| Bleak | 1153 | 3 | 2 | 3 | 28.82 | 30.24 | 27.66 | 54.26 | 79.88 | 71.56 | 62.30 | 43.55 | 30.84 | 57.04 | 64.14 | 41.42 | 78.38 | 76.32 | 17.24 |
| Bleak | 1154 | 3 | 2 | 3 | 23.22 | 22.01 | 22.95 | 38.00 | 58.93 | 51.72 | 45.82 | 35.16 | 23.66 | 45.77 | 50.30 | 32.94 | 58.97 | 54.50 | 13.42 |
| Bleak | 1155 | 3 | 2 | 3 | 32.16 | 29.82 | 28.96 | 47.11 | 74.71 | 70.15 | 60.65 | 45.64 | 33.01 | 54.16 | 61.19 | 41.73 | 78.45 | 73.75 | 17.44 |
| Bleak | 1156 | 3 | 2 | 3 | 25.76 | 26.14 | 25.04 | 47.03 | 71.47 | 64.30 | 54.77 | 41.87 | 32.90 | 50.16 | 56.42 | 40.36 | 70.04 | 65.77 | 15.83 |
| Bleak | 1157 | 3 | 2 | 3 | 30.23 | 25.42 | 24.78 | 50.29 | 77.93 | 72.05 | 60.13 | 45.49 | 38.27 | 55.72 | 62.37 | 38.11 | 66.02 | 61.58 | 16.43 |
| Bleak | 1158 | 3 | 2 | 3 | 30.75 | 26.30 | 25.82 | 41.83 | 61.80 | 54.15 | 51.76 | 37.59 | 27.24 | 57.50 | 58.21 | 35.96 | 70.74 | 64.03 | 15.94 |
| Bleak | 1159 | 3 | 2 | 3 | 20.42 | 20.44 | 20.52 | 38.67 | 55.47 | 46.87 | 43.44 | 33.62 | 25.60 | 43.16 | 47.58 | 30.00 | 52.33 | 48.84 | 12.28 |
| Bleak | 1160 | 3 | 2 | 3 | 25.49 | 22.72 | 23.66 | 47.34 | 66.60 | 57.91 | 54.01 | 35.95 | 30.75 | 50.68 | 55.48 | 34.39 | 64.86 | 60.10 | 14.08 |
| Bleak | 1221 | 3 | 3 | 1 | 29.40 | 28.09 | 27.59 | 44.00 | 70.33 | 60.65 | 52.54 | 42.58 | 32.88 | 50.58 | 54.68 | 37.26 | 67.41 | 61.63 | 15.80 |
| Bleak | 1222 | 3 | 3 | 1 | 24.01 | 20.74 | 19.69 | 36.60 | 55.76 | 51.04 | 44.13 | 31.46 | 24.17 | 38.16 | 43.61 | 28.78 | 52.82 | 51.26 | 11.63 |
| Bleak | 1223 | 3 | 3 | 1 | 24.03 | 20.98 | 20.42 | 36.21 | 57.91 | 54.07 | 43.56 | 30.74 | 23.26 | 42.92 | 47.02 | 28.31 | 56.50 | 53.95 | 11.54 |
| Bleak | 1224 | 3 | 3 | 1 | 28.43 | 24.75 | 24.88 | 43.11 | 67.00 | 59.65 | 51.13 | 37.31 | 29.80 | 55.14 | 59.58 | 35.17 | 67.56 | 62.43 | 16.68 |
| Bleak | 1225 | 3 | 3 | 1 | 29.09 | 25.23 | 23.62 | 44.19 | 64.93 | 56.28 | 51.81 | 36.99 | 33.02 | 49.42 | 54.98 | 34.01 | 64.78 | 62.65 | 13.70 |
| Bleak | 1226 | 3 | 3 | 1 | 20.07 | 18.61 | 18.76 | 32.68 | 50.66 | 45.26 | 39.53 | 29.66 | 22.60 | 37.86 | 42.23 | 26.39 | 47.91 | 44.88 | 11.59 |
| Bleak | 1227 | 3 | 3 | 1 | 22.74 | 22.01 | 20.81 | 37.92 | 59.24 | 51.29 | 44.12 | 34.22 | 28.02 | 40.91 | 46.10 | 31.27 | 54.42 | 52.76 | 13.07 |
| Bleak | 1228 | 3 | 3 | 1 | 24.91 | 22.41 | 21.05 | 36.01 | 60.17 | 54.59 | 43.88 | 34.63 | 23.63 | 44.13 | 48.63 | 29.11 | 58.85 | 58.22 | 12.05 |
| Bleak | 1229 | 3 | 3 | 1 | 23.92 | 23.04 | 22.17 | 37.54 | 57.56 | 48.79 | 42.99 | 33.03 | 23.92 | 43.02 | 48.36 | 30.36 | 57.22 | 56.73 | 12.59 |
| Bleak | 1230 | 3 | 3 | 1 | 29.33 | 25.71 | 24.90 | 40.84 | 66.59 | 59.71 | 50.18 | 40.66 | 29.26 | 49.85 | 55.84 | 35.40 | 61.29 | 59.13 | 14.26 |
| Bleak | 1232 | 3 | 3 | 1 | 23.01 | 22.07 | 20.36 | 39.88 | 59.91 | 53.05 | 45.73 | 33.43 | 23.01 | 41.95 | 47.14 | 31.18 | 55.20 | 52.62 | 12.33 |
| Bleak | 1233 | 3 | 3 | 1 | 33.21 | 31.22 | 31.21 | 57.63 | 91.41 | 81.91 | 66.99 | 50.82 | 36.80 | 62.78 | 69.67 | 44.98 | 81.81 | 75.35 | 19.07 |
| Bleak | 1234 | 3 | 3 | 1 | 27.47 | 23.71 | 22.09 | 38.84 | 65.18 | 59.86 | 47.37 | 39.33 | 27.35 | 47.44 | 52.83 | 33.80 | 57.55 | 55.27 | 14.00 |
| Bleak | 1235 | 3 | 3 | 1 | 26.02 | 24.13 | 23.42 | 44.09 | 63.68 | 54.01 | 49.48 | 35.83 | 30.95 | 45.41 | 50.71 | 33.27 | 63.05 | 61.48 | 13.72 |
| Bleak | 1236 | 3 | 3 | 1 | 26.30 | 23.62 | 22.90 | 36.96 | 59.97 | 54.84 | 46.57 | 34.26 | 26.35 | 44.43 | 50.75 | 32.05 | 61.52 | 59.55 | 12.82 |
| Bleak | 1237 | 3 | 3 | 1 | 24.29 | 22.72 | 21.84 | 57.63 | 58.73 | 52.91 | 44.61 | 32.87 | 20.54 | 41.94 | 46.73 | 28.82 | 54.19 | 52.02 | 12.90 |
| Bleak | 1238 | 3 | 3 | 1 | 25.27 | 23.62 | 22.20 | 42.76 | 66.96 | 60.57 | 50.07 | 37.64 | 28.86 | 44.91 | 51.00 | 33.98 | 59.06 | 56.39 | 13.57 |
| Bleak | 1239 | 3 | 3 | 1 | 26.76 | 23.76 | 24.45 | 46.22 | 68.83 | 60.87 | 54.16 | 40.79 | 28.84 | 45.73 | 53.95 | 34.21 | 61.57 | 59.64 | 14.56 |
| Bleak | 1240 | 3 | 3 | 1 | 25.69 | 22.78 | 22.21 | 40.42 | 63.98 | 57.39 | 46.93 | 35.46 | 26.81 | 45.26 | 50.29 | 32.70 | 61.49 | 58.52 | 13.73 |
| Bleak | 1241 | 3 | 3 | 1 | 24.35 | 22.09 | 22.06 | 35.42 | 59.97 | 52.49 | 42.82 | 35.02 | 26.56 | 43.12 | 48.25 | 30.36 | 57.21 | 54.13 | 14.25 |
| Bleak | 1242 | 3 | 3 | 1 | 28.38 | 26.95 | 26.51 | 47.88 | 73.33 | 64.61 | 55.80 | 42.75 | 31.06 | 53.43 | 58.58 | 36.50 | 67.26 | 63.41 | 14.43 |
| Bleak | 1243 | 3 | 3 | 1 | 26.36 | 24.30 | 23.08 | 47.25 | 68.87 | 59.85 | 52.50 | 39.56 | 34.49 | 49.31 | 52.55 | 36.17 | 62.91 | 57.23 | 14.58 |
| Bleak | 1244 | 3 | 3 | 1 | 25.88 | 24.11 | 24.15 | 42.33 | 67.32 | 60.38 | 53.16 | 38.35 | 25.90 | 49.72 | 53.88 | 35.73 | 64.99 | 60.76 | 15.65 |
| Bleak | 1245 | 3 | 3 | 1 | 25.75 | 21.15 | 21.62 | 38.42 | 61.13 | 60.13 | 49.18 | 34.29 | 21.15 | 39.16 | 44.51 | 31.37 | 53.47 | 51.68 | 12.00 |
| Bleak | 1246 | 3 | 3 | 1 | 24.56 | 22.92 | 22.05 | 39.20 | 60.74 | 51.58 | 46.26 | 37.64 | 31.10 | 49.40 | 52.48 | 32.94 | 59.14 | 52.61 | 14.01 |
| Bleak | 1247 | 3 | 3 | 1 | 23.47 | 22.46 | 21.64 | 39.26 | 61.22 | 54.59 | 46.31 | 34.02 | 23.55 | 44.10 | 49.08 | 31.82 | 58.71 | 56.15 | 13.45 |
| Bleak | 1248 | 3 | 3 | 1 | 27.41 | 24.42 | 23.39 | 40.25 | 60.12 | 51.97 | 47.61 | 36.36 | 30.35 | 45.83 | 48.73 | 33.63 | 58.07 | 52.82 | 13.19 |
| Bleak | 1249 | 3 | 3 | 1 | 18.11 | 16.49 | 15.60 | 27.61 | 40.92 | 35.28 | 31.50 | 22.61 | 17.56 | 33.55 | 34.45 | 21.07 | 39.12 | 35.39 | 8.75 |
| Bleak | 1250 | 3 | 3 | 1 | 19.31 | 19.28 | 18.24 | 33.64 | 50.52 | 43.64 | 37.54 | 27.22 | 21.57 | 36.35 | 39.99 | 25.36 | 47.89 | 45.18 | 10.84 |
| Bleak | 1250 | 3 | 3 | 1 | 22.20 | 19.86 | 20.55 | 36.10 | 55.62 | 49.42 | 43.90 | 31.91 | 22.50 | 40.64 | 45.08 | 28.34 | 52.18 | 49.82 | 12.51 |
| Bleak | 1221 | 3 | 3 | 2 | 29.53 | 27.78 | 27.27 | 42.80 | 70.70 | 61.05 | 53.87 | 42.68 | 31.88 | 49.46 | 53.85 | 37.70 | 68.85 | 61.98 | 16.26 |
| Bleak | 1222 | 3 | 3 | 2 | 23.47 | 20.31 | 19.54 | 36.62 | 55.22 | 50.76 | 44.30 | 31.96 | 24.34 | 39.26 | 43.96 | 28.42 | 53.71 | 52.27 | 11.74 |
| Bleak | 1223 | 3 | 3 | 2 | 23.23 | 20.43 | 20.42 | 36.68 | 56.96 | 53.74 | 44.28 | 31.19 | 24.45 | 41.26 | 44.98 | 28.23 | 57.56 | 54.44 | 11.72 |
| Bleak | 1224 | 3 | 3 | 2 | 28.09 | 25.00 | 24.48 | 43.21 | 66.63 | 59.05 | 52.05 | 39.18 | 29.02 | 55.01 | 59.83 | 35.11 | 67.31 | 62.04 | 16.08 |
| Bleak | 1225 | 3 | 3 | 2 | 27.91 | 26.96 | 23.81 | 44.69 | 64.40 | 54.86 | 50.61 | 36.36 | 33.15 | 49.56 | 54.65 | 34.78 | 66.03 | 63.63 | 13.23 |
| Bleak | 1226 | 3 | 3 | 2 | 19.80 | 18.95 | 18.80 | 33.71 | 52.13 | 45.56 | 38.49 | 29.19 | 23.03 | 37.48 | 42.16 | 26.10 | 47.06 | 43.98 | 11.92 |
| Bleak | 1227 | 3 | 3 | 2 | 22.78 | 23.63 | 21.26 | 37.77 | 59.37 | 51.59 | 44.00 | 34.16 | 28.53 | 40.48 | 45.11 | 30.93 | 52.91 | 50.66 | 12.96 |
| Bleak | 1228 | 3 | 3 | 2 | 26.28 | 22.95 | 20.99 | 36.32 | 59.42 | 53.88 | 43.68 | 34.53 | 23.21 | 42.49 | 47.68 | 29.42 | 59.65 | 58.81 | 12.57 |
| Bleak | 1229 | 3 | 3 | 2 | 23.99 | 22.35 | 22.00 | 39.00 | 57.43 | 48.65 | 43.87 | 32.99 | 23.21 | 43.63 | 49.46 | 29.84 | 57.01 | 56.09 | 12.30 |
| Bleak | 1230 | 3 | 3 | 2 | 29.91 | 25.62 | 24.82 | 41.20 | 67.13 | 59.84 | 50.10 | 38.85 | 27.91 | 51.04 | 55.78 | 33.99 | 61.99 | 58.28 | 14.00 |
| Bleak | 1232 | 3 | 3 | 2 | 23.36 | 21.72 | 20.10 | 40.68 | 59.51 | 51.47 | 45.77 | 33.26 | 22.75 | 41.73 | 47.66 | 31.20 | 55.24 | 53.05 | 12.50 |
| Bleak | 1233 | 3 | 3 | 2 | 33.41 | 30.96 | 30.81 | 57.63 | 90.88 | 81.32 | 67.09 | 50.75 | 38.01 | 61.46 | 66.59 | 45.25 | 80.97 | 75.23 | 19.50 |
| Bleak | 1234 | 3 | 3 | 2 | 27.05 | 23.85 | 22.40 | 37.93 | 66.37 | 60.80 | 46.73 | 39.76 | 27.64 | 47.52 | 53.64 | 34.43 | 58.72 | 55.44 | 13.80 |
| Bleak | 1235 | 3 | 3 | 2 | 24.72 | 23.84 | 23.71 | 44.83 | 64.03 | 53.83 | 49.66 | 36.10 | 30.83 | 45.01 | 51.17 | 33.55 | 63.30 | 61.16 | 13.50 |
| Bleak | 1236 | 3 | 3 | 2 | 25.63 | 23.16 | 22.37 | 37.56 | 59.49 | 54.88 | 46.59 | 35.45 | 26.38 | 44.95 | 52.05 | 32.17 | 60.29 | 58.51 | 13.09 |
| Bleak | 1237 | 3 | 3 | 2 | 24.87 | 21.47 | 21.41 | 36.61 | 57.53 | 51.49 | 43.89 | 32.60 | 21.68 | 40.49 | 46.47 | 29.18 | 51.37 | 51.07 | 12.24 |
| Bleak | 1238 | 3 | 3 | 2 | 25.21 | 23.55 | 22.66 | 43.63 | 68.03 | 61.31 | 50.40 | 38.64 | 29.17 | 45.17 | 50.73 | 34.27 | 58.42 | 56.07 | 13.57 |
| Bleak | 1239 | 3 | 3 | 2 | 27.11 | 24.28 | 24.27 | 47.01 | 71.39 | 63.14 | 54.25 | 39.46 | 28.21 | 45.80 | 54.15 | 33.84 | 59.67 | 58.90 | 14.60 |
| Bleak | 1240 | 3 | 3 | 2 | 24.63 | 22.47 | 21.85 | 40.82 | 64.54 | 57.56 | 46.72 | 36.57 | 25.00 | 46.65 | 51.68 | 32.26 | 60.85 | 58.14 | 13.34 |
| Bleak | 1241 | 3 | 3 | 2 | 24.14 | 22.82 | 22.65 | 35.12 | 59.59 | 51.83 | 42.77 | 35.56 | 27.08 | 43.30 | 48.73 | 31.10 | 56.03 | 52.86 | 13.96 |
| Bleak | 1242 | 3 | 3 | 2 | 27.93 | 26.85 | 26.51 | 47.54 | 74.25 | 63.33 | 54.81 | 43.21 | 30.78 | 53.14 | 57.80 | 36.50 | 66.49 | 61.74 | 14.05 |
| Bleak | 1243 | 3 | 3 | 2 | 24.77 | 23.72 | 23.38 | 47.09 | 70.94 | 60.65 | 52.17 | 41.51 | 35.28 | 48.88 | 52.92 | 35.55 | 64.52 | 57.06 | 14.52 |
| Bleak | 1244 | 3 | 3 | 2 | 25.51 | 24.33 | 24.15 | 42.98 | 65.34 | 58.58 | 51.81 | 38.64 | 28.16 | 49.31 | 53.54 | 35.50 | 65.55 | 60.32 | 15.63 |
| Bleak | 1245 | 3 | 3 | 2 | 25.78 | 21.55 | 21.91 | 38.56 | 61.33 | 60.15 | 49.71 | 34.16 | 20.86 | 38.56 | 44.97 | 31.28 | 53.92 | 52.85 | 11.92 |
| Bleak | 1246 | 3 | 3 | 2 | 24.34 | 23.33 | 22.48 | 40.42 | 61.56 | 51.43 | 45.27 | 36.97 | 31.05 | 50.75 | 52.59 | 33.75 | 61.07 | 54.26 | 14.18 |
| Bleak | 1247 | 3 | 3 | 2 | 23.67 | 22.95 | 21.47 | 37.79 | 58.99 | 53.88 | 45.81 | 33.76 | 24.90 | 43.49 | 47.58 | 31.18 | 57.76 | 54.74 | 13.34 |
| Bleak | 1248 | 3 | 3 | 2 | 27.83 | 24.34 | 23.01 | 46.72 | 36.52 | 29.87 | 48.08 | 49.42 | 33.35 | 46.97 | 50.29 | 33.68 | 57.93 | 52.38 | 13.29 |
| Bleak | 1249 | 3 | 3 | 2 | 16.84 | 17.03 | 15.62 | 27.11 | 41.15 | 35.38 | 31.10 | 22.33 | 17.64 | 32.97 | 34.46 | 20.99 | 40.58 | 36.40 | 8.41 |
| Bleak | 1250 | 3 | 3 | 2 | 19.78 | 18.51 | 18.05 | 33.22 | 49.80 | 42.18 | 36.67 | 26.69 | 22.13 | 36.30 | 40.58 | 25.83 | 47.97 | 45.77 | 11.10 |
| Bleak | 1250 | 3 | 3 | 2 | 22.20 | 20.42 | 20.42 | 36.56 | 55.62 | 49.11 | 43.37 | 31.91 | 23.26 | 41.04 | 44.95 | 28.73 | 52.52 | 49.47 | 12.75 |
| Bleak | 1221 | 3 | 3 | 3 | 29.84 | 28.47 | 27.50 | 42.96 | 69.43 | 59.85 | 53.59 | 42.32 | 31.45 | 51.23 | 54.84 | 37.26 | 67.87 | 61.71 | 16.01 |
| Bleak | 1222 | 3 | 3 | 3 | 24.64 | 21.19 | 19.81 | 37.34 | 55.74 | 50.54 | 44.59 | 31.42 | 24.04 | 39.72 | 44.50 | 28.21 | 52.71 | 52.36 | 11.97 |
| Bleak | 1223 | 3 | 3 | 3 | 22.64 | 19.88 | 20.46 | 36.66 | 57.88 | 54.08 | 43.81 | 30.50 | 23.40 | 43.21 | 46.33 | 27.97 | 57.70 | 54.40 | 11.09 |
| Bleak | 1224 | 3 | 3 | 3 | 28.96 | 24.38 | 24.37 | 42.22 | 66.47 | 59.77 | 52.27 | 38.91 | 28.82 | 56.50 | 59.74 | 35.10 | 67.26 | 63.05 | 15.30 |
| Bleak | 1225 | 3 | 3 | 3 | 28.53 | 25.98 | 22.99 | 45.12 | 64.50 | 55.48 | 51.33 | 36.15 | 33.44 | 48.48 | 53.67 | 35.37 | 66.62 | 64.44 | 13.72 |
| Bleak | 1226 | 3 | 3 | 3 | 21.53 | 19.67 | 18.67 | 33.54 | 51.61 | 45.68 | 38.17 | 29.26 | 21.48 | 39.04 | 42.88 | 25.25 | 47.09 | 44.04 | 11.69 |
| Bleak | 1227 | 3 | 3 | 3 | 24.42 | 23.53 | 21.14 | 37.91 | 59.13 | 51.29 | 43.29 | 34.80 | 28.52 | 39.34 | 45.16 | 31.13 | 54.66 | 52.90 | 12.28 |
| Bleak | 1228 | 3 | 3 | 3 | 24.09 | 22.28 | 21.05 | 36.31 | 59.80 | 54.53 | 43.48 | 34.65 | 25.04 | 42.49 | 48.41 | 29.11 | 59.12 | 58.40 | 12.88 |
| Bleak | 1229 | 3 | 3 | 3 | 23.60 | 21.99 | 22.78 | 37.65 | 55.85 | 46.97 | 43.62 | 31.80 | 23.50 | 43.76 | 49.09 | 30.24 | 57.22 | 55.42 | 12.60 |
| Bleak | 1230 | 3 | 3 | 3 | 27.74 | 24.61 | 24.41 | 41.00 | 66.33 | 59.27 | 49.93 | 40.26 | 28.32 | 49.79 | 55.41 | 34.37 | 62.18 | 58.54 | 14.21 |
| Bleak | 1232 | 3 | 3 | 3 | 22.61 | 22.28 | 20.66 | 39.60 | 58.84 | 51.97 | 45.77 | 33.77 | 22.75 | 41.99 | 47.72 | 30.41 | 52.78 | 52.25 | 12.95 |
| Bleak | 1233 | 3 | 3 | 3 | 33.31 | 31.06 | 30.01 | 57.46 | 89.70 | 80.30 | 67.51 | 50.57 | 37.42 | 62.00 | 67.76 | 45.32 | 81.00 | 75.30 | 19.29 |
| Bleak | 1234 | 3 | 3 | 3 | 27.13 | 23.54 | 22.40 | 38.86 | 65.57 | 59.51 | 46.85 | 39.62 | 27.64 | 46.83 | 53.37 | 34.24 | 59.00 | 55.56 | 13.85 |
| Bleak | 1235 | 3 | 3 | 3 | 25.49 | 24.20 | 23.54 | 43.58 | 63.48 | 53.71 | 48.85 | 35.85 | 31.27 | 45.01 | 50.39 | 33.32 | 62.97 | 61.02 | 13.05 |
| Bleak | 1236 | 3 | 3 | 3 | 26.17 | 24.03 | 23.02 | 37.72 | 61.03 | 55.63 | 46.62 | 34.81 | 26.38 | 45.28 | 51.23 | 32.16 | 61.08 | 61.15 | 13.37 |
| Bleak | 1237 | 3 | 3 | 3 | 24.49 | 22.42 | 21.23 | 35.81 | 57.05 | 51.27 | 42.82 | 32.30 | 22.28 | 40.33 | 46.37 | 28.77 | 51.06 | 51.30 | 11.60 |
| Bleak | 1238 | 3 | 3 | 3 | 24.70 | 23.55 | 22.66 | 41.72 | 66.22 | 60.47 | 50.56 | 37.98 | 29.02 | 43.52 | 49.29 | 34.60 | 59.84 | 56.84 | 13.45 |
| Bleak | 1239 | 3 | 3 | 3 | 26.36 | 23.99 | 24.07 | 47.79 | 70.55 | 62.11 | 53.97 | 39.10 | 28.11 | 46.57 | 53.72 | 34.68 | 61.07 | 59.63 | 14.04 |
| Bleak | 1240 | 3 | 3 | 3 | 25.13 | 22.63 | 21.61 | 39.51 | 62.28 | 55.84 | 46.31 | 35.56 | 25.96 | 45.70 | 50.38 | 32.48 | 61.53 | 58.91 | 13.45 |
| Bleak | 1241 | 3 | 3 | 3 | 23.63 | 22.03 | 22.20 | 36.16 | 60.44 | 52.96 | 42.87 | 34.69 | 26.18 | 44.14 | 49.33 | 30.64 | 57.92 | 54.46 | 14.26 |
| Bleak | 1242 | 3 | 3 | 3 | 28.09 | 26.83 | 26.10 | 48.38 | 73.98 | 64.01 | 54.71 | 43.39 | 30.55 | 54.34 | 58.12 | 36.05 | 66.35 | 61.72 | 14.14 |
| Bleak | 1243 | 3 | 3 | 3 | 25.90 | 24.56 | 23.30 | 47.62 | 71.04 | 61.08 | 51.85 | 40.71 | 35.05 | 50.49 | 52.45 | 35.51 | 62.62 | 57.03 | 13.98 |
| Bleak | 1244 | 3 | 3 | 3 | 26.25 | 24.85 | 23.82 | 42.23 | 65.10 | 57.56 | 51.26 | 39.00 | 28.30 | 50.09 | 54.57 | 35.47 | 66.91 | 61.36 | 15.48 |
| Bleak | 1245 | 3 | 3 | 3 | 24.71 | 21.22 | 21.87 | 38.56 | 61.13 | 59.49 | 48.91 | 34.35 | 21.60 | 38.70 | 44.16 | 31.52 | 54.71 | 53.35 | 11.72 |
| Bleak | 1246 | 3 | 3 | 3 | 24.77 | 23.04 | 23.13 | 40.04 | 61.01 | 51.86 | 46.77 | 37.57 | 29.60 | 50.57 | 52.42 | 33.15 | 60.57 | 53.96 | 14.07 |
| Bleak | 1247 | 3 | 3 | 3 | 24.80 | 22.55 | 21.60 | 38.06 | 60.48 | 55.17 | 46.38 | 33.37 | 24.40 | 44.86 | 48.84 | 31.75 | 59.14 | 56.93 | 13.19 |
| Bleak | 1248 | 3 | 3 | 3 | 27.77 | 23.94 | 22.89 | 41.37 | 63.12 | 53.50 | 47.42 | 36.88 | 30.06 | 46.50 | 47.69 | 33.82 | 58.95 | 52.82 | 13.04 |
| Bleak | 1249 | 3 | 3 | 3 | 18.10 | 17.35 | 15.65 | 27.30 | 40.81 | 34.59 | 31.20 | 22.65 | 17.81 | 32.74 | 33.83 | 21.10 | 40.09 | 36.42 | 8.43 |
| Bleak | 1250 | 3 | 3 | 3 | 19.76 | 18.81 | 18.32 | 32.89 | 50.74 | 43.56 | 36.88 | 27.40 | 21.72 | 35.79 | 39.67 | 24.96 | 47.60 | 44.12 | 10.85 |
| Bleak | 1250 | 3 | 3 | 3 | 21.28 | 20.84 | 20.35 | 37.81 | 55.89 | 48.81 | 43.37 | 32.35 | 23.66 | 40.42 | 44.12 | 28.95 | 52.00 | 48.50 | 12.31 |
| Roach | 1001 | 1 | 1 | 1 | 16.41 | 12.73 | 15.96 | 31.14 | 40.92 | 33.93 | 37.93 | 31.17 | 24.86 | 29.06 | 31.05 | 33.92 | 50.48 | 44.80 | 9.96 |
| Roach | 1002 | 1 | 1 | 1 | 20.85 | 20.19 | 23.01 | 42.52 | 55.20 | 42.45 | 50.55 | 39.38 | 30.90 | 34.61 | 38.85 | 45.67 | 63.70 | 56.65 | 14.03 |
| Roach | 1003 | 1 | 1 | 1 | 24.26 | 25.15 | 26.79 | 48.21 | 63.50 | 52.59 | 57.30 | 47.72 | 32.58 | 34.83 | 42.62 | 52.88 | 76.09 | 71.94 | 15.36 |
| Roach | 1004 | 1 | 1 | 1 | 15.13 | 16.14 | 17.39 | 29.74 | 36.13 | 27.83 | 36.47 | 28.11 | 18.18 | 24.03 | 27.77 | 33.23 | 48.28 | 45.75 | 10.47 |
| Roach | 1005 | 1 | 1 | 1 | 19.07 | 20.98 | 22.18 | 44.20 | 57.00 | 46.90 | 52.49 | 41.08 | 29.68 | 28.81 | 36.33 | 46.01 | 61.54 | 57.41 | 14.41 |
| Roach | 1006 | 1 | 1 | 1 | 25.92 | 25.91 | 27.67 | 47.99 | 61.88 | 50.16 | 59.01 | 46.67 | 34.11 | 35.56 | 45.74 | 53.75 | 72.20 | 68.68 | 16.32 |
| Roach | 1007 | 1 | 1 | 1 | 17.06 | 18.03 | 17.79 | 32.49 | 43.53 | 33.09 | 37.45 | 30.94 | 21.07 | 24.05 | 28.07 | 35.11 | 50.46 | 48.56 | 12.27 |
| Roach | 1008 | 1 | 1 | 1 | 16.14 | 14.31 | 16.18 | 27.62 | 34.39 | 27.22 | 33.39 | 26.96 | 19.34 | 21.98 | 26.86 | 30.99 | 44.59 | 40.85 | 10.28 |
| Roach | 1009 | 1 | 1 | 1 | 18.19 | 17.17 | 18.55 | 34.00 | 41.74 | 32.76 | 38.91 | 30.27 | 21.63 | 25.43 | 30.54 | 33.89 | 48.62 | 45.11 | 10.80 |
| Roach | 1010 | 1 | 1 | 1 | 18.80 | 17.97 | 19.49 | 34.95 | 42.97 | 33.33 | 41.72 | 31.98 | 20.82 | 25.83 | 30.27 | 35.22 | 49.46 | 45.00 | 11.33 |
| Roach | 1011 | 1 | 1 | 1 | 16.18 | 14.75 | 17.02 | 30.36 | 35.71 | 27.78 | 34.82 | 27.36 | 18.25 | 26.11 | 28.94 | 29.91 | 45.43 | 41.48 | 9.67 |
| Roach | 1012 | 1 | 1 | 1 | 18.65 | 17.63 | 19.38 | 38.29 | 47.86 | 38.69 | 43.95 | 33.55 | 25.32 | 29.20 | 34.74 | 37.45 | 54.14 | 48.38 | 11.56 |
| Roach | 1013 | 1 | 1 | 1 | 16.75 | 16.87 | 17.84 | 32.23 | 40.27 | 31.71 | 39.72 | 30.47 | 18.78 | 26.87 | 31.87 | 34.39 | 51.61 | 49.06 | 10.79 |
| Roach | 1014 | 1 | 1 | 1 | 26.31 | 24.28 | 28.03 | 44.18 | 57.37 | 46.89 | 55.76 | 43.46 | 33.23 | 38.31 | 46.37 | 49.65 | 70.91 | 66.04 | 14.49 |
| Roach | 1015 | 1 | 1 | 1 | 26.36 | 24.67 | 27.92 | 43.07 | 61.56 | 47.74 | 53.46 | 44.03 | 32.84 | 37.03 | 43.26 | 50.31 | 73.25 | 70.20 | 15.47 |
| Roach | 1016 | 1 | 1 | 1 | 17.55 | 17.20 | 18.96 | 31.70 | 42.89 | 34.46 | 39.64 | 32.19 | 18.68 | 26.13 | 30.05 | 34.79 | 49.09 | 45.22 | 11.04 |
| Roach | 1017 | 1 | 1 | 1 | 14.19 | 12.98 | 13.94 | 25.92 | 31.92 | 25.28 | 29.88 | 23.32 | 16.05 | 22.81 | 25.14 | 27.11 | 41.70 | 39.03 | 8.26 |
| Roach | 1018 | 1 | 1 | 1 | 21.34 | 21.51 | 23.05 | 38.78 | 50.35 | 39.42 | 46.47 | 37.48 | 26.89 | 30.70 | 36.53 | 42.62 | 62.32 | 58.41 | 12.53 |
| Roach | 1019 | 1 | 1 | 1 | 18.49 | 16.73 | 18.72 | 32.49 | 43.74 | 36.66 | 39.77 | 31.87 | 23.02 | 24.49 | 30.42 | 34.45 | 50.79 | 48.62 | 11.48 |
| Roach | 1020 | 1 | 1 | 1 | 21.48 | 20.74 | 23.79 | 42.65 | 56.37 | 47.37 | 52.82 | 40.80 | 29.73 | 31.11 | 39.54 | 44.70 | 62.13 | 59.96 | 14.77 |
| Roach | 1021 | 1 | 1 | 1 | 19.20 | 18.75 | 20.07 | 39.20 | 52.89 | 44.56 | 47.51 | 36.66 | 26.08 | 30.89 | 39.66 | 40.80 | 59.09 | 56.94 | 13.46 |
| Roach | 1022 | 1 | 1 | 1 | 20.53 | 17.95 | 19.59 | 33.41 | 42.94 | 34.57 | 39.90 | 30.82 | 22.87 | 25.11 | 30.61 | 33.86 | 50.08 | 47.26 | 11.05 |
| Roach | 1023 | 1 | 1 | 1 | 16.43 | 16.13 | 16.17 | 27.24 | 36.45 | 29.26 | 32.77 | 24.86 | 21.21 | 23.51 | 27.43 | 29.13 | 44.12 | 41.83 | 8.29 |
| Roach | 1024 | 1 | 1 | 1 | 21.27 | 20.21 | 21.32 | 32.17 | 43.20 | 32.20 | 40.55 | 33.82 | 22.51 | 27.82 | 33.11 | 37.14 | 54.91 | 52.07 | 12.64 |
| Roach | 1025 | 1 | 1 | 1 | 17.76 | 16.95 | 19.51 | 34.35 | 43.10 | 35.60 | 42.92 | 33.89 | 21.98 | 28.69 | 34.35 | 36.19 | 52.30 | 48.25 | 11.22 |
| Roach | 1026 | 1 | 1 | 1 | 22.81 | 23.30 | 24.65 | 42.76 | 59.03 | 50.34 | 53.68 | 40.95 | 26.81 | 29.03 | 37.34 | 44.78 | 65.40 | 65.60 | 13.92 |
| Roach | 1027 | 1 | 1 | 1 | 20.70 | 18.18 | 20.60 | 37.33 | 47.05 | 38.98 | 46.08 | 34.82 | 26.43 | 29.22 | 33.53 | 38.30 | 52.01 | 47.62 | 11.43 |
| Roach | 1028 | 1 | 1 | 1 | 20.44 | 22.55 | 23.00 | 43.07 | 55.59 | 43.35 | 48.87 | 37.83 | 29.69 | 33.63 | 39.49 | 44.96 | 67.73 | 61.21 | 13.92 |
| Roach | 1029 | 1 | 1 | 1 | 19.19 | 18.28 | 21.34 | 38.90 | 52.13 | 43.67 | 47.10 | 35.75 | 25.48 | 27.22 | 33.96 | 40.52 | 57.28 | 57.19 | 11.82 |
| Roach | 1030 | 1 | 1 | 1 | 16.55 | 20.32 | 20.37 | 33.86 | 46.79 | 39.00 | 41.22 | 33.41 | 23.76 | 26.53 | 32.02 | 38.09 | 52.97 | 50.16 | 11.53 |
| Roach | 1001 | 1 | 1 | 2 | 15.74 | 15.25 | 16.64 | 30.49 | 42.66 | 33.97 | 36.69 | 31.29 | 24.05 | 28.09 | 31.39 | 35.60 | 50.86 | 45.77 | 10.87 |
| Roach | 1002 | 1 | 1 | 2 | 21.30 | 22.89 | 23.99 | 42.86 | 55.46 | 43.96 | 51.03 | 39.64 | 31.27 | 33.26 | 38.96 | 45.89 | 63.44 | 57.58 | 13.99 |
| Roach | 1003 | 1 | 1 | 2 | 22.87 | 26.54 | 27.51 | 48.56 | 64.74 | 51.12 | 57.57 | 46.94 | 31.54 | 34.39 | 43.79 | 52.88 | 75.49 | 72.02 | 14.75 |
| Roach | 1004 | 1 | 1 | 2 | 15.16 | 15.87 | 16.50 | 31.19 | 27.24 | 27.97 | 36.22 | 27.60 | 18.84 | 23.79 | 28.08 | 32.31 | 47.03 | 44.21 | 10.26 |
| Roach | 1005 | 1 | 1 | 2 | 19.36 | 20.34 | 22.18 | 43.12 | 54.27 | 44.46 | 51.75 | 38.88 | 28.61 | 29.89 | 37.28 | 45.83 | 61.80 | 56.66 | 14.48 |
| Roach | 1006 | 1 | 1 | 2 | 25.39 | 26.34 | 27.67 | 46.69 | 61.76 | 49.80 | 57.60 | 46.45 | 34.44 | 35.90 | 46.19 | 52.98 | 70.66 | 67.03 | 15.93 |
| Roach | 1007 | 1 | 1 | 2 | 15.99 | 18.14 | 18.45 | 32.02 | 45.18 | 37.73 | 39.01 | 31.09 | 20.34 | 23.85 | 30.23 | 35.39 | 51.08 | 48.91 | 11.52 |
| Roach | 1008 | 1 | 1 | 2 | 15.17 | 14.15 | 15.81 | 24.91 | 33.63 | 27.16 | 33.46 | 26.58 | 18.71 | 22.21 | 27.23 | 30.32 | 44.11 | 41.58 | 10.26 |
| Roach | 1009 | 1 | 1 | 2 | 16.34 | 15.87 | 18.03 | 34.01 | 41.66 | 33.75 | 39.32 | 29.23 | 21.34 | 25.07 | 31.18 | 34.01 | 49.18 | 46.41 | 11.82 |
| Roach | 1010 | 1 | 1 | 2 | 19.84 | 18.45 | 20.06 | 34.71 | 43.21 | 32.92 | 40.67 | 31.26 | 20.64 | 27.05 | 32.04 | 33.97 | 47.57 | 42.50 | 11.24 |
| Roach | 1011 | 1 | 1 | 2 | 15.82 | 14.83 | 17.00 | 29.73 | 35.79 | 30.20 | 34.80 | 27.89 | 19.35 | 25.91 | 29.35 | 30.59 | 46.38 | 43.34 | 9.95 |
| Roach | 1012 | 1 | 1 | 2 | 18.15 | 19.43 | 19.93 | 36.78 | 46.41 | 36.53 | 42.97 | 33.58 | 23.84 | 31.31 | 35.33 | 36.66 | 51.38 | 48.18 | 10.97 |
| Roach | 1013 | 1 | 1 | 2 | 16.18 | 16.99 | 18.07 | 33.37 | 41.05 | 33.80 | 40.01 | 29.88 | 19.91 | 27.97 | 33.46 | 33.20 | 49.60 | 47.76 | 10.88 |
| Roach | 1014 | 1 | 1 | 2 | 24.96 | 24.18 | 26.90 | 44.96 | 56.61 | 45.97 | 57.68 | 43.29 | 31.82 | 38.78 | 45.93 | 48.86 | 69.08 | 63.12 | 14.71 |
| Roach | 1015 | 1 | 1 | 2 | 26.63 | 25.54 | 27.78 | 43.86 | 60.12 | 47.73 | 54.86 | 43.53 | 32.23 | 36.17 | 44.24 | 50.89 | 73.66 | 69.47 | 16.21 |
| Roach | 1016 | 1 | 1 | 2 | 17.50 | 16.69 | 18.34 | 32.51 | 42.71 | 34.05 | 39.28 | 32.05 | 18.18 | 25.26 | 30.40 | 33.96 | 47.15 | 44.28 | 10.58 |
| Roach | 1017 | 1 | 1 | 2 | 14.39 | 13.41 | 14.48 | 24.81 | 32.93 | 26.18 | 30.57 | 24.41 | 16.52 | 21.77 | 23.70 | 27.07 | 41.67 | 38.04 | 8.49 |
| Roach | 1018 | 1 | 1 | 2 | 20.30 | 20.72 | 22.76 | 37.99 | 51.12 | 39.32 | 46.17 | 37.78 | 27.40 | 28.66 | 36.99 | 43.18 | 61.09 | 59.78 | 12.53 |
| Roach | 1019 | 1 | 1 | 2 | 17.80 | 16.88 | 18.47 | 34.83 | 44.98 | 37.42 | 40.19 | 32.18 | 22.36 | 25.96 | 31.47 | 34.99 | 51.63 | 48.68 | 11.88 |
| Roach | 1020 | 1 | 1 | 2 | 22.51 | 22.15 | 24.10 | 42.37 | 56.37 | 45.18 | 51.85 | 41.19 | 30.48 | 31.44 | 38.54 | 46.76 | 64.32 | 60.94 | 13.84 |
| Roach | 1021 | 1 | 1 | 2 | 19.75 | 20.02 | 21.61 | 39.37 | 52.48 | 44.15 | 47.86 | 36.35 | 25.43 | 31.30 | 38.80 | 41.08 | 57.98 | 56.03 | 13.14 |
| Roach | 1022 | 1 | 1 | 2 | 20.01 | 18.10 | 19.93 | 32.00 | 42.87 | 34.45 | 38.98 | 30.41 | 23.41 | 24.92 | 30.40 | 33.33 | 48.75 | 46.46 | 10.44 |
| Roach | 1023 | 1 | 1 | 2 | 15.96 | 15.52 | 16.80 | 25.40 | 35.25 | 29.37 | 33.61 | 24.84 | 21.04 | 23.10 | 27.52 | 28.79 | 41.59 | 39.53 | 8.93 |
| Roach | 1024 | 1 | 1 | 2 | 19.46 | 19.83 | 21.48 | 31.35 | 42.66 | 31.88 | 39.67 | 34.06 | 23.44 | 27.63 | 33.02 | 38.05 | 55.95 | 52.04 | 12.61 |
| Roach | 1025 | 1 | 1 | 2 | 17.14 | 16.45 | 18.62 | 33.78 | 43.89 | 36.13 | 42.18 | 33.28 | 22.02 | 27.02 | 33.09 | 36.22 | 54.44 | 49.24 | 12.13 |
| Roach | 1026 | 1 | 1 | 2 | 24.18 | 22.32 | 23.86 | 43.53 | 59.13 | 50.49 | 53.61 | 41.21 | 26.58 | 28.92 | 37.91 | 44.94 | 66.70 | 65.46 | 13.78 |
| Roach | 1027 | 1 | 1 | 2 | 20.77 | 18.45 | 20.77 | 37.88 | 47.58 | 40.41 | 46.30 | 34.35 | 26.20 | 30.71 | 33.72 | 38.30 | 53.02 | 48.25 | 11.04 |
| Roach | 1028 | 1 | 1 | 2 | 20.51 | 21.08 | 21.69 | 42.14 | 54.15 | 42.61 | 48.24 | 37.47 | 29.42 | 33.84 | 39.88 | 43.86 | 66.12 | 60.78 | 13.46 |
| Roach | 1029 | 1 | 1 | 2 | 19.05 | 17.20 | 20.74 | 38.58 | 50.28 | 43.51 | 47.69 | 34.98 | 25.76 | 26.71 | 33.02 | 40.96 | 58.67 | 56.43 | 12.67 |
| Roach | 1030 | 1 | 1 | 2 | 17.68 | 17.97 | 19.53 | 32.34 | 45.50 | 38.87 | 42.51 | 33.63 | 23.64 | 27.00 | 33.24 | 34.24 | 47.93 | 44.35 | 11.35 |
| Roach | 1001 | 1 | 1 | 3 | 16.47 | 17.87 | 17.60 | 30.97 | 41.36 | 33.78 | 36.81 | 30.66 | 23.89 | 28.60 | 31.97 | 35.22 | 46.30 | 51.58 | 10.67 |
| Roach | 1002 | 1 | 1 | 3 | 22.42 | 22.53 | 24.41 | 43.11 | 54.78 | 44.75 | 50.37 | 39.81 | 30.85 | 33.66 | 40.58 | 43.16 | 62.66 | 56.37 | 13.54 |
| Roach | 1003 | 1 | 1 | 3 | 22.87 | 24.06 | 25.63 | 47.02 | 62.96 | 52.50 | 59.67 | 46.96 | 29.52 | 35.34 | 43.44 | 53.07 | 76.22 | 73.49 | 14.77 |
| Roach | 1004 | 1 | 1 | 3 | 15.45 | 16.81 | 17.25 | 30.76 | 37.96 | 29.42 | 37.65 | 27.58 | 16.80 | 24.39 | 28.89 | 31.46 | 47.30 | 44.14 | 10.41 |
| Roach | 1005 | 1 | 1 | 3 | 18.30 | 19.68 | 21.14 | 43.65 | 57.00 | 45.95 | 52.12 | 38.70 | 27.34 | 29.30 | 37.78 | 45.11 | 60.27 | 56.83 | 14.61 |
| Roach | 1006 | 1 | 1 | 3 | 24.34 | 25.26 | 26.28 | 48.84 | 61.47 | 49.09 | 58.72 | 46.53 | 33.21 | 33.79 | 45.54 | 53.81 | 68.82 | 66.22 | 15.70 |
| Roach | 1007 | 1 | 1 | 3 | 15.22 | 16.52 | 17.50 | 30.75 | 42.63 | 33.97 | 37.24 | 30.29 | 18.61 | 24.47 | 29.52 | 33.61 | 51.28 | 47.23 | 10.93 |
| Roach | 1008 | 1 | 1 | 3 | 15.40 | 14.38 | 15.22 | 25.87 | 34.07 | 25.71 | 32.01 | 26.23 | 18.33 | 21.18 | 25.98 | 28.94 | 41.61 | 38.48 | 9.65 |
| Roach | 1009 | 1 | 1 | 3 | 17.10 | 16.65 | 17.50 | 32.36 | 40.90 | 32.27 | 38.00 | 28.03 | 21.40 | 24.45 | 28.86 | 33.28 | 48.05 | 44.67 | 11.00 |
| Roach | 1010 | 1 | 1 | 3 | 18.03 | 16.93 | 18.39 | 33.57 | 42.28 | 32.28 | 39.21 | 31.20 | 20.63 | 26.22 | 31.03 | 34.22 | 49.02 | 44.10 | 11.42 |
| Roach | 1011 | 1 | 1 | 3 | 16.03 | 13.91 | 17.20 | 26.09 | 35.78 | 28.40 | 33.94 | 26.98 | 17.26 | 25.77 | 28.34 | 27.85 | 44.98 | 42.28 | 10.55 |
| Roach | 1012 | 1 | 1 | 3 | 17.98 | 18.34 | 19.01 | 37.20 | 45.21 | 35.54 | 42.03 | 32.56 | 22.77 | 30.62 | 34.65 | 36.20 | 51.52 | 47.90 | 11.41 |
| Roach | 1013 | 1 | 1 | 3 | 17.13 | 15.85 | 18.19 | 31.83 | 40.41 | 32.85 | 37.94 | 28.94 | 20.22 | 27.31 | 31.89 | 31.68 | 48.75 | 46.04 | 10.10 |
| Roach | 1014 | 1 | 1 | 3 | 25.32 | 22.75 | 26.22 | 43.57 | 59.24 | 48.39 | 55.74 | 41.33 | 28.57 | 39.08 | 45.77 | 46.50 | 65.79 | 60.75 | 14.65 |
| Roach | 1015 | 1 | 1 | 3 | 23.12 | 24.77 | 27.22 | 42.52 | 59.93 | 47.88 | 53.30 | 42.52 | 30.70 | 35.40 | 43.68 | 47.33 | 70.25 | 66.97 | 14.62 |
| Roach | 1016 | 1 | 1 | 3 | 17.01 | 15.35 | 17.33 | 31.39 | 41.00 | 32.32 | 38.16 | 31.39 | 17.89 | 24.23 | 29.55 | 32.26 | 46.15 | 42.06 | 10.59 |
| Roach | 1017 | 1 | 1 | 3 | 13.76 | 13.21 | 13.65 | 25.42 | 31.30 | 25.15 | 29.30 | 22.47 | 15.41 | 22.49 | 23.78 | 25.17 | 40.23 | 37.22 | 7.38 |
| Roach | 1018 | 1 | 1 | 3 | 19.28 | 19.70 | 21.73 | 37.46 | 49.88 | 38.46 | 46.25 | 36.71 | 25.06 | 30.75 | 37.00 | 41.18 | 58.80 | 56.57 | 12.30 |
| Roach | 1019 | 1 | 1 | 3 | 16.38 | 16.03 | 17.69 | 29.69 | 42.52 | 36.39 | 38.70 | 30.60 | 20.91 | 24.89 | 31.00 | 32.57 | 47.61 | 45.63 | 10.90 |
| Roach | 1020 | 1 | 1 | 3 | 20.71 | 20.62 | 22.77 | 42.09 | 55.47 | 45.31 | 50.73 | 40.32 | 29.42 | 29.35 | 38.84 | 44.06 | 62.16 | 58.25 | 13.45 |
| Roach | 1021 | 1 | 1 | 3 | 18.64 | 18.63 | 20.22 | 38.41 | 51.31 | 43.28 | 46.56 | 35.80 | 25.58 | 30.20 | 37.37 | 40.04 | 57.02 | 54.13 | 12.77 |
| Roach | 1022 | 1 | 1 | 3 | 18.44 | 17.32 | 18.85 | 31.67 | 41.12 | 33.16 | 38.52 | 28.82 | 22.55 | 23.43 | 28.55 | 33.19 | 48.54 | 44.85 | 10.27 |
| Roach | 1023 | 1 | 1 | 3 | 15.59 | 15.43 | 15.55 | 26.56 | 35.36 | 28.45 | 32.06 | 24.38 | 20.25 | 23.49 | 27.61 | 27.98 | 41.92 | 38.34 | 8.65 |
| Roach | 1024 | 1 | 1 | 3 | 19.76 | 19.91 | 20.43 | 30.71 | 41.77 | 31.42 | 39.98 | 32.21 | 20.63 | 27.30 | 32.73 | 36.51 | 53.51 | 50.75 | 12.21 |
| Roach | 1025 | 1 | 1 | 3 | 17.06 | 16.98 | 19.36 | 33.09 | 44.39 | 36.04 | 40.75 | 32.86 | 21.46 | 26.21 | 32.13 | 35.27 | 50.26 | 47.22 | 11.01 |
| Roach | 1026 | 1 | 1 | 3 | 22.08 | 21.01 | 23.73 | 40.56 | 55.97 | 48.19 | 51.70 | 39.96 | 26.61 | 28.75 | 36.26 | 43.38 | 64.15 | 63.25 | 13.97 |
| Roach | 1027 | 1 | 1 | 3 | 21.44 | 18.54 | 20.07 | 34.74 | 45.75 | 39.26 | 45.10 | 34.06 | 25.77 | 28.81 | 33.62 | 36.81 | 50.76 | 46.05 | 11.35 |
| Roach | 1028 | 1 | 1 | 3 | 19.55 | 21.07 | 22.04 | 40.54 | 53.32 | 42.04 | 47.48 | 37.12 | 27.89 | 34.07 | 39.14 | 41.99 | 63.67 | 59.04 | 14.05 |
| Roach | 1029 | 1 | 1 | 3 | 18.40 | 18.37 | 20.61 | 37.86 | 49.58 | 42.21 | 45.68 | 34.51 | 25.05 | 25.89 | 33.44 | 38.90 | 56.28 | 54.42 | 11.88 |
| Roach | 1030 | 1 | 1 | 3 | 17.25 | 19.54 | 20.35 | 33.35 | 45.97 | 38.12 | 40.82 | 33.15 | 22.49 | 26.37 | 31.75 | 33.36 | 46.19 | 42.74 | 11.00 |
| Roach | 1101 | 1 | 2 | 1 | 33.58 | 28.95 | 34.35 | 69.49 | 90.06 | 75.99 | 81.59 | 64.83 | 46.38 | 48.84 | 62.55 | 74.37 | 104.87 | 100.51 | 22.43 |
| Roach | 1102 | 1 | 2 | 1 | 31.68 | 32.23 | 32.34 | 57.75 | 73.07 | 60.40 | 72.46 | 51.24 | 37.12 | 46.91 | 55.76 | 58.16 | 88.40 | 83.74 | 17.19 |
| Roach | 1103 | 1 | 2 | 1 | 33.58 | 30.61 | 33.77 | 58.64 | 76.56 | 64.43 | 74.04 | 55.02 | 45.14 | 40.53 | 51.79 | 62.67 | 88.33 | 82.40 | 20.23 |
| Roach | 1104 | 1 | 2 | 1 | 34.24 | 34.32 | 38.04 | 59.33 | 77.68 | 58.94 | 73.57 | 58.58 | 44.80 | 47.32 | 55.78 | 66.19 | 93.16 | 89.69 | 18.51 |
| Roach | 1105 | 1 | 2 | 1 | 30.62 | 31.29 | 33.32 | 60.38 | 77.83 | 60.75 | 70.40 | 54.25 | 39.49 | 46.13 | 52.00 | 60.11 | 89.43 | 83.13 | 18.09 |
| Roach | 1106 | 1 | 2 | 1 | 24.24 | 21.71 | 25.02 | 42.52 | 55.64 | 44.40 | 52.45 | 40.14 | 31.16 | 39.36 | 42.56 | 46.32 | 70.91 | 62.07 | 13.46 |
| Roach | 1107 | 1 | 2 | 1 | 29.89 | 30.23 | 30.74 | 55.45 | 68.89 | 51.83 | 63.49 | 48.80 | 40.19 | 45.79 | 51.98 | 56.11 | 81.50 | 76.34 | 17.25 |
| Roach | 1108 | 1 | 2 | 1 | 28.02 | 26.47 | 29.27 | 54.73 | 69.69 | 58.33 | 66.30 | 48.83 | 37.07 | 40.33 | 46.96 | 53.65 | 78.86 | 75.08 | 15.38 |
| Roach | 1109 | 1 | 2 | 1 | 32.38 | 31.88 | 34.78 | 52.04 | 71.82 | 59.10 | 68.21 | 53.95 | 40.57 | 39.80 | 49.36 | 60.02 | 82.72 | 76.86 | 17.79 |
| Roach | 1110 | 1 | 2 | 1 | 25.18 | 20.09 | 22.65 | 44.75 | 57.93 | 52.90 | 57.28 | 41.32 | 32.46 | 37.51 | 44.72 | 44.27 | 70.25 | 64.32 | 14.78 |
| Roach | 1111 | 1 | 2 | 1 | 26.62 | 28.34 | 28.64 | 46.04 | 59.51 | 46.23 | 57.95 | 46.46 | 34.83 | 41.08 | 49.41 | 52.11 | 79.59 | 74.43 | 15.48 |
| Roach | 1112 | 1 | 2 | 1 | 26.17 | 25.02 | 25.32 | 44.67 | 54.57 | 43.86 | 51.44 | 37.98 | 33.05 | 31.45 | 40.40 | 44.33 | 67.45 | 63.34 | 15.07 |
| Roach | 1113 | 1 | 2 | 1 | 32.52 | 31.27 | 31.72 | 52.33 | 69.26 | 53.73 | 64.30 | 50.79 | 36.42 | 41.38 | 50.49 | 54.98 | 80.88 | 76.47 | 16.35 |
| Roach | 1114 | 1 | 2 | 1 | 23.33 | 22.92 | 23.26 | 42.37 | 52.87 | 39.69 | 46.60 | 37.25 | 26.39 | 33.77 | 40.08 | 41.89 | 66.11 | 61.78 | 13.73 |
| Roach | 1115 | 1 | 2 | 1 | 28.85 | 24.67 | 28.25 | 47.04 | 62.45 | 52.62 | 59.46 | 44.93 | 30.23 | 37.27 | 46.25 | 51.92 | 74.21 | 70.99 | 15.97 |
| Roach | 1116 | 1 | 2 | 1 | 20.04 | 20.54 | 21.41 | 38.23 | 48.24 | 36.23 | 45.77 | 34.41 | 25.68 | 34.13 | 39.04 | 40.03 | 62.02 | 56.45 | 12.07 |
| Roach | 1117 | 1 | 2 | 1 | 23.77 | 21.31 | 25.62 | 47.53 | 60.58 | 47.37 | 56.55 | 43.10 | 32.17 | 31.36 | 39.88 | 47.08 | 67.81 | 64.02 | 16.41 |
| Roach | 1118 | 1 | 2 | 1 | 21.77 | 25.15 | 26.61 | 43.51 | 57.24 | 47.01 | 52.17 | 39.65 | 30.99 | 35.97 | 41.89 | 46.81 | 70.72 | 66.32 | 15.70 |
| Roach | 1119 | 1 | 2 | 1 | 27.01 | 21.45 | 25.16 | 45.81 | 57.27 | 50.78 | 59.44 | 43.44 | 31.14 | 38.34 | 46.23 | 44.58 | 69.50 | 65.43 | 15.07 |
| Roach | 1120 | 1 | 2 | 1 | 21.35 | 19.56 | 19.63 | 35.37 | 44.81 | 36.35 | 44.48 | 30.89 | 26.07 | 32.97 | 38.82 | 38.58 | 63.67 | 60.08 | 13.29 |
| Roach | 1121 | 1 | 2 | 1 | 26.44 | 24.83 | 24.30 | 43.14 | 54.61 | 42.77 | 49.36 | 35.27 | 26.91 | 34.74 | 39.93 | 43.07 | 68.23 | 64.19 | 13.32 |
| Roach | 1122 | 1 | 2 | 1 | 21.04 | 20.49 | 21.78 | 41.52 | 51.58 | 42.22 | 49.07 | 34.98 | 28.60 | 34.92 | 38.81 | 42.01 | 61.94 | 57.74 | 11.41 |
| Roach | 1123 | 1 | 2 | 1 | 18.39 | 21.05 | 22.10 | 41.57 | 51.44 | 41.09 | 49.26 | 35.01 | 26.47 | 35.43 | 40.15 | 40.06 | 61.39 | 55.12 | 12.30 |
| Roach | 1124 | 1 | 2 | 1 | 21.17 | 21.90 | 22.02 | 42.08 | 56.18 | 46.35 | 50.45 | 37.37 | 28.39 | 29.29 | 36.42 | 43.15 | 64.17 | 62.28 | 12.69 |
| Roach | 1125 | 1 | 2 | 1 | 20.48 | 19.44 | 20.55 | 37.28 | 47.24 | 37.89 | 41.80 | 32.57 | 25.92 | 31.81 | 34.95 | 35.54 | 60.28 | 54.27 | 12.06 |
| Roach | 1126 | 1 | 2 | 1 | 23.91 | 24.75 | 24.52 | 44.93 | 53.87 | 43.43 | 52.00 | 35.38 | 27.39 | 34.03 | 40.80 | 42.08 | 64.44 | 60.39 | 13.47 |
| Roach | 1127 | 1 | 2 | 1 | 30.43 | 27.26 | 30.18 | 54.68 | 70.36 | 58.99 | 68.25 | 50.67 | 40.44 | 41.76 | 50.38 | 56.58 | 80.96 | 73.15 | 18.24 |
| Roach | 1128 | 1 | 2 | 1 | 26.06 | 24.68 | 24.13 | 47.70 | 56.57 | 47.58 | 57.57 | 40.91 | 23.47 | 32.19 | 37.48 | 44.44 | 66.61 | 63.84 | 12.77 |
| Roach | 1129 | 1 | 2 | 1 | 24.94 | 19.39 | 23.36 | 40.72 | 50.55 | 44.67 | 50.55 | 36.19 | 23.23 | 33.76 | 40.60 | 42.40 | 68.02 | 65.79 | 13.22 |
| Roach | 1130 | 1 | 2 | 1 | 19.74 | 20.12 | 20.69 | 36.98 | 48.52 | 40.45 | 42.28 | 32.63 | 25.30 | 25.12 | 30.86 | 36.61 | 55.40 | 53.81 | 9.71 |
| Roach | 1101 | 1 | 2 | 2 | 33.36 | 31.21 | 36.44 | 65.63 | 89.24 | 76.21 | 83.17 | 65.10 | 46.02 | 48.85 | 60.56 | 74.98 | 107.78 | 102.85 | 21.82 |
| Roach | 1102 | 1 | 2 | 2 | 31.48 | 30.35 | 30.41 | 58.89 | 71.12 | 56.92 | 70.75 | 50.64 | 41.18 | 46.77 | 54.80 | 57.72 | 88.10 | 84.97 | 18.54 |
| Roach | 1103 | 1 | 2 | 2 | 32.48 | 30.35 | 33.47 | 60.82 | 77.95 | 61.39 | 74.35 | 56.15 | 43.11 | 41.79 | 52.26 | 62.87 | 89.47 | 83.35 | 19.91 |
| Roach | 1104 | 1 | 2 | 2 | 34.82 | 35.37 | 39.85 | 59.78 | 80.42 | 63.66 | 73.52 | 57.98 | 42.01 | 46.83 | 58.70 | 65.83 | 96.88 | 91.90 | 19.46 |
| Roach | 1105 | 1 | 2 | 2 | 30.38 | 30.93 | 33.08 | 62.26 | 75.90 | 57.35 | 69.34 | 53.80 | 38.81 | 46.39 | 52.67 | 62.23 | 91.12 | 86.11 | 17.64 |
| Roach | 1106 | 1 | 2 | 2 | 23.39 | 22.45 | 26.68 | 40.44 | 55.36 | 42.57 | 50.13 | 41.11 | 29.61 | 35.74 | 42.15 | 43.64 | 66.32 | 60.66 | 13.39 |
| Roach | 1107 | 1 | 2 | 2 | 29.42 | 29.59 | 31.30 | 59.05 | 71.74 | 57.03 | 67.09 | 50.53 | 38.75 | 47.36 | 54.17 | 53.89 | 83.51 | 76.15 | 17.73 |
| Roach | 1108 | 1 | 2 | 2 | 26.59 | 27.99 | 29.87 | 53.45 | 69.35 | 57.90 | 66.71 | 50.20 | 37.61 | 39.17 | 48.10 | 55.46 | 80.93 | 76.35 | 15.83 |
| Roach | 1109 | 1 | 2 | 2 | 32.97 | 31.73 | 33.82 | 51.42 | 71.00 | 58.69 | 67.01 | 54.74 | 40.68 | 40.60 | 50.48 | 58.41 | 81.77 | 77.82 | 18.54 |
| Roach | 1110 | 1 | 2 | 2 | 26.36 | 23.05 | 23.97 | 47.06 | 57.60 | 47.80 | 56.71 | 41.60 | 32.37 | 36.74 | 45.04 | 46.78 | 69.85 | 67.58 | 14.50 |
| Roach | 1111 | 1 | 2 | 2 | 26.98 | 26.84 | 28.57 | 46.75 | 61.36 | 49.68 | 57.78 | 45.70 | 34.78 | 41.18 | 49.67 | 51.69 | 79.43 | 73.28 | 15.93 |
| Roach | 1112 | 1 | 2 | 2 | 25.03 | 23.37 | 25.52 | 42.85 | 54.55 | 45.91 | 53.62 | 37.99 | 31.29 | 32.73 | 40.71 | 46.04 | 68.51 | 64.90 | 13.50 |
| Roach | 1113 | 1 | 2 | 2 | 31.84 | 31.01 | 31.57 | 53.02 | 69.69 | 55.33 | 65.49 | 51.91 | 34.92 | 42.65 | 51.95 | 54.24 | 79.81 | 76.13 | 18.29 |
| Roach | 1114 | 1 | 2 | 2 | 22.46 | 22.76 | 23.32 | 40.04 | 54.09 | 44.31 | 47.82 | 38.71 | 29.04 | 31.79 | 38.21 | 42.75 | 65.17 | 61.44 | 15.07 |
| Roach | 1115 | 1 | 2 | 2 | 26.79 | 24.57 | 27.06 | 48.31 | 63.83 | 52.29 | 58.62 | 46.04 | 30.33 | 39.22 | 47.10 | 52.41 | 76.70 | 72.67 | 16.20 |
| Roach | 1116 | 1 | 2 | 2 | 19.77 | 19.69 | 20.58 | 39.18 | 49.76 | 40.38 | 46.70 | 35.67 | 27.47 | 32.73 | 37.63 | 40.36 | 59.89 | 55.48 | 11.66 |
| Roach | 1117 | 1 | 2 | 2 | 23.80 | 21.57 | 24.72 | 49.29 | 60.94 | 49.43 | 57.47 | 44.03 | 31.96 | 30.33 | 38.70 | 49.83 | 69.74 | 66.37 | 15.30 |
| Roach | 1118 | 1 | 2 | 2 | 20.45 | 23.44 | 24.43 | 41.45 | 58.56 | 50.64 | 53.93 | 39.97 | 32.41 | 30.90 | 38.40 | 45.27 | 67.84 | 62.62 | 15.12 |
| Roach | 1119 | 1 | 2 | 2 | 26.61 | 21.24 | 24.40 | 49.39 | 58.77 | 51.58 | 59.41 | 43.47 | 31.73 | 36.35 | 43.28 | 45.88 | 69.46 | 66.82 | 15.30 |
| Roach | 1120 | 1 | 2 | 2 | 21.04 | 22.06 | 21.31 | 35.88 | 45.28 | 37.55 | 43.83 | 30.75 | 26.87 | 32.80 | 37.24 | 38.94 | 62.46 | 59.09 | 12.67 |
| Roach | 1121 | 1 | 2 | 2 | 25.18 | 24.30 | 23.63 | 44.42 | 54.95 | 47.22 | 51.61 | 35.77 | 27.57 | 33.36 | 38.22 | 43.81 | 66.06 | 62.58 | 13.26 |
| Roach | 1122 | 1 | 2 | 2 | 18.93 | 20.67 | 21.43 | 43.21 | 52.16 | 41.66 | 47.22 | 35.51 | 29.16 | 34.24 | 39.79 | 40.83 | 61.33 | 54.85 | 12.18 |
| Roach | 1123 | 1 | 2 | 2 | 19.74 | 21.31 | 22.98 | 44.00 | 52.17 | 41.09 | 48.83 | 35.41 | 26.00 | 34.22 | 39.30 | 40.20 | 60.88 | 54.52 | 12.85 |
| Roach | 1124 | 1 | 2 | 2 | 21.76 | 22.13 | 23.53 | 43.95 | 55.64 | 45.86 | 51.05 | 37.59 | 27.74 | 33.17 | 39.47 | 42.32 | 62.20 | 58.68 | 13.06 |
| Roach | 1125 | 1 | 2 | 2 | 19.53 | 20.82 | 20.72 | 37.81 | 47.84 | 36.29 | 43.59 | 32.98 | 22.90 | 30.95 | 35.12 | 36.31 | 57.47 | 54.30 | 12.07 |
| Roach | 1126 | 1 | 2 | 2 | 23.60 | 24.83 | 24.97 | 42.20 | 51.88 | 42.07 | 52.40 | 34.56 | 26.17 | 34.42 | 41.02 | 43.81 | 66.08 | 63.37 | 13.50 |
| Roach | 1127 | 1 | 2 | 2 | 29.31 | 28.26 | 30.14 | 60.77 | 71.69 | 59.74 | 70.57 | 52.25 | 38.48 | 41.48 | 48.74 | 59.77 | 84.59 | 76.87 | 17.87 |
| Roach | 1128 | 1 | 2 | 2 | 23.98 | 24.05 | 24.92 | 49.03 | 57.56 | 46.27 | 56.45 | 42.12 | 25.29 | 32.29 | 38.31 | 46.34 | 66.60 | 64.73 | 14.54 |
| Roach | 1129 | 1 | 2 | 2 | 23.82 | 21.79 | 24.71 | 40.39 | 54.22 | 46.51 | 52.94 | 36.44 | 21.16 | 32.77 | 38.96 | 41.96 | 67.37 | 66.09 | 13.70 |
| Roach | 1130 | 1 | 2 | 2 | 18.79 | 18.81 | 21.04 | 36.17 | 48.56 | 40.31 | 45.81 | 33.24 | 23.65 | 23.92 | 30.25 | 36.78 | 54.96 | 54.70 | 10.33 |
| Roach | 1101 | 1 | 2 | 3 | 33.39 | 30.77 | 36.16 | 68.09 | 91.34 | 75.55 | 83.15 | 65.22 | 45.75 | 47.66 | 58.41 | 73.41 | 106.20 | 100.81 | 22.28 |
| Roach | 1102 | 1 | 2 | 3 | 31.64 | 31.82 | 32.48 | 58.06 | 71.18 | 57.06 | 71.50 | 53.01 | 37.72 | 47.80 | 54.85 | 56.70 | 88.73 | 82.56 | 16.45 |
| Roach | 1103 | 1 | 2 | 3 | 34.17 | 29.63 | 33.49 | 60.88 | 76.17 | 62.66 | 74.27 | 55.31 | 43.11 | 41.79 | 53.51 | 62.15 | 88.34 | 84.25 | 19.99 |
| Roach | 1104 | 1 | 2 | 3 | 35.23 | 32.61 | 37.32 | 57.96 | 77.42 | 58.74 | 72.21 | 57.97 | 44.68 | 47.37 | 55.61 | 65.19 | 93.96 | 88.58 | 18.95 |
| Roach | 1105 | 1 | 2 | 3 | 30.97 | 31.98 | 32.20 | 60.77 | 77.83 | 58.78 | 69.89 | 54.05 | 37.61 | 47.20 | 52.71 | 59.85 | 88.94 | 82.95 | 19.89 |
| Roach | 1106 | 1 | 2 | 3 | 23.26 | 20.15 | 23.71 | 41.79 | 55.94 | 44.45 | 51.90 | 40.12 | 29.89 | 37.71 | 42.54 | 45.61 | 67.34 | 61.89 | 12.38 |
| Roach | 1107 | 1 | 2 | 3 | 30.15 | 31.55 | 31.73 | 57.38 | 70.34 | 53.80 | 64.00 | 50.39 | 38.58 | 47.79 | 53.70 | 54.41 | 83.25 | 75.52 | 17.21 |
| Roach | 1108 | 1 | 2 | 3 | 28.83 | 24.28 | 28.52 | 55.14 | 67.04 | 57.42 | 65.98 | 48.84 | 37.64 | 40.34 | 47.20 | 54.33 | 80.88 | 75.73 | 16.06 |
| Roach | 1109 | 1 | 2 | 3 | 29.18 | 29.25 | 32.49 | 50.07 | 70.28 | 58.77 | 67.13 | 54.75 | 39.67 | 41.69 | 52.03 | 58.32 | 83.47 | 80.38 | 19.18 |
| Roach | 1110 | 1 | 2 | 3 | 24.77 | 20.29 | 24.02 | 44.43 | 56.14 | 50.20 | 56.40 | 40.60 | 32.97 | 35.51 | 42.97 | 46.45 | 72.46 | 66.57 | 13.47 |
| Roach | 1111 | 1 | 2 | 3 | 25.73 | 25.66 | 27.93 | 46.85 | 61.21 | 49.24 | 59.14 | 45.00 | 33.31 | 43.31 | 49.71 | 50.54 | 78.94 | 75.33 | 17.12 |
| Roach | 1112 | 1 | 2 | 3 | 25.18 | 25.13 | 25.14 | 43.73 | 54.74 | 46.60 | 54.24 | 38.03 | 29.77 | 33.43 | 41.68 | 43.89 | 67.27 | 63.28 | 13.60 |
| Roach | 1113 | 1 | 2 | 3 | 32.01 | 31.03 | 31.04 | 52.05 | 69.12 | 55.67 | 64.09 | 50.42 | 36.80 | 42.54 | 49.16 | 54.82 | 80.79 | 77.39 | 17.78 |
| Roach | 1114 | 1 | 2 | 3 | 23.80 | 21.85 | 22.32 | 41.39 | 53.84 | 42.71 | 48.20 | 38.93 | 25.54 | 36.37 | 41.77 | 40.24 | 64.95 | 62.08 | 14.01 |
| Roach | 1115 | 1 | 2 | 3 | 27.47 | 25.49 | 28.18 | 47.10 | 62.63 | 53.81 | 60.46 | 47.42 | 29.77 | 38.71 | 45.48 | 50.56 | 75.27 | 71.19 | 15.35 |
| Roach | 1116 | 1 | 2 | 3 | 19.97 | 21.06 | 21.68 | 39.87 | 48.17 | 37.33 | 45.81 | 33.89 | 26.91 | 32.86 | 38.15 | 40.59 | 60.61 | 57.17 | 10.46 |
| Roach | 1117 | 1 | 2 | 3 | 23.26 | 21.85 | 25.46 | 47.41 | 56.98 | 45.16 | 55.82 | 42.90 | 33.59 | 32.15 | 40.72 | 49.34 | 70.70 | 67.15 | 15.79 |
| Roach | 1118 | 1 | 2 | 3 | 21.99 | 24.44 | 25.95 | 42.68 | 60.29 | 50.66 | 52.57 | 38.09 | 28.42 | 35.52 | 42.18 | 43.59 | 64.47 | 61.89 | 14.66 |
| Roach | 1119 | 1 | 2 | 3 | 27.20 | 21.38 | 26.34 | 45.57 | 58.44 | 50.06 | 56.91 | 42.75 | 32.15 | 39.15 | 47.02 | 45.26 | 70.08 | 66.52 | 14.99 |
| Roach | 1120 | 1 | 2 | 3 | 20.81 | 20.63 | 20.59 | 36.37 | 45.91 | 38.40 | 43.66 | 30.74 | 28.21 | 31.63 | 36.85 | 38.47 | 59.88 | 57.57 | 12.21 |
| Roach | 1121 | 1 | 2 | 3 | 22.46 | 23.64 | 23.88 | 41.55 | 53.37 | 44.75 | 52.46 | 35.96 | 24.88 | 33.88 | 39.94 | 41.72 | 66.61 | 62.52 | 14.66 |
| Roach | 1122 | 1 | 2 | 3 | 21.00 | 21.85 | 22.33 | 37.21 | 50.59 | 40.93 | 49.10 | 35.92 | 29.75 | 32.91 | 38.48 | 42.42 | 62.03 | 57.39 | 12.79 |
| Roach | 1123 | 1 | 2 | 3 | 19.88 | 20.12 | 21.37 | 42.13 | 52.69 | 40.79 | 49.45 | 35.36 | 25.86 | 35.35 | 41.08 | 38.57 | 59.22 | 53.89 | 13.13 |
| Roach | 1124 | 1 | 2 | 3 | 21.05 | 22.11 | 22.66 | 45.90 | 56.35 | 45.92 | 51.91 | 36.97 | 29.24 | 31.02 | 36.80 | 43.99 | 66.43 | 61.41 | 12.90 |
| Roach | 1125 | 1 | 2 | 3 | 19.26 | 18.86 | 19.64 | 38.37 | 48.08 | 36.38 | 41.31 | 30.34 | 25.05 | 30.44 | 34.79 | 34.74 | 57.00 | 53.00 | 11.86 |
| Roach | 1126 | 1 | 2 | 3 | 25.49 | 24.86 | 23.80 | 43.51 | 52.45 | 43.51 | 49.10 | 33.35 | 28.59 | 36.64 | 42.36 | 41.84 | 65.53 | 62.66 | 13.25 |
| Roach | 1127 | 1 | 2 | 3 | 30.00 | 27.51 | 30.72 | 53.49 | 68.97 | 57.45 | 68.47 | 49.51 | 39.06 | 43.45 | 50.08 | 59.61 | 84.28 | 75.61 | 16.52 |
| Roach | 1128 | 1 | 2 | 3 | 25.30 | 22.49 | 24.66 | 47.20 | 56.32 | 46.46 | 56.97 | 40.66 | 24.10 | 31.36 | 37.76 | 45.70 | 68.62 | 63.77 | 13.71 |
| Roach | 1129 | 1 | 2 | 3 | 24.33 | 18.84 | 24.44 | 42.55 | 55.14 | 45.16 | 51.56 | 35.98 | 24.41 | 29.99 | 35.81 | 44.50 | 70.21 | 66.17 | 13.13 |
| Roach | 1130 | 1 | 2 | 3 | 19.05 | 21.30 | 20.30 | 36.28 | 47.73 | 38.57 | 42.76 | 31.10 | 22.49 | 26.51 | 32.76 | 34.91 | 54.35 | 55.36 | 10.76 |
| Roach | 1191 | 1 | 3 | 1 | 36.98 | 32.32 | 35.59 | 64.97 | 82.01 | 69.59 | 80.63 | 58.73 | 45.81 | 47.28 | 54.65 | 66.55 | 95.95 | 89.39 | 19.53 |
| Roach | 1192 | 1 | 3 | 1 | 29.53 | 32.82 | 34.00 | 58.38 | 79.90 | 66.65 | 73.14 | 59.28 | 39.83 | 47.10 | 56.85 | 64.97 | 92.01 | 84.40 | 19.54 |
| Roach | 1193 | 1 | 3 | 1 | 25.96 | 29.85 | 33.93 | 56.87 | 73.63 | 57.34 | 69.22 | 55.63 | 38.75 | 41.11 | 45.82 | 63.91 | 88.88 | 83.92 | 16.43 |
| Roach | 1194 | 1 | 3 | 1 | 27.68 | 22.92 | 26.92 | 48.61 | 61.94 | 51.65 | 57.82 | 44.82 | 32.19 | 37.36 | 43.42 | 49.29 | 67.75 | 63.49 | 13.75 |
| Roach | 1195 | 1 | 3 | 1 | 29.87 | 26.89 | 32.63 | 55.24 | 73.01 | 57.57 | 66.82 | 54.75 | 36.53 | 48.05 | 53.17 | 58.06 | 85.64 | 80.12 | 15.54 |
| Roach | 1196 | 1 | 3 | 1 | 32.81 | 32.11 | 35.00 | 58.57 | 76.48 | 62.06 | 71.88 | 55.17 | 43.21 | 38.79 | 49.50 | 62.05 | 84.96 | 82.73 | 16.75 |
| Roach | 1197 | 1 | 3 | 1 | 43.04 | 35.81 | 41.81 | 69.32 | 93.09 | 81.86 | 91.83 | 71.97 | 49.57 | 40.90 | 55.99 | 79.71 | 102.53 | 97.73 | 21.37 |
| Roach | 1198 | 1 | 3 | 1 | 25.59 | 30.01 | 29.92 | 49.13 | 63.35 | 51.25 | 63.71 | 45.62 | 32.26 | 39.26 | 45.27 | 52.54 | 75.83 | 70.55 | 14.02 |
| Roach | 1199 | 1 | 3 | 1 | 25.33 | 27.37 | 30.29 | 52.13 | 66.64 | 50.99 | 62.57 | 49.29 | 38.30 | 42.66 | 49.03 | 57.37 | 88.33 | 80.62 | 15.83 |
| Roach | 1200 | 1 | 3 | 1 | 32.03 | 24.88 | 32.66 | 57.68 | 75.95 | 65.08 | 70.48 | 54.67 | 44.64 | 46.20 | 54.44 | 61.86 | 89.24 | 82.46 | 17.33 |
| Roach | 1201 | 1 | 3 | 1 | 35.37 | 33.25 | 37.17 | 62.41 | 80.50 | 62.86 | 78.65 | 62.76 | 43.45 | 49.33 | 59.59 | 65.38 | 92.61 | 82.43 | 19.04 |
| Roach | 1202 | 1 | 3 | 1 | 24.94 | 28.47 | 30.63 | 51.70 | 71.89 | 61.08 | 64.33 | 51.14 | 31.07 | 39.19 | 45.52 | 54.06 | 73.97 | 67.49 | 14.92 |
| Roach | 1203 | 1 | 3 | 1 | 29.73 | 26.77 | 30.06 | 51.11 | 64.90 | 52.19 | 62.97 | 47.28 | 30.45 | 42.91 | 47.30 | 51.19 | 80.06 | 74.87 | 13.12 |
| Roach | 1204 | 1 | 3 | 1 | 27.60 | 24.20 | 28.39 | 53.47 | 70.77 | 56.77 | 64.75 | 51.37 | 34.51 | 43.18 | 52.09 | 51.55 | 81.50 | 73.19 | 15.82 |
| Roach | 1205 | 1 | 3 | 1 | 32.64 | 30.53 | 33.46 | 53.84 | 67.33 | 50.56 | 64.65 | 50.69 | 36.52 | 41.79 | 47.30 | 56.27 | 83.82 | 79.54 | 16.30 |
| Roach | 1206 | 1 | 3 | 1 | 33.74 | 33.74 | 36.62 | 55.97 | 74.90 | 57.46 | 71.17 | 57.96 | 40.09 | 45.72 | 52.77 | 64.20 | 95.66 | 92.67 | 17.11 |
| Roach | 1207 | 1 | 3 | 1 | 26.60 | 29.34 | 31.25 | 49.50 | 66.23 | 51.10 | 60.79 | 50.28 | 36.24 | 41.01 | 47.90 | 61.22 | 86.13 | 81.11 | 14.98 |
| Roach | 1208 | 1 | 3 | 1 | 25.73 | 30.37 | 30.33 | 49.33 | 68.73 | 58.06 | 63.20 | 49.94 | 35.02 | 40.52 | 46.73 | 56.79 | 89.11 | 84.99 | 15.09 |
| Roach | 1209 | 1 | 3 | 1 | 25.07 | 26.49 | 27.26 | 39.70 | 53.84 | 45.07 | 52.82 | 42.05 | 33.34 | 35.19 | 40.69 | 45.77 | 69.48 | 64.96 | 13.12 |
| Roach | 1210 | 1 | 3 | 1 | 24.90 | 26.21 | 27.13 | 50.58 | 62.76 | 53.21 | 60.58 | 44.39 | 27.31 | 41.18 | 49.55 | 47.16 | 72.39 | 67.26 | 15.54 |
| Roach | 1211 | 1 | 3 | 1 | 26.88 | 26.59 | 25.45 | 44.43 | 52.81 | 39.01 | 48.92 | 37.14 | 30.95 | 35.59 | 40.36 | 40.70 | 61.98 | 58.98 | 10.87 |
| Roach | 1212 | 1 | 3 | 1 | 32.73 | 34.82 | 34.36 | 57.82 | 73.75 | 56.69 | 70.71 | 56.15 | 40.52 | 46.55 | 57.27 | 61.94 | 90.36 | 84.73 | 19.12 |
| Roach | 1213 | 1 | 3 | 1 | 26.13 | 26.86 | 31.65 | 59.22 | 75.14 | 62.06 | 71.80 | 53.99 | 37.51 | 42.46 | 52.09 | 60.22 | 86.89 | 83.18 | 17.23 |
| Roach | 1214 | 1 | 3 | 1 | 28.60 | 27.37 | 28.99 | 59.56 | 70.52 | 55.52 | 69.22 | 54.12 | 40.13 | 44.78 | 51.99 | 58.88 | 89.94 | 87.97 | 14.91 |
| Roach | 1215 | 1 | 3 | 1 | 26.13 | 22.65 | 26.28 | 42.52 | 56.84 | 44.87 | 52.16 | 39.33 | 29.61 | 35.47 | 40.26 | 45.12 | 69.35 | 66.14 | 12.67 |
| Roach | 1216 | 1 | 3 | 1 | 31.02 | 31.23 | 34.10 | 57.99 | 69.71 | 52.18 | 66.54 | 53.67 | 41.50 | 46.83 | 55.21 | 58.34 | 87.82 | 81.50 | 16.73 |
| Roach | 1217 | 1 | 3 | 1 | 33.16 | 34.74 | 35.27 | 61.06 | 75.88 | 62.66 | 71.78 | 50.31 | 32.81 | 38.49 | 44.66 | 54.80 | 81.26 | 79.96 | 13.87 |
| Roach | 1218 | 1 | 3 | 1 | 23.83 | 23.97 | 27.16 | 50.36 | 63.57 | 53.38 | 64.29 | 48.86 | 34.15 | 33.78 | 43.52 | 54.98 | 75.05 | 70.24 | 16.82 |
| Roach | 1219 | 1 | 3 | 1 | 31.22 | 25.69 | 29.73 | 55.38 | 69.70 | 59.21 | 70.33 | 50.32 | 32.11 | 36.45 | 44.53 | 56.43 | 81.26 | 76.49 | 16.46 |
| Roach | 1220 | 1 | 3 | 1 | 20.19 | 18.00 | 20.02 | 37.24 | 49.41 | 43.36 | 47.90 | 35.90 | 26.92 | 28.40 | 33.11 | 40.06 | 59.24 | 51.87 | 11.79 |
| Roach | 1191 | 1 | 3 | 2 | 38.55 | 33.76 | 35.28 | 62.52 | 78.52 | 63.33 | 75.27 | 57.63 | 47.28 | 48.61 | 55.82 | 67.73 | 98.11 | 89.70 | 18.13 |
| Roach | 1192 | 1 | 3 | 2 | 30.13 | 32.03 | 34.27 | 58.50 | 78.67 | 64.20 | 73.23 | 59.68 | 38.07 | 50.22 | 58.23 | 64.04 | 94.20 | 84.76 | 19.25 |
| Roach | 1193 | 1 | 3 | 2 | 28.89 | 28.77 | 31.70 | 53.72 | 73.70 | 55.91 | 65.85 | 54.93 | 36.72 | 39.99 | 47.32 | 60.72 | 86.38 | 84.64 | 16.23 |
| Roach | 1194 | 1 | 3 | 2 | 26.97 | 23.02 | 25.79 | 47.64 | 59.24 | 47.81 | 60.19 | 44.31 | 31.04 | 36.10 | 42.86 | 49.43 | 71.02 | 66.60 | 13.59 |
| Roach | 1195 | 1 | 3 | 2 | 28.54 | 27.50 | 31.66 | 58.95 | 72.88 | 62.39 | 71.36 | 53.35 | 37.17 | 42.91 | 49.85 | 58.31 | 85.25 | 79.75 | 16.33 |
| Roach | 1196 | 1 | 3 | 2 | 33.20 | 31.11 | 34.66 | 59.02 | 75.82 | 62.66 | 75.29 | 56.08 | 37.39 | 42.46 | 52.79 | 61.89 | 86.73 | 86.01 | 16.43 |
| Roach | 1197 | 1 | 3 | 2 | 43.66 | 41.24 | 44.97 | 72.84 | 94.99 | 81.02 | 97.05 | 72.57 | 43.89 | 41.48 | 52.53 | 80.32 | 105.35 | 97.81 | 21.74 |
| Roach | 1198 | 1 | 3 | 2 | 27.59 | 29.52 | 29.61 | 49.89 | 67.85 | 54.82 | 60.77 | 45.34 | 36.27 | 36.33 | 43.01 | 50.04 | 71.37 | 66.70 | 13.12 |
| Roach | 1199 | 1 | 3 | 2 | 28.47 | 29.78 | 32.11 | 49.58 | 64.40 | 52.77 | 66.55 | 49.30 | 34.14 | 44.69 | 50.19 | 56.10 | 86.44 | 83.02 | 14.50 |
| Roach | 1200 | 1 | 3 | 2 | 31.34 | 25.94 | 31.65 | 58.06 | 75.32 | 60.82 | 68.64 | 52.72 | 47.28 | 42.49 | 48.45 | 61.65 | 85.89 | 78.82 | 16.88 |
| Roach | 1201 | 1 | 3 | 2 | 33.98 | 35.44 | 38.95 | 62.18 | 78.58 | 61.96 | 77.71 | 61.20 | 47.27 | 46.11 | 56.66 | 68.60 | 93.52 | 87.03 | 17.73 |
| Roach | 1202 | 1 | 3 | 2 | 26.78 | 29.47 | 31.39 | 50.08 | 68.71 | 59.10 | 62.19 | 50.77 | 31.85 | 39.69 | 45.52 | 53.82 | 74.92 | 72.63 | 14.98 |
| Roach | 1203 | 1 | 3 | 2 | 26.69 | 26.77 | 32.36 | 47.93 | 63.08 | 50.48 | 62.03 | 49.58 | 28.59 | 41.33 | 49.78 | 51.00 | 78.21 | 74.49 | 14.93 |
| Roach | 1204 | 1 | 3 | 2 | 28.23 | 24.83 | 28.11 | 53.09 | 68.42 | 58.52 | 65.56 | 52.09 | 35.26 | 43.04 | 49.10 | 52.75 | 80.67 | 79.94 | 15.38 |
| Roach | 1205 | 1 | 3 | 2 | 31.74 | 32.15 | 34.37 | 52.77 | 66.83 | 51.21 | 67.01 | 51.48 | 34.04 | 36.74 | 46.39 | 55.31 | 79.53 | 76.93 | 17.64 |
| Roach | 1206 | 1 | 3 | 2 | 32.98 | 32.81 | 34.43 | 55.37 | 72.69 | 57.35 | 72.07 | 56.52 | 37.80 | 48.54 | 54.01 | 65.47 | 94.07 | 92.08 | 14.08 |
| Roach | 1207 | 1 | 3 | 2 | 25.99 | 29.72 | 30.80 | 47.52 | 65.92 | 56.74 | 64.29 | 50.98 | 32.66 | 43.65 | 51.67 | 54.90 | 79.31 | 73.40 | 17.91 |
| Roach | 1208 | 1 | 3 | 2 | 28.16 | 25.90 | 29.72 | 50.18 | 69.06 | 60.87 | 64.71 | 50.64 | 34.05 | 41.05 | 47.74 | 56.79 | 82.30 | 81.81 | 15.03 |
| Roach | 1209 | 1 | 3 | 2 | 23.87 | 27.46 | 24.86 | 40.36 | 55.29 | 46.16 | 53.54 | 40.24 | 31.66 | 36.19 | 42.15 | 47.31 | 69.48 | 67.01 | 12.21 |
| Roach | 1210 | 1 | 3 | 2 | 27.60 | 27.14 | 29.85 | 47.58 | 67.28 | 56.21 | 60.82 | 45.29 | 26.43 | 39.10 | 46.55 | 45.53 | 71.98 | 70.11 | 12.73 |
| Roach | 1211 | 1 | 3 | 2 | 22.72 | 25.53 | 24.97 | 41.45 | 52.67 | 42.24 | 50.58 | 37.17 | 30.50 | 32.56 | 39.02 | 40.96 | 60.80 | 58.53 | 11.08 |
| Roach | 1212 | 1 | 3 | 2 | 31.25 | 34.51 | 35.28 | 58.57 | 75.72 | 58.12 | 70.00 | 55.61 | 39.55 | 46.51 | 57.63 | 61.25 | 90.51 | 83.89 | 18.17 |
| Roach | 1213 | 1 | 3 | 2 | 27.99 | 27.22 | 31.28 | 60.23 | 75.08 | 63.10 | 71.80 | 54.93 | 34.43 | 44.61 | 54.93 | 59.74 | 86.22 | 84.96 | 17.73 |
| Roach | 1214 | 1 | 3 | 2 | 27.76 | 29.72 | 30.86 | 56.97 | 75.48 | 59.37 | 66.85 | 55.16 | 42.11 | 43.63 | 54.59 | 61.06 | 94.21 | 93.91 | 16.39 |
| Roach | 1215 | 1 | 3 | 2 | 25.83 | 25.08 | 26.32 | 43.01 | 56.35 | 46.39 | 52.96 | 42.95 | 28.20 | 38.36 | 42.66 | 45.05 | 71.18 | 70.42 | 11.89 |
| Roach | 1216 | 1 | 3 | 2 | 31.97 | 27.90 | 33.47 | 60.82 | 75.99 | 60.68 | 74.50 | 53.85 | 33.45 | 47.18 | 54.67 | 60.09 | 91.31 | 87.63 | 15.38 |
| Roach | 1217 | 1 | 3 | 2 | 31.34 | 32.74 | 34.55 | 56.98 | 74.52 | 62.06 | 70.00 | 51.16 | 31.14 | 41.68 | 50.54 | 53.58 | 79.41 | 78.68 | 15.45 |
| Roach | 1218 | 1 | 3 | 2 | 25.98 | 25.86 | 28.97 | 46.55 | 63.76 | 53.55 | 62.12 | 48.39 | 31.59 | 37.98 | 42.96 | 51.93 | 76.28 | 74.53 | 15.35 |
| Roach | 1219 | 1 | 3 | 2 | 31.60 | 28.11 | 31.70 | 54.93 | 68.56 | 57.14 | 66.47 | 50.73 | 35.30 | 36.06 | 44.69 | 56.35 | 84.29 | 77.07 | 15.61 |
| Roach | 1220 | 1 | 3 | 2 | 22.26 | 22.04 | 20.35 | 37.24 | 44.98 | 37.42 | 47.41 | 34.86 | 26.86 | 28.32 | 31.38 | 38.90 | 58.07 | 52.23 | 11.00 |
| Roach | 1191 | 1 | 3 | 3 | 39.89 | 30.64 | 34.84 | 64.10 | 78.38 | 68.61 | 79.67 | 58.38 | 43.29 | 49.13 | 57.18 | 65.83 | 97.92 | 91.23 | 18.29 |
| Roach | 1192 | 1 | 3 | 3 | 29.75 | 33.69 | 34.41 | 58.41 | 81.55 | 65.24 | 70.50 | 59.23 | 39.92 | 47.90 | 57.06 | 63.57 | 91.60 | 85.48 | 20.36 |
| Roach | 1193 | 1 | 3 | 3 | 26.23 | 28.31 | 32.10 | 61.13 | 76.41 | 59.32 | 69.48 | 53.89 | 37.42 | 38.25 | 44.36 | 61.71 | 88.44 | 81.06 | 17.66 |
| Roach | 1194 | 1 | 3 | 3 | 27.26 | 24.47 | 26.77 | 54.35 | 61.67 | 48.36 | 61.19 | 44.36 | 30.41 | 34.67 | 41.16 | 51.55 | 71.28 | 67.49 | 14.04 |
| Roach | 1195 | 1 | 3 | 3 | 28.89 | 27.71 | 31.70 | 56.14 | 69.98 | 56.80 | 70.47 | 53.54 | 37.42 | 43.24 | 51.55 | 61.77 | 87.95 | 82.65 | 15.85 |
| Roach | 1196 | 1 | 3 | 3 | 33.26 | 31.97 | 34.92 | 59.36 | 74.60 | 59.00 | 69.84 | 55.62 | 41.05 | 43.92 | 52.99 | 58.82 | 84.30 | 79.38 | 17.63 |
| Roach | 1197 | 1 | 3 | 3 | 39.96 | 37.40 | 42.55 | 77.84 | 94.65 | 81.12 | 93.03 | 70.81 | 46.39 | 44.04 | 56.99 | 80.35 | 104.39 | 99.90 | 21.29 |
| Roach | 1198 | 1 | 3 | 3 | 24.72 | 29.73 | 28.66 | 49.68 | 59.82 | 46.67 | 61.16 | 44.91 | 33.57 | 38.49 | 43.81 | 53.17 | 77.65 | 72.84 | 13.11 |
| Roach | 1199 | 1 | 3 | 3 | 23.45 | 27.53 | 29.39 | 50.99 | 66.46 | 51.96 | 64.26 | 49.28 | 37.53 | 44.09 | 49.55 | 57.33 | 87.15 | 81.50 | 15.85 |
| Roach | 1200 | 1 | 3 | 3 | 33.01 | 25.27 | 32.63 | 56.27 | 75.16 | 65.88 | 72.37 | 53.09 | 41.84 | 46.94 | 52.33 | 61.71 | 90.59 | 81.84 | 16.58 |
| Roach | 1201 | 1 | 3 | 3 | 37.11 | 33.84 | 39.05 | 63.27 | 81.15 | 63.11 | 79.25 | 59.92 | 40.52 | 49.35 | 56.99 | 63.96 | 86.32 | 80.79 | 18.62 |
| Roach | 1202 | 1 | 3 | 3 | 26.36 | 28.50 | 31.92 | 49.68 | 67.24 | 59.00 | 68.09 | 51.54 | 30.56 | 39.26 | 46.84 | 59.68 | 81.49 | 75.27 | 15.38 |
| Roach | 1203 | 1 | 3 | 3 | 29.12 | 28.95 | 31.66 | 52.02 | 65.23 | 55.05 | 61.49 | 47.06 | 31.58 | 42.23 | 48.83 | 52.19 | 78.92 | 75.74 | 12.21 |
| Roach | 1204 | 1 | 3 | 3 | 28.61 | 25.14 | 28.71 | 53.87 | 69.76 | 57.63 | 65.96 | 50.47 | 32.60 | 47.04 | 54.56 | 54.67 | 83.71 | 80.84 | 15.38 |
| Roach | 1205 | 1 | 3 | 3 | 32.64 | 29.92 | 32.11 | 58.26 | 67.38 | 54.83 | 68.09 | 51.67 | 36.21 | 40.19 | 46.25 | 55.99 | 82.61 | 78.78 | 16.73 |
| Roach | 1206 | 1 | 3 | 3 | 31.97 | 32.19 | 35.28 | 56.94 | 74.00 | 58.08 | 71.53 | 57.67 | 39.99 | 44.16 | 55.40 | 64.75 | 93.49 | 91.79 | 17.02 |
| Roach | 1207 | 1 | 3 | 3 | 29.39 | 30.86 | 31.20 | 46.02 | 63.46 | 52.53 | 61.78 | 50.43 | 36.27 | 46.75 | 48.79 | 57.56 | 82.31 | 78.00 | 16.88 |
| Roach | 1208 | 1 | 3 | 3 | 30.60 | 28.13 | 30.06 | 49.33 | 67.45 | 57.62 | 65.56 | 49.31 | 33.69 | 40.96 | 48.17 | 55.46 | 84.94 | 83.99 | 13.93 |
| Roach | 1209 | 1 | 3 | 3 | 24.87 | 26.23 | 26.28 | 38.81 | 52.49 | 40.46 | 50.77 | 41.23 | 30.50 | 39.60 | 44.77 | 44.49 | 71.97 | 67.91 | 12.24 |
| Roach | 1210 | 1 | 3 | 3 | 26.96 | 26.12 | 26.67 | 47.33 | 62.12 | 52.33 | 59.94 | 44.39 | 27.26 | 40.80 | 49.58 | 46.83 | 74.82 | 71.42 | 14.37 |
| Roach | 1211 | 1 | 3 | 3 | 23.80 | 24.60 | 24.03 | 43.36 | 53.05 | 39.95 | 50.61 | 37.17 | 29.08 | 36.03 | 42.55 | 40.36 | 60.87 | 57.98 | 11.89 |
| Roach | 1212 | 1 | 3 | 3 | 30.79 | 34.12 | 35.27 | 56.97 | 76.94 | 58.45 | 70.37 | 55.16 | 38.30 | 48.36 | 57.62 | 60.79 | 88.10 | 84.46 | 19.07 |
| Roach | 1213 | 1 | 3 | 3 | 27.71 | 23.87 | 31.07 | 58.26 | 77.28 | 64.35 | 72.51 | 55.63 | 34.78 | 45.75 | 56.08 | 57.34 | 83.78 | 82.82 | 15.98 |
| Roach | 1214 | 1 | 3 | 3 | 29.75 | 28.91 | 31.66 | 55.57 | 72.65 | 59.05 | 70.35 | 54.57 | 34.31 | 50.39 | 57.44 | 58.91 | 99.91 | 98.86 | 13.93 |
| Roach | 1215 | 1 | 3 | 3 | 28.03 | 23.06 | 25.42 | 44.00 | 58.47 | 46.95 | 51.04 | 38.88 | 28.26 | 36.06 | 42.74 | 45.94 | 69.36 | 68.81 | 12.34 |
| Roach | 1216 | 1 | 3 | 3 | 30.41 | 31.54 | 33.25 | 60.29 | 71.23 | 57.54 | 77.58 | 55.16 | 32.81 | 47.27 | 51.91 | 61.33 | 91.61 | 85.65 | 16.78 |
| Roach | 1217 | 1 | 3 | 3 | 31.14 | 32.66 | 35.75 | 56.99 | 76.65 | 67.33 | 72.60 | 49.49 | 31.14 | 38.56 | 46.18 | 54.09 | 83.17 | 81.63 | 16.60 |
| Roach | 1218 | 1 | 3 | 3 | 27.37 | 23.33 | 28.77 | 50.39 | 63.21 | 52.09 | 60.59 | 49.49 | 36.13 | 37.16 | 44.98 | 53.82 | 75.09 | 72.66 | 15.40 |
| Roach | 1219 | 1 | 3 | 3 | 33.64 | 29.72 | 32.58 | 54.89 | 70.43 | 56.51 | 64.98 | 48.39 | 35.00 | 34.78 | 43.47 | 58.70 | 84.18 | 80.78 | 15.73 |
| Roach | 1220 | 1 | 3 | 3 | 22.72 | 17.40 | 19.52 | 35.75 | 47.20 | 40.61 | 48.03 | 34.36 | 26.47 | 27.64 | 31.10 | 39.54 | 57.22 | 53.95 | 12.24 |
| Roach | 1001 | 2 | 1 | 1 | 14.91 | 14.69 | 16.20 | 30.90 | 40.68 | 29.60 | 35.82 | 28.97 | 23.36 | 28.09 | 30.62 | 33.79 | 52.04 | 46.69 | 9.44 |
| Roach | 1002 | 2 | 1 | 1 | 20.94 | 21.28 | 24.47 | 41.70 | 55.44 | 45.49 | 50.98 | 38.72 | 28.13 | 36.28 | 40.04 | 43.70 | 61.52 | 55.07 | 14.56 |
| Roach | 1003 | 2 | 1 | 1 | 21.31 | 23.28 | 24.22 | 45.32 | 62.23 | 49.66 | 55.55 | 45.62 | 30.86 | 35.62 | 41.86 | 51.13 | 71.55 | 70.84 | 13.41 |
| Roach | 1004 | 2 | 1 | 1 | 15.31 | 17.40 | 17.30 | 28.60 | 37.47 | 29.07 | 35.29 | 26.74 | 18.15 | 23.33 | 27.34 | 30.20 | 44.17 | 41.13 | 11.27 |
| Roach | 1005 | 2 | 1 | 1 | 19.12 | 19.97 | 20.57 | 44.57 | 55.78 | 45.67 | 48.64 | 39.39 | 31.20 | 29.34 | 34.24 | 43.54 | 59.78 | 56.00 | 14.26 |
| Roach | 1006 | 2 | 1 | 1 | 25.81 | 23.12 | 26.21 | 45.64 | 59.73 | 47.51 | 56.86 | 44.62 | 32.65 | 36.25 | 43.89 | 51.50 | 69.71 | 62.96 | 16.01 |
| Roach | 1007 | 2 | 1 | 1 | 13.91 | 17.26 | 17.68 | 30.20 | 44.10 | 35.87 | 37.17 | 29.93 | 19.64 | 23.62 | 29.16 | 33.65 | 49.26 | 47.89 | 10.44 |
| Roach | 1008 | 2 | 1 | 1 | 14.77 | 14.87 | 16.96 | 23.99 | 35.56 | 29.18 | 32.26 | 25.50 | 19.43 | 22.01 | 25.97 | 28.96 | 42.56 | 39.40 | 9.78 |
| Roach | 1009 | 2 | 1 | 1 | 15.81 | 15.44 | 16.96 | 31.15 | 39.51 | 32.33 | 38.35 | 28.80 | 22.32 | 24.72 | 29.92 | 33.48 | 48.14 | 43.37 | 10.69 |
| Roach | 1010 | 2 | 1 | 1 | 18.47 | 18.03 | 19.97 | 33.68 | 42.68 | 34.40 | 40.93 | 30.33 | 20.07 | 24.84 | 30.57 | 34.39 | 47.94 | 44.00 | 10.45 |
| Roach | 1011 | 2 | 1 | 1 | 16.80 | 14.23 | 16.38 | 28.62 | 35.10 | 28.22 | 32.78 | 25.20 | 20.03 | 25.54 | 29.38 | 28.99 | 43.87 | 41.25 | 9.63 |
| Roach | 1012 | 2 | 1 | 1 | 19.51 | 19.28 | 20.41 | 34.50 | 44.50 | 35.42 | 42.59 | 32.73 | 22.89 | 29.88 | 32.83 | 35.07 | 51.00 | 46.20 | 11.86 |
| Roach | 1013 | 2 | 1 | 1 | 17.54 | 16.76 | 19.70 | 26.84 | 36.11 | 31.27 | 38.15 | 28.27 | 18.97 | 28.84 | 31.60 | 32.14 | 50.49 | 47.23 | 11.27 |
| Roach | 1014 | 2 | 1 | 1 | 25.69 | 22.37 | 25.22 | 44.73 | 58.53 | 50.89 | 55.57 | 39.93 | 30.87 | 37.89 | 44.13 | 45.33 | 65.33 | 59.29 | 15.46 |
| Roach | 1015 | 2 | 1 | 1 | 25.05 | 24.71 | 28.41 | 39.20 | 59.84 | 48.61 | 53.60 | 43.72 | 30.76 | 34.35 | 41.78 | 46.65 | 69.46 | 64.61 | 15.88 |
| Roach | 1016 | 2 | 1 | 1 | 16.76 | 14.96 | 17.62 | 29.69 | 41.03 | 32.75 | 38.35 | 30.64 | 18.66 | 24.91 | 29.21 | 32.21 | 47.34 | 42.28 | 11.11 |
| Roach | 1017 | 2 | 1 | 1 | 14.90 | 13.87 | 13.99 | 22.41 | 29.93 | 24.09 | 28.75 | 22.08 | 16.25 | 20.04 | 22.85 | 24.84 | 38.67 | 35.69 | 8.32 |
| Roach | 1018 | 2 | 1 | 1 | 20.40 | 21.57 | 23.15 | 35.26 | 48.23 | 39.87 | 45.88 | 36.01 | 25.89 | 31.57 | 37.34 | 41.00 | 56.87 | 56.27 | 11.80 |
| Roach | 1019 | 2 | 1 | 1 | 16.77 | 15.66 | 15.87 | 28.88 | 39.94 | 33.88 | 40.26 | 29.80 | 20.79 | 24.03 | 27.74 | 32.12 | 47.95 | 42.95 | 10.82 |
| Roach | 1020 | 2 | 1 | 1 | 21.44 | 20.62 | 22.62 | 42.76 | 54.13 | 44.41 | 51.45 | 40.50 | 27.99 | 31.10 | 38.12 | 44.16 | 59.82 | 60.31 | 12.56 |
| Roach | 1021 | 2 | 1 | 1 | 18.75 | 18.94 | 21.22 | 36.97 | 49.61 | 39.74 | 45.53 | 35.04 | 23.94 | 30.33 | 37.83 | 39.62 | 56.36 | 54.24 | 13.87 |
| Roach | 1022 | 2 | 1 | 1 | 18.73 | 18.25 | 19.54 | 27.98 | 38.57 | 32.77 | 40.20 | 28.80 | 20.84 | 24.69 | 29.05 | 32.95 | 46.96 | 43.95 | 10.19 |
| Roach | 1023 | 2 | 1 | 1 | 16.70 | 15.39 | 16.62 | 26.62 | 37.18 | 30.75 | 32.99 | 24.94 | 20.34 | 23.59 | 27.53 | 27.87 | 43.99 | 40.74 | 7.81 |
| Roach | 1024 | 2 | 1 | 1 | 19.19 | 19.39 | 19.82 | 30.32 | 42.33 | 32.31 | 39.05 | 32.43 | 21.39 | 29.36 | 34.43 | 34.44 | 54.00 | 50.17 | 12.23 |
| Roach | 1025 | 2 | 1 | 1 | 16.81 | 17.32 | 19.20 | 32.14 | 44.84 | 37.15 | 41.67 | 33.63 | 21.57 | 26.76 | 32.00 | 34.21 | 49.62 | 45.66 | 12.14 |
| Roach | 1026 | 2 | 1 | 1 | 25.53 | 24.62 | 27.56 | 40.81 | 55.73 | 47.88 | 51.26 | 39.80 | 25.95 | 27.30 | 38.57 | 42.06 | 59.72 | 62.84 | 14.68 |
| Roach | 1027 | 2 | 1 | 1 | 20.65 | 17.11 | 19.84 | 33.22 | 45.06 | 38.34 | 44.75 | 32.41 | 27.40 | 30.34 | 33.39 | 36.40 | 52.87 | 47.26 | 12.01 |
| Roach | 1028 | 2 | 1 | 1 | 20.13 | 23.60 | 23.21 | 41.63 | 51.11 | 43.64 | 49.82 | 37.31 | 25.86 | 33.79 | 39.37 | 41.88 | 64.66 | 61.16 | 13.64 |
| Roach | 1029 | 2 | 1 | 1 | 18.80 | 20.36 | 21.60 | 36.78 | 50.08 | 40.78 | 44.61 | 34.38 | 27.73 | 22.41 | 31.58 | 41.07 | 56.64 | 56.22 | 13.20 |
| Roach | 1030 | 2 | 1 | 1 | 16.65 | 20.53 | 21.04 | 31.87 | 44.03 | 35.73 | 40.67 | 31.98 | 22.84 | 26.33 | 31.27 | 33.62 | 47.24 | 42.22 | 11.57 |
| Roach | 1001 | 2 | 1 | 2 | 15.02 | 15.41 | 18.17 | 29.53 | 38.86 | 30.67 | 34.84 | 28.52 | 21.85 | 29.95 | 30.96 | 34.29 | 51.29 | 45.85 | 9.83 |
| Roach | 1002 | 2 | 1 | 2 | 22.38 | 21.41 | 21.93 | 40.01 | 51.28 | 43.61 | 51.25 | 37.65 | 28.20 | 33.54 | 36.25 | 43.30 | 60.30 | 55.75 | 12.97 |
| Roach | 1003 | 2 | 1 | 2 | 21.68 | 24.18 | 25.61 | 46.63 | 62.94 | 50.81 | 55.20 | 43.03 | 30.93 | 34.72 | 44.73 | 52.21 | 74.92 | 74.21 | 15.12 |
| Roach | 1004 | 2 | 1 | 2 | 13.90 | 14.16 | 17.35 | 28.85 | 37.72 | 29.12 | 34.53 | 27.50 | 19.43 | 24.22 | 26.55 | 29.45 | 45.99 | 42.48 | 10.36 |
| Roach | 1005 | 2 | 1 | 2 | 18.94 | 19.95 | 21.45 | 40.89 | 54.45 | 44.74 | 50.55 | 39.31 | 28.83 | 28.62 | 36.29 | 42.40 | 57.03 | 54.09 | 14.89 |
| Roach | 1006 | 2 | 1 | 2 | 23.96 | 24.29 | 26.61 | 45.91 | 61.13 | 49.47 | 57.17 | 44.81 | 33.18 | 33.72 | 42.87 | 52.85 | 68.62 | 65.39 | 16.60 |
| Roach | 1007 | 2 | 1 | 2 | 14.87 | 17.19 | 18.33 | 32.21 | 43.78 | 36.05 | 38.47 | 30.20 | 20.01 | 21.56 | 28.22 | 33.95 | 48.90 | 46.52 | 10.70 |
| Roach | 1008 | 2 | 1 | 2 | 14.59 | 14.18 | 15.62 | 24.98 | 33.27 | 26.10 | 32.39 | 25.31 | 18.70 | 20.83 | 25.89 | 29.07 | 41.72 | 39.11 | 9.50 |
| Roach | 1009 | 2 | 1 | 2 | 17.52 | 16.99 | 19.09 | 28.97 | 39.07 | 32.51 | 37.91 | 28.09 | 23.06 | 22.16 | 27.88 | 33.41 | 47.42 | 43.96 | 9.60 |
| Roach | 1010 | 2 | 1 | 2 | 17.77 | 19.19 | 20.72 | 31.56 | 41.48 | 32.24 | 38.62 | 30.77 | 22.54 | 24.88 | 29.28 | 33.81 | 47.55 | 41.14 | 11.43 |
| Roach | 1011 | 2 | 1 | 2 | 15.60 | 14.66 | 16.40 | 26.82 | 34.25 | 28.64 | 33.19 | 26.12 | 19.41 | 23.06 | 28.60 | 28.45 | 43.10 | 41.12 | 10.52 |
| Roach | 1012 | 2 | 1 | 2 | 19.51 | 18.72 | 18.32 | 36.94 | 46.64 | 37.07 | 41.24 | 30.91 | 23.36 | 29.28 | 33.20 | 35.59 | 50.90 | 46.35 | 11.01 |
| Roach | 1013 | 2 | 1 | 2 | 18.88 | 17.56 | 18.69 | 29.32 | 40.54 | 34.19 | 38.04 | 28.65 | 18.03 | 27.75 | 33.30 | 30.57 | 49.63 | 44.90 | 11.17 |
| Roach | 1014 | 2 | 1 | 2 | 27.46 | 24.19 | 26.60 | 42.46 | 58.37 | 49.14 | 56.89 | 41.44 | 29.38 | 37.56 | 44.24 | 45.63 | 62.98 | 59.06 | 13.58 |
| Roach | 1015 | 2 | 1 | 2 | 25.94 | 24.93 | 28.61 | 39.62 | 60.87 | 48.95 | 52.65 | 41.86 | 29.76 | 35.81 | 42.41 | 44.82 | 65.93 | 61.16 | 16.01 |
| Roach | 1016 | 2 | 1 | 2 | 16.24 | 15.06 | 17.77 | 29.09 | 41.09 | 33.51 | 37.68 | 31.26 | 18.57 | 24.56 | 30.93 | 32.79 | 45.75 | 42.80 | 11.46 |
| Roach | 1017 | 2 | 1 | 2 | 12.63 | 13.40 | 13.52 | 23.58 | 33.24 | 27.14 | 29.67 | 22.50 | 16.72 | 20.05 | 22.28 | 26.26 | 37.84 | 35.15 | 8.30 |
| Roach | 1018 | 2 | 1 | 2 | 19.28 | 20.59 | 22.21 | 36.16 | 49.55 | 38.05 | 45.82 | 35.88 | 25.40 | 27.49 | 34.42 | 40.81 | 58.09 | 56.85 | 12.11 |
| Roach | 1019 | 2 | 1 | 2 | 19.77 | 16.65 | 16.84 | 34.22 | 43.24 | 37.27 | 39.38 | 30.03 | 20.86 | 24.10 | 30.28 | 32.58 | 49.18 | 45.15 | 11.60 |
| Roach | 1020 | 2 | 1 | 2 | 20.09 | 20.04 | 24.42 | 41.07 | 57.43 | 44.57 | 50.12 | 38.95 | 28.06 | 32.65 | 37.62 | 43.77 | 60.79 | 55.22 | 13.81 |
| Roach | 1021 | 2 | 1 | 2 | 19.81 | 18.98 | 20.80 | 38.90 | 50.16 | 41.19 | 44.93 | 35.73 | 26.06 | 28.47 | 36.28 | 39.67 | 57.71 | 53.95 | 13.24 |
| Roach | 1022 | 2 | 1 | 2 | 18.42 | 18.70 | 18.48 | 32.21 | 42.00 | 32.54 | 38.70 | 28.38 | 21.04 | 23.25 | 28.25 | 31.89 | 48.11 | 43.45 | 10.06 |
| Roach | 1023 | 2 | 1 | 2 | 15.12 | 14.85 | 15.33 | 27.00 | 35.29 | 27.82 | 32.33 | 24.67 | 21.64 | 23.21 | 25.41 | 27.66 | 43.09 | 38.48 | 8.05 |
| Roach | 1024 | 2 | 1 | 2 | 18.65 | 19.03 | 20.89 | 30.63 | 41.60 | 33.35 | 40.73 | 32.44 | 22.72 | 28.13 | 32.08 | 35.55 | 50.49 | 49.19 | 12.33 |
| Roach | 1025 | 2 | 1 | 2 | 14.51 | 15.03 | 18.28 | 32.32 | 41.61 | 33.05 | 40.89 | 32.86 | 21.35 | 27.16 | 33.68 | 35.16 | 51.93 | 48.77 | 12.50 |
| Roach | 1026 | 2 | 1 | 2 | 20.99 | 22.49 | 24.74 | 40.07 | 55.03 | 48.96 | 52.24 | 40.65 | 27.77 | 28.87 | 33.99 | 43.80 | 63.12 | 61.81 | 13.89 |
| Roach | 1027 | 2 | 1 | 2 | 20.93 | 19.53 | 21.32 | 33.81 | 45.67 | 37.46 | 42.37 | 32.88 | 26.89 | 30.74 | 34.65 | 36.88 | 52.11 | 45.35 | 12.30 |
| Roach | 1028 | 2 | 1 | 2 | 17.99 | 21.34 | 20.08 | 41.02 | 54.45 | 44.37 | 44.55 | 34.61 | 29.04 | 33.24 | 39.18 | 42.92 | 64.96 | 58.18 | 14.46 |
| Roach | 1029 | 2 | 1 | 2 | 17.85 | 19.69 | 20.75 | 38.21 | 47.63 | 39.34 | 45.55 | 34.23 | 25.06 | 24.91 | 31.76 | 39.20 | 53.69 | 53.62 | 12.18 |
| Roach | 1030 | 2 | 1 | 2 | 17.34 | 17.74 | 19.95 | 32.36 | 40.73 | 30.17 | 40.36 | 31.80 | 24.59 | 27.42 | 31.33 | 36.85 | 49.74 | 47.28 | 10.92 |
| Roach | 1001 | 2 | 1 | 3 | 16.02 | 17.01 | 17.27 | 31.40 | 40.86 | 32.67 | 37.45 | 30.31 | 21.36 | 28.92 | 31.79 | 34.68 | 51.73 | 47.14 | 10.55 |
| Roach | 1002 | 2 | 1 | 3 | 21.49 | 24.42 | 25.85 | 40.20 | 53.53 | 40.27 | 49.10 | 38.64 | 30.49 | 31.60 | 35.35 | 44.47 | 60.79 | 55.73 | 13.92 |
| Roach | 1003 | 2 | 1 | 3 | 23.22 | 23.39 | 24.86 | 45.64 | 62.18 | 49.53 | 55.86 | 45.23 | 30.67 | 34.64 | 43.12 | 52.93 | 74.60 | 75.52 | 14.32 |
| Roach | 1004 | 2 | 1 | 3 | 15.20 | 16.06 | 16.80 | 29.89 | 38.48 | 30.38 | 34.87 | 26.44 | 19.27 | 22.89 | 27.38 | 30.56 | 46.27 | 43.10 | 10.27 |
| Roach | 1005 | 2 | 1 | 3 | 19.60 | 19.86 | 21.62 | 42.13 | 53.15 | 42.91 | 50.39 | 37.83 | 28.78 | 28.17 | 36.31 | 42.49 | 59.85 | 54.94 | 12.91 |
| Roach | 1006 | 2 | 1 | 3 | 23.94 | 24.47 | 28.23 | 45.11 | 61.98 | 49.73 | 55.37 | 45.21 | 33.48 | 33.04 | 41.03 | 51.45 | 67.11 | 62.14 | 14.87 |
| Roach | 1007 | 2 | 1 | 3 | 15.03 | 19.02 | 18.60 | 30.01 | 43.92 | 34.86 | 36.41 | 29.98 | 20.52 | 22.32 | 29.07 | 33.37 | 49.08 | 46.65 | 11.74 |
| Roach | 1008 | 2 | 1 | 3 | 13.30 | 15.00 | 17.27 | 21.99 | 32.67 | 25.30 | 32.08 | 25.80 | 18.46 | 23.54 | 26.67 | 28.56 | 41.84 | 38.51 | 9.30 |
| Roach | 1009 | 2 | 1 | 3 | 16.52 | 15.72 | 17.57 | 32.88 | 42.13 | 35.63 | 39.45 | 28.96 | 21.68 | 23.63 | 28.61 | 33.39 | 47.73 | 43.22 | 10.42 |
| Roach | 1010 | 2 | 1 | 3 | 17.61 | 17.14 | 20.10 | 30.61 | 41.22 | 32.43 | 39.55 | 30.47 | 21.24 | 25.61 | 29.60 | 34.28 | 46.88 | 43.92 | 10.86 |
| Roach | 1011 | 2 | 1 | 3 | 15.39 | 13.84 | 16.20 | 28.62 | 35.73 | 29.84 | 33.31 | 26.27 | 19.18 | 25.55 | 29.29 | 29.88 | 46.49 | 41.65 | 10.21 |
| Roach | 1012 | 2 | 1 | 3 | 18.02 | 19.26 | 19.37 | 35.84 | 46.67 | 37.69 | 42.82 | 32.41 | 23.95 | 27.92 | 32.25 | 37.34 | 53.75 | 47.69 | 11.57 |
| Roach | 1013 | 2 | 1 | 3 | 19.48 | 16.59 | 18.43 | 30.97 | 39.46 | 32.28 | 38.45 | 29.00 | 18.83 | 26.39 | 30.67 | 32.18 | 47.52 | 44.54 | 10.76 |
| Roach | 1014 | 2 | 1 | 3 | 25.68 | 24.87 | 27.44 | 39.63 | 59.78 | 50.75 | 54.20 | 41.73 | 31.81 | 39.09 | 45.02 | 47.65 | 63.16 | 58.45 | 14.37 |
| Roach | 1015 | 2 | 1 | 3 | 23.74 | 24.93 | 28.83 | 38.04 | 59.28 | 50.03 | 52.37 | 43.08 | 29.38 | 34.59 | 41.92 | 44.44 | 66.30 | 61.27 | 12.82 |
| Roach | 1016 | 2 | 1 | 3 | 14.36 | 14.74 | 16.95 | 30.98 | 40.42 | 33.37 | 39.20 | 30.81 | 18.01 | 23.29 | 30.63 | 32.76 | 45.53 | 43.34 | 12.00 |
| Roach | 1017 | 2 | 1 | 3 | 13.38 | 13.38 | 14.80 | 24.04 | 31.21 | 26.05 | 29.90 | 22.62 | 15.77 | 20.01 | 22.63 | 25.10 | 38.88 | 35.79 | 7.80 |
| Roach | 1018 | 2 | 1 | 3 | 19.41 | 21.72 | 22.95 | 35.52 | 49.76 | 39.52 | 46.06 | 37.26 | 25.14 | 31.47 | 36.90 | 40.55 | 61.79 | 57.66 | 13.83 |
| Roach | 1019 | 2 | 1 | 3 | 18.70 | 18.53 | 18.81 | 31.48 | 44.57 | 36.81 | 41.43 | 30.37 | 20.37 | 23.09 | 30.43 | 33.65 | 47.95 | 45.88 | 12.00 |
| Roach | 1020 | 2 | 1 | 3 | 19.81 | 20.52 | 24.02 | 42.43 | 57.06 | 44.73 | 50.02 | 39.53 | 29.95 | 31.49 | 36.45 | 44.44 | 60.92 | 55.62 | 15.22 |
| Roach | 1021 | 2 | 1 | 3 | 17.37 | 19.49 | 21.80 | 37.46 | 51.54 | 43.94 | 45.27 | 34.84 | 25.50 | 29.10 | 37.07 | 38.83 | 56.38 | 55.08 | 13.38 |
| Roach | 1022 | 2 | 1 | 3 | 17.44 | 18.48 | 19.95 | 33.20 | 41.95 | 33.33 | 38.75 | 28.35 | 21.14 | 24.21 | 28.21 | 32.52 | 48.52 | 45.01 | 11.41 |
| Roach | 1023 | 2 | 1 | 3 | 16.29 | 15.17 | 15.48 | 27.09 | 36.57 | 29.62 | 33.22 | 24.63 | 19.88 | 24.59 | 28.75 | 27.31 | 41.21 | 39.68 | 8.86 |
| Roach | 1024 | 2 | 1 | 3 | 18.79 | 20.92 | 21.45 | 32.84 | 42.86 | 34.02 | 40.66 | 34.30 | 23.25 | 27.40 | 32.91 | 35.93 | 55.06 | 51.30 | 12.77 |
| Roach | 1025 | 2 | 1 | 3 | 15.93 | 16.34 | 19.24 | 33.12 | 43.00 | 35.30 | 40.96 | 31.81 | 23.30 | 25.81 | 30.24 | 35.62 | 50.51 | 48.04 | 12.00 |
| Roach | 1026 | 2 | 1 | 3 | 23.72 | 21.79 | 25.58 | 40.80 | 57.43 | 48.72 | 52.25 | 38.02 | 24.30 | 29.75 | 37.31 | 41.74 | 62.64 | 63.41 | 14.34 |
| Roach | 1027 | 2 | 1 | 3 | 20.79 | 19.35 | 21.75 | 33.54 | 45.31 | 35.07 | 43.00 | 33.15 | 26.05 | 31.80 | 33.81 | 36.68 | 51.56 | 45.97 | 9.81 |
| Roach | 1028 | 2 | 1 | 3 | 19.59 | 22.88 | 22.00 | 39.82 | 53.40 | 42.19 | 46.55 | 35.71 | 26.71 | 32.96 | 39.17 | 41.76 | 64.33 | 61.16 | 12.68 |
| Roach | 1029 | 2 | 1 | 3 | 18.19 | 20.05 | 21.98 | 34.42 | 47.59 | 40.47 | 44.41 | 33.90 | 26.78 | 25.37 | 32.94 | 39.18 | 55.55 | 57.56 | 11.88 |
| Roach | 1030 | 2 | 1 | 3 | 16.86 | 18.25 | 19.81 | 32.96 | 45.97 | 38.25 | 42.16 | 32.32 | 22.41 | 28.50 | 31.21 | 34.49 | 47.28 | 44.60 | 12.20 |
| Roach | 1101 | 2 | 2 | 1 | 33.11 | 29.98 | 35.66 | 66.70 | 88.53 | 74.17 | 77.81 | 64.46 | 48.91 | 47.37 | 62.47 | 69.79 | 100.43 | 99.33 | 23.92 |
| Roach | 1102 | 2 | 2 | 1 | 28.64 | 29.26 | 31.51 | 63.02 | 72.89 | 59.29 | 70.15 | 52.29 | 40.69 | 49.43 | 54.26 | 55.97 | 84.35 | 80.05 | 15.90 |
| Roach | 1103 | 2 | 2 | 1 | 30.74 | 30.48 | 34.52 | 61.36 | 78.44 | 61.32 | 72.37 | 54.31 | 42.87 | 40.33 | 51.97 | 61.90 | 87.16 | 83.44 | 17.95 |
| Roach | 1104 | 2 | 2 | 1 | 32.96 | 34.05 | 38.26 | 59.34 | 81.60 | 63.90 | 71.56 | 55.71 | 45.63 | 44.30 | 58.67 | 61.59 | 89.37 | 84.60 | 20.82 |
| Roach | 1105 | 2 | 2 | 1 | 31.71 | 30.06 | 31.51 | 58.13 | 78.02 | 61.56 | 71.98 | 53.71 | 37.14 | 43.45 | 53.18 | 58.75 | 87.53 | 79.54 | 18.42 |
| Roach | 1106 | 2 | 2 | 1 | 23.49 | 21.93 | 24.22 | 41.73 | 53.37 | 41.54 | 49.89 | 39.49 | 31.33 | 37.14 | 38.08 | 45.95 | 64.00 | 56.81 | 13.10 |
| Roach | 1107 | 2 | 2 | 1 | 29.30 | 26.47 | 27.92 | 58.61 | 69.57 | 55.30 | 63.66 | 49.48 | 41.72 | 45.07 | 52.78 | 55.82 | 80.17 | 75.40 | 17.12 |
| Roach | 1108 | 2 | 2 | 1 | 27.86 | 26.50 | 27.41 | 57.06 | 69.41 | 57.64 | 68.01 | 48.82 | 37.66 | 38.14 | 46.86 | 56.41 | 79.56 | 74.69 | 16.80 |
| Roach | 1109 | 2 | 2 | 1 | 29.53 | 29.85 | 33.17 | 54.49 | 71.50 | 59.12 | 68.50 | 54.91 | 41.56 | 38.89 | 50.02 | 63.46 | 88.32 | 84.94 | 18.32 |
| Roach | 1110 | 2 | 2 | 1 | 26.58 | 20.58 | 25.95 | 46.63 | 55.86 | 47.85 | 58.17 | 40.21 | 31.54 | 34.73 | 41.11 | 47.53 | 69.00 | 67.77 | 14.73 |
| Roach | 1111 | 2 | 2 | 1 | 20.25 | 26.45 | 24.53 | 50.21 | 65.54 | 53.15 | 59.33 | 45.69 | 35.88 | 38.84 | 47.93 | 51.92 | 75.55 | 76.86 | 17.42 |
| Roach | 1112 | 2 | 2 | 1 | 23.53 | 23.40 | 23.82 | 44.17 | 55.01 | 44.88 | 52.60 | 38.40 | 30.68 | 31.12 | 41.49 | 44.74 | 64.80 | 62.83 | 14.42 |
| Roach | 1113 | 2 | 2 | 1 | 27.89 | 30.63 | 31.55 | 51.89 | 69.13 | 55.20 | 63.59 | 48.81 | 37.44 | 37.62 | 47.61 | 55.39 | 74.74 | 76.28 | 17.27 |
| Roach | 1114 | 2 | 2 | 1 | 19.49 | 20.25 | 20.84 | 42.69 | 54.32 | 41.53 | 46.51 | 38.98 | 28.56 | 31.15 | 38.16 | 42.99 | 65.66 | 62.81 | 14.80 |
| Roach | 1115 | 2 | 2 | 1 | 21.87 | 26.53 | 29.07 | 45.38 | 63.86 | 53.61 | 59.43 | 46.24 | 30.60 | 36.89 | 48.18 | 50.42 | 74.86 | 69.96 | 16.51 |
| Roach | 1116 | 2 | 2 | 1 | 19.98 | 19.42 | 20.82 | 38.17 | 46.99 | 38.64 | 47.84 | 35.22 | 27.22 | 31.37 | 36.34 | 40.49 | 59.13 | 55.00 | 11.83 |
| Roach | 1117 | 2 | 2 | 1 | 25.59 | 24.71 | 25.05 | 44.84 | 53.79 | 41.84 | 53.05 | 42.00 | 33.74 | 29.59 | 39.61 | 51.00 | 66.03 | 68.86 | 15.00 |
| Roach | 1118 | 2 | 2 | 1 | 23.45 | 22.28 | 25.10 | 42.06 | 57.93 | 49.24 | 51.70 | 40.64 | 34.96 | 31.19 | 39.90 | 45.97 | 62.89 | 62.88 | 14.49 |
| Roach | 1119 | 2 | 2 | 1 | 26.81 | 21.14 | 23.26 | 47.22 | 56.29 | 49.90 | 59.19 | 42.02 | 32.02 | 36.50 | 44.97 | 46.25 | 68.20 | 64.81 | 16.25 |
| Roach | 1120 | 2 | 2 | 1 | 22.09 | 19.04 | 21.30 | 35.21 | 46.63 | 37.60 | 43.00 | 32.25 | 27.23 | 32.53 | 37.67 | 37.94 | 58.57 | 56.31 | 13.26 |
| Roach | 1121 | 2 | 2 | 1 | 24.35 | 24.15 | 22.91 | 42.83 | 54.14 | 46.00 | 50.91 | 36.63 | 27.24 | 31.63 | 36.84 | 40.25 | 61.55 | 58.65 | 13.00 |
| Roach | 1122 | 2 | 2 | 1 | 22.49 | 20.40 | 22.41 | 37.98 | 49.15 | 39.52 | 46.31 | 35.60 | 27.81 | 37.33 | 39.49 | 40.77 | 59.09 | 52.86 | 13.30 |
| Roach | 1123 | 2 | 2 | 1 | 20.36 | 19.93 | 21.25 | 41.54 | 50.29 | 39.61 | 47.86 | 34.87 | 29.87 | 32.62 | 38.08 | 39.75 | 57.69 | 50.91 | 13.85 |
| Roach | 1124 | 2 | 2 | 1 | 19.68 | 22.05 | 23.20 | 46.38 | 56.09 | 48.42 | 50.23 | 38.61 | 32.69 | 29.67 | 35.11 | 43.52 | 62.54 | 60.37 | 12.91 |
| Roach | 1125 | 2 | 2 | 1 | 21.74 | 18.85 | 21.04 | 36.54 | 47.28 | 37.50 | 40.24 | 32.74 | 29.21 | 29.92 | 35.54 | 35.78 | 53.42 | 51.09 | 12.24 |
| Roach | 1126 | 2 | 2 | 1 | 26.69 | 23.35 | 23.31 | 46.15 | 51.70 | 40.29 | 51.69 | 36.21 | 26.37 | 34.59 | 41.05 | 42.26 | 65.97 | 61.20 | 13.20 |
| Roach | 1127 | 2 | 2 | 1 | 26.38 | 25.94 | 29.40 | 57.37 | 69.93 | 58.45 | 66.08 | 50.73 | 44.51 | 42.40 | 49.03 | 60.32 | 85.58 | 76.70 | 18.25 |
| Roach | 1128 | 2 | 2 | 1 | 22.64 | 22.03 | 24.62 | 46.32 | 59.78 | 48.40 | 53.15 | 39.91 | 29.36 | 26.99 | 35.51 | 46.34 | 66.43 | 64.55 | 15.66 |
| Roach | 1129 | 2 | 2 | 1 | 25.38 | 22.41 | 24.78 | 38.11 | 51.14 | 45.21 | 51.03 | 36.90 | 24.97 | 33.61 | 38.52 | 42.03 | 65.67 | 64.93 | 12.22 |
| Roach | 1130 | 2 | 2 | 1 | 18.20 | 19.29 | 20.04 | 39.22 | 48.98 | 41.10 | 44.30 | 32.01 | 22.63 | 26.03 | 33.07 | 36.46 | 55.04 | 57.36 | 11.39 |
| Roach | 1101 | 2 | 2 | 2 | 28.20 | 29.64 | 39.30 | 63.03 | 87.51 | 75.72 | 84.54 | 65.73 | 43.79 | 52.43 | 63.60 | 73.81 | 101.24 | 103.15 | 25.22 |
| Roach | 1102 | 2 | 2 | 2 | 30.13 | 31.41 | 30.15 | 57.90 | 69.90 | 59.15 | 70.58 | 51.71 | 40.79 | 44.98 | 52.28 | 58.51 | 86.47 | 83.76 | 17.41 |
| Roach | 1103 | 2 | 2 | 2 | 32.58 | 30.06 | 33.30 | 62.15 | 76.32 | 60.55 | 74.50 | 55.90 | 42.86 | 40.52 | 56.29 | 62.46 | 87.28 | 82.79 | 21.06 |
| Roach | 1104 | 2 | 2 | 2 | 31.97 | 36.28 | 43.66 | 63.09 | 79.38 | 62.88 | 72.96 | 58.08 | 45.37 | 45.08 | 50.86 | 65.52 | 90.79 | 86.34 | 20.26 |
| Roach | 1105 | 2 | 2 | 2 | 29.65 | 30.59 | 33.47 | 62.32 | 80.23 | 61.28 | 69.58 | 53.44 | 39.34 | 43.89 | 50.02 | 59.70 | 87.03 | 81.91 | 18.99 |
| Roach | 1106 | 2 | 2 | 2 | 23.02 | 21.67 | 23.41 | 40.76 | 56.88 | 45.41 | 50.35 | 40.58 | 31.19 | 36.50 | 42.93 | 44.53 | 64.74 | 59.95 | 15.41 |
| Roach | 1107 | 2 | 2 | 2 | 26.54 | 27.04 | 29.91 | 59.02 | 72.55 | 60.62 | 66.62 | 49.00 | 40.61 | 45.27 | 53.23 | 57.12 | 86.43 | 76.63 | 17.57 |
| Roach | 1108 | 2 | 2 | 2 | 27.86 | 28.11 | 29.41 | 55.81 | 70.77 | 56.26 | 65.99 | 51.22 | 38.77 | 39.60 | 45.42 | 56.66 | 78.92 | 73.55 | 16.41 |
| Roach | 1109 | 2 | 2 | 2 | 30.62 | 27.66 | 33.26 | 55.04 | 71.51 | 58.69 | 67.75 | 54.74 | 38.73 | 42.81 | 52.86 | 60.01 | 81.68 | 79.12 | 17.10 |
| Roach | 1110 | 2 | 2 | 2 | 24.19 | 17.58 | 22.87 | 44.15 | 56.47 | 47.13 | 52.57 | 41.35 | 36.19 | 35.49 | 41.54 | 44.49 | 67.85 | 64.81 | 14.13 |
| Roach | 1111 | 2 | 2 | 2 | 23.64 | 26.90 | 27.61 | 47.27 | 62.44 | 48.52 | 57.63 | 45.10 | 39.04 | 37.21 | 46.78 | 52.38 | 76.18 | 73.83 | 17.43 |
| Roach | 1112 | 2 | 2 | 2 | 23.92 | 21.92 | 23.90 | 43.51 | 57.45 | 46.43 | 51.22 | 38.63 | 32.40 | 30.88 | 39.17 | 43.87 | 63.61 | 63.02 | 13.74 |
| Roach | 1113 | 2 | 2 | 2 | 31.87 | 30.87 | 31.23 | 53.42 | 68.35 | 53.67 | 65.32 | 50.72 | 35.78 | 38.37 | 50.76 | 55.03 | 76.64 | 74.89 | 16.60 |
| Roach | 1114 | 2 | 2 | 2 | 19.42 | 21.14 | 23.00 | 40.78 | 54.37 | 44.15 | 46.84 | 38.44 | 27.81 | 33.44 | 40.87 | 43.26 | 64.76 | 59.77 | 14.95 |
| Roach | 1115 | 2 | 2 | 2 | 24.98 | 25.81 | 26.84 | 49.25 | 64.27 | 52.96 | 58.17 | 44.71 | 31.53 | 36.86 | 48.20 | 46.24 | 76.31 | 69.65 | 16.01 |
| Roach | 1116 | 2 | 2 | 2 | 20.53 | 19.88 | 21.56 | 39.58 | 48.30 | 42.32 | 47.65 | 34.37 | 27.69 | 31.13 | 38.39 | 38.69 | 57.05 | 54.17 | 11.83 |
| Roach | 1117 | 2 | 2 | 2 | 22.03 | 23.52 | 26.65 | 50.80 | 56.77 | 46.28 | 54.42 | 39.96 | 34.54 | 31.88 | 40.11 | 48.64 | 63.77 | 65.67 | 15.00 |
| Roach | 1118 | 2 | 2 | 2 | 22.04 | 22.39 | 23.71 | 42.18 | 56.89 | 47.78 | 53.62 | 38.82 | 30.78 | 34.64 | 40.07 | 43.79 | 65.88 | 62.10 | 14.61 |
| Roach | 1119 | 2 | 2 | 2 | 25.63 | 23.08 | 25.60 | 42.57 | 57.61 | 45.64 | 55.35 | 43.20 | 35.43 | 34.62 | 42.37 | 47.06 | 70.12 | 61.84 | 14.85 |
| Roach | 1120 | 2 | 2 | 2 | 20.65 | 21.03 | 21.46 | 37.46 | 46.42 | 36.60 | 44.66 | 30.99 | 26.60 | 30.62 | 36.45 | 39.04 | 59.08 | 59.02 | 13.60 |
| Roach | 1121 | 2 | 2 | 2 | 21.74 | 23.30 | 23.77 | 42.83 | 53.99 | 44.72 | 51.97 | 36.81 | 27.79 | 31.44 | 39.32 | 40.77 | 62.26 | 59.77 | 15.02 |
| Roach | 1122 | 2 | 2 | 2 | 21.49 | 21.25 | 22.66 | 41.11 | 50.83 | 38.19 | 45.46 | 34.46 | 29.42 | 35.79 | 38.94 | 40.95 | 59.03 | 54.65 | 12.91 |
| Roach | 1123 | 2 | 2 | 2 | 18.64 | 20.97 | 22.31 | 42.56 | 51.12 | 41.88 | 48.69 | 33.42 | 27.51 | 33.47 | 39.05 | 41.21 | 59.41 | 53.80 | 14.51 |
| Roach | 1124 | 2 | 2 | 2 | 21.66 | 20.21 | 24.32 | 40.85 | 58.30 | 49.92 | 49.68 | 38.44 | 30.24 | 30.58 | 36.80 | 42.25 | 62.71 | 57.86 | 13.02 |
| Roach | 1125 | 2 | 2 | 2 | 20.05 | 20.65 | 21.54 | 35.77 | 47.81 | 34.52 | 37.75 | 31.26 | 29.05 | 31.00 | 34.82 | 37.56 | 54.68 | 53.28 | 12.82 |
| Roach | 1126 | 2 | 2 | 2 | 26.60 | 23.60 | 22.71 | 45.32 | 50.55 | 40.75 | 49.49 | 34.62 | 28.22 | 34.07 | 40.50 | 41.61 | 62.96 | 60.24 | 13.80 |
| Roach | 1127 | 2 | 2 | 2 | 25.05 | 25.38 | 26.71 | 61.61 | 70.32 | 56.87 | 65.97 | 50.99 | 43.11 | 43.38 | 52.30 | 59.46 | 85.30 | 77.18 | 18.80 |
| Roach | 1128 | 2 | 2 | 2 | 25.53 | 21.10 | 22.96 | 46.07 | 56.53 | 46.70 | 54.49 | 41.11 | 27.00 | 30.26 | 38.67 | 44.29 | 64.03 | 64.87 | 14.36 |
| Roach | 1129 | 2 | 2 | 2 | 24.96 | 20.29 | 26.95 | 36.62 | 52.62 | 47.65 | 49.12 | 35.81 | 27.98 | 29.42 | 35.58 | 46.58 | 70.65 | 68.65 | 13.29 |
| Roach | 1130 | 2 | 2 | 2 | 18.36 | 21.76 | 22.06 | 41.70 | 47.55 | 39.95 | 45.44 | 30.01 | 21.83 | 23.20 | 31.00 | 37.21 | 52.49 | 52.19 | 11.86 |
| Roach | 1101 | 2 | 2 | 3 | 35.83 | 33.95 | 37.36 | 63.62 | 86.27 | 72.15 | 80.27 | 63.91 | 47.73 | 50.67 | 61.83 | 74.92 | 103.66 | 106.10 | 26.34 |
| Roach | 1102 | 2 | 2 | 3 | 29.29 | 32.87 | 31.65 | 61.25 | 72.23 | 57.12 | 68.76 | 51.08 | 39.13 | 46.61 | 53.01 | 56.37 | 85.58 | 83.57 | 17.74 |
| Roach | 1103 | 2 | 2 | 3 | 37.03 | 34.83 | 35.81 | 63.14 | 78.64 | 64.97 | 72.68 | 54.97 | 46.72 | 40.90 | 51.73 | 61.90 | 82.71 | 83.92 | 20.18 |
| Roach | 1104 | 2 | 2 | 3 | 37.98 | 36.65 | 39.08 | 61.24 | 82.36 | 64.17 | 72.20 | 59.45 | 45.89 | 46.99 | 53.21 | 68.18 | 98.57 | 91.82 | 18.65 |
| Roach | 1105 | 2 | 2 | 3 | 28.99 | 31.40 | 31.92 | 61.15 | 77.41 | 60.32 | 69.44 | 55.57 | 39.37 | 42.21 | 52.60 | 60.79 | 86.82 | 80.85 | 19.91 |
| Roach | 1106 | 2 | 2 | 3 | 22.90 | 22.03 | 25.24 | 43.18 | 53.73 | 41.24 | 50.89 | 40.76 | 31.61 | 37.13 | 40.24 | 47.11 | 67.58 | 59.86 | 14.26 |
| Roach | 1107 | 2 | 2 | 3 | 25.55 | 29.66 | 30.54 | 56.43 | 67.49 | 55.12 | 65.79 | 48.98 | 40.99 | 44.64 | 52.71 | 56.59 | 78.11 | 75.66 | 17.53 |
| Roach | 1108 | 2 | 2 | 3 | 25.58 | 25.80 | 29.41 | 55.58 | 71.09 | 57.08 | 65.85 | 48.40 | 38.41 | 41.02 | 48.77 | 55.51 | 79.00 | 74.96 | 15.60 |
| Roach | 1109 | 2 | 2 | 3 | 27.64 | 28.64 | 33.90 | 53.92 | 71.99 | 57.29 | 67.91 | 54.94 | 39.96 | 42.35 | 52.10 | 61.22 | 83.85 | 81.64 | 19.19 |
| Roach | 1110 | 2 | 2 | 3 | 25.90 | 19.02 | 24.38 | 47.55 | 60.08 | 50.96 | 58.58 | 41.21 | 33.00 | 38.86 | 41.36 | 43.98 | 66.90 | 67.00 | 15.01 |
| Roach | 1111 | 2 | 2 | 3 | 23.52 | 27.25 | 28.23 | 47.68 | 63.38 | 48.88 | 57.14 | 44.46 | 36.38 | 41.44 | 45.85 | 53.07 | 75.67 | 73.44 | 15.62 |
| Roach | 1112 | 2 | 2 | 3 | 21.17 | 22.63 | 25.55 | 42.47 | 55.35 | 45.07 | 50.82 | 40.04 | 32.85 | 33.08 | 39.84 | 44.56 | 64.85 | 61.93 | 14.42 |
| Roach | 1113 | 2 | 2 | 3 | 26.81 | 31.00 | 30.15 | 53.03 | 69.67 | 55.91 | 62.29 | 48.54 | 39.17 | 37.84 | 46.21 | 51.93 | 73.80 | 68.63 | 17.77 |
| Roach | 1114 | 2 | 2 | 3 | 19.35 | 23.37 | 22.01 | 40.41 | 55.74 | 45.02 | 48.53 | 39.23 | 27.53 | 30.35 | 37.11 | 43.77 | 65.55 | 62.68 | 14.68 |
| Roach | 1115 | 2 | 2 | 3 | 18.83 | 25.29 | 26.74 | 51.69 | 63.51 | 53.89 | 54.77 | 46.83 | 31.77 | 38.34 | 48.44 | 51.13 | 75.46 | 70.20 | 16.30 |
| Roach | 1116 | 2 | 2 | 3 | 20.68 | 16.60 | 20.00 | 40.57 | 49.68 | 40.03 | 47.05 | 34.77 | 26.35 | 31.13 | 37.62 | 39.30 | 58.91 | 55.00 | 11.81 |
| Roach | 1117 | 2 | 2 | 3 | 24.74 | 24.55 | 25.76 | 47.36 | 59.18 | 45.41 | 55.45 | 43.51 | 33.54 | 29.51 | 40.70 | 47.89 | 64.54 | 64.86 | 15.88 |
| Roach | 1118 | 2 | 2 | 3 | 23.21 | 25.80 | 25.46 | 38.75 | 55.63 | 48.42 | 52.35 | 40.06 | 32.56 | 33.74 | 42.23 | 46.03 | 65.09 | 64.29 | 14.61 |
| Roach | 1119 | 2 | 2 | 3 | 25.24 | 23.11 | 25.00 | 47.23 | 57.60 | 48.31 | 55.37 | 42.86 | 33.42 | 38.23 | 45.65 | 45.61 | 64.74 | 61.92 | 15.27 |
| Roach | 1120 | 2 | 2 | 3 | 21.91 | 18.32 | 21.28 | 34.10 | 45.17 | 36.35 | 42.81 | 31.64 | 27.95 | 30.72 | 38.51 | 38.36 | 60.41 | 57.76 | 13.41 |
| Roach | 1121 | 2 | 2 | 3 | 25.24 | 24.80 | 23.35 | 41.94 | 52.76 | 45.24 | 51.88 | 36.21 | 26.15 | 31.63 | 39.57 | 42.81 | 64.59 | 60.35 | 15.03 |
| Roach | 1122 | 2 | 2 | 3 | 19.21 | 18.92 | 22.49 | 36.70 | 48.13 | 38.77 | 45.41 | 33.61 | 30.55 | 35.08 | 38.49 | 43.09 | 63.22 | 57.84 | 12.63 |
| Roach | 1123 | 2 | 2 | 3 | 20.09 | 18.16 | 19.83 | 41.97 | 50.19 | 39.55 | 46.70 | 34.69 | 27.89 | 33.69 | 38.11 | 40.36 | 60.53 | 53.79 | 12.85 |
| Roach | 1124 | 2 | 2 | 3 | 19.50 | 22.93 | 23.01 | 42.36 | 56.89 | 48.03 | 49.98 | 38.21 | 31.83 | 32.03 | 35.98 | 42.39 | 62.71 | 59.24 | 14.21 |
| Roach | 1125 | 2 | 2 | 3 | 20.34 | 20.75 | 21.28 | 34.27 | 48.09 | 38.18 | 40.61 | 31.60 | 25.43 | 31.11 | 35.75 | 36.02 | 54.49 | 54.36 | 12.91 |
| Roach | 1126 | 2 | 2 | 3 | 25.44 | 23.51 | 24.62 | 41.78 | 50.22 | 40.30 | 51.99 | 34.60 | 26.82 | 36.44 | 40.95 | 41.81 | 63.52 | 60.22 | 14.42 |
| Roach | 1127 | 2 | 2 | 3 | 29.07 | 30.06 | 30.26 | 57.03 | 70.27 | 53.37 | 65.74 | 49.81 | 42.37 | 42.90 | 52.90 | 57.86 | 80.07 | 75.03 | 18.16 |
| Roach | 1128 | 2 | 2 | 3 | 26.32 | 24.15 | 23.89 | 43.47 | 54.74 | 46.97 | 56.65 | 41.65 | 27.30 | 31.97 | 38.91 | 44.89 | 63.56 | 63.38 | 16.38 |
| Roach | 1129 | 2 | 2 | 3 | 26.03 | 24.31 | 26.66 | 36.61 | 53.89 | 46.87 | 53.07 | 35.92 | 22.70 | 32.74 | 35.48 | 44.11 | 67.77 | 67.92 | 12.09 |
| Roach | 1130 | 2 | 2 | 3 | 15.38 | 18.82 | 19.65 | 42.61 | 50.58 | 40.90 | 44.67 | 30.00 | 24.42 | 23.83 | 30.06 | 38.37 | 53.70 | 55.90 | 11.66 |
| Roach | 1191 | 2 | 3 | 1 | 28.37 | 32.01 | 33.42 | 71.67 | 84.95 | 69.35 | 81.02 | 58.26 | 42.35 | 46.70 | 56.75 | 65.47 | 95.45 | 91.19 | 19.24 |
| Roach | 1192 | 2 | 3 | 1 | 25.23 | 29.16 | 33.54 | 55.37 | 79.14 | 63.38 | 71.90 | 59.50 | 40.50 | 51.63 | 59.30 | 67.71 | 94.50 | 89.15 | 20.14 |
| Roach | 1193 | 2 | 3 | 1 | 28.96 | 26.38 | 31.23 | 56.90 | 71.92 | 56.47 | 67.70 | 55.23 | 36.41 | 41.73 | 41.73 | 59.97 | 89.85 | 85.48 | 18.31 |
| Roach | 1194 | 2 | 3 | 1 | 24.70 | 26.19 | 25.55 | 50.62 | 61.51 | 48.72 | 58.81 | 45.30 | 30.60 | 39.06 | 43.58 | 48.18 | 68.67 | 62.55 | 15.00 |
| Roach | 1195 | 2 | 3 | 1 | 26.76 | 26.57 | 29.40 | 57.58 | 71.18 | 58.43 | 68.98 | 52.43 | 42.52 | 41.84 | 48.62 | 59.29 | 78.60 | 74.55 | 16.27 |
| Roach | 1196 | 2 | 3 | 1 | 32.38 | 33.12 | 36.00 | 58.36 | 75.16 | 59.49 | 68.89 | 54.63 | 41.60 | 38.89 | 52.39 | 62.71 | 84.47 | 83.27 | 19.32 |
| Roach | 1197 | 2 | 3 | 1 | 36.88 | 36.61 | 39.75 | 70.80 | 92.34 | 81.91 | 96.21 | 71.77 | 43.41 | 42.46 | 57.51 | 76.70 | 99.65 | 96.51 | 22.51 |
| Roach | 1198 | 2 | 3 | 1 | 23.48 | 25.68 | 29.59 | 53.08 | 66.95 | 53.85 | 61.62 | 46.48 | 35.75 | 35.30 | 44.86 | 50.89 | 70.72 | 65.73 | 14.71 |
| Roach | 1199 | 2 | 3 | 1 | 25.64 | 29.53 | 29.96 | 54.32 | 67.04 | 53.96 | 64.27 | 49.26 | 36.07 | 45.41 | 52.92 | 56.53 | 82.59 | 84.20 | 17.10 |
| Roach | 1200 | 2 | 3 | 1 | 29.35 | 26.70 | 31.83 | 63.56 | 76.81 | 66.20 | 70.99 | 54.11 | 42.95 | 49.81 | 52.06 | 59.89 | 84.54 | 77.14 | 17.77 |
| Roach | 1201 | 2 | 3 | 1 | 33.77 | 34.32 | 38.78 | 66.95 | 84.46 | 62.71 | 75.58 | 59.71 | 49.18 | 49.25 | 59.53 | 62.99 | 85.14 | 79.07 | 20.48 |
| Roach | 1202 | 2 | 3 | 1 | 22.79 | 29.30 | 31.68 | 51.18 | 70.65 | 60.92 | 64.09 | 49.47 | 31.94 | 37.50 | 46.52 | 53.07 | 73.43 | 67.27 | 15.65 |
| Roach | 1203 | 2 | 3 | 1 | 24.43 | 26.59 | 27.62 | 47.82 | 70.14 | 57.60 | 59.07 | 47.71 | 34.54 | 38.53 | 49.04 | 51.45 | 73.95 | 72.90 | 14.48 |
| Roach | 1204 | 2 | 3 | 1 | 25.83 | 27.38 | 30.81 | 52.57 | 70.01 | 55.70 | 61.96 | 48.63 | 39.77 | 34.55 | 44.43 | 54.95 | 76.76 | 73.83 | 14.75 |
| Roach | 1205 | 2 | 3 | 1 | 30.63 | 30.87 | 31.77 | 59.32 | 73.15 | 59.09 | 68.74 | 51.33 | 34.17 | 37.96 | 47.16 | 57.12 | 78.06 | 76.65 | 18.61 |
| Roach | 1206 | 2 | 3 | 1 | 30.82 | 29.38 | 33.62 | 62.33 | 77.14 | 60.71 | 72.76 | 57.01 | 38.48 | 46.86 | 54.88 | 65.05 | 96.65 | 89.19 | 19.20 |
| Roach | 1207 | 2 | 3 | 1 | 28.15 | 30.60 | 30.78 | 50.41 | 59.00 | 47.37 | 61.03 | 49.52 | 39.62 | 41.26 | 48.52 | 59.41 | 80.27 | 76.94 | 15.60 |
| Roach | 1208 | 2 | 3 | 1 | 31.41 | 28.01 | 30.78 | 52.52 | 70.45 | 62.36 | 63.47 | 51.61 | 34.35 | 43.28 | 53.15 | 56.82 | 84.57 | 83.77 | 17.97 |
| Roach | 1209 | 2 | 3 | 1 | 24.42 | 25.32 | 24.75 | 43.71 | 53.32 | 44.62 | 50.96 | 40.22 | 30.54 | 40.70 | 44.52 | 43.11 | 65.94 | 62.33 | 13.58 |
| Roach | 1210 | 2 | 3 | 1 | 23.98 | 27.17 | 27.36 | 51.99 | 61.87 | 56.49 | 63.24 | 43.53 | 30.26 | 34.83 | 42.35 | 46.43 | 69.52 | 65.82 | 14.78 |
| Roach | 1211 | 2 | 3 | 1 | 24.24 | 22.62 | 21.61 | 41.49 | 51.11 | 46.19 | 52.93 | 35.56 | 33.07 | 31.71 | 35.22 | 43.11 | 60.64 | 56.07 | 14.36 |
| Roach | 1212 | 2 | 3 | 1 | 30.00 | 34.89 | 36.05 | 59.14 | 77.26 | 61.21 | 72.83 | 56.43 | 39.61 | 44.45 | 54.79 | 63.27 | 86.60 | 81.95 | 19.50 |
| Roach | 1213 | 2 | 3 | 1 | 21.14 | 23.63 | 27.68 | 59.21 | 73.91 | 61.50 | 73.76 | 54.49 | 34.93 | 44.40 | 53.47 | 59.09 | 84.24 | 80.99 | 16.93 |
| Roach | 1214 | 2 | 3 | 1 | 29.39 | 28.33 | 30.02 | 54.27 | 71.53 | 60.64 | 69.27 | 54.31 | 40.91 | 36.72 | 49.10 | 60.73 | 86.96 | 85.30 | 17.61 |
| Roach | 1215 | 2 | 3 | 1 | 23.72 | 17.53 | 22.82 | 43.80 | 57.71 | 48.71 | 52.98 | 39.25 | 29.23 | 38.63 | 42.28 | 44.43 | 71.29 | 67.58 | 13.12 |
| Roach | 1216 | 2 | 3 | 1 | 29.81 | 33.17 | 33.36 | 60.14 | 72.15 | 52.70 | 64.54 | 51.71 | 46.60 | 43.23 | 53.67 | 61.63 | 85.79 | 84.20 | 18.01 |
| Roach | 1217 | 2 | 3 | 1 | 31.93 | 33.13 | 33.90 | 60.95 | 77.68 | 64.71 | 69.68 | 49.81 | 38.01 | 37.62 | 46.44 | 58.21 | 81.51 | 80.31 | 16.27 |
| Roach | 1218 | 2 | 3 | 1 | 27.69 | 26.97 | 28.83 | 48.84 | 64.24 | 55.75 | 61.70 | 48.96 | 35.81 | 35.42 | 43.17 | 53.76 | 73.65 | 70.70 | 13.77 |
| Roach | 1219 | 2 | 3 | 1 | 32.95 | 27.78 | 32.19 | 50.19 | 66.08 | 51.45 | 63.00 | 50.71 | 37.47 | 33.83 | 45.35 | 58.27 | 79.29 | 79.54 | 16.89 |
| Roach | 1220 | 2 | 3 | 1 | 21.97 | 18.34 | 19.40 | 41.19 | 47.95 | 40.72 | 47.10 | 33.81 | 24.34 | 29.74 | 34.52 | 37.83 | 52.97 | 50.12 | 11.69 |
| Roach | 1191 | 2 | 3 | 2 | 28.77 | 31.53 | 34.13 | 68.70 | 84.23 | 65.27 | 81.11 | 58.30 | 45.66 | 46.24 | 55.09 | 65.65 | 92.60 | 86.45 | 21.01 |
| Roach | 1192 | 2 | 3 | 2 | 21.67 | 28.22 | 30.14 | 60.96 | 81.60 | 68.70 | 73.59 | 59.44 | 41.95 | 47.53 | 58.67 | 64.87 | 90.15 | 84.64 | 21.06 |
| Roach | 1193 | 2 | 3 | 2 | 26.47 | 29.03 | 29.45 | 57.88 | 74.37 | 58.32 | 68.16 | 54.93 | 36.85 | 39.72 | 48.23 | 61.09 | 88.13 | 82.45 | 17.27 |
| Roach | 1194 | 2 | 3 | 2 | 25.05 | 25.63 | 28.56 | 53.05 | 63.82 | 50.67 | 59.83 | 44.43 | 29.48 | 40.61 | 44.53 | 47.52 | 72.65 | 66.85 | 14.70 |
| Roach | 1195 | 2 | 3 | 2 | 26.89 | 29.39 | 31.54 | 56.69 | 74.95 | 60.20 | 67.87 | 52.21 | 40.45 | 43.65 | 51.61 | 60.47 | 83.52 | 79.07 | 17.44 |
| Roach | 1196 | 2 | 3 | 2 | 34.06 | 31.62 | 36.02 | 57.40 | 74.70 | 56.50 | 71.41 | 54.60 | 39.66 | 42.07 | 54.25 | 61.29 | 84.78 | 81.15 | 20.37 |
| Roach | 1197 | 2 | 3 | 2 | 42.91 | 34.42 | 41.37 | 81.52 | 98.36 | 74.32 | 93.89 | 71.99 | 48.84 | 36.28 | 51.57 | 79.89 | 101.09 | 98.80 | 19.85 |
| Roach | 1198 | 2 | 3 | 2 | 24.84 | 26.05 | 27.38 | 49.56 | 63.30 | 51.11 | 61.64 | 44.85 | 31.59 | 38.95 | 44.27 | 49.96 | 69.56 | 65.58 | 14.73 |
| Roach | 1199 | 2 | 3 | 2 | 27.21 | 28.88 | 32.41 | 49.18 | 63.59 | 52.49 | 63.13 | 47.52 | 38.70 | 43.88 | 49.71 | 57.59 | 88.10 | 83.10 | 16.87 |
| Roach | 1200 | 2 | 3 | 2 | 28.54 | 27.95 | 33.90 | 58.51 | 76.15 | 62.20 | 68.53 | 55.29 | 48.97 | 42.12 | 49.99 | 64.38 | 88.92 | 81.38 | 15.90 |
| Roach | 1201 | 2 | 3 | 2 | 24.83 | 35.98 | 36.31 | 62.27 | 80.24 | 62.63 | 75.96 | 61.44 | 46.81 | 48.98 | 58.77 | 66.61 | 89.14 | 83.21 | 19.86 |
| Roach | 1202 | 2 | 3 | 2 | 24.02 | 26.95 | 30.01 | 47.52 | 70.80 | 60.71 | 66.44 | 49.06 | 30.73 | 34.30 | 45.28 | 51.94 | 73.35 | 67.08 | 16.34 |
| Roach | 1203 | 2 | 3 | 2 | 28.21 | 27.93 | 30.18 | 47.71 | 66.21 | 52.50 | 63.75 | 50.01 | 32.07 | 38.91 | 44.89 | 53.32 | 75.22 | 71.33 | 15.01 |
| Roach | 1204 | 2 | 3 | 2 | 26.05 | 22.39 | 29.18 | 52.24 | 67.68 | 55.04 | 62.62 | 48.95 | 38.08 | 42.07 | 48.36 | 54.76 | 73.82 | 72.66 | 17.17 |
| Roach | 1205 | 2 | 3 | 2 | 27.79 | 29.12 | 29.71 | 57.28 | 66.98 | 51.96 | 66.55 | 54.39 | 39.58 | 39.45 | 46.58 | 54.13 | 80.23 | 75.12 | 17.70 |
| Roach | 1206 | 2 | 3 | 2 | 32.31 | 31.68 | 33.62 | 56.66 | 77.26 | 60.76 | 69.79 | 54.31 | 40.72 | 40.57 | 53.55 | 64.48 | 91.93 | 88.33 | 18.01 |
| Roach | 1207 | 2 | 3 | 2 | 25.89 | 25.99 | 31.21 | 48.66 | 65.09 | 51.45 | 61.15 | 48.61 | 36.00 | 43.02 | 52.68 | 58.11 | 88.17 | 79.39 | 22.07 |
| Roach | 1208 | 2 | 3 | 2 | 27.51 | 27.81 | 31.66 | 50.11 | 70.86 | 58.01 | 62.11 | 51.09 | 33.91 | 39.96 | 50.50 | 55.93 | 83.06 | 82.16 | 18.63 |
| Roach | 1209 | 2 | 3 | 2 | 25.90 | 26.43 | 24.35 | 40.01 | 51.61 | 44.10 | 50.61 | 39.38 | 34.50 | 34.66 | 40.07 | 47.37 | 67.95 | 64.75 | 15.97 |
| Roach | 1210 | 2 | 3 | 2 | 26.65 | 24.30 | 25.62 | 51.13 | 64.08 | 55.74 | 61.29 | 42.63 | 27.97 | 38.11 | 44.15 | 46.74 | 71.38 | 69.35 | 15.24 |
| Roach | 1211 | 2 | 3 | 2 | 27.97 | 25.35 | 24.27 | 43.54 | 49.76 | 42.69 | 48.85 | 34.50 | 32.12 | 30.20 | 39.57 | 43.11 | 61.21 | 61.12 | 11.84 |
| Roach | 1212 | 2 | 3 | 2 | 32.16 | 34.58 | 35.10 | 57.53 | 75.26 | 62.20 | 73.77 | 55.56 | 39.84 | 50.04 | 59.50 | 63.59 | 88.49 | 83.97 | 18.69 |
| Roach | 1213 | 2 | 3 | 2 | 24.55 | 22.40 | 32.95 | 59.52 | 75.45 | 62.45 | 73.44 | 52.70 | 38.01 | 36.63 | 51.79 | 61.25 | 87.08 | 84.08 | 18.01 |
| Roach | 1214 | 2 | 3 | 2 | 30.11 | 31.00 | 30.38 | 53.89 | 70.39 | 58.19 | 67.15 | 53.11 | 43.74 | 38.35 | 49.31 | 63.26 | 90.93 | 89.10 | 16.34 |
| Roach | 1215 | 2 | 3 | 2 | 24.85 | 22.57 | 22.52 | 47.88 | 57.57 | 46.43 | 49.57 | 36.00 | 31.11 | 34.61 | 41.26 | 46.28 | 73.29 | 72.73 | 14.53 |
| Roach | 1216 | 2 | 3 | 2 | 29.85 | 31.80 | 36.05 | 53.14 | 71.29 | 56.27 | 67.82 | 52.61 | 40.46 | 44.06 | 54.34 | 59.11 | 83.63 | 85.04 | 18.72 |
| Roach | 1217 | 2 | 3 | 2 | 31.53 | 31.87 | 34.61 | 59.71 | 76.54 | 62.89 | 68.20 | 50.12 | 36.32 | 33.60 | 47.09 | 54.44 | 80.32 | 81.62 | 15.49 |
| Roach | 1218 | 2 | 3 | 2 | 29.07 | 25.64 | 28.96 | 44.72 | 62.98 | 54.61 | 62.29 | 47.14 | 29.85 | 39.25 | 47.60 | 54.45 | 72.81 | 75.55 | 17.10 |
| Roach | 1219 | 2 | 3 | 2 | 30.54 | 31.67 | 30.18 | 49.65 | 64.86 | 54.97 | 64.49 | 48.66 | 33.99 | 38.92 | 49.84 | 55.85 | 76.82 | 80.08 | 16.51 |
| Roach | 1220 | 2 | 3 | 2 | 21.36 | 21.51 | 21.69 | 37.13 | 47.54 | 40.51 | 45.88 | 33.01 | 26.33 | 28.58 | 32.68 | 38.28 | 53.85 | 49.73 | 11.15 |
| Roach | 1191 | 2 | 3 | 3 | 27.60 | 36.13 | 35.44 | 69.68 | 84.23 | 67.25 | 79.29 | 58.54 | 44.26 | 47.97 | 54.92 | 67.62 | 98.43 | 91.26 | 19.24 |
| Roach | 1192 | 2 | 3 | 3 | 22.62 | 28.03 | 29.70 | 61.50 | 81.49 | 68.62 | 71.94 | 59.47 | 43.42 | 48.14 | 57.93 | 66.08 | 91.16 | 85.34 | 20.71 |
| Roach | 1193 | 2 | 3 | 3 | 23.71 | 27.64 | 29.70 | 57.16 | 71.01 | 53.37 | 67.27 | 55.51 | 37.14 | 39.76 | 48.79 | 61.45 | 90.25 | 84.29 | 19.72 |
| Roach | 1194 | 2 | 3 | 3 | 26.05 | 27.51 | 27.20 | 50.16 | 60.85 | 49.25 | 59.35 | 45.31 | 33.19 | 36.43 | 42.31 | 49.34 | 69.42 | 67.14 | 15.31 |
| Roach | 1195 | 2 | 3 | 3 | 28.03 | 27.80 | 32.70 | 57.65 | 72.54 | 59.46 | 68.90 | 50.71 | 41.35 | 43.40 | 50.80 | 60.27 | 76.27 | 86.39 | 17.86 |
| Roach | 1196 | 2 | 3 | 3 | 32.31 | 35.29 | 36.93 | 54.99 | 73.42 | 57.66 | 70.24 | 54.95 | 42.96 | 36.65 | 50.02 | 63.44 | 82.85 | 84.30 | 18.78 |
| Roach | 1197 | 2 | 3 | 3 | 31.92 | 38.54 | 40.78 | 70.80 | 92.61 | 76.99 | 92.45 | 71.10 | 45.75 | 39.99 | 55.43 | 79.09 | 103.27 | 94.42 | 24.85 |
| Roach | 1198 | 2 | 3 | 3 | 24.93 | 29.29 | 32.21 | 49.31 | 66.79 | 52.95 | 61.87 | 44.81 | 35.44 | 39.59 | 45.36 | 53.12 | 69.69 | 67.62 | 14.70 |
| Roach | 1199 | 2 | 3 | 3 | 26.38 | 26.72 | 30.36 | 51.15 | 69.13 | 55.03 | 61.10 | 49.64 | 37.64 | 45.63 | 52.66 | 55.86 | 86.71 | 83.80 | 16.50 |
| Roach | 1200 | 2 | 3 | 3 | 25.25 | 28.58 | 36.35 | 57.32 | 78.73 | 66.03 | 69.74 | 53.44 | 46.05 | 45.24 | 52.90 | 62.32 | 86.41 | 76.91 | 16.66 |
| Roach | 1201 | 2 | 3 | 3 | 36.78 | 33.82 | 38.63 | 62.38 | 81.97 | 61.63 | 75.56 | 59.79 | 45.63 | 50.98 | 57.56 | 65.05 | 86.32 | 81.69 | 18.46 |
| Roach | 1202 | 2 | 3 | 3 | 23.46 | 29.75 | 31.94 | 50.61 | 71.38 | 58.95 | 62.74 | 50.88 | 33.27 | 36.60 | 46.04 | 53.72 | 74.82 | 67.27 | 18.02 |
| Roach | 1203 | 2 | 3 | 3 | 27.25 | 25.57 | 28.63 | 52.51 | 67.89 | 53.71 | 61.29 | 48.35 | 33.68 | 38.84 | 46.87 | 52.98 | 75.24 | 74.34 | 16.60 |
| Roach | 1204 | 2 | 3 | 3 | 26.97 | 29.29 | 31.84 | 54.39 | 70.54 | 55.72 | 63.55 | 48.81 | 37.57 | 39.33 | 47.84 | 56.91 | 78.28 | 78.79 | 18.04 |
| Roach | 1205 | 2 | 3 | 3 | 25.81 | 27.73 | 31.83 | 55.90 | 69.03 | 56.33 | 65.91 | 51.66 | 38.21 | 37.30 | 45.61 | 54.13 | 78.87 | 75.62 | 16.55 |
| Roach | 1206 | 2 | 3 | 3 | 31.21 | 31.46 | 35.47 | 59.54 | 78.40 | 63.60 | 71.36 | 56.50 | 40.82 | 47.65 | 56.94 | 62.41 | 94.27 | 87.00 | 18.30 |
| Roach | 1207 | 2 | 3 | 3 | 24.45 | 26.80 | 29.40 | 48.63 | 63.56 | 50.94 | 61.24 | 49.53 | 37.32 | 43.21 | 50.63 | 55.70 | 81.85 | 76.48 | 19.24 |
| Roach | 1208 | 2 | 3 | 3 | 26.66 | 29.71 | 30.07 | 49.52 | 69.59 | 61.48 | 65.09 | 49.80 | 34.78 | 41.31 | 52.73 | 57.97 | 85.07 | 82.45 | 16.72 |
| Roach | 1209 | 2 | 3 | 3 | 22.69 | 24.97 | 22.07 | 39.81 | 51.78 | 45.14 | 53.51 | 39.64 | 28.19 | 40.81 | 44.19 | 43.16 | 65.67 | 64.24 | 13.80 |
| Roach | 1210 | 2 | 3 | 3 | 26.46 | 25.27 | 26.84 | 46.45 | 60.63 | 53.79 | 59.95 | 42.48 | 30.38 | 39.08 | 43.63 | 46.57 | 72.47 | 68.05 | 14.42 |
| Roach | 1211 | 2 | 3 | 3 | 26.47 | 25.02 | 24.05 | 40.91 | 50.95 | 43.71 | 50.79 | 35.53 | 31.23 | 39.12 | 44.72 | 37.92 | 58.77 | 56.29 | 11.29 |
| Roach | 1212 | 2 | 3 | 3 | 28.56 | 35.56 | 34.36 | 58.58 | 77.79 | 63.38 | 72.07 | 54.72 | 37.72 | 48.32 | 58.01 | 61.93 | 90.85 | 85.30 | 20.71 |
| Roach | 1213 | 2 | 3 | 3 | 25.83 | 27.96 | 30.31 | 61.15 | 70.68 | 56.34 | 71.73 | 52.95 | 37.92 | 39.19 | 51.25 | 63.11 | 84.70 | 82.39 | 16.83 |
| Roach | 1214 | 2 | 3 | 3 | 32.37 | 28.58 | 30.12 | 57.35 | 75.03 | 63.26 | 69.07 | 54.37 | 42.37 | 42.21 | 51.88 | 60.68 | 86.01 | 84.63 | 16.18 |
| Roach | 1215 | 2 | 3 | 3 | 27.59 | 25.76 | 26.12 | 44.22 | 54.92 | 47.05 | 54.65 | 40.14 | 28.71 | 37.57 | 41.27 | 45.12 | 71.89 | 67.35 | 13.08 |
| Roach | 1216 | 2 | 3 | 3 | 30.00 | 31.81 | 31.62 | 59.20 | 72.98 | 56.86 | 66.04 | 52.75 | 45.92 | 42.71 | 53.27 | 60.16 | 85.18 | 85.56 | 15.95 |
| Roach | 1217 | 2 | 3 | 3 | 34.08 | 32.05 | 35.56 | 54.63 | 75.55 | 63.51 | 70.71 | 51.31 | 36.13 | 33.71 | 44.53 | 56.40 | 82.48 | 83.68 | 17.81 |
| Roach | 1218 | 2 | 3 | 3 | 29.71 | 27.95 | 31.22 | 47.32 | 64.39 | 52.20 | 59.18 | 46.84 | 37.38 | 36.09 | 40.46 | 54.86 | 71.37 | 70.12 | 16.53 |
| Roach | 1219 | 2 | 3 | 3 | 30.42 | 28.45 | 31.80 | 51.96 | 72.39 | 59.98 | 64.00 | 48.60 | 34.61 | 35.90 | 47.10 | 56.26 | 74.81 | 76.66 | 19.00 |
| Roach | 1220 | 2 | 3 | 3 | 20.00 | 21.92 | 20.41 | 36.05 | 46.29 | 33.66 | 44.53 | 32.62 | 24.67 | 27.32 | 31.96 | 37.85 | 53.94 | 50.16 | 12.61 |
| Roach | 1001 | 3 | 1 | 1 | 15.65 | 16.11 | 16.83 | 30.66 | 40.80 | 32.16 | 35.44 | 29.68 | 23.56 | 28.87 | 30.16 | 34.39 | 50.38 | 44.47 | 10.03 |
| Roach | 1002 | 3 | 1 | 1 | 24.49 | 21.34 | 22.80 | 42.29 | 52.69 | 42.29 | 49.77 | 37.25 | 28.56 | 32.35 | 36.83 | 43.33 | 60.47 | 55.68 | 12.88 |
| Roach | 1003 | 3 | 1 | 1 | 22.73 | 24.43 | 25.24 | 46.02 | 61.92 | 48.40 | 56.53 | 45.24 | 29.50 | 34.86 | 42.58 | 51.25 | 73.40 | 71.36 | 13.99 |
| Roach | 1004 | 3 | 1 | 1 | 16.04 | 16.94 | 16.65 | 29.95 | 37.26 | 30.31 | 35.30 | 26.57 | 30.60 | 23.14 | 27.21 | 30.58 | 46.08 | 42.16 | 9.60 |
| Roach | 1005 | 3 | 1 | 1 | 18.81 | 20.53 | 21.81 | 42.92 | 54.54 | 45.32 | 51.49 | 38.21 | 27.27 | 28.47 | 34.58 | 43.21 | 58.95 | 55.07 | 13.23 |
| Roach | 1006 | 3 | 1 | 1 | 24.74 | 24.87 | 25.83 | 45.87 | 61.29 | 49.40 | 56.37 | 45.28 | 32.40 | 37.22 | 43.82 | 50.55 | 69.01 | 63.40 | 15.00 |
| Roach | 1007 | 3 | 1 | 1 | 16.51 | 16.65 | 17.69 | 30.15 | 43.83 | 36.40 | 37.12 | 30.05 | 19.32 | 22.76 | 28.58 | 33.45 | 47.85 | 45.49 | 10.00 |
| Roach | 1008 | 3 | 1 | 1 | 15.17 | 14.61 | 15.50 | 26.46 | 34.92 | 28.12 | 32.53 | 25.50 | 18.03 | 21.27 | 25.68 | 29.16 | 41.21 | 38.51 | 9.63 |
| Roach | 1009 | 3 | 1 | 1 | 16.60 | 16.51 | 17.27 | 32.97 | 39.99 | 33.16 | 39.17 | 27.91 | 19.95 | 23.48 | 28.05 | 32.59 | 47.34 | 42.60 | 10.22 |
| Roach | 1010 | 3 | 1 | 1 | 18.68 | 18.14 | 19.37 | 33.99 | 41.32 | 33.04 | 40.69 | 30.23 | 19.86 | 25.00 | 28.96 | 33.77 | 42.47 | 47.11 | 10.93 |
| Roach | 1011 | 3 | 1 | 1 | 16.10 | 14.64 | 16.08 | 29.90 | 34.90 | 29.00 | 34.95 | 26.12 | 17.57 | 23.06 | 26.88 | 29.71 | 43.54 | 40.28 | 9.23 |
| Roach | 1012 | 3 | 1 | 1 | 18.14 | 18.98 | 19.03 | 36.92 | 45.33 | 37.36 | 43.55 | 31.80 | 23.25 | 28.18 | 31.68 | 37.01 | 50.86 | 46.74 | 10.29 |
| Roach | 1013 | 3 | 1 | 1 | 19.12 | 16.73 | 18.35 | 30.48 | 39.81 | 33.49 | 39.30 | 28.83 | 18.13 | 26.76 | 30.93 | 31.51 | 47.13 | 44.34 | 10.36 |
| Roach | 1014 | 3 | 1 | 1 | 25.37 | 23.36 | 26.23 | 43.64 | 58.60 | 49.76 | 55.33 | 41.36 | 30.28 | 39.53 | 43.94 | 46.93 | 66.78 | 61.00 | 13.92 |
| Roach | 1015 | 3 | 1 | 1 | 23.17 | 25.10 | 27.29 | 42.83 | 60.75 | 49.18 | 53.11 | 42.07 | 30.75 | 35.86 | 42.83 | 48.08 | 69.78 | 65.39 | 14.22 |
| Roach | 1016 | 3 | 1 | 1 | 16.04 | 16.12 | 17.50 | 31.06 | 41.39 | 33.87 | 38.15 | 31.03 | 17.96 | 23.87 | 28.99 | 32.91 | 45.90 | 42.35 | 10.62 |
| Roach | 1017 | 3 | 1 | 1 | 12.88 | 13.57 | 13.96 | 24.88 | 31.56 | 26.71 | 30.59 | 22.50 | 15.49 | 20.20 | 22.77 | 25.26 | 38.35 | 35.55 | 7.35 |
| Roach | 1018 | 3 | 1 | 1 | 19.05 | 20.49 | 21.88 | 36.68 | 48.94 | 39.41 | 45.12 | 36.61 | 26.75 | 28.08 | 35.41 | 41.26 | 58.00 | 57.09 | 11.86 |
| Roach | 1019 | 3 | 1 | 1 | 16.25 | 16.85 | 18.08 | 32.40 | 42.75 | 35.93 | 40.12 | 30.08 | 19.14 | 25.80 | 30.39 | 32.57 | 47.09 | 43.30 | 10.65 |
| Roach | 1020 | 3 | 1 | 1 | 19.70 | 21.82 | 22.46 | 43.39 | 55.31 | 44.92 | 52.37 | 39.05 | 27.53 | 30.27 | 36.28 | 44.23 | 60.94 | 57.12 | 13.27 |
| Roach | 1021 | 3 | 1 | 1 | 19.59 | 19.87 | 21.01 | 38.78 | 49.65 | 40.81 | 45.38 | 34.99 | 25.72 | 29.51 | 36.17 | 39.23 | 53.52 | 56.45 | 12.70 |
| Roach | 1022 | 3 | 1 | 1 | 18.77 | 17.88 | 19.08 | 31.65 | 41.12 | 34.39 | 39.11 | 28.67 | 21.47 | 23.62 | 28.46 | 33.04 | 48.32 | 44.50 | 10.20 |
| Roach | 1023 | 3 | 1 | 1 | 15.44 | 15.97 | 16.37 | 26.35 | 36.36 | 30.40 | 32.64 | 24.06 | 19.48 | 22.98 | 26.46 | 26.35 | 40.67 | 37.96 | 8.40 |
| Roach | 1024 | 3 | 1 | 1 | 19.95 | 19.28 | 20.27 | 31.69 | 42.15 | 32.01 | 38.95 | 31.84 | 21.79 | 26.69 | 31.69 | 36.06 | 53.29 | 49.70 | 12.20 |
| Roach | 1025 | 3 | 1 | 1 | 17.19 | 16.32 | 18.32 | 33.59 | 43.92 | 35.49 | 40.81 | 32.26 | 21.33 | 26.44 | 31.85 | 35.46 | 49.90 | 46.29 | 11.26 |
| Roach | 1026 | 3 | 1 | 1 | 23.12 | 22.21 | 24.05 | 41.62 | 55.95 | 48.72 | 53.17 | 39.39 | 24.45 | 28.49 | 37.22 | 42.29 | 63.36 | 62.64 | 13.62 |
| Roach | 1027 | 3 | 1 | 1 | 19.36 | 17.60 | 19.87 | 36.21 | 45.84 | 38.57 | 44.69 | 33.11 | 26.44 | 28.99 | 32.58 | 37.42 | 52.33 | 46.24 | 10.87 |
| Roach | 1028 | 3 | 1 | 1 | 19.59 | 21.75 | 21.92 | 41.27 | 53.10 | 42.88 | 47.73 | 36.34 | 28.27 | 32.38 | 38.55 | 42.65 | 63.00 | 59.83 | 13.80 |
| Roach | 1029 | 3 | 1 | 1 | 18.79 | 19.07 | 21.33 | 37.02 | 47.33 | 39.42 | 45.78 | 33.42 | 24.21 | 24.89 | 32.18 | 39.03 | 54.43 | 54.66 | 11.65 |
| Roach | 1030 | 3 | 1 | 1 | 16.96 | 18.82 | 19.50 | 33.18 | 45.61 | 37.05 | 40.45 | 32.42 | 22.41 | 25.28 | 32.08 | 34.39 | 48.48 | 43.95 | 10.80 |
| Roach | 1001 | 3 | 1 | 2 | 17.42 | 16.13 | 17.61 | 28.79 | 40.18 | 31.90 | 35.66 | 28.99 | 23.24 | 27.66 | 30.57 | 33.94 | 50.17 | 44.66 | 10.41 |
| Roach | 1002 | 3 | 1 | 2 | 22.09 | 23.00 | 24.45 | 39.07 | 52.66 | 41.88 | 48.94 | 38.25 | 29.74 | 32.73 | 37.70 | 43.41 | 61.01 | 54.29 | 13.81 |
| Roach | 1003 | 3 | 1 | 2 | 23.44 | 25.56 | 26.88 | 44.53 | 61.14 | 48.46 | 54.98 | 44.81 | 31.26 | 33.67 | 42.15 | 51.13 | 72.52 | 71.67 | 13.92 |
| Roach | 1004 | 3 | 1 | 2 | 15.85 | 16.31 | 16.97 | 29.78 | 37.64 | 29.58 | 35.83 | 26.53 | 17.19 | 22.93 | 27.21 | 30.34 | 45.04 | 42.31 | 10.24 |
| Roach | 1005 | 3 | 1 | 2 | 19.52 | 20.71 | 21.77 | 43.33 | 55.46 | 45.34 | 50.17 | 37.05 | 26.51 | 28.56 | 35.75 | 43.84 | 59.11 | 56.01 | 13.67 |
| Roach | 1006 | 3 | 1 | 2 | 25.84 | 25.24 | 27.30 | 44.71 | 60.89 | 49.20 | 55.84 | 44.93 | 33.29 | 35.68 | 44.55 | 51.94 | 69.63 | 63.76 | 15.79 |
| Roach | 1007 | 3 | 1 | 2 | 17.05 | 18.81 | 19.15 | 29.20 | 41.60 | 34.94 | 37.15 | 30.01 | 20.02 | 22.70 | 29.17 | 34.16 | 49.21 | 47.85 | 10.61 |
| Roach | 1008 | 3 | 1 | 2 | 16.43 | 15.31 | 16.74 | 24.44 | 33.97 | 27.09 | 31.59 | 25.93 | 19.67 | 20.57 | 25.53 | 29.99 | 42.49 | 39.56 | 9.60 |
| Roach | 1009 | 3 | 1 | 2 | 17.61 | 17.43 | 18.40 | 31.01 | 40.00 | 32.62 | 38.23 | 28.50 | 21.08 | 24.04 | 28.72 | 33.35 | 47.59 | 44.12 | 10.80 |
| Roach | 1010 | 3 | 1 | 2 | 19.39 | 18.15 | 20.10 | 31.48 | 41.08 | 32.89 | 39.89 | 31.11 | 21.87 | 24.69 | 28.71 | 34.77 | 47.73 | 43.41 | 11.15 |
| Roach | 1011 | 3 | 1 | 2 | 16.79 | 14.75 | 17.62 | 28.19 | 35.32 | 29.01 | 35.81 | 25.93 | 15.46 | 23.95 | 28.37 | 29.74 | 44.44 | 41.60 | 9.91 |
| Roach | 1012 | 3 | 1 | 2 | 19.14 | 20.11 | 20.73 | 33.83 | 45.61 | 37.03 | 41.30 | 31.87 | 25.35 | 28.14 | 32.32 | 36.46 | 51.01 | 46.12 | 11.09 |
| Roach | 1013 | 3 | 1 | 2 | 17.83 | 17.66 | 18.52 | 31.04 | 40.95 | 33.29 | 36.81 | 28.60 | 21.33 | 26.51 | 31.03 | 32.24 | 48.34 | 44.67 | 10.71 |
| Roach | 1014 | 3 | 1 | 2 | 26.47 | 23.84 | 26.56 | 41.93 | 58.60 | 48.52 | 53.96 | 41.14 | 31.85 | 39.22 | 45.15 | 47.22 | 65.94 | 60.62 | 15.00 |
| Roach | 1015 | 3 | 1 | 2 | 27.19 | 25.19 | 28.66 | 38.86 | 58.24 | 48.32 | 52.18 | 41.76 | 31.80 | 35.63 | 42.48 | 48.36 | 69.97 | 65.49 | 15.00 |
| Roach | 1016 | 3 | 1 | 2 | 17.40 | 16.60 | 18.51 | 28.75 | 41.15 | 34.46 | 37.78 | 31.26 | 19.01 | 24.16 | 28.80 | 32.36 | 45.66 | 42.22 | 10.98 |
| Roach | 1017 | 3 | 1 | 2 | 14.10 | 14.95 | 14.90 | 23.48 | 31.71 | 25.59 | 28.53 | 22.20 | 16.45 | 20.79 | 23.52 | 25.00 | 38.23 | 36.04 | 8.00 |
| Roach | 1018 | 3 | 1 | 2 | 20.93 | 20.59 | 22.50 | 35.28 | 49.54 | 40.04 | 44.78 | 36.17 | 27.28 | 28.42 | 34.92 | 41.97 | 60.40 | 57.03 | 12.51 |
| Roach | 1019 | 3 | 1 | 2 | 19.20 | 17.11 | 19.20 | 30.71 | 42.65 | 36.00 | 39.02 | 30.53 | 21.52 | 23.70 | 30.02 | 33.01 | 46.02 | 44.78 | 10.84 |
| Roach | 1020 | 3 | 1 | 2 | 22.80 | 22.31 | 24.46 | 40.64 | 55.42 | 45.37 | 50.12 | 39.23 | 28.63 | 30.79 | 37.45 | 43.90 | 61.58 | 57.99 | 13.90 |
| Roach | 1021 | 3 | 1 | 2 | 20.07 | 20.68 | 22.07 | 36.73 | 49.84 | 42.19 | 45.69 | 34.91 | 25.75 | 28.91 | 36.91 | 40.16 | 56.56 | 54.48 | 13.07 |
| Roach | 1022 | 3 | 1 | 2 | 19.60 | 18.83 | 19.81 | 30.24 | 41.45 | 33.80 | 38.81 | 28.93 | 22.53 | 23.58 | 28.43 | 33.19 | 48.11 | 45.43 | 10.44 |
| Roach | 1023 | 3 | 1 | 2 | 16.50 | 16.01 | 16.51 | 25.98 | 35.74 | 29.70 | 32.07 | 23.97 | 21.36 | 22.62 | 26.23 | 27.74 | 40.97 | 38.12 | 8.51 |
| Roach | 1024 | 3 | 1 | 2 | 19.66 | 20.08 | 21.31 | 29.47 | 42.14 | 33.02 | 39.01 | 32.26 | 22.36 | 26.60 | 32.50 | 35.99 | 53.39 | 50.79 | 12.36 |
| Roach | 1025 | 3 | 1 | 2 | 18.58 | 18.22 | 19.95 | 31.83 | 43.23 | 35.46 | 40.62 | 32.21 | 21.99 | 26.36 | 32.19 | 35.68 | 50.06 | 46.89 | 11.71 |
| Roach | 1026 | 3 | 1 | 2 | 25.96 | 23.13 | 25.34 | 38.88 | 55.42 | 49.23 | 52.21 | 39.23 | 26.41 | 28.12 | 35.93 | 43.01 | 63.19 | 63.17 | 13.56 |
| Roach | 1027 | 3 | 1 | 2 | 21.14 | 18.89 | 21.38 | 33.53 | 45.37 | 37.90 | 43.16 | 32.73 | 27.52 | 30.36 | 33.33 | 37.46 | 52.84 | 45.97 | 11.32 |
| Roach | 1028 | 3 | 1 | 2 | 21.54 | 22.14 | 22.74 | 39.76 | 52.32 | 42.46 | 47.95 | 36.80 | 28.51 | 33.31 | 38.01 | 43.07 | 64.04 | 59.41 | 13.90 |
| Roach | 1029 | 3 | 1 | 2 | 19.66 | 18.69 | 21.56 | 37.22 | 50.04 | 41.58 | 46.07 | 34.36 | 25.23 | 26.10 | 32.25 | 38.90 | 56.36 | 55.64 | 11.67 |
| Roach | 1030 | 3 | 1 | 2 | 18.04 | 19.31 | 20.53 | 31.63 | 44.59 | 36.66 | 41.23 | 32.65 | 21.77 | 25.88 | 31.48 | 34.62 | 48.03 | 44.05 | 11.24 |
| Roach | 1001 | 3 | 1 | 3 | 16.52 | 15.78 | 17.25 | 29.46 | 40.95 | 31.91 | 35.67 | 29.28 | 23.41 | 26.55 | 30.00 | 33.83 | 49.73 | 44.62 | 10.66 |
| Roach | 1002 | 3 | 1 | 3 | 21.89 | 22.61 | 24.46 | 40.35 | 53.50 | 42.76 | 49.55 | 37.74 | 29.46 | 32.65 | 36.94 | 43.32 | 59.93 | 53.79 | 13.98 |
| Roach | 1003 | 3 | 1 | 3 | 23.44 | 24.62 | 26.08 | 44.69 | 62.03 | 49.66 | 54.98 | 45.22 | 31.86 | 33.48 | 41.60 | 51.17 | 72.80 | 72.11 | 14.49 |
| Roach | 1004 | 3 | 1 | 3 | 15.67 | 16.92 | 17.61 | 28.56 | 37.32 | 29.89 | 35.65 | 26.94 | 17.40 | 22.17 | 27.10 | 30.49 | 44.48 | 42.21 | 10.04 |
| Roach | 1005 | 3 | 1 | 3 | 19.74 | 21.09 | 22.41 | 42.92 | 54.71 | 45.03 | 50.62 | 37.04 | 26.43 | 27.93 | 35.01 | 43.98 | 59.18 | 55.42 | 13.80 |
| Roach | 1006 | 3 | 1 | 3 | 25.76 | 24.47 | 27.49 | 44.62 | 61.24 | 50.15 | 56.23 | 45.13 | 33.29 | 35.46 | 43.37 | 51.71 | 69.82 | 64.61 | 15.62 |
| Roach | 1007 | 3 | 1 | 3 | 17.40 | 18.10 | 18.61 | 29.51 | 42.57 | 35.05 | 37.33 | 29.84 | 19.71 | 22.32 | 29.08 | 34.82 | 50.36 | 48.84 | 11.46 |
| Roach | 1008 | 3 | 1 | 3 | 15.90 | 14.51 | 16.32 | 25.24 | 33.47 | 27.68 | 32.01 | 25.51 | 19.36 | 20.83 | 25.38 | 30.19 | 42.39 | 39.68 | 9.70 |
| Roach | 1009 | 3 | 1 | 3 | 17.09 | 16.95 | 18.11 | 31.73 | 40.67 | 32.93 | 37.70 | 28.42 | 21.58 | 24.29 | 28.77 | 33.00 | 46.96 | 43.80 | 10.70 |
| Roach | 1010 | 3 | 1 | 3 | 18.31 | 17.72 | 19.50 | 31.55 | 40.90 | 31.78 | 38.98 | 30.80 | 21.81 | 24.62 | 29.21 | 34.80 | 48.14 | 43.07 | 11.06 |
| Roach | 1011 | 3 | 1 | 3 | 15.91 | 14.24 | 16.65 | 28.64 | 35.75 | 28.65 | 32.41 | 26.45 | 19.21 | 24.66 | 28.25 | 29.11 | 44.35 | 41.06 | 9.61 |
| Roach | 1012 | 3 | 1 | 3 | 19.06 | 19.61 | 20.42 | 33.96 | 46.03 | 36.95 | 40.99 | 32.18 | 24.89 | 28.46 | 32.78 | 36.29 | 51.76 | 46.65 | 11.47 |
| Roach | 1013 | 3 | 1 | 3 | 17.99 | 16.94 | 18.35 | 31.83 | 41.45 | 33.67 | 37.97 | 28.80 | 20.10 | 25.66 | 30.46 | 32.30 | 48.86 | 46.07 | 10.50 |
| Roach | 1014 | 3 | 1 | 3 | 25.43 | 24.25 | 26.56 | 44.00 | 57.50 | 48.44 | 55.21 | 40.69 | 30.82 | 38.65 | 44.20 | 47.52 | 67.42 | 60.32 | 14.99 |
| Roach | 1015 | 3 | 1 | 3 | 26.52 | 26.03 | 28.90 | 39.60 | 58.24 | 48.88 | 52.44 | 41.91 | 31.82 | 35.74 | 42.36 | 48.22 | 70.26 | 66.05 | 14.85 |
| Roach | 1016 | 3 | 1 | 3 | 16.88 | 16.56 | 18.22 | 29.59 | 41.45 | 34.11 | 37.61 | 30.66 | 18.24 | 24.13 | 28.91 | 32.63 | 45.91 | 41.98 | 10.92 |
| Roach | 1017 | 3 | 1 | 3 | 14.28 | 14.98 | 15.40 | 22.89 | 31.10 | 24.14 | 28.68 | 22.60 | 16.56 | 20.23 | 23.50 | 25.36 | 38.05 | 35.61 | 7.90 |
| Roach | 1018 | 3 | 1 | 3 | 20.40 | 20.86 | 22.48 | 35.58 | 49.37 | 39.50 | 44.95 | 36.01 | 26.70 | 28.75 | 35.18 | 41.94 | 58.90 | 57.12 | 12.76 |
| Roach | 1019 | 3 | 1 | 3 | 18.67 | 17.02 | 18.42 | 31.35 | 43.71 | 37.01 | 38.45 | 30.15 | 22.70 | 23.77 | 29.57 | 33.39 | 47.13 | 45.17 | 10.74 |
| Roach | 1020 | 3 | 1 | 3 | 22.05 | 21.65 | 23.61 | 40.39 | 55.32 | 45.60 | 50.93 | 39.61 | 29.01 | 28.89 | 36.40 | 44.92 | 61.56 | 57.96 | 13.93 |
| Roach | 1021 | 3 | 1 | 3 | 20.27 | 19.14 | 21.05 | 37.74 | 50.29 | 41.98 | 45.74 | 34.76 | 26.08 | 28.93 | 35.78 | 39.62 | 56.22 | 53.57 | 12.97 |
| Roach | 1022 | 3 | 1 | 3 | 20.14 | 18.52 | 19.73 | 31.02 | 41.39 | 34.44 | 38.84 | 28.85 | 22.42 | 23.77 | 28.19 | 33.21 | 48.19 | 45.38 | 10.70 |
| Roach | 1023 | 3 | 1 | 3 | 15.85 | 15.17 | 16.35 | 25.59 | 36.03 | 29.68 | 32.38 | 23.84 | 20.51 | 22.92 | 25.65 | 27.72 | 42.37 | 39.05 | 8.28 |
| Roach | 1024 | 3 | 1 | 3 | 19.55 | 20.21 | 21.22 | 29.39 | 41.61 | 32.39 | 38.97 | 32.41 | 22.65 | 26.42 | 32.73 | 36.23 | 52.69 | 50.19 | 12.52 |
| Roach | 1025 | 3 | 1 | 3 | 17.47 | 16.87 | 19.17 | 32.38 | 43.52 | 35.12 | 40.70 | 32.11 | 21.42 | 26.80 | 32.66 | 35.32 | 50.57 | 47.28 | 11.55 |
| Roach | 1026 | 3 | 1 | 3 | 24.53 | 23.87 | 25.29 | 40.69 | 55.73 | 48.62 | 52.88 | 39.54 | 26.49 | 27.38 | 35.66 | 43.98 | 64.76 | 64.37 | 13.51 |
| Roach | 1027 | 3 | 1 | 3 | 21.18 | 19.02 | 20.92 | 34.03 | 44.83 | 37.20 | 43.74 | 33.31 | 26.96 | 29.82 | 32.33 | 37.46 | 52.80 | 45.66 | 11.21 |
| Roach | 1028 | 3 | 1 | 3 | 19.66 | 22.66 | 22.96 | 40.66 | 53.21 | 42.60 | 47.87 | 36.21 | 27.80 | 33.36 | 38.68 | 42.86 | 63.67 | 59.11 | 13.81 |
| Roach | 1029 | 3 | 1 | 3 | 19.33 | 19.76 | 21.66 | 36.92 | 49.68 | 42.06 | 45.49 | 34.10 | 25.54 | 25.48 | 32.51 | 39.44 | 56.36 | 54.44 | 11.96 |
| Roach | 1030 | 3 | 1 | 3 | 17.86 | 18.79 | 20.11 | 32.61 | 46.31 | 38.34 | 40.44 | 31.84 | 22.11 | 26.33 | 31.90 | 34.21 | 47.80 | 44.01 | 11.61 |
| Roach | 1101 | 3 | 2 | 1 | 33.46 | 32.65 | 36.72 | 69.48 | 90.47 | 74.64 | 81.05 | 63.81 | 47.07 | 46.30 | 59.88 | 74.22 | 104.03 | 100.08 | 21.74 |
| Roach | 1102 | 3 | 2 | 1 | 30.29 | 33.01 | 32.69 | 58.88 | 72.56 | 58.93 | 69.45 | 50.87 | 40.68 | 44.98 | 52.01 | 59.16 | 87.81 | 82.37 | 18.20 |
| Roach | 1103 | 3 | 2 | 1 | 33.44 | 30.50 | 33.24 | 61.85 | 79.50 | 64.25 | 73.33 | 54.75 | 45.63 | 38.65 | 49.54 | 63.81 | 87.06 | 82.58 | 20.00 |
| Roach | 1104 | 3 | 2 | 1 | 35.49 | 33.24 | 36.78 | 59.86 | 82.48 | 65.61 | 73.36 | 57.98 | 46.00 | 42.86 | 54.38 | 65.79 | 92.26 | 89.11 | 18.59 |
| Roach | 1105 | 3 | 2 | 1 | 31.09 | 32.92 | 33.42 | 62.75 | 78.85 | 60.69 | 70.68 | 53.01 | 37.09 | 44.25 | 52.01 | 61.42 | 87.70 | 81.81 | 18.81 |
| Roach | 1106 | 3 | 2 | 1 | 23.38 | 22.62 | 24.35 | 42.72 | 57.36 | 46.48 | 51.37 | 40.26 | 30.45 | 37.00 | 40.81 | 46.33 | 70.44 | 62.45 | 14.24 |
| Roach | 1107 | 3 | 2 | 1 | 30.41 | 28.55 | 30.19 | 58.05 | 71.86 | 58.22 | 66.17 | 48.91 | 40.84 | 43.57 | 51.03 | 56.42 | 79.30 | 74.02 | 17.15 |
| Roach | 1108 | 3 | 2 | 1 | 29.13 | 27.00 | 28.51 | 56.75 | 70.54 | 57.68 | 65.86 | 48.76 | 37.84 | 37.24 | 47.08 | 55.62 | 77.12 | 75.44 | 15.99 |
| Roach | 1109 | 3 | 2 | 1 | 31.28 | 28.74 | 32.46 | 54.50 | 72.16 | 61.50 | 71.68 | 54.41 | 36.06 | 39.14 | 50.44 | 60.37 | 81.79 | 79.26 | 18.20 |
| Roach | 1110 | 3 | 2 | 1 | 25.55 | 20.88 | 23.54 | 46.64 | 57.58 | 49.22 | 56.46 | 40.97 | 33.10 | 33.49 | 41.53 | 46.73 | 68.69 | 65.79 | 14.87 |
| Roach | 1111 | 3 | 2 | 1 | 26.63 | 29.56 | 28.87 | 47.53 | 62.38 | 49.63 | 58.36 | 45.38 | 35.35 | 39.01 | 46.90 | 52.19 | 76.28 | 71.87 | 16.51 |
| Roach | 1112 | 3 | 2 | 1 | 25.29 | 24.52 | 24.77 | 44.03 | 55.34 | 44.18 | 52.08 | 37.65 | 30.43 | 31.59 | 40.08 | 44.38 | 63.52 | 62.56 | 13.86 |
| Roach | 1113 | 3 | 2 | 1 | 32.35 | 31.42 | 32.27 | 51.37 | 69.68 | 55.37 | 64.60 | 51.27 | 36.51 | 40.43 | 48.85 | 54.94 | 79.30 | 75.08 | 18.49 |
| Roach | 1114 | 3 | 2 | 1 | 22.63 | 24.15 | 23.30 | 40.99 | 55.21 | 44.00 | 48.07 | 38.96 | 28.78 | 30.12 | 37.31 | 43.69 | 63.65 | 61.06 | 13.58 |
| Roach | 1115 | 3 | 2 | 1 | 26.51 | 26.68 | 27.48 | 46.28 | 63.35 | 52.37 | 57.71 | 46.20 | 31.35 | 37.92 | 45.45 | 51.83 | 75.92 | 71.23 | 16.67 |
| Roach | 1116 | 3 | 2 | 1 | 20.31 | 21.34 | 21.48 | 40.56 | 50.73 | 40.66 | 46.26 | 34.37 | 26.49 | 30.58 | 37.21 | 38.75 | 56.33 | 52.98 | 12.22 |
| Roach | 1117 | 3 | 2 | 1 | 24.01 | 22.61 | 25.09 | 48.10 | 60.01 | 48.05 | 56.43 | 42.78 | 32.69 | 29.63 | 38.13 | 47.42 | 65.34 | 62.12 | 15.15 |
| Roach | 1118 | 3 | 2 | 1 | 23.18 | 25.86 | 25.97 | 42.26 | 58.27 | 48.46 | 51.76 | 39.55 | 33.08 | 31.70 | 38.33 | 46.33 | 67.01 | 63.52 | 15.18 |
| Roach | 1119 | 3 | 2 | 1 | 27.97 | 23.93 | 25.84 | 45.72 | 57.94 | 51.55 | 59.63 | 42.91 | 29.76 | 35.80 | 43.32 | 45.92 | 66.53 | 63.50 | 14.85 |
| Roach | 1120 | 3 | 2 | 1 | 21.33 | 21.54 | 21.12 | 35.10 | 45.00 | 37.23 | 43.28 | 30.91 | 28.92 | 29.60 | 34.97 | 39.39 | 58.36 | 56.94 | 12.33 |
| Roach | 1121 | 3 | 2 | 1 | 24.77 | 24.97 | 24.08 | 42.62 | 55.90 | 46.99 | 51.80 | 35.70 | 26.64 | 30.93 | 37.72 | 43.30 | 64.61 | 61.53 | 13.88 |
| Roach | 1122 | 3 | 2 | 1 | 21.32 | 22.92 | 22.28 | 39.92 | 50.87 | 38.80 | 46.37 | 35.45 | 31.47 | 29.97 | 35.79 | 43.15 | 58.99 | 55.16 | 12.35 |
| Roach | 1123 | 3 | 2 | 1 | 20.64 | 22.62 | 22.66 | 42.76 | 50.94 | 40.86 | 48.73 | 35.02 | 26.63 | 34.02 | 38.86 | 40.25 | 58.63 | 54.71 | 13.16 |
| Roach | 1124 | 3 | 2 | 1 | 21.81 | 23.22 | 23.41 | 42.88 | 56.92 | 48.18 | 50.84 | 37.65 | 29.09 | 29.15 | 35.93 | 41.86 | 61.41 | 58.96 | 13.21 |
| Roach | 1125 | 3 | 2 | 1 | 21.13 | 21.23 | 21.21 | 34.98 | 47.36 | 36.54 | 43.09 | 31.91 | 23.03 | 29.03 | 34.25 | 37.01 | 57.29 | 55.02 | 11.89 |
| Roach | 1126 | 3 | 2 | 1 | 26.76 | 25.21 | 24.62 | 41.48 | 51.84 | 42.86 | 51.10 | 34.82 | 27.18 | 33.42 | 39.78 | 42.69 | 63.55 | 60.35 | 13.58 |
| Roach | 1127 | 3 | 2 | 1 | 28.88 | 29.42 | 30.49 | 60.35 | 72.40 | 60.58 | 72.31 | 51.83 | 34.69 | 40.91 | 49.66 | 59.26 | 81.59 | 74.64 | 17.59 |
| Roach | 1128 | 3 | 2 | 1 | 25.40 | 25.32 | 25.52 | 44.24 | 55.74 | 44.72 | 54.75 | 41.26 | 26.46 | 32.09 | 38.78 | 46.70 | 69.38 | 67.04 | 14.68 |
| Roach | 1129 | 3 | 2 | 1 | 24.69 | 23.09 | 24.51 | 38.40 | 55.45 | 47.70 | 50.37 | 36.61 | 25.97 | 28.80 | 36.91 | 43.08 | 64.20 | 63.35 | 14.30 |
| Roach | 1130 | 3 | 2 | 1 | 20.29 | 21.53 | 21.41 | 40.81 | 48.77 | 41.37 | 48.97 | 32.53 | 18.16 | 23.85 | 30.09 | 37.17 | 55.75 | 55.19 | 11.15 |
| Roach | 1101 | 3 | 2 | 2 | 35.69 | 33.58 | 37.82 | 65.48 | 89.84 | 75.03 | 82.28 | 64.21 | 46.28 | 45.95 | 58.64 | 74.99 | 104.13 | 100.96 | 22.87 |
| Roach | 1102 | 3 | 2 | 2 | 32.99 | 32.20 | 32.45 | 56.87 | 72.28 | 57.90 | 69.89 | 51.97 | 40.37 | 45.71 | 52.49 | 58.28 | 87.45 | 80.83 | 17.90 |
| Roach | 1103 | 3 | 2 | 2 | 34.03 | 31.53 | 34.51 | 61.11 | 79.81 | 66.96 | 75.20 | 55.56 | 45.15 | 38.24 | 50.72 | 63.75 | 86.15 | 81.63 | 20.05 |
| Roach | 1104 | 3 | 2 | 2 | 35.09 | 33.94 | 38.43 | 59.62 | 82.75 | 64.92 | 73.86 | 58.57 | 45.06 | 45.12 | 56.53 | 65.98 | 93.13 | 90.18 | 18.44 |
| Roach | 1105 | 3 | 2 | 2 | 32.04 | 33.15 | 35.21 | 60.66 | 78.86 | 60.51 | 70.08 | 53.86 | 38.61 | 43.45 | 53.56 | 61.48 | 87.64 | 82.87 | 19.76 |
| Roach | 1106 | 3 | 2 | 2 | 25.89 | 22.29 | 24.90 | 42.30 | 57.22 | 46.31 | 51.88 | 40.99 | 30.20 | 68.44 | 68.92 | 40.81 | 66.67 | 59.93 | 14.52 |
| Roach | 1107 | 3 | 2 | 2 | 31.50 | 28.64 | 31.85 | 53.57 | 71.53 | 58.28 | 66.19 | 50.04 | 40.99 | 42.29 | 50.26 | 56.30 | 79.96 | 74.40 | 17.26 |
| Roach | 1108 | 3 | 2 | 2 | 30.77 | 28.31 | 30.45 | 52.95 | 70.38 | 60.26 | 65.81 | 49.26 | 38.29 | 37.89 | 47.46 | 55.06 | 79.06 | 75.59 | 16.66 |
| Roach | 1109 | 3 | 2 | 2 | 32.94 | 31.27 | 34.17 | 51.56 | 70.72 | 59.18 | 68.57 | 54.21 | 39.79 | 38.82 | 49.51 | 60.65 | 82.90 | 79.08 | 18.83 |
| Roach | 1110 | 3 | 2 | 2 | 26.94 | 25.90 | 26.10 | 44.26 | 57.16 | 48.13 | 55.44 | 40.99 | 33.49 | 35.67 | 42.01 | 46.99 | 68.53 | 63.65 | 14.60 |
| Roach | 1111 | 3 | 2 | 2 | 27.92 | 27.27 | 28.82 | 46.25 | 62.60 | 49.15 | 57.92 | 45.87 | 36.83 | 38.97 | 45.97 | 52.37 | 76.81 | 71.85 | 16.61 |
| Roach | 1112 | 3 | 2 | 2 | 26.48 | 24.80 | 26.02 | 42.94 | 57.53 | 46.83 | 52.73 | 38.71 | 29.69 | 33.47 | 40.32 | 44.48 | 65.37 | 63.01 | 14.86 |
| Roach | 1113 | 3 | 2 | 2 | 34.62 | 32.12 | 33.21 | 50.75 | 70.26 | 55.59 | 63.48 | 50.53 | 37.98 | 40.80 | 48.54 | 55.17 | 80.78 | 74.81 | 19.02 |
| Roach | 1114 | 3 | 2 | 2 | 24.19 | 23.69 | 23.88 | 39.27 | 54.79 | 43.31 | 47.23 | 38.57 | 28.74 | 30.24 | 38.22 | 43.35 | 64.37 | 61.96 | 14.38 |
| Roach | 1115 | 3 | 2 | 2 | 27.60 | 25.91 | 28.48 | 44.74 | 63.33 | 53.51 | 57.37 | 46.36 | 33.42 | 39.09 | 46.77 | 52.46 | 76.02 | 71.82 | 16.58 |
| Roach | 1116 | 3 | 2 | 2 | 21.38 | 19.74 | 21.58 | 38.12 | 49.29 | 39.05 | 45.15 | 34.82 | 27.58 | 32.36 | 37.52 | 39.31 | 57.85 | 53.88 | 12.02 |
| Roach | 1117 | 3 | 2 | 2 | 24.51 | 23.08 | 26.30 | 47.78 | 61.33 | 49.08 | 56.57 | 42.91 | 32.36 | 30.02 | 38.49 | 47.94 | 65.48 | 62.27 | 15.81 |
| Roach | 1118 | 3 | 2 | 2 | 23.90 | 25.99 | 26.60 | 41.91 | 57.05 | 47.48 | 51.84 | 39.71 | 33.97 | 31.70 | 38.51 | 46.74 | 67.74 | 64.11 | 15.43 |
| Roach | 1119 | 3 | 2 | 2 | 28.84 | 24.27 | 26.74 | 45.55 | 59.20 | 49.38 | 57.34 | 42.93 | 31.44 | 35.49 | 43.18 | 45.90 | 65.83 | 62.78 | 15.30 |
| Roach | 1120 | 3 | 2 | 2 | 21.61 | 21.82 | 21.77 | 34.99 | 45.08 | 37.69 | 43.38 | 30.94 | 28.08 | 31.29 | 36.03 | 39.57 | 61.43 | 58.48 | 12.90 |
| Roach | 1121 | 3 | 2 | 2 | 25.39 | 25.05 | 24.59 | 41.71 | 55.48 | 47.63 | 51.96 | 35.85 | 26.77 | 32.58 | 38.08 | 43.39 | 65.22 | 61.24 | 14.03 |
| Roach | 1122 | 3 | 2 | 2 | 22.53 | 22.83 | 22.81 | 39.78 | 50.45 | 39.64 | 46.78 | 35.45 | 30.15 | 33.83 | 37.77 | 42.27 | 60.85 | 55.44 | 12.88 |
| Roach | 1123 | 3 | 2 | 2 | 22.36 | 23.02 | 22.78 | 41.37 | 51.22 | 41.05 | 48.52 | 34.37 | 25.48 | 35.24 | 40.26 | 40.58 | 60.35 | 55.41 | 13.92 |
| Roach | 1124 | 3 | 2 | 2 | 22.68 | 23.68 | 24.49 | 40.91 | 56.77 | 47.35 | 50.94 | 38.08 | 28.96 | 30.02 | 36.08 | 42.55 | 63.20 | 59.76 | 13.32 |
| Roach | 1125 | 3 | 2 | 2 | 22.81 | 21.56 | 22.23 | 35.85 | 47.12 | 36.83 | 42.69 | 30.81 | 23.19 | 30.36 | 34.59 | 36.89 | 56.94 | 52.98 | 12.02 |
| Roach | 1126 | 3 | 2 | 2 | 27.01 | 24.77 | 24.90 | 41.92 | 51.60 | 42.23 | 51.31 | 34.65 | 26.26 | 33.75 | 39.78 | 43.16 | 63.63 | 60.29 | 13.65 |
| Roach | 1127 | 3 | 2 | 2 | 30.00 | 29.49 | 31.21 | 59.04 | 72.02 | 59.43 | 69.81 | 52.10 | 38.50 | 42.74 | 49.13 | 58.91 | 82.21 | 74.07 | 17.63 |
| Roach | 1128 | 3 | 2 | 2 | 27.00 | 25.66 | 26.28 | 46.87 | 56.80 | 44.92 | 55.19 | 40.78 | 24.95 | 31.99 | 38.46 | 46.58 | 67.86 | 66.72 | 14.84 |
| Roach | 1129 | 3 | 2 | 2 | 26.37 | 23.53 | 26.13 | 37.20 | 54.03 | 47.56 | 50.71 | 36.62 | 25.67 | 29.10 | 36.30 | 43.44 | 65.03 | 64.27 | 14.22 |
| Roach | 1130 | 3 | 2 | 2 | 20.93 | 20.81 | 21.79 | 37.10 | 47.88 | 40.29 | 46.41 | 32.24 | 20.67 | 23.51 | 31.10 | 37.16 | 55.03 | 56.64 | 11.18 |
| Roach | 1101 | 3 | 2 | 3 | 36.03 | 33.56 | 36.89 | 66.12 | 90.02 | 74.70 | 82.02 | 64.02 | 46.31 | 46.41 | 58.86 | 74.40 | 104.39 | 101.20 | 23.00 |
| Roach | 1102 | 3 | 2 | 3 | 33.06 | 32.65 | 33.05 | 55.78 | 72.90 | 58.81 | 69.88 | 51.88 | 40.25 | 46.02 | 52.11 | 57.83 | 87.21 | 81.26 | 18.01 |
| Roach | 1103 | 3 | 2 | 3 | 33.66 | 32.14 | 34.85 | 61.58 | 79.73 | 65.53 | 74.32 | 55.08 | 44.81 | 39.05 | 51.41 | 63.16 | 85.27 | 81.24 | 20.53 |
| Roach | 1104 | 3 | 2 | 3 | 35.23 | 36.14 | 38.84 | 60.44 | 82.71 | 63.96 | 73.00 | 58.66 | 44.89 | 44.76 | 56.14 | 65.23 | 92.19 | 90.30 | 18.88 |
| Roach | 1105 | 3 | 2 | 3 | 32.30 | 33.48 | 34.97 | 61.23 | 79.07 | 59.67 | 69.76 | 54.01 | 37.99 | 43.90 | 52.93 | 60.86 | 88.15 | 83.88 | 19.75 |
| Roach | 1106 | 3 | 2 | 3 | 24.84 | 23.71 | 25.67 | 42.95 | 57.09 | 46.45 | 52.21 | 40.04 | 30.95 | 37.58 | 69.36 | 40.80 | 67.60 | 60.41 | 13.99 |
| Roach | 1107 | 3 | 2 | 3 | 32.01 | 30.90 | 32.53 | 54.02 | 71.19 | 59.64 | 66.88 | 50.00 | 41.03 | 42.61 | 50.69 | 56.30 | 79.03 | 72.79 | 17.14 |
| Roach | 1108 | 3 | 2 | 3 | 31.11 | 27.69 | 30.33 | 54.10 | 70.10 | 59.25 | 65.96 | 49.45 | 38.40 | 38.99 | 47.29 | 54.50 | 81.50 | 76.38 | 16.55 |
| Roach | 1109 | 3 | 2 | 3 | 32.29 | 31.26 | 34.78 | 51.79 | 72.35 | 61.39 | 70.17 | 54.36 | 39.12 | 38.58 | 49.55 | 61.01 | 82.38 | 78.26 | 18.76 |
| Roach | 1110 | 3 | 2 | 3 | 26.46 | 25.17 | 26.56 | 44.09 | 57.25 | 45.55 | 53.77 | 40.84 | 33.52 | 33.73 | 42.28 | 47.28 | 68.93 | 65.66 | 15.00 |
| Roach | 1111 | 3 | 2 | 3 | 28.53 | 29.69 | 30.01 | 46.72 | 62.62 | 50.19 | 58.24 | 44.74 | 35.86 | 39.53 | 45.94 | 53.29 | 77.03 | 71.75 | 16.54 |
| Roach | 1112 | 3 | 2 | 3 | 26.50 | 25.12 | 26.00 | 42.94 | 55.96 | 45.55 | 52.05 | 37.96 | 30.11 | 33.02 | 40.86 | 43.20 | 63.21 | 61.80 | 14.96 |
| Roach | 1113 | 3 | 2 | 3 | 33.08 | 31.47 | 32.42 | 52.39 | 69.80 | 54.69 | 64.20 | 51.13 | 37.07 | 39.08 | 48.93 | 54.71 | 79.63 | 74.41 | 18.57 |
| Roach | 1114 | 3 | 2 | 3 | 23.93 | 24.22 | 23.92 | 39.55 | 54.64 | 42.84 | 47.33 | 38.67 | 29.05 | 29.93 | 37.39 | 43.52 | 63.32 | 61.54 | 14.14 |
| Roach | 1115 | 3 | 2 | 3 | 27.28 | 26.03 | 28.22 | 44.00 | 62.37 | 52.09 | 57.60 | 46.35 | 32.28 | 39.41 | 47.47 | 51.73 | 75.49 | 71.40 | 16.98 |
| Roach | 1116 | 3 | 2 | 3 | 21.13 | 21.39 | 21.58 | 40.84 | 49.36 | 38.57 | 44.65 | 34.73 | 28.28 | 32.02 | 37.09 | 39.28 | 56.69 | 53.43 | 12.15 |
| Roach | 1117 | 3 | 2 | 3 | 25.62 | 24.16 | 26.68 | 45.88 | 59.81 | 47.86 | 55.85 | 42.82 | 32.56 | 32.50 | 39.00 | 47.21 | 65.75 | 61.71 | 15.13 |
| Roach | 1118 | 3 | 2 | 3 | 23.23 | 25.34 | 26.32 | 40.44 | 57.85 | 47.89 | 51.47 | 39.41 | 34.24 | 31.15 | 38.14 | 46.74 | 67.47 | 63.34 | 15.18 |
| Roach | 1119 | 3 | 2 | 3 | 28.90 | 25.27 | 27.15 | 46.81 | 59.17 | 49.50 | 57.40 | 43.46 | 32.64 | 34.82 | 42.52 | 45.43 | 66.25 | 63.94 | 15.33 |
| Roach | 1120 | 3 | 2 | 3 | 20.98 | 21.66 | 21.32 | 35.37 | 45.95 | 38.38 | 43.79 | 30.64 | 27.30 | 30.57 | 35.90 | 39.54 | 60.94 | 57.84 | 12.94 |
| Roach | 1121 | 3 | 2 | 3 | 25.94 | 25.49 | 24.51 | 41.91 | 55.09 | 47.43 | 51.09 | 35.53 | 28.06 | 31.68 | 36.44 | 43.23 | 64.77 | 60.16 | 13.91 |
| Roach | 1122 | 3 | 2 | 3 | 21.89 | 23.63 | 22.81 | 39.91 | 50.49 | 39.17 | 46.54 | 35.46 | 30.96 | 32.81 | 37.76 | 42.90 | 61.47 | 55.43 | 12.86 |
| Roach | 1123 | 3 | 2 | 3 | 21.99 | 23.40 | 23.24 | 41.20 | 50.95 | 40.17 | 47.78 | 34.24 | 26.02 | 34.47 | 40.08 | 39.76 | 58.67 | 53.66 | 13.55 |
| Roach | 1124 | 3 | 2 | 3 | 22.41 | 24.85 | 24.75 | 41.95 | 56.92 | 47.06 | 50.31 | 37.17 | 28.92 | 28.71 | 35.87 | 42.37 | 63.08 | 58.89 | 13.69 |
| Roach | 1125 | 3 | 2 | 3 | 22.24 | 21.28 | 21.64 | 34.76 | 45.92 | 35.92 | 41.23 | 31.78 | 24.90 | 31.10 | 35.43 | 36.37 | 57.42 | 53.87 | 12.31 |
| Roach | 1126 | 3 | 2 | 3 | 27.31 | 25.88 | 24.79 | 40.63 | 51.43 | 41.90 | 50.50 | 34.82 | 26.72 | 34.87 | 40.94 | 42.49 | 63.55 | 60.31 | 13.35 |
| Roach | 1127 | 3 | 2 | 3 | 30.35 | 30.17 | 31.21 | 58.40 | 69.41 | 56.64 | 70.10 | 51.81 | 38.18 | 42.10 | 49.52 | 59.29 | 82.08 | 75.44 | 17.89 |
| Roach | 1128 | 3 | 2 | 3 | 27.13 | 26.35 | 26.87 | 45.81 | 57.00 | 44.88 | 56.55 | 41.06 | 23.01 | 31.26 | 39.05 | 45.97 | 68.19 | 65.55 | 14.71 |
| Roach | 1129 | 3 | 2 | 3 | 26.10 | 23.84 | 25.46 | 39.75 | 54.60 | 46.07 | 49.77 | 36.18 | 25.65 | 28.95 | 36.42 | 44.70 | 65.66 | 64.63 | 13.73 |
| Roach | 1130 | 3 | 2 | 3 | 20.33 | 21.37 | 21.97 | 38.00 | 48.19 | 40.24 | 46.28 | 32.25 | 20.64 | 22.30 | 30.21 | 37.45 | 54.45 | 56.40 | 11.06 |
| Roach | 1191 | 3 | 3 | 1 | 35.09 | 33.75 | 36.07 | 68.83 | 83.44 | 68.09 | 80.21 | 57.03 | 41.64 | 44.54 | 55.11 | 65.89 | 93.24 | 88.01 | 19.25 |
| Roach | 1192 | 3 | 3 | 1 | 29.25 | 32.60 | 33.85 | 57.78 | 79.92 | 64.66 | 71.53 | 59.26 | 43.78 | 45.28 | 53.07 | 67.36 | 92.66 | 82.67 | 20.18 |
| Roach | 1193 | 3 | 3 | 1 | 26.37 | 28.92 | 30.71 | 59.64 | 76.38 | 59.90 | 67.79 | 54.83 | 36.63 | 39.86 | 46.62 | 61.05 | 88.10 | 80.59 | 17.69 |
| Roach | 1194 | 3 | 3 | 1 | 29.04 | 26.16 | 27.34 | 50.43 | 61.66 | 49.08 | 60.09 | 44.30 | 30.95 | 35.16 | 42.06 | 50.46 | 69.68 | 65.65 | 15.16 |
| Roach | 1195 | 3 | 3 | 1 | 29.07 | 30.06 | 31.57 | 58.94 | 75.79 | 60.89 | 68.47 | 52.72 | 39.44 | 41.59 | 49.25 | 59.70 | 82.07 | 74.27 | 17.97 |
| Roach | 1196 | 3 | 3 | 1 | 32.62 | 33.95 | 35.25 | 58.79 | 77.94 | 61.46 | 70.90 | 55.51 | 42.37 | 35.00 | 48.35 | 60.75 | 77.65 | 77.73 | 18.49 |
| Roach | 1197 | 3 | 3 | 1 | 41.65 | 39.49 | 42.22 | 75.08 | 93.79 | 79.14 | 95.92 | 72.25 | 43.36 | 41.04 | 56.39 | 78.15 | 99.56 | 94.95 | 22.63 |
| Roach | 1198 | 3 | 3 | 1 | 25.62 | 28.72 | 28.93 | 52.46 | 66.24 | 53.88 | 61.54 | 44.64 | 34.96 | 33.63 | 42.84 | 52.93 | 69.80 | 66.85 | 15.83 |
| Roach | 1199 | 3 | 3 | 1 | 26.05 | 28.34 | 28.85 | 52.28 | 65.69 | 52.61 | 63.19 | 48.68 | 35.54 | 41.41 | 49.06 | 55.03 | 81.90 | 78.78 | 16.14 |
| Roach | 1200 | 3 | 3 | 1 | 31.46 | 29.19 | 33.47 | 59.88 | 77.39 | 64.29 | 72.53 | 53.38 | 40.31 | 44.15 | 51.75 | 61.18 | 84.01 | 77.49 | 17.13 |
| Roach | 1201 | 3 | 3 | 1 | 34.09 | 37.25 | 38.50 | 67.39 | 85.93 | 65.41 | 77.91 | 60.74 | 44.22 | 45.15 | 55.70 | 65.50 | 85.41 | 79.07 | 19.70 |
| Roach | 1202 | 3 | 3 | 1 | 26.08 | 29.61 | 31.15 | 49.05 | 70.80 | 60.51 | 62.92 | 49.41 | 31.62 | 35.16 | 43.03 | 53.63 | 72.04 | 65.85 | 16.98 |
| Roach | 1203 | 3 | 3 | 1 | 27.44 | 30.39 | 30.62 | 52.09 | 66.41 | 53.37 | 64.20 | 48.90 | 31.08 | 36.21 | 45.59 | 53.69 | 75.40 | 75.19 | 14.99 |
| Roach | 1204 | 3 | 3 | 1 | 29.01 | 29.67 | 30.34 | 53.15 | 67.36 | 52.70 | 63.98 | 50.14 | 35.91 | 40.03 | 48.50 | 53.89 | 79.46 | 75.58 | 16.26 |
| Roach | 1205 | 3 | 3 | 1 | 30.42 | 31.20 | 31.72 | 59.10 | 69.63 | 53.73 | 69.15 | 50.18 | 34.33 | 36.15 | 45.69 | 56.55 | 81.23 | 76.33 | 17.28 |
| Roach | 1206 | 3 | 3 | 1 | 33.94 | 32.60 | 34.66 | 58.22 | 75.13 | 60.11 | 70.68 | 55.21 | 37.87 | 42.77 | 51.04 | 63.79 | 91.10 | 85.92 | 18.41 |
| Roach | 1207 | 3 | 3 | 1 | 27.89 | 29.38 | 31.05 | 50.88 | 69.31 | 57.43 | 64.05 | 48.57 | 33.01 | 35.09 | 43.18 | 57.05 | 75.10 | 67.51 | 17.10 |
| Roach | 1208 | 3 | 3 | 1 | 27.42 | 29.06 | 29.43 | 51.46 | 69.91 | 59.00 | 62.94 | 49.66 | 32.11 | 41.21 | 48.97 | 55.09 | 83.26 | 79.98 | 17.04 |
| Roach | 1209 | 3 | 3 | 1 | 24.76 | 25.88 | 24.92 | 42.05 | 54.59 | 44.73 | 53.64 | 40.06 | 29.04 | 36.39 | 42.28 | 45.43 | 67.05 | 63.65 | 13.20 |
| Roach | 1210 | 3 | 3 | 1 | 26.06 | 26.61 | 26.75 | 52.04 | 64.04 | 53.37 | 60.54 | 42.16 | 28.36 | 35.93 | 43.30 | 47.77 | 70.86 | 67.59 | 15.00 |
| Roach | 1211 | 3 | 3 | 1 | 25.07 | 24.93 | 25.10 | 43.66 | 55.14 | 44.17 | 50.37 | 36.28 | 33.94 | 30.73 | 35.43 | 42.22 | 58.60 | 53.66 | 12.24 |
| Roach | 1212 | 3 | 3 | 1 | 32.44 | 35.25 | 35.30 | 58.48 | 76.01 | 58.65 | 71.10 | 54.48 | 39.37 | 44.86 | 54.86 | 62.70 | 85.86 | 79.62 | 18.94 |
| Roach | 1213 | 3 | 3 | 1 | 27.59 | 27.64 | 30.91 | 60.45 | 73.66 | 58.96 | 70.24 | 53.43 | 37.07 | 40.62 | 49.54 | 61.41 | 85.80 | 83.63 | 18.06 |
| Roach | 1214 | 3 | 3 | 1 | 30.41 | 29.16 | 30.63 | 56.38 | 74.01 | 58.85 | 67.24 | 54.41 | 42.06 | 40.17 | 49.60 | 59.74 | 84.42 | 82.68 | 16.49 |
| Roach | 1215 | 3 | 3 | 1 | 26.80 | 26.39 | 26.86 | 43.58 | 58.47 | 45.99 | 52.64 | 39.61 | 29.36 | 33.77 | 40.86 | 46.25 | 71.11 | 68.30 | 14.01 |
| Roach | 1216 | 3 | 3 | 1 | 30.36 | 31.60 | 32.75 | 61.91 | 75.06 | 59.76 | 70.71 | 52.73 | 39.23 | 40.56 | 50.14 | 59.83 | 82.26 | 78.66 | 17.02 |
| Roach | 1217 | 3 | 3 | 1 | 32.49 | 36.31 | 36.00 | 62.29 | 78.35 | 61.68 | 70.36 | 50.07 | 33.13 | 33.53 | 43.38 | 57.28 | 80.71 | 80.93 | 16.58 |
| Roach | 1218 | 3 | 3 | 1 | 27.38 | 27.63 | 30.00 | 49.45 | 66.12 | 54.35 | 62.32 | 49.04 | 35.99 | 33.57 | 43.69 | 53.47 | 74.06 | 71.37 | 16.75 |
| Roach | 1219 | 3 | 3 | 1 | 32.38 | 31.34 | 32.70 | 52.63 | 69.19 | 54.61 | 64.49 | 49.66 | 35.04 | 33.08 | 44.82 | 57.13 | 79.50 | 77.18 | 17.08 |
| Roach | 1220 | 3 | 3 | 1 | 21.36 | 21.79 | 21.05 | 38.82 | 49.88 | 40.75 | 46.89 | 34.37 | 23.86 | 29.45 | 33.82 | 38.48 | 56.63 | 51.74 | 12.13 |
| Roach | 1191 | 3 | 3 | 2 | 37.81 | 33.63 | 36.80 | 65.01 | 81.83 | 68.06 | 80.01 | 57.42 | 42.90 | 47.23 | 54.62 | 67.19 | 93.77 | 87.76 | 19.21 |
| Roach | 1192 | 3 | 3 | 2 | 33.17 | 33.68 | 35.12 | 54.71 | 79.21 | 66.42 | 70.91 | 58.23 | 44.00 | 45.30 | 54.53 | 66.48 | 91.16 | 82.69 | 20.34 |
| Roach | 1193 | 3 | 3 | 2 | 26.85 | 29.35 | 31.63 | 56.86 | 75.45 | 59.43 | 67.29 | 54.61 | 35.36 | 41.47 | 49.77 | 60.66 | 88.22 | 80.01 | 17.67 |
| Roach | 1194 | 3 | 3 | 2 | 28.88 | 25.84 | 27.91 | 49.89 | 61.24 | 50.34 | 59.83 | 44.11 | 31.45 | 35.78 | 42.68 | 50.75 | 69.95 | 65.14 | 15.30 |
| Roach | 1195 | 3 | 3 | 2 | 30.57 | 30.87 | 33.06 | 57.16 | 75.94 | 60.82 | 67.54 | 53.05 | 41.01 | 42.29 | 49.84 | 59.34 | 82.57 | 75.33 | 17.90 |
| Roach | 1196 | 3 | 3 | 2 | 34.83 | 34.17 | 35.91 | 57.16 | 77.16 | 62.35 | 70.68 | 54.81 | 43.24 | 36.66 | 48.74 | 61.25 | 78.68 | 76.85 | 18.58 |
| Roach | 1197 | 3 | 3 | 2 | 43.39 | 39.76 | 44.03 | 72.25 | 92.64 | 77.62 | 94.03 | 71.79 | 43.77 | 42.19 | 57.51 | 79.65 | 102.34 | 98.02 | 23.19 |
| Roach | 1198 | 3 | 3 | 2 | 27.75 | 29.68 | 30.41 | 50.75 | 64.99 | 53.11 | 62.73 | 44.59 | 33.62 | 33.64 | 42.75 | 52.38 | 70.74 | 65.59 | 16.10 |
| Roach | 1199 | 3 | 3 | 2 | 29.07 | 30.35 | 30.84 | 49.85 | 66.25 | 53.24 | 62.04 | 48.50 | 36.69 | 43.74 | 50.61 | 54.85 | 83.42 | 78.10 | 16.00 |
| Roach | 1200 | 3 | 3 | 2 | 33.12 | 28.83 | 34.65 | 57.86 | 76.13 | 63.87 | 72.15 | 54.14 | 40.29 | 45.84 | 53.42 | 61.25 | 86.15 | 79.04 | 18.45 |
| Roach | 1201 | 3 | 3 | 2 | 36.53 | 35.75 | 38.31 | 68.93 | 84.67 | 66.66 | 81.64 | 60.45 | 39.82 | 47.68 | 57.28 | 64.87 | 85.44 | 78.76 | 19.04 |
| Roach | 1202 | 3 | 3 | 2 | 28.29 | 29.92 | 31.77 | 47.67 | 69.86 | 60.66 | 63.38 | 49.55 | 30.57 | 37.73 | 46.48 | 53.51 | 72.20 | 66.27 | 16.83 |
| Roach | 1203 | 3 | 3 | 2 | 29.55 | 30.08 | 31.28 | 47.87 | 64.96 | 51.33 | 61.60 | 49.23 | 34.54 | 36.50 | 45.07 | 54.00 | 75.89 | 74.49 | 15.24 |
| Roach | 1204 | 3 | 3 | 2 | 30.00 | 29.90 | 30.67 | 53.42 | 68.04 | 54.08 | 63.16 | 48.99 | 34.12 | 43.47 | 50.73 | 55.03 | 80.47 | 77.60 | 16.64 |
| Roach | 1205 | 3 | 3 | 2 | 34.62 | 30.86 | 33.25 | 53.30 | 68.45 | 54.15 | 67.38 | 50.86 | 36.71 | 37.59 | 46.12 | 55.88 | 78.63 | 76.62 | 16.97 |
| Roach | 1206 | 3 | 3 | 2 | 34.31 | 33.69 | 36.22 | 56.84 | 75.83 | 60.98 | 71.07 | 54.21 | 38.72 | 41.76 | 51.21 | 64.18 | 92.09 | 87.60 | 18.32 |
| Roach | 1207 | 3 | 3 | 2 | 28.23 | 30.75 | 31.80 | 47.84 | 70.15 | 57.14 | 60.75 | 49.23 | 35.56 | 36.72 | 45.64 | 56.01 | 76.65 | 69.46 | 17.23 |
| Roach | 1208 | 3 | 3 | 2 | 29.60 | 29.14 | 30.53 | 48.81 | 69.45 | 59.86 | 62.89 | 49.41 | 33.58 | 40.74 | 48.18 | 54.53 | 79.79 | 76.61 | 16.80 |
| Roach | 1209 | 3 | 3 | 2 | 25.64 | 25.80 | 25.74 | 42.15 | 54.84 | 45.27 | 53.09 | 40.20 | 29.89 | 36.34 | 41.92 | 44.31 | 66.15 | 62.72 | 12.91 |
| Roach | 1210 | 3 | 3 | 2 | 27.48 | 26.61 | 27.76 | 49.23 | 64.03 | 53.25 | 59.43 | 42.32 | 29.08 | 36.80 | 44.43 | 48.67 | 72.40 | 70.25 | 15.07 |
| Roach | 1211 | 3 | 3 | 2 | 25.99 | 25.32 | 25.33 | 41.61 | 54.99 | 42.20 | 49.42 | 36.13 | 33.12 | 30.26 | 35.96 | 41.73 | 58.60 | 53.63 | 12.78 |
| Roach | 1212 | 3 | 3 | 2 | 34.62 | 35.12 | 34.96 | 56.01 | 75.32 | 60.25 | 70.91 | 54.76 | 40.79 | 44.80 | 54.53 | 62.79 | 88.32 | 83.53 | 20.72 |
| Roach | 1213 | 3 | 3 | 2 | 28.58 | 28.90 | 31.81 | 58.69 | 73.85 | 59.87 | 70.05 | 53.93 | 37.66 | 41.43 | 52.11 | 60.95 | 85.39 | 83.41 | 17.93 |
| Roach | 1214 | 3 | 3 | 2 | 31.45 | 29.89 | 31.41 | 56.39 | 74.22 | 58.96 | 66.73 | 54.21 | 43.37 | 39.10 | 50.09 | 60.33 | 87.27 | 86.51 | 16.96 |
| Roach | 1215 | 3 | 3 | 2 | 27.42 | 25.29 | 26.56 | 42.19 | 58.09 | 46.32 | 52.56 | 39.60 | 29.83 | 34.05 | 39.92 | 45.97 | 69.51 | 65.75 | 13.38 |
| Roach | 1216 | 3 | 3 | 2 | 31.40 | 31.49 | 33.70 | 59.13 | 74.18 | 58.67 | 70.23 | 53.33 | 38.90 | 42.34 | 51.72 | 59.27 | 82.39 | 77.86 | 17.41 |
| Roach | 1217 | 3 | 3 | 2 | 35.08 | 34.24 | 35.87 | 57.28 | 76.72 | 62.15 | 70.34 | 49.48 | 32.26 | 33.72 | 44.07 | 55.95 | 79.41 | 78.37 | 17.27 |
| Roach | 1218 | 3 | 3 | 2 | 28.69 | 27.02 | 30.27 | 46.37 | 65.54 | 53.78 | 60.45 | 48.15 | 34.81 | 33.11 | 42.89 | 54.44 | 74.85 | 72.75 | 16.84 |
| Roach | 1219 | 3 | 3 | 2 | 32.34 | 30.09 | 33.03 | 52.09 | 69.57 | 55.30 | 64.47 | 48.81 | 35.17 | 33.78 | 44.73 | 58.14 | 80.89 | 80.33 | 17.36 |
| Roach | 1220 | 3 | 3 | 2 | 21.55 | 21.60 | 21.50 | 37.86 | 49.27 | 40.20 | 45.45 | 34.45 | 25.35 | 30.79 | 33.91 | 38.84 | 56.49 | 51.37 | 12.05 |
| Roach | 1191 | 3 | 3 | 3 | 38.12 | 35.31 | 37.23 | 63.68 | 82.89 | 67.55 | 77.74 | 57.46 | 44.99 | 46.16 | 55.35 | 66.92 | 93.40 | 87.40 | 19.51 |
| Roach | 1192 | 3 | 3 | 3 | 32.59 | 33.25 | 35.28 | 53.68 | 77.97 | 64.81 | 71.46 | 59.44 | 43.84 | 45.55 | 54.40 | 66.00 | 91.00 | 83.15 | 20.78 |
| Roach | 1193 | 3 | 3 | 3 | 26.96 | 29.12 | 31.40 | 56.77 | 74.99 | 58.42 | 67.12 | 55.06 | 36.58 | 41.75 | 48.09 | 61.00 | 87.94 | 79.70 | 18.47 |
| Roach | 1194 | 3 | 3 | 3 | 27.97 | 27.32 | 28.23 | 49.92 | 61.20 | 48.43 | 58.70 | 43.53 | 29.64 | 36.01 | 42.64 | 49.72 | 70.35 | 65.24 | 15.46 |
| Roach | 1195 | 3 | 3 | 3 | 31.37 | 30.70 | 33.22 | 56.19 | 74.66 | 58.82 | 67.69 | 52.64 | 40.09 | 42.45 | 49.87 | 59.63 | 82.46 | 75.41 | 17.43 |
| Roach | 1196 | 3 | 3 | 3 | 34.12 | 32.40 | 35.55 | 57.31 | 79.52 | 64.29 | 70.74 | 54.81 | 43.00 | 36.66 | 48.12 | 61.29 | 82.54 | 79.48 | 18.01 |
| Roach | 1197 | 3 | 3 | 3 | 44.12 | 39.68 | 43.97 | 71.48 | 92.60 | 78.67 | 95.55 | 71.57 | 41.79 | 42.12 | 57.18 | 79.34 | 100.37 | 96.95 | 23.08 |
| Roach | 1198 | 3 | 3 | 3 | 27.52 | 29.82 | 30.00 | 50.64 | 64.63 | 53.01 | 61.80 | 44.39 | 35.22 | 34.43 | 42.40 | 53.18 | 73.26 | 67.45 | 15.80 |
| Roach | 1199 | 3 | 3 | 3 | 28.79 | 28.95 | 30.04 | 51.35 | 66.52 | 53.52 | 62.72 | 48.55 | 36.75 | 43.02 | 49.81 | 55.36 | 82.89 | 78.63 | 16.20 |
| Roach | 1200 | 3 | 3 | 3 | 31.93 | 28.75 | 34.50 | 59.90 | 77.68 | 64.41 | 72.42 | 53.84 | 43.56 | 42.51 | 50.07 | 62.11 | 85.23 | 78.41 | 18.33 |
| Roach | 1201 | 3 | 3 | 3 | 36.75 | 36.85 | 38.38 | 69.95 | 86.02 | 66.27 | 80.73 | 60.06 | 41.60 | 44.28 | 55.02 | 65.46 | 84.52 | 78.59 | 19.23 |
| Roach | 1202 | 3 | 3 | 3 | 26.66 | 30.39 | 31.81 | 46.63 | 69.17 | 59.75 | 63.04 | 49.53 | 30.60 | 38.28 | 46.72 | 53.18 | 72.37 | 66.94 | 16.62 |
| Roach | 1203 | 3 | 3 | 3 | 29.00 | 28.30 | 29.90 | 48.63 | 65.08 | 52.45 | 61.23 | 48.14 | 35.00 | 36.57 | 45.09 | 54.31 | 76.75 | 74.23 | 14.99 |
| Roach | 1204 | 3 | 3 | 3 | 29.80 | 29.58 | 31.13 | 53.12 | 68.84 | 54.51 | 64.25 | 49.67 | 34.06 | 41.60 | 49.37 | 54.74 | 81.36 | 78.77 | 16.23 |
| Roach | 1205 | 3 | 3 | 3 | 33.29 | 32.68 | 33.62 | 55.68 | 69.50 | 53.50 | 66.73 | 51.06 | 37.32 | 38.45 | 46.41 | 55.51 | 81.26 | 75.71 | 17.23 |
| Roach | 1206 | 3 | 3 | 3 | 34.01 | 33.50 | 35.56 | 57.77 | 76.17 | 60.73 | 70.11 | 54.16 | 38.18 | 42.40 | 51.23 | 64.09 | 92.16 | 87.62 | 18.55 |
| Roach | 1207 | 3 | 3 | 3 | 28.82 | 31.63 | 32.81 | 49.79 | 68.19 | 53.98 | 60.80 | 49.73 | 35.64 | 39.10 | 46.55 | 56.70 | 78.12 | 70.54 | 17.62 |
| Roach | 1208 | 3 | 3 | 3 | 27.78 | 29.14 | 30.03 | 48.41 | 69.30 | 59.54 | 62.10 | 49.61 | 34.30 | 39.47 | 47.39 | 54.85 | 81.31 | 78.07 | 17.03 |
| Roach | 1209 | 3 | 3 | 3 | 25.00 | 27.26 | 25.84 | 41.29 | 55.07 | 45.46 | 52.61 | 40.06 | 29.92 | 36.49 | 41.91 | 45.06 | 66.95 | 63.90 | 13.05 |
| Roach | 1210 | 3 | 3 | 3 | 27.27 | 26.14 | 27.61 | 50.01 | 64.27 | 53.37 | 59.05 | 42.02 | 28.62 | 37.73 | 45.09 | 47.77 | 72.20 | 69.44 | 15.18 |
| Roach | 1211 | 3 | 3 | 3 | 26.22 | 26.00 | 25.85 | 42.73 | 55.27 | 44.03 | 49.79 | 36.35 | 32.16 | 32.61 | 38.61 | 41.41 | 57.16 | 53.48 | 12.71 |
| Roach | 1212 | 3 | 3 | 3 | 33.49 | 34.18 | 35.42 | 56.28 | 75.42 | 59.43 | 70.87 | 54.41 | 39.66 | 45.86 | 56.33 | 61.90 | 85.87 | 79.99 | 19.63 |
| Roach | 1213 | 3 | 3 | 3 | 28.51 | 29.18 | 32.10 | 59.34 | 73.91 | 60.03 | 71.14 | 54.11 | 37.56 | 40.70 | 51.46 | 60.81 | 83.76 | 81.65 | 18.08 |
| Roach | 1214 | 3 | 3 | 3 | 30.01 | 29.47 | 32.00 | 53.81 | 72.86 | 57.29 | 67.04 | 54.61 | 41.77 | 38.42 | 49.77 | 60.20 | 87.35 | 84.81 | 16.65 |
| Roach | 1215 | 3 | 3 | 3 | 27.67 | 24.96 | 26.72 | 43.83 | 57.52 | 45.44 | 51.55 | 39.31 | 30.80 | 34.82 | 41.13 | 46.01 | 70.11 | 67.65 | 14.03 |
| Roach | 1216 | 3 | 3 | 3 | 31.87 | 31.60 | 33.47 | 58.11 | 75.23 | 59.89 | 69.80 | 53.07 | 39.74 | 41.94 | 50.87 | 58.81 | 81.99 | 76.70 | 17.70 |
| Roach | 1217 | 3 | 3 | 3 | 33.42 | 34.37 | 36.37 | 61.43 | 77.93 | 62.13 | 70.99 | 49.30 | 32.88 | 34.12 | 43.87 | 57.05 | 82.43 | 80.17 | 17.55 |
| Roach | 1218 | 3 | 3 | 3 | 27.41 | 26.80 | 29.66 | 47.39 | 65.10 | 52.85 | 60.45 | 48.35 | 34.86 | 33.42 | 43.70 | 53.21 | 72.28 | 69.84 | 16.46 |
| Roach | 1219 | 3 | 3 | 3 | 32.41 | 29.40 | 32.12 | 53.21 | 70.72 | 56.54 | 65.12 | 49.21 | 35.06 | 34.09 | 44.41 | 57.63 | 82.12 | 80.12 | 17.38 |
| Roach | 1220 | 3 | 3 | 3 | 22.14 | 20.40 | 20.70 | 38.47 | 47.68 | 38.46 | 46.15 | 34.51 | 24.64 | 29.47 | 33.66 | 39.10 | 57.43 | 52.28 | 12.05 |
| Prussian carp | 1061 | 1 | 1 | 1 | 26.95 | 28.20 | 32.24 | 31.81 | 55.29 | 36.83 | 48.45 | 48.63 | 35.81 | 29.57 | 40.15 | 56.40 | 68.86 | 61.02 | 18.62 |
| Prussian carp | 1062 | 1 | 1 | 1 | 29.75 | 29.70 | 34.28 | 37.39 | 59.08 | 39.19 | 55.83 | 54.60 | 39.25 | 32.80 | 46.58 | 67.37 | 84.81 | 76.64 | 23.78 |
| Prussian carp | 1063 | 1 | 1 | 1 | 27.85 | 26.52 | 32.76 | 38.88 | 60.48 | 41.05 | 54.42 | 52.28 | 35.23 | 32.86 | 43.91 | 60.61 | 77.33 | 69.47 | 19.90 |
| Prussian carp | 1064 | 1 | 1 | 1 | 25.35 | 29.28 | 33.71 | 35.51 | 58.79 | 39.63 | 52.98 | 52.55 | 36.88 | 28.73 | 42.09 | 64.80 | 80.74 | 71.89 | 21.30 |
| Prussian carp | 1065 | 1 | 1 | 1 | 24.81 | 27.21 | 31.19 | 32.41 | 55.48 | 36.57 | 50.04 | 49.84 | 33.53 | 26.34 | 39.36 | 57.91 | 70.30 | 61.70 | 20.50 |
| Prussian carp | 1066 | 1 | 1 | 1 | 28.65 | 29.15 | 34.04 | 33.76 | 56.34 | 38.20 | 53.58 | 49.66 | 35.28 | 30.98 | 41.40 | 58.97 | 75.32 | 64.53 | 19.62 |
| Prussian carp | 1067 | 1 | 1 | 1 | 27.09 | 29.26 | 35.02 | 45.29 | 67.87 | 45.33 | 58.77 | 61.71 | 39.88 | 33.05 | 45.47 | 68.23 | 81.64 | 74.41 | 20.53 |
| Prussian carp | 1068 | 1 | 1 | 1 | 28.88 | 29.32 | 34.23 | 39.19 | 64.38 | 44.07 | 56.57 | 55.06 | 37.57 | 32.60 | 44.99 | 64.96 | 81.10 | 71.15 | 22.52 |
| Prussian carp | 1069 | 1 | 1 | 1 | 27.68 | 25.64 | 31.75 | 31.42 | 51.62 | 33.78 | 49.02 | 46.43 | 33.00 | 27.33 | 37.25 | 56.10 | 68.68 | 60.25 | 19.51 |
| Prussian carp | 1070 | 1 | 1 | 1 | 24.88 | 22.74 | 29.73 | 31.45 | 55.96 | 36.68 | 48.93 | 51.44 | 32.85 | 32.38 | 43.91 | 53.89 | 65.25 | 57.47 | 19.36 |
| Prussian carp | 1071 | 1 | 1 | 1 | 27.22 | 27.41 | 34.35 | 38.47 | 63.65 | 43.69 | 56.73 | 56.64 | 38.91 | 28.65 | 43.67 | 68.17 | 80.31 | 70.01 | 23.07 |
| Prussian carp | 1072 | 1 | 1 | 1 | 30.29 | 31.43 | 38.08 | 42.97 | 66.85 | 43.60 | 61.54 | 59.08 | 43.59 | 30.00 | 45.18 | 71.81 | 85.43 | 77.75 | 23.98 |
| Prussian carp | 1073 | 1 | 1 | 1 | 24.16 | 23.71 | 29.14 | 31.51 | 55.56 | 40.75 | 45.33 | 42.09 | 29.65 | 24.71 | 36.89 | 45.01 | 53.48 | 46.12 | 17.68 |
| Prussian carp | 1074 | 1 | 1 | 1 | 30.46 | 28.26 | 32.76 | 36.41 | 62.23 | 43.30 | 53.74 | 54.30 | 39.62 | 35.01 | 47.08 | 65.71 | 82.80 | 71.94 | 20.32 |
| Prussian carp | 1075 | 1 | 1 | 1 | 26.61 | 26.60 | 34.27 | 36.32 | 64.00 | 44.27 | 56.27 | 57.48 | 41.35 | 25.55 | 41.60 | 66.00 | 74.81 | 65.06 | 22.99 |
| Prussian carp | 1076 | 1 | 1 | 1 | 23.16 | 26.14 | 30.98 | 39.83 | 63.21 | 43.31 | 54.16 | 54.30 | 33.23 | 28.74 | 40.39 | 61.21 | 73.72 | 66.61 | 19.14 |
| Prussian carp | 1077 | 1 | 1 | 1 | 24.37 | 32.83 | 35.41 | 44.93 | 69.39 | 46.42 | 59.25 | 58.49 | 42.22 | 31.39 | 48.60 | 72.87 | 89.74 | 82.17 | 23.71 |
| Prussian carp | 1078 | 1 | 1 | 1 | 25.89 | 22.65 | 31.88 | 34.85 | 60.63 | 44.90 | 53.42 | 53.26 | 32.91 | 27.62 | 39.62 | 57.00 | 65.83 | 57.51 | 20.31 |
| Prussian carp | 1079 | 1 | 1 | 1 | 31.17 | 26.25 | 35.03 | 34.36 | 62.05 | 40.29 | 54.63 | 55.96 | 41.95 | 28.22 | 40.57 | 64.22 | 73.70 | 63.73 | 21.96 |
| Prussian carp | 1080 | 1 | 1 | 1 | 30.44 | 30.27 | 34.57 | 36.07 | 63.14 | 41.51 | 55.22 | 55.71 | 40.65 | 30.83 | 42.75 | 64.36 | 75.53 | 64.28 | 20.66 |
| Prussian carp | 1081 | 1 | 1 | 1 | 28.43 | 28.87 | 33.17 | 32.24 | 56.26 | 34.78 | 49.51 | 51.72 | 37.39 | 28.89 | 39.01 | 58.80 | 67.86 | 59.46 | 17.79 |
| Prussian carp | 1082 | 1 | 1 | 1 | 26.47 | 31.12 | 36.13 | 29.34 | 53.39 | 31.91 | 48.17 | 45.65 | 34.22 | 26.81 | 38.10 | 58.14 | 72.80 | 65.61 | 18.29 |
| Prussian carp | 1083 | 1 | 1 | 1 | 25.41 | 26.55 | 30.80 | 32.84 | 55.79 | 35.72 | 47.74 | 48.64 | 33.99 | 23.46 | 36.50 | 56.71 | 66.78 | 57.46 | 19.50 |
| Prussian carp | 1084 | 1 | 1 | 1 | 28.22 | 27.28 | 33.58 | 31.95 | 62.63 | 44.79 | 52.85 | 53.20 | 34.76 | 27.61 | 40.56 | 59.31 | 73.50 | 66.72 | 19.52 |
| Prussian carp | 1085 | 1 | 1 | 1 | 30.84 | 32.76 | 37.04 | 42.49 | 70.64 | 49.48 | 62.10 | 62.52 | 46.21 | 35.23 | 51.72 | 75.03 | 89.09 | 80.05 | 24.93 |
| Prussian carp | 1086 | 1 | 1 | 1 | 28.14 | 31.01 | 34.58 | 35.82 | 57.96 | 37.16 | 52.74 | 50.74 | 35.89 | 26.95 | 40.43 | 61.79 | 75.32 | 67.28 | 22.10 |
| Prussian carp | 1087 | 1 | 1 | 1 | 23.67 | 25.01 | 31.49 | 35.55 | 59.35 | 38.46 | 51.58 | 54.69 | 36.57 | 25.71 | 38.60 | 60.68 | 72.49 | 67.01 | 19.71 |
| Prussian carp | 1088 | 1 | 1 | 1 | 22.75 | 24.27 | 28.14 | 30.13 | 47.79 | 30.86 | 43.34 | 42.70 | 28.62 | 25.26 | 33.02 | 50.25 | 61.69 | 55.02 | 16.16 |
| Prussian carp | 1089 | 1 | 1 | 1 | 25.79 | 30.04 | 33.67 | 39.43 | 60.66 | 38.58 | 56.63 | 56.81 | 37.10 | 31.60 | 45.86 | 67.11 | 79.54 | 74.60 | 20.14 |
| Prussian carp | 1090 | 1 | 1 | 1 | 23.02 | 24.60 | 27.13 | 35.64 | 52.21 | 34.88 | 49.02 | 47.19 | 29.84 | 25.24 | 35.13 | 55.32 | 68.21 | 61.12 | 17.69 |
| Prussian carp | 1061 | 1 | 1 | 2 | 24.97 | 28.15 | 32.96 | 30.82 | 55.34 | 35.54 | 48.17 | 47.63 | 35.93 | 29.13 | 39.21 | 57.02 | 68.90 | 60.90 | 18.98 |
| Prussian carp | 1062 | 1 | 1 | 2 | 30.06 | 31.76 | 35.15 | 39.52 | 59.44 | 37.34 | 54.47 | 52.94 | 39.60 | 35.05 | 48.67 | 66.50 | 85.33 | 77.80 | 23.03 |
| Prussian carp | 1063 | 1 | 1 | 2 | 27.43 | 27.73 | 32.86 | 38.11 | 61.40 | 42.42 | 54.14 | 52.81 | 37.42 | 31.93 | 43.48 | 62.33 | 77.59 | 70.05 | 21.41 |
| Prussian carp | 1064 | 1 | 1 | 2 | 26.28 | 28.35 | 31.22 | 35.51 | 57.03 | 35.45 | 52.87 | 52.39 | 35.09 | 30.13 | 42.28 | 64.14 | 78.81 | 70.98 | 21.10 |
| Prussian carp | 1065 | 1 | 1 | 2 | 25.69 | 27.79 | 32.00 | 33.42 | 54.75 | 33.76 | 50.56 | 50.96 | 33.42 | 27.09 | 38.55 | 57.39 | 70.20 | 60.90 | 19.00 |
| Prussian carp | 1066 | 1 | 1 | 2 | 29.59 | 30.56 | 33.16 | 34.44 | 55.41 | 37.36 | 51.39 | 48.53 | 35.30 | 33.66 | 42.22 | 59.20 | 76.18 | 63.89 | 19.03 |
| Prussian carp | 1067 | 1 | 1 | 2 | 26.28 | 29.96 | 34.22 | 43.87 | 67.88 | 45.45 | 59.70 | 62.69 | 39.56 | 30.17 | 44.99 | 68.99 | 82.93 | 73.49 | 21.70 |
| Prussian carp | 1068 | 1 | 1 | 2 | 29.05 | 30.72 | 35.30 | 40.52 | 66.51 | 47.69 | 56.40 | 55.17 | 39.35 | 30.95 | 44.07 | 66.19 | 79.97 | 72.52 | 23.81 |
| Prussian carp | 1069 | 1 | 1 | 2 | 25.70 | 25.26 | 30.78 | 31.39 | 52.39 | 32.71 | 48.30 | 46.11 | 32.60 | 26.78 | 36.49 | 53.51 | 69.06 | 59.41 | 17.85 |
| Prussian carp | 1070 | 1 | 1 | 2 | 25.58 | 22.39 | 29.20 | 31.81 | 58.17 | 41.20 | 50.08 | 50.94 | 33.12 | 30.85 | 41.89 | 54.01 | 64.21 | 57.39 | 18.75 |
| Prussian carp | 1071 | 1 | 1 | 2 | 24.33 | 26.82 | 34.15 | 40.18 | 65.48 | 44.24 | 57.42 | 57.20 | 41.50 | 28.44 | 43.04 | 69.30 | 79.55 | 69.14 | 22.83 |
| Prussian carp | 1072 | 1 | 1 | 2 | 32.98 | 36.83 | 40.75 | 41.18 | 65.88 | 43.50 | 61.84 | 57.85 | 40.76 | 28.89 | 43.92 | 71.86 | 86.17 | 77.22 | 24.13 |
| Prussian carp | 1073 | 1 | 1 | 2 | 24.67 | 24.18 | 29.27 | 28.76 | 53.24 | 36.13 | 45.01 | 44.99 | 32.23 | 24.80 | 36.77 | 45.13 | 53.71 | 45.77 | 17.50 |
| Prussian carp | 1074 | 1 | 1 | 2 | 28.52 | 29.30 | 35.01 | 34.54 | 62.45 | 41.08 | 52.99 | 54.94 | 40.76 | 33.48 | 46.51 | 64.57 | 77.95 | 69.93 | 19.98 |
| Prussian carp | 1075 | 1 | 1 | 2 | 25.56 | 27.72 | 36.35 | 35.48 | 64.37 | 44.25 | 55.66 | 56.08 | 40.34 | 26.83 | 42.24 | 65.91 | 78.02 | 66.69 | 23.53 |
| Prussian carp | 1076 | 1 | 1 | 2 | 26.21 | 27.92 | 31.67 | 37.95 | 63.24 | 42.19 | 53.92 | 54.65 | 35.53 | 29.22 | 40.94 | 61.33 | 74.02 | 68.02 | 18.47 |
| Prussian carp | 1077 | 1 | 1 | 2 | 27.35 | 32.85 | 35.89 | 42.71 | 68.31 | 44.72 | 58.19 | 58.18 | 42.52 | 32.79 | 49.45 | 71.69 | 88.03 | 80.36 | 24.11 |
| Prussian carp | 1078 | 1 | 1 | 2 | 24.05 | 24.58 | 32.72 | 31.63 | 60.90 | 43.61 | 51.17 | 54.17 | 34.62 | 27.51 | 38.55 | 57.74 | 69.47 | 58.53 | 20.20 |
| Prussian carp | 1079 | 1 | 1 | 2 | 30.54 | 26.58 | 34.06 | 34.72 | 61.86 | 39.66 | 55.17 | 56.74 | 41.54 | 28.98 | 41.46 | 64.75 | 75.08 | 63.80 | 22.05 |
| Prussian carp | 1080 | 1 | 1 | 2 | 32.69 | 32.94 | 37.37 | 37.01 | 64.33 | 41.16 | 52.74 | 55.50 | 41.51 | 31.15 | 43.14 | 65.62 | 78.66 | 66.16 | 21.52 |
| Prussian carp | 1081 | 1 | 1 | 2 | 28.34 | 29.68 | 32.86 | 32.76 | 55.22 | 33.48 | 49.62 | 50.34 | 34.19 | 29.83 | 40.22 | 58.37 | 68.80 | 59.29 | 17.35 |
| Prussian carp | 1082 | 1 | 1 | 2 | 27.81 | 30.92 | 35.59 | 28.63 | 52.34 | 30.98 | 49.88 | 46.34 | 30.59 | 30.79 | 39.88 | 57.47 | 73.88 | 65.74 | 17.49 |
| Prussian carp | 1083 | 1 | 1 | 2 | 24.99 | 26.13 | 30.84 | 32.30 | 54.75 | 35.10 | 48.17 | 48.27 | 33.16 | 23.23 | 35.78 | 56.39 | 65.56 | 57.47 | 19.00 |
| Prussian carp | 1084 | 1 | 1 | 2 | 29.12 | 26.86 | 33.63 | 33.46 | 61.10 | 42.10 | 51.92 | 52.89 | 33.65 | 29.00 | 41.14 | 59.36 | 74.98 | 68.13 | 19.59 |
| Prussian carp | 1085 | 1 | 1 | 2 | 29.94 | 31.81 | 36.87 | 42.12 | 71.51 | 50.77 | 61.59 | 61.82 | 47.24 | 36.14 | 51.30 | 73.52 | 89.00 | 80.22 | 25.02 |
| Prussian carp | 1086 | 1 | 1 | 2 | 27.97 | 31.37 | 35.04 | 36.27 | 57.26 | 38.45 | 52.92 | 49.94 | 35.12 | 27.66 | 41.33 | 60.87 | 75.15 | 68.69 | 22.00 |
| Prussian carp | 1087 | 1 | 1 | 2 | 23.69 | 26.64 | 32.25 | 37.68 | 60.72 | 39.23 | 51.79 | 54.62 | 35.77 | 27.82 | 40.55 | 59.36 | 71.93 | 66.09 | 18.15 |
| Prussian carp | 1088 | 1 | 1 | 2 | 21.34 | 23.77 | 28.34 | 30.66 | 48.32 | 27.94 | 43.10 | 42.68 | 28.64 | 23.35 | 32.27 | 49.36 | 62.43 | 54.90 | 15.10 |
| Prussian carp | 1089 | 1 | 1 | 2 | 26.01 | 30.07 | 34.92 | 40.42 | 62.81 | 40.87 | 56.50 | 56.10 | 37.96 | 32.49 | 46.53 | 68.01 | 82.54 | 78.10 | 20.51 |
| Prussian carp | 1090 | 1 | 1 | 2 | 22.08 | 26.32 | 29.54 | 36.65 | 53.93 | 34.65 | 47.81 | 45.44 | 31.52 | 24.59 | 35.18 | 56.12 | 68.00 | 62.43 | 18.11 |
| Prussian carp | 1061 | 1 | 1 | 3 | 26.72 | 27.01 | 31.89 | 32.02 | 55.67 | 36.42 | 49.02 | 49.28 | 34.98 | 31.36 | 40.86 | 56.80 | 69.43 | 63.51 | 19.42 |
| Prussian carp | 1062 | 1 | 1 | 3 | 28.49 | 30.55 | 34.82 | 38.80 | 59.86 | 37.74 | 55.06 | 54.38 | 39.44 | 32.35 | 47.22 | 67.35 | 86.42 | 78.56 | 22.94 |
| Prussian carp | 1063 | 1 | 1 | 3 | 27.33 | 26.77 | 32.99 | 37.13 | 60.07 | 41.81 | 55.13 | 53.05 | 36.09 | 32.15 | 43.19 | 62.53 | 78.96 | 70.88 | 20.09 |
| Prussian carp | 1064 | 1 | 1 | 3 | 25.82 | 28.84 | 32.46 | 36.44 | 57.98 | 36.93 | 53.04 | 53.09 | 35.15 | 30.63 | 42.44 | 63.84 | 78.95 | 71.59 | 20.32 |
| Prussian carp | 1065 | 1 | 1 | 3 | 26.82 | 28.75 | 33.36 | 34.30 | 56.13 | 33.36 | 49.52 | 49.63 | 34.57 | 26.04 | 37.44 | 58.28 | 70.68 | 62.58 | 19.70 |
| Prussian carp | 1066 | 1 | 1 | 3 | 28.05 | 29.41 | 33.64 | 35.32 | 55.83 | 36.30 | 52.10 | 50.24 | 35.23 | 31.61 | 41.63 | 60.35 | 76.66 | 66.54 | 21.11 |
| Prussian carp | 1067 | 1 | 1 | 3 | 23.67 | 28.02 | 34.36 | 40.28 | 68.80 | 46.91 | 59.98 | 62.10 | 37.29 | 33.68 | 45.19 | 67.16 | 80.87 | 73.20 | 21.30 |
| Prussian carp | 1068 | 1 | 1 | 3 | 28.76 | 30.17 | 35.83 | 38.65 | 65.43 | 45.30 | 56.89 | 55.11 | 39.15 | 29.42 | 43.28 | 67.10 | 80.53 | 72.25 | 24.13 |
| Prussian carp | 1069 | 1 | 1 | 3 | 25.67 | 27.61 | 33.15 | 29.52 | 52.53 | 33.93 | 48.33 | 46.05 | 33.19 | 26.27 | 37.29 | 55.62 | 68.48 | 59.32 | 18.50 |
| Prussian carp | 1070 | 1 | 1 | 3 | 24.19 | 23.45 | 29.42 | 32.60 | 56.64 | 37.29 | 48.50 | 51.26 | 34.65 | 30.76 | 43.15 | 54.23 | 65.54 | 58.69 | 18.47 |
| Prussian carp | 1071 | 1 | 1 | 3 | 28.10 | 27.76 | 35.30 | 37.95 | 63.64 | 43.18 | 56.34 | 57.06 | 43.11 | 27.20 | 40.76 | 68.78 | 81.76 | 70.91 | 22.91 |
| Prussian carp | 1072 | 1 | 1 | 3 | 30.06 | 35.82 | 41.33 | 43.89 | 67.99 | 45.04 | 60.82 | 59.08 | 45.12 | 30.08 | 45.85 | 73.35 | 85.98 | 78.42 | 24.40 |
| Prussian carp | 1073 | 1 | 1 | 3 | 23.08 | 23.67 | 29.14 | 31.95 | 54.59 | 37.62 | 42.93 | 43.35 | 32.89 | 23.54 | 35.37 | 45.49 | 53.14 | 45.44 | 17.70 |
| Prussian carp | 1074 | 1 | 1 | 3 | 30.23 | 27.92 | 33.59 | 35.41 | 61.58 | 40.94 | 53.92 | 54.28 | 39.67 | 33.75 | 45.84 | 64.10 | 79.03 | 70.04 | 21.10 |
| Prussian carp | 1075 | 1 | 1 | 3 | 24.73 | 27.42 | 35.00 | 38.05 | 63.44 | 42.97 | 56.14 | 56.49 | 40.64 | 25.08 | 41.87 | 64.93 | 75.12 | 63.84 | 23.41 |
| Prussian carp | 1076 | 1 | 1 | 3 | 24.09 | 27.66 | 32.13 | 36.46 | 63.70 | 44.39 | 53.50 | 54.45 | 34.69 | 35.11 | 44.69 | 57.28 | 73.61 | 67.57 | 18.93 |
| Prussian carp | 1077 | 1 | 1 | 3 | 25.17 | 31.29 | 34.58 | 43.88 | 65.75 | 43.75 | 59.39 | 58.77 | 42.21 | 33.93 | 48.03 | 73.81 | 92.12 | 84.91 | 23.93 |
| Prussian carp | 1078 | 1 | 1 | 3 | 27.55 | 22.23 | 31.25 | 35.15 | 59.50 | 41.79 | 51.43 | 52.65 | 33.47 | 27.91 | 39.13 | 57.90 | 68.16 | 60.19 | 19.91 |
| Prussian carp | 1079 | 1 | 1 | 3 | 29.66 | 26.70 | 34.37 | 34.92 | 62.90 | 40.43 | 54.86 | 56.98 | 41.52 | 27.15 | 41.35 | 66.32 | 76.21 | 65.44 | 22.34 |
| Prussian carp | 1080 | 1 | 1 | 3 | 30.27 | 29.74 | 34.91 | 37.46 | 61.97 | 41.00 | 55.48 | 54.45 | 39.79 | 31.72 | 42.58 | 64.27 | 78.10 | 64.43 | 21.68 |
| Prussian carp | 1081 | 1 | 1 | 3 | 27.97 | 28.26 | 32.78 | 32.08 | 56.26 | 34.85 | 49.24 | 51.69 | 36.55 | 29.76 | 40.04 | 58.43 | 70.55 | 60.19 | 18.33 |
| Prussian carp | 1082 | 1 | 1 | 3 | 30.65 | 29.18 | 34.10 | 29.15 | 51.69 | 30.64 | 50.30 | 46.23 | 33.17 | 27.65 | 37.92 | 58.30 | 72.25 | 65.26 | 18.57 |
| Prussian carp | 1083 | 1 | 1 | 3 | 25.92 | 26.27 | 31.04 | 32.20 | 55.84 | 36.68 | 48.78 | 48.47 | 32.15 | 24.28 | 36.74 | 56.81 | 68.51 | 58.20 | 20.53 |
| Prussian carp | 1084 | 1 | 1 | 3 | 29.39 | 26.72 | 33.92 | 33.36 | 61.44 | 42.88 | 53.57 | 54.12 | 33.73 | 27.80 | 40.22 | 60.96 | 74.52 | 68.48 | 20.33 |
| Prussian carp | 1085 | 1 | 1 | 3 | 30.74 | 32.11 | 36.02 | 42.12 | 71.50 | 51.16 | 61.67 | 61.31 | 46.87 | 37.34 | 50.24 | 73.63 | 90.77 | 79.09 | 25.70 |
| Prussian carp | 1086 | 1 | 1 | 3 | 26.54 | 29.15 | 33.35 | 35.23 | 57.35 | 38.84 | 53.55 | 49.88 | 35.48 | 26.51 | 39.18 | 61.94 | 74.27 | 64.67 | 21.99 |
| Prussian carp | 1087 | 1 | 1 | 3 | 23.11 | 24.58 | 31.05 | 34.75 | 58.74 | 37.84 | 52.45 | 53.17 | 34.92 | 26.97 | 39.44 | 61.03 | 73.30 | 65.79 | 18.90 |
| Prussian carp | 1088 | 1 | 1 | 3 | 22.10 | 22.97 | 27.53 | 30.24 | 47.84 | 31.40 | 42.49 | 42.23 | 29.21 | 22.39 | 31.64 | 49.22 | 61.85 | 55.36 | 15.28 |
| Prussian carp | 1089 | 1 | 1 | 3 | 26.15 | 29.86 | 33.31 | 41.27 | 61.86 | 41.71 | 58.68 | 55.99 | 37.28 | 31.28 | 45.46 | 67.92 | 82.00 | 77.50 | 20.50 |
| Prussian carp | 1090 | 1 | 1 | 3 | 23.88 | 24.75 | 28.69 | 35.04 | 51.27 | 34.17 | 49.30 | 45.45 | 30.69 | 24.51 | 34.68 | 57.07 | 68.99 | 62.38 | 17.88 |
| Prussian carp | 1161 | 1 | 2 | 1 | 41.92 | 42.06 | 48.72 | 49.44 | 81.63 | 54.46 | 76.21 | 72.08 | 49.31 | 41.57 | 56.43 | 85.29 | 106.89 | 94.56 | 29.69 |
| Prussian carp | 1162 | 1 | 2 | 1 | 40.91 | 40.62 | 47.08 | 51.78 | 78.04 | 52.97 | 73.97 | 66.61 | 48.79 | 43.98 | 58.19 | 78.98 | 104.82 | 93.01 | 28.96 |
| Prussian carp | 1163 | 1 | 2 | 1 | 40.55 | 36.31 | 46.06 | 52.03 | 79.62 | 58.39 | 76.00 | 67.30 | 49.96 | 38.82 | 52.05 | 83.54 | 108.01 | 93.99 | 27.55 |
| Prussian carp | 1164 | 1 | 2 | 1 | 28.14 | 29.92 | 33.48 | 33.74 | 55.05 | 36.65 | 51.05 | 48.50 | 28.16 | 32.05 | 41.83 | 56.77 | 74.10 | 66.40 | 17.10 |
| Prussian carp | 1165 | 1 | 2 | 1 | 36.78 | 36.08 | 43.41 | 43.30 | 71.97 | 45.74 | 68.31 | 68.67 | 43.36 | 38.79 | 55.11 | 77.58 | 98.71 | 90.14 | 27.97 |
| Prussian carp | 1166 | 1 | 2 | 1 | 32.76 | 36.55 | 38.81 | 42.40 | 69.38 | 48.53 | 63.37 | 58.27 | 45.72 | 36.50 | 48.59 | 70.11 | 91.07 | 84.53 | 22.68 |
| Prussian carp | 1167 | 1 | 2 | 1 | 47.50 | 46.66 | 52.70 | 53.03 | 91.53 | 66.99 | 81.72 | 76.52 | 53.26 | 45.07 | 65.24 | 84.91 | 104.72 | 92.95 | 30.69 |
| Prussian carp | 1168 | 1 | 2 | 1 | 39.27 | 38.29 | 44.67 | 45.56 | 74.07 | 50.06 | 68.80 | 63.48 | 48.97 | 38.38 | 54.04 | 79.47 | 102.92 | 93.76 | 26.17 |
| Prussian carp | 1169 | 1 | 2 | 1 | 23.02 | 21.00 | 25.89 | 29.13 | 48.94 | 35.18 | 42.69 | 42.44 | 28.76 | 21.84 | 30.10 | 43.69 | 51.90 | 43.06 | 15.03 |
| Prussian carp | 1170 | 1 | 2 | 1 | 37.91 | 33.85 | 42.61 | 49.72 | 81.00 | 59.10 | 73.97 | 71.15 | 44.83 | 39.48 | 57.77 | 81.27 | 99.43 | 88.71 | 28.25 |
| Prussian carp | 1171 | 1 | 2 | 1 | 23.80 | 27.41 | 28.63 | 24.53 | 44.29 | 29.56 | 42.66 | 40.40 | 27.76 | 20.43 | 28.54 | 45.73 | 56.02 | 47.78 | 14.36 |
| Prussian carp | 1172 | 1 | 2 | 1 | 25.61 | 24.47 | 24.76 | 24.83 | 44.33 | 30.43 | 41.43 | 39.75 | 26.83 | 21.75 | 30.34 | 43.40 | 55.80 | 51.35 | 14.33 |
| Prussian carp | 1173 | 1 | 2 | 1 | 26.02 | 22.93 | 26.16 | 26.48 | 45.93 | 33.41 | 41.76 | 39.08 | 24.61 | 28.26 | 37.30 | 42.95 | 58.43 | 51.17 | 16.07 |
| Prussian carp | 1174 | 1 | 2 | 1 | 23.27 | 21.29 | 21.28 | 26.14 | 38.35 | 27.16 | 35.90 | 34.52 | 23.11 | 17.00 | 24.66 | 38.78 | 45.76 | 40.53 | 11.86 |
| Prussian carp | 1175 | 1 | 2 | 1 | 20.01 | 21.21 | 23.88 | 22.66 | 36.08 | 22.16 | 34.65 | 32.99 | 21.53 | 18.36 | 25.03 | 38.12 | 46.32 | 41.37 | 13.28 |
| Prussian carp | 1176 | 1 | 2 | 1 | 23.41 | 24.28 | 27.49 | 28.61 | 45.54 | 29.78 | 42.46 | 40.19 | 25.90 | 23.72 | 33.46 | 44.76 | 54.40 | 49.63 | 12.86 |
| Prussian carp | 1177 | 1 | 2 | 1 | 22.96 | 22.03 | 24.77 | 25.92 | 41.02 | 26.83 | 39.07 | 36.65 | 24.32 | 23.46 | 30.37 | 41.68 | 52.13 | 45.69 | 14.23 |
| Prussian carp | 1178 | 1 | 2 | 1 | 21.46 | 20.96 | 22.92 | 26.32 | 37.58 | 25.17 | 37.95 | 32.93 | 20.86 | 18.10 | 26.53 | 37.85 | 46.73 | 42.83 | 12.30 |
| Prussian carp | 1179 | 1 | 2 | 1 | 20.85 | 21.47 | 21.31 | 24.37 | 34.89 | 22.88 | 33.71 | 29.93 | 19.20 | 19.90 | 24.91 | 33.98 | 44.99 | 39.67 | 11.66 |
| Prussian carp | 1180 | 1 | 2 | 1 | 21.19 | 18.43 | 21.36 | 24.96 | 33.88 | 22.44 | 36.81 | 33.81 | 21.36 | 19.07 | 26.16 | 35.74 | 42.87 | 38.78 | 11.59 |
| Prussian carp | 1181 | 1 | 2 | 1 | 19.81 | 20.24 | 21.11 | 22.19 | 37.00 | 24.90 | 34.40 | 31.39 | 20.95 | 17.80 | 22.76 | 34.72 | 43.97 | 37.98 | 12.26 |
| Prussian carp | 1182 | 1 | 2 | 1 | 25.32 | 26.87 | 28.45 | 30.92 | 48.74 | 34.09 | 43.62 | 37.10 | 26.63 | 26.64 | 33.41 | 46.29 | 61.03 | 54.26 | 15.30 |
| Prussian carp | 1183 | 1 | 2 | 1 | 24.92 | 23.58 | 27.79 | 29.10 | 49.36 | 36.66 | 44.48 | 41.70 | 27.94 | 23.41 | 30.74 | 44.77 | 56.34 | 48.62 | 17.09 |
| Prussian carp | 1184 | 1 | 2 | 1 | 27.49 | 27.02 | 30.14 | 35.92 | 51.46 | 35.61 | 49.61 | 44.05 | 32.89 | 26.06 | 35.53 | 53.70 | 64.43 | 57.28 | 16.52 |
| Prussian carp | 1185 | 1 | 2 | 1 | 22.56 | 21.66 | 24.35 | 31.09 | 42.63 | 29.79 | 41.63 | 35.80 | 21.95 | 21.17 | 28.33 | 42.14 | 51.40 | 44.75 | 13.88 |
| Prussian carp | 1186 | 1 | 2 | 1 | 29.41 | 31.87 | 33.76 | 34.42 | 55.41 | 37.00 | 50.30 | 48.25 | 34.86 | 30.00 | 38.54 | 59.41 | 73.62 | 63.74 | 19.14 |
| Prussian carp | 1187 | 1 | 2 | 1 | 24.69 | 22.64 | 25.37 | 29.37 | 42.15 | 30.46 | 43.40 | 36.80 | 22.43 | 21.78 | 30.98 | 43.16 | 55.48 | 49.74 | 15.68 |
| Prussian carp | 1188 | 1 | 2 | 1 | 21.84 | 22.31 | 26.33 | 29.94 | 47.43 | 33.70 | 41.93 | 39.03 | 25.15 | 19.62 | 29.74 | 43.24 | 50.44 | 45.10 | 14.73 |
| Prussian carp | 1189 | 1 | 2 | 1 | 27.37 | 23.74 | 29.82 | 33.73 | 52.36 | 36.81 | 50.05 | 46.67 | 29.04 | 27.97 | 37.13 | 53.13 | 67.07 | 59.65 | 18.10 |
| Prussian carp | 1190 | 1 | 2 | 1 | 20.92 | 21.58 | 22.93 | 29.95 | 38.94 | 26.13 | 37.47 | 33.80 | 22.86 | 22.11 | 29.29 | 38.56 | 50.68 | 46.12 | 14.87 |
| Prussian carp | 1161 | 1 | 2 | 2 | 43.44 | 45.58 | 47.32 | 49.50 | 79.09 | 51.55 | 76.52 | 72.70 | 48.38 | 41.18 | 58.86 | 85.75 | 105.51 | 95.77 | 28.37 |
| Prussian carp | 1162 | 1 | 2 | 2 | 42.74 | 40.74 | 45.82 | 50.47 | 76.35 | 50.09 | 73.76 | 66.97 | 48.86 | 42.99 | 57.79 | 79.77 | 104.93 | 93.83 | 28.63 |
| Prussian carp | 1163 | 1 | 2 | 2 | 37.45 | 33.92 | 44.22 | 50.73 | 77.16 | 55.69 | 78.10 | 68.80 | 48.74 | 35.82 | 52.69 | 82.56 | 104.02 | 91.34 | 28.33 |
| Prussian carp | 1164 | 1 | 2 | 2 | 27.86 | 29.12 | 31.65 | 34.44 | 56.12 | 36.94 | 50.30 | 48.00 | 28.69 | 30.41 | 39.49 | 54.19 | 70.25 | 65.54 | 17.18 |
| Prussian carp | 1165 | 1 | 2 | 2 | 36.42 | 37.53 | 42.63 | 44.10 | 70.89 | 47.30 | 67.99 | 65.90 | 44.00 | 40.30 | 54.44 | 76.22 | 98.80 | 87.84 | 25.32 |
| Prussian carp | 1166 | 1 | 2 | 2 | 32.89 | 35.27 | 38.04 | 42.51 | 68.88 | 47.47 | 63.38 | 56.76 | 41.51 | 37.08 | 50.79 | 68.65 | 90.98 | 84.61 | 22.00 |
| Prussian carp | 1167 | 1 | 2 | 2 | 47.41 | 46.67 | 52.49 | 51.83 | 88.46 | 63.08 | 80.85 | 76.66 | 53.57 | 44.38 | 63.56 | 84.43 | 104.93 | 90.88 | 29.91 |
| Prussian carp | 1168 | 1 | 2 | 2 | 38.62 | 37.63 | 43.73 | 45.41 | 73.02 | 47.38 | 68.63 | 65.26 | 49.01 | 37.85 | 55.22 | 80.87 | 101.64 | 93.30 | 25.92 |
| Prussian carp | 1169 | 1 | 2 | 2 | 24.25 | 22.74 | 25.92 | 26.32 | 46.60 | 33.76 | 43.49 | 41.51 | 26.33 | 20.82 | 29.00 | 47.06 | 56.18 | 47.98 | 14.85 |
| Prussian carp | 1170 | 1 | 2 | 2 | 39.12 | 35.95 | 43.40 | 49.36 | 78.88 | 54.05 | 70.76 | 68.72 | 47.04 | 37.16 | 55.22 | 82.83 | 101.02 | 89.47 | 27.15 |
| Prussian carp | 1171 | 1 | 2 | 2 | 23.83 | 23.54 | 27.03 | 29.31 | 45.03 | 30.98 | 42.42 | 40.20 | 29.04 | 21.38 | 30.87 | 46.13 | 55.67 | 49.89 | 15.45 |
| Prussian carp | 1172 | 1 | 2 | 2 | 24.86 | 24.41 | 25.62 | 27.45 | 43.46 | 29.00 | 40.28 | 38.58 | 26.77 | 22.46 | 29.41 | 45.19 | 59.34 | 53.45 | 15.37 |
| Prussian carp | 1173 | 1 | 2 | 2 | 22.99 | 23.42 | 25.62 | 26.48 | 42.84 | 28.03 | 40.57 | 39.26 | 26.31 | 26.91 | 35.17 | 43.98 | 60.38 | 51.90 | 16.64 |
| Prussian carp | 1174 | 1 | 2 | 2 | 21.57 | 21.24 | 22.92 | 20.59 | 36.05 | 24.88 | 38.18 | 35.42 | 22.51 | 15.73 | 23.87 | 38.45 | 45.94 | 40.75 | 11.79 |
| Prussian carp | 1175 | 1 | 2 | 2 | 20.62 | 21.43 | 22.55 | 23.18 | 36.23 | 23.94 | 34.13 | 32.29 | 21.45 | 18.51 | 26.87 | 37.29 | 49.01 | 42.13 | 11.76 |
| Prussian carp | 1176 | 1 | 2 | 2 | 22.97 | 22.95 | 26.22 | 26.99 | 42.57 | 31.01 | 42.86 | 41.30 | 22.40 | 25.17 | 33.14 | 43.41 | 53.24 | 48.64 | 12.67 |
| Prussian carp | 1177 | 1 | 2 | 2 | 21.47 | 21.98 | 23.93 | 26.97 | 41.04 | 26.23 | 38.08 | 36.22 | 24.87 | 22.56 | 30.16 | 41.55 | 52.88 | 44.99 | 14.87 |
| Prussian carp | 1178 | 1 | 2 | 2 | 20.63 | 18.68 | 21.76 | 27.59 | 37.50 | 24.91 | 35.95 | 33.01 | 22.34 | 16.81 | 26.40 | 36.92 | 45.57 | 41.93 | 12.10 |
| Prussian carp | 1179 | 1 | 2 | 2 | 20.32 | 21.10 | 21.59 | 24.14 | 33.95 | 22.30 | 33.72 | 29.78 | 20.14 | 20.09 | 24.08 | 35.09 | 45.80 | 40.67 | 11.94 |
| Prussian carp | 1180 | 1 | 2 | 2 | 21.85 | 18.78 | 20.95 | 23.84 | 34.80 | 24.08 | 35.80 | 32.47 | 21.59 | 20.67 | 26.89 | 35.84 | 44.32 | 39.31 | 11.11 |
| Prussian carp | 1181 | 1 | 2 | 2 | 19.37 | 20.64 | 20.90 | 22.37 | 35.12 | 22.83 | 34.31 | 32.40 | 20.42 | 17.92 | 24.46 | 35.25 | 44.45 | 38.72 | 11.96 |
| Prussian carp | 1182 | 1 | 2 | 2 | 25.35 | 25.17 | 27.55 | 30.49 | 46.08 | 33.74 | 46.36 | 38.20 | 26.09 | 23.56 | 33.34 | 45.00 | 57.25 | 50.64 | 15.92 |
| Prussian carp | 1183 | 1 | 2 | 2 | 24.43 | 23.64 | 27.73 | 29.57 | 51.72 | 37.67 | 43.92 | 40.79 | 30.47 | 21.73 | 30.15 | 48.06 | 58.07 | 50.90 | 15.72 |
| Prussian carp | 1184 | 1 | 2 | 2 | 28.26 | 26.09 | 30.64 | 35.04 | 50.71 | 33.87 | 50.16 | 43.95 | 31.93 | 26.42 | 36.05 | 54.23 | 65.24 | 58.30 | 16.75 |
| Prussian carp | 1185 | 1 | 2 | 2 | 23.60 | 21.21 | 24.14 | 29.08 | 41.64 | 28.07 | 40.08 | 35.37 | 25.74 | 17.25 | 25.88 | 44.56 | 51.42 | 45.69 | 13.28 |
| Prussian carp | 1186 | 1 | 2 | 2 | 29.14 | 29.78 | 32.66 | 32.35 | 54.06 | 36.81 | 51.60 | 49.12 | 34.29 | 26.52 | 39.13 | 59.41 | 71.91 | 65.17 | 19.76 |
| Prussian carp | 1187 | 1 | 2 | 2 | 25.03 | 23.75 | 25.75 | 29.70 | 41.58 | 27.73 | 42.92 | 36.39 | 22.21 | 22.13 | 29.84 | 43.49 | 55.31 | 49.19 | 15.68 |
| Prussian carp | 1188 | 1 | 2 | 2 | 22.92 | 20.89 | 25.89 | 27.73 | 46.37 | 33.44 | 43.73 | 40.21 | 26.44 | 18.73 | 27.47 | 43.05 | 49.87 | 44.70 | 14.90 |
| Prussian carp | 1189 | 1 | 2 | 2 | 30.46 | 25.12 | 29.37 | 33.46 | 52.04 | 36.98 | 50.44 | 48.23 | 30.90 | 25.94 | 36.73 | 53.22 | 66.69 | 59.31 | 18.49 |
| Prussian carp | 1190 | 1 | 2 | 2 | 21.93 | 22.59 | 22.72 | 26.00 | 39.39 | 26.31 | 36.54 | 33.82 | 25.39 | 20.37 | 27.74 | 38.37 | 48.98 | 45.62 | 13.88 |
| Prussian carp | 1161 | 1 | 2 | 3 | 42.44 | 39.64 | 45.91 | 47.70 | 80.17 | 52.22 | 73.76 | 72.35 | 52.76 | 39.58 | 56.99 | 87.26 | 107.94 | 97.52 | 28.39 |
| Prussian carp | 1162 | 1 | 2 | 3 | 41.06 | 39.30 | 45.53 | 50.00 | 76.68 | 51.05 | 71.89 | 66.91 | 52.79 | 40.95 | 55.22 | 81.44 | 104.78 | 95.56 | 28.66 |
| Prussian carp | 1163 | 1 | 2 | 3 | 41.21 | 35.93 | 45.95 | 51.07 | 76.74 | 55.31 | 78.32 | 68.36 | 48.13 | 36.77 | 54.37 | 83.13 | 106.27 | 96.20 | 29.58 |
| Prussian carp | 1164 | 1 | 2 | 3 | 27.66 | 31.35 | 33.16 | 31.85 | 55.29 | 36.05 | 50.51 | 47.37 | 28.35 | 30.64 | 40.72 | 53.83 | 72.08 | 65.25 | 17.48 |
| Prussian carp | 1165 | 1 | 2 | 3 | 37.27 | 38.71 | 43.43 | 42.12 | 70.90 | 44.61 | 68.04 | 67.56 | 43.81 | 42.33 | 54.27 | 76.18 | 97.46 | 87.26 | 25.41 |
| Prussian carp | 1166 | 1 | 2 | 3 | 33.58 | 35.54 | 39.92 | 41.21 | 68.85 | 50.52 | 62.15 | 56.06 | 45.44 | 37.68 | 50.16 | 68.78 | 90.04 | 82.91 | 22.02 |
| Prussian carp | 1167 | 1 | 2 | 3 | 46.40 | 45.41 | 51.01 | 53.22 | 91.68 | 64.41 | 79.90 | 76.00 | 52.94 | 43.54 | 62.18 | 83.52 | 102.20 | 89.55 | 32.29 |
| Prussian carp | 1168 | 1 | 2 | 3 | 38.62 | 36.30 | 42.65 | 45.17 | 72.42 | 47.95 | 67.77 | 64.89 | 49.04 | 39.27 | 55.41 | 77.84 | 98.07 | 89.41 | 26.87 |
| Prussian carp | 1169 | 1 | 2 | 3 | 26.01 | 22.91 | 25.64 | 30.21 | 47.01 | 32.33 | 44.33 | 41.30 | 25.76 | 20.99 | 29.46 | 47.76 | 58.35 | 50.14 | 14.80 |
| Prussian carp | 1170 | 1 | 2 | 3 | 38.94 | 33.78 | 42.35 | 48.83 | 79.77 | 55.38 | 73.15 | 70.88 | 49.34 | 36.50 | 53.42 | 82.48 | 101.44 | 89.96 | 28.69 |
| Prussian carp | 1171 | 1 | 2 | 3 | 24.83 | 23.82 | 27.19 | 29.29 | 47.02 | 31.85 | 40.51 | 37.71 | 28.90 | 22.38 | 30.03 | 45.11 | 55.28 | 48.83 | 15.15 |
| Prussian carp | 1172 | 1 | 2 | 3 | 24.19 | 22.75 | 24.86 | 26.81 | 43.48 | 30.73 | 42.05 | 39.28 | 26.26 | 22.05 | 28.57 | 44.11 | 56.55 | 51.50 | 14.88 |
| Prussian carp | 1173 | 1 | 2 | 3 | 24.43 | 23.93 | 26.53 | 29.32 | 43.96 | 32.83 | 44.16 | 39.83 | 21.66 | 27.12 | 34.82 | 43.31 | 58.10 | 52.05 | 16.28 |
| Prussian carp | 1174 | 1 | 2 | 3 | 21.46 | 22.66 | 22.91 | 21.57 | 36.77 | 25.84 | 37.96 | 34.98 | 18.93 | 20.11 | 25.47 | 37.32 | 47.02 | 40.97 | 12.39 |
| Prussian carp | 1175 | 1 | 2 | 3 | 18.93 | 18.67 | 20.45 | 24.28 | 35.19 | 21.14 | 35.18 | 32.86 | 21.46 | 18.74 | 24.84 | 37.70 | 46.06 | 40.94 | 12.66 |
| Prussian carp | 1176 | 1 | 2 | 3 | 23.51 | 25.97 | 26.96 | 29.42 | 45.63 | 30.30 | 42.05 | 40.80 | 29.63 | 22.71 | 31.55 | 44.61 | 53.07 | 47.52 | 13.68 |
| Prussian carp | 1177 | 1 | 2 | 3 | 22.15 | 21.52 | 24.34 | 26.68 | 41.47 | 27.43 | 39.45 | 36.27 | 24.32 | 23.26 | 28.84 | 41.52 | 50.82 | 43.34 | 14.12 |
| Prussian carp | 1178 | 1 | 2 | 3 | 21.74 | 19.98 | 22.66 | 25.51 | 37.39 | 26.45 | 37.76 | 32.96 | 20.99 | 19.30 | 27.33 | 37.12 | 46.71 | 42.39 | 11.91 |
| Prussian carp | 1179 | 1 | 2 | 3 | 20.70 | 19.78 | 20.67 | 22.97 | 35.99 | 23.16 | 33.60 | 30.24 | 19.86 | 19.09 | 24.49 | 34.96 | 46.50 | 40.90 | 11.94 |
| Prussian carp | 1180 | 1 | 2 | 3 | 20.86 | 17.95 | 20.80 | 24.68 | 37.38 | 26.79 | 36.02 | 33.46 | 21.64 | 19.29 | 27.04 | 35.88 | 44.10 | 40.26 | 11.59 |
| Prussian carp | 1181 | 1 | 2 | 3 | 19.75 | 21.30 | 20.97 | 22.94 | 36.45 | 24.51 | 33.81 | 31.90 | 21.84 | 16.68 | 23.83 | 35.42 | 43.39 | 37.68 | 12.10 |
| Prussian carp | 1182 | 1 | 2 | 3 | 25.49 | 25.82 | 28.26 | 30.82 | 46.57 | 31.74 | 44.45 | 37.87 | 28.08 | 22.03 | 32.07 | 45.57 | 57.02 | 51.94 | 15.61 |
| Prussian carp | 1183 | 1 | 2 | 3 | 25.16 | 24.24 | 28.06 | 29.97 | 49.40 | 35.98 | 43.49 | 40.77 | 29.14 | 22.60 | 31.55 | 46.79 | 58.82 | 51.05 | 16.69 |
| Prussian carp | 1184 | 1 | 2 | 3 | 27.58 | 26.45 | 29.84 | 35.91 | 52.23 | 35.37 | 50.21 | 44.40 | 31.12 | 26.09 | 35.70 | 53.12 | 65.58 | 57.57 | 17.69 |
| Prussian carp | 1185 | 1 | 2 | 3 | 22.56 | 21.63 | 24.72 | 30.38 | 41.42 | 28.29 | 41.35 | 36.36 | 24.75 | 16.51 | 25.95 | 43.80 | 50.62 | 45.09 | 14.02 |
| Prussian carp | 1186 | 1 | 2 | 3 | 26.98 | 29.75 | 31.88 | 33.71 | 54.85 | 37.36 | 51.20 | 47.82 | 33.57 | 27.47 | 39.14 | 58.85 | 72.37 | 63.56 | 19.52 |
| Prussian carp | 1187 | 1 | 2 | 3 | 24.08 | 23.08 | 25.79 | 30.41 | 43.15 | 29.32 | 43.48 | 37.08 | 21.01 | 21.31 | 29.85 | 42.80 | 55.50 | 50.18 | 15.38 |
| Prussian carp | 1188 | 1 | 2 | 3 | 24.33 | 22.59 | 26.94 | 27.43 | 47.37 | 33.98 | 41.03 | 39.04 | 26.48 | 19.41 | 28.40 | 43.28 | 50.01 | 42.91 | 15.36 |
| Prussian carp | 1189 | 1 | 2 | 3 | 28.28 | 25.16 | 30.45 | 32.74 | 53.69 | 34.74 | 49.28 | 48.09 | 28.15 | 28.31 | 38.79 | 52.98 | 67.12 | 58.83 | 17.97 |
| Prussian carp | 1190 | 1 | 2 | 3 | 20.42 | 22.67 | 22.93 | 25.13 | 39.25 | 27.50 | 37.10 | 34.84 | 25.88 | 20.59 | 28.46 | 38.82 | 49.33 | 46.38 | 14.35 |
| Prussian carp | 1251 | 1 | 3 | 1 | 33.28 | 29.36 | 38.51 | 36.40 | 68.32 | 43.04 | 60.10 | 63.18 | 39.83 | 36.51 | 49.66 | 71.86 | 86.55 | 76.24 | 22.34 |
| Prussian carp | 1252 | 1 | 3 | 1 | 35.95 | 38.09 | 44.31 | 46.83 | 76.14 | 41.25 | 70.94 | 73.99 | 45.87 | 37.89 | 55.74 | 85.73 | 104.22 | 93.24 | 27.73 |
| Prussian carp | 1253 | 1 | 3 | 1 | 33.81 | 34.44 | 42.22 | 42.91 | 76.74 | 51.33 | 70.66 | 72.35 | 46.70 | 36.73 | 53.49 | 81.88 | 94.80 | 83.79 | 27.19 |
| Prussian carp | 1254 | 1 | 3 | 1 | 30.41 | 41.13 | 39.19 | 39.90 | 66.14 | 39.58 | 60.10 | 57.70 | 39.74 | 29.63 | 43.07 | 67.15 | 89.17 | 82.08 | 21.75 |
| Prussian carp | 1255 | 1 | 3 | 1 | 28.84 | 28.76 | 33.48 | 34.59 | 61.27 | 38.68 | 52.49 | 55.79 | 41.92 | 32.41 | 43.31 | 63.13 | 75.50 | 64.50 | 19.23 |
| Prussian carp | 1256 | 1 | 3 | 1 | 34.95 | 36.37 | 39.49 | 35.78 | 64.93 | 43.08 | 58.78 | 56.69 | 42.17 | 30.73 | 46.04 | 66.04 | 81.62 | 73.89 | 22.91 |
| Prussian carp | 1257 | 1 | 3 | 1 | 28.95 | 27.49 | 34.70 | 39.54 | 69.50 | 49.79 | 57.71 | 57.10 | 38.72 | 34.95 | 45.62 | 65.64 | 81.86 | 73.62 | 19.98 |
| Prussian carp | 1258 | 1 | 3 | 1 | 28.77 | 29.26 | 32.55 | 34.64 | 60.25 | 42.43 | 52.44 | 53.10 | 41.27 | 29.09 | 42.34 | 59.97 | 69.17 | 58.20 | 17.14 |
| Prussian carp | 1259 | 1 | 3 | 1 | 26.10 | 28.79 | 33.26 | 35.42 | 59.44 | 42.33 | 55.47 | 52.47 | 37.61 | 26.78 | 39.43 | 60.82 | 76.26 | 69.81 | 20.80 |
| Prussian carp | 1260 | 1 | 3 | 1 | 29.68 | 31.59 | 34.96 | 36.72 | 60.77 | 37.00 | 52.41 | 54.29 | 38.31 | 35.82 | 44.58 | 65.30 | 84.72 | 76.85 | 21.10 |
| Prussian carp | 1261 | 1 | 3 | 1 | 30.10 | 30.27 | 38.88 | 42.28 | 73.95 | 53.30 | 64.36 | 64.30 | 47.44 | 34.93 | 49.56 | 64.93 | 80.56 | 73.59 | 24.83 |
| Prussian carp | 1262 | 1 | 3 | 1 | 29.42 | 28.67 | 33.48 | 35.64 | 56.50 | 35.77 | 51.60 | 50.05 | 37.21 | 27.37 | 40.21 | 56.02 | 67.63 | 59.53 | 19.31 |
| Prussian carp | 1263 | 1 | 3 | 1 | 31.68 | 27.67 | 35.27 | 39.20 | 63.56 | 42.70 | 57.44 | 57.31 | 40.15 | 25.80 | 41.76 | 64.08 | 77.33 | 67.79 | 21.12 |
| Prussian carp | 1264 | 1 | 3 | 1 | 38.52 | 34.89 | 44.64 | 44.46 | 77.50 | 49.61 | 70.98 | 74.88 | 48.17 | 37.17 | 58.66 | 85.17 | 106.48 | 94.67 | 31.36 |
| Prussian carp | 1265 | 1 | 3 | 1 | 29.26 | 27.13 | 34.28 | 36.59 | 63.70 | 42.87 | 55.00 | 56.24 | 42.47 | 31.47 | 43.38 | 68.28 | 84.60 | 75.22 | 22.70 |
| Prussian carp | 1266 | 1 | 3 | 1 | 29.39 | 27.19 | 36.41 | 32.26 | 64.30 | 44.82 | 55.17 | 56.86 | 38.72 | 27.36 | 41.98 | 63.88 | 79.86 | 73.03 | 21.50 |
| Prussian carp | 1267 | 1 | 3 | 1 | 29.80 | 29.63 | 35.78 | 34.49 | 61.13 | 39.77 | 54.78 | 56.06 | 39.05 | 29.83 | 41.14 | 67.78 | 82.20 | 75.49 | 20.04 |
| Prussian carp | 1268 | 1 | 3 | 1 | 33.15 | 29.01 | 38.92 | 35.04 | 66.06 | 42.06 | 56.69 | 63.30 | 42.09 | 31.23 | 47.50 | 69.39 | 86.11 | 73.98 | 24.72 |
| Prussian carp | 1269 | 1 | 3 | 1 | 27.92 | 28.58 | 32.26 | 36.02 | 54.61 | 36.06 | 52.65 | 46.51 | 28.39 | 24.31 | 37.51 | 55.82 | 68.38 | 62.43 | 18.99 |
| Prussian carp | 1270 | 1 | 3 | 1 | 29.54 | 30.31 | 37.02 | 37.35 | 69.98 | 50.82 | 59.04 | 63.90 | 41.92 | 27.55 | 43.10 | 69.80 | 83.81 | 78.95 | 21.45 |
| Prussian carp | 1271 | 1 | 3 | 1 | 35.21 | 35.56 | 37.39 | 36.15 | 60.88 | 40.13 | 54.42 | 52.77 | 40.64 | 32.62 | 42.89 | 62.48 | 76.16 | 64.84 | 21.54 |
| Prussian carp | 1272 | 1 | 3 | 1 | 27.12 | 25.36 | 31.35 | 34.70 | 60.16 | 43.15 | 53.25 | 56.66 | 40.67 | 24.27 | 39.59 | 60.08 | 68.72 | 60.89 | 20.53 |
| Prussian carp | 1273 | 1 | 3 | 1 | 30.04 | 27.43 | 35.32 | 37.56 | 64.63 | 47.23 | 58.00 | 59.29 | 39.73 | 30.66 | 42.87 | 65.95 | 77.46 | 63.78 | 20.65 |
| Prussian carp | 1274 | 1 | 3 | 1 | 30.53 | 30.13 | 35.73 | 37.59 | 63.86 | 44.82 | 58.28 | 55.46 | 38.22 | 27.95 | 42.16 | 62.78 | 74.85 | 65.95 | 22.55 |
| Prussian carp | 1275 | 1 | 3 | 1 | 33.26 | 31.82 | 37.95 | 36.41 | 69.83 | 53.04 | 63.23 | 66.47 | 43.81 | 36.40 | 50.63 | 72.81 | 87.71 | 81.18 | 22.11 |
| Prussian carp | 4030 | 1 | 3 | 1 | 36.46 | 35.83 | 42.37 | 42.35 | 71.44 | 45.74 | 64.79 | 65.90 | 44.18 | 35.55 | 48.47 | 74.10 | 93.96 | 84.56 | 24.54 |
| Prussian carp | 4031 | 1 | 3 | 1 | 30.82 | 28.99 | 34.99 | 32.79 | 61.31 | 40.89 | 53.17 | 54.65 | 40.24 | 24.00 | 40.61 | 64.40 | 76.00 | 72.01 | 20.59 |
| Prussian carp | 4032 | 1 | 3 | 1 | 28.14 | 28.87 | 34.59 | 44.14 | 73.15 | 52.05 | 61.06 | 62.49 | 42.61 | 33.98 | 48.87 | 74.18 | 84.99 | 72.57 | 23.31 |
| Prussian carp | 4033 | 1 | 3 | 1 | 26.31 | 27.85 | 34.53 | 38.68 | 67.89 | 44.67 | 57.70 | 62.15 | 41.67 | 28.82 | 44.88 | 73.06 | 81.68 | 70.77 | 23.31 |
| Prussian carp | 4034 | 1 | 3 | 1 | 33.68 | 30.87 | 40.78 | 41.81 | 77.68 | 56.99 | 67.07 | 70.79 | 52.37 | 31.11 | 51.27 | 79.87 | 87.81 | 77.88 | 25.32 |
| Prussian carp | 1251 | 1 | 3 | 2 | 33.96 | 29.54 | 37.38 | 38.89 | 70.95 | 48.77 | 61.33 | 64.20 | 39.26 | 33.33 | 49.17 | 71.13 | 84.74 | 74.67 | 22.31 |
| Prussian carp | 1252 | 1 | 3 | 2 | 38.30 | 34.44 | 43.69 | 46.03 | 79.48 | 50.32 | 71.74 | 75.56 | 49.65 | 34.64 | 55.71 | 87.40 | 102.92 | 92.28 | 30.02 |
| Prussian carp | 1253 | 1 | 3 | 2 | 34.43 | 32.91 | 41.80 | 48.01 | 77.24 | 49.26 | 69.23 | 73.81 | 48.53 | 37.81 | 53.82 | 81.48 | 95.69 | 83.49 | 26.35 |
| Prussian carp | 1254 | 1 | 3 | 2 | 31.75 | 36.86 | 39.75 | 40.50 | 65.54 | 44.38 | 63.15 | 58.10 | 39.77 | 32.16 | 43.13 | 68.70 | 90.82 | 82.83 | 22.56 |
| Prussian carp | 1255 | 1 | 3 | 2 | 28.20 | 27.94 | 34.03 | 34.65 | 61.75 | 41.27 | 54.32 | 56.66 | 41.06 | 31.01 | 43.02 | 64.50 | 75.31 | 64.63 | 20.62 |
| Prussian carp | 1256 | 1 | 3 | 2 | 33.84 | 32.55 | 37.65 | 39.16 | 68.17 | 46.66 | 59.12 | 58.70 | 40.87 | 30.45 | 46.39 | 65.47 | 79.23 | 70.92 | 23.73 |
| Prussian carp | 1257 | 1 | 3 | 2 | 29.22 | 29.99 | 36.33 | 41.03 | 70.45 | 51.27 | 56.71 | 58.50 | 42.75 | 32.44 | 47.71 | 68.91 | 82.63 | 76.66 | 21.55 |
| Prussian carp | 1258 | 1 | 3 | 2 | 27.36 | 27.21 | 31.38 | 34.52 | 59.44 | 41.30 | 52.60 | 53.76 | 41.66 | 28.76 | 40.60 | 60.53 | 70.55 | 61.40 | 17.79 |
| Prussian carp | 1259 | 1 | 3 | 2 | 26.48 | 26.14 | 33.62 | 37.19 | 60.04 | 42.95 | 54.30 | 53.05 | 40.01 | 27.70 | 40.08 | 63.13 | 78.32 | 71.15 | 20.31 |
| Prussian carp | 1260 | 1 | 3 | 2 | 28.90 | 31.51 | 36.97 | 36.04 | 60.02 | 35.68 | 56.32 | 55.10 | 39.88 | 30.31 | 42.81 | 67.59 | 85.06 | 75.94 | 22.51 |
| Prussian carp | 1261 | 1 | 3 | 2 | 31.32 | 30.99 | 39.63 | 42.94 | 76.29 | 57.61 | 65.04 | 63.81 | 48.12 | 32.48 | 50.53 | 68.98 | 83.92 | 75.98 | 26.37 |
| Prussian carp | 1262 | 1 | 3 | 2 | 28.94 | 27.14 | 32.96 | 34.17 | 56.20 | 36.43 | 53.18 | 50.64 | 36.51 | 29.30 | 39.43 | 58.62 | 70.50 | 62.90 | 20.54 |
| Prussian carp | 1263 | 1 | 3 | 2 | 30.57 | 27.98 | 34.76 | 39.30 | 63.60 | 44.35 | 58.69 | 55.92 | 38.51 | 28.41 | 40.89 | 63.70 | 75.12 | 66.80 | 21.90 |
| Prussian carp | 1264 | 1 | 3 | 2 | 39.27 | 33.33 | 44.36 | 43.77 | 78.85 | 51.04 | 71.05 | 73.44 | 47.06 | 38.06 | 57.34 | 83.87 | 102.64 | 93.33 | 30.75 |
| Prussian carp | 1265 | 1 | 3 | 2 | 27.93 | 26.74 | 33.38 | 39.35 | 63.20 | 41.07 | 56.00 | 56.95 | 41.92 | 30.06 | 42.30 | 69.41 | 86.26 | 77.24 | 22.88 |
| Prussian carp | 1266 | 1 | 3 | 2 | 27.51 | 27.85 | 35.41 | 33.56 | 63.64 | 45.97 | 54.05 | 57.47 | 39.72 | 28.34 | 42.00 | 65.01 | 80.43 | 71.02 | 21.92 |
| Prussian carp | 1267 | 1 | 3 | 2 | 29.20 | 30.98 | 35.92 | 34.61 | 61.48 | 43.59 | 54.20 | 56.08 | 39.59 | 28.02 | 42.20 | 67.47 | 81.71 | 74.67 | 21.88 |
| Prussian carp | 1268 | 1 | 3 | 2 | 30.60 | 28.13 | 38.11 | 34.07 | 67.44 | 44.84 | 57.73 | 62.69 | 39.51 | 33.22 | 49.68 | 69.08 | 82.85 | 73.60 | 25.93 |
| Prussian carp | 1269 | 1 | 3 | 2 | 26.33 | 27.59 | 31.47 | 33.62 | 54.98 | 37.51 | 52.01 | 47.03 | 32.01 | 22.94 | 34.29 | 56.06 | 68.10 | 61.38 | 19.73 |
| Prussian carp | 1270 | 1 | 3 | 2 | 29.93 | 31.19 | 36.74 | 36.67 | 67.69 | 47.01 | 59.12 | 63.31 | 41.66 | 26.77 | 43.76 | 70.29 | 85.21 | 80.45 | 23.60 |
| Prussian carp | 1271 | 1 | 3 | 2 | 36.56 | 34.72 | 36.17 | 37.00 | 61.65 | 40.80 | 54.22 | 53.88 | 42.61 | 30.92 | 42.73 | 64.11 | 77.46 | 64.47 | 21.49 |
| Prussian carp | 1272 | 1 | 3 | 2 | 27.43 | 25.25 | 30.94 | 34.39 | 58.67 | 42.32 | 53.98 | 56.15 | 38.65 | 26.06 | 39.06 | 59.24 | 68.50 | 59.67 | 20.12 |
| Prussian carp | 1273 | 1 | 3 | 2 | 29.83 | 26.68 | 33.20 | 39.88 | 65.05 | 49.18 | 57.00 | 58.53 | 43.09 | 29.09 | 41.82 | 67.27 | 75.89 | 63.57 | 20.43 |
| Prussian carp | 1274 | 1 | 3 | 2 | 30.26 | 30.28 | 36.49 | 38.35 | 63.70 | 44.57 | 57.16 | 54.25 | 38.23 | 29.03 | 41.39 | 63.99 | 77.89 | 67.99 | 23.20 |
| Prussian carp | 1275 | 1 | 3 | 2 | 31.68 | 32.40 | 38.63 | 35.99 | 69.82 | 52.94 | 64.68 | 67.35 | 43.92 | 35.42 | 50.00 | 72.60 | 86.65 | 79.89 | 23.13 |
| Prussian carp | 4030 | 1 | 3 | 2 | 35.41 | 36.54 | 42.23 | 41.93 | 72.76 | 48.08 | 64.51 | 65.48 | 44.53 | 32.85 | 49.06 | 74.02 | 92.29 | 84.94 | 25.52 |
| Prussian carp | 4031 | 1 | 3 | 2 | 32.26 | 29.14 | 34.44 | 32.37 | 61.11 | 40.53 | 51.58 | 54.36 | 41.61 | 24.99 | 39.27 | 63.11 | 74.65 | 69.09 | 20.93 |
| Prussian carp | 4032 | 1 | 3 | 2 | 24.74 | 28.85 | 34.02 | 43.54 | 73.00 | 52.66 | 62.09 | 62.69 | 44.44 | 30.93 | 46.76 | 75.59 | 84.83 | 73.02 | 24.63 |
| Prussian carp | 4033 | 1 | 3 | 2 | 26.82 | 27.03 | 34.44 | 37.23 | 67.01 | 45.71 | 57.26 | 63.50 | 41.15 | 28.84 | 46.49 | 71.53 | 80.68 | 72.59 | 23.11 |
| Prussian carp | 4034 | 1 | 3 | 2 | 33.08 | 30.91 | 40.95 | 43.04 | 78.97 | 56.88 | 66.78 | 69.22 | 51.15 | 30.76 | 50.90 | 78.27 | 87.01 | 76.04 | 26.93 |
| Prussian carp | 1251 | 1 | 3 | 3 | 33.70 | 30.09 | 38.00 | 38.36 | 69.69 | 48.81 | 60.41 | 63.54 | 41.01 | 35.37 | 48.63 | 71.35 | 83.43 | 74.18 | 22.93 |
| Prussian carp | 1252 | 1 | 3 | 3 | 38.29 | 32.11 | 41.88 | 48.43 | 79.76 | 52.84 | 72.76 | 75.29 | 46.61 | 36.60 | 57.53 | 85.98 | 100.31 | 91.11 | 30.04 |
| Prussian carp | 1253 | 1 | 3 | 3 | 34.88 | 32.64 | 42.60 | 46.25 | 77.60 | 50.26 | 70.29 | 74.20 | 49.45 | 35.09 | 52.66 | 82.92 | 96.47 | 85.34 | 26.77 |
| Prussian carp | 1254 | 1 | 3 | 3 | 31.87 | 37.47 | 40.70 | 38.35 | 65.33 | 43.42 | 62.74 | 59.02 | 40.64 | 31.88 | 44.48 | 69.56 | 89.67 | 83.93 | 21.24 |
| Prussian carp | 1255 | 1 | 3 | 3 | 29.20 | 27.68 | 34.22 | 32.83 | 61.02 | 40.74 | 53.34 | 55.86 | 40.90 | 30.19 | 41.53 | 63.75 | 74.77 | 65.54 | 19.38 |
| Prussian carp | 1256 | 1 | 3 | 3 | 34.61 | 33.56 | 38.87 | 37.77 | 67.76 | 47.02 | 59.40 | 57.91 | 40.61 | 30.16 | 46.47 | 66.06 | 80.30 | 71.65 | 23.13 |
| Prussian carp | 1257 | 1 | 3 | 3 | 30.04 | 28.03 | 35.47 | 38.58 | 69.35 | 50.96 | 59.04 | 57.90 | 39.59 | 33.72 | 47.81 | 67.88 | 81.83 | 76.22 | 22.06 |
| Prussian carp | 1258 | 1 | 3 | 3 | 27.25 | 27.98 | 32.16 | 34.40 | 60.92 | 42.87 | 51.20 | 52.53 | 42.65 | 26.33 | 39.79 | 60.11 | 71.45 | 60.92 | 18.02 |
| Prussian carp | 1259 | 1 | 3 | 3 | 27.40 | 26.29 | 32.15 | 36.77 | 59.16 | 43.04 | 54.99 | 51.47 | 38.51 | 27.13 | 39.68 | 62.69 | 79.28 | 72.71 | 20.58 |
| Prussian carp | 1260 | 1 | 3 | 3 | 29.46 | 30.91 | 35.97 | 36.77 | 59.21 | 37.25 | 55.73 | 54.54 | 40.86 | 28.74 | 43.65 | 69.55 | 86.44 | 76.92 | 22.91 |
| Prussian carp | 1261 | 1 | 3 | 3 | 32.66 | 30.15 | 40.38 | 42.69 | 74.91 | 54.82 | 65.56 | 62.93 | 48.08 | 31.58 | 49.83 | 70.07 | 84.59 | 78.66 | 26.25 |
| Prussian carp | 1262 | 1 | 3 | 3 | 30.16 | 26.22 | 32.38 | 35.31 | 56.71 | 38.83 | 51.98 | 50.44 | 37.79 | 27.87 | 39.51 | 56.64 | 68.52 | 60.61 | 20.10 |
| Prussian carp | 1263 | 1 | 3 | 3 | 31.19 | 28.62 | 35.98 | 38.66 | 64.47 | 44.92 | 57.45 | 56.51 | 40.15 | 26.08 | 39.83 | 63.46 | 74.68 | 67.42 | 21.11 |
| Prussian carp | 1264 | 1 | 3 | 3 | 37.84 | 35.58 | 45.34 | 44.90 | 80.21 | 55.71 | 70.62 | 74.14 | 48.57 | 39.22 | 58.55 | 82.79 | 102.17 | 92.12 | 30.53 |
| Prussian carp | 1265 | 1 | 3 | 3 | 28.90 | 24.01 | 34.24 | 38.11 | 62.49 | 40.98 | 53.99 | 55.66 | 43.74 | 29.55 | 43.39 | 68.17 | 86.81 | 77.49 | 21.81 |
| Prussian carp | 1266 | 1 | 3 | 3 | 27.09 | 28.65 | 35.20 | 32.75 | 63.44 | 44.83 | 53.10 | 56.53 | 39.84 | 29.30 | 42.22 | 64.27 | 79.33 | 70.53 | 20.90 |
| Prussian carp | 1267 | 1 | 3 | 3 | 29.66 | 29.55 | 34.52 | 33.64 | 61.62 | 41.97 | 53.86 | 55.59 | 36.09 | 30.70 | 43.49 | 66.12 | 82.12 | 75.60 | 22.18 |
| Prussian carp | 1268 | 1 | 3 | 3 | 31.27 | 28.88 | 38.37 | 35.29 | 67.27 | 42.96 | 58.43 | 61.32 | 37.83 | 32.40 | 48.96 | 68.30 | 81.47 | 72.03 | 24.93 |
| Prussian carp | 1269 | 1 | 3 | 3 | 27.43 | 28.51 | 33.62 | 35.31 | 56.52 | 39.03 | 52.61 | 45.63 | 30.79 | 24.13 | 36.23 | 55.14 | 67.99 | 62.27 | 17.74 |
| Prussian carp | 1270 | 1 | 3 | 3 | 31.30 | 31.29 | 36.27 | 35.29 | 69.41 | 50.20 | 59.09 | 61.71 | 41.23 | 28.04 | 43.84 | 68.36 | 79.70 | 78.17 | 20.66 |
| Prussian carp | 1271 | 1 | 3 | 3 | 34.91 | 34.56 | 36.99 | 36.46 | 62.81 | 41.88 | 55.93 | 54.30 | 41.15 | 31.03 | 42.86 | 63.86 | 77.02 | 64.75 | 21.84 |
| Prussian carp | 1272 | 1 | 3 | 3 | 26.40 | 26.12 | 30.95 | 33.24 | 59.73 | 43.34 | 54.01 | 55.92 | 38.03 | 27.60 | 39.91 | 56.69 | 65.61 | 58.59 | 19.34 |
| Prussian carp | 1273 | 1 | 3 | 3 | 29.15 | 27.81 | 33.67 | 36.22 | 64.26 | 47.50 | 57.16 | 57.50 | 41.23 | 30.27 | 40.95 | 67.19 | 75.02 | 62.49 | 21.79 |
| Prussian carp | 1274 | 1 | 3 | 3 | 28.82 | 32.01 | 36.18 | 38.63 | 64.02 | 44.24 | 56.13 | 53.69 | 39.90 | 27.55 | 42.46 | 61.81 | 75.36 | 64.77 | 22.92 |
| Prussian carp | 1275 | 1 | 3 | 3 | 31.48 | 33.33 | 38.10 | 37.31 | 69.93 | 53.01 | 62.67 | 66.14 | 45.23 | 34.47 | 48.36 | 72.60 | 87.89 | 79.01 | 22.71 |
| Prussian carp | 4030 | 1 | 3 | 3 | 35.94 | 36.68 | 41.50 | 42.49 | 74.01 | 51.45 | 64.85 | 64.28 | 44.49 | 33.73 | 46.75 | 73.17 | 94.62 | 83.43 | 24.18 |
| Prussian carp | 4031 | 1 | 3 | 3 | 30.83 | 30.15 | 34.70 | 32.56 | 60.96 | 40.35 | 52.15 | 53.86 | 40.94 | 24.55 | 38.60 | 62.45 | 72.33 | 68.74 | 20.06 |
| Prussian carp | 4032 | 1 | 3 | 3 | 27.29 | 27.29 | 35.35 | 43.79 | 72.59 | 52.15 | 62.04 | 62.90 | 42.44 | 34.24 | 48.35 | 73.34 | 82.82 | 71.56 | 23.93 |
| Prussian carp | 4033 | 1 | 3 | 3 | 26.22 | 27.43 | 33.93 | 39.15 | 67.10 | 46.94 | 57.55 | 62.49 | 42.45 | 30.29 | 48.15 | 73.62 | 82.98 | 74.23 | 22.53 |
| Prussian carp | 4034 | 1 | 3 | 3 | 31.30 | 33.86 | 42.21 | 43.39 | 79.27 | 55.41 | 66.57 | 69.75 | 52.79 | 30.70 | 51.29 | 79.01 | 85.44 | 76.33 | 25.75 |
| Prussian carp | 1061 | 2 | 1 | 1 | 25.16 | 25.78 | 30.55 | 33.09 | 54.63 | 34.82 | 48.90 | 47.37 | 33.48 | 31.67 | 39.76 | 56.58 | 70.89 | 61.51 | 19.39 |
| Prussian carp | 1062 | 2 | 1 | 1 | 27.73 | 31.15 | 36.60 | 36.01 | 61.37 | 36.43 | 53.95 | 53.00 | 38.93 | 33.63 | 46.65 | 65.36 | 83.43 | 73.50 | 22.87 |
| Prussian carp | 1063 | 2 | 1 | 1 | 28.53 | 27.84 | 34.22 | 36.44 | 58.67 | 39.60 | 54.74 | 52.61 | 34.50 | 32.45 | 44.00 | 58.65 | 73.70 | 67.40 | 20.17 |
| Prussian carp | 1064 | 2 | 1 | 1 | 26.13 | 28.15 | 32.65 | 35.98 | 57.44 | 36.95 | 51.52 | 51.46 | 34.79 | 28.12 | 41.21 | 63.59 | 78.20 | 73.35 | 21.58 |
| Prussian carp | 1065 | 2 | 1 | 1 | 22.31 | 27.39 | 30.32 | 35.81 | 53.92 | 34.36 | 51.09 | 50.47 | 33.52 | 26.63 | 38.26 | 58.72 | 67.62 | 60.83 | 19.54 |
| Prussian carp | 1066 | 2 | 1 | 1 | 26.27 | 28.66 | 33.22 | 35.04 | 54.88 | 36.35 | 52.96 | 49.66 | 34.15 | 30.83 | 42.76 | 59.74 | 74.78 | 64.87 | 21.44 |
| Prussian carp | 1067 | 2 | 1 | 1 | 28.50 | 30.21 | 34.22 | 41.56 | 63.55 | 41.68 | 61.61 | 61.77 | 34.95 | 32.94 | 44.55 | 64.88 | 79.28 | 69.09 | 19.69 |
| Prussian carp | 1068 | 2 | 1 | 1 | 28.39 | 32.54 | 36.60 | 37.90 | 61.70 | 38.84 | 54.33 | 53.71 | 42.43 | 30.93 | 41.69 | 67.19 | 75.39 | 71.18 | 19.82 |
| Prussian carp | 1069 | 2 | 1 | 1 | 29.21 | 26.21 | 32.40 | 31.50 | 52.35 | 32.94 | 48.65 | 46.63 | 33.52 | 26.44 | 34.74 | 54.74 | 66.38 | 57.25 | 19.44 |
| Prussian carp | 1070 | 2 | 1 | 1 | 23.48 | 28.85 | 31.85 | 34.13 | 57.37 | 36.63 | 47.17 | 50.18 | 34.50 | 29.96 | 43.53 | 55.15 | 63.21 | 55.93 | 18.84 |
| Prussian carp | 1071 | 2 | 1 | 1 | 24.72 | 25.57 | 33.18 | 39.91 | 64.51 | 46.63 | 59.08 | 57.76 | 38.87 | 30.50 | 44.75 | 68.90 | 79.61 | 68.72 | 22.39 |
| Prussian carp | 1072 | 2 | 1 | 1 | 25.13 | 33.31 | 39.61 | 46.97 | 68.53 | 40.56 | 60.43 | 61.85 | 43.35 | 31.54 | 46.64 | 71.95 | 82.22 | 74.87 | 22.53 |
| Prussian carp | 1073 | 2 | 1 | 1 | 22.50 | 22.95 | 27.93 | 31.45 | 55.27 | 40.79 | 43.54 | 42.53 | 31.84 | 25.63 | 35.67 | 47.48 | 58.00 | 48.58 | 17.63 |
| Prussian carp | 1074 | 2 | 1 | 1 | 22.55 | 23.10 | 29.53 | 36.95 | 64.01 | 44.14 | 54.56 | 54.31 | 38.33 | 32.82 | 46.04 | 63.02 | 78.27 | 69.69 | 19.09 |
| Prussian carp | 1075 | 2 | 1 | 1 | 28.68 | 24.42 | 30.55 | 44.15 | 67.13 | 44.20 | 54.33 | 57.03 | 40.65 | 28.73 | 42.49 | 64.95 | 75.34 | 65.08 | 21.56 |
| Prussian carp | 1076 | 2 | 1 | 1 | 27.04 | 27.93 | 33.13 | 36.54 | 63.17 | 42.29 | 53.47 | 55.51 | 33.63 | 30.27 | 41.21 | 61.21 | 76.21 | 69.88 | 19.85 |
| Prussian carp | 1077 | 2 | 1 | 1 | 26.07 | 33.16 | 35.43 | 39.38 | 63.69 | 42.38 | 59.98 | 57.92 | 43.01 | 28.12 | 44.08 | 71.89 | 87.78 | 78.96 | 23.68 |
| Prussian carp | 1078 | 2 | 1 | 1 | 25.12 | 24.22 | 34.19 | 32.85 | 60.65 | 44.98 | 52.22 | 50.81 | 32.52 | 25.53 | 39.49 | 56.59 | 66.50 | 57.88 | 21.68 |
| Prussian carp | 1079 | 2 | 1 | 1 | 31.20 | 28.02 | 34.81 | 34.37 | 60.91 | 44.35 | 53.89 | 56.42 | 42.09 | 26.84 | 41.02 | 66.34 | 74.82 | 65.69 | 22.05 |
| Prussian carp | 1080 | 2 | 1 | 1 | 30.35 | 30.30 | 34.00 | 38.75 | 57.97 | 36.26 | 54.74 | 53.81 | 39.39 | 30.06 | 43.11 | 65.66 | 76.98 | 63.21 | 23.51 |
| Prussian carp | 1081 | 2 | 1 | 1 | 30.41 | 31.81 | 36.20 | 39.04 | 59.57 | 37.22 | 55.53 | 54.65 | 38.90 | 31.04 | 42.27 | 63.01 | 73.13 | 62.00 | 20.79 |
| Prussian carp | 1082 | 2 | 1 | 1 | 25.73 | 28.59 | 34.19 | 30.80 | 52.32 | 31.35 | 48.25 | 47.54 | 35.15 | 29.62 | 38.47 | 57.87 | 72.99 | 67.35 | 19.40 |
| Prussian carp | 1083 | 2 | 1 | 1 | 26.91 | 24.20 | 29.70 | 33.69 | 54.51 | 35.96 | 48.44 | 48.38 | 30.12 | 25.23 | 38.16 | 57.21 | 68.40 | 59.75 | 20.88 |
| Prussian carp | 1084 | 2 | 1 | 1 | 26.57 | 32.70 | 36.68 | 34.24 | 63.00 | 42.81 | 52.13 | 54.01 | 34.92 | 28.09 | 39.18 | 58.94 | 69.59 | 64.92 | 17.49 |
| Prussian carp | 1085 | 2 | 1 | 1 | 31.86 | 32.15 | 37.93 | 37.68 | 67.00 | 48.14 | 62.17 | 60.94 | 47.02 | 36.64 | 47.52 | 72.44 | 88.39 | 73.50 | 26.44 |
| Prussian carp | 1086 | 2 | 1 | 1 | 34.62 | 27.81 | 34.86 | 31.53 | 58.05 | 37.94 | 54.98 | 50.50 | 36.02 | 26.86 | 41.10 | 60.82 | 78.53 | 67.31 | 24.78 |
| Prussian carp | 1087 | 2 | 1 | 1 | 23.41 | 27.58 | 32.40 | 37.41 | 60.00 | 45.90 | 51.92 | 55.51 | 38.25 | 26.32 | 40.07 | 61.25 | 74.06 | 67.60 | 19.91 |
| Prussian carp | 1088 | 2 | 1 | 1 | 20.12 | 22.65 | 26.44 | 31.50 | 47.89 | 32.05 | 42.58 | 42.85 | 28.21 | 25.52 | 34.81 | 49.37 | 63.80 | 52.48 | 14.49 |
| Prussian carp | 1089 | 2 | 1 | 1 | 26.03 | 28.51 | 32.30 | 43.54 | 62.83 | 42.68 | 57.92 | 58.19 | 34.07 | 31.42 | 46.49 | 65.01 | 80.79 | 75.62 | 20.41 |
| Prussian carp | 1090 | 2 | 1 | 1 | 23.53 | 27.38 | 30.20 | 34.04 | 49.82 | 32.96 | 49.89 | 45.84 | 28.38 | 24.18 | 35.39 | 54.96 | 66.93 | 59.98 | 18.20 |
| Prussian carp | 1061 | 2 | 1 | 2 | 26.20 | 30.46 | 34.65 | 33.92 | 54.73 | 34.18 | 50.63 | 47.12 | 32.16 | 30.19 | 43.04 | 56.02 | 68.77 | 61.56 | 19.36 |
| Prussian carp | 1062 | 2 | 1 | 2 | 28.45 | 33.16 | 35.42 | 36.22 | 59.00 | 38.65 | 55.58 | 52.12 | 40.14 | 32.11 | 45.93 | 68.32 | 84.12 | 75.00 | 23.91 |
| Prussian carp | 1063 | 2 | 1 | 2 | 26.96 | 27.18 | 34.01 | 39.04 | 59.20 | 40.74 | 54.60 | 50.71 | 37.07 | 31.38 | 41.86 | 61.25 | 76.19 | 70.59 | 20.96 |
| Prussian carp | 1064 | 2 | 1 | 2 | 26.75 | 27.47 | 31.75 | 36.73 | 58.53 | 34.17 | 53.32 | 53.07 | 35.25 | 27.32 | 42.37 | 63.57 | 75.06 | 70.79 | 20.84 |
| Prussian carp | 1065 | 2 | 1 | 2 | 16.80 | 19.67 | 24.81 | 39.76 | 55.81 | 39.14 | 51.15 | 49.67 | 31.86 | 25.36 | 38.50 | 58.45 | 68.59 | 60.69 | 18.95 |
| Prussian carp | 1066 | 2 | 1 | 2 | 27.53 | 27.12 | 32.24 | 34.54 | 55.49 | 34.93 | 50.03 | 49.01 | 35.23 | 31.83 | 42.87 | 58.11 | 73.44 | 63.70 | 19.03 |
| Prussian carp | 1067 | 2 | 1 | 2 | 28.68 | 28.16 | 33.07 | 45.27 | 64.66 | 40.95 | 59.92 | 60.05 | 37.89 | 35.24 | 46.94 | 65.36 | 77.01 | 71.85 | 21.60 |
| Prussian carp | 1068 | 2 | 1 | 2 | 28.79 | 29.43 | 36.00 | 39.03 | 64.63 | 42.12 | 54.78 | 51.66 | 38.72 | 30.81 | 45.90 | 64.00 | 78.42 | 68.87 | 20.76 |
| Prussian carp | 1069 | 2 | 1 | 2 | 27.84 | 26.25 | 32.23 | 32.21 | 51.26 | 35.14 | 48.69 | 45.62 | 31.85 | 27.39 | 37.78 | 53.65 | 66.38 | 56.10 | 18.73 |
| Prussian carp | 1070 | 2 | 1 | 2 | 21.37 | 25.63 | 30.88 | 31.60 | 52.82 | 36.69 | 57.90 | 52.59 | 33.18 | 33.06 | 45.46 | 54.73 | 65.98 | 57.37 | 20.54 |
| Prussian carp | 1071 | 2 | 1 | 2 | 21.50 | 26.65 | 33.23 | 40.56 | 64.67 | 43.27 | 57.72 | 56.81 | 38.81 | 29.23 | 42.78 | 68.97 | 77.66 | 70.40 | 22.81 |
| Prussian carp | 1072 | 2 | 1 | 2 | 28.61 | 37.22 | 42.51 | 41.27 | 66.40 | 43.66 | 61.67 | 57.64 | 40.87 | 31.87 | 47.61 | 72.38 | 85.42 | 77.62 | 23.82 |
| Prussian carp | 1073 | 2 | 1 | 2 | 22.90 | 24.05 | 29.76 | 30.88 | 54.86 | 37.15 | 44.37 | 43.87 | 30.47 | 27.20 | 35.94 | 48.08 | 59.43 | 52.65 | 17.44 |
| Prussian carp | 1074 | 2 | 1 | 2 | 21.80 | 25.07 | 30.38 | 35.67 | 62.04 | 47.09 | 52.19 | 53.30 | 39.66 | 31.48 | 45.67 | 63.79 | 78.97 | 72.55 | 22.21 |
| Prussian carp | 1075 | 2 | 1 | 2 | 18.75 | 23.65 | 31.01 | 41.24 | 63.70 | 41.68 | 57.02 | 56.10 | 38.44 | 28.92 | 41.55 | 64.39 | 75.17 | 67.00 | 23.63 |
| Prussian carp | 1076 | 2 | 1 | 2 | 20.64 | 23.63 | 29.75 | 37.11 | 61.16 | 41.76 | 51.59 | 54.31 | 36.26 | 26.52 | 41.86 | 61.69 | 72.26 | 67.66 | 19.20 |
| Prussian carp | 1077 | 2 | 1 | 2 | 25.85 | 34.83 | 36.07 | 40.71 | 64.29 | 41.50 | 59.58 | 59.45 | 43.65 | 29.90 | 47.95 | 72.86 | 89.44 | 78.00 | 25.42 |
| Prussian carp | 1078 | 2 | 1 | 2 | 24.35 | 21.63 | 30.61 | 31.02 | 61.43 | 44.65 | 52.84 | 54.61 | 32.38 | 27.45 | 39.88 | 56.03 | 65.84 | 55.32 | 21.29 |
| Prussian carp | 1079 | 2 | 1 | 2 | 29.42 | 27.03 | 37.62 | 31.81 | 59.98 | 38.84 | 54.33 | 56.25 | 40.60 | 26.75 | 40.83 | 66.38 | 75.56 | 63.97 | 23.84 |
| Prussian carp | 1080 | 2 | 1 | 2 | 27.91 | 33.91 | 36.45 | 38.81 | 62.35 | 41.43 | 55.96 | 54.50 | 38.70 | 32.65 | 43.71 | 63.90 | 76.43 | 65.25 | 22.67 |
| Prussian carp | 1081 | 2 | 1 | 2 | 29.58 | 33.09 | 36.83 | 38.06 | 61.20 | 38.49 | 55.89 | 55.37 | 37.57 | 30.89 | 43.55 | 63.89 | 76.22 | 64.47 | 22.72 |
| Prussian carp | 1082 | 2 | 1 | 2 | 25.83 | 26.74 | 33.12 | 30.20 | 52.52 | 31.79 | 49.08 | 45.95 | 32.48 | 29.08 | 39.53 | 57.05 | 71.39 | 62.53 | 20.24 |
| Prussian carp | 1083 | 2 | 1 | 2 | 24.19 | 26.82 | 31.23 | 32.69 | 55.42 | 34.59 | 47.70 | 49.01 | 34.04 | 26.03 | 37.67 | 56.69 | 65.54 | 56.54 | 20.30 |
| Prussian carp | 1084 | 2 | 1 | 2 | 27.64 | 26.92 | 33.89 | 34.47 | 61.22 | 40.26 | 52.48 | 52.53 | 33.12 | 28.94 | 39.38 | 60.23 | 75.37 | 68.65 | 18.89 |
| Prussian carp | 1085 | 2 | 1 | 2 | 33.38 | 31.22 | 40.25 | 37.30 | 67.50 | 47.80 | 58.98 | 60.40 | 45.77 | 36.63 | 53.52 | 69.97 | 86.11 | 77.50 | 22.20 |
| Prussian carp | 1086 | 2 | 1 | 2 | 28.62 | 31.44 | 37.68 | 32.10 | 56.45 | 36.34 | 53.05 | 50.33 | 34.63 | 29.60 | 42.31 | 60.60 | 75.62 | 67.55 | 25.51 |
| Prussian carp | 1087 | 2 | 1 | 2 | 24.90 | 26.94 | 30.60 | 37.49 | 58.99 | 40.79 | 50.30 | 52.82 | 38.60 | 27.03 | 37.38 | 59.46 | 71.86 | 66.52 | 19.32 |
| Prussian carp | 1088 | 2 | 1 | 2 | 19.84 | 22.54 | 26.21 | 30.02 | 47.77 | 27.60 | 41.62 | 42.02 | 29.23 | 25.11 | 34.06 | 49.56 | 58.35 | 53.05 | 16.40 |
| Prussian carp | 1089 | 2 | 1 | 2 | 27.10 | 32.29 | 36.10 | 42.01 | 61.14 | 37.19 | 55.59 | 55.16 | 37.25 | 29.07 | 44.75 | 67.51 | 78.92 | 74.74 | 20.54 |
| Prussian carp | 1090 | 2 | 1 | 2 | 23.98 | 24.97 | 29.47 | 35.07 | 51.39 | 34.86 | 51.11 | 46.08 | 30.10 | 22.63 | 34.81 | 55.96 | 66.17 | 60.12 | 17.60 |
| Prussian carp | 1061 | 2 | 1 | 3 | 25.11 | 27.27 | 32.02 | 33.22 | 56.84 | 36.60 | 48.49 | 46.83 | 33.33 | 30.80 | 41.13 | 55.46 | 67.83 | 61.12 | 18.27 |
| Prussian carp | 1062 | 2 | 1 | 3 | 29.50 | 34.63 | 37.46 | 37.60 | 60.30 | 35.90 | 55.56 | 54.41 | 39.74 | 30.87 | 45.14 | 65.79 | 82.42 | 74.28 | 23.85 |
| Prussian carp | 1063 | 2 | 1 | 3 | 26.56 | 27.74 | 33.20 | 38.08 | 59.34 | 44.46 | 52.03 | 51.66 | 36.74 | 31.38 | 42.30 | 59.81 | 74.40 | 67.40 | 20.21 |
| Prussian carp | 1064 | 2 | 1 | 3 | 25.94 | 28.90 | 34.14 | 35.27 | 56.50 | 37.40 | 55.22 | 52.13 | 33.82 | 31.35 | 43.14 | 63.51 | 76.69 | 72.19 | 22.08 |
| Prussian carp | 1065 | 2 | 1 | 3 | 23.92 | 28.18 | 31.54 | 35.10 | 55.30 | 37.96 | 51.14 | 50.49 | 32.40 | 24.97 | 37.28 | 57.17 | 66.87 | 59.27 | 18.45 |
| Prussian carp | 1066 | 2 | 1 | 3 | 25.63 | 29.65 | 32.64 | 35.68 | 55.42 | 34.79 | 51.29 | 49.41 | 38.08 | 29.52 | 40.58 | 60.38 | 71.20 | 62.44 | 19.45 |
| Prussian carp | 1067 | 2 | 1 | 3 | 26.14 | 31.40 | 35.81 | 40.33 | 65.85 | 45.96 | 61.24 | 60.86 | 35.65 | 31.05 | 44.56 | 68.62 | 80.50 | 73.04 | 21.60 |
| Prussian carp | 1068 | 2 | 1 | 3 | 32.23 | 31.70 | 35.48 | 36.78 | 63.59 | 40.11 | 54.12 | 54.91 | 41.49 | 27.39 | 43.99 | 65.11 | 78.42 | 68.34 | 22.85 |
| Prussian carp | 1069 | 2 | 1 | 3 | 27.07 | 26.61 | 32.64 | 30.13 | 51.49 | 34.88 | 49.38 | 46.81 | 32.09 | 28.28 | 37.28 | 53.97 | 65.28 | 57.61 | 19.33 |
| Prussian carp | 1070 | 2 | 1 | 3 | 20.53 | 22.87 | 30.67 | 33.51 | 59.70 | 39.22 | 47.69 | 51.24 | 33.48 | 32.69 | 43.74 | 53.85 | 63.45 | 55.81 | 18.11 |
| Prussian carp | 1071 | 2 | 1 | 3 | 23.49 | 28.79 | 35.01 | 39.79 | 64.92 | 44.16 | 58.18 | 57.61 | 40.25 | 28.27 | 44.80 | 65.86 | 80.80 | 69.53 | 23.24 |
| Prussian carp | 1072 | 2 | 1 | 3 | 29.24 | 32.43 | 41.08 | 39.90 | 66.26 | 42.21 | 59.70 | 56.67 | 43.10 | 30.76 | 49.00 | 69.81 | 85.11 | 76.49 | 24.85 |
| Prussian carp | 1073 | 2 | 1 | 3 | 22.33 | 22.71 | 28.36 | 31.54 | 51.73 | 33.54 | 43.52 | 43.02 | 32.42 | 26.32 | 34.74 | 46.57 | 56.60 | 46.17 | 17.10 |
| Prussian carp | 1074 | 2 | 1 | 3 | 27.38 | 30.24 | 33.65 | 35.13 | 60.59 | 42.04 | 52.50 | 53.16 | 38.50 | 34.71 | 47.73 | 63.16 | 77.23 | 68.05 | 20.38 |
| Prussian carp | 1075 | 2 | 1 | 3 | 24.20 | 23.95 | 32.12 | 38.09 | 63.85 | 42.37 | 54.42 | 56.86 | 40.46 | 25.92 | 41.40 | 64.72 | 74.23 | 62.19 | 23.30 |
| Prussian carp | 1076 | 2 | 1 | 3 | 23.20 | 25.22 | 30.69 | 35.71 | 63.57 | 42.42 | 54.03 | 54.74 | 35.91 | 26.39 | 39.07 | 62.61 | 72.19 | 68.09 | 20.22 |
| Prussian carp | 1077 | 2 | 1 | 3 | 23.05 | 36.41 | 36.18 | 42.09 | 63.31 | 40.04 | 61.36 | 59.83 | 41.16 | 32.10 | 46.18 | 72.97 | 88.64 | 81.16 | 23.72 |
| Prussian carp | 1078 | 2 | 1 | 3 | 27.76 | 22.65 | 32.02 | 32.68 | 59.72 | 43.13 | 53.57 | 54.01 | 32.68 | 26.91 | 38.95 | 56.59 | 63.72 | 55.88 | 19.87 |
| Prussian carp | 1079 | 2 | 1 | 3 | 28.75 | 27.64 | 36.62 | 33.02 | 60.36 | 34.62 | 55.95 | 57.01 | 39.37 | 25.36 | 40.39 | 65.33 | 72.23 | 63.97 | 22.40 |
| Prussian carp | 1080 | 2 | 1 | 3 | 30.37 | 33.09 | 37.41 | 34.33 | 61.92 | 41.94 | 54.74 | 54.62 | 39.85 | 31.59 | 41.38 | 65.15 | 78.81 | 67.88 | 22.09 |
| Prussian carp | 1081 | 2 | 1 | 3 | 32.34 | 31.37 | 34.51 | 36.70 | 59.83 | 37.30 | 56.07 | 52.61 | 39.00 | 29.12 | 42.11 | 64.05 | 73.87 | 63.89 | 23.14 |
| Prussian carp | 1082 | 2 | 1 | 3 | 28.05 | 31.09 | 36.50 | 28.81 | 51.79 | 32.38 | 48.59 | 44.93 | 33.97 | 28.99 | 39.61 | 58.88 | 62.78 | 72.32 | 17.66 |
| Prussian carp | 1083 | 2 | 1 | 3 | 25.97 | 29.94 | 33.60 | 31.22 | 55.93 | 38.83 | 48.08 | 47.83 | 32.60 | 25.85 | 37.18 | 57.07 | 69.60 | 60.26 | 21.00 |
| Prussian carp | 1084 | 2 | 1 | 3 | 28.05 | 30.03 | 35.97 | 32.21 | 60.12 | 39.63 | 51.71 | 54.24 | 33.94 | 29.90 | 41.59 | 60.93 | 73.99 | 67.56 | 19.11 |
| Prussian carp | 1085 | 2 | 1 | 3 | 32.53 | 32.53 | 38.04 | 37.47 | 71.17 | 50.50 | 61.97 | 62.41 | 46.49 | 36.90 | 51.13 | 72.19 | 83.93 | 75.90 | 24.54 |
| Prussian carp | 1086 | 2 | 1 | 3 | 24.69 | 32.09 | 34.20 | 37.49 | 58.18 | 40.60 | 51.61 | 48.94 | 37.38 | 27.45 | 47.54 | 61.08 | 76.12 | 71.85 | 26.15 |
| Prussian carp | 1087 | 2 | 1 | 3 | 21.37 | 24.40 | 29.16 | 35.39 | 58.09 | 39.59 | 50.73 | 53.27 | 35.73 | 28.30 | 39.38 | 61.35 | 74.36 | 66.64 | 19.50 |
| Prussian carp | 1088 | 2 | 1 | 3 | 19.93 | 21.72 | 25.01 | 31.34 | 47.05 | 30.60 | 43.14 | 44.17 | 26.75 | 26.04 | 34.42 | 48.49 | 58.80 | 53.28 | 14.81 |
| Prussian carp | 1089 | 2 | 1 | 3 | 23.37 | 32.85 | 35.40 | 39.36 | 61.23 | 37.45 | 57.70 | 58.45 | 36.03 | 31.04 | 42.97 | 68.58 | 79.17 | 77.91 | 20.24 |
| Prussian carp | 1090 | 2 | 1 | 3 | 22.86 | 26.36 | 29.60 | 37.66 | 51.10 | 32.33 | 51.59 | 47.90 | 28.71 | 23.89 | 38.44 | 59.28 | 70.51 | 65.69 | 19.11 |
| Prussian carp | 1161 | 2 | 2 | 1 | 41.50 | 46.72 | 49.84 | 52.81 | 84.37 | 56.12 | 76.38 | 71.42 | 47.52 | 43.64 | 59.03 | 82.04 | 104.56 | 89.00 | 30.68 |
| Prussian carp | 1162 | 2 | 2 | 1 | 41.05 | 41.83 | 45.95 | 54.65 | 78.14 | 48.84 | 71.04 | 68.28 | 56.76 | 37.86 | 51.09 | 80.88 | 95.88 | 84.85 | 27.58 |
| Prussian carp | 1163 | 2 | 2 | 1 | 40.01 | 42.24 | 51.84 | 52.09 | 78.73 | 48.90 | 75.13 | 66.75 | 47.76 | 38.61 | 54.04 | 81.91 | 103.17 | 92.00 | 26.40 |
| Prussian carp | 1164 | 2 | 2 | 1 | 30.59 | 30.41 | 33.97 | 30.39 | 55.39 | 41.14 | 51.63 | 49.38 | 27.84 | 29.95 | 39.15 | 52.88 | 69.52 | 62.12 | 19.06 |
| Prussian carp | 1165 | 2 | 2 | 1 | 36.43 | 37.69 | 42.67 | 42.31 | 69.20 | 45.35 | 66.86 | 66.78 | 44.79 | 40.35 | 53.51 | 76.29 | 94.54 | 83.70 | 27.93 |
| Prussian carp | 1166 | 2 | 2 | 1 | 31.26 | 34.70 | 38.61 | 40.68 | 64.73 | 47.95 | 65.39 | 57.82 | 44.92 | 35.17 | 48.94 | 68.98 | 89.72 | 82.16 | 23.97 |
| Prussian carp | 1167 | 2 | 2 | 1 | 46.84 | 44.25 | 50.93 | 55.43 | 88.22 | 63.99 | 82.35 | 75.35 | 51.78 | 42.42 | 60.14 | 86.15 | 103.36 | 87.59 | 30.86 |
| Prussian carp | 1168 | 2 | 2 | 1 | 37.98 | 37.93 | 43.28 | 46.30 | 72.08 | 45.73 | 68.17 | 62.99 | 50.68 | 36.75 | 52.51 | 81.94 | 101.01 | 91.34 | 27.41 |
| Prussian carp | 1169 | 2 | 2 | 1 | 23.31 | 20.80 | 24.85 | 30.78 | 47.48 | 36.86 | 47.98 | 42.61 | 24.79 | 20.61 | 29.73 | 46.97 | 55.68 | 47.69 | 16.21 |
| Prussian carp | 1170 | 2 | 2 | 1 | 38.80 | 38.96 | 45.26 | 48.43 | 83.61 | 62.16 | 71.09 | 68.60 | 47.17 | 35.01 | 53.05 | 78.83 | 93.29 | 79.62 | 25.85 |
| Prussian carp | 1171 | 2 | 2 | 1 | 25.14 | 26.50 | 28.66 | 29.12 | 47.93 | 34.73 | 42.00 | 39.35 | 28.04 | 21.83 | 29.11 | 46.60 | 59.05 | 47.48 | 16.30 |
| Prussian carp | 1172 | 2 | 2 | 1 | 25.32 | 23.99 | 27.00 | 27.17 | 43.81 | 27.47 | 39.11 | 38.47 | 27.84 | 25.32 | 32.48 | 43.84 | 60.20 | 53.14 | 16.21 |
| Prussian carp | 1173 | 2 | 2 | 1 | 23.04 | 22.95 | 26.71 | 24.45 | 44.20 | 32.60 | 40.85 | 39.05 | 27.24 | 24.28 | 30.02 | 42.95 | 55.21 | 46.93 | 14.51 |
| Prussian carp | 1174 | 2 | 2 | 1 | 20.15 | 21.68 | 22.50 | 24.93 | 35.56 | 23.58 | 35.52 | 35.12 | 24.44 | 17.29 | 24.23 | 39.73 | 49.09 | 42.90 | 12.99 |
| Prussian carp | 1175 | 2 | 2 | 1 | 19.45 | 20.06 | 21.94 | 22.67 | 36.34 | 23.58 | 34.80 | 34.17 | 19.37 | 19.32 | 26.05 | 35.71 | 44.77 | 37.64 | 12.62 |
| Prussian carp | 1176 | 2 | 2 | 1 | 22.90 | 24.97 | 26.56 | 29.76 | 43.03 | 30.38 | 42.49 | 39.33 | 26.65 | 23.82 | 30.75 | 42.77 | 51.64 | 44.29 | 15.00 |
| Prussian carp | 1177 | 2 | 2 | 1 | 20.16 | 20.61 | 23.46 | 27.33 | 40.84 | 28.70 | 38.24 | 36.52 | 24.45 | 23.88 | 29.04 | 41.30 | 51.30 | 42.64 | 15.28 |
| Prussian carp | 1178 | 2 | 2 | 1 | 19.89 | 18.10 | 22.34 | 24.95 | 36.64 | 28.32 | 38.66 | 33.10 | 20.51 | 18.69 | 26.38 | 36.08 | 45.39 | 40.14 | 12.21 |
| Prussian carp | 1179 | 2 | 2 | 1 | 19.95 | 20.64 | 20.56 | 24.03 | 34.57 | 22.64 | 34.30 | 30.39 | 19.09 | 17.64 | 24.21 | 34.96 | 45.05 | 41.20 | 11.51 |
| Prussian carp | 1180 | 2 | 2 | 1 | 21.49 | 19.19 | 21.17 | 25.58 | 34.92 | 23.95 | 36.49 | 32.97 | 20.81 | 20.40 | 26.39 | 36.13 | 42.90 | 38.04 | 12.06 |
| Prussian carp | 1181 | 2 | 2 | 1 | 20.58 | 19.26 | 22.09 | 21.58 | 35.46 | 23.07 | 34.42 | 31.26 | 19.75 | 20.51 | 25.28 | 35.17 | 44.61 | 36.20 | 12.56 |
| Prussian carp | 1182 | 2 | 2 | 1 | 27.07 | 25.14 | 28.09 | 32.95 | 47.10 | 33.20 | 46.14 | 40.01 | 27.25 | 22.79 | 31.25 | 43.66 | 55.31 | 48.19 | 17.54 |
| Prussian carp | 1183 | 2 | 2 | 1 | 24.40 | 25.72 | 29.25 | 25.76 | 50.11 | 34.58 | 42.58 | 41.53 | 31.54 | 23.21 | 30.66 | 46.79 | 59.43 | 49.31 | 17.88 |
| Prussian carp | 1184 | 2 | 2 | 1 | 29.93 | 26.05 | 30.36 | 37.72 | 52.17 | 35.57 | 50.95 | 43.59 | 32.57 | 25.83 | 34.24 | 54.42 | 62.58 | 56.91 | 15.54 |
| Prussian carp | 1185 | 2 | 2 | 1 | 24.18 | 21.47 | 25.36 | 30.32 | 42.55 | 28.57 | 40.98 | 34.74 | 23.00 | 19.51 | 27.14 | 42.61 | 51.57 | 44.83 | 13.22 |
| Prussian carp | 1186 | 2 | 2 | 1 | 31.96 | 30.57 | 34.59 | 30.21 | 53.24 | 35.86 | 51.63 | 47.70 | 34.46 | 23.80 | 39.24 | 57.47 | 70.53 | 61.90 | 18.26 |
| Prussian carp | 1187 | 2 | 2 | 1 | 22.72 | 25.13 | 26.76 | 27.77 | 41.05 | 26.42 | 43.76 | 36.85 | 18.76 | 21.98 | 29.28 | 42.02 | 53.51 | 47.63 | 15.00 |
| Prussian carp | 1188 | 2 | 2 | 1 | 22.21 | 22.70 | 26.89 | 27.31 | 47.02 | 34.63 | 42.18 | 38.56 | 28.11 | 18.76 | 26.92 | 43.01 | 48.33 | 42.84 | 16.03 |
| Prussian carp | 1189 | 2 | 2 | 1 | 28.71 | 27.54 | 32.41 | 31.63 | 54.28 | 37.71 | 50.60 | 47.12 | 28.72 | 26.80 | 36.54 | 51.80 | 64.18 | 56.61 | 19.36 |
| Prussian carp | 1190 | 2 | 2 | 1 | 21.18 | 22.05 | 24.00 | 25.81 | 39.57 | 27.67 | 37.13 | 35.47 | 28.20 | 20.16 | 28.96 | 37.98 | 51.13 | 46.70 | 15.00 |
| Prussian carp | 1161 | 2 | 2 | 2 | 38.11 | 43.13 | 48.43 | 51.53 | 84.73 | 58.89 | 77.55 | 72.25 | 47.29 | 40.64 | 60.66 | 85.01 | 106.21 | 94.60 | 31.40 |
| Prussian carp | 1162 | 2 | 2 | 2 | 42.07 | 40.04 | 44.41 | 52.06 | 75.76 | 55.72 | 74.05 | 65.71 | 47.75 | 40.33 | 55.58 | 75.68 | 99.03 | 88.70 | 25.51 |
| Prussian carp | 1163 | 2 | 2 | 2 | 39.44 | 36.72 | 45.49 | 51.21 | 77.03 | 54.65 | 80.20 | 69.95 | 46.63 | 35.74 | 52.83 | 82.24 | 101.69 | 90.42 | 27.44 |
| Prussian carp | 1164 | 2 | 2 | 2 | 28.60 | 29.89 | 33.18 | 34.31 | 56.05 | 38.66 | 51.14 | 49.06 | 30.49 | 27.92 | 38.77 | 54.46 | 67.49 | 60.34 | 18.21 |
| Prussian carp | 1165 | 2 | 2 | 2 | 34.01 | 35.44 | 40.42 | 44.43 | 72.93 | 50.04 | 68.10 | 68.16 | 42.84 | 41.65 | 54.34 | 75.41 | 95.06 | 84.58 | 29.40 |
| Prussian carp | 1166 | 2 | 2 | 2 | 31.67 | 37.75 | 41.61 | 42.87 | 66.86 | 50.93 | 64.01 | 58.21 | 46.34 | 35.30 | 47.67 | 69.07 | 89.63 | 83.99 | 24.78 |
| Prussian carp | 1167 | 2 | 2 | 2 | 47.56 | 44.97 | 50.82 | 59.65 | 89.62 | 70.39 | 85.65 | 76.41 | 48.01 | 43.72 | 59.67 | 84.60 | 103.04 | 90.42 | 29.80 |
| Prussian carp | 1168 | 2 | 2 | 2 | 36.67 | 35.16 | 42.16 | 43.00 | 68.85 | 45.58 | 67.33 | 64.11 | 50.71 | 33.65 | 54.40 | 81.99 | 102.62 | 92.55 | 27.42 |
| Prussian carp | 1169 | 2 | 2 | 2 | 23.01 | 20.86 | 26.56 | 28.24 | 45.66 | 42.49 | 45.68 | 43.67 | 28.17 | 20.81 | 29.50 | 47.68 | 55.60 | 47.14 | 15.46 |
| Prussian carp | 1170 | 2 | 2 | 2 | 37.58 | 38.55 | 42.41 | 48.25 | 77.36 | 52.68 | 74.11 | 69.61 | 43.98 | 39.56 | 56.53 | 80.56 | 94.35 | 81.70 | 27.64 |
| Prussian carp | 1171 | 2 | 2 | 2 | 23.38 | 24.19 | 27.15 | 29.32 | 47.42 | 36.13 | 44.22 | 39.63 | 26.81 | 20.15 | 29.38 | 46.98 | 57.90 | 49.03 | 16.00 |
| Prussian carp | 1172 | 2 | 2 | 2 | 22.30 | 24.08 | 25.42 | 26.22 | 43.09 | 28.66 | 39.70 | 35.40 | 30.61 | 24.09 | 29.85 | 43.09 | 55.91 | 50.14 | 15.42 |
| Prussian carp | 1173 | 2 | 2 | 2 | 26.46 | 23.62 | 26.26 | 26.10 | 44.00 | 31.28 | 39.74 | 38.84 | 29.23 | 24.25 | 31.94 | 43.83 | 56.32 | 48.05 | 16.88 |
| Prussian carp | 1174 | 2 | 2 | 2 | 21.07 | 21.97 | 23.73 | 24.23 | 35.25 | 22.73 | 35.34 | 34.45 | 22.47 | 20.01 | 27.19 | 38.82 | 50.10 | 44.08 | 13.50 |
| Prussian carp | 1175 | 2 | 2 | 2 | 18.70 | 21.74 | 22.78 | 23.31 | 36.17 | 22.33 | 33.38 | 33.31 | 24.42 | 15.65 | 24.40 | 36.70 | 46.14 | 40.71 | 12.45 |
| Prussian carp | 1176 | 2 | 2 | 2 | 22.49 | 26.71 | 27.16 | 27.83 | 44.05 | 27.94 | 40.45 | 39.88 | 26.31 | 24.49 | 31.41 | 41.19 | 50.62 | 44.94 | 15.39 |
| Prussian carp | 1177 | 2 | 2 | 2 | 23.28 | 18.64 | 23.90 | 25.70 | 39.74 | 28.42 | 40.58 | 36.51 | 24.01 | 20.32 | 27.31 | 41.92 | 51.40 | 42.81 | 13.51 |
| Prussian carp | 1178 | 2 | 2 | 2 | 20.02 | 19.26 | 22.65 | 25.50 | 37.88 | 27.51 | 38.15 | 32.89 | 22.31 | 17.25 | 24.96 | 38.29 | 46.66 | 42.66 | 13.37 |
| Prussian carp | 1179 | 2 | 2 | 2 | 22.13 | 21.18 | 20.36 | 22.62 | 33.33 | 22.05 | 33.96 | 29.99 | 19.94 | 18.88 | 24.91 | 33.83 | 44.61 | 39.25 | 12.76 |
| Prussian carp | 1180 | 2 | 2 | 2 | 21.72 | 19.26 | 20.26 | 25.25 | 34.73 | 23.80 | 35.01 | 30.92 | 21.07 | 20.34 | 26.35 | 34.96 | 43.04 | 37.41 | 11.30 |
| Prussian carp | 1181 | 2 | 2 | 2 | 20.02 | 21.05 | 21.04 | 21.92 | 35.19 | 24.86 | 34.05 | 31.20 | 22.22 | 19.93 | 23.17 | 35.20 | 44.33 | 35.92 | 13.09 |
| Prussian carp | 1182 | 2 | 2 | 2 | 25.05 | 26.52 | 28.30 | 31.13 | 50.57 | 38.43 | 46.68 | 39.46 | 28.03 | 24.16 | 30.53 | 46.33 | 57.50 | 49.82 | 16.55 |
| Prussian carp | 1183 | 2 | 2 | 2 | 23.49 | 24.76 | 27.61 | 25.29 | 47.38 | 33.04 | 44.09 | 40.99 | 30.15 | 22.28 | 29.64 | 45.83 | 58.21 | 48.80 | 18.05 |
| Prussian carp | 1184 | 2 | 2 | 2 | 26.00 | 26.16 | 30.66 | 35.90 | 52.00 | 36.31 | 51.24 | 46.15 | 32.36 | 25.79 | 36.12 | 53.21 | 63.14 | 55.65 | 17.38 |
| Prussian carp | 1185 | 2 | 2 | 2 | 21.68 | 23.10 | 25.14 | 26.18 | 39.83 | 26.79 | 41.72 | 35.81 | 19.77 | 17.05 | 25.71 | 42.42 | 51.63 | 43.45 | 13.22 |
| Prussian carp | 1186 | 2 | 2 | 2 | 29.75 | 31.96 | 32.48 | 31.79 | 53.41 | 32.73 | 48.10 | 48.30 | 36.03 | 28.47 | 40.61 | 60.60 | 72.08 | 63.99 | 19.27 |
| Prussian carp | 1187 | 2 | 2 | 2 | 22.90 | 24.55 | 26.13 | 31.08 | 41.12 | 26.42 | 42.43 | 36.32 | 21.22 | 21.47 | 29.73 | 42.80 | 56.53 | 50.53 | 16.55 |
| Prussian carp | 1188 | 2 | 2 | 2 | 25.76 | 23.48 | 27.45 | 26.26 | 46.44 | 31.85 | 41.66 | 40.11 | 27.75 | 17.27 | 28.34 | 41.80 | 49.31 | 42.13 | 14.68 |
| Prussian carp | 1189 | 2 | 2 | 2 | 30.09 | 28.09 | 32.73 | 31.48 | 52.35 | 36.15 | 50.39 | 47.56 | 29.62 | 24.51 | 35.63 | 53.05 | 63.97 | 56.28 | 18.47 |
| Prussian carp | 1190 | 2 | 2 | 2 | 21.61 | 23.13 | 23.81 | 24.36 | 38.00 | 25.06 | 35.86 | 33.77 | 24.74 | 22.97 | 30.67 | 37.82 | 50.25 | 47.55 | 14.75 |
| Prussian carp | 1161 | 2 | 2 | 3 | 39.48 | 46.02 | 50.47 | 51.63 | 84.79 | 54.60 | 75.76 | 72.69 | 49.37 | 39.11 | 57.32 | 85.16 | 103.25 | 89.24 | 30.30 |
| Prussian carp | 1162 | 2 | 2 | 3 | 43.11 | 39.79 | 45.04 | 55.89 | 79.31 | 52.47 | 69.15 | 66.58 | 50.93 | 40.70 | 55.81 | 76.45 | 99.59 | 89.44 | 28.90 |
| Prussian carp | 1163 | 2 | 2 | 3 | 39.53 | 43.84 | 51.63 | 52.97 | 76.75 | 50.79 | 77.86 | 66.01 | 47.11 | 40.55 | 55.16 | 82.87 | 101.78 | 92.25 | 27.14 |
| Prussian carp | 1164 | 2 | 2 | 3 | 26.96 | 30.02 | 34.22 | 35.50 | 56.77 | 37.64 | 50.79 | 48.27 | 27.13 | 31.83 | 40.49 | 51.87 | 65.74 | 59.52 | 18.45 |
| Prussian carp | 1165 | 2 | 2 | 3 | 37.76 | 36.64 | 44.45 | 40.14 | 72.01 | 46.04 | 67.47 | 66.00 | 43.87 | 41.46 | 54.09 | 75.48 | 94.57 | 82.51 | 27.11 |
| Prussian carp | 1166 | 2 | 2 | 3 | 33.19 | 38.67 | 38.20 | 43.17 | 65.67 | 47.96 | 64.41 | 58.53 | 43.41 | 36.82 | 49.57 | 68.62 | 89.66 | 82.92 | 22.26 |
| Prussian carp | 1167 | 2 | 2 | 3 | 47.44 | 45.62 | 51.23 | 51.53 | 92.30 | 68.16 | 82.46 | 76.33 | 50.33 | 44.10 | 60.49 | 85.59 | 102.02 | 87.94 | 31.40 |
| Prussian carp | 1168 | 2 | 2 | 3 | 37.95 | 36.77 | 44.37 | 45.26 | 74.65 | 48.91 | 67.63 | 63.93 | 51.44 | 40.23 | 54.11 | 82.24 | 99.16 | 90.34 | 29.40 |
| Prussian carp | 1169 | 2 | 2 | 3 | 25.42 | 25.65 | 27.02 | 26.67 | 44.63 | 31.45 | 45.86 | 42.67 | 27.45 | 20.59 | 27.16 | 48.34 | 56.21 | 48.92 | 14.86 |
| Prussian carp | 1170 | 2 | 2 | 3 | 37.21 | 37.57 | 44.02 | 50.56 | 81.53 | 56.75 | 73.21 | 71.43 | 50.25 | 33.62 | 53.01 | 80.43 | 94.89 | 84.44 | 28.66 |
| Prussian carp | 1171 | 2 | 2 | 3 | 25.94 | 26.90 | 29.27 | 25.22 | 47.47 | 28.61 | 39.95 | 39.50 | 27.82 | 23.83 | 31.04 | 44.83 | 54.84 | 48.15 | 16.15 |
| Prussian carp | 1172 | 2 | 2 | 3 | 24.49 | 26.14 | 26.11 | 25.73 | 44.82 | 29.98 | 36.85 | 38.51 | 34.08 | 20.92 | 29.92 | 47.11 | 62.48 | 54.84 | 18.80 |
| Prussian carp | 1173 | 2 | 2 | 3 | 23.09 | 21.90 | 24.91 | 28.28 | 44.26 | 29.48 | 42.48 | 40.08 | 27.30 | 25.08 | 34.16 | 44.57 | 56.94 | 49.37 | 15.61 |
| Prussian carp | 1174 | 2 | 2 | 3 | 21.82 | 19.99 | 23.07 | 21.37 | 35.03 | 23.18 | 35.88 | 34.02 | 24.91 | 18.90 | 27.12 | 39.22 | 48.61 | 43.36 | 13.21 |
| Prussian carp | 1175 | 2 | 2 | 3 | 19.48 | 22.21 | 22.59 | 23.91 | 37.02 | 27.08 | 34.71 | 32.85 | 22.72 | 16.96 | 25.35 | 37.78 | 46.41 | 40.99 | 14.14 |
| Prussian carp | 1176 | 2 | 2 | 3 | 24.09 | 25.32 | 26.60 | 31.58 | 44.89 | 28.68 | 41.62 | 38.60 | 26.51 | 23.77 | 33.68 | 43.05 | 50.71 | 42.93 | 14.41 |
| Prussian carp | 1177 | 2 | 2 | 3 | 22.81 | 21.26 | 24.63 | 27.66 | 42.55 | 28.20 | 38.54 | 36.76 | 26.54 | 22.15 | 28.38 | 41.71 | 51.83 | 44.47 | 14.43 |
| Prussian carp | 1178 | 2 | 2 | 3 | 20.91 | 21.04 | 22.71 | 27.79 | 38.66 | 27.66 | 38.54 | 33.49 | 21.13 | 17.89 | 24.98 | 36.57 | 43.91 | 39.23 | 12.70 |
| Prussian carp | 1179 | 2 | 2 | 3 | 19.68 | 21.64 | 22.54 | 22.82 | 34.79 | 23.13 | 34.47 | 31.11 | 17.83 | 21.99 | 24.78 | 35.80 | 46.76 | 42.44 | 12.93 |
| Prussian carp | 1180 | 2 | 2 | 3 | 21.52 | 17.98 | 21.17 | 23.83 | 35.88 | 23.98 | 34.97 | 32.46 | 21.27 | 20.46 | 26.83 | 34.63 | 42.33 | 38.13 | 11.58 |
| Prussian carp | 1181 | 2 | 2 | 3 | 19.20 | 19.14 | 21.09 | 24.05 | 36.89 | 23.06 | 33.75 | 31.99 | 20.20 | 18.53 | 24.91 | 37.18 | 46.86 | 40.29 | 11.86 |
| Prussian carp | 1182 | 2 | 2 | 3 | 26.77 | 26.11 | 28.53 | 30.00 | 48.05 | 36.98 | 46.95 | 38.82 | 28.77 | 21.28 | 31.54 | 46.15 | 55.57 | 50.99 | 16.09 |
| Prussian carp | 1183 | 2 | 2 | 3 | 24.17 | 24.69 | 28.53 | 27.48 | 48.33 | 33.69 | 41.59 | 40.08 | 32.32 | 25.02 | 31.65 | 48.61 | 60.13 | 52.24 | 19.02 |
| Prussian carp | 1184 | 2 | 2 | 3 | 28.24 | 24.25 | 29.10 | 36.98 | 52.56 | 34.27 | 48.32 | 43.92 | 31.73 | 28.06 | 36.44 | 49.87 | 64.14 | 52.58 | 18.86 |
| Prussian carp | 1185 | 2 | 2 | 3 | 23.13 | 23.18 | 26.33 | 32.49 | 40.85 | 25.13 | 39.40 | 35.04 | 24.82 | 20.39 | 26.68 | 45.41 | 52.59 | 45.92 | 14.82 |
| Prussian carp | 1186 | 2 | 2 | 3 | 32.21 | 28.51 | 33.40 | 35.09 | 54.55 | 35.96 | 50.08 | 49.61 | 33.70 | 26.21 | 38.69 | 58.98 | 70.78 | 60.20 | 20.03 |
| Prussian carp | 1187 | 2 | 2 | 3 | 21.53 | 23.08 | 24.81 | 30.86 | 41.12 | 27.22 | 44.43 | 36.55 | 22.83 | 21.22 | 28.87 | 43.58 | 56.36 | 50.06 | 16.35 |
| Prussian carp | 1188 | 2 | 2 | 3 | 22.82 | 21.75 | 26.71 | 26.57 | 48.18 | 34.85 | 40.59 | 40.06 | 28.97 | 19.76 | 27.31 | 41.01 | 45.96 | 39.11 | 15.81 |
| Prussian carp | 1189 | 2 | 2 | 3 | 28.96 | 27.38 | 32.60 | 30.05 | 53.08 | 35.44 | 49.47 | 48.06 | 30.61 | 24.58 | 36.90 | 54.37 | 64.81 | 56.46 | 17.87 |
| Prussian carp | 1190 | 2 | 2 | 3 | 22.03 | 22.43 | 23.44 | 25.00 | 39.61 | 29.08 | 36.94 | 33.45 | 28.92 | 18.98 | 26.55 | 39.57 | 50.12 | 45.66 | 15.01 |
| Prussian carp | 1251 | 2 | 3 | 1 | 23.56 | 22.47 | 29.72 | 49.37 | 71.93 | 44.46 | 61.33 | 63.39 | 38.22 | 37.67 | 47.76 | 70.47 | 86.05 | 74.66 | 21.00 |
| Prussian carp | 1252 | 2 | 3 | 1 | 43.26 | 36.29 | 46.21 | 47.80 | 76.77 | 39.40 | 73.34 | 74.17 | 44.60 | 37.40 | 54.25 | 84.44 | 100.69 | 88.44 | 27.61 |
| Prussian carp | 1253 | 2 | 3 | 1 | 34.72 | 32.72 | 41.82 | 48.13 | 76.45 | 50.85 | 69.58 | 72.23 | 48.71 | 35.62 | 54.87 | 81.17 | 93.14 | 82.92 | 26.85 |
| Prussian carp | 1254 | 2 | 3 | 1 | 32.74 | 43.09 | 42.03 | 42.91 | 65.43 | 41.76 | 64.07 | 57.79 | 34.89 | 35.73 | 43.21 | 65.32 | 88.87 | 81.39 | 24.07 |
| Prussian carp | 1255 | 2 | 3 | 1 | 27.92 | 29.37 | 35.79 | 34.26 | 60.08 | 37.83 | 53.70 | 54.92 | 40.23 | 30.98 | 42.69 | 63.54 | 74.35 | 63.87 | 20.53 |
| Prussian carp | 1256 | 2 | 3 | 1 | 33.14 | 33.19 | 38.12 | 39.60 | 67.93 | 46.89 | 59.41 | 58.23 | 39.69 | 31.76 | 46.64 | 62.63 | 75.02 | 65.39 | 24.40 |
| Prussian carp | 1257 | 2 | 3 | 1 | 28.99 | 29.05 | 35.27 | 41.02 | 70.13 | 48.01 | 57.65 | 56.03 | 41.30 | 30.43 | 46.14 | 70.38 | 83.35 | 77.30 | 23.80 |
| Prussian carp | 1258 | 2 | 3 | 1 | 31.11 | 27.74 | 29.21 | 34.87 | 59.95 | 41.75 | 51.79 | 52.39 | 41.78 | 31.51 | 40.44 | 59.79 | 70.76 | 59.33 | 18.17 |
| Prussian carp | 1259 | 2 | 3 | 1 | 25.80 | 26.88 | 33.31 | 36.47 | 60.45 | 40.56 | 53.27 | 52.64 | 37.91 | 27.46 | 41.03 | 60.86 | 75.72 | 68.64 | 20.60 |
| Prussian carp | 1260 | 2 | 3 | 1 | 30.13 | 31.76 | 38.08 | 36.07 | 59.80 | 34.16 | 55.93 | 54.57 | 39.24 | 32.40 | 43.42 | 68.41 | 84.26 | 73.37 | 22.53 |
| Prussian carp | 1261 | 2 | 3 | 1 | 33.08 | 30.84 | 29.46 | 37.80 | 74.33 | 44.75 | 53.28 | 64.78 | 49.04 | 33.81 | 49.58 | 70.23 | 85.12 | 80.58 | 27.30 |
| Prussian carp | 1262 | 2 | 3 | 1 | 30.77 | 28.90 | 32.83 | 36.21 | 55.09 | 35.56 | 53.50 | 49.21 | 34.22 | 28.11 | 42.61 | 56.73 | 66.35 | 59.60 | 19.49 |
| Prussian carp | 1263 | 2 | 3 | 1 | 29.05 | 27.80 | 33.34 | 37.49 | 60.08 | 36.64 | 55.67 | 53.11 | 37.56 | 28.94 | 42.18 | 64.00 | 73.28 | 63.95 | 22.80 |
| Prussian carp | 1264 | 2 | 3 | 1 | 40.16 | 32.00 | 44.10 | 46.22 | 78.01 | 52.35 | 72.14 | 75.57 | 49.87 | 39.12 | 58.58 | 82.52 | 99.86 | 87.82 | 31.80 |
| Prussian carp | 1265 | 2 | 3 | 1 | 27.05 | 27.02 | 34.43 | 37.42 | 63.46 | 41.79 | 57.15 | 54.96 | 39.70 | 31.93 | 41.93 | 67.21 | 84.39 | 69.59 | 25.03 |
| Prussian carp | 1266 | 2 | 3 | 1 | 28.16 | 26.23 | 32.80 | 33.16 | 63.92 | 44.35 | 54.97 | 55.86 | 38.30 | 27.99 | 43.63 | 65.31 | 80.03 | 71.87 | 22.25 |
| Prussian carp | 1267 | 2 | 3 | 1 | 28.03 | 29.49 | 35.43 | 34.44 | 59.77 | 37.48 | 54.74 | 54.10 | 35.67 | 28.59 | 43.03 | 64.65 | 78.75 | 72.42 | 21.68 |
| Prussian carp | 1268 | 2 | 3 | 1 | 30.00 | 29.14 | 39.69 | 33.10 | 65.23 | 44.64 | 58.84 | 63.48 | 37.85 | 33.43 | 49.89 | 70.21 | 84.76 | 74.08 | 25.93 |
| Prussian carp | 1269 | 2 | 3 | 1 | 28.23 | 29.99 | 33.00 | 33.33 | 55.57 | 37.05 | 50.70 | 46.20 | 32.42 | 22.15 | 35.34 | 55.30 | 64.87 | 59.68 | 18.72 |
| Prussian carp | 1270 | 2 | 3 | 1 | 30.77 | 31.59 | 37.23 | 36.37 | 66.17 | 45.08 | 59.44 | 60.85 | 37.16 | 28.77 | 42.44 | 68.61 | 80.18 | 79.39 | 21.81 |
| Prussian carp | 1271 | 2 | 3 | 1 | 34.62 | 32.62 | 35.22 | 36.38 | 60.26 | 38.11 | 56.75 | 53.61 | 41.30 | 27.82 | 41.58 | 61.91 | 71.97 | 61.72 | 22.46 |
| Prussian carp | 1272 | 2 | 3 | 1 | 25.44 | 24.14 | 29.24 | 32.76 | 56.71 | 38.44 | 51.04 | 54.09 | 40.12 | 28.05 | 38.12 | 56.02 | 64.66 | 56.51 | 18.22 |
| Prussian carp | 1273 | 2 | 3 | 1 | 27.91 | 25.95 | 33.28 | 40.02 | 61.18 | 42.31 | 58.31 | 60.27 | 40.25 | 31.50 | 42.47 | 67.33 | 77.81 | 66.58 | 21.45 |
| Prussian carp | 1274 | 2 | 3 | 1 | 30.29 | 33.61 | 37.38 | 34.68 | 60.09 | 40.29 | 57.59 | 55.82 | 41.07 | 25.76 | 39.17 | 63.70 | 74.97 | 62.40 | 21.93 |
| Prussian carp | 1275 | 2 | 3 | 1 | 27.82 | 31.14 | 37.00 | 40.09 | 71.01 | 52.22 | 65.74 | 66.62 | 46.21 | 30.12 | 47.35 | 77.06 | 89.09 | 79.03 | 24.65 |
| Prussian carp | 4030 | 2 | 3 | 1 | 35.03 | 33.81 | 38.78 | 40.94 | 71.07 | 48.50 | 65.82 | 64.81 | 44.78 | 32.35 | 48.16 | 72.57 | 91.08 | 81.08 | 27.02 |
| Prussian carp | 4031 | 2 | 3 | 1 | 30.42 | 30.85 | 34.49 | 37.37 | 61.24 | 39.12 | 53.65 | 53.55 | 40.99 | 19.70 | 37.58 | 68.04 | 80.24 | 75.94 | 20.21 |
| Prussian carp | 4032 | 2 | 3 | 1 | 31.46 | 29.95 | 36.31 | 42.73 | 72.21 | 51.77 | 63.49 | 62.42 | 40.02 | 35.69 | 48.00 | 71.56 | 85.33 | 71.63 | 23.60 |
| Prussian carp | 4033 | 2 | 3 | 1 | 24.96 | 31.88 | 37.49 | 39.12 | 61.61 | 39.26 | 58.57 | 63.64 | 41.98 | 27.37 | 48.20 | 72.93 | 75.98 | 66.87 | 25.23 |
| Prussian carp | 4034 | 2 | 3 | 1 | 38.94 | 36.11 | 44.41 | 43.31 | 78.68 | 52.88 | 68.20 | 67.53 | 49.43 | 28.68 | 48.95 | 78.21 | 84.08 | 77.50 | 26.21 |
| Prussian carp | 1251 | 2 | 3 | 2 | 36.03 | 33.37 | 41.40 | 38.14 | 68.65 | 41.94 | 60.51 | 63.32 | 38.20 | 36.07 | 48.37 | 70.17 | 86.31 | 72.66 | 21.94 |
| Prussian carp | 1252 | 2 | 3 | 2 | 37.03 | 37.69 | 47.71 | 46.38 | 80.63 | 48.97 | 72.84 | 72.61 | 47.52 | 34.39 | 53.00 | 86.45 | 101.28 | 89.96 | 29.10 |
| Prussian carp | 1253 | 2 | 3 | 2 | 32.63 | 32.40 | 43.43 | 45.31 | 77.18 | 56.21 | 70.86 | 73.01 | 49.32 | 33.21 | 54.64 | 83.34 | 92.50 | 83.37 | 26.80 |
| Prussian carp | 1254 | 2 | 3 | 2 | 33.71 | 39.05 | 41.76 | 42.81 | 64.07 | 40.13 | 61.74 | 56.17 | 40.20 | 33.10 | 44.61 | 67.57 | 87.83 | 80.58 | 23.11 |
| Prussian carp | 1255 | 2 | 3 | 2 | 28.05 | 25.35 | 32.11 | 31.63 | 60.72 | 37.96 | 53.51 | 55.06 | 41.18 | 33.94 | 41.50 | 63.51 | 74.20 | 65.07 | 19.59 |
| Prussian carp | 1256 | 2 | 3 | 2 | 34.24 | 34.56 | 38.69 | 39.12 | 64.99 | 42.87 | 59.69 | 56.81 | 40.15 | 31.16 | 46.30 | 66.79 | 79.54 | 69.86 | 23.42 |
| Prussian carp | 1257 | 2 | 3 | 2 | 29.53 | 27.62 | 35.23 | 38.03 | 65.53 | 42.60 | 56.64 | 56.45 | 41.70 | 31.93 | 47.41 | 69.47 | 81.94 | 76.82 | 22.21 |
| Prussian carp | 1258 | 2 | 3 | 2 | 27.79 | 26.37 | 30.42 | 35.13 | 59.91 | 42.34 | 53.62 | 53.88 | 40.76 | 30.43 | 40.73 | 60.80 | 71.94 | 61.51 | 17.71 |
| Prussian carp | 1259 | 2 | 3 | 2 | 24.44 | 27.77 | 33.63 | 37.18 | 60.17 | 39.75 | 53.90 | 50.89 | 36.77 | 25.86 | 37.62 | 58.19 | 76.67 | 68.35 | 20.42 |
| Prussian carp | 1260 | 2 | 3 | 2 | 29.49 | 30.49 | 36.65 | 38.53 | 60.37 | 36.15 | 56.42 | 56.16 | 40.15 | 31.32 | 41.19 | 68.30 | 88.32 | 75.75 | 23.24 |
| Prussian carp | 1261 | 2 | 3 | 2 | 30.24 | 31.15 | 41.14 | 43.69 | 74.69 | 49.66 | 65.50 | 62.75 | 47.31 | 30.50 | 50.81 | 71.23 | 86.08 | 80.35 | 26.25 |
| Prussian carp | 1262 | 2 | 3 | 2 | 30.93 | 26.58 | 34.21 | 34.68 | 57.70 | 37.65 | 53.01 | 49.41 | 35.88 | 29.55 | 43.39 | 56.45 | 67.01 | 60.02 | 22.91 |
| Prussian carp | 1263 | 2 | 3 | 2 | 24.55 | 29.33 | 36.01 | 37.68 | 61.34 | 40.99 | 59.05 | 54.90 | 38.15 | 26.47 | 41.76 | 61.67 | 70.70 | 60.35 | 21.30 |
| Prussian carp | 1264 | 2 | 3 | 2 | 36.90 | 36.13 | 44.51 | 42.66 | 80.50 | 47.53 | 71.59 | 71.76 | 46.82 | 36.91 | 54.10 | 84.30 | 104.61 | 95.76 | 30.40 |
| Prussian carp | 1265 | 2 | 3 | 2 | 27.72 | 27.43 | 33.95 | 38.19 | 60.59 | 34.82 | 55.51 | 55.13 | 41.68 | 27.52 | 41.58 | 69.53 | 84.81 | 74.59 | 22.65 |
| Prussian carp | 1266 | 2 | 3 | 2 | 27.03 | 26.37 | 34.25 | 32.12 | 61.94 | 36.43 | 53.79 | 55.37 | 39.28 | 28.46 | 41.70 | 66.52 | 82.69 | 77.28 | 21.19 |
| Prussian carp | 1267 | 2 | 3 | 2 | 29.97 | 31.56 | 37.23 | 32.32 | 59.02 | 37.75 | 53.90 | 55.16 | 39.64 | 28.61 | 40.93 | 66.58 | 78.44 | 71.28 | 20.00 |
| Prussian carp | 1268 | 2 | 3 | 2 | 32.44 | 27.97 | 38.06 | 35.93 | 67.15 | 42.92 | 59.65 | 63.46 | 39.92 | 33.58 | 47.16 | 69.35 | 84.01 | 74.87 | 26.80 |
| Prussian carp | 1269 | 2 | 3 | 2 | 28.13 | 30.81 | 33.93 | 37.15 | 57.57 | 42.13 | 52.84 | 45.97 | 32.67 | 21.87 | 33.54 | 55.51 | 67.37 | 59.69 | 18.92 |
| Prussian carp | 1270 | 2 | 3 | 2 | 30.44 | 26.59 | 34.40 | 34.88 | 64.50 | 43.37 | 62.98 | 60.38 | 35.37 | 25.33 | 41.87 | 70.99 | 81.61 | 81.43 | 23.37 |
| Prussian carp | 1271 | 2 | 3 | 2 | 37.67 | 35.52 | 37.80 | 34.49 | 64.25 | 41.94 | 55.56 | 53.44 | 39.21 | 32.04 | 44.65 | 63.49 | 75.86 | 63.29 | 23.19 |
| Prussian carp | 1272 | 2 | 3 | 2 | 25.40 | 23.91 | 29.65 | 33.99 | 59.19 | 43.08 | 53.86 | 53.39 | 39.07 | 22.91 | 36.96 | 57.63 | 66.41 | 55.83 | 19.22 |
| Prussian carp | 1273 | 2 | 3 | 2 | 29.09 | 27.13 | 33.80 | 38.30 | 63.58 | 43.07 | 57.30 | 58.36 | 43.01 | 27.79 | 41.79 | 68.06 | 75.52 | 65.29 | 20.36 |
| Prussian carp | 1274 | 2 | 3 | 2 | 27.79 | 30.72 | 37.72 | 38.08 | 68.82 | 48.33 | 57.71 | 54.48 | 38.84 | 27.42 | 40.67 | 63.07 | 73.85 | 63.63 | 22.52 |
| Prussian carp | 1275 | 2 | 3 | 2 | 35.81 | 29.96 | 38.66 | 39.30 | 74.47 | 54.71 | 64.41 | 67.40 | 42.29 | 37.84 | 51.65 | 71.66 | 85.52 | 81.48 | 21.60 |
| Prussian carp | 4030 | 2 | 3 | 2 | 35.44 | 32.88 | 41.29 | 42.96 | 72.90 | 47.41 | 65.99 | 62.93 | 43.52 | 29.98 | 44.57 | 70.91 | 89.75 | 79.26 | 26.10 |
| Prussian carp | 4031 | 2 | 3 | 2 | 32.63 | 28.21 | 35.91 | 31.21 | 61.21 | 38.56 | 52.78 | 53.18 | 39.63 | 22.50 | 40.03 | 63.70 | 75.70 | 68.37 | 22.41 |
| Prussian carp | 4032 | 2 | 3 | 2 | 20.48 | 29.70 | 31.84 | 46.27 | 70.07 | 47.86 | 62.80 | 62.41 | 43.89 | 28.45 | 44.02 | 74.65 | 85.46 | 74.95 | 21.91 |
| Prussian carp | 4033 | 2 | 3 | 2 | 25.13 | 28.96 | 36.65 | 37.14 | 67.29 | 42.20 | 58.34 | 61.33 | 41.38 | 27.69 | 46.82 | 75.90 | 85.53 | 78.81 | 23.73 |
| Prussian carp | 4034 | 2 | 3 | 2 | 31.14 | 34.38 | 40.51 | 44.03 | 72.86 | 50.90 | 66.40 | 66.01 | 51.43 | 30.84 | 48.85 | 79.61 | 90.97 | 80.13 | 24.30 |
| Prussian carp | 1251 | 2 | 3 | 3 | 33.27 | 31.63 | 38.04 | 43.03 | 70.82 | 45.97 | 61.20 | 63.21 | 39.33 | 36.35 | 50.51 | 72.27 | 83.23 | 74.32 | 24.60 |
| Prussian carp | 1252 | 2 | 3 | 3 | 39.74 | 38.56 | 47.01 | 44.44 | 79.00 | 52.33 | 71.71 | 73.19 | 48.46 | 36.40 | 55.05 | 86.01 | 100.47 | 89.32 | 30.37 |
| Prussian carp | 1253 | 2 | 3 | 3 | 34.19 | 33.87 | 40.51 | 46.45 | 75.27 | 50.11 | 69.19 | 72.91 | 48.08 | 38.84 | 55.60 | 81.56 | 97.28 | 87.58 | 25.91 |
| Prussian carp | 1254 | 2 | 3 | 3 | 31.87 | 40.07 | 43.07 | 41.61 | 68.92 | 46.56 | 61.71 | 55.96 | 39.01 | 34.92 | 44.49 | 66.01 | 85.66 | 77.75 | 23.82 |
| Prussian carp | 1255 | 2 | 3 | 3 | 28.71 | 28.82 | 35.27 | 33.66 | 60.00 | 35.67 | 51.92 | 55.21 | 41.22 | 31.45 | 42.68 | 63.47 | 73.03 | 64.65 | 19.76 |
| Prussian carp | 1256 | 2 | 3 | 3 | 35.03 | 36.31 | 41.81 | 37.14 | 64.04 | 38.81 | 59.66 | 57.02 | 40.31 | 30.93 | 45.45 | 66.50 | 81.31 | 72.34 | 23.40 |
| Prussian carp | 1257 | 2 | 3 | 3 | 29.68 | 28.15 | 35.85 | 40.22 | 69.71 | 47.86 | 58.14 | 58.70 | 39.94 | 32.84 | 47.89 | 69.32 | 83.54 | 76.65 | 22.41 |
| Prussian carp | 1258 | 2 | 3 | 3 | 28.57 | 26.65 | 32.27 | 36.07 | 60.67 | 42.46 | 53.95 | 52.24 | 42.01 | 28.36 | 40.45 | 57.55 | 70.33 | 55.85 | 19.59 |
| Prussian carp | 1259 | 2 | 3 | 3 | 29.45 | 30.27 | 34.66 | 37.62 | 60.77 | 41.12 | 54.60 | 50.78 | 37.40 | 25.30 | 39.64 | 60.75 | 73.49 | 69.58 | 21.65 |
| Prussian carp | 1260 | 2 | 3 | 3 | 28.05 | 33.17 | 38.43 | 38.22 | 61.06 | 35.48 | 55.24 | 55.75 | 39.77 | 31.25 | 42.57 | 69.97 | 86.96 | 78.42 | 20.64 |
| Prussian carp | 1261 | 2 | 3 | 3 | 31.85 | 31.97 | 39.65 | 42.01 | 73.05 | 52.93 | 63.26 | 60.82 | 49.54 | 30.79 | 48.30 | 69.34 | 81.70 | 78.22 | 26.44 |
| Prussian carp | 1262 | 2 | 3 | 3 | 28.65 | 27.68 | 33.41 | 31.97 | 54.89 | 38.47 | 53.56 | 52.01 | 38.57 | 24.44 | 40.89 | 56.63 | 65.47 | 57.69 | 18.59 |
| Prussian carp | 1263 | 2 | 3 | 3 | 29.10 | 26.38 | 33.32 | 39.15 | 60.63 | 40.13 | 54.13 | 55.55 | 43.14 | 26.69 | 39.18 | 62.92 | 73.35 | 62.53 | 23.43 |
| Prussian carp | 1264 | 2 | 3 | 3 | 41.65 | 34.63 | 45.92 | 45.83 | 82.91 | 52.19 | 69.82 | 69.66 | 50.13 | 31.89 | 54.37 | 82.75 | 104.04 | 94.39 | 31.62 |
| Prussian carp | 1265 | 2 | 3 | 3 | 29.37 | 28.23 | 33.81 | 40.38 | 62.41 | 37.31 | 53.89 | 54.78 | 45.18 | 29.55 | 39.97 | 71.05 | 83.44 | 71.41 | 25.30 |
| Prussian carp | 1266 | 2 | 3 | 3 | 27.08 | 26.84 | 33.21 | 33.62 | 62.80 | 38.44 | 53.57 | 55.68 | 39.81 | 29.18 | 43.20 | 66.23 | 80.79 | 74.78 | 21.88 |
| Prussian carp | 1267 | 2 | 3 | 3 | 30.34 | 33.96 | 38.36 | 30.77 | 59.82 | 38.40 | 53.89 | 54.27 | 38.28 | 28.28 | 41.74 | 67.65 | 81.85 | 76.02 | 20.90 |
| Prussian carp | 1268 | 2 | 3 | 3 | 32.69 | 28.36 | 36.98 | 35.35 | 65.71 | 42.94 | 58.00 | 61.76 | 39.01 | 30.68 | 47.53 | 68.46 | 79.11 | 69.62 | 26.80 |
| Prussian carp | 1269 | 2 | 3 | 3 | 25.88 | 31.23 | 34.41 | 36.07 | 54.10 | 36.21 | 50.64 | 45.13 | 30.77 | 23.22 | 35.65 | 53.69 | 63.43 | 56.52 | 18.17 |
| Prussian carp | 1270 | 2 | 3 | 3 | 30.13 | 27.73 | 36.89 | 36.21 | 65.55 | 47.82 | 58.67 | 61.72 | 40.65 | 28.23 | 42.87 | 69.64 | 83.17 | 81.75 | 22.59 |
| Prussian carp | 1271 | 2 | 3 | 3 | 37.76 | 38.26 | 37.02 | 32.65 | 59.55 | 38.27 | 54.17 | 52.61 | 39.17 | 33.94 | 45.62 | 62.89 | 76.13 | 66.07 | 22.17 |
| Prussian carp | 1272 | 2 | 3 | 3 | 24.02 | 26.09 | 30.01 | 33.40 | 59.16 | 42.13 | 52.92 | 56.39 | 36.30 | 29.16 | 42.62 | 59.92 | 67.32 | 60.60 | 20.01 |
| Prussian carp | 1273 | 2 | 3 | 3 | 29.37 | 25.68 | 35.22 | 35.30 | 64.37 | 43.15 | 57.29 | 57.78 | 41.00 | 31.01 | 40.97 | 67.00 | 75.76 | 61.97 | 20.55 |
| Prussian carp | 1274 | 2 | 3 | 3 | 29.16 | 34.58 | 39.62 | 38.01 | 64.52 | 43.54 | 58.84 | 57.22 | 40.56 | 28.27 | 39.93 | 64.23 | 74.90 | 65.44 | 24.02 |
| Prussian carp | 1275 | 2 | 3 | 3 | 35.10 | 33.21 | 41.37 | 34.25 | 70.32 | 53.15 | 62.89 | 63.86 | 41.70 | 35.16 | 52.50 | 71.27 | 89.00 | 81.74 | 25.21 |
| Prussian carp | 4030 | 2 | 3 | 3 | 32.21 | 34.58 | 42.09 | 40.62 | 72.81 | 48.70 | 66.02 | 62.59 | 42.19 | 33.49 | 47.54 | 72.11 | 92.22 | 82.60 | 28.43 |
| Prussian carp | 4031 | 2 | 3 | 3 | 33.01 | 32.35 | 35.48 | 31.61 | 58.56 | 39.32 | 52.09 | 55.20 | 41.08 | 23.35 | 39.60 | 64.67 | 77.44 | 68.04 | 22.40 |
| Prussian carp | 4032 | 2 | 3 | 3 | 27.46 | 28.01 | 34.89 | 44.17 | 70.39 | 50.05 | 62.23 | 65.71 | 40.64 | 31.82 | 50.65 | 73.24 | 80.43 | 73.16 | 24.42 |
| Prussian carp | 4033 | 2 | 3 | 3 | 29.26 | 28.86 | 32.97 | 37.51 | 66.40 | 36.20 | 56.65 | 62.19 | 38.87 | 26.50 | 44.29 | 73.63 | 77.48 | 70.15 | 23.11 |
| Prussian carp | 4034 | 2 | 3 | 3 | 33.90 | 37.88 | 45.65 | 40.49 | 73.91 | 49.37 | 68.25 | 66.35 | 52.66 | 28.51 | 50.48 | 81.36 | 87.47 | 78.15 | 26.81 |
| Prussian carp | 1061 | 3 | 1 | 1 | 29.33 | 27.38 | 31.57 | 33.71 | 54.84 | 35.55 | 48.48 | 48.04 | 34.86 | 30.13 | 41.35 | 56.71 | 69.47 | 61.36 | 19.82 |
| Prussian carp | 1062 | 3 | 1 | 1 | 31.04 | 33.27 | 35.77 | 37.60 | 59.62 | 37.67 | 54.30 | 53.37 | 39.01 | 32.66 | 46.33 | 66.76 | 83.97 | 75.30 | 23.24 |
| Prussian carp | 1063 | 3 | 1 | 1 | 27.93 | 27.90 | 33.81 | 36.83 | 60.04 | 41.05 | 54.17 | 51.61 | 36.46 | 29.94 | 42.32 | 61.38 | 75.06 | 67.29 | 20.40 |
| Prussian carp | 1064 | 3 | 1 | 1 | 27.44 | 29.28 | 33.85 | 35.35 | 57.11 | 37.57 | 53.84 | 52.74 | 34.35 | 28.41 | 41.45 | 64.42 | 77.88 | 69.86 | 21.15 |
| Prussian carp | 1065 | 3 | 1 | 1 | 25.76 | 28.00 | 31.95 | 34.67 | 55.90 | 37.37 | 50.49 | 50.01 | 32.79 | 25.04 | 37.17 | 57.76 | 67.74 | 59.66 | 19.35 |
| Prussian carp | 1066 | 3 | 1 | 1 | 30.20 | 29.26 | 33.74 | 32.80 | 55.72 | 37.67 | 51.68 | 49.06 | 36.33 | 30.01 | 41.05 | 60.19 | 72.74 | 63.31 | 20.15 |
| Prussian carp | 1067 | 3 | 1 | 1 | 28.17 | 31.07 | 37.66 | 38.87 | 66.83 | 43.54 | 58.85 | 61.24 | 39.28 | 30.20 | 43.35 | 68.11 | 80.18 | 72.07 | 21.34 |
| Prussian carp | 1068 | 3 | 1 | 1 | 29.28 | 32.03 | 36.05 | 38.25 | 53.87 | 41.31 | 55.33 | 53.87 | 39.09 | 29.68 | 43.33 | 66.13 | 78.73 | 69.04 | 22.45 |
| Prussian carp | 1069 | 3 | 1 | 1 | 26.53 | 24.68 | 31.53 | 30.46 | 51.19 | 32.76 | 47.98 | 46.22 | 33.66 | 26.00 | 36.36 | 55.48 | 68.13 | 59.08 | 18.91 |
| Prussian carp | 1070 | 3 | 1 | 1 | 23.72 | 26.10 | 31.98 | 31.53 | 54.85 | 34.50 | 49.07 | 50.89 | 33.29 | 31.38 | 41.87 | 54.85 | 65.64 | 55.90 | 19.07 |
| Prussian carp | 1071 | 3 | 1 | 1 | 27.28 | 27.87 | 34.83 | 39.33 | 64.04 | 42.26 | 58.44 | 56.81 | 38.01 | 27.45 | 42.21 | 68.59 | 79.13 | 69.52 | 23.03 |
| Prussian carp | 1072 | 3 | 1 | 1 | 31.86 | 33.84 | 38.87 | 40.25 | 66.64 | 44.53 | 62.14 | 58.57 | 42.70 | 27.53 | 44.05 | 72.55 | 85.30 | 74.26 | 24.81 |
| Prussian carp | 1073 | 3 | 1 | 1 | 23.95 | 25.03 | 29.41 | 30.10 | 54.56 | 38.70 | 43.82 | 43.06 | 31.33 | 25.43 | 34.97 | 47.81 | 56.96 | 48.18 | 17.99 |
| Prussian carp | 1074 | 3 | 1 | 1 | 30.92 | 29.38 | 34.07 | 34.70 | 61.75 | 41.20 | 54.05 | 54.31 | 38.46 | 33.55 | 44.81 | 64.20 | 77.52 | 68.64 | 21.60 |
| Prussian carp | 1075 | 3 | 1 | 1 | 26.58 | 27.30 | 33.93 | 38.83 | 63.74 | 42.15 | 55.95 | 56.14 | 37.23 | 26.90 | 40.45 | 64.83 | 75.51 | 64.29 | 23.32 |
| Prussian carp | 1076 | 3 | 1 | 1 | 23.91 | 27.70 | 31.67 | 39.39 | 62.56 | 41.40 | 53.20 | 53.72 | 32.76 | 27.03 | 40.97 | 59.89 | 71.66 | 64.16 | 20.24 |
| Prussian carp | 1077 | 3 | 1 | 1 | 28.90 | 32.83 | 36.16 | 42.46 | 66.10 | 42.48 | 60.25 | 58.81 | 40.66 | 32.50 | 47.83 | 68.33 | 81.23 | 69.51 | 24.30 |
| Prussian carp | 1078 | 3 | 1 | 1 | 28.35 | 23.09 | 32.80 | 35.56 | 59.59 | 41.42 | 52.83 | 52.66 | 31.59 | 25.91 | 39.61 | 56.55 | 65.06 | 55.93 | 19.81 |
| Prussian carp | 1079 | 3 | 1 | 1 | 31.86 | 27.52 | 35.41 | 34.52 | 61.41 | 38.14 | 54.57 | 56.28 | 39.90 | 27.38 | 41.88 | 64.62 | 74.02 | 62.90 | 21.45 |
| Prussian carp | 1080 | 3 | 1 | 1 | 31.58 | 32.40 | 37.20 | 36.66 | 62.53 | 40.81 | 54.69 | 54.01 | 40.39 | 29.79 | 42.12 | 64.20 | 76.06 | 63.85 | 21.59 |
| Prussian carp | 1081 | 3 | 1 | 1 | 26.92 | 29.50 | 33.80 | 31.77 | 56.17 | 34.97 | 49.69 | 50.27 | 35.97 | 28.41 | 37.90 | 58.48 | 68.38 | 58.29 | 18.55 |
| Prussian carp | 1082 | 3 | 1 | 1 | 27.66 | 28.57 | 33.39 | 32.13 | 52.90 | 33.91 | 49.13 | 46.10 | 34.50 | 28.78 | 37.91 | 57.79 | 70.64 | 63.41 | 18.33 |
| Prussian carp | 1083 | 3 | 1 | 1 | 24.72 | 27.16 | 31.22 | 33.39 | 55.38 | 35.25 | 47.85 | 47.95 | 32.29 | 24.66 | 37.50 | 56.60 | 66.74 | 58.94 | 20.55 |
| Prussian carp | 1084 | 3 | 1 | 1 | 28.31 | 29.08 | 35.05 | 34.96 | 63.63 | 44.62 | 52.46 | 52.61 | 35.49 | 26.40 | 38.92 | 61.43 | 73.26 | 66.08 | 19.37 |
| Prussian carp | 1085 | 3 | 1 | 1 | 32.49 | 32.09 | 38.17 | 40.74 | 70.01 | 49.44 | 60.82 | 60.44 | 45.93 | 38.35 | 50.74 | 71.49 | 87.80 | 74.92 | 25.58 |
| Prussian carp | 1086 | 3 | 1 | 1 | 28.90 | 30.74 | 34.90 | 34.24 | 56.71 | 36.66 | 52.21 | 49.51 | 36.38 | 26.27 | 38.54 | 61.53 | 74.87 | 63.89 | 23.02 |
| Prussian carp | 1087 | 3 | 1 | 1 | 24.35 | 27.90 | 33.37 | 34.76 | 59.22 | 38.64 | 52.30 | 52.59 | 34.01 | 27.01 | 39.15 | 60.48 | 72.80 | 65.64 | 19.05 |
| Prussian carp | 1088 | 3 | 1 | 1 | 22.97 | 23.01 | 27.30 | 29.42 | 46.85 | 29.38 | 43.07 | 42.42 | 28.16 | 24.33 | 33.18 | 49.36 | 58.96 | 53.49 | 16.35 |
| Prussian carp | 1089 | 3 | 1 | 1 | 28.52 | 31.41 | 35.71 | 40.69 | 62.41 | 40.51 | 57.49 | 56.68 | 36.60 | 30.98 | 45.76 | 67.40 | 80.60 | 76.03 | 20.06 |
| Prussian carp | 1090 | 3 | 1 | 1 | 23.22 | 25.73 | 29.58 | 35.69 | 51.68 | 34.04 | 48.81 | 45.80 | 30.91 | 23.71 | 34.16 | 56.34 | 67.51 | 60.19 | 17.40 |
| Prussian carp | 1061 | 3 | 1 | 2 | 29.49 | 28.90 | 34.11 | 28.82 | 53.74 | 35.22 | 48.58 | 47.90 | 34.84 | 29.00 | 40.07 | 56.51 | 69.07 | 60.40 | 19.66 |
| Prussian carp | 1062 | 3 | 1 | 2 | 30.28 | 32.39 | 35.78 | 35.40 | 58.32 | 36.50 | 54.29 | 52.68 | 39.97 | 33.01 | 46.37 | 67.61 | 84.60 | 76.07 | 23.37 |
| Prussian carp | 1063 | 3 | 1 | 2 | 28.57 | 26.74 | 32.88 | 37.02 | 59.65 | 41.13 | 53.89 | 51.76 | 36.82 | 31.12 | 42.46 | 61.42 | 76.18 | 67.75 | 20.70 |
| Prussian carp | 1064 | 3 | 1 | 2 | 26.91 | 29.93 | 33.70 | 36.12 | 58.49 | 38.64 | 53.70 | 52.36 | 34.11 | 29.52 | 41.79 | 63.15 | 78.51 | 69.89 | 21.15 |
| Prussian carp | 1065 | 3 | 1 | 2 | 26.63 | 28.63 | 32.25 | 33.82 | 55.35 | 35.30 | 50.19 | 49.71 | 32.85 | 25.37 | 37.69 | 58.54 | 69.03 | 60.03 | 19.65 |
| Prussian carp | 1066 | 3 | 1 | 2 | 29.61 | 30.58 | 33.91 | 34.39 | 55.69 | 37.29 | 52.44 | 49.21 | 36.09 | 30.40 | 40.51 | 59.91 | 74.45 | 63.86 | 20.54 |
| Prussian carp | 1067 | 3 | 1 | 2 | 28.47 | 31.07 | 36.79 | 39.63 | 66.19 | 42.29 | 59.21 | 61.21 | 39.32 | 29.42 | 43.88 | 68.47 | 79.93 | 72.63 | 21.37 |
| Prussian carp | 1068 | 3 | 1 | 2 | 28.89 | 31.13 | 36.20 | 38.01 | 63.93 | 42.29 | 54.97 | 54.19 | 40.29 | 29.25 | 42.31 | 66.23 | 79.48 | 69.57 | 23.13 |
| Prussian carp | 1069 | 3 | 1 | 2 | 26.45 | 25.99 | 31.95 | 30.78 | 51.55 | 32.87 | 48.08 | 46.22 | 32.75 | 27.37 | 36.82 | 54.58 | 66.90 | 57.81 | 19.05 |
| Prussian carp | 1070 | 3 | 1 | 2 | 23.73 | 26.54 | 32.05 | 30.47 | 54.70 | 34.44 | 49.32 | 51.24 | 33.18 | 31.80 | 42.27 | 55.57 | 67.74 | 58.43 | 19.47 |
| Prussian carp | 1071 | 3 | 1 | 2 | 27.84 | 29.03 | 35.86 | 39.26 | 63.14 | 42.23 | 57.63 | 56.71 | 39.76 | 27.97 | 42.58 | 68.55 | 79.45 | 68.93 | 23.25 |
| Prussian carp | 1072 | 3 | 1 | 2 | 33.53 | 36.23 | 40.70 | 40.12 | 66.20 | 45.21 | 63.19 | 58.86 | 42.56 | 27.52 | 43.51 | 73.30 | 86.40 | 77.11 | 24.90 |
| Prussian carp | 1073 | 3 | 1 | 2 | 24.28 | 24.90 | 29.11 | 30.02 | 53.62 | 37.46 | 43.93 | 42.60 | 32.08 | 24.69 | 34.71 | 46.29 | 54.62 | 46.07 | 18.03 |
| Prussian carp | 1074 | 3 | 1 | 2 | 29.93 | 29.96 | 34.51 | 34.43 | 61.99 | 41.18 | 53.18 | 54.46 | 39.37 | 33.33 | 45.32 | 65.17 | 81.00 | 70.55 | 21.53 |
| Prussian carp | 1075 | 3 | 1 | 2 | 29.09 | 30.26 | 36.45 | 36.68 | 63.42 | 42.30 | 54.95 | 56.41 | 39.82 | 27.51 | 41.25 | 65.39 | 76.36 | 64.51 | 22.74 |
| Prussian carp | 1076 | 3 | 1 | 2 | 24.29 | 28.35 | 32.85 | 36.92 | 61.62 | 42.27 | 53.36 | 54.23 | 33.20 | 27.78 | 40.03 | 60.86 | 72.78 | 64.69 | 20.12 |
| Prussian carp | 1077 | 3 | 1 | 2 | 28.69 | 33.32 | 37.53 | 38.85 | 67.39 | 44.55 | 58.27 | 58.37 | 43.23 | 33.45 | 47.03 | 71.29 | 86.76 | 76.53 | 24.46 |
| Prussian carp | 1078 | 3 | 1 | 2 | 28.05 | 23.58 | 33.29 | 33.01 | 58.60 | 41.51 | 52.08 | 52.91 | 32.97 | 25.97 | 39.76 | 57.80 | 66.41 | 57.31 | 20.55 |
| Prussian carp | 1079 | 3 | 1 | 2 | 30.27 | 27.13 | 36.21 | 33.01 | 61.40 | 41.05 | 54.97 | 56.46 | 40.99 | 26.84 | 40.66 | 64.90 | 75.50 | 63.67 | 22.42 |
| Prussian carp | 1080 | 3 | 1 | 2 | 31.16 | 31.53 | 36.32 | 35.24 | 61.56 | 40.31 | 54.24 | 53.86 | 40.51 | 30.06 | 41.22 | 64.45 | 76.59 | 64.97 | 22.79 |
| Prussian carp | 1081 | 3 | 1 | 2 | 29.07 | 30.50 | 34.83 | 31.13 | 55.73 | 33.40 | 49.45 | 49.97 | 36.13 | 28.15 | 39.07 | 58.72 | 68.70 | 58.94 | 18.65 |
| Prussian carp | 1082 | 3 | 1 | 2 | 28.14 | 29.29 | 34.35 | 29.86 | 52.81 | 34.26 | 48.59 | 46.36 | 35.26 | 27.62 | 37.87 | 58.01 | 71.65 | 63.67 | 19.30 |
| Prussian carp | 1083 | 3 | 1 | 2 | 26.73 | 26.62 | 30.80 | 32.47 | 54.80 | 35.16 | 47.54 | 47.84 | 33.35 | 24.11 | 36.11 | 57.43 | 68.00 | 59.23 | 20.58 |
| Prussian carp | 1084 | 3 | 1 | 2 | 29.72 | 27.05 | 32.95 | 36.00 | 60.38 | 39.49 | 51.83 | 52.05 | 35.32 | 26.83 | 39.70 | 61.87 | 74.79 | 68.15 | 19.80 |
| Prussian carp | 1085 | 3 | 1 | 2 | 32.04 | 32.10 | 37.90 | 41.07 | 71.37 | 50.84 | 61.00 | 61.02 | 46.98 | 36.39 | 50.14 | 72.84 | 89.79 | 78.10 | 24.85 |
| Prussian carp | 1086 | 3 | 1 | 2 | 29.03 | 29.76 | 34.17 | 35.13 | 58.13 | 38.64 | 51.92 | 48.76 | 36.51 | 25.99 | 39.54 | 62.16 | 74.95 | 65.21 | 22.68 |
| Prussian carp | 1087 | 3 | 1 | 2 | 25.13 | 27.37 | 33.65 | 34.68 | 58.25 | 36.09 | 52.19 | 53.72 | 35.72 | 26.10 | 38.11 | 61.36 | 73.07 | 65.82 | 19.20 |
| Prussian carp | 1088 | 3 | 1 | 2 | 23.39 | 23.15 | 27.71 | 29.01 | 46.82 | 29.43 | 42.97 | 42.57 | 28.56 | 22.48 | 32.27 | 49.84 | 60.36 | 54.48 | 16.33 |
| Prussian carp | 1089 | 3 | 1 | 2 | 28.18 | 31.01 | 35.84 | 38.58 | 62.38 | 39.49 | 56.80 | 56.65 | 37.54 | 31.08 | 44.96 | 67.47 | 81.19 | 77.17 | 19.95 |
| Prussian carp | 1090 | 3 | 1 | 2 | 25.24 | 25.73 | 30.09 | 34.17 | 50.52 | 31.99 | 49.16 | 46.25 | 30.07 | 23.85 | 33.91 | 56.22 | 68.51 | 60.66 | 18.05 |
| Prussian carp | 1061 | 3 | 1 | 3 | 27.96 | 28.04 | 32.92 | 31.44 | 54.55 | 34.88 | 48.23 | 47.84 | 34.37 | 30.60 | 40.53 | 55.70 | 68.82 | 60.28 | 19.55 |
| Prussian carp | 1062 | 3 | 1 | 3 | 30.08 | 31.72 | 35.46 | 35.86 | 59.12 | 37.85 | 54.30 | 53.07 | 39.38 | 33.49 | 46.95 | 66.51 | 84.33 | 75.04 | 23.89 |
| Prussian carp | 1063 | 3 | 1 | 3 | 27.22 | 26.97 | 33.07 | 36.53 | 58.79 | 40.23 | 53.89 | 51.91 | 37.28 | 30.41 | 41.86 | 62.08 | 76.98 | 68.29 | 21.01 |
| Prussian carp | 1064 | 3 | 1 | 3 | 26.77 | 29.49 | 33.77 | 36.69 | 58.27 | 39.18 | 53.73 | 52.73 | 34.86 | 29.73 | 41.82 | 63.50 | 77.72 | 69.53 | 21.15 |
| Prussian carp | 1065 | 3 | 1 | 3 | 24.86 | 27.36 | 31.94 | 32.81 | 54.92 | 35.46 | 49.94 | 49.57 | 33.60 | 24.67 | 36.90 | 58.54 | 69.10 | 61.02 | 19.31 |
| Prussian carp | 1066 | 3 | 1 | 3 | 28.87 | 28.68 | 32.76 | 34.70 | 55.61 | 36.69 | 51.74 | 49.01 | 36.46 | 30.80 | 40.73 | 60.00 | 73.98 | 63.21 | 20.47 |
| Prussian carp | 1067 | 3 | 1 | 3 | 27.37 | 30.05 | 36.01 | 39.66 | 66.45 | 43.13 | 58.01 | 60.62 | 39.89 | 31.58 | 43.88 | 68.13 | 82.05 | 72.23 | 21.80 |
| Prussian carp | 1068 | 3 | 1 | 3 | 29.13 | 30.89 | 35.67 | 37.09 | 62.81 | 42.13 | 54.33 | 53.85 | 41.07 | 29.71 | 42.72 | 66.36 | 80.46 | 69.37 | 22.60 |
| Prussian carp | 1069 | 3 | 1 | 3 | 25.73 | 25.12 | 31.20 | 31.50 | 51.31 | 31.60 | 48.12 | 46.31 | 33.45 | 26.36 | 36.68 | 55.17 | 67.53 | 58.05 | 19.74 |
| Prussian carp | 1070 | 3 | 1 | 3 | 24.26 | 25.99 | 32.04 | 30.91 | 53.78 | 33.82 | 48.18 | 50.59 | 32.79 | 31.84 | 42.03 | 54.70 | 66.50 | 56.65 | 19.36 |
| Prussian carp | 1071 | 3 | 1 | 3 | 27.12 | 29.75 | 34.98 | 40.90 | 63.21 | 43.10 | 57.61 | 56.71 | 39.76 | 28.11 | 43.04 | 68.04 | 79.99 | 68.69 | 23.40 |
| Prussian carp | 1072 | 3 | 1 | 3 | 32.45 | 33.99 | 39.50 | 39.19 | 65.13 | 44.83 | 62.46 | 58.17 | 42.30 | 28.23 | 42.81 | 72.92 | 86.04 | 75.67 | 24.81 |
| Prussian carp | 1073 | 3 | 1 | 3 | 24.12 | 25.43 | 29.63 | 31.12 | 52.94 | 35.27 | 43.92 | 42.49 | 31.52 | 24.74 | 34.80 | 48.71 | 58.15 | 49.74 | 18.19 |
| Prussian carp | 1074 | 3 | 1 | 3 | 30.64 | 29.67 | 34.66 | 34.65 | 60.43 | 39.53 | 52.95 | 54.31 | 40.88 | 32.52 | 44.20 | 65.93 | 81.08 | 72.00 | 21.53 |
| Prussian carp | 1075 | 3 | 1 | 3 | 26.86 | 28.46 | 35.04 | 36.65 | 62.80 | 41.97 | 54.74 | 56.01 | 39.12 | 27.91 | 41.56 | 65.14 | 76.08 | 64.91 | 23.02 |
| Prussian carp | 1076 | 3 | 1 | 3 | 24.79 | 29.42 | 33.23 | 37.58 | 62.83 | 42.85 | 52.71 | 54.15 | 33.15 | 27.67 | 40.93 | 60.73 | 73.70 | 65.73 | 19.82 |
| Prussian carp | 1077 | 3 | 1 | 3 | 28.44 | 34.26 | 38.21 | 40.44 | 66.37 | 43.46 | 58.70 | 58.01 | 43.01 | 32.01 | 45.84 | 72.75 | 88.03 | 77.92 | 24.82 |
| Prussian carp | 1078 | 3 | 1 | 3 | 27.91 | 24.02 | 33.07 | 33.05 | 58.56 | 41.29 | 52.72 | 52.96 | 32.10 | 26.32 | 39.21 | 57.80 | 66.33 | 57.50 | 20.26 |
| Prussian carp | 1079 | 3 | 1 | 3 | 30.59 | 26.80 | 35.89 | 33.92 | 61.93 | 41.05 | 55.05 | 56.00 | 40.05 | 28.38 | 41.15 | 64.67 | 74.33 | 63.12 | 21.53 |
| Prussian carp | 1080 | 3 | 1 | 3 | 30.57 | 32.44 | 36.20 | 35.53 | 61.46 | 39.52 | 53.82 | 54.42 | 40.60 | 31.13 | 42.62 | 64.32 | 77.04 | 65.09 | 22.89 |
| Prussian carp | 1081 | 3 | 1 | 3 | 28.68 | 29.79 | 34.82 | 31.45 | 55.53 | 33.02 | 49.42 | 50.12 | 35.94 | 28.36 | 38.74 | 58.99 | 68.61 | 59.31 | 18.16 |
| Prussian carp | 1082 | 3 | 1 | 3 | 28.43 | 29.72 | 34.31 | 31.08 | 53.36 | 32.79 | 48.48 | 46.57 | 33.91 | 28.25 | 37.63 | 57.59 | 71.99 | 63.26 | 19.02 |
| Prussian carp | 1083 | 3 | 1 | 3 | 25.81 | 27.34 | 31.38 | 32.63 | 54.55 | 33.83 | 47.52 | 47.81 | 33.22 | 24.19 | 36.62 | 57.18 | 68.03 | 59.85 | 20.26 |
| Prussian carp | 1084 | 3 | 1 | 3 | 29.98 | 28.73 | 34.60 | 33.21 | 59.69 | 40.26 | 52.45 | 52.41 | 34.49 | 27.19 | 39.98 | 60.73 | 74.85 | 67.97 | 19.82 |
| Prussian carp | 1085 | 3 | 1 | 3 | 32.25 | 31.98 | 37.61 | 41.31 | 69.01 | 48.22 | 61.65 | 60.76 | 47.12 | 36.71 | 50.68 | 73.12 | 89.73 | 77.45 | 25.46 |
| Prussian carp | 1086 | 3 | 1 | 3 | 29.40 | 29.97 | 33.87 | 34.83 | 58.50 | 39.31 | 51.90 | 49.35 | 37.46 | 26.44 | 39.59 | 61.76 | 74.86 | 66.18 | 23.25 |
| Prussian carp | 1087 | 3 | 1 | 3 | 25.47 | 27.16 | 33.19 | 34.84 | 58.97 | 37.91 | 52.08 | 53.24 | 35.87 | 25.71 | 38.42 | 60.97 | 72.53 | 65.38 | 19.20 |
| Prussian carp | 1088 | 3 | 1 | 3 | 22.38 | 23.48 | 28.36 | 28.99 | 47.43 | 30.04 | 42.76 | 42.43 | 29.01 | 23.58 | 32.83 | 50.14 | 60.98 | 54.25 | 16.05 |
| Prussian carp | 1089 | 3 | 1 | 3 | 27.73 | 32.13 | 35.98 | 39.03 | 61.14 | 38.14 | 56.30 | 56.07 | 37.59 | 31.69 | 46.12 | 66.66 | 80.63 | 76.70 | 19.92 |
| Prussian carp | 1090 | 3 | 1 | 3 | 24.10 | 25.49 | 30.06 | 32.93 | 49.84 | 31.83 | 49.38 | 46.22 | 29.82 | 23.38 | 34.21 | 56.49 | 68.53 | 61.00 | 18.10 |
| Prussian carp | 1161 | 3 | 2 | 1 | 42.82 | 42.96 | 49.20 | 50.08 | 83.90 | 56.40 | 74.96 | 71.77 | 50.12 | 39.61 | 57.45 | 85.03 | 104.60 | 91.74 | 30.00 |
| Prussian carp | 1162 | 3 | 2 | 1 | 42.93 | 40.35 | 46.83 | 49.17 | 76.54 | 52.61 | 74.11 | 66.26 | 49.84 | 40.49 | 55.28 | 80.60 | 102.85 | 91.58 | 29.40 |
| Prussian carp | 1163 | 3 | 2 | 1 | 39.67 | 39.83 | 47.83 | 49.50 | 76.41 | 52.45 | 75.39 | 66.69 | 49.70 | 37.06 | 51.80 | 83.27 | 105.38 | 93.00 | 28.02 |
| Prussian carp | 1164 | 3 | 2 | 1 | 29.29 | 29.67 | 33.69 | 31.15 | 55.27 | 36.43 | 50.75 | 49.21 | 30.76 | 28.29 | 37.46 | 54.81 | 69.38 | 61.58 | 17.70 |
| Prussian carp | 1165 | 3 | 2 | 1 | 36.47 | 36.32 | 43.07 | 42.09 | 70.43 | 42.76 | 66.40 | 66.18 | 43.90 | 39.71 | 52.76 | 77.50 | 95.46 | 84.79 | 26.89 |
| Prussian carp | 1166 | 3 | 2 | 1 | 33.90 | 39.34 | 40.60 | 42.97 | 67.72 | 48.21 | 62.30 | 56.21 | 45.86 | 34.73 | 47.24 | 68.59 | 87.86 | 80.21 | 22.69 |
| Prussian carp | 1167 | 3 | 2 | 1 | 45.94 | 44.89 | 52.91 | 51.47 | 89.98 | 62.14 | 82.15 | 74.90 | 50.01 | 42.35 | 60.93 | 82.28 | 98.19 | 83.71 | 30.00 |
| Prussian carp | 1168 | 3 | 2 | 1 | 39.46 | 35.77 | 41.82 | 44.88 | 71.99 | 48.52 | 67.48 | 63.50 | 50.08 | 37.51 | 53.57 | 80.61 | 101.38 | 90.79 | 27.60 |
| Prussian carp | 1169 | 3 | 2 | 1 | 24.59 | 22.47 | 26.97 | 27.77 | 47.89 | 33.92 | 43.51 | 42.17 | 28.93 | 20.28 | 29.50 | 47.99 | 56.72 | 49.91 | 16.22 |
| Prussian carp | 1170 | 3 | 2 | 1 | 38.09 | 42.11 | 46.25 | 48.14 | 80.42 | 48.97 | 69.33 | 69.08 | 48.26 | 36.90 | 54.35 | 81.34 | 97.97 | 84.96 | 28.40 |
| Prussian carp | 1171 | 3 | 2 | 1 | 24.31 | 24.39 | 27.79 | 28.68 | 46.67 | 30.52 | 41.73 | 38.80 | 28.28 | 20.56 | 29.67 | 46.95 | 56.31 | 48.73 | 15.82 |
| Prussian carp | 1172 | 3 | 2 | 1 | 25.25 | 26.35 | 27.71 | 26.12 | 43.89 | 28.94 | 40.90 | 39.20 | 27.43 | 20.98 | 29.70 | 45.26 | 57.26 | 50.73 | 15.90 |
| Prussian carp | 1173 | 3 | 2 | 1 | 24.78 | 22.44 | 25.70 | 25.27 | 45.17 | 32.71 | 40.73 | 38.80 | 28.68 | 25.83 | 32.14 | 43.53 | 57.53 | 49.38 | 16.50 |
| Prussian carp | 1174 | 3 | 2 | 1 | 21.81 | 21.39 | 23.81 | 21.60 | 38.54 | 25.60 | 35.81 | 35.67 | 23.03 | 19.29 | 26.69 | 38.87 | 47.89 | 42.12 | 12.63 |
| Prussian carp | 1175 | 3 | 2 | 1 | 19.60 | 20.84 | 22.43 | 23.84 | 35.96 | 22.42 | 34.26 | 32.41 | 22.13 | 17.55 | 24.64 | 38.03 | 46.93 | 40.61 | 12.90 |
| Prussian carp | 1176 | 3 | 2 | 1 | 22.70 | 24.51 | 26.71 | 27.87 | 44.70 | 29.15 | 42.00 | 40.62 | 27.16 | 24.15 | 32.17 | 44.06 | 52.64 | 46.29 | 13.58 |
| Prussian carp | 1177 | 3 | 2 | 1 | 22.66 | 22.16 | 24.54 | 26.74 | 41.55 | 27.75 | 38.76 | 36.61 | 24.18 | 21.87 | 28.74 | 41.31 | 50.61 | 41.92 | 14.32 |
| Prussian carp | 1178 | 3 | 2 | 1 | 20.97 | 19.42 | 22.44 | 26.20 | 37.88 | 26.64 | 37.97 | 33.09 | 20.77 | 17.82 | 25.76 | 37.30 | 45.50 | 41.19 | 12.60 |
| Prussian carp | 1179 | 3 | 2 | 1 | 19.77 | 20.43 | 21.32 | 21.75 | 33.47 | 20.98 | 32.90 | 30.16 | 18.83 | 19.30 | 25.04 | 34.81 | 45.66 | 40.45 | 11.78 |
| Prussian carp | 1180 | 3 | 2 | 1 | 21.51 | 19.79 | 22.31 | 22.85 | 35.38 | 23.46 | 35.97 | 33.04 | 22.17 | 19.39 | 26.46 | 36.34 | 44.31 | 39.02 | 12.23 |
| Prussian carp | 1181 | 3 | 2 | 1 | 19.53 | 20.33 | 21.46 | 22.14 | 35.33 | 23.62 | 33.45 | 31.73 | 21.84 | 18.08 | 24.05 | 36.35 | 44.69 | 38.63 | 12.40 |
| Prussian carp | 1182 | 3 | 2 | 1 | 26.65 | 26.84 | 28.27 | 30.59 | 44.50 | 28.49 | 42.21 | 36.75 | 29.81 | 22.32 | 31.14 | 46.12 | 57.96 | 51.25 | 16.08 |
| Prussian carp | 1183 | 3 | 2 | 1 | 25.74 | 23.97 | 28.18 | 26.99 | 47.54 | 32.90 | 42.48 | 41.36 | 31.23 | 22.01 | 31.19 | 46.84 | 57.50 | 49.92 | 17.13 |
| Prussian carp | 1184 | 3 | 2 | 1 | 29.26 | 26.14 | 30.81 | 33.68 | 51.54 | 36.39 | 50.40 | 44.89 | 31.99 | 25.83 | 36.52 | 53.11 | 64.08 | 56.40 | 18.34 |
| Prussian carp | 1185 | 3 | 2 | 1 | 23.46 | 21.93 | 25.43 | 29.38 | 42.39 | 28.40 | 41.66 | 35.72 | 24.12 | 16.19 | 26.02 | 44.25 | 51.02 | 44.26 | 14.30 |
| Prussian carp | 1186 | 3 | 2 | 1 | 32.20 | 32.29 | 34.99 | 30.36 | 53.49 | 35.93 | 50.71 | 47.64 | 35.77 | 25.77 | 37.04 | 60.17 | 70.60 | 62.54 | 19.75 |
| Prussian carp | 1187 | 3 | 2 | 1 | 25.47 | 23.61 | 26.65 | 28.12 | 42.08 | 28.91 | 42.29 | 36.66 | 23.92 | 20.02 | 28.37 | 43.76 | 55.15 | 48.23 | 15.61 |
| Prussian carp | 1188 | 3 | 2 | 1 | 22.54 | 21.61 | 26.25 | 28.02 | 46.73 | 33.57 | 41.06 | 39.62 | 28.46 | 17.49 | 28.70 | 43.74 | 48.84 | 44.67 | 14.90 |
| Prussian carp | 1189 | 3 | 2 | 1 | 29.18 | 27.09 | 31.84 | 32.40 | 53.41 | 35.86 | 49.46 | 47.15 | 29.72 | 26.16 | 37.00 | 53.02 | 65.40 | 57.97 | 18.30 |
| Prussian carp | 1190 | 3 | 2 | 1 | 21.96 | 23.50 | 24.41 | 26.23 | 40.36 | 26.38 | 37.74 | 33.24 | 24.38 | 17.92 | 26.79 | 39.81 | 49.87 | 45.43 | 14.41 |
| Prussian carp | 1161 | 3 | 2 | 2 | 42.43 | 40.75 | 48.60 | 48.86 | 82.22 | 53.15 | 75.55 | 72.83 | 50.76 | 39.54 | 56.09 | 85.95 | 105.39 | 93.68 | 29.61 |
| Prussian carp | 1162 | 3 | 2 | 2 | 42.69 | 40.56 | 46.62 | 50.59 | 78.29 | 52.12 | 72.17 | 66.61 | 50.89 | 38.71 | 55.37 | 79.49 | 100.11 | 91.10 | 29.42 |
| Prussian carp | 1163 | 3 | 2 | 2 | 39.37 | 39.57 | 47.89 | 50.61 | 77.29 | 52.03 | 75.95 | 66.36 | 47.15 | 40.84 | 57.50 | 82.56 | 106.46 | 95.86 | 30.02 |
| Prussian carp | 1164 | 3 | 2 | 2 | 29.60 | 30.28 | 33.82 | 32.18 | 56.25 | 37.51 | 49.81 | 49.02 | 31.54 | 28.05 | 37.67 | 53.99 | 69.27 | 62.18 | 17.81 |
| Prussian carp | 1165 | 3 | 2 | 2 | 35.41 | 36.62 | 42.28 | 42.69 | 71.12 | 44.54 | 66.09 | 65.80 | 45.12 | 40.64 | 53.76 | 77.84 | 98.85 | 86.31 | 26.46 |
| Prussian carp | 1166 | 3 | 2 | 2 | 33.57 | 37.80 | 40.02 | 42.18 | 67.32 | 47.40 | 62.97 | 57.41 | 46.52 | 33.84 | 46.97 | 70.21 | 88.83 | 80.49 | 22.46 |
| Prussian carp | 1167 | 3 | 2 | 2 | 45.88 | 46.49 | 52.85 | 51.74 | 90.13 | 61.08 | 79.56 | 74.65 | 53.71 | 42.68 | 61.16 | 85.10 | 101.99 | 88.64 | 31.63 |
| Prussian carp | 1168 | 3 | 2 | 2 | 38.35 | 38.09 | 42.70 | 44.69 | 72.50 | 49.22 | 67.33 | 63.24 | 50.08 | 36.71 | 53.18 | 80.35 | 100.67 | 90.60 | 28.00 |
| Prussian carp | 1169 | 3 | 2 | 2 | 24.22 | 21.90 | 26.53 | 28.86 | 47.05 | 32.73 | 43.81 | 41.71 | 28.47 | 19.42 | 29.66 | 48.40 | 57.38 | 50.67 | 15.90 |
| Prussian carp | 1170 | 3 | 2 | 2 | 38.25 | 43.49 | 47.39 | 48.77 | 82.71 | 50.64 | 68.89 | 69.60 | 49.46 | 36.33 | 53.70 | 79.95 | 93.85 | 80.96 | 28.40 |
| Prussian carp | 1171 | 3 | 2 | 2 | 24.19 | 24.06 | 27.25 | 29.12 | 46.46 | 31.22 | 42.16 | 38.87 | 27.39 | 21.29 | 31.03 | 47.16 | 57.64 | 50.40 | 16.23 |
| Prussian carp | 1172 | 3 | 2 | 2 | 24.96 | 24.76 | 26.42 | 25.29 | 45.22 | 31.59 | 40.11 | 38.55 | 29.03 | 20.59 | 29.60 | 44.92 | 56.63 | 50.74 | 16.22 |
| Prussian carp | 1173 | 3 | 2 | 2 | 24.38 | 21.91 | 25.64 | 25.91 | 44.65 | 30.38 | 40.10 | 39.22 | 28.86 | 25.08 | 32.75 | 43.52 | 57.30 | 48.76 | 17.00 |
| Prussian carp | 1174 | 3 | 2 | 2 | 21.29 | 20.92 | 23.68 | 21.54 | 36.55 | 23.63 | 36.90 | 35.48 | 21.80 | 18.62 | 26.27 | 39.67 | 48.86 | 43.19 | 13.20 |
| Prussian carp | 1175 | 3 | 2 | 2 | 19.40 | 20.50 | 22.25 | 23.51 | 35.63 | 22.55 | 34.91 | 32.64 | 21.94 | 17.63 | 24.69 | 38.40 | 46.97 | 40.70 | 12.93 |
| Prussian carp | 1176 | 3 | 2 | 2 | 24.01 | 26.49 | 27.53 | 28.38 | 44.10 | 27.99 | 42.47 | 40.42 | 25.69 | 23.80 | 31.62 | 44.24 | 52.34 | 45.97 | 13.83 |
| Prussian carp | 1177 | 3 | 2 | 2 | 22.07 | 21.82 | 24.19 | 26.24 | 41.21 | 27.06 | 39.69 | 36.96 | 22.57 | 22.75 | 28.75 | 41.62 | 51.01 | 43.65 | 14.70 |
| Prussian carp | 1178 | 3 | 2 | 2 | 20.94 | 19.42 | 22.33 | 26.41 | 37.53 | 25.82 | 37.36 | 33.14 | 21.99 | 17.85 | 25.83 | 37.63 | 46.16 | 41.56 | 12.70 |
| Prussian carp | 1179 | 3 | 2 | 2 | 19.80 | 20.93 | 21.52 | 23.22 | 34.12 | 22.52 | 32.90 | 30.18 | 19.82 | 20.06 | 25.00 | 35.13 | 46.00 | 39.27 | 11.89 |
| Prussian carp | 1180 | 3 | 2 | 2 | 21.20 | 19.89 | 22.58 | 23.42 | 36.08 | 24.11 | 36.67 | 33.21 | 21.28 | 19.00 | 25.93 | 36.43 | 44.58 | 39.42 | 12.20 |
| Prussian carp | 1181 | 3 | 2 | 2 | 19.62 | 20.48 | 21.01 | 22.26 | 35.71 | 23.67 | 33.37 | 31.71 | 21.50 | 17.64 | 24.24 | 36.30 | 44.34 | 37.66 | 12.56 |
| Prussian carp | 1182 | 3 | 2 | 2 | 26.78 | 26.66 | 28.28 | 30.35 | 45.17 | 29.42 | 42.86 | 37.61 | 29.14 | 22.94 | 31.64 | 45.67 | 57.70 | 50.60 | 15.82 |
| Prussian carp | 1183 | 3 | 2 | 2 | 25.22 | 24.55 | 27.77 | 27.80 | 47.71 | 33.53 | 42.71 | 41.04 | 31.50 | 21.97 | 30.72 | 46.51 | 57.13 | 49.73 | 16.50 |
| Prussian carp | 1184 | 3 | 2 | 2 | 28.47 | 27.21 | 31.21 | 35.51 | 51.70 | 35.06 | 49.93 | 44.15 | 32.32 | 24.65 | 35.31 | 53.85 | 63.71 | 56.24 | 17.85 |
| Prussian carp | 1185 | 3 | 2 | 2 | 23.05 | 22.41 | 25.36 | 29.37 | 40.34 | 26.39 | 41.08 | 36.02 | 24.79 | 18.89 | 26.85 | 43.88 | 52.59 | 45.28 | 14.26 |
| Prussian carp | 1186 | 3 | 2 | 2 | 32.76 | 31.12 | 34.57 | 31.55 | 54.32 | 37.21 | 51.04 | 47.54 | 35.35 | 26.00 | 37.15 | 59.33 | 72.76 | 63.02 | 19.37 |
| Prussian carp | 1187 | 3 | 2 | 2 | 25.01 | 23.51 | 26.58 | 29.01 | 41.86 | 27.87 | 42.96 | 36.98 | 22.66 | 20.78 | 29.18 | 43.46 | 55.21 | 49.15 | 15.76 |
| Prussian carp | 1188 | 3 | 2 | 2 | 24.02 | 21.92 | 26.28 | 28.11 | 46.86 | 33.48 | 41.55 | 39.52 | 26.93 | 17.92 | 29.20 | 43.40 | 49.78 | 43.67 | 14.88 |
| Prussian carp | 1189 | 3 | 2 | 2 | 28.46 | 26.89 | 32.19 | 31.86 | 52.43 | 34.21 | 49.03 | 46.97 | 29.51 | 27.20 | 37.50 | 52.93 | 65.84 | 58.00 | 18.61 |
| Prussian carp | 1190 | 3 | 2 | 2 | 20.81 | 23.37 | 24.31 | 25.84 | 38.84 | 24.86 | 37.81 | 33.59 | 24.07 | 18.00 | 26.62 | 39.89 | 51.46 | 46.19 | 14.70 |
| Prussian carp | 1161 | 3 | 2 | 3 | 44.51 | 43.26 | 49.96 | 48.49 | 71.76 | 55.17 | 75.96 | 72.73 | 51.06 | 39.72 | 56.28 | 87.02 | 105.99 | 93.22 | 28.84 |
| Prussian carp | 1162 | 3 | 2 | 3 | 42.55 | 40.81 | 46.50 | 51.09 | 77.79 | 51.16 | 73.04 | 66.04 | 50.66 | 38.62 | 55.85 | 80.29 | 100.74 | 90.40 | 29.10 |
| Prussian carp | 1163 | 3 | 2 | 3 | 39.73 | 40.70 | 48.10 | 49.84 | 77.33 | 53.25 | 75.11 | 66.83 | 49.48 | 37.35 | 52.62 | 83.23 | 105.04 | 92.65 | 28.80 |
| Prussian carp | 1164 | 3 | 2 | 3 | 29.10 | 29.20 | 33.38 | 31.11 | 55.73 | 37.30 | 50.50 | 49.06 | 31.21 | 28.07 | 37.02 | 54.14 | 68.41 | 59.87 | 18.15 |
| Prussian carp | 1165 | 3 | 2 | 3 | 35.58 | 36.65 | 42.70 | 42.03 | 70.16 | 43.10 | 66.39 | 65.96 | 43.80 | 40.43 | 53.91 | 76.27 | 96.88 | 85.52 | 26.25 |
| Prussian carp | 1166 | 3 | 2 | 3 | 33.56 | 37.90 | 40.53 | 42.00 | 67.55 | 47.17 | 62.28 | 56.71 | 45.36 | 32.10 | 46.91 | 69.52 | 88.37 | 80.27 | 22.32 |
| Prussian carp | 1167 | 3 | 2 | 3 | 46.22 | 46.53 | 52.64 | 52.11 | 90.53 | 63.26 | 79.50 | 75.28 | 54.11 | 41.66 | 60.79 | 84.19 | 98.58 | 84.95 | 30.83 |
| Prussian carp | 1168 | 3 | 2 | 3 | 39.25 | 36.28 | 42.28 | 46.25 | 72.81 | 48.77 | 67.04 | 63.10 | 49.86 | 39.10 | 54.46 | 80.38 | 101.47 | 90.79 | 27.60 |
| Prussian carp | 1169 | 3 | 2 | 3 | 24.30 | 24.38 | 27.91 | 28.37 | 47.05 | 32.69 | 43.39 | 41.41 | 26.91 | 21.98 | 31.24 | 47.81 | 57.04 | 50.24 | 16.20 |
| Prussian carp | 1170 | 3 | 2 | 3 | 38.17 | 43.04 | 48.37 | 48.08 | 79.14 | 46.77 | 69.61 | 69.67 | 48.08 | 38.28 | 55.41 | 78.87 | 94.22 | 80.27 | 28.01 |
| Prussian carp | 1171 | 3 | 2 | 3 | 24.42 | 24.25 | 27.86 | 28.81 | 46.95 | 31.71 | 42.81 | 39.70 | 27.60 | 21.06 | 29.79 | 46.46 | 57.57 | 48.34 | 16.78 |
| Prussian carp | 1172 | 3 | 2 | 3 | 25.03 | 25.42 | 26.70 | 26.18 | 45.04 | 29.74 | 39.92 | 38.56 | 29.01 | 20.52 | 29.38 | 45.89 | 57.71 | 51.17 | 15.60 |
| Prussian carp | 1173 | 3 | 2 | 3 | 24.24 | 22.76 | 25.91 | 25.83 | 45.21 | 31.63 | 40.55 | 39.39 | 28.36 | 24.87 | 33.24 | 43.05 | 56.93 | 49.10 | 17.21 |
| Prussian carp | 1174 | 3 | 2 | 3 | 21.56 | 21.25 | 23.63 | 21.08 | 37.49 | 24.61 | 35.39 | 35.47 | 23.82 | 18.85 | 26.24 | 39.83 | 50.01 | 44.70 | 12.67 |
| Prussian carp | 1175 | 3 | 2 | 3 | 19.11 | 20.63 | 22.44 | 23.24 | 32.51 | 21.37 | 34.23 | 32.65 | 21.64 | 17.81 | 24.91 | 37.36 | 45.83 | 39.25 | 13.02 |
| Prussian carp | 1176 | 3 | 2 | 3 | 23.50 | 26.24 | 27.25 | 29.98 | 42.97 | 26.24 | 41.76 | 40.42 | 26.10 | 23.95 | 32.29 | 44.74 | 54.48 | 48.46 | 13.42 |
| Prussian carp | 1177 | 3 | 2 | 3 | 21.32 | 22.14 | 24.43 | 26.05 | 40.70 | 26.15 | 38.69 | 36.59 | 24.22 | 21.26 | 28.67 | 41.60 | 50.11 | 43.14 | 14.22 |
| Prussian carp | 1178 | 3 | 2 | 3 | 20.60 | 20.51 | 23.40 | 26.54 | 37.75 | 26.24 | 37.26 | 33.07 | 21.51 | 18.06 | 26.22 | 37.62 | 46.22 | 41.92 | 12.75 |
| Prussian carp | 1179 | 3 | 2 | 3 | 19.55 | 20.13 | 21.41 | 22.24 | 33.85 | 20.63 | 32.90 | 30.06 | 19.22 | 20.06 | 24.29 | 34.95 | 46.08 | 39.89 | 11.93 |
| Prussian carp | 1180 | 3 | 2 | 3 | 21.16 | 19.31 | 22.31 | 23.36 | 35.60 | 24.47 | 36.29 | 33.02 | 21.90 | 19.20 | 26.17 | 36.06 | 43.85 | 38.89 | 12.62 |
| Prussian carp | 1181 | 3 | 2 | 3 | 19.75 | 20.89 | 21.46 | 22.01 | 36.00 | 24.08 | 33.39 | 31.66 | 21.35 | 18.11 | 24.23 | 35.57 | 43.87 | 37.13 | 12.43 |
| Prussian carp | 1182 | 3 | 2 | 3 | 26.67 | 26.03 | 27.81 | 29.85 | 44.33 | 28.16 | 42.70 | 36.92 | 29.65 | 22.79 | 31.21 | 45.16 | 55.77 | 48.81 | 15.80 |
| Prussian carp | 1183 | 3 | 2 | 3 | 26.18 | 25.05 | 27.91 | 25.80 | 48.26 | 34.55 | 43.38 | 41.06 | 30.35 | 22.37 | 31.06 | 46.68 | 56.70 | 49.79 | 16.92 |
| Prussian carp | 1184 | 3 | 2 | 3 | 28.55 | 26.72 | 31.07 | 34.46 | 52.13 | 35.57 | 49.96 | 44.72 | 33.03 | 24.43 | 35.87 | 54.74 | 65.22 | 57.67 | 18.05 |
| Prussian carp | 1185 | 3 | 2 | 3 | 23.18 | 23.18 | 25.96 | 27.29 | 41.66 | 27.62 | 40.76 | 35.53 | 25.82 | 17.41 | 26.51 | 44.44 | 51.50 | 45.31 | 14.52 |
| Prussian carp | 1186 | 3 | 2 | 3 | 32.29 | 29.29 | 33.50 | 31.86 | 53.86 | 35.98 | 50.72 | 47.69 | 35.78 | 26.06 | 37.09 | 59.36 | 71.19 | 62.51 | 19.93 |
| Prussian carp | 1187 | 3 | 2 | 3 | 25.30 | 23.97 | 26.58 | 28.28 | 41.55 | 27.87 | 42.96 | 37.19 | 23.56 | 20.17 | 28.76 | 43.37 | 54.42 | 47.91 | 15.60 |
| Prussian carp | 1188 | 3 | 2 | 3 | 23.84 | 22.07 | 26.54 | 27.81 | 47.52 | 34.70 | 41.72 | 39.30 | 27.03 | 17.86 | 28.90 | 43.38 | 48.62 | 44.20 | 15.32 |
| Prussian carp | 1189 | 3 | 2 | 3 | 28.44 | 27.38 | 31.97 | 31.65 | 53.02 | 35.58 | 48.39 | 46.67 | 29.21 | 27.33 | 37.99 | 52.58 | 66.17 | 57.85 | 19.21 |
| Prussian carp | 1190 | 3 | 2 | 3 | 21.47 | 23.62 | 24.11 | 24.81 | 39.11 | 25.08 | 37.49 | 34.05 | 25.70 | 18.38 | 26.61 | 40.24 | 51.13 | 45.71 | 14.71 |
| Prussian carp | 1251 | 3 | 3 | 1 | 32.85 | 33.07 | 39.79 | 38.71 | 70.50 | 46.36 | 61.27 | 64.49 | 39.51 | 35.87 | 47.69 | 71.69 | 85.06 | 73.35 | 23.70 |
| Prussian carp | 1252 | 3 | 3 | 1 | 38.93 | 35.27 | 44.97 | 45.35 | 78.11 | 50.18 | 71.04 | 74.30 | 48.33 | 35.70 | 54.45 | 86.42 | 102.32 | 90.47 | 30.20 |
| Prussian carp | 1253 | 3 | 3 | 1 | 36.01 | 34.93 | 44.41 | 44.50 | 77.29 | 48.73 | 69.23 | 73.02 | 49.92 | 35.28 | 51.89 | 82.94 | 96.81 | 84.13 | 26.61 |
| Prussian carp | 1254 | 3 | 3 | 1 | 32.27 | 41.44 | 42.24 | 39.39 | 65.21 | 41.19 | 61.31 | 57.77 | 39.57 | 32.57 | 43.74 | 68.37 | 89.51 | 82.49 | 22.22 |
| Prussian carp | 1255 | 3 | 3 | 1 | 28.25 | 29.34 | 35.30 | 32.23 | 55.99 | 39.71 | 53.88 | 55.66 | 40.92 | 31.47 | 42.12 | 64.22 | 75.34 | 63.39 | 20.16 |
| Prussian carp | 1256 | 3 | 3 | 1 | 34.68 | 33.41 | 39.26 | 38.22 | 66.55 | 45.83 | 60.25 | 57.46 | 39.42 | 30.64 | 45.13 | 65.11 | 78.42 | 69.24 | 24.00 |
| Prussian carp | 1257 | 3 | 3 | 1 | 29.67 | 27.60 | 35.06 | 40.97 | 70.00 | 50.26 | 57.31 | 57.16 | 41.20 | 31.93 | 47.75 | 68.44 | 80.29 | 73.57 | 22.23 |
| Prussian carp | 1258 | 3 | 3 | 1 | 27.47 | 28.01 | 32.11 | 33.89 | 59.42 | 39.67 | 51.53 | 52.67 | 42.76 | 31.85 | 39.52 | 61.60 | 74.28 | 62.78 | 18.93 |
| Prussian carp | 1259 | 3 | 3 | 1 | 27.80 | 27.48 | 34.04 | 36.63 | 57.00 | 38.39 | 54.70 | 51.93 | 39.67 | 25.33 | 38.65 | 63.98 | 77.58 | 70.95 | 21.02 |
| Prussian carp | 1260 | 3 | 3 | 1 | 29.19 | 31.39 | 36.62 | 36.58 | 59.84 | 35.66 | 55.32 | 54.43 | 39.52 | 31.13 | 42.80 | 67.41 | 83.77 | 73.87 | 22.66 |
| Prussian carp | 1261 | 3 | 3 | 1 | 30.76 | 31.30 | 40.84 | 42.84 | 73.72 | 52.38 | 65.05 | 63.22 | 47.96 | 31.97 | 49.43 | 68.88 | 83.57 | 75.55 | 25.86 |
| Prussian carp | 1262 | 3 | 3 | 1 | 28.48 | 28.72 | 33.75 | 33.63 | 56.02 | 37.28 | 52.88 | 50.41 | 36.47 | 27.71 | 41.04 | 57.73 | 68.51 | 61.31 | 20.87 |
| Prussian carp | 1263 | 3 | 3 | 1 | 30.43 | 29.86 | 36.60 | 38.05 | 62.91 | 43.84 | 57.63 | 56.00 | 39.89 | 26.85 | 40.82 | 63.64 | 75.94 | 67.06 | 22.05 |
| Prussian carp | 1264 | 3 | 3 | 1 | 39.62 | 37.28 | 46.96 | 42.35 | 79.03 | 52.24 | 69.46 | 72.93 | 48.23 | 38.78 | 58.37 | 83.41 | 102.48 | 90.59 | 31.81 |
| Prussian carp | 1265 | 3 | 3 | 1 | 28.74 | 27.00 | 35.06 | 37.93 | 63.50 | 41.80 | 54.77 | 56.36 | 42.24 | 29.65 | 41.02 | 68.63 | 85.44 | 73.72 | 23.26 |
| Prussian carp | 1266 | 3 | 3 | 1 | 27.78 | 29.05 | 35.70 | 33.92 | 63.33 | 43.07 | 54.19 | 56.39 | 39.34 | 27.52 | 42.42 | 65.79 | 80.66 | 74.30 | 22.99 |
| Prussian carp | 1267 | 3 | 3 | 1 | 29.39 | 30.58 | 35.94 | 33.82 | 60.62 | 39.39 | 53.58 | 53.98 | 38.14 | 28.37 | 42.43 | 66.59 | 80.69 | 74.44 | 21.41 |
| Prussian carp | 1268 | 3 | 3 | 1 | 32.00 | 29.70 | 39.34 | 32.82 | 66.46 | 42.87 | 57.61 | 62.26 | 40.25 | 31.28 | 49.21 | 68.88 | 81.16 | 69.97 | 26.12 |
| Prussian carp | 1269 | 3 | 3 | 1 | 27.20 | 30.31 | 33.01 | 36.44 | 55.36 | 36.95 | 51.24 | 46.52 | 32.30 | 24.01 | 34.62 | 55.93 | 68.86 | 59.73 | 19.36 |
| Prussian carp | 1270 | 3 | 3 | 1 | 30.27 | 30.51 | 36.86 | 35.87 | 65.45 | 44.38 | 58.62 | 61.48 | 40.69 | 26.42 | 43.50 | 69.19 | 81.65 | 76.73 | 22.54 |
| Prussian carp | 1271 | 3 | 3 | 1 | 36.64 | 36.38 | 37.50 | 35.60 | 60.82 | 39.47 | 55.48 | 53.71 | 40.97 | 29.61 | 42.17 | 63.62 | 75.75 | 64.67 | 22.42 |
| Prussian carp | 1272 | 3 | 3 | 1 | 26.99 | 25.17 | 31.21 | 32.73 | 58.40 | 41.18 | 51.82 | 55.18 | 39.93 | 25.29 | 39.54 | 59.54 | 67.66 | 58.93 | 20.85 |
| Prussian carp | 1273 | 3 | 3 | 1 | 29.17 | 29.29 | 35.59 | 37.66 | 64.02 | 46.37 | 57.28 | 57.91 | 42.80 | 27.45 | 40.54 | 67.41 | 76.00 | 62.77 | 22.16 |
| Prussian carp | 1274 | 3 | 3 | 1 | 29.87 | 31.94 | 37.12 | 38.15 | 65.47 | 44.56 | 54.42 | 53.71 | 40.86 | 27.97 | 40.66 | 63.47 | 76.05 | 65.45 | 22.70 |
| Prussian carp | 1275 | 3 | 3 | 1 | 32.05 | 33.12 | 38.40 | 36.78 | 69.84 | 52.87 | 63.80 | 66.47 | 44.86 | 35.04 | 49.51 | 74.06 | 88.63 | 79.06 | 24.26 |
| Prussian carp | 4030 | 3 | 3 | 1 | 37.31 | 39.52 | 44.15 | 40.50 | 72.24 | 47.18 | 64.21 | 64.63 | 45.86 | 33.22 | 47.89 | 74.29 | 93.18 | 82.81 | 26.01 |
| Prussian carp | 4031 | 3 | 3 | 1 | 30.94 | 29.82 | 35.44 | 32.13 | 59.79 | 37.65 | 51.55 | 54.24 | 41.07 | 24.56 | 40.45 | 65.45 | 77.18 | 72.58 | 21.17 |
| Prussian carp | 4032 | 3 | 3 | 1 | 30.09 | 29.92 | 37.13 | 43.19 | 73.30 | 51.53 | 60.97 | 62.57 | 44.87 | 31.15 | 46.66 | 76.09 | 85.65 | 72.88 | 24.99 |
| Prussian carp | 4033 | 3 | 3 | 1 | 27.73 | 27.53 | 35.45 | 37.09 | 68.50 | 46.79 | 57.71 | 62.42 | 40.32 | 30.47 | 46.60 | 71.03 | 80.95 | 69.63 | 24.47 |
| Prussian carp | 4034 | 3 | 3 | 1 | 32.13 | 36.65 | 43.81 | 44.19 | 76.85 | 52.07 | 67.24 | 68.65 | 50.39 | 30.96 | 51.13 | 79.50 | 88.06 | 77.04 | 27.81 |
| Prussian carp | 1251 | 3 | 3 | 2 | 33.29 | 30.54 | 38.41 | 38.31 | 69.64 | 46.64 | 60.78 | 64.42 | 40.39 | 33.61 | 47.58 | 71.91 | 85.51 | 74.25 | 24.01 |
| Prussian carp | 1252 | 3 | 3 | 2 | 37.32 | 35.57 | 44.93 | 45.77 | 80.27 | 51.32 | 71.26 | 75.24 | 46.42 | 37.91 | 56.66 | 84.81 | 100.48 | 88.06 | 29.55 |
| Prussian carp | 1253 | 3 | 3 | 2 | 34.24 | 34.03 | 43.22 | 46.57 | 77.71 | 49.25 | 68.60 | 72.35 | 49.53 | 35.61 | 51.92 | 82.15 | 95.87 | 83.59 | 26.45 |
| Prussian carp | 1254 | 3 | 3 | 2 | 31.96 | 39.93 | 41.18 | 40.28 | 64.80 | 42.26 | 61.79 | 57.47 | 39.69 | 70.83 | 79.50 | 57.22 | 89.13 | 81.35 | 23.41 |
| Prussian carp | 1255 | 3 | 3 | 2 | 28.59 | 30.22 | 34.82 | 33.22 | 60.46 | 39.44 | 54.45 | 55.52 | 40.20 | 29.77 | 40.96 | 64.52 | 75.41 | 63.80 | 20.35 |
| Prussian carp | 1256 | 3 | 3 | 2 | 33.92 | 31.99 | 37.77 | 38.85 | 65.27 | 43.72 | 59.73 | 58.51 | 40.72 | 29.78 | 44.92 | 65.35 | 79.09 | 68.95 | 23.58 |
| Prussian carp | 1257 | 3 | 3 | 2 | 30.09 | 28.62 | 34.95 | 40.63 | 67.81 | 47.32 | 57.10 | 56.29 | 41.50 | 30.87 | 46.05 | 69.38 | 81.58 | 74.23 | 22.22 |
| Prussian carp | 1258 | 3 | 3 | 2 | 28.79 | 28.09 | 32.43 | 32.95 | 58.65 | 40.34 | 51.40 | 52.68 | 42.78 | 30.60 | 39.55 | 61.60 | 73.36 | 62.92 | 18.10 |
| Prussian carp | 1259 | 3 | 3 | 2 | 27.10 | 26.84 | 33.50 | 36.50 | 59.64 | 41.31 | 55.24 | 52.05 | 36.90 | 26.90 | 39.59 | 62.03 | 76.68 | 70.55 | 21.02 |
| Prussian carp | 1260 | 3 | 3 | 2 | 29.28 | 31.49 | 37.02 | 36.35 | 59.47 | 35.00 | 55.09 | 54.26 | 40.66 | 30.81 | 43.06 | 67.92 | 84.66 | 74.51 | 22.65 |
| Prussian carp | 1261 | 3 | 3 | 2 | 31.29 | 31.42 | 40.51 | 42.61 | 74.10 | 55.13 | 65.02 | 63.44 | 47.54 | 31.99 | 49.23 | 69.61 | 84.34 | 76.69 | 25.83 |
| Prussian carp | 1262 | 3 | 3 | 2 | 29.43 | 28.59 | 34.35 | 34.16 | 56.58 | 36.99 | 52.69 | 50.73 | 36.17 | 28.78 | 40.99 | 57.76 | 69.88 | 61.05 | 20.30 |
| Prussian carp | 1263 | 3 | 3 | 2 | 30.33 | 29.60 | 36.60 | 37.91 | 63.51 | 41.71 | 57.32 | 55.83 | 39.50 | 27.39 | 40.27 | 62.93 | 75.06 | 65.03 | 22.23 |
| Prussian carp | 1264 | 3 | 3 | 2 | 38.85 | 35.86 | 47.27 | 42.01 | 78.97 | 51.43 | 69.87 | 72.87 | 46.17 | 39.72 | 58.50 | 82.52 | 101.08 | 90.15 | 31.62 |
| Prussian carp | 1265 | 3 | 3 | 2 | 27.94 | 26.59 | 34.33 | 37.93 | 64.49 | 43.21 | 55.85 | 56.65 | 41.35 | 29.27 | 41.05 | 68.71 | 84.23 | 72.27 | 22.81 |
| Prussian carp | 1266 | 3 | 3 | 2 | 27.64 | 28.60 | 35.08 | 32.55 | 64.01 | 46.10 | 54.10 | 56.18 | 39.76 | 27.38 | 42.07 | 66.79 | 80.85 | 74.33 | 22.52 |
| Prussian carp | 1267 | 3 | 3 | 2 | 30.12 | 31.63 | 37.17 | 32.72 | 61.40 | 40.39 | 53.90 | 54.94 | 37.70 | 29.18 | 42.65 | 67.08 | 80.40 | 74.88 | 21.77 |
| Prussian carp | 1268 | 3 | 3 | 2 | 32.14 | 28.76 | 39.42 | 32.42 | 66.73 | 43.41 | 57.14 | 61.96 | 40.48 | 31.00 | 48.70 | 68.69 | 80.75 | 70.26 | 26.56 |
| Prussian carp | 1269 | 3 | 3 | 2 | 26.93 | 29.55 | 33.03 | 35.26 | 54.83 | 32.61 | 50.92 | 46.96 | 33.20 | 23.92 | 34.95 | 55.75 | 68.61 | 60.15 | 19.40 |
| Prussian carp | 1270 | 3 | 3 | 2 | 29.80 | 30.37 | 35.78 | 37.07 | 66.42 | 45.48 | 58.02 | 61.76 | 41.30 | 26.38 | 43.62 | 69.29 | 81.96 | 76.67 | 23.00 |
| Prussian carp | 1271 | 3 | 3 | 2 | 36.41 | 35.39 | 37.06 | 36.77 | 61.36 | 38.52 | 55.11 | 53.71 | 41.70 | 29.87 | 42.49 | 63.71 | 76.01 | 64.93 | 21.78 |
| Prussian carp | 1272 | 3 | 3 | 2 | 26.66 | 25.67 | 32.25 | 32.88 | 59.12 | 41.56 | 52.03 | 54.99 | 39.95 | 25.16 | 38.77 | 59.25 | 67.16 | 58.59 | 20.41 |
| Prussian carp | 1273 | 3 | 3 | 2 | 30.84 | 28.96 | 36.02 | 34.35 | 65.03 | 45.88 | 57.49 | 58.06 | 42.59 | 27.30 | 40.80 | 67.13 | 75.48 | 62.23 | 22.05 |
| Prussian carp | 1274 | 3 | 3 | 2 | 29.44 | 30.34 | 36.60 | 36.78 | 64.63 | 42.42 | 56.44 | 52.97 | 38.60 | 27.38 | 40.07 | 63.40 | 76.75 | 65.71 | 23.15 |
| Prussian carp | 1275 | 3 | 3 | 2 | 33.38 | 33.41 | 39.52 | 35.57 | 70.58 | 53.69 | 63.70 | 66.43 | 43.90 | 34.96 | 50.09 | 73.59 | 88.38 | 78.96 | 23.09 |
| Prussian carp | 4030 | 3 | 3 | 2 | 36.10 | 37.50 | 43.01 | 41.02 | 71.78 | 45.73 | 64.81 | 65.21 | 44.98 | 33.17 | 47.35 | 74.13 | 92.29 | 81.25 | 25.96 |
| Prussian carp | 4031 | 3 | 3 | 2 | 30.67 | 31.41 | 36.49 | 31.40 | 60.46 | 39.58 | 52.08 | 54.79 | 40.69 | 24.15 | 40.29 | 64.53 | 75.36 | 70.39 | 22.13 |
| Prussian carp | 4032 | 3 | 3 | 2 | 28.75 | 29.60 | 35.93 | 43.38 | 73.06 | 52.35 | 60.73 | 61.36 | 43.35 | 33.29 | 47.23 | 75.41 | 85.98 | 72.11 | 24.49 |
| Prussian carp | 4033 | 3 | 3 | 2 | 26.21 | 27.36 | 34.66 | 37.06 | 66.66 | 44.78 | 57.29 | 62.23 | 40.62 | 30.39 | 46.44 | 71.66 | 80.51 | 69.18 | 24.03 |
| Prussian carp | 4034 | 3 | 3 | 2 | 32.41 | 35.51 | 43.22 | 43.54 | 77.37 | 51.85 | 66.55 | 68.43 | 50.04 | 31.74 | 50.98 | 78.05 | 87.06 | 75.92 | 27.61 |
| Prussian carp | 1251 | 3 | 3 | 3 | 32.93 | 31.88 | 39.01 | 37.80 | 69.89 | 47.06 | 60.04 | 63.74 | 40.83 | 32.76 | 48.16 | 71.90 | 84.57 | 73.25 | 23.60 |
| Prussian carp | 1252 | 3 | 3 | 3 | 37.96 | 34.86 | 45.12 | 45.58 | 80.51 | 52.78 | 70.89 | 74.25 | 48.53 | 35.99 | 55.76 | 85.97 | 102.42 | 90.31 | 29.60 |
| Prussian carp | 1253 | 3 | 3 | 3 | 35.40 | 34.35 | 43.63 | 45.37 | 78.05 | 49.33 | 68.91 | 72.03 | 49.44 | 36.01 | 52.49 | 82.69 | 96.58 | 83.94 | 27.03 |
| Prussian carp | 1254 | 3 | 3 | 3 | 31.65 | 39.53 | 40.95 | 39.90 | 64.86 | 41.77 | 61.96 | 58.12 | 38.70 | 32.48 | 44.05 | 67.78 | 88.40 | 80.58 | 22.09 |
| Prussian carp | 1255 | 3 | 3 | 3 | 28.09 | 28.83 | 34.55 | 32.46 | 60.12 | 38.89 | 53.05 | 55.66 | 41.86 | 30.69 | 41.24 | 64.31 | 75.34 | 63.79 | 20.74 |
| Prussian carp | 1256 | 3 | 3 | 3 | 33.84 | 32.38 | 38.26 | 38.52 | 66.76 | 46.14 | 60.04 | 58.07 | 39.36 | 30.81 | 44.98 | 65.26 | 78.70 | 69.72 | 24.31 |
| Prussian carp | 1257 | 3 | 3 | 3 | 30.21 | 27.82 | 35.39 | 40.21 | 67.15 | 46.96 | 57.45 | 57.17 | 41.68 | 30.74 | 45.58 | 69.02 | 80.98 | 74.52 | 22.69 |
| Prussian carp | 1258 | 3 | 3 | 3 | 28.96 | 27.90 | 32.41 | 33.60 | 59.05 | 39.81 | 51.55 | 52.85 | 43.70 | 30.39 | 39.29 | 61.21 | 73.91 | 61.78 | 18.84 |
| Prussian carp | 1259 | 3 | 3 | 3 | 27.13 | 26.31 | 33.63 | 35.15 | 57.22 | 39.20 | 54.60 | 52.13 | 39.69 | 24.81 | 38.37 | 63.67 | 76.87 | 70.95 | 20.99 |
| Prussian carp | 1260 | 3 | 3 | 3 | 28.62 | 32.68 | 38.03 | 36.55 | 60.09 | 34.99 | 54.78 | 54.65 | 40.13 | 30.94 | 42.51 | 67.36 | 84.08 | 74.59 | 22.53 |
| Prussian carp | 1261 | 3 | 3 | 3 | 30.96 | 33.87 | 42.10 | 43.14 | 74.51 | 54.31 | 64.76 | 63.38 | 47.40 | 32.70 | 49.25 | 69.52 | 83.87 | 77.08 | 26.08 |
| Prussian carp | 1262 | 3 | 3 | 3 | 30.12 | 28.92 | 34.05 | 33.77 | 56.44 | 36.62 | 52.60 | 51.05 | 36.87 | 27.65 | 41.08 | 56.98 | 68.92 | 60.38 | 20.61 |
| Prussian carp | 1263 | 3 | 3 | 3 | 29.24 | 29.49 | 36.45 | 38.52 | 63.41 | 42.99 | 57.15 | 55.38 | 39.58 | 27.52 | 40.59 | 62.59 | 75.14 | 66.00 | 22.21 |
| Prussian carp | 1264 | 3 | 3 | 3 | 38.29 | 34.77 | 47.21 | 42.58 | 80.68 | 52.06 | 69.92 | 72.95 | 46.55 | 39.52 | 58.44 | 83.18 | 102.29 | 90.76 | 31.56 |
| Prussian carp | 1265 | 3 | 3 | 3 | 27.69 | 27.47 | 34.67 | 38.74 | 63.30 | 41.78 | 55.23 | 56.38 | 42.85 | 28.97 | 40.12 | 68.47 | 84.08 | 72.31 | 23.37 |
| Prussian carp | 1266 | 3 | 3 | 3 | 26.97 | 28.93 | 34.75 | 34.05 | 63.00 | 42.35 | 53.92 | 56.66 | 39.54 | 27.61 | 42.10 | 67.37 | 82.84 | 77.05 | 22.81 |
| Prussian carp | 1267 | 3 | 3 | 3 | 29.38 | 30.46 | 35.71 | 33.23 | 61.05 | 40.73 | 54.57 | 54.19 | 36.33 | 29.84 | 43.39 | 66.71 | 81.66 | 74.88 | 21.57 |
| Prussian carp | 1268 | 3 | 3 | 3 | 32.14 | 28.97 | 39.08 | 33.14 | 67.20 | 43.94 | 57.75 | 62.43 | 39.93 | 32.31 | 49.18 | 67.87 | 80.32 | 70.62 | 26.40 |
| Prussian carp | 1269 | 3 | 3 | 3 | 26.63 | 29.97 | 33.32 | 34.24 | 54.22 | 36.02 | 50.71 | 45.92 | 32.14 | 23.37 | 34.82 | 55.63 | 66.91 | 59.49 | 19.20 |
| Prussian carp | 1270 | 3 | 3 | 3 | 29.98 | 30.76 | 36.41 | 35.21 | 64.99 | 44.86 | 58.60 | 61.91 | 41.20 | 26.50 | 43.30 | 69.58 | 82.95 | 77.93 | 22.70 |
| Prussian carp | 1271 | 3 | 3 | 3 | 35.78 | 36.28 | 37.65 | 35.90 | 60.85 | 39.28 | 54.62 | 53.41 | 41.68 | 31.73 | 42.15 | 63.48 | 74.69 | 64.88 | 21.95 |
| Prussian carp | 1272 | 3 | 3 | 3 | 26.90 | 25.38 | 32.40 | 33.17 | 59.67 | 41.08 | 52.21 | 55.40 | 38.72 | 26.50 | 39.87 | 59.42 | 67.21 | 58.64 | 20.71 |
| Prussian carp | 1273 | 3 | 3 | 3 | 30.21 | 27.26 | 35.15 | 36.94 | 64.33 | 45.60 | 57.39 | 57.96 | 43.69 | 27.35 | 39.45 | 67.19 | 74.84 | 62.19 | 21.69 |
| Prussian carp | 1274 | 3 | 3 | 3 | 29.70 | 31.51 | 37.01 | 38.58 | 63.97 | 42.85 | 57.01 | 53.12 | 38.12 | 27.94 | 40.79 | 63.54 | 76.59 | 64.73 | 22.96 |
| Prussian carp | 1275 | 3 | 3 | 3 | 31.87 | 33.38 | 39.03 | 35.75 | 70.46 | 52.62 | 64.01 | 66.87 | 44.61 | 34.80 | 49.28 | 73.16 | 87.69 | 78.26 | 23.92 |
| Prussian carp | 4030 | 3 | 3 | 3 | 37.45 | 37.99 | 43.65 | 41.04 | 71.76 | 45.91 | 64.71 | 64.66 | 45.61 | 31.83 | 47.29 | 74.75 | 92.06 | 81.69 | 26.11 |
| Prussian carp | 4031 | 3 | 3 | 3 | 31.34 | 29.41 | 35.22 | 31.24 | 61.09 | 38.98 | 51.87 | 54.37 | 41.10 | 23.15 | 40.18 | 64.11 | 75.04 | 70.24 | 21.32 |
| Prussian carp | 4032 | 3 | 3 | 3 | 27.71 | 29.71 | 35.56 | 45.38 | 72.84 | 51.87 | 60.64 | 62.42 | 43.21 | 33.68 | 47.48 | 74.95 | 86.18 | 72.54 | 24.67 |
| Prussian carp | 4033 | 3 | 3 | 3 | 26.27 | 27.96 | 34.91 | 37.17 | 68.36 | 47.26 | 57.39 | 62.57 | 41.30 | 29.10 | 46.39 | 72.82 | 81.03 | 70.14 | 24.22 |
| Prussian carp | 4034 | 3 | 3 | 3 | 31.46 | 35.65 | 43.42 | 44.23 | 76.21 | 51.59 | 66.52 | 68.71 | 51.21 | 30.40 | 50.88 | 79.41 | 87.13 | 77.33 | 27.00 |
