# Supplementary material for: Repeatability, Reproducibility, Separative Power and Subjectivity of Different Fish Morphometric Analysis Methods
Source: PLoS One. 2016 Jun 21;11(6):e0157890. doi: 10.1371/journal.pone.0157890 (PMC4915670; doi:10.1371/journal.pone.0157890)
Supplement: S4 Table — (DOCX) [file pone.0157890.s004.docx]

Supplementary Table 4 Raw dataset of the TRA analyses, for codes see text.

| Species | individual code | Measu-rer | Site | repeat | SL | 1 | 2 | 3 | 4 | 5 | 6 | 7 | 8 | 9 | 10 | 11 | 12 | 13 | 14 | 15 |
| --- | --- | --- | --- | --- | --- | --- | --- | --- | --- | --- | --- | --- | --- | --- | --- | --- | --- | --- | --- | --- |
| Bleak | 1031 | 1 | 1 | 1 | 96.36 | 14.95 | 5.35 | 9.97 | 21.09 | 21.61 | 20.12 | 40.69 | 16.45 | 53.83 | 19.26 | 62.79 | 15.64 | 16.39 | 9.76 | 22.95 |
| Bleak | 1032 | 1 | 1 | 1 | 121.99 | 15.43 | 6.51 | 10.48 | 20.88 | 22.11 | 19.89 | 44.17 | 15.73 | 52.79 | 18.86 | 62.20 | 13.51 | 14.46 | 8.49 | 21.70 |
| Bleak | 1033 | 1 | 1 | 1 | 126.34 | 15.76 | 5.45 | 9.71 | 21.29 | 23.51 | 19.70 | 43.29 | 13.42 | 52.76 | 18.46 | 63.16 | 13.61 | 15.15 | 9.41 | 22.90 |
| Bleak | 1034 | 1 | 1 | 1 | 95.73 | 14.91 | 5.71 | 8.69 | 20.44 | 23.24 | 19.18 | 42.46 | 13.42 | 52.24 | 16.30 | 60.31 | 11.93 | 11.45 | 9.24 | 22.25 |
| Bleak | 1035 | 1 | 1 | 1 | 77.93 | 14.80 | 5.84 | 9.22 | 20.85 | 22.40 | 15.43 | 42.21 | 13.35 | 53.41 | 16.19 | 61.34 | 12.52 | 16.45 | 8.84 | 22.59 |
| Bleak | 1036 | 1 | 1 | 1 | 117.39 | 15.86 | 5.08 | 9.81 | 20.70 | 22.30 | 18.34 | 41.70 | 15.26 | 51.10 | 18.01 | 60.15 | 14.13 | 17.04 | 9.90 | 23.36 |
| Bleak | 1037 | 1 | 1 | 1 | 102.1 | 15.06 | 5.79 | 9.47 | 19.62 | 22.00 | 18.84 | 44.23 | 14.63 | 54.44 | 18.16 | 60.99 | 14.00 | 16.52 | 9.75 | 22.17 |
| Bleak | 1038 | 1 | 1 | 1 | 104.61 | 15.56 | 5.52 | 9.87 | 19.74 | 20.85 | 17.47 | 43.47 | 13.92 | 50.47 | 18.21 | 61.25 | 12.01 | 15.20 | 9.58 | 24.19 |
| Bleak | 1039 | 1 | 1 | 1 | 89 | 14.65 | 5.41 | 10.08 | 21.31 | 22.91 | 17.85 | 42.93 | 13.82 | 52.58 | 18.53 | 60.12 | 13.40 | 11.95 | 8.91 | 22.81 |
| Bleak | 1040 | 1 | 1 | 1 | 126.72 | 14.17 | 5.86 | 10.01 | 21.02 | 22.52 | 20.19 | 41.72 | 15.34 | 49.89 | 19.85 | 60.85 | 13.22 | 15.97 | 9.21 | 23.30 |
| Bleak | 1041 | 1 | 1 | 1 | 79.57 | 14.74 | 5.49 | 9.58 | 21.46 | 23.03 | 19.34 | 42.06 | 12.50 | 52.20 | 18.24 | 63.43 | 13.28 | 13.67 | 8.89 | 22.67 |
| Bleak | 1042 | 1 | 1 | 1 | 99.66 | 15.94 | 6.30 | 9.97 | 20.86 | 23.08 | 19.01 | 44.44 | 15.78 | 51.88 | 20.53 | 60.85 | 14.59 | 16.03 | 9.59 | 23.15 |
| Bleak | 1043 | 1 | 1 | 1 | 109.39 | 15.10 | 5.19 | 8.90 | 19.34 | 21.73 | 19.81 | 42.11 | 14.55 | 50.87 | 18.66 | 60.51 | 13.81 | 13.51 | 9.39 | 22.90 |
| Bleak | 1044 | 1 | 1 | 1 | 89.62 | 13.71 | 5.19 | 8.23 | 20.45 | 22.81 | 17.92 | 42.48 | 13.94 | 53.75 | 19.77 | 61.71 | 13.72 | 14.68 | 8.52 | 19.73 |
| Bleak | 1045 | 1 | 1 | 1 | 96.13 | 15.80 | 5.37 | 8.49 | 17.70 | 18.34 | 17.57 | 41.54 | 13.35 | 52.41 | 16.86 | 59.77 | 12.62 | 12.77 | 9.02 | 23.22 |
| Bleak | 1046 | 1 | 1 | 1 | 115.36 | 14.96 | 5.85 | 10.48 | 21.50 | 22.54 | 19.17 | 43.90 | 14.61 | 51.88 | 17.78 | 61.56 | 14.83 | 16.56 | 9.43 | 21.37 |
| Bleak | 1047 | 1 | 1 | 1 | 114.09 | 14.28 | 5.18 | 10.58 | 20.97 | 23.50 | 19.98 | 44.04 | 14.79 | 53.49 | 18.41 | 64.15 | 12.78 | 17.55 | 9.20 | 21.94 |
| Bleak | 1048 | 1 | 1 | 1 | 96.85 | 15.88 | 6.29 | 9.74 | 21.07 | 23.87 | 19.31 | 44.07 | 14.57 | 54.34 | 17.82 | 60.38 | 12.95 | 14.76 | 9.66 | 22.20 |
| Bleak | 1049 | 1 | 1 | 1 | 123.23 | 15.20 | 5.47 | 8.95 | 20.35 | 21.82 | 18.63 | 42.54 | 13.23 | 52.02 | 17.78 | 64.46 | 12.87 | 13.83 | 8.85 | 24.03 |
| Bleak | 1050 | 1 | 1 | 1 | 91.94 | 14.00 | 5.63 | 9.46 | 19.91 | 21.24 | 19.04 | 42.80 | 12.44 | 51.39 | 17.43 | 59.81 | 13.24 | 14.94 | 8.67 | 20.45 |
| Bleak | 1051 | 1 | 1 | 1 | 112.01 | 15.23 | 5.72 | 8.38 | 20.06 | 21.01 | 19.57 | 44.83 | 14.03 | 52.95 | 16.95 | 62.04 | 13.13 | 14.53 | 8.70 | 20.38 |
| Bleak | 1052 | 1 | 1 | 1 | 118.22 | 14.95 | 6.29 | 10.17 | 19.77 | 21.72 | 18.37 | 44.17 | 14.64 | 52.48 | 19.09 | 61.88 | 12.39 | 13.78 | 9.31 | 23.41 |
| Bleak | 1053 | 1 | 1 | 1 | 110.89 | 16.00 | 6.46 | 9.14 | 20.64 | 22.65 | 18.80 | 43.87 | 15.18 | 52.71 | 20.59 | 61.81 | 13.69 | 12.93 | 8.77 | 22.64 |
| Bleak | 1054 | 1 | 1 | 1 | 88.22 | 14.82 | 5.61 | 9.49 | 21.31 | 21.39 | 18.93 | 40.61 | 13.94 | 48.56 | 21.31 | 59.83 | 12.54 | 15.61 | 9.04 | 22.78 |
| Bleak | 1055 | 1 | 1 | 1 | 100.01 | 15.81 | 5.62 | 8.97 | 20.02 | 22.19 | 17.60 | 41.02 | 14.04 | 50.20 | 18.90 | 58.74 | 13.22 | 14.44 | 8.99 | 22.83 |
| Bleak | 1056 | 1 | 1 | 1 | 73.68 | 14.22 | 6.20 | 9.46 | 21.05 | 23.57 | 15.50 | 44.82 | 12.21 | 51.22 | 19.09 | 58.78 | 13.87 | 14.34 | 9.65 | 20.94 |
| Bleak | 1057 | 1 | 1 | 1 | 78.9 | 15.34 | 6.15 | 10.13 | 21.14 | 22.81 | 19.70 | 43.50 | 14.72 | 53.08 | 18.53 | 64.23 | 13.35 | 17.76 | 9.37 | 22.23 |
| Bleak | 1058 | 1 | 1 | 1 | 98.91 | 15.23 | 6.51 | 10.44 | 22.04 | 23.84 | 19.14 | 47.09 | 15.04 | 54.39 | 20.66 | 65.58 | 14.20 | 13.44 | 9.33 | 24.88 |
| Bleak | 1059 | 1 | 1 | 1 | 95.66 | 14.88 | 5.60 | 9.16 | 20.00 | 22.06 | 17.75 | 41.58 | 12.24 | 53.05 | 16.83 | 61.46 | 11.97 | 11.84 | 8.25 | 20.64 |
| Bleak | 1060 | 1 | 1 | 1 | 96.53 | 15.67 | 5.61 | 9.31 | 20.16 | 22.12 | 18.06 | 44.79 | 14.22 | 52.80 | 18.69 | 62.46 | 12.47 | 17.82 | 9.82 | 23.22 |
| Bleak | 1031 | 1 | 1 | 2 | 96.71 | 15.72 | 5.59 | 8.96 | 20.30 | 22.50 | 19.95 | 41.40 | 14.54 | 50.89 | 18.87 | 61.29 | 13.89 | 11.93 | 9.18 | 22.85 |
| Bleak | 1032 | 1 | 1 | 2 | 122.86 | 16.36 | 5.96 | 8.65 | 20.25 | 22.07 | 19.48 | 43.09 | 14.80 | 51.68 | 18.61 | 59.57 | 13.75 | 9.82 | 7.75 | 21.05 |
| Bleak | 1033 | 1 | 1 | 2 | 126.49 | 14.87 | 5.59 | 9.27 | 20.93 | 22.30 | 19.04 | 43.10 | 14.89 | 52.51 | 19.01 | 61.53 | 13.48 | 10.92 | 8.63 | 22.33 |
| Bleak | 1034 | 1 | 1 | 2 | 94.93 | 15.13 | 5.52 | 8.44 | 20.37 | 22.10 | 15.00 | 41.12 | 14.08 | 51.83 | 15.55 | 61.05 | 13.21 | 11.78 | 9.02 | 21.58 |
| Bleak | 1035 | 1 | 1 | 2 | 76.48 | 15.10 | 6.19 | 8.98 | 18.29 | 22.35 | 18.36 | 41.11 | 16.92 | 52.26 | 17.25 | 61.62 | 13.27 | 11.61 | 8.93 | 22.29 |
| Bleak | 1036 | 1 | 1 | 2 | 118.94 | 14.93 | 5.31 | 8.66 | 20.74 | 22.29 | 18.74 | 40.27 | 14.12 | 49.28 | 17.56 | 56.73 | 12.58 | 13.11 | 8.85 | 18.29 |
| Bleak | 1037 | 1 | 1 | 2 | 104.03 | 15.17 | 5.00 | 7.92 | 19.82 | 21.58 | 19.11 | 42.25 | 13.84 | 52.83 | 17.91 | 59.97 | 13.58 | 10.94 | 9.02 | 21.24 |
| Bleak | 1038 | 1 | 1 | 2 | 104.22 | 15.53 | 5.70 | 8.29 | 20.67 | 22.25 | 17.99 | 38.84 | 12.18 | 49.33 | 18.51 | 60.43 | 11.81 | 10.96 | 9.50 | 23.35 |
| Bleak | 1039 | 1 | 1 | 2 | 89.49 | 15.10 | 5.22 | 9.17 | 20.61 | 22.12 | 17.22 | 41.61 | 13.45 | 49.25 | 18.22 | 59.74 | 12.74 | 10.74 | 8.86 | 23.06 |
| Bleak | 1040 | 1 | 1 | 2 | 127.84 | 15.54 | 5.86 | 8.94 | 20.34 | 22.60 | 19.78 | 43.08 | 14.97 | 53.12 | 19.88 | 59.69 | 12.58 | 10.53 | 8.58 | 22.57 |
| Bleak | 1041 | 1 | 1 | 2 | 79.82 | 14.19 | 4.85 | 8.53 | 21.58 | 22.33 | 18.16 | 41.60 | 12.57 | 48.90 | 17.88 | 59.82 | 14.23 | 12.04 | 8.27 | 22.01 |
| Bleak | 1042 | 1 | 1 | 2 | 101.85 | 14.87 | 5.45 | 8.06 | 19.87 | 21.76 | 18.67 | 41.12 | 13.92 | 49.66 | 20.23 | 57.78 | 12.93 | 11.00 | 8.67 | 22.01 |
| Bleak | 1043 | 1 | 1 | 2 | 109.63 | 13.21 | 5.39 | 8.39 | 20.36 | 21.32 | 18.62 | 40.06 | 13.75 | 49.28 | 18.23 | 59.23 | 14.87 | 9.21 | 8.96 | 21.77 |
| Bleak | 1044 | 1 | 1 | 2 | 89.44 | 14.37 | 5.76 | 8.28 | 20.41 | 21.96 | 18.32 | 41.58 | 14.47 | 52.63 | 17.91 | 60.09 | 13.94 | 11.29 | 7.91 | 18.22 |
| Bleak | 1045 | 1 | 1 | 2 | 97.48 | 15.37 | 5.24 | 8.35 | 18.97 | 20.23 | 17.34 | 40.26 | 12.79 | 50.25 | 17.71 | 58.68 | 13.16 | 11.51 | 8.52 | 22.15 |
| Bleak | 1046 | 1 | 1 | 2 | 115.28 | 15.84 | 5.34 | 8.40 | 20.34 | 22.41 | 19.43 | 42.44 | 14.36 | 52.42 | 19.89 | 60.36 | 13.58 | 10.82 | 8.83 | 20.61 |
| Bleak | 1047 | 1 | 1 | 2 | 114.07 | 15.44 | 5.33 | 9.25 | 20.85 | 22.72 | 19.11 | 43.66 | 14.34 | 49.78 | 21.97 | 60.35 | 13.63 | 13.19 | 8.77 | 21.67 |
| Bleak | 1048 | 1 | 1 | 2 | 97.57 | 15.66 | 5.88 | 9.25 | 20.75 | 22.07 | 19.17 | 41.79 | 14.26 | 43.27 | 19.02 | 51.60 | 12.50 | 12.66 | 9.07 | 21.72 |
| Bleak | 1049 | 1 | 1 | 2 | 125.33 | 15.73 | 5.30 | 8.62 | 19.86 | 20.45 | 16.81 | 39.53 | 12.83 | 50.94 | 17.29 | 61.94 | 12.55 | 11.04 | 8.72 | 24.38 |
| Bleak | 1050 | 1 | 1 | 2 | 90.83 | 15.23 | 5.69 | 8.45 | 20.09 | 22.13 | 18.66 | 41.95 | 12.96 | 51.32 | 17.01 | 60.41 | 13.16 | 10.92 | 8.40 | 20.58 |
| Bleak | 1051 | 1 | 1 | 2 | 113.68 | 12.67 | 6.02 | 8.34 | 19.50 | 21.48 | 18.20 | 42.92 | 12.89 | 43.62 | 15.44 | 59.59 | 14.27 | 11.79 | 8.27 | 19.24 |
| Bleak | 1052 | 1 | 1 | 2 | 118.76 | 15.49 | 5.56 | 9.14 | 20.08 | 21.52 | 17.91 | 42.65 | 14.02 | 51.07 | 19.38 | 59.92 | 12.37 | 11.47 | 8.66 | 22.36 |
| Bleak | 1053 | 1 | 1 | 2 | 114.12 | 14.66 | 5.69 | 8.33 | 19.90 | 21.10 | 18.53 | 40.97 | 14.10 | 51.04 | 17.49 | 59.09 | 13.67 | 10.07 | 8.41 | 21.14 |
| Bleak | 1054 | 1 | 1 | 2 | 88.81 | 14.91 | 5.57 | 8.47 | 19.79 | 21.41 | 18.04 | 39.36 | 14.58 | 50.53 | 19.03 | 58.94 | 12.42 | 8.07 | 8.45 | 21.77 |
| Bleak | 1055 | 1 | 1 | 2 | 99.55 | 14.96 | 5.48 | 9.51 | 19.71 | 20.64 | 18.25 | 40.94 | 13.35 | 49.06 | 17.65 | 60.78 | 12.64 | 9.27 | 8.31 | 22.55 |
| Bleak | 1056 | 1 | 1 | 2 | 74.52 | 13.94 | 5.46 | 7.98 | 19.77 | 18.30 | 14.89 | 49.87 | 12.91 | 50.82 | 18.22 | 62.02 | 13.80 | 10.03 | 8.67 | 20.91 |
| Bleak | 1057 | 1 | 1 | 2 | 79.14 | 15.53 | 5.21 | 9.09 | 21.19 | 22.10 | 19.12 | 41.02 | 14.16 | 51.38 | 17.66 | 60.26 | 11.95 | 11.75 | 8.68 | 22.07 |
| Bleak | 1058 | 1 | 1 | 2 | 98.59 | 17.03 | 5.91 | 9.37 | 22.14 | 22.88 | 19.63 | 43.31 | 13.23 | 52.43 | 20.05 | 63.74 | 13.89 | 10.75 | 9.12 | 24.42 |
| Bleak | 1059 | 1 | 1 | 2 | 94.6 | 14.74 | 5.66 | 8.01 | 19.70 | 21.26 | 18.20 | 40.60 | 12.95 | 50.94 | 17.58 | 59.18 | 12.52 | 9.37 | 8.40 | 19.98 |
| Bleak | 1060 | 1 | 1 | 2 | 98.88 | 14.82 | 5.17 | 8.08 | 18.47 | 20.69 | 18.35 | 41.06 | 14.40 | 49.05 | 17.54 | 60.23 | 13.01 | 12.86 | 8.56 | 22.30 |
| Bleak | 1031 | 1 | 1 | 3 | 97.3 | 16.26 | 5.13 | 8.81 | 20.28 | 22.10 | 20.68 | 42.06 | 14.75 | 51.86 | 19.65 | 61.46 | 13.71 | 11.82 | 9.45 | 22.74 |
| Bleak | 1032 | 1 | 1 | 3 | 121.46 | 15.01 | 5.26 | 8.31 | 20.54 | 21.87 | 19.45 | 42.61 | 14.63 | 50.86 | 18.54 | 60.18 | 13.38 | 12.32 | 7.78 | 20.67 |
| Bleak | 1033 | 1 | 1 | 3 | 126.82 | 16.27 | 5.60 | 9.13 | 19.76 | 19.35 | 19.61 | 42.06 | 15.45 | 52.62 | 19.17 | 61.03 | 13.78 | 11.76 | 8.66 | 22.44 |
| Bleak | 1034 | 1 | 1 | 3 | 95.12 | 14.84 | 5.07 | 8.66 | 21.01 | 22.67 | 18.36 | 41.20 | 14.11 | 51.17 | 16.58 | 60.76 | 14.00 | 11.35 | 8.89 | 22.08 |
| Bleak | 1035 | 1 | 1 | 3 | 76.28 | 15.07 | 5.42 | 9.19 | 20.77 | 22.44 | 17.60 | 41.03 | 11.76 | 52.34 | 16.37 | 61.92 | 12.42 | 10.79 | 8.43 | 21.60 |
| Bleak | 1036 | 1 | 1 | 3 | 117.98 | 14.40 | 5.35 | 9.04 | 20.74 | 22.51 | 18.61 | 39.36 | 13.20 | 49.18 | 16.88 | 57.23 | 17.71 | 11.56 | 8.80 | 22.36 |
| Bleak | 1037 | 1 | 1 | 3 | 104.09 | 14.78 | 5.19 | 8.20 | 20.12 | 21.58 | 18.32 | 41.35 | 13.80 | 52.07 | 18.37 | 59.31 | 13.40 | 12.29 | 8.87 | 21.54 |
| Bleak | 1038 | 1 | 1 | 3 | 103.98 | 15.17 | 5.48 | 7.61 | 20.57 | 22.03 | 18.16 | 40.08 | 12.38 | 48.07 | 17.76 | 60.56 | 11.93 | 11.56 | 8.96 | 23.73 |
| Bleak | 1039 | 1 | 1 | 3 | 89.25 | 14.62 | 4.81 | 9.06 | 19.71 | 21.80 | 18.41 | 40.75 | 12.32 | 49.19 | 19.85 | 60.54 | 13.75 | 9.47 | 8.86 | 22.64 |
| Bleak | 1041 | 1 | 1 | 3 | 126.043 | 14.78 | 5.50 | 9.19 | 20.97 | 23.18 | 19.88 | 42.51 | 14.99 | 51.47 | 20.72 | 59.77 | 13.19 | 10.62 | 8.67 | 22.57 |
| Bleak | 1042 | 1 | 1 | 3 | 80.81 | 14.85 | 5.05 | 9.04 | 20.83 | 22.13 | 15.87 | 41.09 | 11.94 | 48.72 | 17.67 | 59.91 | 14.48 | 10.47 | 8.21 | 21.52 |
| Bleak | 1043 | 1 | 1 | 3 | 100.95 | 15.71 | 5.81 | 8.21 | 20.14 | 22.31 | 18.73 | 41.81 | 14.95 | 50.39 | 18.94 | 59.45 | 13.46 | 11.86 | 8.73 | 21.77 |
| Bleak | 1044 | 1 | 1 | 3 | 109.7 | 14.61 | 4.97 | 7.90 | 19.33 | 20.71 | 18.80 | 40.24 | 13.99 | 49.16 | 17.41 | 58.66 | 14.25 | 9.87 | 8.63 | 20.92 |
| Bleak | 1044 | 1 | 1 | 3 | 89.31 | 14.15 | 5.49 | 7.91 | 19.94 | 21.21 | 18.00 | 41.44 | 14.42 | 52.15 | 15.46 | 57.41 | 15.11 | 12.23 | 8.13 | 19.87 |
| Bleak | 1045 | 1 | 1 | 3 | 97.35 | 15.62 | 5.54 | 7.90 | 19.99 | 21.01 | 16.59 | 39.23 | 13.02 | 49.57 | 18.13 | 57.61 | 12.33 | 11.76 | 10.59 | 21.70 |
| Bleak | 1046 | 1 | 1 | 3 | 115.28 | 14.99 | 5.65 | 8.36 | 20.71 | 22.92 | 19.00 | 41.12 | 13.36 | 51.96 | 17.65 | 59.43 | 14.44 | 11.30 | 8.52 | 20.75 |
| Bleak | 1047 | 1 | 1 | 3 | 115.38 | 15.96 | 5.96 | 8.81 | 20.93 | 22.71 | 19.76 | 42.59 | 14.66 | 50.50 | 19.06 | 59.86 | 13.22 | 12.74 | 9.18 | 21.55 |
| Bleak | 1048 | 1 | 1 | 3 | 98.47 | 15.43 | 5.81 | 9.75 | 21.44 | 22.65 | 19.40 | 41.27 | 13.57 | 51.43 | 18.26 | 57.93 | 12.72 | 10.94 | 9.05 | 21.73 |
| Bleak | 1049 | 1 | 1 | 3 | 127.86 | 15.32 | 5.19 | 8.51 | 18.58 | 20.27 | 16.65 | 39.24 | 13.65 | 50.07 | 17.70 | 59.26 | 12.01 | 11.00 | 8.26 | 23.26 |
| Bleak | 1050 | 1 | 1 | 3 | 91.85 | 15.24 | 5.77 | 8.00 | 19.89 | 21.30 | 16.76 | 41.25 | 13.04 | 47.00 | 17.12 | 59.64 | 13.38 | 11.21 | 8.46 | 19.79 |
| Bleak | 1051 | 1 | 1 | 3 | 112.73 | 14.93 | 5.46 | 8.68 | 20.62 | 21.64 | 17.45 | 42.87 | 13.04 | 51.04 | 17.19 | 58.22 | 13.82 | 10.82 | 8.61 | 19.55 |
| Bleak | 1052 | 1 | 1 | 3 | 119.62 | 15.20 | 5.17 | 8.49 | 19.92 | 21.43 | 16.43 | 40.65 | 14.14 | 50.65 | 18.57 | 60.22 | 13.96 | 12.25 | 8.12 | 21.88 |
| Bleak | 1053 | 1 | 1 | 3 | 113.98 | 15.51 | 5.67 | 8.57 | 19.81 | 22.06 | 18.48 | 41.26 | 13.83 | 50.29 | 14.21 | 59.01 | 14.43 | 11.33 | 8.57 | 21.39 |
| Bleak | 1054 | 1 | 1 | 3 | 88.36 | 14.99 | 5.19 | 8.32 | 19.81 | 20.98 | 18.15 | 39.65 | 14.50 | 48.79 | 17.41 | 59.13 | 12.50 | 13.13 | 8.19 | 21.56 |
| Bleak | 1055 | 1 | 1 | 3 | 100.78 | 14.66 | 5.11 | 7.86 | 18.89 | 20.09 | 17.19 | 39.33 | 12.44 | 51.35 | 16.68 | 60.00 | 12.16 | 11.97 | 10.33 | 21.90 |
| Bleak | 1056 | 1 | 1 | 3 | 74.22 | 15.12 | 5.59 | 7.32 | 20.81 | 21.89 | 14.68 | 42.73 | 12.00 | 50.46 | 17.74 | 61.92 | 13.34 | 10.92 | 8.71 | 20.08 |
| Bleak | 1057 | 1 | 1 | 3 | 78.08 | 14.98 | 5.40 | 9.33 | 20.85 | 22.24 | 19.13 | 43.32 | 13.19 | 51.68 | 16.80 | 60.04 | 12.58 | 10.85 | 8.91 | 21.62 |
| Bleak | 1058 | 1 | 1 | 3 | 99.41 | 16.10 | 5.68 | 9.90 | 22.10 | 23.77 | 19.43 | 44.23 | 14.19 | 52.68 | 19.89 | 64.12 | 13.60 | 10.32 | 8.93 | 23.57 |
| Bleak | 1059 | 1 | 1 | 3 | 95.31 | 15.07 | 5.32 | 9.13 | 20.36 | 21.98 | 18.37 | 41.30 | 13.00 | 52.85 | 17.45 | 61.27 | 11.65 | 9.57 | 8.25 | 20.46 |
| Bleak | 1060 | 1 | 1 | 3 | 97.41 | 14.56 | 4.93 | 8.56 | 19.19 | 20.65 | 17.67 | 40.64 | 14.04 | 49.96 | 18.56 | 61.31 | 12.50 | 12.08 | 8.47 | 21.98 |
| Bleak | 1131 | 1 | 2 | 1 | 102.81 | 14.68 | 6.31 | 8.30 | 19.92 | 20.70 | 19.13 | 41.07 | 13.24 | 53.39 | 20.25 | 55.62 | 11.72 | 14.63 | 9.85 | 23.07 |
| Bleak | 1132 | 1 | 2 | 1 | 85.06 | 14.77 | 6.27 | 8.80 | 20.28 | 22.26 | 17.36 | 42.86 | 12.98 | 50.74 | 19.56 | 59.91 | 14.55 | 14.42 | 8.95 | 22.29 |
| Bleak | 1133 | 1 | 2 | 1 | 86.88 | 14.37 | 5.72 | 7.72 | 18.90 | 19.44 | 21.70 | 40.09 | 15.39 | 50.04 | 18.68 | 61.04 | 13.89 | 11.28 | 9.75 | 22.43 |
| Bleak | 1134 | 1 | 2 | 1 | 75.46 | 15.07 | 6.08 | 10.04 | 21.66 | 20.90 | 18.76 | 36.39 | 15.92 | 52.77 | 19.13 | 61.38 | 14.06 | 11.42 | 9.53 | 24.41 |
| Bleak | 1135 | 1 | 2 | 1 | 82.8 | 13.65 | 5.77 | 8.60 | 19.74 | 20.90 | 16.07 | 39.34 | 13.17 | 50.90 | 17.26 | 60.72 | 13.53 | 13.98 | 9.40 | 21.98 |
| Bleak | 1136 | 1 | 2 | 1 | 77.39 | 15.47 | 6.69 | 8.45 | 21.03 | 21.45 | 18.86 | 40.87 | 14.23 | 51.62 | 17.88 | 61.53 | 13.09 | 16.39 | 10.25 | 23.89 |
| Bleak | 1137 | 1 | 2 | 1 | 91.85 | 15.11 | 5.63 | 8.26 | 19.81 | 22.25 | 17.91 | 42.46 | 14.82 | 51.19 | 16.89 | 59.27 | 11.99 | 15.38 | 8.83 | 22.79 |
| Bleak | 1138 | 1 | 2 | 1 | 92.83 | 14.84 | 5.95 | 8.79 | 19.83 | 20.88 | 21.21 | 43.05 | 14.11 | 51.59 | 15.03 | 60.75 | 13.23 | 9.75 | 9.59 | 23.03 |
| Bleak | 1139 | 1 | 2 | 1 | 113.96 | 15.58 | 5.46 | 9.30 | 19.84 | 20.39 | 18.56 | 39.50 | 13.03 | 51.95 | 18.49 | 63.57 | 12.45 | 15.30 | 9.50 | 24.83 |
| Bleak | 1140 | 1 | 2 | 1 | 89.25 | 14.39 | 5.77 | 7.59 | 19.62 | 21.42 | 19.74 | 41.61 | 14.74 | 51.91 | 18.58 | 60.33 | 14.41 | 12.67 | 8.92 | 21.63 |
| Bleak | 1141 | 1 | 2 | 1 | 65.45 | 15.29 | 5.90 | 8.03 | 19.78 | 22.36 | 21.20 | 41.71 | 14.23 | 52.40 | 18.36 | 63.65 | 12.02 | 12.50 | 9.90 | 23.26 |
| Bleak | 1142 | 1 | 2 | 1 | 107.51 | 13.82 | 5.04 | 8.66 | 19.66 | 20.61 | 18.90 | 41.01 | 13.86 | 50.40 | 18.57 | 61.14 | 13.39 | 11.50 | 8.93 | 21.69 |
| Bleak | 1143 | 1 | 2 | 1 | 87.38 | 13.88 | 6.03 | 8.93 | 18.78 | 19.54 | 19.62 | 37.85 | 14.11 | 49.42 | 19.98 | 56.73 | 13.31 | 11.43 | 8.41 | 22.04 |
| Bleak | 1144 | 1 | 2 | 1 | 93.15 | 14.27 | 5.53 | 8.86 | 19.68 | 22.72 | 19.51 | 42.73 | 13.46 | 53.36 | 18.25 | 62.65 | 12.00 | 12.69 | 9.38 | 23.83 |
| Bleak | 1145 | 1 | 2 | 1 | 74.94 | 13.82 | 6.01 | 8.58 | 19.84 | 21.30 | 19.38 | 41.67 | 13.03 | 52.65 | 17.35 | 61.37 | 12.40 | 12.43 | 8.75 | 21.70 |
| Bleak | 1146 | 1 | 2 | 1 | 103.89 | 15.22 | 5.73 | 8.63 | 20.02 | 21.71 | 20.17 | 44.10 | 13.19 | 51.68 | 20.26 | 61.98 | 13.92 | 17.40 | 9.05 | 24.35 |
| Bleak | 1147 | 1 | 2 | 1 | 104.75 | 14.10 | 5.85 | 8.78 | 20.10 | 21.46 | 19.39 | 42.31 | 14.22 | 53.10 | 18.98 | 62.97 | 14.19 | 15.49 | 9.49 | 22.93 |
| Bleak | 1148 | 1 | 2 | 1 | 103.83 | 14.51 | 6.11 | 8.28 | 21.95 | 22.08 | 18.54 | 41.44 | 14.08 | 51.66 | 18.59 | 61.82 | 12.99 | 15.14 | 9.56 | 22.66 |
| Bleak | 1149 | 1 | 2 | 1 | 106.08 | 13.57 | 6.03 | 8.85 | 19.96 | 21.09 | 18.93 | 41.04 | 16.72 | 49.50 | 16.38 | 58.82 | 12.23 | 16.00 | 8.40 | 22.12 |
| Bleak | 1150 | 1 | 2 | 1 | 85.95 | 14.62 | 5.13 | 8.33 | 20.44 | 21.99 | 18.14 | 43.08 | 13.99 | 51.75 | 18.29 | 62.63 | 13.58 | 11.86 | 9.34 | 24.39 |
| Bleak | 1151 | 1 | 2 | 1 | 82.67 | 14.79 | 5.80 | 8.62 | 19.72 | 21.18 | 19.67 | 46.00 | 14.42 | 50.22 | 19.62 | 55.11 | 13.29 | 15.56 | 9.01 | 23.60 |
| Bleak | 1152 | 1 | 2 | 1 | 104.39 | 14.31 | 6.24 | 7.36 | 19.75 | 19.91 | 17.88 | 41.76 | 15.30 | 51.34 | 19.37 | 59.49 | 14.05 | 13.74 | 8.02 | 22.09 |
| Bleak | 1153 | 1 | 2 | 1 | 105.66 | 14.04 | 4.99 | 9.23 | 20.41 | 20.88 | 20.07 | 41.93 | 14.55 | 52.16 | 18.77 | 60.79 | 14.18 | 10.88 | 9.09 | 21.84 |
| Bleak | 1154 | 1 | 2 | 1 | 81.2 | 15.18 | 5.61 | 8.77 | 20.09 | 20.97 | 19.34 | 40.50 | 13.43 | 47.71 | 17.67 | 58.56 | 12.43 | 13.87 | 9.00 | 22.33 |
| Bleak | 1155 | 1 | 2 | 1 | 104.24 | 15.53 | 5.81 | 9.81 | 20.73 | 23.50 | 17.44 | 42.47 | 14.36 | 52.67 | 20.31 | 60.85 | 13.81 | 13.02 | 10.21 | 23.62 |
| Bleak | 1156 | 1 | 2 | 1 | 95.12 | 13.85 | 5.44 | 8.04 | 19.66 | 22.65 | 17.85 | 42.07 | 15.34 | 52.96 | 18.83 | 62.90 | 13.29 | 12.99 | 9.58 | 23.10 |
| Bleak | 1157 | 1 | 2 | 1 | 100.86 | 15.41 | 6.36 | 9.62 | 19.44 | 22.32 | 19.37 | 45.11 | 13.78 | 54.32 | 19.03 | 64.15 | 14.12 | 9.66 | 9.49 | 22.03 |
| Bleak | 1158 | 1 | 2 | 1 | 89.71 | 14.66 | 5.95 | 8.35 | 19.51 | 23.10 | 19.12 | 44.20 | 13.55 | 50.05 | 20.17 | 61.16 | 14.03 | 13.12 | 9.00 | 21.40 |
| Bleak | 1159 | 1 | 2 | 1 | 75.09 | 14.90 | 6.42 | 10.00 | 19.93 | 22.42 | 18.53 | 41.27 | 14.20 | 51.81 | 18.95 | 60.63 | 13.25 | 13.11 | 9.74 | 23.02 |
| Bleak | 1160 | 1 | 2 | 1 | 88.71 | 14.93 | 5.46 | 8.41 | 19.78 | 21.50 | 19.17 | 42.18 | 15.16 | 51.54 | 18.81 | 63.12 | 13.88 | 13.83 | 8.57 | 21.72 |
| Bleak | 1131 | 1 | 2 | 2 | 105.45 | 14.46 | 5.80 | 8.48 | 19.36 | 21.07 | 19.72 | 42.00 | 11.81 | 52.31 | 16.51 | 61.14 | 13.16 | 11.21 | 9.04 | 22.82 |
| Bleak | 1132 | 1 | 2 | 2 | 85.32 | 15.10 | 5.50 | 8.13 | 19.22 | 21.12 | 17.40 | 39.76 | 14.68 | 48.68 | 17.96 | 57.56 | 13.81 | 10.08 | 8.84 | 22.41 |
| Bleak | 1133 | 1 | 2 | 2 | 86.87 | 14.91 | 5.66 | 8.89 | 19.93 | 21.47 | 20.34 | 39.91 | 15.40 | 48.34 | 17.96 | 59.04 | 13.51 | 13.24 | 8.97 | 21.39 |
| Bleak | 1134 | 1 | 2 | 2 | 75.6 | 15.39 | 6.43 | 9.66 | 19.81 | 20.78 | 18.36 | 40.27 | 13.11 | 51.00 | 17.83 | 59.30 | 13.60 | 9.67 | 9.13 | 22.60 |
| Bleak | 1135 | 1 | 2 | 2 | 84.11 | 14.17 | 5.33 | 8.93 | 18.45 | 21.48 | 17.94 | 41.64 | 12.82 | 50.17 | 19.19 | 59.34 | 13.56 | 9.98 | 8.93 | 22.32 |
| Bleak | 1136 | 1 | 2 | 2 | 77.5 | 14.92 | 5.43 | 8.60 | 19.66 | 20.72 | 19.39 | 40.75 | 13.31 | 48.50 | 16.40 | 60.43 | 11.28 | 12.83 | 8.55 | 23.14 |
| Bleak | 1137 | 1 | 2 | 2 | 91.99 | 15.32 | 5.98 | 8.88 | 20.05 | 21.64 | 18.76 | 40.30 | 15.07 | 50.16 | 19.11 | 59.71 | 13.06 | 11.23 | 8.28 | 22.02 |
| Bleak | 1138 | 1 | 2 | 2 | 92.05 | 15.08 | 5.88 | 9.16 | 20.80 | 22.80 | 20.32 | 42.33 | 15.15 | 50.95 | 21.07 | 60.17 | 13.83 | 12.69 | 7.88 | 21.22 |
| Bleak | 1139 | 1 | 2 | 2 | 114.53 | 15.63 | 5.70 | 9.20 | 20.87 | 20.69 | 18.19 | 41.40 | 11.29 | 48.47 | 17.25 | 60.95 | 12.48 | 11.42 | 9.16 | 25.63 |
| Bleak | 1140 | 1 | 2 | 2 | 90.06 | 14.22 | 5.82 | 8.16 | 19.13 | 20.14 | 17.86 | 43.29 | 13.81 | 52.19 | 20.32 | 60.10 | 13.68 | 10.71 | 8.66 | 20.66 |
| Bleak | 1141 | 1 | 2 | 2 | 66.99 | 16.28 | 4.95 | 9.45 | 20.37 | 21.38 | 19.20 | 42.50 | 14.11 | 52.70 | 17.30 | 60.43 | 12.46 | 13.10 | 9.16 | 23.12 |
| Bleak | 1142 | 1 | 2 | 2 | 106.94 | 14.47 | 5.58 | 7.37 | 18.28 | 20.62 | 18.98 | 38.03 | 13.79 | 50.41 | 19.09 | 60.68 | 13.25 | 10.46 | 8.82 | 22.11 |
| Bleak | 1143 | 1 | 2 | 2 | 85.33 | 14.07 | 5.87 | 8.49 | 18.70 | 19.76 | 18.53 | 40.05 | 13.69 | 50.02 | 19.87 | 60.16 | 14.43 | 11.06 | 8.35 | 21.75 |
| Bleak | 1144 | 1 | 2 | 2 | 93.29 | 14.84 | 5.82 | 8.31 | 19.02 | 20.24 | 18.96 | 39.85 | 14.06 | 51.02 | 16.60 | 60.81 | 13.09 | 11.29 | 8.95 | 22.80 |
| Bleak | 1145 | 1 | 2 | 2 | 77.59 | 14.40 | 5.89 | 7.85 | 19.33 | 20.99 | 18.45 | 42.69 | 12.99 | 50.87 | 16.59 | 59.79 | 12.33 | 12.53 | 8.34 | 22.08 |
| Bleak | 1146 | 1 | 2 | 2 | 106.13 | 15.17 | 5.30 | 8.04 | 19.55 | 21.39 | 20.00 | 41.46 | 15.04 | 49.10 | 21.10 | 59.87 | 13.59 | 10.04 | 8.43 | 24.09 |
| Bleak | 1147 | 1 | 2 | 2 | 106.78 | 15.12 | 6.09 | 8.22 | 20.36 | 22.07 | 17.31 | 41.15 | 13.49 | 51.29 | 17.42 | 60.09 | 13.04 | 11.49 | 8.82 | 22.45 |
| Bleak | 1148 | 1 | 2 | 2 | 104.08 | 14.20 | 5.32 | 8.73 | 19.85 | 21.66 | 18.40 | 42.12 | 13.28 | 52.26 | 17.15 | 59.29 | 11.96 | 13.45 | 8.24 | 22.43 |
| Bleak | 1149 | 1 | 2 | 2 | 102.11 | 15.63 | 5.76 | 7.34 | 20.04 | 21.65 | 19.44 | 41.85 | 13.67 | 52.88 | 18.11 | 62.84 | 13.46 | 10.57 | 8.86 | 23.50 |
| Bleak | 1150 | 1 | 2 | 2 | 89.96 | 14.93 | 5.15 | 8.92 | 19.88 | 21.65 | 18.46 | 41.56 | 13.45 | 51.14 | 16.91 | 59.32 | 13.03 | 10.16 | 8.82 | 23.99 |
| Bleak | 1151 | 1 | 2 | 2 | 82.4 | 15.61 | 5.40 | 8.52 | 19.08 | 21.61 | 18.82 | 42.01 | 14.53 | 48.85 | 17.11 | 58.88 | 12.57 | 11.46 | 8.32 | 23.43 |
| Bleak | 1152 | 1 | 2 | 2 | 102.87 | 14.10 | 5.46 | 8.60 | 19.52 | 21.66 | 16.73 | 41.97 | 14.09 | 51.29 | 18.70 | 59.68 | 12.37 | 11.22 | 7.99 | 22.21 |
| Bleak | 1153 | 1 | 2 | 2 | 107.13 | 14.51 | 4.96 | 8.60 | 19.98 | 21.15 | 18.48 | 44.66 | 14.50 | 51.84 | 18.57 | 59.21 | 12.61 | 9.64 | 8.51 | 20.66 |
| Bleak | 1154 | 1 | 2 | 2 | 81.3 | 17.14 | 8.48 | 12.00 | 20.06 | 20.97 | 18.33 | 40.51 | 13.05 | 48.75 | 19.45 | 57.33 | 12.93 | 11.77 | 8.94 | 21.83 |
| Bleak | 1155 | 1 | 2 | 2 | 105.34 | 14.87 | 5.59 | 8.45 | 20.47 | 22.85 | 19.43 | 41.90 | 14.07 | 50.64 | 18.64 | 59.65 | 13.99 | 11.70 | 9.52 | 23.94 |
| Bleak | 1156 | 1 | 2 | 2 | 96.8 | 14.81 | 5.31 | 8.89 | 19.97 | 22.17 | 18.66 | 42.04 | 14.31 | 51.49 | 18.33 | 59.99 | 13.85 | 11.78 | 8.46 | 23.29 |
| Bleak | 1157 | 1 | 2 | 2 | 101.5 | 13.96 | 5.66 | 8.52 | 19.68 | 20.86 | 20.19 | 41.38 | 13.99 | 54.48 | 18.65 | 59.80 | 12.65 | 11.55 | 8.80 | 21.58 |
| Bleak | 1158 | 1 | 2 | 2 | 91.89 | 15.27 | 5.26 | 8.27 | 19.42 | 21.08 | 17.73 | 39.50 | 12.34 | 48.25 | 18.27 | 56.42 | 13.16 | 11.12 | 8.54 | 21.90 |
| Bleak | 1159 | 1 | 2 | 2 | 73.24 | 15.17 | 4.90 | 8.75 | 21.35 | 27.83 | 18.83 | 42.99 | 13.58 | 52.24 | 17.68 | 61.40 | 14.15 | 12.60 | 9.33 | 23.67 |
| Bleak | 1160 | 1 | 2 | 2 | 90.44 | 14.71 | 5.31 | 8.20 | 18.83 | 22.24 | 17.61 | 39.71 | 13.06 | 49.12 | 17.66 | 60.56 | 12.77 | 12.83 | 8.30 | 21.94 |
| Bleak | 1131 | 1 | 2 | 3 | 107.28 | 13.32 | 5.45 | 8.19 | 19.35 | 20.28 | 18.91 | 40.16 | 13.85 | 50.43 | 18.33 | 59.72 | 12.34 | 11.12 | 8.69 | 21.48 |
| Bleak | 1132 | 1 | 2 | 3 | 84.7 | 14.76 | 5.16 | 7.45 | 19.33 | 21.56 | 16.34 | 41.63 | 12.72 | 49.95 | 20.09 | 60.06 | 12.33 | 9.38 | 8.80 | 21.46 |
| Bleak | 1133 | 1 | 2 | 3 | 87.02 | 14.61 | 4.69 | 7.90 | 19.99 | 21.40 | 20.97 | 38.18 | 15.84 | 49.17 | 18.24 | 58.52 | 14.00 | 11.32 | 8.67 | 22.06 |
| Bleak | 1134 | 1 | 2 | 3 | 74.9 | 14.27 | 5.51 | 7.92 | 24.97 | 26.34 | 18.27 | 41.84 | 13.95 | 49.45 | 18.66 | 56.52 | 14.14 | 9.56 | 8.35 | 21.67 |
| Bleak | 1135 | 1 | 2 | 3 | 82.26 | 15.91 | 5.47 | 7.54 | 19.85 | 20.83 | 19.50 | 42.38 | 11.36 | 49.01 | 16.64 | 61.48 | 13.12 | 13.17 | 9.36 | 22.83 |
| Bleak | 1136 | 1 | 2 | 3 | 76.15 | 14.86 | 5.70 | 7.81 | 20.14 | 22.02 | 19.27 | 41.33 | 14.93 | 52.05 | 17.72 | 60.39 | 14.11 | 12.71 | 8.80 | 23.53 |
| Bleak | 1137 | 1 | 2 | 3 | 91.64 | 14.35 | 4.82 | 7.92 | 19.09 | 21.62 | 17.71 | 41.86 | 15.18 | 50.57 | 12.87 | 60.58 | 12.77 | 13.43 | 8.73 | 22.09 |
| Bleak | 1138 | 1 | 2 | 3 | 92.43 | 14.42 | 5.67 | 8.68 | 19.97 | 22.33 | 20.10 | 43.05 | 14.12 | 51.07 | 19.12 | 61.26 | 11.44 | 14.20 | 7.53 | 21.62 |
| Bleak | 1139 | 1 | 2 | 3 | 113.44 | 15.76 | 5.44 | 8.56 | 20.51 | 21.47 | 17.93 | 41.01 | 12.88 | 51.79 | 18.83 | 64.07 | 11.25 | 11.99 | 9.27 | 24.84 |
| Bleak | 1140 | 1 | 2 | 3 | 90.5 | 14.90 | 5.56 | 7.39 | 19.84 | 20.83 | 18.89 | 42.23 | 13.89 | 52.97 | 18.07 | 59.62 | 13.12 | 9.77 | 8.69 | 21.74 |
| Bleak | 1141 | 1 | 2 | 3 | 66.7 | 14.59 | 5.78 | 8.00 | 20.20 | 21.06 | 19.41 | 43.37 | 13.17 | 52.72 | 17.74 | 58.89 | 13.05 | 11.34 | 8.52 | 26.22 |
| Bleak | 1142 | 1 | 2 | 3 | 107.8 | 14.11 | 5.27 | 7.16 | 19.53 | 21.41 | 18.35 | 41.17 | 13.37 | 51.49 | 17.06 | 61.75 | 12.05 | 7.98 | 8.79 | 21.33 |
| Bleak | 1143 | 1 | 2 | 3 | 87.71 | 14.09 | 4.51 | 7.60 | 19.07 | 20.45 | 18.39 | 39.30 | 13.90 | 48.94 | 18.98 | 58.87 | 13.64 | 13.76 | 7.96 | 20.93 |
| Bleak | 1144 | 1 | 2 | 3 | 93.24 | 14.15 | 5.78 | 7.89 | 18.88 | 21.33 | 17.42 | 43.30 | 12.56 | 52.42 | 16.88 | 60.69 | 12.46 | 13.07 | 8.50 | 22.20 |
| Bleak | 1145 | 1 | 2 | 3 | 76.98 | 14.78 | 5.87 | 9.07 | 19.56 | 21.09 | 19.65 | 42.68 | 14.76 | 48.53 | 20.64 | 60.98 | 13.15 | 13.08 | 9.56 | 23.44 |
| Bleak | 1146 | 1 | 2 | 3 | 104.22 | 15.01 | 4.62 | 6.88 | 19.81 | 20.92 | 18.04 | 41.38 | 14.37 | 51.47 | 17.00 | 61.09 | 14.82 | 10.46 | 9.06 | 22.58 |
| Bleak | 1147 | 1 | 2 | 3 | 106.84 | 14.66 | 5.58 | 8.26 | 19.95 | 21.74 | 17.94 | 43.16 | 12.04 | 49.40 | 17.44 | 60.77 | 14.27 | 12.86 | 8.45 | 22.56 |
| Bleak | 1148 | 1 | 2 | 3 | 103.58 | 14.07 | 5.61 | 8.18 | 19.83 | 21.15 | 17.89 | 42.34 | 13.60 | 52.11 | 17.06 | 59.57 | 12.30 | 13.39 | 7.89 | 22.79 |
| Bleak | 1149 | 1 | 2 | 3 | 104.03 | 14.43 | 5.89 | 7.46 | 19.30 | 20.61 | 18.93 | 42.21 | 13.68 | 51.13 | 12.55 | 60.68 | 12.77 | 11.73 | 8.94 | 24.20 |
| Bleak | 1150 | 1 | 2 | 3 | 88.88 | 14.55 | 4.88 | 7.55 | 19.83 | 22.18 | 18.85 | 41.89 | 14.30 | 51.35 | 17.72 | 60.44 | 12.28 | 7.41 | 8.46 | 23.85 |
| Bleak | 1151 | 1 | 2 | 3 | 82.77 | 15.17 | 4.84 | 8.32 | 19.90 | 21.43 | 18.24 | 41.84 | 14.09 | 48.73 | 17.99 | 60.65 | 13.29 | 12.21 | 8.93 | 23.21 |
| Bleak | 1152 | 1 | 2 | 3 | 101.41 | 14.35 | 5.49 | 8.10 | 19.56 | 21.59 | 16.80 | 43.34 | 12.26 | 51.31 | 17.11 | 60.96 | 12.44 | 11.43 | 8.05 | 22.24 |
| Bleak | 1153 | 1 | 2 | 3 | 108.16 | 13.88 | 5.10 | 8.21 | 18.16 | 20.20 | 19.31 | 41.60 | 13.92 | 51.09 | 18.62 | 58.71 | 13.58 | 11.42 | 8.16 | 21.48 |
| Bleak | 1154 | 1 | 2 | 3 | 80.41 | 14.94 | 5.68 | 8.50 | 18.98 | 16.80 | 17.42 | 45.03 | 13.26 | 49.51 | 18.21 | 59.00 | 13.74 | 12.25 | 9.87 | 21.68 |
| Bleak | 1155 | 1 | 2 | 3 | 104.68 | 14.80 | 5.72 | 7.30 | 21.56 | 23.40 | 17.36 | 42.19 | 14.72 | 51.12 | 19.82 | 60.21 | 13.58 | 12.07 | 9.39 | 24.43 |
| Bleak | 1156 | 1 | 2 | 3 | 97.34 | 14.69 | 5.16 | 8.66 | 19.55 | 20.93 | 18.48 | 42.14 | 13.47 | 50.33 | 17.44 | 60.44 | 12.68 | 11.41 | 8.26 | 18.25 |
| Bleak | 1157 | 1 | 2 | 3 | 101.85 | 13.84 | 5.31 | 8.63 | 19.01 | 20.19 | 19.32 | 43.03 | 14.08 | 54.28 | 17.91 | 62.68 | 13.21 | 13.24 | 9.04 | 21.63 |
| Bleak | 1158 | 1 | 2 | 3 | 89.8 | 15.83 | 5.92 | 7.91 | 19.75 | 19.99 | 17.92 | 41.46 | 12.82 | 48.12 | 18.61 | 60.16 | 13.33 | 11.42 | 8.88 | 22.40 |
| Bleak | 1159 | 1 | 2 | 3 | 74.38 | 15.39 | 4.74 | 8.88 | 20.12 | 22.10 | 18.12 | 42.90 | 17.02 | 51.53 | 18.29 | 59.44 | 13.82 | 12.06 | 9.89 | 23.70 |
| Bleak | 1160 | 1 | 2 | 3 | 91.46 | 15.37 | 4.95 | 7.57 | 19.02 | 20.66 | 17.95 | 40.50 | 12.48 | 50.33 | 13.61 | 61.55 | 12.76 | 14.88 | 7.96 | 20.65 |
| Bleak | 1221 | 1 | 3 | 1 | 103.4 | 16.79 | 5.46 | 7.74 | 18.94 | 21.58 | 17.74 | 42.47 | 13.35 | 54.61 | 17.08 | 62.81 | 11.81 | 14.31 | 9.52 | 24.15 |
| Bleak | 1222 | 1 | 3 | 1 | 86.4 | 13.81 | 5.15 | 8.50 | 19.34 | 20.33 | 19.20 | 39.72 | 14.12 | 52.02 | 16.93 | 60.53 | 13.29 | 14.07 | 8.22 | 21.39 |
| Bleak | 1223 | 1 | 3 | 1 | 87.26 | 14.45 | 6.04 | 8.59 | 18.55 | 21.40 | 18.71 | 42.17 | 13.31 | 52.75 | 18.16 | 60.21 | 12.63 | 14.79 | 8.53 | 21.84 |
| Bleak | 1224 | 1 | 3 | 1 | 103.3 | 14.72 | 5.12 | 8.23 | 18.62 | 20.97 | 17.75 | 44.33 | 13.33 | 50.77 | 18.23 | 59.89 | 13.16 | 18.17 | 9.37 | 22.64 |
| Bleak | 1225 | 1 | 3 | 1 | 100.57 | 14.75 | 5.52 | 8.61 | 20.39 | 21.85 | 18.40 | 42.04 | 13.81 | 49.97 | 17.87 | 62.34 | 13.68 | 15.57 | 8.86 | 20.73 |
| Bleak | 1226 | 1 | 3 | 1 | 77.52 | 13.87 | 4.59 | 9.05 | 19.82 | 21.49 | 18.12 | 40.09 | 14.37 | 51.89 | 18.80 | 59.84 | 12.85 | 13.90 | 8.89 | 21.20 |
| Bleak | 1227 | 1 | 3 | 1 | 84.32 | 15.05 | 4.41 | 8.52 | 21.30 | 22.48 | 19.06 | 44.12 | 14.84 | 52.47 | 17.83 | 61.71 | 13.87 | 15.26 | 8.86 | 22.63 |
| Bleak | 1228 | 1 | 3 | 1 | 87.86 | 15.56 | 6.15 | 9.39 | 16.59 | 22.50 | 17.04 | 39.85 | 13.53 | 53.93 | 15.81 | 61.63 | 11.57 | 13.26 | 8.87 | 21.14 |
| Bleak | 1229 | 1 | 3 | 1 | 87.36 | 14.94 | 5.46 | 8.82 | 19.25 | 21.73 | 17.72 | 37.63 | 13.01 | 49.84 | 18.50 | 58.76 | 13.27 | 11.41 | 8.79 | 21.98 |
| Bleak | 1230 | 1 | 3 | 1 | 100.29 | 14.09 | 5.22 | 8.25 | 18.52 | 20.93 | 18.28 | 40.63 | 12.68 | 51.43 | 16.59 | 60.49 | 13.62 | 13.88 | 9.30 | 22.42 |
| Bleak | 1231 | 1 | 3 | 1 | 86.71 | 14.11 | 6.08 | 9.03 | 20.55 | 21.32 | 20.60 | 42.43 | 15.47 | 51.65 | 18.81 | 61.32 | 13.34 | 11.56 | 9.36 | 24.06 |
| Bleak | 1232 | 1 | 3 | 1 | 127.99 | 14.80 | 6.11 | 8.91 | 19.92 | 22.38 | 18.25 | 43.05 | 13.66 | 53.49 | 17.84 | 60.83 | 13.15 | 14.48 | 9.18 | 22.74 |
| Bleak | 1233 | 1 | 3 | 1 | 93.38 | 13.87 | 6.55 | 9.23 | 20.52 | 25.82 | 16.79 | 41.80 | 12.69 | 53.35 | 17.06 | 54.85 | 11.34 | 12.65 | 9.39 | 23.56 |
| Bleak | 1234 | 1 | 3 | 1 | 96.14 | 14.34 | 6.18 | 8.68 | 19.37 | 20.71 | 19.43 | 41.70 | 13.77 | 50.77 | 17.83 | 62.74 | 13.17 | 12.99 | 8.30 | 20.89 |
| Bleak | 1235 | 1 | 3 | 1 | 93.49 | 13.81 | 5.56 | 9.91 | 20.69 | 22.72 | 17.87 | 41.48 | 13.60 | 51.75 | 18.87 | 59.84 | 13.26 | 12.38 | 8.82 | 21.51 |
| Bleak | 1236 | 1 | 3 | 1 | 85.68 | 15.41 | 5.58 | 8.23 | 20.54 | 23.01 | 18.90 | 40.51 | 13.37 | 52.81 | 19.62 | 60.23 | 12.36 | 10.89 | 8.87 | 22.57 |
| Bleak | 1237 | 1 | 3 | 1 | 98.76 | 14.55 | 5.24 | 8.45 | 20.34 | 22.28 | 17.82 | 42.83 | 12.51 | 51.59 | 16.43 | 61.25 | 11.51 | 14.50 | 8.61 | 21.98 |
| Bleak | 1238 | 1 | 3 | 1 | 98.94 | 15.47 | 5.76 | 9.10 | 21.40 | 23.57 | 19.19 | 43.91 | 14.27 | 53.36 | 20.12 | 63.02 | 13.22 | 11.37 | 9.25 | 23.59 |
| Bleak | 1239 | 1 | 3 | 1 | 93.51 | 13.92 | 5.87 | 9.72 | 19.97 | 21.08 | 19.68 | 38.98 | 14.23 | 51.58 | 17.48 | 59.80 | 13.72 | 11.92 | 8.88 | 22.30 |
| Bleak | 1240 | 1 | 3 | 1 | 90.71 | 15.87 | 5.27 | 8.89 | 19.46 | 20.40 | 18.60 | 38.48 | 13.12 | 53.04 | 16.42 | 59.42 | 12.83 | 15.91 | 9.87 | 21.34 |
| Bleak | 1241 | 1 | 3 | 1 | 106.52 | 16.23 | 6.30 | 8.87 | 20.90 | 23.12 | 19.16 | 42.80 | 15.03 | 45.47 | 20.28 | 61.89 | 15.09 | 15.02 | 8.73 | 23.36 |
| Bleak | 1242 | 1 | 3 | 1 | 99.5 | 14.06 | 4.83 | 8.86 | 18.52 | 20.95 | 18.58 | 41.02 | 13.19 | 51.25 | 18.54 | 61.82 | 12.91 | 13.40 | 9.12 | 23.04 |
| Bleak | 1243 | 1 | 3 | 1 | 99.05 | 14.87 | 6.28 | 8.25 | 19.37 | 20.47 | 16.97 | 40.98 | 14.23 | 52.17 | 19.24 | 59.67 | 13.39 | 13.97 | 9.70 | 23.35 |
| Bleak | 1244 | 1 | 3 | 1 | 87.61 | 14.80 | 5.83 | 8.72 | 20.60 | 24.74 | 18.77 | 45.05 | 14.58 | 56.00 | 19.53 | 63.07 | 12.84 | 13.14 | 9.06 | 23.52 |
| Bleak | 1245 | 1 | 3 | 1 | 91.77 | 14.22 | 6.01 | 9.28 | 19.56 | 20.75 | 20.23 | 40.76 | 11.12 | 53.08 | 18.28 | 63.93 | 14.66 | 15.76 | 9.80 | 22.59 |
| Bleak | 1246 | 1 | 3 | 1 | 90.04 | 15.77 | 5.83 | 9.61 | 20.12 | 22.34 | 19.07 | 47.28 | 15.02 | 51.79 | 19.30 | 58.64 | 13.85 | 16.34 | 9.11 | 22.64 |
| Bleak | 1247 | 1 | 3 | 1 | 95.4 | 14.51 | 6.28 | 7.03 | 20.79 | 19.20 | 16.94 | 40.58 | 13.62 | 49.83 | 17.33 | 59.23 | 14.20 | 13.72 | 9.54 | 22.30 |
| Bleak | 1248 | 1 | 3 | 1 | 61.81 | 15.76 | 6.79 | 10.05 | 19.35 | 20.98 | 19.77 | 43.94 | 13.81 | 51.99 | 19.69 | 61.49 | 14.62 | 11.76 | 8.94 | 20.96 |
| Bleak | 1249 | 1 | 3 | 1 | 73.92 | 13.22 | 5.28 | 8.24 | 19.81 | 20.77 | 19.50 | 42.92 | 15.82 | 50.83 | 20.42 | 60.01 | 13.58 | 14.03 | 8.76 | 21.43 |
| Bleak | 1250 | 1 | 3 | 1 | 82.14 | 15.09 | 5.57 | 9.10 | 17.24 | 18.00 | 17.51 | 40.98 | 12.67 | 52.32 | 18.71 | 61.10 | 13.85 | 13.38 | 8.56 | 22.66 |
| Bleak | 1221 | 1 | 3 | 2 | 102.36 | 16.10 | 5.60 | 9.03 | 19.75 | 21.92 | 17.45 | 40.72 | 13.21 | 52.28 | 17.79 | 62.04 | 12.61 | 12.06 | 9.09 | 23.97 |
| Bleak | 1222 | 1 | 3 | 2 | 86.77 | 14.60 | 4.63 | 8.61 | 18.67 | 20.39 | 19.31 | 42.58 | 13.75 | 52.66 | 19.87 | 60.94 | 12.38 | 11.43 | 7.82 | 21.40 |
| Bleak | 1223 | 1 | 3 | 2 | 84.56 | 14.38 | 5.27 | 9.36 | 20.91 | 23.07 | 18.53 | 44.04 | 13.96 | 53.59 | 16.33 | 60.67 | 11.79 | 15.94 | 7.85 | 21.93 |
| Bleak | 1224 | 1 | 3 | 2 | 105.31 | 14.46 | 5.46 | 8.33 | 20.24 | 22.00 | 18.78 | 40.33 | 13.10 | 50.59 | 16.64 | 59.84 | 11.71 | 13.43 | 8.47 | 21.33 |
| Bleak | 1225 | 1 | 3 | 2 | 100.8 | 14.76 | 5.70 | 8.73 | 21.36 | 22.17 | 18.48 | 40.65 | 14.11 | 47.94 | 18.21 | 63.20 | 13.53 | 12.27 | 8.81 | 20.81 |
| Bleak | 1226 | 1 | 3 | 2 | 77.62 | 14.94 | 5.10 | 9.32 | 19.93 | 21.67 | 20.73 | 41.37 | 14.72 | 50.39 | 17.91 | 60.19 | 12.69 | 12.26 | 8.72 | 21.76 |
| Bleak | 1227 | 1 | 3 | 2 | 87.47 | 15.43 | 5.18 | 8.14 | 21.39 | 22.35 | 19.90 | 39.77 | 14.14 | 51.13 | 18.66 | 64.35 | 13.77 | 12.36 | 8.90 | 22.05 |
| Bleak | 1228 | 1 | 3 | 2 | 91.03 | 14.31 | 5.29 | 9.18 | 19.42 | 21.33 | 16.09 | 51.31 | 12.53 | 50.07 | 15.63 | 57.98 | 12.18 | 13.90 | 8.19 | 21.26 |
| Bleak | 1229 | 1 | 3 | 2 | 87.5 | 15.17 | 5.00 | 7.75 | 20.57 | 21.66 | 17.76 | 37.16 | 14.18 | 49.92 | 19.19 | 60.16 | 13.53 | 13.27 | 8.65 | 20.95 |
| Bleak | 1230 | 1 | 3 | 2 | 100.48 | 14.38 | 5.32 | 8.76 | 20.09 | 21.95 | 18.25 | 41.82 | 13.98 | 50.82 | 17.49 | 59.91 | 13.32 | 10.60 | 9.67 | 22.73 |
| Bleak | 1231 | 1 | 3 | 2 | 86.98 | 14.10 | 6.04 | 8.82 | 20.37 | 21.07 | 18.31 | 39.48 | 16.34 | 51.96 | 18.09 | 61.33 | 13.46 | 11.18 | 9.20 | 23.23 |
| Bleak | 1232 | 1 | 3 | 2 | 128.15 | 14.67 | 6.00 | 8.43 | 19.95 | 21.67 | 18.59 | 43.07 | 14.17 | 52.52 | 18.29 | 59.84 | 13.58 | 13.35 | 8.97 | 22.63 |
| Bleak | 1233 | 1 | 3 | 2 | 94.31 | 14.88 | 5.44 | 8.62 | 21.11 | 22.13 | 18.88 | 40.13 | 13.51 | 52.78 | 17.02 | 59.80 | 12.46 | 13.27 | 8.95 | 22.49 |
| Bleak | 1234 | 1 | 3 | 2 | 94.61 | 14.69 | 4.97 | 7.57 | 19.28 | 20.57 | 19.92 | 40.54 | 14.00 | 50.78 | 17.98 | 64.12 | 15.19 | 15.54 | 9.19 | 21.55 |
| Bleak | 1235 | 1 | 3 | 2 | 93.77 | 14.94 | 5.64 | 8.67 | 21.40 | 22.27 | 17.52 | 40.46 | 14.40 | 50.73 | 18.52 | 62.53 | 12.33 | 11.60 | 8.68 | 21.31 |
| Bleak | 1236 | 1 | 3 | 2 | 86.22 | 15.29 | 5.12 | 8.57 | 19.18 | 21.12 | 17.83 | 42.65 | 14.07 | 52.28 | 19.67 | 60.86 | 13.57 | 12.58 | 8.83 | 21.35 |
| Bleak | 1237 | 1 | 3 | 2 | 97.55 | 14.67 | 5.86 | 9.32 | 20.32 | 22.32 | 18.45 | 43.61 | 13.60 | 52.82 | 18.05 | 61.84 | 11.69 | 13.69 | 8.52 | 22.47 |
| Bleak | 1238 | 1 | 3 | 2 | 99.72 | 15.35 | 5.70 | 9.18 | 18.45 | 20.21 | 19.36 | 42.23 | 14.09 | 52.61 | 19.18 | 63.69 | 13.22 | 12.05 | 8.85 | 23.64 |
| Bleak | 1239 | 1 | 3 | 2 | 92.97 | 14.50 | 6.24 | 8.77 | 23.70 | 23.44 | 19.80 | 44.39 | 13.61 | 51.88 | 19.00 | 61.24 | 12.82 | 10.88 | 9.18 | 22.28 |
| Bleak | 1240 | 1 | 3 | 2 | 90.11 | 15.33 | 5.61 | 8.43 | 21.41 | 22.06 | 17.74 | 40.07 | 12.81 | 51.19 | 18.03 | 58.29 | 13.22 | 13.54 | 9.55 | 22.01 |
| Bleak | 1241 | 1 | 3 | 2 | 108.41 | 14.72 | 5.24 | 8.21 | 21.28 | 22.30 | 19.73 | 39.92 | 15.18 | 52.10 | 19.62 | 60.67 | 14.15 | 12.69 | 8.32 | 22.91 |
| Bleak | 1242 | 1 | 3 | 2 | 101.12 | 14.42 | 5.53 | 9.70 | 19.75 | 20.23 | 21.31 | 41.78 | 14.84 | 50.63 | 19.70 | 62.72 | 14.12 | 14.62 | 8.69 | 22.76 |
| Bleak | 1243 | 1 | 3 | 2 | 99.19 | 14.64 | 5.68 | 8.15 | 18.75 | 20.32 | 18.75 | 42.72 | 14.47 | 47.99 | 18.43 | 59.22 | 13.86 | 13.14 | 9.45 | 23.99 |
| Bleak | 1244 | 1 | 3 | 2 | 90.03 | 15.23 | 5.33 | 10.15 | 21.46 | 24.89 | 20.01 | 40.68 | 15.77 | 53.96 | 16.85 | 59.79 | 13.79 | 12.45 | 8.41 | 22.38 |
| Bleak | 1245 | 1 | 3 | 2 | 91.49 | 14.88 | 6.02 | 8.72 | 18.77 | 19.07 | 20.17 | 39.01 | 16.08 | 50.02 | 20.89 | 60.82 | 14.77 | 14.71 | 9.91 | 23.34 |
| Bleak | 1246 | 1 | 3 | 2 | 91.1 | 14.74 | 5.34 | 9.05 | 21.02 | 22.10 | 18.56 | 39.29 | 16.26 | 50.37 | 18.43 | 55.91 | 11.17 | 12.79 | 8.70 | 20.92 |
| Bleak | 1247 | 1 | 3 | 2 | 95.74 | 14.92 | 5.64 | 8.66 | 19.61 | 21.78 | 18.57 | 40.90 | 14.17 | 60.34 | 18.39 | 61.80 | 12.89 | 12.61 | 8.64 | 20.83 |
| Bleak | 1248 | 1 | 3 | 2 | 61.06 | 15.67 | 5.60 | 8.40 | 21.23 | 23.06 | 20.01 | 44.89 | 17.25 | 53.60 | 18.65 | 65.16 | 13.69 | 13.17 | 8.03 | 22.17 |
| Bleak | 1249 | 1 | 3 | 2 | 75.32 | 14.96 | 5.74 | 8.17 | 20.16 | 21.89 | 19.04 | 39.48 | 14.27 | 50.36 | 19.85 | 61.34 | 13.31 | 13.84 | 8.49 | 21.59 |
| Bleak | 1250 | 1 | 3 | 2 | 82.04 | 17.38 | 5.90 | 8.78 | 19.72 | 23.28 | 19.21 | 42.94 | 16.21 | 50.64 | 18.76 | 59.84 | 13.36 | 11.99 | 8.88 | 21.34 |
| Bleak | 1221 | 1 | 3 | 3 | 102.83 | 15.83 | 4.88 | 9.21 | 20.15 | 21.87 | 17.68 | 42.54 | 16.35 | 50.56 | 17.47 | 60.05 | 12.59 | 11.17 | 9.42 | 22.94 |
| Bleak | 1222 | 1 | 3 | 3 | 86.36 | 14.47 | 5.25 | 7.84 | 18.87 | 21.05 | 19.07 | 41.33 | 12.28 | 52.41 | 16.89 | 61.85 | 13.25 | 11.72 | 8.46 | 21.00 |
| Bleak | 1223 | 1 | 3 | 3 | 86.59 | 15.19 | 5.10 | 8.37 | 20.97 | 23.09 | 19.41 | 41.89 | 14.73 | 51.79 | 18.66 | 60.05 | 13.10 | 15.31 | 8.06 | 21.69 |
| Bleak | 1224 | 1 | 3 | 3 | 102.68 | 14.86 | 5.15 | 8.58 | 20.12 | 21.70 | 18.42 | 44.22 | 13.57 | 50.69 | 17.27 | 60.26 | 11.26 | 11.22 | 8.28 | 21.80 |
| Bleak | 1225 | 1 | 3 | 3 | 100.3 | 14.69 | 5.35 | 8.98 | 20.60 | 21.56 | 18.22 | 43.12 | 14.05 | 48.92 | 17.70 | 62.73 | 13.98 | 13.99 | 8.71 | 21.25 |
| Bleak | 1226 | 1 | 3 | 3 | 77.6 | 15.05 | 5.06 | 9.16 | 19.31 | 21.34 | 18.06 | 42.07 | 13.56 | 50.39 | 16.59 | 60.91 | 11.65 | 11.88 | 8.33 | 23.26 |
| Bleak | 1227 | 1 | 3 | 3 | 87.41 | 15.56 | 4.67 | 9.87 | 20.29 | 21.40 | 20.51 | 42.56 | 15.19 | 54.25 | 19.87 | 65.83 | 14.12 | 12.54 | 9.11 | 24.02 |
| Bleak | 1228 | 1 | 3 | 3 | 90.62 | 15.05 | 5.79 | 8.84 | 19.82 | 20.26 | 16.92 | 40.94 | 14.46 | 50.50 | 15.35 | 58.53 | 13.36 | 15.76 | 8.29 | 21.33 |
| Bleak | 1229 | 1 | 3 | 3 | 87.22 | 15.66 | 4.53 | 8.34 | 17.75 | 19.29 | 20.59 | 40.97 | 13.65 | 47.93 | 18.47 | 58.01 | 12.67 | 11.10 | 8.36 | 22.07 |
| Bleak | 1230 | 1 | 3 | 3 | 100.25 | 14.68 | 5.45 | 9.14 | 20.72 | 22.33 | 17.66 | 42.15 | 15.17 | 51.86 | 17.46 | 60.73 | 13.47 | 11.49 | 9.06 | 22.06 |
| Bleak | 1231 | 1 | 3 | 3 | 87.35 | 15.21 | 5.10 | 8.57 | 20.00 | 21.15 | 19.56 | 41.29 | 15.24 | 51.94 | 18.25 | 60.43 | 12.55 | 10.45 | 9.30 | 22.96 |
| Bleak | 1232 | 1 | 3 | 3 | 128.06 | 15.08 | 5.37 | 8.02 | 19.87 | 22.17 | 17.09 | 44.39 | 13.76 | 52.71 | 18.98 | 60.50 | 12.66 | 12.87 | 8.84 | 22.88 |
| Bleak | 1233 | 1 | 3 | 3 | 93.69 | 13.75 | 5.26 | 8.79 | 20.88 | 21.86 | 19.01 | 41.09 | 13.02 | 53.88 | 16.36 | 60.23 | 12.14 | 10.86 | 9.33 | 21.76 |
| Bleak | 1234 | 1 | 3 | 3 | 95.34 | 13.77 | 4.88 | 7.79 | 18.97 | 19.50 | 19.03 | 42.75 | 16.76 | 48.80 | 17.88 | 61.37 | 13.19 | 12.25 | 8.43 | 24.53 |
| Bleak | 1235 | 1 | 3 | 3 | 93.32 | 13.87 | 4.90 | 9.10 | 20.17 | 21.67 | 19.25 | 39.89 | 14.50 | 49.57 | 17.73 | 61.67 | 12.27 | 13.03 | 8.48 | 21.52 |
| Bleak | 1236 | 1 | 3 | 3 | 86.71 | 15.15 | 4.37 | 7.91 | 19.02 | 19.55 | 18.18 | 41.02 | 14.18 | 50.07 | 19.53 | 60.02 | 13.11 | 11.59 | 8.63 | 21.95 |
| Bleak | 1237 | 1 | 3 | 3 | 97.96 | 15.16 | 5.11 | 8.93 | 20.15 | 21.68 | 18.01 | 41.82 | 13.68 | 51.66 | 17.43 | 62.21 | 10.70 | 13.02 | 8.52 | 22.39 |
| Bleak | 1238 | 1 | 3 | 3 | 101.3 | 15.10 | 5.77 | 10.15 | 20.24 | 22.49 | 19.11 | 45.30 | 14.71 | 52.21 | 19.37 | 62.44 | 12.72 | 12.36 | 8.83 | 23.53 |
| Bleak | 1239 | 1 | 3 | 3 | 93.5 | 14.50 | 5.08 | 9.34 | 21.41 | 22.94 | 19.15 | 43.27 | 13.49 | 51.52 | 19.01 | 58.90 | 11.41 | 9.87 | 8.76 | 21.83 |
| Bleak | 1240 | 1 | 3 | 3 | 90.37 | 14.75 | 6.09 | 8.43 | 20.21 | 22.05 | 17.01 | 38.72 | 12.93 | 51.44 | 14.50 | 60.53 | 12.05 | 14.70 | 9.05 | 21.45 |
| Bleak | 1241 | 1 | 3 | 3 | 109.45 | 14.85 | 4.59 | 7.90 | 21.07 | 22.37 | 19.82 | 38.45 | 14.70 | 50.89 | 17.90 | 59.06 | 14.33 | 11.85 | 8.21 | 21.44 |
| Bleak | 1242 | 1 | 3 | 3 | 100.51 | 13.38 | 5.45 | 9.32 | 19.33 | 21.57 | 18.85 | 41.19 | 13.01 | 51.39 | 20.59 | 63.54 | 12.38 | 11.84 | 8.99 | 22.29 |
| Bleak | 1243 | 1 | 3 | 3 | 101.48 | 14.61 | 5.39 | 7.18 | 18.31 | 20.79 | 18.34 | 41.68 | 13.64 | 47.92 | 16.55 | 58.12 | 12.25 | 14.13 | 9.55 | 22.30 |
| Bleak | 1244 | 1 | 3 | 3 | 87.18 | 16.25 | 5.78 | 9.70 | 22.05 | 25.11 | 19.30 | 43.27 | 13.85 | 55.70 | 17.82 | 61.69 | 12.58 | 12.23 | 8.63 | 23.67 |
| Bleak | 1245 | 1 | 3 | 3 | 91.39 | 15.26 | 4.85 | 8.70 | 18.92 | 20.45 | 19.94 | 39.53 | 15.21 | 50.67 | 17.91 | 59.56 | 12.66 | 14.42 | 10.11 | 22.99 |
| Bleak | 1246 | 1 | 3 | 3 | 90.01 | 14.85 | 5.06 | 9.67 | 20.81 | 21.92 | 18.71 | 41.06 | 14.30 | 53.01 | 18.33 | 61.63 | 13.19 | 10.73 | 8.92 | 22.11 |
| Bleak | 1247 | 1 | 3 | 3 | 95.61 | 14.81 | 5.38 | 7.84 | 19.36 | 22.34 | 17.13 | 40.56 | 14.06 | 51.05 | 17.08 | 60.94 | 11.82 | 13.01 | 8.84 | 21.91 |
| Bleak | 1248 | 1 | 3 | 3 | 62.44 | 15.47 | 6.40 | 9.41 | 20.64 | 21.48 | 19.49 | 42.42 | 14.14 | 51.86 | 17.65 | 61.47 | 12.63 | 11.30 | 8.58 | 21.57 |
| Bleak | 1249 | 1 | 3 | 3 | 74.25 | 14.67 | 5.30 | 8.50 | 20.61 | 22.65 | 19.12 | 40.74 | 13.34 | 51.73 | 17.83 | 62.16 | 14.33 | 14.73 | 8.90 | 21.12 |
| Bleak | 1250 | 1 | 3 | 3 | 82.93 | 13.91 | 4.76 | 9.26 | 20.79 | 22.63 | 18.46 | 41.64 | 13.35 | 51.75 | 19.33 | 60.06 | 14.45 | 11.01 | 9.21 | 22.68 |
| Bleak | 1031 | 2 | 1 | 1 | 98.06 | 14.568 | 5.076 | 9.477 | 19.818 | 19.134 | 19.887 | 37.984 | 15.602 | 50.844 | 19.718 | 59.183 | 15.442 | 19.018 | 9.530 | 22.555 |
| Bleak | 1032 | 2 | 1 | 1 | 124.75 | 13.623 | 3.969 | 9.585 | 19.573 | 19.615 | 19.434 | 41.004 | 16.300 | 50.202 | 18.519 | 60.336 | 13.530 | 18.706 | 7.875 | 20.975 |
| Bleak | 1033 | 2 | 1 | 1 | 130.43 | 15.861 | 5.793 | 10.212 | 20.611 | 20.352 | 19.741 | 40.627 | 15.213 | 53.251 | 17.789 | 61.315 | 14.292 | 18.556 | 8.769 | 22.559 |
| Bleak | 1034 | 2 | 1 | 1 | 97.25 | 15.107 | 4.612 | 9.253 | 20.038 | 18.840 | 18.172 | 41.321 | 15.639 | 52.900 | 17.595 | 60.255 | 14.280 | 19.180 | 9.256 | 22.182 |
| Bleak | 1035 | 2 | 1 | 1 | 78.66 | 14.657 | 5.944 | 10.423 | 22.155 | 20.948 | 18.239 | 41.366 | 12.597 | 54.866 | 15.821 | 61.668 | 13.481 | 19.409 | 8.626 | 22.617 |
| Bleak | 1036 | 2 | 1 | 1 | 122.15 | 14.137 | 5.157 | 10.220 | 21.178 | 20.501 | 19.168 | 38.486 | 14.563 | 50.457 | 18.103 | 59.392 | 13.711 | 19.705 | 9.426 | 22.322 |
| Bleak | 1037 | 2 | 1 | 1 | 106.72 | 12.978 | 4.752 | 8.896 | 19.400 | 18.872 | 19.878 | 39.596 | 14.149 | 52.880 | 18.044 | 58.604 | 13.831 | 17.590 | 8.953 | 21.592 |
| Bleak | 1038 | 2 | 1 | 1 | 103.38 | 13.792 | 5.569 | 9.713 | 20.882 | 20.142 | 18.129 | 40.953 | 14.086 | 52.298 | 19.177 | 61.061 | 13.394 | 19.876 | 9.688 | 25.065 |
| Bleak | 1039 | 2 | 1 | 1 | 91.53 | 14.178 | 5.289 | 10.481 | 21.726 | 20.926 | 19.070 | 40.835 | 14.578 | 50.448 | 18.395 | 61.569 | 13.117 | 18.212 | 9.294 | 23.076 |
| Bleak | 1040 | 2 | 1 | 1 | 128.63 | 15.241 | 5.136 | 7.901 | 22.040 | 22.440 | 21.060 | 41.881 | 14.729 | 51.780 | 19.570 | 61.386 | 13.191 | 20.546 | 9.283 | 23.306 |
| Bleak | 1041 | 2 | 1 | 1 | 81.29 | 14.814 | 5.162 | 9.171 | 21.651 | 20.978 | 18.154 | 42.331 | 12.748 | 52.163 | 18.912 | 59.930 | 15.332 | 18.640 | 8.658 | 22.324 |
| Bleak | 1042 | 2 | 1 | 1 | 102.7 | 14.493 | 4.326 | 9.812 | 19.974 | 19.429 | 18.753 | 43.825 | 15.012 | 52.402 | 19.526 | 60.509 | 14.861 | 18.354 | 9.199 | 22.616 |
| Bleak | 1043 | 2 | 1 | 1 | 110.65 | 15.002 | 5.192 | 10.487 | 20.600 | 19.972 | 20.086 | 40.811 | 15.514 | 51.207 | 19.292 | 59.248 | 13.816 | 17.008 | 9.298 | 22.495 |
| Bleak | 1044 | 2 | 1 | 1 | 94.01 | 13.746 | 5.183 | 9.166 | 20.479 | 20.276 | 19.347 | 42.882 | 15.032 | 51.005 | 19.338 | 60.222 | 13.876 | 21.103 | 8.639 | 19.650 |
| Bleak | 1045 | 2 | 1 | 1 | 99.26 | 16.133 | 5.569 | 9.128 | 19.900 | 17.637 | 17.803 | 38.173 | 14.318 | 49.729 | 18.303 | 59.852 | 13.274 | 18.386 | 8.726 | 23.385 |
| Bleak | 1046 | 2 | 1 | 1 | 118.49 | 14.850 | 5.211 | 10.926 | 21.769 | 21.001 | 20.354 | 42.136 | 16.697 | 51.535 | 18.633 | 61.556 | 14.375 | 17.042 | 9.047 | 20.653 |
| Bleak | 1047 | 2 | 1 | 1 | 117.1 | 14.799 | 5.022 | 10.660 | 21.936 | 21.610 | 19.441 | 44.166 | 14.870 | 50.183 | 20.185 | 61.864 | 14.988 | 20.753 | 9.282 | 21.978 |
| Bleak | 1048 | 2 | 1 | 1 | 99.53 | 15.380 | 5.816 | 10.756 | 22.181 | 21.817 | 19.674 | 42.621 | 15.511 | 53.575 | 19.208 | 60.761 | 13.137 | 19.529 | 9.524 | 21.808 |
| Bleak | 1049 | 2 | 1 | 1 | 124.46 | 14.825 | 4.609 | 9.899 | 20.994 | 20.900 | 19.062 | 41.948 | 15.516 | 54.746 | 16.915 | 64.490 | 13.190 | 19.937 | 9.008 | 24.784 |
| Bleak | 1050 | 2 | 1 | 1 | 93.99 | 14.645 | 5.257 | 9.914 | 20.721 | 20.424 | 18.413 | 39.770 | 15.096 | 52.908 | 17.294 | 61.457 | 14.033 | 18.507 | 8.600 | 20.181 |
| Bleak | 1051 | 2 | 1 | 1 | 114.88 | 13.573 | 5.661 | 9.477 | 20.885 | 20.666 | 19.188 | 42.193 | 16.412 | 51.885 | 16.261 | 61.156 | 14.488 | 19.683 | 9.016 | 19.937 |
| Bleak | 1052 | 2 | 1 | 1 | 119.54 | 14.574 | 5.011 | 9.992 | 20.080 | 19.188 | 19.190 | 41.080 | 15.776 | 51.243 | 19.132 | 59.147 | 14.026 | 19.544 | 9.086 | 22.862 |
| Bleak | 1053 | 2 | 1 | 1 | 112.07 | 14.531 | 5.888 | 10.942 | 22.445 | 22.996 | 20.341 | 43.277 | 16.624 | 52.164 | 19.802 | 63.254 | 14.408 | 19.241 | 8.758 | 22.465 |
| Bleak | 1054 | 2 | 1 | 1 | 90.19 | 14.337 | 5.296 | 9.535 | 19.525 | 20.058 | 18.539 | 39.501 | 15.021 | 50.588 | 19.018 | 60.182 | 13.820 | 17.741 | 8.931 | 22.624 |
| Bleak | 1055 | 2 | 1 | 1 | 100.38 | 15.766 | 5.397 | 10.030 | 20.353 | 21.841 | 18.308 | 40.998 | 14.508 | 49.647 | 18.464 | 61.838 | 13.628 | 18.025 | 9.748 | 23.007 |
| Bleak | 1056 | 2 | 1 | 1 | 76.77 | 14.477 | 5.240 | 9.670 | 20.009 | 17.278 | 16.057 | 42.071 | 13.663 | 52.924 | 18.187 | 59.853 | 14.619 | 20.380 | 8.901 | 21.333 |
| Bleak | 1057 | 2 | 1 | 1 | 82.96 | 15.604 | 4.958 | 10.418 | 21.287 | 19.996 | 19.527 | 40.690 | 14.915 | 50.923 | 18.165 | 58.747 | 12.363 | 19.605 | 8.760 | 21.879 |
| Bleak | 1058 | 2 | 1 | 1 | 100.25 | 16.610 | 5.772 | 11.203 | 23.645 | 23.729 | 20.282 | 44.904 | 15.459 | 54.425 | 19.292 | 65.480 | 15.171 | 19.104 | 9.595 | 24.953 |
| Bleak | 1059 | 2 | 1 | 1 | 97.41 | 14.347 | 5.770 | 9.406 | 20.873 | 20.096 | 19.590 | 40.425 | 12.426 | 53.019 | 17.599 | 61.236 | 12.546 | 16.867 | 9.132 | 20.420 |
| Bleak | 1060 | 2 | 1 | 1 | 99.32 | 13.685 | 4.813 | 10.058 | 19.575 | 18.585 | 18.293 | 39.987 | 14.223 | 52.950 | 17.548 | 60.428 | 12.149 | 19.941 | 9.093 | 23.097 |
| Bleak | 1031 | 2 | 1 | 2 | 97.99 | 15.678 | 5.496 | 8.940 | 20.764 | 20.555 | 19.830 | 40.657 | 14.589 | 53.001 | 18.857 | 58.961 | 14.221 | 18.000 | 9.834 | 23.176 |
| Bleak | 1032 | 2 | 1 | 2 | 125.15 | 14.854 | 4.476 | 9.499 | 20.357 | 19.757 | 19.384 | 42.741 | 14.682 | 51.957 | 18.411 | 59.383 | 12.911 | 17.982 | 7.804 | 21.081 |
| Bleak | 1033 | 2 | 1 | 2 | 128.45 | 15.827 | 4.786 | 10.369 | 20.636 | 21.396 | 20.014 | 43.453 | 14.650 | 53.839 | 18.443 | 61.380 | 13.816 | 17.233 | 8.920 | 22.563 |
| Bleak | 1034 | 2 | 1 | 2 | 96.66 | 15.030 | 4.933 | 10.109 | 21.643 | 21.241 | 19.530 | 40.974 | 13.469 | 53.967 | 16.655 | 62.473 | 13.028 | 16.360 | 9.419 | 22.534 |
| Bleak | 1035 | 2 | 1 | 2 | 77.84 | 14.675 | 5.656 | 10.988 | 21.155 | 20.989 | 18.453 | 42.485 | 12.706 | 54.584 | 16.913 | 61.522 | 13.767 | 19.246 | 8.970 | 21.934 |
| Bleak | 1036 | 2 | 1 | 2 | 121.69 | 14.984 | 4.752 | 9.949 | 20.275 | 21.580 | 18.999 | 39.818 | 12.165 | 50.520 | 18.737 | 55.923 | 13.522 | 20.477 | 9.347 | 22.876 |
| Bleak | 1037 | 2 | 1 | 2 | 104.18 | 14.409 | 4.920 | 9.104 | 19.839 | 20.216 | 19.125 | 39.981 | 15.706 | 53.574 | 17.883 | 60.425 | 13.384 | 17.711 | 9.374 | 25.812 |
| Bleak | 1038 | 2 | 1 | 2 | 105.18 | 15.466 | 5.211 | 9.157 | 21.230 | 22.036 | 16.886 | 43.034 | 14.169 | 52.236 | 18.293 | 61.222 | 12.829 | 17.170 | 9.837 | 24.461 |
| Bleak | 1039 | 2 | 1 | 2 | 91.81 | 15.254 | 5.166 | 9.851 | 21.630 | 21.118 | 18.384 | 42.656 | 14.280 | 51.879 | 18.661 | 62.771 | 13.639 | 18.000 | 9.288 | 22.960 |
| Bleak | 1040 | 2 | 1 | 2 | 130.17 | 14.295 | 5.050 | 10.656 | 20.975 | 21.760 | 20.182 | 43.939 | 15.270 | 52.364 | 20.427 | 59.165 | 13.422 | 21.158 | 8.937 | 22.915 |
| Bleak | 1041 | 2 | 1 | 2 | 80.99 | 15.005 | 5.400 | 10.755 | 21.979 | 22.366 | 19.067 | 42.382 | 13.169 | 53.679 | 18.985 | 61.983 | 14.135 | 17.837 | 9.127 | 22.145 |
| Bleak | 1042 | 2 | 1 | 2 | 101.56 | 15.600 | 5.102 | 9.513 | 21.263 | 22.159 | 19.311 | 44.211 | 15.218 | 51.827 | 18.747 | 59.389 | 13.217 | 17.412 | 9.354 | 23.577 |
| Bleak | 1043 | 2 | 1 | 2 | 111.19 | 14.334 | 5.122 | 9.819 | 20.135 | 20.360 | 18.866 | 41.525 | 15.229 | 51.323 | 18.127 | 58.181 | 13.671 | 16.639 | 8.516 | 22.980 |
| Bleak | 1044 | 2 | 1 | 2 | 93.71 | 13.610 | 4.806 | 9.229 | 19.798 | 18.776 | 18.112 | 40.274 | 15.380 | 51.817 | 17.442 | 57.281 | 13.069 | 18.715 | 8.024 | 18.978 |
| Bleak | 1045 | 2 | 1 | 2 | 98.68 | 14.928 | 5.279 | 8.813 | 20.328 | 21.557 | 17.860 | 41.047 | 14.683 | 51.939 | 16.238 | 59.478 | 12.481 | 18.401 | 9.158 | 22.525 |
| Bleak | 1046 | 2 | 1 | 2 | 117.47 | 14.504 | 4.891 | 10.810 | 21.986 | 21.976 | 19.326 | 40.489 | 14.310 | 52.039 | 17.598 | 59.933 | 14.530 | 19.091 | 9.171 | 20.758 |
| Bleak | 1047 | 2 | 1 | 2 | 118.31 | 15.038 | 5.001 | 10.420 | 22.049 | 22.592 | 19.566 | 43.666 | 14.584 | 50.605 | 19.179 | 61.169 | 14.181 | 20.732 | 9.184 | 22.134 |
| Bleak | 1048 | 2 | 1 | 2 | 101.14 | 14.714 | 4.885 | 9.948 | 22.579 | 21.460 | 20.213 | 43.189 | 13.684 | 51.322 | 18.613 | 59.689 | 13.311 | 17.318 | 9.342 | 21.619 |
| Bleak | 1049 | 2 | 1 | 2 | 124.65 | 16.232 | 4.372 | 9.037 | 20.813 | 18.940 | 17.693 | 42.139 | 13.215 | 52.606 | 20.324 | 62.666 | 14.173 | 18.279 | 8.774 | 23.373 |
| Bleak | 1050 | 2 | 1 | 2 | 93.63 | 14.685 | 5.611 | 9.674 | 20.839 | 20.408 | 18.158 | 41.648 | 13.681 | 52.828 | 17.363 | 61.531 | 12.810 | 18.565 | 8.641 | 19.847 |
| Bleak | 1051 | 2 | 1 | 2 | 114.49 | 14.898 | 5.068 | 10.247 | 20.630 | 20.471 | 19.017 | 41.070 | 13.523 | 52.422 | 16.440 | 59.882 | 14.102 | 19.175 | 8.489 | 20.008 |
| Bleak | 1052 | 2 | 1 | 2 | 122.85 | 15.389 | 5.118 | 9.514 | 19.675 | 20.451 | 18.568 | 38.476 | 15.064 | 50.496 | 17.646 | 59.042 | 12.791 | 17.427 | 8.407 | 22.651 |
| Bleak | 1053 | 2 | 1 | 2 | 112.85 | 16.062 | 5.265 | 10.452 | 21.690 | 21.485 | 18.784 | 42.286 | 13.799 | 51.688 | 18.306 | 60.498 | 13.372 | 21.000 | 8.744 | 21.892 |
| Bleak | 1054 | 2 | 1 | 2 | 89.4 | 14.883 | 5.466 | 9.375 | 19.952 | 20.988 | 19.078 | 41.618 | 14.231 | 52.663 | 18.337 | 57.089 | 13.022 | 20.024 | 8.757 | 22.407 |
| Bleak | 1055 | 2 | 1 | 2 | 99.55 | 15.142 | 5.552 | 9.469 | 20.800 | 20.251 | 18.049 | 41.394 | 11.893 | 52.219 | 18.364 | 61.313 | 11.753 | 16.548 | 9.141 | 22.846 |
| Bleak | 1056 | 2 | 1 | 2 | 76.22 | 14.671 | 5.873 | 9.746 | 20.465 | 19.740 | 18.740 | 42.828 | 12.293 | 53.499 | 17.437 | 60.660 | 12.824 | 18.743 | 9.037 | 21.313 |
| Bleak | 1057 | 2 | 1 | 2 | 77.1 | 15.172 | 5.764 | 10.203 | 22.764 | 22.749 | 20.373 | 44.644 | 13.120 | 56.333 | 19.098 | 64.101 | 13.453 | 20.459 | 9.462 | 22.815 |
| Bleak | 1058 | 2 | 1 | 2 | 101.89 | 17.124 | 4.581 | 11.140 | 23.019 | 22.203 | 19.579 | 45.446 | 13.428 | 57.304 | 20.592 | 62.311 | 14.449 | 17.081 | 9.182 | 24.439 |
| Bleak | 1059 | 2 | 1 | 2 | 96.67 | 13.874 | 5.686 | 9.706 | 21.882 | 22.716 | 18.926 | 42.477 | 12.655 | 53.118 | 17.306 | 61.472 | 13.599 | 17.372 | 8.916 | 20.754 |
| Bleak | 1060 | 2 | 1 | 2 | 97.52 | 15.239 | 5.226 | 9.912 | 20.612 | 21.398 | 19.002 | 41.732 | 13.939 | 50.757 | 18.827 | 63.512 | 12.135 | 18.073 | 8.982 | 22.934 |
| Bleak | 1031 | 2 | 1 | 3 | 97.95 | 14.633 | 4.488 | 9.251 | 20.554 | 20.097 | 19.997 | 38.355 | 14.961 | 54.450 | 19.588 | 60.264 | 14.096 | 18.057 | 9.322 | 22.938 |
| Bleak | 1032 | 2 | 1 | 3 | 123.36 | 16.546 | 5.484 | 10.119 | 21.548 | 20.630 | 19.355 | 44.845 | 15.317 | 51.529 | 17.956 | 61.250 | 14.681 | 17.922 | 9.514 | 21.065 |
| Bleak | 1033 | 2 | 1 | 3 | 125.28 | 15.015 | 5.181 | 9.783 | 22.150 | 22.399 | 20.812 | 43.799 | 14.783 | 54.113 | 17.667 | 62.623 | 13.685 | 16.930 | 9.141 | 23.381 |
| Bleak | 1034 | 2 | 1 | 3 | 95.43 | 14.779 | 4.389 | 9.975 | 20.682 | 22.301 | 18.289 | 43.027 | 15.011 | 53.141 | 18.091 | 62.339 | 13.532 | 16.226 | 9.493 | 22.969 |
| Bleak | 1035 | 2 | 1 | 3 | 77 | 14.388 | 4.508 | 10.720 | 20.690 | 20.084 | 19.122 | 41.501 | 14.186 | 53.561 | 16.695 | 61.991 | 13.892 | 17.836 | 9.100 | 22.871 |
| Bleak | 1036 | 2 | 1 | 3 | 118.85 | 16.907 | 5.195 | 9.518 | 20.851 | 22.188 | 19.750 | 39.751 | 15.224 | 50.481 | 18.223 | 60.342 | 13.477 | 21.325 | 9.442 | 22.815 |
| Bleak | 1037 | 2 | 1 | 3 | 106.39 | 14.459 | 5.938 | 9.913 | 22.607 | 17.117 | 18.942 | 36.568 | 14.454 | 47.537 | 18.600 | 55.547 | 13.644 | 17.972 | 9.209 | 22.026 |
| Bleak | 1038 | 2 | 1 | 3 | 106.74 | 13.985 | 4.561 | 9.481 | 20.081 | 20.556 | 17.639 | 40.887 | 13.980 | 53.014 | 17.258 | 59.488 | 13.089 | 18.089 | 9.409 | 23.971 |
| Bleak | 1039 | 2 | 1 | 3 | 91.83 | 14.746 | 5.493 | 10.561 | 20.707 | 21.419 | 18.928 | 42.837 | 13.388 | 49.759 | 18.279 | 63.096 | 13.018 | 18.038 | 9.139 | 23.072 |
| Bleak | 1041 | 2 | 1 | 3 | 79.75 | 13.973 | 5.206 | 9.352 | 21.898 | 19.894 | 19.652 | 43.039 | 13.067 | 54.298 | 17.508 | 62.721 | 14.423 | 18.115 | 9.056 | 22.491 |
| Bleak | 1042 | 2 | 1 | 3 | 100.76 | 15.364 | 5.164 | 9.672 | 20.105 | 20.767 | 19.656 | 43.855 | 14.182 | 51.782 | 19.410 | 59.475 | 14.700 | 17.979 | 8.748 | 22.983 |
| Bleak | 1043 | 2 | 1 | 3 | 110.34 | 14.978 | 5.085 | 9.501 | 20.683 | 20.062 | 19.480 | 41.244 | 14.037 | 49.875 | 17.847 | 60.725 | 14.505 | 16.868 | 8.764 | 22.858 |
| Bleak | 1044 | 2 | 1 | 3 | 127.03 | 14.467 | 5.573 | 10.010 | 21.291 | 18.888 | 20.696 | 42.570 | 14.937 | 51.896 | 20.078 | 60.851 | 13.712 | 20.014 | 9.309 | 23.529 |
| Bleak | 1044 | 2 | 1 | 3 | 92.82 | 13.593 | 5.023 | 9.388 | 20.880 | 21.479 | 18.419 | 44.153 | 14.412 | 51.703 | 17.589 | 59.404 | 13.972 | 18.491 | 8.399 | 19.388 |
| Bleak | 1045 | 2 | 1 | 3 | 99.49 | 15.552 | 5.614 | 9.806 | 19.503 | 19.531 | 17.091 | 38.873 | 13.811 | 50.039 | 16.621 | 58.809 | 13.210 | 18.355 | 8.775 | 23.319 |
| Bleak | 1046 | 2 | 1 | 3 | 116.73 | 14.774 | 5.673 | 10.205 | 21.225 | 22.103 | 19.473 | 42.915 | 14.101 | 52.133 | 17.440 | 61.257 | 13.611 | 21.839 | 9.318 | 20.185 |
| Bleak | 1047 | 2 | 1 | 3 | 118.72 | 14.199 | 4.966 | 9.963 | 22.041 | 22.047 | 19.529 | 41.165 | 14.059 | 49.880 | 19.834 | 59.967 | 14.682 | 20.746 | 9.088 | 21.162 |
| Bleak | 1048 | 2 | 1 | 3 | 101.32 | 15.181 | 5.048 | 10.406 | 21.839 | 22.094 | 19.661 | 42.651 | 14.880 | 51.648 | 19.008 | 61.440 | 13.503 | 16.319 | 9.288 | 21.330 |
| Bleak | 1049 | 2 | 1 | 3 | 126.95 | 15.032 | 5.132 | 8.926 | 19.291 | 18.645 | 18.514 | 42.224 | 13.508 | 51.808 | 17.700 | 58.506 | 13.287 | 18.248 | 9.112 | 23.673 |
| Bleak | 1050 | 2 | 1 | 3 | 95.4 | 14.601 | 6.173 | 9.163 | 20.719 | 20.436 | 17.725 | 40.761 | 13.574 | 50.011 | 17.223 | 60.841 | 12.196 | 16.150 | 8.429 | 20.207 |
| Bleak | 1051 | 2 | 1 | 3 | 113.54 | 14.907 | 5.011 | 9.362 | 21.270 | 21.324 | 19.510 | 42.758 | 13.360 | 51.278 | 16.264 | 61.023 | 14.077 | 17.737 | 8.755 | 20.080 |
| Bleak | 1052 | 2 | 1 | 3 | 107.45 | 14.974 | 5.252 | 9.830 | 21.096 | 20.882 | 19.130 | 42.076 | 13.951 | 52.121 | 18.177 | 60.790 | 13.572 | 18.364 | 9.070 | 22.266 |
| Bleak | 1053 | 2 | 1 | 3 | 113.55 | 13.231 | 5.781 | 9.893 | 21.319 | 21.374 | 19.733 | 43.121 | 15.664 | 51.562 | 18.651 | 60.331 | 14.172 | 20.455 | 8.737 | 22.066 |
| Bleak | 1054 | 2 | 1 | 3 | 88.62 | 14.882 | 4.988 | 9.542 | 20.548 | 20.851 | 19.614 | 41.430 | 15.207 | 52.418 | 17.687 | 61.841 | 13.862 | 21.350 | 8.906 | 22.976 |
| Bleak | 1055 | 2 | 1 | 3 | 101.87 | 14.963 | 4.677 | 8.769 | 19.692 | 19.736 | 18.530 | 39.446 | 14.324 | 51.759 | 17.230 | 58.608 | 12.828 | 17.275 | 8.782 | 22.323 |
| Bleak | 1056 | 2 | 1 | 3 | 76.61 | 14.752 | 5.020 | 9.873 | 18.100 | 21.518 | 17.918 | 42.364 | 12.193 | 51.914 | 15.465 | 62.814 | 13.610 | 18.990 | 8.869 | 21.495 |
| Bleak | 1057 | 2 | 1 | 3 | 80.82 | 16.392 | 5.424 | 10.975 | 22.155 | 23.021 | 19.885 | 45.159 | 13.982 | 52.586 | 16.542 | 61.696 | 13.217 | 19.507 | 8.991 | 22.157 |
| Bleak | 1058 | 2 | 1 | 3 | 101.65 | 15.866 | 5.202 | 9.760 | 24.009 | 22.111 | 19.612 | 45.545 | 14.417 | 55.289 | 18.905 | 62.886 | 15.017 | 17.981 | 9.049 | 23.708 |
| Bleak | 1059 | 2 | 1 | 3 | 95.76 | 15.331 | 6.027 | 9.126 | 22.134 | 21.932 | 18.879 | 44.479 | 14.058 | 55.223 | 16.893 | 61.624 | 12.654 | 16.659 | 8.591 | 21.801 |
| Bleak | 1060 | 2 | 1 | 3 | 100.62 | 14.667 | 5.345 | 9.330 | 20.653 | 21.104 | 18.471 | 46.164 | 13.343 | 50.642 | 17.330 | 61.219 | 12.611 | 18.795 | 9.078 | 22.978 |
| Bleak | 1131 | 2 | 2 | 1 | 107.82 | 14.122 | 5.139 | 8.753 | 20.292 | 18.856 | 19.319 | 38.376 | 14.801 | 53.039 | 18.394 | 57.944 | 13.392 | 18.593 | 8.631 | 23.218 |
| Bleak | 1132 | 2 | 2 | 1 | 88.08 | 14.910 | 5.087 | 9.317 | 19.901 | 19.392 | 17.778 | 43.079 | 13.994 | 48.968 | 17.837 | 58.628 | 14.550 | 19.982 | 8.990 | 23.256 |
| Bleak | 1133 | 2 | 2 | 1 | 88.63 | 14.186 | 5.835 | 10.016 | 20.677 | 19.876 | 21.178 | 41.908 | 17.091 | 48.081 | 20.047 | 60.699 | 14.831 | 18.608 | 8.938 | 22.654 |
| Bleak | 1134 | 2 | 2 | 1 | 75.53 | 14.278 | 5.383 | 8.108 | 21.488 | 19.724 | 20.675 | 37.495 | 15.567 | 53.098 | 20.292 | 57.607 | 13.722 | 17.304 | 9.496 | 24.128 |
| Bleak | 1135 | 2 | 2 | 1 | 86.65 | 15.311 | 5.886 | 9.416 | 20.833 | 20.675 | 19.325 | 41.411 | 14.776 | 47.633 | 16.183 | 59.405 | 13.826 | 23.209 | 8.674 | 22.530 |
| Bleak | 1136 | 2 | 2 | 1 | 78.19 | 15.370 | 5.290 | 9.239 | 20.730 | 20.033 | 18.206 | 39.264 | 15.597 | 54.594 | 19.276 | 57.784 | 14.291 | 18.487 | 8.344 | 23.285 |
| Bleak | 1137 | 2 | 2 | 1 | 92.63 | 14.286 | 6.305 | 9.630 | 20.649 | 21.292 | 16.835 | 40.458 | 13.220 | 51.634 | 17.349 | 60.049 | 12.623 | 18.715 | 8.928 | 23.221 |
| Bleak | 1138 | 2 | 2 | 1 | 94.38 | 14.846 | 5.419 | 9.137 | 20.936 | 18.862 | 19.256 | 39.253 | 15.270 | 50.756 | 19.784 | 58.365 | 14.763 | 18.382 | 8.207 | 22.021 |
| Bleak | 1139 | 2 | 2 | 1 | 116.33 | 14.896 | 4.595 | 9.159 | 20.196 | 19.199 | 19.188 | 40.993 | 14.183 | 51.680 | 18.363 | 61.414 | 13.263 | 17.884 | 9.230 | 25.194 |
| Bleak | 1140 | 2 | 2 | 1 | 94.54 | 13.266 | 5.667 | 8.719 | 20.676 | 19.950 | 18.007 | 41.600 | 13.481 | 48.961 | 19.386 | 58.301 | 14.098 | 18.199 | 8.214 | 20.666 |
| Bleak | 1141 | 2 | 2 | 1 | 69.08 | 15.700 | 5.928 | 9.094 | 21.731 | 20.231 | 19.137 | 38.808 | 15.639 | 53.261 | 19.610 | 58.244 | 15.175 | 19.863 | 9.214 | 23.088 |
| Bleak | 1142 | 2 | 2 | 1 | 107.01 | 14.420 | 5.288 | 9.481 | 21.351 | 20.514 | 19.664 | 40.710 | 15.179 | 52.776 | 19.856 | 60.844 | 13.633 | 16.478 | 9.332 | 22.670 |
| Bleak | 1143 | 2 | 2 | 1 | 87.95 | 13.541 | 4.706 | 8.641 | 20.171 | 17.978 | 18.623 | 40.302 | 14.749 | 50.372 | 19.659 | 56.621 | 14.666 | 20.285 | 7.707 | 21.429 |
| Bleak | 1144 | 2 | 2 | 1 | 95.38 | 13.252 | 5.747 | 9.420 | 20.642 | 20.572 | 18.115 | 41.533 | 13.110 | 52.986 | 17.378 | 61.945 | 12.584 | 19.683 | 8.593 | 22.248 |
| Bleak | 1145 | 2 | 2 | 1 | 79.63 | 15.010 | 5.263 | 8.747 | 20.924 | 18.347 | 20.459 | 39.264 | 14.777 | 50.404 | 20.885 | 56.616 | 14.076 | 19.037 | 9.166 | 23.388 |
| Bleak | 1146 | 2 | 2 | 1 | 109.35 | 14.230 | 4.772 | 8.321 | 19.991 | 19.945 | 17.555 | 42.864 | 14.214 | 51.996 | 18.132 | 60.673 | 14.351 | 18.745 | 9.018 | 22.624 |
| Bleak | 1147 | 2 | 2 | 1 | 107.99 | 14.247 | 5.533 | 8.809 | 21.146 | 20.189 | 18.475 | 41.968 | 14.507 | 53.087 | 19.261 | 60.447 | 13.659 | 19.730 | 8.971 | 22.894 |
| Bleak | 1148 | 2 | 2 | 1 | 102.94 | 15.562 | 6.139 | 9.449 | 20.968 | 22.049 | 18.741 | 43.100 | 14.078 | 53.226 | 18.147 | 60.153 | 13.517 | 20.676 | 8.478 | 24.123 |
| Bleak | 1149 | 2 | 2 | 1 | 105.71 | 15.127 | 5.375 | 9.535 | 20.473 | 19.839 | 18.981 | 42.402 | 14.819 | 49.841 | 18.445 | 62.688 | 13.269 | 16.470 | 9.191 | 24.233 |
| Bleak | 1150 | 2 | 2 | 1 | 88.89 | 15.101 | 5.290 | 9.169 | 21.773 | 20.397 | 19.353 | 41.762 | 14.760 | 52.976 | 19.607 | 59.094 | 13.988 | 19.372 | 9.315 | 24.164 |
| Bleak | 1151 | 2 | 2 | 1 | 84.95 | 15.182 | 5.262 | 9.321 | 20.929 | 20.588 | 16.877 | 39.871 | 13.679 | 48.965 | 19.973 | 55.378 | 14.178 | 19.813 | 8.725 | 23.713 |
| Bleak | 1152 | 2 | 2 | 1 | 104.84 | 14.252 | 5.404 | 9.612 | 19.792 | 20.398 | 17.691 | 42.621 | 13.155 | 50.038 | 17.223 | 59.718 | 13.210 | 17.753 | 8.333 | 22.744 |
| Bleak | 1153 | 2 | 2 | 1 | 105.93 | 14.683 | 4.324 | 9.869 | 20.606 | 19.918 | 20.688 | 41.470 | 15.623 | 52.932 | 20.719 | 58.666 | 14.967 | 17.817 | 8.639 | 22.290 |
| Bleak | 1154 | 2 | 2 | 1 | 83.61 | 14.761 | 4.390 | 9.636 | 20.699 | 19.093 | 18.377 | 38.064 | 13.395 | 47.280 | 18.759 | 54.725 | 15.159 | 17.763 | 8.603 | 22.532 |
| Bleak | 1155 | 2 | 2 | 1 | 105.17 | 15.876 | 6.029 | 10.130 | 21.750 | 21.761 | 19.145 | 42.227 | 14.901 | 53.770 | 19.069 | 60.916 | 15.880 | 17.625 | 9.393 | 24.611 |
| Bleak | 1156 | 2 | 2 | 1 | 97.17 | 14.399 | 5.235 | 9.851 | 20.883 | 20.364 | 19.705 | 40.576 | 14.220 | 50.867 | 18.247 | 60.517 | 13.731 | 17.996 | 9.153 | 23.670 |
| Bleak | 1157 | 2 | 2 | 1 | 102.45 | 13.906 | 5.291 | 9.347 | 20.932 | 20.130 | 19.632 | 40.828 | 15.138 | 54.085 | 18.614 | 62.140 | 14.193 | 17.865 | 9.106 | 22.286 |
| Bleak | 1158 | 2 | 2 | 1 | 93.09 | 13.900 | 6.021 | 7.925 | 20.707 | 20.439 | 17.766 | 41.090 | 13.467 | 47.035 | 18.945 | 56.880 | 14.363 | 18.939 | 8.990 | 21.618 |
| Bleak | 1159 | 2 | 2 | 1 | 76.26 | 16.220 | 5.754 | 9.142 | 20.227 | 19.097 | 19.105 | 37.769 | 14.994 | 52.841 | 18.565 | 62.486 | 13.393 | 19.326 | 9.561 | 24.402 |
| Bleak | 1160 | 2 | 2 | 1 | 94.21 | 14.275 | 5.244 | 8.122 | 19.140 | 16.929 | 18.610 | 37.933 | 14.268 | 47.735 | 19.045 | 59.725 | 14.888 | 20.668 | 8.128 | 21.154 |
| Bleak | 1131 | 2 | 2 | 2 | 107.77 | 15.419 | 5.285 | 8.552 | 19.569 | 20.274 | 18.836 | 41.524 | 14.449 | 51.036 | 18.684 | 59.924 | 15.498 | 18.520 | 9.007 | 23.032 |
| Bleak | 1132 | 2 | 2 | 2 | 84.49 | 15.257 | 6.010 | 8.992 | 20.902 | 22.128 | 17.678 | 43.247 | 14.054 | 50.142 | 18.453 | 60.645 | 13.243 | 16.260 | 9.807 | 23.801 |
| Bleak | 1133 | 2 | 2 | 2 | 88.38 | 14.320 | 5.168 | 8.839 | 19.890 | 20.939 | 21.354 | 40.928 | 14.906 | 50.383 | 18.381 | 60.792 | 13.731 | 17.170 | 8.973 | 22.423 |
| Bleak | 1134 | 2 | 2 | 2 | 76.28 | 15.681 | 6.101 | 7.957 | 19.679 | 18.852 | 17.952 | 39.062 | 15.319 | 51.748 | 19.265 | 61.772 | 13.329 | 16.120 | 9.784 | 23.701 |
| Bleak | 1135 | 2 | 2 | 2 | 84.22 | 14.880 | 6.541 | 9.327 | 20.198 | 20.187 | 19.679 | 40.684 | 13.675 | 48.825 | 19.535 | 58.572 | 13.278 | 19.943 | 9.379 | 23.336 |
| Bleak | 1136 | 2 | 2 | 2 | 79.91 | 14.795 | 5.133 | 8.380 | 22.544 | 21.958 | 19.149 | 38.710 | 15.485 | 51.579 | 16.932 | 58.557 | 13.635 | 16.080 | 8.366 | 21.795 |
| Bleak | 1137 | 2 | 2 | 2 | 91.43 | 14.731 | 6.115 | 9.245 | 20.328 | 20.546 | 19.195 | 41.699 | 14.759 | 51.910 | 16.389 | 61.523 | 13.349 | 19.809 | 8.859 | 23.592 |
| Bleak | 1138 | 2 | 2 | 2 | 92.49 | 15.257 | 5.515 | 9.246 | 21.078 | 21.763 | 21.794 | 44.428 | 15.058 | 53.166 | 18.238 | 62.219 | 14.086 | 19.937 | 8.313 | 25.835 |
| Bleak | 1139 | 2 | 2 | 2 | 115.58 | 16.128 | 4.867 | 9.398 | 20.703 | 19.202 | 19.030 | 40.785 | 14.172 | 52.186 | 17.952 | 63.466 | 12.278 | 20.939 | 9.117 | 24.714 |
| Bleak | 1140 | 2 | 2 | 2 | 91.48 | 14.893 | 5.484 | 8.834 | 20.413 | 20.291 | 19.048 | 43.078 | 14.847 | 53.083 | 18.067 | 58.772 | 14.121 | 16.008 | 8.865 | 21.642 |
| Bleak | 1141 | 2 | 2 | 2 | 70.14 | 14.987 | 5.947 | 8.617 | 20.618 | 21.457 | 19.531 | 43.054 | 14.609 | 50.943 | 18.526 | 61.517 | 14.448 | 18.714 | 9.069 | 23.047 |
| Bleak | 1142 | 2 | 2 | 2 | 109.53 | 15.365 | 5.600 | 8.797 | 22.513 | 20.758 | 17.734 | 40.749 | 14.141 | 52.093 | 18.412 | 62.275 | 13.490 | 17.807 | 8.700 | 21.712 |
| Bleak | 1143 | 2 | 2 | 2 | 87.86 | 14.539 | 5.333 | 7.726 | 18.715 | 19.032 | 20.341 | 39.702 | 14.837 | 50.614 | 18.640 | 59.028 | 14.211 | 17.268 | 8.374 | 21.652 |
| Bleak | 1144 | 2 | 2 | 2 | 94.65 | 13.918 | 5.280 | 8.451 | 20.222 | 20.009 | 19.083 | 42.577 | 14.367 | 50.678 | 18.763 | 63.232 | 12.461 | 19.316 | 8.512 | 22.931 |
| Bleak | 1145 | 2 | 2 | 2 | 79.82 | 14.130 | 4.365 | 8.192 | 20.127 | 18.752 | 20.391 | 39.059 | 13.985 | 48.296 | 18.587 | 61.556 | 13.613 | 17.835 | 9.302 | 24.458 |
| Bleak | 1146 | 2 | 2 | 2 | 104.45 | 15.489 | 4.823 | 9.679 | 20.870 | 19.299 | 19.061 | 42.513 | 14.843 | 53.078 | 19.981 | 62.508 | 14.268 | 18.945 | 9.370 | 24.102 |
| Bleak | 1147 | 2 | 2 | 2 | 107.58 | 14.670 | 5.340 | 8.837 | 20.617 | 19.142 | 19.095 | 40.238 | 13.854 | 51.696 | 19.078 | 61.229 | 14.253 | 17.971 | 9.466 | 22.571 |
| Bleak | 1148 | 2 | 2 | 2 | 106.44 | 14.067 | 5.364 | 9.265 | 19.797 | 20.722 | 18.989 | 40.631 | 14.059 | 51.278 | 16.849 | 59.293 | 12.276 | 20.866 | 8.334 | 23.351 |
| Bleak | 1149 | 2 | 2 | 2 | 106.96 | 14.756 | 5.463 | 9.603 | 19.512 | 19.632 | 20.175 | 42.605 | 14.493 | 49.527 | 19.524 | 59.620 | 13.251 | 19.684 | 9.244 | 23.937 |
| Bleak | 1150 | 2 | 2 | 2 | 90.5 | 15.955 | 4.792 | 9.996 | 21.280 | 20.494 | 18.235 | 41.522 | 13.782 | 51.097 | 17.142 | 60.825 | 13.463 | 15.597 | 9.020 | 24.211 |
| Bleak | 1151 | 2 | 2 | 2 | 85.44 | 16.080 | 5.298 | 9.377 | 21.062 | 19.896 | 19.488 | 41.722 | 14.636 | 48.846 | 18.676 | 59.026 | 12.709 | 18.630 | 9.241 | 23.206 |
| Bleak | 1152 | 2 | 2 | 2 | 103.12 | 15.310 | 5.405 | 9.525 | 20.489 | 20.880 | 19.070 | 41.979 | 13.839 | 53.909 | 16.990 | 60.192 | 13.964 | 19.075 | 8.219 | 22.717 |
| Bleak | 1153 | 2 | 2 | 2 | 108.13 | 14.502 | 4.454 | 9.055 | 19.831 | 19.308 | 20.499 | 41.169 | 14.959 | 51.352 | 18.311 | 57.919 | 13.489 | 16.756 | 8.481 | 21.504 |
| Bleak | 1154 | 2 | 2 | 2 | 82.85 | 14.930 | 6.005 | 8.330 | 19.845 | 20.979 | 18.891 | 39.570 | 13.997 | 48.519 | 19.346 | 58.463 | 13.438 | 16.906 | 9.166 | 23.679 |
| Bleak | 1155 | 2 | 2 | 2 | 106.28 | 15.892 | 5.718 | 8.347 | 21.649 | 19.818 | 20.248 | 41.304 | 14.671 | 52.797 | 18.673 | 61.723 | 15.223 | 17.467 | 9.980 | 22.850 |
| Bleak | 1156 | 2 | 2 | 2 | 98.03 | 14.374 | 5.385 | 9.559 | 20.359 | 20.319 | 19.377 | 40.492 | 14.386 | 51.108 | 17.661 | 62.756 | 13.065 | 19.212 | 8.582 | 22.948 |
| Bleak | 1157 | 2 | 2 | 2 | 102 | 13.913 | 5.610 | 10.596 | 20.840 | 21.055 | 19.789 | 42.647 | 15.855 | 52.850 | 18.437 | 62.269 | 14.590 | 19.305 | 10.146 | 22.778 |
| Bleak | 1158 | 2 | 2 | 2 | 93.49 | 15.482 | 5.609 | 7.773 | 20.185 | 21.983 | 20.399 | 42.750 | 14.141 | 50.487 | 16.631 | 59.099 | 12.620 | 18.602 | 8.871 | 21.501 |
| Bleak | 1159 | 2 | 2 | 2 | 77.41 | 14.798 | 4.826 | 9.046 | 18.815 | 20.454 | 17.968 | 39.792 | 14.570 | 51.093 | 17.592 | 59.978 | 14.024 | 18.404 | 9.216 | 22.226 |
| Bleak | 1160 | 2 | 2 | 2 | 92.79 | 14.358 | 5.129 | 7.942 | 19.403 | 18.372 | 17.997 | 40.104 | 14.457 | 49.760 | 18.967 | 61.544 | 14.848 | 18.820 | 8.204 | 20.894 |
| Bleak | 1131 | 2 | 2 | 3 | 107.94 | 14.970 | 5.312 | 9.535 | 20.623 | 20.821 | 19.389 | 39.689 | 14.412 | 50.675 | 19.232 | 60.423 | 13.417 | 18.646 | 9.084 | 23.120 |
| Bleak | 1132 | 2 | 2 | 3 | 85.88 | 15.793 | 5.418 | 8.788 | 20.957 | 21.025 | 17.324 | 41.437 | 12.562 | 48.757 | 17.579 | 59.697 | 13.929 | 17.627 | 9.163 | 22.945 |
| Bleak | 1133 | 2 | 2 | 3 | 89.84 | 14.935 | 4.710 | 9.271 | 19.221 | 19.950 | 19.905 | 37.861 | 15.261 | 47.270 | 17.937 | 59.062 | 13.278 | 18.044 | 8.587 | 22.293 |
| Bleak | 1134 | 2 | 2 | 3 | 76.07 | 14.735 | 5.291 | 10.132 | 21.859 | 21.071 | 20.101 | 44.720 | 14.560 | 52.926 | 19.658 | 62.949 | 14.314 | 21.963 | 9.356 | 23.459 |
| Bleak | 1135 | 2 | 2 | 3 | 84.62 | 16.255 | 5.711 | 9.511 | 21.899 | 19.363 | 19.548 | 41.754 | 13.421 | 52.541 | 17.413 | 58.117 | 13.103 | 21.416 | 8.916 | 23.879 |
| Bleak | 1136 | 2 | 2 | 3 | 78.38 | 15.433 | 5.001 | 8.747 | 19.697 | 19.346 | 16.728 | 39.677 | 14.946 | 54.545 | 18.282 | 59.091 | 13.868 | 19.635 | 8.178 | 22.702 |
| Bleak | 1137 | 2 | 2 | 3 | 93.15 | 14.443 | 5.286 | 8.683 | 20.766 | 20.958 | 18.400 | 40.427 | 13.429 | 52.473 | 16.673 | 59.955 | 12.968 | 18.803 | 9.245 | 22.783 |
| Bleak | 1138 | 2 | 2 | 3 | 93.99 | 14.338 | 5.278 | 9.531 | 20.680 | 17.452 | 19.392 | 43.098 | 15.292 | 51.554 | 19.660 | 61.457 | 14.187 | 19.951 | 7.785 | 21.940 |
| Bleak | 1139 | 2 | 2 | 3 | 115.54 | 14.621 | 5.751 | 10.063 | 20.026 | 19.006 | 18.338 | 41.202 | 14.011 | 52.155 | 17.254 | 65.285 | 12.983 | 17.942 | 8.866 | 24.623 |
| Bleak | 1140 | 2 | 2 | 3 | 93.57 | 14.890 | 4.959 | 9.088 | 20.376 | 20.897 | 19.514 | 40.491 | 14.162 | 49.902 | 17.537 | 60.314 | 13.157 | 17.696 | 8.191 | 21.003 |
| Bleak | 1141 | 2 | 2 | 3 | 69.38 | 15.502 | 5.688 | 10.075 | 20.657 | 21.103 | 20.697 | 43.196 | 17.190 | 50.317 | 18.328 | 60.533 | 13.006 | 19.201 | 9.053 | 23.395 |
| Bleak | 1142 | 2 | 2 | 3 | 106.74 | 15.215 | 5.212 | 9.598 | 21.157 | 21.121 | 18.949 | 40.524 | 13.694 | 51.020 | 18.565 | 61.993 | 14.244 | 17.788 | 9.006 | 22.403 |
| Bleak | 1143 | 2 | 2 | 3 | 91.16 | 14.114 | 4.831 | 8.075 | 18.250 | 18.968 | 18.028 | 39.245 | 14.353 | 50.084 | 17.446 | 54.607 | 13.319 | 18.336 | 8.056 | 20.461 |
| Bleak | 1144 | 2 | 2 | 3 | 95.33 | 13.716 | 5.179 | 8.987 | 20.103 | 18.099 | 18.184 | 41.698 | 13.440 | 50.691 | 18.382 | 62.887 | 12.316 | 17.555 | 8.475 | 22.556 |
| Bleak | 1145 | 2 | 2 | 3 | 77.51 | 15.186 | 5.225 | 9.442 | 20.483 | 20.852 | 21.012 | 42.053 | 15.336 | 50.267 | 20.507 | 62.105 | 14.298 | 18.717 | 9.044 | 24.890 |
| Bleak | 1146 | 2 | 2 | 3 | 106.33 | 14.338 | 5.615 | 8.116 | 20.916 | 21.091 | 18.750 | 42.214 | 13.653 | 52.771 | 17.474 | 58.714 | 15.635 | 19.677 | 9.471 | 22.985 |
| Bleak | 1147 | 2 | 2 | 3 | 107.76 | 14.720 | 5.214 | 8.285 | 20.954 | 22.083 | 18.992 | 40.989 | 15.093 | 52.630 | 17.691 | 61.658 | 13.444 | 18.820 | 8.962 | 22.209 |
| Bleak | 1148 | 2 | 2 | 3 | 104.84 | 15.063 | 4.971 | 9.097 | 21.129 | 19.971 | 19.843 | 42.066 | 13.108 | 51.202 | 17.513 | 61.635 | 13.464 | 20.995 | 8.575 | 23.021 |
| Bleak | 1149 | 2 | 2 | 3 | 106.56 | 15.164 | 4.714 | 8.892 | 19.126 | 20.315 | 19.731 | 40.803 | 14.769 | 50.029 | 18.667 | 62.340 | 12.867 | 18.164 | 9.259 | 23.986 |
| Bleak | 1150 | 2 | 2 | 3 | 85.74 | 14.440 | 5.404 | 9.857 | 21.678 | 20.099 | 19.077 | 43.684 | 14.517 | 52.013 | 17.180 | 64.135 | 13.482 | 18.511 | 9.627 | 25.295 |
| Bleak | 1151 | 2 | 2 | 3 | 83.26 | 15.864 | 5.386 | 9.515 | 20.122 | 20.774 | 19.958 | 41.979 | 14.156 | 49.690 | 18.645 | 61.901 | 13.065 | 19.399 | 8.834 | 24.063 |
| Bleak | 1152 | 2 | 2 | 3 | 102.58 | 13.975 | 5.558 | 9.287 | 20.641 | 20.361 | 18.640 | 41.948 | 13.306 | 52.635 | 18.260 | 61.745 | 13.853 | 18.631 | 8.145 | 22.360 |
| Bleak | 1153 | 2 | 2 | 3 | 109.17 | 14.638 | 5.293 | 8.195 | 19.505 | 19.798 | 20.651 | 38.445 | 14.597 | 51.492 | 18.501 | 58.854 | 13.618 | 15.853 | 8.539 | 21.539 |
| Bleak | 1154 | 2 | 2 | 3 | 83.72 | 14.343 | 4.992 | 8.506 | 18.837 | 19.635 | 17.988 | 38.548 | 16.780 | 48.090 | 16.495 | 55.831 | 13.016 | 18.985 | 8.259 | 21.801 |
| Bleak | 1155 | 2 | 2 | 3 | 107.74 | 14.659 | 5.582 | 9.724 | 21.627 | 21.934 | 18.105 | 42.031 | 15.123 | 52.254 | 19.556 | 59.333 | 14.648 | 18.263 | 10.033 | 23.936 |
| Bleak | 1156 | 2 | 2 | 3 | 98.83 | 14.780 | 5.260 | 8.857 | 21.536 | 20.621 | 18.839 | 40.777 | 13.610 | 51.033 | 17.870 | 60.275 | 14.298 | 19.269 | 9.352 | 23.196 |
| Bleak | 1157 | 2 | 2 | 3 | 102.12 | 14.338 | 5.309 | 9.692 | 21.407 | 20.393 | 19.586 | 42.559 | 14.871 | 54.133 | 18.073 | 63.044 | 13.646 | 15.634 | 8.085 | 22.228 |
| Bleak | 1158 | 2 | 2 | 3 | 91.85 | 15.143 | 5.471 | 8.211 | 21.019 | 21.088 | 18.977 | 42.828 | 14.024 | 50.044 | 18.150 | 60.324 | 12.440 | 17.876 | 8.768 | 23.459 |
| Bleak | 1159 | 2 | 2 | 3 | 78.23 | 15.424 | 5.287 | 10.106 | 19.117 | 20.172 | 18.503 | 39.929 | 14.039 | 50.963 | 18.977 | 59.010 | 12.598 | 15.845 | 8.940 | 23.824 |
| Bleak | 1160 | 2 | 2 | 3 | 93.43 | 14.548 | 4.811 | 7.799 | 19.409 | 18.509 | 19.313 | 40.033 | 14.593 | 48.377 | 18.305 | 61.504 | 14.351 | 19.257 | 8.534 | 21.109 |
| Bleak | 1221 | 2 | 3 | 1 | 105.65 | 15.959 | 5.077 | 9.806 | 22.308 | 23.221 | 17.548 | 40.655 | 13.800 | 51.345 | 17.275 | 63.027 | 12.110 | 18.185 | 9.205 | 22.928 |
| Bleak | 1222 | 2 | 3 | 1 | 87.62 | 14.905 | 4.814 | 10.175 | 20.389 | 21.193 | 18.523 | 41.023 | 13.978 | 52.259 | 19.078 | 61.251 | 13.024 | 17.589 | 8.076 | 21.602 |
| Bleak | 1223 | 2 | 3 | 1 | 85.96 | 14.931 | 4.912 | 9.887 | 21.411 | 21.827 | 18.752 | 44.714 | 12.895 | 53.384 | 19.089 | 63.790 | 13.818 | 17.814 | 8.628 | 21.810 |
| Bleak | 1224 | 2 | 3 | 1 | 105.02 | 15.105 | 4.041 | 10.081 | 20.093 | 19.929 | 18.668 | 39.850 | 14.618 | 51.397 | 17.281 | 59.030 | 12.339 | 18.994 | 8.988 | 22.067 |
| Bleak | 1225 | 2 | 3 | 1 | 101.38 | 15.423 | 5.264 | 10.142 | 20.782 | 19.455 | 18.843 | 42.032 | 14.198 | 49.953 | 17.736 | 63.293 | 14.134 | 19.034 | 9.187 | 21.671 |
| Bleak | 1226 | 2 | 3 | 1 | 77.96 | 15.021 | 4.235 | 10.767 | 21.581 | 21.428 | 19.260 | 39.019 | 15.108 | 51.385 | 17.835 | 62.065 | 16.905 | 20.032 | 9.597 | 22.716 |
| Bleak | 1227 | 2 | 3 | 1 | 90.31 | 14.747 | 5.192 | 9.921 | 21.345 | 24.173 | 18.997 | 42.612 | 14.930 | 48.395 | 18.069 | 62.332 | 12.892 | 19.219 | 9.048 | 22.388 |
| Bleak | 1228 | 2 | 3 | 1 | 92.61 | 13.851 | 5.382 | 10.138 | 21.323 | 20.323 | 16.692 | 40.855 | 14.948 | 49.199 | 16.800 | 55.615 | 13.187 | 21.482 | 9.087 | 21.840 |
| Bleak | 1229 | 2 | 3 | 1 | 88.88 | 15.818 | 5.181 | 9.523 | 20.773 | 20.160 | 17.890 | 40.716 | 14.063 | 49.256 | 19.263 | 60.672 | 12.881 | 17.284 | 8.903 | 22.478 |
| Bleak | 1230 | 2 | 3 | 1 | 102.03 | 14.608 | 5.134 | 10.365 | 22.226 | 20.401 | 18.092 | 39.820 | 14.267 | 50.925 | 16.354 | 61.168 | 12.734 | 17.859 | 9.017 | 22.305 |
| Bleak | 1231 | 2 | 3 | 1 | 88.61 | 13.493 | 4.878 | 10.504 | 22.222 | 20.176 | 19.692 | 42.225 | 14.540 | 51.566 | 19.152 | 62.068 | 13.259 | 15.392 | 9.169 | 23.551 |
| Bleak | 1232 | 2 | 3 | 1 | 130.84 | 16.197 | 5.146 | 9.462 | 20.751 | 21.023 | 17.542 | 41.547 | 14.180 | 52.263 | 19.088 | 61.017 | 13.997 | 18.538 | 8.984 | 22.595 |
| Bleak | 1233 | 2 | 3 | 1 | 94.71 | 13.337 | 4.845 | 9.678 | 23.324 | 24.689 | 18.161 | 44.714 | 14.716 | 50.204 | 18.232 | 60.469 | 11.790 | 15.085 | 9.613 | 23.644 |
| Bleak | 1234 | 2 | 3 | 1 | 94.86 | 15.064 | 4.919 | 9.846 | 20.926 | 19.537 | 19.657 | 42.461 | 15.064 | 51.238 | 18.370 | 63.392 | 16.016 | 18.580 | 8.852 | 22.437 |
| Bleak | 1235 | 2 | 3 | 1 | 93.77 | 13.819 | 4.938 | 9.794 | 20.861 | 21.454 | 18.505 | 41.771 | 15.465 | 52.456 | 19.082 | 61.070 | 13.206 | 20.265 | 9.373 | 22.274 |
| Bleak | 1236 | 2 | 3 | 1 | 87.07 | 16.039 | 5.340 | 9.682 | 21.269 | 21.968 | 18.432 | 42.604 | 14.619 | 52.477 | 17.732 | 60.516 | 12.873 | 19.228 | 10.020 | 22.898 |
| Bleak | 1237 | 2 | 3 | 1 | 99.91 | 15.366 | 5.375 | 9.742 | 21.311 | 21.435 | 18.602 | 41.845 | 14.515 | 52.335 | 18.637 | 59.983 | 12.441 | 17.503 | 8.828 | 22.663 |
| Bleak | 1238 | 2 | 3 | 1 | 102.76 | 15.511 | 5.585 | 10.652 | 21.401 | 21.490 | 19.037 | 42.884 | 14.767 | 50.295 | 18.194 | 63.174 | 12.411 | 18.865 | 8.938 | 23.017 |
| Bleak | 1239 | 2 | 3 | 1 | 95.99 | 14.509 | 4.797 | 9.345 | 21.233 | 19.839 | 17.715 | 40.547 | 12.849 | 50.219 | 17.706 | 58.826 | 13.820 | 18.339 | 9.087 | 21.848 |
| Bleak | 1240 | 2 | 3 | 1 | 90.8 | 14.994 | 5.345 | 10.089 | 21.681 | 20.716 | 16.424 | 39.585 | 14.178 | 51.812 | 17.093 | 60.763 | 11.914 | 17.797 | 9.705 | 22.036 |
| Bleak | 1241 | 2 | 3 | 1 | 110.29 | 14.645 | 5.525 | 10.098 | 21.823 | 22.297 | 19.301 | 41.913 | 14.619 | 51.862 | 18.721 | 60.755 | 14.330 | 20.804 | 8.714 | 22.641 |
| Bleak | 1242 | 2 | 3 | 1 | 100.78 | 16.096 | 4.884 | 8.623 | 20.053 | 17.609 | 19.855 | 41.478 | 16.178 | 51.088 | 19.135 | 63.250 | 12.516 | 19.326 | 8.968 | 22.536 |
| Bleak | 1243 | 2 | 3 | 1 | 100.64 | 14.471 | 4.756 | 8.982 | 19.693 | 19.623 | 17.762 | 38.441 | 13.555 | 51.431 | 18.180 | 58.164 | 14.763 | 19.101 | 9.821 | 24.149 |
| Bleak | 1244 | 2 | 3 | 1 | 91.53 | 15.423 | 5.736 | 10.119 | 22.954 | 23.203 | 18.309 | 45.932 | 13.434 | 54.828 | 18.700 | 62.517 | 12.907 | 18.095 | 8.955 | 22.693 |
| Bleak | 1245 | 2 | 3 | 1 | 93.67 | 14.886 | 5.473 | 9.368 | 20.251 | 19.840 | 19.722 | 37.938 | 14.922 | 51.425 | 19.007 | 57.192 | 13.537 | 19.323 | 10.096 | 22.911 |
| Bleak | 1246 | 2 | 3 | 1 | 91.31 | 14.696 | 4.950 | 10.594 | 22.222 | 22.345 | 19.504 | 41.435 | 15.627 | 52.365 | 17.653 | 59.560 | 13.795 | 18.858 | 9.548 | 22.111 |
| Bleak | 1247 | 2 | 3 | 1 | 95.51 | 14.271 | 4.975 | 9.539 | 20.859 | 21.307 | 19.473 | 41.028 | 13.977 | 50.938 | 19.011 | 61.103 | 14.354 | 21.272 | 9.537 | 21.515 |
| Bleak | 1248 | 2 | 3 | 1 | 63.15 | 14.284 | 5.651 | 11.416 | 22.112 | 21.134 | 20.001 | 40.578 | 15.268 | 48.816 | 19.114 | 63.329 | 14.563 | 18.476 | 9.069 | 21.876 |
| Bleak | 1249 | 2 | 3 | 1 | 74.27 | 14.829 | 5.134 | 10.587 | 21.309 | 22.566 | 20.576 | 43.485 | 15.808 | 52.658 | 18.848 | 62.933 | 14.377 | 18.956 | 8.759 | 21.699 |
| Bleak | 1250 | 2 | 3 | 1 | 82.81 | 15.526 | 4.755 | 10.189 | 21.219 | 22.471 | 18.437 | 45.020 | 14.914 | 50.936 | 18.389 | 62.494 | 13.763 | 20.094 | 9.658 | 23.998 |
| Bleak | 1221 | 2 | 3 | 2 | 104.38 | 15.995 | 5.874 | 10.749 | 20.977 | 21.073 | 18.268 | 40.684 | 14.380 | 50.146 | 17.365 | 60.580 | 11.884 | 19.069 | 9.740 | 23.711 |
| Bleak | 1222 | 2 | 3 | 2 | 87.24 | 14.203 | 4.735 | 9.156 | 20.652 | 20.850 | 19.712 | 44.638 | 14.498 | 54.181 | 18.174 | 62.351 | 14.218 | 18.228 | 8.685 | 21.454 |
| Bleak | 1223 | 2 | 3 | 2 | 88.79 | 13.891 | 4.967 | 11.098 | 20.826 | 21.860 | 19.384 | 40.890 | 15.506 | 52.862 | 18.372 | 61.078 | 12.816 | 19.524 | 8.509 | 21.031 |
| Bleak | 1224 | 2 | 3 | 2 | 104.99 | 15.295 | 5.055 | 10.736 | 20.414 | 20.843 | 18.626 | 40.675 | 14.893 | 50.979 | 17.958 | 61.054 | 12.632 | 20.716 | 8.897 | 22.489 |
| Bleak | 1225 | 2 | 3 | 2 | 101.28 | 14.965 | 5.327 | 10.468 | 20.120 | 21.517 | 18.995 | 41.844 | 13.774 | 50.023 | 18.426 | 63.598 | 13.517 | 17.199 | 8.878 | 21.530 |
| Bleak | 1226 | 2 | 3 | 2 | 78.54 | 14.811 | 5.203 | 10.227 | 20.739 | 20.082 | 19.335 | 39.520 | 15.397 | 51.502 | 18.189 | 62.444 | 13.498 | 18.481 | 9.103 | 22.554 |
| Bleak | 1227 | 2 | 3 | 2 | 88.55 | 14.937 | 4.728 | 10.247 | 21.209 | 20.834 | 19.726 | 45.065 | 13.556 | 49.952 | 18.068 | 62.014 | 12.068 | 19.662 | 9.960 | 22.412 |
| Bleak | 1228 | 2 | 3 | 2 | 90.53 | 15.833 | 6.406 | 9.917 | 21.894 | 21.975 | 17.611 | 41.637 | 13.132 | 53.412 | 18.179 | 59.888 | 11.647 | 19.752 | 8.654 | 22.213 |
| Bleak | 1229 | 2 | 3 | 2 | 87.06 | 16.305 | 3.949 | 10.629 | 20.671 | 19.180 | 20.601 | 38.431 | 14.970 | 52.450 | 19.263 | 57.919 | 13.969 | 19.196 | 10.021 | 22.330 |
| Bleak | 1230 | 2 | 3 | 2 | 100.45 | 15.349 | 4.968 | 10.128 | 20.997 | 19.852 | 18.558 | 37.530 | 14.412 | 53.917 | 16.366 | 61.686 | 12.482 | 17.838 | 9.606 | 23.608 |
| Bleak | 1231 | 2 | 3 | 2 | 87.69 | 13.556 | 4.287 | 9.641 | 20.593 | 19.885 | 21.179 | 39.004 | 15.154 | 52.949 | 17.709 | 61.511 | 12.819 | 16.252 | 9.535 | 23.386 |
| Bleak | 1232 | 2 | 3 | 2 | 128.82 | 15.963 | 5.195 | 10.263 | 21.256 | 21.048 | 18.636 | 41.293 | 14.338 | 52.679 | 18.014 | 59.652 | 13.412 | 19.152 | 9.117 | 22.972 |
| Bleak | 1233 | 2 | 3 | 2 | 95.49 | 14.944 | 5.882 | 9.999 | 21.605 | 24.015 | 17.202 | 41.861 | 13.827 | 55.466 | 17.188 | 60.628 | 11.660 | 16.816 | 10.107 | 23.239 |
| Bleak | 1234 | 2 | 3 | 2 | 99.68 | 14.851 | 4.630 | 9.999 | 20.267 | 19.709 | 19.462 | 40.103 | 14.259 | 47.780 | 17.551 | 62.055 | 13.453 | 19.989 | 8.798 | 21.570 |
| Bleak | 1235 | 2 | 3 | 2 | 94.44 | 13.894 | 4.952 | 9.708 | 21.868 | 20.268 | 19.091 | 40.123 | 16.152 | 52.071 | 18.933 | 58.675 | 13.486 | 21.103 | 9.290 | 21.687 |
| Bleak | 1236 | 2 | 3 | 2 | 86.83 | 16.145 | 4.793 | 9.790 | 21.270 | 21.503 | 18.634 | 45.099 | 13.721 | 52.830 | 19.406 | 60.356 | 12.804 | 19.490 | 9.513 | 23.358 |
| Bleak | 1237 | 2 | 3 | 2 | 99.06 | 15.328 | 5.160 | 9.833 | 20.037 | 22.045 | 18.563 | 42.161 | 13.988 | 52.516 | 15.653 | 63.152 | 10.946 | 19.883 | 9.606 | 23.454 |
| Bleak | 1238 | 2 | 3 | 2 | 101.91 | 15.102 | 5.159 | 10.587 | 21.831 | 21.999 | 19.394 | 42.638 | 15.069 | 52.185 | 18.451 | 63.932 | 12.872 | 18.672 | 8.788 | 23.293 |
| Bleak | 1239 | 2 | 3 | 2 | 94.68 | 14.353 | 5.021 | 10.925 | 20.421 | 21.663 | 19.661 | 41.958 | 14.782 | 52.314 | 18.206 | 60.161 | 14.908 | 19.371 | 9.380 | 22.502 |
| Bleak | 1240 | 2 | 3 | 2 | 90.07 | 15.361 | 4.405 | 10.655 | 21.443 | 21.611 | 18.219 | 38.680 | 14.112 | 51.209 | 17.385 | 61.393 | 12.995 | 19.612 | 9.672 | 22.396 |
| Bleak | 1241 | 2 | 3 | 2 | 110.93 | 14.914 | 4.422 | 9.697 | 20.945 | 21.584 | 20.145 | 39.100 | 15.527 | 50.785 | 18.476 | 61.087 | 14.333 | 20.319 | 8.438 | 22.584 |
| Bleak | 1242 | 2 | 3 | 2 | 97.46 | 13.394 | 4.880 | 8.704 | 18.950 | 18.611 | 20.169 | 41.131 | 13.966 | 55.202 | 20.482 | 62.498 | 14.584 | 17.337 | 9.954 | 24.174 |
| Bleak | 1243 | 2 | 3 | 2 | 100.27 | 15.578 | 4.311 | 9.443 | 19.909 | 19.470 | 17.075 | 40.598 | 13.898 | 51.560 | 18.151 | 57.039 | 14.030 | 19.459 | 10.301 | 23.284 |
| Bleak | 1244 | 2 | 3 | 2 | 89.26 | 15.573 | 5.202 | 10.739 | 23.248 | 23.777 | 18.782 | 44.145 | 14.324 | 54.985 | 19.654 | 61.950 | 13.522 | 19.046 | 9.073 | 23.830 |
| Bleak | 1245 | 2 | 3 | 2 | 92.71 | 15.381 | 4.935 | 9.811 | 21.008 | 19.360 | 20.307 | 41.607 | 16.191 | 50.843 | 19.450 | 63.007 | 13.113 | 20.184 | 10.226 | 24.103 |
| Bleak | 1246 | 2 | 3 | 2 | 91.4 | 14.925 | 5.148 | 9.985 | 21.575 | 21.592 | 20.046 | 42.946 | 15.677 | 50.118 | 16.931 | 60.127 | 12.932 | 18.936 | 9.211 | 22.759 |
| Bleak | 1247 | 2 | 3 | 2 | 94.52 | 15.154 | 5.339 | 10.708 | 21.768 | 21.319 | 19.046 | 41.979 | 14.894 | 50.339 | 18.683 | 61.545 | 14.704 | 21.209 | 9.959 | 21.997 |
| Bleak | 1248 | 2 | 3 | 2 | 63.97 | 15.636 | 4.674 | 10.722 | 21.987 | 22.536 | 19.103 | 45.902 | 14.628 | 50.602 | 20.023 | 64.025 | 12.131 | 18.040 | 8.618 | 21.105 |
| Bleak | 1249 | 2 | 3 | 2 | 75.19 | 14.551 | 4.662 | 10.446 | 20.647 | 21.208 | 20.217 | 42.283 | 16.044 | 53.659 | 18.546 | 61.528 | 12.834 | 19.321 | 8.861 | 21.944 |
| Bleak | 1250 | 2 | 3 | 2 | 81.05 | 15.525 | 5.493 | 9.797 | 22.249 | 22.075 | 19.963 | 45.329 | 14.878 | 50.985 | 19.230 | 63.363 | 14.916 | 18.907 | 10.307 | 23.576 |
| Bleak | 1221 | 2 | 3 | 3 | 101.19 | 15.758 | 4.040 | 9.665 | 21.040 | 20.095 | 18.029 | 42.572 | 14.674 | 54.447 | 17.389 | 63.266 | 12.320 | 16.963 | 10.021 | 25.008 |
| Bleak | 1222 | 2 | 3 | 3 | 86.84 | 13.422 | 4.567 | 9.190 | 21.535 | 20.475 | 17.502 | 43.369 | 13.456 | 54.518 | 18.026 | 62.723 | 12.199 | 19.766 | 8.801 | 22.108 |
| Bleak | 1223 | 2 | 3 | 3 | 88.12 | 14.435 | 4.663 | 10.090 | 20.463 | 19.516 | 19.214 | 39.308 | 14.162 | 52.748 | 17.481 | 59.043 | 12.626 | 20.687 | 8.482 | 21.574 |
| Bleak | 1224 | 2 | 3 | 3 | 105.39 | 14.667 | 4.595 | 9.256 | 20.385 | 20.996 | 18.357 | 41.024 | 13.847 | 52.413 | 18.308 | 57.683 | 11.593 | 19.670 | 8.745 | 22.537 |
| Bleak | 1225 | 2 | 3 | 3 | 100.69 | 15.267 | 4.225 | 9.448 | 21.035 | 20.955 | 18.933 | 44.426 | 14.782 | 50.432 | 18.065 | 63.512 | 13.088 | 17.189 | 9.111 | 21.277 |
| Bleak | 1226 | 2 | 3 | 3 | 77.86 | 14.317 | 4.355 | 9.205 | 19.069 | 19.819 | 20.263 | 39.571 | 15.196 | 50.397 | 16.448 | 59.585 | 12.671 | 18.512 | 8.956 | 22.432 |
| Bleak | 1227 | 2 | 3 | 3 | 88.38 | 13.569 | 4.351 | 9.880 | 21.248 | 21.202 | 18.627 | 45.593 | 15.986 | 51.165 | 19.065 | 62.438 | 13.644 | 19.293 | 8.820 | 22.765 |
| Bleak | 1228 | 2 | 3 | 3 | 91.3 | 14.581 | 5.015 | 9.313 | 18.722 | 20.511 | 16.701 | 38.098 | 14.335 | 51.667 | 16.335 | 59.513 | 12.744 | 21.279 | 8.691 | 21.218 |
| Bleak | 1229 | 2 | 3 | 3 | 86.93 | 15.952 | 4.562 | 9.902 | 21.157 | 20.811 | 17.053 | 42.931 | 15.295 | 53.417 | 17.823 | 62.272 | 14.085 | 19.257 | 9.003 | 23.027 |
| Bleak | 1230 | 2 | 3 | 3 | 100.23 | 14.340 | 4.622 | 9.177 | 21.303 | 20.273 | 19.120 | 39.134 | 15.237 | 54.146 | 17.195 | 60.340 | 13.703 | 18.826 | 9.490 | 23.062 |
| Bleak | 1231 | 2 | 3 | 3 | 87.45 | 14.172 | 4.623 | 8.863 | 19.752 | 19.063 | 20.054 | 38.023 | 14.578 | 52.007 | 19.571 | 58.774 | 11.910 | 18.053 | 9.283 | 23.966 |
| Bleak | 1232 | 2 | 3 | 3 | 128.97 | 15.042 | 5.373 | 8.862 | 21.331 | 20.584 | 19.208 | 42.114 | 13.968 | 52.608 | 18.136 | 62.051 | 13.533 | 18.941 | 8.855 | 22.587 |
| Bleak | 1233 | 2 | 3 | 3 | 97.6 | 13.844 | 5.579 | 10.649 | 22.486 | 20.963 | 18.779 | 43.468 | 13.392 | 52.514 | 16.952 | 61.358 | 12.615 | 19.462 | 9.453 | 22.457 |
| Bleak | 1234 | 2 | 3 | 3 | 96.79 | 13.307 | 3.822 | 8.177 | 20.351 | 19.328 | 19.395 | 40.768 | 14.757 | 52.408 | 18.500 | 60.899 | 13.515 | 18.605 | 8.896 | 21.590 |
| Bleak | 1235 | 2 | 3 | 3 | 92.43 | 14.450 | 4.487 | 9.822 | 21.290 | 20.948 | 18.584 | 40.274 | 14.941 | 51.802 | 15.879 | 59.744 | 12.958 | 20.935 | 9.302 | 22.758 |
| Bleak | 1236 | 2 | 3 | 3 | 87.43 | 14.208 | 4.803 | 8.888 | 20.631 | 22.688 | 20.246 | 43.928 | 14.308 | 52.610 | 18.797 | 59.056 | 13.356 | 19.594 | 9.087 | 22.566 |
| Bleak | 1237 | 2 | 3 | 3 | 99.78 | 15.950 | 5.198 | 9.891 | 20.948 | 20.751 | 18.976 | 41.102 | 13.524 | 49.917 | 17.633 | 62.368 | 12.132 | 21.168 | 8.888 | 22.937 |
| Bleak | 1238 | 2 | 3 | 3 | 103.91 | 14.537 | 5.632 | 9.595 | 20.301 | 20.857 | 18.034 | 42.831 | 13.592 | 52.338 | 18.055 | 60.238 | 12.203 | 17.802 | 8.929 | 23.226 |
| Bleak | 1239 | 2 | 3 | 3 | 94.88 | 15.041 | 4.877 | 10.592 | 21.054 | 23.163 | 20.154 | 40.149 | 14.430 | 53.634 | 17.856 | 61.328 | 13.964 | 19.588 | 9.535 | 21.285 |
| Bleak | 1240 | 2 | 3 | 3 | 91.41 | 15.019 | 6.074 | 10.432 | 20.990 | 20.762 | 19.389 | 40.475 | 13.407 | 52.263 | 16.866 | 59.619 | 13.289 | 19.029 | 9.517 | 21.851 |
| Bleak | 1241 | 2 | 3 | 3 | 108.63 | 14.487 | 5.792 | 9.779 | 22.451 | 21.884 | 19.159 | 41.833 | 15.450 | 52.353 | 18.564 | 62.138 | 14.139 | 19.816 | 9.155 | 23.038 |
| Bleak | 1242 | 2 | 3 | 3 | 100.01 | 13.872 | 5.389 | 9.644 | 21.183 | 22.337 | 20.599 | 40.851 | 16.348 | 53.348 | 19.157 | 62.410 | 13.211 | 20.548 | 9.724 | 24.036 |
| Bleak | 1243 | 2 | 3 | 3 | 100.13 | 14.411 | 3.940 | 8.848 | 19.373 | 18.332 | 19.089 | 39.532 | 14.070 | 50.240 | 18.037 | 54.917 | 14.416 | 20.310 | 9.218 | 24.170 |
| Bleak | 1244 | 2 | 3 | 3 | 85.9 | 16.058 | 5.963 | 11.448 | 23.283 | 23.994 | 19.156 | 45.040 | 15.545 | 57.986 | 18.266 | 63.496 | 14.524 | 19.580 | 9.318 | 24.087 |
| Bleak | 1245 | 2 | 3 | 3 | 94.05 | 15.243 | 5.905 | 9.255 | 20.512 | 21.763 | 19.092 | 40.343 | 15.366 | 50.693 | 19.135 | 60.526 | 13.902 | 21.073 | 10.048 | 22.773 |
| Bleak | 1246 | 2 | 3 | 3 | 90.46 | 14.757 | 5.010 | 9.936 | 21.932 | 21.263 | 19.662 | 41.401 | 15.419 | 53.681 | 18.862 | 59.690 | 15.674 | 17.102 | 9.515 | 21.972 |
| Bleak | 1247 | 2 | 3 | 3 | 96 | 14.902 | 6.021 | 8.068 | 19.502 | 21.464 | 18.865 | 44.700 | 13.747 | 50.608 | 18.684 | 63.374 | 14.505 | 20.117 | 9.572 | 23.090 |
| Bleak | 1248 | 2 | 3 | 3 | 61.81 | 15.859 | 3.381 | 10.558 | 21.801 | 20.634 | 22.418 | 43.260 | 16.668 | 54.525 | 18.333 | 61.428 | 15.537 | 19.538 | 8.366 | 22.819 |
| Bleak | 1249 | 2 | 3 | 3 | 74.89 | 15.046 | 3.800 | 9.828 | 20.907 | 20.364 | 19.974 | 39.669 | 14.327 | 54.954 | 20.080 | 58.129 | 13.100 | 19.960 | 8.908 | 21.748 |
| Bleak | 1250 | 2 | 3 | 3 | 83.72 | 14.984 | 4.794 | 9.599 | 21.284 | 20.986 | 19.776 | 43.454 | 15.594 | 52.567 | 17.303 | 61.412 | 12.711 | 19.561 | 9.409 | 22.069 |
| Bleak | 1031 | 3 | 1 | 1 | 97.87 | 15.72 | 5.53 | 8.85 | 20.82 | 24.47 | 20.62 | 43.36 | 13.51 | 53.04 | 18.23 | 60.45 | 14.10 | 17.15 | 9.62 | 23.62 |
| Bleak | 1032 | 3 | 1 | 1 | 125.05 | 16.78 | 5.95 | 10.08 | 20.47 | 21.66 | 19.29 | 43.53 | 14.52 | 54.61 | 17.21 | 59.27 | 12.86 | 17.09 | 7.90 | 20.68 |
| Bleak | 1033 | 3 | 1 | 1 | 129.37 | 16.05 | 5.23 | 10.21 | 20.73 | 22.54 | 19.83 | 44.83 | 14.74 | 55.00 | 18.96 | 59.56 | 13.21 | 17.32 | 7.99 | 23.09 |
| Bleak | 1034 | 3 | 1 | 1 | 96.12 | 15.14 | 5.34 | 9.90 | 20.65 | 22.63 | 19.33 | 41.42 | 13.56 | 53.48 | 15.81 | 62.96 | 12.41 | 15.20 | 9.19 | 22.31 |
| Bleak | 1035 | 3 | 1 | 1 | 77.78 | 15.65 | 5.76 | 9.45 | 20.71 | 22.89 | 18.02 | 43.46 | 12.50 | 56.01 | 15.86 | 61.10 | 11.81 | 15.66 | 8.68 | 22.43 |
| Bleak | 1036 | 3 | 1 | 1 | 119.75 | 15.81 | 5.75 | 8.85 | 20.60 | 23.42 | 18.17 | 40.72 | 14.38 | 50.96 | 17.49 | 57.48 | 12.96 | 21.12 | 9.66 | 22.99 |
| Bleak | 1037 | 3 | 1 | 1 | 105.42 | 14.37 | 5.42 | 8.61 | 20.34 | 22.53 | 18.15 | 42.68 | 13.72 | 53.09 | 17.94 | 60.34 | 13.11 | 16.78 | 9.54 | 22.35 |
| Bleak | 1038 | 3 | 1 | 1 | 103.57 | 16.70 | 5.03 | 9.40 | 21.11 | 22.34 | 18.15 | 44.73 | 13.90 | 53.74 | 16.59 | 61.41 | 12.09 | 19.23 | 9.93 | 25.53 |
| Bleak | 1039 | 3 | 1 | 1 | 91.06 | 16.89 | 5.49 | 9.61 | 20.64 | 21.23 | 18.89 | 42.72 | 11.97 | 56.09 | 17.90 | 61.52 | 12.72 | 18.69 | 9.27 | 23.47 |
| Bleak | 1040 | 3 | 1 | 1 | 130.86 | 13.93 | 5.21 | 9.35 | 21.21 | 22.46 | 20.74 | 43.69 | 13.94 | 54.60 | 18.93 | 60.00 | 12.82 | 19.61 | 9.41 | 23.57 |
| Bleak | 1041 | 3 | 1 | 1 | 80.55 | 16.09 | 4.17 | 7.95 | 21.41 | 23.29 | 18.42 | 43.26 | 11.37 | 55.83 | 17.06 | 62.01 | 12.94 | 18.75 | 8.68 | 22.90 |
| Bleak | 1042 | 3 | 1 | 1 | 102.15 | 15.06 | 4.44 | 8.96 | 20.42 | 22.78 | 18.48 | 43.93 | 13.69 | 52.26 | 17.51 | 58.91 | 12.73 | 18.68 | 9.21 | 23.23 |
| Bleak | 1043 | 3 | 1 | 1 | 108.69 | 14.84 | 4.68 | 7.27 | 19.04 | 22.48 | 19.74 | 42.07 | 14.72 | 54.08 | 18.84 | 60.08 | 14.07 | 18.85 | 9.28 | 23.69 |
| Bleak | 1044 | 3 | 1 | 1 | 92.01 | 14.06 | 4.59 | 8.54 | 20.53 | 21.63 | 18.17 | 43.48 | 13.19 | 54.03 | 17.33 | 59.53 | 12.72 | 20.95 | 8.31 | 19.25 |
| Bleak | 1045 | 3 | 1 | 1 | 98.69 | 15.22 | 4.44 | 7.98 | 20.34 | 21.19 | 16.66 | 41.98 | 12.51 | 53.35 | 16.17 | 57.21 | 12.02 | 17.71 | 9.03 | 23.39 |
| Bleak | 1046 | 3 | 1 | 1 | 115.52 | 15.33 | 5.57 | 9.53 | 20.94 | 23.11 | 20.62 | 43.37 | 13.19 | 57.13 | 18.59 | 62.74 | 13.78 | 18.31 | 9.45 | 21.16 |
| Bleak | 1047 | 3 | 1 | 1 | 116.14 | 16.01 | 4.71 | 9.55 | 21.98 | 25.51 | 19.17 | 44.42 | 15.26 | 56.25 | 18.48 | 63.42 | 13.77 | 19.28 | 9.23 | 22.07 |
| Bleak | 1048 | 3 | 1 | 1 | 99.40 | 15.63 | 5.33 | 9.56 | 21.43 | 24.41 | 18.48 | 43.95 | 14.04 | 54.25 | 17.26 | 61.10 | 12.01 | 18.85 | 9.39 | 21.97 |
| Bleak | 1049 | 3 | 1 | 1 | 127.40 | 16.95 | 4.33 | 8.26 | 20.62 | 21.94 | 17.31 | 44.89 | 12.31 | 52.87 | 16.12 | 63.04 | 13.22 | 17.91 | 8.90 | 24.08 |
| Bleak | 1050 | 3 | 1 | 1 | 93.86 | 15.11 | 6.61 | 8.54 | 20.63 | 22.80 | 16.16 | 41.56 | 12.92 | 53.58 | 15.22 | 59.74 | 12.36 | 18.95 | 8.56 | 21.17 |
| Bleak | 1051 | 3 | 1 | 1 | 115.77 | 14.62 | 5.26 | 8.31 | 21.24 | 22.73 | 18.14 | 42.87 | 11.83 | 53.18 | 16.37 | 59.95 | 13.29 | 19.21 | 8.80 | 20.75 |
| Bleak | 1052 | 3 | 1 | 1 | 120.87 | 15.70 | 5.39 | 9.92 | 21.03 | 23.35 | 17.30 | 43.86 | 12.78 | 53.64 | 18.21 | 60.63 | 13.90 | 18.62 | 8.65 | 22.98 |
| Bleak | 1053 | 3 | 1 | 1 | 112.86 | 15.38 | 4.81 | 9.11 | 21.20 | 23.91 | 19.11 | 43.67 | 14.63 | 54.10 | 17.47 | 60.12 | 12.75 | 18.18 | 8.91 | 22.38 |
| Bleak | 1054 | 3 | 1 | 1 | 89.75 | 15.41 | 4.94 | 9.59 | 20.38 | 21.33 | 18.47 | 40.07 | 13.71 | 53.95 | 18.49 | 59.80 | 13.22 | 20.67 | 9.17 | 22.70 |
| Bleak | 1055 | 3 | 1 | 1 | 100.69 | 14.81 | 4.04 | 9.39 | 19.62 | 21.57 | 18.80 | 42.40 | 12.58 | 53.41 | 16.87 | 60.16 | 12.32 | 17.60 | 8.94 | 22.50 |
| Bleak | 1056 | 3 | 1 | 1 | 75.65 | 15.02 | 4.73 | 9.45 | 20.72 | 22.42 | 14.77 | 43.67 | 12.67 | 53.51 | 18.28 | 60.95 | 11.71 | 20.59 | 9.14 | 21.48 |
| Bleak | 1057 | 3 | 1 | 1 | 78.49 | 15.83 | 5.63 | 9.64 | 21.83 | 23.70 | 18.27 | 45.99 | 13.72 | 56.54 | 16.37 | 62.70 | 13.15 | 19.48 | 9.36 | 23.09 |
| Bleak | 1058 | 3 | 1 | 1 | 99.30 | 14.95 | 5.23 | 9.94 | 22.74 | 25.18 | 19.48 | 45.63 | 15.01 | 59.63 | 18.02 | 64.85 | 13.94 | 19.39 | 9.22 | 16.00 |
| Bleak | 1059 | 3 | 1 | 1 | 97.43 | 15.30 | 6.08 | 8.93 | 20.89 | 23.32 | 17.72 | 41.12 | 11.59 | 54.90 | 16.58 | 61.40 | 10.90 | 17.51 | 8.73 | 20.62 |
| Bleak | 1060 | 3 | 1 | 1 | 99.28 | 14.84 | 5.35 | 9.14 | 20.65 | 22.76 | 18.54 | 42.22 | 13.37 | 53.86 | 17.71 | 62.33 | 11.46 | 18.98 | 9.01 | 23.17 |
| Bleak | 1031 | 3 | 1 | 2 | 97.77 | 15.68 | 5.52 | 10.15 | 21.29 | 22.38 | 20.35 | 43.25 | 13.12 | 55.03 | 18.93 | 59.58 | 13.13 | 15.85 | 9.39 | 23.32 |
| Bleak | 1032 | 3 | 1 | 2 | 125.74 | 14.56 | 5.09 | 9.06 | 20.16 | 21.31 | 19.50 | 42.60 | 13.60 | 51.95 | 17.95 | 60.39 | 12.39 | 18.20 | 7.77 | 21.36 |
| Bleak | 1033 | 3 | 1 | 2 | 128.40 | 15.62 | 5.11 | 9.90 | 20.68 | 22.59 | 20.25 | 44.11 | 14.35 | 54.47 | 18.36 | 60.98 | 13.45 | 17.17 | 8.85 | 22.61 |
| Bleak | 1034 | 3 | 1 | 2 | 96.71 | 15.43 | 5.44 | 8.82 | 20.83 | 22.56 | 19.32 | 40.80 | 13.14 | 52.51 | 15.06 | 60.64 | 12.35 | 17.36 | 9.42 | 22.17 |
| Bleak | 1035 | 3 | 1 | 2 | 78.04 | 16.30 | 5.88 | 10.81 | 20.98 | 21.02 | 18.24 | 41.90 | 12.13 | 55.36 | 16.37 | 60.04 | 13.04 | 18.26 | 8.88 | 22.58 |
| Bleak | 1036 | 3 | 1 | 2 | 119.71 | 16.06 | 5.43 | 9.65 | 20.98 | 23.09 | 19.01 | 42.56 | 13.65 | 51.30 | 17.52 | 59.31 | 12.84 | 19.84 | 9.79 | 22.85 |
| Bleak | 1037 | 3 | 1 | 2 | 105.35 | 14.34 | 5.42 | 8.93 | 20.88 | 22.09 | 19.36 | 43.00 | 13.45 | 54.51 | 17.97 | 59.11 | 13.05 | 19.12 | 8.24 | 22.24 |
| Bleak | 1038 | 3 | 1 | 2 | 104.42 | 15.36 | 5.37 | 9.75 | 20.98 | 23.41 | 17.12 | 43.46 | 11.95 | 52.89 | 17.01 | 61.37 | 10.88 | 17.72 | 9.74 | 24.52 |
| Bleak | 1039 | 3 | 1 | 2 | 90.92 | 14.47 | 4.70 | 10.82 | 21.36 | 22.64 | 18.68 | 41.87 | 11.83 | 54.69 | 16.51 | 60.09 | 13.23 | 17.50 | 9.87 | 23.45 |
| Bleak | 1040 | 3 | 1 | 2 | 130.15 | 14.39 | 5.58 | 9.94 | 21.27 | 24.17 | 20.07 | 43.58 | 14.19 | 53.37 | 19.28 | 59.70 | 13.02 | 19.26 | 9.15 | 23.78 |
| Bleak | 1041 | 3 | 1 | 2 | 82.07 | 14.86 | 5.04 | 9.88 | 21.16 | 21.47 | 18.40 | 40.27 | 11.89 | 53.27 | 16.57 | 60.75 | 13.91 | 18.75 | 8.61 | 21.65 |
| Bleak | 1042 | 3 | 1 | 2 | 102.92 | 15.76 | 5.51 | 9.54 | 20.67 | 22.66 | 18.12 | 44.80 | 14.22 | 47.60 | 17.90 | 58.19 | 12.88 | 20.90 | 9.38 | 23.05 |
| Bleak | 1043 | 3 | 1 | 2 | 111.16 | 14.79 | 5.10 | 9.42 | 19.53 | 20.83 | 19.87 | 41.30 | 14.72 | 51.43 | 17.47 | 59.31 | 15.33 | 16.98 | 8.87 | 22.49 |
| Bleak | 1044 | 3 | 1 | 2 | 94.15 | 13.55 | 5.41 | 9.86 | 20.23 | 21.42 | 19.17 | 40.51 | 13.17 | 51.74 | 16.26 | 58.26 | 13.72 | 17.46 | 7.99 | 18.82 |
| Bleak | 1045 | 3 | 1 | 2 | 99.53 | 15.07 | 5.24 | 8.10 | 19.30 | 20.98 | 16.65 | 39.48 | 13.12 | 52.78 | 16.63 | 57.78 | 12.22 | 19.67 | 8.83 | 22.59 |
| Bleak | 1046 | 3 | 1 | 2 | 118.06 | 14.39 | 4.87 | 9.94 | 21.58 | 22.26 | 19.33 | 40.36 | 13.47 | 53.95 | 18.18 | 58.46 | 12.71 | 17.50 | 9.81 | 20.62 |
| Bleak | 1047 | 3 | 1 | 2 | 116.45 | 16.43 | 5.45 | 10.01 | 21.86 | 24.07 | 19.42 | 44.20 | 14.62 | 53.88 | 18.85 | 62.12 | 13.40 | 19.09 | 9.19 | 22.28 |
| Bleak | 1048 | 3 | 1 | 2 | 99.65 | 15.77 | 5.72 | 9.24 | 21.69 | 23.23 | 19.31 | 42.03 | 13.07 | 53.76 | 18.57 | 59.22 | 13.06 | 18.39 | 9.49 | 22.13 |
| Bleak | 1049 | 3 | 1 | 2 | 126.68 | 15.64 | 5.51 | 9.19 | 20.10 | 21.23 | 17.44 | 42.06 | 12.55 | 52.06 | 15.75 | 63.38 | 13.38 | 17.33 | 8.83 | 24.37 |
| Bleak | 1050 | 3 | 1 | 2 | 92.81 | 14.87 | 5.66 | 9.07 | 20.30 | 22.35 | 18.49 | 41.82 | 13.50 | 53.87 | 15.94 | 58.39 | 12.74 | 18.05 | 8.32 | 20.12 |
| Bleak | 1051 | 3 | 1 | 2 | 115.24 | 15.08 | 5.65 | 9.54 | 20.70 | 22.33 | 18.94 | 42.38 | 12.79 | 53.92 | 15.85 | 60.84 | 13.63 | 18.97 | 9.12 | 19.92 |
| Bleak | 1052 | 3 | 1 | 2 | 120.31 | 14.66 | 6.36 | 9.26 | 20.61 | 21.98 | 19.01 | 42.89 | 13.28 | 52.96 | 17.44 | 61.03 | 12.79 | 19.01 | 8.68 | 22.62 |
| Bleak | 1053 | 3 | 1 | 2 | 114.70 | 14.82 | 5.88 | 9.45 | 20.79 | 22.44 | 19.17 | 42.56 | 13.38 | 53.69 | 17.34 | 59.70 | 13.19 | 18.30 | 8.96 | 22.26 |
| Bleak | 1054 | 3 | 1 | 2 | 89.26 | 15.39 | 5.25 | 8.21 | 20.72 | 22.30 | 19.16 | 40.66 | 14.23 | 53.18 | 17.01 | 58.90 | 12.53 | 20.26 | 8.94 | 22.67 |
| Bleak | 1055 | 3 | 1 | 2 | 101.75 | 14.13 | 5.38 | 9.24 | 19.70 | 22.15 | 16.87 | 41.15 | 12.34 | 53.22 | 16.04 | 58.56 | 11.08 | 18.58 | 9.11 | 22.92 |
| Bleak | 1056 | 3 | 1 | 2 | 75.50 | 14.80 | 4.46 | 9.92 | 20.80 | 22.74 | 18.40 | 43.46 | 12.64 | 50.24 | 17.29 | 61.23 | 12.81 | 18.89 | 9.20 | 22.26 |
| Bleak | 1057 | 3 | 1 | 2 | 80.90 | 16.22 | 5.14 | 10.00 | 21.59 | 22.40 | 18.99 | 43.29 | 13.18 | 53.96 | 16.96 | 60.66 | 12.39 | 19.13 | 8.86 | 22.39 |
| Bleak | 1058 | 3 | 1 | 2 | 100.54 | 15.63 | 5.82 | 10.66 | 22.57 | 24.03 | 19.75 | 44.58 | 13.39 | 56.38 | 17.94 | 66.01 | 13.92 | 16.97 | 9.00 | 24.01 |
| Bleak | 1059 | 3 | 1 | 2 | 96.47 | 14.73 | 5.50 | 10.05 | 20.26 | 21.50 | 17.47 | 42.50 | 12.63 | 54.71 | 17.01 | 61.61 | 12.09 | 18.11 | 8.87 | 21.01 |
| Bleak | 1060 | 3 | 1 | 2 | 98.70 | 14.73 | 4.79 | 9.83 | 19.79 | 21.12 | 18.49 | 43.09 | 12.28 | 52.32 | 16.76 | 59.55 | 12.63 | 19.73 | 9.05 | 23.05 |
| Bleak | 1031 | 3 | 1 | 3 | 98.00 | 15.78 | 4.63 | 9.80 | 21.32 | 22.19 | 20.36 | 42.19 | 14.37 | 52.23 | 17.90 | 59.12 | 13.20 | 17.63 | 9.19 | 22.93 |
| Bleak | 1032 | 3 | 1 | 3 | 124.12 | 15.79 | 5.73 | 8.97 | 20.55 | 21.77 | 19.61 | 43.12 | 15.08 | 52.22 | 16.31 | 59.57 | 13.18 | 16.07 | 8.07 | 21.57 |
| Bleak | 1033 | 3 | 1 | 3 | 129.68 | 14.70 | 5.35 | 10.22 | 20.77 | 22.24 | 19.94 | 44.00 | 15.15 | 53.25 | 18.64 | 59.00 | 12.82 | 18.31 | 8.80 | 22.41 |
| Bleak | 1034 | 3 | 1 | 3 | 97.51 | 15.19 | 5.41 | 10.15 | 20.15 | 21.44 | 19.37 | 42.27 | 14.00 | 54.49 | 16.35 | 59.30 | 12.45 | 17.18 | 9.27 | 22.95 |
| Bleak | 1035 | 3 | 1 | 3 | 77.94 | 15.64 | 5.14 | 10.03 | 21.18 | 22.29 | 19.59 | 42.35 | 12.91 | 54.35 | 17.46 | 61.70 | 11.42 | 17.51 | 8.47 | 22.26 |
| Bleak | 1036 | 3 | 1 | 3 | 119.91 | 15.14 | 5.23 | 9.97 | 21.36 | 23.03 | 19.53 | 42.51 | 15.12 | 51.38 | 18.28 | 59.25 | 12.45 | 17.85 | 9.48 | 23.06 |
| Bleak | 1037 | 3 | 1 | 3 | 105.42 | 14.45 | 5.42 | 9.19 | 20.54 | 21.73 | 19.06 | 42.97 | 13.34 | 55.36 | 17.45 | 59.98 | 12.26 | 18.42 | 9.01 | 22.54 |
| Bleak | 1038 | 3 | 1 | 3 | 103.68 | 15.31 | 5.64 | 9.79 | 20.99 | 23.26 | 17.36 | 42.60 | 12.16 | 52.92 | 15.61 | 62.60 | 11.28 | 17.25 | 9.52 | 24.48 |
| Bleak | 1039 | 3 | 1 | 3 | 89.79 | 14.89 | 4.89 | 10.45 | 21.57 | 23.46 | 19.09 | 41.99 | 12.82 | 54.61 | 18.68 | 63.06 | 12.20 | 17.89 | 9.16 | 23.00 |
| Bleak | 1041 | 3 | 1 | 3 | 128.49 | 13.95 | 5.44 | 12.50 | 21.13 | 23.88 | 20.73 | 42.65 | 14.55 | 52.41 | 19.60 | 60.23 | 13.05 | 18.79 | 9.06 | 22.94 |
| Bleak | 1042 | 3 | 1 | 3 | 110.78 | 15.60 | 5.03 | 10.17 | 20.95 | 22.36 | 17.44 | 41.91 | 12.08 | 56.41 | 17.23 | 60.44 | 13.54 | 19.47 | 8.66 | 22.67 |
| Bleak | 1043 | 3 | 1 | 3 | 81.03 | 15.22 | 5.63 | 9.74 | 20.78 | 21.87 | 18.56 | 43.06 | 14.64 | 53.64 | 19.68 | 58.61 | 13.29 | 19.11 | 9.19 | 22.43 |
| Bleak | 1044 | 3 | 1 | 3 | 102.58 | 14.23 | 5.29 | 8.54 | 19.86 | 21.61 | 20.07 | 41.91 | 13.85 | 54.33 | 18.30 | 58.45 | 13.45 | 16.71 | 8.88 | 22.18 |
| Bleak | 1044 | 3 | 1 | 3 | 92.50 | 15.00 | 5.30 | 8.69 | 20.66 | 22.21 | 18.58 | 42.35 | 13.92 | 52.95 | 17.01 | 57.52 | 14.03 | 18.57 | 7.97 | 19.18 |
| Bleak | 1045 | 3 | 1 | 3 | 98.67 | 14.26 | 5.10 | 8.83 | 19.66 | 21.52 | 16.78 | 40.64 | 12.57 | 52.89 | 17.13 | 56.89 | 12.47 | 16.19 | 8.86 | 22.85 |
| Bleak | 1046 | 3 | 1 | 3 | 116.96 | 14.74 | 6.26 | 9.94 | 21.77 | 23.68 | 19.15 | 41.74 | 15.02 | 53.96 | 17.56 | 58.76 | 13.80 | 15.52 | 9.32 | 20.28 |
| Bleak | 1047 | 3 | 1 | 3 | 114.43 | 15.45 | 5.35 | 10.53 | 22.47 | 23.48 | 19.75 | 44.34 | 15.29 | 55.67 | 18.69 | 61.86 | 12.79 | 19.92 | 9.24 | 22.42 |
| Bleak | 1048 | 3 | 1 | 3 | 99.65 | 15.39 | 5.86 | 10.23 | 21.59 | 22.57 | 19.33 | 42.59 | 13.54 | 52.33 | 17.64 | 59.06 | 12.14 | 17.59 | 9.44 | 21.88 |
| Bleak | 1049 | 3 | 1 | 3 | 126.44 | 15.09 | 5.46 | 9.42 | 20.55 | 21.84 | 17.45 | 44.79 | 12.97 | 54.22 | 16.07 | 62.31 | 12.36 | 17.96 | 8.55 | 23.61 |
| Bleak | 1050 | 3 | 1 | 3 | 93.82 | 14.63 | 6.14 | 9.10 | 19.91 | 21.40 | 18.09 | 40.82 | 13.07 | 52.32 | 16.77 | 59.36 | 12.59 | 15.44 | 8.41 | 20.25 |
| Bleak | 1051 | 3 | 1 | 3 | 114.20 | 14.95 | 6.03 | 9.28 | 20.20 | 22.67 | 19.43 | 42.92 | 13.36 | 51.27 | 15.71 | 60.96 | 14.12 | 18.52 | 8.83 | 20.20 |
| Bleak | 1052 | 3 | 1 | 3 | 120.13 | 15.00 | 5.90 | 9.39 | 20.63 | 22.34 | 17.99 | 42.85 | 14.18 | 53.54 | 17.31 | 62.41 | 13.29 | 18.16 | 8.66 | 23.18 |
| Bleak | 1053 | 3 | 1 | 3 | 114.80 | 14.84 | 4.82 | 8.86 | 20.58 | 25.24 | 19.41 | 41.80 | 13.62 | 52.24 | 18.15 | 59.23 | 12.04 | 17.79 | 8.79 | 21.70 |
| Bleak | 1054 | 3 | 1 | 3 | 89.00 | 14.76 | 4.95 | 9.08 | 20.40 | 23.57 | 18.76 | 41.68 | 13.79 | 53.30 | 19.14 | 58.97 | 13.07 | 19.78 | 8.85 | 22.62 |
| Bleak | 1055 | 3 | 1 | 3 | 101.23 | 14.84 | 5.07 | 9.69 | 20.23 | 21.02 | 18.02 | 41.15 | 12.89 | 51.18 | 17.64 | 60.13 | 11.98 | 15.93 | 8.89 | 22.92 |
| Bleak | 1056 | 3 | 1 | 3 | 75.43 | 14.72 | 4.60 | 9.22 | 20.82 | 22.74 | 17.10 | 42.63 | 12.00 | 53.06 | 18.15 | 60.50 | 13.43 | 17.93 | 9.19 | 21.35 |
| Bleak | 1057 | 3 | 1 | 3 | 79.89 | 15.58 | 5.70 | 10.48 | 21.93 | 22.91 | 19.21 | 45.01 | 13.53 | 59.91 | 17.45 | 60.83 | 12.57 | 17.00 | 9.12 | 23.14 |
| Bleak | 1058 | 3 | 1 | 3 | 98.73 | 16.10 | 4.72 | 10.97 | 23.15 | 24.31 | 19.70 | 45.87 | 13.52 | 57.76 | 18.83 | 63.32 | 13.08 | 19.03 | 8.73 | 25.05 |
| Bleak | 1059 | 3 | 1 | 3 | 105.87 | 14.13 | 4.81 | 8.73 | 19.18 | 20.16 | 16.50 | 38.77 | 11.11 | 49.50 | 15.59 | 52.00 | 11.73 | 14.15 | 8.01 | 18.77 |
| Bleak | 1060 | 3 | 1 | 3 | 99.54 | 14.50 | 4.44 | 8.26 | 19.79 | 21.70 | 17.86 | 42.16 | 13.05 | 52.80 | 18.32 | 60.68 | 11.28 | 18.60 | 8.82 | 23.18 |
| Bleak | 1131 | 3 | 2 | 1 | 108.89 | 13.82 | 5.59 | 8.95 | 19.56 | 22.02 | 19.61 | 39.72 | 13.72 | 52.82 | 18.04 | 61.05 | 12.95 | 17.91 | 8.86 | 22.84 |
| Bleak | 1132 | 3 | 2 | 1 | 85.27 | 15.31 | 5.65 | 8.61 | 20.55 | 21.64 | 19.17 | 42.28 | 14.35 | 52.19 | 18.04 | 57.93 | 13.54 | 16.64 | 9.51 | 22.96 |
| Bleak | 1133 | 3 | 2 | 1 | 87.24 | 15.64 | 5.00 | 8.83 | 20.36 | 21.17 | 21.25 | 41.99 | 15.05 | 51.69 | 17.89 | 58.74 | 15.28 | 19.14 | 8.99 | 22.82 |
| Bleak | 1134 | 3 | 2 | 1 | 76.26 | 14.57 | 6.02 | 9.34 | 21.93 | 23.61 | 19.58 | 44.25 | 14.87 | 51.57 | 18.01 | 61.90 | 13.88 | 17.36 | 9.96 | 23.67 |
| Bleak | 1135 | 3 | 2 | 1 | 84.97 | 15.11 | 6.17 | 8.43 | 21.27 | 22.79 | 19.66 | 43.16 | 14.20 | 51.83 | 16.97 | 58.60 | 13.41 | 21.19 | 9.04 | 23.71 |
| Bleak | 1136 | 3 | 2 | 1 | 78.10 | 14.59 | 5.44 | 7.98 | 19.77 | 21.35 | 19.07 | 39.57 | 14.50 | 52.71 | 17.27 | 60.11 | 13.30 | 18.23 | 8.59 | 22.81 |
| Bleak | 1137 | 3 | 2 | 1 | 92.77 | 14.25 | 5.43 | 8.72 | 20.05 | 21.46 | 18.52 | 41.82 | 14.03 | 52.62 | 17.01 | 61.28 | 13.55 | 19.75 | 9.28 | 23.38 |
| Bleak | 1138 | 3 | 2 | 1 | 92.77 | 14.60 | 5.50 | 9.07 | 21.44 | 23.10 | 21.72 | 44.19 | 13.14 | 54.66 | 19.05 | 61.62 | 14.42 | 20.14 | 8.24 | 22.97 |
| Bleak | 1139 | 3 | 2 | 1 | 116.35 | 15.60 | 4.85 | 9.74 | 21.11 | 22.40 | 18.27 | 41.78 | 12.99 | 53.21 | 16.87 | 62.69 | 12.27 | 17.77 | 9.57 | 25.81 |
| Bleak | 1140 | 3 | 2 | 1 | 92.69 | 15.55 | 5.16 | 9.62 | 20.77 | 23.05 | 19.35 | 42.74 | 13.54 | 53.37 | 17.43 | 60.42 | 14.74 | 17.55 | 9.11 | 22.23 |
| Bleak | 1141 | 3 | 2 | 1 | 69.26 | 14.73 | 5.22 | 9.46 | 21.23 | 22.03 | 19.42 | 42.56 | 14.28 | 53.25 | 17.68 | 59.36 | 14.32 | 16.90 | 9.14 | 23.80 |
| Bleak | 1142 | 3 | 2 | 1 | 108.84 | 14.04 | 5.03 | 8.19 | 20.25 | 21.66 | 19.46 | 41.21 | 13.62 | 51.54 | 18.52 | 61.06 | 13.02 | 19.06 | 9.23 | 22.40 |
| Bleak | 1143 | 3 | 2 | 1 | 89.14 | 13.53 | 4.87 | 8.15 | 19.65 | 21.02 | 19.53 | 39.60 | 14.65 | 49.32 | 18.28 | 55.82 | 14.35 | 20.21 | 8.24 | 21.19 |
| Bleak | 1144 | 3 | 2 | 1 | 93.46 | 14.72 | 5.52 | 8.76 | 20.60 | 21.47 | 19.51 | 43.00 | 14.59 | 54.91 | 17.61 | 62.40 | 13.49 | 19.09 | 9.08 | 22.96 |
| Bleak | 1145 | 3 | 2 | 1 | 79.45 | 15.09 | 4.73 | 8.39 | 19.61 | 21.56 | 20.02 | 43.01 | 14.41 | 49.39 | 18.48 | 60.10 | 13.78 | 17.47 | 9.57 | 24.32 |
| Bleak | 1146 | 3 | 2 | 1 | 105.96 | 14.96 | 5.03 | 9.34 | 20.27 | 21.81 | 20.13 | 43.65 | 14.45 | 52.50 | 18.11 | 62.03 | 13.98 | 17.87 | 9.36 | 23.79 |
| Bleak | 1147 | 3 | 2 | 1 | 108.12 | 15.28 | 5.30 | 8.66 | 20.77 | 22.33 | 18.36 | 43.35 | 14.42 | 52.99 | 18.34 | 61.93 | 14.37 | 20.21 | 9.58 | 22.67 |
| Bleak | 1148 | 3 | 2 | 1 | 105.11 | 14.19 | 5.46 | 8.77 | 21.05 | 22.65 | 19.71 | 43.60 | 14.24 | 53.57 | 15.87 | 60.27 | 12.33 | 21.50 | 8.73 | 23.79 |
| Bleak | 1149 | 3 | 2 | 1 | 106.23 | 14.36 | 5.43 | 8.09 | 19.71 | 22.18 | 19.03 | 42.54 | 13.47 | 54.11 | 17.35 | 61.21 | 14.36 | 18.08 | 9.33 | 24.23 |
| Bleak | 1150 | 3 | 2 | 1 | 89.62 | 14.76 | 4.86 | 8.23 | 20.19 | 22.04 | 19.51 | 42.21 | 14.11 | 52.21 | 17.00 | 61.59 | 15.43 | 18.38 | 9.23 | 24.20 |
| Bleak | 1151 | 3 | 2 | 1 | 84.02 | 16.31 | 4.83 | 8.53 | 20.39 | 22.72 | 20.14 | 42.98 | 13.79 | 50.43 | 18.91 | 60.10 | 13.56 | 20.27 | 9.53 | 22.82 |
| Bleak | 1152 | 3 | 2 | 1 | 102.22 | 15.27 | 6.05 | 8.67 | 20.44 | 21.32 | 18.78 | 45.75 | 13.52 | 53.18 | 16.90 | 60.32 | 13.51 | 17.94 | 8.60 | 23.28 |
| Bleak | 1153 | 3 | 2 | 1 | 107.62 | 13.89 | 4.94 | 8.67 | 19.98 | 22.05 | 20.68 | 42.33 | 13.96 | 52.12 | 19.26 | 60.35 | 13.94 | 15.17 | 9.10 | 22.49 |
| Bleak | 1154 | 3 | 2 | 1 | 82.31 | 15.44 | 4.84 | 8.00 | 20.34 | 21.47 | 19.04 | 41.40 | 13.88 | 53.37 | 18.29 | 57.70 | 13.91 | 19.73 | 9.72 | 23.64 |
| Bleak | 1155 | 3 | 2 | 1 | 105.60 | 15.95 | 5.80 | 10.31 | 21.54 | 23.34 | 20.17 | 44.12 | 14.80 | 52.09 | 19.20 | 61.19 | 15.17 | 19.98 | 9.85 | 25.32 |
| Bleak | 1156 | 3 | 2 | 1 | 96.92 | 15.07 | 5.14 | 8.47 | 20.94 | 22.31 | 19.36 | 42.74 | 14.91 | 53.87 | 17.63 | 62.25 | 13.31 | 18.22 | 9.37 | 23.48 |
| Bleak | 1157 | 3 | 2 | 1 | 102.52 | 14.68 | 5.75 | 8.97 | 20.68 | 23.06 | 19.09 | 43.52 | 14.36 | 56.50 | 17.50 | 61.19 | 12.93 | 17.51 | 9.22 | 22.71 |
| Bleak | 1158 | 3 | 2 | 1 | 91.80 | 15.31 | 6.00 | 8.24 | 21.20 | 21.72 | 18.79 | 43.85 | 14.30 | 51.66 | 18.09 | 60.58 | 13.83 | 19.43 | 9.35 | 22.01 |
| Bleak | 1159 | 3 | 2 | 1 | 76.48 | 15.62 | 5.42 | 8.98 | 20.94 | 24.02 | 18.83 | 43.33 | 15.38 | 52.87 | 18.21 | 60.38 | 12.64 | 19.23 | 9.52 | 23.24 |
| Bleak | 1160 | 3 | 2 | 1 | 92.90 | 14.37 | 4.95 | 7.77 | 20.09 | 22.36 | 18.58 | 41.07 | 15.08 | 52.25 | 17.74 | 58.08 | 14.57 | 21.26 | 8.72 | 20.76 |
| Bleak | 1131 | 3 | 2 | 2 | 108.91 | 14.16 | 4.86 | 7.93 | 20.18 | 21.80 | 19.18 | 42.14 | 13.59 | 52.29 | 17.18 | 57.21 | 12.58 | 18.34 | 9.01 | 23.49 |
| Bleak | 1132 | 3 | 2 | 2 | 86.32 | 15.10 | 5.68 | 8.65 | 20.87 | 23.14 | 17.69 | 41.11 | 13.26 | 51.62 | 16.66 | 60.95 | 12.46 | 18.76 | 9.47 | 23.49 |
| Bleak | 1133 | 3 | 2 | 2 | 89.02 | 15.01 | 5.39 | 8.56 | 19.60 | 21.17 | 20.64 | 40.73 | 14.62 | 52.91 | 17.84 | 58.96 | 13.27 | 18.00 | 8.98 | 22.94 |
| Bleak | 1134 | 3 | 2 | 2 | 76.40 | 15.11 | 6.03 | 9.36 | 21.07 | 23.36 | 20.54 | 44.19 | 14.65 | 52.71 | 19.28 | 60.59 | 13.40 | 16.68 | 9.48 | 23.91 |
| Bleak | 1135 | 3 | 2 | 2 | 86.96 | 14.79 | 5.56 | 8.43 | 19.80 | 20.82 | 19.49 | 39.71 | 12.68 | 50.83 | 16.82 | 56.11 | 12.69 | 19.22 | 8.89 | 24.06 |
| Bleak | 1136 | 3 | 2 | 2 | 79.06 | 15.50 | 5.19 | 8.68 | 20.62 | 21.97 | 19.28 | 41.31 | 15.14 | 53.10 | 17.33 | 57.94 | 13.95 | 16.60 | 8.64 | 23.38 |
| Bleak | 1137 | 3 | 2 | 2 | 93.97 | 14.39 | 5.35 | 8.66 | 19.71 | 21.28 | 18.86 | 42.15 | 13.90 | 52.69 | 16.34 | 59.48 | 12.06 | 19.17 | 9.06 | 23.04 |
| Bleak | 1138 | 3 | 2 | 2 | 93.64 | 14.71 | 5.15 | 9.45 | 21.16 | 23.10 | 21.13 | 44.92 | 13.13 | 54.42 | 17.62 | 61.11 | 13.73 | 17.49 | 8.36 | 22.29 |
| Bleak | 1139 | 3 | 2 | 2 | 116.17 | 15.72 | 4.73 | 10.57 | 20.90 | 22.15 | 19.13 | 41.21 | 14.03 | 53.82 | 16.83 | 62.58 | 12.63 | 18.00 | 9.65 | 25.44 |
| Bleak | 1140 | 3 | 2 | 2 | 92.84 | 15.18 | 5.19 | 7.95 | 20.21 | 22.08 | 19.59 | 41.28 | 14.10 | 52.35 | 18.64 | 59.95 | 13.36 | 18.17 | 8.90 | 21.90 |
| Bleak | 1141 | 3 | 2 | 2 | 69.20 | 15.37 | 5.32 | 8.80 | 20.52 | 22.03 | 20.03 | 42.99 | 12.73 | 52.59 | 17.51 | 59.79 | 14.21 | 19.48 | 9.24 | 23.82 |
| Bleak | 1142 | 3 | 2 | 2 | 108.11 | 14.70 | 4.56 | 9.23 | 20.18 | 21.88 | 20.04 | 39.62 | 13.20 | 51.37 | 18.33 | 60.91 | 13.84 | 15.59 | 9.14 | 22.59 |
| Bleak | 1143 | 3 | 2 | 2 | 88.49 | 14.31 | 4.92 | 8.35 | 19.77 | 20.94 | 18.48 | 40.27 | 14.21 | 50.92 | 18.09 | 57.04 | 13.64 | 18.05 | 8.59 | 21.87 |
| Bleak | 1144 | 3 | 2 | 2 | 94.73 | 14.17 | 4.86 | 8.00 | 19.82 | 21.85 | 18.63 | 41.51 | 13.94 | 54.10 | 17.79 | 62.81 | 13.77 | 19.27 | 8.69 | 22.82 |
| Bleak | 1145 | 3 | 2 | 2 | 78.82 | 14.82 | 4.26 | 8.46 | 20.55 | 21.64 | 20.31 | 41.59 | 14.77 | 51.23 | 18.62 | 62.51 | 14.18 | 20.31 | 9.47 | 24.44 |
| Bleak | 1146 | 3 | 2 | 2 | 106.53 | 14.47 | 4.34 | 8.59 | 19.76 | 21.78 | 19.15 | 40.94 | 14.54 | 53.51 | 17.75 | 61.04 | 13.29 | 18.73 | 9.06 | 23.67 |
| Bleak | 1147 | 3 | 2 | 2 | 108.50 | 14.69 | 5.61 | 8.38 | 20.65 | 21.72 | 17.26 | 41.06 | 14.63 | 51.87 | 18.30 | 61.74 | 13.95 | 19.07 | 9.20 | 23.07 |
| Bleak | 1148 | 3 | 2 | 2 | 104.04 | 14.36 | 5.56 | 8.53 | 20.81 | 22.27 | 19.22 | 43.13 | 13.84 | 53.81 | 16.73 | 59.76 | 11.92 | 21.42 | 8.55 | 24.06 |
| Bleak | 1149 | 3 | 2 | 2 | 107.06 | 14.89 | 4.67 | 8.21 | 19.70 | 21.89 | 20.01 | 41.05 | 13.31 | 54.67 | 16.03 | 56.26 | 13.61 | 18.05 | 9.24 | 24.33 |
| Bleak | 1150 | 3 | 2 | 2 | 90.76 | 14.71 | 5.18 | 8.67 | 19.83 | 22.02 | 17.81 | 42.02 | 13.84 | 51.56 | 16.27 | 59.65 | 12.46 | 16.29 | 9.12 | 24.20 |
| Bleak | 1151 | 3 | 2 | 2 | 84.40 | 15.40 | 5.08 | 8.65 | 20.51 | 22.53 | 19.90 | 41.17 | 13.15 | 51.78 | 16.71 | 59.27 | 12.38 | 17.56 | 8.98 | 23.71 |
| Bleak | 1152 | 3 | 2 | 2 | 103.39 | 14.57 | 5.74 | 8.56 | 20.30 | 22.83 | 18.39 | 42.20 | 13.11 | 55.80 | 16.52 | 61.65 | 12.89 | 18.70 | 8.89 | 22.76 |
| Bleak | 1153 | 3 | 2 | 2 | 108.80 | 14.35 | 4.80 | 8.85 | 19.74 | 21.50 | 20.08 | 42.08 | 13.84 | 53.01 | 18.09 | 59.38 | 13.82 | 17.74 | 9.00 | 22.05 |
| Bleak | 1154 | 3 | 2 | 2 | 82.54 | 15.40 | 4.87 | 8.67 | 20.54 | 21.38 | 18.98 | 39.91 | 13.65 | 48.64 | 17.89 | 56.66 | 13.08 | 18.06 | 9.45 | 23.06 |
| Bleak | 1155 | 3 | 2 | 2 | 106.70 | 14.98 | 5.67 | 8.63 | 20.96 | 23.00 | 19.81 | 42.42 | 14.51 | 52.20 | 18.99 | 60.79 | 14.16 | 18.90 | 9.83 | 25.14 |
| Bleak | 1156 | 3 | 2 | 2 | 97.40 | 15.23 | 5.32 | 8.53 | 21.12 | 22.09 | 18.65 | 42.97 | 13.71 | 51.24 | 17.01 | 61.17 | 13.29 | 18.57 | 9.39 | 24.43 |
| Bleak | 1157 | 3 | 2 | 2 | 103.20 | 13.93 | 4.62 | 9.02 | 20.99 | 22.80 | 19.58 | 43.20 | 14.20 | 56.53 | 18.05 | 64.25 | 13.81 | 18.20 | 9.30 | 22.66 |
| Bleak | 1158 | 3 | 2 | 2 | 89.66 | 15.13 | 5.51 | 8.64 | 21.69 | 24.63 | 18.93 | 42.78 | 13.94 | 52.93 | 17.73 | 63.45 | 14.15 | 17.37 | 9.16 | 23.75 |
| Bleak | 1159 | 3 | 2 | 2 | 77.90 | 14.82 | 5.15 | 9.41 | 20.03 | 22.62 | 19.01 | 44.25 | 14.48 | 54.29 | 17.07 | 60.22 | 12.96 | 19.02 | 9.15 | 23.91 |
| Bleak | 1160 | 3 | 2 | 2 | 92.75 | 14.75 | 4.44 | 8.21 | 18.99 | 20.72 | 17.48 | 41.69 | 13.75 | 52.32 | 17.45 | 59.22 | 13.17 | 16.29 | 10.03 | 20.42 |
| Bleak | 1131 | 3 | 2 | 3 | **107.75** | 14.01 | 6.09 | 8.97 | 20.22 | 22.04 | 19.16 | 39.96 | 13.28 | 52.53 | 17.85 | 59.41 | 12.68 | 18.40 | 8.84 | 23.19 |
| Bleak | 1132 | 3 | 2 | 3 | **85.92** | 15.51 | 4.63 | 8.99 | 20.41 | 21.69 | 18.30 | 40.20 | 14.68 | 52.43 | 17.01 | 58.05 | 13.76 | 18.44 | 9.37 | 23.30 |
| Bleak | 1133 | 3 | 2 | 3 | **87.82** | 15.22 | 5.43 | 8.87 | 19.91 | 20.92 | 20.56 | 39.34 | 15.32 | 52.03 | 18.39 | 60.81 | 14.19 | 17.66 | 8.96 | 22.89 |
| Bleak | 1134 | 3 | 2 | 3 | **77.06** | 15.21 | 6.64 | 9.50 | 20.62 | 22.10 | 19.17 | 44.07 | 16.35 | 52.31 | 18.54 | 61.14 | 14.90 | 16.49 | 8.99 | 23.54 |
| Bleak | 1135 | 3 | 2 | 3 | **84.73** | 15.04 | 5.48 | 8.51 | 20.43 | 20.80 | 19.64 | 41.79 | 14.19 | 50.22 | 16.39 | 56.78 | 12.11 | 19.75 | 9.03 | 23.59 |
| Bleak | 1136 | 3 | 2 | 3 | **78.71** | 14.36 | 5.79 | 9.38 | 19.79 | 20.56 | 19.73 | 38.86 | 14.77 | 51.53 | 16.91 | 59.78 | 13.61 | 17.72 | 8.43 | 22.69 |
| Bleak | 1137 | 3 | 2 | 3 | **91.77** | 14.58 | 5.17 | 8.97 | 21.11 | 22.61 | 19.96 | 41.94 | 13.44 | 54.61 | 16.26 | 60.20 | 12.34 | 17.05 | 9.29 | 22.79 |
| Bleak | 1138 | 3 | 2 | 3 | **94.20** | 13.94 | 5.26 | 9.58 | 21.26 | 22.44 | 21.51 | 44.33 | 14.73 | 54.08 | 19.02 | 60.63 | 13.56 | 18.84 | 7.67 | 21.88 |
| Bleak | 1139 | 3 | 2 | 3 | **117.23** | 15.06 | 4.98 | 9.55 | 21.14 | 22.36 | 19.28 | 42.77 | 13.80 | 52.73 | 17.46 | 62.25 | 12.16 | 16.22 | 9.20 | 25.15 |
| Bleak | 1140 | 3 | 2 | 3 | **92.00** | 14.12 | 5.89 | 9.02 | 20.46 | 22.02 | 19.57 | 42.04 | 14.69 | 54.67 | 18.12 | 61.02 | 14.01 | 16.94 | 8.34 | 21.70 |
| Bleak | 1141 | 3 | 2 | 3 | **68.53** | 15.35 | 5.93 | 8.42 | 21.45 | 22.05 | 20.24 | 42.99 | 12.51 | 52.20 | 17.56 | 61.83 | 12.23 | 18.93 | 8.89 | 23.17 |
| Bleak | 1142 | 3 | 2 | 3 | **108.30** | 14.20 | 5.05 | 8.44 | 20.13 | 20.89 | 19.03 | 41.00 | 14.06 | 52.11 | 18.19 | 60.02 | 12.86 | 18.14 | 9.22 | 22.37 |
| Bleak | 1143 | 3 | 2 | 3 | **80.40** | 15.53 | 5.46 | 9.51 | 21.40 | 21.90 | 20.30 | 43.32 | 15.69 | 55.79 | 19.75 | 63.08 | 15.32 | 18.82 | 9.10 | 23.74 |
| Bleak | 1144 | 3 | 2 | 3 | **94.49** | 13.82 | 5.91 | 9.30 | 20.32 | 22.23 | 19.23 | 44.26 | 13.19 | 54.02 | 16.50 | 60.98 | 12.64 | 17.86 | 8.42 | 22.28 |
| Bleak | 1145 | 3 | 2 | 3 | **79.65** | 14.67 | 5.52 | 8.98 | 20.08 | 22.72 | 19.54 | 41.90 | 14.75 | 51.19 | 18.86 | 58.82 | 13.92 | 17.52 | 9.14 | 23.60 |
| Bleak | 1146 | 3 | 2 | 3 | **105.52** | 15.75 | 5.86 | 8.71 | 20.61 | 21.56 | 19.56 | 42.45 | 15.28 | 52.87 | 17.78 | 60.67 | 14.35 | 17.72 | 9.07 | 23.56 |
| Bleak | 1147 | 3 | 2 | 3 | **107.98** | 15.00 | 5.68 | 8.50 | 21.11 | 22.46 | 18.62 | 42.59 | 14.12 | 53.31 | 17.75 | 60.03 | 14.10 | 16.90 | 8.90 | 23.22 |
| Bleak | 1148 | 3 | 2 | 3 | **105.74** | 14.40 | 5.46 | 8.92 | 20.48 | 21.53 | 18.90 | 41.02 | 14.14 | 52.43 | 16.91 | 59.93 | 12.84 | 19.30 | 8.14 | 22.91 |
| Bleak | 1149 | 3 | 2 | 3 | **105.21** | 14.98 | 5.26 | 8.59 | 21.17 | 22.89 | 19.45 | 41.82 | 15.21 | 54.17 | 17.44 | 60.68 | 13.24 | 18.44 | 9.45 | 24.50 |
| Bleak | 1150 | 3 | 2 | 3 | **90.58** | 15.12 | 5.30 | 8.70 | 21.21 | 22.00 | 16.46 | 41.50 | 14.77 | 51.95 | 16.07 | 59.30 | 12.85 | 17.61 | 9.06 | 24.23 |
| Bleak | 1151 | 3 | 2 | 3 | **84.59** | 15.29 | 5.40 | 9.08 | 20.94 | 22.27 | 19.86 | 42.64 | 13.75 | 51.63 | 16.93 | 59.07 | 11.94 | 19.39 | 9.00 | 23.65 |
| Bleak | 1152 | 3 | 2 | 3 | **104.55** | 14.00 | 6.01 | 8.94 | 20.27 | 21.39 | 18.51 | 41.41 | 12.66 | 53.06 | 16.41 | 60.76 | 11.90 | 16.09 | 8.14 | 22.37 |
| Bleak | 1153 | 3 | 2 | 3 | **107.43** | 14.80 | 5.37 | 9.01 | 20.36 | 21.77 | 20.65 | 43.27 | 13.73 | 53.34 | 18.53 | 60.60 | 12.91 | 16.40 | 8.86 | 22.58 |
| Bleak | 1154 | 3 | 2 | 3 | **80.93** | 15.57 | 4.93 | 8.05 | 20.43 | 20.66 | 19.28 | 40.20 | 12.93 | 53.21 | 18.06 | 58.52 | 12.75 | 17.45 | 8.82 | 23.39 |
| Bleak | 1155 | 3 | 2 | 3 | **106.00** | 14.90 | 5.57 | 9.04 | 21.02 | 22.88 | 20.41 | 41.85 | 14.63 | 50.79 | 18.23 | 60.02 | 14.02 | 17.85 | 9.79 | 25.24 |
| Bleak | 1156 | 3 | 2 | 3 | **97.43** | 15.02 | 5.91 | 10.34 | 20.68 | 23.22 | 19.30 | 43.15 | 14.26 | 52.29 | 17.89 | 62.57 | 12.75 | 18.57 | 9.08 | 23.76 |
| Bleak | 1157 | 3 | 2 | 3 | **102.86** | 13.78 | 5.73 | 10.08 | 21.26 | 22.91 | 20.47 | 44.19 | 14.58 | 56.38 | 18.25 | 62.80 | 13.28 | 17.17 | 8.97 | 22.41 |
| Bleak | 1158 | 3 | 2 | 3 | **90.01** | 16.36 | 6.31 | 9.21 | 21.34 | 23.91 | 19.36 | 43.09 | 14.59 | 54.77 | 18.31 | 59.36 | 12.34 | 17.91 | 8.94 | 23.18 |
| Bleak | 1159 | 3 | 2 | 3 | **76.77** | 14.96 | 5.45 | 10.18 | 21.44 | 23.54 | 18.56 | 44.63 | 14.65 | 52.89 | 17.75 | 58.95 | 13.29 | 16.15 | 9.17 | 23.68 |
| Bleak | 1160 | 3 | 2 | 3 | **92.24** | 14.72 | 4.82 | 8.43 | 20.08 | 23.25 | 19.07 | 41.13 | 13.56 | 52.96 | 16.75 | 57.45 | 14.04 | 17.98 | 8.08 | 21.30 |
| Bleak | 1221 | 3 | 3 | 1 | 103.95 | 16.13 | 4.98 | 7.69 | 19.66 | 21.10 | 18.37 | 40.69 | 13.15 | 52.29 | 17.07 | 62.50 | 12.24 | 14.67 | 9.96 | 23.57 |
| Bleak | 1222 | 3 | 3 | 1 | 86.68 | 13.93 | 6.44 | 9.68 | 19.95 | 22.38 | 19.34 | 44.36 | 13.24 | 53.88 | 16.97 | 61.84 | 13.23 | 18.42 | 8.59 | 22.39 |
| Bleak | 1223 | 3 | 3 | 1 | 87.71 | 15.13 | 5.23 | 8.86 | 19.18 | 22.52 | 18.98 | 44.46 | 12.73 | 54.30 | 18.71 | 61.52 | 12.95 | 17.98 | 8.94 | 21.67 |
| Bleak | 1224 | 3 | 3 | 1 | 105.01 | 14.76 | 4.92 | 9.27 | 19.11 | 22.52 | 18.47 | 42.71 | 13.91 | 52.23 | 17.44 | 60.53 | 11.92 | 21.98 | 9.65 | 22.30 |
| Bleak | 1225 | 3 | 3 | 1 | 102.98 | 14.08 | 5.88 | 8.94 | 19.61 | 21.25 | 19.04 | 43.36 | 12.62 | 51.94 | 17.35 | 61.50 | 12.61 | 18.53 | 9.15 | 22.05 |
| Bleak | 1226 | 3 | 3 | 1 | 78.84 | 14.79 | 5.31 | 8.20 | 19.51 | 22.31 | 19.34 | 43.94 | 14.41 | 53.19 | 17.87 | 60.54 | 14.09 | 18.86 | 9.10 | 22.79 |
| Bleak | 1227 | 3 | 3 | 1 | 88.88 | 14.01 | 4.90 | 9.97 | 19.36 | 22.28 | 19.99 | 43.29 | 13.65 | 52.89 | 16.97 | 63.65 | 12.20 | 19.01 | 9.13 | 23.74 |
| Bleak | 1228 | 3 | 3 | 1 | 92.07 | 15.25 | 6.03 | 9.10 | 20.90 | 22.02 | 16.62 | 41.51 | 12.24 | 52.82 | 14.69 | 59.74 | 12.50 | 18.96 | 8.91 | 21.48 |
| Bleak | 1229 | 3 | 3 | 1 | 88.19 | 15.71 | 5.02 | 9.67 | 19.03 | 22.01 | 18.51 | 42.08 | 13.98 | 51.32 | 19.00 | 60.24 | 13.06 | 18.97 | 9.20 | 23.98 |
| Bleak | 1230 | 3 | 3 | 1 | 101.40 | 14.05 | 5.72 | 8.38 | 19.80 | 22.03 | 19.01 | 41.84 | 13.74 | 52.67 | 16.46 | 60.54 | 12.52 | 17.64 | 9.27 | 23.76 |
| Bleak | 1231 | 3 | 3 | 1 | 87.75 | 13.22 | 5.65 | 8.76 | 19.65 | 21.63 | 20.50 | 44.35 | 14.55 | 53.27 | 17.20 | 59.35 | 11.91 | 18.22 | 9.56 | 23.89 |
| Bleak | 1232 | 3 | 3 | 1 | 130.22 | 15.01 | 5.59 | 8.72 | 19.42 | 21.49 | 17.92 | 43.87 | 12.98 | 53.18 | 16.83 | 59.18 | 13.68 | 18.77 | 9.40 | 23.17 |
| Bleak | 1233 | 3 | 3 | 1 | 96.35 | 14.77 | 6.24 | 9.12 | 21.01 | 23.35 | 18.80 | 41.96 | 12.52 | 54.13 | 17.54 | 58.73 | 11.06 | 18.66 | 9.47 | 23.43 |
| Bleak | 1234 | 3 | 3 | 1 | 98.99 | 14.40 | 4.62 | 8.86 | 18.28 | 19.84 | 18.56 | 43.99 | 12.70 | 50.60 | 17.25 | 58.28 | 13.49 | 18.44 | 9.21 | 21.67 |
| Bleak | 1235 | 3 | 3 | 1 | 93.54 | 16.14 | 5.79 | 9.49 | 20.89 | 22.91 | 18.89 | 44.07 | 13.93 | 51.61 | 18.58 | 61.09 | 13.35 | 19.21 | 9.41 | 22.80 |
| Bleak | 1236 | 3 | 3 | 1 | 88.16 | 14.96 | 5.25 | 8.80 | 19.68 | 23.07 | 18.62 | 46.70 | 12.92 | 52.85 | 17.22 | 61.31 | 12.24 | 18.62 | 9.52 | 23.39 |
| Bleak | 1237 | 3 | 3 | 1 | 99.22 | 14.38 | 5.95 | 7.41 | 19.81 | 21.01 | 18.56 | 45.80 | 14.61 | 56.18 | 17.03 | 64.37 | 12.94 | 18.57 | 8.88 | 23.61 |
| Bleak | 1238 | 3 | 3 | 1 | 103.38 | 15.11 | 4.35 | 8.64 | 19.47 | 21.82 | 18.74 | 44.57 | 12.47 | 52.08 | 16.39 | 61.32 | 12.02 | 15.78 | 8.69 | 23.56 |
| Bleak | 1239 | 3 | 3 | 1 | 95.31 | 14.56 | 5.59 | 8.97 | 19.38 | 22.58 | 19.87 | 42.72 | 13.54 | 54.41 | 17.54 | 62.00 | 13.89 | 18.21 | 9.19 | 22.19 |
| Bleak | 1240 | 3 | 3 | 1 | 90.82 | 14.80 | 5.26 | 9.44 | 20.05 | 22.48 | 18.69 | 40.63 | 13.21 | 53.86 | 15.64 | 61.67 | 11.56 | 17.71 | 9.65 | 22.54 |
| Bleak | 1241 | 3 | 3 | 1 | 110.93 | 15.47 | 5.72 | 9.17 | 21.16 | 23.18 | 19.34 | 43.49 | 14.42 | 53.56 | 18.92 | 58.84 | 14.12 | 17.99 | 8.89 | 22.50 |
| Bleak | 1242 | 3 | 3 | 1 | 101.13 | 13.69 | 5.72 | 8.86 | 19.58 | 21.42 | 18.52 | 42.45 | 13.99 | 53.25 | 17.24 | 65.50 | 12.50 | 17.85 | 9.19 | 24.04 |
| Bleak | 1243 | 3 | 3 | 1 | 97.90 | 16.86 | 6.11 | 7.95 | 19.02 | 20.87 | 19.21 | 40.44 | 13.61 | 52.94 | 17.54 | 62.55 | 13.22 | 17.22 | 9.54 | 24.38 |
| Bleak | 1244 | 3 | 3 | 1 | 90.01 | 15.24 | 5.98 | 9.60 | 21.03 | 24.93 | 18.80 | 45.36 | 13.54 | 56.99 | 18.68 | 63.23 | 13.18 | 15.64 | 8.86 | 23.71 |
| Bleak | 1245 | 3 | 3 | 1 | 93.25 | 14.87 | 5.28 | 8.30 | 18.85 | 20.21 | 20.07 | 41.14 | 13.87 | 51.55 | 18.57 | 62.43 | 13.67 | 19.56 | 10.09 | 23.49 |
| Bleak | 1246 | 3 | 3 | 1 | 92.72 | 13.61 | 6.12 | 8.00 | 20.36 | 23.77 | 19.17 | 39.76 | 13.62 | 52.02 | 18.36 | 62.39 | 11.17 | 16.87 | 9.11 | 22.84 |
| Bleak | 1247 | 3 | 3 | 1 | 96.29 | 15.32 | 5.47 | 7.57 | 19.15 | 21.58 | 18.01 | 41.03 | 13.40 | 50.59 | 18.93 | 59.38 | 13.48 | 14.60 | 9.14 | 22.77 |
| Bleak | 1248 | 3 | 3 | 1 | 63.75 | 15.02 | 5.60 | 9.97 | 20.34 | 22.34 | 17.85 | 43.52 | 13.81 | 52.29 | 16.68 | 61.15 | 11.16 | 18.64 | 8.62 | 22.16 |
| Bleak | 1249 | 3 | 3 | 1 | 75.09 | 14.25 | 5.64 | 9.87 | 20.40 | 22.02 | 19.22 | 44.53 | 13.37 | 53.26 | 18.11 | 62.41 | 11.35 | 18.51 | 8.81 | 22.68 |
| Bleak | 1250 | 3 | 3 | 1 | 83.75 | 14.13 | 5.90 | 8.29 | 19.05 | 23.22 | 19.40 | 44.50 | 12.43 | 53.17 | 18.04 | 61.58 | 12.21 | 16.42 | 9.44 | 22.72 |
| Bleak | 1221 | 3 | 3 | 2 | 103.72 | 16.25 | 5.69 | 9.87 | 20.62 | 22.59 | 18.10 | 43.22 | 13.73 | 53.94 | 16.53 | 62.74 | 12.14 | 16.48 | 10.06 | 24.42 |
| Bleak | 1222 | 3 | 3 | 2 | 86.67 | 13.86 | 5.34 | 9.37 | 20.17 | 23.18 | 20.05 | 40.27 | 13.70 | 52.56 | 18.20 | 61.95 | 13.35 | 17.55 | 8.74 | 21.35 |
| Bleak | 1223 | 3 | 3 | 2 | 87.08 | 14.77 | 4.63 | 9.44 | 20.78 | 23.74 | 20.30 | 43.85 | 15.61 | 53.71 | 18.77 | 61.52 | 12.78 | 17.14 | 8.63 | 21.35 |
| Bleak | 1224 | 3 | 3 | 2 | 105.86 | 14.76 | 5.51 | 9.42 | 19.80 | 22.10 | 18.61 | 42.20 | 14.76 | 51.61 | 17.55 | 59.40 | 12.30 | 19.11 | 9.17 | 22.04 |
| Bleak | 1225 | 3 | 3 | 2 | 100.64 | 14.54 | 5.60 | 9.84 | 19.85 | 21.52 | 20.34 | 45.56 | 13.43 | 51.04 | 19.39 | 62.76 | 14.10 | 18.71 | 9.00 | 22.19 |
| Bleak | 1226 | 3 | 3 | 2 | 78.20 | 14.78 | 4.77 | 9.60 | 20.11 | 22.44 | 20.58 | 41.95 | 14.86 | 53.82 | 17.51 | 60.17 | 13.92 | 19.21 | 9.66 | 22.28 |
| Bleak | 1227 | 3 | 3 | 2 | 88.68 | 14.71 | 5.29 | 9.73 | 20.21 | 23.72 | 19.28 | 42.41 | 14.54 | 53.65 | 17.16 | 63.18 | 12.88 | 19.71 | 9.45 | 22.83 |
| Bleak | 1228 | 3 | 3 | 2 | 89.65 | 14.45 | 5.53 | 9.31 | 20.00 | 22.70 | 17.34 | 43.52 | 12.87 | 53.46 | 15.91 | 61.06 | 12.31 | 19.85 | 8.41 | 22.32 |
| Bleak | 1229 | 3 | 3 | 2 | 86.50 | 16.54 | 5.85 | 8.24 | 19.84 | 22.11 | 19.56 | 42.56 | 15.14 | 53.56 | 18.71 | 60.93 | 13.33 | 20.00 | 9.60 | 22.76 |
| Bleak | 1230 | 3 | 3 | 2 | 100.38 | 14.72 | 5.65 | 8.75 | 20.42 | 23.11 | 19.77 | 41.20 | 13.96 | 53.20 | 17.87 | 60.09 | 13.08 | 18.45 | 9.58 | 23.13 |
| Bleak | 1231 | 3 | 3 | 2 | 87.81 | 13.67 | 5.78 | 9.39 | 20.30 | 22.72 | 21.59 | 42.56 | 14.97 | 53.01 | 17.82 | 62.20 | 12.65 | 16.38 | 9.50 | 23.70 |
| Bleak | 1232 | 3 | 3 | 2 | 128.92 | 15.30 | 6.36 | 8.80 | 20.03 | 22.53 | 19.58 | 43.80 | 13.39 | 55.30 | 17.13 | 61.58 | 13.20 | 17.98 | 9.52 | 23.11 |
| Bleak | 1233 | 3 | 3 | 2 | 95.73 | 14.58 | 6.17 | 9.68 | 21.54 | 24.10 | 18.62 | 40.58 | 12.53 | 55.50 | 16.05 | 58.69 | 11.92 | 20.30 | 9.39 | 23.36 |
| Bleak | 1234 | 3 | 3 | 2 | 98.06 | 14.43 | 5.63 | 8.33 | 19.40 | 20.39 | 18.54 | 42.36 | 14.17 | 50.26 | 17.08 | 59.53 | 12.69 | 18.01 | 9.04 | 21.69 |
| Bleak | 1235 | 3 | 3 | 2 | 93.67 | 14.51 | 6.24 | 8.87 | 20.86 | 23.42 | 18.36 | 42.11 | 15.94 | 53.12 | 19.25 | 61.24 | 11.67 | 19.64 | 9.66 | 22.95 |
| Bleak | 1236 | 3 | 3 | 2 | 87.89 | 15.33 | 4.64 | 8.46 | 20.25 | 23.33 | 19.20 | 44.44 | 14.43 | 54.75 | 18.18 | 60.49 | 12.98 | 18.66 | 9.33 | 22.55 |
| Bleak | 1237 | 3 | 3 | 2 | 100.06 | 14.94 | 5.81 | 8.59 | 19.46 | 21.12 | 18.36 | 42.08 | 13.01 | 52.63 | 15.66 | 61.49 | 11.76 | 17.24 | 9.07 | 23.13 |
| Bleak | 1238 | 3 | 3 | 2 | 102.48 | 14.71 | 5.83 | 9.17 | 20.09 | 23.67 | 18.72 | 43.87 | 13.91 | 52.53 | 16.11 | 62.32 | 12.69 | 17.79 | 8.96 | 23.63 |
| Bleak | 1239 | 3 | 3 | 2 | 95.36 | 14.11 | 5.21 | 9.80 | 20.53 | 23.07 | 19.06 | 42.28 | 14.07 | 52.86 | 18.34 | 58.28 | 13.47 | 17.65 | 9.44 | 22.50 |
| Bleak | 1240 | 3 | 3 | 2 | 90.86 | 15.05 | 5.64 | 7.76 | 19.72 | 22.51 | 18.04 | 40.90 | 11.92 | 53.11 | 16.07 | 59.66 | 11.33 | 17.94 | 9.40 | 22.02 |
| Bleak | 1241 | 3 | 3 | 2 | 109.23 | 14.75 | 6.23 | 9.79 | 21.31 | 22.96 | 20.14 | 42.40 | 14.92 | 53.78 | 18.49 | 60.69 | 14.58 | 19.83 | 9.26 | 22.94 |
| Bleak | 1242 | 3 | 3 | 2 | 101.27 | 15.29 | 5.52 | 9.14 | 19.67 | 21.45 | 19.72 | 41.72 | 15.89 | 53.46 | 19.34 | 63.08 | 12.02 | 19.34 | 9.54 | 24.03 |
| Bleak | 1243 | 3 | 3 | 2 | 98.50 | 15.53 | 4.71 | 7.83 | 19.13 | 21.94 | 19.68 | 42.40 | 14.83 | 53.50 | 18.79 | 62.55 | 13.80 | 18.62 | 9.61 | 24.27 |
| Bleak | 1244 | 3 | 3 | 2 | 89.73 | 15.56 | 5.33 | 9.26 | 22.56 | 26.63 | 18.89 | 43.34 | 15.39 | 55.84 | 18.34 | 62.52 | 13.69 | 16.60 | 9.05 | 24.17 |
| Bleak | 1245 | 3 | 3 | 2 | 92.08 | 14.58 | 4.90 | 8.65 | 20.10 | 21.79 | 19.87 | 40.67 | 14.98 | 52.87 | 18.81 | 60.77 | 13.19 | 19.38 | 10.60 | 23.96 |
| Bleak | 1246 | 3 | 3 | 2 | 92.12 | 14.36 | 6.31 | 8.75 | 20.37 | 23.99 | 19.01 | 40.52 | 16.20 | 50.54 | 17.85 | 60.67 | 11.93 | 16.57 | 9.92 | 22.29 |
| Bleak | 1247 | 3 | 3 | 2 | 96.41 | 15.03 | 5.80 | 8.48 | 19.60 | 23.15 | 17.85 | 45.16 | 13.35 | 51.31 | 15.93 | 60.89 | 12.89 | 15.97 | 9.03 | 21.83 |
| Bleak | 1248 | 3 | 3 | 2 | 63.57 | 15.14 | 5.68 | 10.21 | 19.21 | 22.11 | 18.61 | 45.54 | 14.58 | 56.61 | 17.41 | 60.69 | 13.00 | 18.27 | 8.46 | 22.56 |
| Bleak | 1249 | 3 | 3 | 2 | 75.30 | 14.61 | 5.72 | 9.30 | 19.55 | 23.29 | 20.09 | 44.38 | 16.41 | 51.96 | 18.40 | 62.47 | 13.01 | 17.08 | 9.13 | 22.12 |
| Bleak | 1250 | 3 | 3 | 2 | 81.78 | 15.09 | 5.71 | 10.21 | 20.95 | 24.66 | 18.77 | 44.28 | 14.01 | 56.27 | 16.78 | 61.59 | 13.72 | 17.63 | 9.90 | 23.20 |
| Bleak | 1221 | 3 | 3 | 3 | 104.38 | 16.46 | 5.56 | 9.73 | 20.48 | 22.87 | 17.70 | 42.94 | 13.62 | 52.74 | 16.63 | 60.92 | 11.92 | 18.09 | 9.54 | 24.07 |
| Bleak | 1222 | 3 | 3 | 3 | 86.80 | 13.49 | 5.16 | 9.98 | 21.04 | 22.75 | 18.21 | 41.89 | 12.60 | 54.29 | 17.72 | 61.08 | 12.74 | 17.61 | 8.12 | 22.25 |
| Bleak | 1223 | 3 | 3 | 3 | 88.68 | 14.67 | 5.20 | 9.18 | 20.04 | 22.36 | 20.08 | 41.72 | 13.23 | 52.73 | 17.92 | 60.06 | 13.03 | 17.35 | 8.37 | 21.15 |
| Bleak | 1224 | 3 | 3 | 3 | 105.34 | 14.35 | 5.21 | 10.25 | 20.19 | 22.10 | 19.15 | 37.24 | 13.46 | 51.89 | 17.25 | 59.25 | 11.94 | 20.34 | 9.17 | 21.77 |
| Bleak | 1225 | 3 | 3 | 3 | 101.26 | 14.87 | 5.68 | 9.37 | 20.62 | 21.61 | 20.11 | 42.94 | 14.61 | 52.26 | 17.58 | 61.70 | 12.99 | 17.74 | 8.85 | 21.37 |
| Bleak | 1226 | 3 | 3 | 3 | 78.58 | 15.05 | 5.83 | 10.95 | 21.30 | 22.58 | 17.45 | 43.13 | 13.29 | 53.24 | 16.24 | 60.32 | 11.63 | 18.17 | 9.04 | 22.61 |
| Bleak | 1227 | 3 | 3 | 3 | 88.68 | 14.61 | 5.73 | 10.17 | 20.99 | 22.58 | 21.10 | 43.09 | 12.94 | 52.20 | 15.90 | 61.41 | 12.32 | 17.61 | 8.48 | 22.17 |
| Bleak | 1228 | 3 | 3 | 3 | 92.89 | 13.89 | 5.71 | 9.61 | 20.71 | 21.73 | 18.07 | 41.05 | 11.81 | 50.70 | 14.90 | 59.54 | 11.77 | 19.30 | 8.29 | 21.66 |
| Bleak | 1229 | 3 | 3 | 3 | 87.73 | 15.87 | 6.23 | 9.48 | 21.26 | 24.23 | 18.12 | 41.30 | 13.79 | 51.00 | 17.04 | 59.01 | 13.80 | 17.76 | 8.77 | 22.33 |
| Bleak | 1230 | 3 | 3 | 3 | 100.79 | 14.48 | 5.77 | 9.67 | 21.23 | 22.92 | 18.74 | 40.47 | 13.88 | 53.41 | 17.45 | 60.24 | 12.16 | 18.65 | 9.08 | 23.43 |
| Bleak | 1231 | 3 | 3 | 3 | 88.50 | 14.34 | 5.71 | 10.44 | 20.68 | 22.02 | 20.50 | 44.31 | 14.42 | 52.85 | 17.40 | 60.78 | 12.69 | 17.17 | 9.16 | 23.22 |
| Bleak | 1232 | 3 | 3 | 3 | 129.45 | 15.21 | 6.62 | 9.25 | 20.66 | 22.32 | 18.61 | 43.40 | 13.30 | 52.46 | 16.32 | 61.48 | 13.04 | 18.23 | 9.08 | 23.06 |
| Bleak | 1233 | 3 | 3 | 3 | 95.28 | 14.18 | 5.70 | 10.47 | 21.80 | 22.21 | 18.56 | 40.56 | 12.59 | 54.38 | 16.46 | 60.00 | 12.05 | 18.49 | 9.48 | 22.90 |
| Bleak | 1234 | 3 | 3 | 3 | 98.92 | 14.41 | 4.79 | 10.04 | 20.21 | 20.96 | 19.86 | 41.90 | 14.14 | 50.62 | 17.03 | 62.04 | 12.37 | 17.34 | 8.39 | 21.23 |
| Bleak | 1235 | 3 | 3 | 3 | 94.46 | 14.45 | 5.99 | 9.59 | 20.84 | 22.60 | 19.00 | 41.66 | 14.32 | 52.28 | 18.41 | 60.58 | 12.90 | 18.06 | 8.93 | 21.88 |
| Bleak | 1236 | 3 | 3 | 3 | 87.32 | 14.85 | 5.02 | 10.12 | 20.80 | 22.30 | 19.76 | 43.17 | 13.50 | 53.76 | 15.95 | 60.68 | 12.23 | 16.43 | 8.77 | 23.01 |
| Bleak | 1237 | 3 | 3 | 3 | 100.18 | 14.93 | 5.87 | 9.66 | 20.33 | 22.62 | 19.45 | 40.97 | 13.09 | 52.02 | 16.65 | 60.41 | 11.62 | 17.44 | 8.41 | 22.10 |
| Bleak | 1238 | 3 | 3 | 3 | 103.77 | 14.54 | 5.11 | 9.78 | 20.12 | 22.53 | 18.50 | 43.87 | 12.83 | 51.90 | 17.52 | 61.67 | 12.03 | 17.31 | 8.69 | 23.27 |
| Bleak | 1239 | 3 | 3 | 3 | 95.52 | 14.04 | 4.91 | 9.87 | 20.78 | 22.45 | 20.39 | 42.69 | 14.42 | 52.39 | 17.25 | 60.77 | 13.78 | 17.20 | 8.78 | 22.04 |
| Bleak | 1240 | 3 | 3 | 3 | 91.28 | 14.67 | 5.20 | 9.85 | 21.10 | 21.95 | 18.92 | 39.11 | 12.67 | 53.73 | 16.11 | 59.95 | 11.72 | 18.07 | 9.26 | 21.71 |
| Bleak | 1241 | 3 | 3 | 3 | 110.68 | 15.04 | 5.51 | 9.75 | 21.55 | 23.67 | 19.99 | 41.47 | 14.69 | 52.10 | 16.60 | 59.14 | 13.99 | 18.34 | 8.45 | 22.72 |
| Bleak | 1242 | 3 | 3 | 3 | 101.26 | 13.99 | 5.49 | 9.40 | 19.80 | 21.94 | 19.08 | 41.32 | 14.26 | 54.06 | 18.21 | 61.81 | 12.91 | 17.65 | 9.01 | 23.38 |
| Bleak | 1243 | 3 | 3 | 3 | 100.85 | 15.08 | 5.35 | 8.89 | 19.96 | 21.67 | 18.02 | 38.88 | 13.21 | 49.39 | 18.42 | 59.03 | 12.56 | 18.63 | 9.17 | 23.45 |
| Bleak | 1244 | 3 | 3 | 3 | 89.09 | 15.21 | 6.05 | 10.81 | 22.99 | 25.11 | 18.62 | 46.41 | 14.67 | 57.95 | 18.64 | 61.64 | 13.53 | 18.56 | 8.47 | 23.55 |
| Bleak | 1245 | 3 | 3 | 3 | 94.03 | 14.67 | 4.89 | 8.98 | 20.74 | 20.90 | 20.03 | 40.86 | 14.67 | 50.82 | 19.45 | 60.23 | 13.53 | 18.60 | 9.67 | 22.36 |
| Bleak | 1246 | 3 | 3 | 3 | 91.54 | 14.80 | 5.58 | 9.38 | 21.43 | 23.24 | 19.07 | 42.10 | 13.30 | 54.09 | 17.34 | 59.34 | 12.60 | 17.55 | 9.03 | 22.10 |
| Bleak | 1247 | 3 | 3 | 3 | 96.87 | 14.83 | 5.91 | 7.84 | 20.05 | 22.21 | 17.56 | 41.43 | 13.29 | 49.90 | 17.88 | 61.29 | 13.45 | 18.54 | 8.54 | 22.04 |
| Bleak | 1248 | 3 | 3 | 3 | 62.39 | 15.30 | 6.43 | 10.90 | 21.68 | 22.91 | 19.71 | 42.72 | 14.51 | 54.40 | 18.71 | 62.88 | 13.76 | 15.39 | 8.63 | 22.07 |
| Bleak | 1249 | 3 | 3 | 3 | 75.48 | 14.19 | 5.29 | 10.61 | 20.74 | 22.86 | 20.32 | 43.25 | 12.70 | 53.59 | 18.09 | 60.44 | 12.72 | 17.99 | 8.72 | 21.13 |
| Bleak | 1250 | 3 | 3 | 3 | 83.25 | 15.57 | 5.69 | 10.18 | 21.37 | 23.67 | 18.23 | 42.89 | 14.16 | 53.18 | 16.54 | 62.26 | 12.46 | 18.91 | 9.31 | 22.72 |
| Roach | 1001 | 1 | 1 | 1 | 90.76 | 16.8 | 6.27 | 9.47 | 21.65 | 22.7 | 15.21 | 41.42 | 16.47 | 44.76 | 22.52 | 62.34 | 13.92 | 20.96 | 9.41 | 27.99 |
| Roach | 1002 | 1 | 1 | 1 | 114.1 | 20.66 | 8.17 | 13.37 | 27.82 | 27.79 | 21.48 | 54.09 | 19.99 | 57.96 | 25.96 | 82.26 | 20.28 | 20.37 | 12.05 | 37.05 |
| Roach | 1003 | 1 | 1 | 1 | 132.79 | 25.11 | 8.52 | 13.96 | 30.32 | 29.83 | 23.07 | 60.69 | 23.27 | 64.66 | 30.6 | 91.12 | 19.43 | 21.6 | 14.07 | 44.31 |
| Roach | 1004 | 1 | 1 | 1 | 83.1 | 16.73 | 5.6 | 9.7 | 20.46 | 20.14 | 14.28 | 39.33 | 14.23 | 41.05 | 21 | 58.89 | 13.38 | 15.54 | 7.93 | 26.19 |
| Roach | 1005 | 1 | 1 | 1 | 115.04 | 23.47 | 7.44 | 14.35 | 29.46 | 29.03 | 16.27 | 58.19 | 20.22 | 61.3 | 24.86 | 85.63 | 18.82 | 18.85 | 13 | 34.91 |
| Roach | 1006 | 1 | 1 | 1 | 130.73 | 28.16 | 8.15 | 15.16 | 31.08 | 29.96 | 22.66 | 62.02 | 23.26 | 66.28 | 31.12 | 94.17 | 20.08 | 22.83 | 14.96 | 41.37 |
| Roach | 1007 | 1 | 1 | 1 | 89.67 | 18.18 | 6.29 | 10.56 | 21.94 | 22.3 | 15.11 | 42.15 | 14.89 | 46.41 | 19.95 | 62.76 | 13.71 | 16.02 | 9.38 | 28.54 |
| Roach | 1008 | 1 | 1 | 1 | 78.4 | 15.63 | 5.53 | 9.86 | 19.54 | 20.65 | 13.5 | 35.56 | 14.05 | 37.29 | 19.16 | 55.79 | 12.16 | 15.62 | 8.09 | 24.74 |
| Roach | 1009 | 1 | 1 | 1 | 88.83 | 18.56 | 6.22 | 10.03 | 22.07 | 22.09 | 15.97 | 42.63 | 15.98 | 44.48 | 20.54 | 61.75 | 13.9 | 16.29 | 9.23 | 27.57 |
| Roach | 1010 | 1 | 1 | 1 | 89.96 | 18.98 | 5.99 | 11.03 | 23.56 | 23.15 | 16.86 | 44.51 | 16.2 | 46.76 | 21.79 | 68.23 | 14.04 | 17.29 | 10.3 | 29.64 |
| Roach | 1011 | 1 | 1 | 1 | 81.25 | 16.2 | 4.83 | 9.72 | 20.01 | 20.51 | 14.08 | 38.73 | 13.91 | 39.06 | 20.18 | 50.22 | 11.78 | 16.62 | 8.3 | 25.25 |
| Roach | 1012 | 1 | 1 | 1 | 99.53 | 19.94 | 6.44 | 10.04 | 24.12 | 24.42 | 16.73 | 49.17 | 17.62 | 51.55 | 23.85 | 71.38 | 15.16 | 20.28 | 9.93 | 30.71 |
| Roach | 1013 | 1 | 1 | 1 | 91.38 | 18.18 | 6.37 | 10.17 | 21.66 | 20.88 | 18 | 42.2 | 15.51 | 46.63 | 20.91 | 61.95 | 14.9 | 20.54 | 9.74 | 27.62 |
| Roach | 1014 | 1 | 1 | 1 | 128.79 | 24.31 | 8.86 | 15.65 | 30.21 | 28.89 | 22.63 | 57.26 | 21.68 | 62.6 | 28.57 | 85.94 | 18.14 | 26.09 | 13.53 | 39.79 |
| Roach | 1015 | 1 | 1 | 1 | 125.97 | 23.77 | 8.45 | 14.56 | 31.57 | 30.23 | 22.66 | 58.46 | 22.55 | 62.69 | 26.2 | 90.48 | 17.22 | 22.28 | 13.73 | 38.94 |
| Roach | 1016 | 1 | 1 | 1 | 86.39 | 17.89 | 6.14 | 10.39 | 19.99 | 22.27 | 16.53 | 43.58 | 15.21 | 47.18 | 21.13 | 60.26 | 13.51 | 15.32 | 9.74 | 29.2 |
| Roach | 1016 | 1 | 1 | 1 | 70.69 | 11.66 | 4.26 | 8.91 | 18.23 | 17.63 | 10.82 | 34.24 | 12.81 | 36.1 | 18.7 | 49.96 | 11.7 | 14.38 | 6.41 | 22.43 |
| Roach | 1017 | 1 | 1 | 1 | 108.94 | 20.57 | 7.02 | 12.44 | 25.01 | 26.34 | 20.78 | 49.47 | 20.39 | 52.95 | 26.79 | 75.86 | 18.18 | 20.44 | 11.07 | 35.05 |
| Roach | 1019 | 1 | 1 | 1 | 91.8 | 18.96 | 6.3 | 10.51 | 22.2 | 23.03 | 15.98 | 45.73 | 15.3 | 49.05 | 21.33 | 66.15 | 15.18 | 13.75 | 9.45 | 29.01 |
| Roach | 1020 | 1 | 1 | 1 | 117.63 | 22.63 | 7.11 | 12.99 | 26.45 | 29.1 | 20.12 | 56.18 | 20.51 | 60.62 | 27.22 | 84.35 | 17.66 | 17.15 | 12.06 | 38.45 |
| Roach | 1021 | 1 | 1 | 1 | 110.86 | 20.36 | 7.06 | 12.04 | 25.63 | 26.96 | 20.05 | 53.8 | 19.02 | 58.5 | 26.76 | 77.77 | 17.35 | 17.54 | 12.29 | 33.81 |
| Roach | 1022 | 1 | 1 | 1 | 94.05 | 18.65 | 5.96 | 12.68 | 23.93 | 24.47 | 13.64 | 46.78 | 15.56 | 46.21 | 19.36 | 68.43 | 13.38 | 18.52 | 9.49 | 26.32 |
| Roach | 1023 | 1 | 1 | 1 | 80.35 | 14.95 | 4.85 | 8.59 | 19.66 | 18.98 | 13.04 | 38.22 | 14.58 | 40.02 | 19.1 | 57.94 | 12.91 | 15.25 | 8.24 | 23.26 |
| Roach | 1024 | 1 | 1 | 1 | 95.67 | 20.71 | 6.73 | 10.7 | 23.34 | 24.85 | 15.41 | 44.15 | 18.29 | 46.77 | 24.92 | 66.58 | 14.98 | 18.64 | 10.46 | 31.43 |
| Roach | 1025 | 1 | 1 | 1 | 95.31 | 18.09 | 5.92 | 10.84 | 23.89 | 24.73 | 17.38 | 46.95 | 16.07 | 46.6 | 24 | 65.93 | 15.18 | 16.43 | 10 | 31 |
| Roach | 1026 | 1 | 1 | 1 | 117.96 | 21.48 | 7.95 | 12.9 | 28.02 | 28.51 | 19.34 | 56.06 | 18.67 | 61.53 | 24.95 | 83.57 | 16.87 | 18.01 | 12.26 | 38.35 |
| Roach | 1027 | 1 | 1 | 1 | 100.7 | 20.01 | 6.06 | 12.03 | 23.85 | 26.32 | 17.42 | 48.08 | 15.74 | 51.07 | 24.53 | 84.38 | 16.82 | 17.54 | 10.34 | 31.15 |
| Roach | 1028 | 1 | 1 | 1 | 114.21 | 21.47 | 8.18 | 12.25 | 27.27 | 26.21 | 21.03 | 53.43 | 20.97 | 56.27 | 25.81 | 82.08 | 16.21 | 20.18 | 11.71 | 34.64 |
| Roach | 1029 | 1 | 1 | 1 | 105.22 | 20.36 | 7.11 | 11.12 | 24.25 | 25.3 | 17.32 | 51.22 | 18.44 | 55.49 | 25.69 | 75.99 | 16 | 15.51 | 10.56 | 32 |
| Roach | 1030 | 1 | 1 | 1 | 94.66 | 19.5 | 5.62 | 10.58 | 23.09 | 24.48 | 15.59 | 47.41 | 15.34 | 51.28 | 20.69 | 66.7 | 14.54 | 17.46 | 10.12 | 31.02 |
| Roach | 1001 | 1 | 1 | 2 | 89.87 | 17.15 | 5.27 | 10.06 | 21.51 | 22.54 | 14.92 | 40.69 | 15.34 | 44.03 | 21.95 | 62.24 | 13.85 | 19.94 | 9.43 | 28.3 |
| Roach | 1002 | 1 | 1 | 2 | 113.43 | 22.22 | 7.13 | 13.88 | 27.12 | 27.64 | 19.99 | 55.34 | 20.15 | 55.36 | 27.73 | 81.82 | 17.62 | 20.76 | 12.12 | 37.52 |
| Roach | 1003 | 1 | 1 | 2 | 131.58 | 25.38 | 7.93 | 14.31 | 29.32 | 30.15 | 23.15 | 60 | 22.9 | 61.15 | 30.75 | 91.74 | 20.05 | 21.53 | 14.06 | 43.62 |
| Roach | 1004 | 1 | 1 | 2 | 83.36 | 16.66 | 5.14 | 8.77 | 19.64 | 20.77 | 15.34 | 48.91 | 14.65 | 48.91 | 20.31 | 59.16 | 12.88 | 17.6 | 8.39 | 25.62 |
| Roach | 1005 | 1 | 1 | 2 | 116.01 | 22.4 | 7 | 14.56 | 28.24 | 29.22 | 19.31 | 56.48 | 19.67 | 56.6 | 25.95 | 81.61 | 17.41 | 19.67 | 12.11 | 39 |
| Roach | 1006 | 1 | 1 | 2 | 130.34 | 24.9 | 8 | 15.16 | 29.91 | 31.05 | 23.37 | 61.59 | 22.76 | 64.09 | 31.1 | 93.63 | 19.31 | 23.7 | 14.54 | 43.38 |
| Roach | 1007 | 1 | 1 | 2 | 89.47 | 17.59 | 5.44 | 9.89 | 21.56 | 23.12 | 17.83 | 42.13 | 15.11 | 44.39 | 19.54 | 61.7 | 13.31 | 15.37 | 9.18 | 28.87 |
| Roach | 1008 | 1 | 1 | 2 | 77.61 | 15.49 | 5.18 | 8.32 | 19.48 | 20.19 | 13.53 | 34.91 | 13.18 | 34.91 | 17.38 | 54.13 | 11.28 | 17.06 | 7.77 | 25.11 |
| Roach | 1009 | 1 | 1 | 2 | 89.56 | 18.06 | 4.82 | 9.64 | 21.79 | 22.94 | 16.12 | 43.06 | 15.55 | 43.14 | 21.92 | 63.95 | 14.38 | 16.37 | 9.92 | 28.32 |
| Roach | 1010 | 1 | 1 | 2 | 90.6 | 18.05 | 5.68 | 10.47 | 22.73 | 23.45 | 17.77 | 43.68 | 16.92 | 46.04 | 22.13 | 64.84 | 14.57 | 18.75 | 10.19 | 30.05 |
| Roach | 1011 | 1 | 1 | 2 | 81.01 | 15.96 | 4.5 | 9.25 | 19.53 | 20.36 | 14.78 | 37.78 | 13.41 | 38.99 | 19.4 | 55.91 | 12.13 | 16.63 | 8.28 | 26.1 |
| Roach | 1012 | 1 | 1 | 2 | 99.58 | 18.67 | 5.85 | 10.57 | 23.09 | 23.88 | 17.37 | 46.86 | 16.84 | 49.91 | 22.52 | 69.49 | 15.83 | 18.76 | 9.75 | 30.86 |
| Roach | 1013 | 1 | 1 | 2 | 91.59 | 18.21 | 5.35 | 9.42 | 20.96 | 22.42 | 16.42 | 41.84 | 15.3 | 45.68 | 21.61 | 62.73 | 14.31 | 18.55 | 9.21 | 28.2 |
| Roach | 1014 | 1 | 1 | 2 | 129.22 | 24.49 | 7.59 | 14.33 | 29.94 | 30.55 | 22.22 | 60.54 | 23.11 | 64.61 | 30.4 | 89.86 | 18.41 | 23.31 | 12.79 | 38.29 |
| Roach | 1015 | 1 | 1 | 2 | 127.42 | 26.8 | 8.1 | 14.2 | 29.28 | 30.41 | 23.69 | 59.55 | 22.43 | 60.74 | 27.37 | 91.02 | 19.01 | 22.72 | 12.76 | 39.83 |
| Roach | 1016 | 1 | 1 | 2 | 86.67 | 17.55 | 5.02 | 10.07 | 21.11 | 21.97 | 15.98 | 40.01 | 15.87 | 42.66 | 21.56 | 59.9 | 12.96 | 18.18 | 9.68 | 29.66 |
| Roach | 1016 | 1 | 1 | 2 | 70.05 | 13.61 | 4.25 | 7.65 | 17.41 | 18.01 | 12.78 | 32.51 | 13.56 | 32.51 | 18.67 | 49.03 | 12.55 | 15.83 | 7.17 | 21.75 |
| Roach | 1017 | 1 | 1 | 2 | 109.42 | 20.35 | 6.34 | 11.54 | 24.92 | 25.69 | 19.25 | 49.47 | 20.14 | 51.08 | 27.8 | 76.21 | 17.68 | 20.86 | 11.74 | 35.37 |
| Roach | 1019 | 1 | 1 | 2 | 91.84 | 17.4 | 5.64 | 10.74 | 22.06 | 24.27 | 17.03 | 45.19 | 15.8 | 48.17 | 21.44 | 64.41 | 14.5 | 16.53 | 9.97 | 29.1 |
| Roach | 1020 | 1 | 1 | 2 | 117.68 | 24.42 | 7.11 | 13.92 | 27.93 | 29.37 | 20.67 | 54.81 | 19.98 | 58.35 | 27.14 | 84 | 18.23 | 21.98 | 12.02 | 39.39 |
| Roach | 1021 | 1 | 1 | 2 | 110.87 | 21.98 | 6.29 | 12.11 | 25.46 | 26.13 | 19.27 | 52.51 | 19.51 | 56.64 | 25.72 | 77.61 | 18.21 | 19.71 | 11.36 | 34.14 |
| Roach | 1022 | 1 | 1 | 2 | 93.04 | 19 | 5.53 | 10.92 | 22.91 | 23.7 | 16.37 | 44.23 | 16.23 | 46.34 | 19.51 | 67.5 | 15.44 | 19.37 | 9.35 | 26.94 |
| Roach | 1023 | 1 | 1 | 2 | 79.9 | 15.12 | 4.38 | 7.89 | 18.68 | 19.94 | 12.57 | 37.04 | 12.09 | 38.87 | 17.79 | 56.67 | 12.8 | 17.41 | 8.38 | 23.34 |
| Roach | 1024 | 1 | 1 | 2 | 95.73 | 19.66 | 6.14 | 10.46 | 23.79 | 25.13 | 17.49 | 42.01 | 18.4 | 43.91 | 24.16 | 66.43 | 16.31 | 20.8 | 11.04 | 31.89 |
| Roach | 1025 | 1 | 1 | 2 | 94.36 | 18.32 | 5.37 | 11.28 | 22.52 | 24.36 | 17.68 | 44.88 | 17.94 | 45.8 | 23.71 | 65.5 | 14.71 | 17.34 | 9.99 | 31.25 |
| Roach | 1026 | 1 | 1 | 2 | 115.69 | 23.44 | 7.38 | 13.67 | 28.96 | 29.63 | 20.13 | 58.4 | 19.23 | 61.33 | 24.79 | 82.68 | 16.72 | 20.67 | 12.12 | 37.57 |
| Roach | 1027 | 1 | 1 | 2 | 99.73 | 19.36 | 5.76 | 11.94 | 23.54 | 24.43 | 17.76 | 46.24 | 16.47 | 49.21 | 23.79 | 72.69 | 15.45 | 21.62 | 10.16 | 31.67 |
| Roach | 1028 | 1 | 1 | 2 | 115.28 | 21.87 | 7.08 | 11.69 | 26 | 27.03 | 20.29 | 54.79 | 19.78 | 56.42 | 26.11 | 81.95 | 17.43 | 21.53 | 11.74 | 35.1 |
| Roach | 1029 | 1 | 1 | 2 | 106.66 | 20.68 | 6.63 | 10.94 | 24.9 | 26.15 | 17.85 | 52.18 | 18.43 | 52.65 | 25.04 | 73.74 | 17.06 | 19.59 | 10.89 | 33.13 |
| Roach | 1030 | 1 | 1 | 2 | 93.92 | 19.3 | 5.48 | 10.6 | 22.81 | 24.68 | 16.6 | 44.68 | 15.74 | 46.5 | 21.63 | 66.85 | 14.42 | 19.92 | 10.25 | 31.42 |
| Roach | 1001 | 1 | 1 | 3 | 90.97 | 17.33 | 5.14 | 9.99 | 21.31 | 22.93 | 15.58 | 40.7 | 15.44 | 43.49 | 22.03 | 62.51 | 14.4 | 17.5 | 9.06 | 28.7 |
| Roach | 1002 | 1 | 1 | 3 | 112.76 | 21.39 | 6.48 | 13.07 | 26.61 | 27.94 | 20.74 | 54.23 | 21.09 | 57.16 | 28.87 | 81.68 | 16.47 | 19.96 | 11.72 | 36.66 |
| Roach | 1003 | 1 | 1 | 3 | 131.42 | 24.78 | 7.87 | 14.62 | 29.72 | 31.09 | 22.71 | 60.52 | 23.29 | 64.09 | 29.95 | 89.96 | 19.86 | 20.36 | 13.64 | 43.43 |
| Roach | 1004 | 1 | 1 | 3 | 82.74 | 16.57 | 4.62 | 8.71 | 20.01 | 21.03 | 14.78 | 40.35 | 15.27 | 42.71 | 23.81 | 57.27 | 14.31 | 16.54 | 8.75 | 25.81 |
| Roach | 1005 | 1 | 1 | 3 | 116 | 22.88 | 6.98 | 14.57 | 28.71 | 30 | 19.21 | 56.88 | 19.15 | 58.33 | 26.72 | 81.41 | 17.5 | 20.8 | 11.9 | 38.26 |
| Roach | 1006 | 1 | 1 | 3 | 129.18 | 24.23 | 7.94 | 15.04 | 30.1 | 31.51 | 23.02 | 61.9 | 22.74 | 64.44 | 30.16 | 93.72 | 19.58 | 23.24 | 13.97 | 43.66 |
| Roach | 1007 | 1 | 1 | 3 | 88.98 | 17.22 | 5.84 | 9.35 | 20.93 | 21.93 | 15.78 | 42.54 | 14.66 | 44.06 | 19.89 | 62.23 | 13.92 | 17.93 | 9.69 | 28.24 |
| Roach | 1008 | 1 | 1 | 3 | 78.01 | 15.66 | 5.24 | 8.32 | 19.23 | 20.37 | 13.21 | 35.35 | 14.27 | 37.01 | 18.01 | 54.74 | 12.16 | 17.91 | 8.15 | 25.27 |
| Roach | 1009 | 1 | 1 | 3 | 87.8 | 17.91 | 5.42 | 9.51 | 21.86 | 22.64 | 15.97 | 42.86 | 15.29 | 45.32 | 21.19 | 63.42 | 14.55 | 16.45 | 9.27 | 27.57 |
| Roach | 1010 | 1 | 1 | 3 | 92.55 | 18.76 | 5.88 | 9.89 | 22.46 | 23.06 | 16.21 | 43.57 | 16.2 | 47.36 | 21.81 | 63.78 | 14.19 | 18.96 | 10.23 | 29.9 |
| Roach | 1011 | 1 | 1 | 3 | 80.74 | 15.96 | 4.34 | 9.11 | 19.78 | 20.44 | 14.91 | 37.53 | 13.95 | 38.43 | 19.64 | 56.15 | 12.15 | 16.77 | 8.07 | 26.07 |
| Roach | 1012 | 1 | 1 | 3 | 98.77 | 19 | 6.37 | 10.98 | 22.73 | 24 | 15.76 | 46.52 | 17.85 | 48.5 | 23.14 | 69.65 | 15.58 | 20.1 | 9.98 | 31.38 |
| Roach | 1013 | 1 | 1 | 3 | 90.92 | 17.24 | 5.55 | 9.74 | 21.66 | 22.49 | 16.88 | 41.77 | 16.79 | 43.9 | 21.33 | 62.73 | 14.83 | 19.31 | 9.07 | 28.02 |
| Roach | 1014 | 1 | 1 | 3 | 127.88 | 23.49 | 8.18 | 14.78 | 29.46 | 30.63 | 22.75 | 59.28 | 22.2 | 63.95 | 30.3 | 88.99 | 17.65 | 25.22 | 13.2 | 39.31 |
| Roach | 1015 | 1 | 1 | 3 | 127.25 | 25.19 | 7.78 | 14 | 28.87 | 30.81 | 23.15 | 60.19 | 22.28 | 64.1 | 25.9 | 89.6 | 19.52 | 22.75 | 12.88 | 40.57 |
| Roach | 1016 | 1 | 1 | 3 | 86.82 | 18.15 | 6.07 | 9.65 | 20.8 | 21.64 | 15.78 | 40.08 | 15.91 | 43.76 | 20.85 | 61.26 | 13.23 | 18.93 | 10.1 | 29.77 |
| Roach | 1016 | 1 | 1 | 3 | 69.28 | 13.28 | 4.08 | 7.86 | 17.99 | 18.47 | 12.09 | 32.79 | 12.61 | 32.95 | 17.74 | 49.04 | 12.16 | 16.19 | 6.61 | 21.49 |
| Roach | 1017 | 1 | 1 | 3 | 109.35 | 21.94 | 6.6 | 11.74 | 24.43 | 26.03 | 20.71 | 50.27 | 21.14 | 52.66 | 26.18 | 75.36 | 17.5 | 19.97 | 11.09 | 34.45 |
| Roach | 1019 | 1 | 1 | 3 | 91.16 | 17.12 | 5.58 | 10.44 | 22.83 | 23.74 | 16.22 | 44.93 | 15.44 | 47.04 | 22.68 | 64.84 | 14.88 | 16.7 | 9.07 | 28.91 |
| Roach | 1020 | 1 | 1 | 3 | 116.72 | 22.84 | 7.32 | 13.33 | 27.4 | 28.85 | 20.06 | 56.08 | 20.38 | 60.96 | 26.29 | 83.19 | 18.57 | 19.12 | 11.26 | 38.14 |
| Roach | 1021 | 1 | 1 | 3 | 110.42 | 21.2 | 6.05 | 12.07 | 25.56 | 26.96 | 20.2 | 51.62 | 19.37 | 55.01 | 27.17 | 76.49 | 17.43 | 19.04 | 11.44 | 34.5 |
| Roach | 1022 | 1 | 1 | 3 | 91.77 | 17.99 | 5.54 | 10.1 | 21.76 | 22.58 | 12.98 | 45.17 | 16.32 | 46.76 | 20.53 | 67.49 | 13.82 | 16.81 | 9.04 | 26.92 |
| Roach | 1023 | 1 | 1 | 3 | 81.22 | 15.07 | 4.53 | 6.81 | 18.9 | 19.57 | 13.49 | 37.83 | 12.63 | 40.78 | 20.68 | 56.83 | 13.25 | 16.54 | 8.03 | 23.12 |
| Roach | 1024 | 1 | 1 | 3 | 96.95 | 19.75 | 5.91 | 11.23 | 23.44 | 24.6 | 18.66 | 43.8 | 18.72 | 44.97 | 24.78 | 65.29 | 16.55 | 19.61 | 10.45 | 32.15 |
| Roach | 1025 | 1 | 1 | 3 | 94.25 | 18.8 | 6.31 | 10.7 | 22.4 | 24.51 | 16.96 | 45.11 | 16.27 | 46.45 | 22.03 | 66.43 | 14.69 | 19.02 | 10.29 | 31.87 |
| Roach | 1026 | 1 | 1 | 3 | 116.64 | 23.42 | 7.74 | 14.6 | 28.78 | 29.76 | 19.76 | 57.79 | 19.4 | 61.2 | 25.53 | 82.1 | 17.15 | 20.1 | 12.11 | 37.1 |
| Roach | 1027 | 1 | 1 | 3 | 100.35 | 19.02 | 6.05 | 11.59 | 24.28 | 25.31 | 17.37 | 45.41 | 15.45 | 50.69 | 23.49 | 73 | 16.33 | 21.45 | 10.5 | 31.23 |
| Roach | 1028 | 1 | 1 | 3 | 115.51 | 22.07 | 6.17 | 12.83 | 26.41 | 27.3 | 20.4 | 52.84 | 19.75 | 56.24 | 25.68 | 79.29 | 16.91 | 21.18 | 11.57 | 34.87 |
| Roach | 1029 | 1 | 1 | 3 | 106.14 | 20.81 | 6.76 | 10.49 | 24.83 | 26.25 | 17.49 | 51.42 | 19.04 | 53.66 | 26.19 | 74.95 | 17.04 | 17.38 | 10.34 | 33.74 |
| Roach | 1030 | 1 | 1 | 3 | 94.27 | 19.46 | 6.02 | 10.57 | 22.54 | 24.47 | 16.82 | 45.55 | 15.75 | 46.38 | 22.38 | 66.59 | 13.86 | 18.4 | 10.3 | 31.91 |
| Roach | 1101 | 2 | 1 | 1 | 128.71 | 23.09 | 8.23 | 15.09 | 29.68 | 30.5 | 21.83 | 61.33 | 23.63 | 64.71 | 29.78 | 89.69 | 20.42 | 20.51 | 14.02 | 39.8 |
| Roach | 1102 | 2 | 1 | 1 | 109.89 | 19.58 | 6.98 | 12.97 | 26.46 | 25.24 | 18.99 | 50.2 | 19.7 | 53.56 | 25.34 | 78.13 | 16.22 | 21.41 | 12.03 | 33.32 |
| Roach | 1103 | 2 | 1 | 1 | 114.09 | 23.56 | 7.58 | 13.94 | 28.05 | 29.61 | 19.1 | 57.01 | 20.12 | 60.62 | 24.55 | 84.46 | 17.62 | 20.02 | 12.27 | 34.92 |
| Roach | 1104 | 2 | 1 | 1 | 115.55 | 22.39 | 6.66 | 13.75 | 27.45 | 26.76 | 18.66 | 54.21 | 17.74 | 56.86 | 24.8 | 83.43 | 16.95 | 16.37 | 12.27 | 37.21 |
| Roach | 1105 | 2 | 1 | 1 | 109.41 | 22.03 | 7.19 | 11.74 | 26.67 | 25.59 | 19.46 | 54.1 | 17.74 | 55.16 | 23.9 | 78.32 | 14.72 | 19.18 | 11.84 | 33.75 |
| Roach | 1106 | 2 | 1 | 1 | 83.3 | 17.81 | 6.04 | 9.53 | 20.92 | 21.72 | 16 | 39.36 | 14.4 | 41.49 | 19.73 | 59.67 | 13.13 | 16.33 | 8.67 | 26.03 |
| Roach | 1107 | 2 | 1 | 1 | 104.51 | 19.61 | 7.03 | 11.36 | 26.12 | 25.55 | 17.17 | 47.53 | 17.78 | 51.77 | 25.11 | 76.04 | 15.99 | 18.34 | 10.62 | 31.88 |
| Roach | 1108 | 2 | 1 | 1 | 104.26 | 19.18 | 6.37 | 10.36 | 23.63 | 25.25 | 18.38 | 50 | 17.24 | 52.41 | 23.02 | 76.35 | 15.51 | 14.48 | 10.09 | 31.74 |
| Roach | 1109 | 2 | 1 | 1 | 104.17 | 19.75 | 7.04 | 13.12 | 25.97 | 25.92 | 19.03 | 49.82 | 19.56 | 52.46 | 26.52 | 72.95 | 18.72 | 19.82 | 11.2 | 34.44 |
| Roach | 1110 | 2 | 1 | 1 | 87.14 | 16.44 | 6.1 | 9.44 | 20.53 | 21.4 | 13.91 | 39.66 | 14.72 | 42.15 | 18.47 | 61.53 | 12.54 | 16.25 | 9.02 | 25.9 |
| Roach | 1111 | 2 | 1 | 1 | 94.2 | 17.22 | 6.32 | 10.81 | 20.4 | 21.94 | 15.63 | 43.73 | 15.4 | 47.02 | 20.8 | 65.8 | 14.51 | 20.17 | 10.3 | 29.92 |
| Roach | 1112 | 2 | 1 | 1 | 85.21 | 15.57 | 5.72 | 9.38 | 20.49 | 21.05 | 14.47 | 42.03 | 14.18 | 41.57 | 17.65 | 64.35 | 12.56 | 16.23 | 9.16 | 23.46 |
| Roach | 1113 | 2 | 1 | 1 | 102.85 | 20.24 | 7.1 | 11.36 | 25.38 | 24.89 | 16.56 | 48.6 | 18.33 | 52.43 | 22.57 | 70.39 | 14.22 | 16.02 | 10.98 | 32.1 |
| Roach | 1114 | 2 | 1 | 1 | 79.61 | 14.92 | 4.75 | 9.66 | 19.1 | 20.18 | 13.54 | 37.75 | 13.82 | 41.04 | 19.16 | 56.52 | 11.51 | 15.78 | 8.52 | 25.58 |
| Roach | 1115 | 2 | 1 | 1 | 95.26 | 18.59 | 6.12 | 11.52 | 23.01 | 23.63 | 15.46 | 44.12 | 15.32 | 45.1 | 23.29 | 65.62 | 13.26 | 17.49 | 9.45 | 27.66 |
| Roach | 1116 | 2 | 1 | 1 | 76.06 | 15.42 | 4.72 | 9.06 | 17.83 | 19.71 | 12.95 | 34.19 | 13.48 | 38.25 | 17.02 | 53.18 | 12.01 | 17.39 | 7.29 | 22.57 |
| Roach | 1117 | 2 | 1 | 1 | 85.63 | 17.4 | 5.91 | 9.87 | 20.26 | 22.55 | 15.66 | 40.59 | 14.83 | 43.76 | 20.71 | 62.47 | 12.76 | 16.16 | 9.99 | 27.35 |
| Roach | 1118 | 2 | 1 | 1 | 86.36 | 17.18 | 5.18 | 11.19 | 22.11 | 22.73 | 12.58 | 41.41 | 13.28 | 44.27 | 19.6 | 60.09 | 13.16 | 14.31 | 8.3 | 24.64 |
| Roach | 1119 | 2 | 1 | 1 | 87.15 | 17.44 | 6.24 | 10 | 20.79 | 22.07 | 14.27 | 44.33 | 14.77 | 42.89 | 20.11 | 62.91 | 13.52 | 16.74 | 9 | 27.03 |
| Roach | 1120 | 2 | 1 | 1 | 73.16 | 12.99 | 5.46 | 8.96 | 18.48 | 17.81 | 11.92 | 33.76 | 11.06 | 35.67 | 18.09 | 49.48 | 11.07 | 13.37 | 6.92 | 20.23 |
| Roach | 1121 | 2 | 1 | 1 | 82.95 | 15.37 | 5.37 | 9.87 | 18.98 | 20.27 | 12.98 | 41.79 | 13.74 | 41.68 | 17.8 | 58.64 | 11.76 | 16.09 | 8.08 | 23.16 |
| Roach | 1122 | 2 | 1 | 1 | 79.41 | 14.29 | 5.22 | 8.99 | 18.79 | 20.6 | 14.15 | 36.95 | 15.51 | 39.27 | 20.44 | 55.61 | 11.84 | 16.33 | 7.22 | 24.14 |
| Roach | 1123 | 2 | 1 | 1 | 77.15 | 14.53 | 5.61 | 9.53 | 19.61 | 21.69 | 11.9 | 38.57 | 11.12 | 39.98 | 18.05 | 55.99 | 11.24 | 15.02 | 8.51 | 23.02 |
| Roach | 1124 | 2 | 1 | 1 | 81.33 | 16.45 | 6.5 | 10.64 | 20.8 | 20.69 | 12.83 | 38.69 | 12.24 | 39.88 | 18.46 | 55.3 | 10.92 | 13.77 | 7.97 | 23.96 |
| Roach | 1125 | 2 | 1 | 1 | 70.15 | 13.78 | 5.25 | 8.1 | 17.86 | 18.84 | 11.23 | 33.64 | 11.55 | 34.4 | 15.91 | 50.67 | 10.95 | 14.67 | 7.44 | 20.72 |
| Roach | 1126 | 2 | 1 | 1 | 64.47 | 12.73 | 4.79 | 7.75 | 16.6 | 18.15 | 10.41 | 31.54 | 9.77 | 33.64 | 14.85 | 44.74 | 9.44 | 14.05 | 6.04 | 17.11 |
| Roach | 1127 | 2 | 1 | 1 | 100.44 | 19.31 | 7.2 | 12.49 | 24.08 | 25.58 | 17.27 | 49.17 | 17.48 | 49.7 | 23.26 | 72.93 | 14.75 | 18.43 | 10.74 | 30.66 |
| Roach | 1128 | 2 | 1 | 1 | 80.12 | 15.15 | 5.11 | 9.35 | 19.9 | 20.87 | 14.4 | 38.67 | 12.58 | 41.86 | 17.75 | 57.43 | 11.71 | 13.85 | 8.04 | 24.62 |
| Roach | 1129 | 2 | 1 | 1 | 76.12 | 15.38 | 6.05 | 9.23 | 19.74 | 20.38 | 12.67 | 38.22 | 14.16 | 39.92 | 17.68 | 54.89 | 10.73 | 12.55 | 7.91 | 22.18 |
| Roach | 1130 | 2 | 1 | 1 | 70.02 | 13.09 | 5.66 | 8.13 | 17.03 | 19 | 11.13 | 35.3 | 12.53 | 34.05 | 16.11 | 49.68 | 10.9 | 16.02 | 6.66 | 19.81 |
| Roach | 1101 | 2 | 1 | 2 | 127.36 | 24.02 | 7.71 | 14.16 | 29.49 | 30.89 | 22.9 | 61.52 | 23.06 | 63.98 | 30.28 | 90.07 | 19.26 | 19.9 | 13.32 | 40.71 |
| Roach | 1102 | 2 | 1 | 2 | 109.98 | 18.98 | 6.73 | 11.51 | 24.85 | 25.52 | 19.49 | 55.65 | 19.39 | 53.4 | 25.56 | 79.56 | 16.51 | 18.04 | 11.38 | 32.68 |
| Roach | 1103 | 2 | 1 | 2 | 114.12 | 22.68 | 7.99 | 13.91 | 27.91 | 29.72 | 19.1 | 53.78 | 20.66 | 56.54 | 26.16 | 84.34 | 17.72 | 16.89 | 11.36 | 34.51 |
| Roach | 1104 | 2 | 1 | 2 | 113.94 | 21.76 | 6.78 | 13.03 | 26.6 | 27.91 | 19.27 | 52.09 | 18.75 | 56.38 | 23.91 | 83.31 | 16.4 | 15.47 | 11.07 | 36.38 |
| Roach | 1105 | 2 | 1 | 2 | 109.62 | 21.57 | 7.08 | 11.57 | 26.59 | 27.06 | 20.55 | 50.22 | 20.05 | 53.72 | 24.73 | 76.98 | 16.96 | 17.54 | 11.35 | 34.09 |
| Roach | 1106 | 2 | 1 | 2 | 82.05 | 16.1 | 5.23 | 9.41 | 20.23 | 21.12 | 16.18 | 39.07 | 15.49 | 40.76 | 21.1 | 58.1 | 14.63 | 15.6 | 8.48 | 25.42 |
| Roach | 1107 | 2 | 1 | 2 | 104.04 | 19.8 | 6.53 | 11.29 | 24.46 | 25.78 | 16.84 | 48.18 | 19.1 | 50.43 | 24.15 | 74.48 | 16.83 | 15.85 | 10.26 | 30.48 |
| Roach | 1108 | 2 | 1 | 2 | 100.41 | 19.23 | 6.52 | 10.28 | 23.05 | 24.26 | 18.14 | 47.81 | 18.01 | 49.71 | 25.49 | 74.06 | 16.39 | 14.43 | 10.57 | 31.57 |
| Roach | 1109 | 2 | 1 | 2 | 104.17 | 20.85 | 7.06 | 11.69 | 25.37 | 26.38 | 19.49 | 48.35 | 19.81 | 50.56 | 26.57 | 73.46 | 16.66 | 14.95 | 11.09 | 34.37 |
| Roach | 1110 | 2 | 1 | 2 | 86.3 | 16.42 | 4.99 | 7.79 | 20.27 | 21.32 | 14.3 | 39.92 | 13.43 | 41.67 | 19.49 | 61.48 | 12.74 | 19.17 | 8.38 | 25.7 |
| Roach | 1111 | 2 | 1 | 2 | 96.02 | 17.98 | 5.86 | 9.44 | 21.17 | 22.04 | 16.43 | 44.05 | 16.62 | 45.21 | 18.14 | 66.47 | 14.54 | 18.68 | 9.59 | 29.14 |
| Roach | 1112 | 2 | 1 | 2 | 85.5 | 15.06 | 4.99 | 9.06 | 21.23 | 21.43 | 13.38 | 40.18 | 14.3 | 42.12 | 18.78 | 60.49 | 11.45 | 15.46 | 8.44 | 23.74 |
| Roach | 1113 | 2 | 1 | 2 | 103.26 | 20.54 | 7.07 | 11.2 | 24.06 | 24.54 | 18.91 | 48.65 | 18.16 | 50.8 | 23.75 | 73.91 | 14.99 | 15.51 | 10.21 | 32.5 |
| Roach | 1114 | 2 | 1 | 2 | 79.9 | 15.14 | 4.46 | 8.67 | 19.36 | 20.33 | 14.52 | 37.11 | 14.31 | 37.5 | 18.8 | 57.21 | 12.12 | 14.34 | 8.62 | 25.48 |
| Roach | 1115 | 2 | 1 | 2 | 91.43 | 17.56 | 6.57 | 10.32 | 22.84 | 23.98 | 14.14 | 42.56 | 16.09 | 44.92 | 21.83 | 63.81 | 14.69 | 15.96 | 9.69 | 29.67 |
| Roach | 1116 | 2 | 1 | 2 | 73.35 | 14.76 | 4.53 | 8.04 | 18.52 | 19.34 | 13.06 | 35.06 | 14.47 | 36.22 | 17.25 | 50.28 | 12.49 | 14.77 | 7.58 | 21.76 |
| Roach | 1117 | 2 | 1 | 2 | 86.14 | 16.69 | 5.99 | 8.96 | 20.04 | 21.91 | 14.89 | 41.18 | 15.72 | 43.43 | 20.93 | 61.95 | 12.45 | 16.67 | 9.41 | 27.81 |
| Roach | 1118 | 2 | 1 | 2 | 84.85 | 16.46 | 5.56 | 9.55 | 21.97 | 23.24 | 14.55 | 39.46 | 14.85 | 40.97 | 19.77 | 61.49 | 12.71 | 14.23 | 8.15 | 25.48 |
| Roach | 1119 | 2 | 1 | 2 | 87.16 | 17.56 | 5.85 | 9.27 | 22.35 | 22.36 | 14.89 | 41.52 | 13.39 | 42.66 | 15.23 | 63.25 | 13.09 | 17.55 | 9.16 | 26.27 |
| Roach | 1120 | 2 | 1 | 2 | 72.55 | 12.52 | 4.26 | 7.74 | 16.97 | 18.3 | 12.23 | 33.59 | 11.66 | 33.07 | 17 | 50.66 | 11.07 | 15.94 | 6.77 | 20.04 |
| Roach | 1121 | 2 | 1 | 2 | 81.56 | 14.88 | 5.16 | 8.83 | 19.98 | 21.03 | 14.6 | 39.87 | 13.07 | 40.8 | 18.55 | 58.2 | 12.93 | 14.99 | 7.93 | 22.89 |
| Roach | 1122 | 2 | 1 | 2 | 78.3 | 14.61 | 5.29 | 8.53 | 18.45 | 20.18 | 13.94 | 36.36 | 15.38 | 37.23 | 20.92 | 54.83 | 13.29 | 16.31 | 7.91 | 22.81 |
| Roach | 1123 | 2 | 1 | 2 | 76.39 | 14.28 | 5.22 | 8.58 | 18.61 | 19.7 | 13.39 | 36.63 | 12.85 | 38.65 | 19.37 | 54.24 | 12.09 | 15.44 | 7.97 | 22.54 |
| Roach | 1124 | 2 | 1 | 2 | 80.43 | 15.47 | 5.05 | 9.38 | 19.99 | 21.43 | 13.5 | 38.14 | 14.07 | 40.17 | 18.37 | 57.6 | 11.64 | 14.53 | 7.73 | 23.74 |
| Roach | 1125 | 2 | 1 | 2 | 69.33 | 13.56 | 4.03 | 7.1 | 17.63 | 18.46 | 10.45 | 32.48 | 10.78 | 33.31 | 16.93 | 47.74 | 11.46 | 15.63 | 7.3 | 20.19 |
| Roach | 1126 | 2 | 1 | 2 | 64.79 | 12.04 | 4.18 | 6.89 | 16.4 | 17.35 | 10.78 | 30.01 | 9.33 | 31.09 | 15.11 | 46.96 | 10.58 | 14.39 | 6.37 | 17.85 |
| Roach | 1127 | 2 | 1 | 2 | 100 | 19.66 | 6.91 | 10.75 | 24.45 | 25.91 | 17.55 | 46.93 | 18.38 | 49.52 | 24.18 | 69.82 | 13.82 | 16.96 | 10.33 | 31.06 |
| Roach | 1128 | 2 | 1 | 2 | 78.8 | 15.34 | 4.9 | 8.96 | 19.78 | 20.48 | 14.55 | 37.48 | 13.97 | 37.66 | 19.62 | 57.44 | 12.11 | 15.23 | 8.56 | 24 |
| Roach | 1129 | 2 | 1 | 2 | 74.44 | 14.94 | 5.34 | 8.92 | 19.94 | 20.95 | 14.15 | 36.09 | 14.41 | 36.93 | 18.2 | 53.94 | 12.53 | 15.19 | 7.34 | 22.64 |
| Roach | 1130 | 2 | 1 | 2 | 67.04 | 12.49 | 4.69 | 6.79 | 17.12 | 17.85 | 11.32 | 32.09 | 11.89 | 33.69 | 16.04 | 47.86 | 10.2 | 13.06 | 6.77 | 18.79 |
| Roach | 1101 | 2 | 1 | 3 | 128.1 | 24.68 | 7.51 | 14.52 | 29.17 | 30.74 | 22.01 | 59.59 | 23.6 | 61.99 | 30.53 | 90.37 | 19.47 | 20.5 | 13.44 | 40.01 |
| Roach | 1102 | 2 | 1 | 3 | 110.66 | 20.12 | 6.71 | 11.5 | 25.4 | 26.35 | 17.38 | 52.55 | 19.72 | 54.24 | 24.66 | 77.43 | 16.44 | 20.11 | 11.29 | 31.81 |
| Roach | 1103 | 2 | 1 | 3 | 114.62 | 22.19 | 7.74 | 13.6 | 28.23 | 29.01 | 18.75 | 55.94 | 19.78 | 58.04 | 25.74 | 83.83 | 16.72 | 18.7 | 11.46 | 35.11 |
| Roach | 1104 | 2 | 1 | 3 | 114.93 | 22.97 | 6.96 | 12.95 | 27.11 | 27.6 | 18.58 | 53.17 | 18.56 | 57 | 23.98 | 81.69 | 16.61 | 20.28 | 11.14 | 36.98 |
| Roach | 1105 | 2 | 1 | 3 | 109.76 | 22.14 | 7.16 | 12.17 | 25.78 | 26.3 | 20.57 | 51.95 | 19.72 | 53.7 | 25.69 | 78.1 | 16 | 18.32 | 11.34 | 24.61 |
| Roach | 1106 | 2 | 1 | 3 | 82.53 | 15.96 | 4.92 | 9.17 | 19.94 | 20.46 | 15.83 | 38.91 | 15.12 | 40.55 | 20.26 | 57.72 | 14.09 | 16.89 | 8.53 | 26.05 |
| Roach | 1107 | 2 | 1 | 3 | 103.63 | 20.69 | 6.46 | 10.99 | 24.43 | 25.11 | 18.6 | 48.93 | 19.12 | 50.66 | 25.65 | 75.88 | 17.58 | 15.9 | 10.03 | 31.37 |
| Roach | 1108 | 2 | 1 | 3 | 101.67 | 19.55 | 6.39 | 10.5 | 22.62 | 23.77 | 18.99 | 48.58 | 17.65 | 51.27 | 25.36 | 73.51 | 16.58 | 16.25 | 10.05 | 31.18 |
| Roach | 1109 | 2 | 1 | 3 | 104.2 | 20.83 | 7.13 | 11.94 | 26.39 | 27.12 | 19.49 | 48.82 | 20 | 50.6 | 26.9 | 73.19 | 17.96 | 18.51 | 11.23 | 33.97 |
| Roach | 1110 | 2 | 1 | 3 | 86.63 | 16.16 | 5.39 | 8 | 19.57 | 20.64 | 14.33 | 40 | 14.71 | 41.02 | 19.46 | 62.25 | 13.72 | 18.04 | 8.53 | 26.12 |
| Roach | 1111 | 2 | 1 | 3 | 94.93 | 17.67 | 5.39 | 10 | 21.6 | 22.09 | 15.94 | 42.82 | 16.7 | 44.18 | 22.85 | 66.68 | 14.31 | 16.21 | 9.9 | 29 |
| Roach | 1112 | 2 | 1 | 3 | 85.4 | 16.2 | 5.12 | 9.24 | 20.98 | 21.67 | 14.17 | 40.68 | 14.86 | 41.71 | 18.89 | 60.06 | 12.33 | 16.03 | 8.33 | 24.1 |
| Roach | 1113 | 2 | 1 | 3 | 103.47 | 20.64 | 6.81 | 11.49 | 24.58 | 25.4 | 18.36 | 47.97 | 17.95 | 51.31 | 23.33 | 73.03 | 15.06 | 16.85 | 10.28 | 21.07 |
| Roach | 1114 | 2 | 1 | 3 | 79.65 | 15.76 | 4.62 | 8.7 | 19.54 | 20.61 | 13.54 | 37.95 | 13.99 | 40 | 18.93 | 54.2 | 12.02 | 16.21 | 8.5 | 25.12 |
| Roach | 1115 | 2 | 1 | 3 | 91.12 | 17.98 | 5.8 | 10.2 | 22.15 | 23.17 | 15.12 | 42.44 | 15.57 | 44.53 | 23.73 | 63.68 | 14.66 | 17.87 | 9.87 | 29.44 |
| Roach | 1116 | 2 | 1 | 3 | 74.3 | 14.18 | 4.76 | 7.8 | 18.66 | 19.29 | 13.04 | 34.95 | 14.06 | 34.79 | 18.31 | 51.6 | 13.5 | 16.32 | 7.41 | 21.8 |
| Roach | 1117 | 2 | 1 | 3 | 85.15 | 16.61 | 5.8 | 8.76 | 20.85 | 22.61 | 15.09 | 40.38 | 15.12 | 41.51 | 20.67 | 61.09 | 13.33 | 15.69 | 9.56 | 27.22 |
| Roach | 1118 | 2 | 1 | 3 | 86.21 | 16.75 | 5.3 | 9.4 | 21.12 | 22.26 | 14.41 | 39.22 | 14.16 | 41.12 | 18.3 | 59.91 | 13.39 | 15.33 | 8.31 | 24.51 |
| Roach | 1119 | 2 | 1 | 3 | 86.96 | 16.6 | 5.4 | 8.35 | 20.58 | 21.5 | 14.62 | 42.85 | 14.98 | 44.19 | 19.32 | 61.72 | 13.51 | 18.28 | 8.78 | 26.67 |
| Roach | 1120 | 2 | 1 | 3 | 72.64 | 13.5 | 4.71 | 7.33 | 18.3 | 18.04 | 11.03 | 32.67 | 12.43 | 33.54 | 16.07 | 51.85 | 10.8 | 12.76 | 7.17 | 19.4 |
| Roach | 1121 | 2 | 1 | 3 | 82.88 | 15.15 | 5.16 | 8.85 | 20.18 | 21.58 | 13.89 | 38.84 | 13.76 | 41.34 | 18.8 | 57.76 | 13 | 14.58 | 7.84 | 22.95 |
| Roach | 1122 | 2 | 1 | 3 | 79.05 | 14.44 | 4.75 | 9.06 | 19.9 | 20.77 | 14.65 | 37.06 | 14.62 | 38.59 | 19.6 | 54.39 | 13.42 | 14.65 | 7.59 | 22.76 |
| Roach | 1123 | 2 | 1 | 3 | 77.07 | 14.82 | 4.87 | 8.07 | 19.35 | 20.48 | 13.06 | 36.93 | 13.09 | 38.69 | 17.13 | 53.88 | 12.22 | 14.06 | 8 | 22.5 |
| Roach | 1124 | 2 | 1 | 3 | 80.33 | 15.34 | 5.28 | 8.82 | 19.66 | 21.52 | 13.14 | 36.57 | 13.33 | 40.07 | 18.32 | 57.56 | 12.42 | 13.61 | 7.51 | 24.3 |
| Roach | 1125 | 2 | 1 | 3 | 70.3 | 13.82 | 4.55 | 8.04 | 17.38 | 18.14 | 10.83 | 32.6 | 11.55 | 34.71 | 17.52 | 49.92 | 10.6 | 13.13 | 6.94 | 19.77 |
| Roach | 1126 | 2 | 1 | 3 | 65.01 | 12.44 | 4.68 | 6.72 | 16.6 | 17.41 | 10.57 | 30.83 | 12.02 | 32.02 | 15.69 | 45.95 | 10.53 | 14.35 | 6.34 | 17.83 |
| Roach | 1127 | 2 | 1 | 3 | 99.62 | 19.92 | 6.7 | 10.69 | 24.01 | 25.32 | 16.96 | 46.03 | 17.33 | 48.45 | 23.34 | 70.23 | 15.54 | 18.95 | 10.25 | 30.62 |
| Roach | 1128 | 2 | 1 | 3 | 79.3 | 14.83 | 5.15 | 9.51 | 19.64 | 20.5 | 14.33 | 39.34 | 11.79 | 39.26 | 17.71 | 57.18 | 12.48 | 13.8 | 7.95 | 24.47 |
| Roach | 1129 | 2 | 1 | 3 | 75.43 | 15.17 | 4.99 | 8.54 | 19.09 | 20.08 | 13.66 | 36.5 | 14.52 | 37.29 | 19 | 54.57 | 11.19 | 14.15 | 7.39 | 22.67 |
| Roach | 1130 | 2 | 1 | 3 | 68.9 | 13.46 | 4.65 | 6.54 | 16.93 | 17.86 | 11.36 | 33.01 | 11.49 | 33.98 | 16.64 | 49.17 | 9.98 | 13.52 | 6.44 | 18.57 |
| Roach | 1191 | 3 | 1 | 1 | 116.9 | 21.86 | 7.06 | 13.53 | 29.13 | 28.59 | 22.4 | 59.36 | 21.13 | 58.1 | 27.1 | 84.38 | 17.69 | 17.12 | 11.61 | 34.48 |
| Roach | 1192 | 3 | 1 | 1 | 109 | 20.04 | 6.28 | 12.97 | 25.25 | 25.81 | 18.64 | 51.55 | 17.52 | 56.99 | 23.12 | 79.34 | 16.35 | 17.7 | 10.69 | 35.64 |
| Roach | 1193 | 3 | 1 | 1 | 99.21 | 18.82 | 6.73 | 10.6 | 21.43 | 23.65 | 17.27 | 50.12 | 14.29 | 51.31 | 24.84 | 73.05 | 14.58 | 17.78 | 11.42 | 34.1 |
| Roach | 1194 | 3 | 1 | 1 | 88.33 | 18.16 | 6.43 | 8.44 | 20.92 | 22.77 | 14.22 | 43.95 | 14.17 | 45.8 | 18.66 | 63.69 | 13.69 | 17.99 | 9.27 | 27.69 |
| Roach | 1195 | 3 | 1 | 1 | 105.16 | 19.65 | 7.05 | 12.46 | 23.28 | 24.35 | 15.82 | 51.55 | 16.53 | 52.77 | 21.71 | 76.66 | 15.84 | 18.58 | 10.77 | 32.97 |
| Roach | 1196 | 3 | 1 | 1 | 110.56 | 20.68 | 7.19 | 15.62 | 27.4 | 26.48 | 20.34 | 54.06 | 17.98 | 60.4 | 24.97 | 68.04 | 17.62 | 18.81 | 10.94 | 34.39 |
| Roach | 1197 | 3 | 1 | 1 | 126.99 | 26.03 | 8.12 | 15.29 | 31.78 | 33.07 | 22.57 | 60.71 | 20.49 | 64.43 | 28.27 | 92.42 | 19.16 | 19.77 | 12.95 | 44.09 |
| Roach | 1198 | 3 | 1 | 1 | 92.38 | 18.24 | 5.72 | 10.49 | 21.97 | 22.93 | 15.33 | 45.26 | 15.72 | 47.72 | 19.99 | 68.08 | 14.64 | 16.56 | 9.18 | 28.66 |
| Roach | 1199 | 3 | 1 | 1 | 100.38 | 17.49 | 6.01 | 11.23 | 22.66 | 23.78 | 15.45 | 45.36 | 18.11 | 49.34 | 23.41 | 69.1 | 14.4 | 18.54 | 9.74 | 29.69 |
| Roach | 1200 | 3 | 1 | 1 | 109.7 | 21.07 | 6.88 | 13.17 | 25.11 | 27.88 | 19.36 | 52.8 | 17.37 | 55.15 | 23.75 | 77.9 | 17.08 | 21.89 | 10.46 | 33.25 |
| Roach | 1201 | 3 | 1 | 1 | 113.92 | 21.9 | 6.42 | 12.41 | 27.03 | 27.36 | 19.51 | 55.5 | 20.62 | 57.26 | 26.88 | 82.07 | 19.13 | 20.56 | 12.25 | 37.85 |
| Roach | 1202 | 3 | 1 | 1 | 92.32 | 19.13 | 5.84 | 10.74 | 22.1 | 23.44 | 15.77 | 45.43 | 15.89 | 48.31 | 24.06 | 64.63 | 14 | 16.32 | 10 | 29.38 |
| Roach | 1203 | 3 | 1 | 1 | 94.32 | 17.99 | 5.59 | 8.01 | 22.09 | 22.54 | 14.9 | 42.86 | 17.07 | 48.18 | 23.72 | 65.43 | 12.61 | 15.28 | 8.18 | 29.74 |
| Roach | 1204 | 3 | 1 | 1 | 98.77 | 18.14 | 5.52 | 11.87 | 23.75 | 24.56 | 15.71 | 49.04 | 17.15 | 53.06 | 20.72 | 69.4 | 14.57 | 16.24 | 8.58 | 31 |
| Roach | 1205 | 3 | 1 | 1 | 99.47 | 15.67 | 5.72 | 10.69 | 23.62 | 24.87 | 16.32 | 48.04 | 14.55 | 49.1 | 21.48 | 72.93 | 14.88 | 15.76 | 9.6 | 31.33 |
| Roach | 1206 | 3 | 1 | 1 | 108.75 | 21.16 | 7.85 | 12.74 | 26.51 | 26.26 | 19.39 | 49.26 | 17.59 | 52.11 | 23.96 | 76.04 | 17.38 | 20.57 | 10.9 | 34.98 |
| Roach | 1207 | 3 | 1 | 1 | 95.78 | 18.49 | 5.69 | 12.01 | 26.62 | 25.19 | 19.13 | 46.76 | 15.38 | 51.16 | 24.31 | 66.81 | 16.19 | 17.95 | 9.77 | 30.32 |
| Roach | 1208 | 3 | 1 | 1 | 96.19 | 18.55 | 6.33 | 10.71 | 22.69 | 23.05 | 16.35 | 46.64 | 15.46 | 48.23 | 21.73 | 67.06 | 18.32 | 16.62 | 9.76 | 30.41 |
| Roach | 1209 | 3 | 1 | 1 | 80.37 | 15.34 | 4.98 | 8.5 | 19.68 | 21.1 | 13.55 | 37.54 | 12.98 | 38.23 | 17.81 | 57.9 | 11.19 | 17.04 | 7.93 | 23.88 |
| Roach | 1210 | 3 | 1 | 1 | 91.78 | 17.22 | 6.46 | 9.32 | 22.07 | 23.59 | 17.03 | 45.59 | 15.55 | 46.43 | 19.05 | 64.18 | 13.79 | 14.47 | 8.89 | 26.13 |
| Roach | 1211 | 3 | 1 | 1 | 80.02 | 14.21 | 5 | 8.32 | 19.08 | 20.32 | 13.58 | 37.78 | 12.42 | 41.56 | 18.1 | 56.61 | 13.13 | 15.14 | 6.73 | 21.83 |
| Roach | 1212 | 3 | 1 | 1 | 108.46 | 20.55 | 7.24 | 11.56 | 25.09 | 26.09 | 18.49 | 52.21 | 19.1 | 54.41 | 24.91 | 74.62 | 17.78 | 17.13 | 10.55 | 33.76 |
| Roach | 1213 | 3 | 1 | 1 | 107.04 | 19.62 | 6.25 | 11.58 | 23.27 | 25.66 | 18.39 | 47.59 | 17.16 | 52.06 | 23.24 | 72.27 | 14.52 | 19.44 | 10.16 | 33.19 |
| Roach | 1214 | 3 | 1 | 1 | 103.13 | 19.11 | 6.19 | 11.62 | 24.22 | 25.06 | 18.72 | 50.07 | 15.3 | 51.68 | 29.07 | 74.07 | 14.41 | 16.87 | 8.84 | 33 |
| Roach | 1215 | 3 | 1 | 1 | 85.3 | 15.2 | 5.52 | 8.72 | 19.54 | 20.7 | 16.15 | 39.71 | 14.1 | 43.59 | 18.89 | 58.77 | 12.34 | 18.45 | 8.19 | 24.88 |
| Roach | 1216 | 3 | 1 | 1 | 104.84 | 20.15 | 6.09 | 10.84 | 24.46 | 26.05 | 20.77 | 51.91 | 17.87 | 57.78 | 25.13 | 72.39 | 18.1 | 19.4 | 10.14 | 32.15 |
| Roach | 1217 | 3 | 1 | 1 | 103.96 | 20.79 | 7.33 | 12.17 | 26.46 | 27.5 | 19.26 | 52.38 | 18.86 | 57.46 | 24.87 | 76.07 | 15.72 | 17.99 | 9.99 | 31.19 |
| Roach | 1218 | 3 | 1 | 1 | 92.04 | 18.26 | 6.57 | 9.13 | 23.49 | 23.6 | 14.89 | 45.68 | 15.58 | 49.64 | 20.73 | 65.55 | 14.5 | 17.11 | 9.84 | 30.28 |
| Roach | 1219 | 3 | 1 | 1 | 98.62 | 18.77 | 6.08 | 11.7 | 23.47 | 23.28 | 18.4 | 50.64 | 15.38 | 52.3 | 22.43 | 71.05 | 15.52 | 15.84 | 9.53 | 30.26 |
| Roach | 1220 | 3 | 1 | 1 | 71.54 | 13.65 | 4.62 | 7.68 | 16.39 | 15.4 | 12.13 | 36.01 | 13.1 | 37.35 | 16.31 | 54.36 | 11.43 | 15.22 | 6.99 | 21.21 |
| Roach | 1191 | 3 | 1 | 2 | 119.45 | 23.57 | 8.16 | 13.65 | 27.8 | 29.48 | 20.86 | 55.82 | 21.27 | 57.34 | 29.06 | 85.19 | 19.71 | 20.75 | 11.15 | 35.54 |
| Roach | 1192 | 3 | 1 | 2 | 110.45 | 20.06 | 7.21 | 12.32 | 26.63 | 27.54 | 18.75 | 51.32 | 18.8 | 51.88 | 25.02 | 78.73 | 17.08 | 18.79 | 10.89 | 36.46 |
| Roach | 1193 | 3 | 1 | 2 | 100.14 | 19.91 | 6.32 | 10.71 | 23.4 | 24.7 | 20.1 | 46.81 | 17.23 | 47.65 | 26.19 | 71.75 | 15.56 | 17.81 | 11.01 | 33.77 |
| Roach | 1194 | 3 | 1 | 2 | 86.71 | 17.84 | 5.56 | 8.69 | 20.71 | 20.91 | 14.69 | 42.71 | 15.2 | 43.7 | 19.38 | 60.81 | 13.94 | 18.76 | 9.35 | 27.28 |
| Roach | 1195 | 3 | 1 | 2 | 104.83 | 21.03 | 6.97 | 11.5 | 24 | 25.2 | 17.15 | 48.59 | 18.26 | 51.43 | 23.34 | 75.07 | 15.65 | 18.74 | 10.74 | 33.39 |
| Roach | 1196 | 3 | 1 | 2 | 110.15 | 20.73 | 6.99 | 11.91 | 26.41 | 27.9 | 18.38 | 49.64 | 18.64 | 51.86 | 26.46 | 78.07 | 17.78 | 19.74 | 10.78 | 34.8 |
| Roach | 1197 | 3 | 1 | 2 | 124.22 | 26.51 | 8.98 | 15.21 | 32.21 | 34.08 | 22.91 | 59.44 | 22.46 | 60.62 | 30.85 | 91.89 | 20.04 | 22.54 | 12.76 | 44.39 |
| Roach | 1198 | 3 | 1 | 2 | 92.01 | 19.31 | 5.67 | 10.41 | 21.64 | 22.67 | 15.78 | 43.9 | 16.55 | 45.52 | 20.35 | 66.9 | 15.6 | 18.82 | 9.04 | 28.28 |
| Roach | 1199 | 3 | 1 | 2 | 101.65 | 18.07 | 5.87 | 10.04 | 22.49 | 23.5 | 17.88 | 45.39 | 16.29 | 47.19 | 24.27 | 68.96 | 15.14 | 19.78 | 10.32 | 30.02 |
| Roach | 1200 | 3 | 1 | 2 | 111.07 | 20.95 | 6.8 | 12.28 | 25.34 | 26.25 | 18.73 | 51.9 | 17.47 | 54.46 | 24.16 | 77.3 | 16.87 | 21.01 | 9.93 | 33.28 |
| Roach | 1201 | 3 | 1 | 2 | 112.48 | 23.07 | 6.61 | 12.51 | 26.88 | 27.84 | 19.43 | 53.76 | 20.41 | 55.63 | 26.82 | 84.1 | 18.89 | 20.51 | 12.98 | 37.78 |
| Roach | 1202 | 3 | 1 | 2 | 92.34 | 19.41 | 6.23 | 12.64 | 22.71 | 23.92 | 16.26 | 42.16 | 17.33 | 44.71 | 22.99 | 63.55 | 15.81 | 16.95 | 9.77 | 30.75 |
| Roach | 1203 | 3 | 1 | 2 | 94.98 | 18.39 | 5.34 | 9.7 | 24.85 | 23.43 | 17.02 | 44.37 | 16.27 | 46.15 | 23.85 | 66.45 | 14.68 | 16.6 | 8.25 | 28.55 |
| Roach | 1204 | 3 | 1 | 2 | 99.04 | 18.46 | 6.33 | 12.39 | 23.94 | 25.52 | 18.39 | 46.93 | 16.65 | 49.5 | 22.98 | 69.21 | 15.71 | 19.06 | 8.91 | 31.24 |
| Roach | 1205 | 3 | 1 | 2 | 101.85 | 20.41 | 6.14 | 11.05 | 24.28 | 25.28 | 17.52 | 42.42 | 16.72 | 48.74 | 22.81 | 73.33 | 16.11 | 17.27 | 10.16 | 31.3 |
| Roach | 1206 | 3 | 1 | 2 | 109.75 | 21.32 | 6.22 | 12.34 | 25.86 | 26.93 | 19.3 | 51.34 | 19.66 | 52.53 | 23.56 | 75.33 | 14.87 | 19.74 | 11.71 | 35.37 |
| Roach | 1207 | 3 | 1 | 2 | 95.9 | 19.52 | 6.55 | 11.4 | 23.6 | 24.59 | 17.58 | 43.95 | 17.56 | 46.23 | 25.25 | 66.58 | 16.35 | 17.24 | 10.01 | 30.98 |
| Roach | 1208 | 3 | 1 | 2 | 97.36 | 18.5 | 6.49 | 10.53 | 21.63 | 22.4 | 17.41 | 42.42 | 17.17 | 46.44 | 23.41 | 68.44 | 16.22 | 17.37 | 9.29 | 29.84 |
| Roach | 1209 | 3 | 1 | 2 | 81.15 | 15.75 | 5.78 | 7.44 | 18.75 | 20.27 | 14.78 | 36.84 | 14.13 | 39.21 | 18.78 | 55.83 | 12.85 | 16.37 | 7.94 | 24.02 |
| Roach | 1210 | 3 | 1 | 2 | 88.34 | 17.28 | 6.36 | 10.36 | 22.09 | 23.37 | 16.35 | 43.52 | 16.01 | 47.3 | 21.07 | 63 | 14.48 | 15.72 | 9.32 | 26.51 |
| Roach | 1211 | 3 | 1 | 2 | 79.19 | 15.52 | 5.71 | 7.7 | 20.81 | 20.81 | 13.17 | 35.15 | 12.42 | 40.64 | 19.37 | 58.39 | 12.6 | 15.56 | 7.66 | 22.42 |
| Roach | 1212 | 3 | 1 | 2 | 108.22 | 20.78 | 7.28 | 10.61 | 24.73 | 25.89 | 19.01 | 50.65 | 19.73 | 52.71 | 25.63 | 74.36 | 18.17 | 18.69 | 10.34 | 32.74 |
| Roach | 1213 | 3 | 1 | 2 | 105.87 | 20.63 | 6.56 | 11.65 | 25.9 | 25.7 | 17.01 | 48.01 | 17.53 | 50.58 | 24.71 | 72.98 | 15.48 | 19.45 | 10.31 | 32.97 |
| Roach | 1214 | 3 | 1 | 2 | 102.57 | 19.71 | 6.98 | 10.78 | 23.96 | 25.29 | 17.34 | 47.19 | 16.44 | 48.47 | 27.77 | 74.89 | 16.26 | 19.24 | 9.85 | 33.72 |
| Roach | 1215 | 3 | 1 | 2 | 85.54 | 16.44 | 4.95 | 8.99 | 19.66 | 20.34 | 14.76 | 37.42 | 14.86 | 39.57 | 17.92 | 59.48 | 11.83 | 15.98 | 8.33 | 23.98 |
| Roach | 1216 | 3 | 1 | 2 | 105.62 | 20.69 | 6.98 | 11.97 | 24.75 | 26.61 | 19.61 | 48.02 | 19.78 | 50.29 | 26.25 | 73.55 | 18.94 | 20.11 | 10.94 | 32.28 |
| Roach | 1217 | 3 | 1 | 2 | 105 | 20.97 | 7.42 | 13.43 | 26.14 | 28.07 | 19.51 | 50.6 | 19.02 | 53.7 | 25.45 | 76.1 | 15.3 | 17.11 | 9.66 | 30.85 |
| Roach | 1218 | 3 | 1 | 2 | 91.77 | 19.01 | 7.35 | 10.02 | 22.75 | 23.49 | 16.64 | 43.73 | 16.83 | 45.09 | 22.11 | 65.51 | 14.91 | 16.82 | 9.98 | 30 |
| Roach | 1219 | 3 | 1 | 2 | 98.95 | 19.79 | 6.82 | 10.45 | 23.36 | 25.16 | 19.34 | 47.09 | 17.87 | 49.48 | 26.44 | 71.11 | 15.39 | 18.51 | 10.51 | 31.05 |
| Roach | 1220 | 3 | 1 | 2 | 70 | 12.96 | 4.26 | 6.42 | 16.9 | 18.85 | 13.07 | 34.05 | 13.17 | 36.01 | 18.92 | 49.43 | 13.31 | 14.19 | 6.59 | 20.71 |
| Roach | 1191 | 3 | 1 | 3 | 119.4 | 23.59 | 7.11 | 13.07 | 27.68 | 28.68 | 22.9 | 56.26 | 21.41 | 58.66 | 28.55 | 84.72 | 19.72 | 20.85 | 11.75 | 35.47 |
| Roach | 1192 | 3 | 1 | 3 | 110.25 | 20.59 | 6.99 | 12.44 | 26.16 | 27.44 | 18.55 | 51.09 | 17.56 | 52.87 | 27.21 | 76.36 | 16.99 | 20.03 | 11.13 | 36.26 |
| Roach | 1193 | 3 | 1 | 3 | 100.54 | 20.52 | 6.17 | 10.75 | 23.29 | 24.57 | 18.51 | 46.86 | 18.53 | 47.88 | 26.09 | 70.84 | 16.12 | 16.31 | 10.28 | 34.59 |
| Roach | 1194 | 3 | 1 | 3 | 88.11 | 16.57 | 6.54 | 8.89 | 16.61 | 20.9 | 14.89 | 42.52 | 16.16 | 43.2 | 18.92 | 63.92 | 14.07 | 16.39 | 9.24 | 28.01 |
| Roach | 1195 | 3 | 1 | 3 | 104.58 | 21.43 | 6.39 | 10.78 | 24.08 | 25.03 | 16.7 | 48.31 | 17.76 | 50.68 | 23.23 | 74.47 | 15.62 | 18.55 | 10.74 | 33.33 |
| Roach | 1196 | 3 | 1 | 3 | 106.64 | 21.05 | 7.2 | 12.33 | 26.17 | 27.59 | 19.59 | 50.54 | 19.02 | 54.37 | 25.32 | 79 | 18.02 | 18.45 | 11.46 | 35.34 |
| Roach | 1197 | 3 | 1 | 3 | 124.75 | 26.62 | 8.21 | 15.11 | 30.94 | 32.53 | 22.86 | 60.79 | 22.3 | 63.11 | 31.44 | 93.34 | 19.74 | 21.61 | 12.73 | 44.19 |
| Roach | 1198 | 3 | 1 | 3 | 92.72 | 18.86 | 5.44 | 10.05 | 21.71 | 23.76 | 14.91 | 44.13 | 16.37 | 46 | 21.07 | 67.52 | 16.05 | 17.57 | 10.35 | 29.25 |
| Roach | 1199 | 3 | 1 | 3 | 100.56 | 18.17 | 7.25 | 10.95 | 23.01 | 24.03 | 17.33 | 43.73 | 16.58 | 44.86 | 24.11 | 68.38 | 15.51 | 19.62 | 10.65 | 30.63 |
| Roach | 1200 | 3 | 1 | 3 | 110.52 | 21.76 | 6.8 | 11.2 | 25.1 | 26.47 | 18.84 | 50.93 | 18.6 | 52.61 | 24.76 | 77.56 | 17.52 | 21.32 | 9.81 | 33.5 |
| Roach | 1201 | 3 | 1 | 3 | 113.86 | 21.85 | 7.41 | 13.07 | 27.24 | 28.52 | 18.94 | 43.71 | 20.73 | 56.37 | 28.14 | 81.02 | 19.93 | 20.96 | 11.89 | 37.95 |
| Roach | 1202 | 3 | 1 | 3 | 93.72 | 18.73 | 6.25 | 9.59 | 20.33 | 21.2 | 15.65 | 43.02 | 16.89 | 45.37 | 25.41 | 64.06 | 14.08 | 18.11 | 10.18 | 31.39 |
| Roach | 1203 | 3 | 1 | 3 | 93.42 | 18.04 | 5.38 | 10.43 | 22.66 | 23.4 | 16.81 | 43.55 | 16.21 | 44.6 | 26.9 | 66.09 | 14.31 | 15.94 | 7.84 | 29.83 |
| Roach | 1204 | 3 | 1 | 3 | 99.62 | 18.25 | 6.95 | 11.4 | 24.06 | 24.69 | 18.57 | 46.4 | 16.88 | 48.02 | 23.06 | 70.14 | 14.39 | 18.17 | 9.08 | 32.25 |
| Roach | 1205 | 3 | 1 | 3 | 101.97 | 19.98 | 6.35 | 11.18 | 22.89 | 24.23 | 17.09 | 46.85 | 19.01 | 48.9 | 24.97 | 79.07 | 14.45 | 15.8 | 9.38 | 31 |
| Roach | 1206 | 3 | 1 | 3 | 109.64 | 21.75 | 7.83 | 11.9 | 25.54 | 27.1 | 19.37 | 49.68 | 19.66 | 51.17 | 24.19 | 77 | 18.35 | 19.35 | 11.49 | 35.73 |
| Roach | 1207 | 3 | 1 | 3 | 94.67 | 19.62 | 6.24 | 12 | 23.83 | 25.22 | 20.56 | 43.01 | 17.21 | 44.62 | 25.39 | 66.69 | 16.79 | 15.45 | 8.98 | 30.8 |
| Roach | 1208 | 3 | 1 | 3 | 97.63 | 18.41 | 6.47 | 10.62 | 21.99 | 23.32 | 16.19 | 43.74 | 16.59 | 45.78 | 23.72 | 66.66 | 18.76 | 16.93 | 9.55 | 30.77 |
| Roach | 1209 | 3 | 1 | 3 | 82.38 | 14.39 | 5.79 | 8.82 | 18.56 | 20.25 | 12.69 | 38.18 | 13.36 | 40.07 | 18.08 | 57.92 | 12.73 | 15.22 | 8.28 | 24.76 |
| Roach | 1210 | 3 | 1 | 3 | 91.61 | 16.62 | 6.31 | 9.29 | 21.06 | 22.51 | 15.995 | 44.19 | 16.15 | 48.95 | 21.66 | 63.09 | 15.09 | 14.37 | 8.34 | 25.81 |
| Roach | 1211 | 3 | 1 | 3 | 79.54 | 15.89 | 5.1 | 8.01 | 19.69 | 21.04 | 12.9 | 37.22 | 12.02 | 39.32 | 20.17 | 59.39 | 12.91 | 16.39 | 7.98 | 21.79 |
| Roach | 1213 | 3 | 1 | 3 | 106.41 | 21.2 | 6.27 | 11.78 | 24.44 | 25.7 | 17.89 | 46.53 | 18.22 | 47.42 | 23.87 | 73.89 | 15.87 | 17.96 | 10.37 | 33.07 |
| Roach | 1212 | 3 | 1 | 3 | 108.49 | 21.91 | 6.71 | 11.07 | 25.66 | 26.45 | 19.84 | 50.6 | 19.07 | 53.21 | 26.51 | 76.3 | 18.01 | 19.18 | 10.71 | 33.75 |
| Roach | 1214 | 3 | 1 | 3 | 101.97 | 19.26 | 6.48 | 11.95 | 23.93 | 25.48 | 17.92 | 48.31 | 16.07 | 48.42 | 27.31 | 73.46 | 16.2 | 16.69 | 9.69 | 34.07 |
| Roach | 1215 | 3 | 1 | 3 | 84.22 | 15.42 | 4.53 | 8.15 | 19.28 | 20.5 | 14.72 | 38.16 | 13.55 | 39.72 | 18.8 | 58.46 | 12.92 | 18.42 | 7.97 | 23.78 |
| Roach | 1216 | 3 | 1 | 3 | 105.52 | 21.47 | 6.25 | 11.82 | 23.95 | 25.56 | 20.6 | 49.47 | 19.08 | 53.09 | 25.86 | 74.37 | 18.65 | 17.81 | 10.5 | 33.93 |
| Roach | 1217 | 3 | 1 | 3 | 107.37 | 21.2 | 6.83 | 12.08 | 26.33 | 27.16 | 19.07 | 49.86 | 18.48 | 50.14 | 24.5 | 75.65 | 16.73 | 15.55 | 8.82 | 39.79 |
| Roach | 1218 | 3 | 1 | 3 | 90.9 | 19.34 | 6.25 | 11.52 | 22.91 | 24.75 | 16.22 | 42.48 | 15.96 | 44.76 | 21.61 | 63.02 | 14.94 | 13.99 | 9.16 | 30.03 |
| Roach | 1219 | 3 | 1 | 3 | 98.49 | 18.85 | 6.13 | 9.85 | 23.73 | 25.3 | 18.07 | 36.47 | 17.39 | 47.83 | 23.89 | 69.87 | 14.74 | 14.92 | 9.54 | 30.99 |
| Roach | 1220 | 3 | 1 | 3 | 69.87 | 13.86 | 4.52 | 7.04 | 16.82 | 18.58 | 10.97 | 34.45 | 12.64 | 35.64 | 16.55 | 39.55 | 11.65 | 14.64 | 6.62 | 16.92 |
| Roach | 1001 | 1 | 2 | 1 | 91.76 | 16.18 | 5.84 | 10.12 | 22.21 | 21.92 | 14.94 | 43.19 | 15.82 | 43.68 | 20.55 | 62.71 | 14 | 20.02 | 10.05 | 28.92 |
| Roach | 1002 | 1 | 2 | 1 | 114 | 20.72 | 5.82 | 13.86 | 27.58 | 26.39 | 21.45 | 54.6 | 20.5 | 59.9 | 27.5 | 82.33 | 18.1 | 19.49 | 13.28 | 36.7 |
| Roach | 1003 | 1 | 2 | 1 | 134.22 | 23.28 | 8.11 | 16.61 | 31.25 | 29.47 | 24.61 | 61.51 | 23.4 | 61.47 | 30.52 | 93.71 | 18.8 | 27.49 | 15.25 | 42.98 |
| Roach | 1004 | 1 | 2 | 1 | 85.62 | 15.71 | 4.76 | 10.31 | 20.07 | 19.7 | 13.68 | 41.11 | 15.82 | 41.12 | 22.09 | 60.29 | 13.1 | 16.77 | 9.24 | 26.11 |
| Roach | 1005 | 1 | 2 | 1 | 118.57 | 21.33 | 6.94 | 14.97 | 28.46 | 26.89 | 18.85 | 56.74 | 20.2 | 60.33 | 26.97 | 84.58 | 17.64 | 21.98 | 12.66 | 38.5 |
| Roach | 1006 | 1 | 2 | 1 | 132.23 | 24.44 | 7.62 | 16.55 | 30.03 | 28.82 | 24.3 | 63.19 | 22.29 | 63.92 | 30.27 | 95.33 | 18.55 | 23.06 | 14.61 | 42.75 |
| Roach | 1007 | 1 | 2 | 1 | 92.72 | 16.49 | 4.52 | 9.86 | 22.03 | 20.81 | 17.06 | 42.48 | 15.66 | 47.46 | 19.92 | 61.77 | 13.77 | 18.43 | 9.77 | 28.55 |
| Roach | 1008 | 1 | 2 | 1 | 78.25 | 14.46 | 4.65 | 9.43 | 20.13 | 18.66 | 13.27 | 37.21 | 12.41 | 38.44 | 18.34 | 55.56 | 11.5 | 14.38 | 8.33 | 24.86 |
| Roach | 1009 | 1 | 2 | 1 | 90.71 | 15.38 | 4.37 | 9.94 | 21.09 | 19.45 | 15.58 | 42.38 | 15.18 | 45.62 | 20.45 | 64.46 | 14.3 | 16.41 | 9.6 | 27.71 |
| Roach | 1010 | 1 | 2 | 1 | 92.98 | 18.35 | 6.16 | 10.66 | 23.39 | 22.64 | 16.52 | 45.18 | 16.79 | 44.41 | 22.6 | 66.98 | 13.86 | 18.61 | 10.53 | 29.57 |
| Roach | 1011 | 1 | 2 | 1 | 83.77 | 14.32 | 4.61 | 8.41 | 20.15 | 18.37 | 14.75 | 35.13 | 14.16 | 40.53 | 19.87 | 55.48 | 13.37 | 16.21 | 8.62 | 25.34 |
| Roach | 1012 | 1 | 2 | 1 | 99.4 | 17.85 | 5.33 | 11.27 | 23.44 | 20.61 | 17.76 | 47 | 17.85 | 52.61 | 23.25 | 69.11 | 15.05 | 19.92 | 9.99 | 30.08 |
| Roach | 1013 | 1 | 2 | 1 | 92.43 | 16.02 | 5.26 | 10.51 | 21.71 | 20.01 | 16.92 | 42.46 | 16.95 | 46.32 | 23.14 | 60.97 | 14.34 | 18.08 | 9.31 | 27.69 |
| Roach | 1014 | 1 | 2 | 1 | 127.28 | 22.73 | 6.89 | 15.35 | 28.81 | 27.73 | 23.95 | 58.9 | 25.03 | 66.72 | 30.09 | 88.67 | 17.99 | 24.65 | 13.85 | 39.09 |
| Roach | 1015 | 1 | 2 | 1 | 130.45 | 22.85 | 8.08 | 15.74 | 31.2 | 30.04 | 23.6 | 62.79 | 23.23 | 66.15 | 28.36 | 89.68 | 18.43 | 25.41 | 13.7 | 40.21 |
| Roach | 1016 | 1 | 2 | 1 | 90.46 | 16.74 | 5.42 | 9.41 | 22.47 | 20.54 | 16.8 | 40.88 | 15.65 | 43.07 | 20.98 | 61.39 | 14.85 | 19.12 | 9.79 | 29.66 |
| Roach | 1017 | 1 | 2 | 1 | 72.85 | 12.92 | 3.9 | 8.43 | 18.17 | 16.78 | 13.35 | 32 | 13.52 | 34.58 | 18.17 | 51.88 | 12.55 | 14.43 | 7.3 | 22.37 |
| Roach | 1018 | 1 | 2 | 1 | 110.28 | 20.93 | 6.94 | 12.68 | 26.34 | 25.68 | 20.33 | 50.64 | 19.8 | 51.06 | 27.4 | 76.59 | 17.77 | 20.26 | 11.6 | 35.54 |
| Roach | 1019 | 1 | 2 | 1 | 92.28 | 16.69 | 4.69 | 10.64 | 22.45 | 22.17 | 15.55 | 45.96 | 15.71 | 46.01 | 22.64 | 66.71 | 14.49 | 16.94 | 9.68 | 29.93 |
| Roach | 1020 | 1 | 2 | 1 | 118.47 | 21.73 | 6.18 | 14.81 | 27.96 | 26.98 | 21.31 | 56.64 | 21.69 | 58.01 | 26.79 | 85.88 | 18.9 | 20.78 | 12.91 | 37.62 |
| Roach | 1021 | 1 | 2 | 1 | 111.77 | 20 | 5.42 | 12.57 | 25.95 | 23.91 | 21.02 | 49.69 | 19.52 | 56.73 | 26.87 | 76.46 | 17.63 | 20.22 | 11.84 | 33.48 |
| Roach | 1022 | 1 | 2 | 1 | 95.43 | 17.91 | 5.03 | 11.08 | 25.145 | 23.69 | 17.27 | 46.21 | 16.32 | 48.06 | 19.89 | 66.39 | 14.82 | 16.81 | 9.43 | 27.68 |
| Roach | 1023 | 1 | 2 | 1 | 80.64 | 13.49 | 4.02 | 8.75 | 19.33 | 17.95 | 14.2 | 37.76 | 13.86 | 43.67 | 18.45 | 58.89 | 13.05 | 18.36 | 8.18 | 23.5 |
| Roach | 1024 | 1 | 2 | 1 | 98.05 | 19.54 | 5.32 | 11.73 | 23.91 | 21.54 | 19.18 | 43.56 | 18.65 | 48.14 | 24.35 | 64.04 | 16.56 | 16.76 | 10.95 | 31.24 |
| Roach | 1025 | 1 | 2 | 1 | 94.94 | 17.55 | 5.58 | 10.98 | 22.01 | 20.59 | 16.74 | 45.85 | 17.21 | 48.69 | 23.45 | 64.71 | 14.82 | 16.47 | 10.41 | 31.5 |
| Roach | 1026 | 1 | 2 | 1 | 120.48 | 21.52 | 6.47 | 14 | 28.58 | 26.88 | 20.92 | 54.85 | 20.02 | 60.63 | 25.11 | 83.69 | 16.64 | 23.71 | 12.98 | 37.13 |
| Roach | 1027 | 1 | 2 | 1 | 101.57 | 19.27 | 5.67 | 11.69 | 24.36 | 21.23 | 18.22 | 46.7 | 16.58 | 52.61 | 24.32 | 71.22 | 16.57 | 21.18 | 10.73 | 31.67 |
| Roach | 1028 | 1 | 2 | 1 | 117.58 | 21 | 6.19 | 13.62 | 28.15 | 26.87 | 22.08 | 57.45 | 19.81 | 54.94 | 25.96 | 82.43 | 17.55 | 22.95 | 12.08 | 35.55 |
| Roach | 1029 | 1 | 2 | 1 | 105.28 | 18.82 | 6.06 | 14.15 | 25.38 | 23.68 | 18.26 | 52.88 | 17.94 | 52.74 | 24.99 | 77.29 | 16.57 | 22.1 | 10.96 | 33.18 |
| Roach | 1030 | 1 | 2 | 1 | 96.58 | 18.46 | 5.53 | 11.05 | 22.87 | 21.42 | 17.27 | 43.45 | 17.01 | 51.97 | 21.93 | 66.09 | 14.55 | 18.06 | 10.08 | 31.7 |
| Roach | 1001 | 1 | 2 | 2 | 91.13 | 15.75 | 4.43 | 10.38 | 20.79 | 19.19 | 16.14 | 40.01 | 15.79 | 44.67 | 23.4 | 61.98 | 14.47 | 17.9 | 9.4 | 28.69 |
| Roach | 1002 | 1 | 2 | 2 | 113.98 | 21 | 6.82 | 13.54 | 29.09 | 27.1 | 21.77 | 57.13 | 21.36 | 57.07 | 27.04 | 82.62 | 17.28 | 20.33 | 12.29 | 36.56 |
| Roach | 1003 | 1 | 2 | 2 | 135.51 | 24.32 | 7.96 | 15.18 | 29.83 | 29.59 | 25.1 | 61.4 | 23.64 | 66.39 | 30.5 | 91.59 | 20.45 | 27.01 | 14.86 | 43.82 |
| Roach | 1004 | 1 | 2 | 2 | 85.36 | 15.27 | 4.27 | 8.81 | 19.93 | 19.09 | 14.62 | 41.07 | 15.56 | 42.39 | 22.16 | 59.85 | 13.4 | 16.55 | 9.19 | 25.88 |
| Roach | 1005 | 1 | 2 | 2 | 116.17 | 22.91 | 6.9 | 14.08 | 28.44 | 27.37 | 18.6 | 56.36 | 21.13 | 55.66 | 26.2 | 84.02 | 18.28 | 21.57 | 12.61 | 37.71 |
| Roach | 1006 | 1 | 2 | 2 | 133.19 | 23.52 | 7.78 | 15.45 | 31.26 | 29.05 | 24.8 | 61.59 | 24.13 | 68.6 | 28.38 | 92.69 | 18.49 | 26.34 | 14.5 | 43.06 |
| Roach | 1007 | 1 | 2 | 2 | 92.6 | 16.17 | 4.96 | 9.85 | 21.68 | 20.63 | 16.11 | 41.52 | 15.64 | 45.7 | 20.63 | 60.85 | 13.53 | 19.71 | 10.01 | 28.69 |
| Roach | 1008 | 1 | 2 | 2 | 80.21 | 16.3 | 3.92 | 9.41 | 19.3 | 18.63 | 13.66 | 37.03 | 13.11 | 36.03 | 19.08 | 56.33 | 11.74 | 17.41 | 8.62 | 25.03 |
| Roach | 1009 | 1 | 2 | 2 | 91.24 | 16.86 | 5.61 | 9.76 | 22.46 | 21.69 | 15.59 | 45.36 | 15.45 | 47.03 | 21.41 | 63.17 | 13.13 | 15.34 | 9.92 | 28.18 |
| Roach | 1010 | 1 | 2 | 2 | 92.1 | 18.48 | 6.28 | 11.03 | 24.05 | 23.17 | 17.85 | 46 | 15.92 | 47.17 | 20.45 | 65.75 | 14.12 | 18.83 | 10.5 | 30.08 |
| Roach | 1011 | 1 | 2 | 2 | 83.14 | 14.91 | 4.32 | 10.16 | 20.24 | 19.9 | 15.4 | 39.27 | 14.41 | 39.56 | 19.66 | 59.04 | 12.24 | 16.41 | 8.62 | 25.97 |
| Roach | 1012 | 1 | 2 | 2 | 98.58 | 18.37 | 5.23 | 11.59 | 22.69 | 19.75 | 18.37 | 46.15 | 17.43 | 52.54 | 24.07 | 70.79 | 15.81 | 18.37 | 9.97 | 30.7 |
| Roach | 1013 | 1 | 2 | 2 | 93.1 | 15.57 | 5.05 | 11.04 | 21.57 | 20.45 | 16.73 | 42.29 | 17.06 | 43.83 | 23.26 | 63.56 | 13.99 | 18.91 | 9.68 | 28.13 |
| Roach | 1014 | 1 | 2 | 2 | 129.17 | 25.16 | 6.76 | 14.98 | 29.09 | 27.02 | 22.74 | 59.44 | 23.06 | 64.93 | 29.91 | 87.46 | 19.22 | 25.66 | 13.73 | 39.48 |
| Roach | 1015 | 1 | 2 | 2 | 130.33 | 23.34 | 7.5 | 14.07 | 30.31 | 29.93 | 24.18 | 57.4 | 21.42 | 62.87 | 27.64 | 88.43 | 17.18 | 24.61 | 14.13 | 40.55 |
| Roach | 1016 | 1 | 2 | 2 | 88.93 | 16.64 | 5.19 | 9.04 | 20.99 | 20.57 | 15.52 | 41.93 | 15.88 | 46.38 | 20 | 60.65 | 12.22 | 17.63 | 9.53 | 29.58 |
| Roach | 1017 | 1 | 2 | 2 | 73.06 | 12.83 | 4.02 | 8.19 | 17.38 | 16.82 | 12.66 | 32.98 | 14.68 | 32.1 | 18.56 | 50.82 | 12.74 | 13.93 | 7.04 | 21.82 |
| Roach | 1018 | 1 | 2 | 2 | 111.9 | 21.12 | 6.19 | 12.27 | 25.2 | 23.69 | 20.68 | 50.98 | 17.5 | 52.23 | 26.16 | 77.07 | 16.64 | 20.31 | 11.33 | 35.31 |
| Roach | 1019 | 1 | 2 | 2 | 92.5 | 17.02 | 5.35 | 10.18 | 22.53 | 22.57 | 16.09 | 45.39 | 16.37 | 46.07 | 21.36 | 66.05 | 14.49 | 18.88 | 10.18 | 29.65 |
| Roach | 1020 | 1 | 2 | 2 | 118.8 | 22.65 | 6.15 | 14.05 | 27.87 | 27.4 | 21.44 | 57.75 | 21.23 | 57.79 | 27.97 | 86.4 | 17.46 | 21.76 | 12.63 | 38.64 |
| Roach | 1021 | 1 | 2 | 2 | 112.39 | 19.19 | 5.91 | 12.03 | 25.8 | 25.59 | 20.9 | 53.57 | 18.87 | 57.17 | 27.1 | 79.47 | 16.74 | 20.79 | 12.09 | 34.9 |
| Roach | 1022 | 1 | 2 | 2 | 94.25 | 18.13 | 6.1 | 11.59 | 24.76 | 24.28 | 16.28 | 49.76 | 15.47 | 49.48 | 21.1 | 66.34 | 14.67 | 16.09 | 10.31 | 27.49 |
| Roach | 1023 | 1 | 2 | 2 | 82.26 | 14.58 | 4.67 | 8.98 | 19.6 | 18.32 | 15.52 | 38.78 | 14.13 | 39.49 | 17.17 | 59.28 | 13.73 | 18.83 | 8.24 | 24.14 |
| Roach | 1024 | 1 | 2 | 2 | 98.1 | 18.81 | 5.87 | 11.72 | 24.14 | 22.52 | 17.25 | 45.88 | 17 | 46.03 | 23.54 | 68.21 | 16.3 | 20.26 | 10.49 | 31.77 |
| Roach | 1025 | 1 | 2 | 2 | 95.88 | 16.9 | 6.31 | 10.01 | 23.42 | 22.67 | 18.08 | 42.42 | 18.29 | 49.31 | 23.87 | 65.33 | 15.23 | 18.41 | 10.35 | 31.04 |
| Roach | 1026 | 1 | 2 | 2 | 118.6 | 24.05 | 6.41 | 14.03 | 27.83 | 26.94 | 20.72 | 58.78 | 21.73 | 57.36 | 26.29 | 84.42 | 16.22 | 21.13 | 12.34 | 37.27 |
| Roach | 1027 | 1 | 2 | 2 | 101.12 | 19.14 | 5.51 | 11.44 | 24.1 | 22.06 | 18.66 | 46.39 | 15.12 | 50.48 | 24.54 | 69.02 | 16.05 | 19.82 | 11.1 | 31.48 |
| Roach | 1028 | 1 | 2 | 2 | 117.1 | 20.76 | 6.83 | 13.04 | 27.5 | 25.72 | 20.03 | 54.15 | 18.41 | 57.3 | 26.06 | 81.92 | 15.86 | 21.35 | 12.38 | 34.47 |
| Roach | 1029 | 1 | 2 | 2 | 108.24 | 20.67 | 6.61 | 12.67 | 25.73 | 24.32 | 18.41 | 51.76 | 17.66 | 55.2 | 25.12 | 75.84 | 16.34 | 20.94 | 11.46 | 32.7 |
| Roach | 1030 | 1 | 2 | 2 | 95.13 | 19.31 | 5.26 | 11.57 | 23.26 | 22.16 | 17.43 | 49.23 | 17.11 | 49.35 | 22.01 | 66.66 | 13.49 | 18.33 | 9.95 | 31.52 |
| Roach | 1001 | 1 | 2 | 3 | 92.48 | 15.94 | 5.9 | 10.69 | 21.45 | 21.26 | 16.02 | 40.14 | 15.76 | 47.31 | 20.81 | 62.41 | 13.77 | 19.05 | 9.82 | 29.26 |
| Roach | 1002 | 1 | 2 | 3 | 113.28 | 21.09 | 7.13 | 13.64 | 27.35 | 27.15 | 21.59 | 58.39 | 19.9 | 60.43 | 28.44 | 82.57 | 17.69 | 21.33 | 12.75 | 36.5 |
| Roach | 1003 | 1 | 2 | 3 | 132.58 | 25.68 | 7.81 | 15.38 | 31.06 | 28.48 | 25.46 | 61.48 | 22.59 | 67.4 | 30.2 | 91.45 | 18.99 | 23 | 14.61 | 43.67 |
| Roach | 1004 | 1 | 2 | 3 | 85.36 | 15.92 | 4.86 | 10.14 | 20.39 | 18.59 | 14.56 | 38.46 | 14.43 | 42.77 | 20.8 | 56.59 | 13.07 | 17.69 | 8.88 | 26.01 |
| Roach | 1005 | 1 | 2 | 3 | 116.61 | 19.11 | 6.92 | 15.33 | 29.3 | 27.26 | 19.3 | 54.53 | 20.58 | 58.06 | 26.96 | 85.32 | 17.44 | 21.23 | 12.68 | 37.88 |
| Roach | 1006 | 1 | 2 | 3 | 132.89 | 24.19 | 6.91 | 16.2 | 30.35 | 29.36 | 24.8 | 63.13 | 22.01 | 65.39 | 31.15 | 95.63 | 18.55 | 24.91 | 14.59 | 42.41 |
| Roach | 1007 | 1 | 2 | 3 | 93.32 | 16.1 | 6.2 | 11.47 | 22.87 | 22.57 | 16 | 44.63 | 15.78 | 50.63 | 19.78 | 65.01 | 12.76 | 17.12 | 10.31 | 28.74 |
| Roach | 1008 | 1 | 2 | 3 | 74.01 | 14.49 | 4.05 | 9.45 | 17.15 | 18.47 | 15.04 | 30.12 | 13.73 | 34.87 | 17.57 | 47.13 | 12.03 | 15.19 | 8.69 | 24.57 |
| Roach | 1009 | 1 | 2 | 3 | 90.18 | 16.11 | 5.08 | 10.09 | 21.43 | 19.02 | 16.5 | 45.93 | 15.68 | 43.2 | 23.19 | 64.1 | 13.42 | 18.28 | 9.61 | 27.68 |
| Roach | 1010 | 1 | 2 | 3 | 93.48 | 18.16 | 5.39 | 10.79 | 23.04 | 20.99 | 17.62 | 44.04 | 14.04 | 48.83 | 20.8 | 63.4 | 13.88 | 19.06 | 10.55 | 29.42 |
| Roach | 1011 | 1 | 2 | 3 | 82.43 | 15.48 | 4.41 | 9.53 | 19.7 | 18.34 | 15.63 | 34.73 | 13.64 | 40.47 | 19.66 | 54.39 | 12.26 | 16.69 | 8.51 | 25.25 |
| Roach | 1012 | 1 | 2 | 3 | 98.4 | 19.17 | 5.37 | 11.51 | 22.43 | 22.98 | 17.04 | 50.43 | 18.82 | 50.68 | 21.29 | 69.79 | 15.98 | 20.59 | 10.07 | 31.14 |
| Roach | 1013 | 1 | 2 | 3 | 90.93 | 16.95 | 5.58 | 10.85 | 21.55 | 20.52 | 17.8 | 42.02 | 16.97 | 45.11 | 21.69 | 61.06 | 14.09 | 17.4 | 9.49 | 28.12 |
| Roach | 1014 | 1 | 2 | 3 | 128 | 24.27 | 7.32 | 14.52 | 28.15 | 27.14 | 23.17 | 55.93 | 22.58 | 66.73 | 29.93 | 88.24 | 18.32 | 24.03 | 13.48 | 39.23 |
| Roach | 1015 | 1 | 2 | 3 | 130.36 | 22.9 | 7.15 | 14.62 | 29.78 | 29.02 | 24.02 | 57.93 | 23.23 | 66.13 | 29.52 | 91.37 | 18.59 | 24.83 | 13.9 | 40.42 |
| Roach | 1016 | 1 | 2 | 3 | 86.56 | 16.31 | 5.1 | 9.54 | 21.46 | 20.75 | 16.29 | 42.04 | 16.21 | 45.84 | 21.25 | 60.69 | 12.96 | 17.45 | 9.88 | 29.16 |
| Roach | 1017 | 1 | 2 | 3 | 72.79 | 12.71 | 4.73 | 8.62 | 17.52 | 17.04 | 13 | 31.21 | 13.15 | 34.32 | 19.12 | 49.8 | 13.06 | 13.81 | 7.13 | 21.79 |
| Roach | 1018 | 1 | 2 | 3 | 110.27 | 21.39 | 6.07 | 11.92 | 24.69 | 23.54 | 21.02 | 47.02 | 18.68 | 54.41 | 26.36 | 75.94 | 17.04 | 23.26 | 11.77 | 35.15 |
| Roach | 1019 | 1 | 2 | 3 | 91.15 | 16.58 | 6.02 | 10.5 | 22.7 | 22.92 | 16.57 | 45.82 | 16.17 | 46.5 | 20.75 | 66.48 | 14.59 | 20.91 | 9.87 | 29.49 |
| Roach | 1020 | 1 | 2 | 3 | 118.11 | 21.07 | 6.17 | 13.78 | 26.88 | 24.17 | 21.83 | 51.41 | 21.57 | 60.8 | 25.64 | 82.99 | 16.14 | 20.9 | 12.93 | 37.01 |
| Roach | 1021 | 1 | 2 | 3 | 112.16 | 19.79 | 6.49 | 12.18 | 23.93 | 22.57 | 21.27 | 51.15 | 20.27 | 54.43 | 28.23 | 76.93 | 16.57 | 22.97 | 11.74 | 33.74 |
| Roach | 1022 | 1 | 2 | 3 | 95.22 | 18.62 | 5.49 | 10.71 | 23.58 | 22.42 | 17.32 | 45.81 | 15.5 | 46.79 | 26.1 | 70.28 | 13.17 | 17.92 | 9.6 | 28.32 |
| Roach | 1023 | 1 | 2 | 3 | 82.98 | 14.61 | 4.3 | 8.32 | 18.68 | 18.32 | 13.73 | 38.02 | 13.02 | 43.02 | 19.96 | 55.57 | 12.64 | 16.32 | 8.3 | 23.56 |
| Roach | 1024 | 1 | 2 | 3 | 97.87 | 18.84 | 6.53 | 13.07 | 25.5 | 24.57 | 18.74 | 48.15 | 17.6 | 47.2 | 24.58 | 67.28 | 15.94 | 18.4 | 11.34 | 31.96 |
| Roach | 1025 | 1 | 2 | 3 | 96.56 | 18.4 | 5.31 | 10.73 | 22.77 | 22.03 | 18.09 | 47.61 | 18.48 | 48.31 | 24.96 | 66.67 | 14.14 | 16.71 | 10.81 | 31.42 |
| Roach | 1026 | 1 | 2 | 3 | 118.21 | 21.58 | 7.05 | 14.37 | 29.91 | 28.45 | 20.09 | 58.74 | 19.99 | 60.93 | 25.43 | 83.26 | 16.48 | 23.91 | 12.62 | 37.42 |
| Roach | 1027 | 1 | 2 | 3 | 101.5 | 17.88 | 5.41 | 11.54 | 24.43 | 21.8 | 18.59 | 47.35 | 17.27 | 54.89 | 23.74 | 70.63 | 15.59 | 21.77 | 10.52 | 31.62 |
| Roach | 1028 | 1 | 2 | 3 | 118.21 | 20.27 | 6.93 | 12.95 | 27.01 | 25.61 | 19.84 | 55.72 | 19.33 | 57.71 | 25.54 | 81.26 | 15.98 | 21.81 | 12.39 | 35.43 |
| Roach | 1029 | 1 | 2 | 3 | 106.79 | 19.28 | 5.26 | 12.78 | 25.53 | 24.3 | 18.38 | 51.79 | 18.87 | 55.82 | 26.2 | 75.23 | 14.97 | 21.39 | 10.88 | 32.2 |
| Roach | 1030 | 1 | 2 | 3 | 95.3 | 18.75 | 5.34 | 10.71 | 22.11 | 21.48 | 16.69 | 43.37 | 15.76 | 48.66 | 21.89 | 64.77 | 15.13 | 17.86 | 10.67 | 30.96 |
| Roach | 1101 | 2 | 2 | 1 | 130.43 | 23.51 | 6.75 | 16.35 | 29.77 | 29.35 | 22.78 | 63.3 | 22.79 | 62.54 | 30.9 | 93.18 | 19.05 | 26.05 | 13.94 | 40.49 |
| Roach | 1102 | 2 | 2 | 1 | 111.39 | 21.3 | 5.41 | 11.66 | 26.41 | 24.28 | 20.55 | 53.29 | 19.02 | 53.41 | 25.38 | 78.36 | 16.12 | 21.14 | 11.64 | 33.26 |
| Roach | 1103 | 2 | 2 | 1 | 117.63 | 21.9 | 6.65 | 14.27 | 28.22 | 27.44 | 19.38 | 56.42 | 21.14 | 56.86 | 27.19 | 87.57 | 18.23 | 19.82 | 12.24 | 35.16 |
| Roach | 1104 | 2 | 2 | 1 | 112 | 23.27 | 5.66 | 13.16 | 26.17 | 25.28 | 18.2 | 46.95 | 18.41 | 53.5 | 24.37 | 85.26 | 18.54 | 22.22 | 11.91 | 37.28 |
| Roach | 1105 | 2 | 2 | 1 | 111.8 | 20.63 | 6.22 | 12.5 | 27.09 | 25.4 | 19.94 | 54.13 | 20.72 | 55.5 | 24.57 | 78.88 | 16.84 | 19.52 | 12.48 | 34.98 |
| Roach | 1106 | 2 | 2 | 1 | 84.06 | 16.33 | 5.31 | 9.29 | 19.43 | 18.59 | 14.95 | 38.76 | 15.43 | 41.51 | 21.58 | 58.52 | 13.89 | 16.29 | 9.65 | 25.96 |
| Roach | 1107 | 2 | 2 | 1 | 103.58 | 19.35 | 5.89 | 11.46 | 24.96 | 22.46 | 18.98 | 47.62 | 18.51 | 52.77 | 26.15 | 74.21 | 17.19 | 18.13 | 10.63 | 32.43 |
| Roach | 1108 | 2 | 2 | 1 | 101.46 | 18.51 | 5.95 | 12.65 | 24.88 | 22.62 | 18.92 | 44.25 | 18.11 | 51.88 | 21.4 | 71.18 | 15.91 | 19.2 | 10.91 | 31.61 |
| Roach | 1109 | 2 | 2 | 1 | 105.47 | 19.93 | 5.52 | 12.26 | 25.2 | 23.97 | 18.95 | 46.88 | 20.37 | 53.42 | 26.34 | 71.5 | 16.79 | 17.76 | 11.73 | 34.62 |
| Roach | 1110 | 2 | 2 | 1 | 88.51 | 16.27 | 4.92 | 10.98 | 21.61 | 21.98 | 16.02 | 42.93 | 14.42 | 44.5 | 18.48 | 64.69 | 11.98 | 14.3 | 9.38 | 26.11 |
| Roach | 1111 | 2 | 2 | 1 | 97.08 | 17.38 | 5.12 | 11.65 | 22.6 | 21.48 | 17.03 | 43.63 | 17.46 | 48.4 | 23.11 | 66.34 | 14.16 | 18.65 | 10.09 | 29.79 |
| Roach | 1112 | 2 | 2 | 1 | 87.29 | 15.18 | 5.28 | 10.38 | 21.26 | 20.79 | 14.63 | 42.52 | 15.1 | 43.82 | 19.49 | 61.08 | 13.54 | 14.83 | 9.18 | 24.96 |
| Roach | 1113 | 2 | 2 | 1 | 104.54 | 19.83 | 6.14 | 12.51 | 26.19 | 24.55 | 18.27 | 51.85 | 17.95 | 54.12 | 23.4 | 75.92 | 13.9 | 17.93 | 11.24 | 32.95 |
| Roach | 1114 | 2 | 2 | 1 | 81.8 | 15.94 | 4.86 | 10.47 | 20.58 | 20.05 | 14.27 | 40.17 | 14.7 | 40.6 | 19.94 | 57.08 | 12.91 | 16.36 | 9.36 | 25.4 |
| Roach | 1115 | 2 | 2 | 1 | 95.9 | 17.72 | 5.53 | 11.51 | 22.39 | 21.89 | 18.04 | 44.31 | 15.99 | 46.54 | 22.33 | 63.05 | 13.96 | 15.8 | 10.84 | 30.16 |
| Roach | 1116 | 2 | 2 | 1 | 77.72 | 13.41 | 4.28 | 8.34 | 18.3 | 18.1 | 13.81 | 36.7 | 15.24 | 38.2 | 17.34 | 52.94 | 12.6 | 14.39 | 7.78 | 21.74 |
| Roach | 1117 | 2 | 2 | 1 | 88.25 | 15.95 | 4.99 | 9.98 | 21.49 | 19.68 | 15.91 | 43.58 | 13.87 | 42.89 | 18.88 | 63.04 | 12.7 | 16.74 | 9.88 | 27.36 |
| Roach | 1118 | 2 | 2 | 1 | 88.3 | 16.72 | 4.97 | 10.99 | 21.45 | 20.25 | 14.75 | 41.74 | 15.08 | 45.77 | 19.56 | 63.07 | 13.8 | 17.63 | 8.71 | 25.28 |
| Roach | 1119 | 2 | 2 | 1 | 89.63 | 16.66 | 5.1 | 11.09 | 22.22 | 21.36 | 14.94 | 42.86 | 16.4 | 47.12 | 18.79 | 63.19 | 13.05 | 15.7 | 9.51 | 27.92 |
| Roach | 1120 | 2 | 2 | 1 | 75.24 | 11.99 | 4.28 | 9.34 | 17.76 | 18.49 | 12.06 | 35.24 | 12.6 | 38.43 | 15.59 | 53.71 | 9.95 | 13.91 | 7.93 | 20.78 |
| Roach | 1121 | 2 | 2 | 1 | 84.73 | 15.22 | 4.67 | 9.26 | 20.46 | 19.99 | 14.23 | 39.18 | 13.78 | 42.45 | 18.88 | 56.5 | 12.06 | 16.63 | 8.58 | 23.15 |
| Roach | 1122 | 2 | 2 | 1 | 78.98 | 14.22 | 4.21 | 8.43 | 18.65 | 18.11 | 15.18 | 37.59 | 14.88 | 38.6 | 19.32 | 56.77 | 12.51 | 16.57 | 8.11 | 23.58 |
| Roach | 1123 | 2 | 2 | 1 | 78.87 | 15.26 | 5 | 10.07 | 19.47 | 18.89 | 13.25 | 39.07 | 13.21 | 39.89 | 17.72 | 52.36 | 9.82 | 14.99 | 8.59 | 23.06 |
| Roach | 1124 | 2 | 2 | 1 | 81.04 | 16.26 | 4.24 | 11.25 | 21.44 | 20.25 | 13.93 | 39.05 | 13.24 | 42.67 | 19.48 | 57.57 | 11.8 | 15.28 | 7.97 | 24.76 |
| Roach | 1125 | 2 | 2 | 1 | 71.64 | 14.36 | 4.6 | 8.6 | 18.51 | 18.19 | 12.31 | 33.09 | 12.73 | 32.03 | 16.04 | 50.03 | 10.94 | 12.58 | 7.83 | 20.89 |
| Roach | 1126 | 2 | 2 | 1 | 66.73 | 11.54 | 3.62 | 8.01 | 17.76 | 17.02 | 11.89 | 32.54 | 11.47 | 35.29 | 15.7 | 44.91 | 10.59 | 13.19 | 6.31 | 17.76 |
| Roach | 1127 | 2 | 2 | 1 | 103.15 | 18.92 | 6.11 | 12.13 | 25.09 | 23.34 | 17.56 | 49.16 | 17.59 | 52.1 | 23.41 | 73.51 | 15.75 | 17.3 | 10.34 | 31.42 |
| Roach | 1128 | 2 | 2 | 1 | 81.67 | 15.9 | 5.26 | 10.16 | 21.92 | 21.42 | 13.9 | 39.3 | 14.3 | 41.25 | 18.04 | 58.58 | 12.15 | 13.5 | 8.89 | 25.29 |
| Roach | 1129 | 2 | 2 | 1 | 77.77 | 14.8 | 4.44 | 10.01 | 19.98 | 19.57 | 14.2 | 38.28 | 13.99 | 39.48 | 17.96 | 57.44 | 11.35 | 13.27 | 7.87 | 22.52 |
| Roach | 1130 | 2 | 2 | 1 | 70.34 | 13.15 | 4.17 | 9.14 | 17.62 | 17.42 | 12.72 | 34.45 | 12.94 | 32.74 | 16.64 | 51.63 | 10.4 | 14.35 | 7.21 | 19.86 |
| Roach | 1101 | 2 | 2 | 2 | 130.68 | 23.71 | 7.11 | 16.92 | 30.27 | 29.76 | 23.37 | 63.04 | 23.97 | 63.99 | 30.62 | 93.51 | 20.27 | 25.45 | 14.31 | 40.79 |
| Roach | 1102 | 2 | 2 | 2 | 112.56 | 21.61 | 6.23 | 13.88 | 26.64 | 26.4 | 19.83 | 52.8 | 20.19 | 52.82 | 26.1 | 81.63 | 16.5 | 23.06 | 11.5 | 33.29 |
| Roach | 1103 | 2 | 2 | 2 | 118.57 | 22 | 7.13 | 13.59 | 27.94 | 25.88 | 19.76 | 54.49 | 20.27 | 60.66 | 26.24 | 83.01 | 16.9 | 21.7 | 11.95 | 35.07 |
| Roach | 1104 | 2 | 2 | 2 | 111.87 | 19.17 | 6.41 | 13.54 | 27.9 | 26.58 | 18.46 | 54.46 | 18.78 | 58.18 | 25.64 | 86.38 | 16.94 | 21.59 | 11.66 | 33.53 |
| Roach | 1105 | 2 | 2 | 2 | 113.42 | 21.19 | 6.18 | 11.91 | 25.26 | 24.76 | 21.1 | 49.79 | 20.71 | 58.42 | 26.34 | 76.4 | 16.93 | 21.21 | 11.68 | 34.09 |
| Roach | 1106 | 2 | 2 | 2 | 84.51 | 16.07 | 4.37 | 10.17 | 20.26 | 20.18 | 16.14 | 40.14 | 15.09 | 41.44 | 20.32 | 58.4 | 14.23 | 17.76 | 8.91 | 26.66 |
| Roach | 1107 | 2 | 2 | 2 | 106.2 | 18.9 | 5.41 | 11.5 | 25.71 | 24.77 | 19.48 | 46.91 | 19.46 | 57.1 | 24.64 | 73.49 | 17.18 | 20.59 | 10.8 | 32.04 |
| Roach | 1108 | 2 | 2 | 2 | 103.91 | 19.33 | 5.66 | 12.2 | 25.8 | 24.48 | 19.45 | 50.7 | 19.89 | 51.4 | 24.09 | 76.72 | 16.19 | 19.05 | 10.66 | 31.92 |
| Roach | 1109 | 2 | 2 | 2 | 106.67 | 20.18 | 5.98 | 13.01 | 26.49 | 26 | 19.12 | 53.12 | 20.45 | 53.76 | 27.2 | 76.84 | 18.41 | 18.21 | 11.62 | 34.42 |
| Roach | 1110 | 2 | 2 | 2 | 88.05 | 16.91 | 5.03 | 10.7 | 21.62 | 21.33 | 14.34 | 43.7 | 15.31 | 43.25 | 18.92 | 64.713 | 12.72 | 16.97 | 9.21 | 28.78 |
| Roach | 1111 | 2 | 2 | 2 | 96.66 | 17.85 | 5.66 | 10.4 | 23.13 | 23.55 | 15.99 | 45.27 | 16.81 | 47.45 | 23.27 | 65.75 | 13.61 | 19.2 | 10.33 | 29.43 |
| Roach | 1112 | 2 | 2 | 2 | 86.64 | 14.33 | 4.57 | 9.4 | 20.43 | 19.92 | 14.41 | 43.87 | 14.3 | 41.48 | 20.46 | 59.35 | 13.26 | 15.89 | 9.2 | 24.72 |
| Roach | 1113 | 2 | 2 | 2 | 104.82 | 19.2 | 5.55 | 11.24 | 25.53 | 25.55 | 17.79 | 52.98 | 18.1 | 53.64 | 22.15 | 74.68 | 14.38 | 17.75 | 11.12 | 32.13 |
| Roach | 1114 | 2 | 2 | 2 | 81.88 | 16.03 | 4.33 | 10.34 | 20.55 | 20.44 | 14.13 | 40.4 | 13.97 | 40.5 | 19.22 | 56.38 | 12.02 | 15.47 | 8.79 | 25.31 |
| Roach | 1115 | 2 | 2 | 2 | 94.11 | 17.65 | 5.83 | 10.93 | 21.69 | 22.65 | 15.7 | 44.66 | 15.5 | 45.03 | 21.4 | 65.41 | 15.26 | 19.3 | 10.33 | 30.08 |
| Roach | 1116 | 2 | 2 | 2 | 75.83 | 13.56 | 4.41 | 8.48 | 18.52 | 18.85 | 12.64 | 36.9 | 12.6 | 37.38 | 16.4 | 54.46 | 11.56 | 15.9 | 8.09 | 22.78 |
| Roach | 1117 | 2 | 2 | 2 | 89.96 | 15.29 | 4.45 | 9.73 | 20.78 | 19.05 | 16.56 | 42.43 | 16.88 | 42.42 | 20.67 | 65.38 | 11.85 | 16.76 | 9.91 | 27.23 |
| Roach | 1118 | 2 | 2 | 2 | 89.77 | 16.39 | 4.78 | 11.27 | 22.51 | 22.37 | 14.46 | 41.22 | 15.88 | 41.9 | 20.66 | 64.03 | 13.18 | 17.75 | 8.53 | 25.68 |
| Roach | 1119 | 2 | 2 | 2 | 89.83 | 16.02 | 5.35 | 9.55 | 22.49 | 22.26 | 15.79 | 46.07 | 15.83 | 45.47 | 19.13 | 65.59 | 12.85 | 18.01 | 9.54 | 27.83 |
| Roach | 1120 | 2 | 2 | 2 | 74.68 | 13.47 | 4.41 | 8.69 | 17.91 | 16.49 | 13.51 | 33.9 | 12.07 | 37.44 | 15.68 | 51.25 | 10.07 | 13.82 | 7.45 | 20.34 |
| Roach | 1121 | 2 | 2 | 2 | 84.14 | 14.67 | 4.79 | 10.11 | 21.06 | 20.21 | 15.13 | 40.72 | 14.16 | 41.23 | 17.55 | 60.39 | 12.81 | 16.61 | 8.66 | 22.97 |
| Roach | 1122 | 2 | 2 | 2 | 80.6 | 14.95 | 4.48 | 8.63 | 19.31 | 19.06 | 15.33 | 37.76 | 15.2 | 38.27 | 18.82 | 56.55 | 12.87 | 17.14 | 8.17 | 23.9 |
| Roach | 1123 | 2 | 2 | 2 | 78.73 | 14.74 | 4.35 | 10.21 | 19.11 | 19.03 | 14 | 39.63 | 13.08 | 39.74 | 18.6 | 56.71 | 12.21 | 16.28 | 8.33 | 23.17 |
| Roach | 1124 | 2 | 2 | 2 | 82.31 | 14.01 | 5.39 | 11.54 | 21.31 | 21.13 | 13.1 | 36.87 | 13.3 | 43.69 | 18.82 | 57.35 | 11.24 | 15.35 | 7.94 | 24.24 |
| Roach | 1125 | 2 | 2 | 2 | 72.53 | 14.14 | 4.31 | 9.13 | 18.24 | 18.01 | 12.36 | 33.03 | 12.31 | 36.43 | 17.02 | 48.28 | 10.49 | 13.94 | 7.4 | 21.03 |
| Roach | 1126 | 2 | 2 | 2 | 65.44 | 12.31 | 3.47 | 8.45 | 17.05 | 16.62 | 10.58 | 31.4 | 11.22 | 34.15 | 15.4 | 46.31 | 11 | 13.19 | 6.71 | 17.35 |
| Roach | 1127 | 2 | 2 | 2 | 103.28 | 18.44 | 5.61 | 12.48 | 24.86 | 23.91 | 16.27 | 51.18 | 17.38 | 51.81 | 23.45 | 72.18 | 14.61 | 18.95 | 10.5 | 30.56 |
| Roach | 1128 | 2 | 2 | 2 | 81.92 | 13.96 | 5.06 | 9.16 | 19.88 | 19.4 | 15.55 | 37.9 | 14.55 | 40.38 | 19.36 | 56.05 | 12 | 15.12 | 8.91 | 24.93 |
| Roach | 1129 | 2 | 2 | 2 | 78.24 | 14.57 | 4.65 | 10.37 | 20.66 | 19.54 | 14.07 | 38.77 | 14.65 | 36.09 | 17.77 | 56.02 | 11.99 | 15.84 | 8.04 | 23.17 |
| Roach | 1130 | 2 | 2 | 2 | 67.86 | 13.89 | 4.21 | 8.8 | 19.12 | 18.74 | 12.21 | 33.27 | 12.68 | 34.65 | 16.52 | 51.91 | 11.01 | 13.85 | 6.95 | 19.79 |
| Roach | 1101 | 2 | 2 | 3 | 130.27 | 23.23 | 7 | 14.77 | 29.85 | 29.55 | 23.43 | 62.52 | 23.11 | 63.75 | 30.68 | 93.48 | 20.82 | 26.84 | 14.2 | 40.09 |
| Roach | 1102 | 2 | 2 | 3 | 112.62 | 19.59 | 6.29 | 12.22 | 25.16 | 25.22 | 20.11 | 51.44 | 19.43 | 56.55 | 23.87 | 79.75 | 16.57 | 22.69 | 11.95 | 33.6 |
| Roach | 1103 | 2 | 2 | 3 | 115.51 | 22.22 | 6.25 | 14.07 | 28.19 | 27.61 | 19.47 | 56.92 | 19.96 | 57.65 | 26.84 | 85.15 | 17.1 | 21.67 | 12.44 | 35.6 |
| Roach | 1104 | 2 | 2 | 3 | 116.84 | 23.05 | 6.55 | 13.71 | 28 | 26.3 | 19.48 | 55.8 | 19.46 | 54.96 | 25.69 | 81.44 | 16.99 | 22.56 | 11.71 | 37.68 |
| Roach | 1105 | 2 | 2 | 3 | 113.29 | 21.67 | 5.44 | 12.91 | 26.6 | 25.63 | 20.27 | 54.63 | 20.04 | 59.25 | 27.08 | 78.24 | 17.59 | 20.18 | 12.26 | 34.52 |
| Roach | 1106 | 2 | 2 | 3 | 83.98 | 16.39 | 4.78 | 9.54 | 19.79 | 18.42 | 15.19 | 37.29 | 15.64 | 41.27 | 22.09 | 54.77 | 12.74 | 17.64 | 8.43 | 25.06 |
| Roach | 1107 | 2 | 2 | 3 | 106.15 | 19.51 | 5.94 | 11.62 | 25.33 | 23.16 | 19.35 | 47.19 | 18.36 | 54.67 | 24.68 | 72.55 | 16.84 | 20.04 | 10.75 | 31.89 |
| Roach | 1108 | 2 | 2 | 3 | 103.31 | 18.44 | 5.93 | 11.96 | 23.4 | 21.86 | 19.88 | 47.13 | 19.632 | 54.6 | 25.36 | 73.37 | 16.21 | 18.04 | 10.88 | 31.53 |
| Roach | 1109 | 2 | 2 | 3 | 106.99 | 19.31 | 5.9 | 13.21 | 25.8 | 25.13 | 19.16 | 52.1 | 19.17 | 55.06 | 26.19 | 75.18 | 18.15 | 19.22 | 11.87 | 34.96 |
| Roach | 1110 | 2 | 2 | 3 | 88.26 | 16.42 | 4.46 | 9.89 | 20.25 | 19.86 | 14.85 | 38.25 | 15.58 | 43.86 | 18.55 | 60.59 | 12.83 | 18.65 | 9.15 | 25.86 |
| Roach | 1111 | 2 | 2 | 3 | 96.58 | 17.4 | 5.46 | 11.26 | 22.33 | 22.71 | 17.13 | 46.91 | 16.92 | 48.87 | 22.05 | 66.94 | 14.44 | 20.05 | 10.41 | 29.33 |
| Roach | 1112 | 2 | 2 | 3 | 86.74 | 15.59 | 5.08 | 10.64 | 22.5 | 22.13 | 14.701 | 42.42 | 14.79 | 42.09 | 18.42 | 61.25 | 13.13 | 16.18 | 8.93 | 25.1 |
| Roach | 1113 | 2 | 2 | 3 | 105.82 | 19.89 | 5.55 | 11.94 | 24.78 | 22.72 | 18.48 | 48.34 | 18.99 | 55.95 | 22.63 | 69.96 | 14.85 | 20.36 | 11.1 | 32.88 |
| Roach | 1114 | 2 | 2 | 3 | 81.22 | 15.44 | 4.22 | 10 | 20.24 | 18.88 | 14.72 | 34.67 | 14.15 | 42.06 | 18.73 | 55.4 | 12.14 | 16.3 | 9.1 | 25.7 |
| Roach | 1115 | 2 | 2 | 3 | 94.89 | 17.43 | 4.99 | 10.63 | 21.81 | 20.81 | 15.48 | 45.37 | 17.94 | 47.09 | 22.46 | 63.4 | 15.6 | 18.77 | 10.66 | 29.37 |
| Roach | 1116 | 2 | 2 | 3 | 74.43 | 13.73 | 4.21 | 9.35 | 18.28 | 18.18 | 13.17 | 38.65 | 13.51 | 37.13 | 15.89 | 52.13 | 11 | 15.53 | 8.08 | 22.61 |
| Roach | 1117 | 2 | 2 | 3 | 89.69 | 16.48 | 5.19 | 9.79 | 20.79 | 19.44 | 15.7 | 41.97 | 15.07 | 46.48 | 20.22 | 63.76 | 12.33 | 16.33 | 9.8 | 28.01 |
| Roach | 1118 | 2 | 2 | 3 | 88.05 | 17.61 | 5.53 | 11.03 | 21.64 | 20.56 | 13.93 | 41.77 | 14.57 | 46.37 | 18.01 | 62.77 | 12.3 | 16.69 | 8.62 | 26.19 |
| Roach | 1119 | 2 | 2 | 3 | 88.4 | 15.71 | 5.54 | 9.21 | 20.97 | 19.84 | 15.13 | 41.11 | 15.29 | 47.82 | 18.88 | 62.58 | 14.05 | 16.25 | 9.29 | 27.77 |
| Roach | 1120 | 2 | 2 | 3 | 74.96 | 12.82 | 3.54 | 8.61 | 17.79 | 16.41 | 13.23 | 32.78 | 12.09 | 37.72 | 15.51 | 50.15 | 11.71 | 14.67 | 7.2 | 20.17 |
| Roach | 1121 | 2 | 2 | 3 | 84.72 | 15.35 | 4.74 | 9.93 | 20.3 | 20.19 | 13.75 | 40.4 | 15.65 | 43.68 | 18.38 | 58.17 | 13.93 | 15.95 | 9.08 | 23.14 |
| Roach | 1122 | 2 | 2 | 3 | 79.89 | 13.42 | 4.3 | 8.23 | 18.71 | 18.12 | 14.74 | 40.02 | 15.42 | 37.57 | 20.09 | 54.98 | 13.44 | 17.56 | 8.53 | 22.9 |
| Roach | 1123 | 2 | 2 | 3 | 79.29 | 13.77 | 4.36 | 9.79 | 18.33 | 17.34 | 13.48 | 34.43 | 12.36 | 40.76 | 18.8 | 55.2 | 11.61 | 14.64 | 8.34 | 22.51 |
| Roach | 1124 | 2 | 2 | 3 | 82.71 | 16.01 | 4.72 | 10.48 | 21.78 | 20.05 | 13.27 | 41.78 | 13.8 | 43.97 | 17.48 | 59.72 | 12.96 | 16.8 | 8.31 | 24 |
| Roach | 1125 | 2 | 2 | 3 | 73.17 | 12.91 | 3.64 | 8.36 | 17.76 | 16.69 | 12.71 | 32.95 | 12.09 | 33.32 | 17.14 | 50.22 | 11.74 | 13.89 | 8.02 | 20.5 |
| Roach | 1126 | 2 | 2 | 3 | 66.16 | 12.11 | 3.4 | 7.81 | 16.4 | 15.44 | 12.03 | 32.01 | 10.98 | 33.59 | 15.26 | 45.53 | 10.09 | 13.27 | 6.69 | 18.17 |
| Roach | 1127 | 2 | 2 | 3 | 100.98 | 19.39 | 5.21 | 12.57 | 25.05 | 24.83 | 17.77 | 50.79 | 17.99 | 48.6 | 25.44 | 75.4 | 13.85 | 18.65 | 11.49 | 31.08 |
| Roach | 1128 | 2 | 2 | 3 | 80.16 | 13.5 | 4.24 | 9.01 | 20.47 | 19.49 | 14.81 | 41.15 | 15.21 | 42.16 | 17.78 | 54.51 | 11.65 | 15.62 | 8.72 | 25.11 |
| Roach | 1129 | 2 | 2 | 3 | 76.58 | 14.78 | 4.15 | 10.28 | 20.14 | 19.87 | 13.77 | 37.92 | 15.6 | 38.4 | 17.97 | 55.26 | 12.33 | 15.24 | 7.78 | 22.72 |
| Roach | 1130 | 2 | 2 | 3 | 70.78 | 12.66 | 4.38 | 9.12 | 17.43 | 17.2 | 11.44 | 34.5 | 12.07 | 35.02 | 16 | 50.45 | 11.42 | 14.59 | 6.95 | 20.05 |
| Roach | 1191 | 3 | 2 | 1 | 118.24 | 22.4 | 6.49 | 12.99 | 28.29 | 26.63 | 23.6 | 59.08 | 21.02 | 62.26 | 27.95 | 86.18 | 18.93 | 24.29 | 11.96 | 36.14 |
| Roach | 1192 | 3 | 2 | 1 | 111.46 | 20.42 | 6.22 | 12.74 | 26.26 | 25.58 | 19.32 | 58.42 | 20.07 | 56.82 | 24.73 | 78.64 | 16.99 | 20.27 | 12.15 | 36.81 |
| Roach | 1193 | 3 | 2 | 1 | 102.25 | 17.38 | 5.27 | 11.64 | 22.63 | 23.07 | 20.11 | 50.28 | 18.9 | 49.64 | 26.71 | 74.24 | 15.87 | 19.43 | 11.25 | 34.1 |
| Roach | 1194 | 3 | 2 | 1 | 91.14 | 16.49 | 4.58 | 9.45 | 22.87 | 22.16 | 16.55 | 45.52 | 16.45 | 44.08 | 22.25 | 64.17 | 14.05 | 18.81 | 9.39 | 27.83 |
| Roach | 1195 | 3 | 2 | 1 | 107.17 | 18.92 | 5.58 | 11.9 | 25.09 | 23.36 | 18.98 | 52.45 | 18.67 | 55.34 | 23.67 | 75.92 | 15.66 | 20.49 | 10.9 | 33.69 |
| Roach | 1196 | 3 | 2 | 1 | 111.49 | 20.38 | 5.81 | 13.59 | 26.48 | 27.39 | 20.55 | 54.4 | 21.26 | 55.3 | 25.58 | 79.82 | 18.31 | 20.56 | 11.97 | 33.37 |
| Roach | 1197 | 3 | 2 | 1 | 129.73 | 25.23 | 8.09 | 17.46 | 33.85 | 30.41 | 23.22 | 62.57 | 23.83 | 67.75 | 30.13 | 94.44 | 20.09 | 23.49 | 13.61 | 43.48 |
| Roach | 1198 | 3 | 2 | 1 | 96.28 | 16.72 | 5.17 | 11.67 | 23.13 | 21.95 | 17.01 | 46.75 | 17.38 | 47.25 | 21.36 | 68.01 | 15.59 | 18.48 | 9.3 | 28.21 |
| Roach | 1199 | 3 | 2 | 1 | 100.83 | 16.88 | 6.16 | 12.35 | 22.69 | 21.55 | 19.41 | 46.13 | 18.43 | 49.6 | 25.37 | 68.7 | 14.96 | 20.56 | 9.96 | 30.86 |
| Roach | 1200 | 3 | 2 | 1 | 110.59 | 20.93 | 6.04 | 12.74 | 26.62 | 25.97 | 19.9 | 53.78 | 18.03 | 55.33 | 24.82 | 80.9 | 16.19 | 22.27 | 11.4 | 33.6 |
| Roach | 1201 | 3 | 2 | 1 | 116.56 | 24 | 7.25 | 13.49 | 28.18 | 27.48 | 21.13 | 54.12 | 20.05 | 56.85 | 27.4 | 82.77 | 18.03 | 23.55 | 12.39 | 38.14 |
| Roach | 1202 | 3 | 2 | 1 | 94.61 | 18.39 | 5.26 | 12.95 | 24.69 | 22.33 | 16.72 | 45.37 | 16.22 | 48.91 | 23.43 | 65.26 | 15.97 | 18.7 | 10.58 | 30.79 |
| Roach | 1203 | 3 | 2 | 1 | 96.37 | 15.85 | 5.25 | 10.81 | 23.34 | 20.21 | 15.56 | 42.51 | 17.71 | 46.85 | 23.01 | 65.84 | 15.39 | 17.41 | 9.03 | 29.07 |
| Roach | 1204 | 3 | 2 | 1 | 101.2 | 17.94 | 6.1 | 12.65 | 25.76 | 25.02 | 19.06 | 48.98 | 16.48 | 48.79 | 21.93 | 71.95 | 14.08 | 21.24 | 9.38 | 31.18 |
| Roach | 1205 | 3 | 2 | 1 | 104.91 | 18.64 | 5.71 | 12.26 | 25.38 | 23.4 | 18.64 | 50.81 | 17.47 | 51.28 | 23.1 | 74.42 | 15.05 | 19.38 | 11.11 | 31.64 |
| Roach | 1206 | 3 | 2 | 1 | 107.76 | 21.52 | 5.28 | 13.44 | 27.39 | 26.01 | 19.72 | 53.49 | 19.14 | 53.17 | 25.55 | 76.78 | 18.04 | 22.11 | 12.47 | 34.86 |
| Roach | 1207 | 3 | 2 | 1 | 96.92 | 20.62 | 5.39 | 11.96 | 25.72 | 24.55 | 19.01 | 45.11 | 18.93 | 47.48 | 22.61 | 69.1 | 16.22 | 19.92 | 10.57 | 31.43 |
| Roach | 1208 | 3 | 2 | 1 | 99.57 | 18.19 | 5.38 | 12.29 | 23.68 | 24.5 | 17.27 | 47.52 | 18.31 | 47.27 | 21.56 | 69.63 | 16.75 | 20.07 | 9.96 | 30.79 |
| Roach | 1209 | 3 | 2 | 1 | 83.26 | 14.38 | 4.83 | 8.57 | 19.95 | 18.32 | 13.7 | 37.56 | 13.84 | 42.18 | 16.85 | 58.84 | 12.41 | 14.48 | 8.49 | 25.07 |
| Roach | 1210 | 3 | 2 | 1 | 94.58 | 16.3 | 5.35 | 10.8 | 22.75 | 21.86 | 17.44 | 48.56 | 16.05 | 48.57 | 20.53 | 62.78 | 13.48 | 17.16 | 9.39 | 26.63 |
| Roach | 1211 | 3 | 2 | 1 | 80.7 | 14.95 | 5.2 | 10.43 | 20.17 | 19.73 | 14.64 | 39.84 | 12.8 | 40.81 | 17.72 | 57.11 | 13.54 | 15.44 | 7.89 | 22.69 |
| Roach | 1212 | 3 | 2 | 1 | 108.21 | 19.99 | 6.63 | 12.48 | 27.21 | 25.77 | 20.2 | 52.03 | 18.75 | 55.67 | 28.32 | 75.84 | 18.3 | 20.04 | 10.89 | 34.47 |
| Roach | 1213 | 3 | 2 | 1 | 108.47 | 19.16 | 5.21 | 12.12 | 23.74 | 22.2 | 19.77 | 51.21 | 17.67 | 49.81 | 24.15 | 74.1 | 15.47 | 19.33 | 11.45 | 32.97 |
| Roach | 1214 | 3 | 2 | 1 | 104.93 | 19.46 | 5.36 | 12.34 | 24.05 | 23.05 | 19.14 | 47.6 | 18.08 | 51.02 | 27.61 | 74.78 | 14.49 | 18.53 | 11.04 | 32.75 |
| Roach | 1215 | 3 | 2 | 1 | 84.46 | 17.62 | 4.86 | 9.04 | 20.32 | 18.64 | 15.24 | 42.36 | 14.13 | 45.7 | 18.66 | 59.46 | 12.62 | 18.24 | 7.82 | 24.44 |
| Roach | 1216 | 3 | 2 | 1 | 106.58 | 21.26 | 6.31 | 13.2 | 26.33 | 24.07 | 21.08 | 51.19 | 20.16 | 52.94 | 25.96 | 77.55 | 18.04 | 20.28 | 11.62 | 33.04 |
| Roach | 1217 | 3 | 2 | 1 | 106.88 | 19.27 | 5.72 | 12.4 | 27.53 | 23.24 | 20.53 | 51.51 | 18.13 | 56.49 | 23.88 | 76.85 | 15.84 | 20.17 | 10.39 | 30.83 |
| Roach | 1218 | 3 | 2 | 1 | 93.59 | 18.2 | 5.85 | 12.22 | 24.47 | 23.64 | 16.41 | 45.35 | 16.07 | 41.78 | 21.9 | 66.01 | 16.35 | 18.68 | 9.83 | 30.39 |
| Roach | 1219 | 3 | 2 | 1 | 100.33 | 19.62 | 5.36 | 12.02 | 24.09 | 25.39 | 19.3 | 49.61 | 18.53 | 49.22 | 23.32 | 71.59 | 14.86 | 19.52 | 10.72 | 30.63 |
| Roach | 1220 | 3 | 2 | 1 | 70.98 | 12.18 | 4.01 | 8.95 | 18.12 | 16.44 | 13.69 | 33.95 | 12.61 | 34.01 | 16.77 | 48.35 | 12.01 | 14.13 | 7.36 | 21.89 |
| Roach | 1191 | 3 | 2 | 2 | 120.61 | 22.71 | 7.2 | 14.83 | 28.23 | 28.13 | 24.4 | 61.72 | 21.01 | 60.12 | 30.19 | 87.34 | 18.25 | 24.64 | 11.94 | 35.85 |
| Roach | 1192 | 3 | 2 | 2 | 111.56 | 20.29 | 6.46 | 12.98 | 27.7 | 24.52 | 19.16 | 48.11 | 19.34 | 52.88 | 24.68 | 76.88 | 16.63 | 21.49 | 11.39 | 36.27 |
| Roach | 1193 | 3 | 2 | 2 | 101.64 | 18.81 | 5.63 | 11.58 | 24.2 | 22.71 | 20.42 | 48.5 | 19.43 | 50.09 | 25.46 | 73.18 | 16.86 | 18.46 | 10.63 | 34.3 |
| Roach | 1194 | 3 | 2 | 2 | 91.75 | 17.13 | 5.87 | 10.6 | 23.57 | 22.88 | 16.66 | 44.45 | 16.5 | 43.22 | 19.71 | 65.51 | 14.99 | 15.91 | 9.44 | 28.24 |
| Roach | 1195 | 3 | 2 | 2 | 106.48 | 19.3 | 6.32 | 13.71 | 24.65 | 23.4 | 17.32 | 46.53 | 18.23 | 52.62 | 24.07 | 77.01 | 15.54 | 17.08 | 10.92 | 33.32 |
| Roach | 1196 | 3 | 2 | 2 | 110.71 | 19.72 | 5.77 | 12.28 | 25.89 | 25.34 | 20.11 | 52.85 | 19.19 | 57.99 | 23.46 | 78.82 | 17.16 | 22.09 | 11.6 | 34.74 |
| Roach | 1197 | 3 | 2 | 2 | 128.23 | 24.38 | 8.14 | 16.25 | 33.54 | 30.88 | 23.6 | 59.63 | 22.83 | 64.84 | 31.11 | 93.45 | 19.87 | 24.81 | 13.94 | 43.99 |
| Roach | 1198 | 3 | 2 | 2 | 96.45 | 17.91 | 5.37 | 11.69 | 23.54 | 23.68 | 17.76 | 45.81 | 16.98 | 46.96 | 20.93 | 72.97 | 13.71 | 17.45 | 9.19 | 28.97 |
| Roach | 1199 | 3 | 2 | 2 | 101.25 | 17.37 | 5.55 | 10.26 | 23.71 | 23.57 | 17.93 | 46.25 | 18.03 | 47.33 | 24.97 | 70.59 | 14.69 | 19.44 | 11.02 | 30.65 |
| Roach | 1200 | 3 | 2 | 2 | 113.56 | 20.17 | 6.11 | 12.67 | 24.92 | 24.21 | 20.58 | 52.36 | 18.91 | 55.76 | 25.11 | 78.56 | 16.43 | 22.24 | 11.42 | 33.73 |
| Roach | 1201 | 3 | 2 | 2 | 116.66 | 21.05 | 6.94 | 14.3 | 28.35 | 27.93 | 20.29 | 58.19 | 20.89 | 61.81 | 27.41 | 82.59 | 18.03 | 22.69 | 13.24 | 37.36 |
| Roach | 1202 | 3 | 2 | 2 | 94.38 | 18.71 | 5.85 | 11.77 | 24.3 | 23.1 | 19.2 | 45.18 | 17.88 | 47.46 | 24.53 | 67.07 | 16.85 | 18.73 | 10.75 | 30.92 |
| Roach | 1203 | 3 | 2 | 2 | 95.08 | 16.81 | 6 | 11.03 | 22.84 | 22.24 | 17.65 | 45.77 | 16.34 | 46.25 | 22.23 | 68.04 | 14.71 | 15.29 | 9.43 | 29.69 |
| Roach | 1204 | 3 | 2 | 2 | 99.97 | 18.53 | 5.6 | 12.07 | 25.03 | 24.6 | 18.67 | 49.47 | 17.11 | 46.81 | 21.7 | 73.63 | 14.39 | 20.22 | 9.661 | 31.17 |
| Roach | 1205 | 3 | 2 | 2 | 103.91 | 17.77 | 6.26 | 12.77 | 24.8 | 24.4 | 18.69 | 52.1 | 17.95 | 52.74 | 22.78 | 72.61 | 16.98 | 16.72 | 10.81 | 31.51 |
| Roach | 1206 | 3 | 2 | 2 | 109.71 | 21 | 5.92 | 12.24 | 26.23 | 26.03 | 20.16 | 52.53 | 20.26 | 53.25 | 23.48 | 77.63 | 17.3 | 22.76 | 12.11 | 36.08 |
| Roach | 1207 | 3 | 2 | 2 | 97.58 | 18.69 | 5.73 | 12 | 24.04 | 22.36 | 18.93 | 45.34 | 18.31 | 49.78 | 23.9 | 71.85 | 15.75 | 19.19 | 10.71 | 31.13 |
| Roach | 1208 | 3 | 2 | 2 | 97.72 | 17.97 | 5.31 | 12.33 | 24.62 | 23.86 | 17.83 | 44.37 | 17.73 | 47.84 | 22.7 | 67.71 | 17.27 | 18.91 | 10.07 | 30.92 |
| Roach | 1209 | 3 | 2 | 2 | 83.24 | 14.56 | 5.06 | 8.53 | 20.11 | 19.39 | 14.18 | 37.42 | 14.24 | 40.94 | 18.79 | 56.98 | 12.69 | 16.73 | 8.47 | 24.35 |
| Roach | 1210 | 3 | 2 | 2 | 93.71 | 16.69 | 5.63 | 10.8 | 23.14 | 22.66 | 17.87 | 45.87 | 16.96 | 46.4 | 21.88 | 65.92 | 14.66 | 17.72 | 9.43 | 27.11 |
| Roach | 1211 | 3 | 2 | 2 | 80.62 | 15.13 | 4.68 | 9.5 | 20.79 | 20.87 | 15.22 | 39.09 | 14.46 | 39.72 | 17.49 | 57.85 | 12.27 | 14.41 | 8.12 | 22.8 |
| Roach | 1212 | 3 | 2 | 2 | 108.01 | 19.13 | 6.72 | 12.51 | 27.11 | 25.26 | 19.73 | 53.55 | 19.71 | 53.86 | 25.44 | 80.88 | 16.58 | 18.35 | 12.54 | 33.88 |
| Roach | 1213 | 3 | 2 | 2 | 108.73 | 19.07 | 6.64 | 11.61 | 24.12 | 23.2 | 17.09 | 50.42 | 18.36 | 54.26 | 24.62 | 75.76 | 16.06 | 19.53 | 11.01 | 32.9 |
| Roach | 1214 | 3 | 2 | 2 | 106.72 | 19.32 | 6.23 | 13.17 | 24.99 | 24.65 | 20.33 | 50.79 | 18.55 | 48.21 | 27.71 | 76.57 | 15.11 | 19.07 | 10.15 | 34.11 |
| Roach | 1215 | 3 | 2 | 2 | 86.28 | 14.03 | 4.62 | 9.42 | 20.29 | 18.46 | 16.23 | 39.18 | 15.5 | 43.28 | 18.19 | 57.94 | 13.33 | 16.94 | 8.51 | 24.63 |
| Roach | 1216 | 3 | 2 | 2 | 107.93 | 19.36 | 5.66 | 11.63 | 24.4 | 23.72 | 21.46 | 48.52 | 19.38 | 53.16 | 25.99 | 75.37 | 17.41 | 20.51 | 11.5 | 33.06 |
| Roach | 1217 | 3 | 2 | 2 | 106.82 | 20.19 | 5.69 | 12.38 | 26.77 | 25.42 | 20.62 | 51.64 | 19.2 | 56.33 | 24.7 | 75.59 | 15.74 | 21.62 | 10.35 | 30.56 |
| Roach | 1218 | 3 | 2 | 2 | 94.34 | 17.36 | 5.87 | 10.19 | 23.44 | 22.95 | 16.27 | 44.25 | 16.88 | 45.03 | 22.37 | 66.29 | 15.18 | 19.21 | 10.09 | 29.96 |
| Roach | 1219 | 3 | 2 | 2 | 98.47 | 19.06 | 5.85 | 12.61 | 24.25 | 24.05 | 18.93 | 45.77 | 19.17 | 49.03 | 24.68 | 69.26 | 15.24 | 19.19 | 10.96 | 30.68 |
| Roach | 1220 | 3 | 2 | 2 | 71.26 | 12.48 | 3.59 | 8.16 | 17.03 | 16.74 | 14.52 | 35.02 | 12.47 | 36.17 | 18.01 | 50.91 | 12.12 | 13.16 | 7.26 | 21.13 |
| Roach | 1191 | 3 | 2 | 3 | 120.32 | 21.51 | 6.97 | 14.3 | 28.36 | 26.63 | 22.6 | 58.8 | 20.77 | 61.32 | 28.04 | 83.87 | 19.36 | 22.6 | 11.87 | 35.3 |
| Roach | 1192 | 3 | 2 | 3 | 111.95 | 20.35 | 5.86 | 13.06 | 26.54 | 24.84 | 19.19 | 52.83 | 19.84 | 57.22 | 27.23 | 78.51 | 18 | 19.19 | 11.83 | 37.23 |
| Roach | 1193 | 3 | 2 | 3 | 100.9 | 18.64 | 6.05 | 11.34 | 23.19 | 21.64 | 19.26 | 47.25 | 18.44 | 50.93 | 25.29 | 70.4 | 16.13 | 19.26 | 11.62 | 33.81 |
| Roach | 1194 | 3 | 2 | 3 | 91.19 | 17.12 | 6.15 | 9.13 | 20.82 | 20.02 | 16.36 | 42.61 | 15.93 | 44.44 | 20.64 | 62.62 | 14.99 | 16.36 | 9.28 | 27.68 |
| Roach | 1195 | 3 | 2 | 3 | 109.11 | 19.4 | 5.31 | 11.4 | 24.8 | 23.42 | 19.39 | 47.42 | 18.5 | 51.17 | 23.24 | 73.43 | 15.59 | 19.39 | 11.03 | 33.07 |
| Roach | 1196 | 3 | 2 | 3 | 111.76 | 25.23 | 6.8 | 13.58 | 26.82 | 24.17 | 21.84 | 52.8 | 19.15 | 56.38 | 26.19 | 79.19 | 17.63 | 21.84 | 11.4 | 35.38 |
| Roach | 1197 | 3 | 2 | 3 | 128.48 | 25.8 | 7.62 | 15.24 | 32.75 | 32.24 | 22.67 | 59.35 | 24.18 | 60.88 | 29.14 | 91.31 | 22.23 | 22.67 | 13.82 | 43.79 |
| Roach | 1198 | 3 | 2 | 3 | 95.52 | 17.52 | 4.6 | 10.76 | 22.99 | 22.86 | 17.82 | 45.62 | 18.03 | 47.31 | 20.82 | 72.27 | 15.19 | 17.82 | 9.85 | 28.73 |
| Roach | 1199 | 3 | 2 | 3 | 100.41 | 18 | 5.39 | 11.97 | 23.44 | 21.94 | 18.22 | 46.49 | 17.28 | 45.27 | 23.23 | 71.02 | 16.17 | 18.22 | 10.02 | 29.93 |
| Roach | 1200 | 3 | 2 | 3 | 111.93 | 19.96 | 6.93 | 12.29 | 25.96 | 25.65 | 19.15 | 52.23 | 18.05 | 54.81 | 24.87 | 75.61 | 16.52 | 19.15 | 11.31 | 33.57 |
| Roach | 1201 | 3 | 2 | 3 | 115.44 | 21.99 | 6.78 | 12.86 | 27.14 | 24.32 | 20.8 | 55.39 | 20.1 | 56.11 | 26.7 | 83.2 | 18.346 | 20.8 | 12.77 | 37.98 |
| Roach | 1202 | 3 | 2 | 3 | 94.32 | 19.25 | 5.49 | 12.16 | 23.39 | 21.47 | 19.27 | 44.31 | 17.78 | 47.51 | 25.03 | 66.71 | 16.73 | 19.27 | 10.52 | 31.11 |
| Roach | 1203 | 3 | 2 | 3 | 96.13 | 18.14 | 5.55 | 10.85 | 22.37 | 20.21 | 19.05 | 43.35 | 17.9 | 47.63 | 23.88 | 66.37 | 14.85 | 19.05 | 8.82 | 31.02 |
| Roach | 1204 | 3 | 2 | 3 | 100.17 | 19.09 | 6.79 | 11.06 | 23.58 | 22.37 | 19.29 | 47.36 | 16.99 | 48.56 | 22.24 | 72.33 | 14.25 | 19.29 | 9.52 | 31.38 |
| Roach | 1205 | 3 | 2 | 3 | 100.53 | 18.65 | 5.59 | 11.48 | 25.37 | 25.16 | 18.99 | 51.61 | 17.68 | 52.27 | 24 | 71.36 | 14.01 | 18.99 | 13.29 | 31.26 |
| Roach | 1206 | 3 | 2 | 3 | 110.75 | 20.06 | 6.05 | 13.52 | 27.75 | 25.433 | 20.44 | 50.51 | 21.21 | 53.68 | 25.71 | 77.38 | 17.93 | 20.44 | 12.16 | 35.21 |
| Roach | 1207 | 3 | 2 | 3 | 96.53 | 18.57 | 6.12 | 13.15 | 23.58 | 22.55 | 19.72 | 45.7 | 17.93 | 45.58 | 25.23 | 69.31 | 16.38 | 19.72 | 10.43 | 31.02 |
| Roach | 1208 | 3 | 2 | 3 | 99.13 | 18.06 | 5.06 | 11.34 | 24.02 | 22.35 | 16.1 | 46.37 | 18.43 | 50.17 | 21.76 | 69.27 | 14.93 | 16.1 | 10.26 | 31.02 |
| Roach | 1209 | 3 | 2 | 3 | 83.84 | 14.5 | 4.26 | 9.06 | 19.48 | 18.23 | 16.02 | 36.07 | 14.31 | 43.41 | 17.92 | 57.67 | 12.82 | 16.02 | 8.53 | 24.65 |
| Roach | 1210 | 3 | 2 | 3 | 94.16 | 16.82 | 4.97 | 10.99 | 22.97 | 22.53 | 16.19 | 46.78 | 15.27 | 50.8 | 19.27 | 64.38 | 13.73 | 16.19 | 9.17 | 25.34 |
| Roach | 1211 | 3 | 2 | 3 | 80.78 | 14.94 | 4.62 | 9.03 | 20.07 | 19.3 | 14.63 | 39.3 | 17.97 | 41.15 | 14.39 | 54.54 | 12.99 | 14.63 | 7.92 | 22.5 |
| Roach | 1212 | 3 | 2 | 3 | 111.26 | 19.6 | 6.1 | 12.12 | 25.93 | 23.76 | 21.46 | 45.28 | 19.84 | 56.61 | 26.75 | 75.33 | 18.3 | 21.46 | 11.4 | 33.73 |
| Roach | 1213 | 3 | 2 | 3 | 106.41 | 19.19 | 5.88 | 11.59 | 25.12 | 23.71 | 18.68 | 48.24 | 17.83 | 50.01 | 24.49 | 77.92 | 16.07 | 18.68 | 11.42 | 33.42 |
| Roach | 1214 | 3 | 2 | 3 | 102.57 | 20.09 | 6.43 | 11.94 | 24.06 | 22.23 | 18.5 | 46.83 | 18.78 | 50.99 | 27.81 | 73.74 | 15.17 | 18.5 | 10.46 | 34.01 |
| Roach | 1215 | 3 | 2 | 3 | 87.69 | 15.44 | 5.23 | 10.76 | 20.6 | 20.56 | 15.63 | 42.28 | 14.92 | 42.4 | 18.91 | 58.96 | 13.12 | 15.63 | 8.34 | 24.58 |
| Roach | 1216 | 3 | 2 | 3 | 108.36 | 18.12 | 6.15 | 11.65 | 25.71 | 25.47 | 21.35 | 50.86 | 20.6 | 53.85 | 25.46 | 75.83 | 18.95 | 21.35 | 11.63 | 32.64 |
| Roach | 1217 | 3 | 2 | 3 | 108.94 | 19.83 | 5.99 | 12.13 | 25.4 | 25.18 | 20.76 | 52.98 | 18.99 | 56 | 23.43 | 74.88 | 15.67 | 20.76 | 9.97 | 30.87 |
| Roach | 1218 | 3 | 2 | 3 | 94.54 | 18.59 | 5.5 | 10.6 | 23.36 | 21.99 | 18.1 | 46.39 | 17.13 | 45.5 | 22.09 | 67.34 | 16.46 | 18.1 | 9.96 | 30.88 |
| Roach | 1219 | 3 | 2 | 3 | 101.73 | 18.48 | 5.53 | 11.36 | 24.94 | 23.65 | 19.11 | 47.34 | 16.23 | 49.73 | 24.96 | 69.76 | 14.92 | 19.11 | 10.43 | 30.69 |
| Roach | 1220 | 3 | 2 | 3 | 71.93 | 13.49 | 4.25 | 8.01 | 17.75 | 16.43 | 13.55 | 35.85 | 12.14 | 35.72 | 16.48 | 53.88 | 10.8 | 13.55 | 7.86 | 21.57 |
| Roach | 1001 | 1 | 3 | 1 | 92.53 | 15.25 | 4.99 | 10.5 | 21.35 | 21.06 | 16.79 | 41.57 | 16.42 | 43.85 | 21.95 | 63.32 | 13.37 | 20.01 | 9.48 | 28.61 |
| Roach | 1002 | 1 | 3 | 1 | 113.6 | 20.6 | 6.28 | 13.48 | 28.2 | 26.91 | 21.38 | 54.98 | 20.62 | 56.47 | 26.98 | 81.41 | 15.9 | 20.48 | 12.21 | 36.49 |
| Roach | 1003 | 1 | 3 | 1 | 135.65 | 23.2 | 7.56 | 15.33 | 30.45 | 29.88 | 24.2 | 61.66 | 23.08 | 63.83 | 30.38 | 92.77 | 20.5 | 27.56 | 14.12 | 42.88 |
| Roach | 1004 | 1 | 3 | 1 | 84.36 | 15.95 | 4.6 | 10.69 | 20.45 | 20 | 15.96 | 39.26 | 15.41 | 42.7 | 20.01 | 59.06 | 14.16 | 16.3 | 8.63 | 25.62 |
| Roach | 1005 | 1 | 3 | 1 | 117.45 | 20.41 | 6.83 | 14.37 | 28.25 | 28.25 | 20.24 | 56.68 | 20.37 | 59.85 | 24.73 | 84.38 | 17.46 | 20.51 | 12.45 | 38.26 |
| Roach | 1006 | 1 | 3 | 1 | 132.14 | 23.28 | 7.37 | 16.05 | 31 | 30.39 | 23.69 | 60.68 | 23.73 | 65.51 | 30.11 | 93.69 | 19.86 | 26.83 | 14.16 | 42.62 |
| Roach | 1007 | 1 | 3 | 1 | 92.72 | 17.26 | 5.9 | 10.89 | 21.79 | 21.35 | 16.48 | 43.62 | 15.28 | 44.91 | 19.59 | 62.81 | 13.41 | 17.89 | 9.98 | 28.86 |
| Roach | 1008 | 1 | 3 | 1 | 79.24 | 14.76 | 5.42 | 9.36 | 19.61 | 19.29 | 13.94 | 37.93 | 12.85 | 38.67 | 18.48 | 54.56 | 11.55 | 17 | 8.06 | 24.79 |
| Roach | 1009 | 1 | 3 | 1 | 89.01 | 15.3 | 5.14 | 10.85 | 21.87 | 20.96 | 16.36 | 43.19 | 16.01 | 44.95 | 20.52 | 65.06 | 13.19 | 17.42 | 9.62 | 27.78 |
| Roach | 1010 | 1 | 3 | 1 | 93.1 | 17.63 | 5.17 | 11.39 | 22.91 | 22.25 | 17.5 | 45.05 | 15.94 | 46.58 | 20.6 | 67.66 | 14.2 | 17.58 | 10.39 | 30.03 |
| Roach | 1011 | 1 | 3 | 1 | 83.14 | 15.73 | 4.93 | 10.52 | 20.41 | 20.53 | 15.21 | 38.11 | 13.75 | 40.11 | 19.53 | 57.52 | 12.62 | 16.31 | 8.12 | 25.34 |
| Roach | 1012 | 1 | 3 | 1 | 99.47 | 17.73 | 5.9 | 11.57 | 23.68 | 22.02 | 18.38 | 46.86 | 17.38 | 50.71 | 22.24 | 77 | 16.03 | 19.71 | 9.93 | 31.23 |
| Roach | 1013 | 1 | 3 | 1 | 92 | 16.16 | 5.18 | 10.55 | 21.59 | 20.83 | 17.07 | 43.79 | 16.62 | 45.01 | 20.81 | 63.83 | 14.22 | 20.14 | 9.32 | 27.64 |
| Roach | 1014 | 1 | 3 | 1 | 128.6 | 22.87 | 8.1 | 15.89 | 29.71 | 29.46 | 23.32 | 61.29 | 23.12 | 63.94 | 31.49 | 89.44 | 19 | 26.34 | 13.41 | 39.83 |
| Roach | 1015 | 1 | 3 | 1 | 128.42 | 22.97 | 7.59 | 15.15 | 29.56 | 28.63 | 23.7 | 61.63 | 22.82 | 64.17 | 26.69 | 91.74 | 19.01 | 25.12 | 13.46 | 40.04 |
| Roach | 1016 | 1 | 3 | 1 | 88.53 | 16.06 | 5.71 | 11.13 | 22.1 | 20.81 | 16.5 | 41.83 | 15.39 | 44.64 | 20.28 | 60.96 | 13.14 | 18.65 | 9.92 | 29.11 |
| Roach | 1017 | 1 | 3 | 1 | 72.13 | 13.18 | 4.57 | 9.34 | 18.61 | 18.13 | 12.22 | 35.68 | 13.1 | 36.72 | 17.77 | 52.21 | 11.25 | 15.49 | 7 | 21.04 |
| Roach | 1018 | 1 | 3 | 1 | 109.4 | 20.52 | 6.44 | 12.64 | 25.58 | 25.4 | 20.3 | 51.07 | 19.69 | 53.1 | 25.22 | 77.43 | 17.42 | 23.02 | 11.61 | 35.34 |
| Roach | 1019 | 1 | 3 | 1 | 92.76 | 16.31 | 5.51 | 11.02 | 22.43 | 22.98 | 15.05 | 45.28 | 15.8 | 48.19 | 20.72 | 66.63 | 14.95 | 17.15 | 10 | 29.22 |
| Roach | 1020 | 1 | 3 | 1 | 118.16 | 21.48 | 5.98 | 15.16 | 28.28 | 27.08 | 21.14 | 54.95 | 20.59 | 59.33 | 25.29 | 85.97 | 18.15 | 22.06 | 12.05 | 38.01 |
| Roach | 1021 | 1 | 3 | 1 | 111.96 | 20.58 | 6.13 | 12.99 | 26.36 | 25.81 | 20.87 | 52.18 | 19.41 | 54.88 | 25.96 | 78.48 | 17.88 | 22.79 | 11.69 | 34.15 |
| Roach | 1022 | 1 | 3 | 1 | 95.4 | 17.52 | 5.44 | 12.03 | 23.26 | 22.81 | 17.39 | 43.87 | 14.9 | 47.68 | 19.58 | 68.32 | 12.83 | 18.64 | 9.32 | 27.37 |
| Roach | 1023 | 1 | 3 | 1 | 82.9 | 14.52 | 4.67 | 8.82 | 19.61 | 19.23 | 14.95 | 39.24 | 13.56 | 42.22 | 16.94 | 57.61 | 12.81 | 17.5 | 8.22 | 23.98 |
| Roach | 1024 | 1 | 3 | 1 | 96.87 | 18.4 | 6.09 | 12.22 | 24.27 | 23.56 | 18.85 | 44.82 | 17.68 | 45.48 | 23.84 | 67.32 | 15.87 | 20.5 | 10.7 | 31.77 |
| Roach | 1025 | 1 | 3 | 1 | 95.21 | 16.56 | 5.45 | 11.73 | 22.41 | 23.33 | 18.18 | 46.04 | 17.61 | 46.97 | 23.78 | 66.54 | 15.3 | 19.68 | 10.08 | 31.51 |
| Roach | 1026 | 1 | 3 | 1 | 121.4 | 20.63 | 7.31 | 14.36 | 28.99 | 29.41 | 21.61 | 59.66 | 19.06 | 60.5 | 25.07 | 84.59 | 16.29 | 23.42 | 12.17 | 37.46 |
| Roach | 1027 | 1 | 3 | 1 | 100.56 | 19.94 | 6.03 | 11.78 | 24.21 | 22.9 | 18.74 | 48.78 | 17.03 | 51.74 | 23.13 | 72.85 | 15.38 | 20.93 | 10.76 | 31.84 |
| Roach | 1028 | 1 | 3 | 1 | 117.12 | 20.98 | 6.96 | 13.81 | 26.08 | 25.35 | 21.59 | 54.6 | 20.37 | 56.18 | 24.89 | 83.05 | 16.34 | 23.82 | 11.58 | 35.39 |
| Roach | 1029 | 1 | 3 | 1 | 108.71 | 19.91 | 5.93 | 12.35 | 24.57 | 24.23 | 18.29 | 52.61 | 18.31 | 54.49 | 23 | 77.77 | 16.85 | 20.23 | 11.04 | 32.72 |
| Roach | 1030 | 1 | 3 | 1 | 94.74 | 17.97 | 5.96 | 11.5 | 23.16 | 23.11 | 17.02 | 46.98 | 16.52 | 52.99 | 21.23 | 66.85 | 14.38 | 18.9 | 10.08 | 31.73 |
| Roach | 1001 | 1 | 3 | 2 | 93.03 | 16.19 | 5.35 | 10.3 | 21.41 | 21.41 | 16.6 | 40.83 | 16.43 | 44.07 | 21.58 | 62.89 | 14.04 | 21 | 9.18 | 28.51 |
| Roach | 1002 | 1 | 3 | 2 | 113.92 | 22.21 | 7.3 | 14.47 | 28.08 | 28.67 | 21.79 | 55.83 | 21.01 | 57.42 | 26.82 | 81.55 | 17.39 | 18.75 | 11.85 | 36.92 |
| Roach | 1003 | 1 | 3 | 2 | 134.83 | 23.09 | 7.75 | 15.23 | 31.09 | 29.59 | 23.77 | 61.28 | 24.24 | 62.77 | 29.82 | 91.68 | 20.52 | 27.47 | 14.35 | 42.82 |
| Roach | 1004 | 1 | 3 | 2 | 84.79 | 15.43 | 5.55 | 10.52 | 20.75 | 20.64 | 15.4 | 41.3 | 14.72 | 41.6 | 20.8 | 58.91 | 13.44 | 16.84 | 8.47 | 25.75 |
| Roach | 1005 | 1 | 3 | 2 | 117.25 | 21.21 | 6.98 | 15.07 | 27.96 | 29.07 | 19.8 | 56.09 | 20.25 | 58.23 | 25.63 | 83.53 | 17.27 | 21.16 | 12.33 | 38.21 |
| Roach | 1006 | 1 | 3 | 2 | 131.15 | 23.15 | 8.16 | 15.56 | 31.33 | 30.67 | 23.73 | 62 | 23.43 | 65.23 | 30.33 | 92.37 | 19.12 | 27.02 | 14.45 | 42.14 |
| Roach | 1007 | 1 | 3 | 2 | 92.54 | 16.32 | 5.98 | 10.87 | 22.04 | 21.24 | 15.88 | 44.28 | 15.65 | 44.64 | 18.99 | 62.65 | 13.57 | 17.79 | 9.38 | 29.07 |
| Roach | 1008 | 1 | 3 | 2 | 78.95 | 14.91 | 4.72 | 9.36 | 19.43 | 19.2 | 14.32 | 37.06 | 13.5 | 38.62 | 17.52 | 54.82 | 11.64 | 16.83 | 7.88 | 24.68 |
| Roach | 1009 | 1 | 3 | 2 | 90.02 | 16 | 5.34 | 10.55 | 21.84 | 21.66 | 16.24 | 42.48 | 15.6 | 44.16 | 20.49 | 63.44 | 14.06 | 17.12 | 9.32 | 27.85 |
| Roach | 1010 | 1 | 3 | 2 | 93.13 | 17.4 | 5.91 | 11.13 | 23.24 | 22.32 | 17.65 | 43.9 | 15.84 | 47.54 | 21.56 | 66 | 14.06 | 19.6 | 10.14 | 30.23 |
| Roach | 1011 | 1 | 3 | 2 | 84.14 | 15.01 | 4.94 | 9.26 | 19.91 | 19.62 | 15.46 | 37.45 | 14.71 | 39.59 | 19.03 | 57.43 | 12.79 | 16.25 | 8.2 | 25.34 |
| Roach | 1012 | 1 | 3 | 2 | 99.68 | 17.58 | 6.67 | 11.48 | 23.61 | 22.72 | 19.55 | 48.38 | 18.34 | 51.01 | 22.48 | 71.04 | 14.53 | 19.2 | 9.75 | 30.16 |
| Roach | 1013 | 1 | 3 | 2 | 91.82 | 16.52 | 5.84 | 10.44 | 21.67 | 20.95 | 17.15 | 42.63 | 16.58 | 44.37 | 20.35 | 62.99 | 14.29 | 19.15 | 9.41 | 27.87 |
| Roach | 1014 | 1 | 3 | 2 | 128.84 | 24.66 | 8.46 | 15.81 | 30.91 | 29.63 | 22.74 | 61.64 | 23.43 | 63.6 | 30.69 | 89.04 | 20.48 | 27.31 | 13.37 | 38.97 |
| Roach | 1015 | 1 | 3 | 2 | 130.63 | 23.18 | 8.08 | 15.46 | 31.48 | 29.61 | 24.16 | 60.43 | 22.41 | 64.54 | 27.36 | 90.39 | 18.86 | 25.72 | 13.1 | 40.13 |
| Roach | 1016 | 1 | 3 | 2 | 89.05 | 16.23 | 6.05 | 10.61 | 21.83 | 21.27 | 16.93 | 42.9 | 16.39 | 44.39 | 20.33 | 61.44 | 13.4 | 18.84 | 9.94 | 29.33 |
| Roach | 1017 | 1 | 3 | 2 | 73.31 | 12.87 | 4.37 | 9.48 | 18.47 | 18.28 | 12.29 | 34.97 | 13.34 | 36.1 | 16.2 | 52.12 | 11.44 | 15.22 | 6.98 | 21.44 |
| Roach | 1018 | 1 | 3 | 2 | 110.26 | 20.53 | 5.9 | 13 | 25.33 | 24.64 | 20.25 | 51 | 20.46 | 52.73 | 25.24 | 76.36 | 17.92 | 23.32 | 11.32 | 35.18 |
| Roach | 1019 | 1 | 3 | 2 | 93.48 | 16.49 | 6.16 | 11.35 | 23.37 | 22.64 | 16.22 | 46.03 | 16.47 | 48.68 | 21.05 | 66.74 | 15.29 | 16.89 | 9.95 | 28.94 |
| Roach | 1020 | 1 | 3 | 2 | 118.68 | 20.74 | 6.58 | 14.79 | 28.22 | 26.46 | 21.79 | 54.87 | 20.62 | 59.49 | 26.52 | 84.18 | 17.74 | 22.06 | 12.06 | 38.21 |
| Roach | 1021 | 1 | 3 | 2 | 112.18 | 19.71 | 6.29 | 13.32 | 26.44 | 25.71 | 20.89 | 53.33 | 20.55 | 55.78 | 25.82 | 76.85 | 18.22 | 22.85 | 11.76 | 34.13 |
| Roach | 1022 | 1 | 3 | 2 | 94.93 | 18.25 | 6.29 | 12.09 | 23.44 | 23.91 | 16.1 | 45.71 | 16.52 | 47.43 | 20.23 | 68.64 | 13.85 | 17.53 | 9.29 | 27.36 |
| Roach | 1023 | 1 | 3 | 2 | 82.85 | 15 | 5.02 | 9.47 | 19.38 | 18.85 | 14.32 | 38.55 | 13.35 | 41.66 | 17.31 | 57.23 | 12.89 | 17 | 7.85 | 23.2 |
| Roach | 1024 | 1 | 3 | 2 | 97.69 | 18.35 | 5.98 | 11.89 | 24.05 | 23.15 | 18.39 | 43.43 | 18.51 | 44.44 | 24.7 | 66.1 | 16.36 | 19.4 | 10.54 | 31.78 |
| Roach | 1025 | 1 | 3 | 2 | 96.09 | 16.25 | 6.08 | 12.18 | 22.79 | 23.21 | 17.66 | 45.82 | 17.98 | 47.12 | 23.69 | 66.91 | 14.08 | 19.22 | 10.21 | 30.91 |
| Roach | 1026 | 1 | 3 | 2 | 121.16 | 20.68 | 7.09 | 13.7 | 28.27 | 27.97 | 20.01 | 59.94 | 18.94 | 60.15 | 24.75 | 84.26 | 17.18 | 22.23 | 11.94 | 37.5 |
| Roach | 1027 | 1 | 3 | 2 | 102.43 | 19.05 | 6.54 | 12.27 | 24.52 | 24.84 | 18.92 | 48.87 | 15.81 | 51.49 | 23.09 | 72.07 | 16.13 | 21.51 | 10.46 | 31.57 |
| Roach | 1028 | 1 | 3 | 2 | 117.37 | 19.23 | 7.48 | 12.89 | 27.02 | 25.65 | 22.07 | 56.06 | 20.01 | 56.99 | 24.61 | 82.86 | 18.35 | 24.7 | 11.64 | 35.22 |
| Roach | 1029 | 1 | 3 | 2 | 108.14 | 19.61 | 6.13 | 13.19 | 24.18 | 23.67 | 18.45 | 52.63 | 19.1 | 55.1 | 24.36 | 75.97 | 16.21 | 21.2 | 10.87 | 33.01 |
| Roach | 1030 | 1 | 3 | 2 | 95.42 | 17.97 | 5.8 | 11.17 | 23.22 | 23.22 | 16.99 | 47.14 | 16.6 | 49.75 | 21.89 | 66.69 | 14.58 | 17.94 | 10.13 | 31.49 |
| Roach | 1001 | 1 | 3 | 3 | 93.61 | 15.79 | 4.9 | 10.19 | 20.9 | 20.37 | 16.31 | 41.73 | 16.5 | 45.38 | 21.45 | 63.91 | 13.15 | 20.48 | 9.63 | 28.64 |
| Roach | 1002 | 1 | 3 | 3 | 113.3 | 20.71 | 7 | 14.4 | 28.21 | 27.83 | 21.27 | 55.09 | 20.52 | 57.44 | 27.55 | 81.92 | 17.45 | 19.89 | 12.17 | 36.67 |
| Roach | 1003 | 1 | 3 | 3 | 135.16 | 21.91 | 7.38 | 14.55 | 30.31 | 29.21 | 24.75 | 61.26 | 22.99 | 64.11 | 30.99 | 92.78 | 19.61 | 27.83 | 14.37 | 43.1 |
| Roach | 1004 | 1 | 3 | 3 | 84.95 | 15.88 | 5.18 | 10.21 | 20.33 | 20.04 | 15.87 | 41.25 | 14.82 | 42.56 | 21.12 | 59.39 | 12.93 | 17.63 | 8.37 | 25.86 |
| Roach | 1005 | 1 | 3 | 3 | 117.56 | 20.58 | 7.17 | 15.44 | 28.09 | 28.45 | 19.65 | 57.7 | 20.13 | 59.86 | 26.22 | 84.88 | 17.92 | 22.72 | 12.5 | 37.85 |
| Roach | 1006 | 1 | 3 | 3 | 131.43 | 23.26 | 7.5 | 16.3 | 31.32 | 29.69 | 22.9 | 61.48 | 23.46 | 65.82 | 29.78 | 93.93 | 18.77 | 26.64 | 13.97 | 42.38 |
| Roach | 1007 | 1 | 3 | 3 | 92.28 | 16.23 | 5.63 | 10.24 | 21.97 | 22.46 | 16.52 | 44.37 | 15.57 | 45.12 | 18.91 | 62.58 | 13.41 | 17.11 | 9.32 | 29.28 |
| Roach | 1008 | 1 | 3 | 3 | 79.07 | 14.82 | 4.97 | 10.01 | 19.4 | 18.45 | 14.47 | 35.76 | 13.73 | 37.76 | 18.14 | 55.14 | 12.19 | 16.78 | 7.68 | 24.63 |
| Roach | 1009 | 1 | 3 | 3 | 89.61 | 16.22 | 5.5 | 10.62 | 21.51 | 20.72 | 17.35 | 43.47 | 16.44 | 44.37 | 21.43 | 63.9 | 14.38 | 18.41 | 8.99 | 27.96 |
| Roach | 1010 | 1 | 3 | 3 | 93.36 | 16.38 | 5.96 | 11.52 | 23.49 | 22.91 | 17.46 | 45.23 | 16.66 | 47.7 | 20.65 | 66.43 | 13.64 | 17.52 | 10.07 | 29.83 |
| Roach | 1011 | 1 | 3 | 3 | 83.78 | 14.58 | 5.07 | 10.31 | 20.05 | 19.52 | 15.57 | 38.33 | 14.6 | 39.01 | 19.42 | 57.43 | 13.09 | 16.93 | 8.3 | 25.33 |
| Roach | 1012 | 1 | 3 | 3 | 100.42 | 16.57 | 5.99 | 11.21 | 23.72 | 22.86 | 18.81 | 48.16 | 17.52 | 50.72 | 22.27 | 71.35 | 14.94 | 20.35 | 9.57 | 30.89 |
| Roach | 1013 | 1 | 3 | 3 | 92.01 | 15.97 | 5.58 | 10.2 | 21.79 | 21.11 | 17.18 | 44.11 | 16.66 | 45.62 | 20.89 | 63.8 | 14.05 | 20.56 | 8.91 | 27.57 |
| Roach | 1014 | 1 | 3 | 3 | 127.8 | 23.58 | 8.33 | 15.71 | 30.59 | 30.04 | 23.01 | 60.67 | 22.55 | 64.26 | 29.2 | 90.81 | 18.31 | 26.24 | 12.79 | 39.53 |
| Roach | 1015 | 1 | 3 | 3 | 129.54 | 25.09 | 8.58 | 16.02 | 30.49 | 29.98 | 23.94 | 61.61 | 22.85 | 63.16 | 27.71 | 90.35 | 19.35 | 24.58 | 13.44 | 40.31 |
| Roach | 1016 | 1 | 3 | 3 | 88.81 | 16.42 | 6.12 | 10.78 | 21.31 | 21 | 16.68 | 42.15 | 15.37 | 45 | 19.62 | 61.79 | 13.57 | 18.26 | 9.68 | 28.91 |
| Roach | 1017 | 1 | 3 | 3 | 73.33 | 13.13 | 4.22 | 9.23 | 17.99 | 17.32 | 13.04 | 34.81 | 13.5 | 36.47 | 17.85 | 51.48 | 11.59 | 15.15 | 7.03 | 21.19 |
| Roach | 1018 | 1 | 3 | 3 | 110.33 | 20.29 | 5.87 | 12.63 | 25.33 | 24.85 | 20.02 | 50.61 | 19.65 | 52.88 | 25.86 | 76.97 | 17.1 | 24.4 | 11.43 | 34.95 |
| Roach | 1019 | 1 | 3 | 3 | 93.48 | 16.43 | 6.07 | 11.13 | 22.69 | 22.65 | 16.24 | 45.97 | 15.85 | 48.7 | 20.65 | 66.86 | 14.72 | 17.1 | 9.47 | 29.33 |
| Roach | 1020 | 1 | 3 | 3 | 118.65 | 20.83 | 6.57 | 14.95 | 27.59 | 26 | 21.18 | 54.9 | 20.6 | 59.52 | 25.66 | 84.04 | 18 | 22.16 | 11.74 | 38.43 |
| Roach | 1021 | 1 | 3 | 3 | 111.56 | 19.48 | 5.88 | 13.41 | 26.06 | 26.06 | 20.48 | 53.43 | 19.6 | 55.46 | 25.06 | 77.81 | 17.73 | 22.61 | 11.78 | 34.24 |
| Roach | 1022 | 1 | 3 | 3 | 95.28 | 17.5 | 5.55 | 12.33 | 23.47 | 22.96 | 17.09 | 45.2 | 15.85 | 46.96 | 20.02 | 68.71 | 14.42 | 17.88 | 8.67 | 27.56 |
| Roach | 1023 | 1 | 3 | 3 | 83.01 | 14.71 | 4.77 | 9.49 | 19.61 | 19.37 | 14.04 | 38.99 | 13.43 | 42.35 | 16.75 | 57.41 | 12.21 | 18.01 | 7.96 | 23.09 |
| Roach | 1024 | 1 | 3 | 3 | 97 | 18.71 | 6.17 | 11.91 | 24.52 | 23.41 | 18.76 | 42.86 | 18.15 | 44 | 24.84 | 67.03 | 15.83 | 19.95 | 10.46 | 31.55 |
| Roach | 1025 | 1 | 3 | 3 | 95.58 | 16.81 | 5.77 | 10.84 | 22.64 | 22.87 | 17.84 | 46.28 | 17.14 | 47.01 | 24.14 | 66.55 | 14.43 | 19.73 | 10.1 | 31.24 |
| Roach | 1026 | 1 | 3 | 3 | 121.55 | 20.24 | 7.46 | 14.07 | 29.15 | 28.26 | 21.37 | 59.4 | 19.25 | 62.18 | 25.48 | 85.14 | 17.04 | 24.35 | 11.57 | 36.81 |
| Roach | 1027 | 1 | 3 | 3 | 101.84 | 19.06 | 6.18 | 12.15 | 24.43 | 23.72 | 18.62 | 49.45 | 16 | 51.74 | 23.61 | 72.14 | 15.47 | 22.27 | 10.55 | 31.55 |
| Roach | 1028 | 1 | 3 | 3 | 117.43 | 19.58 | 6.9 | 12.98 | 26.9 | 25.62 | 22.39 | 55.75 | 20.36 | 56.43 | 24.49 | 82.01 | 17.11 | 25.28 | 11.39 | 35.07 |
| Roach | 1029 | 1 | 3 | 3 | 108.45 | 18.91 | 6.01 | 12.53 | 25.3 | 25.3 | 18.38 | 52.04 | 17.95 | 53.5 | 24.75 | 76.46 | 16.25 | 21.79 | 10.41 | 32.33 |
| Roach | 1030 | 1 | 3 | 3 | 95.88 | 17.95 | 6.34 | 11.48 | 23.31 | 22.73 | 18.13 | 46.96 | 16.46 | 50.08 | 20.32 | 66.73 | 14.05 | 18.13 | 9.75 | 31.79 |
| Roach | 1101 | 2 | 3 | 1 | 130.78 | 21.11 | 7.28 | 15.48 | 29.86 | 28.52 | 23.49 | 62.23 | 22.95 | 65.94 | 29.36 | 91 | 20.35 | 23.15 | 13.25 | 39.66 |
| Roach | 1102 | 2 | 3 | 1 | 110.58 | 19.48 | 6.76 | 12.59 | 25.9 | 25 | 20.44 | 51.75 | 19.85 | 55.04 | 25.1 | 78.26 | 15.86 | 22.16 | 10.88 | 33.19 |
| Roach | 1103 | 2 | 3 | 1 | 115.46 | 21.19 | 6.88 | 14.34 | 28.27 | 27.49 | 20.64 | 54.84 | 19.95 | 58.28 | 25.2 | 83.78 | 16.36 | 20.78 | 12.04 | 35.03 |
| Roach | 1104 | 2 | 3 | 1 | 116.13 | 21.63 | 6.53 | 13.87 | 26.52 | 24.47 | 20.75 | 54.29 | 18.9 | 56.94 | 23.55 | 81.26 | 16.45 | 22.71 | 11.44 | 37.78 |
| Roach | 1105 | 2 | 3 | 1 | 110.61 | 19.73 | 6.96 | 12.53 | 27.14 | 24.39 | 20.76 | 52.23 | 19.35 | 55.99 | 23.04 | 78 | 16.57 | 19.4 | 11.42 | 34.55 |
| Roach | 1106 | 2 | 3 | 1 | 85.62 | 15.8 | 4.96 | 9.84 | 20.15 | 18.73 | 16.55 | 39.27 | 15.25 | 42.17 | 19.36 | 60.1 | 12.95 | 17.65 | 8.13 | 26 |
| Roach | 1107 | 2 | 3 | 1 | 105.54 | 18.71 | 6.68 | 12.08 | 25.39 | 23.5 | 19.58 | 50.14 | 19.28 | 54.86 | 24.84 | 75.1 | 15.49 | 19.83 | 10.22 | 31.09 |
| Roach | 1108 | 2 | 3 | 1 | 102.84 | 18.3 | 5.91 | 11.6 | 24.32 | 23.33 | 20.93 | 50.33 | 18.38 | 52.16 | 23.97 | 73.72 | 15.44 | 17.18 | 10.29 | 31.55 |
| Roach | 1109 | 2 | 3 | 1 | 107.43 | 19.68 | 6.33 | 13.31 | 26.79 | 23.5 | 20.01 | 51.5 | 19.81 | 52.28 | 24.24 | 74.03 | 17.58 | 20.11 | 11.23 | 34.42 |
| Roach | 1110 | 2 | 3 | 1 | 87.32 | 15.62 | 5.12 | 9.71 | 20.63 | 19.8 | 14.16 | 41.72 | 14.79 | 43.04 | 17.26 | 62.72 | 12.02 | 16.67 | 8.58 | 26.15 |
| Roach | 1111 | 2 | 3 | 1 | 94.9 | 18.12 | 5.24 | 10.39 | 22.57 | 21.51 | 16.64 | 44.32 | 17.13 | 47.2 | 21.56 | 66.93 | 14.39 | 18.36 | 9.8 | 29.44 |
| Roach | 1112 | 2 | 3 | 1 | 86.26 | 15.63 | 5.51 | 10.14 | 20.7 | 20.35 | 15.42 | 42.93 | 14.94 | 44.14 | 17.59 | 60.68 | 11.77 | 16.05 | 8.77 | 24.74 |
| Roach | 1113 | 2 | 3 | 1 | 103.72 | 18.42 | 6.45 | 11.97 | 25.21 | 23.66 | 19.14 | 50.41 | 18.9 | 54.25 | 22.13 | 74.74 | 13.87 | 20.36 | 10.65 | 32.35 |
| Roach | 1114 | 2 | 3 | 1 | 81.54 | 14.47 | 4.6 | 9.99 | 19.94 | 19.1 | 14.58 | 38.11 | 14.27 | 41.93 | 17.66 | 55.97 | 11.57 | 16.67 | 8.12 | 24.63 |
| Roach | 1115 | 2 | 3 | 1 | 94.25 | 16.81 | 6.2 | 10.33 | 22.22 | 22.53 | 16.63 | 44.37 | 16.57 | 45.12 | 21.36 | 64.08 | 14.55 | 17.46 | 10.05 | 29.35 |
| Roach | 1116 | 2 | 3 | 1 | 76.07 | 13.52 | 4.5 | 8.48 | 18.3 | 18.49 | 13.59 | 36.83 | 14 | 39.28 | 16.33 | 53.91 | 11.28 | 13.95 | 7.24 | 21.83 |
| Roach | 1117 | 2 | 3 | 1 | 88.87 | 16.33 | 5.59 | 9.72 | 20.17 | 19.5 | 17.21 | 43.6 | 15.69 | 45.3 | 18.93 | 63.57 | 11.85 | 17.77 | 9.19 | 27.8 |
| Roach | 1118 | 2 | 3 | 1 | 88.27 | 16.34 | 4.53 | 10.99 | 21.75 | 21.6 | 15.71 | 40.95 | 15.42 | 45.68 | 17.55 | 62.57 | 13.12 | 16.11 | 8.23 | 25.49 |
| Roach | 1119 | 2 | 3 | 1 | 87.61 | 15.8 | 5.32 | 10.21 | 21.6 | 20.59 | 15.33 | 43.57 | 14.28 | 46.19 | 18.79 | 62.06 | 12.29 | 16.43 | 9 | 27.19 |
| Roach | 1120 | 2 | 3 | 1 | 73.83 | 13.24 | 4.25 | 9.18 | 18.01 | 17.57 | 12.91 | 33.57 | 11.24 | 38.56 | 15.7 | 51.07 | 9.76 | 14.34 | 6.64 | 19.97 |
| Roach | 1121 | 2 | 3 | 1 | 84.57 | 14.91 | 5.03 | 10.86 | 20.16 | 20.1 | 15.12 | 40.88 | 14.35 | 43.25 | 17.76 | 58.67 | 12.54 | 16.63 | 8.18 | 22.97 |
| Roach | 1122 | 2 | 3 | 1 | 79.15 | 14.19 | 4.78 | 9.24 | 18.89 | 19.14 | 15.63 | 37.33 | 15.29 | 37.96 | 19.61 | 57.26 | 11.7 | 16.71 | 7.85 | 22.51 |
| Roach | 1123 | 2 | 3 | 1 | 78.75 | 14.17 | 4.76 | 9.68 | 18.94 | 19.28 | 14.23 | 38.64 | 12.71 | 40.37 | 18.04 | 54.94 | 11.58 | 15.15 | 8.19 | 22.46 |
| Roach | 1124 | 2 | 3 | 1 | 81.07 | 14.58 | 5.11 | 10.21 | 20.2 | 19.72 | 14.28 | 36.64 | 14.06 | 42.71 | 16.34 | 58.86 | 12.14 | 13.62 | 7.85 | 23.69 |
| Roach | 1125 | 2 | 3 | 1 | 69.64 | 12.8 | 4.12 | 8.93 | 17.69 | 18.18 | 12.81 | 35.04 | 12.22 | 36.27 | 14.93 | 49.12 | 9.94 | 13.84 | 7.31 | 19.72 |
| Roach | 1126 | 2 | 3 | 1 | 65.23 | 11.82 | 4.28 | 7.44 | 16.96 | 17.49 | 10.9 | 33.03 | 11.97 | 34.1 | 14.05 | 46.01 | 9.46 | 12.97 | 6.43 | 17.72 |
| Roach | 1127 | 2 | 3 | 1 | 101.88 | 18.21 | 7.01 | 12.53 | 24.52 | 24.19 | 18.09 | 48.3 | 17.33 | 51.75 | 23.05 | 74.22 | 14.19 | 18.73 | 10.16 | 30.7 |
| Roach | 1128 | 2 | 3 | 1 | 80.67 | 15.17 | 5.32 | 9.97 | 21.45 | 19.53 | 15.5 | 39.94 | 13.78 | 41.94 | 18.68 | 56.89 | 11.7 | 14.97 | 7.73 | 24.66 |
| Roach | 1129 | 2 | 3 | 1 | 77 | 14.89 | 4.93 | 10.15 | 19.5 | 19.46 | 14.37 | 38.58 | 14.56 | 39.32 | 16.03 | 54.78 | 11.28 | 14.38 | 7.71 | 22.26 |
| Roach | 1130 | 2 | 3 | 1 | 70.97 | 12.66 | 4.84 | 8.66 | 17.84 | 17.41 | 11.83 | 34.05 | 12.45 | 35.71 | 15.97 | 49.12 | 10.15 | 13.56 | 6.57 | 18.59 |
| Roach | 1101 | 2 | 3 | 2 | 130.63 | 22.22 | 8.24 | 15.52 | 29.8 | 29.26 | 22.97 | 60.93 | 23.31 | 65.17 | 29.85 | 91.63 | 20.37 | 22.98 | 14 | 40.18 |
| Roach | 1102 | 2 | 3 | 2 | 109.83 | 19.24 | 6.53 | 12.17 | 25.9 | 24.85 | 20.22 | 50.99 | 20.36 | 53.08 | 24.2 | 78.28 | 15.8 | 21.59 | 10.47 | 33.26 |
| Roach | 1103 | 2 | 3 | 2 | 115.65 | 21.75 | 7.36 | 14.42 | 28.12 | 27.36 | 20.52 | 55.51 | 19.96 | 59.13 | 25.65 | 83.2 | 15.95 | 18.97 | 11.99 | 34.7 |
| Roach | 1104 | 2 | 3 | 2 | 116.17 | 21.96 | 6.53 | 14.36 | 27.51 | 25.69 | 20.97 | 54.19 | 18.19 | 57.8 | 23.51 | 82.07 | 15.48 | 21.17 | 11.03 | 37.21 |
| Roach | 1105 | 2 | 3 | 2 | 110.75 | 20.68 | 6.53 | 13.77 | 27.16 | 24.98 | 20.94 | 52.44 | 20.14 | 56.22 | 24.08 | 78.77 | 16.58 | 20.21 | 11.48 | 34.44 |
| Roach | 1106 | 2 | 3 | 2 | 84.72 | 15.97 | 5.13 | 10.05 | 20.26 | 18.85 | 17.23 | 39.12 | 15.98 | 41.88 | 19.89 | 58.19 | 13.2 | 17.03 | 8.4 | 26.07 |
| Roach | 1107 | 2 | 3 | 2 | 105.5 | 19.33 | 6.78 | 12.2 | 25.62 | 24.24 | 19.42 | 49.97 | 20.17 | 53.63 | 24.08 | 74.35 | 16.04 | 19.98 | 9.81 | 31.19 |
| Roach | 1108 | 2 | 3 | 2 | 103.82 | 18.27 | 6.31 | 11.42 | 23.94 | 22.31 | 20.29 | 49.94 | 17.88 | 51.8 | 23.78 | 73.59 | 16.4 | 18.97 | 10.06 | 31.39 |
| Roach | 1109 | 2 | 3 | 2 | 105.74 | 19.66 | 7.26 | 13.46 | 25.81 | 24.07 | 19.01 | 50.76 | 20.08 | 52.97 | 24.55 | 75.14 | 16.19 | 20.25 | 10.99 | 33.98 |
| Roach | 1110 | 2 | 3 | 2 | 87.22 | 15.88 | 5.4 | 9.65 | 21.19 | 19.62 | 14.36 | 40.8 | 14.93 | 42.99 | 18.5 | 62.5 | 12.71 | 17.52 | 8.15 | 26.04 |
| Roach | 1111 | 2 | 3 | 2 | 95.49 | 17.3 | 5.73 | 10.84 | 23.33 | 22.07 | 17.32 | 45.13 | 17.85 | 46.64 | 21.65 | 65.99 | 14.39 | 18.58 | 9.81 | 28.98 |
| Roach | 1112 | 2 | 3 | 2 | 86.17 | 15.23 | 5.47 | 9.67 | 20.46 | 19.57 | 15.33 | 41.09 | 14.64 | 44.42 | 17.55 | 61.26 | 11.43 | 15.95 | 8.79 | 24.44 |
| Roach | 1113 | 2 | 3 | 2 | 103.68 | 18.62 | 6.38 | 11.97 | 26.03 | 23.48 | 18.7 | 48.88 | 18.11 | 53.2 | 22.65 | 75.19 | 14.04 | 20.23 | 10.82 | 31.88 |
| Roach | 1114 | 2 | 3 | 2 | 81.82 | 14.18 | 5.1 | 10.06 | 20.36 | 19 | 15.18 | 37.61 | 14.1 | 41.18 | 19.12 | 56.74 | 11.56 | 16.01 | 8.17 | 24.97 |
| Roach | 1115 | 2 | 3 | 2 | 94 | 17.18 | 6.24 | 11.21 | 23.24 | 22.44 | 16.19 | 43.38 | 16.4 | 45.88 | 20.95 | 63.61 | 13.78 | 17.04 | 9.63 | 29.71 |
| Roach | 1116 | 2 | 3 | 2 | 75.95 | 13.23 | 4.82 | 8.87 | 18.68 | 18.56 | 13.38 | 37 | 13.03 | 37.84 | 16.67 | 53.4 | 10.9 | 15.08 | 7.19 | 21.77 |
| Roach | 1117 | 2 | 3 | 2 | 88.51 | 15.57 | 5.54 | 10.09 | 21.19 | 19.85 | 17.14 | 40.7 | 15.62 | 44.64 | 19.06 | 63.25 | 12.37 | 18.95 | 9.3 | 27.64 |
| Roach | 1118 | 2 | 3 | 2 | 88.03 | 16.14 | 5.47 | 10.87 | 21.51 | 20.9 | 14.47 | 40.96 | 15.13 | 44.93 | 17.24 | 63.27 | 12.44 | 18.15 | 8.5 | 25.23 |
| Roach | 1119 | 2 | 3 | 2 | 87.86 | 16.04 | 5.36 | 10.25 | 21.91 | 20.16 | 16.72 | 42.26 | 15.3 | 45.46 | 18.63 | 62.73 | 12.51 | 17.64 | 8.98 | 27.59 |
| Roach | 1120 | 2 | 3 | 2 | 74.79 | 13.33 | 4.6 | 9.26 | 18.45 | 17.01 | 13.7 | 34.47 | 11.83 | 35.91 | 15.16 | 50.7 | 9.94 | 14.95 | 6.99 | 19.82 |
| Roach | 1121 | 2 | 3 | 2 | 83.8 | 15.43 | 5.55 | 10.73 | 20.85 | 20.53 | 15.32 | 41.29 | 14.39 | 46.64 | 18.41 | 57.95 | 12.22 | 16.7 | 7.72 | 22.81 |
| Roach | 1122 | 2 | 3 | 2 | 79.59 | 13.97 | 4.93 | 9.21 | 18.92 | 18.33 | 16.49 | 37.13 | 15.73 | 38.76 | 19.14 | 55.78 | 12.08 | 16.06 | 7.56 | 22.47 |
| Roach | 1123 | 2 | 3 | 2 | 77.37 | 13.59 | 4.73 | 9.58 | 19.31 | 19.78 | 14.75 | 38.41 | 13.99 | 39.09 | 16.91 | 54.55 | 11.81 | 16.07 | 7.53 | 23.04 |
| Roach | 1124 | 2 | 3 | 2 | 81.1 | 14.08 | 5.51 | 10.28 | 20.54 | 19.86 | 14.62 | 38.38 | 14.05 | 41.73 | 17.05 | 58.51 | 12.12 | 14.61 | 7.91 | 23.55 |
| Roach | 1125 | 2 | 3 | 2 | 70.92 | 13.22 | 4.57 | 8.9 | 18.11 | 17.62 | 13.11 | 33.74 | 12.05 | 36.04 | 15.72 | 50.46 | 10.06 | 13.72 | 6.94 | 19.2 |
| Roach | 1126 | 2 | 3 | 2 | 65.11 | 12.33 | 4.19 | 7.61 | 17.02 | 16.39 | 12.59 | 32.83 | 10.49 | 34.82 | 15.17 | 46.1 | 9.92 | 12.34 | 6.14 | 17.53 |
| Roach | 1127 | 2 | 3 | 2 | 101.28 | 17.98 | 7.12 | 13.36 | 25.49 | 24.63 | 17.84 | 49.16 | 17.54 | 50.85 | 22.6 | 73.77 | 14.14 | 20.42 | 10.98 | 31.09 |
| Roach | 1128 | 2 | 3 | 2 | 81.93 | 15.24 | 5.22 | 8.9 | 21.13 | 19.78 | 15.82 | 39.72 | 14.02 | 40.68 | 18.23 | 55.48 | 11.5 | 15.2 | 8.1 | 24.79 |
| Roach | 1129 | 2 | 3 | 2 | 77.14 | 15.86 | 5.09 | 10.71 | 19.99 | 19.51 | 14.38 | 38.51 | 15 | 39.73 | 17.39 | 55.65 | 11.17 | 14.94 | 7.62 | 22.22 |
| Roach | 1130 | 2 | 3 | 2 | 71.14 | 12.12 | 4.62 | 9.08 | 17.69 | 16.84 | 12.39 | 34.55 | 12.8 | 35.78 | 15.8 | 49.67 | 9.93 | 14.73 | 6.43 | 18.25 |
| Roach | 1101 | 2 | 3 | 3 | 130 | 21.54 | 7.29 | 15.11 | 29.46 | 28.28 | 23.82 | 61.77 | 23.56 | 64.89 | 28.95 | 92.68 | 19.58 | 23.97 | 13.14 | 39.97 |
| Roach | 1102 | 2 | 3 | 3 | 111.06 | 19.72 | 6.74 | 12.8 | 26.67 | 25.58 | 21 | 51.69 | 19.97 | 54.56 | 25.15 | 80.28 | 14.92 | 22.33 | 11.02 | 33.4 |
| Roach | 1103 | 2 | 3 | 3 | 116.49 | 21.75 | 7.63 | 14.45 | 28.05 | 26.37 | 20.24 | 53.91 | 20.75 | 59.33 | 25.22 | 84.64 | 17 | 21.17 | 11.64 | 34.56 |
| Roach | 1104 | 2 | 3 | 3 | 116.51 | 21.41 | 6.52 | 14.72 | 27.57 | 25.97 | 20.2 | 55.25 | 19.01 | 57.41 | 23.48 | 83.52 | 16.41 | 22.67 | 10.69 | 37.63 |
| Roach | 1105 | 2 | 3 | 3 | 110.75 | 20.73 | 6.47 | 12.71 | 26.98 | 25.04 | 21 | 53.38 | 18.09 | 57.03 | 23.2 | 78.76 | 15.67 | 19.59 | 11.73 | 34.66 |
| Roach | 1106 | 2 | 3 | 3 | 84.39 | 15.7 | 4.61 | 10.29 | 20.45 | 19.48 | 16.26 | 39.15 | 15.31 | 42.28 | 20.35 | 59.67 | 12.92 | 16.21 | 8.1 | 25.94 |
| Roach | 1107 | 2 | 3 | 3 | 105.74 | 19.09 | 6.45 | 12.58 | 26.35 | 23.96 | 20.06 | 49 | 19.13 | 51.64 | 23.74 | 74.21 | 16.58 | 21.47 | 10.44 | 31.46 |
| Roach | 1108 | 2 | 3 | 3 | 104.29 | 18.57 | 6.04 | 11.72 | 24.58 | 23.04 | 20.81 | 49.67 | 17.38 | 52.95 | 24.59 | 75.32 | 16.15 | 17.7 | 10.26 | 31.68 |
| Roach | 1109 | 2 | 3 | 3 | 105.78 | 19.79 | 5.89 | 11.96 | 26.59 | 24.04 | 19.86 | 52.35 | 20.32 | 53.88 | 24.7 | 77.13 | 17.22 | 19.26 | 11.38 | 34.43 |
| Roach | 1110 | 2 | 3 | 3 | 87.54 | 15.96 | 5.53 | 9.92 | 21.6 | 20.13 | 15.13 | 41.82 | 15.42 | 44.08 | 17.89 | 62.51 | 12.04 | 17.53 | 8.57 | 26.16 |
| Roach | 1111 | 2 | 3 | 3 | 95.83 | 17.73 | 5.43 | 10.4 | 22.04 | 21.04 | 17.26 | 43.16 | 17.28 | 47.45 | 20.82 | 66.99 | 13.75 | 19.95 | 9.51 | 29.35 |
| Roach | 1112 | 2 | 3 | 3 | 86.17 | 15.72 | 5.48 | 10.11 | 21.2 | 19.89 | 15.03 | 40.97 | 15.4 | 44.76 | 17.77 | 61.28 | 11.46 | 15.78 | 8.88 | 24.7 |
| Roach | 1113 | 2 | 3 | 3 | 102.93 | 18.62 | 6.94 | 11.92 | 25.72 | 23.72 | 19.63 | 48.61 | 18.54 | 54.74 | 22.67 | 74.51 | 13.9 | 20.31 | 10.42 | 32.51 |
| Roach | 1114 | 2 | 3 | 3 | 81.93 | 14.54 | 5.26 | 10.16 | 19.59 | 19.09 | 14.93 | 37.7 | 14.56 | 39.88 | 18.36 | 56.22 | 11.22 | 16.41 | 8.16 | 25.14 |
| Roach | 1115 | 2 | 3 | 3 | 94.44 | 17.52 | 6.01 | 10.75 | 22.55 | 21.98 | 17.07 | 43.35 | 16.84 | 46.7 | 22.4 | 65.02 | 13.2 | 18.57 | 9.98 | 29.66 |
| Roach | 1116 | 2 | 3 | 3 | 75.91 | 13.35 | 4.27 | 9.15 | 18.97 | 18.71 | 13.67 | 36.6 | 13.69 | 38.5 | 17.75 | 53.7 | 12.22 | 15.74 | 7.72 | 21.72 |
| Roach | 1117 | 2 | 3 | 3 | 88.55 | 15.7 | 5.78 | 9.94 | 21.03 | 20.95 | 16.95 | 42.78 | 15.71 | 45.43 | 19.54 | 63.81 | 12.68 | 17.71 | 9.28 | 27.93 |
| Roach | 1118 | 2 | 3 | 3 | 87.93 | 16.46 | 5.18 | 11.31 | 21.68 | 21.12 | 14.91 | 41.29 | 15.66 | 44.65 | 17.96 | 63.6 | 12.2 | 16.77 | 8.08 | 25.48 |
| Roach | 1119 | 2 | 3 | 3 | 88.27 | 15.87 | 5.26 | 10.67 | 22.56 | 20.93 | 16.21 | 44 | 15.14 | 45.93 | 17.53 | 62.97 | 12.75 | 17.4 | 9.16 | 27.58 |
| Roach | 1120 | 2 | 3 | 3 | 74.72 | 13.32 | 4.95 | 8.57 | 17.81 | 16.91 | 13.06 | 35.02 | 11.92 | 36.8 | 14.32 | 51.42 | 10.7 | 15.11 | 7.07 | 20.56 |
| Roach | 1121 | 2 | 3 | 3 | 83.77 | 14.86 | 5.33 | 10.27 | 20.69 | 20 | 15.93 | 41.44 | 14.34 | 43.53 | 17.81 | 57.69 | 12.98 | 15.23 | 8.32 | 23.08 |
| Roach | 1122 | 2 | 3 | 3 | 79.69 | 14.07 | 4.3 | 9.49 | 19.35 | 18.48 | 15.08 | 37.84 | 16.36 | 39.37 | 17.79 | 57.06 | 12.05 | 16.98 | 7.94 | 23.1 |
| Roach | 1123 | 2 | 3 | 3 | 78.71 | 13.65 | 5.02 | 9.72 | 19.12 | 18 | 14.45 | 38.86 | 13.45 | 40.44 | 17.73 | 54.82 | 12.11 | 15.21 | 8.11 | 22.47 |
| Roach | 1124 | 2 | 3 | 3 | 81.7 | 14.45 | 5.51 | 10.49 | 20.82 | 19.86 | 14.56 | 38.65 | 13.98 | 41.3 | 17.39 | 58.28 | 11.51 | 15.26 | 7.5 | 23.87 |
| Roach | 1125 | 2 | 3 | 3 | 70.79 | 12.96 | 4.43 | 8.41 | 17.67 | 17.12 | 11.94 | 35.07 | 12.89 | 36.51 | 14.28 | 49.72 | 10.31 | 13.79 | 7.24 | 19.87 |
| Roach | 1126 | 2 | 3 | 3 | 66.26 | 11.57 | 4.45 | 7.46 | 16.78 | 16.78 | 12.41 | 33.55 | 11.74 | 35.03 | 14.77 | 46.19 | 10.7 | 12.91 | 6.15 | 17.9 |
| Roach | 1127 | 2 | 3 | 3 | 102.03 | 17.76 | 6.92 | 12.25 | 24.71 | 23.93 | 17.88 | 48.89 | 17.55 | 53.89 | 23.27 | 75.47 | 14.61 | 19.84 | 10.52 | 30.93 |
| Roach | 1128 | 2 | 3 | 3 | 81.62 | 15.57 | 5 | 10.14 | 21.06 | 19.59 | 15.72 | 40.62 | 14.24 | 41.33 | 17.34 | 57.65 | 11.5 | 15.6 | 7.91 | 25.2 |
| Roach | 1129 | 2 | 3 | 3 | 77.74 | 14.44 | 5.11 | 10.19 | 20.41 | 19.73 | 14.42 | 39.12 | 14.46 | 39.58 | 16.5 | 54.94 | 11.78 | 15.05 | 7.59 | 22.52 |
| Roach | 1130 | 2 | 3 | 3 | 71.13 | 12.22 | 4.69 | 8.6 | 17.48 | 17.18 | 11.95 | 34.31 | 13.23 | 35.22 | 15.84 | 49.93 | 10.26 | 15.32 | 6.74 | 18.88 |
| Roach | 1191 | 3 | 3 | 1 | 120.34 | 20.97 | 7.21 | 14.41 | 28.67 | 27.43 | 24.02 | 57.28 | 21.33 | 60.47 | 28.89 | 87.85 | 18.56 | 25.93 | 11.66 | 35.68 |
| Roach | 1192 | 3 | 3 | 1 | 110.67 | 19.98 | 5.54 | 13.96 | 26.51 | 26.46 | 19.57 | 53.21 | 19.46 | 55.29 | 24.46 | 79.22 | 17.95 | 20.63 | 10.78 | 36.59 |
| Roach | 1193 | 3 | 3 | 1 | 101.83 | 18.6 | 6.3 | 11.94 | 24.78 | 24.78 | 20.53 | 48.78 | 19.05 | 52.01 | 24.36 | 73.19 | 14.13 | 18.64 | 10.89 | 34.22 |
| Roach | 1194 | 3 | 3 | 1 | 90.16 | 16.55 | 6.78 | 10.56 | 22.61 | 22.61 | 16.87 | 43.82 | 15.02 | 46.49 | 19.52 | 64.99 | 14.02 | 16.79 | 9.04 | 28.23 |
| Roach | 1195 | 3 | 3 | 1 | 106.32 | 18.83 | 6.3 | 12.14 | 25.58 | 24.65 | 18.34 | 50.44 | 18.01 | 53.38 | 21.97 | 77.61 | 15.98 | 19.69 | 10.36 | 33.56 |
| Roach | 1196 | 3 | 3 | 1 | 112.67 | 20.01 | 6.52 | 13.44 | 27.37 | 26.67 | 20.31 | 53.8 | 18.6 | 58.5 | 22.56 | 80.91 | 16.46 | 20.86 | 10.82 | 34.96 |
| Roach | 1197 | 3 | 3 | 1 | 125.12 | 24.56 | 8 | 16.88 | 33.38 | 33 | 23.33 | 62.46 | 22.77 | 64.44 | 29.76 | 94.29 | 19.66 | 21.8 | 13.24 | 44.86 |
| Roach | 1198 | 3 | 3 | 1 | 94.64 | 17.55 | 5.18 | 11.41 | 23.05 | 23.04 | 17.22 | 47.34 | 16.16 | 48.98 | 19.57 | 69.51 | 14.15 | 17.53 | 8.68 | 28.86 |
| Roach | 1199 | 3 | 3 | 1 | 102.39 | 18.25 | 6.41 | 11.66 | 22.88 | 22.32 | 18.55 | 45.42 | 17.88 | 47.82 | 22.53 | 69.89 | 16.07 | 19.65 | 9.7 | 30.02 |
| Roach | 1200 | 3 | 3 | 1 | 111.87 | 20.24 | 6.91 | 13.81 | 26.58 | 27.03 | 19.88 | 54.44 | 18.39 | 58.76 | 23.13 | 82.19 | 16.15 | 21.78 | 10.15 | 33.78 |
| Roach | 1201 | 3 | 3 | 1 | 114.03 | 21.23 | 6.65 | 13.91 | 28.21 | 26.07 | 20.31 | 53.76 | 19.93 | 61.25 | 26.76 | 83.86 | 18.35 | 22.28 | 12.16 | 38.36 |
| Roach | 1202 | 3 | 3 | 1 | 95.04 | 17.69 | 5.18 | 11.82 | 24.09 | 24.11 | 18.02 | 44.71 | 16.09 | 48.91 | 22.9 | 66.48 | 14.49 | 18.81 | 10.26 | 30.66 |
| Roach | 1203 | 3 | 3 | 1 | 93.54 | 17.11 | 5.66 | 11.46 | 22.26 | 23.1 | 17.03 | 45.37 | 17.24 | 47.62 | 22.02 | 66.37 | 15.14 | 16.64 | 8.73 | 29.63 |
| Roach | 1204 | 3 | 3 | 1 | 100.88 | 18.04 | 6.24 | 12.28 | 25.47 | 25.46 | 19.11 | 49 | 18.33 | 52.42 | 21.41 | 72.14 | 14.55 | 20.5 | 9.3 | 31.12 |
| Roach | 1205 | 3 | 3 | 1 | 103.06 | 19.56 | 6.6 | 12.2 | 24.97 | 23.8 | 18.8 | 48.23 | 18.06 | 49.13 | 22.98 | 73.59 | 14.59 | 19.61 | 10.63 | 31.1 |
| Roach | 1206 | 3 | 3 | 1 | 110.52 | 20.56 | 6.64 | 12.73 | 26.35 | 26.35 | 20.05 | 52.48 | 19.1 | 55.98 | 23.38 | 76.41 | 16.7 | 22.03 | 11.59 | 35.39 |
| Roach | 1207 | 3 | 3 | 1 | 97.62 | 20.13 | 6.68 | 12.48 | 24.68 | 23.81 | 19.19 | 46.08 | 19.11 | 50.58 | 24 | 70.65 | 14.97 | 19.27 | 9.84 | 31.23 |
| Roach | 1208 | 3 | 3 | 1 | 98.45 | 18.11 | 5.74 | 12.66 | 24.43 | 23.45 | 18.32 | 47.21 | 17.83 | 49.18 | 21.27 | 68.3 | 16.29 | 19.88 | 9.25 | 30.88 |
| Roach | 1209 | 3 | 3 | 1 | 82.8 | 14.67 | 4.95 | 9.86 | 20.38 | 21.3 | 15.27 | 39.8 | 14.41 | 41.92 | 16.16 | 58.37 | 12.32 | 16.47 | 7.94 | 25.19 |
| Roach | 1210 | 3 | 3 | 1 | 93.33 | 16.4 | 6.06 | 11.84 | 23.18 | 24.57 | 17.56 | 46.3 | 15.78 | 50.95 | 20.98 | 65.24 | 14.33 | 16.69 | 9.22 | 26.57 |
| Roach | 1211 | 3 | 3 | 1 | 80.74 | 14.83 | 5.56 | 9.49 | 20.2 | 19.07 | 14.49 | 39.05 | 14.16 | 42.39 | 16.4 | 58.86 | 12.38 | 16.73 | 7.23 | 22.53 |
| Roach | 1212 | 3 | 3 | 1 | 108.73 | 19.99 | 7.32 | 12.55 | 27.95 | 26.81 | 20.06 | 53.76 | 19.51 | 56.44 | 25.48 | 77.29 | 16.96 | 18.64 | 11.01 | 34.2 |
| Roach | 1213 | 3 | 3 | 1 | 108.63 | 18.41 | 6.18 | 12.81 | 24.55 | 25.44 | 18.37 | 51.11 | 18.48 | 53.82 | 23.71 | 77.57 | 15.96 | 21.56 | 11 | 33.93 |
| Roach | 1214 | 3 | 3 | 1 | 103.74 | 18.45 | 6.22 | 12.31 | 24.5 | 25.06 | 17.88 | 48.34 | 17.71 | 49.96 | 24.23 | 74.84 | 15.51 | 20.11 | 9.86 | 34.23 |
| Roach | 1215 | 3 | 3 | 1 | 84.66 | 14.88 | 5.32 | 9.31 | 21.45 | 20.89 | 16.12 | 41.03 | 15.1 | 42.21 | 17.18 | 60.62 | 12.76 | 17.03 | 7.7 | 24.36 |
| Roach | 1216 | 3 | 3 | 1 | 107.23 | 19.65 | 5.9 | 13.62 | 26.06 | 26.65 | 22.84 | 50.43 | 20.52 | 55.62 | 25.34 | 78.08 | 18.85 | 19.5 | 10.67 | 33.97 |
| Roach | 1217 | 3 | 3 | 1 | 106.6 | 20.32 | 6.92 | 12.72 | 26.81 | 26.79 | 20.16 | 55.22 | 19.55 | 56.46 | 24.52 | 76.83 | 15.66 | 19.01 | 10.14 | 31.2 |
| Roach | 1218 | 3 | 3 | 1 | 92.57 | 16.31 | 5.94 | 12.48 | 23.74 | 23.74 | 16.43 | 45.21 | 16.63 | 48.43 | 21.83 | 66.61 | 14.41 | 17.73 | 9.88 | 29.61 |
| Roach | 1219 | 3 | 3 | 1 | 101.88 | 18.41 | 6.68 | 11.91 | 24.38 | 24.23 | 19.2 | 48.75 | 17.11 | 49.02 | 22.84 | 71.94 | 14.73 | 19.03 | 10.33 | 30.88 |
| Roach | 1220 | 3 | 3 | 1 | 71.21 | 13.19 | 4.41 | 8.52 | 17.84 | 18.86 | 12.99 | 34.48 | 12.58 | 37.34 | 15.79 | 50.53 | 12.1 | 13.88 | 6.46 | 21.16 |
| Roach | 1191 | 3 | 3 | 2 | 121.03 | 21.37 | 6.87 | 14.34 | 29.05 | 29.05 | 22.71 | 58.13 | 21.47 | 61.03 | 28.12 | 86.22 | 17.88 | 23.5 | 11.63 | 36.25 |
| Roach | 1192 | 3 | 3 | 2 | 110.86 | 19.04 | 6.75 | 13.59 | 27.35 | 27.8 | 19.05 | 53.15 | 17.39 | 55.87 | 24.03 | 79.57 | 16.81 | 21.31 | 11.02 | 37.1 |
| Roach | 1193 | 3 | 3 | 2 | 101.04 | 18.19 | 6.01 | 11.74 | 23.9 | 22.9 | 20.71 | 49.12 | 18.62 | 49.73 | 22.96 | 71.94 | 14.88 | 19.65 | 11.06 | 34.14 |
| Roach | 1194 | 3 | 3 | 2 | 90.48 | 16.62 | 6.21 | 10.61 | 22.56 | 23.29 | 16.52 | 45.13 | 15.9 | 48.18 | 18.14 | 64.06 | 13.79 | 17.81 | 9.07 | 27.77 |
| Roach | 1195 | 3 | 3 | 2 | 106 | 19.83 | 6.76 | 12.39 | 25.74 | 25.05 | 18.06 | 51.52 | 17.34 | 53.88 | 22.13 | 76.19 | 13.48 | 20.44 | 10.06 | 33.53 |
| Roach | 1196 | 3 | 3 | 2 | 111.88 | 19.45 | 7.01 | 13.37 | 27.04 | 28.27 | 20.43 | 53.44 | 18.24 | 58.38 | 22.85 | 79.59 | 16.73 | 21.06 | 10.93 | 30.86 |
| Roach | 1197 | 3 | 3 | 2 | 125.92 | 23.27 | 8.75 | 17.49 | 33.21 | 33.92 | 23.87 | 65.03 | 21.3 | 65.03 | 29.43 | 94.45 | 19.11 | 22.22 | 12.99 | 43.92 |
| Roach | 1198 | 3 | 3 | 2 | 96.06 | 16.92 | 6.76 | 11.9 | 24.04 | 24.54 | 16.65 | 47.5 | 16.7 | 49.54 | 19.33 | 69.07 | 14.89 | 19.66 | 8.96 | 28.87 |
| Roach | 1199 | 3 | 3 | 2 | 101.93 | 17.41 | 6.4 | 11.55 | 23.09 | 23.08 | 18.85 | 45.57 | 16.33 | 50.58 | 21.58 | 69.63 | 15.27 | 20.95 | 9.81 | 29.71 |
| Roach | 1200 | 3 | 3 | 2 | 111.69 | 19.72 | 5.56 | 13.45 | 26.14 | 26.84 | 19.51 | 53.12 | 18.11 | 56.14 | 24.02 | 80.83 | 16.32 | 22.99 | 10.33 | 33.55 |
| Roach | 1201 | 3 | 3 | 2 | 115.17 | 21.24 | 7.23 | 13.32 | 28.03 | 27.44 | 19.94 | 57.75 | 20.51 | 60.11 | 26.18 | 83.03 | 18.05 | 22.63 | 11.55 | 38.27 |
| Roach | 1202 | 3 | 3 | 2 | 94.39 | 18.43 | 5.59 | 11.92 | 24.21 | 23.67 | 17.35 | 45.27 | 16.66 | 48.72 | 21.47 | 67.42 | 14.83 | 18.38 | 10.01 | 31.01 |
| Roach | 1203 | 3 | 3 | 2 | 95.28 | 16.64 | 5.56 | 10.59 | 22.3 | 23.23 | 17.13 | 45.06 | 16.91 | 46.53 | 21.15 | 69.07 | 14.48 | 17 | 8.4 | 29.18 |
| Roach | 1204 | 3 | 3 | 2 | 101.42 | 17.86 | 6.41 | 11.89 | 25.97 | 25.7 | 18.6 | 49.36 | 16.87 | 52.5 | 21.06 | 72.52 | 14.06 | 19.96 | 9.31 | 30.78 |
| Roach | 1205 | 3 | 3 | 2 | 103.22 | 18.93 | 6.8 | 11.65 | 25.63 | 25.21 | 17.77 | 50.63 | 16.83 | 53.34 | 22.15 | 72.64 | 15.15 | 17.53 | 10.04 | 31.51 |
| Roach | 1206 | 3 | 3 | 2 | 109.76 | 20.29 | 7 | 12.43 | 26.83 | 26.13 | 20.19 | 52.33 | 18.79 | 54.39 | 22.42 | 77.85 | 16.15 | 22.06 | 10.63 | 35.31 |
| Roach | 1207 | 3 | 3 | 2 | 97.83 | 18.65 | 6.6 | 12.88 | 25.36 | 25.6 | 19.66 | 45.44 | 17.93 | 52.89 | 23.31 | 70.14 | 14.63 | 18.73 | 10.16 | 31.39 |
| Roach | 1208 | 3 | 3 | 2 | 99.29 | 18.13 | 6.96 | 12.11 | 23.25 | 23.3 | 18.67 | 47.63 | 18.1 | 48.68 | 20.62 | 68.67 | 15.54 | 20.18 | 9.96 | 30.25 |
| Roach | 1209 | 3 | 3 | 2 | 82.9 | 15.41 | 5.25 | 9.46 | 20.25 | 21.1 | 14.81 | 39.44 | 14.01 | 43.21 | 16.07 | 58.17 | 12.17 | 16.4 | 7.81 | 24.75 |
| Roach | 1210 | 3 | 3 | 2 | 93.45 | 16.23 | 6.31 | 10.36 | 22.4 | 23.2 | 17.56 | 44.47 | 16.4 | 52.3 | 21.29 | 64.97 | 14.83 | 17.46 | 9.14 | 26.51 |
| Roach | 1211 | 3 | 3 | 2 | 80.87 | 15.07 | 5.12 | 9.55 | 21.01 | 20.73 | 13.15 | 41.02 | 13.49 | 43.49 | 17.34 | 58.41 | 11.85 | 15.13 | 7.45 | 22.21 |
| Roach | 1212 | 3 | 3 | 2 | 107.95 | 20.64 | 7.17 | 12.67 | 27.14 | 26.04 | 20.32 | 53.46 | 18.03 | 55.57 | 25.92 | 76.23 | 16.87 | 20 | 10.63 | 33.53 |
| Roach | 1213 | 3 | 3 | 2 | 108.69 | 17.86 | 6.33 | 12.48 | 25.25 | 25.25 | 18.09 | 51.87 | 16.9 | 53.5 | 23.24 | 77.26 | 15.18 | 21.07 | 10.77 | 33.65 |
| Roach | 1214 | 3 | 3 | 2 | 103.2 | 18.77 | 6.64 | 12.69 | 25.61 | 26.68 | 18.59 | 49.73 | 18.08 | 49.85 | 23.94 | 74.22 | 15.56 | 20.59 | 9.75 | 33.99 |
| Roach | 1215 | 3 | 3 | 2 | 85.58 | 15.28 | 5.27 | 9.49 | 20.84 | 21.16 | 15.9 | 43.4 | 13.28 | 45.3 | 18.09 | 59.46 | 11.53 | 16.61 | 7.6 | 24.56 |
| Roach | 1216 | 3 | 3 | 2 | 107.01 | 19.9 | 6.31 | 12.65 | 26.23 | 25.85 | 21.53 | 51.78 | 19.47 | 56.54 | 24.87 | 79.19 | 17.57 | 21.06 | 10.62 | 33.14 |
| Roach | 1217 | 3 | 3 | 2 | 106.85 | 19.62 | 6.68 | 13.79 | 28.04 | 28.86 | 21.14 | 53.55 | 19.35 | 54.7 | 24.26 | 76.07 | 16.29 | 17.24 | 10.44 | 30.44 |
| Roach | 1218 | 3 | 3 | 2 | 91.9 | 16.37 | 6.42 | 11.19 | 23.94 | 24.8 | 17.18 | 46.15 | 16.56 | 48.99 | 20.08 | 65.97 | 14.9 | 16.54 | 9.73 | 30.11 |
| Roach | 1219 | 3 | 3 | 2 | 101.54 | 18.29 | 6.44 | 11.93 | 24.63 | 24.95 | 18.24 | 49.2 | 17.09 | 50.71 | 21.41 | 72.06 | 15.01 | 20.24 | 10.11 | 30.76 |
| Roach | 1220 | 3 | 3 | 2 | 70.06 | 12.71 | 4.71 | 8.15 | 17.68 | 18.44 | 12.9 | 36.14 | 12.09 | 36.36 | 14.17 | 50.17 | 10.35 | 15.29 | 7.23 | 21.43 |
| Roach | 1191 | 3 | 3 | 3 | 121.13 | 21.8 | 6.95 | 14.63 | 28.7 | 27.8 | 23.06 | 57.63 | 21.82 | 60.7 | 26.16 | 86.69 | 18.36 | 24.79 | 11 | 35.98 |
| Roach | 1192 | 3 | 3 | 3 | 111.42 | 20.01 | 6.73 | 13.49 | 27.07 | 25.94 | 20.26 | 52.32 | 19.18 | 56.04 | 23.9 | 79.09 | 16.53 | 21.84 | 11.41 | 36.65 |
| Roach | 1193 | 3 | 3 | 3 | 102.48 | 18.26 | 6.13 | 12.07 | 24.43 | 23.22 | 21.15 | 48 | 18.35 | 50.83 | 24.6 | 71.96 | 15.63 | 19.59 | 10.68 | 34.19 |
| Roach | 1194 | 3 | 3 | 3 | 90.54 | 16.71 | 6.33 | 11.16 | 22.29 | 21.99 | 16.8 | 44.36 | 15.52 | 46.76 | 18.36 | 63.6 | 13.4 | 17.14 | 8.83 | 27.71 |
| Roach | 1195 | 3 | 3 | 3 | 106.44 | 18.51 | 6.25 | 11.96 | 25.21 | 24.36 | 18.8 | 50.15 | 17.64 | 53.95 | 22.1 | 76.82 | 14.93 | 20.37 | 10.57 | 32.98 |
| Roach | 1196 | 3 | 3 | 3 | 111.1 | 20.49 | 6.96 | 14.02 | 27.55 | 26.53 | 20.84 | 53.65 | 18.36 | 60.77 | 24.37 | 79.36 | 17.55 | 21.56 | 11.21 | 34.72 |
| Roach | 1197 | 3 | 3 | 3 | 126.93 | 23.66 | 8.32 | 17.46 | 32.83 | 32.12 | 24.06 | 63.36 | 22.91 | 68.16 | 29.63 | 93.64 | 18.88 | 20.91 | 12.96 | 44.47 |
| Roach | 1198 | 3 | 3 | 3 | 95.18 | 17.12 | 5.58 | 11.97 | 23.46 | 22.84 | 17.52 | 46.84 | 17.11 | 50.1 | 20.95 | 69.06 | 15.23 | 17.91 | 8.87 | 28.76 |
| Roach | 1199 | 3 | 3 | 3 | 102.84 | 16.81 | 6.36 | 11.34 | 22.85 | 22.47 | 20.57 | 45.93 | 16.99 | 47.97 | 22.88 | 69.24 | 14.86 | 19.93 | 9.94 | 29.97 |
| Roach | 1200 | 3 | 3 | 3 | 111.94 | 19.74 | 6.24 | 13.22 | 25.7 | 25.7 | 20.43 | 52.28 | 18.41 | 58.04 | 24.66 | 80.18 | 16.12 | 21.49 | 10.31 | 33.19 |
| Roach | 1201 | 3 | 3 | 3 | 115.84 | 21.35 | 6.65 | 13.2 | 27.22 | 26.41 | 20.58 | 55.66 | 21.18 | 60.82 | 26.47 | 83.12 | 18.35 | 23.45 | 11.94 | 38.06 |
| Roach | 1202 | 3 | 3 | 3 | 95.47 | 17.94 | 5.6 | 12.05 | 23.55 | 21.77 | 19.48 | 44.92 | 18 | 51.79 | 23.19 | 67.5 | 14.26 | 18.49 | 10.38 | 30.89 |
| Roach | 1203 | 3 | 3 | 3 | 95.74 | 16.7 | 6.03 | 11 | 22.74 | 21.98 | 17.58 | 46.29 | 17.41 | 47.27 | 21.34 | 66.92 | 15.14 | 17.26 | 8.69 | 30.23 |
| Roach | 1204 | 3 | 3 | 3 | 100.96 | 17.21 | 6.26 | 12.69 | 25.38 | 24.8 | 19.02 | 49.22 | 17.52 | 52.5 | 23.12 | 71.91 | 14.5 | 20.01 | 9.01 | 30.95 |
| Roach | 1205 | 3 | 3 | 3 | 103.09 | 18.25 | 6.38 | 11.39 | 24.82 | 23 | 18.76 | 50.07 | 17.84 | 51.34 | 21.5 | 74.64 | 14.71 | 16.85 | 9.95 | 31.41 |
| Roach | 1206 | 3 | 3 | 3 | 109.33 | 18.7 | 6.73 | 12.62 | 26.89 | 26.29 | 19.58 | 52.14 | 20.08 | 54.9 | 23.43 | 77.67 | 15.77 | 22.9 | 11.1 | 34.95 |
| Roach | 1207 | 3 | 3 | 3 | 97.47 | 19.28 | 6.37 | 12.1 | 24.63 | 23.47 | 20.88 | 46.84 | 19.22 | 51.21 | 22.66 | 70.01 | 15.63 | 18.56 | 10.26 | 31.13 |
| Roach | 1208 | 3 | 3 | 3 | 100.1 | 18.25 | 6.77 | 12.34 | 23.89 | 23.02 | 18.22 | 46.79 | 18.27 | 49.29 | 23.03 | 68.91 | 15.96 | 20.38 | 9.4 | 30.22 |
| Roach | 1209 | 3 | 3 | 3 | 82.81 | 14.09 | 5.2 | 9.55 | 20.18 | 19.62 | 15.03 | 39.55 | 14.26 | 42.17 | 17.03 | 56.78 | 13.09 | 16.49 | 7.91 | 24.92 |
| Roach | 1210 | 3 | 3 | 3 | 93.24 | 16.26 | 6.43 | 10.54 | 23.06 | 22.57 | 17.81 | 45.59 | 15.83 | 50.08 | 22.6 | 65.06 | 14.53 | 16.71 | 8.83 | 26.09 |
| Roach | 1211 | 3 | 3 | 3 | 80.55 | 14.97 | 5.34 | 9.4 | 19.86 | 19.65 | 14.06 | 39.45 | 14.28 | 43.49 | 16.8 | 57.3 | 11.73 | 14.69 | 7.58 | 22.06 |
| Roach | 1212 | 3 | 3 | 3 | 107.8 | 19.85 | 6.65 | 12.7 | 26.59 | 26.39 | 21.61 | 52 | 19.35 | 55.44 | 24.91 | 76.68 | 16.57 | 18.82 | 10.27 | 33.64 |
| Roach | 1213 | 3 | 3 | 3 | 108.35 | 18.72 | 6.4 | 12.46 | 24.93 | 25.49 | 18.7 | 50.92 | 18.84 | 54.17 | 22.38 | 76.63 | 16.14 | 21.29 | 10.17 | 33.39 |
| Roach | 1214 | 3 | 3 | 3 | 104.03 | 18.8 | 6.02 | 12.5 | 25.1 | 24.67 | 19.7 | 49.42 | 18.73 | 50.91 | 25.61 | 73.02 | 15.31 | 19.73 | 9.65 | 33.95 |
| Roach | 1215 | 3 | 3 | 3 | 84.73 | 14.84 | 4.89 | 9.82 | 20.5 | 19.84 | 15.26 | 41.78 | 14.14 | 42.23 | 17.53 | 60.7 | 11.11 | 16.15 | 8.22 | 24.15 |
| Roach | 1216 | 3 | 3 | 3 | 107.57 | 19.1 | 5.51 | 13.27 | 25.88 | 24.6 | 21.48 | 50.31 | 19.47 | 55.59 | 25.03 | 76.23 | 18.32 | 21.03 | 10.55 | 33.3 |
| Roach | 1217 | 3 | 3 | 3 | 106.42 | 19.78 | 6.89 | 13.51 | 28.2 | 26.12 | 21.04 | 53.86 | 19.66 | 57.4 | 23.82 | 78.77 | 15.42 | 17.96 | 9.95 | 31.12 |
| Roach | 1218 | 3 | 3 | 3 | 93.19 | 16.87 | 5.65 | 11.31 | 23.36 | 23.11 | 17.88 | 45.89 | 17.2 | 49.78 | 21.33 | 65.53 | 15.02 | 17.19 | 9.56 | 30.28 |
| Roach | 1219 | 3 | 3 | 3 | 101.64 | 18.8 | 6.48 | 12.35 | 24.95 | 24.65 | 19.6 | 48.71 | 17.98 | 50.49 | 21.92 | 72.69 | 15.24 | 18.63 | 10.1 | 31.1 |
| Roach | 1220 | 3 | 3 | 3 | 69.57 | 12.51 | 4.44 | 8.78 | 17.84 | 17.84 | 13.84 | 36.05 | 13.4 | 38.26 | 16.06 | 50.57 | 10.84 | 13.4 | 6.29 | 21.09 |
| Prussian carp | 1061 | 1 | 1 | 1 | 110.66 | 75.43 | 47.56 | 46.05 | 28.87 | 30.49 | 13.91 | 9.52 | 27.41 | 43.46 | 13.36 | 17.13 | 19.11 | 17.55 | 19.48 | 15.52 |
| Prussian carp | 1062 | 1 | 1 | 1 | 128.13 | 77.56 | 45.78 | 47.92 | 28.91 | 30.17 | 14.74 | 8.91 | 26.54 | 42.06 | 10.82 | 17.82 | 20.43 | 15.55 | 21.29 | 13.90 |
| Prussian carp | 1063 | 1 | 1 | 1 | 122.71 | 78.42 | 49.57 | 48.82 | 31.10 | 30.33 | 14.43 | 8.85 | 27.08 | 42.95 | 12.87 | 15.49 | 19.44 | 17.52 | 21.40 | 16.19 |
| Prussian carp | 1064 | 1 | 1 | 1 | 120.69 | 75.67 | 47.23 | 46.78 | 31.75 | 30.66 | 15.38 | 9.68 | 27.02 | 42.94 | 11.54 | 17.14 | 20.25 | 16.40 | 22.97 | 17.00 |
| Prussian carp | 1065 | 1 | 1 | 1 | 111.71 | 76.66 | 48.58 | 46.73 | 30.17 | 29.52 | 14.58 | 9.40 | 28.08 | 44.65 | 11.47 | 16.48 | 20.96 | 17.31 | 21.96 | 17.14 |
| Prussian carp | 1066 | 1 | 1 | 1 | 119.66 | 78.48 | 46.53 | 48.08 | 29.58 | 29.34 | 13.66 | 9.07 | 25.18 | 40.00 | 11.25 | 15.96 | 19.69 | 14.54 | 21.80 | 13.37 |
| Prussian carp | 1067 | 1 | 1 | 1 | 129.93 | 80.14 | 49.21 | 48.44 | 29.84 | 29.96 | 14.89 | 8.40 | 26.50 | 45.98 | 14.21 | 15.98 | 19.64 | 14.75 | 22.20 | 16.21 |
| Prussian carp | 1068 | 1 | 1 | 1 | 126.44 | 79.98 | 47.01 | 48.15 | 30.76 | 29.90 | 13.75 | 9.12 | 27.52 | 43.31 | 8.52 | 17.44 | 21.29 | 18.16 | 22.56 | 19.54 |
| Prussian carp | 1069 | 1 | 1 | 1 | 105.48 | 78.43 | 48.08 | 49.88 | 30.98 | 30.90 | 13.72 | 9.82 | 27.24 | 43.60 | 11.66 | 17.42 | 18.94 | 17.77 | 22.15 | 17.90 |
| Prussian carp | 1070 | 1 | 1 | 1 | 108.00 | 75.13 | 47.24 | 49.33 | 30.21 | 32.01 | 15.26 | 9.28 | 28.40 | 47.16 | 12.14 | 16.96 | 20.02 | 16.73 | 22.70 | 14.06 |
| Prussian carp | 1071 | 1 | 1 | 1 | 127.98 | 81.07 | 51.26 | 48.11 | 32.32 | 30.63 | 14.62 | 9.25 | 29.05 | 43.00 | 9.64 | 17.42 | 18.87 | 19.06 | 22.46 | 17.82 |
| Prussian carp | 1072 | 1 | 1 | 1 | 136.09 | 78.32 | 48.16 | 47.35 | 31.06 | 30.79 | 14.37 | 9.50 | 26.36 | 42.76 | 10.97 | 16.42 | 19.61 | 18.08 | 22.26 | 16.98 |
| Prussian carp | 1073 | 1 | 1 | 1 | 103.41 | 76.59 | 53.45 | 48.59 | 31.76 | 30.43 | 14.85 | 10.62 | 26.16 | 41.95 | 11.90 | 16.79 | 18.16 | 17.86 | 20.73 | 17.64 |
| Prussian carp | 1074 | 1 | 1 | 1 | 126.26 | 78.61 | 47.47 | 47.12 | 30.84 | 29.98 | 14.62 | 8.35 | 25.37 | 42.14 | 9.52 | 16.10 | 19.57 | 16.13 | 22.15 | 14.86 |
| Prussian carp | 1075 | 1 | 1 | 1 | 124.04 | 78.21 | 53.58 | 50.34 | 31.17 | 31.14 | 15.18 | 9.61 | 28.46 | 44.26 | 14.07 | 17.78 | 18.12 | 14.99 | 21.37 | 14.72 |
| Prussian carp | 1076 | 1 | 1 | 1 | 116.47 | 79.16 | 53.39 | 51.31 | 32.57 | 31.82 | 16.25 | 9.17 | 26.52 | 46.88 | 11.89 | 16.72 | 21.93 | 20.15 | 25.47 | 18.48 |
| Prussian carp | 1077 | 1 | 1 | 1 | 136.65 | 79.12 | 47.17 | 47.12 | 29.53 | 28.18 | 14.20 | 8.40 | 26.47 | 42.67 | 12.93 | 17.48 | 18.58 | 17.92 | 22.71 | 16.49 |
| Prussian carp | 1078 | 1 | 1 | 1 | 111.54 | 76.45 | 52.18 | 48.54 | 33.35 | 31.76 | 15.76 | 9.01 | 29.56 | 47.41 | 10.18 | 18.50 | 20.77 | 16.81 | 22.45 | 15.88 |
| Prussian carp | 1079 | 1 | 1 | 1 | 118.76 | 82.21 | 51.61 | 47.35 | 30.03 | 28.96 | 14.28 | 8.61 | 28.85 | 47.44 | 8.10 | 17.64 | 21.87 | 19.05 | 24.44 | 17.27 |
| Prussian carp | 1080 | 1 | 1 | 1 | 123.55 | 79.60 | 50.14 | 46.83 | 29.36 | 30.13 | 15.50 | 8.66 | 25.95 | 45.29 | 10.82 | 16.50 | 20.93 | 22.13 | 22.27 | 20.30 |
| Prussian carp | 1081 | 1 | 1 | 1 | 110.08 | 77.69 | 48.01 | 48.36 | 31.17 | 31.54 | 16.11 | 9.33 | 27.38 | 46.04 | 12.85 | 16.11 | 20.82 | 17.04 | 23.54 | 17.94 |
| Prussian carp | 1082 | 1 | 1 | 1 | 111.72 | 78.50 | 46.23 | 43.01 | 27.81 | 28.75 | 14.24 | 8.41 | 27.33 | 42.19 | 13.28 | 15.95 | 20.89 | 20.88 | 22.07 | 16.83 |
| Prussian carp | 1083 | 1 | 1 | 1 | 105.47 | 79.63 | 48.32 | 46.69 | 30.57 | 31.15 | 14.81 | 9.57 | 27.25 | 45.62 | 12.67 | 17.45 | 20.91 | 21.64 | 22.87 | 16.23 |
| Prussian carp | 1084 | 1 | 1 | 1 | 120.07 | 78.40 | 50.49 | 48.51 | 32.31 | 30.61 | 14.36 | 9.33 | 28.02 | 43.74 | 9.60 | 16.65 | 18.22 | 15.33 | 20.63 | 15.08 |
| Prussian carp | 1085 | 1 | 1 | 1 | 142.51 | 78.62 | 49.52 | 46.13 | 28.75 | 29.37 | 13.10 | 8.62 | 25.18 | 41.59 | 11.75 | 16.14 | 18.16 | 16.26 | 21.50 | 16.92 |
| Prussian carp | 1086 | 1 | 1 | 1 | 120.54 | 76.60 | 47.68 | 48.32 | 30.62 | 30.15 | 15.30 | 9.64 | 26.02 | 41.01 | 11.06 | 16.57 | 19.30 | 19.98 | 22.86 | 16.27 |
| Prussian carp | 1087 | 1 | 1 | 1 | 112.90 | 80.47 | 52.03 | 49.48 | 29.82 | 31.20 | 14.72 | 8.43 | 28.44 | 46.57 | 12.49 | 17.08 | 19.94 | 17.88 | 22.38 | 16.44 |
| Prussian carp | 1088 | 1 | 1 | 1 | 97.98 | 76.33 | 44.71 | 48.72 | 30.86 | 29.74 | 15.05 | 7.99 | 26.24 | 44.28 | 11.66 | 15.79 | 18.07 | 17.93 | 24.48 | 15.51 |
| Prussian carp | 1089 | 1 | 1 | 1 | 126.89 | 77.98 | 46.68 | 46.31 | 30.96 | 30.69 | 14.85 | 9.66 | 27.13 | 43.93 | 10.83 | 15.69 | 19.34 | 17.83 | 23.20 | 16.80 |
| Prussian carp | 1090 | 1 | 1 | 1 | 107.91 | 78.46 | 47.29 | 47.71 | 30.88 | 29.80 | 14.28 | 8.77 | 26.73 | 42.74 | 10.96 | 16.70 | 20.42 | 16.51 | 22.29 | 15.90 |
| Prussian carp | 1061 | 1 | 1 | 2 | 112.96 | 78.35 | 47.15 | 46.03 | 31.64 | 30.00 | 14.88 | 9.22 | 27.21 | 43.38 | 10.68 | 16.62 | 18.02 | 16.01 | 18.89 | 16.53 |
| Prussian carp | 1062 | 1 | 1 | 2 | 129.22 | 78.73 | 46.65 | 45.74 | 29.98 | 28.59 | 14.92 | 8.46 | 28.18 | 40.20 | 11.61 | 16.99 | 18.11 | 14.53 | 21.59 | 13.33 |
| Prussian carp | 1063 | 1 | 1 | 2 | 121.83 | 80.25 | 50.49 | 48.43 | 31.76 | 30.26 | 14.77 | 9.65 | 27.33 | 43.19 | 13.24 | 15.52 | 18.53 | 16.01 | 21.11 | 15.99 |
| Prussian carp | 1064 | 1 | 1 | 2 | 118.46 | 78.30 | 47.66 | 46.23 | 31.59 | 29.91 | 14.70 | 10.34 | 28.00 | 45.15 | 9.79 | 17.17 | 21.04 | 18.45 | 21.29 | 15.86 |
| Prussian carp | 1065 | 1 | 1 | 2 | 111.38 | 78.80 | 48.48 | 47.57 | 32.43 | 30.23 | 14.12 | 9.74 | 27.82 | 45.26 | 10.32 | 16.78 | 18.46 | 19.11 | 22.27 | 15.95 |
| Prussian carp | 1066 | 1 | 1 | 2 | 117.40 | 79.14 | 48.53 | 47.25 | 29.18 | 29.14 | 13.24 | 8.74 | 26.65 | 41.59 | 10.95 | 16.13 | 16.98 | 15.41 | 22.72 | 13.54 |
| Prussian carp | 1067 | 1 | 1 | 2 | 129.47 | 79.98 | 51.81 | 48.10 | 32.65 | 30.77 | 15.18 | 9.27 | 27.40 | 46.95 | 11.91 | 16.14 | 17.16 | 15.87 | 22.26 | 17.58 |
| Prussian carp | 1068 | 1 | 1 | 2 | 126.05 | 80.01 | 49.86 | 47.82 | 33.47 | 30.94 | 15.43 | 8.80 | 27.43 | 43.19 | 9.41 | 17.69 | 19.34 | 19.43 | 22.44 | 17.51 |
| Prussian carp | 1069 | 1 | 1 | 2 | 105.84 | 79.04 | 50.76 | 48.73 | 32.09 | 30.79 | 14.71 | 9.38 | 28.80 | 44.34 | 14.13 | 16.81 | 18.40 | 18.99 | 22.23 | 18.11 |
| Prussian carp | 1070 | 1 | 1 | 2 | 109.10 | 76.17 | 48.96 | 45.82 | 33.42 | 29.79 | 14.97 | 8.98 | 29.64 | 46.41 | 15.15 | 16.72 | 16.53 | 20.20 | 21.97 | 13.91 |
| Prussian carp | 1071 | 1 | 1 | 2 | 127.29 | 82.55 | 51.55 | 48.33 | 31.55 | 30.73 | 15.27 | 10.19 | 28.84 | 43.54 | 9.18 | 17.37 | 19.47 | 17.84 | 22.16 | 18.83 |
| Prussian carp | 1072 | 1 | 1 | 2 | 132.28 | 81.59 | 51.30 | 47.77 | 33.10 | 31.43 | 15.39 | 9.81 | 27.81 | 43.75 | 11.24 | 16.50 | 17.97 | 20.83 | 23.29 | 17.03 |
| Prussian carp | 1073 | 1 | 1 | 2 | 103.72 | 80.58 | 53.55 | 47.72 | 33.47 | 31.27 | 16.17 | 9.75 | 26.88 | 41.79 | 10.89 | 16.54 | 17.40 | 17.12 | 19.94 | 18.40 |
| Prussian carp | 1074 | 1 | 1 | 2 | 126.48 | 77.66 | 46.77 | 44.60 | 29.75 | 28.49 | 15.17 | 8.63 | 27.71 | 42.83 | 9.82 | 15.55 | 17.23 | 16.92 | 21.23 | 15.03 |
| Prussian carp | 1075 | 1 | 1 | 2 | 123.36 | 80.75 | 53.57 | 51.25 | 32.19 | 30.10 | 15.63 | 9.09 | 27.16 | 44.74 | 10.31 | 18.01 | 17.82 | 16.62 | 21.53 | 14.59 |
| Prussian carp | 1076 | 1 | 1 | 2 | 120.72 | 76.16 | 49.44 | 47.18 | 32.17 | 30.48 | 15.67 | 9.08 | 26.26 | 44.95 | 11.67 | 16.20 | 19.67 | 16.64 | 25.21 | 17.04 |
| Prussian carp | 1077 | 1 | 1 | 2 | 136.04 | 76.86 | 48.39 | 45.82 | 29.14 | 27.60 | 14.75 | 8.68 | 28.07 | 42.85 | 12.76 | 17.78 | 14.98 | 18.26 | 21.92 | 17.93 |
| Prussian carp | 1078 | 1 | 1 | 2 | 108.98 | 81.20 | 51.60 | 49.04 | 32.36 | 30.95 | 16.26 | 10.08 | 29.59 | 47.28 | 11.39 | 18.26 | 18.02 | 21.27 | 22.28 | 15.67 |
| Prussian carp | 1079 | 1 | 1 | 2 | 117.62 | 84.74 | 50.93 | 47.12 | 32.25 | 29.74 | 14.87 | 9.22 | 30.41 | 47.93 | 11.20 | 17.92 | 19.43 | 18.22 | 25.72 | 17.38 |
| Prussian carp | 1080 | 1 | 1 | 2 | 124.31 | 79.58 | 50.66 | 48.06 | 31.16 | 29.58 | 15.18 | 8.75 | 27.61 | 45.56 | 12.37 | 16.63 | 17.74 | 18.23 | 23.07 | 17.26 |
| Prussian carp | 1081 | 1 | 1 | 2 | 110.78 | 76.68 | 48.11 | 46.72 | 32.99 | 31.06 | 15.59 | 8.94 | 29.10 | 45.72 | 12.68 | 16.18 | 19.12 | 16.97 | 23.40 | 17.01 |
| Prussian carp | 1082 | 1 | 1 | 2 | 110.60 | 79.83 | 47.20 | 44.67 | 31.03 | 29.54 | 14.44 | 9.31 | 27.94 | 43.89 | 13.32 | 16.25 | 18.98 | 17.91 | 23.10 | 16.93 |
| Prussian carp | 1083 | 1 | 1 | 2 | 104.77 | 81.04 | 48.91 | 45.87 | 31.73 | 30.27 | 15.39 | 8.99 | 28.79 | 46.46 | 12.42 | 17.99 | 17.60 | 19.13 | 22.46 | 16.24 |
| Prussian carp | 1084 | 1 | 1 | 2 | 118.87 | 78.89 | 51.22 | 47.48 | 32.80 | 30.76 | 14.82 | 9.30 | 29.71 | 45.28 | 10.31 | 16.57 | 18.35 | 15.84 | 21.67 | 15.32 |
| Prussian carp | 1085 | 1 | 1 | 2 | 141.22 | 79.82 | 50.99 | 48.33 | 32.99 | 30.26 | 14.02 | 9.66 | 26.47 | 36.84 | 11.53 | 16.60 | 18.14 | 15.89 | 21.38 | 19.62 |
| Prussian carp | 1086 | 1 | 1 | 2 | 119.30 | 79.88 | 47.81 | 46.83 | 33.40 | 30.32 | 15.23 | 8.88 | 27.17 | 42.08 | 12.19 | 16.71 | 17.36 | 14.62 | 22.85 | 17.79 |
| Prussian carp | 1087 | 1 | 1 | 2 | 113.19 | 80.53 | 52.33 | 49.76 | 32.20 | 31.02 | 15.32 | 8.83 | 28.82 | 46.68 | 11.88 | 16.77 | 18.81 | 17.72 | 23.25 | 15.53 |
| Prussian carp | 1088 | 1 | 1 | 2 | 97.78 | 78.34 | 44.92 | 47.34 | 32.33 | 29.85 | 14.71 | 8.24 | 25.96 | 44.89 | 13.77 | 15.79 | 17.65 | 16.67 | 21.66 | 15.22 |
| Prussian carp | 1089 | 1 | 1 | 2 | 128.25 | 77.32 | 45.96 | 47.31 | 33.60 | 30.49 | 14.45 | 8.96 | 27.19 | 43.60 | 11.48 | 15.49 | 18.31 | 17.84 | 23.50 | 16.11 |
| Prussian carp | 1090 | 1 | 1 | 2 | 107.89 | 78.20 | 45.45 | 45.50 | 31.80 | 30.05 | 15.06 | 9.42 | 27.37 | 43.71 | 12.15 | 15.50 | 20.19 | 17.37 | 22.93 | 15.49 |
| Prussian carp | 1061 | 1 | 1 | 3 | 111.60 | 77.42 | 47.79 | 45.97 | 31.03 | 29.67 | 14.06 | 9.84 | 26.52 | 43.59 | 11.00 | 16.69 | 19.94 | 17.17 | 20.18 | 15.16 |
| Prussian carp | 1062 | 1 | 1 | 3 | 116.60 | 87.34 | 48.37 | 51.46 | 34.08 | 32.15 | 16.17 | 9.85 | 29.70 | 46.61 | 12.42 | 19.56 | 20.10 | 15.54 | 22.94 | 14.80 |
| Prussian carp | 1063 | 1 | 1 | 3 | 120.10 | 79.39 | 51.10 | 48.77 | 33.67 | 30.45 | 14.94 | 8.85 | 28.20 | 44.18 | 10.99 | 16.32 | 17.91 | 17.66 | 23.16 | 16.68 |
| Prussian carp | 1064 | 1 | 1 | 3 | 119.10 | 79.56 | 48.41 | 46.46 | 32.51 | 30.61 | 14.58 | 9.44 | 28.25 | 44.55 | 11.92 | 17.02 | 19.38 | 18.08 | 21.19 | 16.14 |
| Prussian carp | 1065 | 1 | 1 | 3 | 111.08 | 81.37 | 49.73 | 53.51 | 31.09 | 29.59 | 14.88 | 10.04 | 28.01 | 45.33 | 10.99 | 16.35 | 18.94 | 19.14 | 22.28 | 16.80 |
| Prussian carp | 1066 | 1 | 1 | 3 | 118.43 | 78.80 | 45.87 | 45.90 | 31.63 | 29.26 | 13.87 | 9.40 | 27.01 | 41.75 | 10.57 | 16.09 | 16.14 | 16.74 | 22.70 | 13.97 |
| Prussian carp | 1067 | 1 | 1 | 3 | 128.31 | 79.86 | 52.83 | 48.77 | 33.00 | 30.12 | 15.40 | 10.04 | 27.63 | 47.80 | 12.16 | 15.87 | 19.67 | 13.91 | 22.87 | 18.36 |
| Prussian carp | 1068 | 1 | 1 | 3 | 127.80 | 80.43 | 49.87 | 48.16 | 33.10 | 30.25 | 15.95 | 8.55 | 27.60 | 43.93 | 10.90 | 17.06 | 18.84 | 17.78 | 21.89 | 17.44 |
| Prussian carp | 1069 | 1 | 1 | 3 | 104.41 | 81.43 | 48.46 | 47.50 | 31.92 | 30.85 | 14.45 | 9.01 | 29.84 | 45.71 | 10.34 | 16.94 | 17.94 | 19.69 | 22.91 | 17.83 |
| Prussian carp | 1070 | 1 | 1 | 3 | 107.84 | 78.39 | 48.70 | 47.43 | 31.64 | 29.53 | 15.25 | 9.46 | 27.49 | 47.60 | 13.66 | 17.25 | 17.91 | 16.92 | 22.17 | 13.56 |
| Prussian carp | 1071 | 1 | 1 | 3 | 127.76 | 81.35 | 51.23 | 48.09 | 32.33 | 30.97 | 14.86 | 10.25 | 29.53 | 43.85 | 10.36 | 17.57 | 19.61 | 17.98 | 21.12 | 18.74 |
| Prussian carp | 1072 | 1 | 1 | 3 | 133.21 | 82.05 | 52.38 | 49.91 | 33.46 | 30.84 | 15.46 | 8.50 | 28.24 | 43.55 | 12.62 | 16.80 | 18.24 | 18.12 | 22.01 | 17.57 |
| Prussian carp | 1073 | 1 | 1 | 3 | 102.51 | 77.57 | 52.15 | 48.59 | 33.83 | 31.49 | 15.63 | 9.07 | 21.22 | 41.70 | 9.93 | 16.12 | 16.75 | 16.82 | 19.41 | 14.35 |
| Prussian carp | 1074 | 1 | 1 | 3 | 126.17 | 78.00 | 48.74 | 44.99 | 32.06 | 29.99 | 15.24 | 9.03 | 26.78 | 42.37 | 9.94 | 15.45 | 18.39 | 16.15 | 21.84 | 14.93 |
| Prussian carp | 1075 | 1 | 1 | 3 | 124.35 | 81.27 | 52.20 | 48.15 | 32.39 | 30.81 | 16.19 | 8.92 | 29.31 | 45.25 | 11.80 | 17.67 | 17.53 | 17.25 | 20.94 | 15.85 |
| Prussian carp | 1076 | 1 | 1 | 3 | 118.14 | 79.35 | 51.76 | 48.70 | 32.80 | 30.73 | 16.38 | 9.84 | 28.92 | 46.37 | 9.71 | 16.41 | 18.66 | 14.85 | 25.12 | 16.48 |
| Prussian carp | 1077 | 1 | 1 | 3 | 135.95 | 78.41 | 49.38 | 46.68 | 31.70 | 29.41 | 14.50 | 8.91 | 26.19 | 44.16 | 13.16 | 17.17 | 17.22 | 16.71 | 21.18 | 17.13 |
| Prussian carp | 1078 | 1 | 1 | 3 | 110.47 | 81.95 | 54.28 | 48.82 | 34.26 | 31.53 | 16.78 | 10.13 | 29.39 | 47.99 | 10.62 | 18.33 | 16.34 | 17.86 | 23.04 | 15.32 |
| Prussian carp | 1079 | 1 | 1 | 3 | 116.62 | 84.11 | 50.96 | 47.79 | 32.04 | 30.15 | 14.31 | 8.23 | 30.33 | 49.25 | 9.74 | 17.73 | 23.37 | 18.31 | 24.83 | 16.91 |
| Prussian carp | 1080 | 1 | 1 | 3 | 122.82 | 80.89 | 48.15 | 45.45 | 33.39 | 31.15 | 15.35 | 9.83 | 27.65 | 47.11 | 13.66 | 16.63 | 19.02 | 17.24 | 23.64 | 16.10 |
| Prussian carp | 1081 | 1 | 1 | 3 | 111.86 | 78.30 | 46.57 | 44.75 | 32.82 | 30.93 | 14.42 | 9.12 | 28.03 | 45.26 | 12.42 | 16.15 | 19.34 | 15.74 | 22.97 | 17.54 |
| Prussian carp | 1082 | 1 | 1 | 3 | 111.32 | 79.22 | 45.74 | 45.54 | 32.19 | 29.77 | 14.34 | 9.81 | 27.49 | 42.97 | 13.00 | 16.69 | 19.22 | 17.41 | 22.12 | 17.24 |
| Prussian carp | 1083 | 1 | 1 | 3 | 106.08 | 80.85 | 47.43 | 48.89 | 31.53 | 30.24 | 14.69 | 9.61 | 28.28 | 45.57 | 11.58 | 17.58 | 18.31 | 16.03 | 22.10 | 15.99 |
| Prussian carp | 1084 | 1 | 1 | 3 | 118.53 | 77.05 | 48.60 | 47.53 | 34.22 | 31.57 | 15.80 | 9.72 | 30.26 | 45.94 | 10.07 | 15.58 | 19.06 | 15.84 | 21.75 | 15.66 |
| Prussian carp | 1085 | 1 | 1 | 3 | 131.72 | 84.30 | 55.97 | 51.24 | 33.98 | 32.11 | 15.08 | 10.24 | 28.72 | 46.16 | 13.58 | 17.58 | 17.13 | 17.47 | 23.46 | 18.35 |
| Prussian carp | 1086 | 1 | 1 | 3 | 117.33 | 80.13 | 47.82 | 49.91 | 33.36 | 31.26 | 15.59 | 9.45 | 27.20 | 43.25 | 12.43 | 16.62 | 18.08 | 16.95 | 23.51 | 19.01 |
| Prussian carp | 1087 | 1 | 1 | 3 | 114.01 | 77.31 | 49.14 | 46.69 | 31.96 | 29.50 | 15.00 | 8.22 | 28.88 | 46.80 | 10.60 | 16.22 | 17.47 | 16.24 | 22.59 | 15.77 |
| Prussian carp | 1088 | 1 | 1 | 3 | 97.03 | 77.96 | 48.89 | 46.00 | 29.90 | 28.97 | 16.53 | 8.66 | 26.75 | 35.00 | 11.55 | 15.64 | 16.96 | 15.96 | 22.72 | 16.93 |
| Prussian carp | 1089 | 1 | 1 | 3 | 128.11 | 76.52 | 47.73 | 46.61 | 31.96 | 29.74 | 14.90 | 9.26 | 27.51 | 43.77 | 11.67 | 15.83 | 19.81 | 17.72 | 23.01 | 17.03 |
| Prussian carp | 1090 | 1 | 1 | 3 | 108.16 | 78.81 | 46.96 | 45.59 | 31.30 | 29.55 | 13.31 | 8.67 | 27.39 | 42.91 | 11.04 | 15.78 | 19.97 | 15.50 | 22.71 | 14.65 |
| Prussian carp | 1061 | 1 | 2 | 1 | 108.02 | 78.47 | 48.50 | 51.43 | 27.76 | 29.98 | 17.21 | 6.28 | 25.91 | 43.12 | 18.40 | 17.12 | 20.10 | 24.74 | 21.76 | 21.92 |
| Prussian carp | 1062 | 1 | 2 | 1 | 107.53 | 79.10 | 47.55 | 46.42 | 28.21 | 31.31 | 16.86 | 7.23 | 25.74 | 41.30 | 17.29 | 17.91 | 19.71 | 21.00 | 21.38 | 18.98 |
| Prussian carp | 1063 | 1 | 2 | 1 | 108.61 | 80.69 | 49.56 | 47.19 | 28.66 | 30.66 | 15.80 | 7.05 | 26.69 | 44.26 | 19.03 | 16.30 | 21.43 | 23.86 | 21.95 | 21.13 |
| Prussian carp | 1064 | 1 | 2 | 1 | 71.06 | 80.61 | 48.41 | 49.52 | 29.33 | 33.50 | 16.69 | 9.18 | 24.34 | 43.63 | 17.94 | 17.71 | 21.11 | 22.56 | 20.93 | 20.56 |
| Prussian carp | 1065 | 1 | 2 | 1 | 95.65 | 78.74 | 48.07 | 48.83 | 30.15 | 31.71 | 16.57 | 8.51 | 26.63 | 44.08 | 18.58 | 16.66 | 20.56 | 23.79 | 19.87 | 22.09 |
| Prussian carp | 1066 | 1 | 2 | 1 | 92.18 | 79.50 | 46.70 | 48.24 | 28.56 | 31.23 | 16.58 | 8.02 | 26.68 | 41.55 | 19.16 | 16.42 | 20.31 | 22.60 | 22.35 | 20.36 |
| Prussian carp | 1067 | 1 | 2 | 1 | 115.28 | 79.84 | 51.05 | 46.28 | 27.94 | 32.24 | 16.36 | 8.38 | 27.29 | 48.53 | 18.07 | 16.71 | 20.75 | 25.25 | 21.01 | 21.49 |
| Prussian carp | 1068 | 1 | 2 | 1 | 102.71 | 82.86 | 47.94 | 47.91 | 30.72 | 32.07 | 16.19 | 7.35 | 29.43 | 43.30 | 18.44 | 17.61 | 20.70 | 23.55 | 22.93 | 21.63 |
| Prussian carp | 1069 | 1 | 2 | 1 | 59.40 | 81.46 | 51.50 | 50.87 | 30.17 | 31.22 | 16.97 | 7.09 | 27.38 | 43.75 | 19.48 | 16.64 | 18.93 | 24.67 | 22.03 | 22.79 |
| Prussian carp | 1070 | 1 | 2 | 1 | 99.44 | 73.74 | 51.18 | 45.27 | 25.39 | 29.68 | 18.09 | 6.52 | 26.64 | 47.05 | 21.13 | 17.53 | 19.40 | 23.35 | 22.30 | 17.35 |
| Prussian carp | 1071 | 1 | 2 | 1 | 59.92 | 82.47 | 50.56 | 50.01 | 28.29 | 31.76 | 16.84 | 7.84 | 26.71 | 42.97 | 17.62 | 16.38 | 20.52 | 24.47 | 21.66 | 23.08 |
| Prussian carp | 1072 | 1 | 2 | 1 | 60.65 | 81.61 | 49.56 | 47.90 | 30.89 | 30.60 | 16.31 | 8.23 | 26.60 | 42.59 | 17.10 | 16.93 | 20.50 | 24.76 | 23.19 | 20.29 |
| Prussian carp | 1073 | 1 | 2 | 1 | 60.79 | 79.87 | 51.61 | 50.57 | 29.39 | 32.47 | 17.80 | 7.58 | 26.73 | 42.43 | 19.09 | 16.50 | 19.34 | 22.16 | 20.61 | 20.48 |
| Prussian carp | 1074 | 1 | 2 | 1 | 50.02 | 77.18 | 50.50 | 48.85 | 29.53 | 29.28 | 17.36 | 7.73 | 26.31 | 42.34 | 15.97 | 15.99 | 19.31 | 21.98 | 20.64 | 18.15 |
| Prussian carp | 1075 | 1 | 2 | 1 | 48.15 | 82.59 | 52.64 | 49.37 | 29.26 | 32.56 | 17.51 | 8.12 | 28.96 | 45.52 | 17.42 | 18.36 | 21.28 | 24.94 | 21.06 | 20.59 |
| Prussian carp | 1076 | 1 | 2 | 1 | 58.35 | 79.20 | 50.21 | 50.57 | 28.88 | 31.34 | 16.75 | 7.12 | 25.70 | 44.48 | 19.13 | 17.14 | 20.54 | 24.48 | 25.91 | 23.16 |
| Prussian carp | 1077 | 1 | 2 | 1 | 54.42 | 80.15 | 49.38 | 46.13 | 30.30 | 28.75 | 16.72 | 7.59 | 25.75 | 43.51 | 18.19 | 15.21 | 20.97 | 24.43 | 21.20 | 19.30 |
| Prussian carp | 1078 | 1 | 2 | 1 | 49.82 | 78.17 | 51.96 | 49.37 | 26.55 | 29.96 | 17.01 | 6.90 | 28.30 | 47.29 | 18.37 | 17.19 | 20.82 | 22.91 | 22.87 | 20.83 |
| Prussian carp | 1079 | 1 | 2 | 1 | 47.31 | 80.98 | 47.91 | 48.22 | 28.07 | 31.12 | 16.53 | 6.19 | 27.53 | 47.45 | 16.90 | 17.69 | 21.47 | 22.29 | 24.75 | 19.52 |
| Prussian carp | 1080 | 1 | 2 | 1 | 49.00 | 79.33 | 52.52 | 46.82 | 28.13 | 31.51 | 18.17 | 7.72 | 27.70 | 44.82 | 18.55 | 17.23 | 21.13 | 23.89 | 22.62 | 21.81 |
| Prussian carp | 1081 | 1 | 2 | 1 | 48.34 | 78.50 | 50.04 | 49.62 | 29.63 | 31.95 | 17.09 | 7.83 | 29.82 | 44.78 | 20.30 | 16.40 | 20.02 | 23.53 | 22.02 | 21.10 |
| Prussian carp | 1082 | 1 | 2 | 1 | 51.81 | 82.59 | 49.34 | 46.49 | 27.70 | 30.13 | 16.80 | 7.89 | 28.73 | 42.28 | 19.80 | 17.20 | 22.60 | 25.15 | 23.48 | 22.35 |
| Prussian carp | 1083 | 1 | 2 | 1 | 49.92 | 80.09 | 49.83 | 51.86 | 31.61 | 32.24 | 18.98 | 8.59 | 26.95 | 45.73 | 18.29 | 15.11 | 20.53 | 24.21 | 21.47 | 21.54 |
| Prussian carp | 1084 | 1 | 2 | 1 | 57.49 | 79.86 | 47.37 | 52.59 | 31.83 | 32.47 | 17.00 | 8.22 | 26.91 | 44.79 | 18.47 | 16.88 | 19.02 | 25.02 | 21.29 | 19.31 |
| Prussian carp | 1085 | 1 | 2 | 1 | 44.16 | 79.25 | 50.72 | 47.96 | 29.12 | 32.21 | 16.66 | 8.87 | 29.27 | 42.34 | 18.35 | 17.04 | 20.96 | 22.78 | 21.67 | 19.86 |
| Prussian carp | 1086 | 1 | 2 | 1 | 60.58 | 79.55 | 49.18 | 49.38 | 31.08 | 32.88 | 18.23 | 7.30 | 26.23 | 41.79 | 17.42 | 18.86 | 21.72 | 21.91 | 22.98 | 19.49 |
| Prussian carp | 1087 | 1 | 2 | 1 | 47.67 | 82.99 | 50.27 | 51.67 | 29.14 | 30.21 | 16.66 | 5.86 | 28.75 | 47.68 | 17.43 | 17.11 | 20.15 | 22.71 | 21.69 | 21.42 |
| Prussian carp | 1088 | 1 | 2 | 1 | 44.60 | 76.92 | 47.01 | 48.24 | 27.45 | 30.38 | 17.28 | 6.30 | 25.97 | 45.50 | 16.44 | 16.98 | 20.08 | 24.02 | 21.47 | 21.46 |
| Prussian carp | 1089 | 1 | 2 | 1 | 52.60 | 77.29 | 47.73 | 50.93 | 31.30 | 32.29 | 16.91 | 8.51 | 25.76 | 42.69 | 18.71 | 16.25 | 20.00 | 20.28 | 22.51 | 19.77 |
| Prussian carp | 1090 | 1 | 2 | 1 | 44.14 | 79.60 | 49.29 | 48.95 | 25.74 | 30.28 | 16.23 | 7.94 | 26.86 | 43.71 | 18.59 | 17.31 | 21.18 | 23.47 | 23.18 | 20.03 |
| Prussian carp | 1061 | 1 | 2 | 2 | 108.60 | 78.63 | 51.55 | 46.64 | 27.23 | 31.90 | 16.46 | 6.23 | 26.49 | 42.01 | 16.79 | 16.77 | 19.96 | 22.22 | 20.36 | 19.22 |
| Prussian carp | 1062 | 1 | 2 | 2 | 106.47 | 79.96 | 44.81 | 47.96 | 28.37 | 30.84 | 16.38 | 7.14 | 26.74 | 41.13 | 15.94 | 18.12 | 19.68 | 20.53 | 21.53 | 19.55 |
| Prussian carp | 1063 | 1 | 2 | 2 | 107.60 | 79.95 | 47.18 | 51.19 | 30.50 | 29.95 | 16.24 | 7.54 | 25.16 | 42.50 | 18.64 | 15.89 | 20.66 | 22.19 | 21.00 | 19.78 |
| Prussian carp | 1064 | 1 | 2 | 2 | 71.85 | 79.04 | 47.77 | 49.11 | 29.28 | 31.08 | 16.34 | 7.46 | 26.14 | 43.39 | 17.73 | 17.84 | 20.36 | 22.28 | 21.64 | 21.31 |
| Prussian carp | 1065 | 1 | 2 | 2 | 95.65 | 80.87 | 50.07 | 50.99 | 28.39 | 31.27 | 17.54 | 7.52 | 26.82 | 44.84 | 19.72 | 17.11 | 21.41 | 23.61 | 21.84 | 22.35 |
| Prussian carp | 1066 | 1 | 2 | 2 | 93.82 | 79.11 | 48.39 | 47.25 | 27.99 | 30.71 | 16.84 | 7.12 | 25.44 | 40.65 | 16.86 | 16.41 | 18.52 | 21.51 | 20.37 | 18.43 |
| Prussian carp | 1067 | 1 | 2 | 2 | 114.55 | 81.81 | 47.45 | 48.06 | 29.00 | 29.76 | 16.15 | 8.27 | 27.19 | 47.33 | 17.14 | 16.48 | 18.92 | 24.13 | 22.71 | 21.29 |
| Prussian carp | 1068 | 1 | 2 | 2 | 101.34 | 82.72 | 49.15 | 53.12 | 30.67 | 32.07 | 16.49 | 8.32 | 26.99 | 43.46 | 18.46 | 17.83 | 20.50 | 22.77 | 22.69 | 20.08 |
| Prussian carp | 1069 | 1 | 2 | 2 | 59.85 | 80.31 | 50.84 | 51.15 | 29.04 | 32.44 | 16.77 | 6.91 | 27.30 | 43.48 | 18.36 | 17.32 | 17.65 | 21.25 | 21.58 | 20.54 |
| Prussian carp | 1070 | 1 | 2 | 2 | 100.44 | 77.45 | 49.55 | 49.66 | 27.65 | 31.54 | 17.69 | 7.25 | 28.38 | 48.31 | 20.64 | 18.12 | 20.83 | 24.14 | 21.54 | 17.30 |
| Prussian carp | 1071 | 1 | 2 | 2 | 60.02 | 83.25 | 49.80 | 48.55 | 29.18 | 30.48 | 16.50 | 7.19 | 25.69 | 43.41 | 16.89 | 17.46 | 21.29 | 24.33 | 22.26 | 22.04 |
| Prussian carp | 1072 | 1 | 2 | 2 | 60.50 | 80.84 | 47.33 | 50.92 | 28.16 | 31.05 | 16.17 | 7.88 | 26.89 | 42.40 | 16.31 | 16.61 | 20.18 | 23.81 | 22.76 | 19.59 |
| Prussian carp | 1073 | 1 | 2 | 2 | 60.60 | 85.23 | 52.87 | 55.50 | 30.99 | 33.50 | 17.79 | 7.25 | 27.04 | 42.20 | 15.92 | 16.76 | 19.77 | 21.34 | 20.65 | 20.92 |
| Prussian carp | 1074 | 1 | 2 | 2 | 50.18 | 77.60 | 48.89 | 48.39 | 28.98 | 31.00 | 16.68 | 7.63 | 25.29 | 43.19 | 19.42 | 15.98 | 19.64 | 22.40 | 21.29 | 20.19 |
| Prussian carp | 1075 | 1 | 2 | 2 | 48.17 | 81.93 | 51.56 | 51.11 | 28.28 | 31.11 | 17.72 | 7.40 | 27.68 | 45.79 | 17.88 | 18.50 | 20.80 | 23.76 | 21.13 | 20.35 |
| Prussian carp | 1076 | 1 | 2 | 2 | 57.82 | 82.17 | 47.88 | 51.54 | 30.18 | 30.92 | 17.57 | 7.20 | 27.08 | 47.10 | 17.22 | 18.49 | 23.87 | 23.34 | 25.43 | 24.25 |
| Prussian carp | 1077 | 1 | 2 | 2 | 54.79 | 79.66 | 48.61 | 46.10 | 26.56 | 28.59 | 16.29 | 7.86 | 27.04 | 42.78 | 16.59 | 17.87 | 18.95 | 22.42 | 22.52 | 22.09 |
| Prussian carp | 1078 | 1 | 2 | 2 | 49.69 | 82.95 | 51.84 | 50.60 | 28.37 | 31.18 | 16.91 | 6.90 | 28.27 | 47.65 | 17.59 | 18.91 | 21.36 | 22.51 | 24.05 | 21.80 |
| Prussian carp | 1079 | 1 | 2 | 2 | 48.01 | 82.70 | 49.58 | 50.19 | 28.38 | 31.19 | 15.64 | 7.96 | 28.66 | 46.73 | 17.61 | 17.69 | 22.99 | 23.31 | 23.94 | 22.52 |
| Prussian carp | 1080 | 1 | 2 | 2 | 48.13 | 78.77 | 46.67 | 48.94 | 26.94 | 31.39 | 16.39 | 7.54 | 27.52 | 44.58 | 17.17 | 17.40 | 21.21 | 23.49 | 21.81 | 22.00 |
| Prussian carp | 1081 | 1 | 2 | 2 | 47.86 | 80.30 | 50.77 | 48.97 | 28.87 | 30.95 | 17.53 | 7.51 | 28.25 | 45.54 | 18.52 | 17.51 | 21.39 | 24.94 | 21.72 | 21.82 |
| Prussian carp | 1082 | 1 | 2 | 2 | 52.33 | 78.79 | 49.01 | 46.80 | 27.83 | 31.51 | 16.38 | 8.19 | 27.61 | 42.00 | 18.95 | 16.64 | 21.93 | 24.20 | 22.44 | 21.47 |
| Prussian carp | 1083 | 1 | 2 | 2 | 49.83 | 81.52 | 47.76 | 52.92 | 28.32 | 29.91 | 16.74 | 6.54 | 26.87 | 45.43 | 17.17 | 17.93 | 23.52 | 23.10 | 21.70 | 20.92 |
| Prussian carp | 1084 | 1 | 2 | 2 | 58.06 | 81.27 | 51.22 | 53.39 | 29.14 | 31.64 | 16.63 | 7.39 | 27.32 | 44.06 | 18.44 | 16.82 | 20.50 | 21.95 | 21.81 | 20.69 |
| Prussian carp | 1085 | 1 | 2 | 2 | 43.66 | 79.64 | 49.98 | 52.77 | 28.36 | 30.35 | 15.67 | 8.25 | 26.32 | 43.44 | 17.42 | 16.80 | 21.02 | 22.57 | 22.43 | 21.19 |
| Prussian carp | 1086 | 1 | 2 | 2 | 60.09 | 78.41 | 52.48 | 48.36 | 28.28 | 31.67 | 17.45 | 6.36 | 27.71 | 41.46 | 18.41 | 17.84 | 19.80 | 24.38 | 22.49 | 20.33 |
| Prussian carp | 1087 | 1 | 2 | 2 | 47.51 | 81.49 | 49.54 | 48.03 | 27.23 | 28.11 | 17.02 | 6.80 | 29.02 | 46.89 | 16.70 | 16.54 | 20.25 | 23.78 | 22.47 | 20.73 |
| Prussian carp | 1088 | 1 | 2 | 2 | 46.06 | 76.43 | 47.00 | 49.00 | 28.97 | 30.68 | 17.83 | 7.23 | 25.31 | 45.34 | 19.14 | 16.42 | 18.67 | 24.71 | 22.13 | 21.25 |
| Prussian carp | 1089 | 1 | 2 | 2 | 54.89 | 79.15 | 46.43 | 50.36 | 28.27 | 32.26 | 17.03 | 7.91 | 26.21 | 44.55 | 17.69 | 16.18 | 20.85 | 24.55 | 21.85 | 22.91 |
| Prussian carp | 1090 | 1 | 2 | 2 | 45.80 | 81.26 | 47.44 | 50.53 | 27.93 | 29.98 | 16.06 | 7.60 | 26.37 | 43.72 | 17.04 | 16.70 | 21.50 | 22.62 | 22.32 | 21.17 |
| Prussian carp | 1061 | 1 | 2 | 3 | 106.83 | 79.52 | 53.80 | 50.92 | 29.46 | 32.04 | 16.71 | 7.10 | 27.14 | 42.70 | 18.72 | 16.90 | 18.98 | 22.04 | 21.62 | 20.50 |
| Prussian carp | 1062 | 1 | 2 | 3 | 106.78 | 79.20 | 45.21 | 45.49 | 26.66 | 29.53 | 15.45 | 6.55 | 26.21 | 40.58 | 17.84 | 17.19 | 19.18 | 21.31 | 20.89 | 18.98 |
| Prussian carp | 1063 | 1 | 2 | 3 | 107.35 | 78.50 | 50.04 | 47.94 | 28.18 | 31.23 | 15.56 | 8.78 | 26.65 | 44.07 | 16.25 | 16.51 | 20.87 | 22.50 | 21.15 | 19.79 |
| Prussian carp | 1064 | 1 | 2 | 3 | 70.27 | 80.17 | 46.81 | 50.81 | 28.43 | 30.93 | 15.76 | 7.60 | 25.85 | 43.04 | 18.30 | 17.21 | 21.55 | 21.50 | 21.86 | 21.13 |
| Prussian carp | 1065 | 1 | 2 | 3 | 95.91 | 78.50 | 48.95 | 46.69 | 27.86 | 31.46 | 16.39 | 8.37 | 26.46 | 43.98 | 14.48 | 16.92 | 20.29 | 24.42 | 23.03 | 21.32 |
| Prussian carp | 1066 | 1 | 2 | 3 | 93.66 | 79.98 | 48.85 | 50.25 | 28.62 | 31.15 | 16.94 | 7.74 | 27.70 | 42.07 | 18.70 | 16.13 | 20.27 | 22.35 | 22.24 | 18.33 |
| Prussian carp | 1067 | 1 | 2 | 3 | 115.36 | 81.05 | 49.32 | 50.11 | 27.35 | 30.73 | 16.18 | 7.11 | 25.14 | 46.22 | 17.85 | 16.82 | 20.46 | 24.81 | 21.53 | 21.09 |
| Prussian carp | 1068 | 1 | 2 | 3 | 101.76 | 80.54 | 51.31 | 50.41 | 29.74 | 32.21 | 16.84 | 8.03 | 26.24 | 42.15 | 18.18 | 17.65 | 20.15 | 23.62 | 21.73 | 22.56 |
| Prussian carp | 1069 | 1 | 2 | 3 | 60.48 | 79.33 | 50.05 | 50.03 | 29.56 | 31.29 | 16.51 | 7.29 | 28.34 | 43.49 | 17.77 | 17.49 | 20.35 | 21.03 | 21.20 | 20.31 |
| Prussian carp | 1070 | 1 | 2 | 3 | 99.23 | 75.48 | 47.87 | 48.60 | 28.16 | 31.22 | 18.22 | 6.68 | 26.64 | 47.77 | 20.79 | 17.38 | 20.07 | 22.80 | 23.65 | 17.33 |
| Prussian carp | 1071 | 1 | 2 | 3 | 59.22 | 81.96 | 49.68 | 50.69 | 28.69 | 30.75 | 16.43 | 7.84 | 26.63 | 43.11 | 14.91 | 17.37 | 19.59 | 23.91 | 24.09 | 22.43 |
| Prussian carp | 1072 | 1 | 2 | 3 | 61.10 | 80.29 | 47.15 | 51.12 | 29.11 | 31.76 | 16.63 | 6.69 | 26.23 | 42.56 | 16.63 | 16.28 | 19.33 | 24.90 | 20.65 | 19.63 |
| Prussian carp | 1073 | 1 | 2 | 3 | 60.28 | 82.70 | 51.59 | 51.40 | 29.36 | 29.81 | 17.63 | 8.00 | 25.95 | 41.70 | 17.27 | 16.62 | 19.27 | 21.70 | 20.33 | 20.62 |
| Prussian carp | 1074 | 1 | 2 | 3 | 50.21 | 77.81 | 50.58 | 45.93 | 26.92 | 30.28 | 16.70 | 6.75 | 25.27 | 42.69 | 14.14 | 15.30 | 21.05 | 23.12 | 21.77 | 19.79 |
| Prussian carp | 1075 | 1 | 2 | 3 | 47.81 | 84.55 | 51.52 | 48.94 | 28.84 | 31.37 | 17.10 | 7.09 | 27.96 | 46.55 | 17.41 | 18.31 | 18.76 | 21.64 | 21.89 | 20.95 |
| Prussian carp | 1076 | 1 | 2 | 3 | 57.59 | 80.26 | 47.80 | 51.74 | 28.75 | 32.07 | 16.26 | 7.25 | 27.02 | 46.75 | 16.47 | 17.06 | 21.82 | 26.13 | 25.18 | 23.01 |
| Prussian carp | 1077 | 1 | 2 | 3 | 53.59 | 80.24 | 48.60 | 46.80 | 28.20 | 29.52 | 15.34 | 7.39 | 27.11 | 42.67 | 15.16 | 17.79 | 19.76 | 23.57 | 20.60 | 20.61 |
| Prussian carp | 1078 | 1 | 2 | 3 | 49.50 | 80.45 | 49.32 | 52.20 | 27.37 | 31.62 | 16.86 | 6.77 | 28.82 | 47.19 | 15.24 | 18.97 | 20.97 | 21.75 | 21.57 | 18.43 |
| Prussian carp | 1079 | 1 | 2 | 3 | 48.13 | 82.62 | 50.08 | 46.98 | 26.45 | 29.13 | 16.27 | 5.72 | 28.83 | 46.09 | 18.21 | 16.50 | 22.69 | 24.75 | 24.97 | 22.42 |
| Prussian carp | 1080 | 1 | 2 | 3 | 49.63 | 81.71 | 51.05 | 48.01 | 26.76 | 31.69 | 16.58 | 7.97 | 24.77 | 45.01 | 17.76 | 16.99 | 21.65 | 22.54 | 23.74 | 21.96 |
| Prussian carp | 1081 | 1 | 2 | 3 | 47.74 | 79.32 | 53.18 | 45.46 | 27.46 | 30.95 | 17.82 | 6.78 | 26.99 | 45.46 | 17.14 | 16.81 | 19.54 | 26.17 | 21.59 | 19.24 |
| Prussian carp | 1082 | 1 | 2 | 3 | 51.97 | 77.66 | 48.66 | 44.88 | 25.43 | 30.25 | 16.67 | 6.25 | 26.68 | 42.84 | 19.21 | 16.56 | 20.68 | 23.66 | 22.04 | 21.94 |
| Prussian carp | 1083 | 1 | 2 | 3 | 49.89 | 80.38 | 51.81 | 51.01 | 29.72 | 32.08 | 17.66 | 6.90 | 28.09 | 46.26 | 17.99 | 18.26 | 20.29 | 24.00 | 20.22 | 19.23 |
| Prussian carp | 1084 | 1 | 2 | 3 | 57.55 | 82.80 | 50.13 | 52.36 | 31.12 | 32.70 | 15.45 | 7.35 | 28.25 | 44.65 | 19.66 | 16.47 | 20.67 | 22.63 | 21.28 | 21.13 |
| Prussian carp | 1085 | 1 | 2 | 3 | 45.39 | 80.43 | 54.35 | 49.14 | 27.20 | 29.75 | 15.91 | 6.93 | 26.36 | 41.62 | 16.12 | 16.95 | 22.36 | 22.66 | 22.70 | 19.18 |
| Prussian carp | 1086 | 1 | 2 | 3 | 60.88 | 79.71 | 50.11 | 46.39 | 26.67 | 31.58 | 17.12 | 7.61 | 26.25 | 41.97 | 17.51 | 17.43 | 21.54 | 23.72 | 23.11 | 20.71 |
| Prussian carp | 1087 | 1 | 2 | 3 | 47.50 | 80.44 | 50.11 | 48.21 | 29.25 | 31.25 | 16.34 | 7.07 | 27.56 | 47.86 | 17.51 | 18.18 | 19.77 | 22.53 | 21.24 | 21.95 |
| Prussian carp | 1088 | 1 | 2 | 3 | 45.65 | 80.51 | 49.10 | 48.45 | 26.32 | 30.48 | 17.38 | 6.89 | 24.94 | 45.47 | 18.51 | 16.44 | 19.80 | 21.46 | 22.64 | 19.53 |
| Prussian carp | 1089 | 1 | 2 | 3 | 53.65 | 79.70 | 48.76 | 50.27 | 29.34 | 32.89 | 17.55 | 7.72 | 27.18 | 45.09 | 16.52 | 16.32 | 21.41 | 20.59 | 22.85 | 21.11 |
| Prussian carp | 1090 | 1 | 2 | 3 | 44.20 | 81.71 | 50.02 | 47.75 | 28.61 | 30.53 | 15.72 | 7.45 | 25.99 | 43.37 | 19.25 | 17.18 | 21.24 | 24.32 | 22.63 | 20.94 |
| Prussian carp | 1061 | 1 | 3 | 1 | 134.46 | 79.525 | 48.725 | 48.224 | 31.932 | 31.990 | 16.193 | 8.590 | 26.751 | 43.869 | 17.238 | 16.955 | 20.548 | 21.749 | 20.932 | 21.28407 |
| Prussian carp | 1062 | 1 | 3 | 1 | 154.39 | 78.280 | 48.728 | 48.204 | 29.810 | 31.780 | 16.607 | 8.836 | 26.631 | 41.661 | 16.413 | 18.137 | 20.530 | 21.932 | 21.801 | 20.65571 |
| Prussian carp | 1063 | 1 | 3 | 1 | 152.74 | 79.802 | 50.361 | 49.933 | 30.760 | 31.143 | 16.679 | 8.561 | 26.729 | 43.950 | 17.757 | 15.934 | 21.568 | 21.668 | 21.742 | 20.85709 |
| Prussian carp | 1064 | 1 | 3 | 1 | 135.82 | 79.149 | 48.097 | 49.704 | 31.459 | 32.084 | 16.787 | 9.122 | 27.755 | 44.027 | 17.509 | 17.507 | 21.864 | 22.908 | 22.531 | 21.79834 |
| Prussian carp | 1065 | 1 | 3 | 1 | 123.29 | 79.515 | 50.546 | 49.800 | 31.361 | 31.729 | 16.236 | 8.886 | 27.824 | 45.436 | 19.304 | 16.904 | 21.610 | 23.594 | 21.791 | 21.45927 |
| Prussian carp | 1066 | 1 | 3 | 1 | 132.95 | 79.615 | 46.779 | 46.997 | 29.581 | 31.432 | 15.044 | 9.267 | 25.612 | 40.723 | 17.807 | 15.679 | 19.878 | 21.152 | 22.386 | 20.30289 |
| Prussian carp | 1067 | 1 | 3 | 1 | 134.03 | 80.516 | 49.866 | 48.888 | 29.993 | 30.679 | 16.000 | 8.906 | 26.522 | 46.408 | 17.325 | 16.167 | 20.745 | 21.917 | 21.707 | 20.42085 |
| Prussian carp | 1068 | 1 | 3 | 1 | 118.58 | 83.764 | 51.298 | 50.077 | 31.819 | 32.139 | 16.683 | 8.465 | 26.724 | 43.446 | 17.007 | 17.204 | 21.281 | 22.662 | 22.491 | 22.4734 |
| Prussian carp | 1069 | 1 | 3 | 1 | 122.48 | 83.502 | 50.210 | 50.204 | 30.500 | 31.768 | 16.328 | 8.690 | 28.498 | 44.615 | 18.908 | 17.338 | 19.735 | 24.222 | 22.979 | 22.2731 |
| Prussian carp | 1070 | 1 | 3 | 1 | 125.05 | 78.875 | 49.675 | 46.396 | 29.450 | 30.919 | 16.583 | 8.195 | 26.956 | 47.232 | 20.605 | 17.711 | 18.373 | 21.563 | 22.973 | 19.84144 |
| Prussian carp | 1071 | 1 | 3 | 1 | 140.71 | 83.691 | 49.792 | 49.683 | 31.800 | 31.702 | 17.676 | 9.609 | 25.357 | 43.189 | 15.269 | 17.385 | 19.787 | 23.369 | 22.513 | 21.95158 |
| Prussian carp | 1072 | 1 | 3 | 1 | 117.41 | 81.625 | 53.860 | 51.001 | 31.319 | 32.447 | 16.721 | 9.828 | 27.697 | 43.471 | 17.392 | 16.629 | 20.095 | 24.212 | 22.027 | 21.64268 |
| Prussian carp | 1073 | 1 | 3 | 1 | 123.87 | 82.025 | 57.974 | 52.365 | 33.759 | 33.691 | 17.908 | 9.479 | 28.773 | 42.734 | 15.526 | 17.083 | 19.725 | 21.737 | 22.142 | 20.02801 |
| Prussian carp | 1074 | 1 | 3 | 1 | 154.53 | 80.029 | 48.824 | 48.065 | 30.888 | 31.102 | 17.066 | 8.073 | 24.668 | 42.931 | 17.522 | 15.986 | 19.758 | 21.174 | 21.818 | 20.59663 |
| Prussian carp | 1075 | 1 | 3 | 1 | 133.61 | 82.681 | 51.783 | 51.204 | 30.855 | 32.922 | 17.664 | 9.405 | 29.572 | 45.540 | 16.581 | 18.294 | 21.088 | 21.690 | 22.207 | 20.44001 |
| Prussian carp | 1076 | 1 | 3 | 1 | 123.27 | 81.867 | 50.662 | 51.007 | 32.153 | 33.419 | 17.440 | 9.453 | 26.821 | 47.116 | 17.695 | 17.068 | 22.877 | 22.858 | 24.890 | 23.19309 |
| Prussian carp | 1077 | 1 | 3 | 1 | 123.28 | 82.803 | 51.295 | 49.382 | 29.519 | 30.100 | 16.170 | 7.608 | 25.950 | 43.213 | 16.538 | 17.956 | 18.793 | 22.243 | 21.114 | 21.54652 |
| Prussian carp | 1078 | 1 | 3 | 1 | 127.71 | 81.403 | 50.817 | 50.588 | 33.353 | 32.597 | 17.287 | 9.976 | 28.176 | 47.341 | 15.998 | 18.659 | 21.575 | 22.952 | 23.410 | 21.27251 |
| Prussian carp | 1079 | 1 | 3 | 1 | 115.51 | 82.882 | 50.380 | 49.610 | 28.267 | 30.530 | 15.723 | 7.655 | 27.076 | 46.846 | 17.515 | 17.519 | 22.835 | 23.264 | 25.206 | 22.88649 |
| Prussian carp | 1080 | 1 | 3 | 1 | 129.21 | 81.468 | 51.257 | 50.967 | 31.726 | 32.320 | 16.614 | 8.569 | 26.629 | 46.412 | 14.982 | 16.891 | 21.176 | 22.569 | 23.811 | 22.73524 |
| Prussian carp | 1081 | 1 | 3 | 1 | 125.76 | 80.504 | 51.418 | 48.614 | 32.721 | 33.059 | 16.923 | 9.981 | 28.883 | 46.822 | 19.352 | 16.439 | 20.144 | 22.876 | 23.411 | 22.43302 |
| Prussian carp | 1082 | 1 | 3 | 1 | 115.85 | 81.081 | 48.993 | 46.795 | 29.967 | 31.399 | 16.475 | 8.747 | 27.431 | 43.086 | 17.779 | 17.062 | 22.243 | 24.158 | 23.600 | 22.16515 |
| Prussian carp | 1083 | 1 | 3 | 1 | 126.44 | 81.785 | 49.061 | 49.686 | 32.221 | 33.771 | 16.957 | 8.121 | 26.554 | 46.773 | 17.047 | 18.252 | 21.869 | 23.478 | 22.720 | 21.39323 |
| Prussian carp | 1084 | 1 | 3 | 1 | 128.56 | 81.507 | 51.828 | 49.781 | 32.256 | 32.119 | 17.670 | 9.865 | 28.087 | 45.274 | 17.733 | 16.808 | 20.939 | 22.471 | 21.917 | 20.36823 |
| Prussian carp | 1085 | 1 | 3 | 1 | 138.92 | 78.350 | 49.402 | 47.889 | 28.695 | 30.476 | 15.097 | 8.467 | 26.013 | 41.721 | 16.928 | 16.545 | 20.672 | 20.175 | 21.818 | 19.66841 |
| Prussian carp | 1086 | 1 | 3 | 1 | 141.16 | 78.396 | 48.450 | 48.951 | 30.863 | 32.514 | 17.396 | 9.692 | 26.652 | 41.660 | 18.355 | 16.975 | 21.185 | 23.828 | 23.113 | 21.68819 |
| Prussian carp | 1087 | 1 | 3 | 1 | 117.63 | 81.525 | 48.524 | 49.215 | 30.348 | 30.863 | 16.404 | 7.045 | 27.573 | 46.850 | 17.776 | 16.880 | 20.593 | 22.312 | 21.710 | 20.4358 |
| Prussian carp | 1088 | 1 | 3 | 1 | 139.92 | 78.113 | 48.097 | 47.822 | 31.244 | 31.137 | 16.857 | 7.507 | 26.825 | 45.196 | 17.360 | 16.528 | 19.454 | 22.455 | 22.233 | 21.85278 |
| Prussian carp | 1089 | 1 | 3 | 1 | 127.53 | 80.001 | 50.147 | 49.312 | 30.969 | 32.121 | 16.456 | 9.133 | 27.049 | 43.829 | 20.299 | 16.149 | 20.663 | 22.049 | 22.925 | 21.7094 |
| Prussian carp | 1090 | 1 | 3 | 1 | 145.55 | 81.867 | 48.320 | 48.021 | 30.608 | 31.576 | 16.551 | 8.582 | 26.106 | 43.462 | 18.391 | 16.420 | 21.291 | 24.437 | 23.141 | 20.97731 |
| Prussian carp | 1061 | 1 | 3 | 2 | 136.64 | 79.150 | 50.276 | 48.650 | 30.646 | 32.079 | 16.495 | 9.552 | 26.554 | 43.766 | 16.668 | 17.213 | 20.653 | 22.838 | 20.109 | 20.96755 |
| Prussian carp | 1062 | 1 | 3 | 2 | 155.46 | 81.607 | 48.352 | 46.093 | 29.177 | 31.838 | 16.758 | 8.406 | 25.290 | 41.361 | 16.630 | 17.757 | 20.043 | 22.612 | 21.952 | 19.91733 |
| Prussian carp | 1063 | 1 | 3 | 2 | 153.34 | 80.896 | 49.866 | 49.303 | 30.030 | 31.186 | 16.680 | 8.653 | 26.871 | 43.913 | 18.099 | 15.928 | 21.457 | 21.295 | 22.422 | 20.05587 |
| Prussian carp | 1064 | 1 | 3 | 2 | 137.45 | 81.034 | 48.799 | 49.545 | 31.016 | 32.407 | 16.406 | 8.601 | 27.413 | 44.153 | 17.286 | 17.424 | 21.586 | 22.228 | 22.235 | 21.50893 |
| Prussian carp | 1065 | 1 | 3 | 2 | 120.68 | 82.133 | 49.171 | 50.134 | 30.629 | 31.393 | 16.244 | 9.446 | 26.741 | 45.192 | 17.635 | 16.542 | 21.543 | 23.692 | 21.494 | 21.26654 |
| Prussian carp | 1066 | 1 | 3 | 2 | 131.60 | 79.960 | 47.723 | 47.887 | 29.255 | 31.257 | 15.812 | 9.225 | 25.567 | 40.521 | 17.938 | 15.667 | 19.667 | 21.567 | 21.870 | 19.58647 |
| Prussian carp | 1067 | 1 | 3 | 2 | 131.26 | 83.114 | 51.149 | 48.746 | 28.879 | 30.678 | 16.076 | 8.389 | 26.839 | 46.890 | 17.541 | 16.796 | 20.920 | 21.526 | 22.228 | 20.78046 |
| Prussian carp | 1068 | 1 | 3 | 2 | 117.45 | 83.003 | 48.796 | 48.784 | 30.147 | 31.563 | 16.186 | 8.905 | 26.340 | 42.872 | 16.413 | 17.623 | 21.200 | 21.753 | 22.982 | 22.36741 |
| Prussian carp | 1069 | 1 | 3 | 2 | 121.86 | 78.005 | 49.031 | 47.512 | 28.406 | 30.951 | 16.270 | 7.945 | 28.678 | 43.064 | 19.181 | 16.385 | 20.565 | 21.696 | 21.799 | 20.8449 |
| Prussian carp | 1070 | 1 | 3 | 2 | 125.58 | 78.779 | 54.355 | 47.960 | 29.918 | 30.458 | 17.281 | 7.813 | 27.005 | 47.405 | 19.919 | 17.068 | 18.218 | 20.760 | 22.967 | 21.79274 |
| Prussian carp | 1071 | 1 | 3 | 2 | 146.48 | 85.403 | 54.293 | 51.059 | 31.562 | 33.221 | 16.258 | 10.307 | 26.307 | 43.756 | 14.274 | 17.793 | 20.428 | 23.022 | 21.706 | 22.51354 |
| Prussian carp | 1072 | 1 | 3 | 2 | 118.81 | 81.062 | 53.070 | 50.460 | 31.074 | 31.638 | 15.986 | 8.976 | 26.018 | 42.711 | 16.509 | 16.091 | 19.647 | 21.820 | 22.157 | 21.01194 |
| Prussian carp | 1073 | 1 | 3 | 2 | 125.88 | 80.617 | 54.395 | 49.873 | 31.057 | 32.496 | 17.706 | 8.997 | 26.651 | 42.277 | 17.115 | 16.872 | 19.463 | 21.046 | 21.679 | 20.0623 |
| Prussian carp | 1074 | 1 | 3 | 2 | 150.97 | 80.365 | 49.936 | 47.049 | 29.430 | 31.356 | 15.972 | 8.498 | 25.932 | 41.977 | 16.989 | 15.477 | 19.882 | 21.562 | 21.033 | 19.71319 |
| Prussian carp | 1075 | 1 | 3 | 2 | 130.45 | 79.731 | 49.652 | 47.986 | 28.673 | 30.620 | 16.572 | 8.786 | 25.950 | 43.765 | 16.244 | 17.474 | 20.238 | 20.869 | 21.190 | 20.27394 |
| Prussian carp | 1076 | 1 | 3 | 2 | 124.89 | 80.322 | 51.550 | 51.160 | 32.205 | 32.900 | 17.589 | 9.075 | 26.724 | 46.394 | 17.890 | 16.743 | 23.115 | 22.641 | 25.499 | 22.96629 |
| Prussian carp | 1077 | 1 | 3 | 2 | 123.74 | 83.439 | 50.888 | 50.008 | 29.577 | 30.415 | 16.425 | 8.194 | 25.457 | 42.667 | 17.551 | 17.988 | 19.791 | 21.103 | 22.607 | 22.14105 |
| Prussian carp | 1078 | 1 | 3 | 2 | 128.92 | 80.254 | 52.363 | 51.635 | 31.349 | 31.761 | 17.138 | 8.756 | 27.716 | 42.213 | 16.286 | 18.496 | 20.975 | 20.947 | 22.673 | 22.77118 |
| Prussian carp | 1079 | 1 | 3 | 2 | 112.92 | 83.155 | 50.073 | 49.381 | 29.423 | 30.763 | 15.742 | 7.891 | 26.706 | 46.517 | 17.223 | 17.271 | 21.841 | 25.275 | 24.402 | 24.21529 |
| Prussian carp | 1080 | 1 | 3 | 2 | 129.29 | 82.642 | 52.266 | 51.208 | 30.851 | 31.553 | 17.207 | 9.035 | 27.051 | 44.933 | 14.059 | 17.243 | 21.426 | 22.817 | 23.189 | 23.09397 |
| Prussian carp | 1081 | 1 | 3 | 2 | 126.43 | 82.851 | 51.199 | 51.146 | 33.580 | 33.414 | 17.360 | 9.426 | 31.150 | 46.790 | 17.862 | 16.384 | 21.548 | 22.980 | 22.709 | 21.12126 |
| Prussian carp | 1082 | 1 | 3 | 2 | 117.10 | 78.550 | 47.639 | 46.503 | 29.795 | 31.133 | 15.798 | 8.556 | 26.278 | 42.762 | 18.886 | 16.721 | 21.391 | 22.548 | 23.115 | 20.11093 |
| Prussian carp | 1083 | 1 | 3 | 2 | 127.24 | 83.150 | 49.000 | 49.926 | 31.672 | 33.798 | 16.687 | 9.418 | 27.309 | 46.729 | 18.726 | 18.182 | 22.142 | 22.518 | 22.376 | 20.49524 |
| Prussian carp | 1084 | 1 | 3 | 2 | 121.82 | 81.394 | 50.273 | 50.343 | 31.541 | 31.690 | 16.810 | 8.730 | 27.587 | 44.604 | 17.788 | 16.255 | 20.937 | 20.036 | 21.555 | 20.91325 |
| Prussian carp | 1085 | 1 | 3 | 2 | 138.49 | 79.583 | 51.599 | 48.410 | 29.053 | 30.544 | 15.878 | 9.102 | 25.183 | 41.767 | 18.007 | 16.640 | 19.117 | 20.028 | 21.085 | 19.29175 |
| Prussian carp | 1086 | 1 | 3 | 2 | 141.94 | 80.386 | 49.558 | 49.942 | 30.028 | 32.787 | 17.306 | 9.377 | 27.129 | 42.649 | 18.611 | 17.128 | 21.649 | 22.061 | 23.805 | 22.58456 |
| Prussian carp | 1087 | 1 | 3 | 2 | 119.09 | 81.614 | 47.975 | 48.998 | 28.773 | 30.232 | 17.032 | 6.549 | 27.264 | 46.745 | 15.782 | 16.832 | 20.733 | 21.633 | 22.409 | 20.66786 |
| Prussian carp | 1088 | 1 | 3 | 2 | 140.03 | 78.988 | 47.572 | 48.120 | 29.569 | 30.129 | 16.783 | 7.640 | 25.144 | 44.693 | 19.344 | 16.292 | 19.877 | 21.363 | 22.200 | 21.69339 |
| Prussian carp | 1089 | 1 | 3 | 2 | 129.95 | 81.760 | 47.203 | 47.123 | 29.681 | 32.313 | 16.372 | 8.799 | 26.179 | 43.277 | 18.638 | 15.746 | 21.364 | 21.921 | 22.407 | 21.72472 |
| Prussian carp | 1090 | 1 | 3 | 2 | 148.42 | 79.787 | 48.366 | 49.001 | 29.345 | 30.619 | 16.382 | 8.795 | 25.538 | 43.288 | 17.406 | 18.362 | 21.735 | 22.866 | 23.451 | 21.88161 |
| Prussian carp | 1061 | 1 | 3 | 3 | 136.68 | 80.595 | 49.571 | 48.272 | 48.364 | 32.289 | 15.935 | 9.529 | 26.914 | 43.355 | 17.962 | 16.936 | 20.768 | 22.955 | 20.377 | 20.8145 |
| Prussian carp | 1062 | 1 | 3 | 3 | 154.90 | 81.287 | 47.847 | 46.970 | 47.356 | 31.239 | 16.888 | 8.604 | 25.756 | 41.469 | 17.293 | 17.820 | 21.533 | 21.694 | 21.856 | 20.51664 |
| Prussian carp | 1063 | 1 | 3 | 3 | 153.35 | 79.699 | 50.932 | 50.377 | 50.651 | 32.061 | 17.253 | 9.453 | 27.011 | 44.128 | 18.821 | 15.806 | 21.503 | 21.219 | 21.802 | 20.85188 |
| Prussian carp | 1064 | 1 | 3 | 3 | 135.90 | 81.666 | 49.391 | 49.544 | 49.795 | 31.625 | 17.035 | 9.272 | 27.596 | 44.301 | 18.744 | 17.524 | 21.403 | 22.864 | 23.169 | 21.7533 |
| Prussian carp | 1065 | 1 | 3 | 3 | 120.04 | 82.099 | 48.844 | 47.856 | 47.982 | 31.062 | 16.183 | 9.391 | 26.753 | 44.403 | 17.533 | 16.637 | 21.910 | 23.492 | 22.188 | 21.65709 |
| Prussian carp | 1066 | 1 | 3 | 3 | 132.86 | 79.521 | 47.209 | 46.059 | 46.308 | 31.877 | 15.523 | 9.406 | 26.411 | 40.681 | 19.140 | 15.800 | 19.493 | 20.729 | 21.792 | 21.18476 |
| Prussian carp | 1067 | 1 | 3 | 3 | 133.05 | 81.412 | 51.127 | 48.717 | 49.131 | 31.566 | 16.574 | 9.958 | 28.422 | 46.346 | 18.256 | 16.380 | 21.134 | 22.171 | 22.680 | 20.96389 |
| Prussian carp | 1068 | 1 | 3 | 3 | 116.82 | 82.574 | 49.849 | 50.008 | 50.398 | 32.103 | 17.270 | 9.418 | 27.792 | 42.792 | 16.710 | 17.637 | 20.535 | 21.470 | 22.045 | 21.62163 |
| Prussian carp | 1069 | 1 | 3 | 3 | 122.15 | 81.443 | 49.559 | 48.396 | 48.406 | 31.193 | 16.619 | 8.917 | 27.803 | 43.335 | 19.807 | 16.803 | 20.911 | 22.895 | 22.522 | 21.55358 |
| Prussian carp | 1070 | 1 | 3 | 3 | 124.57 | 77.729 | 51.447 | 47.546 | 47.613 | 30.567 | 16.962 | 8.730 | 28.306 | 47.474 | 20.834 | 17.223 | 19.976 | 21.587 | 21.884 | 20.99672 |
| Prussian carp | 1071 | 1 | 3 | 3 | 146.30 | 85.553 | 50.739 | 49.902 | 50.311 | 32.076 | 16.872 | 10.141 | 25.969 | 42.912 | 15.047 | 17.556 | 19.837 | 22.586 | 22.131 | 22.20921 |
| Prussian carp | 1072 | 1 | 3 | 3 | 117.01 | 81.257 | 51.654 | 48.003 | 48.483 | 32.053 | 16.266 | 8.924 | 25.080 | 43.318 | 16.836 | 16.684 | 18.576 | 22.251 | 21.792 | 22.55269 |
| Prussian carp | 1073 | 1 | 3 | 3 | 124.53 | 82.610 | 54.778 | 51.373 | 51.309 | 32.856 | 18.317 | 8.997 | 26.556 | 42.131 | 16.121 | 16.580 | 19.657 | 21.324 | 21.167 | 20.64669 |
| Prussian carp | 1074 | 1 | 3 | 3 | 153.78 | 79.841 | 48.266 | 47.579 | 47.933 | 31.015 | 17.005 | 8.251 | 26.048 | 42.349 | 16.732 | 15.403 | 19.633 | 20.951 | 21.755 | 21.46342 |
| Prussian carp | 1075 | 1 | 3 | 3 | 130.76 | 83.156 | 54.517 | 51.009 | 51.325 | 31.603 | 17.392 | 8.963 | 26.887 | 45.169 | 17.031 | 18.666 | 20.767 | 21.245 | 21.860 | 20.64737 |
| Prussian carp | 1076 | 1 | 3 | 3 | 125.38 | 80.116 | 51.543 | 51.257 | 51.507 | 32.398 | 17.991 | 9.165 | 27.003 | 45.521 | 17.741 | 16.974 | 23.565 | 23.945 | 25.472 | 23.98651 |
| Prussian carp | 1077 | 1 | 3 | 3 | 124.74 | 81.407 | 51.206 | 50.501 | 51.015 | 30.416 | 16.555 | 8.645 | 27.001 | 43.119 | 15.976 | 18.449 | 19.311 | 21.599 | 21.946 | 22.71214 |
| Prussian carp | 1078 | 1 | 3 | 3 | 127.38 | 82.006 | 54.827 | 52.599 | 52.693 | 32.273 | 17.934 | 9.770 | 27.489 | 47.737 | 16.438 | 18.512 | 21.934 | 21.995 | 23.487 | 20.61573 |
| Prussian carp | 1079 | 1 | 3 | 3 | 114.03 | 82.129 | 48.429 | 47.039 | 47.332 | 29.874 | 15.782 | 7.534 | 27.202 | 45.480 | 15.720 | 17.432 | 22.491 | 24.662 | 24.804 | 22.58528 |
| Prussian carp | 1080 | 1 | 3 | 3 | 129.75 | 82.621 | 50.396 | 48.935 | 49.262 | 32.277 | 16.995 | 9.320 | 26.485 | 45.080 | 15.351 | 17.169 | 21.109 | 23.166 | 23.697 | 22.7983 |
| Prussian carp | 1081 | 1 | 3 | 3 | 124.67 | 79.187 | 51.796 | 50.701 | 50.786 | 31.816 | 17.344 | 8.341 | 28.091 | 46.060 | 20.212 | 16.721 | 21.447 | 22.814 | 23.293 | 22.02797 |
| Prussian carp | 1082 | 1 | 3 | 3 | 116.69 | 78.402 | 48.056 | 47.563 | 47.686 | 31.528 | 16.149 | 8.367 | 27.008 | 42.331 | 18.843 | 16.599 | 21.839 | 24.317 | 23.218 | 22.084 |
| Prussian carp | 1083 | 1 | 3 | 3 | 127.53 | 83.260 | 48.912 | 49.855 | 49.837 | 33.105 | 17.080 | 8.906 | 26.862 | 45.996 | 16.195 | 17.166 | 22.002 | 22.883 | 22.659 | 21.66966 |
| Prussian carp | 1084 | 1 | 3 | 3 | 126.13 | 81.121 | 49.968 | 49.985 | 50.238 | 31.876 | 16.869 | 9.549 | 27.575 | 44.823 | 18.039 | 16.197 | 21.355 | 21.479 | 21.863 | 21.10663 |
| Prussian carp | 1085 | 1 | 3 | 3 | 139.87 | 81.266 | 51.336 | 47.682 | 48.277 | 31.674 | 15.888 | 9.031 | 25.149 | 42.042 | 18.683 | 16.220 | 19.893 | 20.344 | 22.077 | 21.06186 |
| Prussian carp | 1086 | 1 | 3 | 3 | 147.17 | 78.259 | 48.206 | 48.435 | 48.674 | 31.754 | 16.844 | 8.837 | 25.840 | 41.821 | 18.118 | 17.088 | 20.790 | 22.814 | 23.436 | 22.29764 |
| Prussian carp | 1087 | 1 | 3 | 3 | 119.35 | 78.834 | 47.183 | 47.227 | 47.393 | 30.275 | 16.250 | 8.079 | 27.600 | 45.921 | 17.880 | 16.836 | 20.231 | 22.283 | 21.623 | 21.27515 |
| Prussian carp | 1088 | 1 | 3 | 3 | 139.19 | 78.744 | 47.218 | 47.621 | 47.463 | 30.518 | 16.774 | 8.061 | 25.130 | 44.667 | 20.346 | 16.061 | 19.579 | 21.274 | 22.073 | 21.71198 |
| Prussian carp | 1089 | 1 | 3 | 3 | 131.39 | 78.766 | 48.173 | 47.010 | 47.395 | 32.323 | 16.421 | 9.470 | 26.752 | 43.423 | 18.776 | 15.884 | 20.670 | 22.278 | 22.428 | 22.03433 |
| Prussian carp | 1090 | 1 | 3 | 3 | 149.46 | 81.846 | 48.308 | 47.849 | 47.878 | 31.326 | 16.312 | 8.896 | 25.699 | 43.488 | 17.346 | 17.344 | 21.412 | 22.774 | 22.680 | 22.27585 |
| Prussian carp | 1161 | 2 | 1 | 1 | 112.06 | 78.55 | 49.95 | 47.45 | 31.14 | 29.78 | 14.66 | 9.50 | 28.55 | 44.63 | 9.08 | 15.98 | 20.45 | 15.50 | 23.90 | 13.77 |
| Prussian carp | 1162 | 2 | 1 | 1 | 129.62 | 76.87 | 48.74 | 47.20 | 32.93 | 30.93 | 16.09 | 9.92 | 26.76 | 40.27 | 11.50 | 16.24 | 17.95 | 16.93 | 21.51 | 14.07 |
| Prussian carp | 1163 | 2 | 1 | 1 | 123.01 | 76.59 | 50.69 | 51.44 | 30.30 | 30.71 | 16.56 | 8.93 | 28.55 | 39.56 | 9.98 | 15.58 | 15.88 | 14.41 | 20.23 | 14.63 |
| Prussian carp | 1164 | 2 | 1 | 1 | 121.44 | 77.66 | 50.92 | 45.33 | 31.31 | 30.65 | 15.64 | 10.04 | 27.89 | 45.88 | 13.82 | 16.39 | 19.07 | 16.46 | 20.64 | 16.27 |
| Prussian carp | 1165 | 2 | 1 | 1 | 114.28 | 75.65 | 50.13 | 47.41 | 32.01 | 30.94 | 15.50 | 8.91 | 29.30 | 45.15 | 11.27 | 16.90 | 17.86 | 15.72 | 21.58 | 14.99 |
| Prussian carp | 1166 | 2 | 1 | 1 | 120.81 | 79.70 | 52.95 | 49.68 | 32.57 | 30.66 | 15.80 | 8.76 | 27.35 | 40.21 | 10.16 | 15.61 | 19.15 | 17.32 | 21.37 | 15.63 |
| Prussian carp | 1167 | 2 | 1 | 1 | 129.18 | 76.51 | 53.92 | 50.06 | 32.58 | 30.95 | 16.08 | 9.73 | 28.50 | 42.06 | 11.78 | 16.54 | 16.50 | 14.05 | 21.32 | 15.25 |
| Prussian carp | 1168 | 2 | 1 | 1 | 127.98 | 79.17 | 47.31 | 45.95 | 32.36 | 30.03 | 14.95 | 8.97 | 27.65 | 42.14 | 9.83 | 16.80 | 18.95 | 14.06 | 20.99 | 13.48 |
| Prussian carp | 1169 | 2 | 1 | 1 | 107.55 | 79.73 | 52.53 | 49.85 | 32.87 | 31.45 | 16.30 | 7.88 | 29.17 | 47.32 | 9.13 | 17.20 | 19.25 | 18.94 | 24.48 | 16.56 |
| Prussian carp | 1170 | 2 | 1 | 1 | 109.82 | 80.45 | 49.49 | 47.38 | 32.20 | 32.09 | 16.62 | 9.49 | 28.96 | 46.16 | 10.04 | 17.05 | 17.98 | 18.76 | 22.93 | 17.78 |
| Prussian carp | 1171 | 2 | 1 | 1 | 130.43 | 77.43 | 52.19 | 46.67 | 31.98 | 30.39 | 14.78 | 10.33 | 28.43 | 43.70 | 12.39 | 16.89 | 17.07 | 16.48 | 22.12 | 16.81 |
| Prussian carp | 1172 | 2 | 1 | 1 | 135.94 | 78.19 | 50.27 | 47.43 | 30.99 | 30.16 | 13.50 | 8.88 | 27.35 | 42.09 | 10.86 | 15.58 | 20.42 | 17.67 | 23.52 | 17.26 |
| Prussian carp | 1173 | 2 | 1 | 1 | 103.52 | 76.46 | 51.15 | 46.90 | 32.63 | 30.98 | 14.92 | 8.31 | 26.26 | 42.25 | 12.29 | 15.69 | 16.52 | 17.23 | 20.56 | 13.91 |
| Prussian carp | 1174 | 2 | 1 | 1 | 127.69 | 79.98 | 49.52 | 47.50 | 31.30 | 30.79 | 14.73 | 9.04 | 26.82 | 48.59 | 13.93 | 17.02 | 18.76 | 18.41 | 23.27 | 18.12 |
| Prussian carp | 1175 | 2 | 1 | 1 | 124.19 | 79.01 | 49.01 | 47.34 | 32.93 | 31.81 | 15.96 | 10.17 | 27.83 | 47.38 | 9.89 | 16.24 | 17.06 | 17.92 | 23.71 | 16.99 |
| Prussian carp | 1176 | 2 | 1 | 1 | 119.28 | 78.69 | 51.08 | 48.49 | 31.60 | 30.04 | 13.91 | 9.43 | 29.19 | 44.77 | 11.54 | 16.06 | 19.66 | 19.46 | 24.63 | 17.55 |
| Prussian carp | 1177 | 2 | 1 | 1 | 135.56 | 80.06 | 50.55 | 48.64 | 33.16 | 31.75 | 14.95 | 8.84 | 28.25 | 42.74 | 11.86 | 16.17 | 18.14 | 19.18 | 19.35 | 14.15 |
| Prussian carp | 1178 | 2 | 1 | 1 | 112.36 | 78.60 | 54.25 | 50.25 | 32.74 | 31.65 | 14.39 | 9.25 | 28.33 | 43.51 | 12.41 | 17.30 | 20.54 | 16.56 | 23.00 | 15.38 |
| Prussian carp | 1179 | 2 | 1 | 1 | 122.09 | 77.76 | 50.40 | 46.66 | 31.24 | 29.93 | 13.40 | 9.22 | 26.24 | 42.04 | 15.61 | 16.94 | 15.34 | 12.59 | 22.33 | 17.86 |
| Prussian carp | 1180 | 2 | 1 | 1 | 125.25 | 75.87 | 48.38 | 43.80 | 33.13 | 31.43 | 17.40 | 8.62 | 28.18 | 46.59 | 10.97 | 17.40 | 20.25 | 20.64 | 21.69 | 16.63 |
| Prussian carp | 1181 | 2 | 1 | 1 | 113.89 | 76.32 | 50.41 | 47.52 | 33.79 | 30.81 | 14.22 | 8.85 | 28.16 | 43.85 | 14.00 | 16.50 | 16.97 | 17.05 | 20.94 | 17.92 |
| Prussian carp | 1182 | 2 | 1 | 1 | 112.63 | 80.31 | 52.55 | 50.25 | 33.77 | 31.88 | 14.22 | 9.25 | 26.68 | 41.72 | 12.68 | 15.65 | 15.98 | 18.65 | 18.92 | 16.62 |
| Prussian carp | 1183 | 2 | 1 | 1 | 107.7 | 83.33 | 52.82 | 49.09 | 30.71 | 29.36 | 14.25 | 8.47 | 28.02 | 43.03 | 10.68 | 17.53 | 14.28 | 14.49 | 23.46 | 17.49 |
| Prussian carp | 1184 | 2 | 1 | 1 | 119.59 | 77.28 | 51.73 | 46.31 | 32.31 | 31.46 | 15.72 | 9.91 | 28.11 | 40.27 | 12.05 | 16.39 | 16.93 | 19.37 | 20.95 | 14.85 |
| Prussian carp | 1185 | 2 | 1 | 1 | 143.46 | 82.67 | 52.33 | 48.53 | 31.65 | 30.05 | 13.79 | 9.07 | 28.47 | 45.73 | 9.32 | 17.15 | 21.04 | 16.80 | 23.51 | 16.93 |
| Prussian carp | 1186 | 2 | 1 | 1 | 118.89 | 79.46 | 48.61 | 47.77 | 30.78 | 29.71 | 14.90 | 8.20 | 26.72 | 41.85 | 13.19 | 17.38 | 14.42 | 17.92 | 21.29 | 15.23 |
| Prussian carp | 1187 | 2 | 1 | 1 | 114 | 76.47 | 48.57 | 47.03 | 31.22 | 30.09 | 12.22 | 7.95 | 27.17 | 42.17 | 12.94 | 16.75 | 19.39 | 17.24 | 23.22 | 14.92 |
| Prussian carp | 1188 | 2 | 1 | 1 | 97.73 | 81.97 | 52.99 | 51.40 | 34.17 | 32.35 | 16.37 | 9.23 | 28.39 | 50.40 | 9.83 | 17.44 | 19.38 | 21.61 | 23.00 | 16.39 |
| Prussian carp | 1189 | 2 | 1 | 1 | 130.28 | 78.34 | 51.25 | 49.30 | 34.53 | 31.86 | 17.08 | 8.95 | 29.11 | 49.74 | 13.57 | 18.50 | 20.16 | 18.35 | 24.37 | 17.36 |
| Prussian carp | 1190 | 2 | 1 | 1 | 108.38 | 77.88 | 51.54 | 47.15 | 32.92 | 30.39 | 14.60 | 9.20 | 27.34 | 41.58 | 10.75 | 17.08 | 16.92 | 14.26 | 22.93 | 15.49 |
| Prussian carp | 1161 | 2 | 1 | 2 | 113.35 | 77.58 | 49.32 | 47.37 | 31.64 | 29.55 | 15.32 | 9.17 | 28.66 | 42.69 | 12.17 | 16.18 | 19.61 | 11.28 | 22.92 | 18.17 |
| Prussian carp | 1162 | 2 | 1 | 2 | 131.71 | 77.29 | 48.39 | 47.75 | 31.38 | 30.59 | 16.18 | 8.72 | 28.25 | 39.42 | 11.12 | 16.29 | 17.36 | 15.88 | 19.84 | 15.04 |
| Prussian carp | 1163 | 2 | 1 | 2 | 124.73 | 77.34 | 47.69 | 48.12 | 31.35 | 29.56 | 16.08 | 9.37 | 28.54 | 40.34 | 11.42 | 15.53 | 16.48 | 18.27 | 20.44 | 14.22 |
| Prussian carp | 1164 | 2 | 1 | 2 | 121.57 | 78.80 | 51.27 | 48.25 | 31.81 | 30.63 | 14.24 | 8.93 | 28.48 | 44.46 | 11.72 | 16.22 | 17.66 | 16.20 | 19.93 | 15.29 |
| Prussian carp | 1165 | 2 | 1 | 2 | 113.15 | 78.20 | 49.77 | 47.16 | 31.95 | 29.76 | 15.38 | 8.79 | 28.60 | 44.49 | 11.26 | 17.00 | 17.71 | 14.66 | 21.03 | 14.45 |
| Prussian carp | 1166 | 2 | 1 | 2 | 120.27 | 79.14 | 49.61 | 47.49 | 32.61 | 30.24 | 15.74 | 8.29 | 26.72 | 39.42 | 11.61 | 15.41 | 19.67 | 17.98 | 21.13 | 15.72 |
| Prussian carp | 1167 | 2 | 1 | 2 | 130.23 | 76.72 | 53.58 | 49.25 | 33.48 | 30.90 | 15.35 | 9.06 | 26.35 | 42.36 | 12.75 | 16.60 | 16.74 | 16.50 | 21.04 | 15.97 |
| Prussian carp | 1168 | 2 | 1 | 2 | 126.97 | 79.87 | 48.31 | 47.33 | 32.33 | 29.03 | 15.23 | 8.72 | 26.16 | 41.14 | 12.36 | 17.13 | 18.64 | 14.62 | 21.13 | 15.19 |
| Prussian carp | 1169 | 2 | 1 | 2 | 107.91 | 81.86 | 51.63 | 50.48 | 32.89 | 31.06 | 16.47 | 8.60 | 27.34 | 46.58 | 10.26 | 17.28 | 22.07 | 19.54 | 23.87 | 17.90 |
| Prussian carp | 1170 | 2 | 1 | 2 | 107.87 | 80.17 | 50.23 | 48.54 | 32.93 | 31.82 | 16.90 | 9.45 | 29.61 | 46.02 | 11.07 | 17.43 | 19.72 | 20.36 | 22.73 | 18.08 |
| Prussian carp | 1171 | 2 | 1 | 2 | 128.71 | 78.29 | 47.75 | 45.83 | 30.88 | 29.46 | 15.68 | 9.79 | 26.99 | 43.19 | 12.60 | 16.27 | 16.21 | 17.61 | 19.51 | 15.40 |
| Prussian carp | 1172 | 2 | 1 | 2 | 136.54 | 81.12 | 48.96 | 47.06 | 31.86 | 30.28 | 13.67 | 8.48 | 26.96 | 41.68 | 11.13 | 15.31 | 19.05 | 18.04 | 21.62 | 17.79 |
| Prussian carp | 1173 | 2 | 1 | 2 | 102.96 | 79.11 | 49.86 | 45.87 | 31.45 | 29.84 | 15.09 | 9.62 | 26.08 | 42.36 | 12.60 | 16.19 | 18.03 | 20.64 | 20.11 | 15.78 |
| Prussian carp | 1174 | 2 | 1 | 2 | 126.08 | 77.89 | 50.38 | 46.56 | 32.27 | 30.92 | 15.20 | 9.31 | 27.81 | 48.62 | 11.43 | 16.72 | 18.11 | 18.97 | 23.11 | 18.51 |
| Prussian carp | 1175 | 2 | 1 | 2 | 123.98 | 82.04 | 50.64 | 47.36 | 31.51 | 30.60 | 15.71 | 7.27 | 26.22 | 46.18 | 9.51 | 17.54 | 19.68 | 16.09 | 21.87 | 18.17 |
| Prussian carp | 1176 | 2 | 1 | 2 | 117.45 | 80.14 | 50.15 | 48.23 | 31.72 | 30.84 | 15.07 | 8.88 | 27.42 | 45.97 | 15.56 | 15.49 | 21.87 | 17.57 | 23.36 | 18.63 |
| Prussian carp | 1177 | 2 | 1 | 2 | 136.83 | 79.32 | 47.39 | 45.99 | 32.37 | 31.20 | 15.63 | 8.03 | 27.07 | 43.65 | 10.11 | 16.25 | 17.20 | 17.62 | 21.44 | 16.90 |
| Prussian carp | 1178 | 2 | 1 | 2 | 111.12 | 81.48 | 53.65 | 50.29 | 33.76 | 31.20 | 15.71 | 8.83 | 28.34 | 43.13 | 11.14 | 16.69 | 19.06 | 17.57 | 21.05 | 17.10 |
| Prussian carp | 1179 | 2 | 1 | 2 | 122.01 | 75.86 | 50.50 | 47.37 | 29.91 | 29.60 | 15.05 | 7.73 | 29.19 | 43.02 | 14.55 | 17.11 | 19.19 | 13.11 | 18.40 | 15.22 |
| Prussian carp | 1180 | 2 | 1 | 2 | 125.02 | 81.43 | 53.62 | 51.24 | 32.90 | 32.92 | 17.43 | 9.01 | 30.20 | 46.10 | 10.82 | 17.11 | 18.29 | 20.35 | 22.69 | 15.87 |
| Prussian carp | 1181 | 2 | 1 | 2 | 111.95 | 79.78 | 55.05 | 52.52 | 32.37 | 31.09 | 15.10 | 9.23 | 26.99 | 43.44 | 10.41 | 17.17 | 20.50 | 18.77 | 20.87 | 18.51 |
| Prussian carp | 1182 | 2 | 1 | 2 | 113.23 | 80.98 | 51.71 | 49.42 | 33.39 | 31.50 | 13.68 | 9.35 | 27.04 | 41.68 | 11.16 | 15.99 | 17.31 | 16.36 | 19.47 | 15.93 |
| Prussian carp | 1183 | 2 | 1 | 2 | 107.27 | 82.91 | 47.16 | 46.51 | 30.70 | 29.39 | 14.36 | 8.21 | 28.89 | 45.00 | 9.59 | 17.52 | 15.75 | 16.50 | 22.56 | 17.92 |
| Prussian carp | 1184 | 2 | 1 | 2 | 119.51 | 80.64 | 52.02 | 47.72 | 31.45 | 30.02 | 15.43 | 9.09 | 26.56 | 42.29 | 13.78 | 15.09 | 17.07 | 18.87 | 22.14 | 17.68 |
| Prussian carp | 1185 | 2 | 1 | 2 | 141.93 | 82.19 | 50.86 | 49.51 | 30.49 | 29.01 | 14.10 | 9.05 | 29.41 | 45.07 | 10.62 | 17.72 | 20.28 | 16.80 | 22.77 | 17.95 |
| Prussian carp | 1186 | 2 | 1 | 2 | 120.74 | 82.22 | 48.59 | 47.85 | 30.30 | 29.73 | 15.01 | 8.62 | 27.49 | 42.75 | 14.37 | 17.76 | 12.09 | 17.57 | 22.81 | 18.65 |
| Prussian carp | 1187 | 2 | 1 | 2 | 115.61 | 78.01 | 47.81 | 47.05 | 32.38 | 30.02 | 12.67 | 8.91 | 25.26 | 42.11 | 10.08 | 17.29 | 18.20 | 17.29 | 22.99 | 14.99 |
| Prussian carp | 1188 | 2 | 1 | 2 | 97.59 | 78.73 | 52.26 | 49.51 | 31.42 | 30.49 | 16.49 | 7.26 | 30.13 | 45.80 | 8.29 | 17.53 | 19.94 | 17.91 | 22.18 | 19.44 |
| Prussian carp | 1189 | 2 | 1 | 2 | 127.86 | 75.51 | 50.17 | 46.28 | 31.39 | 28.98 | 14.90 | 7.98 | 29.70 | 46.59 | 13.36 | 17.66 | 19.54 | 15.06 | 21.84 | 14.90 |
| Prussian carp | 1190 | 2 | 1 | 2 | 108.01 | 75.93 | 50.77 | 48.40 | 31.98 | 29.99 | 14.79 | 8.72 | 27.66 | 40.31 | 10.56 | 17.15 | 19.01 | 13.82 | 19.92 | 14.92 |
| Prussian carp | 1161 | 2 | 1 | 3 | 111.81 | 80.80 | 49.37 | 47.39 | 32.11 | 30.33 | 15.94 | 9.54 | 27.94 | 44.90 | 11.47 | 16.39 | 20.96 | 16.24 | 23.17 | 16.80 |
| Prussian carp | 1162 | 2 | 1 | 3 | 130.84 | 76.74 | 49.62 | 47.31 | 32.87 | 31.51 | 16.25 | 8.74 | 27.85 | 41.48 | 13.70 | 15.62 | 17.31 | 15.72 | 20.67 | 15.79 |
| Prussian carp | 1163 | 2 | 1 | 3 | 123 | 79.66 | 50.99 | 50.31 | 33.50 | 31.68 | 17.31 | 8.70 | 30.02 | 40.71 | 13.59 | 15.52 | 17.05 | 16.10 | 20.32 | 14.12 |
| Prussian carp | 1164 | 2 | 1 | 3 | 123.79 | 79.93 | 44.70 | 48.58 | 32.94 | 31.11 | 16.23 | 9.46 | 27.19 | 47.80 | 10.76 | 16.74 | 19.27 | 15.92 | 19.87 | 16.94 |
| Prussian carp | 1165 | 2 | 1 | 3 | 113.93 | 77.58 | 49.33 | 47.70 | 31.74 | 30.21 | 15.62 | 8.63 | 29.19 | 45.50 | 13.20 | 16.73 | 18.28 | 13.82 | 21.69 | 15.91 |
| Prussian carp | 1166 | 2 | 1 | 3 | 120.26 | 78.84 | 49.83 | 47.37 | 33.42 | 30.56 | 15.41 | 8.11 | 25.94 | 40.39 | 13.91 | 15.63 | 19.59 | 16.35 | 20.73 | 15.29 |
| Prussian carp | 1167 | 2 | 1 | 3 | 131.14 | 77.16 | 55.77 | 50.61 | 33.95 | 32.08 | 16.29 | 7.60 | 26.90 | 42.27 | 11.59 | 16.72 | 18.64 | 16.36 | 21.28 | 15.84 |
| Prussian carp | 1168 | 2 | 1 | 3 | 127.21 | 79.75 | 50.62 | 48.07 | 32.49 | 30.22 | 14.63 | 8.68 | 25.93 | 41.13 | 10.10 | 16.73 | 18.27 | 16.26 | 20.81 | 14.31 |
| Prussian carp | 1169 | 2 | 1 | 3 | 107.76 | 81.04 | 51.51 | 49.46 | 32.71 | 31.29 | 16.35 | 8.72 | 26.58 | 47.34 | 11.28 | 17.63 | 19.55 | 21.46 | 21.26 | 15.07 |
| Prussian carp | 1170 | 2 | 1 | 3 | 109.18 | 81.26 | 52.22 | 49.31 | 33.57 | 32.06 | 17.43 | 10.07 | 29.25 | 46.29 | 11.75 | 16.48 | 18.60 | 18.35 | 22.73 | 16.62 |
| Prussian carp | 1171 | 2 | 1 | 3 | 129.6 | 83.36 | 53.23 | 49.41 | 32.84 | 32.28 | 16.08 | 9.26 | 27.64 | 43.91 | 9.52 | 16.94 | 17.04 | 17.62 | 21.37 | 14.39 |
| Prussian carp | 1172 | 2 | 1 | 3 | 137.86 | 76.90 | 49.07 | 47.82 | 29.71 | 28.32 | 13.38 | 7.97 | 26.64 | 42.33 | 8.73 | 16.09 | 19.50 | 17.37 | 23.11 | 15.82 |
| Prussian carp | 1173 | 2 | 1 | 3 | 105.34 | 76.85 | 51.64 | 48.17 | 33.86 | 30.82 | 16.39 | 8.91 | 26.47 | 43.80 | 12.69 | 16.56 | 17.48 | 22.47 | 20.63 | 17.44 |
| Prussian carp | 1174 | 2 | 1 | 3 | 126.99 | 78.83 | 49.19 | 47.15 | 33.43 | 31.20 | 16.40 | 9.60 | 27.37 | 47.83 | 12.89 | 16.64 | 18.33 | 13.81 | 22.53 | 17.01 |
| Prussian carp | 1175 | 2 | 1 | 3 | 122.82 | 80.35 | 50.13 | 47.64 | 32.60 | 30.59 | 16.19 | 9.42 | 25.87 | 48.56 | 10.87 | 16.89 | 21.95 | 17.55 | 19.62 | 15.19 |
| Prussian carp | 1176 | 2 | 1 | 3 | 118.58 | 80.00 | 51.94 | 49.87 | 32.11 | 30.41 | 15.17 | 10.52 | 28.07 | 47.09 | 14.06 | 15.67 | 19.73 | 19.89 | 22.98 | 16.97 |
| Prussian carp | 1177 | 2 | 1 | 3 | 137.42 | 83.23 | 52.61 | 50.72 | 35.08 | 31.92 | 15.71 | 9.02 | 27.46 | 47.45 | 13.41 | 17.59 | 19.92 | 17.02 | 16.45 | 14.56 |
| Prussian carp | 1178 | 2 | 1 | 3 | 111.11 | 81.46 | 50.92 | 48.27 | 34.03 | 31.46 | 16.06 | 9.41 | 28.73 | 43.79 | 9.25 | 16.94 | 17.15 | 17.39 | 23.55 | 16.25 |
| Prussian carp | 1179 | 2 | 1 | 3 | 121.89 | 78.39 | 48.17 | 46.62 | 32.02 | 29.37 | 15.23 | 10.40 | 28.25 | 42.00 | 11.85 | 15.81 | 17.16 | 13.57 | 18.85 | 15.60 |
| Prussian carp | 1180 | 2 | 1 | 3 | 124.5 | 77.67 | 53.09 | 48.85 | 32.73 | 30.88 | 17.54 | 8.26 | 27.58 | 44.06 | 12.00 | 16.58 | 21.97 | 16.23 | 22.65 | 17.75 |
| Prussian carp | 1181 | 2 | 1 | 3 | 113.95 | 78.85 | 51.17 | 47.05 | 32.79 | 30.89 | 16.05 | 9.62 | 28.32 | 45.44 | 10.03 | 17.46 | 19.34 | 21.42 | 18.81 | 18.89 |
| Prussian carp | 1182 | 2 | 1 | 3 | 112.64 | 78.78 | 51.12 | 50.15 | 34.71 | 32.38 | 16.41 | 9.24 | 27.97 | 40.04 | 13.78 | 16.37 | 14.57 | 16.57 | 16.46 | 17.68 |
| Prussian carp | 1183 | 2 | 1 | 3 | 106.33 | 82.49 | 50.05 | 48.01 | 30.63 | 30.57 | 15.74 | 8.50 | 27.23 | 43.76 | 6.19 | 18.04 | 19.65 | 15.43 | 22.44 | 16.41 |
| Prussian carp | 1184 | 2 | 1 | 3 | 118.29 | 76.91 | 53.30 | 48.57 | 31.79 | 29.93 | 15.63 | 10.16 | 28.27 | 45.84 | 13.68 | 17.01 | 17.51 | 18.37 | 22.08 | 15.13 |
| Prussian carp | 1185 | 2 | 1 | 3 | 141.95 | 82.31 | 51.20 | 47.61 | 30.07 | 28.27 | 13.34 | 8.71 | 26.62 | 44.91 | 12.51 | 17.22 | 20.60 | 15.17 | 19.71 | 16.37 |
| Prussian carp | 1186 | 2 | 1 | 3 | 119.07 | 80.73 | 49.31 | 46.58 | 29.12 | 29.01 | 15.25 | 9.53 | 27.17 | 41.55 | 7.90 | 16.98 | 14.75 | 16.35 | 21.00 | 14.42 |
| Prussian carp | 1187 | 2 | 1 | 3 | 114.93 | 76.98 | 49.09 | 47.08 | 30.74 | 28.67 | 14.07 | 10.04 | 25.04 | 44.55 | 10.90 | 15.99 | 21.69 | 17.25 | 21.14 | 15.69 |
| Prussian carp | 1188 | 2 | 1 | 3 | 98.86 | 77.86 | 50.56 | 49.67 | 32.23 | 30.38 | 15.87 | 8.73 | 32.69 | 47.51 | 10.19 | 18.35 | 22.10 | 19.67 | 25.25 | 15.97 |
| Prussian carp | 1189 | 2 | 1 | 3 | 127.54 | 77.04 | 51.33 | 47.59 | 31.59 | 29.40 | 16.72 | 9.59 | 30.41 | 49.08 | 10.43 | 18.54 | 21.21 | 16.35 | 24.42 | 15.24 |
| Prussian carp | 1190 | 2 | 1 | 3 | 108.46 | 78.35 | 52.13 | 49.41 | 33.72 | 31.47 | 16.18 | 8.25 | 24.90 | 45.76 | 13.69 | 17.31 | 19.93 | 17.00 | 22.25 | 18.56 |
| Prussian carp | 1161 | 2 | 2 | 1 | 111.24 | 79.22 | 47.42 | 47.95 | 29.00 | 31.66 | 17.49 | 8.45 | 27.91 | 42.63 | 18.01 | 17.02 | 22.20 | 19.88 | 22.59 | 20.52 |
| Prussian carp | 1162 | 2 | 2 | 1 | 107.77 | 81.26 | 49.77 | 51.00 | 29.82 | 33.00 | 18.33 | 8.09 | 26.74 | 40.74 | 15.86 | 15.70 | 18.60 | 21.29 | 21.13 | 18.18 |
| Prussian carp | 1163 | 2 | 2 | 1 | 108.2 | 78.95 | 49.30 | 49.80 | 29.67 | 32.49 | 18.93 | 7.27 | 27.52 | 41.17 | 17.17 | 15.92 | 19.93 | 21.48 | 19.54 | 17.49 |
| Prussian carp | 1164 | 2 | 2 | 1 | 72.19 | 77.22 | 53.17 | 47.74 | 29.21 | 32.27 | 16.89 | 6.77 | 26.19 | 45.08 | 19.60 | 16.35 | 19.97 | 20.28 | 22.70 | 19.10 |
| Prussian carp | 1165 | 2 | 2 | 1 | 96.32 | 79.19 | 49.03 | 48.61 | 28.40 | 33.37 | 17.78 | 7.44 | 28.07 | 45.42 | 20.20 | 18.18 | 19.52 | 22.75 | 21.56 | 19.73 |
| Prussian carp | 1166 | 2 | 2 | 1 | 94.14 | 83.08 | 50.72 | 49.61 | 32.03 | 34.08 | 17.39 | 7.18 | 26.61 | 40.23 | 17.47 | 16.06 | 21.94 | 20.72 | 20.09 | 20.31 |
| Prussian carp | 1167 | 2 | 2 | 1 | 114.11 | 80.98 | 56.32 | 47.53 | 29.86 | 33.00 | 18.29 | 7.45 | 29.12 | 42.27 | 19.28 | 17.21 | 21.41 | 23.97 | 20.96 | 19.83 |
| Prussian carp | 1168 | 2 | 2 | 1 | 102.45 | 83.73 | 48.89 | 50.61 | 30.52 | 31.98 | 16.71 | 8.19 | 27.59 | 41.50 | 16.54 | 17.34 | 20.54 | 22.12 | 21.03 | 19.47 |
| Prussian carp | 1169 | 2 | 2 | 1 | 60.68 | 86.87 | 50.99 | 53.89 | 29.55 | 31.88 | 18.63 | 7.15 | 27.17 | 47.88 | 17.75 | 18.12 | 22.25 | 28.35 | 22.31 | 21.91 |
| Prussian carp | 1170 | 2 | 2 | 1 | 99.81 | 80.00 | 53.61 | 48.23 | 30.13 | 33.52 | 20.20 | 7.91 | 27.29 | 46.54 | 17.45 | 17.81 | 17.89 | 21.67 | 24.16 | 22.32 |
| Prussian carp | 1171 | 2 | 2 | 1 | 61.35 | 82.50 | 51.91 | 50.51 | 31.37 | 33.57 | 16.34 | 8.24 | 27.99 | 44.89 | 13.68 | 17.07 | 17.95 | 21.33 | 20.55 | 18.24 |
| Prussian carp | 1172 | 2 | 2 | 1 | 60.87 | 82.48 | 45.63 | 51.68 | 30.26 | 31.43 | 17.81 | 7.59 | 25.75 | 44.83 | 19.84 | 17.13 | 23.04 | 25.30 | 23.74 | 20.22 |
| Prussian carp | 1173 | 2 | 2 | 1 | 63.6 | 75.05 | 51.03 | 42.11 | 26.42 | 29.76 | 15.94 | 7.26 | 26.15 | 42.29 | 19.47 | 16.47 | 19.35 | 22.97 | 21.22 | 18.74 |
| Prussian carp | 1174 | 2 | 2 | 1 | 52.06 | 80.90 | 46.70 | 47.16 | 29.56 | 31.95 | 16.95 | 7.93 | 27.11 | 48.22 | 14.21 | 18.94 | 19.35 | 18.75 | 23.32 | 21.07 |
| Prussian carp | 1175 | 2 | 2 | 1 | 48.87 | 81.80 | 50.99 | 48.17 | 27.31 | 30.79 | 17.23 | 6.99 | 26.92 | 46.38 | 15.95 | 17.54 | 19.73 | 24.86 | 22.81 | 23.89 |
| Prussian carp | 1176 | 2 | 2 | 1 | 58.03 | 79.94 | 54.04 | 50.05 | 29.24 | 31.68 | 16.54 | 6.89 | 29.66 | 46.67 | 16.44 | 14.91 | 24.24 | 20.24 | 19.32 | 24.51 |
| Prussian carp | 1177 | 2 | 2 | 1 | 55.61 | 76.59 | 50.83 | 45.88 | 28.16 | 30.09 | 17.12 | 6.76 | 24.80 | 46.01 | 20.52 | 17.89 | 20.57 | 24.75 | 20.38 | 21.55 |
| Prussian carp | 1178 | 2 | 2 | 1 | 51.34 | 83.78 | 52.93 | 52.28 | 29.04 | 32.01 | 17.59 | 7.71 | 28.20 | 45.21 | 16.32 | 17.60 | 20.55 | 22.42 | 23.46 | 21.67 |
| Prussian carp | 1179 | 2 | 2 | 1 | 49.58 | 79.66 | 47.96 | 49.35 | 29.20 | 30.36 | 16.68 | 7.89 | 26.05 | 42.00 | 21.57 | 16.66 | 18.81 | 20.38 | 19.60 | 19.09 |
| Prussian carp | 1180 | 2 | 2 | 1 | 50.85 | 75.45 | 54.06 | 49.84 | 27.87 | 33.13 | 16.85 | 6.82 | 30.02 | 45.97 | 20.14 | 16.89 | 19.41 | 25.10 | 25.27 | 21.92 |
| Prussian carp | 1181 | 2 | 2 | 1 | 49.99 | 80.14 | 47.82 | 52.93 | 30.89 | 32.43 | 18.26 | 7.71 | 28.01 | 45.80 | 22.27 | 21.18 | 20.44 | 23.67 | 20.23 | 21.90 |
| Prussian carp | 1182 | 2 | 2 | 1 | 53.82 | 82.31 | 45.43 | 50.28 | 29.94 | 31.29 | 17.35 | 6.81 | 25.76 | 41.38 | 20.86 | 16.12 | 17.93 | 20.04 | 19.08 | 19.08 |
| Prussian carp | 1183 | 2 | 2 | 1 | 52.79 | 82.64 | 51.02 | 48.97 | 27.83 | 29.83 | 16.80 | 6.60 | 26.71 | 44.75 | 19.24 | 17.90 | 18.84 | 21.90 | 22.11 | 18.93 |
| Prussian carp | 1184 | 2 | 2 | 1 | 60.17 | 78.45 | 50.26 | 45.42 | 28.61 | 32.54 | 15.77 | 6.72 | 26.47 | 40.66 | 17.74 | 16.39 | 18.40 | 23.02 | 21.93 | 20.60 |
| Prussian carp | 1185 | 2 | 2 | 1 | 46.25 | 83.75 | 53.85 | 49.88 | 28.08 | 31.03 | 17.12 | 7.68 | 27.28 | 44.60 | 17.36 | 15.70 | 20.56 | 24.08 | 20.43 | 21.54 |
| Prussian carp | 1186 | 2 | 2 | 1 | 62 | 79.88 | 52.30 | 45.77 | 28.19 | 30.74 | 15.86 | 7.48 | 26.83 | 42.75 | 19.74 | 16.84 | 19.99 | 18.19 | 21.10 | 19.24 |
| Prussian carp | 1187 | 2 | 2 | 1 | 49.75 | 77.07 | 49.59 | 42.36 | 25.30 | 29.75 | 15.97 | 6.69 | 25.34 | 41.14 | 19.60 | 17.36 | 21.82 | 15.50 | 21.44 | 16.41 |
| Prussian carp | 1188 | 2 | 2 | 1 | 46.78 | 82.82 | 50.66 | 51.04 | 31.22 | 33.94 | 18.09 | 7.26 | 28.59 | 48.41 | 14.35 | 17.58 | 19.72 | 26.25 | 25.12 | 21.95 |
| Prussian carp | 1189 | 2 | 2 | 1 | 56.33 | 80.77 | 49.08 | 47.75 | 29.92 | 32.62 | 18.81 | 7.24 | 29.79 | 48.32 | 18.84 | 18.46 | 22.63 | 22.25 | 23.28 | 20.74 |
| Prussian carp | 1190 | 2 | 2 | 1 | 46.77 | 77.30 | 48.43 | 47.16 | 25.83 | 28.85 | 16.53 | 7.65 | 24.28 | 43.18 | 20.84 | 16.25 | 20.83 | 21.28 | 18.39 | 17.53 |
| Prussian carp | 1161 | 2 | 2 | 2 | 110.46 | 80.55 | 51.24 | 53.21 | 30.55 | 31.33 | 18.89 | 8.76 | 25.96 | 42.84 | 18.13 | 16.54 | 21.83 | 23.69 | 23.33 | 19.57 |
| Prussian carp | 1162 | 2 | 2 | 2 | 108.15 | 81.94 | 49.28 | 50.83 | 30.62 | 33.67 | 18.24 | 8.43 | 26.88 | 40.13 | 18.57 | 16.44 | 18.91 | 22.48 | 22.55 | 20.08 |
| Prussian carp | 1163 | 2 | 2 | 2 | 109.83 | 79.53 | 46.08 | 49.78 | 28.25 | 32.79 | 18.83 | 8.45 | 27.71 | 40.96 | 18.83 | 15.94 | 19.51 | 24.59 | 22.03 | 20.10 |
| Prussian carp | 1164 | 2 | 2 | 2 | 74.32 | 77.13 | 50.03 | 45.70 | 28.80 | 32.30 | 17.23 | 7.31 | 27.48 | 45.01 | 17.78 | 16.91 | 20.59 | 22.33 | 20.53 | 20.37 |
| Prussian carp | 1165 | 2 | 2 | 2 | 98.69 | 79.07 | 46.04 | 47.54 | 30.24 | 32.14 | 17.25 | 7.04 | 25.95 | 43.75 | 16.79 | 19.68 | 19.38 | 22.96 | 21.10 | 19.91 |
| Prussian carp | 1166 | 2 | 2 | 2 | 95.39 | 82.01 | 50.92 | 51.88 | 31.11 | 32.45 | 17.95 | 7.35 | 26.50 | 39.56 | 16.10 | 16.22 | 21.52 | 22.35 | 22.29 | 19.56 |
| Prussian carp | 1167 | 2 | 2 | 2 | 114.13 | 82.30 | 53.50 | 53.01 | 30.72 | 33.13 | 17.59 | 8.88 | 28.76 | 42.19 | 17.67 | 18.24 | 20.71 | 26.02 | 22.31 | 20.87 |
| Prussian carp | 1168 | 2 | 2 | 2 | 102.94 | 82.25 | 52.79 | 46.68 | 27.91 | 30.31 | 16.85 | 7.30 | 27.27 | 40.95 | 16.40 | 17.42 | 21.65 | 23.72 | 21.74 | 20.08 |
| Prussian carp | 1169 | 2 | 2 | 2 | 60.72 | 79.78 | 49.44 | 49.43 | 28.46 | 32.17 | 17.40 | 6.61 | 26.50 | 47.48 | 18.59 | 17.97 | 22.22 | 26.54 | 23.31 | 22.05 |
| Prussian carp | 1170 | 2 | 2 | 2 | 101.44 | 84.24 | 47.33 | 50.74 | 27.54 | 33.58 | 17.92 | 7.66 | 27.65 | 44.65 | 17.88 | 18.29 | 22.99 | 23.72 | 21.69 | 21.36 |
| Prussian carp | 1171 | 2 | 2 | 2 | 61.09 | 77.74 | 52.46 | 48.93 | 28.68 | 30.63 | 17.78 | 8.96 | 27.73 | 45.20 | 16.93 | 17.19 | 17.89 | 21.77 | 22.26 | 21.25 |
| Prussian carp | 1172 | 2 | 2 | 2 | 61.88 | 77.79 | 48.43 | 45.42 | 26.05 | 29.93 | 18.11 | 6.11 | 25.04 | 42.52 | 16.82 | 15.71 | 22.80 | 26.75 | 24.93 | 21.54 |
| Prussian carp | 1173 | 2 | 2 | 2 | 61.78 | 81.30 | 49.66 | 50.01 | 29.67 | 31.28 | 15.46 | 8.09 | 25.61 | 43.93 | 14.51 | 17.09 | 18.55 | 22.72 | 20.27 | 19.67 |
| Prussian carp | 1174 | 2 | 2 | 2 | 51.88 | 80.21 | 44.11 | 46.60 | 29.15 | 31.78 | 17.29 | 5.80 | 26.34 | 48.29 | 14.84 | 17.11 | 19.27 | 24.12 | 22.23 | 20.44 |
| Prussian carp | 1175 | 2 | 2 | 2 | 49.74 | 80.52 | 49.24 | 49.29 | 26.41 | 31.80 | 15.97 | 6.59 | 26.49 | 46.31 | 16.82 | 18.91 | 24.15 | 21.73 | 23.78 | 21.68 |
| Prussian carp | 1176 | 2 | 2 | 2 | 59.16 | 80.96 | 55.76 | 53.92 | 32.04 | 33.23 | 16.73 | 8.01 | 28.62 | 48.02 | 14.49 | 14.88 | 21.72 | 19.27 | 23.78 | 24.53 |
| Prussian carp | 1177 | 2 | 2 | 2 | 56.08 | 82.62 | 51.52 | 48.90 | 32.20 | 31.87 | 17.08 | 8.46 | 27.31 | 44.12 | 16.88 | 18.35 | 20.55 | 21.62 | 21.16 | 18.76 |
| Prussian carp | 1178 | 2 | 2 | 2 | 52.04 | 75.89 | 50.68 | 48.98 | 29.37 | 31.77 | 17.04 | 7.77 | 26.92 | 42.56 | 17.57 | 18.11 | 19.54 | 22.97 | 23.04 | 20.23 |
| Prussian carp | 1179 | 2 | 2 | 2 | 50.2 | 72.71 | 50.74 | 47.84 | 28.96 | 31.20 | 15.68 | 7.96 | 26.73 | 42.24 | 20.76 | 16.46 | 17.33 | 20.19 | 20.19 | 18.20 |
| Prussian carp | 1180 | 2 | 2 | 2 | 49.46 | 80.14 | 53.72 | 47.42 | 27.12 | 31.91 | 17.50 | 7.74 | 28.47 | 47.54 | 17.41 | 17.96 | 23.83 | 22.51 | 25.27 | 20.83 |
| Prussian carp | 1181 | 2 | 2 | 2 | 50.48 | 81.02 | 48.54 | 50.45 | 28.26 | 30.81 | 17.96 | 6.22 | 28.57 | 44.53 | 17.48 | 17.36 | 21.69 | 23.68 | 20.33 | 25.25 |
| Prussian carp | 1182 | 2 | 2 | 2 | 53.59 | 83.78 | 51.00 | 48.56 | 29.46 | 32.81 | 17.99 | 8.16 | 25.79 | 40.17 | 17.48 | 16.75 | 19.25 | 22.29 | 21.27 | 18.67 |
| Prussian carp | 1183 | 2 | 2 | 2 | 52.22 | 85.15 | 48.13 | 46.94 | 28.40 | 30.92 | 17.30 | 6.50 | 25.74 | 43.77 | 16.66 | 17.98 | 19.53 | 23.65 | 22.56 | 20.13 |
| Prussian carp | 1184 | 2 | 2 | 2 | 59.59 | 83.55 | 51.24 | 49.90 | 29.77 | 31.29 | 17.02 | 5.93 | 25.12 | 42.01 | 19.09 | 17.48 | 24.04 | 18.42 | 20.80 | 20.46 |
| Prussian carp | 1185 | 2 | 2 | 2 | 45.72 | 85.28 | 45.02 | 44.43 | 27.70 | 30.86 | 17.18 | 6.88 | 27.20 | 45.38 | 16.74 | 18.26 | 22.10 | 24.90 | 22.55 | 24.08 |
| Prussian carp | 1186 | 2 | 2 | 2 | 62.35 | 78.09 | 55.15 | 50.87 | 30.68 | 31.75 | 14.95 | 6.07 | 25.23 | 42.40 | 17.22 | 16.54 | 19.20 | 23.08 | 20.29 | 20.09 |
| Prussian carp | 1187 | 2 | 2 | 2 | 49.49 | 81.17 | 44.27 | 47.78 | 28.97 | 30.81 | 16.58 | 7.38 | 26.87 | 42.66 | 16.40 | 21.53 | 21.29 | 23.02 | 22.78 | 21.72 |
| Prussian carp | 1188 | 2 | 2 | 2 | 48.11 | 81.58 | 48.96 | 48.30 | 28.16 | 32.26 | 18.04 | 7.93 | 28.76 | 47.22 | 16.56 | 17.84 | 21.38 | 28.45 | 23.78 | 20.61 |
| Prussian carp | 1189 | 2 | 2 | 2 | 56.5 | 76.98 | 52.09 | 47.64 | 30.17 | 31.29 | 16.06 | 7.62 | 29.47 | 46.08 | 18.97 | 17.88 | 22.32 | 25.43 | 22.61 | 19.83 |
| Prussian carp | 1190 | 2 | 2 | 2 | 45.76 | 79.44 | 48.08 | 48.48 | 27.10 | 32.00 | 15.31 | 7.45 | 27.47 | 42.86 | 16.87 | 17.43 | 22.12 | 23.16 | 21.00 | 19.91 |
| Prussian carp | 1161 | 2 | 2 | 3 | 110.17 | 81.07 | 49.94 | 46.97 | 28.14 | 29.99 | 19.12 | 7.74 | 25.93 | 43.11 | 16.60 | 16.25 | 21.96 | 26.15 | 22.79 | 20.26 |
| Prussian carp | 1162 | 2 | 2 | 3 | 106.36 | 81.44 | 51.59 | 50.44 | 29.28 | 32.93 | 17.27 | 8.14 | 27.61 | 41.38 | 16.47 | 16.55 | 20.96 | 21.96 | 22.55 | 19.43 |
| Prussian carp | 1163 | 2 | 2 | 3 | 109.6 | 80.69 | 48.78 | 44.36 | 28.33 | 32.04 | 17.26 | 7.65 | 27.52 | 39.26 | 19.25 | 15.86 | 20.13 | 23.27 | 19.46 | 18.89 |
| Prussian carp | 1164 | 2 | 2 | 3 | 71.68 | 75.94 | 49.21 | 46.81 | 30.03 | 32.16 | 18.35 | 7.26 | 30.04 | 46.31 | 18.80 | 17.22 | 21.18 | 25.71 | 22.24 | 20.42 |
| Prussian carp | 1165 | 2 | 2 | 3 | 98.16 | 79.16 | 47.09 | 47.61 | 30.63 | 32.76 | 17.74 | 7.00 | 26.43 | 44.85 | 18.60 | 16.89 | 20.49 | 22.56 | 22.57 | 20.34 |
| Prussian carp | 1166 | 2 | 2 | 3 | 95.07 | 83.09 | 50.10 | 48.98 | 30.32 | 30.93 | 19.29 | 7.83 | 26.16 | 40.19 | 17.16 | 15.57 | 21.72 | 21.89 | 21.51 | 20.33 |
| Prussian carp | 1167 | 2 | 2 | 3 | 116.67 | 78.82 | 54.97 | 49.73 | 30.65 | 31.40 | 17.57 | 8.31 | 27.95 | 41.10 | 16.66 | 18.52 | 20.68 | 24.36 | 20.95 | 19.86 |
| Prussian carp | 1168 | 2 | 2 | 3 | 104.44 | 79.85 | 51.56 | 46.37 | 28.97 | 29.32 | 16.98 | 7.53 | 26.59 | 40.40 | 17.57 | 16.84 | 21.07 | 22.01 | 21.01 | 20.51 |
| Prussian carp | 1169 | 2 | 2 | 3 | 63.44 | 79.45 | 49.38 | 51.02 | 27.82 | 30.85 | 17.21 | 7.57 | 29.28 | 46.09 | 16.53 | 13.84 | 18.82 | 27.06 | 21.75 | 21.38 |
| Prussian carp | 1170 | 2 | 2 | 3 | 101.99 | 81.71 | 49.78 | 48.67 | 29.70 | 33.49 | 19.31 | 7.58 | 28.62 | 45.22 | 18.33 | 17.27 | 21.64 | 25.82 | 22.52 | 21.61 |
| Prussian carp | 1171 | 2 | 2 | 3 | 62.14 | 79.23 | 54.32 | 48.15 | 30.93 | 31.16 | 18.56 | 6.80 | 28.65 | 42.92 | 15.51 | 17.20 | 19.93 | 22.87 | 21.53 | 22.61 |
| Prussian carp | 1172 | 2 | 2 | 3 | 61.54 | 81.20 | 49.47 | 48.05 | 24.66 | 27.79 | 16.37 | 6.27 | 26.99 | 42.30 | 15.01 | 16.29 | 21.37 | 24.83 | 20.59 | 22.08 |
| Prussian carp | 1173 | 2 | 2 | 3 | 62.76 | 74.78 | 48.90 | 44.55 | 27.20 | 30.50 | 16.11 | 7.71 | 27.14 | 42.78 | 19.58 | 16.66 | 18.33 | 27.88 | 17.65 | 19.31 |
| Prussian carp | 1174 | 2 | 2 | 3 | 52.3 | 81.80 | 42.93 | 47.91 | 27.86 | 31.32 | 18.28 | 6.92 | 25.82 | 47.61 | 16.55 | 16.66 | 18.26 | 22.85 | 22.03 | 20.54 |
| Prussian carp | 1175 | 2 | 2 | 3 | 50.16 | 80.36 | 52.98 | 48.89 | 28.57 | 30.36 | 16.38 | 7.87 | 27.16 | 45.73 | 16.50 | 13.71 | 21.11 | 23.78 | 22.31 | 19.69 |
| Prussian carp | 1176 | 2 | 2 | 3 | 58.74 | 82.21 | 51.92 | 58.22 | 30.65 | 34.81 | 16.41 | 5.93 | 28.32 | 47.68 | 14.28 | 14.52 | 22.89 | 23.39 | 24.65 | 19.80 |
| Prussian carp | 1177 | 2 | 2 | 3 | 54.35 | 83.39 | 48.82 | 53.73 | 28.08 | 30.20 | 20.06 | 8.42 | 29.64 | 45.37 | 17.93 | 18.42 | 20.87 | 25.90 | 21.77 | 20.91 |
| Prussian carp | 1178 | 2 | 2 | 3 | 51.31 | 77.42 | 50.36 | 53.64 | 28.82 | 30.10 | 18.36 | 8.58 | 27.45 | 44.39 | 16.37 | 18.06 | 20.27 | 22.71 | 20.92 | 20.57 |
| Prussian carp | 1179 | 2 | 2 | 3 | 49.72 | 82.04 | 53.19 | 45.89 | 25.82 | 29.28 | 16.06 | 6.02 | 25.36 | 42.13 | 17.81 | 15.94 | 20.31 | 22.66 | 21.09 | 20.51 |
| Prussian carp | 1180 | 2 | 2 | 3 | 50.11 | 78.50 | 49.59 | 45.74 | 30.01 | 32.00 | 18.01 | 8.51 | 25.79 | 45.55 | 17.80 | 16.31 | 20.22 | 24.06 | 23.69 | 21.90 |
| Prussian carp | 1181 | 2 | 2 | 3 | 49.68 | 80.86 | 45.34 | 48.74 | 29.79 | 32.66 | 18.65 | 7.42 | 28.39 | 44.60 | 17.65 | 17.60 | 20.14 | 24.37 | 20.08 | 20.96 |
| Prussian carp | 1182 | 2 | 2 | 3 | 53.29 | 81.30 | 51.48 | 52.63 | 29.92 | 29.92 | 16.53 | 7.52 | 25.37 | 40.38 | 17.10 | 16.45 | 18.98 | 21.41 | 18.91 | 20.07 |
| Prussian carp | 1183 | 2 | 2 | 3 | 51.28 | 79.92 | 49.08 | 49.21 | 25.93 | 29.60 | 15.42 | 7.05 | 29.34 | 45.13 | 15.13 | 18.27 | 20.55 | 25.08 | 24.25 | 20.58 |
| Prussian carp | 1184 | 2 | 2 | 3 | 60.43 | 81.33 | 48.09 | 48.65 | 29.61 | 31.88 | 16.73 | 7.93 | 27.00 | 41.91 | 18.46 | 16.65 | 19.68 | 25.13 | 20.00 | 20.42 |
| Prussian carp | 1185 | 2 | 2 | 3 | 46.13 | 83.20 | 46.01 | 52.22 | 28.99 | 30.36 | 15.92 | 7.99 | 27.75 | 44.82 | 16.18 | 18.18 | 21.72 | 26.54 | 21.64 | 22.30 |
| Prussian carp | 1186 | 2 | 2 | 3 | 63.74 | 77.00 | 51.34 | 46.57 | 28.60 | 29.09 | 16.02 | 7.37 | 27.98 | 41.01 | 16.74 | 17.20 | 19.94 | 23.47 | 21.13 | 21.31 |
| Prussian carp | 1187 | 2 | 2 | 3 | 50.18 | 75.20 | 46.78 | 49.69 | 25.15 | 28.09 | 14.98 | 6.00 | 26.37 | 41.48 | 19.69 | 16.58 | 21.14 | 22.67 | 21.56 | 20.69 |
| Prussian carp | 1188 | 2 | 2 | 3 | 48.35 | 79.94 | 55.56 | 46.45 | 28.95 | 31.47 | 18.63 | 6.52 | 28.11 | 47.28 | 15.19 | 17.58 | 20.11 | 29.80 | 24.20 | 20.69 |
| Prussian carp | 1189 | 2 | 2 | 3 | 56.97 | 78.50 | 54.07 | 50.05 | 30.17 | 31.32 | 17.28 | 7.01 | 28.34 | 46.88 | 18.19 | 16.96 | 17.64 | 25.86 | 24.50 | 20.80 |
| Prussian carp | 1190 | 2 | 2 | 3 | 46.17 | 82.21 | 48.87 | 51.44 | 28.87 | 30.26 | 17.60 | 7.20 | 26.99 | 42.77 | 23.44 | 17.94 | 20.14 | 23.77 | 21.57 | 18.95 |
| Prussian carp | 1161 | 2 | 3 | 1 | 138.46 | 80.340 | 48.699 | 49.641 | 31.069 | 32.306 | 16.670 | 9.295 | 28.293 | 42.596 | 18.064 | 15.842 | 21.459 | 22.618 | 22.162 | 20.67263 |
| Prussian carp | 1162 | 2 | 3 | 1 | 156.59 | 81.577 | 50.331 | 51.659 | 33.400 | 34.120 | 17.565 | 9.525 | 27.018 | 40.842 | 20.277 | 15.879 | 20.697 | 21.812 | 21.996 | 19.81421 |
| Prussian carp | 1163 | 2 | 3 | 1 | 153.71 | 79.669 | 51.891 | 50.263 | 30.811 | 31.820 | 16.742 | 8.913 | 28.363 | 40.042 | 20.045 | 16.085 | 19.500 | 20.328 | 20.309 | 18.51153 |
| Prussian carp | 1164 | 2 | 3 | 1 | 141.51 | 81.219 | 51.627 | 51.335 | 32.660 | 32.150 | 16.817 | 9.696 | 27.496 | 44.673 | 20.932 | 16.698 | 20.413 | 22.420 | 23.217 | 20.84947 |
| Prussian carp | 1165 | 2 | 3 | 1 | 120.9 | 80.359 | 47.904 | 47.746 | 31.418 | 32.890 | 17.131 | 8.497 | 27.512 | 45.066 | 20.713 | 16.996 | 20.310 | 21.440 | 22.140 | 19.75048 |
| Prussian carp | 1166 | 2 | 3 | 1 | 134.6 | 81.286 | 51.682 | 50.695 | 32.317 | 32.146 | 17.067 | 8.185 | 27.628 | 40.998 | 18.222 | 15.773 | 21.423 | 23.412 | 22.327 | 20.78844 |
| Prussian carp | 1167 | 2 | 3 | 1 | 135.81 | 79.856 | 54.305 | 51.818 | 31.777 | 33.143 | 16.330 | 9.918 | 27.312 | 41.623 | 16.818 | 16.779 | 20.112 | 21.520 | 21.694 | 20.34956 |
| Prussian carp | 1168 | 2 | 3 | 1 | 118.48 | 81.795 | 48.869 | 50.306 | 31.311 | 31.503 | 16.143 | 8.870 | 25.584 | 40.338 | 16.484 | 17.082 | 22.256 | 22.839 | 21.538 | 20.00513 |
| Prussian carp | 1169 | 2 | 3 | 1 | 125.3 | 81.568 | 51.270 | 50.819 | 33.572 | 32.832 | 17.956 | 9.438 | 26.817 | 47.233 | 15.414 | 18.124 | 22.068 | 25.546 | 24.655 | 23.85985 |
| Prussian carp | 1170 | 2 | 3 | 1 | 124.08 | 82.711 | 51.554 | 49.952 | 32.714 | 33.458 | 17.895 | 8.180 | 27.758 | 45.022 | 16.663 | 17.299 | 21.539 | 23.376 | 23.434 | 22.33396 |
| Prussian carp | 1171 | 2 | 3 | 1 | 145.51 | 80.414 | 47.285 | 50.264 | 33.620 | 33.081 | 17.625 | 9.120 | 27.429 | 45.459 | 17.790 | 16.652 | 20.786 | 22.328 | 22.759 | 21.44587 |
| Prussian carp | 1172 | 2 | 3 | 1 | 119.23 | 81.183 | 49.595 | 49.726 | 33.915 | 32.471 | 16.405 | 9.111 | 25.577 | 44.186 | 19.358 | 15.934 | 21.208 | 21.965 | 22.680 | 21.71738 |
| Prussian carp | 1173 | 2 | 3 | 1 | 125.98 | 81.107 | 50.573 | 49.722 | 32.932 | 32.187 | 16.465 | 9.083 | 26.022 | 43.356 | 19.862 | 16.246 | 19.717 | 22.528 | 20.806 | 19.91005 |
| Prussian carp | 1174 | 2 | 3 | 1 | 153.48 | 80.947 | 50.417 | 50.070 | 33.129 | 32.152 | 17.308 | 7.338 | 27.468 | 47.848 | 16.740 | 17.122 | 18.622 | 20.525 | 23.308 | 20.82334 |
| Prussian carp | 1175 | 2 | 3 | 1 | 132.34 | 82.520 | 47.592 | 51.006 | 31.606 | 31.741 | 17.698 | 8.139 | 26.457 | 47.007 | 16.604 | 17.748 | 22.820 | 25.549 | 22.618 | 21.06322 |
| Prussian carp | 1176 | 2 | 3 | 1 | 127.72 | 81.358 | 47.253 | 48.234 | 33.065 | 32.266 | 16.882 | 9.262 | 27.785 | 47.575 | 16.674 | 15.524 | 23.548 | 21.429 | 24.613 | 23.9374 |
| Prussian carp | 1177 | 2 | 3 | 1 | 126.38 | 81.108 | 51.669 | 50.083 | 33.713 | 32.449 | 16.856 | 8.699 | 26.811 | 45.917 | 16.119 | 17.646 | 21.370 | 23.341 | 21.015 | 21.24012 |
| Prussian carp | 1178 | 2 | 3 | 1 | 128.97 | 80.642 | 51.901 | 52.454 | 33.057 | 32.092 | 16.701 | 8.141 | 27.367 | 44.368 | 18.497 | 17.013 | 19.833 | 23.303 | 23.062 | 21.38233 |
| Prussian carp | 1179 | 2 | 3 | 1 | 116.72 | 76.606 | 49.269 | 49.512 | 33.542 | 31.469 | 16.026 | 8.020 | 26.729 | 42.713 | 19.832 | 15.823 | 18.590 | 21.144 | 20.114 | 19.24456 |
| Prussian carp | 1180 | 2 | 3 | 1 | 127.26 | 81.539 | 51.814 | 50.563 | 34.246 | 33.204 | 17.719 | 7.825 | 27.806 | 45.874 | 16.655 | 17.094 | 22.534 | 24.874 | 23.895 | 20.92984 |
| Prussian carp | 1181 | 2 | 3 | 1 | 127.96 | 77.944 | 51.381 | 51.049 | 32.679 | 31.677 | 16.940 | 8.478 | 27.266 | 44.642 | 19.605 | 17.278 | 19.440 | 21.185 | 20.701 | 19.79267 |
| Prussian carp | 1182 | 2 | 3 | 1 | 117.06 | 84.601 | 51.258 | 49.532 | 32.885 | 32.169 | 16.968 | 9.243 | 25.111 | 39.078 | 20.850 | 15.211 | 19.491 | 21.791 | 21.701 | 21.14534 |
| Prussian carp | 1183 | 2 | 3 | 1 | 129.34 | 82.840 | 51.766 | 51.431 | 32.616 | 31.358 | 17.030 | 8.110 | 25.650 | 44.503 | 15.522 | 17.400 | 20.485 | 23.563 | 24.341 | 21.66916 |
| Prussian carp | 1184 | 2 | 3 | 1 | 128.94 | 79.799 | 53.301 | 49.966 | 33.908 | 32.177 | 16.563 | 9.087 | 25.896 | 41.806 | 21.040 | 15.789 | 20.330 | 19.690 | 22.383 | 22.87857 |
| Prussian carp | 1185 | 2 | 3 | 1 | 138.2 | 83.076 | 51.242 | 52.283 | 32.106 | 30.689 | 15.781 | 8.196 | 27.775 | 44.982 | 16.253 | 17.502 | 21.459 | 22.806 | 21.912 | 20.44676 |
| Prussian carp | 1186 | 2 | 3 | 1 | 145.46 | 79.815 | 49.425 | 50.428 | 33.957 | 32.121 | 16.325 | 8.882 | 28.504 | 43.553 | 19.342 | 17.293 | 20.343 | 17.086 | 21.641 | 19.31716 |
| Prussian carp | 1187 | 2 | 3 | 1 | 118.61 | 79.943 | 50.005 | 51.438 | 32.350 | 30.518 | 15.952 | 7.133 | 27.101 | 42.525 | 20.258 | 16.742 | 20.229 | 21.878 | 21.522 | 17.46761 |
| Prussian carp | 1188 | 2 | 3 | 1 | 141.34 | 80.786 | 54.602 | 52.067 | 34.126 | 32.326 | 18.126 | 9.114 | 29.040 | 46.828 | 20.294 | 18.195 | 21.752 | 23.069 | 23.001 | 22.85809 |
| Prussian carp | 1189 | 2 | 3 | 1 | 129.76 | 78.461 | 53.011 | 49.858 | 34.460 | 33.014 | 17.546 | 9.516 | 25.805 | 47.440 | 19.507 | 17.602 | 21.255 | 22.913 | 23.940 | 21.40248 |
| Prussian carp | 1190 | 2 | 3 | 1 | 149.53 | 78.176 | 51.764 | 50.744 | 32.295 | 30.775 | 15.674 | 8.156 | 26.106 | 41.394 | 16.887 | 17.066 | 19.994 | 21.897 | 22.088 | 17.25859 |
| Prussian carp | 1161 | 2 | 3 | 2 | 138.71 | 80.971 | 50.600 | 51.067 | 31.060 | 33.139 | 16.804 | 8.470 | 28.828 | 43.502 | 16.752 | 17.197 | 21.325 | 22.663 | 23.011 | 21.12462 |
| Prussian carp | 1162 | 2 | 3 | 2 | 155.71 | 81.219 | 49.370 | 52.231 | 32.102 | 32.903 | 18.205 | 9.386 | 26.229 | 40.869 | 17.341 | 16.153 | 20.478 | 21.368 | 21.506 | 19.61196 |
| Prussian carp | 1163 | 2 | 3 | 2 | 151.96 | 78.401 | 49.692 | 50.761 | 31.076 | 31.679 | 17.653 | 9.201 | 26.947 | 40.046 | 19.521 | 16.028 | 19.392 | 20.326 | 19.873 | 17.5409 |
| Prussian carp | 1164 | 2 | 3 | 2 | 138.09 | 81.651 | 51.567 | 50.172 | 33.102 | 33.815 | 16.668 | 9.485 | 27.882 | 44.782 | 17.544 | 17.608 | 20.673 | 21.666 | 20.393 | 20.34525 |
| Prussian carp | 1165 | 2 | 3 | 2 | 120.68 | 79.332 | 47.391 | 48.214 | 31.253 | 32.563 | 17.693 | 9.239 | 27.542 | 44.695 | 16.842 | 17.550 | 16.716 | 22.138 | 21.502 | 20.16429 |
| Prussian carp | 1166 | 2 | 3 | 2 | 134.01 | 82.010 | 50.503 | 50.746 | 31.980 | 32.512 | 17.326 | 8.190 | 27.246 | 40.463 | 17.896 | 15.844 | 22.053 | 21.793 | 21.841 | 20.57074 |
| Prussian carp | 1167 | 2 | 3 | 2 | 135.55 | 79.432 | 55.066 | 52.633 | 31.548 | 33.592 | 16.678 | 9.569 | 27.984 | 41.689 | 15.556 | 16.598 | 19.719 | 22.041 | 21.169 | 20.22718 |
| Prussian carp | 1168 | 2 | 3 | 2 | 118.31 | 79.778 | 48.821 | 48.304 | 31.386 | 31.056 | 16.006 | 8.670 | 24.924 | 40.425 | 16.608 | 17.188 | 22.383 | 23.112 | 21.783 | 19.38207 |
| Prussian carp | 1169 | 2 | 3 | 2 | 122.85 | 81.649 | 54.702 | 50.718 | 33.426 | 32.684 | 17.430 | 9.270 | 27.935 | 47.140 | 18.869 | 18.110 | 22.609 | 22.193 | 24.559 | 21.63377 |
| Prussian carp | 1170 | 2 | 3 | 2 | 125.61 | 82.756 | 50.377 | 49.480 | 31.770 | 32.978 | 17.237 | 9.548 | 27.425 | 44.516 | 15.724 | 17.530 | 21.755 | 23.351 | 22.644 | 22.05039 |
| Prussian carp | 1171 | 2 | 3 | 2 | 145.54 | 82.101 | 50.314 | 50.269 | 33.877 | 32.685 | 17.378 | 9.028 | 27.054 | 43.750 | 16.331 | 17.145 | 20.593 | 21.815 | 20.792 | 18.88148 |
| Prussian carp | 1172 | 2 | 3 | 2 | 118.23 | 81.309 | 47.889 | 50.443 | 32.155 | 30.855 | 15.776 | 7.985 | 25.942 | 42.732 | 16.459 | 16.579 | 21.983 | 24.282 | 22.357 | 21.84966 |
| Prussian carp | 1173 | 2 | 3 | 2 | 126.94 | 76.233 | 48.010 | 45.615 | 32.842 | 31.541 | 16.324 | 9.399 | 24.015 | 41.883 | 20.683 | 15.177 | 18.911 | 20.471 | 20.200 | 18.16977 |
| Prussian carp | 1174 | 2 | 3 | 2 | 154.8 | 81.251 | 48.134 | 46.441 | 32.590 | 33.395 | 16.303 | 8.962 | 27.743 | 47.834 | 16.392 | 17.606 | 19.235 | 23.332 | 22.506 | 23.27894 |
| Prussian carp | 1175 | 2 | 3 | 2 | 132.96 | 81.811 | 48.043 | 50.322 | 32.282 | 32.315 | 17.007 | 7.571 | 28.713 | 46.087 | 17.037 | 17.823 | 23.670 | 23.042 | 23.516 | 21.2479 |
| Prussian carp | 1176 | 2 | 3 | 2 | 127.39 | 82.524 | 53.611 | 52.070 | 33.249 | 33.316 | 17.309 | 9.443 | 28.500 | 48.133 | 15.274 | 15.379 | 24.599 | 24.842 | 24.000 | 25.99435 |
| Prussian carp | 1177 | 2 | 3 | 2 | 125.08 | 83.447 | 48.607 | 50.729 | 31.380 | 31.753 | 17.291 | 9.311 | 27.278 | 45.171 | 17.772 | 17.196 | 20.444 | 23.615 | 20.668 | 22.92018 |
| Prussian carp | 1178 | 2 | 3 | 2 | 128.86 | 81.062 | 50.946 | 51.519 | 32.140 | 31.400 | 17.118 | 9.136 | 27.527 | 43.379 | 20.486 | 16.743 | 21.612 | 22.120 | 20.569 | 19.86061 |
| Prussian carp | 1179 | 2 | 3 | 2 | 116.29 | 78.528 | 48.743 | 49.102 | 33.098 | 31.059 | 16.279 | 9.097 | 28.090 | 42.760 | 22.109 | 15.949 | 21.051 | 21.043 | 20.835 | 20.04849 |
| Prussian carp | 1180 | 2 | 3 | 2 | 132.53 | 82.577 | 50.454 | 50.721 | 33.793 | 32.764 | 17.271 | 7.574 | 28.205 | 45.183 | 18.905 | 17.163 | 22.391 | 24.281 | 23.495 | 22.49126 |
| Prussian carp | 1181 | 2 | 3 | 2 | 129.2 | 80.211 | 50.516 | 48.118 | 34.323 | 31.806 | 16.525 | 9.122 | 27.199 | 45.819 | 18.112 | 18.217 | 21.455 | 22.381 | 21.828 | 22.08525 |
| Prussian carp | 1182 | 2 | 3 | 2 | 117.09 | 82.243 | 53.165 | 49.659 | 32.762 | 31.922 | 16.489 | 9.535 | 26.012 | 39.390 | 16.846 | 14.390 | 17.052 | 22.703 | 20.166 | 19.22651 |
| Prussian carp | 1183 | 2 | 3 | 2 | 129.88 | 87.308 | 55.476 | 48.575 | 30.971 | 30.063 | 16.678 | 7.381 | 29.076 | 45.471 | 15.498 | 17.594 | 19.837 | 20.333 | 21.077 | 19.93431 |
| Prussian carp | 1184 | 2 | 3 | 2 | 129.37 | 81.032 | 50.790 | 50.383 | 33.032 | 33.146 | 17.392 | 9.500 | 26.125 | 41.617 | 16.136 | 16.632 | 22.120 | 24.589 | 21.533 | 22.48099 |
| Prussian carp | 1185 | 2 | 3 | 2 | 140.07 | 81.051 | 50.289 | 49.438 | 32.157 | 31.077 | 15.096 | 8.087 | 26.783 | 42.274 | 18.819 | 18.206 | 21.145 | 25.064 | 21.870 | 20.79726 |
| Prussian carp | 1186 | 2 | 3 | 2 | 145.61 | 81.022 | 49.089 | 49.290 | 35.693 | 31.936 | 17.262 | 9.387 | 26.989 | 42.523 | 16.488 | 18.014 | 19.253 | 21.494 | 20.857 | 20.21178 |
| Prussian carp | 1187 | 2 | 3 | 2 | 119.41 | 78.701 | 49.052 | 50.543 | 31.475 | 30.747 | 14.858 | 8.438 | 27.329 | 42.724 | 19.155 | 17.056 | 21.373 | 21.316 | 21.007 | 20.07328 |
| Prussian carp | 1188 | 2 | 3 | 2 | 136.62 | 79.367 | 52.288 | 51.675 | 33.486 | 31.686 | 18.314 | 9.469 | 26.506 | 46.889 | 17.862 | 16.870 | 18.772 | 21.933 | 23.921 | 23.00404 |
| Prussian carp | 1189 | 2 | 3 | 2 | 128.71 | 80.569 | 53.126 | 49.372 | 34.047 | 31.885 | 17.308 | 8.450 | 28.219 | 46.385 | 19.938 | 17.786 | 21.058 | 21.967 | 23.827 | 22.74806 |
| Prussian carp | 1190 | 2 | 3 | 2 | 149.48 | 78.258 | 50.354 | 50.244 | 34.243 | 31.822 | 16.929 | 9.791 | 26.130 | 41.138 | 18.378 | 16.631 | 18.735 | 22.309 | 19.485 | 20.00652 |
| Prussian carp | 1161 | 2 | 3 | 3 | 137.98 | 80.791 | 48.383 | 49.847 | 30.944 | 32.375 | 16.368 | 9.971 | 27.652 | 43.585 | 17.652 | 16.535 | 22.234 | 22.240 | 22.856 | 20.56051 |
| Prussian carp | 1162 | 2 | 3 | 3 | 153.16 | 80.998 | 49.283 | 51.198 | 31.824 | 33.125 | 17.507 | 9.480 | 25.044 | 40.434 | 17.454 | 15.951 | 18.856 | 20.213 | 20.329 | 19.56719 |
| Prussian carp | 1163 | 2 | 3 | 3 | 153.79 | 78.124 | 49.992 | 51.975 | 31.776 | 32.710 | 17.351 | 9.246 | 27.573 | 40.051 | 17.725 | 15.661 | 20.100 | 20.119 | 20.456 | 17.3752 |
| Prussian carp | 1164 | 2 | 3 | 3 | 143.05 | 79.401 | 51.029 | 50.451 | 33.210 | 32.168 | 17.284 | 10.158 | 28.053 | 45.908 | 18.954 | 17.219 | 20.888 | 21.938 | 21.475 | 20.02737 |
| Prussian carp | 1165 | 2 | 3 | 3 | 122.24 | 80.287 | 49.144 | 49.324 | 31.280 | 32.120 | 17.859 | 9.332 | 27.862 | 45.508 | 19.002 | 17.296 | 19.738 | 20.948 | 21.839 | 20.26683 |
| Prussian carp | 1166 | 2 | 3 | 3 | 133.54 | 80.313 | 52.192 | 49.313 | 31.912 | 32.179 | 16.523 | 8.070 | 26.397 | 40.891 | 18.655 | 15.943 | 21.780 | 21.865 | 21.010 | 19.32518 |
| Prussian carp | 1167 | 2 | 3 | 3 | 131.76 | 79.281 | 54.069 | 52.511 | 32.071 | 33.391 | 17.322 | 9.931 | 27.260 | 42.417 | 15.435 | 16.532 | 20.603 | 20.715 | 21.520 | 19.34848 |
| Prussian carp | 1168 | 2 | 3 | 3 | 118.45 | 79.508 | 49.771 | 48.496 | 31.030 | 31.171 | 16.534 | 9.890 | 26.055 | 40.402 | 16.890 | 17.059 | 22.264 | 22.183 | 21.294 | 19.37681 |
| Prussian carp | 1169 | 2 | 3 | 3 | 126.06 | 82.366 | 52.487 | 51.605 | 33.588 | 32.892 | 17.642 | 8.521 | 27.770 | 46.079 | 16.818 | 17.417 | 22.118 | 22.992 | 22.181 | 21.76213 |
| Prussian carp | 1170 | 2 | 3 | 3 | 126.51 | 81.142 | 50.884 | 49.279 | 31.563 | 33.356 | 16.983 | 9.193 | 27.960 | 44.801 | 17.230 | 17.640 | 22.405 | 23.416 | 22.553 | 21.72809 |
| Prussian carp | 1171 | 2 | 3 | 3 | 144.35 | 80.887 | 47.838 | 51.555 | 33.142 | 32.799 | 16.479 | 8.875 | 26.653 | 43.561 | 15.438 | 16.812 | 20.863 | 20.415 | 19.888 | 19.97645 |
| Prussian carp | 1172 | 2 | 3 | 3 | 117.4 | 82.411 | 48.914 | 49.223 | 31.873 | 31.170 | 15.965 | 8.889 | 24.941 | 41.992 | 16.388 | 15.811 | 21.665 | 24.000 | 22.781 | 21.15533 |
| Prussian carp | 1173 | 2 | 3 | 3 | 127.28 | 79.569 | 50.776 | 47.138 | 32.203 | 31.468 | 16.024 | 9.569 | 26.023 | 44.431 | 17.584 | 15.019 | 20.881 | 21.391 | 21.099 | 19.22922 |
| Prussian carp | 1174 | 2 | 3 | 3 | 155.48 | 81.320 | 50.052 | 48.531 | 33.104 | 32.150 | 17.412 | 8.557 | 27.592 | 47.406 | 16.121 | 16.925 | 19.804 | 22.705 | 24.030 | 22.17996 |
| Prussian carp | 1175 | 2 | 3 | 3 | 130.89 | 79.869 | 47.306 | 47.575 | 32.133 | 31.582 | 18.375 | 8.146 | 26.549 | 48.678 | 17.787 | 17.608 | 22.375 | 19.906 | 21.239 | 22.03927 |
| Prussian carp | 1176 | 2 | 3 | 3 | 125.44 | 82.504 | 50.662 | 50.834 | 32.344 | 32.822 | 16.506 | 8.903 | 26.605 | 47.998 | 14.380 | 14.499 | 23.623 | 25.767 | 24.605 | 24.32937 |
| Prussian carp | 1177 | 2 | 3 | 3 | 126.61 | 82.771 | 50.695 | 50.858 | 34.232 | 33.305 | 16.252 | 8.083 | 27.621 | 45.093 | 17.727 | 18.447 | 21.671 | 22.015 | 23.856 | 20.88703 |
| Prussian carp | 1178 | 2 | 3 | 3 | 127.64 | 78.966 | 53.467 | 53.283 | 33.189 | 32.671 | 17.783 | 10.579 | 27.509 | 45.066 | 19.005 | 17.324 | 19.919 | 20.659 | 23.103 | 20.87561 |
| Prussian carp | 1179 | 2 | 3 | 3 | 117.02 | 78.795 | 46.927 | 49.629 | 32.558 | 31.058 | 17.188 | 9.419 | 26.339 | 42.839 | 20.133 | 15.626 | 20.162 | 20.389 | 17.886 | 18.60698 |
| Prussian carp | 1180 | 2 | 3 | 3 | 129.27 | 81.489 | 53.489 | 50.793 | 35.467 | 33.957 | 17.062 | 9.189 | 29.291 | 46.738 | 19.334 | 18.470 | 21.982 | 25.019 | 23.802 | 22.19777 |
| Prussian carp | 1181 | 2 | 3 | 3 | 129.9 | 82.484 | 51.943 | 50.375 | 33.813 | 32.025 | 16.775 | 9.088 | 25.805 | 45.318 | 18.954 | 16.940 | 21.347 | 22.155 | 22.925 | 22.52676 |
| Prussian carp | 1182 | 2 | 3 | 3 | 117.13 | 78.362 | 50.541 | 49.578 | 31.026 | 31.491 | 16.644 | 9.586 | 26.634 | 39.464 | 15.757 | 16.218 | 16.843 | 20.887 | 21.523 | 18.1562 |
| Prussian carp | 1183 | 2 | 3 | 3 | 126.82 | 81.681 | 54.288 | 50.306 | 32.096 | 30.452 | 16.223 | 8.525 | 27.685 | 44.128 | 18.589 | 17.415 | 19.259 | 19.910 | 20.765 | 20.63201 |
| Prussian carp | 1184 | 2 | 3 | 3 | 129.51 | 80.232 | 52.193 | 51.102 | 33.249 | 31.777 | 17.600 | 9.769 | 26.497 | 41.369 | 19.872 | 17.051 | 20.021 | 22.446 | 22.621 | 21.5301 |
| Prussian carp | 1185 | 2 | 3 | 3 | 138.25 | 82.364 | 52.329 | 51.228 | 33.045 | 31.465 | 15.938 | 8.535 | 27.851 | 44.049 | 17.684 | 17.822 | 21.113 | 22.362 | 23.215 | 21.40923 |
| Prussian carp | 1186 | 2 | 3 | 3 | 147.8 | 82.145 | 49.204 | 50.196 | 34.248 | 32.398 | 17.474 | 10.059 | 27.708 | 43.150 | 18.434 | 16.831 | 19.900 | 21.175 | 22.097 | 19.14458 |
| Prussian carp | 1187 | 2 | 3 | 3 | 117.15 | 78.501 | 46.915 | 49.713 | 32.788 | 31.757 | 15.544 | 8.292 | 25.700 | 41.547 | 17.797 | 17.191 | 20.040 | 21.454 | 21.172 | 22.48144 |
| Prussian carp | 1188 | 2 | 3 | 3 | 139.2 | 79.260 | 56.081 | 51.421 | 34.219 | 32.300 | 17.764 | 8.861 | 26.664 | 46.837 | 17.653 | 17.686 | 19.982 | 23.331 | 24.841 | 21.90099 |
| Prussian carp | 1189 | 2 | 3 | 3 | 129.11 | 78.845 | 53.511 | 50.455 | 32.838 | 32.007 | 17.380 | 9.220 | 28.387 | 46.575 | 17.986 | 17.289 | 21.223 | 21.775 | 20.804 | 20.04949 |
| Prussian carp | 1190 | 2 | 3 | 3 | 150.54 | 77.053 | 51.455 | 48.915 | 33.351 | 31.839 | 15.605 | 8.778 | 26.085 | 41.734 | 18.171 | 17.909 | 20.782 | 25.834 | 25.318 | 25.31975 |
| Prussian carp | 1251 | 3 | 1 | 1 | 111.79 | 80.47 | 54.01 | 50.28 | 31.23 | 32.02 | 15.89 | 8.21 | 28.02 | 47.37 | 13.70 | 17.91 | 19.84 | 18.08 | 20.79 | 18.43 |
| Prussian carp | 1252 | 3 | 1 | 1 | 129.18 | 79.87 | 53.59 | 52.29 | 32.50 | 31.36 | 16.25 | 8.67 | 28.30 | 48.23 | 7.86 | 18.00 | 19.25 | 21.62 | 21.98 | 16.65 |
| Prussian carp | 1253 | 3 | 1 | 1 | 122.26 | 80.72 | 54.65 | 50.02 | 29.43 | 30.72 | 7.96 | 7.33 | 27.14 | 46.20 | 9.56 | 17.20 | 22.16 | 21.58 | 21.32 | 19.49 |
| Prussian carp | 1254 | 3 | 1 | 1 | 120.59 | 80.82 | 55.50 | 50.31 | 32.22 | 33.98 | 16.61 | 8.95 | 28.55 | 42.39 | 13.70 | 17.61 | 19.84 | 19.74 | 23.35 | 15.88 |
| Prussian carp | 1255 | 3 | 1 | 1 | 112.04 | 78.43 | 55.87 | 44.68 | 30.22 | 29.95 | 14.69 | 8.63 | 26.67 | 44.38 | 16.34 | 16.74 | 19.04 | 22.08 | 19.42 | 19.34 |
| Prussian carp | 1256 | 3 | 1 | 1 | 121.13 | 77.51 | 52.67 | 50.92 | 31.17 | 32.56 | 14.70 | 9.39 | 27.01 | 42.47 | 12.99 | 15.84 | 17.66 | 19.03 | 18.95 | 18.47 |
| Prussian carp | 1257 | 3 | 1 | 1 | 132.05 | 82.04 | 54.72 | 44.88 | 28.23 | 29.31 | 15.83 | 7.65 | 26.69 | 42.86 | 13.15 | 16.09 | 15.70 | 20.73 | 22.07 | 15.33 |
| Prussian carp | 1258 | 3 | 1 | 1 | 127.02 | 76.29 | 53.29 | 45.61 | 29.60 | 30.74 | 14.81 | 9.08 | 26.89 | 43.42 | 11.54 | 16.58 | 20.49 | 22.94 | 20.30 | 17.77 |
| Prussian carp | 1259 | 3 | 1 | 1 | 105.46 | 82.09 | 47.99 | 48.70 | 31.89 | 30.84 | 16.91 | 8.71 | 25.97 | 42.43 | 10.65 | 16.78 | 20.77 | 20.84 | 23.81 | 17.41 |
| Prussian carp | 1260 | 3 | 1 | 1 | 109.53 | 80.41 | 45.47 | 48.05 | 31.80 | 31.69 | 14.58 | 8.89 | 27.73 | 43.26 | 10.40 | 17.15 | 20.25 | 18.59 | 23.61 | 16.78 |
| Prussian carp | 1261 | 3 | 1 | 1 | 129.6 | 81.86 | 55.46 | 49.89 | 31.99 | 31.05 | 15.64 | 8.58 | 27.92 | 39.20 | 10.86 | 17.35 | 19.89 | 15.16 | 19.99 | 21.17 |
| Prussian carp | 1262 | 3 | 1 | 1 | 135.12 | 78.83 | 53.67 | 57.13 | 31.46 | 30.62 | 15.96 | 8.43 | 26.99 | 42.90 | 12.92 | 16.34 | 19.92 | 22.33 | 20.72 | 17.19 |
| Prussian carp | 1263 | 3 | 1 | 1 | 102.45 | 82.42 | 55.19 | 52.37 | 30.82 | 32.63 | 16.80 | 8.77 | 28.66 | 44.78 | 10.89 | 16.41 | 20.25 | 19.90 | 21.36 | 18.71 |
| Prussian carp | 1264 | 3 | 1 | 1 | 126.68 | 71.56 | 50.81 | 45.82 | 28.25 | 32.02 | 14.03 | 8.04 | 25.88 | 46.32 | 10.55 | 17.68 | 17.45 | 20.96 | 21.20 | 20.08 |
| Prussian carp | 1265 | 3 | 1 | 1 | 123.79 | 75.43 | 50.32 | 47.11 | 33.04 | 31.35 | 15.19 | 9.64 | 27.90 | 41.47 | 9.87 | 17.02 | 16.34 | 17.25 | 20.49 | 15.82 |
| Prussian carp | 1266 | 3 | 1 | 1 | 119.59 | 79.20 | 48.40 | 46.52 | 29.38 | 31.06 | 15.67 | 8.12 | 26.96 | 45.23 | 10.38 | 17.37 | 21.14 | 18.74 | 19.61 | 19.10 |
| Prussian carp | 1267 | 3 | 1 | 1 | 135.6 | 79.85 | 56.16 | 48.47 | 29.90 | 32.71 | 15.14 | 8.61 | 25.66 | 44.38 | 12.19 | 17.24 | 18.74 | 20.78 | 20.56 | 17.79 |
| Prussian carp | 1268 | 3 | 1 | 1 | 111.32 | 78.28 | 44.16 | 42.75 | 29.24 | 30.84 | 14.27 | 8.65 | 27.93 | 48.22 | 17.19 | 24.08 | 19.30 | 19.59 | 22.55 | 19.23 |
| Prussian carp | 1269 | 3 | 1 | 1 | 121.99 | 78.09 | 52.04 | 51.10 | 31.06 | 32.55 | 17.55 | 9.32 | 29.48 | 40.15 | 13.24 | 15.74 | 19.87 | 14.66 | 20.02 | 17.53 |
| Prussian carp | 1270 | 3 | 1 | 1 | 125.77 | 80.92 | 48.75 | 47.35 | 32.40 | 30.95 | 14.64 | 9.55 | 27.41 | 48.44 | 8.30 | 18.52 | 21.99 | 16.81 | 23.36 | 17.47 |
| Prussian carp | 1271 | 3 | 1 | 1 | 110.63 | 81.78 | 50.83 | 52.36 | 33.77 | 33.40 | 14.62 | 10.57 | 26.39 | 42.20 | 14.75 | 16.18 | 19.07 | 16.05 | 19.86 | 16.81 |
| Prussian carp | 1272 | 3 | 1 | 1 | 112.46 | 77.26 | 51.58 | 49.51 | 30.89 | 31.01 | 15.30 | 8.88 | 27.05 | 47.37 | 12.21 | 16.64 | 20.22 | 18.92 | 20.40 | 17.46 |
| Prussian carp | 1273 | 3 | 1 | 1 | 105.72 | 80.16 | 53.62 | 51.00 | 32.89 | 31.02 | 16.32 | 8.28 | 28.13 | 45.15 | 15.09 | 16.89 | 19.03 | 14.50 | 22.79 | 16.12 |
| Prussian carp | 1274 | 3 | 1 | 1 | 118.7 | 78.70 | 50.92 | 49.28 | 32.51 | 30.22 | 16.79 | 8.47 | 28.52 | 42.39 | 11.41 | 16.33 | 20.46 | 19.77 | 18.51 | 17.41 |
| Prussian carp | 1275 | 3 | 1 | 1 | 145.27 | 78.05 | 54.49 | 48.93 | 29.58 | 31.16 | 17.52 | 8.27 | 26.78 | 45.84 | 10.78 | 16.89 | 19.60 | 18.34 | 21.98 | 21.79 |
| Prussian carp | 4030 | 3 | 1 | 1 | 119.45 | 79.41 | 49.97 | 49.44 | 35.94 | 33.96 | 16.46 | 9.70 | 30.08 | 45.32 | 13.66 | 16.59 | 18.60 | 20.29 | 21.26 | 21.50 |
| Prussian carp | 4031 | 3 | 1 | 1 | 115.16 | 82.01 | 53.27 | 48.65 | 31.08 | 31.57 | 12.63 | 8.69 | 27.46 | 45.14 | 16.03 | 21.11 | 21.47 | 20.24 | 21.91 | 23.25 |
| Prussian carp | 4032 | 3 | 1 | 1 | 98.19 | 75.95 | 57.53 | 52.03 | 29.51 | 30.90 | 15.58 | 7.27 | 25.33 | 45.45 | 12.21 | 15.04 | 20.75 | 19.71 | 22.56 | 19.00 |
| Prussian carp | 4033 | 3 | 1 | 1 | 129.18 | 79.82 | 54.16 | 49.51 | 31.18 | 30.62 | 14.38 | 9.96 | 28.42 | 47.98 | 10.22 | 18.69 | 20.37 | 19.34 | 21.65 | 18.44 |
| Prussian carp | 4034 | 3 | 1 | 1 | 108.92 | 83.57 | 57.28 | 52.61 | 33.09 | 32.24 | 15.13 | 7.77 | 28.53 | 45.17 | 9.49 | 17.72 | 16.63 | 18.35 | 22.19 | 17.02 |
| Prussian carp | 1251 | 3 | 1 | 2 | 111.9 | 78.15 | 49.95 | 48.45 | 32.60 | 30.09 | 16.14 | 8.49 | 27.70 | 48.11 | 11.52 | 17.48 | 17.60 | 16.19 | 20.45 | 15.18 |
| Prussian carp | 1252 | 3 | 1 | 2 | 129.6 | 79.84 | 51.49 | 50.42 | 34.04 | 30.66 | 17.16 | 8.86 | 28.31 | 35.38 | 9.99 | 17.95 | 17.66 | 17.56 | 21.47 | 15.58 |
| Prussian carp | 1253 | 3 | 1 | 2 | 122.6 | 79.09 | 53.23 | 49.51 | 32.07 | 30.04 | 16.32 | 7.72 | 28.49 | 45.84 | 10.95 | 17.30 | 19.47 | 15.55 | 21.37 | 16.41 |
| Prussian carp | 1254 | 3 | 1 | 2 | 120.56 | 79.80 | 47.30 | 49.69 | 33.78 | 30.75 | 16.62 | 8.92 | 28.05 | 42.21 | 11.74 | 16.43 | 21.63 | 17.96 | 24.30 | 16.97 |
| Prussian carp | 1255 | 3 | 1 | 2 | 112.69 | 81.74 | 50.13 | 46.69 | 33.55 | 30.37 | 14.92 | 9.37 | 28.12 | 45.51 | 11.43 | 16.36 | 17.97 | 16.87 | 19.90 | 14.56 |
| Prussian carp | 1256 | 3 | 1 | 2 | 121.4 | 78.70 | 52.47 | 48.56 | 32.62 | 30.70 | 15.52 | 8.30 | 24.64 | 44.31 | 11.00 | 16.28 | 17.42 | 16.14 | 18.71 | 14.60 |
| Prussian carp | 1257 | 3 | 1 | 2 | 130.73 | 80.70 | 53.60 | 48.87 | 32.35 | 30.20 | 15.60 | 8.40 | 29.47 | 44.86 | 14.49 | 16.42 | 14.72 | 13.60 | 21.02 | 18.02 |
| Prussian carp | 1258 | 3 | 1 | 2 | 128.12 | 80.63 | 49.53 | 47.05 | 31.59 | 30.19 | 14.91 | 8.97 | 25.82 | 43.95 | 15.76 | 16.77 | 15.53 | 17.96 | 22.26 | 17.91 |
| Prussian carp | 1259 | 3 | 1 | 2 | 109.6 | 78.63 | 51.62 | 48.28 | 32.92 | 29.69 | 15.74 | 9.68 | 25.91 | 43.35 | 9.41 | 16.46 | 17.60 | 15.99 | 22.19 | 15.65 |
| Prussian carp | 1260 | 3 | 1 | 2 | 109.7 | 79.91 | 46.08 | 47.56 | 33.05 | 30.11 | 14.11 | 9.63 | 28.79 | 44.08 | 9.72 | 17.23 | 19.31 | 14.46 | 23.35 | 14.73 |
| Prussian carp | 1261 | 3 | 1 | 2 | 128.51 | 78.83 | 55.18 | 48.07 | 33.56 | 29.80 | 15.63 | 8.35 | 26.92 | 43.85 | 13.15 | 16.01 | 16.26 | 15.67 | 19.70 | 14.78 |
| Prussian carp | 1262 | 3 | 1 | 2 | 136.69 | 79.26 | 53.52 | 47.98 | 32.75 | 30.12 | 15.70 | 8.90 | 27.97 | 42.26 | 13.53 | 16.05 | 17.35 | 17.47 | 21.36 | 15.43 |
| Prussian carp | 1263 | 3 | 1 | 2 | 103.9 | 82.21 | 55.11 | 50.01 | 32.00 | 30.48 | 15.89 | 9.21 | 28.81 | 44.24 | 10.76 | 16.40 | 16.87 | 14.49 | 20.96 | 14.83 |
| Prussian carp | 1264 | 3 | 1 | 2 | 128 | 75.73 | 47.91 | 44.91 | 30.19 | 30.70 | 15.22 | 8.23 | 26.60 | 48.59 | 11.56 | 17.81 | 15.08 | 17.28 | 21.23 | 15.93 |
| Prussian carp | 1265 | 3 | 1 | 2 | 127.86 | 80.65 | 52.95 | 48.09 | 34.08 | 30.57 | 15.27 | 9.10 | 26.52 | 42.79 | 11.44 | 17.04 | 18.41 | 17.12 | 20.97 | 16.15 |
| Prussian carp | 1266 | 3 | 1 | 2 | 119.75 | 79.75 | 50.41 | 45.41 | 31.82 | 29.38 | 14.06 | 9.10 | 28.48 | 45.79 | 10.34 | 16.42 | 19.32 | 18.89 | 18.44 | 16.02 |
| Prussian carp | 1267 | 3 | 1 | 2 | 134.54 | 80.59 | 53.00 | 48.29 | 34.65 | 31.49 | 16.96 | 9.53 | 26.42 | 44.65 | 9.55 | 16.60 | 17.37 | 16.09 | 22.39 | 16.82 |
| Prussian carp | 1268 | 3 | 1 | 2 | 112.63 | 78.90 | 46.51 | 45.01 | 34.31 | 31.24 | 15.56 | 9.88 | 28.28 | 48.98 | 8.60 | 18.19 | 17.01 | 18.12 | 20.83 | 16.09 |
| Prussian carp | 1269 | 3 | 1 | 2 | 122.2 | 79.93 | 54.75 | 52.28 | 35.33 | 33.20 | 16.83 | 10.10 | 27.81 | 41.75 | 10.42 | 16.53 | 19.82 | 14.26 | 19.19 | 15.46 |
| Prussian carp | 1270 | 3 | 1 | 2 | 124.49 | 79.83 | 48.40 | 46.54 | 32.57 | 30.72 | 14.82 | 9.86 | 28.10 | 48.38 | 8.67 | 17.01 | 20.91 | 19.58 | 23.98 | 16.60 |
| Prussian carp | 1271 | 3 | 1 | 2 | 109.96 | 80.06 | 52.65 | 50.87 | 36.71 | 32.14 | 14.04 | 10.02 | 27.27 | 42.43 | 11.92 | 15.54 | 17.17 | 15.54 | 19.13 | 16.67 |
| Prussian carp | 1272 | 3 | 1 | 2 | 113.85 | 77.51 | 49.65 | 46.66 | 32.54 | 29.71 | 15.75 | 9.03 | 27.07 | 46.50 | 14.44 | 16.19 | 17.09 | 15.55 | 19.53 | 15.86 |
| Prussian carp | 1273 | 3 | 1 | 2 | 105.77 | 80.91 | 55.72 | 50.53 | 34.58 | 31.02 | 16.03 | 9.61 | 27.44 | 45.51 | 14.40 | 16.88 | 19.28 | 14.21 | 22.53 | 15.99 |
| Prussian carp | 1274 | 3 | 1 | 2 | 120 | 81.91 | 55.61 | 51.14 | 36.30 | 33.05 | 17.58 | 9.73 | 29.47 | 45.26 | 13.06 | 17.13 | 18.45 | 18.68 | 20.49 | 15.44 |
| Prussian carp | 1275 | 3 | 1 | 2 | 144.75 | 79.55 | 52.45 | 48.32 | 33.13 | 28.73 | 15.48 | 8.69 | 26.92 | 47.61 | 9.69 | 17.08 | 21.69 | 18.78 | 21.33 | 16.83 |
| Prussian carp | 4030 | 3 | 1 | 2 | 118.31 | 80.67 | 53.02 | 46.41 | 35.65 | 32.98 | 16.03 | 9.67 | 29.84 | 45.82 | 12.39 | 16.40 | 17.50 | 16.29 | 19.63 | 12.56 |
| Prussian carp | 4031 | 3 | 1 | 2 | 115 | 83.39 | 47.50 | 46.72 | 33.74 | 31.20 | 15.35 | 9.37 | 26.83 | 45.25 | 8.86 | 16.48 | 17.33 | 19.36 | 21.69 | 16.62 |
| Prussian carp | 4032 | 3 | 1 | 2 | 99.06 | 80.14 | 52.46 | 48.44 | 32.95 | 29.23 | 14.90 | 7.19 | 25.97 | 46.39 | 13.61 | 17.54 | 18.12 | 17.94 | 23.44 | 17.04 |
| Prussian carp | 4033 | 3 | 1 | 2 | 129.4 | 77.37 | 50.86 | 48.08 | 31.84 | 29.32 | 14.06 | 8.68 | 27.13 | 47.97 | 10.97 | 17.86 | 19.24 | 16.17 | 22.69 | 16.71 |
| Prussian carp | 4034 | 3 | 1 | 2 | 109.85 | 81.10 | 54.93 | 49.32 | 33.98 | 31.10 | 16.05 | 8.44 | 28.09 | 46.70 | 12.06 | 16.79 | 15.28 | 17.58 | 21.07 | 14.59 |
| Prussian carp | 1251 | 3 | 1 | 3 | 111.79 | 78.96 | 52.61 | 50.52 | 32.55 | 29.65 | 15.90 | 8.48 | 26.92 | 48.45 | 12.02 | 17.26 | 18.96 | 18.21 | 21.06 | 15.62 |
| Prussian carp | 1252 | 3 | 1 | 3 | 129.83 | 78.99 | 49.69 | 51.37 | 34.21 | 30.55 | 16.56 | 8.98 | 29.31 | 47.69 | 10.31 | 17.67 | 18.07 | 17.58 | 22.00 | 14.13 |
| Prussian carp | 1253 | 3 | 1 | 3 | 121.58 | 80.66 | 52.36 | 47.34 | 31.84 | 29.52 | 16.07 | 7.36 | 29.01 | 46.38 | 10.01 | 16.68 | 21.13 | 15.44 | 21.75 | 15.72 |
| Prussian carp | 1254 | 3 | 1 | 3 | 120.54 | 80.19 | 48.61 | 51.41 | 35.12 | 32.82 | 16.73 | 8.53 | 28.55 | 41.73 | 10.03 | 16.37 | 20.41 | 15.84 | 24.29 | 18.70 |
| Prussian carp | 1255 | 3 | 1 | 3 | 113.79 | 82.32 | 50.22 | 46.52 | 34.69 | 30.50 | 15.04 | 9.50 | 28.68 | 44.96 | 10.20 | 17.10 | 19.63 | 15.39 | 20.46 | 12.47 |
| Prussian carp | 1256 | 3 | 1 | 3 | 121.44 | 78.84 | 52.66 | 49.02 | 33.25 | 30.70 | 15.30 | 9.47 | 29.03 | 45.05 | 13.96 | 15.81 | 16.47 | 13.28 | 17.05 | 13.41 |
| Prussian carp | 1257 | 3 | 1 | 3 | 130.72 | 79.02 | 50.96 | 47.84 | 31.75 | 29.14 | 15.04 | 7.94 | 28.08 | 44.40 | 12.89 | 16.05 | 13.51 | 12.56 | 19.96 | 16.96 |
| Prussian carp | 1258 | 3 | 1 | 3 | 128.57 | 81.64 | 48.40 | 46.60 | 33.41 | 30.76 | 15.41 | 8.95 | 26.56 | 44.71 | 12.02 | 16.90 | 17.54 | 18.46 | 21.19 | 19.86 |
| Prussian carp | 1259 | 3 | 1 | 3 | 107.41 | 78.78 | 50.81 | 49.14 | 33.77 | 30.46 | 15.09 | 9.26 | 27.57 | 43.71 | 9.10 | 16.52 | 17.72 | 16.91 | 23.23 | 14.84 |
| Prussian carp | 1260 | 3 | 1 | 3 | 110.49 | 80.58 | 48.05 | 46.43 | 32.70 | 30.52 | 14.50 | 7.95 | 28.03 | 43.71 | 12.22 | 16.74 | 18.92 | 15.72 | 22.55 | 14.41 |
| Prussian carp | 1261 | 3 | 1 | 3 | 129.76 | 78.80 | 50.89 | 48.81 | 32.38 | 28.21 | 14.51 | 9.30 | 31.01 | 44.33 | 10.24 | 16.85 | 19.66 | 17.75 | 21.55 | 15.50 |
| Prussian carp | 1262 | 3 | 1 | 3 | 135.45 | 79.47 | 53.49 | 47.40 | 32.37 | 29.85 | 15.29 | 9.10 | 29.35 | 43.11 | 12.21 | 16.37 | 16.97 | 16.35 | 20.94 | 15.74 |
| Prussian carp | 1263 | 3 | 1 | 3 | 103.74 | 83.32 | 55.21 | 52.40 | 33.61 | 30.62 | 15.77 | 9.24 | 29.26 | 45.37 | 10.29 | 16.24 | 17.93 | 17.33 | 20.58 | 16.51 |
| Prussian carp | 1264 | 3 | 1 | 3 | 127.44 | 76.11 | 48.60 | 45.59 | 32.44 | 30.48 | 15.50 | 8.07 | 27.52 | 47.38 | 10.05 | 17.21 | 15.49 | 16.67 | 20.46 | 16.70 |
| Prussian carp | 1265 | 3 | 1 | 3 | 123.8 | 79.50 | 50.52 | 47.29 | 33.66 | 30.49 | 15.51 | 9.26 | 28.33 | 43.33 | 10.14 | 17.26 | 16.76 | 18.08 | 21.42 | 16.23 |
| Prussian carp | 1266 | 3 | 1 | 3 | 119.96 | 75.68 | 48.46 | 44.90 | 31.53 | 29.10 | 13.71 | 9.21 | 27.15 | 46.29 | 10.45 | 17.53 | 19.06 | 17.25 | 22.36 | 17.17 |
| Prussian carp | 1267 | 3 | 1 | 3 | 136.01 | 78.32 | 47.90 | 46.37 | 33.34 | 31.33 | 13.91 | 9.53 | 26.90 | 44.04 | 10.14 | 16.62 | 16.82 | 15.09 | 21.56 | 17.18 |
| Prussian carp | 1268 | 3 | 1 | 3 | 111.5 | 76.43 | 49.79 | 46.14 | 32.64 | 30.74 | 16.30 | 10.45 | 27.76 | 48.42 | 12.07 | 18.09 | 15.51 | 15.05 | 20.40 | 17.56 |
| Prussian carp | 1269 | 3 | 1 | 3 | 123.92 | 78.09 | 47.69 | 54.03 | 32.19 | 30.34 | 15.99 | 9.08 | 28.63 | 40.39 | 11.58 | 15.63 | 19.69 | 15.52 | 19.84 | 14.09 |
| Prussian carp | 1270 | 3 | 1 | 3 | 125.2 | 80.73 | 49.00 | 46.80 | 33.16 | 30.69 | 14.34 | 9.67 | 27.92 | 47.64 | 8.30 | 17.65 | 21.53 | 16.69 | 23.34 | 16.66 |
| Prussian carp | 1271 | 3 | 1 | 3 | 111.19 | 80.72 | 52.04 | 49.06 | 34.37 | 30.99 | 14.36 | 9.60 | 28.54 | 44.00 | 11.18 | 15.32 | 18.68 | 14.82 | 18.32 | 16.33 |
| Prussian carp | 1272 | 3 | 1 | 3 | 113.62 | 78.26 | 53.88 | 49.00 | 32.14 | 29.59 | 14.49 | 9.19 | 28.41 | 46.81 | 12.98 | 16.70 | 20.76 | 19.00 | 20.05 | 15.53 |
| Prussian carp | 1273 | 3 | 1 | 3 | 105.91 | 82.49 | 53.22 | 48.05 | 32.94 | 30.79 | 15.46 | 9.63 | 27.17 | 44.69 | 14.62 | 17.86 | 17.43 | 14.67 | 21.04 | 16.54 |
| Prussian carp | 1274 | 3 | 1 | 3 | 120.51 | 79.50 | 54.62 | 46.84 | 33.23 | 30.36 | 16.53 | 8.99 | 30.98 | 44.43 | 11.94 | 16.47 | 18.94 | 17.90 | 20.38 | 14.98 |
| Prussian carp | 1275 | 3 | 1 | 3 | 143.62 | 77.19 | 49.21 | 46.82 | 32.58 | 29.69 | 15.43 | 10.27 | 27.26 | 46.39 | 16.94 | 17.55 | 17.95 | 19.59 | 23.04 | 16.26 |
| Prussian carp | 4030 | 3 | 1 | 3 | 120.08 | 74.90 | 49.69 | 45.42 | 34.88 | 31.86 | 16.38 | 9.35 | 28.49 | 44.07 | 10.78 | 16.29 | 16.01 | 15.16 | 21.24 | 15.00 |
| Prussian carp | 4031 | 3 | 1 | 3 | 116.12 | 82.20 | 48.86 | 47.35 | 33.82 | 31.04 | 15.48 | 8.31 | 25.90 | 45.48 | 9.60 | 17.00 | 18.79 | 21.13 | 21.37 | 19.15 |
| Prussian carp | 4032 | 3 | 1 | 3 | 98.71 | 81.98 | 50.50 | 47.41 | 33.49 | 29.55 | 14.97 | 8.00 | 28.90 | 46.75 | 11.62 | 16.57 | 19.25 | 16.83 | 22.56 | 15.89 |
| Prussian carp | 4033 | 3 | 1 | 3 | 129.76 | 78.35 | 48.88 | 46.58 | 31.87 | 29.19 | 14.18 | 8.41 | 26.35 | 47.85 | 10.89 | 17.59 | 19.35 | 16.87 | 22.05 | 14.38 |
| Prussian carp | 4034 | 3 | 1 | 3 | 108.45 | 79.57 | 57.12 | 48.44 | 32.23 | 29.64 | 15.50 | 8.37 | 29.14 | 46.83 | 9.34 | 16.48 | 16.92 | 17.34 | 21.98 | 15.66 |
| Prussian carp | 1251 | 3 | 2 | 1 | 110.02 | 77.45 | 50.26 | 47.21 | 28.85 | 31.38 | 18.89 | 6.33 | 27.87 | 46.90 | 17.72 | 17.61 | 20.42 | 25.11 | 21.79 | 20.82 |
| Prussian carp | 1252 | 3 | 2 | 1 | 105.51 | 79.07 | 51.74 | 51.65 | 32.13 | 36.07 | 19.00 | 7.57 | 26.67 | 47.96 | 18.06 | 18.72 | 21.05 | 25.57 | 21.73 | 20.49 |
| Prussian carp | 1253 | 3 | 2 | 1 | 109.17 | 80.71 | 48.74 | 45.38 | 32.15 | 36.62 | 18.39 | 6.36 | 27.85 | 45.47 | 17.15 | 17.46 | 22.87 | 23.49 | 22.36 | 22.51 |
| Prussian carp | 1254 | 3 | 2 | 1 | 72.46 | 75.82 | 48.94 | 50.28 | 29.21 | 32.26 | 18.02 | 8.44 | 26.63 | 40.75 | 17.16 | 16.30 | 21.12 | 26.25 | 23.70 | 22.34 |
| Prussian carp | 1255 | 3 | 2 | 1 | 96.77 | 81.06 | 50.37 | 44.37 | 26.03 | 31.22 | 17.82 | 7.55 | 30.35 | 46.34 | 20.70 | 17.08 | 20.39 | 23.17 | 21.93 | 20.97 |
| Prussian carp | 1256 | 3 | 2 | 1 | 93.76 | 76.18 | 55.11 | 52.59 | 29.94 | 32.25 | 16.03 | 8.12 | 28.51 | 44.63 | 19.46 | 16.53 | 18.93 | 21.03 | 18.90 | 18.05 |
| Prussian carp | 1257 | 3 | 2 | 1 | 115.79 | 81.48 | 51.43 | 46.18 | 26.80 | 30.08 | 17.76 | 5.17 | 28.17 | 42.58 | 16.71 | 15.86 | 21.11 | 19.66 | 23.99 | 21.71 |
| Prussian carp | 1258 | 3 | 2 | 1 | 103.14 | 81.45 | 52.26 | 47.68 | 27.64 | 31.36 | 17.11 | 7.63 | 26.38 | 44.66 | 21.11 | 17.27 | 20.57 | 25.31 | 21.62 | 20.95 |
| Prussian carp | 1259 | 3 | 2 | 1 | 61 | 80.79 | 48.35 | 49.55 | 29.85 | 31.26 | 17.23 | 6.61 | 26.04 | 42.48 | 16.32 | 17.17 | 20.76 | 25.26 | 23.53 | 20.59 |
| Prussian carp | 1260 | 3 | 2 | 1 | 101 | 82.96 | 49.12 | 46.31 | 26.88 | 30.23 | 16.99 | 6.68 | 26.05 | 44.47 | 18.05 | 17.94 | 21.14 | 21.29 | 22.52 | 22.82 |
| Prussian carp | 1261 | 3 | 2 | 1 | 59.79 | 81.70 | 51.19 | 49.63 | 27.26 | 31.45 | 17.55 | 6.69 | 29.05 | 43.90 | 17.44 | 18.13 | 19.56 | 23.12 | 19.78 | 20.27 |
| Prussian carp | 1262 | 3 | 2 | 1 | 61.27 | 80.97 | 50.65 | 49.29 | 28.89 | 30.11 | 15.87 | 6.97 | 28.99 | 43.96 | 19.13 | 15.88 | 20.86 | 21.48 | 19.98 | 22.20 |
| Prussian carp | 1263 | 3 | 2 | 1 | 60.83 | 83.49 | 51.01 | 50.19 | 29.48 | 30.32 | 17.51 | 7.29 | 27.16 | 45.36 | 16.75 | 16.59 | 19.42 | 21.14 | 19.80 | 19.14 |
| Prussian carp | 1264 | 3 | 2 | 1 | 51.14 | 74.51 | 51.98 | 48.93 | 31.82 | 36.01 | 17.18 | 7.77 | 26.77 | 46.25 | 18.44 | 20.28 | 20.26 | 25.57 | 22.07 | 22.01 |
| Prussian carp | 1265 | 3 | 2 | 1 | 49.44 | 80.82 | 47.45 | 48.04 | 30.66 | 28.62 | 17.63 | 5.26 | 26.55 | 44.51 | 16.72 | 18.03 | 20.20 | 23.65 | 20.86 | 20.19 |
| Prussian carp | 1266 | 3 | 2 | 1 | 58.5 | 75.29 | 45.02 | 47.97 | 26.05 | 29.86 | 16.33 | 6.20 | 25.50 | 44.40 | 16.38 | 16.92 | 20.13 | 24.63 | 22.11 | 22.23 |
| Prussian carp | 1267 | 3 | 2 | 1 | 54.43 | 81.93 | 49.63 | 51.44 | 28.05 | 30.50 | 17.29 | 8.09 | 26.27 | 44.31 | 16.46 | 16.88 | 21.00 | 21.55 | 20.05 | 22.22 |
| Prussian carp | 1268 | 3 | 2 | 1 | 51.5 | 81.25 | 48.11 | 47.30 | 30.00 | 32.26 | 18.38 | 6.32 | 27.87 | 47.66 | 19.22 | 14.20 | 20.32 | 23.75 | 23.27 | 19.76 |
| Prussian carp | 1269 | 3 | 2 | 1 | 49.27 | 79.93 | 51.30 | 50.85 | 29.34 | 33.03 | 18.78 | 7.72 | 28.07 | 38.35 | 18.16 | 16.01 | 20.67 | 21.07 | 20.25 | 18.75 |
| Prussian carp | 1270 | 3 | 2 | 1 | 49.93 | 85.15 | 49.15 | 53.49 | 30.64 | 33.18 | 16.24 | 7.41 | 27.80 | 48.61 | 17.19 | 18.30 | 21.75 | 26.32 | 25.37 | 23.76 |
| Prussian carp | 1271 | 3 | 2 | 1 | 49.63 | 80.15 | 53.67 | 50.19 | 27.34 | 30.25 | 15.84 | 7.37 | 26.46 | 41.22 | 17.42 | 15.94 | 21.40 | 21.41 | 20.42 | 18.92 |
| Prussian carp | 1272 | 3 | 2 | 1 | 53.4 | 80.66 | 52.53 | 49.32 | 32.03 | 32.94 | 17.28 | 8.79 | 27.49 | 47.96 | 14.80 | 16.91 | 19.66 | 22.75 | 20.37 | 22.59 |
| Prussian carp | 1273 | 3 | 2 | 1 | 51.78 | 83.11 | 49.68 | 47.21 | 29.98 | 32.27 | 18.06 | 7.62 | 27.51 | 44.90 | 17.07 | 17.33 | 19.24 | 22.79 | 19.90 | 19.84 |
| Prussian carp | 1274 | 3 | 2 | 1 | 58.98 | 79.22 | 50.54 | 51.28 | 28.85 | 31.26 | 16.86 | 7.05 | 26.98 | 43.77 | 18.63 | 16.84 | 20.87 | 22.53 | 18.54 | 19.94 |
| Prussian carp | 1275 | 3 | 2 | 1 | 45.62 | 82.04 | 51.47 | 52.10 | 29.31 | 31.50 | 16.75 | 7.90 | 26.90 | 47.37 | 17.97 | 14.68 | 22.63 | 24.37 | 21.55 | 23.08 |
| Prussian carp | 4030 | 3 | 2 | 1 | 61.05 | 80.47 | 53.41 | 47.75 | 28.62 | 33.73 | 18.79 | 7.85 | 28.51 | 43.77 | 17.28 | 16.59 | 19.45 | 25.48 | 23.17 | 21.01 |
| Prussian carp | 4031 | 3 | 2 | 1 | 48.63 | 81.75 | 51.26 | 50.69 | 25.79 | 30.18 | 17.03 | 6.13 | 28.32 | 46.55 | 14.56 | 17.50 | 19.27 | 26.14 | 21.43 | 19.50 |
| Prussian carp | 4032 | 3 | 2 | 1 | 47.41 | 80.64 | 50.43 | 49.16 | 28.94 | 31.21 | 17.80 | 5.97 | 25.55 | 46.32 | 17.50 | 17.23 | 20.58 | 23.69 | 22.69 | 22.12 |
| Prussian carp | 4033 | 3 | 2 | 1 | 56.44 | 77.23 | 48.95 | 47.67 | 27.94 | 29.51 | 15.77 | 6.12 | 27.09 | 49.28 | 19.32 | 17.48 | 20.49 | 23.94 | 22.73 | 21.72 |
| Prussian carp | 4034 | 3 | 2 | 1 | 46.37 | 82.26 | 49.23 | 52.43 | 29.09 | 32.21 | 17.92 | 6.15 | 29.07 | 45.39 | 16.92 | 17.80 | 18.78 | 21.87 | 21.64 | 20.19 |
| Prussian carp | 1251 | 3 | 2 | 2 | 109.35 | 78.97 | 46.81 | 49.85 | 31.18 | 31.21 | 17.82 | 6.57 | 27.56 | 47.67 | 17.79 | 18.11 | 19.99 | 23.45 | 21.19 | 19.90 |
| Prussian carp | 1252 | 3 | 2 | 2 | 107.07 | 80.37 | 45.43 | 49.37 | 29.76 | 33.09 | 18.09 | 7.06 | 28.20 | 47.56 | 17.72 | 18.56 | 19.97 | 24.32 | 21.81 | 20.20 |
| Prussian carp | 1253 | 3 | 2 | 2 | 109.95 | 80.66 | 47.29 | 48.62 | 27.82 | 31.60 | 18.18 | 5.73 | 28.71 | 46.63 | 16.88 | 17.93 | 23.51 | 23.39 | 22.31 | 20.56 |
| Prussian carp | 1254 | 3 | 2 | 2 | 71.75 | 80.13 | 48.74 | 51.63 | 29.12 | 30.47 | 18.14 | 6.70 | 28.42 | 41.47 | 16.91 | 16.75 | 21.82 | 26.07 | 25.35 | 22.55 |
| Prussian carp | 1255 | 3 | 2 | 2 | 97.85 | 83.12 | 49.30 | 47.41 | 27.72 | 30.77 | 16.93 | 6.93 | 27.28 | 47.09 | 18.76 | 16.53 | 21.06 | 22.07 | 21.20 | 21.76 |
| Prussian carp | 1256 | 3 | 2 | 2 | 93.5 | 79.27 | 52.12 | 49.16 | 30.06 | 30.23 | 17.02 | 8.27 | 28.09 | 43.02 | 19.54 | 16.54 | 18.40 | 19.43 | 19.44 | 17.39 |
| Prussian carp | 1257 | 3 | 2 | 2 | 116.59 | 78.48 | 51.41 | 46.09 | 28.00 | 31.01 | 16.97 | 7.51 | 25.69 | 42.66 | 16.98 | 19.06 | 16.57 | 22.68 | 17.03 | 19.98 |
| Prussian carp | 1258 | 3 | 2 | 2 | 104.09 | 82.06 | 52.12 | 49.74 | 28.84 | 32.87 | 16.79 | 7.50 | 25.96 | 44.68 | 18.88 | 17.24 | 20.52 | 24.86 | 20.82 | 22.19 |
| Prussian carp | 1259 | 3 | 2 | 2 | 60.76 | 79.01 | 46.46 | 45.55 | 29.39 | 30.25 | 18.02 | 7.66 | 26.13 | 43.11 | 18.06 | 18.03 | 22.10 | 24.60 | 23.80 | 19.56 |
| Prussian carp | 1260 | 3 | 2 | 2 | 102.02 | 82.83 | 48.90 | 48.67 | 27.97 | 30.95 | 17.00 | 7.38 | 28.62 | 43.81 | 19.09 | 17.43 | 20.51 | 21.87 | 22.55 | 17.69 |
| Prussian carp | 1261 | 3 | 2 | 2 | 60.51 | 81.61 | 50.88 | 50.29 | 28.81 | 30.01 | 17.65 | 6.33 | 26.64 | 43.63 | 16.90 | 17.28 | 20.28 | 21.85 | 21.61 | 20.35 |
| Prussian carp | 1262 | 3 | 2 | 2 | 61.58 | 79.75 | 52.84 | 49.07 | 29.46 | 32.54 | 17.75 | 5.43 | 28.65 | 43.62 | 20.37 | 17.03 | 19.18 | 21.88 | 18.83 | 18.86 |
| Prussian carp | 1263 | 3 | 2 | 2 | 64.24 | 79.84 | 51.61 | 45.27 | 26.88 | 30.12 | 16.03 | 7.22 | 26.57 | 43.81 | 16.02 | 16.99 | 20.62 | 20.36 | 20.94 | 18.74 |
| Prussian carp | 1264 | 3 | 2 | 2 | 51.11 | 75.51 | 47.23 | 46.21 | 25.23 | 30.70 | 17.39 | 5.90 | 26.77 | 47.03 | 16.11 | 18.09 | 19.34 | 24.34 | 20.71 | 21.30 |
| Prussian carp | 1265 | 3 | 2 | 2 | 48.78 | 77.52 | 50.09 | 43.84 | 26.89 | 28.99 | 16.24 | 6.56 | 26.55 | 43.01 | 20.22 | 17.97 | 17.47 | 23.47 | 22.53 | 20.77 |
| Prussian carp | 1266 | 3 | 2 | 2 | 57.59 | 78.38 | 46.74 | 50.32 | 25.39 | 28.61 | 15.45 | 7.28 | 25.58 | 45.01 | 17.59 | 14.81 | 21.15 | 23.03 | 24.22 | 22.23 |
| Prussian carp | 1267 | 3 | 2 | 2 | 55.24 | 81.82 | 51.53 | 50.89 | 30.32 | 31.18 | 15.37 | 7.59 | 25.70 | 45.30 | 15.72 | 17.35 | 19.51 | 22.24 | 20.72 | 21.34 |
| Prussian carp | 1268 | 3 | 2 | 2 | 52.13 | 78.58 | 52.34 | 44.84 | 27.30 | 30.42 | 17.85 | 7.11 | 28.76 | 47.86 | 16.49 | 17.70 | 20.27 | 24.27 | 20.97 | 20.00 |
| Prussian carp | 1269 | 3 | 2 | 2 | 48.85 | 81.28 | 53.24 | 48.18 | 29.87 | 32.66 | 17.45 | 8.43 | 27.11 | 39.72 | 17.98 | 15.88 | 20.72 | 22.36 | 19.60 | 18.88 |
| Prussian carp | 1270 | 3 | 2 | 2 | 49.87 | 76.08 | 49.41 | 46.94 | 28.56 | 31.97 | 16.37 | 7.38 | 26.18 | 47.51 | 16.11 | 17.81 | 22.26 | 23.41 | 22.01 | 20.93 |
| Prussian carp | 1271 | 3 | 2 | 2 | 49.32 | 81.49 | 52.31 | 53.35 | 30.71 | 31.70 | 15.00 | 8.19 | 27.24 | 41.34 | 19.41 | 16.02 | 18.99 | 20.60 | 20.90 | 19.23 |
| Prussian carp | 1272 | 3 | 2 | 2 | 54.07 | 80.22 | 50.44 | 48.30 | 28.34 | 30.93 | 18.16 | 7.98 | 27.69 | 47.83 | 18.62 | 16.19 | 20.55 | 22.13 | 21.20 | 20.13 |
| Prussian carp | 1273 | 3 | 2 | 2 | 51.38 | 80.73 | 50.04 | 50.52 | 29.50 | 30.93 | 16.74 | 7.62 | 29.06 | 45.02 | 17.61 | 17.60 | 20.79 | 22.83 | 21.83 | 19.76 |
| Prussian carp | 1274 | 3 | 2 | 2 | 58.43 | 77.95 | 51.86 | 48.89 | 31.10 | 26.70 | 16.71 | 8.05 | 28.65 | 43.02 | 18.40 | 16.75 | 19.62 | 19.97 | 19.28 | 21.04 |
| Prussian carp | 1275 | 3 | 2 | 2 | 46.05 | 79.56 | 44.02 | 48.37 | 28.51 | 29.99 | 17.85 | 5.87 | 26.11 | 46.19 | 17.37 | 17.53 | 20.39 | 24.51 | 22.37 | 22.66 |
| Prussian carp | 4030 | 3 | 2 | 2 | 62.42 | 78.74 | 51.32 | 50.49 | 30.33 | 32.72 | 18.12 | 7.59 | 27.44 | 43.77 | 17.20 | 16.86 | 19.76 | 23.19 | 21.99 | 22.04 |
| Prussian carp | 4031 | 3 | 2 | 2 | 49.11 | 83.73 | 48.10 | 49.38 | 30.18 | 33.10 | 18.77 | 9.98 | 28.09 | 45.78 | 14.38 | 17.51 | 19.52 | 25.96 | 21.66 | 20.59 |
| Prussian carp | 4032 | 3 | 2 | 2 | 47.42 | 78.73 | 49.97 | 45.58 | 28.13 | 30.21 | 17.32 | 8.12 | 27.05 | 47.43 | 17.99 | 18.34 | 21.71 | 24.07 | 23.85 | 22.20 |
| Prussian carp | 4033 | 3 | 2 | 2 | 56.26 | 80.29 | 48.17 | 47.80 | 25.57 | 28.26 | 15.29 | 7.37 | 27.58 | 49.58 | 17.59 | 18.87 | 20.24 | 24.10 | 23.24 | 20.96 |
| Prussian carp | 4034 | 3 | 2 | 2 | 46.2 | 79.35 | 52.47 | 46.82 | 26.52 | 28.81 | 16.50 | 7.12 | 28.41 | 46.06 | 16.44 | 17.80 | 21.73 | 19.76 | 21.77 | 19.79 |
| Prussian carp | 1251 | 3 | 2 | 3 | 109.11 | 80.84 | 50.68 | 49.87 | 29.88 | 32.28 | 17.21 | 6.25 | 25.96 | 47.23 | 17.65 | 17.42 | 20.36 | 24.33 | 21.56 | 18.99 |
| Prussian carp | 1252 | 3 | 2 | 3 | 107.33 | 79.46 | 49.12 | 49.78 | 28.66 | 31.92 | 18.26 | 7.86 | 28.01 | 48.46 | 18.28 | 18.69 | 20.84 | 22.29 | 22.09 | 19.55 |
| Prussian carp | 1253 | 3 | 2 | 3 | 109.69 | 81.68 | 47.99 | 47.15 | 26.59 | 30.79 | 17.29 | 6.86 | 28.48 | 46.93 | 18.18 | 17.74 | 23.57 | 24.07 | 23.45 | 21.37 |
| Prussian carp | 1254 | 3 | 2 | 3 | 71.52 | 78.40 | 50.54 | 49.51 | 28.58 | 29.88 | 16.96 | 8.00 | 25.30 | 40.52 | 14.55 | 16.16 | 21.01 | 26.13 | 24.49 | 22.59 |
| Prussian carp | 1255 | 3 | 2 | 3 | 96.49 | 83.71 | 48.75 | 49.89 | 26.85 | 30.53 | 16.74 | 6.05 | 27.13 | 45.85 | 18.62 | 17.42 | 19.43 | 22.85 | 20.58 | 20.21 |
| Prussian carp | 1256 | 3 | 2 | 3 | 93.84 | 80.67 | 52.24 | 45.34 | 25.65 | 30.98 | 15.94 | 7.03 | 27.07 | 43.05 | 18.81 | 16.54 | 19.11 | 20.52 | 19.47 | 17.77 |
| Prussian carp | 1257 | 3 | 2 | 3 | 115.73 | 83.99 | 50.65 | 50.87 | 29.67 | 31.71 | 18.09 | 5.72 | 29.55 | 44.44 | 18.02 | 17.17 | 16.38 | 20.27 | 24.19 | 22.21 |
| Prussian carp | 1258 | 3 | 2 | 3 | 104.12 | 80.49 | 52.28 | 50.84 | 28.14 | 32.70 | 18.26 | 6.93 | 25.92 | 43.84 | 16.31 | 17.21 | 18.54 | 23.68 | 20.26 | 21.91 |
| Prussian carp | 1259 | 3 | 2 | 3 | 61.54 | 78.85 | 48.90 | 46.36 | 25.82 | 29.90 | 16.04 | 7.84 | 25.15 | 41.71 | 18.01 | 17.04 | 22.16 | 23.65 | 22.69 | 18.10 |
| Prussian carp | 1260 | 3 | 2 | 3 | 101.44 | 77.57 | 48.21 | 45.83 | 26.05 | 28.90 | 16.12 | 6.88 | 26.26 | 43.41 | 15.38 | 17.11 | 21.22 | 22.44 | 22.77 | 19.40 |
| Prussian carp | 1261 | 3 | 2 | 3 | 61.11 | 82.83 | 54.15 | 51.78 | 26.85 | 32.77 | 18.11 | 7.22 | 30.24 | 44.37 | 17.25 | 17.81 | 20.61 | 22.12 | 20.38 | 20.23 |
| Prussian carp | 1262 | 3 | 2 | 3 | 61.7 | 78.39 | 51.34 | 45.58 | 29.46 | 30.53 | 17.48 | 5.79 | 28.69 | 43.07 | 18.37 | 16.94 | 20.95 | 23.52 | 21.04 | 20.24 |
| Prussian carp | 1263 | 3 | 2 | 3 | 60.49 | 80.31 | 53.80 | 43.60 | 28.76 | 32.02 | 17.50 | 7.42 | 28.63 | 44.35 | 16.89 | 16.54 | 20.28 | 21.31 | 19.97 | 19.40 |
| Prussian carp | 1264 | 3 | 2 | 3 | 51.25 | 77.91 | 47.79 | 46.34 | 24.79 | 30.17 | 17.46 | 6.32 | 25.88 | 45.49 | 18.80 | 18.02 | 18.51 | 24.85 | 20.43 | 21.46 |
| Prussian carp | 1265 | 3 | 2 | 3 | 49.38 | 80.77 | 49.82 | 51.41 | 28.51 | 30.92 | 18.08 | 6.57 | 27.22 | 44.29 | 19.65 | 17.96 | 20.35 | 23.00 | 22.11 | 20.35 |
| Prussian carp | 1266 | 3 | 2 | 3 | 58.13 | 78.42 | 50.52 | 48.16 | 26.95 | 28.94 | 15.38 | 7.26 | 25.79 | 45.81 | 15.48 | 17.74 | 20.77 | 26.36 | 22.88 | 21.63 |
| Prussian carp | 1267 | 3 | 2 | 3 | 54.31 | 82.07 | 48.83 | 50.08 | 29.67 | 30.33 | 15.98 | 8.56 | 25.82 | 43.62 | 16.92 | 17.22 | 19.91 | 22.62 | 21.28 | 19.87 |
| Prussian carp | 1268 | 3 | 2 | 3 | 50.9 | 79.12 | 49.42 | 45.10 | 28.18 | 30.60 | 18.68 | 6.99 | 28.89 | 49.01 | 16.57 | 18.93 | 20.96 | 26.06 | 22.52 | 19.27 |
| Prussian carp | 1269 | 3 | 2 | 3 | 49.43 | 80.46 | 52.21 | 51.98 | 28.63 | 31.42 | 17.49 | 8.08 | 28.24 | 39.67 | 18.59 | 16.08 | 20.34 | 21.90 | 19.58 | 17.52 |
| Prussian carp | 1270 | 3 | 2 | 3 | 48.42 | 82.47 | 46.90 | 47.38 | 28.83 | 30.74 | 16.46 | 7.61 | 28.22 | 49.27 | 14.95 | 18.26 | 22.54 | 25.73 | 24.50 | 24.25 |
| Prussian carp | 1271 | 3 | 2 | 3 | 48.92 | 80.99 | 52.52 | 48.28 | 28.35 | 28.87 | 15.45 | 7.71 | 26.96 | 41.12 | 17.95 | 16.30 | 19.06 | 22.22 | 19.82 | 19.33 |
| Prussian carp | 1272 | 3 | 2 | 3 | 53.79 | 81.37 | 50.75 | 48.91 | 29.30 | 32.48 | 17.05 | 8.44 | 27.29 | 48.87 | 18.04 | 16.92 | 20.29 | 21.91 | 21.66 | 21.39 |
| Prussian carp | 1273 | 3 | 2 | 3 | 51.38 | 85.38 | 49.28 | 50.36 | 30.06 | 31.32 | 17.43 | 7.35 | 25.93 | 46.26 | 18.75 | 17.28 | 19.52 | 23.67 | 23.18 | 21.60 |
| Prussian carp | 1274 | 3 | 2 | 3 | 58.88 | 80.30 | 52.76 | 51.88 | 28.51 | 32.00 | 18.29 | 7.76 | 28.07 | 43.56 | 16.96 | 18.69 | 20.25 | 22.25 | 19.79 | 20.84 |
| Prussian carp | 1275 | 3 | 2 | 3 | 45.41 | 82.02 | 49.36 | 48.15 | 30.16 | 31.50 | 17.74 | 5.98 | 26.80 | 47.11 | 17.50 | 17.06 | 21.39 | 24.38 | 23.43 | 23.45 |
| Prussian carp | 4030 | 3 | 2 | 3 | 61.29 | 79.62 | 49.90 | 49.04 | 32.73 | 33.39 | 17.97 | 9.69 | 27.59 | 43.45 | 17.77 | 16.39 | 19.03 | 21.02 | 21.49 | 17.81 |
| Prussian carp | 4031 | 3 | 2 | 3 | 48.88 | 84.30 | 50.56 | 47.97 | 26.41 | 31.08 | 16.66 | 6.03 | 28.01 | 47.26 | 15.15 | 17.96 | 21.59 | 24.63 | 23.43 | 21.11 |
| Prussian carp | 4032 | 3 | 2 | 3 | 47.04 | 79.24 | 50.58 | 48.84 | 27.69 | 30.86 | 15.61 | 5.64 | 27.06 | 46.64 | 16.84 | 17.77 | 20.97 | 23.28 | 23.27 | 22.15 |
| Prussian carp | 4033 | 3 | 2 | 3 | 56.83 | 83.32 | 49.01 | 46.40 | 28.18 | 28.85 | 15.53 | 6.56 | 26.26 | 48.95 | 17.43 | 19.00 | 20.72 | 24.10 | 22.63 | 22.09 |
| Prussian carp | 4034 | 3 | 2 | 3 | 46.45 | 81.59 | 56.68 | 48.75 | 27.75 | 32.00 | 16.99 | 6.91 | 26.83 | 45.86 | 17.78 | 14.85 | 19.63 | 24.68 | 22.05 | 21.19 |
| Prussian carp | 1251 | 3 | 3 | 1 | 138.25 | 79.181 | 52.943 | 53.843 | 30.677 | 32.593 | 17.619 | 9.412 | 27.289 | 46.873 | 16.683 | 17.457 | 20.620 | 22.804 | 22.201 | 21.267 |
| Prussian carp | 1252 | 3 | 3 | 1 | 157.72 | 83.076 | 50.326 | 54.534 | 32.767 | 33.297 | 18.707 | 9.148 | 27.559 | 47.251 | 18.763 | 17.564 | 19.754 | 22.837 | 21.849 | 19.35926 |
| Prussian carp | 1253 | 3 | 3 | 1 | 150.68 | 78.912 | 50.647 | 51.300 | 30.436 | 31.687 | 17.596 | 7.733 | 28.866 | 47.778 | 17.022 | 17.914 | 24.730 | 20.676 | 22.849 | 20.65159 |
| Prussian carp | 1254 | 3 | 3 | 1 | 138.62 | 79.341 | 52.889 | 51.100 | 30.891 | 34.320 | 17.191 | 8.945 | 29.798 | 41.896 | 15.588 | 16.250 | 23.532 | 24.640 | 25.702 | 22.54376 |
| Prussian carp | 1255 | 3 | 3 | 1 | 124.38 | 77.229 | 52.386 | 50.919 | 30.324 | 31.295 | 15.120 | 8.385 | 26.926 | 45.282 | 19.927 | 16.193 | 19.389 | 21.842 | 21.981 | 19.84599 |
| Prussian carp | 1256 | 3 | 3 | 1 | 133.82 | 78.434 | 55.790 | 52.157 | 33.374 | 33.027 | 17.221 | 10.049 | 27.375 | 43.519 | 19.172 | 16.002 | 19.025 | 20.946 | 19.974 | 19.90741 |
| Prussian carp | 1257 | 3 | 3 | 1 | 137.36 | 81.068 | 49.727 | 48.448 | 29.874 | 30.982 | 16.008 | 7.723 | 25.914 | 42.418 | 17.736 | 15.936 | 17.361 | 17.056 | 23.021 | 22.06767 |
| Prussian carp | 1258 | 3 | 3 | 1 | 117.1 | 83.749 | 51.681 | 49.490 | 31.343 | 32.729 | 17.372 | 8.490 | 27.933 | 45.380 | 21.183 | 17.350 | 20.072 | 25.943 | 22.208 | 22.64843 |
| Prussian carp | 1259 | 3 | 3 | 1 | 123.14 | 79.868 | 49.601 | 50.261 | 31.987 | 32.713 | 16.866 | 9.322 | 25.998 | 42.993 | 16.959 | 17.476 | 21.172 | 22.978 | 24.554 | 20.5504 |
| Prussian carp | 1260 | 3 | 3 | 1 | 125.44 | 82.230 | 46.182 | 51.574 | 30.593 | 31.430 | 16.396 | 8.478 | 27.099 | 44.188 | 18.660 | 17.526 | 20.984 | 23.621 | 23.473 | 20.86578 |
| Prussian carp | 1261 | 3 | 3 | 1 | 146.72 | 78.931 | 54.432 | 50.473 | 28.815 | 31.670 | 16.773 | 8.743 | 26.893 | 44.396 | 17.545 | 17.353 | 20.455 | 21.587 | 20.503 | 18.83249 |
| Prussian carp | 1262 | 3 | 3 | 1 | 119.04 | 81.081 | 51.951 | 50.347 | 30.529 | 31.942 | 16.719 | 9.067 | 28.600 | 42.972 | 18.815 | 16.275 | 20.323 | 21.706 | 21.496 | 20.6747 |
| Prussian carp | 1263 | 3 | 3 | 1 | 127.47 | 82.502 | 54.609 | 50.670 | 31.429 | 32.948 | 17.561 | 8.399 | 27.542 | 44.033 | 16.051 | 16.544 | 20.207 | 21.132 | 20.903 | 18.93511 |
| Prussian carp | 1264 | 3 | 3 | 1 | 154.73 | 76.340 | 51.592 | 50.640 | 30.511 | 31.801 | 17.712 | 9.542 | 28.143 | 46.981 | 16.182 | 17.605 | 20.202 | 23.203 | 21.630 | 21.10137 |
| Prussian carp | 1265 | 3 | 3 | 1 | 137.51 | 76.914 | 49.575 | 48.986 | 30.238 | 29.732 | 15.834 | 7.023 | 25.572 | 41.172 | 19.158 | 16.576 | 19.141 | 24.571 | 21.800 | 20.89386 |
| Prussian carp | 1266 | 3 | 3 | 1 | 127.32 | 77.235 | 47.613 | 47.629 | 29.577 | 32.783 | 16.876 | 9.855 | 26.385 | 45.944 | 16.377 | 17.225 | 20.819 | 23.380 | 22.774 | 22.33872 |
| Prussian carp | 1267 | 3 | 3 | 1 | 125.7 | 81.345 | 50.201 | 51.690 | 33.032 | 32.588 | 17.061 | 10.800 | 27.674 | 44.367 | 18.626 | 17.127 | 21.902 | 22.941 | 22.455 | 22.97027 |
| Prussian carp | 1268 | 3 | 3 | 1 | 128.85 | 79.764 | 49.364 | 52.204 | 33.659 | 32.831 | 18.117 | 9.097 | 29.186 | 48.290 | 18.505 | 18.473 | 20.433 | 24.527 | 23.017 | 21.89591 |
| Prussian carp | 1269 | 3 | 3 | 1 | 116.42 | 80.384 | 53.905 | 51.512 | 31.802 | 32.843 | 17.494 | 9.710 | 28.151 | 40.642 | 16.564 | 16.160 | 20.859 | 21.924 | 20.007 | 18.65933 |
| Prussian carp | 1270 | 3 | 3 | 1 | 132.28 | 78.026 | 48.192 | 49.384 | 31.140 | 32.773 | 17.293 | 8.457 | 25.521 | 46.790 | 18.140 | 17.615 | 22.764 | 25.214 | 23.365 | 22.90618 |
| Prussian carp | 1271 | 3 | 3 | 1 | 129.32 | 82.306 | 53.881 | 52.775 | 33.838 | 33.735 | 15.494 | 10.550 | 27.916 | 41.531 | 18.837 | 15.903 | 19.217 | 21.518 | 20.240 | 19.41494 |
| Prussian carp | 1272 | 3 | 3 | 1 | 116.84 | 82.033 | 51.673 | 50.485 | 32.643 | 32.762 | 17.178 | 9.648 | 27.081 | 47.931 | 22.105 | 16.558 | 20.501 | 22.124 | 21.270 | 21.38141 |
| Prussian carp | 1273 | 3 | 3 | 1 | 129.22 | 83.482 | 54.087 | 49.079 | 32.060 | 33.024 | 17.356 | 8.895 | 25.217 | 45.517 | 20.804 | 17.328 | 21.228 | 22.434 | 22.075 | 22.69793 |
| Prussian carp | 1274 | 3 | 3 | 1 | 128 | 78.037 | 51.179 | 51.839 | 32.474 | 32.224 | 17.722 | 8.577 | 27.984 | 44.034 | 21.250 | 16.993 | 20.308 | 21.705 | 19.389 | 19.53605 |
| Prussian carp | 1275 | 3 | 3 | 1 | 133.83 | 79.953 | 50.284 | 46.825 | 29.084 | 27.944 | 16.312 | 8.540 | 24.183 | 45.280 | 17.934 | 17.539 | 21.071 | 22.411 | 19.638 | 19.53394 |
| Prussian carp | 4030 | 3 | 3 | 1 | 144.5 | 79.327 | 49.108 | 52.589 | 35.604 | 35.065 | 18.832 | 10.308 | 28.094 | 44.828 | 20.860 | 16.829 | 20.855 | 23.005 | 22.112 | 21.82889 |
| Prussian carp | 4031 | 3 | 3 | 1 | 119.18 | 82.567 | 46.451 | 51.856 | 31.605 | 32.968 | 17.297 | 8.921 | 27.196 | 45.691 | 16.981 | 16.671 | 21.385 | 22.204 | 21.811 | 23.74643 |
| Prussian carp | 4032 | 3 | 3 | 1 | 140.63 | 80.392 | 52.484 | 50.634 | 30.681 | 31.780 | 17.070 | 7.776 | 26.284 | 46.048 | 17.583 | 17.229 | 20.800 | 23.195 | 23.504 | 22.186 |
| Prussian carp | 4033 | 3 | 3 | 1 | 128.42 | 82.424 | 56.645 | 52.146 | 34.838 | 33.840 | 16.741 | 8.206 | 26.930 | 44.658 | 20.135 | 18.884 | 20.401 | 20.513 | 19.118 | 21.5277 |
| Prussian carp | 4034 | 3 | 3 | 1 | 150.68 | 80.065 | 54.052 | 49.946 | 30.554 | 31.680 | 17.226 | 8.674 | 27.120 | 45.562 | 15.998 | 17.578 | 20.662 | 22.915 | 21.807 | 21.40387 |
| Prussian carp | 1251 | 3 | 3 | 2 | 136.08 | 78.920 | 52.353 | 54.588 | 31.503 | 33.120 | 17.010 | 8.844 | 28.183 | 48.095 | 18.986 | 17.657 | 21.379 | 25.207 | 22.107 | 20.32034 |
| Prussian carp | 1252 | 3 | 3 | 2 | 156.8 | 81.792 | 53.185 | 54.366 | 33.172 | 34.728 | 17.487 | 9.712 | 29.950 | 47.609 | 21.364 | 17.769 | 20.088 | 21.725 | 22.025 | 20.13512 |
| Prussian carp | 1253 | 3 | 3 | 2 | 152.3 | 83.584 | 52.577 | 52.922 | 30.048 | 32.383 | 17.343 | 7.924 | 27.524 | 46.439 | 18.386 | 17.488 | 23.681 | 22.411 | 22.936 | 22.27776 |
| Prussian carp | 1254 | 3 | 3 | 2 | 137.39 | 80.918 | 50.598 | 52.125 | 31.222 | 33.760 | 17.674 | 8.860 | 27.686 | 42.126 | 16.174 | 16.478 | 24.941 | 26.667 | 25.994 | 23.28623 |
| Prussian carp | 1255 | 3 | 3 | 2 | 123.3 | 78.656 | 51.102 | 50.087 | 31.378 | 31.445 | 15.673 | 8.248 | 26.378 | 45.478 | 19.296 | 16.469 | 19.436 | 22.871 | 22.633 | 19.79129 |
| Prussian carp | 1256 | 3 | 3 | 2 | 133.14 | 77.614 | 53.929 | 52.583 | 33.665 | 33.476 | 17.051 | 9.769 | 29.971 | 43.790 | 19.436 | 16.234 | 19.845 | 19.809 | 19.138 | 18.71896 |
| Prussian carp | 1257 | 3 | 3 | 2 | 133.47 | 82.109 | 52.260 | 49.572 | 31.084 | 30.520 | 15.719 | 7.344 | 25.802 | 43.839 | 17.838 | 15.994 | 17.333 | 17.135 | 24.229 | 21.8217 |
| Prussian carp | 1258 | 3 | 3 | 2 | 117.51 | 80.120 | 51.255 | 53.022 | 32.286 | 32.411 | 17.196 | 9.329 | 28.601 | 45.218 | 22.400 | 16.878 | 20.012 | 25.793 | 22.567 | 22.29968 |
| Prussian carp | 1259 | 3 | 3 | 2 | 121.64 | 82.592 | 51.819 | 52.404 | 31.666 | 32.783 | 17.325 | 8.929 | 25.759 | 43.774 | 18.273 | 18.208 | 22.469 | 23.885 | 24.242 | 20.45278 |
| Prussian carp | 1260 | 3 | 3 | 2 | 128.6 | 80.386 | 45.377 | 50.511 | 30.668 | 29.907 | 15.911 | 8.049 | 26.533 | 42.470 | 17.515 | 16.906 | 20.189 | 22.060 | 22.256 | 19.85327 |
| Prussian carp | 1261 | 3 | 3 | 2 | 146.45 | 79.290 | 52.896 | 50.851 | 30.763 | 30.653 | 16.966 | 7.412 | 26.664 | 44.626 | 18.310 | 17.473 | 20.536 | 21.277 | 20.906 | 19.9723 |
| Prussian carp | 1262 | 3 | 3 | 2 | 120 | 81.607 | 49.543 | 50.307 | 31.108 | 30.947 | 16.810 | 7.542 | 27.910 | 42.610 | 19.567 | 16.264 | 20.794 | 21.235 | 21.475 | 20.03477 |
| Prussian carp | 1263 | 3 | 3 | 2 | 126.82 | 82.313 | 53.466 | 51.260 | 31.778 | 32.649 | 17.492 | 8.155 | 27.427 | 44.185 | 18.530 | 16.671 | 20.057 | 21.796 | 20.985 | 19.74981 |
| Prussian carp | 1264 | 3 | 3 | 2 | 156.82 | 76.197 | 53.193 | 48.165 | 29.775 | 30.865 | 16.163 | 9.650 | 26.863 | 46.280 | 17.157 | 17.583 | 20.550 | 21.529 | 21.382 | 20.50191 |
| Prussian carp | 1265 | 3 | 3 | 2 | 131.55 | 79.746 | 50.325 | 51.024 | 31.101 | 30.559 | 17.045 | 7.593 | 25.835 | 43.290 | 22.020 | 17.746 | 19.122 | 24.176 | 23.397 | 20.79814 |
| Prussian carp | 1266 | 3 | 3 | 2 | 127.6 | 78.321 | 45.821 | 48.933 | 31.273 | 31.499 | 16.180 | 9.495 | 28.218 | 44.456 | 17.673 | 17.044 | 21.923 | 22.858 | 21.150 | 22.23331 |
| Prussian carp | 1267 | 3 | 3 | 2 | 125.86 | 80.207 | 49.841 | 51.354 | 32.026 | 31.272 | 16.998 | 10.746 | 26.277 | 43.493 | 19.462 | 17.270 | 21.254 | 23.990 | 22.444 | 21.99145 |
| Prussian carp | 1268 | 3 | 3 | 2 | 126.35 | 80.967 | 51.514 | 50.091 | 32.322 | 32.543 | 18.202 | 9.642 | 28.634 | 49.504 | 17.964 | 18.926 | 21.401 | 24.446 | 23.321 | 21.97939 |
| Prussian carp | 1269 | 3 | 3 | 2 | 115.4 | 81.296 | 53.244 | 53.544 | 31.853 | 33.743 | 17.145 | 9.307 | 28.636 | 40.873 | 20.770 | 15.977 | 20.504 | 21.943 | 20.749 | 18.74028 |
| Prussian carp | 1270 | 3 | 3 | 2 | 131.6 | 78.770 | 47.798 | 49.455 | 30.949 | 32.832 | 16.965 | 8.099 | 27.602 | 46.795 | 16.753 | 17.580 | 22.417 | 23.520 | 23.973 | 23.78192 |
| Prussian carp | 1271 | 3 | 3 | 2 | 131.3 | 81.870 | 51.485 | 52.040 | 33.972 | 33.349 | 15.821 | 10.981 | 26.581 | 40.240 | 18.719 | 15.257 | 19.680 | 20.517 | 21.188 | 20.26721 |
| Prussian carp | 1272 | 3 | 3 | 2 | 117.96 | 81.091 | 50.990 | 50.809 | 32.104 | 32.509 | 17.600 | 9.136 | 25.810 | 47.621 | 21.867 | 16.296 | 19.809 | 21.996 | 20.117 | 20.64347 |
| Prussian carp | 1273 | 3 | 3 | 2 | 130.13 | 83.253 | 52.844 | 50.204 | 32.224 | 31.695 | 16.469 | 7.816 | 25.708 | 44.940 | 18.395 | 16.945 | 21.887 | 22.574 | 21.764 | 21.36072 |
| Prussian carp | 1274 | 3 | 3 | 2 | 128.76 | 78.747 | 54.148 | 50.336 | 31.338 | 33.175 | 17.078 | 7.945 | 28.206 | 43.648 | 19.408 | 16.297 | 20.713 | 21.649 | 19.980 | 18.99958 |
| Prussian carp | 1275 | 3 | 3 | 2 | 142.73 | 76.936 | 51.404 | 49.119 | 30.942 | 30.798 | 15.270 | 7.849 | 25.326 | 45.611 | 17.009 | 16.964 | 20.384 | 23.953 | 21.122 | 22.58628 |
| Prussian carp | 4030 | 3 | 3 | 2 | 143.28 | 81.774 | 51.799 | 52.202 | 36.396 | 34.996 | 17.886 | 10.792 | 28.022 | 44.533 | 20.728 | 16.712 | 22.235 | 21.901 | 22.055 | 19.62229 |
| Prussian carp | 4031 | 3 | 3 | 2 | 119.56 | 82.895 | 45.985 | 52.010 | 31.997 | 31.470 | 16.687 | 6.980 | 27.979 | 45.667 | 18.098 | 16.622 | 21.291 | 22.469 | 23.252 | 22.50594 |
| Prussian carp | 4032 | 3 | 3 | 2 | 138.88 | 82.194 | 52.833 | 49.921 | 31.490 | 31.001 | 16.636 | 6.310 | 26.884 | 45.958 | 18.351 | 16.853 | 20.928 | 23.607 | 23.838 | 22.51961 |
| Prussian carp | 4033 | 3 | 3 | 2 | 129.22 | 79.695 | 50.184 | 48.937 | 30.335 | 30.195 | 16.242 | 8.895 | 27.978 | 48.613 | 18.853 | 18.926 | 20.143 | 22.151 | 23.050 | 21.02589 |
| Prussian carp | 4034 | 3 | 3 | 2 | 151.09 | 83.021 | 53.848 | 50.263 | 31.033 | 32.123 | 16.813 | 8.055 | 28.193 | 44.403 | 16.081 | 17.198 | 21.514 | 24.563 | 21.932 | 21.15434 |
| Prussian carp | 1251 | 3 | 3 | 3 | 137.4 | 80.711 | 50.506 | 51.836 | 31.108 | 31.427 | 17.214 | 8.248 | 26.752 | 47.872 | 17.730 | 17.394 | 20.427 | 22.271 | 22.214 | 22.31411 |
| Prussian carp | 1252 | 3 | 3 | 3 | 157.7 | 83.013 | 52.356 | 51.448 | 33.566 | 34.482 | 17.032 | 8.352 | 28.554 | 46.981 | 19.936 | 17.709 | 19.804 | 23.867 | 22.421 | 19.17448 |
| Prussian carp | 1253 | 3 | 3 | 3 | 152.82 | 84.853 | 49.165 | 51.067 | 29.994 | 31.869 | 17.975 | 7.641 | 28.458 | 46.200 | 17.580 | 17.786 | 23.124 | 23.921 | 22.564 | 22.1635 |
| Prussian carp | 1254 | 3 | 3 | 3 | 136.77 | 80.315 | 48.625 | 50.210 | 31.790 | 33.158 | 17.137 | 8.896 | 28.629 | 42.477 | 18.667 | 16.339 | 23.159 | 26.806 | 25.776 | 23.87701 |
| Prussian carp | 1255 | 3 | 3 | 3 | 122.96 | 80.619 | 51.449 | 48.634 | 29.722 | 30.301 | 15.757 | 7.757 | 27.086 | 45.880 | 18.496 | 16.082 | 20.437 | 22.896 | 22.053 | 20.47362 |
| Prussian carp | 1256 | 3 | 3 | 3 | 133.4 | 78.718 | 54.657 | 52.260 | 32.825 | 33.189 | 17.540 | 10.285 | 27.683 | 43.123 | 21.585 | 15.799 | 19.549 | 20.516 | 19.581 | 18.77383 |
| Prussian carp | 1257 | 3 | 3 | 3 | 134.55 | 83.622 | 51.757 | 47.968 | 30.236 | 30.561 | 16.005 | 7.922 | 26.140 | 43.147 | 17.519 | 16.008 | 18.023 | 17.573 | 24.479 | 20.75527 |
| Prussian carp | 1258 | 3 | 3 | 3 | 117.7 | 79.490 | 50.539 | 49.193 | 32.451 | 32.579 | 16.613 | 9.066 | 28.211 | 44.725 | 17.547 | 17.046 | 20.697 | 24.814 | 21.922 | 22.10144 |
| Prussian carp | 1259 | 3 | 3 | 3 | 125.13 | 79.912 | 48.092 | 51.726 | 31.599 | 31.802 | 17.444 | 9.002 | 25.757 | 42.510 | 18.130 | 16.793 | 21.873 | 21.992 | 24.974 | 19.7856 |
| Prussian carp | 1260 | 3 | 3 | 3 | 127.28 | 80.713 | 46.514 | 49.387 | 32.182 | 31.842 | 15.827 | 8.629 | 27.012 | 43.036 | 18.837 | 16.885 | 19.850 | 21.196 | 22.647 | 18.81757 |
| Prussian carp | 1261 | 3 | 3 | 3 | 145.93 | 81.381 | 53.040 | 51.783 | 30.175 | 30.383 | 17.037 | 6.557 | 26.290 | 43.176 | 18.140 | 17.159 | 19.786 | 21.684 | 21.851 | 20.68794 |
| Prussian carp | 1262 | 3 | 3 | 3 | 120.88 | 80.092 | 49.715 | 50.522 | 31.240 | 30.847 | 16.393 | 7.586 | 28.022 | 42.871 | 18.363 | 16.151 | 20.388 | 20.990 | 21.469 | 20.12878 |
| Prussian carp | 1263 | 3 | 3 | 3 | 127.86 | 83.584 | 51.425 | 51.095 | 31.797 | 33.044 | 17.892 | 9.138 | 28.393 | 43.988 | 16.622 | 16.349 | 20.598 | 21.307 | 20.488 | 19.73352 |
| Prussian carp | 1264 | 3 | 3 | 3 | 156.72 | 77.848 | 50.611 | 50.573 | 30.968 | 32.193 | 16.991 | 8.633 | 27.032 | 46.530 | 17.834 | 17.503 | 20.207 | 23.483 | 21.409 | 20.11692 |
| Prussian carp | 1265 | 3 | 3 | 3 | 131.52 | 79.466 | 48.523 | 49.904 | 30.708 | 30.002 | 16.579 | 7.326 | 26.235 | 43.133 | 21.730 | 17.305 | 19.509 | 23.649 | 23.118 | 20.93417 |
| Prussian carp | 1266 | 3 | 3 | 3 | 127.2 | 77.979 | 46.916 | 49.331 | 31.617 | 31.021 | 16.159 | 9.958 | 27.283 | 44.598 | 17.973 | 16.641 | 20.586 | 23.012 | 22.039 | 22.26524 |
| Prussian carp | 1267 | 3 | 3 | 3 | 124.48 | 81.759 | 49.799 | 50.265 | 30.870 | 32.390 | 16.792 | 10.633 | 26.958 | 44.072 | 19.003 | 17.362 | 21.239 | 22.724 | 22.169 | 23.52005 |
| Prussian carp | 1268 | 3 | 3 | 3 | 126.74 | 82.425 | 54.338 | 51.811 | 32.587 | 32.813 | 18.005 | 9.789 | 28.679 | 49.040 | 19.738 | 18.323 | 21.555 | 23.610 | 23.097 | 21.81349 |
| Prussian carp | 1269 | 3 | 3 | 3 | 115.26 | 80.139 | 52.815 | 53.781 | 32.542 | 34.228 | 17.593 | 8.823 | 27.839 | 41.544 | 19.996 | 15.962 | 20.167 | 21.652 | 21.107 | 19.32039 |
| Prussian carp | 1270 | 3 | 3 | 3 | 131.44 | 79.693 | 47.035 | 50.510 | 32.332 | 31.916 | 17.116 | 9.755 | 27.848 | 47.473 | 16.073 | 17.102 | 22.371 | 25.721 | 23.214 | 22.79727 |
| Prussian carp | 1271 | 3 | 3 | 3 | 131.34 | 81.569 | 51.762 | 53.033 | 34.238 | 33.450 | 15.717 | 11.523 | 26.755 | 40.731 | 17.396 | 15.443 | 19.079 | 20.807 | 20.303 | 20.24493 |
| Prussian carp | 1272 | 3 | 3 | 3 | 116.73 | 81.299 | 51.623 | 51.036 | 32.208 | 32.324 | 16.861 | 9.232 | 26.599 | 48.035 | 20.430 | 16.113 | 20.575 | 21.712 | 21.684 | 20.96797 |
| Prussian carp | 1273 | 3 | 3 | 3 | 130.02 | 81.877 | 51.683 | 50.644 | 31.735 | 31.989 | 16.824 | 8.265 | 26.286 | 44.932 | 19.195 | 16.776 | 21.321 | 22.203 | 22.391 | 21.36112 |
| Prussian carp | 1274 | 3 | 3 | 3 | 128.32 | 80.226 | 52.417 | 52.074 | 32.565 | 32.508 | 17.579 | 8.316 | 28.828 | 42.915 | 20.046 | 16.473 | 20.825 | 21.871 | 19.857 | 19.20402 |
| Prussian carp | 1275 | 3 | 3 | 3 | 141.07 | 79.850 | 50.307 | 49.467 | 30.425 | 30.208 | 15.600 | 7.545 | 26.114 | 45.545 | 17.612 | 16.794 | 21.525 | 23.801 | 21.969 | 21.81292 |
| Prussian carp | 4030 | 3 | 3 | 3 | 146.04 | 78.508 | 50.065 | 52.519 | 34.635 | 35.077 | 17.810 | 10.455 | 28.413 | 43.962 | 20.037 | 16.003 | 20.806 | 21.942 | 21.645 | 19.51109 |
| Prussian carp | 4031 | 3 | 3 | 3 | 118.13 | 83.489 | 45.921 | 51.616 | 33.089 | 32.783 | 17.987 | 8.884 | 28.160 | 46.434 | 18.095 | 16.888 | 18.332 | 24.491 | 22.277 | 22.74435 |
| Prussian carp | 4032 | 3 | 3 | 3 | 137.89 | 82.436 | 52.620 | 51.247 | 31.570 | 31.898 | 17.126 | 6.892 | 26.766 | 46.420 | 19.146 | 17.220 | 21.605 | 22.978 | 23.475 | 21.26426 |
| Prussian carp | 4033 | 3 | 3 | 3 | 129.94 | 80.881 | 50.105 | 47.376 | 29.783 | 29.469 | 15.783 | 9.226 | 26.484 | 48.209 | 20.436 | 18.553 | 19.785 | 22.639 | 23.219 | 20.84957 |
| Prussian carp | 4034 | 3 | 3 | 3 | 150.98 | 82.949 | 54.339 | 50.537 | 31.392 | 33.204 | 16.781 | 8.278 | 26.000 | 44.500 | 17.019 | 17.013 | 20.018 | 22.483 | 21.502 | 20.50462 |
