# Supplementary material for: Repeatability, Reproducibility, Separative Power and Subjectivity of Different Fish Morphometric Analysis Methods
Source: PLoS One. 2016 Jun 21;11(6):e0157890. doi: 10.1371/journal.pone.0157890 (PMC4915670; doi:10.1371/journal.pone.0157890)
Supplement: S5 Table — „R” values and significance levels (* = p<0.05; ** = p<0.01) of pairwise Mantel tests made on Euclidean distance matrices of the repeated measurement data of the same measurers. (DOCX) [file pone.0157890.s005.docx]

**Supplementary Table 5** Results of repeatability computations. „R” values and significance levels (* = p<0.05; **= p<0.01) of pairwise Mantel tests made on Euclidean distance matrices of the repeated measurement data of the same measurers

| **species** | **measurer** | **repeated measurement comparison** | **GMB** | | |  | **GMS** | | |  | **TRU** | | |  | **TRA** | | |
| --- | --- | --- | --- | --- | --- | --- | --- | --- | --- | --- | --- | --- | --- | --- | --- | --- | --- |
|  |  |  | **Site1** | **Site2** | **Site3** |  | **Site1** | **Site2** | **Site3** |  | **Site1** | **Site2** | **Site3** |  | **Site1** | **Site2** | **Site3** |
| bleak | M1 | 1 vs 2 | 0.942** | 0.807** | 0.951** |  | 0.522** | 0.856** | 0.824** |  | 0.263* | 0.584** | 0.579** |  | 0.255** | 0.015 | 0.019 |
|  |  | 1 vs 3 | 0.950** | 0.843** | 0.941** |  | 0.406** | 0.777** | 0.771** |  | 0.323** | 0.446** | 0.649** |  | 0.279** | 0.065 | 0.085 |
|  |  | 2 vs 3 | 0.959** | 0.854** | 0.946** |  | 0.544** | 0.852** | 0.864** |  | 0.277* | 0.308** | 0.591** |  | 0.239* | 0.162 | 0.364** |
|  | M2 | 1 vs 2 | 0.947** | 0.859** | 0.983** |  | 0.577** | 0.762** | 0.456** |  | 0.483** | 0.248* | 0.678** |  | 0.361** | 0.150 | 0.338** |
|  |  | 1 vs 3 | 0.945** | 0.847** | 0.970** |  | 0.696** | 0.851** | 0.371** |  | 0.436** | 0.353** | 0.385** |  | 0.142 | 0.346** | 0.208* |
|  |  | 2 vs 3 | 0.965** | 0.875** | 0.981** |  | 0.752** | 0.857** | 0.728** |  | 0.554** | 0.408** | 0.358** |  | 0.274** | 0.336** | 0.108 |
|  | M3 | 1 vs 2 | 0.981** | 0.927** | 0.985** |  | 0.838** | 0.820** | 0.307* |  | 0.656** | 0.409** | 0.613** |  | 0.369** | 0.350** | 0.187* |
|  |  | 1 vs 3 | 0.981** | 0.918** | 0.985** |  | 0.487** | 0.810** | 0.174 |  | 0.616** | 0.635** | 0.501** |  | 0.312* | 0.213* | 0.311** |
|  |  | 2 vs 3 | 0.976** | 0.918** | 0.983** |  | 0.480** | 0.839** | 0.559** |  | 0.581** | 0.616** | 0.466** |  | 0.220* | 0.221* | 0.174 |
| roach | M1 | 1 vs 2 | 0.919** | 0.888** | 0.938** |  | 0.405** | 0.346** | 0.807** |  | 0.545** | 0.619** | 0.408** |  | 0.094 | 0.190* | 0.381* |
|  |  | 1 vs 3 | 0.915** | 0.912** | 0.927** |  | 0.451** | 0.465** | 0.777** |  | 0.710** | 0.666** | 0.529** |  | 0.229* | 0.182* | 0.427** |
|  |  | 2 vs 3 | 0.921** | 0.935** | 0.923** |  | 0.501** | 0.522** | 0.765** |  | 0.523** | 0.642** | 0.396** |  | 0.330** | 0.244** | 0.654** |
|  | M2 | 1 vs 2 | 0.955** | 0.953** | 0.926** |  | 0.459** | 0.601** | 0.549** |  | 0.402** | 0.365** | 0.527** |  | 0.127 | 0.174 | 0.513** |
|  |  | 1 vs 3 | 0.948** | 0.967** | 0.935** |  | 0.376** | 0.542** | 0.526** |  | 0.312** | 0.411** | 0.474** |  | 0.091 | 0.178* | 0.518* |
|  |  | 2 vs 3 | 0.960** | 0.962** | 0.961** |  | 0.700** | 0.521** | 0.558** |  | 0.427** | 0.660** | 0.548** |  | 0.168 | 0.198* | 0.444** |
|  | M3 | 1 vs 2 | 0.938** | 0.982** | 0.963** |  | 0.481** | 0.312** | 0.650** |  | 0.348** | 0.243** | 0.474** |  | 0.191* | 0.200* | 0.494** |
|  |  | 1 vs 3 | 0.927** | 0.977** | 0.954** |  | 0.501** | 0.221* | 0.630** |  | 0.335** | 0.444** | 0.654** |  | 0.257** | 0.175* | 0.560** |
|  |  | 2 vs 3 | 0.923** | 0.975** | 0.964** |  | 0.361** | 0.464** | 0.767** |  | 0.760** | 0.589** | 0.696** |  | 0.210** | 0.051 | 0.617** |
| prussian carp | M1 | 1 vs 2 | 0.691** | 0.681** | 0.901** |  | 0.898** | 0.765** | 0.894** |  | 0.378** | 0.650** | 0.441** |  | 0.208* | 0.456** | 0.158 |
|  |  | 1 vs 3 | 0.749** | 0.618** | 0.897** |  | 0.893** | 0.825** | 0.885** |  | 0.416** | 0.627** | 0.695** |  | 0.258* | 0.200* | 0.198* |
|  |  | 2 vs 3 | 0.820** | 0.581** | 0.891** |  | 0.883** | 0.884** | 0.861** |  | 0.353** | 0.678** | 0.355** |  | 0.292* | 0.214* | 0.297** |
|  | M2 | 1 vs 2 | 0.923** | 0.630** | 0.923** |  | 0.808** | 0.825** | 0.895** |  | 0.535** | 0.562** | 0.340** |  | 0.250* | 0.268* | 0.356** |
|  |  | 1 vs 3 | 0.884** | 0.537** | 0.884** |  | 0.855** | 0.652** | 0.909** |  | 0.563** | 0.580** | 0.397** |  | 0.242* | 0.431** | 0.365** |
|  |  | 2 vs 3 | 0.932** | 0.606** | 0.932** |  | 0.898** | 0.736** | 0.916** |  | 0.533** | 0.453** | 0.608** |  | 0.246* | 0.276* | 0.242* |
|  | M3 | 1 vs 2 | 0.908** | 0.860** | 0.950** |  | 0.898** | 0.765** | 0.894** |  | 0.410** | 0.444** | 0.404** |  | 0.585** | 0.367** | 0.604** |
|  |  | 1 vs 3 | 0.863** | 0.838** | 0.966** |  | 0.893** | 0.825** | 0.885** |  | 0.372** | 0.430** | 0.398** |  | 0.651** | 0.344** | 0.571** |
|  |  | 2 vs 3 | 0.925** | 0.790** | 0.972** |  | 0.883** | 0.884** | 0.861** |  | 0.602** | 0.589** | 0.489** |  | 0.567** | 0.489** | 0.675** |
