# Supplementary material for: Repeatability, Reproducibility, Separative Power and Subjectivity of Different Fish Morphometric Analysis Methods
Source: PLoS One. 2016 Jun 21;11(6):e0157890. doi: 10.1371/journal.pone.0157890 (PMC4915670; doi:10.1371/journal.pone.0157890)
Supplement: S6 Table — „R” values and significance levels (* = p<0.05; ** = p<0.01) of pairwise Mantel tests made on Euclidean distance matrices of the datasets of two different measurers. (DOCX) [file pone.0157890.s006.docx]

**Supplemetary Table 6** Results of reproduciblity computations. „R” values and significance levels (* = p<0.05; **= p<0.01) of pairwise Mantel tests made on Euclidean distance matrices of the datasets of two different measurers

| **Measu-rerA** | **Measu-rerB** | **Site** | **Repeat comparison** | **Bleak** | | | |  | **Roach** | | | |  | **Prussian carp** | | | |
| --- | --- | --- | --- | --- | --- | --- | --- | --- | --- | --- | --- | --- | --- | --- | --- | --- | --- |
|  |  |  |  | **GMB** | **GMS** | **TRU** | **TRA** |  | **GMB** | **GMS** | **TRU** | **TRA** |  | **GMB** | **GMS** | **TRU** | **TRA** |
| M1 | M2 | Site1 | 1 vs 1 | 0.866** | 0.516** | 0.166 | 0.477** |  | 0.915** | 0.414** | 0.428** | 0.150 |  | 0.793** | 0.789** | 0.628** | 0.326** |
| M1 | M2 | Site1 | 1 vs 2 | 0.818** | 0.277* | 0.175 | 0.290** |  | 0.922** | 0.302** | 0.252** | 0.086 |  | 0.856** | 0.803** | 0.430** | 0.482** |
| M1 | M2 | Site1 | 1 vs 3 | 0.848** | 0.329** | 0.104 | 0.230** |  | 0.909** | 0.354** | 0.251** | 0.059 |  | 0.767** | 0.798** | 0.572** | 0.420** |
| M1 | M2 | Site1 | 2 vs 1 | 0.813** | 0.611** | 0.499** | 0.035 |  | 0.907** | 0.353** | 0.169 | 0.082 |  | 0.731** | 0.816** | 0.586** | 0.319** |
| M1 | M2 | Site1 | 2 vs 2 | 0.817** | 0.503** | 0.523** | 0.040 |  | 0.914** | 0.320** | 0.179 | 0.081 |  | 0.648** | 0.831** | 0.446** | 0.362** |
| M1 | M2 | Site1 | 2 vs 3 | 0.857** | 0.572** | 0.439** | 0.197* |  | 0.901** | 0.334** | 0.163 | 0.076 |  | 0.635** | 0.790** | 0.617** | 0.414** |
| M1 | M2 | Site1 | 3 vs 1 | 0.852** | 0.484** | 0.328** | 0.409** |  | 0.915** | 0.312** | 0.449** | 0.333** |  | 0.736** | 0.824** | 0.600** | 0.101 |
| M1 | M2 | Site1 | 3 vs 2 | 0.827** | 0.571** | 0.201* | 0.398** |  | 0.917** | 0.335** | 0.320** | 0.285** |  | 0.692** | 0.806** | 0.383** | 0.319** |
| M1 | M2 | Site1 | 3 vs 3 | 0.880** | 0.492** | 0.143 | 0.201* |  | 0.914** | 0.399** | 0.411** | 0.270** |  | 0.741** | 0.806** | 0.563** | 0.227* |
| M1 | M2 | Site2 | 1 vs 1 | 0.922** | 0.723** | 0.327** | 0.151 |  | 0.887** | 0.279** | 0.293** | 0.174* |  | 0.445** | 0.685** | 0.478** | 0.216** |
| M1 | M2 | Site2 | 1 vs 2 | 0.942** | 0.747** | 0.392** | 0.108 |  | 0.905** | 0.399** | 0.391** | 0.010 |  | 0.597** | 0.675** | 0.470** | 0.256** |
| M1 | M2 | Site2 | 1 vs 3 | 0.941** | 0.787** | 0.525** | 0.278* |  | 0.908** | 0.295* | 0.396** | 0.010 |  | 0.594** | 0.673** | 0.259* | 0.230* |
| M1 | M2 | Site2 | 2 vs 1 | 0.948** | 0.775** | 0.420** | 0.220* |  | 0.944** | 0.479** | 0.422** | 0.061 |  | 0.447** | 0.625** | 0.471** | 0.292** |
| M1 | M2 | Site2 | 2 vs 2 | 0.953** | 0.733** | 0.377** | 0.135 |  | 0.937** | 0.545** | 0.481** | 0.020 |  | 0.493** | 0.556** | 0.403** | 0.288** |
| M1 | M2 | Site2 | 2 vs 3 | 0.945** | 0.799** | 0.395** | 0.212* |  | 0.948** | 0.268* | 0.539** | 0.137 |  | 0.548** | 0.630** | 0.493** | 0.138 |
| M1 | M2 | Site2 | 3 vs 1 | 0.951** | 0.742** | 0.202* | 0.258** |  | 0.933** | 0.552** | 0.415** | 0.060 |  | 0.466** | 0.615** | 0.346** | 0.188* |
| M1 | M2 | Site2 | 3 vs 2 | 0.947** | 0.715** | 0.296** | 0.162 |  | 0.929** | 0.613** | 0.548** | 0.113 |  | 0.454** | 0.567** | 0.362** | 0.127 |
| M1 | M2 | Site2 | 3 vs 3 | 0.965** | 0.774** | 0.443** | 0.400** |  | 0.939** | 0.436** | 0.452** | 0.090 |  | 0.527** | 0.573** | 0.393** | 0.317** |
| M1 | M2 | Site3 | 1 vs 1 | 0.954** | 0.197* | 0.497** | 0.238* |  | 0.905** | 0.196* | 0.406** | 0.161 |  | 0.866** | 0.782** | 0.611** | 0.129 |
| M1 | M2 | Site3 | 1 vs 2 | 0.949** | 0.439** | 0.438** | 0.090 |  | 0.915** | 0.299** | 0.551** | 0.040 |  | 0.868** | 0.786** | 0.495** | 0.129 |
| M1 | M2 | Site3 | 1 vs 3 | 0.933** | 0.655** | 0.461** | 0.030 |  | 0.934** | 0.221* | 0.504** | 0.060 |  | 0.872** | 0.803** | 0.609** | 0.285** |
| M1 | M2 | Site3 | 2 vs 1 | 0.949** | 0.196 | 0.496** | 0.272* |  | 0.916** | 0.181* | 0.304** | 0.060 |  | 0.886** | 0.799** | 0.581** | 0.316** |
| M1 | M2 | Site3 | 2 vs 2 | 0.948** | 0.313** | 0.441** | 0.066 |  | 0.906** | 0.361** | 0.530** | 0.172* |  | 0.892** | 0.783** | 0.540** | 0.253** |
| M1 | M2 | Site3 | 2 vs 3 | 0.936** | 0.572** | 0.418** | 0.331** |  | 0.916** | 0.156* | 0.385** | 0.290** |  | 0.909** | 0.803** | 0.639** | 0.374** |
| M1 | M2 | Site3 | 3 vs 1 | 0.960** | 0.300** | 0.532** | 0.308** |  | 0.905** | 0.249* | 0.299** | 0.070 |  | 0.867** | 0.749** | 0.476** | 0.337** |
| M1 | M2 | Site3 | 3 vs 2 | 0.964** | 0.359** | 0.460** | 0.177 |  | 0.931** | 0.324** | 0.471** | 0.060 |  | 0.882** | 0.760** | 0.601** | 0.303** |
| M1 | M2 | Site3 | 3 vs 3 | 0.957** | 0.576** | 0.378** | 0.238* |  | 0.943** | 0.156* | 0.429** | 0.210* |  | 0.898** | 0.776** | 0.608** | 0.403** |
| M1 | M3 | Site1 | 1 vs 1 | 0.855** | 0.191 | 0.138 | 0.581** |  | 0.911** | 0.318** | 0.401** | 0.130 |  | 0.854** | 0.841** | 0.742** | 0.130 |
| M1 | M3 | Site1 | 1 vs 2 | 0.869** | 0.249* | 0.283* | 0.462** |  | 0.926** | 0.417** | 0.558** | 0.221* |  | 0.862** | 0.859** | 0.840** | 0.131 |
| M1 | M3 | Site1 | 1 vs 3 | 0.851** | 0.406** | 0.273* | 0.349* |  | 0.923** | 0.310** | 0.611** | 0.172 |  | 0.842** | 0.889** | 0.744** | 0.254* |
| M1 | M3 | Site1 | 2 vs 1 | 0.827** | 0.245* | 0.464** | 0.053 |  | 0.897** | 0.301** | 0.786** | 0.172 |  | 0.655** | 0.928** | 0.704** | 0.218* |
| M1 | M3 | Site1 | 2 vs 2 | 0.843** | 0.234* | 0.693** | 0.110 |  | 0.908** | 0.331** | 0.485** | 0.109 |  | 0.758** | 0.893** | 0.837** | 0.467** |
| M1 | M3 | Site1 | 2 vs 3 | 0.839** | 0.480** | 0.612** | 0.188 |  | 0.916** | 0.270** | 0.473** | 0.057 |  | 0.774** | 0.900** | 0.753** | 0.313** |
| M1 | M3 | Site1 | 3 vs 1 | 0.860** | 0.322* | 0.391** | 0.584** |  | 0.923** | 0.417** | 0.339** | 0.220* |  | 0.659** | 0.850** | 0.678** | 0.249* |
| M1 | M3 | Site1 | 3 vs 2 | 0.870** | 0.395** | 0.532** | 0.435** |  | 0.909** | 0.479** | 0.675** | 0.402** |  | 0.811** | 0.877** | 0.810** | 0.399** |
| M1 | M3 | Site1 | 3 vs 3 | 0.851** | 0.570** | 0.466** | 0.255* |  | 0.922** | 0.422** | 0.713** | 0.419** |  | 0.826** | 0.890** | 0.616** | 0.351** |
| M1 | M3 | Site2 | 1 vs 1 | 0.949** | 0.785** | 0.651** | 0.270* |  | 0.932** | 0.382* | 0.520** | 0.224* |  | 0.640** | 0.753** | 0.600** | 0.472** |
| M1 | M3 | Site2 | 1 vs 2 | 0.951** | 0.807** | 0.625** | 0.243* |  | 0.926** | 0.385* | 0.105 | 0.220* |  | 0.658** | 0.754** | 0.611** | 0.410** |
| M1 | M3 | Site2 | 1 vs 3 | 0.957** | 0.735** | 0.560** | 0.226* |  | 0.922** | 0.372* | 0.192 | 0.211* |  | 0.653** | 0.730** | 0.513** | 0.576** |
| M1 | M3 | Site2 | 2 vs 1 | 0.952** | 0.816** | 0.614** | 0.553** |  | 0.942** | 0.622** | 0.664** | 0.154 |  | 0.658** | 0.643** | 0.712** | 0.536** |
| M1 | M3 | Site2 | 2 vs 2 | 0.945** | 0.803** | 0.629** | 0.138 |  | 0.947** | 0.602** | 0.183 | 0.106 |  | 0.692** | 0.863** | 0.636** | 0.324** |
| M1 | M3 | Site2 | 2 vs 3 | 0.957** | 0.722** | 0.672** | 0.083 |  | 0.955** | 0.604** | 0.289** | 0.280** |  | 0.685** | 0.836** | 0.703** | 0.471** |
| M1 | M3 | Site2 | 3 vs 1 | 0.971** | 0.765** | 0.484** | 0.182 |  | 0.940** | 0.597** | 0.448** | 0.020 |  | 0.592** | 0.676** | 0.679** | 0.355** |
| M1 | M3 | Site2 | 3 vs 2 | 0.964** | 0.779** | 0.451** | 0.271* |  | 0.940** | 0.589** | 0.150 | 0.130 |  | 0.634** | 0.668** | 0.608** | 0.180 |
| M1 | M3 | Site2 | 3 vs 3 | 0.972** | 0.653** | 0.461** | 0.201* |  | 0.947** | 0.594** | 0.170 | 0.100 |  | 0.598** | 0.653** | 0.640** | 0.318** |
| M1 | M3 | Site3 | 1 vs 1 | 0.956** | 0.172 | 0.343** | 0.274** |  | 0.930** | 0.448** | 0.570** | 0.060 |  | 0.873** | 0.854** | 0.477** | 0.133 |
| M1 | M3 | Site3 | 1 vs 2 | 0.959** | 0.423** | 0.415** | 0.309** |  | 0.934** | 0.549** | 0.526** | 0.280* |  | 0.909** | 0.842** | 0.516** | 0.133 |
| M1 | M3 | Site3 | 1 vs 3 | 0.952** | 0.375** | 0.600** | 0.309* |  | 0.915** | 0.472** | 0.545** | 0.282** |  | 0.899** | 0.845** | 0.727** | 0.254** |
| M1 | M3 | Site3 | 2 vs 1 | 0.957** | 0.173 | 0.246 | 0.289** |  | 0.918** | 0.470** | 0.397** | 0.457** |  | 0.917** | 0.903** | 0.831** | 0.313** |
| M1 | M3 | Site3 | 2 vs 2 | 0.955** | 0.303* | 0.501** | 0.233* |  | 0.931** | 0.358** | 0.402** | 0.459** |  | 0.920** | 0.836** | 0.484** | 0.466** |
| M1 | M3 | Site3 | 2 vs 3 | 0.953** | 0.278* | 0.582** | 0.271* |  | 0.928** | 0.261* | 0.442** | 0.568** |  | 0.886** | 0.840** | 0.816** | 0.218* |
| M1 | M3 | Site3 | 3 vs 1 | 0.971** | 0.219* | 0.223* | 0.211* |  | 0.948** | 0.403** | 0.497** | 0.070 |  | 0.879** | 0.817** | 0.762** | 0.249** |
| M1 | M3 | Site3 | 3 vs 2 | 0.959** | 0.315* | 0.540** | 0.114 |  | 0.946** | 0.265* | 0.571** | 0.118 |  | 0.905** | 0.788** | 0.446** | 0.399** |
| M1 | M3 | Site3 | 3 vs 3 | 0.968** | 0.271* | 0.575** | 0.375** |  | 0.935** | 0.372** | 0.577** | 0.326** |  | 0.910** | 0.810** | 0.801** | 0.351** |
| M2 | M3 | Site1 | 1 vs 1 | 0.879** | 0.156 | 0.596** | 0.541** |  | 0.961** | 0.315** | 0.427** | 0.272** |  | 0.835** | 0.776** | 0.674** | 0.210* |
| M2 | M3 | Site1 | 1 vs 2 | 0.901** | 0.171 | 0.552** | 0.461** |  | 0.965** | 0.342** | 0.466** | 0.436** |  | 0.863** | 0.798** | 0.637** | 0.254* |
| M2 | M3 | Site1 | 1 vs 3 | 0.887** | 0.413** | 0.507** | 0.295* |  | 0.958** | 0.288** | 0.502** | 0.412** |  | 0.868** | 0.816** | 0.657** | 0.275** |
| M2 | M3 | Site1 | 2 vs 1 | 0.868** | 0.142 | 0.451** | 0.564** |  | 0.963** | 0.231** | 0.462** | 0.338** |  | 0.926** | 0.844** | 0.534** | 0.590** |
| M2 | M3 | Site1 | 2 vs 2 | 0.896** | 0.210* | 0.620** | 0.342** |  | 0.969** | 0.231* | 0.381** | 0.244** |  | 0.893** | 0.831** | 0.481** | 0.268** |
| M2 | M3 | Site1 | 2 vs 3 | 0.870** | 0.348** | 0.483** | 0.327** |  | 0.971** | 0.176* | 0.450** | 0.225* |  | 0.895** | 0.804** | 0.593** | 0.499** |
| M2 | M3 | Site1 | 3 vs 1 | 0.916** | 0.262* | 0.385** | 0.277* |  | 0.953** | 0.471** | 0.418** | 0.070 |  | 0.768** | 0.754** | 0.552** | 0.336** |
| M2 | M3 | Site1 | 3 vs 2 | 0.914** | 0.258* | 0.435** | 0.144 |  | 0.959** | 0.433** | 0.587** | 0.168 |  | 0.867** | 0.802** | 0.553** | 0.338** |
| M2 | M3 | Site1 | 3 vs 3 | 0.908** | 0.376** | 0.392** | 0.115 |  | 0.953** | 0.358** | 0.589** | 0.238* |  | 0.818** | 0.808** | 0.617** | 0.387** |
| M2 | M3 | Site2 | 1 vs 1 | 0.955** | 0.707** | 0.374** | 0.232* |  | 0.966** | 0.270* | 0.404** | 0.340** |  | 0.862** | 0.732** | 0.504** | 0.310** |
| M2 | M3 | Site2 | 1 vs 2 | 0.944** | 0.783** | 0.488** | 0.273** |  | 0.965** | 0.284* | 0.476** | 0.208** |  | 0.851** | 0.766** | 0.610** | 0.236* |
| M2 | M3 | Site2 | 1 vs 3 | 0.961** | 0.646** | 0.223* | 0.177* |  | 0.970** | 0.339** | 0.449** | 0.223** |  | 0.802** | 0.690** | 0.515** | 0.316** |
| M2 | M3 | Site2 | 2 vs 1 | 0.963** | 0.741** | 0.378** | 0.324** |  | 0.945** | 0.569** | 0.483** | 0.137 |  | 0.726** | 0.710** | 0.550** | 0.338** |
| M2 | M3 | Site2 | 2 vs 2 | 0.966** | 0.691** | 0.422** | 0.364** |  | 0.955** | 0.569** | 0.532** | 0.120 |  | 0.664** | 0.703** | 0.581** | 0.290** |
| M2 | M3 | Site2 | 2 vs 3 | 0.961** | 0.611** | 0.298** | 0.237* |  | 0.967** | 0.546** | 0.507** | 0.170 |  | 0.646** | 0.651** | 0.507** | 0.309** |
| M2 | M3 | Site2 | 3 vs 1 | 0.957** | 0.788** | 0.548** | 0.473** |  | 0.970** | 0.245* | 0.591** | 0.226** |  | 0.668** | 0.707** | 0.508** | 0.320** |
| M2 | M3 | Site2 | 3 vs 2 | 0.962** | 0.799** | 0.526** | 0.327** |  | 0.973** | 0.215* | 0.634** | 0.173* |  | 0.617** | 0.639** | 0.547** | 0.518** |
| M2 | M3 | Site2 | 3 vs 3 | 0.949** | 0.706** | 0.446** | 0.376** |  | 0.976** | 0.257* | 0.630** | 0.121 |  | 0.637** | 0.622** | 0.527** | 0.460** |
| M2 | M3 | Site3 | 1 vs 1 | 0.980** | 0.297** | 0.223* | 0.265* |  | 0.910** | 0.207* | 0.466** | 0.186 |  | 0.929** | 0.777** | 0.598** | 0.320** |
| M2 | M3 | Site3 | 1 vs 2 | 0.977** | 0.226* | 0.315** | 0.312** |  | 0.940** | 0.090 | 0.403** | 0.349** |  | 0.921** | 0.725** | 0.430** | 0.500** |
| M2 | M3 | Site3 | 1 vs 3 | 0.978** | 0.285** | 0.410** | 0.434** |  | 0.937** | 0.191* | 0.400** | 0.231* |  | 0.939** | 0.755** | 0.527** | 0.480** |
| M2 | M3 | Site3 | 2 vs 1 | 0.984** | 0.217* | 0.166 | 0.191* |  | 0.963** | 0.386** | 0.553** | 0.080 |  | 0.945** | 0.763** | 0.573** | 0.337** |
| M2 | M3 | Site3 | 2 vs 2 | 0.980** | 0.385** | 0.341** | 0.292** |  | 0.952** | 0.242* | 0.524** | 0.100 |  | 0.945** | 0.744** | 0.346* | 0.439** |
| M2 | M3 | Site3 | 2 vs 3 | 0.986** | 0.383** | 0.403** | 0.250* |  | 0.948** | 0.296** | 0.568** | 0.179 |  | 0.948** | 0.749** | 0.659** | 0.490** |
| M2 | M3 | Site3 | 3 vs 1 | 0.975** | 0.229* | 0.186** | 0.276* |  | 0.959** | 0.314** | 0.653** | 0.178 |  | 0.924** | 0.800** | 0.614** | 0.281** |
| M2 | M3 | Site3 | 3 vs 2 | 0.972** | 0.468** | 0.364** | 0.265* |  | 0.963** | 0.275** | 0.620** | 0.248* |  | 0.941** | 0.767** | 0.311* | 0.436** |
| M2 | M3 | Site3 | 3 vs 3 | 0.976** | 0.447** | 0.467** | 0.547** |  | 0.942** | 0.282** | 0.583** | 0.272** |  | 0.942** | 0.786** | 0.688** | 0.520** |
